# Recent status and trends regarding oxidative stress in gliomas (2013 - 2025): a systematic review and bibliometric analysis

**DOI:** 10.3389/fonc.2025.1586515

**Published:** 2025-05-16

**Authors:** Yijun Zeng, Xiaowen Lu, Yadan Wang, Jihong He, Hui Cao, Lushun Zhang, Li Cheng

**Affiliations:** ^1^ Department of Neurosurgery, The Third Affiliated Hospital of Chengdu Medical College, Chengdu Pidu District People’s Hospital, Chengdu, China; ^2^ School of Clinical Medicine, Chengdu Medical College, Chengdu, China; ^3^ Department of Pathology and Pathophysiology, Chengdu Medical College, Chengdu, China; ^4^ Graduate School, Chengdu Medical College, Chengdu, China

**Keywords:** oxidative stress, gliomas, bibliometrics, Citespace, VOSviewer, visualization

## Abstract

**Background:**

Glioma, a prevalent brain tumor originating from glial cells, exhibits rapid growth, high recurrence, and significant invasiveness. Standard treatments include surgery, radiotherapy, and chemotherapy, yet their effectiveness remains unsatisfactory. Recent studies implicate oxidative stress in promoting glioma cell proliferation and migration, as well as enhancing survival rates, suggesting antioxidant therapy as a potential tumor treatment strategy.

**Objective:**

The aim of this review is to summarize the research hotspots on antioxidant treatment options for gliomas in the last twelve years and analyze the future trends through bibliometric analysis.

**Method:**

We collected articles on oxidative stress in gliomas published between January 1, 2013, and April 5, 2025, using the Web of Science (WOS) database. We also visualized and analyzed annual publications, countries, and journals using VOSviewer, Citespace, and pajek.

**Result:**

The search yielded a total of 1020 publications. Visual analyses show that the number of articles on this topic has increased annually over the last twelve years. Most of the studies came from China, followed by the United States. The three most cited journals were International Journal of Molecular Sciences, Cancer and Frontiers in Oncology. The author who published the most articles on this topic was Wang HD.

**Conclusion:**

Through a systematic analysis, we found that current research hotspots mainly focus on the dose of reactive oxygen species (ROS) and tumor proliferation, inflammatory response, apoptosis, etc. in relation to oxidative stress. In addition, we analyzed the direction of future research: a possible focus on the treatment of gliomas via ‘tumor microenvironment’, ‘blood-brain barrier’, ‘anti-inflammatory’ and ‘ ferroptosis induction ‘ routes.

## 1 Introduction

Gliomas are primary malignant tumors of the central nervous system (CNS) and constitute the most prevalent type of adult CNS tumors, accounting for 81% of all malignant brain tumors (1). They are traditionally classified into four grades based on morphological changes. Grade I gliomas are benign, non-invasive tumors with a favorable prognosis and infrequent occurrence. Grade II gliomas are infiltrative and low proliferative, with a tendency to recur. Grade III gliomas represent highly malignant types, such as anaplastic astrocytoma. Grade IV gliomas are recognized as glioblastoma (GBM) (2), the most invasive and prevalent brain cancer in adults, accounting for approximately 60% of all cases (1).

GBM is an immunosuppressive deadly brain cancer containing glioblastoma stem-like cells. GBM progresses rapidly, exhibits strong resistance to treatment, and has a high recurrence rate, attributed to factors such as rapid growth, molecular heterogeneity, invasive potential into critical brain structures, and regenerative capabilities of drug-resistant cancer stem cells. Clinical management of gliomas includes surgery, radiotherapy, chemotherapy, targeted therapy, and immunotherapy (3).

The management of glioblastoma remains a profound challenge for researchers and clinicians, yet recent breakthroughs in oxidative stress research have offered novel therapeutic paradigms for long-term glioma intervention (2). The reduction of molecular oxygen in mitochondria generates ROS as part of normal cellular metabolism. Oxidative stress arises from the ongoing imbalance between the production of ROS/free radicals and the cellular antioxidant defenses that neutralize them (4). Mitochondria serve as the primary loci of ROS generation, accounting for approximately 90% of cellular ROS production. Under hypoxic or bioenergetic stress, dysfunction in the electron transport chain leads to electron leakage from Complex I (NADH dehydrogenase) and Complex II (succinate dehydrogenase), facilitating the reduction of molecular oxygen to superoxide anions, the precursor of downstream ROS species (5). Current evidence underscores the dual role of ROS in gliomagenesis: physiologic ROS levels act as signaling molecules to promote tumor cell proliferation and survival through mitogenic pathways, whereas excessive ROS induces cytotoxicity via DNA/RNA strand breaks, protein carbonylation, and lipid peroxidation, thereby regulating glioma cell cycle progression and apoptotic cell death (6, 7). Beyond these effects, ROS modulate key hallmarks of tumor malignancy, including invasive migration, neovascularization, and therapeutic resistance, establishing their centrality in glioma progression and treatment response (5).

Consequently, contemporary therapeutic strategies predominantly aim to exploit oxidative stress for inducing glioma cell apoptosis (8).This is achieved through various strategies, such as enhancing ROS levels by targeted mitochondrial interventions, activating NADPH oxidase (NOX) to elevate ROS levels in tumor cells, and inhibiting TrxR activity to accumulate ROS *in vivo* (9). Once ROS levels surpass the cellular tolerance threshold of tumor cells, oxidative damage ensues, triggering mitochondria-mediated apoptotic cascades via intrinsic signaling pathways (10).

To systematically characterize the current landscape and emerging trajectories of oxidative stress research in glioma biology, we employed bibliometric methodologies to conduct quantitative analysis and visual mapping of scholarly literature in this domain. As a widely adopted framework for integrative analysis of research outputs, bibliometrics enables qualitative and quantitative assessment of contributions from countries, institutions, and collaborative networks (11, 12). By deciphering the knowledge architecture and identifying focal research areas, this analytical tool provides a structured foundation for informing future investigative directions (13, 14).

## 2 Materials and methods

### 2.1 Data collection

**Figure 1:**
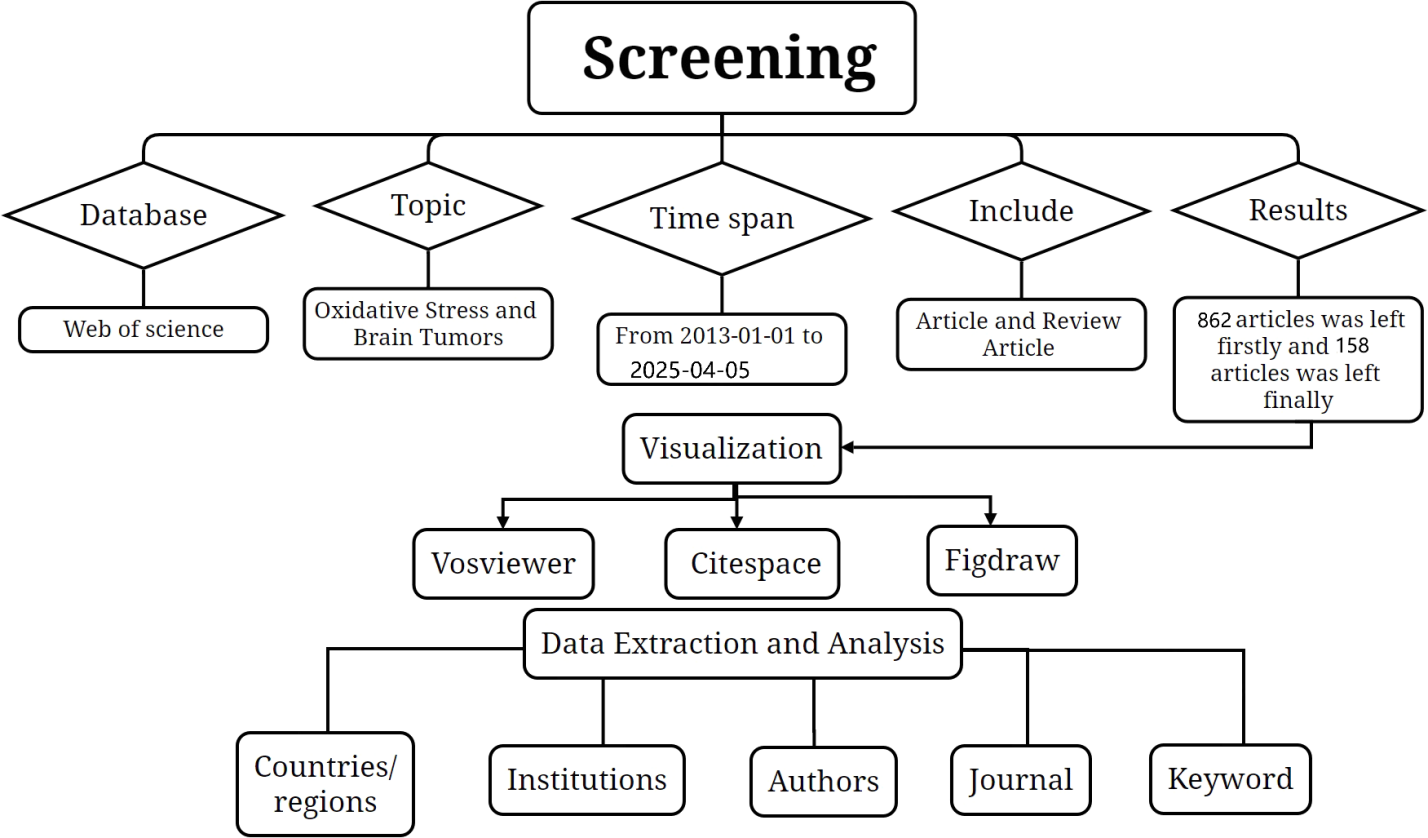
The flowchart illustrating the search strategy and selection process.

The database of this study was collected from WOS, which is known to be a high-quality database of digital bibliographic resources. Most researchers consider it as the most appropriate database for bibliometric analyses, with more than 12,000 high-quality journals. Based on the literature regarding oxidative stress and glioma, we expanded his keywords first and then searched the authors who published the most on this topic and by this we collected the free words about the topic. By respectively expanding the free words for glioma and oxidative stress and then intersecting the two, we came up with the search formula. The search strategy is shown in **Supplementary Material 1 (S1)**. Only the English-language papers and review papers published between 1 January 2013 and 5 April 2025 were selected. The truncation symbol “*” was used with the aim of preventing the omission of valid keywords. By setting the time, article type, keyword expansion and reading screening, 1020 English papers and review papers were finally identified as the research objects to start the topic discussion (**Figure 1**).

### 2.2 Data analysis and visualization

Quantitative analysis and knowledge domain visualization were performed using two prominent bibliometric software packages:

VOSviewer 1.6.19 was utilized to construct co-citation and co-occurrence networks, generating visual maps for authors, journals, and keywords based on bibliographic coupling or term co-occurrence data (15). This tool enabled the visualization of collaborative patterns and thematic clusters through density- and similarity-based mapping.

CiteSpace 6.2.R3 (developed by Prof. Chaomei Chen) was employed for temporal trend analysis and critical node identification, leveraging citation burst detection and time-zone visualization to uncover emerging research frontiers and pivotal milestones in the oxidative stress–glioma field.

For descriptive statistical analysis, Microsoft Excel 2019 was used to tabulate essential bibliometric indicators, including annual publication trends, author productivity, country/region and institutional contributions, journal distributions, citation counts, and keyword co-occurrence frequencies. To systematically evaluate research impact, H-indices for leading authors, countries, and institutions were extracted, alongside 2022 journal metrics—specifically, impact factors and Journal Citation Reports quartile rankings—for the top 10 journals by publication output in this domain.

Mechanistic schematics depicting the role of oxidative stress in glioma biology, including molecular pathways and therapeutic targets, were visualized using Figdraw. These diagrams synthesized key findings from the literature analysis to illustrate the bidirectional relationship between redox homeostasis and glioma progression, enhancing the interpretability of complex biological interactions.

## 3 Results

### 3.1 Publication and citation analysis

**Figure 2:**
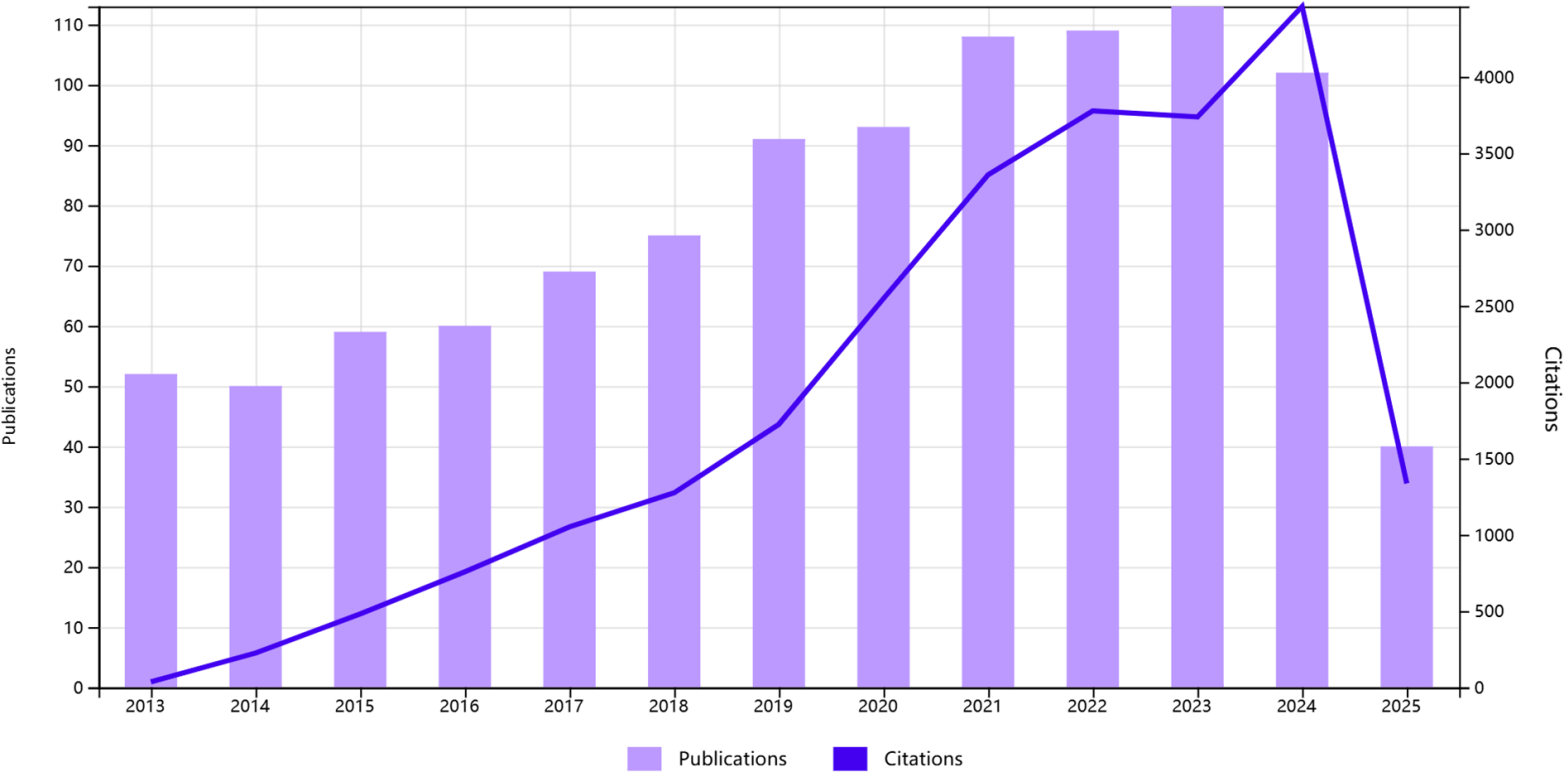
Annual number of studies on topics related to glioma and oxidative stress.

1020 English-language articles on oxidative stress and gliomas obtained in our search, 158 reviews and 862 papers were included. The total number of citations among them was around 18,000, excluding the number of self-citations about 17,000. The average number of citations for publications was 21.59 As shown in **Figure 2**, overall trend of the number of publications showed a gradual increase. Analyzing the data from the papers shows that the number of annual publications has gradually increased from 52 in 2013 to 119 in 2023. From 2013 to 2017 the number of publications was more stable, fluctuating around 60. It indicates that there is not much research progress during that period. From 2017 to 2025, the number of publications increased year by year, and surpassed 100 in 2021, indicating a breakthrough in research on oxidative stress. It is predictable that oxidative stress will be a major hotspot for glioma treatment in the future.

### 3.2 Country analyses

**Figure 3:**
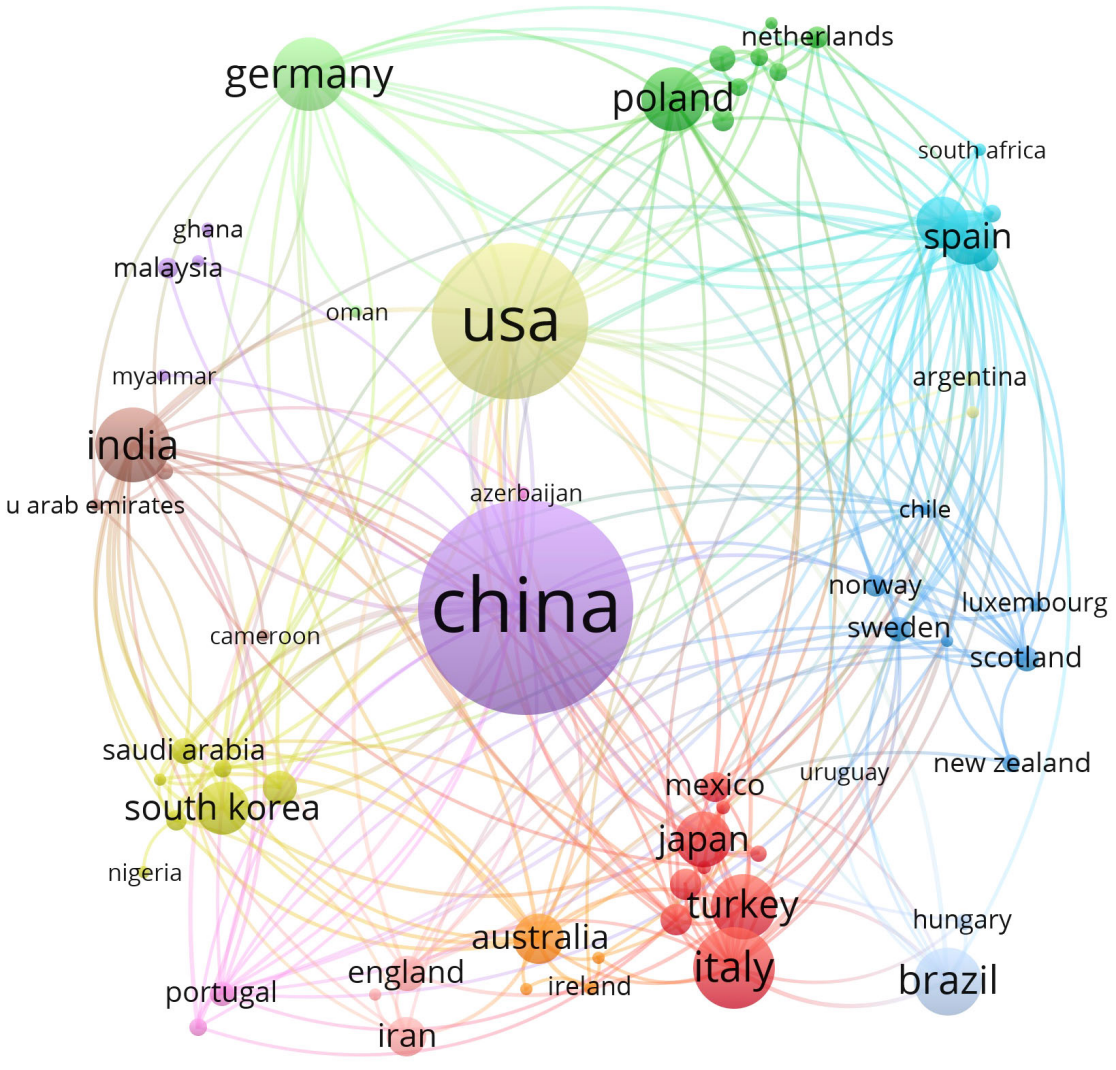
The visualization of countries on research of oxidative stress in gliomas.

A total of 71 countries were involved in the study of oxidative stress in gliomas from 1 January 2013 to 5 April 2025, **Table 1** lists the top ten countries with the highest number of publications, with the highest number of publications in China (count = 313) followed by the United States (count = 189) and Italy (count = 72) In terms of centrality, the United States ranked as the world’s first (centrality = 0.55) followed by China, (centrality = 0.24). Both countries have a centrality degree greater than 0.2, indicating that both countries have a strong academic influence in this field. VOSviewer was used to display the 71 countries mentioned above in the field of international cooperation, as shown in **Figure 3**. Circles and labels form an element, the size of the element depends on the degree of the node, the strength of the linkage, the number of citations, etc., and the colors of the element represent the cluster it belongs to, and different clusters are indicated by different colors. This indicates that China and the United States are the most connected in this area.

**Table 1:** Top 10 countries on research of oxidative stress in gliomas.

Rank 1	Count	Centrality	Year	Countries/Regions
1	313	0.24	2013	CHINA
2	189	0.55	2013	USA
3	72	0.09	2013	ITALY
4	57	0.18	2013	INDIA
5	54	0.18	2013	GERMANY
6	47	0.08	2013	BRAZIL
7	46	0.06	2013	TURKEY
8	40	0.03	2014	POLAND
9	36	0.03	2014	TAIWAN, China
10	35	0.11	2013	JAPAN
11	35	0.11	2013	SPAIN

### 3.3 Author analysis

In our analysis of oxidative stress in gliomas, the top 10 most prolific authors based on publication count were listed in **Table 2**. Among them, Wang, HD. emerged as the most productive author with 9 publications and a total citation count of 283, marking a high level of both output and impact. A comparative review of authors’ total citations (**Table 3**) revealed that most of the top 10 publishing authors had citation counts exceeding 100; however, two authors exhibited a discrepancy between productivity and influence, with citation counts as low as 48.

**Table 2:** Top 10 authors on research of oxidative stress in gliomas.

Rank 2	Count	Centrality	Authors	Times Cited	Average per item	H-Index
1	9	0.88	Wang HD	283	31.44	8
2	8	0.78	Kusaczuk M	194	24.25	6
3	8	0.78	Turkez H	188	23.5	8
4	8	0.78	Huntosova V	123	15.38	6
5	8	0.78	Ge P	359	44.88	8
6	7	0.68	Bona NP	48	6.86	5
7	7	0.68	Naziroglu M	192	27.43	6
8	7	0.68	Zhang X	189	27	6
9	7	0.68	StarkPedra N	48	6.86	5
10	7	0.68	Naziroglu M	229	32.71	7

**Table 3:** Top 10 co-authors on research of oxidative stress in gliomas.

Rank 3	Count	Centrality	Year	Co-Authors
1	240	0.06	2013	STUPP R
2	179	0.01	2013	LOUIS DN
3	152	0.02	2015	OSTROM QT
4	84	0.02	2013	WANG J
5	73	0.12	2013	WEN PY
6	70	0.02	2013	CHEN J
7	70	0.06	2017	ZHANG Y
8	60	0.12	2013	OHGAKI H
9	59	0.08	2013	VERHAAK RGW
10	59	0.06	2013	TRACHOOTHAM D

**Figure 4:**
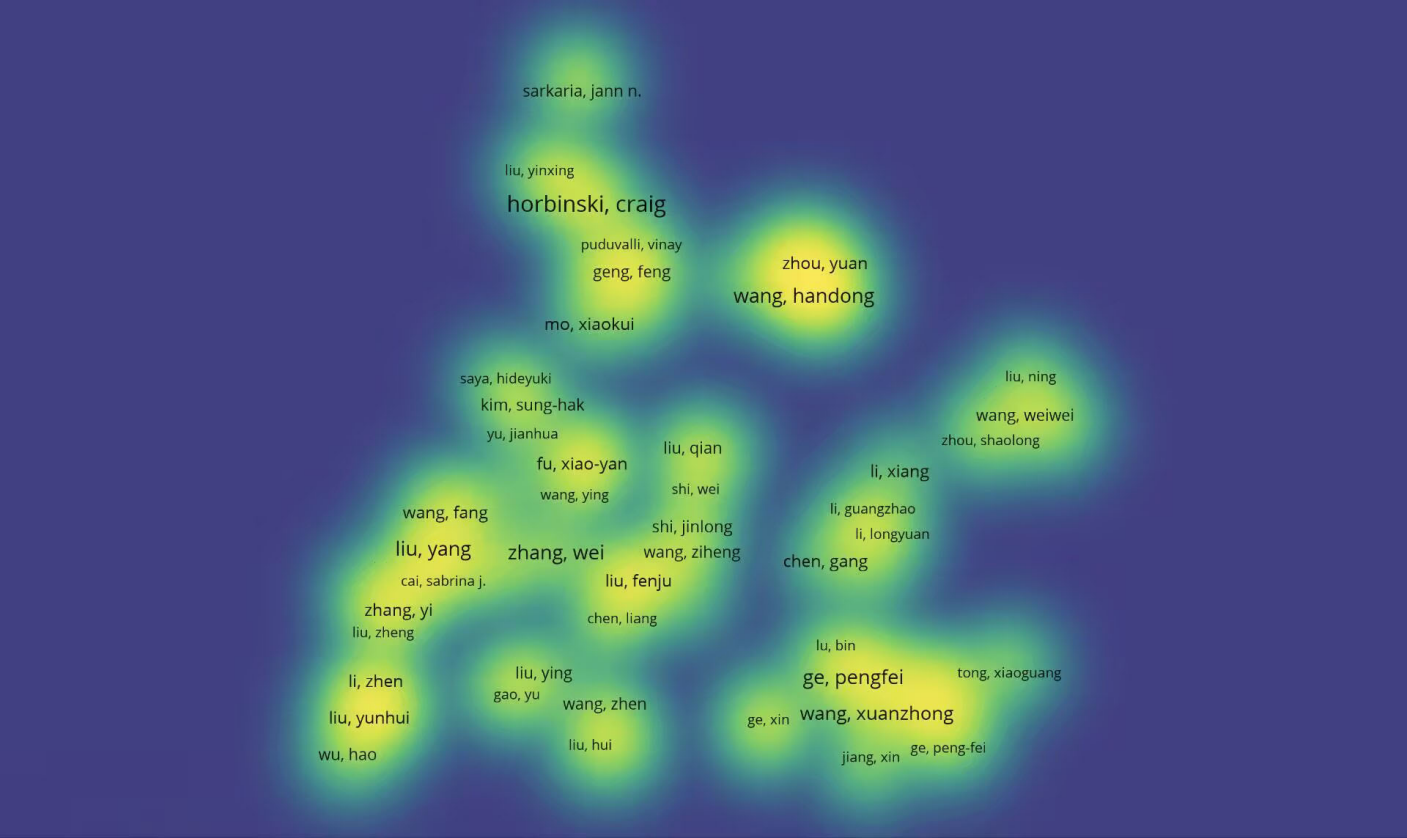
Density map of authors’ co-occurrence of glioma and oxidative stress-related topics.

**Figure 5:**
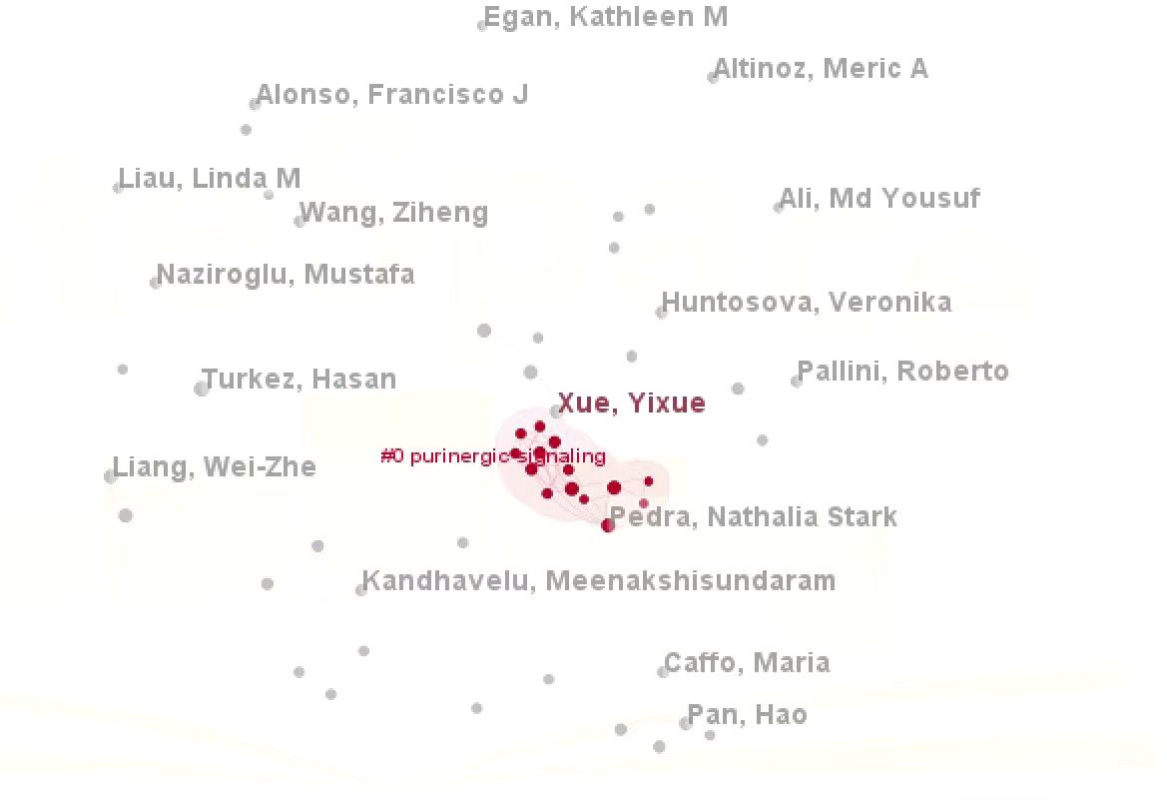
Clustering of authors on topics related to glioma and oxidative stress.

Notably, Ge, P stood out with a total citation count of 359, which was significantly higher than that of all other authors, solidifying their status as the most influential contributor in this field. As can be seen from the density plot of VOSviewer, the area with the brightest color (e.g. yellow) represents the highest co-citation density, in which the authors (e.g. Zhou Y, Wang HD, Wang F, Liu Y, etc.) are at the core of the academic network, suggesting that their research results on glioma and oxidative stress have been widely cited, and have a high impact in this field(**Figure 4**). Complementary analysis using Citespace, where nodes represent author clusters, further illustrated a fragmented network: clusters were dispersed across the map, demonstrating minimal interconnectivity between different groups (**Figure 5**). These findings highlight the need for enhanced interdisciplinary and international collaboration to foster breakthroughs in mechanistic research and therapeutic development for gliomas.

### 3.4 Journal analysis

There were about 396 journals in total and the journal with the highest number of publications was INTERNATIONAL JOURNAL OF MOLECULAR SCIENCES with 43 articles published and the 2023 JOURNAL IMPACT FACTOR is 4.9. The number of citations was 1047 and the average number of citations was 24.35. The next highest number of publications was CANCERS with 28 publications, cited 442 times with an average of 15.79 citations. The impact factor (IF) of journals in 2023 is shown in **Table 4**. The top 10 journals with the highest number of articles published are shown in **Table 4**. The top 10 high-productivity journals are divided equally between Q2 and Q1. In addition, we find that the journal with the highest impact factor is MEDICINE AND CELLULAR.

**Table 4:** Top 10 journals on research of oxidative stress in gliomas.

Rank 4	Journal	Count	Times Cited	Average per item	H-Index	2023 JOURNAL IMPACT FACTOR	JIF QUARTILE
1	INTERNATIONAL JOURNAL OF MOLECULAR SCIENCES	43	1047	24.35	16	4.9	Q1
2	CANCERS	28	442	15.79	14	4.5	Q1
3	FRONTIERS IN ONCOLOGY	21	343	16.33	10	3.5	Q2
4	CELLS	20	320	16	10	5.1	Q2
5	SCIENTIFIC REPORTS	19	362	18.1	11	3.8	Q1
6	OXIDATIVE MEDICINE AND CELLULAR LONGEVITY	19	365	19.21	11	7.31	Q2
7	PLOS ONE	19	636	33.47	14	2.9	Q1
8	FREE RADICAL BIOLOGY AND MEDICINE	18	306	17	11	7.1	Q1
9	ONCOTARGET	16	724	45.25	15	5.168	Q2
10	NEUROCHEMISTRY INTERNATIONAL	14	332	23.71	10	4.4	Q2

### 3.5 Keywords analysis

**Figure 6:**
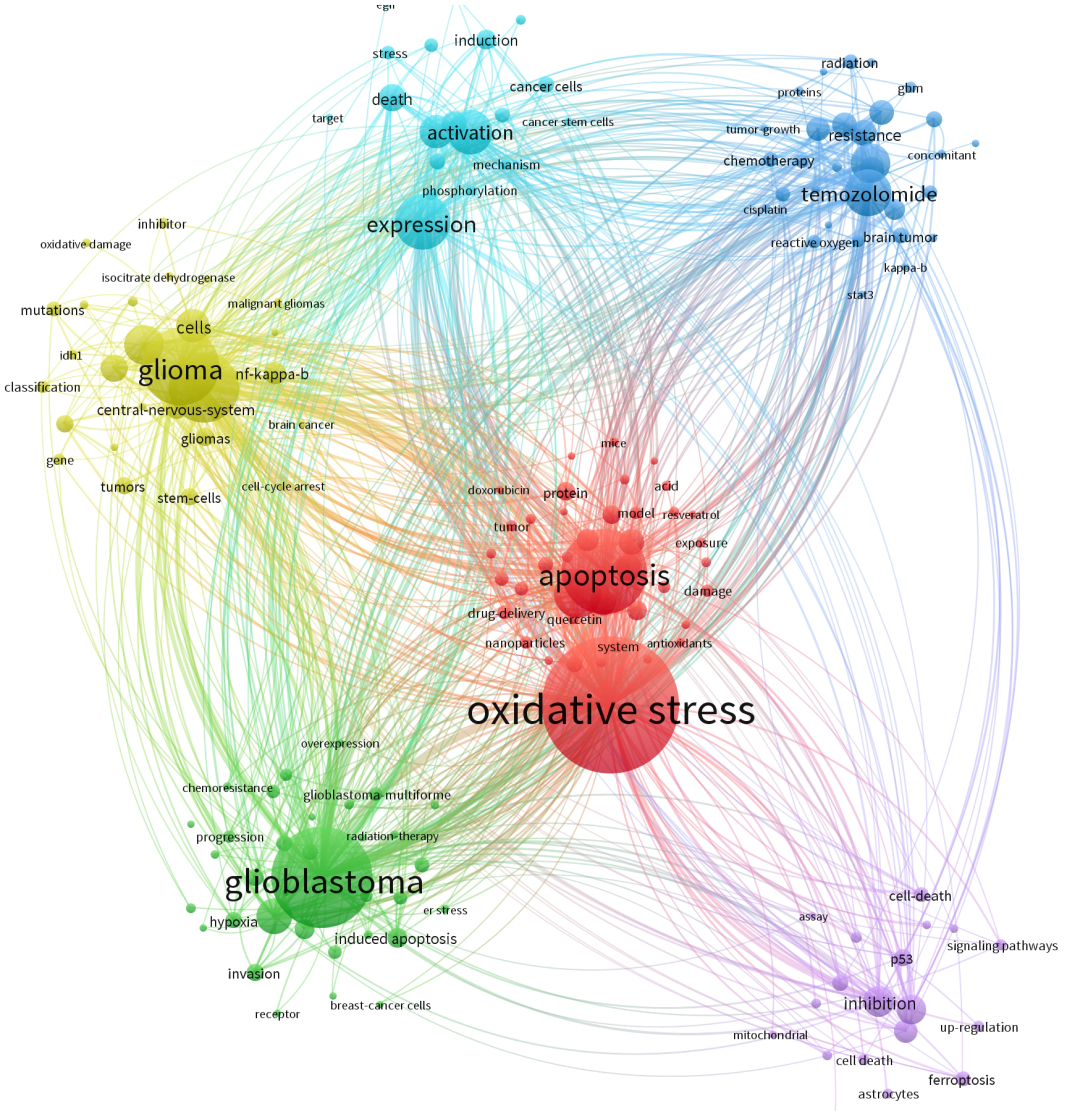
The cluster of keywords on research of oxidative stress.

Keywords typically reflect the thematic focus of scholarly publications. Using VOSviewer, we extracted 4,838 keywords from an initial corpus of 1,020 articles for co-occurrence analysis and clustering, yielding the six-cluster co-occurrence network depicted in **Figure 6**. Circular nodes represent keywords, with size indicating frequency of occurrence, larger nodes denote more representative and frequently appearing hotspots, while connecting lines signify association strength, where thicker lines indicate higher co-occurrence frequency within the same documents. Node colors demarcate distinct clusters corresponding to unique research themes.

The blue cluster focuses on mechanisms of cancer cell activation and target discovery under oxidative stress in glioma, incorporating keywords such as “induction,” “activation,” “expression,” “cancer cells,” and “cancer stem cells.” This theme centers on the activation processes of cancerous and cancer stem cells under oxidative stress conditions, examining molecular mechanisms like phosphorylation and investigating targets linked to cell death to elucidate molecular activity patterns and potential intervention points in glioma cells experiencing oxidative stress. The light-blue cluster, featuring keywords including “radiation,” “tumor growth,” “resistance,” “reactive oxygen,” “kappa-b,” and “signal transducer and activator of transcription 3,” addresses glioma responses to radiotherapy, chemotherapy, and agents like temozolomide within oxidative stress contexts, exploring tumor growth dynamics and resistance mechanisms mediated by these factors.

The purple cluster, characterized by “signaling pathways,” “ferroptosis,” and “inhibition,” is dedicated to uncovering how oxidative stress modulates glioma-related signaling pathways to regulate cell death processes such as ferroptosis. The green cluster revolves around treatment resistance and disease progression in glioblastoma, with core keywords “glioblastoma,” “chemoresistance,” and “progression,” dissecting how oxidative stress influences therapeutic responses and tumor advancement in this aggressive glioma subtype. The yellow cluster emphasizes intrinsic glioma properties, including “glioma,” “mutations,” and “stem cells,” focusing on molecular alterations like genetic mutations and stem cell characteristics that may interact bidirectionally with oxidative stress environments. Lastly, the red cluster, anchored by “oxidative stress” and “apoptosis,” directly addresses the regulatory relationship between oxidative stress and apoptosis, investigating how oxidative stress impacts apoptotic pathways and their functional interplay in glioma biology. Following keyword collation, the ten most frequently occurring keywords are listed in order as: oxidative stress, cancer, apoptosis, expression, cancer cells, *in vitro*, activation, growth, glioblastoma multiforme, and temozolomide. Among these, “cancer cells” exhibits the highest centrality (**Table 5**), underscoring its pivotal role in contextualizing discussions around oxidative stress.

**Table 5:** Top 20 keywords on research of oxidative stress in gliomas.

Rank 5	Count	Centrality	Keywords
1	593	0.01	oxidative stress
2	195	0.03	cancer
3	180	0.02	apoptosis
4	164	0.03	expression
5	129	0.07	cancer cells
6	126	0.02	*in vitro*
7	117	0.03	activation
8	101	0.03	growth
9	84	0.05	glioblastoma multiforme
10	80	0.05	temozolomide

**Figure 7:**
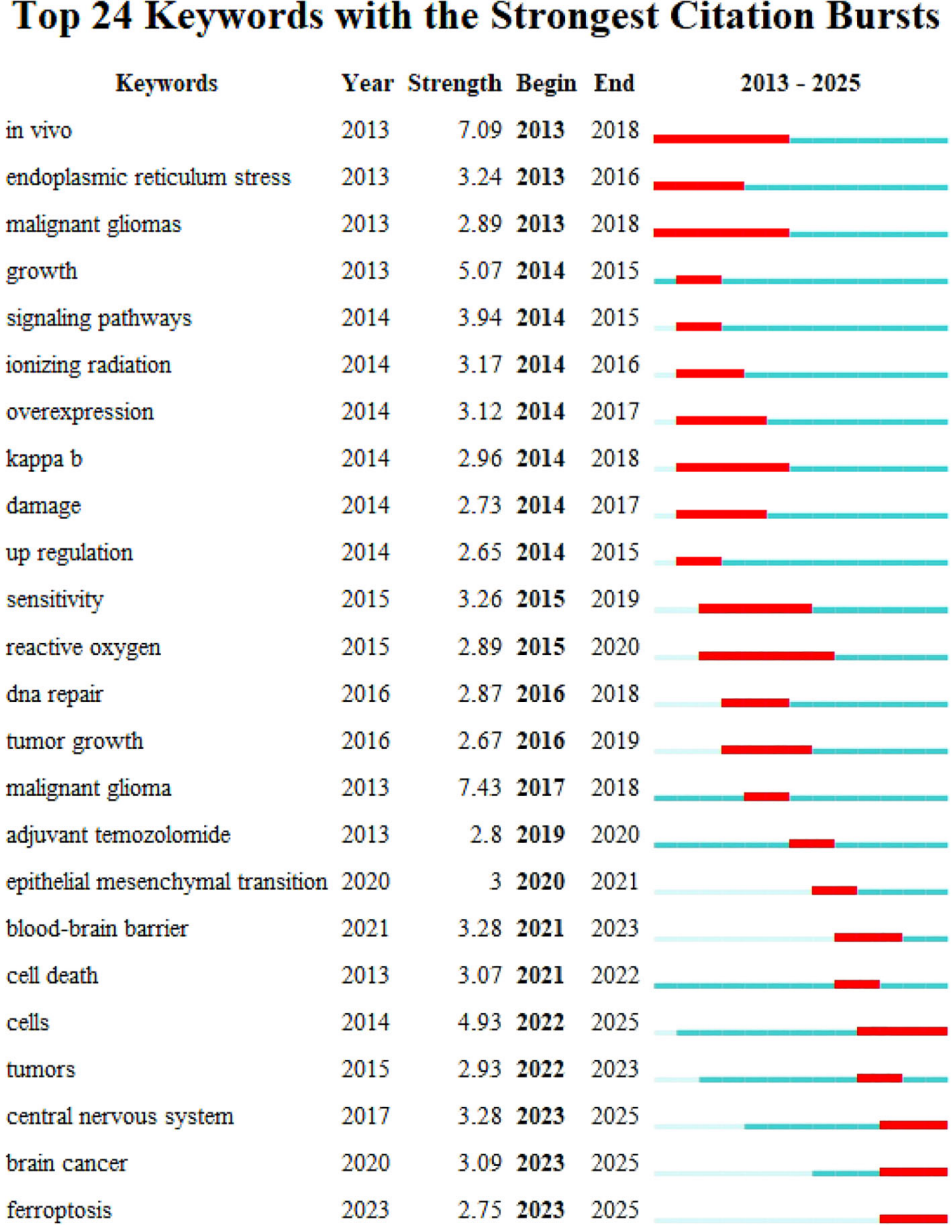
Keyword emergence map for topics related to glioma and oxidative stress: the top 25 keywords with the strongest citation explosion are mainly shown.

**Figure 8:**
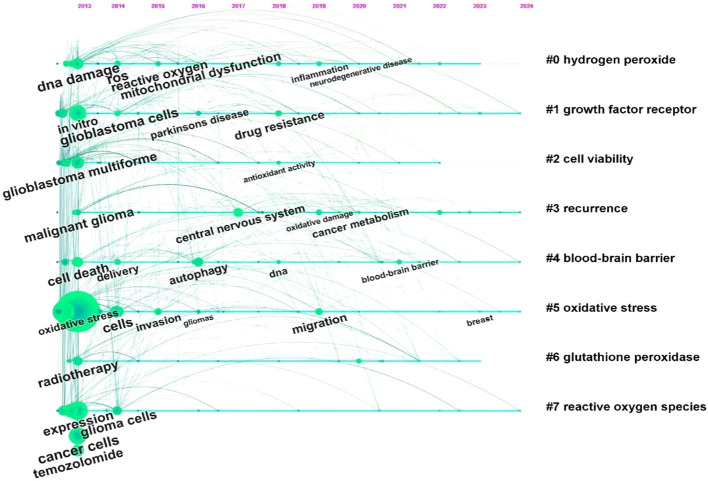
Timeline diagram of topics related to glioma and oxidative stress.

Using CiteSpace, we analyzed the burst strength of keywords and identified the top 25 with significant temporal dynamics (**Figure 7**). The parameters “strength,” “start,” and “end” denote the intensity of the burst, its initiation year, and termination year, respectively, while red highlights the duration of the burst period. Integrating these data with the timeline graph (**Figure 8**) reveals that “inflammation” and “tumor microenvironment” have shown the highest citation burst intensities in recent years, reflecting sustained research momentum. Conversely, “blood brain barrier (BBB)” and “ferroptosis” have emerged as emerging hotspots, indicating rising investigative focus in these areas.

### 3.6 Institution analysis

As shown in **Table 6**, we collected the top 10 institutions from the WOS database. The geographical distribution reveals that four institutions are from the United States, three from China, two from France, and one from Poland. The University of Texas System stands out with the highest citation frequency (1,055 times) and the highest average citations per article (70.33), underscoring its significant influence in the field. The Polish Academy of Sciences has the largest publication output, contributing 17 articles, while Harvard University exhibits the highest centrality score (0.1), indicating its pivotal role in connecting research networks.

**Table 6:** Top 20 institutions on research of oxidative stress in gliomas.

Rank 6	Count	Centrality	Year	Institutions	Times Cited	Average per item	H-Index
1	17	0.01	2019	Polish Academy of Sciences	414	20.7	12
2	16	0.02	2013	Institut National de la Sante et de la Recherche Medicale (Inserm)	658	38.71	12
3	15	0	2013	Jilin University	706	35.3	15
4	13	0.07	2013	University of California System	813	54.2	11
5	12	0.03	2013	Centre National de la Recherche Scientifique (CNRS)	447	31.93	11
6	12	0.1	2016	Harvard University	627	44.79	10
7	12	0	2013	Nanjing University	319	24.54	9
8	11	0.02	2013	Emory University	193	17.55	7
9	11	0.01	2016	University of Texas System	1055	70.33	15
10	10	0.01	2021	Nanjing Medical University	159	11.36	7

**Figure 9:**
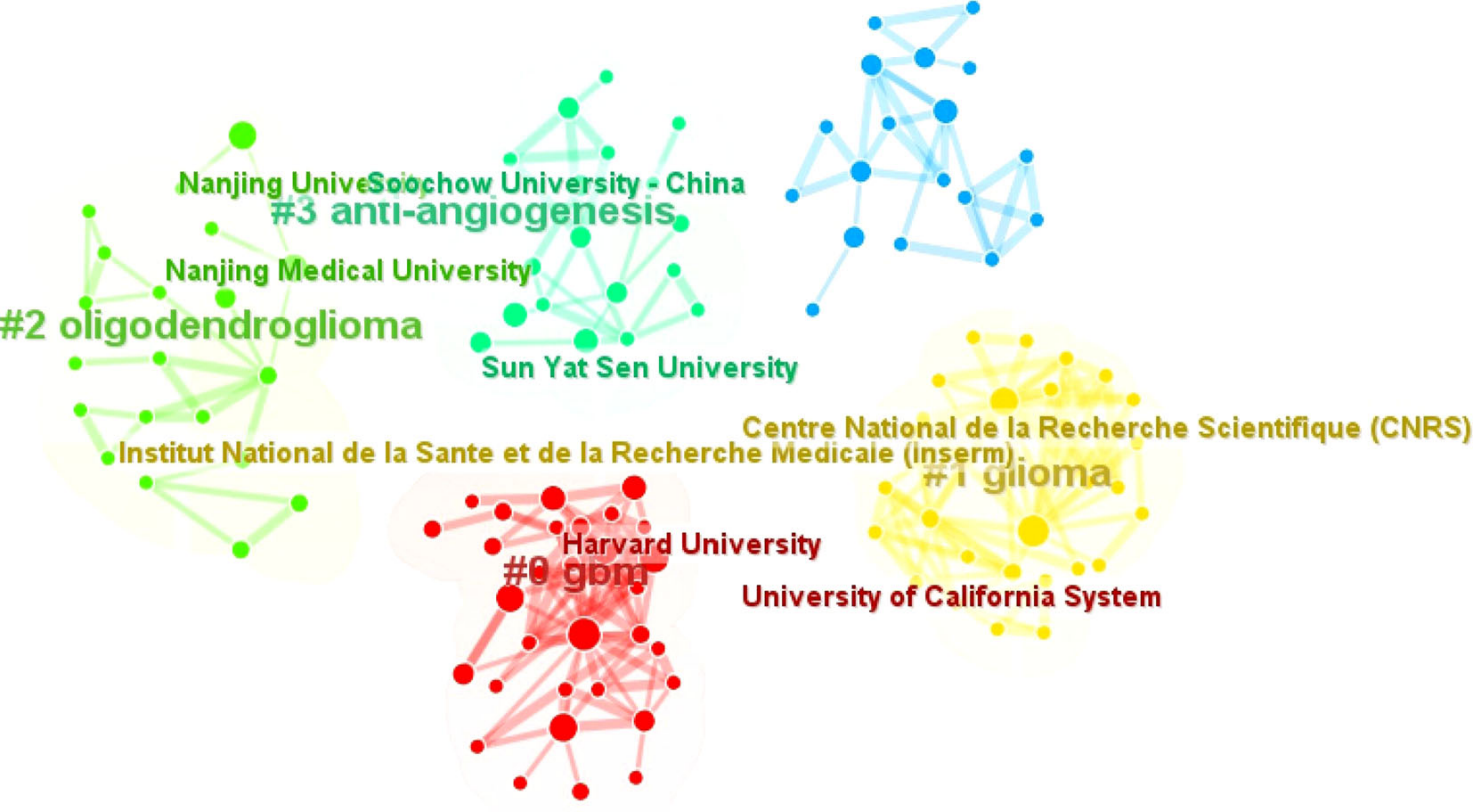
Clustering of institutions associated with glioma and oxidative stress.

Among the three Chinese institutions, Jilin University has the most publications (n=15), reflecting its active research engagement. The CiteSpace visualization classifies these institutions into five thematic clusters based on the thematic trends of their publications, with consistent colors denoting shared research focuses (**Figure 9**). Notably, Harvard University and the University of California System are grouped in the same cluster, with their research primarily centered on the analysis of glioblastoma.

Collectively, these top 10 institutions have contributed 129 articles, making substantial contributions to advancing the field through their research on glioma biology, oxidative stress mechanisms, and translational applications.

## 4 Discussion

To the best of my knowledge, this is the first bibliometric study to systematically characterize research trends at the interface of oxidative stress and gliomas over the 2013–2025 period. As illustrated in **Figure 2**, there has been a consistent upward trend in scholarly publications addressing the relationship between oxidative stress and gliomas. This observed growth can be attributed to both heightened scientific interest in the health implications of oxidative mechanisms and ongoing advancements in optimizing therapeutic strategies for glioma treatment. Through quantitative and visual analyses of 1,020 scholarly documents, our study provides a comprehensive overview of hotspots in this domain, identifying contributions from 71 countries and regions where the USA and China lead in both publication output and collaborative research networks.

Despite advancements in surgical resection, radiotherapy, and chemotherapy for gliomas, the disease remains highly lethal due to incomplete understanding of its molecular pathogenesis and limitations of emerging therapies such as immunotherapy, whose efficacy is substantially constrained by the blood-brain barrier and immunosuppressive tumor microenvironment (16). These unmet clinical needs have spurred increasing interest in oxidative stress pathways as critical therapeutic targets. By analyzing keyword frequencies, burst dynamics, and temporal trends, our study reveals that current research intensively explores the roles of ROS in glioma proliferation, invasion, and resistance to therapy, alongside investigations into redox-modulating agents and their interactions with inflammatory processes.

Building on these insights, future research is poised to focus on deciphering the bidirectional relationship between inflammation and oxidative stress, such as the crosstalk between ROS mediated signaling and inflammatory pathways like NF-κB, in the context of glioma progression, as well as developing oxidative therapies that target both the blood-brain barrier to enhance drug delivery and the tumor microenvironment to exploit its redox vulnerabilities. Additionally, leveraging molecular heterogeneity defined by The Cancer Genome Atlas glioblastoma subtypes (proneural, neural, classical, mesenchymal), studies may aim to design precision interventions that capitalize on subtype-specific redox signatures, such as augmenting ROS levels in mesenchymal glioblastoma cells with elevated antioxidant capacities (17).

To ensure translational relevance, our work integrates genetic factors and environmental influences that modulate redox homeostasis, offering a mechanistic framework for understanding how inflammation and oxidative stress collectively impact glioma treatment responses. By synthesizing bibliometric evidence with molecular insights, this study underscores the centrality of oxidative stress in glioma biology and highlights potential targets for overcoming therapeutic resistance, thereby informing the development of innovative, redox based strategies to improve clinical outcomes for patients with these aggressive brain tumors.

### 4.1 Oxidative stress and tumor proliferation

Mitochondria are the primary sites of ROS production. ROS can be generated through mitochondrial electron transport chains, endogenous pathways (such as cell signaling, metabolic processes, and inflammation), or exogenous pathways (including X-rays, gamma rays, alpha particles, oxidants, or ultraviolet light) (18). Research indicates that elevated ROS concentrations in cancer cells arise from multiple mechanisms: heightened cellular metabolism, mitochondrial dysfunction induced by hypoxia or autophagy, peroxisome activation, dysregulation of growth factor mediated signaling pathways, oncogene activation, and increased activity of ROS-generating enzymes like xanthine oxidase and peroxidases. In contrast, normal cells maintain redox homeostasis through a sophisticated antioxidant system, which is critical for preventing oxidative damage (19, 20).

For example, in the central nervous system, the glutathione system composed of reduced and oxidized forms of glutathione plays a crucial role in protecting astrocytes and neurons from oxidative damage (21). However, when levels of hydrogen peroxide and other ROS increase, glioblastoma cells respond robustly by activating antioxidant defense mechanisms (22). Research indicates that under conditions of elevated ROS levels, glioma cells invoke antioxidant defense mechanisms including manganese superoxide dismutase (MnSOD), catalase, and glutathione peroxidase (GPx) to combat oxidative stress and ensure cell survival (23, 24). MnSOD, located within mitochondria, catalyzes the breakdown of superoxide radicals into oxygen and hydrogen peroxide, thereby alleviating oxidative damage at the mitochondrial level (25). Catalase, predominantly found in peroxisomes, effectively decomposes hydrogen peroxide into water and oxygen, thereby inhibiting the spread of ROS-induced cell damage (26). GPx, a selenium-dependent enzyme, clears lipid hydroperoxides and H2O2 while utilizing reduced glutathione (GSH) as a cofactor, thereby enhancing cellular defenses against oxidative damage (27). This coordinated activation of MnSOD, catalase, and GPx enables glioblastoma cells to tolerate high ROS microenvironments, facilitating survival in metabolically stressed or inflamed tumor niches. Concurrently, oxidative stress influences glioma malignancy through multifaceted mechanisms: inhibiting apoptosis, modulating tumor-associated gene expression (e.g., redox-sensitive transcription factors like Nrf2 and HIF-1α), and perturbing energy metabolism—processes that collectively enhance invasive and metastatic potential (28).

In the pathogenesis of gliomas, the inhibition of apoptosis is a defining feature that promotes tumor cell survival, a trait markedly distinct from normal cellular homeostasis (8). Malignant transformation involves dysregulation of apoptotic signaling pathways, enabling tumor cells to evade both intrinsic and extrinsic programmed cell death stimuli (29), which underlies the uncontrolled proliferation and invasive phenotype of glioblastomas. A key resistance mechanism involves disruption of both death receptor–mediated extrinsic and mitochondria-driven intrinsic apoptotic pathways (30). Within the extrinsic pathway, the tumor necrosis factor receptor superfamily member Fas (Apol/CD95) is central to apoptotic regulation (31). Its trimeric ligand, Fas ligand, targets cytotoxic T lymphocytes and mediates apoptosis by binding to Fas receptors on target cells (32). Dysregulation of the Fas-Fas ligand axis in gliomas is recognized as a mechanism that promotes tumor cell survival by blocking apoptotic signaling pathways. Furthermore, mitochondria serve as key initiators of the intrinsic apoptotic pathway under conditions such as DNA damage, hypoxia, and metabolic disturbances (33). In this cascade, the Bcl-2 protein family plays a crucial regulatory role by modulating mitochondrial membrane permeability and controlling the release of apoptotic factors (34). Notably, oxidative stress is a hallmark feature of the glioma microenvironment. During oxidative stress, ROS inhibit pro-apoptotic proteins (such as p53, Bcl-2, and caspase-9), thereby intervening in apoptotic regulation (35). This inhibition is mediated through activation of the phosphoinositide 3-kinase (PI3K) and Akt signaling pathways, collectively hindering apoptosis in tumor cells and granting survival advantage under adverse conditions (36). The intricate interactions among apoptotic regulatory mechanisms underscore the formidable challenge of combating gliomas, emphasizing the necessity for targeted interventions aimed at restoring apoptotic competence in tumor cells.

Notably, ROS-induced apoptosis may have a negative impact on health. Therefore, a new technology - using pyroelectric sensors made of polyvinylidene fluoride (37)- has been proposed, which can indirectly assess the health status of cells by detecting changes in their thermal properties (e.g., thermal resistance and heat capacity). For example, oxidative stress may lead to changes in cell membrane structure, protein denaturation, or metabolic changes that affect thermal conductivity and heat capacity (38, 39). If this thermoelectric analysis method is used before and after treatment, changes in these parameters can be monitored and thus the effectiveness of the treatment can be assessed. This technique, with its high sensitivity, low sample requirement and dynamic response (40), has promising applications in both basic research and clinical translation of oxidative stress for gliomas (40).

**Figure 10:**
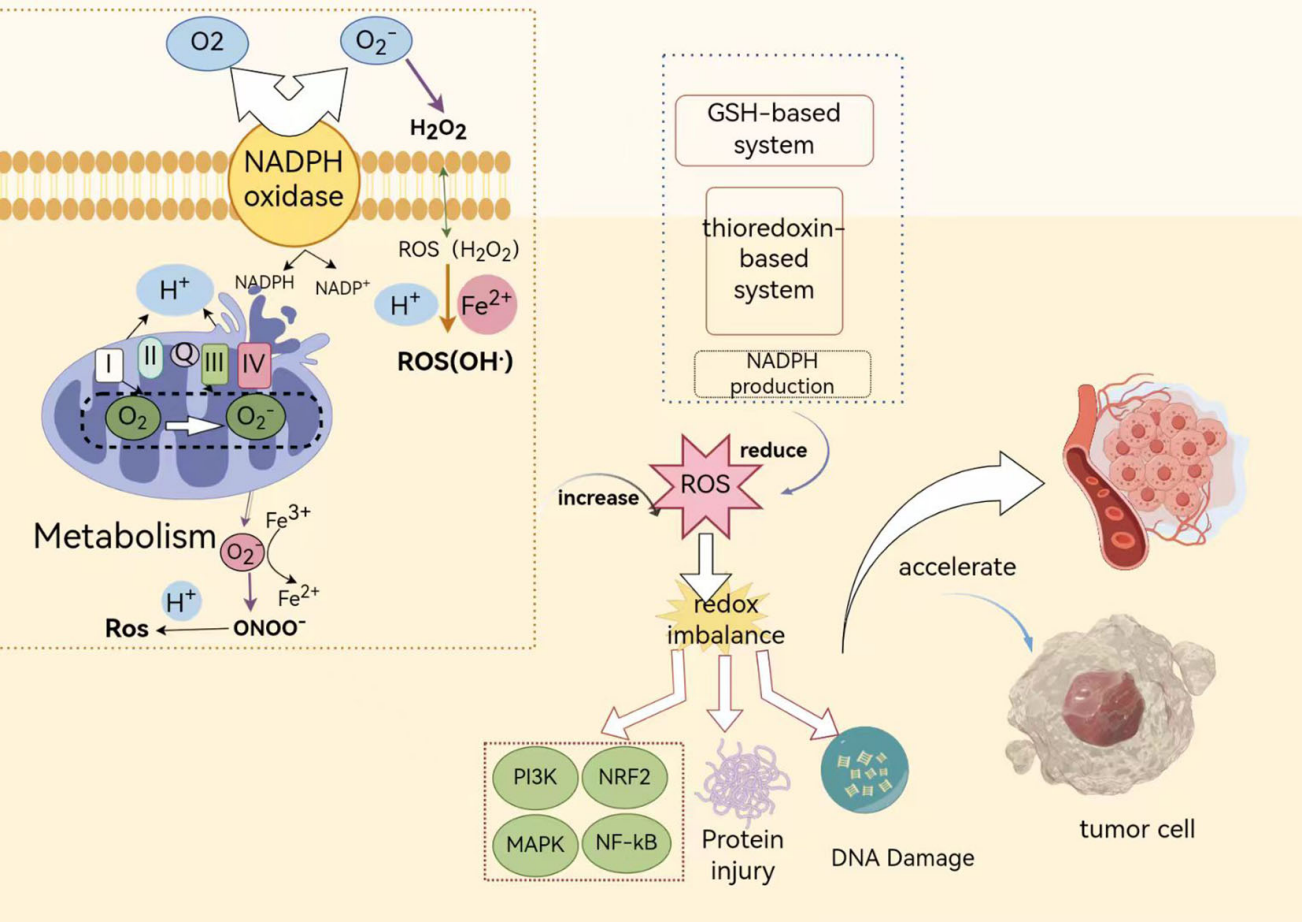
Illustrates the mechanisms by which oxidative stress regulates tumor development and progression. In this figure, the green boxes encompass the pathways of ROS production inside and outside the cell, while the blue dashed boxes represent pathways that antagonize ROS production. PI3K, Phosphoinositide 3-kinase; Nrf2, Nuclear factor erythroid 2-related factor 2; MAPK, Mitogen-activated protein kinase; NF-κB, Nuclear factor kappa B; GSH-based system: A biological system based on GSH; Thioredoxin-based system: A biological system centered around thioredoxin.

The transition from normal cellular physiology to malignant tumors triggers a series of events, including activation of the body’s immune response and subsequent inflammatory processes (41). Following cell transformation, various inflammatory cells, including macrophages and neutrophils, are recruited, creating an environment rich in ROS (16). These ROS act as critical secondary messengers, coordinating the activation of key signaling pathways such as mitogen-activated protein kinase, Ras-extracellular signal-regulated kinase, and activator protein-1 pathways. Collectively, these pathways profoundly impact the proliferation of glioblastoma cells and the dynamics of the cell cycle (**Figure 10**).

### 4.2 Oxidative stress and inflammatory responses

Persistent oxidative stress is an intrinsic mechanism triggering chronic inflammation, which in turn can mediate various related diseases such as cancer, diabetes, cardiovascular diseases, and neurological disorders. During the inflammatory process, mast cells and leukocytes are recruited to the damaged site, leading to increased release and accumulation of ROS due to enhanced oxygen uptake, resulting in a “respiratory burst” (42). Therefore, ROS not only serve as signaling molecules but also play a critical role as mediators in inflammation, where the generation of ROS and reactive nitrogen species drives the inflammatory process (43). In the treatment of GBM, induced inflammation promotes tumor cell apoptosis through ROS production. Thus, the intricate interactions between ROS, oxidative stress pathways, and inflammation significantly influence treatment resistance (44).

Two main inflammatory pathways, the NF-κB signaling pathway and the Nrf-2 pathway, significantly impact the relationship between oxidative stress and tumors (45). Numerous studies indicate that the NF-κB signaling pathway can promote tumor development. The basic NF-κB signaling pathway comprises receptor and receptor-proximal signal adapter proteins, IκB kinase complexes, IκB proteins, and NF-κB dimers. Upon various endogenous and exogenous stimuli, IκB kinase is activated, leading to the phosphorylation and ubiquitination of IκB proteins, followed by their degradation and the release of NF-κB dimers (46). These dimers translocate into the nucleus, producing pro-inflammatory cytokines (such as IL-6), inducible nitric oxide synthase (iNOS or NOS2), and cyclooxygenase-2 (COX-2) (47, 48), thereby promoting inflammation and signaling survival cues for tumors in a carcinogenic environment. Additionally, oxidative stress, inflammation, and other factors can activate the Nrf-2 pathway. The Nrf-2 pathway plays a dual role in tumorigenesis: it can prevent chemically induced carcinogenesis by activating detoxifying enzymes, yet its overexpression is associated with tumor development and drug resistance. In the treatment of GBM, Nrf-2 acts as a critical transcription factor regulating antioxidant responses to maintain redox balance, crucial in mitigating DNA damage induction and the effects of carcinogens (49).

**Figure 11:**
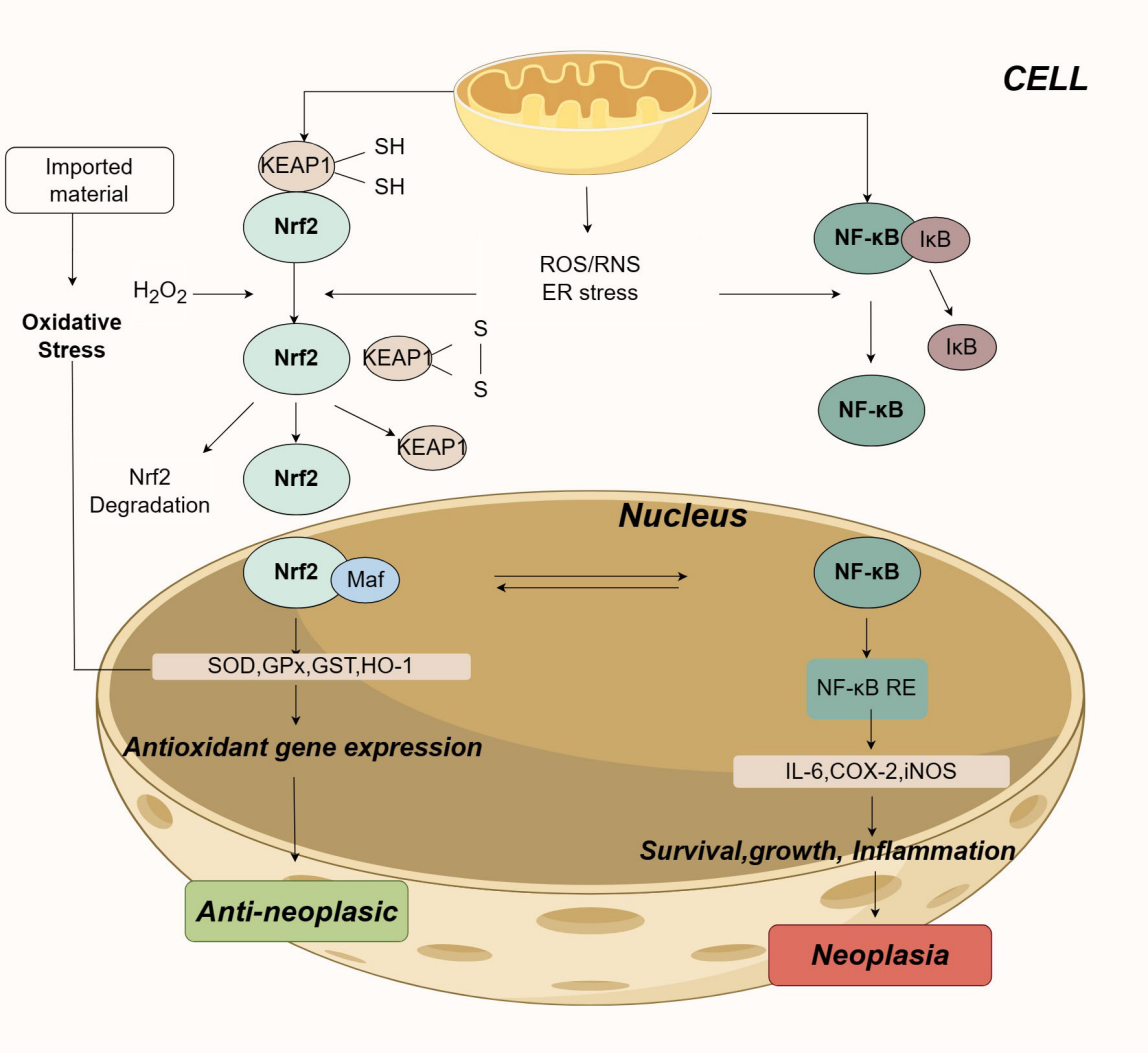
Two pathways affecting the relationship between oxidative stress and tumors: the NF-κB signaling pathway and the Nrf-2 pathway.

Under oxidative stress conditions, Nrf-2 rapidly dissociates from the Keap1 complex and translocates to the nucleus (50). Within the nucleus, it forms heterodimers with Maf proteins, promoting the transcription of phase II detoxifying enzymes and antioxidant proteins such as SOD, GPx, GST, and HO-1, thereby serving as a defense mechanism against oxidative damage and exerting an anti-tumor effect. However, due to its dual role, increased levels of Nrf-2 can also promote tumor cell survival. Fan et al. reported that increased expression of Nrf-2 in gliomas, coupled with decreased expression of Keap1, significantly accelerates cell proliferation, promotes tumor formation, and impedes programmed cell death (51) (**Figure 11**).

However, due to genetic and environmental differences in the oxidative stress response, the therapeutic effects may vary in different individuals. Bigner et al. (1988) found that high expression of CYBB (NADPH oxidase subunit) was significantly correlated with copy number gain of chromosome 7, suggesting that it may be the genetic basis for oxidative stress dysregulation in mesenchymal glioblastoma (52). Chromosome 7 gain was accompanied by overexpression of oncogenes such as EGFR and MEOX2 and enhanced mitochondrial antioxidant capacity through activation of the CYBB/Nrf2/SOD2 axis, thereby mediating chemoresistance (53, 54). In addition, radiotherapy exacerbated DNA damage by increasing mitochondrial ROS levels, whereas glioma cells with high CYBB expression inhibited ROS accumulation by activating the Nrf2/SOD2 pathway, thereby enhancing resistance to radiotherapy (55).

### 4.3 Active oxygen-based anticancer drugs

In recent years, researchers have proposed targeting the upregulation of ROS to induce mitochondrial release of apoptotic factors. Additionally, NOX are proteins involved in electron transfer across biological membranes, crucial for various physiological processes but implicated in diseases such as tumors, diabetes, hypertension, and heart disease when overexpressed (56). Zhu Daqian’s team suggested using NOX inhibitors to suppress H_2_O_2_ and O_2_-mediated tumor cell proliferation (57). Boyi Niu et al. proposed enhancing ROS levels by depleting GSH for cancer treatment. Elevated GSH levels in cancer cells can scavenge excessive ROS, promoting tumorigenesis. Using GSH inhibitors enhances oxidative stress responses, thus inducing tumor cell apoptosis (58).

## 5 Future trend

### 5.1 Tumor microenvironment dynamics -based therapies

Gliomas feature a unique microenvironment including hypoxia, acidic pH, and oxidative stress. Within the tumor microenvironment (TME), glioma-associated microglia/macrophages play a crucial role. However, tumor-released anti-inflammatory factors promote M2 polarization of macrophages. This shift stimulates the release of growth factors like vascular endothelial growth factor and promotes tumor proliferation, invasion, and metastasis (59, 60). In the TME, ROS is generated through various pathways, including the mitochondrial electron transport chain, Nox transmembrane protein catalysis, and the Fenton reaction. Studies indicate that overexpression of the mitochondrial membrane protein Romo1 enhances ROS production via mTORC1 signaling, significantly inhibiting the TME. Furthermore, a prognosis model assessing GBM and ROS levels found that high expression of ROS related genes, such as HSPB1, LSP1, and PTX3, correlates not only with the presence of M2 macrophages but also with shortened survival in GBM patients (61).

These findings underscore the potential of ROS related genes as therapeutic targets for GBM. Strategies aimed at inhibiting M2 macrophage polarization may thus benefit GBM treatment. Additionally, myeloid derived suppressor cells (MDSCs) constitute a significant proportion (30%-50%) in the solid glioma, exerting profound immunosuppressive effects in the TME (62). MDSCs inhibit T cell activation and promote tumor immune evasion through upregulation of ARG-1 expression, secretion of immunosuppressive factors, and expression of inhibitory ligands (63). Moreover, MDSCs enhance angiogenesis by releasing vascular endothelial growth factors and IL-10, thus facilitating tumor growth and invasion (64). MDSCs thrive in environments rich in ROS, with elevated Nrf-2 expression promoting their survival. ROS also maintains MDSCs in an undifferentiated state, sustaining expression of immunosuppressive molecules (65). Targeting MDSCs represents a promising strategy for glioma therapy.

### 5.2 BBB based therapies

The BBB consists of endothelial cells, astrocyte end-feet, and pericytes, collectively protecting the brain from systemic circulation, shielding it from harmful substances, and regulating transport of crucial molecules to maintain a stable microenvironment (66). However, the BBB hinders intravenous administration, with chemotherapy drugs achieving penetration rates in the brain of only 1-2%. This results in extensive dispersion of drugs into healthy tissues and organs, causing severe toxicity. (67) Temozolomide, a first line alkylating agent for gliomas, represents a notable exception due to its near-100% BBB permeability and broad antitumor activity (68). It induces DNA methylation mediated strand breaks, thereby inhibiting tumor cell proliferation, arresting the cell cycle, and promoting apoptosis mechanisms that have been clinically validated to slow glioblastoma progression and extend patient survival.

In recent years, scientists have proposed integrating nanotechnology into the BBB as a novel treatment strategy. Advances in nanotechnology have facilitated the production of nanoparticles capable of crossing or bypassing the BBB, revolutionizing glioma therapy. Surface modification of nanoparticles with BBB targeting peptides or cell penetrating peptides enhances their penetration across the BBB, facilitating precise targeted therapy for tumor cells. Researchers have pioneered ROS based nanomedicine technologies for cancer treatment, including photodynamic therapy, photothermal therapy, chemotherapy, sonodynamic therapy, radiotherapy, and other modalities (57, 69). These methods utilize nano sensitizers and exogenous/endogenous stimuli such as light, ultrasound, X-rays, and hydrogen peroxide to induce ROS production in tumor cells. The disruption of redox balance has been shown to effectively eliminate tumor cells (70).

By comparing nanocarriers such as NanoTherm^®^, NU-0129, Onyvide^®^ (polyethylene glycolated irinotecan liposomes), and Caelyx^®^ (polyethylene glycolated adriamycin liposomes), which are currently being used in clinical trials for patients with recurrent GBM, academic Juan Aparicio Blanco found that NanoTherm^®^, administered via intratumoral delivery and combined with alternating magnetic field thermal ablation, resulted in a median overall survival of 13.4 months, which was significantly better than that of the historical control group of 6.2 months. The technology received CE Mark approval as a medical device for GBM treatment in 2013, a strong indication that nanotechnology has made great strides in the treatment of gliomas (71).

### 5.3 Active oxygen-based anti-inflammatory therapies

Inflammation regulated by NF-κB and Nrf-2 signaling pathways is a pivotal contributor to carcinogenesis and disease progression, driving growing interest in antioxidants as potential cancer therapeutics. These molecules mitigate cancer risk by scavenging free radicals, with molecular hydrogen (H_2_), a novel antioxidant notable for its small molecular size and high bioavailability, exhibiting unique intracellular interactions that exert antioxidant, anti-inflammatory, and anti-apoptotic effects (72). Preclinical and clinical evidence highlights H_2_ as a promising adjunct for GBM therapy, demonstrating tumor growth inhibition through dual mechanisms: direct ROS scavenging and modulation of gene transcription involved in cell survival and proliferation, which may extend patient survival (73). Elevated ROS levels are associated with disease progression and drug resistance, highlighting their importance as therapeutic targets. Under specific conditions, H_2_ and various H_2_ donors effectively reduce oxidative stress and exhibit anti-inflammatory properties, offering a new treatment strategy for cancer therapy. Administration methods for H_2_ include inhalation, drinking hydrogen-rich water, or injection of hydrogen saline (73, 74). Despite the therapeutic potential of anti-inflammatory agents, their clinical application is hindered by significant limitations. For instance, cyclooxygenase-2 inhibitors have demonstrated efficacy in preclinical and clinical studies for cancer treatment, yet their use is associated with an elevated risk of cardiovascular events. To address this, strategies such as intermittent dosing of celecoxib are being explored to determine whether this approach can maintain antitumor activity while reducing cardiotoxicity. As a precaution, these drugs are contraindicated in patients with a history of or high risk for cardiovascular disease (75).

### 5.4 Oxidative stress detection based on temperature/SAR monitoring techniques

The non thermal biological effects of electromagnetic radiation (EMR), such as oxidative stress induction, represent a critical frontier in current research. Existing literature indicates that prolonged exposure to electromagnetic fields (EMFs) emitted by wireless networks may disrupt cellular homeostasis via oxidative stress pathways, despite radiation intensities under established safety guidelines generally falling below thermal threshold levels (76). To quantify such risks, the present study developed a real-time monitoring system integrating synthetic aperture radar (SAR) and infrared thermometry sensors to dynamically track energy absorption profiles and temperature changes (ΔT) in human tissues during electromagnetic exposure.

Elevated temperatures, even minor increases of 1–2°C, can induce free radical generation through thermally activated biochemical reactions, while localized SAR spikes from high-frequency EMFs (e.g., 27 GHz) may interfere with mitochondrial electron transport chain function, exacerbating oxidative damage. For instance, the localized heating induced by high-frequency electromagnetic waves has been shown to disrupt mitochondrial ETC complexes, leading to increased ROS production. Glioma cells, characterized by high metabolic activity, exhibit heightened sensitivity to such oxidative perturbations, making them particularly vulnerable to EMR-induced redox imbalance. The developed system provides quantitative SAR/ΔT correlation data, offering real-time exposure metrics to support research on oxidative stress biomarkers (e.g., ROS, 8-OHdG) and their clinical relevance in EMR-associated pathologies (77).

### 5.5 Oxidative stress treatment based on iron death

In recent years, investigations into the regulatory mechanisms of ferroptosis have yielded novel targeting strategies for glioma treatment. Studies demonstrate that dihydroartemisinin (DHA) exerts anti-glioma effects by inducing iron-dependent oxidative stress, characterized by ROS accumulation and lipid peroxidation (78). However, this process concurrently triggers endoplasmic reticulum stress and activates the PERK-ATF4-HSPA5 signaling axis, leading to compensatory upregulation of glutathione peroxidase 4 (GPX4)—an enzyme that neutralizes lipid peroxides and attenuates ferroptotic cell death (79). Conversely, genetic or pharmacological inhibition of HSPA5 disrupts this compensatory pathway, significantly enhancing DHA-induced tumor cell demise (80). On the mechanistic front, activation of SIRT1 promotes ATF3 nuclear translocation via a NAD^+^-dependent mechanism, which in turn suppresses the expression of SLC7A11 and GPX4. This leads to impaired cystine uptake, glutathione depletion, and iron overload, ultimately potentiating RSL3-induced lipid peroxidation–driven ferroptosis (81). Collectively, these findings reveal the bidirectional plasticity of the ferroptosis regulatory network: targeted inhibition of pro-survival compensatory pathways (e.g., the PERK-ATF4-HSPA5 axis) or activation of pro-death signaling (e.g., the SIRT1-ATF3 axis) can overwhelm tumor cell antioxidant defenses by amplifying oxidative stress, offering a dual intervention strategy of “enhancing pro-death signals” and “blocking negative feedback” to optimize ferroptosis-based glioma therapies.

## 6 Limitations and expectations

This study is subject to several limitations stemming from objective factors. Firstly, the literature included in this study was sourced exclusively from the Web of Science database, potentially leading to the omission of relevant publications. Furthermore, the dynamic nature of the Web of Science database, continually updated for bibliometric analyses, may result in findings that lag behind current research progress. Future research could draw on well-established physical-mathematical modelling ideas in fields such as environmental engineering to further advance the precision of oxidative stress treatments (82).

To address these gaps, future studies could adopt a multi-database approach (incorporating platforms like PubMed, Scopus, and Embase) to minimize selection bias and enhance data diversity. Moreover, integrating well-established physical-mathematical modeling frameworks from disciplines such as environmental engineering and systems biology—including kinetic simulations of redox signaling pathways or computational models of tumor microenvironment interactions—holds promise for advancing the precision of oxidative stress-targeted therapies. Such interdisciplinary methodologies may help identify context-specific therapeutic windows and patient-specific redox signatures, thereby facilitating the development of personalized interventions that account for interindividual variability in treatment response.

## 7 Conclusion

Through a comprehensive bibliometric analysis, this study systematically examined 1,020 academic articles on oxidative stress in glioma, identifying research hotspots and emerging trends in this interdisciplinary field. By synthesizing data from measurement tools, we characterized key thematic clusters centered around terms such as “inflammation,” “oxidative stress,” “glioma,” “apoptosis,” “glioblastoma,” and “neuroglioma” — these terms dominate the current research landscape, reflecting a sustained interest in the molecular interactions between redox imbalance and glioma pathogenesis.

Future research trajectories are anticipated to focus on developing precise therapeutic approaches, leveraging four interconnected axes: anti - inflammatory interventions to disrupt pro -tumoral immune microenvironments, BBB targeted drug delivery systems to enhance treatment of brain tumors, ferroptosis, and TME modulation strategies that exploit the dependence of glioma on oxidative stress adaptation. These trends highlight a shift toward translational approaches, which integrate mechanistic insights into redox signaling with clinical needs, such as overcoming BBB impermeability and reversing immunosuppression.
