# Supplementary material for: Recent status and trends regarding oxidative stress in gliomas (2013 - 2025): a systematic review and bibliometric analysis
Source: Front Oncol. 2025 May 16;15:1586515. doi: 10.3389/fonc.2025.1586515 (PMC12122519; doi:10.3389/fonc.2025.1586515)
Supplement: Supplementary file 1 [file Table1.docx]

Search strategy

(TI=("Glioma*") OR AK=("Glioma*") OR TI=("Glial Cell Tumor*") OR AK=("Glial Cell Tumor*") OR TI=("Mixed Glioma*") OR AK=("Mixed Glioma*") OR TI=( "Malignant Glioma*") OR AK=( "Malignant Glioma*") OR TI=(''high grade glioma'') OR AK=(''high grade glioma'') OR TI=(''glioblastoma'') OR AK=(''glioblastoma'') ) AND TS=("Oxidative Stress*" OR "Antioxidative Stress*" OR "Oxidative Damage*" OR "Oxidative Stress Injur*" OR "Oxidative Injur*" OR " Oxidative Cleavage*" OR "Oxidative DNA Damage*" OR " Oxidative Nitrative Stress*" OR "Nitro-Oxidative Stress*")
