# Supplementary material for: Recent status and trends regarding oxidative stress in gliomas (2013 - 2025): a systematic review and bibliometric analysis
Source: Front Oncol. 2025 May 16;15:1586515. doi: 10.3389/fonc.2025.1586515 (PMC12122519; doi:10.3389/fonc.2025.1586515)
Supplement: Supplementary file 2 [file Table2.docx]

FN Clarivate Analytics Web of Science

VR 1.0

PT J

AU Bastian, PE

Daca, A

Ploska, A

Kuban-Jankowska, A

Kalinowski, L

Gorska-Ponikowska, M

AF Bastian, Paulina Emilia

Daca, Agnieszka

Ploska, Agata

Kuban-Jankowska, Alicja

Kalinowski, Leszek

Gorska-Ponikowska, Magdalena

TI 2-Methoxyestradiol Damages DNA in Glioblastoma Cells by Regulating nNOS

and Heat Shock Proteins

SO ANTIOXIDANTS

LA English

DT Article

DE 2-methoxyestradiol; glioblastoma; oxidative stress; reactive nitrogen

species; nitric oxide synthase; heat shock protein

ID NITRIC-OXIDE SYNTHASE; ENDOGENOUS ESTROGEN METABOLITE; GLIOMA-CELLS;

MITOCHONDRIAL DYSFUNCTION; OXIDATIVE STRESS; CANCER; APOPTOSIS; HSP70;

TUMORS; HEAT-SHOCK-PROTEIN-90

AB Gliomas are the most prevalent primary tumors of the central nervous system (CNS), accounting for over fifty percent of all primary intracranial neoplasms. Glioblastoma (GBM) is the most prevalent form of malignant glioma and is often incurable. The main distinguishing trait of GBM is the presence of hypoxic regions accompanied by enhanced angiogenesis. 2-Methoxyestradiol (2-ME) is a well-established antiangiogenic and antiproliferative drug. In current clinical studies, 2-ME, known as Panzem, was examined for breast, ovarian, prostate, and multiple myeloma. The SW1088 grade III glioma cell line was treated with pharmacological and physiological doses of 2-ME. The induction of apoptosis and necrosis, oxidative stress, cell cycle arrest, and mitochondrial membrane potential were established by flow cytometry. Confocal microscopy was used to detect DNA damage. The Western blot technique determined the level of nitric oxide synthase and heat shock proteins. Here, for the first time, 2-ME is shown to induce nitro-oxidative stress with the concomitant modulation of heat shock proteins (HSPs) in the SW1088 grade III glioma cell line. Crucial therapeutic strategies for GMB should address both cell proliferation and angiogenesis, and due to the above, 2-ME seems to be a perfect candidate for GBM therapy.

C1 [Bastian, Paulina Emilia; Kuban-Jankowska, Alicja; Gorska-Ponikowska, Magdalena] Med Univ Gdansk, Dept Med Chem, PL-80210 Gdansk, Poland.

[Daca, Agnieszka] Med Univ Gdansk, Dept Pathol & Expt Rheumatol, PL-80210 Gdansk, Poland.

[Ploska, Agata; Kalinowski, Leszek] Med Univ Gdansk, Fac Pharm, Dept Med Lab Diagnost Fahrenheit Biobank BBMRI Pl, PL-80211 Gdansk, Poland.

[Kalinowski, Leszek] Gdansk Univ Technol, BioTechMed Ctr, Dept Mech Mat & Struct, Narutowicza St 11-12, PL-80233 Gdansk, Poland.

[Gorska-Ponikowska, Magdalena] Univ Stuttgart, Inst Biomat & Biomol Syst, Dept Biophys, D-70569 Stuttgart, Germany.

[Gorska-Ponikowska, Magdalena] Euromediterranean Inst Sci & Technol, I-90139 Palermo, Italy.

C3 Fahrenheit Universities; Medical University Gdansk; Fahrenheit

Universities; Medical University Gdansk; Fahrenheit Universities;

Medical University Gdansk; Fahrenheit Universities; Gdansk University of

Technology; University of Stuttgart

RP Bastian, PE; Gorska-Ponikowska, M (corresponding author), Med Univ Gdansk, Dept Med Chem, PL-80210 Gdansk, Poland.; Gorska-Ponikowska, M (corresponding author), Univ Stuttgart, Inst Biomat & Biomol Syst, Dept Biophys, D-70569 Stuttgart, Germany.; Gorska-Ponikowska, M (corresponding author), Euromediterranean Inst Sci & Technol, I-90139 Palermo, Italy.

EM paulina.bastian@gumed.edu.pl; magdalena.gorska-ponikowska@gumed.edu.pl

RI Kuban-Jankowska, Alicja/LIG-4327-2024; Daca, Agnieszka/AAW-8557-2020;

Kalinowski, Leszek/N-3076-2019

OI Bastian, Paulina/0000-0003-3875-2534; Kalinowski,

Leszek/0000-0001-7270-1592; Daca, Agnieszka/0000-0003-1485-3090; Ploska,

Agata/0000-0002-0408-5262; gorska-ponikowska,

magdalena/0000-0002-7366-8429

FU Medical University of Gdansk, Gdansk, Poland [01-0419/08/259]; ST46

(Medical University of Gdansk, Gdansk, Poland) funding

FX The part of study was a funded research task of the Young Researcher No.

01-0419/08/259 (Medical University of Gdansk, Gdansk, Poland). The part

of study concerning nitro-oxidative stress as well as manuscript

publication was funded by ST46 (Medical University of Gdansk, Gdansk,

Poland) funding.

CR Averna M, 2008, FEBS J, V275, P2501, DOI 10.1111/j.1742-4658.2008.06394.x

Banerjee A, 2017, NEURO-ONCOLOGY, V19, P1135, DOI 10.1093/neuonc/now282

Bastian P, 2021, ANTIOXIDANTS-BASEL, V10, DOI 10.3390/antiox10020248

Boudesco C, 2018, METHODS MOL BIOL, V1709, P371, DOI 10.1007/978-1-4939-7477-1_27

Braeuninger S, 2005, CLIN NEUROPATHOL, V24, P175

Braunstein S, 2017, J NEURO-ONCOL, V134, P541, DOI 10.1007/s11060-017-2393-0

Bruce JY, 2012, INVEST NEW DRUG, V30, P794, DOI 10.1007/s10637-010-9618-9

Bukau B, 1998, CELL, V92, P351, DOI 10.1016/S0092-8674(00)80928-9

Cappello F, 2005, BMC CANCER, V5, DOI 10.1186/1471-2407-5-139

Cappello F, 2006, EUR J HISTOCHEM, V50, P25

Chamaon K, 2005, J NEURO-ONCOL, V72, P11, DOI 10.1007/s11060-004-2158-4

Chang I, 2013, MOL CANCER THER, V12, P1049, DOI 10.1158/1535-7163.MCT-12-1187

Chen R, 2017, NEUROTHERAPEUTICS, V14, P284, DOI 10.1007/s13311-017-0519-x

Choi S, 2018, NEURO-ONCOLOGY, V20, P1300, DOI 10.1093/neuonc/noy016

Davis AK, 2020, MOL PHARMACOL, V98, P243, DOI 10.1124/mol.120.119990

Davoodpour P, 2005, J BIOL CHEM, V280, P14773, DOI 10.1074/jbc.M414470200

Djavaheri-Mergny M, 2003, ONCOGENE, V22, P2558, DOI 10.1038/sj.onc.1206356

Fucarino A, 2020, J ONCOL, V2020, DOI 10.1155/2020/4701868

Gao XJ, 2020, BIOMED PHARMACOTHER, V129, DOI 10.1016/j.biopha.2020.110460

Golebiewska J, 2002, ACTA BIOCHIM POL, V49, P59

Rojas NG, 2020, J INTEGR NEUROSCI, V19, P187, DOI 10.31083/j.jin.2020.01.3

Gorska M, 2016, MOL NEUROBIOL, V53, P5030, DOI 10.1007/s12035-015-9434-5

Gorska M, 2016, CURR MED CHEM, V23, P1513, DOI 10.2174/0929867323666160316123443

Gorska M, 2016, ANTICANCER RES, V36, P1693

Gorska M, 2015, ONCOTARGET, V6, P15449, DOI 10.18632/oncotarget.3913

Gorska M, 2014, MOLECULES, V19, P13267, DOI 10.3390/molecules190913267

Gorska-Ponikowska M, 2021, SCI REP-UK, V11, DOI 10.1038/s41598-020-80816-x

Gorska-Ponikowska M, 2020, REDOX BIOL, V32, DOI 10.1016/j.redox.2020.101522

Gorska-Ponikowska M, 2020, INT J MOL SCI, V21, DOI 10.3390/ijms21020616

Gorska-Ponikowska M, 2018, CANCER GENOM PROTEOM, V15, P73, DOI 10.21873/cgp.20067

Guntuku L, 2016, CURR NEUROPHARMACOL, V14, P567, DOI 10.2174/1570159X14666160121115641

Harrison MR, 2011, INVEST NEW DRUG, V29, P1465, DOI 10.1007/s10637-010-9455-x

Huang TF, 2021, AUTOPHAGY, V17, P3592, DOI 10.1080/15548627.2021.1885203

Jego G, 2013, CANCER LETT, V332, P275, DOI 10.1016/j.canlet.2010.10.014

Jooma Rashid, 2019, Asian J Neurosurg, V14, P356, DOI 10.4103/ajns.AJNS_24_18

Kamm A, 2019, J ONCOL, V2019, DOI 10.1155/2019/9293416

Kamm A, 2019, NITRIC OXIDE-BIOL CH, V93, P102, DOI 10.1016/j.niox.2019.09.005

Kang SH, 2006, CANCER RES, V66, P11991, DOI 10.1158/0008-5472.CAN-06-1320

Kirches E, 2009, ANTI-CANCER AGENT ME, V9, P55, DOI 10.2174/187152009787047725

Kulke MH, 2011, CANCER CHEMOTH PHARM, V68, P293, DOI 10.1007/s00280-010-1478-7

Kumar AP, 2003, CARCINOGENESIS, V24, P209, DOI 10.1093/carcin/24.2.209

Lis A, 2004, CANCER LETT, V213, P57, DOI 10.1016/j.canlet.2004.04.021

Lleonart ME, 2017, MED RES REV, V37, P1275, DOI 10.1002/med.21459

Louis DN, 2007, ACTA NEUROPATHOL, V114, P97, DOI 10.1007/s00401-007-0243-4

Louis DN, 2016, ACTA NEUROPATHOL, V131, P803, DOI 10.1007/s00401-016-1545-1

Marina N, 2004, ONCOLOGIST, V9, P422, DOI 10.1634/theoncologist.9-4-422

Mikeladze MA, 2021, INT J MOL SCI, V22, DOI 10.3390/ijms22041520

Musial C, 2022, REDOX BIOL, V55, DOI 10.1016/j.redox.2022.102395

Nagy A, 2015, BRAIN RES, V1595, P127, DOI 10.1016/j.brainres.2014.10.062

Nuydens R, 1999, J NEUROSCI METH, V92, P153, DOI 10.1016/S0165-0270(99)00107-7

Orlicka-Plocka M, 2021, ANTIOXIDANTS-BASEL, V10, DOI 10.3390/antiox10060950

Parada-Bustamante A, 2015, MINI-REV MED CHEM, V15, P427, DOI 10.2174/1389557515666150226121052

Peng HM, 2009, BIOCHEMISTRY-US, V48, P8483, DOI 10.1021/bi901058g

Picard D, 2002, CELL MOL LIFE SCI, V59, P1640, DOI 10.1007/PL00012491

Pistritto G, 2016, AGING-US, V8, P603, DOI 10.18632/aging.100934

PRYOR WA, 1995, AM J PHYSIOL-LUNG C, V268, pL699, DOI 10.1152/ajplung.1995.268.5.L699

Qadan LR, 2001, BIOCHEM BIOPH RES CO, V285, P1259, DOI 10.1006/bbrc.2001.5320

Rappa F, 2012, ANTICANCER RES, V32, P5139

Reardon DA, 2020, JAMA ONCOL, V6, P1003, DOI 10.1001/jamaoncol.2020.1024

Ren L, 2015, ONCOTARGET, V6, P29469, DOI 10.18632/oncotarget.5177

Rérole AL, 2011, METHODS MOL BIOL, V787, P205, DOI 10.1007/978-1-61779-295-3_16

Rodrigues T, 2020, BIOCHEM PHARMACOL, V182, DOI 10.1016/j.bcp.2020.114282

Sbodio JI, 2019, ANTIOXID REDOX SIGN, V30, P1450, DOI 10.1089/ars.2017.7321

Schneider J, 1999, ANTICANCER RES, V19, P2141

She MR, 2007, ACTA PHARMACOL SIN, V28, P1037, DOI 10.1111/j.1745-7254.2007.00604.x

Sheng LX, 2020, BIOMOLECULES, V10, DOI 10.3390/biom10010123

Stankiewicz AR, 2005, J BIOL CHEM, V280, P38729, DOI 10.1074/jbc.M509497200

Stepien Aleksandra, 2007, Postepy Hig Med Dosw (Online), V61, P420

Sun H, 2020, DNA CELL BIOL, V39, P1228, DOI 10.1089/dna.2020.5425

Szkatula M., 2008, THESIS AKAD MEDYCZNA

Tan AS, 2014, BBA-GEN SUBJECTS, V1840, P1454, DOI 10.1016/j.bbagen.2013.10.016

Tang HP, 2016, SCI REP-UK, V6, DOI 10.1038/srep28388

Taylor OG, 2019, FRONT ONCOL, V9, DOI 10.3389/fonc.2019.00963

Tsai YP, 2009, CARCINOGENESIS, V30, P1049, DOI 10.1093/carcin/bgp087

Tzivion G, 2001, ONCOGENE, V20, P6331, DOI 10.1038/sj.onc.1204777

Valko M, 2006, CHEM-BIOL INTERACT, V160, P1, DOI 10.1016/j.cbi.2005.12.009

Vara-Perez M, 2019, CELLS-BASEL, V8, DOI 10.3390/cells8050493

Vijayanathan V, 2006, CLIN CANCER RES, V12, P2038, DOI 10.1158/1078-0432.CCR-05-2172

Wan Q., 2022, J CLEAN PROD, V373, P133781, DOI [10.1155/2022/4948943, DOI 10.1155/2022/1101015, 10.1016/j.jclepro.2022.133781, DOI 10.1016/J.JCLEPRO.2022.133781]

Wang XX, 2014, INT J ONCOL, V45, P18, DOI 10.3892/ijo.2014.2399

West AB, 2005, TRENDS NEUROSCI, V28, P348, DOI 10.1016/j.tins.2005.05.002

Yue TL, 1997, MOL PHARMACOL, V51, P951, DOI 10.1124/mol.51.6.951

Zhang Q, 2011, CANCER LETT, V313, P201, DOI 10.1016/j.canlet.2011.09.005

Zhang XY, 2010, ACTA BIOCH BIOPH SIN, V42, P615, DOI 10.1093/abbs/gmq065

Zhou X, 2018, RHEUMATOLOGY, V57, P1675, DOI 10.1093/rheumatology/key166

Zou HC, 2007, BRAIN RES, V1185, P231, DOI 10.1016/j.brainres.2007.07.092

NR 86

TC 5

Z9 6

U1 0

U2 4

PU MDPI

PI BASEL

PA ST ALBAN-ANLAGE 66, CH-4052 BASEL, SWITZERLAND

EI 2076-3921

J9 ANTIOXIDANTS-BASEL

JI Antioxidants

PD OCT

PY 2022

VL 11

IS 10

AR 2013

DI 10.3390/antiox11102013

PG 20

WC Biochemistry & Molecular Biology; Chemistry, Medicinal; Food Science &

Technology

WE Science Citation Index Expanded (SCI-EXPANDED)

SC Biochemistry & Molecular Biology; Pharmacology & Pharmacy; Food Science

& Technology

GA 5N9SQ

UT WOS:000872126100001

PM 36290736

OA gold, Green Published

DA 2025-04-09

ER

PT J

AU Guo, JS

Liu, KH

Wang, JY

Jiang, H

Zhang, MY

Liu, Y

Shan, CL

Hu, FZ

Fu, WZ

Zhang, CZ

Li, J

Chen, Y

AF Guo, Jianshuang

Liu, Kaihui

Wang, Jiyan

Jiang, Hao

Zhang, Mengyi

Liu, Yang

Shan, Changliang

Hu, Fangzhong

Fu, Wenzheng

Zhang, Chunze

Li, Jing

Chen, Yue

TI A rational foundation for micheliolide-based combination strategy by

targeting redox and metabolic circuit in cancer cells

SO BIOCHEMICAL PHARMACOLOGY

LA English

DT Article

DE Micheliolide; Oxidative stress; Glutathione metabolism; Mitochondrial

dysfunction; Glioblastoma; Leukemia

ID OXIDATIVE STRESS; SESQUITERPENE LACTONES; GLUTATHIONE; MITOCHONDRIA;

ROS; MODULATION; SYSTEMS; ROLES; AMPK

AB Accumulating evidence has supported that targeting oxidative stress and metabolic alterations of cancer is an effective strategy to combat cancer. We previously reported that Dimethylaminomicheliolide (DMAMCL) and its active metabolite micheliolide (MCL) can cause oxidative stress and cell death in leukemia and glioblastoma. However, the detailed mechanism underlying MCL or DMAMCL triggered oxidative stress remains elusive. Herein, using leukemia HL60 cells and glioblastoma U118MG cells as models, we found that MCL-induced oxidative stress is mainly mediated by reduced glutathione (GSH). Overproduced reactive oxygen species (ROS) can lead to oxidative damage to mitochondrial, impairing the ability of the tricarboxylic acid (TCA) cycle and causing dysfunction of mitochondrial respiratory chain. On the other hand, the depletion of GSH activates GSH biosynthesis pathway and has possibility to give rise to more GSH to scavenge ROS in cancer cells. Targeting this redox and metabolic circuit, we identified L-buthionine sulfoximine (BSO), an inhibitor in GSH biosynthesis, as an agent that can enhance MCL regimen to inhibit GSH compensatory event and thereby further facilitate cancer cell oxidative stress. Together, these results illustrate that targeting redox and metabolic pathway by MCL/DMAMCL combination with BSO is a potent therapeutic intervention for the treatments of glioblastoma and acute-myelocytic leukemia.

C1 [Guo, Jianshuang; Liu, Kaihui; Wang, Jiyan; Jiang, Hao; Zhang, Mengyi; Liu, Yang; Shan, Changliang; Hu, Fangzhong; Li, Jing; Chen, Yue] Nankai Univ, Coll Pharm, Coll Chem, State Key Lab Med Chem Biol, Haihe Educ Pk,38 Tongyan Rd, Tianjin 300353, Peoples R China.

[Fu, Wenzheng; Zhang, Chunze] Tianjin Union Med Ctr, Dept Colorectal Surg, Tianjin 300121, Peoples R China.

C3 Nankai University

RP Li, J; Chen, Y (corresponding author), Nankai Univ, Coll Pharm, Coll Chem, State Key Lab Med Chem Biol, Haihe Educ Pk,38 Tongyan Rd, Tianjin 300353, Peoples R China.

EM jinglink@nankai.edu.cn; yuechen@nankai.edu.cn

RI Shan, changliang/L-9029-2016; Li, Jing/HHC-5348-2022

OI Shan, Changliang/0000-0002-4906-1686

FU Accendatech Co., Ltd.; National Natural Science Foundation of China

[82073879, 81573282]; Postdoctoral Science Foundation of China

[2021M701791]; Natural Science Foundation of Tianjin [21JCYBJC00190];

National Science Fund for Distinguished Young Scholars [81625021]

FX We gratefully acknowledge the financial support of Accendatech Co.,

Ltd., and grants from the National Natural Science Foundation of China

(82073879, 81573282), the Postdoctoral Science Foundation of China

(2021M701791), the Natural Science Foundation of Tianjin (21JCYBJC00190)

and the National Science Fund for Distinguished Young Scholars

(81625021).

CR Adam-Vizi V, 2006, TRENDS PHARMACOL SCI, V27, P639, DOI 10.1016/j.tips.2006.10.005

An YH, 2015, PLOS ONE, V10, DOI 10.1371/journal.pone.0116202

Bailey HH, 1998, CHEM-BIOL INTERACT, V112, P239, DOI 10.1016/S0009-2797(97)00164-6

Bansal A, 2018, J CELL BIOL, V217, P2291, DOI 10.1083/jcb.201804161

Boonstra J, 2004, GENE, V337, P1, DOI 10.1016/j.gene.2004.04.032

Certo M, 2006, CANCER CELL, V9, P351, DOI 10.1016/j.ccr.2006.03.027

Circu ML, 2012, BBA-MOL CELL RES, V1823, P1767, DOI 10.1016/j.bbamcr.2012.06.019

Circu ML, 2010, FREE RADICAL BIO MED, V48, P749, DOI 10.1016/j.freeradbiomed.2009.12.022

Deus CM, 2020, TRENDS MOL MED, V26, P71, DOI 10.1016/j.molmed.2019.10.009

Di Pietro G, 2010, EXPERT OPIN DRUG MET, V6, P153, DOI 10.1517/17425250903427980

Estrela JM, 2006, CRIT REV CL LAB SCI, V43, P143, DOI 10.1080/10408360500523878

Gorrini C, 2013, NAT REV DRUG DISCOV, V12, P931, DOI 10.1038/nrd4002

Guo JS, 2019, FRONT ONCOL, V9, DOI 10.3389/fonc.2019.00993

Hardie DG, 2012, NAT REV MOL CELL BIO, V13, P251, DOI 10.1038/nrm3311

Hatem E, 2017, ANTIOXID REDOX SIGN, V27, P1217, DOI 10.1089/ars.2017.7134

Hayes JD, 2005, ANNU REV PHARMACOL, V45, P51, DOI 10.1146/annurev.pharmtox.45.120403.095857

Jagust P, 2020, WORLD J STEM CELLS, V12, DOI 10.4252/wjsc.v12.i11.1410

Leadsham JE, 2013, CELL METAB, V18, P279, DOI 10.1016/j.cmet.2013.07.005

Li QY, 2020, J MOL MED, V98, P263, DOI 10.1007/s00109-019-01839-0

Mihaylova MM, 2011, NAT CELL BIOL, V13, P1016, DOI 10.1038/ncb2329

Moloney JN, 2018, SEMIN CELL DEV BIOL, V80, P50, DOI 10.1016/j.semcdb.2017.05.023

Mondet J, 2021, EXP HEMATOL, V98, P53, DOI 10.1016/j.exphem.2021.03.001

Murphy MP, 2011, CELL METAB, V13, P361, DOI 10.1016/j.cmet.2011.03.010

O'Malley J, 2020, TRENDS CANCER, V6, P688, DOI 10.1016/j.trecan.2020.04.009

Perry G, 2000, FREE RADICAL BIO MED, V28, P831, DOI 10.1016/S0891-5849(00)00158-1

Poprac P, 2017, TRENDS PHARMACOL SCI, V38, P592, DOI 10.1016/j.tips.2017.04.005

Postovit L, 2018, OXID MED CELL LONGEV, V2018, DOI 10.1155/2018/6135739

Prasad S, 2017, CANCER LETT, V387, P95, DOI 10.1016/j.canlet.2016.03.042

Quinlan CL, 2014, J BIOL CHEM, V289, P8312, DOI 10.1074/jbc.M113.545301

Schafer FQ, 2001, FREE RADICAL BIO MED, V30, P1191, DOI 10.1016/S0891-5849(01)00480-4

Schmidt TJ, 1999, BIOORGAN MED CHEM, V7, P2849, DOI 10.1016/S0968-0896(99)00234-5

Sporn MB, 2012, NAT REV CANCER, V12, P564, DOI 10.1038/nrc3278

Tonekaboni SAM, 2018, BRIEF BIOINFORM, V19, P263, DOI 10.1093/bib/bbw104

TOYOKUNI S, 1995, FEBS LETT, V358, P1, DOI 10.1016/0014-5793(94)01368-B

Trachootham D, 2009, NAT REV DRUG DISCOV, V8, P579, DOI 10.1038/nrd2803

Traverso N, 2013, OXID MED CELL LONGEV, V2013, DOI 10.1155/2013/972913

Wang K, 2019, TRENDS BIOCHEM SCI, V44, P401, DOI 10.1016/j.tibs.2019.01.001

Xi X.-N., J CHROMATOGR B, V1104

Xie H, 2017, J BIOL CHEM, V292, P16825, DOI 10.1074/jbc.R117.799973

Zhang Q, 2012, J MED CHEM, V55, P8757, DOI 10.1021/jm301064b

NR 40

TC 6

Z9 7

U1 1

U2 28

PU PERGAMON-ELSEVIER SCIENCE LTD

PI OXFORD

PA THE BOULEVARD, LANGFORD LANE, KIDLINGTON, OXFORD OX5 1GB, ENGLAND

SN 0006-2952

EI 1873-2968

J9 BIOCHEM PHARMACOL

JI Biochem. Pharmacol.

PD JUN

PY 2022

VL 200

AR 115037

DI 10.1016/j.bcp.2022.115037

EA APR 2022

PG 12

WC Pharmacology & Pharmacy

WE Science Citation Index Expanded (SCI-EXPANDED)

SC Pharmacology & Pharmacy

GA 2S6WJ

UT WOS:000821930700007

PM 35427571

DA 2025-04-09

ER

PT J

AU Liu, SY

Dong, LH

Shi, WY

Zheng, ZZ

Liu, ZJ

Meng, LB

Xin, Y

Jiang, X

AF Liu, Shiyu

Dong, Lihua

Shi, Weiyan

Zheng, Zhuangzhuang

Liu, Zijing

Meng, Lingbin

Xin, Ying

Jiang, Xin

TI Potential targets and treatments affect oxidative stress in gliomas: An

overview of molecular mechanisms

SO FRONTIERS IN PHARMACOLOGY

LA English

DT Review

DE Reactive Oxygen Species (ROS); gliomas; oxidative stress; target gene;

therapeutic strategy

ID CENTRAL-NERVOUS-SYSTEM; FREE-RADICALS; CELL-DEATH; IN-VITRO; INDUCE

APOPTOSIS; ROS PRODUCTION; GLIOBLASTOMA; ACTIVATION; ACCUMULATION;

INFLAMMATION

AB Oxidative stress refers to the imbalance between oxidation and antioxidant activity in the body. Oxygen is reduced by electrons as part of normal metabolism leading to the formation of various reactive oxygen species (ROS). ROS are the main cause of oxidative stress and can be assessed through direct detection. Oxidative stress is a double-edged phenomenon in that it has protective mechanisms that help to destroy bacteria and pathogens, however, increased ROS accumulation can lead to host cell apoptosis and damage. Glioma is one of the most common malignant tumors of the central nervous system and is characterized by changes in the redox state. Therapeutic regimens still encounter multiple obstacles and challenges. Glioma occurrence is related to increased free radical levels and decreased antioxidant defense responses. Oxidative stress is particularly important in the pathogenesis of gliomas, indicating that antioxidant therapy may be a means of treating tumors. This review evaluates oxidative stress and its effects on gliomas, describes the potential targets and therapeutic drugs in detail, and clarifies the effects of radiotherapy and chemotherapy on oxidative stress. These data may provide a reference for the development of precise therapeutic regimes of gliomas based on oxidative stress.

C1 [Liu, Shiyu; Dong, Lihua; Shi, Weiyan; Zheng, Zhuangzhuang; Liu, Zijing; Jiang, Xin] First Hosp Jilin Univ, Jilin Prov Key Lab Radiat Oncol & Therapy, Changchun, Peoples R China.

[Liu, Shiyu; Dong, Lihua; Shi, Weiyan; Zheng, Zhuangzhuang; Liu, Zijing; Jiang, Xin] First Hosp Jilin Univ, Dept Radiat Oncol, Changchun, Peoples R China.

[Liu, Shiyu; Dong, Lihua; Shi, Weiyan; Zheng, Zhuangzhuang; Liu, Zijing; Jiang, Xin] Jilin Univ, Sch Publ Hlth, NHC Key Lab Radiobiol, Changchun, Peoples R China.

[Meng, Lingbin] H Lee Moffitt Canc Ctr & Res Inst, Dept Hematol & Med Oncol, Tampa, FL USA.

[Xin, Ying] Jilin Univ, Key Lab Pathobiol, Minist Educ, Changchun, Peoples R China.

C3 Jilin University; Jilin University; Jilin University; H Lee Moffitt

Cancer Center & Research Institute; Jilin University

RP Jiang, X (corresponding author), First Hosp Jilin Univ, Jilin Prov Key Lab Radiat Oncol & Therapy, Changchun, Peoples R China.; Jiang, X (corresponding author), First Hosp Jilin Univ, Dept Radiat Oncol, Changchun, Peoples R China.; Jiang, X (corresponding author), Jilin Univ, Sch Publ Hlth, NHC Key Lab Radiobiol, Changchun, Peoples R China.; Xin, Y (corresponding author), Jilin Univ, Key Lab Pathobiol, Minist Educ, Changchun, Peoples R China.

EM xiny@jlu.edu.cn; jiangx@jlu.edu.cn

RI liu, shiyu/JSL-3045-2023; Shi, Weiyan/JOK-7836-2023; Jiang,

Xin/AHA-9454-2022

OI Xin, Ying/0000-0001-7591-9423; Jiang, Xin/0000-0002-4613-7438

FU Jilin Provincial Science and Technology Foundation [20190201200JC]

FX This research was funded by the Jilin Provincial Science and Technology

Foundation (Grant number: 20190201200JC).

CR Abbott NJ, 2010, NEUROBIOL DIS, V37, P13, DOI 10.1016/j.nbd.2009.07.030

Ahmadov U, 2021, CELL DEATH DIS, V12, DOI 10.1038/s41419-021-04146-0

Amaral RF, 2021, J NEUROCHEM, V156, P499, DOI 10.1111/jnc.15097

AMES BN, 1983, SCIENCE, V221, P1256, DOI 10.1126/science.6351251

Aruoma OI, 2006, BIOFACTORS, V27, P1, DOI 10.1002/biof.5520270101

Baird L, 2020, MOL CELL BIOL, V40, DOI 10.1128/MCB.00099-20

Barciszewska AM, 2019, CELLS-BASEL, V8, DOI 10.3390/cells8091065

Behrend L, 2003, BIOCHEM SOC T, V31, P1441

Betteridge DJ, 2000, METABOLISM, V49, P3, DOI 10.1016/S0026-0495(00)80077-3

Brown DI, 2009, FREE RADICAL BIO MED, V47, P1239, DOI 10.1016/j.freeradbiomed.2009.07.023

Burtenshaw D, 2019, FRONT CARDIOVASC MED, V6, DOI 10.3389/fcvm.2019.00089

Chang JY, 2007, J NEURO-ONCOL, V84, P9, DOI 10.1007/s11060-007-9347-x

Chang MZ, 2017, ONCOL REP, V38, P1251, DOI 10.3892/or.2017.5780

Chen D, 2017, ONCOGENE, V36, P5593, DOI 10.1038/onc.2017.146

Chen MH, 2021, NANOMATERIALS-BASEL, V11, DOI 10.3390/nano11071661

Chen TC, 2020, REDOX BIOL, V30, DOI 10.1016/j.redox.2019.101413

Chen ZH, 2018, FRONT IMMUNOL, V9, DOI 10.3389/fimmu.2018.01004

Cheng X, 2020, CELL METAB, V32, P229, DOI 10.1016/j.cmet.2020.06.002

Cholia RP, 2018, METAB BRAIN DIS, V33, P1307, DOI 10.1007/s11011-018-0233-3

DEDUVE C, 1966, PHYSIOL REV, V46, P323, DOI 10.1152/physrev.1966.46.2.323

Deryugina EI, 1997, J CELL SCI, V110, P2473

Dixit D, 2014, CELL DEATH DIS, V5, DOI 10.1038/cddis.2014.179

Ersoz M, 2020, PHARM DEV TECHNOL, V25, P757, DOI 10.1080/10837450.2020.1740933

Feng J, 2016, ONCOL REP, V35, P1395, DOI 10.3892/or.2015.4477

Fleming AM, 2017, DNA REPAIR, V56, P75, DOI 10.1016/j.dnarep.2017.06.009

FLINT DH, 1993, J BIOL CHEM, V268, P14732

Fridovich I, 1997, J BIOL CHEM, V272, P18515, DOI 10.1074/jbc.272.30.18515

Gao Z, 2008, FREE RADICAL BIO MED, V45, P1501, DOI 10.1016/j.freeradbiomed.2008.08.009

Geng Y, 2010, NEURO-ONCOLOGY, V12, P473, DOI 10.1093/neuonc/nop048

GERSCHMAN R, 1954, SCIENCE, V119, P623, DOI 10.1126/science.119.3097.623

Gorrini C, 2013, NAT REV DRUG DISCOV, V12, P931, DOI 10.1038/nrd4002

Grabowski MM, 2021, J NEURO-ONCOL, V151, P3, DOI 10.1007/s11060-020-03483-y

HALLIWELL B, 1992, FEBS LETT, V307, P108, DOI 10.1016/0014-5793(92)80911-Y

Ham SW, 2019, CELL DEATH DIFFER, V26, P409, DOI 10.1038/s41418-018-0126-3

Han S, 2019, EXP MOL MED, V51, DOI 10.1038/s12276-019-0351-y

Hayashi S, 1999, CIRC RES, V85, P663, DOI 10.1161/01.RES.85.8.663

Hoelzinger DB, 2007, J NATL CANCER I, V99, P1583, DOI 10.1093/jnci/djm187

Hsieh CH, 2011, PLOS ONE, V6, DOI 10.1371/journal.pone.0023945

Hsieh CH, 2010, ONCOL REP, V24, P1629, DOI 10.3892/or_00001027

Huang HH, 2021, NAT COMMUN, V12, DOI 10.1038/s41467-021-24108-6

Huang LY, 2015, MOL NEUROBIOL, V52, P626, DOI 10.1007/s12035-014-8888-1

Huangfu MJ, 2021, FEBS OPEN BIO, V11, P456, DOI 10.1002/2211-5463.13069

Iida T, 2001, NEURO-ONCOLOGY, V3, P73, DOI 10.1093/neuonc/3.2.73

Illán-Cabeza NA, 2020, J INORG BIOCHEM, V207, DOI 10.1016/j.jinorgbio.2020.111053

Jahani-Asl A, 2016, NAT NEUROSCI, V19, P798, DOI 10.1038/nn.4295

Jha P, 2014, NEURO-ONCOLOGY, V16, P1607, DOI 10.1093/neuonc/nou113

Kanzaki H, 2013, J BIOL CHEM, V288, P23009, DOI 10.1074/jbc.M113.478545

Kensler TW, 2007, ANNU REV PHARMACOL, V47, P89, DOI 10.1146/annurev.pharmtox.46.120604.141046

Kitagawa T, 2015, ONCOL REP, V33, P583, DOI 10.3892/or.2014.3618

Krylova NG, 2019, MOL CELL BIOCHEM, V462, P195, DOI 10.1007/s11010-019-03622-8

Lee SY, 2016, GENES DIS, V3, P198, DOI 10.1016/j.gendis.2016.04.007

Lei KC, 2020, J HEMATOL ONCOL, V13, DOI 10.1186/s13045-020-00979-y

Lei QQ, 2016, ONCOTARGET, V7, P5007, DOI 10.18632/oncotarget.6652

Li SZ, 2014, BIOCHEM BIOPH RES CO, V444, P6, DOI 10.1016/j.bbrc.2013.12.136

Liang J, 2017, CELL RES, V27, P329, DOI 10.1038/cr.2016.159

LIOCHEV SI, 1994, FREE RADICAL BIO MED, V16, P29, DOI 10.1016/0891-5849(94)90239-9

Liu C, 2018, NEUROCHEM RES, V43, P1317, DOI 10.1007/s11064-018-2547-2

Liu SY, 2021, J CANCER, V12, P1094, DOI 10.7150/jca.51107

Liu W, 2020, ONCOTARGETS THER, V13, P2275, DOI 10.2147/OTT.S243953

Liu XR, 2015, EUR REV MED PHARMACO, V19, P4068

Liu XH, 2019, J EXP CLIN CANC RES, V38, DOI 10.1186/s13046-019-1173-4

Liu YY, 2016, BIOCHEM BIOPH RES CO, V480, P415, DOI 10.1016/j.bbrc.2016.10.064

Louis DN, 2021, NEURO-ONCOLOGY, V23, P1231, DOI 10.1093/neuonc/noab106

Louis DN, 2016, ACTA NEUROPATHOL, V131, P803, DOI 10.1007/s00401-016-1545-1

Lu B, 2017, ACTA PHARMACOL SIN, V38, P1543, DOI 10.1038/aps.2017.112

Ma DD, 2016, CANCER LETT, V371, P194, DOI 10.1016/j.canlet.2015.11.044

Massi P, 2006, CELL MOL LIFE SCI, V63, P2057, DOI 10.1007/s00018-006-6156-x

MCCORD JM, 1969, J BIOL CHEM, V244, P6049

McKelvey KJ, 2021, FRONT ONCOL, V11, DOI 10.3389/fonc.2021.633210

Meyer N, 2021, AUTOPHAGY, V17, P3424, DOI 10.1080/15548627.2021.1874208

Milkovic L, 2014, CURR PHARM DESIGN, V20, P6529, DOI 10.2174/1381612820666140826152822

Mittal M, 2014, ANTIOXID REDOX SIGN, V20, P1126, DOI 10.1089/ars.2012.5149

Mitteer RA Jr, 2015, SCI REP-UK, V5, DOI 10.1038/srep13961

Mudassar F, 2020, J EXP CLIN CANC RES, V39, DOI 10.1186/s13046-020-01724-6

Nomura J, 2013, PLOS ONE, V8, DOI 10.1371/journal.pone.0075527

Nosaka Y, 2017, CHEM REV, V117, P11302, DOI 10.1021/acs.chemrev.7b00161

Omuro A, 2013, JAMA-J AM MED ASSOC, V310, P1842, DOI 10.1001/jama.2013.280319

Ostrom QT, 2021, NEURO-ONCOLOGY, V23, P1, DOI 10.1093/neuonc/noab200

Ostrom QT, 2014, NEURO-ONCOLOGY, V16, P896, DOI 10.1093/neuonc/nou087

Ozyerli-Goknar E, 2019, CELL DEATH DIS, V10, DOI 10.1038/s41419-019-2107-y

Pan H, 2013, WORLD NEUROSURG, V80, P363, DOI 10.1016/j.wneu.2011.06.063

Racoma IO, 2013, PLOS ONE, V8, DOI 10.1371/journal.pone.0072882

Reczek CR, 2017, ANNU REV CANC BIOL, V1, P79, DOI 10.1146/annurev-cancerbio-041916-065808

Reuter S, 2010, FREE RADICAL BIO MED, V49, P1603, DOI 10.1016/j.freeradbiomed.2010.09.006

Sharanek A, 2020, NAT COMMUN, V11, DOI 10.1038/s41467-020-17885-z

Shi YK, 2017, J HEMATOL ONCOL, V10, DOI 10.1186/s13045-017-0439-6

Shono T, 2008, INT J CANCER, V123, P787, DOI 10.1002/ijc.23569

Silva L, 2019, FUTURE MED CHEM, V11, P645, DOI 10.4155/fmc-2018-0251

Singer E, 2015, CELL DEATH DIS, V6, DOI 10.1038/cddis.2014.566

SOHAL RS, 1990, EXP GERONTOL, V25, P499, DOI 10.1016/0531-5565(90)90017-V

Takabe H, 2018, PHARMACEUTICS, V10, DOI 10.3390/pharmaceutics10020060

Tan AC, 2020, CA-CANCER J CLIN, V70, P299, DOI 10.3322/caac.21613

Tavana E, 2020, BIOFACTORS, V46, P356, DOI 10.1002/biof.1605

Thannickal VJ, 2000, AM J PHYSIOL-LUNG C, V279, pL1005

Tomar MS, 2021, BBA-REV CANCER, V1876, DOI 10.1016/j.bbcan.2021.188616

Trachootham D, 2009, NAT REV DRUG DISCOV, V8, P579, DOI 10.1038/nrd2803

Valko M, 2006, CHEM-BIOL INTERACT, V160, P1, DOI 10.1016/j.cbi.2005.12.009

Valko M, 2007, INT J BIOCHEM CELL B, V39, P44, DOI 10.1016/j.biocel.2006.07.001

Wada T, 2004, ONCOGENE, V23, P2838, DOI 10.1038/sj.onc.1207556

Wang CC, 2020, CELL DEATH DIS, V11, DOI 10.1038/s41419-020-02866-3

WANG GL, 1995, J BIOL CHEM, V270, P1230, DOI 10.1074/jbc.270.3.1230

Wang JT, 2012, J PINEAL RES, V53, P180, DOI 10.1111/j.1600-079X.2012.00985.x

Wang K, 2016, NEUROCHEM RES, V41, P1439, DOI 10.1007/s11064-016-1854-8

Wang PF, 2018, J NEUROINFLAMM, V15, DOI 10.1186/s12974-018-1187-4

Wang YH, 2017, INT J NANOMED, V12, P1369, DOI 10.2147/IJN.S124276

Wang YJ, 2019, ONCOTARGETS THER, V12, P1867, DOI 10.2147/OTT.S195329

Waris Gulam, 2006, J Carcinog, V5, P14

Wei JL, 2019, CANCER MANAG RES, V11, P167, DOI 10.2147/CMAR.S188655

Wu LQ, 2019, J CELL BIOCHEM, V120, P19044, DOI 10.1002/jcb.29227

Yang HQ, 2020, INT J NANOMED, V15, P7791, DOI 10.2147/IJN.S267120

Yang L, 2017, PLOS ONE, V12, DOI 10.1371/journal.pone.0174839

Yang WW, 2012, MOL CELL, V48, P771, DOI 10.1016/j.molcel.2012.09.028

Yang WW, 2012, NAT CELL BIOL, V14, P1295, DOI 10.1038/ncb2629

Zhao P, 2012, BMC CANCER, V12, DOI 10.1186/1471-2407-12-617

Zheng LJ, 2017, MOL NEUROBIOL, V54, P3492, DOI 10.1007/s12035-016-9926-y

Zhong H, 1999, CANCER RES, V59, P5830

Zhou HX, 2020, OXID MED CELL LONGEV, V2020, DOI 10.1155/2020/7126976

Zhu JH, 2014, ONCOL REP, V32, P443, DOI 10.3892/or.2014.3259

NR 118

TC 16

Z9 18

U1 3

U2 20

PU FRONTIERS MEDIA SA

PI LAUSANNE

PA AVENUE DU TRIBUNAL FEDERAL 34, LAUSANNE, CH-1015, SWITZERLAND

EI 1663-9812

J9 FRONT PHARMACOL

JI Front. Pharmacol.

PD JUL 22

PY 2022

VL 13

AR 921070

DI 10.3389/fphar.2022.921070

PG 16

WC Pharmacology & Pharmacy

WE Science Citation Index Expanded (SCI-EXPANDED)

SC Pharmacology & Pharmacy

GA 3P2KG

UT WOS:000837367600001

PM 35935861

OA gold, Green Published

DA 2025-04-09

ER

PT J

AU Liang, XS

Wang, ZY

Dai, ZY

Liu, J

Zhang, H

Wen, J

Zhang, N

Zhang, J

Luo, P

Liu, ZQ

Liu, ZX

Cheng, Q

AF Liang, Xisong

Wang, Zeyu

Dai, Ziyu

Liu, Jian

Zhang, Hao

Wen, Jie

Zhang, Nan

Zhang, Jian

Luo, Peng

Liu, Zaoqu

Liu, Zhixiong

Cheng, Quan

TI Oxidative stress is involved in immunosuppression and macrophage

regulation in glioblastoma

SO CLINICAL IMMUNOLOGY

LA English

DT Article

DE Oxidative stress; Glioblastoma; SOD3; Microglia; Macrophage

ID EXTRACELLULAR-SUPEROXIDE DISMUTASE; CENTRAL-NERVOUS-SYSTEM; CELL-DEATH;

EXPRESSION; CANCER; CLASSIFICATION; CATABOLISM; LYSOSOMES; PHENOTYPE;

SIGNATURE

AB Oxidative stress dually affected cancer progression, while its effect on glioblastomas remained unclear. Herein, we clustered the multicenter glioblastoma cohorts based on the oxidative-stress-responsive genes (OSS) expression. We found that cluster 2 with high OSS levels suffered a worse prognosis. Functional analyses and immune-related analyses results exhibited that M2-like pro-tumoral macrophages and neutrophils were enriched in cluster 2, while Natural killer cells' infiltration was decreased. The increased M2-like pro-tumoral macrophages in cluster 2 was confirmed by immunofluorescence. An integrated single-cell analysis validated the malignant features of cluster 2 neoplastic cells and discovered their crosstalk with M2-like pro-tumoral macrophages. Moreover, we observed that SOD3 knockdown might decrease the M2-like pro-tumoral transformation of macrophage in vitro and in vivo. Comprehensively, we revealed oxidative stress' prognostic and immunosuppressive potential in glioblastoma and discovered SOD3's potential role in regulating macrophage M2-like pro-tumoral transformation.

C1 [Liang, Xisong; Wang, Zeyu; Dai, Ziyu; Zhang, Hao; Wen, Jie; Liu, Zhixiong; Cheng, Quan] Cent South Univ, Xiangya Hosp, Dept Neurosurg, Changsha 410008, Hunan, Peoples R China.

[Liang, Xisong; Wang, Zeyu; Dai, Ziyu; Zhang, Hao; Wen, Jie; Liu, Zhixiong; Cheng, Quan] Cent South Univ, Xiangya Hosp, Natl Clin Res Ctr Geriatr Disorders, Changsha 410008, Hunan, Peoples R China.

[Liu, Jian] Hunan Univ Chinese Med, Expt Ctr Med Innovat, Hosp 1, Changsha 410007, Hunan, Peoples R China.

[Zhang, Nan] Huazhong Univ Sci & Technol, Coll Life Sci & Technol, Wuhan 430074, Hubei, Peoples R China.

[Zhang, Jian; Luo, Peng] Southern Med Univ, Zhujiang Hosp, Dept Oncol, Guangzhou 510000, Guangdong, Peoples R China.

[Liu, Zaoqu] Zhengzhou Univ, Dept Intervent Radiol, Affiliated Hosp 1, Zhengzhou 450052, Henan, Peoples R China.

[Cheng, Quan] Cent South Univ, Xiangya Hosp, Dept Clin Pharmacol, Changsha 410008, Hunan, Peoples R China.

C3 Central South University; Central South University; Hunan University of

Chinese Medicine; Huazhong University of Science & Technology; Southern

Medical University - China; Zhengzhou University; Central South

University

RP Liu, ZX; Cheng, Q (corresponding author), Cent South Univ, Xiangya Hosp, Dept Neurosurg, Changsha 410008, Hunan, Peoples R China.

EM zhixiongliu@csu.edu.cn; chengquan@csu.edu.cn

RI Luo, Peng/I-4790-2019; CHENG, QUAN/AAJ-6264-2021; Liu,

Zaoqu/AAV-9348-2021; Wang, Zeyu/AAB-1057-2022; wang, nan/KHW-4897-2024;

Luo, Peng/C-5323-2017; Ziyu, Dai/GRR-6048-2022

OI Luo, Peng/0000-0002-8215-2045; Ziyu, Dai/0000-0003-2855-741X; Wang,

Zeyu/0000-0002-6675-5399; Cheng, Quan/0000-0003-2401-5349

FU Hunan Provincial Natural Science Foundation of China [20221120095];

National Natural Science Foundation of China [82073893, 82172685]; Hunan

Pro-vincial Health Committee Foundation of China [202204044869]

FX This work was supported by the Hunan Provincial Natural Science

Foundation of China [NO. 20221120095] ; the National Natural Science

Foundation of China [NO. 82073893, NO. 82172685] ; the Hunan Pro-vincial

Health Committee Foundation of China [NO. 202204044869] .

CR Aran D, 2017, GENOME BIOL, V18, DOI 10.1186/s13059-017-1349-1

Bauernhofer T, 2003, EUR J IMMUNOL, V33, P119, DOI 10.1002/immu.200390014

Castanheira FVS, 2019, BLOOD, V133, P2178, DOI 10.1182/blood-2018-11-844530

Chang HW, 2021, CELL SIGNAL, V77, DOI 10.1016/j.cellsig.2020.109820

Che MX, 2016, DRUG DISCOV TODAY, V21, P143, DOI 10.1016/j.drudis.2015.10.001

Cheng X, 2020, CELL METAB, V32, P229, DOI 10.1016/j.cmet.2020.06.002

Cholia RP, 2018, METAB BRAIN DIS, V33, P1307, DOI 10.1007/s11011-018-0233-3

Delgado-Rizo V, 2017, FRONT IMMUNOL, V8, DOI 10.3389/fimmu.2017.00081

Dong ST, 2022, ACTA PHARM SIN B, V12, P1163, DOI 10.1016/j.apsb.2021.08.020

Fang X, 2021, BIOCHEM BIOPH RES CO, V560, P165, DOI 10.1016/j.bbrc.2021.05.003

Fauster A, 2019, CELL DEATH DIFFER, V26, P1138, DOI 10.1038/s41418-018-0192-6

Feng L, 2017, SCI REP-UK, V7, DOI 10.1038/s41598-017-05538-z

Galluzzi L, 2017, ANNU REV PATHOL-MECH, V12, P103, DOI 10.1146/annurev-pathol-052016-100247

Gao RL, 2021, NAT BIOTECHNOL, V39, P599, DOI 10.1038/s41587-020-00795-2

Giampietri Claudia, 2014, Int J Cell Biol, V2014, P490275, DOI 10.1155/2014/490275

Goji T, 2017, J BIOL CHEM, V292, P19721, DOI 10.1074/jbc.M117.814392

Griess B, 2020, EPIGENETICS-US, V15, P1325, DOI 10.1080/15592294.2020.1777666

Griess B, 2017, FREE RADICAL BIO MED, V112, P464, DOI 10.1016/j.freeradbiomed.2017.08.013

Guicciardi ME, 2004, ONCOGENE, V23, P2881, DOI 10.1038/sj.onc.1207512

Gusev Y, 2018, SCI DATA, V5, DOI 10.1038/sdata.2018.158

Hänzelmann S, 2013, BMC BIOINFORMATICS, V14, DOI 10.1186/1471-2105-14-7

Hao YH, 2021, CELL, V184, P3573, DOI 10.1016/j.cell.2021.04.048

Hayes JD, 2020, CANCER CELL, V38, P167, DOI 10.1016/j.ccell.2020.06.001

Holze C, 2018, NAT IMMUNOL, V19, P130, DOI 10.1038/s41590-017-0013-y

Hu LL, 2019, REDOX BIOL, V26, DOI 10.1016/j.redox.2019.101268

Ishimoto T, 2014, CARCINOGENESIS, V35, P1003, DOI 10.1093/carcin/bgt402

Jin SQ, 2021, NAT COMMUN, V12, DOI 10.1038/s41467-021-21246-9

Joo KM, 2013, CELL REP, V3, P260, DOI 10.1016/j.celrep.2012.12.013

Joy A, 2014, PLOS ONE, V9, DOI 10.1371/journal.pone.0100827

Kavcic N, 2017, BIOL CHEM, V398, P289, DOI 10.1515/hsz-2016-0252

Kawaguchi A, 2013, CANCER SCI, V104, P1205, DOI 10.1111/cas.12214

Khan MA, 2017, SCI REP-UK, V7, DOI 10.1038/srep41749

Kuo CL, 2020, CANCER LETT, V474, P138, DOI 10.1016/j.canlet.2020.01.019

Laurila JP, 2009, PLOS ONE, V4, DOI 10.1371/journal.pone.0005786

Levine B, 2019, CELL, V176, P11, DOI 10.1016/j.cell.2018.09.048

Li JA, 2021, FRONT GENET, V12, DOI 10.3389/fgene.2021.615834

Liu J, 2020, CANCER GENE THER, V27, P267, DOI 10.1038/s41417-019-0134-6

Louis DN, 2007, ACTA NEUROPATHOL, V114, P547, DOI 10.1007/s00401-007-0278-6

Louis DN, 2021, NEURO-ONCOLOGY, V23, P1231, DOI 10.1093/neuonc/noab106

Louis DN, 2016, ACTA NEUROPATHOL, V131, P803, DOI 10.1007/s00401-016-1545-1

Luo LX, 2022, MAR DRUGS, V20, DOI 10.3390/md20010029

Malta TM, 2018, NEURO-ONCOLOGY, V20, P608, DOI 10.1093/neuonc/nox183

Martínez-Reyes I, 2021, NAT REV CANCER, V21, P669, DOI 10.1038/s41568-021-00378-6

Martins I, 2017, BIOMED J, V40, P133, DOI 10.1016/j.bj.2017.05.001

Matés JM, 2020, ARCH TOXICOL, V94, P2603, DOI 10.1007/s00204-020-02838-8

Mensurado S, 2018, PLOS BIOL, V16, DOI 10.1371/journal.pbio.2004990

Mira E, 2018, NAT COMMUN, V9, DOI 10.1038/s41467-018-03079-1

Mohrenz IV, 2013, APOPTOSIS, V18, P1416, DOI 10.1007/s10495-013-0877-8

Molinaro AM, 2019, NAT REV NEUROL, V15, P405, DOI 10.1038/s41582-019-0220-2

Mootha VK, 2003, NAT GENET, V34, P267, DOI 10.1038/ng1180

Morotti M, 2021, BRIT J CANCER, V124, P494, DOI 10.1038/s41416-020-01113-y

Mukha A, 2021, THERANOSTICS, V11, P7844, DOI 10.7150/thno.58655

Murat A, 2008, J CLIN ONCOL, V26, P3015, DOI 10.1200/JCO.2007.15.7164

Neftel C, 2019, CELL, V178, P835, DOI 10.1016/j.cell.2019.06.024

Newman AM, 2015, NAT METHODS, V12, P453, DOI [10.1038/NMETH.3337, 10.1038/nmeth.3337]

Nissen SE, 2022, JAMA-J AM MED ASSOC, V327, P1679, DOI 10.1001/jama.2022.5050

Olivier C, 2021, FRONT MOL BIOSCI, V7, DOI 10.3389/fmolb.2020.620677

Rapoport BL, 2020, MOLECULES, V25, DOI 10.3390/molecules25071618

Robinson N, 2019, REDOX BIOL, V26, DOI 10.1016/j.redox.2019.101239

Rojo AI, 2004, J NEUROSCI, V24, P7324, DOI 10.1523/JNEUROSCI.2111-04.2004

Skendros P, 2018, FRONT CELL DEV BIOL, V6, DOI 10.3389/fcell.2018.00109

Song XX, 2018, GASTROENTEROLOGY, V154, P1480, DOI 10.1053/j.gastro.2017.12.004

Sun G, 2020, AGING-US, V12, P1114, DOI 10.18632/aging.102648

Teoh MLT, 2009, CANCER RES, V69, P6355, DOI 10.1158/0008-5472.CAN-09-1195

Teoh-Fitzgerald MLT, 2012, MOL CANCER RES, V10, P40, DOI 10.1158/1541-7786.MCR-11-0501

Tibshirani R, 1997, STAT MED, V16, P385, DOI 10.1002/(SICI)1097-0258(19970228)16:4<385::AID-SIM380>3.0.CO;2-3

Tong YY, 2021, MOL CELL, V81, P2303, DOI 10.1016/j.molcel.2021.04.002

Tsai M.L., 2021, Int. J. Mol. Sci., V23

Verhaak RGW, 2010, CANCER CELL, V17, P98, DOI 10.1016/j.ccr.2009.12.020

Wang J, 2022, CANCER LETT, V543, DOI 10.1016/j.canlet.2022.215766

Xuan WJ, 2021, TRENDS IMMUNOL, V42, P280, DOI 10.1016/j.it.2021.02.004

Yang CD, 2009, CANCER RES, V69, P7986, DOI 10.1158/0008-5472.CAN-09-2266

Yang KY, 2022, MOL CANCER, V21, DOI 10.1186/s12943-022-01513-z

Ye Y, 2021, CELL DEATH DISCOV, V7, DOI 10.1038/s41420-021-00451-x

Yoshihara K, 2013, NAT COMMUN, V4, DOI 10.1038/ncomms3612

Yu XX, 2016, ONCOTARGET, V7, P14161, DOI 10.18632/oncotarget.7416

Zhang LY, 2021, CANCER LETT, V500, P87, DOI 10.1016/j.canlet.2020.12.013

Zhang N, 2020, J AGR FOOD CHEM, V68, P11182, DOI 10.1021/acs.jafc.0c04041

Zhang YQ, 2020, NAR GENOM BIOINFORM, V2, DOI 10.1093/nargab/lqaa078

Zhao P, 2012, BMC CANCER, V12, DOI 10.1186/1471-2407-12-617

Zhao X, 2022, EUR J PHARMACOL, V929, DOI 10.1016/j.ejphar.2022.175115

Zhao Z, 2021, GENOM PROTEOM BIOINF, V19, P1, DOI 10.1016/j.gpb.2020.10.005

NR 83

TC 6

Z9 6

U1 1

U2 9

PU ACADEMIC PRESS INC ELSEVIER SCIENCE

PI SAN DIEGO

PA 525 B ST, STE 1900, SAN DIEGO, CA 92101-4495 USA

SN 1521-6616

EI 1521-7035

J9 CLIN IMMUNOL

JI Clin. Immunol.

PD JAN

PY 2024

VL 258

AR 109802

DI 10.1016/j.clim.2023.109802

EA DEC 2023

PG 20

WC Immunology

WE Science Citation Index Expanded (SCI-EXPANDED)

SC Immunology

GA EB6Z6

UT WOS:001136501500001

PM 37866784

DA 2025-04-09

ER

PT J

AU Sahin, B

Ergul, M

AF Sahin, Bilal

Ergul, Mustafa

TI Captopril exhibits protective effects through anti-inflammatory and

anti-apoptotic pathways against hydrogen peroxide-induced oxidative

stress in C6 glioma cells

SO METABOLIC BRAIN DISEASE

LA English

DT Article

DE Captopril; Inflammation; Hydrogen Peroxide; Oxidative Stress; Apoptosis;

C6 cells

ID NF-KAPPA-B; RENIN-ANGIOTENSIN; ANTIOXIDANT; BRAIN; ACTIVATION;

INHIBITOR; NEURODEGENERATION; DETOXIFICATION; MECHANISMS; PHYSIOLOGY

AB Recent studies have shown that angiotensin-converting enzyme (ACE) inhibitors have reduced oxidative damage in the central nervous system (CNS). Accumulating evidence have also demonstrated that captopril, an ACE inhibitor, has protective effects on the CNS. However, its effects on hydrogen peroxide (H2O2)-induced oxidative damage in glial cells and interaction with the inflammatory system are still uncertain. Therefore, this study was aimed to investigate the protective effect of captopril on glial cell damage after H2O2-induced oxidative stress involved in the inflammatory and apoptotic pathways. The control group was without any treatment, and the H2O2 group was treated with 0.5 mM H2O2 for 24 h. The captopril group was treated with various concentrations of captopril for 24 h. The captopril +H2O2 group was pre-treated with captopril for 1 h and then exposed to 0.5 mM H2O2 for 24 h. In the captopril +H2O2 group, captopril at all concentrations significantly increased the cell viability in C6 cells. It also significantly increased the TAS and decreased the TOS levels which are an indicator of oxidative stress. Moreover, captopril significantly reduced the inflammation markers including NF-kB, IL-1 beta, COX-1, and COX-2 levels. Flow cytometry results also exhibited that captopril pretreatment significantly decreased the apoptosis rate. Besides, captopril significantly reduced apoptotic Bax and raised anti-apoptotic Bcl-2 protein levels. In conclusion, captopril has protective effects on C6 cells after H2O2-induced oxidative damage by inhibiting oxidative stress, inflammation, and apoptosis. However, further studies need to be conducted to evaluate the potential of captopril as a neuroprotective agent.

C1 [Sahin, Bilal] Sivas Cumhuriyet Univ, Sch Med, Dept Physiol, TR-58140 Sivas, Turkey.

[Ergul, Mustafa] Sivas Cumhuriyet Univ, Sch Pharm, Dept Biochem, TR-58140 Sivas, Turkey.

C3 Cumhuriyet University; Cumhuriyet University

RP Sahin, B (corresponding author), Sivas Cumhuriyet Univ, Sch Med, Dept Physiol, TR-58140 Sivas, Turkey.

EM bilalsahin@cumhuriyet.edu.tr

OI SAHIN, Bilal/0000-0002-4419-1385; Ergul, Mustafa/0000-0003-4303-2996

CR Abareshi A, 2017, INT J PREVENTIVE MED, V8, DOI 10.4103/ijpvm.IJPVM_322_16

Abbassi YA, 2016, ADV PHARM BULL, V6, P531, DOI 10.15171/apb.2016.067

Andersen JK, 2004, NAT MED, V10, pS18, DOI 10.1038/nrn1434

Asraf K, 2018, FRONT CELL NEUROSCI, V12, DOI 10.3389/fncel.2018.00116

Bhat SA, 2016, MOL NEUROBIOL, V53, P6950, DOI 10.1007/s12035-015-9584-5

Blesa J, 2015, FRONT NEUROANAT, V9, DOI 10.3389/fnana.2015.00091

Bonizzi G, 2004, TRENDS IMMUNOL, V25, P280, DOI 10.1016/j.it.2004.03.008

Boskabadi J, 2018, EXP LUNG RES, V44, P191, DOI 10.1080/01902148.2018.1473530

Cabezas R, 2012, NEUROSCI RES, V74, P80, DOI 10.1016/j.neures.2012.07.008

Constantinescu CS, 1998, IMMUNOL LETT, V62, P25, DOI 10.1016/S0165-2478(98)00025-X

COYLE JT, 1993, SCIENCE, V262, P689, DOI 10.1126/science.7901908

Cunningham C, 2013, GLIA, V61, P71, DOI 10.1002/glia.22350

Dielis AWJH, 2005, HYPERTENSION, V46, P1236, DOI 10.1161/01.HYP.0000193538.20705.23

Dringen R, 2005, J NEUROSCI RES, V79, P157, DOI 10.1002/jnr.20280

Dringen R, 1998, BRAIN RES PROTOC, V2, P223, DOI 10.1016/S1385-299X(97)00047-0

Eid BG, 2021, BIOMED PHARMACOTHER, V139, DOI 10.1016/j.biopha.2021.111670

El-Ashmawy NE, 2018, DIGEST DIS SCI, V63, P1497, DOI 10.1007/s10620-018-5036-1

Erel O, 2004, CLIN BIOCHEM, V37, P112, DOI 10.1016/j.clinbiochem.2003.10.014

Erel O, 2005, CLIN BIOCHEM, V38, P1103, DOI 10.1016/j.clinbiochem.2005.08.008

Ergul M, 2020, CHEM-BIOL INTERACT, V332, DOI 10.1016/j.cbi.2020.109288

Ergul M, 2019, ANTI-CANCER AGENT ME, V19, P1846, DOI 10.2174/1871520619666190618162828

Fernandez-Fernandez S, 2012, BIOCHEM J, V443, P3, DOI 10.1042/BJ20111943

Forman HJ, 2007, FREE RADICAL BIO MED, V42, P926, DOI 10.1016/j.freeradbiomed.2007.01.011

Forrester SJ, 2018, PHYSIOL REV, V98, P1627, DOI 10.1152/physrev.00038.2017

Gandhi S, 2012, OXID MED CELL LONGEV, V2012, DOI 10.1155/2012/428010

Garden GA, 2016, GLIA, V64, P1755, DOI 10.1002/glia.22998

Glass CK, 2010, CELL, V140, P918, DOI 10.1016/j.cell.2010.02.016

Halliwell B, 2006, J NEUROCHEM, V97, P1634, DOI 10.1111/j.1471-4159.2006.03907.x

Jensen CJ, 2013, J NEUROIMMUNE PHARM, V8, P824, DOI 10.1007/s11481-013-9480-6

Kurosaki R, 2005, EUR NEUROPSYCHOPHARM, V15, P57, DOI 10.1016/j.euroneuro.2004.05.007

Lawrence Toby, 2009, Cold Spring Harb Perspect Biol, V1, pa001651, DOI 10.1101/cshperspect.a001651

Liddelow SA, 2017, NATURE, V541, P481, DOI 10.1038/nature21029

Miners S, 2009, AM J TRANSL RES, V1, P163

Moon JH, 2019, MOL NEUROBIOL, V56, P4192, DOI 10.1007/s12035-018-1370-8

Odaka C, 2000, CLIN EXP IMMUNOL, V121, P515, DOI 10.1046/j.1365-2249.2000.01323.x

Paseban M, 2019, BIOMOLECULES, V9, DOI 10.3390/biom9040118

Pourahmad J, 2011, PESTIC BIOCHEM PHYS, V99, P105, DOI 10.1016/j.pestbp.2010.11.006

Quincozes-Santos A, 2013, PLOS ONE, V8, DOI 10.1371/journal.pone.0064372

Quincozes-Santos A, 2010, TOXICOL IN VITRO, V24, P916, DOI 10.1016/j.tiv.2009.11.016

Ray PD, 2012, CELL SIGNAL, V24, P981, DOI 10.1016/j.cellsig.2012.01.008

Rizor A, 2019, ANTIOXIDANTS-BASEL, V8, DOI 10.3390/antiox8080265

Saglam IY, 2013, TURK NEUROSURG, V23, P366, DOI 10.5137/1019-5149.JTN.7100-12.2

Sofroniew MV, 2010, ACTA NEUROPATHOL, V119, P7, DOI 10.1007/s00401-009-0619-8

Sofroniew MV, 2009, TRENDS NEUROSCI, V32, P638, DOI 10.1016/j.tins.2009.08.002

Tacconi MT, 1998, NEUROCHEM RES, V23, P759, DOI 10.1023/A:1022463527474

Takeuchi H., 2013, Clinical and Experimental Neuroimmunology, V4, P2

Taskiran AS, 2021, NEUROSCI LETT, V741, DOI 10.1016/j.neulet.2020.135504

Tastemur Y, 2020, TROP J PHARM RES, V19, P637, DOI 10.4314/tjpr.v19i3.26

Ulivi V, 2008, J CELL BIOCHEM, V104, P1393, DOI 10.1002/jcb.21717

Verkhratsky A, 2018, PHYSIOL REV, V98, P239, DOI 10.1152/physrev.00042.2016

Verkhratsky A, 2014, BIOCHEM SOC T, V42, P1291, DOI 10.1042/BST20140107

von Bohlen und Halbach O, 2006, CELL TISSUE RES, V326, P599, DOI 10.1007/s00441-006-0190-8

Wang Y, 2018, CLIN EXP PHARMACOL P, V45, P1135, DOI 10.1111/1440-1681.13001

NR 53

TC 9

Z9 9

U1 2

U2 15

PU SPRINGER/PLENUM PUBLISHERS

PI NEW YORK

PA 233 SPRING ST, NEW YORK, NY 10013 USA

SN 0885-7490

EI 1573-7365

J9 METAB BRAIN DIS

JI Metab. Brain Dis.

PD APR

PY 2022

VL 37

IS 4

BP 1221

EP 1230

DI 10.1007/s11011-022-00948-z

EA MAR 2022

PG 10

WC Endocrinology & Metabolism; Neurosciences

WE Science Citation Index Expanded (SCI-EXPANDED)

SC Endocrinology & Metabolism; Neurosciences & Neurology

GA 0U7LF

UT WOS:000768628800001

PM 35286533

DA 2025-04-09

ER

PT J

AU Kim, SH

Kwon, CH

Nakano, I

AF Kim, Sung-Hak

Kwon, Chang-Hyuk

Nakano, Ichiro

TI Detoxification of Oxidative Stress in Glioma Stem Cells: Mechanism,

Clinical Relevance, and Therapeutic Development

SO JOURNAL OF NEUROSCIENCE RESEARCH

LA English

DT Review

DE cancer stem cell; glioblastoma; oxidative stress; glioma stem cell;

reactive oxygen species

ID MITOCHONDRIAL ELECTRON-TRANSPORT; INTEGRATED GENOMIC ANALYSIS; HUMAN

LUNG-CANCER; GLIOBLASTOMA-MULTIFORME; BRAIN-TUMORS; OXYGEN;

PEROXIREDOXIN; CHEMOTHERAPY; SURVIVAL; GROWTH

AB Neural oncogenesis is currently incurable and invariably lethal. The development of innovative treatments for this devastating cancer will require a deeper molecular understanding of how cancer cells survive, proliferate, and escape from current therapies. In high-grade gliomas (HGGs), glioma stem cells (GSCs) may causally contribute to tumor initiation and propagation, therapeutic resistance, and subsequent recurrence of tumors. Within a tumor mass, GSCs are enriched in a hypoxic niche in which the oxidative stress levels are substantially elevated. Paradoxically, however, recent studies suggest that GSCs appear to generate less reactive oxygen species (ROS), a chemical component responsible for elevation of oxidative stress levels. To date, molecular mechanisms for how GSCs reduce oxidative stress to allow preferential survival in hypoxic areas in tumors remains elusive. This review article summarizes recent studies on the role of ROS-reducing enzymes, including peroxiredoxin 4, in detoxifying oxidative stress preferentially for GSCs in HGGs. In addition, the therapeutic potential of some of the recently identified antioxidant chemotherapeutic agents and avenues for future research in this area are discussed. (C) 2014 Wiley Periodicals, Inc.

C1 [Kim, Sung-Hak; Kwon, Chang-Hyuk; Nakano, Ichiro] Ohio State Univ, Dept Neurol Surg, Wexner Med Ctr, Dardinger Neurooncol Ctr, Columbus, OH 43210 USA.

[Kwon, Chang-Hyuk] Ohio State Univ, Solid Tumor Program, Wexner Med Ctr, Columbus, OH 43210 USA.

[Nakano, Ichiro] Ohio State Univ, James Comprehens Canc Ctr, Wexner Med Ctr, Columbus, OH 43210 USA.

C3 University System of Ohio; Ohio State University; University System of

Ohio; Ohio State University; James Cancer Hospital & Solove Research

Institute; University System of Ohio; Ohio State University

RP Nakano, I (corresponding author), OSUCCC, 385 Wiseman Hall, Columbus, OH 43210 USA.

EM Chang-Hyuk.Kwon@osumc.edu; Ichiro.nakano@osumc.edu

RI Wan, Kim/AAI-8744-2020; Nakano, Ichiro/AAR-9562-2020; Kwon,

Chang-hyuk/E-3450-2011

FU American Cancer Society; American Cancer Society [MRSG-08-108-01]; Ohio

State University (OSU) Comprehensive Cancer Center (CCC) Intramural

Research Program; OSU CCC Start-Up Fund; NIH [R21CA175875, R01NS087913,

R01NS083767, P01CA16320, R21CA135013]; American Brain Tumor Association

FX Contract grant sponsor: American Cancer Society ( to C.-H.K.); Contract

grant sponsor: American Cancer Society; Contract grant number:

MRSG-08-108-01 (to I.N.); Contract grant sponsor: Ohio State University

(OSU) Comprehensive Cancer Center (CCC) Intramural Research Program (to

C.-H.K.); Contract grant sponsor: OSU CCC Start-Up Fund (to C.-H.K.);

Contract grant sponsor: NIH; Contract grant number: R21CA175875;

Contract grant number: R01NS087913; Contract grant number: R01NS083767;

Contract grant number: P01CA16320; Contract grant number: R21CA135013

(to I.N.); Contract grant sponsor: American Brain Tumor Association (to

I.N).

CR Acharya A, 2010, OXID MED CELL LONGEV, V3, P23, DOI 10.4161/oxim.3.1.10095

Ayuso-Sacido Angel, 2006, Curr Stem Cell Res Ther, V1, P387

Badr CE, 2013, JNCI-J NATL CANCER I, V105, P643, DOI 10.1093/jnci/djt037

Bao SD, 2006, NATURE, V444, P756, DOI 10.1038/nature05236

Bezerra DP, 2008, J APPL TOXICOL, V28, P599, DOI 10.1002/jat.1311

Brennan C, 2009, PLOS ONE, V4, DOI 10.1371/journal.pone.0007752

Cabarcas SM, 2011, INT J CANCER, V129, P2315, DOI 10.1002/ijc.26312

Chang KP, 2011, J PROTEOME RES, V10, P4935, DOI 10.1021/pr200311p

Chen J, 2012, NATURE, V488, P522, DOI 10.1038/nature11287

Chen TC, 2011, CANCER LETT, V302, P100, DOI 10.1016/j.canlet.2010.11.008

Chin L, 2008, NATURE, V455, P1061, DOI 10.1038/nature07385

Eramo A, 2006, CELL DEATH DIFFER, V13, P1238, DOI 10.1038/sj.cdd.4401872

Fang YZ, 2002, NUTRITION, V18, P872, DOI 10.1016/S0899-9007(02)00916-4

Golovine KV, 2013, PROSTATE, V73, P23, DOI 10.1002/pros.22535

Han SS, 2013, LEUKEMIA RES, V37, P146, DOI 10.1016/j.leukres.2012.11.009

Iuchi Y, 2009, BIOCHEM J, V419, P149, DOI 10.1042/BJ20081526

Jacinto FV, 2007, DNA REPAIR, V6, P1155, DOI 10.1016/j.dnarep.2007.03.013

Jijiwa M, 2011, PLOS ONE, V6, DOI 10.1371/journal.pone.0024217

Karihtala P, 2011, BMC CANCER, V11, DOI 10.1186/1471-2407-11-262

Kim TH, 2014, NEURO-ONCOLOGY, V16, P1354, DOI 10.1093/neuonc/nou088

Kim TH, 2014, PLOS ONE, V7

Kleihues PCW., 2000, WORLD HLTH ORG CLASS

Kwon CH, 2008, CANCER RES, V68, P3286, DOI 10.1158/0008-5472.CAN-07-6867

Lathia JD, 2011, CELL STEM CELL, V8, P482, DOI 10.1016/j.stem.2011.04.013

Lee J, 2006, CANCER CELL, V9, P391, DOI 10.1016/j.ccr.2006.03.030

Li MH, 2004, INT J ONCOL, V24, P305

Lin CJ, 2012, PLOS ONE, V7, DOI 10.1371/journal.pone.0038706

Liu HL, 2013, J CHROMATOGR B, V928, P78, DOI 10.1016/j.jchromb.2013.03.021

Liu JM, 2013, BIOCHEM BIOPH RES CO, V437, P87, DOI 10.1016/j.bbrc.2013.06.042

Liu XD, 2009, CANCER INVEST, V27, P345, DOI 10.1080/07357900802438577

Martin KR, 2002, HUM EXP TOXICOL, V21, P71, DOI 10.1191/0960327102ht213oa

Martinez-Outschoorn UE, 2010, CELL CYCLE, V9, P3515, DOI 10.4161/cc.9.17.12928

Miyazaki T, 2012, CLIN CANCER RES, V18, P1268, DOI 10.1158/1078-0432.CCR-11-1795

Mohyeldin A, 2010, CELL STEM CELL, V7, P150, DOI 10.1016/j.stem.2010.07.007

Nakano I, 2008, J NEUROSCI RES, V86, P48, DOI 10.1002/jnr.21471

Nakano I, 2011, NEURO-ONCOLOGY, V13, P622, DOI 10.1093/neuonc/nor023

Oliva CR, 2011, PLOS ONE, V6, DOI 10.1371/journal.pone.0024665

Paradies G, 2000, FEBS LETT, V466, P323, DOI 10.1016/S0014-5793(00)01082-6

Park HJ, 2008, J PROTEOME RES, V7, P1138, DOI 10.1021/pr7007237

Parsons DW, 2008, SCIENCE, V321, P1807, DOI 10.1126/science.1164382

Pennington JD, 2005, DRUG RESIST UPDATE, V8, P322, DOI 10.1016/j.drup.2005.09.002

Phillips HS, 2006, CANCER CELL, V9, P157, DOI 10.1016/j.ccr.2006.02.019

Prados MD, 1998, SEMIN SURG ONCOL, V14, P88

Pritchard C, 2009, CANCER RES, V69, P1739, DOI 10.1158/0008-5472.CAN-07-6817

Raj L, 2011, NATURE, V475, P231, DOI 10.1038/nature10167

Santos CXC, 2009, ANTIOXID REDOX SIGN, V11, P2409, DOI [10.1089/ars.2009.2625, 10.1089/ARS.2009.2625]

Shi XK, 2012, ANTIOXID REDOX SIGN, V16, P1215, DOI 10.1089/ars.2012.4529

Stupp R, 2005, NEW ENGL J MED, V352, P987, DOI 10.1056/NEJMoa043330

Tavender TJ, 2010, J CELL SCI, V123, P2672, DOI 10.1242/jcs.067843

Venere M, 2011, GLIA, V59, P1148, DOI 10.1002/glia.21185

Verhaak RGW, 2010, CANCER CELL, V17, P98, DOI 10.1016/j.ccr.2009.12.020

Visnyei K, 2011, MOL CANCER THER, V10, P1818, DOI 10.1158/1535-7163.MCT-11-0268

Vlashi E, 2011, P NATL ACAD SCI USA, V108, P16062, DOI 10.1073/pnas.1106704108

Wei QO, 2011, P NATL ACAD SCI USA, V108, P7004, DOI 10.1073/pnas.1013012108

Wellen KE, 2010, MOL CELL, V40, P323, DOI 10.1016/j.molcel.2010.10.004

Yuan Y, 2012, CNS NEUROSCI THER, V18, P536, DOI 10.1111/j.1755-5949.2012.00319.x

Zhou BBS, 2009, NAT REV DRUG DISCOV, V8, P806, DOI 10.1038/nrd2137

Zito E, 2010, MOL CELL, V40, P787, DOI 10.1016/j.molcel.2010.11.010

NR 58

TC 40

Z9 45

U1 2

U2 63

PU WILEY-BLACKWELL

PI HOBOKEN

PA 111 RIVER ST, HOBOKEN 07030-5774, NJ USA

SN 0360-4012

EI 1097-4547

J9 J NEUROSCI RES

JI J. Neurosci. Res.

PD NOV

PY 2014

VL 92

IS 11

BP 1419

EP 1424

DI 10.1002/jnr.23431

PG 6

WC Neurosciences

WE Science Citation Index Expanded (SCI-EXPANDED)

SC Neurosciences & Neurology

GA AN4CJ

UT WOS:000340534900001

PM 25043479

DA 2025-04-09

ER

PT J

AU Tong, S

Xia, MQ

Xu, Y

Sun, Q

Ye, LG

Yuan, FE

Wang, YX

Cai, JY

Ye, Z

Tian, DF

AF Tong, Shiao

Xia, Minqi

Xu, Yang

Sun, Qian

Ye, Liguo

Yuan, Fanen

Wang, Yixuan

Cai, Jiayang

Ye, Zhang

Tian, Daofeng

TI Identification and validation of a novel prognostic signature based on

mitochondria and oxidative stress related genes for glioblastoma

SO JOURNAL OF TRANSLATIONAL MEDICINE

LA English

DT Article

DE Glioblastoma; Prognosis; Risk score; Mitochondria; Oxidative stress

ID MTH1; DYSFUNCTION

AB BackgroundMitochondria represent a major source of reactive oxygen species (ROS) in cells, and the direct increase in ROS content is the primary cause of oxidative stress, which plays an important role in tumor proliferation, invasion, angiogenesis, and treatment. However, the relationship between mitochondrial oxidative stress-related genes and glioblastoma (GBM) remains unclear. This study aimed to investigate the value of mitochondria and oxidative stress-related genes in the prognosis and therapeutic targets of GBM.MethodsWe retrieved mitochondria and oxidative stress-related genes from several public databases. The LASSO regression and Cox analyses were utilized to build a risk model and the ROC curve was used to assess its performance. Then, we analyzed the correlation between the model and immunity and mutation. Furthermore, CCK8 and EdU assays were utilized to verify the proliferative capacity of GBM cells and flow cytometry was used to analyze apoptosis rates. Finally, the JC-1 assay and ATP levels were utilized to detect mitochondrial function, and the intracellular ROS levels were determined using MitoSOX and BODIPY 581/591 C11.Results5 mitochondrial oxidative stress-related genes (CTSL, TXNRD2, NUDT1, STOX1, CYP2E1) were screened by differential expression analysis and Cox analysis and incorporated in a risk model which yielded a strong prediction accuracy (AUC value = 0.967). Furthermore, this model was strongly related to immune cell infiltration and mutation status and could identify potential targeted therapeutic drugs for GBM. Finally, we selected NUDT1 for further validation in vitro. The results showed that NUDT1 was elevated in GBM, and knockdown of NUDT1 inhibited the proliferation and induced apoptosis of GBM cells, while knockdown of NUDT1 damaged mitochondrial homeostasis and induced oxidative stress in GBM cells.ConclusionOur study was the first to propose a prognostic model of mitochondria and oxidative stress-related genes, which provided potential therapeutic strategies for GBM patients.

C1 [Tong, Shiao; Xu, Yang; Sun, Qian; Ye, Liguo; Yuan, Fanen; Wang, Yixuan; Cai, Jiayang; Ye, Zhang; Tian, Daofeng] Wuhan Univ, Dept Neurosurg, Renmin Hosp, Wuhan, Peoples R China.

[Xia, Minqi] Wuhan Univ, Dept Endocrinol & Metab, Renmin Hosp, Wuhan, Peoples R China.

C3 Wuhan University; Wuhan University

RP Tian, DF (corresponding author), Wuhan Univ, Dept Neurosurg, Renmin Hosp, Wuhan, Peoples R China.

EM tiandaofeng@hotmail.com

RI sun, qian/JMQ-8920-2023; wang, yixuan/JGM-3893-2023

OI tian, daofeng/0000-0002-2023-0060; Yuan, Fanen/0000-0001-9484-5183

CR Aird KM, 2012, BREAST CANCER RES TR, V132, P109, DOI 10.1007/s10549-011-1568-1

Apostolova N, 2015, ANTIOXID REDOX SIGN, V22, P686, DOI 10.1089/ars.2014.5952

Brown TJ, 2016, JAMA ONCOL, V2, P1460, DOI 10.1001/jamaoncol.2016.1373

Chang CH, 2021, J EXP CLIN CANC RES, V40, DOI 10.1186/s13046-021-01960-4

Chin L, 2008, NATURE, V455, P1061, DOI 10.1038/nature07385

Corsello SM, 2020, NAT CANCER, V1, P235, DOI 10.1038/s43018-019-0018-6

Dai HM, 2023, CELL DEATH DIFFER, V30, P794, DOI 10.1038/s41418-022-01083-z

de Sa PL, 2017, OXID MED CELL LONGEV, V2017, DOI 10.1155/2017/2467940

Dong Q, 2022, J CANCER RES CLIN, V148, P599, DOI 10.1007/s00432-021-03843-9

Doridot L, 2014, ANTIOXID REDOX SIGN, V21, P819, DOI 10.1089/ars.2013.5661

Fan KX, 2022, OXID MED CELL LONGEV, V2022, DOI 10.1155/2022/5652586

Friedman JR, 2014, NATURE, V505, P335, DOI 10.1038/nature12985

Gatenby RA, 2004, NAT REV CANCER, V4, P891, DOI 10.1038/nrc1478

Gencheva R, 2022, ANNU REV PHARMACOL, V62, P177, DOI 10.1146/annurev-pharmtox-052220-102509

Haorah J, 2008, FREE RADICAL BIO MED, V45, P1542, DOI 10.1016/j.freeradbiomed.2008.08.030

Hsu CC, 2016, EXP BIOL MED, V241, P1281, DOI 10.1177/1535370216641787

Huang CZ, 2022, CANCERS, V14, DOI 10.3390/cancers14153735

Jin FQ, 2021, OPEN LIFE SCI, V16, P1164, DOI 10.1515/biol-2021-0119

Kalpathy-Cranner J, 2014, CANCER RES, V74, P4622, DOI 10.1158/0008-5472.CAN-14-0383

Kim MR, 2003, BIOCHEM BIOPH RES CO, V304, P119, DOI 10.1016/S0006-291X(03)00547-3

Kim R, 2006, IMMUNOLOGY, V119, P254, DOI 10.1111/j.1365-2567.2006.02430.x

Kirkpatrick JP, 2014, SEMIN RADIAT ONCOL, V24, P289, DOI 10.1016/j.semradonc.2014.06.006

Liu Xia, 2015, Asian Pac J Cancer Prev, V16, P6201

Lu HQ, 2013, AUTOPHAGY, V9, P1720, DOI 10.4161/auto.26550

Luo WX, 2022, GENET RES, V2022, DOI 10.1155/2022/2249909

McKinnon C, 2021, BMJ-BRIT MED J, V374, DOI 10.1136/bmj.n1560

Nair GG, 2019, NAT CELL BIOL, V21, P792, DOI 10.1038/s41556-019-0316-3

Nakabeppu Y, 2006, DNA REPAIR, V5, P761, DOI 10.1016/j.dnarep.2006.03.003

Ning WC, 2022, FRONT IMMUNOL, V13, DOI 10.3389/fimmu.2022.862049

Olar A, 2014, J PATHOL, V232, P165, DOI 10.1002/path.4282

Park W, 2018, BIOCHEM BIOPH RES CO, V503, P3155, DOI 10.1016/j.bbrc.2018.08.109

Rath S, 2021, NUCLEIC ACIDS RES, V49, pD1541, DOI 10.1093/nar/gkaa1011

Ritchie ME, 2015, NUCLEIC ACIDS RES, V43, DOI 10.1093/nar/gkv007

Safran M, 2010, DATABASE-OXFORD, DOI 10.1093/database/baq020

Shergalis A, 2018, PHARMACOL REV, V70, P412, DOI 10.1124/pr.117.014944

Shi HW, 2021, FRONT PHARMACOL, V12, DOI 10.3389/fphar.2021.733805

Shu W, 2023, COMB CHEM HIGH T SCR, V26, P1488, DOI 10.2174/1386207325666221005122554

Tai SH, 2021, TRANSL CANCER RES, V10, P3906, DOI 10.21037/tcr-20-3426

Tang Kailin, 2021, Nucleic Acids Res, V49, pe99, DOI 10.1093/nar/gkab554

Tilokani L, 2018, ESSAYS BIOCHEM, V62, P341, DOI 10.1042/EBC20170104

Tu YY, 2016, AM J TRANSL RES, V8, P2803

Wang J, 2020, PHARMACOL RES, V156, DOI 10.1016/j.phrs.2020.104771

Wang Y, 2022, HELIYON, V8, DOI 10.1016/j.heliyon.2022.e11343

Wang YY, 2015, NEURO-ONCOLOGY, V17, P282, DOI 10.1093/neuonc/nou130

Witthayanuwat Supapan, 2018, Asian Pac J Cancer Prev, V19, P2613

Wu L, 2015, CHEM SOC REV, V44, P2963, DOI 10.1039/c4cs00370e

Wu Y, 2021, OXID MED CELL LONGEV, V2021, DOI 10.1155/2021/9939331

Ye LG, 2021, CANCER MED-US, V10, P8100, DOI 10.1002/cam4.4320

Yu YW, 2021, J PHARM SCI-US, V110, P3431, DOI 10.1016/j.xphs.2021.06.025

Zhan DK, 2020, CANCER BIOTHER RADIO, V35, P223, DOI 10.1089/cbr.2019.3031

Zhang Q, 2020, CELL DEATH DIS, V11, DOI 10.1038/s41419-020-2696-5

Zhao Z, 2017, SCI DATA, V4, DOI 10.1038/sdata.2017.24

Zhou SG, 2022, FRONT ONCOL, V12, DOI 10.3389/fonc.2022.1049773

NR 53

TC 12

Z9 13

U1 1

U2 13

PU BMC

PI LONDON

PA CAMPUS, 4 CRINAN ST, LONDON N1 9XW, ENGLAND

EI 1479-5876

J9 J TRANSL MED

JI J. Transl. Med.

PD FEB 22

PY 2023

VL 21

IS 1

AR 136

DI 10.1186/s12967-023-03970-6

PG 18

WC Medicine, Research & Experimental

WE Science Citation Index Expanded (SCI-EXPANDED)

SC Research & Experimental Medicine

GA 9Q4NS

UT WOS:000944943500003

PM 36814293

OA gold, Green Published

DA 2025-04-09

ER

PT J

AU Wu, LQ

Wang, F

Xu, A

Chen, ZB

AF Wu, Liquan

Wang, Fang

Xu, Iang

Chen, Zhibiao

TI PTPN2 induced by inflammatory response and oxidative stress contributed

to glioma progression

SO JOURNAL OF CELLULAR BIOCHEMISTRY

LA English

DT Article

DE glioma; H2O2; IFN-gamma; PTPN2; TNF-alpha

ID PROTEIN-TYROSINE-PHOSPHATASE; CELL-DEATH; INTESTINAL INFLAMMATION;

NONRECEPTOR TYPE-2; ACTIVATION; GROWTH; ROS

AB Malignant glioma remains the most frequent form of primary brain tumors all over the world. The gliomagenesis is characterized by various molecular processes such as neoplastic transformation, dysregulation of the cell cycle, and angiogenesis. Among these biomolecular events, the existence of inflammation and oxidative stress pathways in the development of glioma has been reported. PTPN2 is associated with several inflammatory disorders. However, the biological role of PTPN2 in inflammation responses and oxidative stress pathways involved in glioma remains poorly known. Here, we focused on its function in glioma development. Here, we observed that PTPN2 was significantly increased in glioma especially in a grade-dependent manner. Meanwhile, interferon-gamma and tumor necrosis factor-alpha, which have been identified as crucial inflammation cytokines, were able to trigger PTPN2 expression in a dose-dependent course in T98G cells. Then, we found that PTPN2 was oxidated and inactivated by H2O2. Meanwhile, H2O2 induced glioma cell colony formation capacity and increased ki-67 expression confirmed by flow cytometry assay. Finally, T98G cells were transfected with PTPN2 shRNA and it was shown that knockdown of PTPN2 obviously inhibited T98G cell colony formation and induced cell apoptosis. In summary, our findings indicated that PTPN2 could be induced by inflammatory response and oxidative stress and its deficiency depressed glioma cell growth.

C1 [Wu, Liquan; Chen, Zhibiao] Wuhan Univ, Renmin Hosp, Dept Neurosurg, Wuhan, Hubei, Peoples R China.

[Wang, Fang] Huazhong Univ Sci & Technol, Tongji Med Coll, Wuhan, Hubei, Peoples R China.

[Xu, Iang] Xuzhou Med Univ, Affiliated Hosp, Huaian Peoples Hosp 2, Dept Rehabil, Huaian, Peoples R China.

C3 Wuhan University; Huazhong University of Science & Technology; Xuzhou

Medical University

RP Wu, LQ; Chen, ZB (corresponding author), Wuhan Univ, Renmin Hosp, Dept Neurosurg, Wuhan, Hubei, Peoples R China.

EM wuliquan@whu.edu.cn; chzbiao@126.com

RI wang, fang/GYD-4295-2022; chen, Zhibiao/W-4719-2019

CR [Anonymous], OXID MED CELL LONGEV

Aradi B, 2015, ARTHRITIS RHEUMATOL, V67, P2624, DOI 10.1002/art.39256

Chen J, 2012, NATURE, V488, P522, DOI 10.1038/nature11287

Del Vecchio CA, 2012, EXPERT REV VACCINES, V11, P133, DOI [10.1586/erv.11.177, 10.1586/ERV.11.177]

Feng Y, 2017, SCI REP-UK, V7, DOI 10.1038/s41598-017-00850-0

Fuller GN, 2007, BRAIN PATHOL, V17, P304, DOI 10.1111/j.1750-3639.2007.00084.x

Ishii M, 2007, ANTICANCER RES, V27, P3987

Karlsson E, 2015, BREAST CANCER RES TR, V153, P31, DOI 10.1007/s10549-015-3516-y

Kim JH, 2013, CELL DEATH DIS, V4, DOI 10.1038/cddis.2013.117

Kleppe M, 2011, HAEMATOL-HEMATOL J, V96, P1723, DOI 10.3324/haematol.2011.041921

Kros JM, 2015, NEURO-ONCOLOGY, V17, P343, DOI 10.1093/neuonc/nou207

Lee YW, 2001, NEUROCHEM RES, V26, P337, DOI 10.1023/A:1010993428770

Li LL, 2015, CELL MOL NEUROBIOL, V35, P615, DOI 10.1007/s10571-015-0166-x

Li S, 2017, REDOX REP, V22, P190, DOI 10.1080/13510002.2016.1173327

Liu XR, 2015, EUR REV MED PHARMACO, V19, P4068

Louis DN, 2007, ACTA NEUROPATHOL, V114, P97, DOI [10.1007/s00401-007-0243-4, DOI 10.1007/s00401-007-0243-4]

Ludwig K, 2017, J NEURO-ONCOL, V134, P505, DOI 10.1007/s11060-017-2379-y

Manguso RT, 2017, NATURE, V547, P413, DOI 10.1038/nature23270

Raza MH, 2017, J CANCER RES CLIN, V143, P1789, DOI 10.1007/s00432-017-2464-9

Rinaldi M, 2016, INT J MOL SCI, V17, DOI 10.3390/ijms17060984

Scharl M, 2011, GUT, V60, P189, DOI 10.1136/gut.2010.216606

Scharl M, 2010, INFLAMM BOWEL DIS, V16, P2055, DOI 10.1002/ibd.21325

Spalinger MR, 2015, MUCOSAL IMMUNOL, V8, P918, DOI 10.1038/mi.2014.122

Spalinger MR, 2018, MOL CELL ONCOL, V5, DOI 10.1080/23723556.2018.1465013

Spalinger MR, 2018, CELL REP, V22, P1835, DOI 10.1016/j.celrep.2018.01.052

Van Meir EG, 2010, CA-CANCER J CLIN, V60, P166, DOI 10.3322/caac.20069

Wang PF, 2018, J NEUROINFLAMM, V15, DOI 10.1186/s12974-018-1187-4

Wiede F, 2011, J CLIN INVEST, V121, P4758, DOI 10.1172/JCI59492

Zhang L, 2013, J CANCER RES CLIN, V139, P719, DOI 10.1007/s00432-013-1387-3

NR 29

TC 21

Z9 24

U1 0

U2 27

PU WILEY

PI HOBOKEN

PA 111 RIVER ST, HOBOKEN 07030-5774, NJ USA

SN 0730-2312

EI 1097-4644

J9 J CELL BIOCHEM

JI J. Cell. Biochem.

PD NOV

PY 2019

VL 120

IS 11

BP 19044

EP 19051

DI 10.1002/jcb.29227

PG 8

WC Biochemistry & Molecular Biology; Cell Biology

WE Science Citation Index Expanded (SCI-EXPANDED)

SC Biochemistry & Molecular Biology; Cell Biology

GA IX9JQ

UT WOS:000486004800046

PM 31241223

DA 2025-04-09

ER

PT J

AU Cholia, RP

Dhiman, M

Kumar, R

Mantha, AK

AF Cholia, Ravi P.

Dhiman, Monisha

Kumar, Raj

Mantha, Anil K.

TI Oxidative stress stimulates invasive potential in rat C6 and human U-87

MG glioblastoma cells via activation and cross-talk between PKM2, ENPP2

and APE1 enzymes

SO METABOLIC BRAIN DISEASE

LA English

DT Article

DE GBM; ROS; APE1; PKM2; ENPP2

ID LYSOPHOSPHATIDIC ACID LPA; APURINIC/APYRIMIDINIC ENDONUCLEASE ACTIVITY;

PYRUVATE-KINASE; CANCER CELLS; LYSOPHOSPHOLIPASE-D; AUTOTAXIN;

APE1/REF-1; DNA; PROTEIN; GROWTH

AB Maintaining genomic integrity is essential for cell survival and viability. Reactive oxygen species (ROS) overproduction results in oxidative stress leading to the genomic instability via generation of small base lesions in DNA and these unrepaired DNA damages lead to various cellular consequences including cancer. Recent data support the concept "oxidative stress is an indispensable participant in fostering proliferation, survival, and migration" in various cancer cell types including glioblastoma cells. In this study we demonstrate that treatment of non-cytotoxic doses of oxidants such as amyloid beta [A beta(25-35)] peptide, glucose oxidase (GO), and hydrogen peroxide (H2O2) for 24 h and 48 h time points found to increase the expression level and activity of a multifunctional enzyme Apurinic/apyrimidinic endonuclease (APE1), a key enzyme of base excision repair (BER) pathway which takes care of base damages; and also resulted in modulation in the expression levels of downstream BER-pathway enzymes viz. PARP-1, XRCC1, DNA pol beta, and ligase III alpha was observed upon oxidative stress in C6 and U-87 MG cells. Oxidants treatment to the C6 and U-87 MG cells also resulted in an elevation in the intracellular expression of glycolytic pathway enzyme Pyruvate kinase M2 (PKM2) and the metastasis inducer protein Ectonucleotide pyrophosphatase/phosphodiesterase 2 (ENPP2) as analyzed using Western blotting and Immunofluorescence microscopic studies. Our study also reports that oxidative stress induced for 24 h and 48 h in C6 and U-87 MG cells resulted in extracellular secretion of APE1 and ENPP2 as analyzed using Western blotting in conditioned media. However, the biological significance of extracellular secreted APE1 remains elusive. Oxidative stress also elevated the ENPP2's LysoPLD activity in conditioned media of C6 and U-87 MG cells. Our results also demonstrate that oxidative stress affects the expression level and localization of APE1, PKM2, and ENPP2 in C6 and U-87 MG cells. As evidenced by the colocalization pattern at 24 h and 48 h time points, it can be attributed that oxidative stress mediates crosstalk between APE1, PKM2, and ENPP2. In addition, when C6 and U-87 MG cells were treated with lysophosphatidic acid (LPA), a bioactive lipid that negatively regulates ENPP2's LysoPLD activity at 10 mu M concentration, demonstrated strong migratory potential in C6 and U-87 MG cells, and also induced migration upon oxidative stress. Altogether, the findings demonstrate the potential of C6 and U-87 MG cells to utilize three proteins viz. APE1, PKM2, and ENPP2 towards migration and survival of gliomas. Thus the knowledge on oxidative stress induced APE1's interaction with PKM2 and ENPP2 opens a new channel for the therapeutic target(s) for gliomas.

C1 [Cholia, Ravi P.; Mantha, Anil K.] Cent Univ Punjab, Dept Anim Sci, Sch Basic & Appl Sci, Bathinda 151001, Punjab, India.

[Dhiman, Monisha] Cent Univ Punjab, Dept Biochem & Microbial Sci, Sch Basic & Appl Sci, Bathinda, Punjab, India.

[Kumar, Raj] Cent Univ Punjab, Sch Basic & Appl Sci, Dept Pharmaceut Sci & Nat Prod, Bathinda, Punjab, India.

C3 Central University of Punjab; Central University of Punjab; Central

University of Punjab

RP Mantha, AK (corresponding author), Cent Univ Punjab, Dept Anim Sci, Sch Basic & Appl Sci, Bathinda 151001, Punjab, India.

EM anilmantha@gmail.com

RI Kumar, Raj/AAY-1522-2021; Mantha, Anil/AAM-5035-2021; Cholia,

Ravi/AAH-8077-2021

OI Cholia, Ravi/0000-0002-6694-3955; Dhiman, Monisha/0000-0001-5923-3384; ,

Raj/0000-0001-5113-6627

FU BSR-startup grant received from the University Grants Commission (UGC),

New Delhi, India; Central University of Punjab, Bathinda (CUPB); Indian

Council for Medical Research (ICMR), New Delhi, India

FX This work is supported to A.K.M. by the BSR-startup grant received from

the University Grants Commission (UGC), New Delhi, India, and the funds

received under the scheme Research Seed Money (RSM) from the Central

University of Punjab, Bathinda (CUPB). R.P.C. acknowledges financial

support in the form of a senior research fellowship (SRF) from the

Indian Council for Medical Research (ICMR), New Delhi, India. The

confocal laser scanning microscope (Olympus) facility of the Central

Instrumentation Laboratory (CIL), CUPB is thankfully acknowledged.

Because of the limited focus of the article, many relevant and

appropriate references could not be included, for which the authors

apologize.

CR Alía M, 2006, TOXICOL APPL PHARM, V212, P110, DOI 10.1016/j.taap.2005.07.014

Awada R, 2012, FREE RADICAL BIO MED, V52, P516, DOI 10.1016/j.freeradbiomed.2011.11.014

Babu R, 2016, J NEUROSURG, V124, P998, DOI 10.3171/2015.4.JNS142200

Benesch MGK, 2015, J LIPID RES, V56, P1134, DOI 10.1194/jlr.M057661

Benesch MGK, 2014, FEBS LETT, V588, P2712, DOI 10.1016/j.febslet.2014.02.009

Bhakat KK, 2009, ANTIOXID REDOX SIGN, V11, P621, DOI [10.1089/ars.2008.2198, 10.1089/ARS.2008.2198]

Bobola MS, 2001, CLIN CANCER RES, V7, P3510

Capdevila C, 2017, J CELL PHYSIOL, V232, P1596, DOI 10.1002/jcp.25502

Chaneton B, 2012, TRENDS BIOCHEM SCI, V37, P309, DOI 10.1016/j.tibs.2012.04.003

CHEN DS, 1991, NUCLEIC ACIDS RES, V19, P5907, DOI 10.1093/nar/19.21.5907

Choi EO, 2016, INT J MOL MED, V37, P798, DOI 10.3892/ijmm.2016.2460

Choi S, 2013, BIOCHEM BIOPH RES CO, V435, P403, DOI 10.1016/j.bbrc.2013.04.101

Cholia RP, 2017, METAB BRAIN DIS, V32, P1705, DOI 10.1007/s11011-017-0057-6

Cortés-Cros M, 2013, P NATL ACAD SCI USA, V110, P489, DOI 10.1073/pnas.1212780110

Costa B, 2013, PLOS ONE, V8, DOI 10.1371/journal.pone.0072281

Dai R., 2009, CHIN GER J CLIN ONCO, V8, P110

Desai S, 2014, ONCOTARGET, V5, P8202, DOI 10.18632/oncotarget.1159

Desmaret S, 2005, BIOL CHEM, V386, P1137, DOI 10.1515/BC.2005.130

Dhiman M, 2012, PLOS ONE, V7, DOI 10.1371/journal.pone.0028449

Duthie SJ, 1997, MUTAT RES-GEN TOX EN, V393, P223, DOI 10.1016/S1383-5718(97)00107-1

Fukushima N, 2000, DEV BIOL, V228, P6, DOI 10.1006/dbio.2000.9930

Harris I, 2012, CELL RES, V22, P447, DOI 10.1038/cr.2011.203

Hausmann Jens, 2013, Advances in Biological Regulation, V53, P112, DOI 10.1016/j.jbior.2012.09.010

Hoelzinger DB, 2008, J NEURO-ONCOL, V86, P297, DOI 10.1007/s11060-007-9480-6

Hu YL, 2001, J NATL CANCER I, V93, P762, DOI 10.1093/jnci/93.10.762

Johannessen TCA, 2012, EXPERT REV ANTICANC, V12, P635, DOI [10.1586/ERA.12.37, 10.1586/era.12.37]

Katsifa A, 2015, PLOS ONE, V10, DOI 10.1371/journal.pone.0143083

Kelman Z, 1997, ONCOGENE, V14, P629, DOI 10.1038/sj.onc.1200886

Kishi Y, 2006, J BIOL CHEM, V281, P17492, DOI 10.1074/jbc.M601803200

Lee YR, 2015, ONCOTARGET, V6, P23383, DOI 10.18632/oncotarget.4345

Liang J, 2017, CELL RES, V27, P329, DOI 10.1038/cr.2016.159

Mantha AK, 2012, J NEUROSCI RES, V90, P1230, DOI 10.1002/jnr.23018

Mazurek S., 2005, SEM CANC BIOL

Mazurek S, 2011, INT J BIOCHEM CELL B, V43, P969, DOI 10.1016/j.biocel.2010.02.005

Montaldi AP, 2015, MUTAT RES-GEN TOX EN, V793, P19, DOI 10.1016/j.mrgentox.2015.06.001

Mukherjee J, 2013, PLOS ONE, V8, DOI 10.1371/journal.pone.0057610

Nath S, 2017, CELL SIGNAL, V39, P18, DOI 10.1016/j.cellsig.2017.07.019

Redaelli A, 1998, TERATOGEN CARCIN MUT, V18, P17, DOI 10.1002/(SICI)1520-6866(1998)18:1<17::AID-TCM3>3.3.CO;2-I

Sabarinathan D, 2011, CHEM-BIOL INTERACT, V189, P26, DOI 10.1016/j.cbi.2010.09.028

Schleicher SM, 2011, PLOS ONE, V6, DOI 10.1371/journal.pone.0022182

Shida D, 2003, CANCER RES, V63, P1706

Silber JR, 2002, CLIN CANCER RES, V8, P3008

Singh S, 2012, FREE RADICAL BIO MED, V53, P1782, DOI 10.1016/j.freeradbiomed.2012.07.025

Sosa V, 2013, AGEING RES REV, V12, P376, DOI 10.1016/j.arr.2012.10.004

Tamada M., 2012, PYRUVATE KINASE M2 M

Tell G, 2010, CELL MOL LIFE SCI, V67, P3589, DOI 10.1007/s00018-010-0486-4

Tell G, 2009, ANTIOXID REDOX SIGN, V11, P601, DOI 10.1089/ars.2008.2194

Umezu-Goto M, 2002, J CELL BIOL, V158, P227, DOI 10.1083/jcb.200204026

Valko M, 2006, CHEM-BIOL INTERACT, V160, P1, DOI 10.1016/j.cbi.2005.12.009

Wrensch M, 2002, NEURO-ONCOLOGY, V4, P278, DOI 10.1093/neuonc/4.4.278

Wu JM, 2010, MOL CANCER, V9, DOI 10.1186/1476-4598-9-71

Yamada T, 2004, J BIOL CHEM, V279, P6595, DOI 10.1074/jbc.M308133200

Yang WW, 2013, CELL CYCLE, V12, P3154, DOI 10.4161/cc.26182

Ye XY, 2017, ONCOTARGET, V8, P6984, DOI 10.18632/oncotarget.14346

NR 54

TC 27

Z9 28

U1 1

U2 9

PU SPRINGER/PLENUM PUBLISHERS

PI NEW YORK

PA 233 SPRING ST, NEW YORK, NY 10013 USA

SN 0885-7490

EI 1573-7365

J9 METAB BRAIN DIS

JI Metab. Brain Dis.

PD AUG

PY 2018

VL 33

IS 4

BP 1307

EP 1326

DI 10.1007/s11011-018-0233-3

PG 20

WC Endocrinology & Metabolism; Neurosciences

WE Science Citation Index Expanded (SCI-EXPANDED)

SC Endocrinology & Metabolism; Neurosciences & Neurology

GA GL7QG

UT WOS:000437396300030

PM 29721771

DA 2025-04-09

ER

PT J

AU Saurty-Seerunghen, MS

Daubon, T

Bellenger, L

Delaunay, V

Castro, G

Guyon, J

Rezk, A

Fabrega, S

Idbaih, A

Almairac, F

Burel-Vandenbos, F

Turchi, L

Virolle, T

Peyrin, JM

Antoniewski, C

Chneiweiss, H

Junier, MP

El-Habr, EA

AF Saurty-Seerunghen, M. S.

Daubon, T.

Bellenger, L.

Delaunay, V.

Castro, G.

Guyon, J.

Rezk, A.

Fabrega, S.

Idbaih, A.

Almairac, F.

Burel-Vandenbos, F.

Turchi, L.

Virolle, T.

Peyrin, J. -M.

Antoniewski, C.

Chneiweiss, H.

Junier, M. -P.

El-Habr, E. A.

TI Glioblastoma cell motility depends on enhanced oxidative stress coupled

with mobilization of a sulfurtransferase

SO GLIA

LA English

DT Meeting Abstract

CT 16th European Meeting on Glial Cells in Health and Disease

CY JUL 08-11, 2023

CL Berlin, GERMANY

C1 [Saurty-Seerunghen, M. S.; Daubon, T.; Bellenger, L.; Delaunay, V.; Castro, G.; Guyon, J.; Rezk, A.; Fabrega, S.; Idbaih, A.; Almairac, F.; Burel-Vandenbos, F.; Turchi, L.; Virolle, T.; Peyrin, J. -M.; Antoniewski, C.; Chneiweiss, H.; Junier, M. -P.; El-Habr, E. A.] Neurosci Paris Seine IBPS, Paris, France.

RI Junier, Marie-Pierre/KRR-0050-2024

NR 0

TC 0

Z9 0

U1 0

U2 0

PU WILEY

PI HOBOKEN

PA 111 RIVER ST, HOBOKEN 07030-5774, NJ USA

SN 0894-1491

EI 1098-1136

J9 GLIA

JI Glia

PD JUL

PY 2023

VL 71

SU 1

MA T24-020B

BP E1182

EP E1182

PG 1

WC Neurosciences

WE Science Citation Index Expanded (SCI-EXPANDED); Conference Proceedings Citation Index - Science (CPCI-S)

SC Neurosciences & Neurology

GA MC2V9

UT WOS:001191372500988

DA 2025-04-09

ER

PT J

AU Gilbert, M

Liu, YX

Sunkara, M

Morris, A

Pittman, T

Kyprianou, N

Horbinski, C

AF Gilbert, Misty

Liu, Yinxing

Sunkara, Manjula

Morris, Andrew

Pittman, Thomas

Kyprianou, Natasha

Horbinski, Craig

TI Effects of D-2-Hydroxyglutarate on Proliferation, Apoptosis, Autophagy,

and Oxidative Stress in Gliomas

SO JOURNAL OF NEUROPATHOLOGY AND EXPERIMENTAL NEUROLOGY

LA English

DT Meeting Abstract

CT 89th Annual Meeting of the American-Association-of-Neuropathologists-Inc

CY JUN 20-23, 2013

CL Charleston, SC

SP Amer Assoc Neuropathologists Inc

C1 [Gilbert, Misty; Liu, Yinxing; Sunkara, Manjula; Morris, Andrew; Pittman, Thomas; Kyprianou, Natasha; Horbinski, Craig] Univ Kentucky, Lexington, KY 40506 USA.

C3 University of Kentucky

RI sunkara, Manjula/H-7944-2016

NR 0

TC 0

Z9 0

U1 0

U2 8

PU LIPPINCOTT WILLIAMS & WILKINS

PI PHILADELPHIA

PA 530 WALNUT ST, PHILADELPHIA, PA 19106-3621 USA

SN 0022-3069

J9 J NEUROPATH EXP NEUR

JI J. Neuropathol. Exp. Neurol.

PD JUN

PY 2013

VL 72

IS 6

MA 2

BP 540

EP 540

PG 1

WC Clinical Neurology; Neurosciences; Pathology

WE Science Citation Index Expanded (SCI-EXPANDED); Conference Proceedings Citation Index - Science (CPCI-S)

SC Neurosciences & Neurology; Pathology

GA 151IR

UT WOS:000319454400010

DA 2025-04-09

ER

PT J

AU Guffens, L

Derua, R

Janssens, V

AF Guffens, Liesbeth

Derua, Rita

Janssens, Veerle

TI PME-1 sensitizes glioblastoma cells to oxidative stress-induced cell

death by attenuating PP2A-B55α-mediated inactivation of MAPKAPK2-RIPK1

signaling

SO CELL DEATH DISCOVERY

LA English

DT Article

ID PROTEIN PHOSPHATASE 2A; REGULATORY SUBUNIT; CATALYTIC SUBUNIT;

HYDROGEN-PEROXIDE; TUMOR-GROWTH; PP2A; CANCER; PHOSPHORYLATION;

INHIBITION; KINASE

AB Glioblastoma (GBM) is the most common primary brain tumor in adults. Current standard therapy is surgery followed by radiotherapy, with concurrent and adjuvant temozolomide chemotherapy. GBM is characterized by almost uniformly fatal outcomes, highlighting the unmet clinical need for more efficient, biomarker-guided treatments. Protein phosphatase methylesterase-1 (PME-1), a regulator of the tumor suppressive phosphatase PP2A, promotes PP2A demethylation and inactivation, and is overexpressed in 44% of GBM, associated with increased tumor grade and cellular proliferation. Here, we aimed to investigate how reactive oxygen species (ROS), a frequent by-product of radiotherapy and temozolomide chemotherapy, regulate PP2A function via its methylesterase PME-1, and how PME-1 overexpression impacts the response of GBM cells to oxidative stress. We found that in two glioblastoma cell lines, U87MG and U251MG, expression of PME-1 is positively correlated with the sensitivity of the cells to H2O2 or t-BHP-induced oxidative stress. Experiments using the irreversible pharmacologic PME-1 inhibitor, AMZ30, and different PME-1 mutants, revealed that the methylesterase function, the PP2A binding capacity, and the nuclear localization of PME-1 are all important for the sensitizing effect of PME-1 expression. Furthermore, we identified increased nuclear localization of the PP2A-B55a subunit, increased binding of PP2A-B55a to PME-1, and increased B55a-bound PP2A-C demethylation upon oxidative stress. Lastly, we uncovered increased stress-induced phosphorylation and activity of MAPKAPK2 and RIPK1 in PME-1 overexpressing U87MG cells, which caused the observed sensitization to t-BHP treatment. Our data reveal a novel role for PME-1 in oxidative stress-induced GBM cell death, regulating nuclear PP2A-B55a activity and MAPKAPK2-RIPK1 signaling. Patients with GBM tumors overexpressing PME-1, although having a worse prognosis due to increased cellular proliferation of the tumor, could actually be more responsive to oxidative stress-inducing therapies.

C1 [Guffens, Liesbeth; Derua, Rita; Janssens, Veerle] Katholieke Univ Leuven, Dept Cellular & Mol Med, Lab Prot Phosphorylat & Proteom, B-3000 Leuven, Belgium.

[Guffens, Liesbeth; Janssens, Veerle] Katholieke Univ Leuven, Canc Inst LKI, B-3000 Leuven, Belgium.

[Derua, Rita] Katholieke Univ Leuven, SyBioMa, B-3000 Leuven, Belgium.

C3 KU Leuven; KU Leuven; KU Leuven

RP Janssens, V (corresponding author), Katholieke Univ Leuven, Dept Cellular & Mol Med, Lab Prot Phosphorylat & Proteom, B-3000 Leuven, Belgium.; Janssens, V (corresponding author), Katholieke Univ Leuven, Canc Inst LKI, B-3000 Leuven, Belgium.

EM veerle.janssens@kuleuven.be

RI Janssens, Veerle/I-7059-2016

OI Guffens, Liesbeth/0000-0001-8820-8022

FU Research Foundation-Flanders (FWO-Vlaanderen) [G0B1719N]

FX This work was funded by a senior project grant from the Research

Foundation-Flanders (FWO-Vlaanderen, G0B1719N).

CR Amin P, 2022, ONCOGENE, V41, P1, DOI 10.1038/s41388-021-02068-x

Bachovchin DA, 2011, J MED CHEM, V54, P5229, DOI 10.1021/jm200502u

Chandana SR, 2008, AM FAM PHYSICIAN, V77, P1423

Chen L, 2009, INT J BIOCHEM CELL B, V41, P1284, DOI 10.1016/j.biocel.2008.10.029

Cucinotta L, 2022, INT J MOL SCI, V23, DOI 10.3390/ijms232415717

Cundell MJ, 2016, J CELL BIOL, V214, P539, DOI 10.1083/jcb.201606033

De Baere I, 1999, BIOCHEMISTRY-US, V38, P16539, DOI 10.1021/bi991646a

Degterev A, 2008, NAT CHEM BIOL, V4, P313, DOI 10.1038/nchembio.83

Dondelinger Y, 2017, NAT CELL BIOL, V19, P1237, DOI 10.1038/ncb3608

Du BY, 2020, CANCER MANAG RES, V12, P2937, DOI 10.2147/CMAR.S252873

Dun MD, 2020, LEUKEMIA, V34, P3393, DOI 10.1038/s41375-020-0814-0

Elgenaidi IS, 2019, PHARMACOL THERAPEUT, V198, P68, DOI 10.1016/j.pharmthera.2019.02.011

Foley TD, 2007, NEUROCHEM RES, V32, P1957, DOI 10.1007/s11064-007-9394-x

Gordon IK, 2015, MOL CANCER THER, V14, P1540, DOI 10.1158/1535-7163.MCT-14-0614

Gu Y, 2015, ONCOTARGET, V6, P42322, DOI 10.18632/oncotarget.5996

Guo CY, 2002, J BIOL CHEM, V277, P4839, DOI 10.1074/jbc.M110092200

Han XZ, 2012, CARCINOGENESIS, V33, P868, DOI 10.1093/carcin/bgs029

Hofstetter CP, 2012, PLOS ONE, V7, DOI 10.1371/journal.pone.0030059

Hombauer H, 2007, PLOS BIOL, V5, P1355, DOI 10.1371/journal.pbio.0050155

Jaco I, 2017, MOL CELL, V66, P698, DOI 10.1016/j.molcel.2017.05.003

Janssens V, 2001, BIOCHEM J, V353, P417, DOI 10.1042/0264-6021:3530417

Janssens V, 2012, CURR MOL MED, V12, P268, DOI 10.2174/156652412799218930

Janssens V, 2008, TRENDS BIOCHEM SCI, V33, P113, DOI 10.1016/j.tibs.2007.12.004

Jiang T, 2016, NEUROREPORT, V27, P960, DOI 10.1097/WNR.0000000000000638

Kashani E, 2022, CANCERS, V14, DOI 10.3390/cancers14215227

Kauko O, 2018, INT J BIOCHEM CELL B, V96, P157, DOI 10.1016/j.biocel.2018.01.005

Kaur A, 2016, BIOCHEM SOC T, V44, P1683, DOI 10.1042/BST20160161

Kaur A, 2016, CANCER RES, V76, P7001, DOI 10.1158/0008-5472.CAN-16-1134

Khanna A, 2020, MOL CANCER RES, V18, P709, DOI 10.1158/1541-7786.MCR-19-0934

Kong M, 2009, MOL CELL, V36, P51, DOI 10.1016/j.molcel.2009.09.025

Kruse T, 2020, EMBO J, V39, DOI 10.15252/embj.2019103695

Laurent A, 2005, CANCER RES, V65, P948

Lee CW, 2014, J BIOL CHEM, V289, P21108, DOI 10.1074/jbc.M113.540229

Lee HY, 2018, SCI SIGNAL, V11, DOI 10.1126/scisignal.aam7893

Li J, 2014, CANCER BIOL THER, V15, P128, DOI 10.4161/cbt.27146

Li YT, 2022, ELIFE, V11, DOI 10.7554/eLife.79736

Liu ZW, 2017, OXID MED CELL LONGEV, V2017, DOI 10.1155/2017/2525967

Longin S, 2004, BIOCHEM J, V380, P111, DOI 10.1042/BJ20031643

Longin S, 2008, EXP CELL RES, V314, P68, DOI 10.1016/j.yexcr.2007.07.030

Longin S, 2007, J BIOL CHEM, V282, P26971, DOI 10.1074/jbc.M704059200

Low ICC, 2014, BLOOD, V124, P2223, DOI 10.1182/blood-2014-03-563296

Lu J, 2009, P NATL ACAD SCI USA, V106, P11697, DOI 10.1073/pnas.0905930106

Lyons SP, 2021, SCI REP-UK, V11, DOI 10.1038/s41598-021-02456-z

Mäkelä E, 2021, CLIN CANCER RES, V27, P2848, DOI 10.1158/1078-0432.CCR-20-3679

Martínez-Limón A, 2020, INT J MOL SCI, V21, DOI 10.3390/ijms21061913

Meeusen B, 2018, INT J BIOCHEM CELL B, V96, P98, DOI 10.1016/j.biocel.2017.10.002

Menon MB, 2017, NAT CELL BIOL, V19, P1248, DOI 10.1038/ncb3614

Merisaari J, 2020, BRAIN COMMUN, V2, DOI 10.1093/braincomms/fcaa002

Narla G, 2018, CELL MOL LIFE SCI, V75, P2695, DOI 10.1007/s00018-018-2826-8

Ogris E, 1999, J BIOL CHEM, V274, P14382, DOI 10.1074/jbc.274.20.14382

Ohama T, 2010, J BIOL CHEM, V285, P8711, DOI 10.1074/jbc.M109.099788

Olivier C, 2021, FRONT MOL BIOSCI, V7, DOI 10.3389/fmolb.2020.620677

Park HJ, 2018, J NEUROPATH EXP NEUR, V77, P139, DOI 10.1093/jnen/nlx110

Perrotti D, 2013, LANCET ONCOL, V14, pE229, DOI 10.1016/S1470-2045(12)70558-2

Pokharel YR, 2015, MOL CELL PROTEOMICS, V14, P3274, DOI 10.1074/mcp.M115.050773

Puustinen P, 2009, CANCER RES, V69, P2870, DOI 10.1158/0008-5472.CAN-08-2760

Qin SS, 2018, MOL CARCINOGEN, V57, P687, DOI 10.1002/mc.22789

Raman D, 2019, REDOX BIOL, V27, DOI 10.1016/j.redox.2019.101105

Rao RK, 2002, BIOCHEM BIOPH RES CO, V293, P610, DOI 10.1016/S0006-291X(02)00268-1

Rinaldi M, 2016, INT J MOL SCI, V17, DOI 10.3390/ijms17060984

Ronkina N, 2022, ANNU REV BIOCHEM, V91, P505, DOI 10.1146/annurev-biochem-081720-114505

Ruvolo PP, 2016, BBA CLIN, V6, P87, DOI 10.1016/j.bbacli.2016.08.002

Sablina AA, 2010, CANCER RES, V70, P10474, DOI 10.1158/0008-5472.CAN-10-2855

Sangodkar J, 2016, FEBS J, V283, P1004, DOI 10.1111/febs.13573

Sents W, 2013, FEBS J, V280, P644, DOI 10.1111/j.1742-4658.2012.08579.x

Stupp R, 2009, LANCET ONCOL, V10, P459, DOI 10.1016/S1470-2045(09)70025-7

Sun L, 2007, J BIOL CHEM, V282, P3766, DOI 10.1074/jbc.M607347200

Tan PL, 2015, INT J BIOCHEM CELL B, V62, P72, DOI 10.1016/j.biocel.2015.02.015

Tang S, 2018, LIFE SCI, V213, P166, DOI 10.1016/j.lfs.2018.10.029

Wandzioch E, 2014, CANCER RES, V74, P4295, DOI 10.1158/0008-5472.CAN-13-3130

Wang BL, 2022, INT J MED SCI, V19, P1965, DOI 10.7150/ijms.69992

Wepf A, 2009, NAT METHODS, V6, P203, DOI [10.1038/nmeth.1302, 10.1038/NMETH.1302]

Wlodarchak N, 2013, CELL RES, V23, P931, DOI 10.1038/cr.2013.77

Wondrak GT, 2009, ANTIOXID REDOX SIGN, V11, P3013, DOI [10.1089/ars.2009.2541, 10.1089/ARS.2009.2541]

Xing YN, 2008, CELL, V133, P154, DOI 10.1016/j.cell.2008.02.041

Xu YH, 2008, MOL CELL, V31, P873, DOI 10.1016/j.molcel.2008.08.006

Xu YH, 2006, CELL, V127, P1239, DOI 10.1016/j.cell.2006.11.033

Yabe R, 2018, FEBS OPEN BIO, V8, P1486, DOI 10.1002/2211-5463.12485

Yabe R, 2015, PLOS ONE, V10, DOI 10.1371/journal.pone.0145226

Yu XX, 2001, MOL BIOL CELL, V12, P185, DOI 10.1091/mbc.12.1.185

NR 80

TC 2

Z9 2

U1 2

U2 4

PU SPRINGERNATURE

PI LONDON

PA CAMPUS, 4 CRINAN ST, LONDON, N1 9XW, ENGLAND

EI 2058-7716

J9 CELL DEATH DISCOV

JI Cell Death Discov.

PD JUL 27

PY 2023

VL 9

IS 1

AR 265

DI 10.1038/s41420-023-01572-1

PG 12

WC Cell Biology

WE Science Citation Index Expanded (SCI-EXPANDED)

SC Cell Biology

GA N7KF4

UT WOS:001038749000003

PM 37500619

OA gold, Green Published

DA 2025-04-09

ER

PT J

AU Tomkova, S

Misuth, M

Lenkayska, L

Miskovsky, P

Huntosova, V

AF Tomkova, Silvia

Misuth, Matus

Lenkayska, Lenka

Miskovsky, Pavol

Huntosova, Veronika

TI In vitro identification of mitochondrial oxidative stress

production by time-resolved fluorescence imaging of glioma cells

SO BIOCHIMICA ET BIOPHYSICA ACTA-MOLECULAR CELL RESEARCH

LA English

DT Article

DE Mitochondria; Go 6976; Rottlerin; Time-resolved microscopy; Oxidative

stress; Glutathione; Superoxide

ID PROTEIN-KINASE-C; PKC-DELTA; TYROSINE PHOSPHORYLATION;

HYDROGEN-PEROXIDE; GLUTATHIONE DEPLETION; SUPEROXIDE-PRODUCTION;

APOPTOSIS; ACTIVATION; INHIBITOR; CYSTEINE

AB Oxidative phosphorylation and glycolysis are important features, by which cells could bypass oxidative stress. The level of oxidative stress, and the ability of cells to promote oxidative phosphorylation or glycolysis, significantly determined proliferation or cell demise. In the present work, we have employed selective mitochondrial probe MitoTracker (TM) Orange CMTM/Ros (MTO) to estimate the level of oxidative stress in cancer cells at different stressed conditions. MTO is partially sensitive to decrease of mitochondrial membrane potential and to reactive oxygen species (ROS) generated in mitochondria. We have demonstrated, that fluorescence lifetime of MTO is much more sensitive to oxidative stress than intensity-based approaches. This method was validated in different cancer cell lines. Our approach revealed, at relatively low ROS levels, that Go 6976, a protein kinase C (PKC) alpha inhibitor, and rottlerin, an indirect PKC delta inhibitor, increased mitochondrial ROS level in glioma cell. Their involvement in oxidative phosphorylation and apoptosis was investigated with oxygen consumption rate estimation, western blot and flow-cytometric analysis. Our study brings new insight to identify feeble differences in ROS production in living cells.

C1 [Tomkova, Silvia; Misuth, Matus; Lenkayska, Lenka] PJ Safarik Univ Kosice, Fac Sci, Dept Biophys, Jesenna 5, Kosice 04154, Slovakia.

[Miskovsky, Pavol; Huntosova, Veronika] PJ Safarik Univ Kosice, Ctr Interdisciplinary Biosci Technol & Innovat Pk, Jesenna 5, Kosice 04154, Slovakia.

[Miskovsky, Pavol] SAFTRA Photon Ltd, Jesenna 5, Kosice 04154, Slovakia.

C3 University of Pavol Jozef Safarik Kosice; University of Pavol Jozef

Safarik Kosice

RP Huntosova, V (corresponding author), PJ Safarik Univ Kosice, Ctr Interdisciplinary Biosci Technol & Innovat Pk, Jesenna 5, Kosice 04154, Slovakia.

EM veronika.huntosova@upjs.sk

RI Huntosova, Veronika/AAX-4977-2021

OI Misuth, Matus/0000-0002-5867-3094; Huntosova,

Veronika/0000-0003-0042-2097

FU Slovak Research and Development Agency [VEGA 1-0425-15, APVV-15-0485]

FX This work was funded by the Slovak Research and Development Agency VEGA

1-0425-15 and APVV-15-0485. The authors strongly appreciate this

support.

CR Acin-Perez R, 2010, FASEB J, V24, P5033, DOI 10.1096/fj.10-166934

Baracca A, 2003, BBA-BIOENERGETICS, V1606, P137, DOI 10.1016/S0005-2728(03)00110-5

Becker W, 2012, J MICROSC-OXFORD, V247, P119, DOI 10.1111/j.1365-2818.2012.03618.x

Bienert GP, 2006, BBA-BIOMEMBRANES, V1758, P994, DOI 10.1016/j.bbamem.2006.02.015

Bienert GP, 2014, BBA-GEN SUBJECTS, V1840, P1596, DOI 10.1016/j.bbagen.2013.09.017

Biswas DK, 2000, P NATL ACAD SCI USA, V97, P8542, DOI 10.1073/pnas.97.15.8542

Campello S, 2014, BBA-BIOENERGETICS, V1837, P451, DOI 10.1016/j.bbabio.2013.11.010

Cieslak D, 2007, MOL CELLS, V24, P224

Clerc P, 2012, PLOS ONE, V7, DOI 10.1371/journal.pone.0034465

Crump KE, 2012, EUR J IMMUNOL, V42, P2152, DOI 10.1002/eji.201142289

DeVries-Seimon TA, 2007, J BIOL CHEM, V282, P22307, DOI 10.1074/jbc.M703661200

Domenicotti C, 2003, FREE RADICAL BIO MED, V35, P504, DOI 10.1016/S0891-5849(03)00332-0

Filomeni G, 2010, AUTOPHAGY, V6, P999, DOI 10.4161/auto.6.7.12754

Giles NM, 2003, BIOCHEM BIOPH RES CO, V300, P1, DOI 10.1016/S0006-291X(02)02770-5

Go YM, 2010, ANTIOXID REDOX SIGN, V13, P489, DOI 10.1089/ars.2009.3021

Gomel R, 2007, MOL CANCER RES, V5, P627, DOI 10.1158/1541-7786.MCR-06-0255

Gordon R, 2016, NEUROBIOL DIS, V93, P96, DOI 10.1016/j.nbd.2016.04.008

Grandage VL, 2006, BRIT J HAEMATOL, V135, P303, DOI 10.1111/j.1365-2141.2006.06291.x

Han D, 2003, MOL PHARMACOL, V64, P1136, DOI 10.1124/mol.64.5.1136

Hansford RG, 1997, J BIOENERG BIOMEMBR, V29, P89, DOI 10.1023/A:1022420007908

He Y, 2007, J CELL BIOCHEM, V101, P1210, DOI 10.1002/jcb.21243

Hill BG, 2009, BIOCHEM J, V424, P99, DOI 10.1042/BJ20090934

Huntosova V, 2017, TOXICOL IN VITRO, V40, P184, DOI 10.1016/j.tiv.2017.01.005

Huntosova V, 2014, METALLOMICS, V6, P2279, DOI 10.1039/c4mt00190g

Huntosova V, 2012, PHOTOCH PHOTOBIO SCI, V11, P1428, DOI 10.1039/c2pp05409d

Kato K, 2009, AM J PHYSIOL-HEART C, V297, pH2253, DOI 10.1152/ajpheart.00274.2009

Kessel D, 2014, PHOTOCHEM PHOTOBIOL, V90, P1211, DOI 10.1111/php.12283

Kim YA, 2013, BIOMOL THER, V21, P358, DOI 10.4062/biomolther.2013.065

Klepinin A, 2014, J BIOENERG BIOMEMBR, V46, P17, DOI 10.1007/s10863-013-9529-5

Koivunen J, 2004, CANCER RES, V64, P5693, DOI 10.1158/0008-5472.CAN-03-3511

Kondaveeti Y, 2015, CANCER LETT, V364, P44, DOI 10.1016/j.canlet.2015.04.025

Konishi H, 1997, P NATL ACAD SCI USA, V94, P11233, DOI 10.1073/pnas.94.21.11233

Kowalczyk JE, 2009, NEUROCHEM INT, V55, P157, DOI 10.1016/j.neuint.2009.01.009

Krämer AC, 2016, FREE RADICAL BIO MED, V97, P544, DOI 10.1016/j.freeradbiomed.2016.07.010

Kumar D, 2013, MOL CANCER, V12, DOI 10.1186/1476-4598-12-171

Kweon SM, 2001, BIOSCIENCE REP, V21, P341, DOI 10.1023/A:1013290316939

Lambert AJ, 2004, BIOCHEM J, V382, P511, DOI 10.1042/BJ20040485

Lasfer M, 2006, FEBS LETT, V580, P2547, DOI 10.1016/j.febslet.2006.03.089

Li NY, 2003, J BIOL CHEM, V278, P8516, DOI 10.1074/jbc.M210432200

Li YB, 1998, BIOCHEM BIOPH RES CO, V253, P295, DOI 10.1006/bbrc.1998.9729

Liu YB, 2002, J NEUROCHEM, V80, P780, DOI 10.1046/j.0022-3042.2002.00744.x

Lubos E, 2011, ANTIOXID REDOX SIGN, V15, P1957, DOI 10.1089/ars.2010.3586

Macho A, 1996, CYTOMETRY, V25, P333, DOI 10.1002/(SICI)1097-0320(19961201)25:4<333::AID-CYTO4>3.0.CO;2-E

Maioli E, 2012, SCI WORLD J, DOI 10.1100/2012/350826

Majumder PK, 2001, CELL GROWTH DIFFER, V12, P465

Marques-Santos LF, 2003, BIOSCIENCE REP, V23, P199, DOI 10.1023/B:BIRE.0000007693.33521.18

MARTINYBARON G, 1993, J BIOL CHEM, V268, P9194

Melo EP, 2017, BMC BIOL, V15, DOI 10.1186/s12915-017-0367-5

Misuth M, 2017, PHOTODIAGN PHOTODYN, V18, P267, DOI 10.1016/j.pdpdt.2017.03.018

Misuth M, 2017, J BIOPHOTONICS, V10, P423, DOI 10.1002/jbio.201500332

Misuth M, 2017, CELL SIGNAL, V34, P11, DOI 10.1016/j.cellsig.2017.02.020

Morad SAF, 2013, NAT REV CANCER, V13, P51, DOI 10.1038/nrc3398

Muller FL, 2004, J BIOL CHEM, V279, P49064, DOI 10.1074/jbc.M407715200

Nakamura K, 2001, J BIOL CHEM, V276, P34402, DOI 10.1074/jbc.M103766200

Nietzel T, 2017, MITOCHONDRION, V33, P72, DOI 10.1016/j.mito.2016.07.010

Oda T, 1999, J BIOCHEM-TOKYO, V126, P715, DOI 10.1093/oxfordjournals.jbchem.a022508

Okhrimenko H, 2005, J BIOL CHEM, V280, P23643, DOI 10.1074/jbc.M501374200

Parent N, 2011, INT J ONCOL, V38, P313, DOI 10.3892/ijo.2010.881

Paulsen CE, 2010, ACS CHEM BIOL, V5, P47, DOI 10.1021/cb900258z

Salvioli S, 1997, FEBS LETT, V411, P77, DOI 10.1016/S0014-5793(97)00669-8

Schuchmann S, 2000, FREE RADICAL BIO MED, V28, P235, DOI 10.1016/S0891-5849(99)00226-9

Scorrano L., 1999, J BIOL CHEM, V274

Sedlák E, 2010, FREE RADICAL BIO MED, V49, P1574, DOI 10.1016/j.freeradbiomed.2010.08.019

Sepulveda MF, 2005, INFLAMM RES, V54, P97, DOI 10.1007/s00011-004-1329-2

Shah BH, 2005, MOL PHARMACOL, V67, P184, DOI 10.1124/mol.104.003533

Sies H, 1999, FREE RADICAL BIO MED, V27, P916, DOI 10.1016/S0891-5849(99)00177-X

Sies H, 2014, J BIOL CHEM, V289, P8735, DOI 10.1074/jbc.R113.544635

Singh BN, 2012, BIOCHEM PHARMACOL, V84, P1154, DOI 10.1016/j.bcp.2012.08.007

Soltoff SP, 2001, J BIOL CHEM, V276, P37986

Tada-Oikawa S, 2003, LIFE SCI, V73, P3277, DOI 10.1016/j.lfs.2003.06.013

Tapia JA, 2006, BBA-MOL CELL RES, V1763, P25, DOI 10.1016/j.bbamcr.2005.10.007

Vlashi E, 2011, P NATL ACAD SCI USA, V108, P16062, DOI 10.1073/pnas.1106704108

WHITAKER JE, 1991, BIOCHEM BIOPH RES CO, V175, P387, DOI 10.1016/0006-291X(91)91576-X

Wu DF, 2001, ALCOHOL CLIN EXP RES, V25, P619, DOI 10.1097/00000374-200104000-00021

Wu M, 2007, AM J PHYSIOL-CELL PH, V292, pC125, DOI 10.1152/ajpcell.00247.2006

Yang J, 2009, AM J PHYSIOL-RENAL, V297, pF1220, DOI 10.1152/ajprenal.00314.2009

Yumoto R, 1999, J PHARMACOL EXP THER, V289, P149

Zheng J, 2012, ONCOL LETT, V4, P1151, DOI 10.3892/ol.2012.928

NR 78

TC 20

Z9 20

U1 2

U2 19

PU ELSEVIER

PI AMSTERDAM

PA RADARWEG 29, 1043 NX AMSTERDAM, NETHERLANDS

SN 0167-4889

EI 1879-2596

J9 BBA-MOL CELL RES

JI Biochim. Biophys. Acta-Mol. Cell Res.

PD APR

PY 2018

VL 1865

IS 4

BP 616

EP 628

DI 10.1016/j.bbamcr.2018.01.012

PG 13

WC Biochemistry & Molecular Biology; Cell Biology

WE Science Citation Index Expanded (SCI-EXPANDED)

SC Biochemistry & Molecular Biology; Cell Biology

GA FZ1KO

UT WOS:000427335700008

PM 29410069

DA 2025-04-09

ER

PT J

AU Yang, CY

Wang, HY

Xia, ZY

Li, T

Chen, YM

Liu, GQ

Sun, Y

Ma, J

Wu, Y

Wang, XY

Wang, P

Wang, GL

AF Yang, C. Y.

Wang, H. Y.

Xia, Z. Y.

Li, T.

Chen, Y. M.

Liu, G. Q.

Sun, Y.

Ma, J.

Wu, Y.

Wang, X. Y.

Wang, P.

Wang, G. L.

TI Propofol suppresses oxidative stress in gliomas through down-regulating

divalent metal transporter 1

SO BRITISH JOURNAL OF ANAESTHESIA

LA English

DT Meeting Abstract

CT 26th Annual Meeting of the Chinese-Society-of-Anesthesiology

CY NOV 01-05, 2018

CL Beijing, PEOPLES R CHINA

SP Chinese Soc Anesthesiol

C1 [Yang, C. Y.; Wang, H. Y.; Li, T.; Chen, Y. M.; Liu, G. Q.; Sun, Y.; Ma, J.; Wu, Y.; Wang, X. Y.] Tianjin Med Univ, Cent Clin Coll 3, Cent Affiliated Hosp 3, Dept Anaesthesiol,Nankai Univ, Tianjin, Peoples R China.

[Wang, H. Y.; Wang, G. L.] Tianjin Med Univ, Gen Hosp, Tianjin Res Inst Anaesthesiol, Tianjin, Peoples R China.

[Xia, Z. Y.] Univ Hong Kong, Dept Anaesthesiol, Pokfulam, Hong Kong, Peoples R China.

[Wang, H. Y.; Li, T.; Chen, Y. M.; Liu, G. Q.; Sun, Y.; Ma, J.; Wu, Y.; Wang, X. Y.; Wang, P.] Third Cent Hosp Tianjin, Tianjin Key Lab Artificial Cell, Tianjin Inst Hepatobiliary Dis, Artificial Cell Engn Res Ctr,Minist Hlth, Tianjin, Peoples R China.

C3 Nankai University; Tianjin Medical University; Tianjin Medical

University; University of Hong Kong

RI Irwin, Michael/C-4286-2009

FU National Natural Science Foundation of China [81071059, 81100984,

81571054]; Tianjin Research Program of Application Foundation and

Advanced Technology [15JCYBJC25600]; Tianjin Municipal Planning

Commission Science and Technology project [15KG117]

FX National Natural Science Foundation of China (No. 81071059, 81100984,

and 81571054), Tianjin Research Program of Application Foundation and

Advanced Technology (No. 15JCYBJC25600), and the Tianjin Municipal

Planning Commission Science and Technology project (No. 15KG117).

CR Chen XY, 2017, EUR J PHARMACOL, V795, P150, DOI 10.1016/j.ejphar.2016.12.017

Romuk EB, 2016, ADV CLIN EXP MED, V25, P815, DOI 10.17219/acem/36459

White RS, 2016, MOL BRAIN, V9, DOI 10.1186/s13041-016-0220-8

NR 3

TC 1

Z9 1

U1 0

U2 3

PU ELSEVIER SCI LTD

PI OXFORD

PA THE BOULEVARD, LANGFORD LANE, KIDLINGTON, OXFORD OX5 1GB, OXON, ENGLAND

SN 0007-0912

EI 1471-6771

J9 BRIT J ANAESTH

JI Br. J. Anaesth.

PD MAR

PY 2019

VL 122

IS 3

BP E40

EP E41

DI 10.1016/j.bja.2018.10.018

PG 2

WC Anesthesiology

WE Science Citation Index Expanded (SCI-EXPANDED); Conference Proceedings Citation Index - Science (CPCI-S)

SC Anesthesiology

GA HL2DN

UT WOS:000458513600036

OA Bronze

DA 2025-04-09

ER

PT J

AU Hegge, B

Sjottem, E

Mikkola, I

AF Hegge, Beate

Sjottem, Eva

Mikkola, Ingvild

TI Generation of a PAX6 knockout glioblastoma cell line with changes in

cell cycle distribution and sensitivity to oxidative stress

SO BMC CANCER

LA English

DT Article

DE PAX6; U251; Glioblastoma; Cell cycle; Oxidative stress; CRISPR-Cas9;

Migration; Proliferation; Morphology; Colony-formation

ID ENDOTHELIAL GROWTH-FACTOR; TRANSCRIPTION FACTOR; HYDROGEN-PEROXIDE;

HUMAN GLIOMAS; LUNG-CANCER; STEM-CELLS; EXPRESSION; PROLIFERATION; GENE;

PROMOTES

AB Background: The transcription factor PAX6 is expressed in various cancers. In anaplastic astrocytic glioma, PAX6 expression is inversely related to tumor grade, resulting in low PAX6 expression in Glioblastoma, the highest-grade astrocytic glioma. The aim of the present study was to develop a PAX6 knock out cell line as a tool for molecular studies of the roles PAX6 have in attenuating glioblastoma tumor progression.

Methods: The CRISPR-Cas9 technique was used to knock out PAX6 in U251 N cells. Viral transduction of a doxycycline inducible EGFP-PAX6 expression vector was used to re-introduce (rescue) PAX6 expression in the PAX6 knock out cells. The knock out and rescued cells were rigorously characterized by analyzing morphology, proliferation, colony forming abilities and responses to oxidative stress and chemotherapeutic agents.

Results: The knock out cells had increased proliferation and colony forming abilities compared to wild type cells, consistent with clinical observations indicating that PAX6 functions as a tumor-suppressor. Cell cycle distribution and sensitivity to H2O2 induced oxidative stress were further studied, as well as the effect of different chemotherapeutic agents. For the PAX6 knock out cells, the percentage of cells in G2/M phase increased compared to PAX6 control cells, indicating that PAX6 keeps U251 N cells in the G1 phase of the cell cycle. Interestingly, PAX6 knock out cells were more resilient to H2O2 induced oxidative stress than wild type cells. Chemotherapy treatment is known to generate oxidative stress, hence the effect of several chemotherapeutic agents were tested. We discovered interesting differences in the sensitivity to chemotherapeutic drugs (Temozolomide, Withaferin A and Sulforaphane) between the PAX6 expressing and non-expressing cells.

Conclusions: The U251 N PAX6 knock out cell lines generated can be used as a tool to study the molecular functions and mechanisms of PAX6 as a tumor suppressor with regard to tumor progression and treatment of glioblastoma.

C1 [Hegge, Beate; Mikkola, Ingvild] Univ Tromso, Dept Pharm, Res Grp Pharmacol, N-9037 Tromso, Norway.

[Sjottem, Eva] Univ Tromso, Dept Med Biol, Mol Canc Res Grp, N-9037 Tromso, Norway.

C3 UiT The Arctic University of Tromso; UiT The Arctic University of Tromso

RP Mikkola, I (corresponding author), Univ Tromso, Dept Pharm, Res Grp Pharmacol, N-9037 Tromso, Norway.

EM ingvild.mikkola@uit.no

OI Sjottem, Eva/0000-0003-2668-1708; Mikkola, Ingvild/0000-0001-6442-7626

FU University of Tromso - The Arctic University of Norway; Northern Norway

Regional Health Authority; UiT - The Arctic University of Norway

FX The project was supported by a PhD grant for BH from the University of

Tromso - The Arctic University of Norway, and by funding from the

Northern Norway Regional Health Authority. The publication charges for

this article have been funded by a grant from the publication fund of

UiT - The Arctic University of Norway. None of the funding sources have

played any role in the design of the study, nor in collection, analysis,

interpretation of data or writing of the manuscript.

CR Aoki T, 2007, EXPERT OPIN PHARMACO, V8, P3133, DOI 10.1517/14656566.8.18.3133

Ashery-Padan R, 2004, DEV BIOL, V269, P479, DOI 10.1016/j.ydbio.2004.01.040

Bai RY, 2011, TRENDS MOL MED, V17, P301, DOI 10.1016/j.molmed.2011.01.011

Benzing K, 2011, PLOS GENET, V7, DOI 10.1371/journal.pgen.1002099

Bonnefont J, 2011, CELL DEATH DIFFER, V18, P293, DOI 10.1038/cdd.2010.102

Cao XM, 2013, ONCOL REP, V29, P1013, DOI 10.3892/or.2012.2206

Cartier L, 2006, J NEUROBIOL, V66, P421, DOI 10.1002/neu.20225

Chang JY, 2007, J NEURO-ONCOL, V84, P9, DOI 10.1007/s11060-007-9347-x

Cheng Q, 2017, MOL MED REP, V15, P597, DOI 10.3892/mmr.2016.6078

Cheng Q, 2014, MOL MED REP, V10, P399, DOI 10.3892/mmr.2014.2150

Di Lullo E, 2011, DEVELOPMENT, V138, P4991, DOI 10.1242/dev.066282

Dorà N, 2008, DEV DYNAM, V237, P1295, DOI 10.1002/dvdy.21528

Duparc RH, 2007, DEV BIOL, V301, P374, DOI 10.1016/j.ydbio.2006.11.006

Farhy C, 2013, PLOS ONE, V8, DOI 10.1371/journal.pone.0076489

Furnari FB, 2007, GENE DEV, V21, P2683, DOI 10.1101/gad.1596707

Grogan PT, 2014, INVEST NEW DRUG, V32, P604, DOI 10.1007/s10637-014-0084-7

Grogan PT, 2013, INVEST NEW DRUG, V31, P545, DOI 10.1007/s10637-012-9888-5

Hsieh YW, 2009, NEURAL DEV, V4, DOI 10.1186/1749-8104-4-32

Hu BL, 2016, CELL, V167, P1281, DOI 10.1016/j.cell.2016.10.039

Huang BS, 2013, ONCOL REP, V30, P2263, DOI 10.3892/or.2013.2683

Huang TY, 2012, CELL BIOCHEM BIOPHYS, V63, P247, DOI 10.1007/s12013-012-9360-3

Khasraw M, 2010, CURR ONCOL REP, V12, P26, DOI 10.1007/s11912-009-0077-4

Kiselev Y, 2012, PLOS ONE, V7, DOI 10.1371/journal.pone.0031915

Kubota H, 2001, GENE CHROMOSOME CANC, V31, P125, DOI 10.1002/gcc.1126

Lan FM, 2016, INT J ONCOL, V48, P559, DOI 10.3892/ijo.2015.3271

Livak KJ, 2001, METHODS, V25, P402, DOI 10.1006/meth.2001.1262

Ma Q, 2013, ANNU REV PHARMACOL, V53, P401, DOI 10.1146/annurev-pharmtox-011112-140320

Manuel MN, 2015, FRONT CELL NEUROSCI, V9, DOI 10.3389/fncel.2015.00070

Martin S, 2009, BBA-MOL CELL RES, V1793, P354, DOI 10.1016/j.bbamcr.2008.09.019

Mascarenhas JB, 2009, J BIOL CHEM, V284, P27524, DOI 10.1074/jbc.M109.047209

Mayes DA, 2006, CANCER RES, V66, P9809, DOI 10.1158/0008-5472.CAN-05-3877

Meng B, 2014, INT J MOL MED, V34, P399, DOI 10.3892/ijmm.2014.1812

Meyer-Ficca ML, 2004, ANAL BIOCHEM, V334, P9, DOI 10.1016/j.ab.2004.07.011

Mischel PS, 2003, BRAIN PATHOL, V13, P52

Mizuguchi H, 2001, BBA-GEN SUBJECTS, V1568, P21, DOI 10.1016/S0304-4165(01)00195-7

Muratovska A, 2003, ONCOGENE, V22, P7989, DOI 10.1038/sj.onc.1206766

Nobusawa S, 2010, BRAIN PATHOL, V20, P936, DOI 10.1111/j.1750-3639.2010.00395.x

Nojima Hiroshi, 2004, Methods Mol Biol, V280, P3

Osumi N, 2008, STEM CELLS, V26, P1663, DOI 10.1634/stemcells.2007-0884

Ouyang J, 2006, INVEST OPHTH VIS SCI, V47, P2397, DOI 10.1167/iovs.05-1083

Pavlakis E, 2017, ONCOL REP, V37, P1579, DOI 10.3892/or.2017.5411

Pinto GR, 2007, GENET MOL RES, V6, P1019

Ran FA, 2013, NAT PROTOC, V8, P2281, DOI 10.1038/nprot.2013.143

Sakurai K, 2008, J NEUROSCI, V28, P4604, DOI 10.1523/JNEUROSCI.5074-07.2008

Sarica Feyzi Birol, 2012, Asian J Neurosurg, V7, P181, DOI 10.4103/1793-5482.106650

Schedl A, 1996, CELL, V86, P71, DOI 10.1016/S0092-8674(00)80078-1

Schumacker PT, 2006, CANCER CELL, V10, P175, DOI 10.1016/j.ccr.2006.08.015

Schwartzbaum JA, 2006, NAT CLIN PRACT NEURO, V2, P494, DOI 10.1038/ncpneuro0289

Sengupta S, 2012, CLIN DEV IMMUNOL, DOI 10.1155/2012/831090

Shih YL, 2016, CLIN EPIGENETICS, V8, DOI 10.1186/s13148-016-0208-3

Shimizu N, 2009, BIOL PHARM BULL, V32, P999, DOI 10.1248/bpb.32.999

Stacey DW, 2003, CURR OPIN CELL BIOL, V15, P158, DOI 10.1016/S0955-0674(03)00008-5

Sun J, 2015, NUCLEIC ACIDS RES, V43, P6827, DOI 10.1093/nar/gkv589

SZATROWSKI TP, 1991, CANCER RES, V51, P794

Thompson SL, 2011, CHROMOSOME RES, V19, P433, DOI 10.1007/s10577-010-9179-y

Torsvik A, 2014, CANCER MED-US, V3, P812, DOI 10.1002/cam4.219

TOYOKUNI S, 1995, FEBS LETT, V358, P1, DOI 10.1016/0014-5793(94)01368-B

Untergasser A, 2007, NUCLEIC ACIDS RES, V35, pW71, DOI 10.1093/nar/gkm306

Wang JY, 2012, J NEURO-ONCOL, V106, P473, DOI 10.1007/s11060-011-0692-4

Yamasaki T, 2001, DEVELOPMENT, V128, P3133

Yang K, 2006, CELL DIV, V1, DOI 10.1186/1747-1028-1-32

Zhang Jihong, 2012, Curr Mol Pharmacol, V5, P102

Zhang X, 2005, J CLIN NEUROSCI, V12, P166, DOI 10.1016/j.jocn.2004.03.036

Zhang XX, 2015, INT J CLIN EXP PATHO, V8, P11452

Zhang Z, 2016, SPRINGERPLUS, V5, DOI 10.1186/s40064-016-1910-5

Zhao X., 2014, Mathematical Problems in Engineering, V2014, P1, DOI DOI 10.1371/J0URNAL.P0NE.0108020

Zhao Y, 2016, TUMOR BIOL, V37, P8691, DOI 10.1007/s13277-015-4645-y

Zhou YH, 2005, J NEURO-ONCOL, V71, P223, DOI 10.1007/s11060-004-1720-4

Zhou YH, 2003, CLIN CANCER RES, V9, P3369

Zhou YH, 2010, J NEURO-ONCOL, V96, P191, DOI 10.1007/s11060-009-9963-8

Zieba M, 2000, RESP MED, V94, P800, DOI 10.1053/rmed.2000.0825

NR 71

TC 23

Z9 26

U1 1

U2 7

PU BIOMED CENTRAL LTD

PI LONDON

PA 236 GRAYS INN RD, FLOOR 6, LONDON WC1X 8HL, ENGLAND

SN 1471-2407

J9 BMC CANCER

JI BMC Cancer

PD MAY 2

PY 2018

VL 18

AR 496

DI 10.1186/s12885-018-4394-6

PG 19

WC Oncology

WE Science Citation Index Expanded (SCI-EXPANDED)

SC Oncology

GA GE5NF

UT WOS:000431268600006

PM 29716531

OA Green Published, gold

DA 2025-04-09

ER

PT J

AU Wang, JL

Chen, S

Xiang, W

Zhu, Q

Ren, NJ

AF Wang, Jiali

Chen, Shuai

Xiang, Wang

Zhu, Qing

Ren, Nianjun

TI NRF1 Alleviated Oxidative Stress of Glioblastoma Cells by Regulating

NOR1

SO FOLIA BIOLOGICA

LA English

DT Article

AB Oxidored-nitro domain-containing protein 1 (NOR1) is a critical tumour suppressor gene, though its regulatory mechanism in oxidative stress of glioblastoma (GBM) remains unclear. Hence, further study is needed to unravel the function of NOR1 in the progression of oxidative stress in GBM. In this study, we evaluated the expression of NOR1 and nuclear respiratory factor 1 (NRF1) in GBM tissue and normal brain tissue (NBT) using quantitative realtime polymerase chain reaction (qRT-PCR) and Western blot (WB), and investigated their relationship. We then induced oxidative stress in U251 cells through H2O2 treatment and conducted Cell Counting Kit-8, Transwell and wound healing assays to analyse cell proliferation, invasion and migration. Cell apoptosis was assessed by flow cytometry and TUNEL staining. We also measured the activities of superoxide dismutase and catalase, as well as the level of reactive oxygen species (ROS) using biochemical techniques. Via qRT-PCR and WB, the mRNA and protein expression levels of NOR1 and NRF1 were determined. Chromatin immunoprecipitation (ChIP) assays were applied to validate NRF1's interaction with NOR1. Our results showed that the expression of NOR1 and NRF1 was low in GBM, and their expression levels were positively correlated. H2O2-induced oxidative stress reduced NRF1 and NOR1 expression levels and increased the ROS level. The ChIP assay confirmed the binding of NRF1 to NOR1. Over expression of NRF1 attenuated the inhibitory effect of oxidative stress on the proliferation, migration and invasion of U251 cells, which was reversed by knockdown of NOR1.

C1 [Wang, Jiali] Cent South Univ, Hunan Canc Hosp, Dept Colon & Rectal Surg, Changsha 410013, Hunan, Peoples R China.

[Wang, Jiali; Chen, Shuai; Xiang, Wang; Zhu, Qing; Ren, Nianjun] Cent South Univ, Affiliated Canc Hosp, Xiangya Sch Med, 283 Tongzipo Rd, Changsha 410013, Hunan, Peoples R China.

[Chen, Shuai; Ren, Nianjun] Cent South Univ, Hunan Canc Hosp, Dept Neurosurg, 283 Tongzipo Rd, Changsha 410013, Hunan, Peoples R China.

[Xiang, Wang] Cent South Univ, Hunan Canc Hosp, Radiol Diag Ctr, Changsha 410013, Hunan, Peoples R China.

[Zhu, Qing] Cent South Univ, Hunan Canc Hosp, Dept Pharm, 283 Tongzipo Rd, Changsha 410013, Hunan, Peoples R China.

C3 Central South University; Central South University; Central South

University; Central South University; Central South University

RP Zhu, Q; Ren, NJ (corresponding author), Cent South Univ, Affiliated Canc Hosp, Xiangya Sch Med, 283 Tongzipo Rd, Changsha 410013, Hunan, Peoples R China.; Ren, NJ (corresponding author), Cent South Univ, Hunan Canc Hosp, Dept Neurosurg, 283 Tongzipo Rd, Changsha 410013, Hunan, Peoples R China.; Zhu, Q (corresponding author), Cent South Univ, Hunan Canc Hosp, Dept Pharm, 283 Tongzipo Rd, Changsha 410013, Hunan, Peoples R China.

EM zhuqing@hnca.org.cn; rennianjun@hnca.org.cn

RI xiang, wang/JKH-6962-2023

FU General Project of Hunan vincial Natural Science Foundation of China

[2019JJ40182]

FX This study was supported by the General Project of Hunan vincial Natural

Science Foundation of China (2019JJ40182) .

CR Asija S, 2022, INT REV IMMUNOL, V41, P582, DOI 10.1080/08830185.2022.2101647

Bugno M, 2015, BBA-GENE REGUL MECH, V1849, P1260, DOI 10.1016/j.bbagrm.2015.08.001

Grochans S, 2022, CANCERS, V14, DOI 10.3390/cancers14102412

Hayes JD, 2020, CANCER CELL, V38, P167, DOI 10.1016/j.ccell.2020.06.001

Herring JA, 2019, CELLS-BASEL, V8, DOI 10.3390/cells8111373

Hu SF, 2022, REDOX BIOL, V57, DOI 10.1016/j.redox.2022.102470

Jelic MD, 2021, J CANCER RES THER, V17, P22, DOI 10.4103/jcrt.JCRT_862_16

Kuo CL, 2022, J BIOMED SCI, V29, DOI 10.1186/s12929-022-00859-2

Li WJ, 2011, CARCINOGENESIS, V32, P1305, DOI 10.1093/carcin/bgr174

McNamara C, 2022, NEURORADIOLOGY, V64, P1919, DOI 10.1007/s00234-022-03008-6

Nanjaiah ND, 2019, CELL BIOL INT, V43, P1443, DOI 10.1002/cbin.11193

Northrop A, 2020, MOL BIOL CELL, V31, P2158, DOI 10.1091/mbc.E20-04-0238

Olivier C, 2021, FRONT MOL BIOSCI, V7, DOI 10.3389/fmolb.2020.620677

Ostrowski RP, 2022, NEUROCHEM INT, V154, DOI 10.1016/j.neuint.2022.105281

Pla-Martin D, 2020, EMBO J, V39, DOI 10.15252/embj.2019102731

Rong L, 2022, J EXP CLIN CANC RES, V41, DOI 10.1186/s13046-022-02349-7

Ruvkun G, 2023, CSH PERSPECT BIOL, V15, DOI 10.1101/cshperspect.a041266

Sun LJ, 2023, TECHNOL CANCER RES T, V22, DOI 10.1177/15330338231161141

Sun Q, 2018, CELL DEATH DIFFER, V25, P1160, DOI 10.1038/s41418-017-0034-y

Xu YY, 2012, ENVIRON HEALTH PERSP, V120, P865, DOI 10.1289/ehp.1204987

Yuan BY, 2022, HUM VACC IMMUNOTHER, V18, DOI 10.1080/21645515.2022.2055417

Yuan JX, 2018, TOXICOL APPL PHARM, V360, P273, DOI 10.1016/j.taap.2018.09.037

Zhang SW, 2020, OXID MED CELL LONGEV, V2020, DOI 10.1155/2020/5097109

Zhang YG, 2016, BIOCHEM J, V473, P961, DOI 10.1042/BJ20151182

NR 24

TC 2

Z9 2

U1 0

U2 0

PU CHARLES UNIV PRAGUE, FIRST FACULTY MEDICINE

PI PRAGUE 6

PA FLEMINGOVO NAM. 2, PRAGUE 6 166 37, CZECH REPUBLIC

SN 0015-5500

J9 FOLIA BIOL-PRAGUE

JI Folia Biol.-Prague

PY 2023

VL 69

IS 1

BP 13

EP 21

DI 10.14712/fb2023069010013

PG 9

WC Biochemistry & Molecular Biology; Biology; Oncology; Cell Biology

WE Science Citation Index Expanded (SCI-EXPANDED)

SC Biochemistry & Molecular Biology; Life Sciences & Biomedicine - Other

Topics; Oncology; Cell Biology

GA CQ5L5

UT WOS:001126721700002

PM 37962027

OA Bronze

DA 2025-04-09

ER

PT J

AU Fu, Q

Liu, DH

Li, ZX

Zheng, XP

Mutemwa, C

Agbo, E

AF Fu, Qiang

Liu, Dong Hai

Li, Zi Xuan

Zheng, Xiao Peng

Mutemwa, Cynthia

Agbo, Elvis

TI Experimental Study on the Anti-tumor and Pro-apoptotic Effects of

Paeonol in Human Gliomas

SO IRANIAN RED CRESCENT MEDICAL JOURNAL

LA English

DT Article

DE Antioxidants; Apoptosis; Caspase-3; Glioma; Oxidative stress

ID OXIDATIVE STRESS; INHIBITION; MIGRATION; INVASION; CANCER; CELLS; RNA

AB Background: Current studies have demonstrated the anti-cancer effects of paeonol in some tumors; however, its effect on gliomas remains unknown.

Objectives: This study aimed to investigate the anti-tumor effect of paeonol in human glioma tissues and cells including its effect and connection with apoptosis and oxidative stress in gliomas.

Methods: Cell Counting Kit-8 (CCK-8) was used to detect the antiproliferative effect of paeonol in human U251 glioma cells. Transwell and colony-forming assays were employed to assess the effect of paeonol on the ability of invasion and colony formation of U251 cells. Superoxide dismutase (SOD) activity, malondialdehyde (MDA) content, total antioxidant capacity (T-AOC), and catalase activity (CAT) were measured to evaluate the effect of paeonol on oxidative stress in U251 cells. Quantitative real-time polymerase chain reaction (RT-q) PCR and western blot were utilized to detect caspase-3 expression levels. Terminal deoxynucleotidyl transferase dUTP nick end-labeling (TUNEL) staining detected the effect of paeonol on U251 cell apoptosis.

Results: Paeonol decreased cell viability, as well as the proliferation, invasion, and colony formation ability of U251 cells. Paeonol reduced MDA content and increased the activities of SOD, CAT, and T-AOC in U251 cells. Caspase-3 expression was lower in human glioma tissues than in normal tissues of the human brain. Paeonol promoted U251 cell apoptosis as revealed by TUNEL staining results and the significant up-regulation of caspase-3 expression in U251 cells.

Conclusion: These results indicated that paeonol has anti-tumor and pro-apoptotic effects in gliomas via oxidative stress regulation and the caspase-3 pathway. Our study, therefore, provides new ideas for the clinical treatment of gliomas.

C1 [Fu, Qiang; Liu, Dong Hai; Mutemwa, Cynthia] Jinggangshan Univ, Jian Key Lab Biomed, Coll Med, Jian, Jiangxi, Peoples R China.

[Li, Zi Xuan; Zheng, Xiao Peng] Jiamusi Univ, Coll Med, Jiamusi, Heilongjiang, Peoples R China.

[Agbo, Elvis] Jinggangshan Univ, Dept Human Anat Histol & Embryol, Coll Med, Jian, Jiangxi, Peoples R China.

C3 Jinggangshan University; Jiamusi University; Jinggangshan University

RP Agbo, E (corresponding author), Jinggangshan Univ, Dept Human Anat Histol & Embryol, Coll Med, Jian, Jiangxi, Peoples R China.

EM dr.elvis.agbo@gmail.com

FU doctoral Start-up Fund of the Natural Science Foundation of Jinggangshan

University, China [JZB1925]

FX This work was supported by the doctoral Start-up Fund of the Natural

Science Foundation of Jinggangshan University, China (Grant No.

JZB1925).

CR Alharthy SA, 2022, EVID-BASED COMPL ALT, V2022, DOI 10.1155/2022/7658899

Bona NP, 2022, NEUROCHEM RES, V47, P1541, DOI 10.1007/s11064-022-03547-7

Chang H-M, 2014, PHARM APPL CHINESE M, VI

Chen BD, 2012, MOLECULES, V17, P4672, DOI 10.3390/molecules17044672

Cheng CS, 2020, CANCER MANAG RES, V12, P641, DOI 10.2147/CMAR.S224416

Cholia RP, 2018, METAB BRAIN DIS, V33, P1307, DOI 10.1007/s11011-018-0233-3

Christofi T, 2019, CANCERS, V11, DOI 10.3390/cancers11101472

Ding Y, 2016, PLOS ONE, V11, DOI 10.1371/journal.pone.0154375

Silva GAF, 2018, CLINICS, V73

Gai ZH, 2019, BIOSCI BIOTECH BIOCH, V83, P1992, DOI 10.1080/09168451.2019.1648203

George S, 2020, ANTIOXIDANTS-BASEL, V9, DOI 10.3390/antiox9111156

Goldar Samira, 2015, Asian Pac J Cancer Prev, V16, P2129

Hafez HM, 2019, HUM EXP TOXICOL, V38, P510, DOI 10.1177/0960327118818254

Klaunig JE, 2018, CURR PHARM DESIGN, V24, P4771, DOI 10.2174/1381612825666190215121712

Lin YS, 2022, CHEM RES TOXICOL, V35, P880, DOI 10.1021/acs.chemrestox.2c00033

Liu Lu, 2020, Zhongguo Yi Xue Ke Xue Yuan Xue Bao, V42, P591, DOI 10.3881/j.issn.1000-503X.11853

Liu MH, 2014, MEDIAT INFLAMM, V2014, DOI 10.1155/2014/651890

Liu XR, 2015, EUR REV MED PHARMACO, V19, P4068

Louis DN, 2016, ACTA NEUROPATHOL, V131, P803, DOI 10.1007/s00401-016-1545-1

Lu JC, 2019, CANCER CELL INT, V19, DOI 10.1186/s12935-019-0972-1

Luo JW, 2015, EUR REV MED PHARMACO, V19, P1630

Mohamed MZ, 2020, ANDROLOGIA, V52, DOI 10.1111/and.13599

Moore LM, 2010, EXPERT OPIN THER TAR, V14, P1247, DOI 10.1517/14728222.2010.527334

Mu ML, 2020, ONCOGENE, V39, P6879, DOI 10.1038/s41388-020-01466-x

Neth BJ, 2022, NEUROLOGIST, V27, P119, DOI 10.1097/NRL.0000000000000393

Ostrom QT, 2014, NEURO-ONCOLOGY, V16, P896, DOI 10.1093/neuonc/nou087

Pak F, 2019, Z NATURFORSCH C, V74, P295, DOI 10.1515/znc-2019-0098

Pisoschi AM, 2015, EUR J MED CHEM, V97, P55, DOI 10.1016/j.ejmech.2015.04.040

Razali NSC, 2022, SCI REP-UK, V12, DOI 10.1038/s41598-022-16274-4

Ren LW, 2022, ACTA PHARMACOL SIN, V43, P194, DOI 10.1038/s41401-021-00752-y

Reuter S, 2010, FREE RADICAL BIO MED, V49, P1603, DOI 10.1016/j.freeradbiomed.2010.09.006

Wang JT, 2012, J PINEAL RES, V53, P180, DOI 10.1111/j.1600-079X.2012.00985.x

Wong RSY, 2011, J EXP CLIN CANC RES, V30, DOI 10.1186/1756-9966-30-87

Wu LQ, 2019, J CELL BIOCHEM, V120, P19044, DOI 10.1002/jcb.29227

Yuan ZH, 2022, J BIOCHEM MOL TOXIC, V36, DOI 10.1002/jbt.23138

Zhang L, 2020, FRONT PHARMACOL, V11, DOI 10.3389/fphar.2020.572616

Zhao P, 2012, BMC CANCER, V12, DOI 10.1186/1471-2407-12-617

Zheng JF, 2020, CHEM PHARM BULL, V68, P1163, DOI 10.1248/cpb.c20-00524

Zheng XY, 2022, WORLD J SURG ONCOL, V20, DOI 10.1186/s12957-022-02533-1

Zhou HX, 2020, OXID MED CELL LONGEV, V2020, DOI 10.1155/2020/7126976

NR 40

TC 0

Z9 0

U1 1

U2 5

PU ZAMENSALAMATI PUBL CO

PI MASHHAD

PA KHORASAN, MASHHAD, SANABAD-EBNE SINA ST, MASHHAD, IRAN

SN 2074-1804

EI 2074-1812

J9 IRAN RED CRESCENT ME

JI Iran. Red Crescent Med. J.

PD APR

PY 2023

VL 25

IS 4

AR e1883

DI 10.32592/ircmj.2023.25.4.1883

PG 8

WC Medicine, General & Internal

WE Science Citation Index Expanded (SCI-EXPANDED)

SC General & Internal Medicine

GA N3CI1

UT WOS:001035829900002

OA hybrid

DA 2025-04-09

ER

PT J

AU Zheng, LJ

Wang, C

Luo, TF

Lu, B

Ma, HX

Zhou, ZJ

Zhu, D

Chi, GF

Ge, PF

Luo, YN

AF Zheng, Linjie

Wang, Chen

Luo, Tianfei

Lu, Bin

Ma, Hongxi

Zhou, Zijian

Zhu, Dong

Chi, Guangfan

Ge, Pengfei

Luo, Yinan

TI JNK Activation Contributes to Oxidative Stress-Induced Parthanatos in

Glioma Cells via Increase of Intracellular ROS Production

SO MOLECULAR NEUROBIOLOGY

LA English

DT Article

DE PARP-1; JNK; ROS; Parthanatos; Glioma; Oxidative stress

ID POLY ADP-RIBOSE; HYDROGEN-PEROXIDE; CANCER-CELLS; APOPTOSIS; DEATH;

INHIBITION; CARCINOMA; AUTOPHAGY; PATHWAY; EXPRESSION

AB Parthanatos is a form of PARP-1-dependent programmed cell death. The induction of parthanatos is emerging as a new strategy to kill gliomas which are the most common type of primary malignant brain tumor. Oxidative stress is thought to be a critical factor triggering parthanatos, but its underlying mechanism is poorly understood. In this study, we used glioma cell lines and H2O2 to investigate the role of JNK in glioma cell parthanatos induced by oxidative stress. We found that exposure to H2O2 not only induced intracellular accumulation of ROS but also resulted in glioma cell death in a concentration- and incubation time-dependent manner, which was accompanied with cytoplasmic formation of PAR polymer, expressional upregulation of PARP-1, mitochondrial depolarization, and AIF translocation to nucleus. Pharmacological inhibition of PARP-1 with 3AB or genetic knockdown of its level with siRNA rescued glioma cell death, as well as suppressed cytoplasmic accumulation of PAR polymer and nuclear translocation of AIF, which were consistent with the definition of parthanatos. Moreover, the phosphorylated level of JNK increased markedly with the extension of H2O2 exposure time. Either attenuation of intracellular ROS with antioxidant NAC or inhibition of JNK phosphorylation with SP600125 or JNK siRNA could significantly prevent H2O2-induced parthanatos in glioma cells. Additionally, inhibition of JNK with SP600125 alleviated intracellular accumulation of ROS and attenuated mitochondrial generation of superoxide. Thus, we demonstrated that JNK activation contributes to glioma cell parthanatos caused by oxidative stress via increase of intracellular ROS generation.

C1 [Zheng, Linjie; Wang, Chen; Lu, Bin; Zhou, Zijian; Ge, Pengfei; Luo, Yinan] Jilin Univ, Hosp 1, Dept Neurosurg, 71 Xinmin Ave, Changchun 130021, Jilin Province, Peoples R China.

[Zheng, Linjie; Wang, Chen; Luo, Tianfei; Lu, Bin; Zhou, Zijian; Ge, Pengfei] Jilin Univ, Hosp 1, Res Ctr Neurosci, Changchun 130021, Peoples R China.

[Luo, Tianfei] Jilin Univ, Hosp 1, Dept Neurol, Changchun 130021, Peoples R China.

[Ma, Hongxi] Jilin Univ, Hosp 1, Dept Pathol, Changchun 130021, Peoples R China.

[Zhu, Dong] Jilin Univ, Hosp 1, Dept Orthopaed, Changchun 130021, Peoples R China.

[Chi, Guangfan] Jilin Univ, Minist Educ, Key Lab Pathobiol, Changchun 130021, Peoples R China.

C3 Jilin University; Jilin University; Jilin University; Jilin University;

Jilin University; Jilin University

RP Ge, PF; Luo, YN (corresponding author), Jilin Univ, Hosp 1, Dept Neurosurg, 71 Xinmin Ave, Changchun 130021, Jilin Province, Peoples R China.; Ge, PF (corresponding author), Jilin Univ, Hosp 1, Res Ctr Neurosci, Changchun 130021, Peoples R China.

EM gepf@jlu.edu.cn; yinanluo@gmail.com

RI Zhu, Dong/AAE-5951-2019; Lu, Bin/JTS-4256-2023; Zhou,

Zijian/JAN-7139-2023

FU National Nature Science Foundation of China [81171234, 81372697,

11432016, 11272134]; Changbaishan Scholar Project of Jilin province

[2013026]; Scientific Research Foundation of Jilin province

[20150414013GH, 20121809]; Bethune project B of Jilin University

[2012203]

FX This work was supported by the National Nature Science Foundation of

China (81171234, 81372697, 11432016, and 11272134), the Changbaishan

Scholar Project of Jilin province (2013026), the Scientific Research

Foundation of Jilin province (20150414013GH, 20121809), and the Bethune

project B of Jilin University (no.2012203).

CR Akhiani AA, 2014, PLOS ONE, V9, DOI 10.1371/journal.pone.0089646

Andrabi SA, 2006, P NATL ACAD SCI USA, V103, P18308, DOI 10.1073/pnas.0606526103

Andrabi SA, 2014, P NATL ACAD SCI USA, V111, P10209, DOI 10.1073/pnas.1405158111

Barbouti A, 2002, FREE RADICAL BIO MED, V33, P691, DOI 10.1016/S0891-5849(02)00967-X

Beck C, 2014, EXP CELL RES, V329, P18, DOI 10.1016/j.yexcr.2014.07.003

Byun YJ, 2009, NEUROSCI LETT, V461, P131, DOI 10.1016/j.neulet.2009.06.011

Chen Y, 2008, CELL DEATH DIFFER, V15, P171, DOI 10.1038/sj.cdd.4402233

Chiu LY, 2011, BIOCHEM PHARMACOL, V81, P459, DOI 10.1016/j.bcp.2010.10.016

Datta K, 2002, INT J BIOCHEM CELL B, V34, P148, DOI 10.1016/S1357-2725(01)00106-6

Dixit D, 2014, CELL DEATH DIS, V5, DOI 10.1038/cddis.2014.179

Fatokun AA, 2014, BRIT J PHARMACOL, V171, P2000, DOI 10.1111/bph.12416

Galia A, 2012, EUR J HISTOCHEM, V56, P45, DOI 10.4081/ejh.2012.e9

Hanawa N, 2008, J BIOL CHEM, V283, P13565, DOI 10.1074/jbc.M708916200

Karpel-Massler G, 2014, PLOS ONE, V9, DOI 10.1371/journal.pone.0114583

Kase M, 2011, RADIOTHER ONCOL, V101, P127, DOI 10.1016/j.radonc.2011.06.024

Lee Y, 2014, BMB REP, V47, P424, DOI 10.5483/BMBRep.2014.47.8.119

Lennicke C, 2015, CELL COMMUN SIGNAL, V13, DOI 10.1186/s12964-015-0118-6

Lu P, 2014, J NEUROSCI, V34, P15975, DOI 10.1523/JNEUROSCI.2499-14.2014

Ma DD, 2016, CANCER LETT, V371, P194, DOI 10.1016/j.canlet.2015.11.044

Mao YB, 2006, CELL BIOL INT, V30, P332, DOI 10.1016/j.cellbi.2005.12.008

Mashimo M, 2013, P NATL ACAD SCI USA, V110, P18964, DOI 10.1073/pnas.1312783110

McKeague AL, 2008, BRIT J CANCER, V88, P125

Mohammad G, 2013, MEDIAT INFLAMM, V2013, DOI 10.1155/2013/510451

Nomura J, 2013, PLOS ONE, V8, DOI 10.1371/journal.pone.0075527

Palit S, 2015, J CELL PHYSIOL, V230, P1729, DOI 10.1002/jcp.24818

Paul M, 2015, PLOS ONE, V10, DOI 10.1371/journal.pone.0127558

Singh M, 2007, MITOCHONDRION, V7, P367, DOI 10.1016/j.mito.2007.07.003

Tentori L, 2014, BMC CANCER, V14, DOI 10.1186/1471-2407-14-151

Wang XY, 2008, J EXP CLIN CANC RES, V27, DOI 10.1186/1756-9966-27-44

Wang YF, 2009, EXP NEUROL, V218, P193, DOI 10.1016/j.expneurol.2009.03.020

Wen PY, 2008, NEW ENGL J MED, V359, P492, DOI 10.1056/NEJMra0708126

Win S, 2015, J HEPATOL, V62, P1367, DOI 10.1016/j.jhep.2015.01.032

Yang ZY, 2014, J IMMUNOL, V193, P6114, DOI 10.4049/jimmunol.1400359

Zhang C, 2013, CANCER LETT, V340, P51, DOI 10.1016/j.canlet.2013.06.021

Zhang HY, 2009, TOXICOL SCI, V110, P376, DOI 10.1093/toxsci/kfp101

Zhang L, 2014, TOXICOL LETT, V228, P248, DOI 10.1016/j.toxlet.2014.05.015

Zhao HL, 2015, KIDNEY INT, V87, P738, DOI 10.1038/ki.2014.388

Zhao N, 2015, ONCOTARGET, V6, P18445, DOI 10.18632/oncotarget.4315

Zhou J, 2006, BOUND VALUE PROBL, DOI 10.1155/BVP/2006/21830

NR 39

TC 50

Z9 55

U1 1

U2 35

PU HUMANA PRESS INC

PI TOTOWA

PA 999 RIVERVIEW DRIVE SUITE 208, TOTOWA, NJ 07512 USA

SN 0893-7648

EI 1559-1182

J9 MOL NEUROBIOL

JI Mol. Neurobiol.

PD JUL

PY 2017

VL 54

IS 5

BP 3492

EP 3505

DI 10.1007/s12035-016-9926-y

PG 14

WC Neurosciences

WE Science Citation Index Expanded (SCI-EXPANDED)

SC Neurosciences & Neurology

GA EV9IQ

UT WOS:000402100100034

PM 27181592

DA 2025-04-09

ER

PT J

AU Zhu, Y

Zhuang, JX

Wang, Q

Zhang, HY

Yang, P

AF Zhu, Yu

Zhuang, Jun-Xue

Wang, Qin

Zhang, Hai-Yan

Yang, Ping

TI Inhibitory Effect of Benzyl Isothiocyanate on Proliferation in

vitro of Human Glioma Cells

SO ASIAN PACIFIC JOURNAL OF CANCER PREVENTION

LA English

DT Article

DE Glioma; benzyl isothiocyanate; oxidative stress; preventive potential

ID OXIDATIVE STRESS; ANTIOXIDANT; BIOMARKERS; RATS

AB Malignant glioma, also known as brain cancer, is the most common intracranial tumor, having an extremely high mortality and recurrence rate. The survival rate of the affected patients is very low and treatment is difficult. Hence, growth inhibition of glioma has become a hot topic in the study of brain cancer treatment. Among the various isothiocyanate compounds, it has been confirmed that benzyl isothiocyanate (BITC) can inhibit the growth of a variety of tumors, including leukemia, glioma and lung cancer, both inside and outside the body. This study explored inhibitory effects of BITC on human glioma U87MG cells, as well as potential mechanisms. It was found that BITC could inhibit proliferation, induce apoptosis and arrest cell cycling of U87MG cells. In addition, it inhibited the expression of SOD and GSH, and caused oxidative stress to tumor cells. Therefore, it is believed that BITC can inhibit the growth of U87MG cells outside the body. Its mechanism may be related to the fact that BITC can cause oxidative stress to tumor cells.

C1 [Zhu, Yu; Wang, Qin; Yang, Ping] Tianjin Huan Hu Hosp, Dept Clin Lab, Tianjin, Peoples R China.

[Zhuang, Jun-Xue; Zhang, Hai-Yan] Tianjin Baodi Hosp, Dept Pharm, Tianjin, Peoples R China.

RP Zhu, Y (corresponding author), Tianjin Huan Hu Hosp, Dept Clin Lab, Tianjin, Peoples R China.

EM zhuyutj@126.com

RI Zhang, Haiyan/IUN-2627-2023

CR Deeb Dorrah, 2012, J Exp Ther Oncol, V10, P51

Deng JS, 2012, J ETHNOPHARMACOL, V142, P795, DOI 10.1016/j.jep.2012.06.003

Fidler IJ, 2011, SEMIN CANCER BIOL, V21, P107, DOI 10.1016/j.semcancer.2010.12.009

Gayathri R, 2009, ASIAN PAC J CANCER P, V10, P933

Zandalinas SI, 2012, J AGR FOOD CHEM, V60, P8648, DOI 10.1021/jf302482y

Kohsaka S, 2013, CANCER LETT, V331, P68, DOI 10.1016/j.canlet.2012.12.005

Martinez-Outschoorn UE, 2012, CELL CYCLE, V11, P4402, DOI 10.4161/cc.22776

Noureen A, 2013, PAK J PHARM SCI, V26, P113

Pawlik A, 2012, FOOD CHEM TOXICOL, V50, P3577, DOI 10.1016/j.fct.2012.07.043

Singh SV, 2012, JNCI-J NATL CANCER I, V104, P1228, DOI 10.1093/jnci/djs321

Tabatabai G, 2012, CURR NEUROL NEUROSCI, V12, P302, DOI 10.1007/s11910-012-0263-x

NR 11

TC 28

Z9 32

U1 0

U2 4

PU ASIAN PACIFIC ORGANIZATION CANCER PREVENTION

PI GYEONGGI-DO

PA APJCP HEAD OFFICE, KOREAN NATL CANCER CENTER, 323 ILAN -RO,

ILSANDONG-GU, GOYANG-SI, GYEONGGI-DO, 410-769, SOUTH KOREA

SN 1513-7368

J9 ASIAN PAC J CANCER P

JI Asian Pac. J. Cancer Prev.

PY 2013

VL 14

IS 4

BP 2607

EP 2610

DI 10.7314/APJCP.2013.14.4.2607

PG 4

WC Oncology

WE Science Citation Index Expanded (SCI-EXPANDED)

SC Oncology

GA 188NC

UT WOS:000322198700080

PM 23725183

OA gold

DA 2025-04-09

ER

PT J

AU Hwang, E

Kim, S

AF Hwang, E.

Kim, S.

TI Effects of Momordica charantia extract on proliferation,

oxidative stress, and apoptosis of C6 glioma cells

SO FEBS OPEN BIO

LA English

DT Meeting Abstract

C1 [Hwang, E.; Kim, S.] Hoseo Univ, Asan, South Korea.

C3 Hoseo University

FU "Cooperative Research Program for Agriculture Science & Technology

Development" Rural Development Administration, Republic of Korea

[PJ01323005]

FX This work was carried out with the support of "Cooperative Research

Program for Agriculture Science & Technology Development (Project No.

PJ01323005)" Rural Development Administration, Republic of Korea.

NR 0

TC 0

Z9 0

U1 0

U2 0

PU WILEY

PI HOBOKEN

PA 111 RIVER ST, HOBOKEN 07030-5774, NJ USA

SN 2211-5463

J9 FEBS OPEN BIO

JI FEBS Open Bio

PD JUL

PY 2019

VL 9

SU 1

MA P-01-038

BP 79

EP 79

PG 1

WC Biochemistry & Molecular Biology

WE Science Citation Index Expanded (SCI-EXPANDED)

SC Biochemistry & Molecular Biology

GA IZ3GH

UT WOS:000486972401038

DA 2025-04-09

ER

PT J

AU Lei, KC

Tora, MS

Neill, SG

Nagarajan, PP

Federici, T

Canoll, P

Boulis, NM

AF Lei, Kecheng

Tora, Muhibullah S.

Neill, Stewart G.

Nagarajan, Purva P.

Federici, Thais

Canoll, Peter

Boulis, Nicholas M.

TI Oxidative stress triggers tumor edge progression of tumor

microenvironment in the minipig spinal cord glioma model

SO CANCER RESEARCH

LA English

DT Meeting Abstract

CT Annual Meeting of the American-Association-for-Cancer-Research (AACR)

CY APR 08-13, 2022

CL New Orleans, LA

SP Amer Assoc Canc Res

C1 [Lei, Kecheng; Tora, Muhibullah S.; Neill, Stewart G.; Nagarajan, Purva P.; Federici, Thais; Boulis, Nicholas M.] Emory Univ, Atlanta, GA 30322 USA.

[Canoll, Peter] Columbia Univ, New York, NY USA.

C3 Emory University; Columbia University

RI Lei, Kecheng/ABI-4314-2020; Tora, Muhibullah/AAV-5949-2020

NR 0

TC 0

Z9 0

U1 0

U2 0

PU AMER ASSOC CANCER RESEARCH

PI PHILADELPHIA

PA 615 CHESTNUT ST, 17TH FLOOR, PHILADELPHIA, PA 19106-4404 USA

SN 0008-5472

EI 1538-7445

J9 CANCER RES

JI Cancer Res.

PD JUN 15

PY 2022

VL 82

IS 12

SU S

MA 1609

PG 1

WC Oncology

WE Science Citation Index Expanded (SCI-EXPANDED); Conference Proceedings Citation Index - Science (CPCI-S)

SC Oncology

GA 6R7WQ

UT WOS:000892509506545

DA 2025-04-09

ER

PT J

AU Hsu, TI

AF Hsu, Tsung-I

TI Cytochrome P450 17A1 (CYP17A1) Attenuates Oxidative Stress through

Regulating Protein Stability of SAR1a/b in Glioblastoma

SO FASEB JOURNAL

LA English

DT Meeting Abstract

CT Annual Meeting on Experimental Biology

CY APR 04-07, 2020

CL San Diego, CA

SP Amer Assoc Anatomists, Amer Physiol Soc, Amer Soc Biochem & Mol Biol, Amer Soc Investigat Pathol, Amer Soc Nutr, Amer Soc Pharmacol & Expt Therapeut

C1 [Hsu, Tsung-I] Taipei Med Univ, Taipei, Taiwan.

C3 Taipei Medical University

RI Hsu, Tsung-I/AAK-9942-2020

OI Hsu, Tsung-I/0000-0002-7524-7740

FU Ministry of Science and Technology of Taiwan [MOST 108-2628-B-038-005-]

FX This study was supported by the Ministry of Science and Technology of

Taiwan (MOST 108-2628-B-038-005-).

NR 0

TC 0

Z9 0

U1 0

U2 2

PU WILEY

PI HOBOKEN

PA 111 RIVER ST, HOBOKEN 07030-5774, NJ USA

SN 0892-6638

EI 1530-6860

J9 FASEB J

JI Faseb J.

PD APR

PY 2020

VL 34

SU S1

DI 10.1096/fasebj.2020.34.s1.02017

PG 2

WC Biochemistry & Molecular Biology; Biology; Cell Biology

WE Science Citation Index Expanded (SCI-EXPANDED); Conference Proceedings Citation Index - Science (CPCI-S)

SC Biochemistry & Molecular Biology; Life Sciences & Biomedicine - Other

Topics; Cell Biology

GA MG4SK

UT WOS:000546023103623

OA Bronze

DA 2025-04-09

ER

PT J

AU Tiek, D

Song, X

Wu, RX

Yu, XZ

Iglesia, R

Hu, B

Cheng, SY

AF Tiek, Deanna

Song, Xiao

Wu, Runxin

Yu, Xiaozhou

Iglesia, Rebeca

Hu, Bo

Cheng, Shiyuan

TI OXIDATIVE STRESS INDUCED PROTEIN AGGREGATION VIA GGCT PRODUCED

PYROGLUTAMIC ACID IN DRUG RESISTANT GLIOBLASTOMA

SO NEURO-ONCOLOGY

LA English

DT Meeting Abstract

CT 29th Annual Meeting and Education Day of the Society-for-Neuro-Oncology

(SNO)

CY NOV 21-24, 2024

CL Houston, TX

SP Soc Neuro Oncol

C1 [Tiek, Deanna; Song, Xiao; Wu, Runxin; Yu, Xiaozhou; Iglesia, Rebeca; Hu, Bo; Cheng, Shiyuan] Northwestern, Chicago, IL USA.

C3 Northwestern University

NR 0

TC 0

Z9 0

U1 0

U2 0

PU OXFORD UNIV PRESS INC

PI CARY

PA JOURNALS DEPT, 2001 EVANS RD, CARY, NC 27513 USA

SN 1522-8517

EI 1523-5866

J9 NEURO-ONCOLOGY

JI Neuro-Oncology

PD NOV 11

PY 2024

VL 26

SU _8

SI SI

MA TMET-31

DI 10.1093/neuonc/noae165.1169

PG 1

WC Oncology; Clinical Neurology

WE Science Citation Index Expanded (SCI-EXPANDED); Conference Proceedings Citation Index - Science (CPCI-S)

SC Oncology; Neurosciences & Neurology

GA N2G4O

UT WOS:001362581000020

DA 2025-04-09

ER

PT J

AU Majewska, E

Márquez, J

Albrecht, J

Szeliga, M

AF Majewska, Ewelina

Marquez, Javier

Albrecht, Jan

Szeliga, Monika

TI Transfection with GLS2 Glutaminase (GAB) Sensitizes Human Glioblastoma

Cell Lines to Oxidative Stress by a Common Mechanism Involving

Suppression of the PI3K/AKT Pathway

SO CANCERS

LA English

DT Article

DE GLS2 glutaminase; human glioblastoma; PI3K/AKT signaling pathway;

oxidative stress

ID INDUCED AKT PHOSPHORYLATION; NF-KAPPA-B; GENE-EXPRESSION; MESSENGER-RNA;

INHIBITION; METABOLISM; ISOFORMS; PTEN; PDGF; PROLIFERATION

AB GLS-encoded glutaminase promotes tumorigenesis, while GLS2-encoded glutaminase displays tumor-suppressive properties. In glioblastoma (GBM), the most aggressive brain tumor, GLS is highly expressed and in most cases GLS2 is silenced. Previously, it was shown that transfection with a sequence encoding GAB, the main GLS2 isoform, decreased the survival, growth, and ability to migrate of human GBM cells T98G and increased their sensitivity towards an alkylating agent temozolomide (TMZ) and oxidative stress compared to the controls, by a not well-defined mechanism. In this study we report that GAB transfection inhibits growth and increases susceptibility towards TMZ and H2O2-mediated oxidative stress of two other GBM cell lines, U87MG and LN229. We also show that in GAB-transfected cells treated with H2O2, the PI3K/AKT pathway is less induced compared to the pcDNA-transfected counterparts and that pretreatment with PDGF-BB, an activator of AKT, protects GAB-transfected cells from death caused by the H2O2 treatment. In conclusion, our results show that (i) GAB suppresses the malignant phenotype of the GBM cells of different tumorigenic potentials and genetic backgrounds and (ii) the GAB-mediated increase of sensitivity to oxidative stress is causally related to the inhibition of the PI3K/AKT pathway. The upregulation of the GLS2 expression and the inhibition of the PI3K/AKT pathway may become a novel combined therapeutic strategy for anti-glioma preclinical investigations.

C1 [Majewska, Ewelina; Albrecht, Jan; Szeliga, Monika] Polish Acad Sci, Mossakowski Med Res Ctr, Dept Neurotoxicol, 5 Pawinskiego St, PL-02106 Warsaw, Poland.

[Marquez, Javier] Univ Malaga, Inst Invest Biomed Malaga IBIMA, Cancerom Lab, Dept Mol Biol & Biochem,Fac Sci, Campus Teatinos, E-29071 Malaga, Spain.

C3 Polish Academy of Sciences; Mossakowski Medical Research Institute of

the Polish Academy of Sciences; Instituto de Investigacion Biomedica de

Malaga y Plataforma en Nanomedicina (IBIMA); Universidad de Malaga

RP Szeliga, M (corresponding author), Polish Acad Sci, Mossakowski Med Res Ctr, Dept Neurotoxicol, 5 Pawinskiego St, PL-02106 Warsaw, Poland.

EM emajewska@imdik.pan.pl; marquez@uma.es; jalbrecht@imdik.pan.pl;

mszeliga@imdik.pan.pl

RI Albrecht, Jan/AAQ-1158-2021; MARQUEZ GOMEZ, FRANCISCO JAVIER/K-3289-2012

OI Majewska, Ewelina/0000-0002-3777-1744; MARQUEZ GOMEZ, FRANCISCO

JAVIER/0000-0003-3317-3963; Szeliga, Monika/0000-0003-2973-4692

FU National Science Centre of Poland [2016/23/N/NZ5/01428,

2013/11/D/NZ7/00925, 2017/25/B/NZ7/00388]; Spanish Ministry of Economy

and Competitivity [SAF2015-64501-R]; National Leading Research Centre

(KNOW-MMRC1) project

FX This research was funded by the National Science Centre of Poland grant

numbers 2016/23/N/NZ5/01428 (to E.M.) and 2013/11/D/NZ7/00925 and

2017/25/B/NZ7/00388 (to M.S.) and by the National Leading Research

Centre (KNOW-MMRC1) project (E.M.). J.M. was supported by Grant

SAF2015-64501-R from the Spanish Ministry of Economy and Competitivity.

CR Aldape K, 2015, ACTA NEUROPATHOL, V129, P829, DOI 10.1007/s00401-015-1432-1

Aledo JC, 2000, MAMM GENOME, V11, P1107, DOI 10.1007/s003350010190

Bai D, 2009, INT J CANCER, V125, P2863, DOI 10.1002/ijc.24748

Campos-Sandoval JA, 2015, NEUROCHEM INT, V88, P1, DOI 10.1016/j.neuint.2015.03.006

Cardona C, 2015, GLIA, V63, P365, DOI 10.1002/glia.22758

Cheng TL, 2011, P NATL ACAD SCI USA, V108, P8674, DOI 10.1073/pnas.1016627108

DeBerardinis RJ, 2010, ONCOGENE, V29, P313, DOI 10.1038/onc.2009.358

Donadio AC, 2008, J CELL BIOCHEM, V103, P800, DOI 10.1002/jcb.21449

Esencay M, 2013, BMC CANCER, V13, DOI 10.1186/1471-2407-13-347

Gao P, 2009, NATURE, V458, P762, DOI 10.1038/nature07823

Hensley CT, 2013, J CLIN INVEST, V123, P3678, DOI 10.1172/JCI69600

Hu WW, 2010, P NATL ACAD SCI USA, V107, P7455, DOI 10.1073/pnas.1001006107

Ishii N, 1999, BRAIN PATHOL, V9, P469, DOI 10.1111/j.1750-3639.1999.tb00536.x

Jacque N, 2015, BLOOD, V126, P1346, DOI 10.1182/blood-2015-01-621870

Kahlert UD, 2016, INT J CANCER, V138, P1246, DOI 10.1002/ijc.29873

Liu J, 2014, ONCOTARGET, V5, P2635, DOI 10.18632/oncotarget.1862

Livak KJ, 2001, METHODS, V25, P402, DOI 10.1006/meth.2001.1262

Lobo C, 2000, BIOCHEM J, V348, P257, DOI 10.1042/0264-6021:3480257

Majewska E, 2017, NEUROCHEM RES, V42, P918, DOI 10.1007/s11064-016-2044-4

Martín-Rufián M, 2014, J MOL MED, V92, P277, DOI 10.1007/s00109-013-1105-2

Martín-Rufián M, 2012, PLOS ONE, V7, DOI 10.1371/journal.pone.0038380

Massacesi C, 2016, ONCOTARGETS THER, V9, P203, DOI 10.2147/OTT.S89967

Olalla L, 2002, J BIOL CHEM, V277, P38939, DOI 10.1074/jbc.C200373200

Olalla L, 2001, FEBS LETT, V488, P116, DOI 10.1016/S0014-5793(00)02373-5

Pérez-Gómez C, 2005, BIOCHEM J, V386, P535, DOI 10.1042/BJ20040996

Ramao A, 2012, PROTEOME SCI, V10, DOI 10.1186/1477-5956-10-53

Rauch BH, 2000, FEBS LETT, V481, P3, DOI 10.1016/S0014-5793(00)01957-8

Sadidi M, 2009, BIOCHIMIE, V91, P577, DOI 10.1016/j.biochi.2009.01.010

Sonoda Y, 2001, CANCER RES, V61, P6674

Sonoda Y, 1999, J BIOL CHEM, V274, P10566, DOI 10.1074/jbc.274.15.10566

Sotelo NS, 2012, J CELL BIOCHEM, V113, P2661, DOI 10.1002/jcb.24141

Suzuki S, 2010, P NATL ACAD SCI USA, V107, P7461, DOI 10.1073/pnas.1002459107

Szeliga M, 2005, NEUROSCI LETT, V374, P171, DOI 10.1016/j.neulet.2004.10.051

Szeliga M, 2008, NEUROCHEM RES, V33, P808, DOI 10.1007/s11064-007-9507-6

Szeliga M, 2016, MOL CARCINOGEN, V55, P1309, DOI 10.1002/mc.22372

Szeliga M, 2012, J NEUROCHEM, V123, P428, DOI 10.1111/j.1471-4159.2012.07917.x

Szeliga M, 2009, GLIA, V57, P1014, DOI 10.1002/glia.20825

Szeliga M, 2009, NEUROCHEM INT, V55, P71, DOI 10.1016/j.neuint.2009.01.008

Takahashi Y, 2006, EMBO J, V25, P910, DOI 10.1038/sj.emboj.7600979

Wang JB, 2010, CANCER CELL, V18, P207, DOI 10.1016/j.ccr.2010.08.009

Zheng LS, 2010, J NEUROSCI RES, V88, P1273, DOI 10.1002/jnr.22302

NR 41

TC 19

Z9 21

U1 0

U2 5

PU MDPI

PI BASEL

PA ST ALBAN-ANLAGE 66, CH-4052 BASEL, SWITZERLAND

SN 2072-6694

J9 CANCERS

JI Cancers

PD JAN

PY 2019

VL 11

IS 1

AR 115

DI 10.3390/cancers11010115

PG 18

WC Oncology

WE Science Citation Index Expanded (SCI-EXPANDED)

SC Oncology

GA HJ5PG

UT WOS:000457233300063

PM 30669455

OA Green Published, gold, Green Submitted

DA 2025-04-09

ER

PT J

AU Erukainure, O

Ebuehi, O

Choudhary, I

Ashraf, N

Navqi, A

Muham-Mad, A

Zaruwa, M

Elemo, G

AF Erukainure, Ochuko

Ebuehi, Osaretin

Choudhary, Iqbal

Ashraf, Nadia

Navqi, Asma

Muham-Mad, Aliyu

Zaruwa, Moses

Elemo, Gloria

TI Dietary Fatty Acids from a local Nigerian Spice (Clerodendrum volubile)

Suppresses Tumor Cell Migration and Invasion, Attenuates Oxidative

Stress, and Regulates Cell Cycle Progression in Human Neuronal

Glioblastoma Cells

SO ANNALS OF NUTRITION AND METABOLISM

LA English

DT Meeting Abstract

DE Spices; Unsaturated fatty acids; Cancer; Tumor migration; and Oxidative

stress

C1 [Erukainure, Ochuko; Elemo, Gloria] FIIRO, Dept Food Technol, Lagos, Nigeria.

[Ebuehi, Osaretin] Univ Lagos, Dept Biochem, Lagos, Nigeria.

[Choudhary, Iqbal; Ashraf, Nadia; Navqi, Asma] Univ Karachi, Int Ctr Chem & Biol Sci, Karachi 75270, Pakistan.

[Muham-Mad, Aliyu] Ahmadu Bello Univ, Dept Biochem, Zaria, Nigeria.

[Zaruwa, Moses] Adamawa State Univ, Fac Sci, Mubi, Nigeria.

C3 University of Lagos; University of Karachi; Ahmadu Bello University

RI Muhammad, Aliyu/AAC-6717-2019; Erukainure, Ochuko/G-9888-2014

NR 0

TC 0

Z9 0

U1 0

U2 2

PU KARGER

PI BASEL

PA ALLSCHWILERSTRASSE 10, CH-4009 BASEL, SWITZERLAND

SN 0250-6807

EI 1421-9697

J9 ANN NUTR METAB

JI Ann. Nutr. Metab.

PY 2015

VL 67

SU 1

MA 149/1239

BP 454

EP 455

PG 2

WC Endocrinology & Metabolism; Nutrition & Dietetics

WE Science Citation Index Expanded (SCI-EXPANDED)

SC Endocrinology & Metabolism; Nutrition & Dietetics

GA DK5VM

UT WOS:000374988802139

DA 2025-04-09

ER

PT J

AU Tan, Q

Yan, XQ

Song, L

Yi, HX

Li, P

Sun, GB

Yu, DF

Li, L

Zeng, Z

Guo, ZL

AF Tan, Qian

Yan, Xiaoqiong

Song, Lin

Yi, Hongxiang

Li, Ping

Sun, Guobin

Yu, Danfang

Li, Le

Zeng, Zheng

Guo, Zhenli

TI Induction of Mitochondrial Dysfunction and Oxidative Damage by

Antibiotic Drug Doxycycline Enhances the Responsiveness of Glioblastoma

to Chemotherapy

SO MEDICAL SCIENCE MONITOR

LA English

DT Article

DE Doxycycline; Glioblastoma; Mitochondria; Oxidative Stress

ID INHIBITS PROLIFERATION; CANCER; CELLS; METABOLISM; APOPTOSIS; STRESS

AB Background: Inducing mitochondrial dysfunction has been recently demonstrated to be an alternative therapeutic strategy for cancer treatment. Doxycycline is an antibiotic that has been shown to have anti-cancer activities in various cancers by way of targeting mitochondria. In this work, we examined whether doxycycline can be repurposed for glioblastoma treatment.

Material/Methods: The effects of doxycycline on the growth, survival, and mitochondrial metabolisms of glioblastoma were investigated. The efficacy of a combination of doxycycline with temozolomide was examined using xenograft mouse model in total number of 40 mice.

Results: Doxycycline targeted glioblastoma cell lines, regardless of their origin, through inhibiting growth and inducing cell death, accompanied by a significant decrease in proliferating cell nuclear antigen (PCNA) and increase in cleaved caspase-3. In addition, doxycycline significantly sensitized glioblastoma cell response to temozolomide in vitro and in vivo. Mechanistically, doxycycline disrupted mitochondrial functions through decreasing mitochondrial membrane potential and mitochondrial respiration. Inducing mitochondrial dysfunctions by using doxycycline led to energy crisis, oxidative stress, and damage as shown by the decreased levels of ATP and the elevated levels of mitochondrial superoxide, intracellular ROS, 8-OHdG, protein carbonylation, and lipid peroxidation. An antioxidant N-acetyl-L-cysteine (NAC) significantly abolished the anti-proliferative and pro-apoptotic effects of doxycycline, demonstrating that doxycycline acts on glioblastoma via inducing oxidative stress.

Conclusions: In our study, we show that the antibiotic doxycycline is effective in targeting glioblastoma through inducing mitochondrial dysfunctions and oxidative stress. Our work also demonstrated the importance of mitochondrial metabolism in glioblastoma.

C1 [Tan, Qian; Yan, Xiaoqiong; Song, Lin; Yi, Hongxiang; Li, Ping; Sun, Guobin; Yu, Danfang; Li, Le; Zeng, Zheng; Guo, Zhenli] Hubei Hosp Integrated Tradit Chinese & Western Me, Dept Neurol, Wuhan, Hubei, Peoples R China.

RP Guo, ZL (corresponding author), Hubei Hosp Integrated Tradit Chinese & Western Me, Dept Neurol, Wuhan, Hubei, Peoples R China.

EM gzlys156@126.com

RI Li, Ping/GON-7455-2022

FU National Natural Science Funds of China [81603095]

FX This work was supported by a research grant provided by National Natural

Science Funds of China (Grant No. 81603095)

CR Ahler E, 2013, PLOS ONE, V8, DOI 10.1371/journal.pone.0064561

Brennan CW, 2013, CELL, V155, P462, DOI 10.1016/j.cell.2013.09.034

CHOPRA I, 1992, J ANTIMICROB CHEMOTH, V29, P245, DOI 10.1093/jac/29.3.245

Dolecek TA, 2012, NEURO-ONCOLOGY, V14, pv1, DOI 10.1093/neuonc/nos218

Duivenvoorden WCM, 2002, CANCER RES, V62, P1588

Gerstner ER, 2012, CANCER J, V18, P45, DOI 10.1097/PPO.0b013e3182431c6f

Kerksick Chad, 2005, J Int Soc Sports Nutr, V2, P38, DOI 10.1186/1550-2783-2-2-38

Kudryavtseva AV, 2016, ONCOTARGET, V7, P44879, DOI 10.18632/oncotarget.9821

Lamb R, 2015, ONCOTARGET, V6, P4569, DOI 10.18632/oncotarget.3174

Miyazaki T, 2015, AM J CASE REP, V16, P255, DOI 10.12659/AJCR.893026

Moreno-Sánchez R, 2007, FEBS J, V274, P1393, DOI 10.1111/j.1742-4658.2007.05686.x

Patel AP, 2014, SCIENCE, V344, P1396, DOI 10.1126/science.1254257

Pedersen P L, 1978, Prog Exp Tumor Res, V22, P190

Phillips HS, 2006, CANCER CELL, V9, P157, DOI 10.1016/j.ccr.2006.02.019

Skrtic M, 2011, CANCER CELL, V20, P674, DOI 10.1016/j.ccr.2011.10.015

Song MJ, 2016, BIOMED PHARMACOTHER, V84, P1137, DOI 10.1016/j.biopha.2016.10.034

Stefano GB, 2017, MED SCI MONITOR, V23, P101, DOI 10.12659/MSM.899478

Varum S, 2011, PLOS ONE, V6, DOI 10.1371/journal.pone.0020914

Wen SJ, 2013, FUTURE MED CHEM, V5, P53, DOI 10.4155/fmc.12.190

Xie Y, 2015, EBIOMEDICINE, V2, P1351, DOI 10.1016/j.ebiom.2015.08.026

Yang BL, 2015, PLOS ONE, V10, DOI 10.1371/journal.pone.0129138

Zhang XN, 2015, INT J MOL SCI, V16, P27313, DOI 10.3390/ijms161126020

Zhao Y, 2016, CAN J PHYSIOL PHARM, V94, P526, DOI 10.1139/cjpp-2015-0481

Zu XL, 2004, BIOCHEM BIOPH RES CO, V313, P459, DOI 10.1016/j.bbrc.2003.11.136

NR 24

TC 28

Z9 28

U1 1

U2 27

PU INT SCIENTIFIC INFORMATION, INC

PI MELVILLE

PA 150 BROADHOLLOW RD, STE 114, MELVILLE, NY 11747 USA

SN 1643-3750

J9 MED SCI MONITOR

JI Med. Sci. Monitor

PD AUG 26

PY 2017

VL 23

DI 10.12659/MSM.903245

PG 9

WC Medicine, Research & Experimental

WE Science Citation Index Expanded (SCI-EXPANDED)

SC Research & Experimental Medicine

GA FF8HM

UT WOS:000409257500001

PM 28842551

OA Green Submitted, Green Published

DA 2025-04-09

ER

PT J

AU Sen, E

AF Sen, Ellora

TI Targeting oxidative stress in glioblastoma: implications in therapy

SO INTERNATIONAL JOURNAL OF MOLECULAR MEDICINE

LA English

DT Meeting Abstract

C1 [Sen, Ellora] Natl Brain Res Ctr, Manesar 122051, Haryana, India.

C3 Department of Biotechnology (DBT) India; National Brain Research Centre

(NBRC)

NR 0

TC 0

Z9 0

U1 0

U2 0

PU SPANDIDOS PUBL LTD

PI ATHENS

PA POB 18179, ATHENS, 116 10, GREECE

SN 1107-3756

EI 1791-244X

J9 INT J MOL MED

JI Int. J. Mol. Med.

PY 2014

VL 34

SU 1

MA 407

BP S81

EP S81

PG 1

WC Medicine, Research & Experimental

WE Science Citation Index Expanded (SCI-EXPANDED)

SC Research & Experimental Medicine

GA AO3ZM

UT WOS:000341276000308

DA 2025-04-09

ER

PT J

AU Gao, ZM

Wu, JL

Bi, HY

Ding, YQ

Zhang, XN

Li, HL

Yang, LY

Xu, FH

Wang, ZX

AF Gao, Zhenmei

Wu, Jianlin

Bi, Hongyan

Ding, Yuanqing

Zhang, Xiaoning

Li, Haili

Yang, Leiying

Xu, Fenghua

Wang, Zhaoxia

TI Oncogene RPP25 Promotes Glioblastoma Progression by Inhibiting

Ferroptosis and Oxidative Stress

SO JOURNAL OF BIOLOGICAL REGULATORS AND HOMEOSTATIC AGENTS

LA English

DT Article

DE GBM; RPP25; ITGB8; TGF-0; Smad4

ID CANCER-CELLS; TGF-BETA; METASTASIS; DEPENDENCY; EXPRESSION

AB Background: Glioblastoma (GBM) are the most prevalent malignant brain tumors with extensive morphological and genetic heterogeneity. The objective of this study was to investigate the role of the oncogene Ribonuclease P/MRP Subunit P25 (RPP25) in GBM progression and its underlying molecular mechanisms.Methods: Glioma cell lines were used to evaluate the effects of RPP25 on ferroptosis-related proteins, oxidative stress levels, and reactive oxygen species (ROS) content. In addition, GBM mouse models were established, including an RPP25 overexpression group (OE-RPP25) and an RPP25 overexpression with integrin beta-8 (ITGB8) knockdown group (OE-RPP25+sh-ITGB8). Tumor volume and weight were measured to assess tumor growth. The levels of ferroptosis and oxidative stress were also evaluated using lipid peroxidation and ROS content assays.Results: It was observed that RPP25 overexpression significantly elevated both tumor volume and weight (p < 0.05) compared to the control group. However, when ITGB8 was knocked down in the OE-RPP25 group, tumor volume and weight were reduced. Furthermore, RPP25 overexpression upregulated the expression of ITGB8, hypoxia-inducible factor (FIH1), and glutathione peroxidase 4 (GPX4), while ITGB8 knockdown reversed these effects. Additionally, RPP25 overexpression suppressed the expression of apoptosis-related proteins B-cell lymphoma-2 associated X (Bax) and Caspase-3 and increased (p < 0.05) the expression of anti-apoptotic protein B-cell lymphoma-2 (Bcl-2). However, ITGB8 knockdown promoted apoptosis in tumor cells by counteracting these effects. Moreover, RPP25 overexpression inhibited ferroptosis and oxidative stress, while ITGB8 knockdown reversed these effects, promoting lipid peroxidation and glutathione depletion.Conclusions: These findings suggest that RPP25 plays a crucial role in GBM progression by upregulating the ITGB8/transforming growth factor-beta (TGF-0)/Smad4 axis, inhibiting ferroptosis and oxidative stress, and suppressing apoptosis. These findings provide important insights into the interaction between RPP25 and ITGB8 and their potential applications in GBM therapy. Targeting the RPP25-ITGB8 axis may represent a promising strategy for the treatment of GBM by modulating ferroptosis and apoptosis pathways. The findings also suggest that ferroptosis and oxidative stress may be potential targets for GBM therapy.

C1 [Gao, Zhenmei; Bi, Hongyan] Shandong Univ Tradit Chinese Med, Dept Rehabil, Affiliated Hosp, Jinan 250014, Shandong, Peoples R China.

[Wu, Jianlin] Shandong Univ Tradit Chinese, Med Basic Med Coll, Jinan 250300, Shandong, Peoples R China.

[Ding, Yuanqing] Shandong Univ Tradit Chinese Med, Dept Tradit Chinese Med Class, Affiliated Hosp, Jinan 250014, Shandong, Peoples R China.

[Zhang, Xiaoning] Shandong First Med Univ & Shandong Acad Med Sci, Sch Clin & Basic Med Sci, Dept Med Expt, Jinan 250117, Shandong, Peoples R China.

[Li, Haili] Shandong First Med Univ & Shandong Acad Med Sci, Sch Clin & Basic Med Sci, Dept Human Anat, Jinan 250117, Shandong, Peoples R China.

[Yang, Leiying; Xu, Fenghua; Wang, Zhaoxia] Shandong First Med Univ & Shandong Acad Med Sci, Sch Clin & Basic Med Sci, Dept Morphol Lab, Jinan 250117, Shandong, Peoples R China.

C3 Shandong University of Traditional Chinese Medicine; Shandong University

of Traditional Chinese Medicine; Shandong University of Traditional

Chinese Medicine; Shandong First Medical University & Shandong Academy

of Medical Sciences; Shandong First Medical University & Shandong

Academy of Medical Sciences; Shandong First Medical University &

Shandong Academy of Medical Sciences

RP Wang, ZX (corresponding author), Shandong First Med Univ & Shandong Acad Med Sci, Sch Clin & Basic Med Sci, Dept Morphol Lab, Jinan 250117, Shandong, Peoples R China.

EM wzxia66@126.com

RI Xu, Fenghua/HLP-4335-2023

FU ogy Project [2020Z04, 2019-0197]; Qilu Medical School TCM Aca-demic

School Construction Project [132, 45, 46]; Shandong Traditional Chinese

Medicine Science and Technology Project [2020M063]; Natural Sci-ence

Foundation of Shandong Province [ZR2021QH242]; 2020 Shandong Medical and

Health Science and Technology Development Plan Project [202002080697]

FX This research was funded by 2022 National Famous and Old Traditional

Chinese Medicine Expert Inheritance Studio Construction Project

(Approval No. Guozhong Pharmaceutical Renjiaohan [2002] No. 75) ; 2020

Shandong Traditional Chinese Medicine Science and Technology Project

(Project No. 2020Z04) ; 2019 Shandong Traditional Chinese Medicine

Science and Technology Project (Project No. 2019-0197) ; Qilu Medical

School TCM Academic School Construction Project (Approval No. LWH [2020]

No. 132, LWH [2012] No. 45) ; 2020 Shandong Traditional Chinese Medicine

Science and Technology Project (Approval No. LWH [2021] No. 46, Project

No. N4) ; 2020 Shandong Traditional Chinese Medicine Science and

Technology Project (Project No. 2020M063) ; Natural Science Foundation

of Shandong Province in 2021 (Project No. ZR2021QH242) ; 2020 Shandong

Medical and Health Science and Technology Development Plan Project

(Project No. 202002080697) .r ogy Project (Project No. 2020Z04) ; 2019

Shandong Tra-ditional Chinese Medicine Science and Technology Project

(Project No. 2019-0197) ; Qilu Medical School TCM Aca-demic School

Construction Project (Approval No. LWH [2020] No. 132, LWH [2012] No.

45) ; 2020 Shandong Tra-ditional Chinese Medicine Science and Technology

Project (Approval No. LWH [2021] No. 46, Project No. N4) ; 2020 Shandong

Traditional Chinese Medicine Science and Technology Project (Project No.

2020M063) ; Natural Sci-ence Foundation of Shandong Province in 2021

(Project No. ZR2021QH242) ; 2020 Shandong Medical and Health Science and

Technology Development Plan Project (Project No. 202002080697) .

CR Aldape K, 2019, NAT REV CLIN ONCOL, V16, P509, DOI 10.1038/s41571-019-0177-5

Bersuker K, 2019, NATURE, V575, P688, DOI 10.1038/s41586-019-1705-2

Brennan CW, 2013, CELL, V155, P462, DOI 10.1016/j.cell.2013.09.034

Bu LG, 2022, THERIOGENOLOGY, V180, P130, DOI 10.1016/j.theriogenology.2021.12.022

Desert R, 2023, HEPATOLOGY, V78, P741, DOI 10.1097/HEP.0000000000000362

Dixit D, 2021, CANCER DISCOV, V11, P480, DOI 10.1158/2159-8290.CD-20-0331

Feng L, 2022, ANTI-CANCER DRUG, V33, pE155, DOI 10.1097/CAD.0000000000001167

Galvin Amy, 2019, Curr Protoc Cytom, V87, pe50, DOI 10.1002/cpcy.50

Gold LI, 1999, CRIT REV ONCOGENESIS, V10, P303

Guerrier-Takada C, 2002, RNA, V8, P290, DOI 10.1017/S1355838202027954

Hangauer MJ, 2017, NATURE, V551, P247, DOI 10.1038/nature24297

Hassannia B, 2019, CANCER CELL, V35, P830, DOI 10.1016/j.ccell.2019.04.002

Jiang XJ, 2021, NAT REV MOL CELL BIO, V22, P266, DOI 10.1038/s41580-020-00324-8

Kaminska B, 2020, ADV EXP MED BIOL, V1202, P179, DOI 10.1007/978-3-030-30651-9_9

Kim SY, 2019, CELL MOL LIFE SCI, V76, P653, DOI 10.1007/s00018-018-2949-y

Kumar V, 2015, BIOL REPROD, V92, DOI 10.1095/biolreprod.114.122838

Lei G, 2022, NAT REV CANCER, V22, P381, DOI 10.1038/s41568-022-00459-0

Li SB, 2021, OXID MED CELL LONGEV, V2021, DOI 10.1155/2021/2915019

Liu XH, 2019, J EXP CLIN CANC RES, V38, DOI 10.1186/s13046-019-1173-4

Louis DN, 2016, ACTA NEUROPATHOL, V131, P803, DOI 10.1007/s00401-016-1545-1

Mahler M, 2013, ARTHRITIS RES THER, V15, DOI 10.1186/ar4210

Qiu S, 2023, Chinese Medical Journal

Stupp R, 2005, NEW ENGL J MED, V352, P987, DOI 10.1056/NEJMoa043330

Tan AC, 2020, CA-CANCER J CLIN, V70, P299, DOI 10.3322/caac.21613

Tu YM, 2019, J CELL MOL MED, V23, P6907, DOI 10.1111/jcmm.14574

Ursini F, 2020, FREE RADICAL BIO MED, V152, P175, DOI 10.1016/j.freeradbiomed.2020.02.027

Viswanathan VS, 2017, NATURE, V547, P453, DOI 10.1038/nature23007

Wan RJ, 2021, CNS NEUROSCI THER, V27, P973, DOI 10.1111/cns.13654

Wei CM, 2020, NEOPLASMA, V67, P802, DOI 10.4149/neo_2020_190723N657

Wu J, 2019, NATURE, V572, P402, DOI 10.1038/s41586-019-1426-6

Wu PC, 2019, PEERJ, V7, DOI 10.7717/peerj.8299

Xiao DD, 2022, FRONT ONCOL, V11, DOI 10.3389/fonc.2021.714904

Xie BM, 2021, CELL DEATH DISCOV, V7, DOI 10.1038/s41420-021-00483-3

Xu ZG, 2012, ANAT REC, V295, P1446, DOI 10.1002/ar.22521

Yang J, 2020, IN VITRO CELL DEV-AN, V56, P145, DOI 10.1007/s11626-019-00425-5

Zhang YZ, 2020, FRONT CELL DEV BIOL, V8, DOI 10.3389/fcell.2020.567682

Zou WH, 2021, OPEN LIFE SCI, V16, P442, DOI 10.1515/biol-2021-0048

Zou YL, 2020, NATURE, V585, P603, DOI 10.1038/s41586-020-2732-8

NR 38

TC 0

Z9 0

U1 4

U2 6

PU BIOLIFE SAS

PI SILVA MARINA (TE)

PA VIA S STEFANO 39 BIS, 64029 SILVA MARINA (TE), ITALY

SN 0393-974X

EI 1724-6083

J9 J BIOL REG HOMEOS AG

JI J. Biol. Regul. Homeost. Agents

PD DEC

PY 2023

VL 37

IS 12

BP 7119

EP 7131

DI 10.23812/j.biol.regul.homeost.agents.20233712.670

PG 13

WC Endocrinology & Metabolism; Immunology; Medicine, Research &

Experimental; Physiology

WE Science Citation Index Expanded (SCI-EXPANDED)

SC Endocrinology & Metabolism; Immunology; Research & Experimental

Medicine; Physiology

GA DX3B8

UT WOS:001135332800001

DA 2025-04-09

ER

PT J

AU Almeida, MB

Costa-Malaquias, A

Nascimento, JLM

Oliveira, KR

Herculano, AM

Crespo-López, ME

AF Almeida, M. B.

Costa-Malaquias, A.

Nascimento, J. L. M.

Oliveira, K. R.

Herculano, A. M.

Crespo-Lopez, M. E.

TI Therapeutic concentration of morphine reduces oxidative stress in glioma

cell line

SO BRAZILIAN JOURNAL OF MEDICAL AND BIOLOGICAL RESEARCH

LA English

DT Article

DE Morphine; Glia; Oxidative stress; Hydrogen peroxide; Lipid peroxidation;

Opiod

ID INDUCED DEATH; NEUROTOXICITY; MICROGLIA; BRAIN

AB Morphine is a potent analgesic opioid used extensively for pain treatment. During the last decade, global consumption grew more than 4-fold. However, molecular mechanisms elicited by morphine are not totally understood. Thus, a growing literature indicates that there are additional actions to the analgesic effect. Previous studies about morphine and oxidative stress are controversial and used concentrations outside the range of clinical practice. Therefore, in this study, we hypothesized that a therapeutic concentration of morphine (1 mM) would show a protective effect in a traditional model of oxidative stress. We exposed the C6 glioma cell line to hydrogen peroxide (H2O2) and/or morphine for 24 h and evaluated cell viability, lipid peroxidation, and levels of sulfhydryl groups (an indicator of the redox state of the cell). Morphine did not prevent the decrease in cell viability provoked by H2O2 but partially prevented lipid peroxidation caused by 0.0025% H2O2 (a concentration allowing more than 90% cell viability). Interestingly, this opioid did not alter the increased levels of sulfhydryl groups produced by exposure to 0.0025% H2O2, opening the possibility that alternative molecular mechanisms (a direct scavenging activity or the inhibition of NAPDH oxidase) may explain the protective effect registered in the lipid peroxidation assay. Our results demonstrate, for the first time, that morphine in usual analgesic doses may contribute to minimizing oxidative stress in cells of glial origin. This study supports the importance of employing concentrations similar to those used in clinical practice for a better approximation between experimental models and the clinical setting.

C1 [Almeida, M. B.; Costa-Malaquias, A.; Crespo-Lopez, M. E.] Fed Univ Para, Inst Ciencias Biol, Lab Farmacol Mol, BR-66059 Belem, Para, Brazil.

[Nascimento, J. L. M.] Fed Univ Para, Inst Ciencias Biol, Lab Neuroquim Mol & Celular, BR-66059 Belem, Para, Brazil.

[Oliveira, K. R.; Herculano, A. M.] Fed Univ Para, Inst Ciencias Biol, Neuroendocrinol Lab, BR-66059 Belem, Para, Brazil.

C3 Universidade Federal do Para; Universidade Federal do Para; Universidade

Federal do Para

RP Crespo-López, ME (corresponding author), UFPA, Inst Ciencias Biol, Lab Farmacol Mol, Rua Augusto Correa 1, BR-66075110 Belem, Para, Brazil.

RI Oliveira, Karen/LGZ-4927-2024; Herculano, Anderson/M-9016-2017;

Crespo-Lopez, Maria/X-7912-2018

OI Crespo-Lopez, Maria Elena/0000-0002-1335-6853; do Nascimento,

jose/0000-0003-3647-9124; Herculano, Anderson/0000-0003-4022-8096

FU CNPq [303110/2010-4, 478580/2012-6]; FAPESPA; Pro-Reitoria de

Pesquisa/Universidade Federal do Para (PROPESP/UFPA)

FX The authors thank Cristalia (Brazil) for the kind donation of morphine.

Research supported by CNPq (#303110/2010-4 and #478580/2012-6). A.

Costa-Malaquias thanks FAPESPA for his grant. J.L.M. Nascimento, A. M.

Herculano, and M. E. Crespo-Lopez thank CNPq for their grants. The

authors also thank Pro-Reitoria de Pesquisa/Universidade Federal do Para

(PROPESP/UFPA) for support.

CR Abdel-Zaher AO, 2013, BEHAV BRAIN RES, V247, P17, DOI 10.1016/j.bbr.2013.02.034

BIRD RP, 1984, METHOD ENZYMOL, V105, P299

Bohn LM, 1998, J NEUROCHEM, V70, P1819

BRADFORD MM, 1976, ANAL BIOCHEM, V72, P248, DOI 10.1016/0003-2697(76)90527-3

ELLMAN GL, 1959, ARCH BIOCHEM BIOPHYS, V82, P70, DOI 10.1016/0003-9861(59)90090-6

Feng Y, 2008, LIFE SCI, V82, P752, DOI 10.1016/j.lfs.2008.01.004

Gülçin I, 2004, PHARMACOL RES, V49, P59, DOI 10.1016/j.phrs.2003.07.012

Guzmán DC, 2006, NEUROCHEM RES, V31, P549, DOI 10.1007/s11064-006-9053-7

Hu SX, 2002, NEUROPHARMACOLOGY, V42, P829, DOI 10.1016/S0028-3908(02)00030-8

International Narcotics Control Board (INCB), 2012, REP 2012 EST WORLD R

Kanesaki T, 1999, EUR J PHARMACOL, V372, P319, DOI 10.1016/S0014-2999(99)00206-X

Laux-Biehlmann A, 2013, NEUROSCIENCE, V233, P95, DOI 10.1016/j.neuroscience.2012.12.013

Lee J, 2004, IMMUNOPHARM IMMUNOT, V26, P17, DOI 10.1081/IPH-120029941

MOSMANN T, 1983, J IMMUNOL METHODS, V65, P55, DOI 10.1016/0022-1759(83)90303-4

NEUMANN PB, 1982, PAIN, V13, P247, DOI 10.1016/0304-3959(82)90014-8

Özmen I, 2007, NEUROCHEM RES, V32, P19, DOI 10.1007/s11064-006-9217-5

Qian L, 2007, J IMMUNOL, V179, P1198, DOI 10.4049/jimmunol.179.2.1198

Rozisky JR, 2013, NEUROCHEM RES, V38, P494, DOI 10.1007/s11064-012-0941-8

Sen CK, 1997, J NUTR BIOCHEM, V8, P660, DOI 10.1016/S0955-2863(97)00113-7

Zhou JQ, 2011, NEUROTOX RES, V20, P334, DOI 10.1007/s12640-011-9247-x

NR 20

TC 22

Z9 22

U1 0

U2 13

PU ASSOC BRAS DIVULG CIENTIFICA

PI RIBEIRAO PRETO

PA FACULDADE MEDICINA, CASA 10, 14049 RIBEIRAO PRETO, RIBEIRAO PRETO, SP

14049, BRAZIL

SN 0100-879X

EI 1414-431X

J9 BRAZ J MED BIOL RES

JI Brazilian J. Med. Biol. Res.

PD FEB

PY 2014

VL 47

IS 5

BP 398

EP 402

DI 10.1590/1414-431X20143697

PG 5

WC Biology; Medicine, Research & Experimental

WE Science Citation Index Expanded (SCI-EXPANDED)

SC Life Sciences & Biomedicine - Other Topics; Research & Experimental

Medicine

GA AH3QO

UT WOS:000336040100007

PM 24728211

OA Green Published, gold

DA 2025-04-09

ER

PT J

AU Wu, H

Yuan, YH

Kang, SX

Zhou, GX

Gu, Y

Yuan, XY

Li, JJ

Gu, N

AF Wu, Hao

Yuan, Yuehui

Kang, Shixiong

Zhou, Gaoxin

Gu, Yue

Yuan, Xingyi

Li, Jiajie

Gu, Ning

TI Autophagy inhibitor-loaded mesoporous AgNPs@SiO2 nanoplatform

for synergistically enhanced glioma radiotherapy

SO SCIENCE CHINA-MATERIALS

LA English

DT Article

DE glioma; radiotherapy; autophagy; mesoporous silica; silver nanoparticles

ID SILVER NANOPARTICLES; SILICA NANOPARTICLES; OXIDATIVE STRESS;

CHEMORESISTANCE; NANOSILVER; PATHWAY; PROTEIN; CELLS; MODEL

AB Our previous studies demonstrated that silver nanoparticles (AgNPs) could be used as a potential radiosensitizer for glioma radiotherapy enhancement, which, however, is restricted by autophagy elicitation. As one of the most promising drug-delivery carriers, mesoporous silica nanospheres (MSNs) have made great contributions to the developments of biomedicine due to their excellent drug loading performance, inherent biocompatibility, and tunable pore size. Herein, we designed autophagy inhibitor (3-methyladenine)-loaded AgNPs-cored MSNs, which exhibited excellent synergistic anticancer efficacy in vitro and in vivo. Besides, it was also confirmed that by inhibition of autophagy, the outcome of radiotherapy can be further enhanced. Moreover, we also explored the enhancing mechanisms from the perspective of the nuclear transcription factor Nrf2, including autophagy inhibition-related enhancement of radiation induced oxidative stress injury and the interaction of Nrf2 with autophagy. This study provided a vision of utilizing AgNPs as a potential radiosensitizer in combination with autophagy inhibitor and proposed a feasible strategy for its translation into the clinic.

C1 [Wu, Hao; Yuan, Yuehui; Kang, Shixiong; Zhou, Gaoxin; Gu, Yue; Yuan, Xingyi; Li, Jiajie; Gu, Ning] Nanjing Med Univ, Sch Biomed Engn & Informat, Nanjing 211166, Peoples R China.

[Gu, Ning] Nanjing Univ, Med Sch, Nanjing 210093, Peoples R China.

C3 Nanjing Medical University; Nanjing University

RP Gu, N (corresponding author), Nanjing Med Univ, Sch Biomed Engn & Informat, Nanjing 211166, Peoples R China.; Gu, N (corresponding author), Nanjing Univ, Med Sch, Nanjing 210093, Peoples R China.

EM guning@seu.edu.cn

FU National Key Research and Development Program of China [2019YFA0210103,

2017YFA0104302]; National Natural Science Foundation of China [81901873,

51832001, 81971701]; National Key Laboratory of Science and Technology

on Strong Electromagnetic Environment Simulation and Protection

[6142205190402]; Natural Science Foundation of Jiangsu Province

[BK20201352]; Program of Jiangsu Specially Appointed Professor

FX We highly appreciate financial supports from the National Key Research

and Development Program of China (2019YFA0210103 and 2017YFA0104302),

the National Natural Science Foundation of China (81901873, 51832001,

and 81971701), the National Key Laboratory of Science and Technology on

Strong Electromagnetic Environment Simulation and Protection

(6142205190402), the Natural Science Foundation of Jiangsu Province

(BK20201352), and the Program of Jiangsu Specially Appointed Professor.

CR Björkblom B, 2022, NEURO-ONCOLOGY, V24, P1454, DOI 10.1093/neuonc/noac042

Bourdenx M, 2021, CELL, V184, P2696, DOI 10.1016/j.cell.2021.03.048

Chaloupka K, 2010, TRENDS BIOTECHNOL, V28, P580, DOI 10.1016/j.tibtech.2010.07.006

Che S, 2004, NATURE, V429, P281, DOI 10.1038/nature02529

Chen Q, 2021, SCI CHINA MATER, V64, P510, DOI 10.1007/s40843-020-1431-5

Chen X, 2008, TOXICOL LETT, V176, P1, DOI 10.1016/j.toxlet.2007.10.004

Chen XY, 2018, NAT COMMUN, V9, DOI 10.1038/s41467-018-05373-4

Clement S, 2020, ADV SCI, V7, DOI 10.1002/advs.202003584

de la Vega MR, 2018, CANCER CELL, V34, P21, DOI 10.1016/j.ccell.2018.03.022

El Yamani N, 2022, NANO TODAY, V46, DOI 10.1016/j.nantod.2022.101581

Fleischmann DF, 2021, RADIAT ONCOL, V16, DOI 10.1186/s13014-021-01886-3

Goutas A, 2018, FREE RADICAL BIO MED, V126, P122, DOI 10.1016/j.freeradbiomed.2018.08.003

Griveau A, 2018, CANCER CELL, V33, P874, DOI 10.1016/j.ccell.2018.03.020

Guan L, 2016, HEPATOLOGY, V63, P1914, DOI 10.1002/hep.28496

Han L, 2011, CHEM COMMUN, V47, P8536, DOI 10.1039/c1cc12718g

Hsin YH, 2008, TOXICOL LETT, V179, P130, DOI 10.1016/j.toxlet.2008.04.015

Huang SS, 2020, SCI CHINA MATER, V63, P864, DOI 10.1007/s40843-019-1235-1

Ichimura Y, 2013, MOL CELL, V51, P618, DOI 10.1016/j.molcel.2013.08.003

Jing Manman, 2021, Small, V17, pe2105995, DOI [10.1002/smll.202102295, 10.1002/smll.202105995]

Levy JMM, 2017, NAT REV CANCER, V17, P528, DOI 10.1038/nrc.2017.53

Li HY, 2020, SIGNAL TRANSDUCT TAR, V5, DOI 10.1038/s41392-019-0089-y

Li YN, 2017, SCI CHINA MATER, V60, P543, DOI 10.1007/s40843-016-5151-6

Li ZX, 2012, CHEM SOC REV, V41, P2590, DOI 10.1039/c1cs15246g

Lin J, 2018, SMALL, V14, DOI 10.1002/smll.201703711

Lin J, 2014, AUTOPHAGY, V10, P2006, DOI 10.4161/auto.36293

Liu C, 2021, ADV MATER, V33, DOI 10.1002/adma.202102054

Liu PD, 2016, INT J NANOMED, V11, P5003, DOI 10.2147/IJN.S115473

Liu PD, 2013, NANOSCALE, V5, P11829, DOI 10.1039/c3nr01351k

Liu ZJ, 2018, ARTIF CELL NANOMED B, V46, pS922, DOI 10.1080/21691401.2018.1518912

Lu J, 2021, SCI CHINA MATER, V64, P769, DOI 10.1007/s40843-020-1445-x

Lv HM, 2019, CELL DEATH DIS, V10, DOI 10.1038/s41419-019-1543-z

Manzano M, 2020, ADV FUNCT MATER, V30, DOI 10.1002/adfm.201902634

Maremonti E, 2020, FREE RADICAL BIO MED, V152, P583, DOI 10.1016/j.freeradbiomed.2019.11.037

Mathew R, 2007, NAT REV CANCER, V7, P961, DOI 10.1038/nrc2254

McDonald JT, 2010, CANCER RES, V70, P8886, DOI 10.1158/0008-5472.CAN-10-0171

Mukha A, 2021, AUTOPHAGY, V17, P3879, DOI 10.1080/15548627.2021.1962682

Ostrom QT, 2018, JAMA ONCOL, V4, P1254, DOI 10.1001/jamaoncol.2018.1789

Pan LM, 2012, J AM CHEM SOC, V134, P5722, DOI 10.1021/ja211035w

Pisoschi AM, 2021, EUR J MED CHEM, V209, DOI 10.1016/j.ejmech.2020.112891

Rubinsztein DC, 2011, CELL, V146, P682, DOI 10.1016/j.cell.2011.07.030

Shi JJ, 2021, SMALL, V17, DOI 10.1002/smll.202104722

Singh I, 2015, CELL RES, V25, P837, DOI 10.1038/cr.2015.67

Soumya R.S., 2013, Der. Pharmacia. Lett, V5, P189

Sun X, 2017, J APPL TOXICOL, V37, P1428, DOI 10.1002/jat.3511

Tao SS, 2019, ENVIRON TOXICOL, V34, P594, DOI 10.1002/tox.22726

Tao S, 2014, CANCER RES, V74, P7430, DOI 10.1158/0008-5472.CAN-14-1439

Tarn D, 2013, ACCOUNTS CHEM RES, V46, P792, DOI 10.1021/ar3000986

Thomassen LCJ, 2010, LANGMUIR, V26, P328, DOI 10.1021/la902050k

Tonelli C, 2018, ANTIOXID REDOX SIGN, V29, P1727, DOI 10.1089/ars.2017.7342

Toulany M, 2019, GENES-BASEL, V10, DOI 10.3390/genes10010025

Towers CG, 2019, DEV CELL, V50, P690, DOI 10.1016/j.devcel.2019.07.010

Vera-Ramirez L, 2018, NAT COMMUN, V9, DOI 10.1038/s41467-018-04070-6

Walker A, 2018, FREE RADICAL BIO MED, V120, P407, DOI 10.1016/j.freeradbiomed.2018.04.009

Wan XJ, 2013, MACROMOL RAPID COMM, V34, P341, DOI 10.1002/marc.201200673

Wang GY, 2017, SCI CHINA MATER, V60, P995, DOI 10.1007/s40843-017-9107-x

Wang XW, 2021, CHEM SOC REV, V50, P8669, DOI 10.1039/d0cs00461h

Wang Y, 2016, BIOMATERIALS, V101, P207, DOI 10.1016/j.biomaterials.2016.06.004

Wang Z, 2022, CHEM-EUR J, V28

Wu H, 2016, BIOMATERIALS, V101, P1, DOI 10.1016/j.biomaterials.2016.05.031

Wu H, 2015, BIOMATERIALS, V62, P47, DOI 10.1016/j.biomaterials.2015.05.033

Wu T, 2011, CHEM MATER, V23, P2370, DOI 10.1021/cm200102g

Xia HJ, 2021, NAT REV CANCER, V21, P281, DOI 10.1038/s41568-021-00344-2

Yang X, 2020, NANO RES, V13, P2579, DOI 10.1007/s12274-020-2722-z

Yang Y, 2017, SCI CHINA MATER, V60, P892, DOI 10.1007/s40843-017-9088-9

Zhang YZ, 2010, J CONTROL RELEASE, V145, P257, DOI 10.1016/j.jconrel.2010.04.029

Zhao J, 2019, INT J NANOMED, V14, P9483, DOI 10.2147/IJN.S224160

Zheng NF, 2018, SCI CHINA MATER, V61, P1129, DOI 10.1007/s40843-018-9213-4

Zhu LY, 2017, NANOSCALE, V9, P5489, DOI 10.1039/c6nr08188f

NR 68

TC 3

Z9 4

U1 5

U2 32

PU SCIENCE PRESS

PI BEIJING

PA 16 DONGHUANGCHENGGEN NORTH ST, BEIJING 100717, PEOPLES R CHINA

SN 2095-8226

EI 2199-4501

J9 SCI CHINA MATER

JI Sci. China-Mater.

PD JUL

PY 2023

VL 66

IS 7

BP 2902

EP 2912

DI 10.1007/s40843-022-2395-y

EA APR 2023

PG 11

WC Materials Science, Multidisciplinary

WE Science Citation Index Expanded (SCI-EXPANDED)

SC Materials Science

GA M0GN1

UT WOS:000975178100001

OA Bronze

DA 2025-04-09

ER

PT J

AU Mohammadzadeh, Z

Khaksari, M

Nematollahi, MH

Kheirandish, R

Moslemizadeh, A

Delshad, S

Faramarz, S

Tezerji, SS

Torkashvand, M

Shahba, S

Bashiri, H

AF Mohammadzadeh, Zahra

Khaksari, Mohammad

Nematollahi, Mohammad Hadi

Kheirandish, Reza

Moslemizadeh, Amirhossein

Delshad, Sina

Faramarz, Sanaz

Tezerji, Sara Sheibani

Torkashvand, Mohammad

Shahba, Samira

Bashiri, Hamideh

TI Therapeutic efficacy to dose-dependent toxicity of Cabazitaxel in

C6-induced glioblastoma model of rats

SO TOXICOLOGY RESEARCH

LA English

DT Article

DE glioblastoma; Temozolomide (TMZ); Cabazitaxel (CBZ); cognitive behavior;

inflammatory factors; oxidative stress

ID TOTAL ANTIOXIDANT CAPACITY; RESISTANT PROSTATE-CANCER; DNA-DAMAGE;

GLIOMA; TEMOZOLOMIDE; DOCETAXEL; ADULT; ALPHA

AB This study was designed to adjust effective chemotherapy doses of cabazitaxel (CBZ) on cognitive behaviors, inflammatory cytokines and oxidative stress parameters, and survival rate in C6-induced GBM of rats. Male Sprague-Dawley rats bearing intra-caudate nucleus (CN) C6 inoculation were randomly divided into nine groups as follows: sham, tumor, Temozolomide (TMZ) vehicle, TMZ, CBZ vehicle, CBZ at doses of 0.5, 1, 2 and 4 mg/kg. Behavioral tests survival rate, histopathology, immunohistochemistry, oxidative stress, and inflammatory cytokines were evaluated. All drug treatments reduced the volume and number of tumor cells dose-dependently and CBZ4 was able to cause the greatest reduction. The %Survival rate of animals using CBZ1 significantly increased compared to other treatment groups. CBZ1 reduced anxiety-like behaviors and increased the balance of the animal with GBM. CBZ1 and CBZ2 groups improved C6-induced learning disabilities. Treatments could ameliorate tumor-induced dysregulation of oxidative stress. TNF-alpha/IL-10 decreased in the CBZ1 group compared to other treatment groups, which may indicate an improvement in inflammatory balance. Our findings demonstrate that the administration of CBZ at a dosage of 1 mg/kg exerts advantageous impacts on both the survival rate and neurocognitive performance of rats within the GBM model. However, our results showed that CBZ may have toxic effects, especially in a dose of 4 mg/kg.

C1 [Mohammadzadeh, Zahra] Kerman Univ Med Sci, Afzalipour Sch Med, Dept Physiol & Pharmacol, Haft Bagh Highway,POB 76169 13555, Kerman, Iran.

[Khaksari, Mohammad] Kerman Univ Med Sci, Inst Basic & Clin Physiol Sci, Endocrinol & Metab Res Ctr, Haft Bagh Highway,POB 76169 13555, Kerman, Iran.

[Nematollahi, Mohammad Hadi] Kerman Univ Med Sci, Physiol Res Ctr, Afzalipour Sch Med, Haft Bagh Highway,POB 76169 13555, Kerman, Iran.

[Kheirandish, Reza; Delshad, Sina] Shahid Bahonar Univ Kerman, Fac Vet Med, Dept Pathobiol, Imam Khomeni Highway,POB 76169 13439, Kerman, Iran.

[Moslemizadeh, Amirhossein] Univ Tehran Med Sci, Dept Immunol, Dameshgh St,Vali e Asr Ave,POB 14167 53955, Tehran, Iran.

[Faramarz, Sanaz] Kerman Univ Med Sci, Dept Biochem, Haft Bagh Highway,POB 76169 13555, Kerman, Iran.

[Tezerji, Sara Sheibani] Univ Regensburg, Regensburg Ctr Neurosci, Dept Behav & Mol Neurobiol, Univ Str 31, D-93053 Regensburg, Germany.

[Torkashvand, Mohammad] Univ Tehran, Coll Engn, Dameshgh St,Vali e Asr Ave,POB 14167 53955, Tehran, Iran.

[Shahba, Samira] Semnan Univ Med Sci, Sch Med, Dept Biotechnol, Bassij Blvd,POB 35147-99442, Semnan, Iran.

[Bashiri, Hamideh] Kerman Univ Med Sci, Inst Neuropharmacol, Neurosci Res Ctr, Haft Bagh Highway,POB 76169 13555, Kerman, Iran.

C3 Kerman University of Medical Sciences; Kerman University of Medical

Sciences; Kerman University of Medical Sciences; Shahid Bahonar

University of Kerman (SBUK); Tehran University of Medical Sciences;

Kerman University of Medical Sciences; University of Regensburg;

University of Tehran; Semnan University of Medical Sciences; Kerman

University of Medical Sciences

RP Bashiri, H (corresponding author), Kerman Univ Med Sci, Inst Neuropharmacol, Neurosci Res Ctr, Afzalipour Sch Med,Dept Physiol & Pharmacol, POB 76169 13555, Kerman, Iran.

EM h.bashiri@kmu.ac.ir

FU Neuroscience Research Center of Kerman University of Medical Sciences;

Kerman Neuroscience Research Center

FX The authors would like to acknowledge the Kerman Neuroscience Research

Center for their support of the study-also, many thanks to 2 anonymous

reviewers whose insightful comments greatly improved the manuscript.

CR Aggarwal Sarita, 2006, J Cancer Res Ther, V2, P24

Albulescu R, 2013, MEDIAT INFLAMM, V2013, DOI 10.1155/2013/979748

[Anonymous], Oncol Rep, V15, DOI [DOI 10.3892/OR.2011.1198, 10.3892/or.15.1.7, DOI 10.3892/OR.15.1.7]

Bashiri H, 2023, IMMUNOTHERAPY-UK, V15, DOI 10.2217/imt-2022-0212

Bteich J, 2018, BIOCONJUGATE CHEM, V29, P2009, DOI 10.1021/acs.bioconjchem.8b00220

Cevik O, 2020, J BIOCHEM MOL TOXIC, V34, DOI 10.1002/jbt.22542

Cicero G, 2017, ONCOLOGY-BASEL, V92, P94, DOI 10.1159/000452491

Dietrich Jorg, 2020, Continuum (Minneap Minn), V26, P1646, DOI 10.1212/CON.0000000000000943

Duran GE, 2018, CANCER CHEMOTH PHARM, V81, P1095, DOI 10.1007/s00280-018-3572-1

Egeland M, 2017, TRANSL PSYCHIAT, V7, DOI 10.1038/tp.2017.68

Frontiñán-Rubio J, 2018, RADIOTHER ONCOL, V128, P236, DOI 10.1016/j.radonc.2018.04.033

Ghoochani A, 2016, ONCOTARGET, V7, P38306, DOI 10.18632/oncotarget.9439

Grochans S, 2022, CANCERS, V14, DOI 10.3390/cancers14102412

Hanimoglu H, 2007, CLIN NEUROL NEUROSUR, V109, P561, DOI 10.1016/j.clineuro.2007.04.007

Jacob J, 2018, RADIOTHER ONCOL, V128, P221, DOI 10.1016/j.radonc.2018.05.027

Jiang T, 2016, CANCER LETT, V375, P263, DOI 10.1016/j.canlet.2016.01.024

Joerger M, 2016, CANCER CHEMOTH PHARM, V77, P221, DOI 10.1007/s00280-015-2893-6

Johannessen AL, 2006, PATHOL ONCOL RES, V12, P143, DOI 10.1007/BF02893360

Karavelioglu E, 2016, J NEUROL SCI, V360, P66, DOI 10.1016/j.jns.2015.11.033

Kosaka T, 2017, ONCOTARGET, V8, P87675, DOI 10.18632/oncotarget.21147

Li J, 2021, J NANOBIOTECHNOL, V19, DOI 10.1186/s12951-021-01048-3

Li JY, 2020, BRAIN BEHAV IMMUN, V87, P645, DOI 10.1016/j.bbi.2020.02.009

Li XZ, 2017, INT J NEUROSCI, V127, P1005, DOI 10.1080/00207454.2017.1288624

Lo Dico A, 2019, CELLS-BASEL, V8, DOI 10.3390/cells8111315

McKinnon C, 2021, BMJ-BRIT MED J, V374, DOI 10.1136/bmj.n1560

Meymandi MS, 2020, INT J DEV NEUROSCI, V80, P500, DOI 10.1002/jdn.10046

Moore RJ, 1999, NAT MED, V5, P828, DOI 10.1038/10552

Moslemizadeh A, 2022, LIFE SCI, V305, DOI 10.1016/j.lfs.2022.120744

Mostofa AGM, 2017, BIOMOLECULES, V7, DOI 10.3390/biom7020034

Neshasteh-Riz A, 2018, CELL BIOL INT, V42, P815, DOI 10.1002/cbin.10940

Oudard S, 2017, J CLIN ONCOL, V35, P3189, DOI 10.1200/JCO.2016.72.1068

Pautler M, 2010, INT J NANOMED, V5, P803, DOI 10.2147/IJN.S13816

Pessina F, 2016, ANN SURG ONCOL, V23, P3040, DOI 10.1245/s10434-016-5222-3

Popivanova BK, 2008, J CLIN INVEST, V118, P560, DOI [10.1172/JC132453, 10.1172/JCI32453]

Popov B., 2003, Archives of Physiology and Biochemistry, V111, P455, DOI 10.3109/13813450312331342328

Pournajaf S, 2024, REV NEUROSCIENCE, V35, P183, DOI 10.1515/revneuro-2023-0067

Schagen SB, 2022, NAT REV NEUROL, V18, P173, DOI 10.1038/s41582-021-00617-2

Sémiond D, 2013, CANCER CHEMOTH PHARM, V72, P515, DOI 10.1007/s00280-013-2214-x

Shafaghi A, 2022, NEUROTOXICOL TERATOL, V89, DOI 10.1016/j.ntt.2021.107050

Shahrbabaki SSV, 2022, PHYSIOL BEHAV, V249, DOI 10.1016/j.physbeh.2022.113739

Sharifzad F, 2019, J CELL PHYSIOL, V234, P22493, DOI 10.1002/jcp.28813

Sharma P, 2018, INT J MOL SCI, V19, DOI 10.3390/ijms19113326

Siegel RL, 2023, CA-CANCER J CLIN, V73, P17, DOI 10.3322/caac.21763

Singer E, 2015, CELL DEATH DIS, V6, DOI 10.1038/cddis.2014.566

Son MJ, 2006, INT J ONCOL, V28, P53

Stupp R, 2017, JAMA-J AM MED ASSOC, V318, P2306, DOI 10.1001/jama.2017.18718

Sulheim Einar, 2019, Nanotheranostics, V3, P103, DOI 10.7150/ntno.31479

Sung H, 2021, CA-CANCER J CLIN, V71, P209, DOI 10.3322/caac.21660

Taillibert S, 2016, CURR NEUROL NEUROSCI, V16, DOI 10.1007/s11910-016-0686-x

Tisdale MJ., 2004, Arch Surg, V389, P299, DOI [10.1007/s00423-004-0486-7, DOI 10.1007/S00423-004-0486-7]

Trachootham D, 2009, NAT REV DRUG DISCOV, V8, P579, DOI 10.1038/nrd2803

Tuzgen S, 2007, CLIN ONCOL-UK, V19, P177, DOI 10.1016/j.clon.2006.11.012

Wu SY, 2022, J CONTROL RELEASE, V345, P685, DOI 10.1016/j.jconrel.2022.03.047

Yin XY, 2019, ASIAN J PHARM SCI, V14, P658, DOI 10.1016/j.ajps.2018.10.004

NR 54

TC 0

Z9 0

U1 0

U2 0

PU OXFORD UNIV PRESS

PI OXFORD

PA GREAT CLARENDON ST, OXFORD OX2 6DP, ENGLAND

SN 2045-452X

EI 2045-4538

J9 TOXICOL RES-UK

JI Toxicol. Res.

PD APR

PY 2025

VL 14

IS 2

AR tfaf048

DI 10.1093/toxres/tfaf048

PG 15

WC Toxicology

WE Science Citation Index Expanded (SCI-EXPANDED)

SC Toxicology

GA 0VS3R

UT WOS:001457153000001

PM 40182916

DA 2025-04-09

ER

PT J

AU Qin, BL

Panickar, KS

Anderson, RA

AF Qin, Bolin

Panickar, Kiran S.

Anderson, Richard A.

TI Cinnamon polyphenols attenuate the hydrogen peroxide-induced down

regulation of S100β secretion by regulating sirtuin 1 in C6 rat glioma

cells

SO LIFE SCIENCES

LA English

DT Article

DE Polyphenols; S100 beta; SIRT1; Oxidative stress

ID NF-KAPPA-B; INSULIN-RESISTANCE; GLUTAMATE UPTAKE; S100B SECRETION;

EXTRACT; RESVERATROL; EXPRESSION; GLUCOSE; OVERPRODUCTION; INHIBITION

AB Aims: It is well established that the brain is particularly susceptible to oxidative damage due to its high consumption of oxygen. The objective of this study was to investigate the protective effects of a water soluble polyphenol-rich extract of cinnamon and the possible mechanisms, under conditions of oxidative stress-induced by hydrogen peroxide, in rat C6 glioma cells. Main methods: After 24 h of H2O2 incubation, the secretion and intracellular expression of S100 beta were determined by immunoprecitation/immunoblotting and immunofluorescence imaging. Key findings: Cinnamon polyphenols (CP) counteracted the oxidative effects of H2O2 on S100 beta secretion and expression. CP also enhanced the impaired protein levels of sirtuins I, 2, and 3, which are deacetylases important in cell survival. H2O2 also induced the overexpression of the proinflammatory factors, TNF-alpha, phospho-NF-kB p65, as well as of Bcl-xl, Bax and Caspase-3, which are all the members of the Bc1-2 family. CP not only suppressed the expression of these proteins but also attenuated the phosphorylation induced by H2O2. CP also upregulated the decreased Bc1-2 protein levels in H2O2 treated C6 cells. The effects of CP on H2O2-induced downregulation of S100 beta secretion were blocked by SIRTI siRNA demonstrating that SIRT1 plays a regulatory role in CP-mediated prevention by H2O2. Significance: These data demonstrate that Cinnamon polyphenols may exert neuroprotective effects in glial cells by the regulation of BcI-2 family members and enhancing SIRT1 expression during oxidative stress. (c) 2014 Elsevier Inc. All rights reserved.

C1 [Qin, Bolin] IN Ingredients Inc, Columbia, TN 38401 USA.

[Qin, Bolin; Panickar, Kiran S.; Anderson, Richard A.] USDA ARS, Beltsville Human Nutr Res Ctr, Diet Genom & Immunol Lab, Beltsville, MD 20705 USA.

[Panickar, Kiran S.] Univ Maryland, Sch Med, Dept Pediat, Baltimore, MD 21201 USA.

C3 United States Department of Agriculture (USDA); University System of

Maryland; University of Maryland Baltimore

RP Qin, BL (corresponding author), USDA ARS BHNRC DGIL, Bldg 307C,Rm 215,10300 Baltimore Ave, Beltsville, MD 20705 USA.

EM Bolin.Qin@ars.usda.gov

FU USDA Cooperative Research and Development Agreement [58-3k95-7-1184];

Integrity Nutraceuticals International, Spring Hill, TN.

FX This work was supported in part by a USDA Cooperative Research and

Development Agreement (CRADA No. 58-3k95-7-1184) with Integrity

Nutraceuticals International, Spring Hill, TN. BQ has a joint

appointment with the USDA and IN Ingredients Inc. (formerly Integrity

Nutraceuticals, Columbia, TN, USA). The authors would like to thank Dr.

Tom Wang for reviewing the manuscript. Mention of trade names or

commercial products is solely for the purpose of providing specific

information and does not imply recommendation or endorsement by the U.S.

Department of Agriculture. The USDA is an equal opportunity provider and

employer.

CR Abib RT, 2010, J MED FOOD, V13, P1111, DOI 10.1089/jmf.2009.0255

Anderson RA, 2004, J AGR FOOD CHEM, V52, P65, DOI 10.1021/jf034916b

Baeuerle PA, 1996, CELL, V87, P13, DOI 10.1016/S0092-8674(00)81318-5

Bhardwaj A, 2007, BLOOD, V109, P2293, DOI 10.1182/blood-2006-02-003988

Broadhurst CL, 2000, J AGR FOOD CHEM, V48, P849, DOI 10.1021/jf9904517

Byun YJ, 2009, NEUROSCI LETT, V461, P131, DOI 10.1016/j.neulet.2009.06.011

Cai DS, 2012, AGING-US, V4, P98, DOI 10.18632/aging.100431

Cao H, 2008, J NUTR, V138, P833, DOI 10.1093/jn/138.5.833

Donato R, 2001, INT J BIOCHEM CELL B, V33, P637, DOI 10.1016/S1357-2725(01)00046-2

Donato R, 2009, BBA-MOL CELL RES, V1793, P1008, DOI 10.1016/j.bbamcr.2008.11.009

Dringen R, 2005, J NEUROSCI RES, V79, P157, DOI 10.1002/jnr.20280

Edwards MM, 2006, J NEURAL TRANSM, V113, P1709, DOI 10.1007/s00702-006-0479-5

Furukawa A, 2007, CELL PHYSIOL BIOCHEM, V20, P45, DOI 10.1159/000104152

Guerra MC, 2011, J NEUROINFLAMM, V8, DOI 10.1186/1742-2094-8-128

Halliwell B, 2006, J NEUROCHEM, V97, P1634, DOI 10.1111/j.1471-4159.2006.03907.x

He X, 2012, BIOCHEM BIOPH RES CO, V417, P468, DOI 10.1016/j.bbrc.2011.11.141

HYSLOP PA, 1995, BRAIN RES, V671, P181, DOI 10.1016/0006-8993(94)01291-O

Killday KB, 2011, J NAT PROD, V74, P1833, DOI 10.1021/np1007944

Kim DH, 2011, AM J PHYSIOL-RENAL, V301, pF427, DOI 10.1152/ajprenal.00258.2010

Kim SH, 2011, PLOS ONE, V6, DOI [10.1371/journal.pone.0014731, 10.1371/journal.pone.0026214]

Kwon HK, 2010, BMC CANCER, V10, DOI 10.1186/1471-2407-10-392

Lee Jeong-Sun, 2003, Journal of Medicinal Food, V6, P183

Mccoy MK, 2008, J NEUROINFLAMM, V5, DOI 10.1186/1742-2094-5-45

MULLER CM, 1993, J CHEM NEUROANAT, V6, P215, DOI 10.1016/0891-0618(93)90043-4

Nishikawa T, 2006, J HEPATOL, V44, P1074, DOI 10.1016/j.jhep.2005.11.045

Pallas Merce, 2008, Recent Pat CNS Drug Discov, V3, P61, DOI 10.2174/157488908783421492

Panickar KS, 2012, NEUROSCIENCE, V202, P87, DOI 10.1016/j.neuroscience.2011.11.051

Panickar KS, 2009, EXP NEUROL, V216, P420, DOI 10.1016/j.expneurol.2008.12.024

Peterson DW, 2009, J ALZHEIMERS DIS, V17, P585, DOI 10.3233/JAD-2009-1083

Qin B, 2009, HORM METAB RES, V41, P516, DOI 10.1055/s-0029-1202813

Qin BL, 2007, DIABETES, V56, P450, DOI 10.2337/db06-0518

Qin BL, 2014, NUTRITION, V30, P210, DOI 10.1016/j.nut.2013.07.001

Qin Bolin, 2010, J Diabetes Sci Technol, V4, P685

Roussel AM, 2009, J AM COLL NUTR, V28, P16, DOI 10.1080/07315724.2009.10719756

Tamatani M, 1999, J BIOL CHEM, V274, P8531, DOI 10.1074/jbc.274.13.8531

Van Eldik LJ, 2003, RESTOR NEUROL NEUROS, V21, P97

de Almeida LMV, 2008, NEUROCHEM RES, V33, P8, DOI 10.1007/s11064-007-9399-5

de Almeida LMV, 2007, CELL MOL NEUROBIOL, V27, P661, DOI 10.1007/s10571-007-9152-2

Wang JG, 2007, FERTIL STERIL, V88, P240, DOI 10.1016/j.fertnstert.2006.11.082

Zhu J, 2008, BIOCHEM BIOPH RES CO, V369, P471, DOI 10.1016/j.bbrc.2008.02.034

Ziegenfuss TN, 2006, J INT SOC SPORT NUTR, V3, DOI 10.1186/1550-2783-3-2-45

NR 41

TC 13

Z9 17

U1 0

U2 24

PU PERGAMON-ELSEVIER SCIENCE LTD

PI OXFORD

PA THE BOULEVARD, LANGFORD LANE, KIDLINGTON, OXFORD OX5 1GB, ENGLAND

SN 0024-3205

EI 1879-0631

J9 LIFE SCI

JI Life Sci.

PD APR 25

PY 2014

VL 102

IS 1

BP 72

EP 79

DI 10.1016/j.lfs.2014.02.038

PG 8

WC Medicine, Research & Experimental; Pharmacology & Pharmacy

WE Science Citation Index Expanded (SCI-EXPANDED)

SC Research & Experimental Medicine; Pharmacology & Pharmacy

GA AG0GR

UT WOS:000335094400010

PM 24631135

DA 2025-04-09

ER

PT J

AU Gratton, R

Tricarico, PM

Celsi, F

Grovella, S

AF Gratton, Rossella

Tricarico, Paola Maura

Celsi, Fulvio

Grovella, Sergio

TI Prolonged treatment with mevalonolactone induces oxidative stress

response with reactive oxygen species production, mitochondrial

depolarization and inflammation in human glioblastoma U-87 MG cells

SO NEUROCHEMISTRY INTERNATIONAL

LA English

DT Article

DE Mevalonate kinase deficiency; Mevalonolactone; Human glioblastoma U-87

MG cells; Oxidative stress; Mitochondrial damage; Inflammation

ID MEVALONATE KINASE-DEFICIENCY; PERIODIC FEVER SYNDROME;

HYPERIMMUNOGLOBULINEMIA-D; ACIDURIA; PATHWAY; AUTOPHAGY; SPECTRUM; GENE

AB Mevalonate pathway impairment has been observed in diverse diseases, including Mevalonate Kinase Deficiency (MKD). MKD is a hereditary auto-inflammatory disorder, due to mutations at mevalonate kinase gene (MVK), encoding mevalonate kinase (MK) enzyme. To date, the most accredited MKD pathogenic hypothesis suggests that the typical MKD phenotypes might be due to a decreased isoprenoid production rather than to the excess and accumulation of mevalonic acid, as initially supported. Nevertheless, recent studies provide clear evidences that accumulating metabolites might be involved in MKD pathophysiology by exerting a toxic effect. Our work aims at describing the effects of accumulating mevalonolactone, mostly produced by a dehydration reaction due to mevalonic acid accumulation, using an in vitro cellular model mimicking the glial component of the central nervous system (human glioblastoma U-87 MG cells). In order to mimic its progressive increase, occurring during the disease, U-87 MG cells have been treated repeatedly with growing doses of mevalonolactone, followed by the assessment of oxidative stress response (evaluated by measuring SOD2 and HemeOX expression levels), ROS production, mitochondrial damage and inflammatory response (evaluated by measuring ILIB expression levels). Our results suggest that protracted treatments with mevalonolactone induce oxidative stress with augmented ROS production and mitochondria] damage accompanied by membrane depolarization. Furthermore, an increment in ILIB expression has been observed, thus correlating the accumulation of the metabolite with the development of a neuroinflammatory response.

Our experimental work suggests to reconsider the presence of a possible synergy between the two major MKD pathogenic hypotheses in attempt of unravelling the different pathogenic pathways responsible for the disease.

C1 [Gratton, Rossella; Tricarico, Paola Maura; Celsi, Fulvio; Grovella, Sergio] IRCCS Barba Garofolo, Inst Maternal & Child Hlth, Via Istria 65-1, I-34137 Trieste, Italy.

[Tricarico, Paola Maura; Grovella, Sergio] Univ Trieste, Trieste, Italy.

C3 University of Trieste

RP Gratton, R (corresponding author), IRCCS Barba Garofolo, Inst Maternal & Child Hlth, Via Istria 65-1, I-34137 Trieste, Italy.

EM rossella.gratton@gmail.com; tricaricopa@gmail.com;

fulvio.celsi@gmail.com; sergio.crovella@burlo.trieste.it

RI Celsi, Fulvio/H-5134-2013; tricarico, paola/AAB-8972-2019

OI Celsi, Fulvio/0000-0002-9760-8463; TRICARICO, PAOLA

MAURA/0000-0002-1954-4126; Gratton, Rossella/0000-0002-7592-2443

CR Bekkering S, 2018, CELL, V172, P135, DOI 10.1016/j.cell.2017.11.025

Cecatto C, 2017, NEUROCHEM INT, V108, P133, DOI 10.1016/j.neuint.2017.03.005

Curtin JF, 2002, J IMMUNOL METHODS, V265, P49, DOI 10.1016/S0022-1759(02)00070-4

DRENTH JPH, 1994, MEDICINE, V73, P133, DOI 10.1097/00005792-199405000-00002

Favier LA, 2016, APPL CLIN GENET, V9, DOI 10.2147/TACG.S93933

Frenkel J, 2002, ARTHRITIS RHEUM, V46, P2794, DOI 10.1002/art.10550

Haas D, 2006, ORPHANET J RARE DIS, V1, DOI 10.1186/1750-1172-1-13

HOFFMANN GF, 1993, PEDIATRICS, V91, P915

Houten SM, 2003, J BIOL CHEM, V278, P5736, DOI 10.1074/jbc.M206564200

Houten SM, 2002, HUM MOL GENET, V11, P3115, DOI 10.1093/hmg/11.25.3115

McDermott MF, 2001, NETH J MED, V59, P118, DOI 10.1016/S0300-2977(01)00149-8

Mezzavilla M, 2018, RHEUMATOL INT, V38, P121, DOI 10.1007/s00296-017-3890-3

Moura R, 2015, RHEUMATOL INT, V35, P657, DOI 10.1007/s00296-014-3115-y

Prasad C, 2012, MOL GENET METAB, V107, P756, DOI 10.1016/j.ymgme.2012.10.019

Tricarico PM, 2017, INT J BIOCHEM CELL B, V92, P26, DOI 10.1016/j.biocel.2017.09.007

Tricarico PM, 2017, CELL PHYSIOL BIOCHEM, V41, P1649, DOI 10.1159/000471235

Tricarico PM, 2015, INT J MOL SCI, V16, P16067, DOI 10.3390/ijms160716067

van der Burgh R, 2014, J BIOL CHEM, V289, P5000, DOI 10.1074/jbc.M113.536920

van der Burgh R, 2013, CLIN IMMUNOL, V147, P197, DOI 10.1016/j.clim.2012.09.011

VANDERMEER JWM, 1984, LANCET, V1, P1087

Zablocka A, 2015, CELL MOL NEUROBIOL, V35, P977, DOI 10.1007/s10571-015-0192-8

NR 21

TC 8

Z9 9

U1 0

U2 8

PU PERGAMON-ELSEVIER SCIENCE LTD

PI OXFORD

PA THE BOULEVARD, LANGFORD LANE, KIDLINGTON, OXFORD OX5 1GB, ENGLAND

SN 0197-0186

EI 1872-9754

J9 NEUROCHEM INT

JI Neurochem. Int.

PD NOV

PY 2018

VL 120

BP 233

EP 237

DI 10.1016/j.neuint.2018.05.003

PG 5

WC Biochemistry & Molecular Biology; Neurosciences

WE Science Citation Index Expanded (SCI-EXPANDED)

SC Biochemistry & Molecular Biology; Neurosciences & Neurology

GA GX5VR

UT WOS:000447819600024

PM 29753116

DA 2025-04-09

ER

PT J

AU Hari, AD

Vegi, NG

Das, UN

AF Hari, Anasuya Devi

Vegi, Naidu G.

Das, Undurti N.

TI Arachidonic and eicosapentaenoic acids induce oxidative stress to

suppress proliferation of human glioma cells

SO ARCHIVES OF MEDICAL SCIENCE

LA English

DT Letter

ID POLYUNSATURATED FATTY-ACIDS; COLON-CANCER CELLS; TUMORICIDAL ACTION;

LINOLEIC-ACID; APOPTOSIS; SENSITIVITY; ONCOGENES; N-6

C1 [Hari, Anasuya Devi] BioSci Res Ctr, GVP Coll Engn Campus, Visakhapatnam, Andhra Pradesh, India.

[Vegi, Naidu G.] NIPER, Hyderabad, Telangana, India.

[Das, Undurti N.] UND Life Sci, 2221 NW 5th St, Battle Ground, WA 98604 USA.

C3 Gayatri Vidya Parishad College of Engineering; National Institute of

Pharmaceutical Education & Research, Hyderabad; National Institute of

Pharmaceutical Education & Research, S.A.S. Nagar (Mohali)

RP Das, UN (corresponding author), UND Life Sci, 2221 NW 5th St, Battle Ground, WA 98604 USA.

EM undurti@hotmail.com

RI Das, Undurti/A-7918-2009

CR Abdi J, 2014, J NUTR BIOCHEM, V25, P1254, DOI 10.1016/j.jnutbio.2014.06.013

Arwert EN, 2010, P NATL ACAD SCI USA, V107, P19903, DOI 10.1073/pnas.1007404107

BEGIN ME, 1986, JNCI-J NATL CANCER I, V77, P1053

DAS UN, 1991, CANCER LETT, V56, P235, DOI 10.1016/0304-3835(91)90008-6

DAS UN, 1995, CANCER LETT, V94, P147, DOI 10.1016/0304-3835(95)03844-M

Das UN, 2018, J ADV RES, V11, P33

Das UN, 2007, MED SCI MONITOR, V13, pRA119

Das UN, 2018, J ADV RES, V11, P67, DOI 10.1016/j.jare.2018.02.004

Das UN, 2011, CLIN LIPIDOL, V6, P463, DOI 10.2217/CLP.11.34

Das UN, 2013, CLIN LIPIDOL, V8, P437, DOI 10.2217/CLP.13.31

Gao XS, 1995, ANTICANCER RES, V15, P1911

Guchelaar HJ, 1997, PHARM WORLD SCI, V19, P119, DOI 10.1023/A:1008654316572

Hamamoto T, 2005, J RADIAT RES, V46, P197, DOI 10.1269/jrr.46.197

Lu XF, 2010, J ZHEJIANG UNIV-SC B, V11, P923, DOI 10.1631/jzus.B1000125

Lu XF, 2010, LIPIDS HEALTH DIS, V9, DOI 10.1186/1476-511X-9-106

MADHAVI N, 1994, CANCER LETT, V84, P31, DOI 10.1016/0304-3835(94)90355-7

Mantovani A, 2009, NATURE, V457, P36, DOI 10.1038/457036b

McLean MH, 2011, PLOS ONE, V6, DOI 10.1371/journal.pone.0015366

NAIDU MRC, 1992, PROSTAG LEUKOTR ESS, V45, P181, DOI 10.1016/0952-3278(92)90110-5

Polavarapu S, 2018, PROSTAG LEUKOTR ESS, V132, P16, DOI 10.1016/j.plefa.2018.04.001

Polavarapu S, 2014, PLOS ONE, V9, DOI 10.1371/journal.pone.0114766

Reddy DR, 1998, J CLIN NEUROSCI, V5, P36, DOI 10.1016/S0967-5868(98)90199-0

Sandrone SS, 2014, NUTRITION, V30, P1104, DOI 10.1016/j.nut.2014.01.009

Shalini S, 2015, CELL DEATH DIFFER, V22, P526, DOI 10.1038/cdd.2014.216

Skender B, 2014, BBA-MOL CELL BIOL L, V1841, P1308, DOI 10.1016/j.bbalip.2014.06.005

Yu HN, 2015, ARCH MED SCI, V11, P282, DOI 10.5114/aoms.2015.50962

Zhang CC, 2015, ARCH MED SCI, V11, P1081, DOI 10.5114/aoms.2015.54865

NR 27

TC 8

Z9 9

U1 0

U2 10

PU TERMEDIA PUBLISHING HOUSE LTD

PI POZNAN

PA KLEEBERGA ST 2, POZNAN, 61-615, POLAND

SN 1734-1922

EI 1896-9151

J9 ARCH MED SCI

JI Arch. Med. Sci.

PD JUN

PY 2020

VL 16

IS 4

BP 974

EP 983

DI 10.5114/aoms.2020.92293

PG 10

WC Medicine, General & Internal

WE Science Citation Index Expanded (SCI-EXPANDED)

SC General & Internal Medicine

GA LX0AL

UT WOS:000539503400030

PM 32542101

OA Green Published, gold

DA 2025-04-09

ER

PT J

AU Olivier, C

Oliver, L

Lalier, L

Vallette, FM

AF Olivier, Christophe

Oliver, Lisa

Lalier, Lisenn

Vallette, Francois M.

TI Drug Resistance in Glioblastoma: The Two Faces of Oxidative Stress

SO FRONTIERS IN MOLECULAR BIOSCIENCES

LA English

DT Review

DE glioblastoma; oxidative stress; drug resistance; tumor microenvironment;

nutrition

AB Glioblastomas (GBM) are the most common primary brain tumor with a median survival of 15 months. A population of cells with stem cell properties (glioblastoma stem cells, GSCs) drives the initiation and progression of GBM and is localized in specialized microenvironments which support their behavior. GBM are characterized as extremely resistant to therapy, resulting in tumor recurrence. Reactive oxygen species (ROS) control the cellular stability by influencing different signaling pathways. Normally, redox systems prevent cell oxidative damage; however, in gliomagenesis, the cellular redox mechanisms are highly impaired. Herein we review the dual nature of the redox status in drug resistance. ROS generation in tumor cells affects the cell cycle and is involved in tumor progression and drug resistance in GBM. However, excess ROS production has been found to induce cell death programs such as apoptosis and autophagy. Since GBM cells have a high metabolic rate and produce high levels of ROS, metabolic adaptation in these cells plays an essential role in resistance to oxidative stress-induced cell death. Finally, the microenvironment with the stromal components participates in the enhancement of the oxidative stress to promote tumor progression and drug resistance.

C1 [Olivier, Christophe] Fac Sci Pharmaceut & Biol, Nantes, France.

[Olivier, Christophe; Oliver, Lisa; Lalier, Lisenn; Vallette, Francois M.] Univ Nantes, INSERM, CRCINA, Nantes, France.

[Oliver, Lisa] CHU Nantes, Nantes, France.

[Lalier, Lisenn; Vallette, Francois M.] ICO, LaBCT, St Herblain, France.

C3 Institut National de la Sante et de la Recherche Medicale (Inserm);

Nantes Universite; Nantes Universite; CHU de Nantes; UNICANCER; Institut

de Cancerologie de l'Ouest (ICO)

RP Olivier, C (corresponding author), Fac Sci Pharmaceut & Biol, Nantes, France.; Olivier, C (corresponding author), Univ Nantes, INSERM, CRCINA, Nantes, France.; Oliver, L (corresponding author), CHU Nantes, Nantes, France.

EM christophe.olivier@univ-nantes.fr; lisa.oliver@univ-nantes.fr

RI Oliver, Lisa/L-3070-2015; Lalier, Lisenn/K-9230-2015; Vallette,

Francois/K-9047-2015

OI Vallette, Francois/0000-0002-3296-8572

FU INSERM/University of Nantes

FX This work was supported by INSERM/University of Nantes.

CR Agostinelli E, 2006, AMINO ACIDS, V31, P341, DOI 10.1007/s00726-005-0271-8

Allen M, 2011, J PATHOL, V223, P162, DOI 10.1002/path.2803

Annabi B, 2009, J NEUROINFLAMM, V6, DOI 10.1186/1742-2094-6-8

Anrather J, 2006, J BIOL CHEM, V281, P5657, DOI 10.1074/jbc.M506172200

Babior BM, 1999, BLOOD, V93, P1464, DOI 10.1182/blood.V93.5.1464.405a32_1464_1476

Badie B, 2000, NEUROSURGERY, V46, P957, DOI 10.1097/00006123-200004000-00035

Baker JR, 2016, SCI REP-UK, V6, DOI 10.1038/srep35871

Bao SD, 2006, NATURE, V444, P756, DOI 10.1038/nature05236

Baulch JE, 2016, ENVIRON MOL MUTAGEN, V57, P405, DOI 10.1002/em.21988

Beier CP, 2012, STEM CELLS DEV, V21, P2753, DOI 10.1089/scd.2011.0660

Bhat KPL, 2013, CANCER CELL, V24, P331, DOI 10.1016/j.ccr.2013.08.001

Bissell MJ, 2001, NAT REV CANCER, V1, P46, DOI 10.1038/35094059

Brantley EC, 2008, MOL CANCER RES, V6, P675, DOI 10.1158/1541-7786.MCR-07-2180

Brierley DJ, 2013, ANTIOXID REDOX SIGN, V18, P2420, DOI 10.1089/ars.2012.4994

Brizel DM, 2001, INT J RADIAT ONCOL, V51, P349, DOI 10.1016/S0360-3016(01)01630-3

Brooks LJ, 2017, CURR OPIN NEUROBIOL, V47, P8, DOI 10.1016/j.conb.2017.06.008

Buccarelli M, 2018, CELL DEATH DIS, V9, DOI 10.1038/s41419-018-0864-7

BURDON RH, 1990, FREE RADICAL RES COM, V11, P65, DOI 10.3109/10715769009109669

CERUTTI PA, 1985, SCIENCE, V227, P375, DOI 10.1126/science.2981433

Chang KY, 2017, BIOCHEM BIOPH RES CO, V493, P14, DOI 10.1016/j.bbrc.2017.09.095

Chang KY, 2017, REDOX BIOL, V13, P655, DOI 10.1016/j.redox.2017.08.005

Chen HL, 2002, CANCER CAUSE CONTROL, V13, P647, DOI 10.1023/A:1019527225197

Chen J, 2012, NATURE, V488, P522, DOI 10.1038/nature11287

Chien CH, 2019, J BIOMED SCI, V26, DOI 10.1186/s12929-019-0565-2

Comito G, 2011, FREE RADICAL BIO MED, V51, P893, DOI 10.1016/j.freeradbiomed.2011.05.042

Conti A, 2007, BRAIN RES REV, V54, P205, DOI 10.1016/j.brainresrev.2007.01.013

Conti Alfredo, 2010, Cancers (Basel), V2, P693, DOI 10.3390/cancers2020693

Coppé JP, 2010, ANNU REV PATHOL-MECH, V5, P99, DOI 10.1146/annurev-pathol-121808-102144

Costa A, 2014, SEMIN CANCER BIOL, V25, P23, DOI 10.1016/j.semcancer.2013.12.007

Da Ros M, 2018, INT J MOL SCI, V19, DOI 10.3390/ijms19102879

Deshors P, 2019, CELL DEATH DIS, V10, DOI 10.1038/s41419-019-2055-6

Dewhirst MW, 2008, NAT REV CANCER, V8, P425, DOI 10.1038/nrc2397

Dhar SK, 2012, FREE RADICAL BIO MED, V52, P2209, DOI 10.1016/j.freeradbiomed.2012.03.009

Diehn M, 2009, NATURE, V458, P780, DOI 10.1038/nature07733

Dittmann LM, 2012, ONCOGENE, V31, P3409, DOI 10.1038/onc.2011.513

Djavaheri-Mergny M, 2004, FEBS LETT, V578, P111, DOI 10.1016/j.febslet.2004.10.082

Fan QW, 2010, SCI SIGNAL, V3, DOI 10.1126/scisignal.2001017

Fan Z, 2017, ONCOGENESIS, V6, DOI 10.1038/oncsis.2017.65

Fang JS, 2008, SEMIN CANCER BIOL, V18, P330, DOI 10.1016/j.semcancer.2008.03.011

Feng F, 2019, BIOMED PHARMACOTHER, V120, DOI 10.1016/j.biopha.2019.109441

Fiaschi Tania, 2012, Int J Cell Biol, V2012, P762825, DOI 10.1155/2012/762825

Ford K, 2020, CANCER RES, V80, P1846, DOI 10.1158/0008-5472.CAN-19-3158

Frijhoff J, 2014, ANTIOXID REDOX SIGN, V20, P1994, DOI 10.1089/ars.2013.5643

Frisch J, 2019, CANCERS, V11, DOI 10.3390/cancers11040457

Gatenby RA, 2004, NAT REV CANCER, V4, P891, DOI 10.1038/nrc1478

Giannoni E, 2012, ANTIOXID REDOX SIGN, V16, P1248, DOI 10.1089/ars.2011.4280

Goetze K, 2011, INT J ONCOL, V39, P453, DOI 10.3892/ijo.2011.1055

Greaves M, 2012, NATURE, V481, P306, DOI 10.1038/nature10762

Grivennikov SI, 2010, CELL, V140, P883, DOI 10.1016/j.cell.2010.01.025

Groussard C, 2000, J APPL PHYSIOL, V89, P169, DOI 10.1152/jappl.2000.89.1.169

Haas B, 2018, INT J MOL SCI, V19, DOI 10.3390/ijms19102874

Han SR, 2015, MOL MED REP, V12, P2239, DOI 10.3892/mmr.2015.3625

Heddleston JM, 2009, CELL CYCLE, V8, P3274, DOI 10.4161/cc.8.20.9701

Hegi M., 2004, Eur. J. Cancer Suppl, V2, P14, DOI [10.1016/S1359-6349(04)80039-9, DOI 10.1016/S1359-6349(04)80039-9]

Heiden MGV, 2009, SCIENCE, V324, P1029, DOI 10.1126/science.1160809

Hervouet E, 2013, NUTR CANCER, V65, P686, DOI 10.1080/01635581.2013.789541

Hira VVV, 2017, BBA-MOL CELL RES, V1864, P594, DOI 10.1016/j.bbamcr.2016.12.021

Hira VVV, 2015, J HISTOCHEM CYTOCHEM, V63, P481, DOI 10.1369/0022155415581689

Hirschhaeuser F, 2011, CANCER RES, V71, P6921, DOI 10.1158/0008-5472.CAN-11-1457

Hjelmeland AB, 2011, CELL DEATH DIFFER, V18, P829, DOI 10.1038/cdd.2010.150

Hsieh CH, 2015, CLIN CANCER RES, V21, P460, DOI 10.1158/1078-0432.CCR-14-0618

Hsieh CH, 2012, FREE RADICAL BIO MED, V53, P649, DOI 10.1016/j.freeradbiomed.2012.06.009

Ito K, 2014, NAT REV MOL CELL BIO, V15, P243, DOI 10.1038/nrm3772

Janiszewska M, 2012, GENE DEV, V26, P1926, DOI 10.1101/gad.188292.112

Jaramillo MC, 2013, GENE DEV, V27, P2179, DOI 10.1101/gad.225680.113

Jiang G, 2012, CURR MED CHEM, V19, P3886, DOI 10.2174/092986712802002446

Jin X, 2017, NAT MED, V23, P1352, DOI 10.1038/nm.4415

Jin ZS, 2017, BIOSCI BIOTECH BIOCH, V81, P1899, DOI 10.1080/09168451.2017.1364965

Johnson DE, 2015, ENDOCR-RELAT CANCER, V22, pT1, DOI 10.1530/ERC-14-0005

Kabat GC, 2010, CANCER EPIDEM BIOMAR, V19, P2421, DOI 10.1158/1055-9965.EPI-10-0658

Kahlon Arunpreet Singh, 2016, Proc (Bayl Univ Med Cent), V29, P313

Kairisalo M, 2007, BIOCHEM BIOPH RES CO, V364, P138, DOI 10.1016/j.bbrc.2007.09.115

Kalluri R, 2016, J CLIN INVEST, V126, P1208, DOI 10.1172/JCI81135

Kanzawa T, 2004, CELL DEATH DIFFER, V11, P448, DOI 10.1038/sj.cdd.4401359

Ke C, 2014, ONCOTARGET, V5, P1657, DOI 10.18632/oncotarget.1823

Kensler TW, 2007, ANNU REV PHARMACOL, V47, P89, DOI 10.1146/annurev.pharmtox.46.120604.141046

Kim SH, 2017, ONCOTARGET, V8, P111581, DOI 10.18632/oncotarget.22875

Kim SS, 2013, BIOCHEM BIOPH RES CO, V440, P658, DOI 10.1016/j.bbrc.2013.09.120

Kim SH, 2014, J NEUROSCI RES, V92, P1419, DOI 10.1002/jnr.23431

Klimova T, 2008, CELL DEATH DIFFER, V15, P660, DOI 10.1038/sj.cdd.4402307

KOESTNER A, 1971, AM J PATHOL, V63, P37

Kong Q, 2000, MED HYPOTHESES, V55, P29, DOI 10.1054/mehy.1999.0982

Kore RA, 2018, BIOCHEM BIOPHYS REP, V14, P104, DOI 10.1016/j.bbrep.2018.03.008

Krause MS, 2007, CELL BIOCHEM FUNCT, V25, P23, DOI 10.1002/cbf.1343

Kubli DA, 2012, CIRC RES, V111, P1208, DOI 10.1161/CIRCRESAHA.112.265819

Kyritsis AP, 2011, NUTR CANCER, V63, P174, DOI 10.1080/01635581.2011.523807

Lagadec C, 2012, STEM CELLS, V30, P833, DOI 10.1002/stem.1058

Lan ML, 2012, PLOS ONE, V7, DOI 10.1371/journal.pone.0050048

Lan XY, 2017, NATURE, V549, P227, DOI 10.1038/nature23666

Lee HC, 2004, INT J MOL MED, V13, P883

Lee JH, 2018, NATURE, V560, P243, DOI 10.1038/s41586-018-0389-3

Li X, 2011, TOXICOL APPL PHARM, V251, P146, DOI 10.1016/j.taap.2010.12.007

Li Z, 2009, CANCER CELL, V15, P501, DOI 10.1016/j.ccr.2009.03.018

Lin CC, 2007, EUR J PHARMACOL, V560, P101, DOI 10.1016/j.ejphar.2007.01.025

Lin CJ, 2012, FREE RADICAL BIO MED, V52, P377, DOI 10.1016/j.freeradbiomed.2011.10.487

Liu HG, 2015, AM J CANCER RES, V5, P880

Liu Y, 2016, ONCOTARGET, V7, P42740, DOI 10.18632/oncotarget.8600

Lo Dico A, 2019, CELLS-BASEL, V8, DOI 10.3390/cells8111315

Lo Dico A, 2018, FRONT ONCOL, V8, DOI 10.3389/fonc.2018.00249

Lomonaco SL, 2009, INT J CANCER, V125, P717, DOI 10.1002/ijc.24402

Louis DN, 2016, ACTA NEUROPATHOL, V131, P803, DOI 10.1007/s00401-016-1545-1

Lu X, 2010, CANCER RES, V70, P3905, DOI 10.1158/0008-5472.CAN-09-3739

Lyakhovich A, 2016, OXID MED CELL LONGEV, V2016, DOI 10.1155/2016/1716341

Ma Q, 2013, ANNU REV PHARMACOL, V53, P401, DOI 10.1146/annurev-pharmtox-011112-140320

MacLeod G, 2019, CELL REP, V27, P971, DOI 10.1016/j.celrep.2019.03.047

Maier P, 2016, INT J MOL SCI, V17, DOI 10.3390/ijms17010102

Mao P, 2013, P NATL ACAD SCI USA, V110, P8644, DOI 10.1073/pnas.1221478110

Marks PA, 2006, SEMIN CANCER BIOL, V16, P436, DOI 10.1016/j.semcancer.2006.09.005

Marnett LJ, 2000, CARCINOGENESIS, V21, P361, DOI 10.1093/carcin/21.3.361

Matschke J, 2016, ANTIOXID REDOX SIGN, V25, P89, DOI 10.1089/ars.2015.6589

Mittal M, 2014, ANTIOXID REDOX SIGN, V20, P1126, DOI 10.1089/ars.2012.5149

Morgan MJ, 2011, CELL RES, V21, P103, DOI 10.1038/cr.2010.178

Murphy MP, 2009, BIOCHEM J, V417, P1, DOI 10.1042/BJ20081386

Nagai S, 2002, J NEUROSURG, V96, P909, DOI 10.3171/jns.2002.96.5.0909

Nakai E, 2009, CANCER INVEST, V27, P901, DOI 10.3109/07357900801946679

Nanjaiah ND, 2019, CELL BIOL INT, V43, P1443, DOI 10.1002/cbin.11193

Narayanan D, 2020, CANCERS, V12, DOI 10.3390/cancers12071706

Nathan C, 2013, NAT REV IMMUNOL, V13, P349, DOI 10.1038/nri3423

Nishiya T, 2000, BIOCHEM BIOPH RES CO, V275, P268, DOI 10.1006/bbrc.2000.3293

Oliva CR, 2011, PLOS ONE, V6, DOI 10.1371/journal.pone.0024665

Orimo A, 2005, CELL, V121, P335, DOI 10.1016/j.cell.2005.02.034

Ortega Angel L, 2011, Cancers (Basel), V3, P1285, DOI 10.3390/cancers3011285

Ostrom Q.T, 2015, NEURO-ONCOLOGY, V17, P1

Pan H, 2013, NEUROL RES, V35, P71, DOI 10.1179/1743132812Y.0000000094

Pani G, 2000, J BIOL CHEM, V275, P38891, DOI 10.1074/jbc.M007319200

Papandreou I, 2006, CELL METAB, V3, P187, DOI 10.1016/j.cmet.2006.01.012

Park HK, 2019, CANCER RES, V79, P1369, DOI 10.1158/0008-5472.CAN-18-2558

Park SH, 2000, CLIN CANCER RES, V6, P4915

Peppicelli S, 2017, CELL MOL LIFE SCI, V74, P2761, DOI 10.1007/s00018-017-2496-y

Pham CG, 2004, CELL, V119, P529, DOI 10.1016/j.cell.2004.10.017

Pistollato F, 2010, STEM CELLS, V28, P851, DOI 10.1002/stem.415

Polewski MD, 2016, MOL CANCER RES, V14, P1229, DOI 10.1158/1541-7786.MCR-16-0028

Poschmann G, 2015, BBA-PROTEINS PROTEOM, V1854, P624, DOI 10.1016/j.bbapap.2014.11.011

Pouliquen D, 2008, INT J CANCER, V123, P288, DOI 10.1002/ijc.23513

Rademakers SE, 2008, MOL ONCOL, V2, P41, DOI 10.1016/j.molonc.2008.03.006

Raghunand N, 2000, DRUG RESIST UPDATE, V3, P39, DOI 10.1054/drup.2000.0119

Rajesh Y, 2019, BIOCHEM PHARMACOL, V164, P1, DOI 10.1016/j.bcp.2019.03.025

Rocha CRR, 2016, ONCOTARGET, V7, P48081, DOI 10.18632/oncotarget.10129

Rhee S G, 2000, Sci STKE, V2000, ppe1

Rojo AI, 2004, J NEUROSCI, V24, P7324, DOI 10.1523/JNEUROSCI.2111-04.2004

Rong Y, 2006, J NEUROPATH EXP NEUR, V65, P529, DOI 10.1097/00005072-200606000-00001

Roos WP, 2007, ONCOGENE, V26, P186, DOI 10.1038/sj.onc.1209785

Saitoh M, 1998, EMBO J, V17, P2596, DOI 10.1093/emboj/17.9.2596

Salaud C, 2020, BIOCHEM BIOPH RES CO, V533, P139, DOI 10.1016/j.bbrc.2020.08.101

Santos P, 2020, CELLS-BASEL, V9, DOI 10.3390/cells9061450

Sattler UGA, 2010, RADIOTHER ONCOL, V94, P102, DOI 10.1016/j.radonc.2009.11.007

Scherz-Shouval R, 2007, EMBO J, V26, P1749, DOI 10.1038/sj.emboj.7601623

Schiffer D, 2019, CANCERS, V11, DOI 10.3390/cancers11010005

Schreiber J, 2006, P NATL ACAD SCI USA, V103, P5899, DOI 10.1073/pnas.0510996103

Sedlak TW, 2004, PEDIATRICS, V113, P1776, DOI 10.1542/peds.113.6.1776

Sharapov MG, 2019, BIOCHEMISTRY-MOSCOW+, V84, P79, DOI 10.1134/S0006297919020019

Shibao S, 2018, NEURO-ONCOLOGY, V20, P343, DOI 10.1093/neuonc/nox170

Siddique HR, 2012, STEM CELLS, V30, P372, DOI 10.1002/stem.1035

Smith AG, 2019, J PATHOL, V247, P708, DOI 10.1002/path.5222

Smith WL, 1996, J BIOL CHEM, V271, P33157, DOI 10.1074/jbc.271.52.33157

Soda Y, 2011, P NATL ACAD SCI USA, V108, P4274, DOI 10.1073/pnas.1016030108

Sporn MB, 2012, NAT REV CANCER, V12, P564, DOI 10.1038/nrc3278

Stepkowski TM, 2011, FREE RADICAL BIO MED, V50, P1186, DOI 10.1016/j.freeradbiomed.2011.01.033

Storz P, 2005, FRONT BIOSCI-LANDMRK, V10, P1881, DOI 10.2741/1667

Stupp R, 2005, NEW ENGL J MED, V352, P987, DOI 10.1056/NEJMoa043330

Stupp R, 2009, LANCET ONCOL, V10, P459, DOI 10.1016/S1470-2045(09)70025-7

Sullivan R, 2017, FRONT ENDOCRINOL, V8, DOI 10.3389/fendo.2017.00194

Svendsen A, 2011, ACTA NEUROPATHOL, V122, P495, DOI 10.1007/s00401-011-0867-2

Tamari Y, 2017, J RADIAT RES, V58, P412, DOI 10.1093/jrr/rrw084

Tang Z, 2019, ONCOL LETT, V18, P2509, DOI 10.3892/ol.2019.10574

Tebay LE, 2015, FREE RADICAL BIO MED, V88, P108, DOI 10.1016/j.freeradbiomed.2015.06.021

Tedeschi-Blok N, 2006, BMC CANCER, V6, DOI 10.1186/1471-2407-6-148

Tivnan A, 2015, FRONT NEUROSCI-SWITZ, V9, DOI 10.3389/fnins.2015.00218

Tlsty TD, 2006, ANNU REV PATHOL-MECH, V1, P119, DOI 10.1146/annurev.pathol.1.110304.100224

Tomasetti C, 2017, SCIENCE, V355, P1330, DOI 10.1126/science.aaf9011

Trachootham D, 2009, NAT REV DRUG DISCOV, V8, P579, DOI 10.1038/nrd2803

Traverso N, 2013, OXID MED CELL LONGEV, V2013, DOI 10.1155/2013/972913

Tsai JY, 2014, EXP CELL RES, V323, P28, DOI 10.1016/j.yexcr.2014.02.014

Tsujii M, 1998, CELL, V93, P705, DOI 10.1016/S0092-8674(00)81433-6

VAN MEIR E, 1990, CANCER RES, V50, P6683

Vander Heiden MG, 2017, CELL, V168, DOI 10.1016/j.cell.2016.12.039

Vaupel P, 2007, CANCER METAST REV, V26, P225, DOI 10.1007/s10555-007-9055-1

Vaupel P, 2017, FRONT IMMUNOL, V8, DOI 10.3389/fimmu.2017.01887

Vlashi E, 2011, P NATL ACAD SCI USA, V108, P16062, DOI 10.1073/pnas.1106704108

Wang H, 2009, STEM CELLS, V27, P2393, DOI 10.1002/stem.188

Wang J, 2008, CANCER BIOL THER, V7, P1875, DOI 10.4161/cbt.7.12.7067

Wang K, 2011, AUTOPHAGY, V7, P297, DOI 10.4161/auto.7.3.14502

Wang XJ, 2008, CARCINOGENESIS, V29, P1235, DOI 10.1093/carcin/bgn095

Weinberg F, 2019, CANCERS, V11, DOI 10.3390/cancers11081191

Whiteside TL, 2016, ADV CLIN CHEM, V74, P103, DOI 10.1016/bs.acc.2015.12.005

Xu P, 1996, GENOMICS, V34, P173, DOI 10.1006/geno.1996.0262

Yan YL, 2016, J EXP CLIN CANC RES, V35, DOI 10.1186/s13046-016-0303-5

Ye F, 2013, PLOS ONE, V8, DOI 10.1371/journal.pone.0080397

Yeh SH, 2011, NUCLEIC ACIDS RES, V39, P5412, DOI 10.1093/nar/gkr161

Yoshida S, 2013, P NATL ACAD SCI USA, V110, pE1604, DOI 10.1073/pnas.1220659110

Zhang PX, 2017, ONCOL LETT, V14, P2053, DOI 10.3892/ol.2017.6397

Zhang WB, 2010, J BIOL CHEM, V285, P40461, DOI 10.1074/jbc.M110.164046

Zhang ZP, 2020, CANCER IMMUNOL RES, V8, P966, DOI 10.1158/2326-6066.CIR-19-0759

Zhou M, 2019, SCI REP-UK, V9, DOI 10.1038/s41598-019-42313-8

Zhou Y, 2013, ONCOL REP, V29, P394, DOI 10.3892/or.2012.2115

Zhu JH, 2014, ONCOL REP, V32, P1170, DOI 10.3892/or.2014.3320

Zhu JH, 2013, BMC CANCER, V13, DOI 10.1186/1471-2407-13-380

Zhu ZL, 2018, J NEUROCHEM, V144, P93, DOI 10.1111/jnc.14250

NR 198

TC 114

Z9 116

U1 1

U2 28

PU FRONTIERS MEDIA SA

PI LAUSANNE

PA AVENUE DU TRIBUNAL FEDERAL 34, LAUSANNE, CH-1015, SWITZERLAND

EI 2296-889X

J9 FRONT MOL BIOSCI

JI Front. Mol. Biosci.

PD JAN 27

PY 2021

VL 7

AR 620677

DI 10.3389/fmolb.2020.620677

PG 16

WC Biochemistry & Molecular Biology

WE Science Citation Index Expanded (SCI-EXPANDED)

SC Biochemistry & Molecular Biology

GA QE9JD

UT WOS:000616519000001

PM 33585565

OA Green Published, gold

DA 2025-04-09

ER

PT J

AU Lu, D

Yang, N

Wang, S

Liu, WY

Zhang, D

Wang, J

Huang, B

Li, XG

AF Lu, Di

Yang, Ning

Wang, Shuai

Liu, Wenyu

Zhang, Di

Wang, Jian

Huang, Bin

Li, Xingang

TI Identifying the Predictive Role of Oxidative Stress Genes in the

Prognosis of Glioma Patients

SO MEDICAL SCIENCE MONITOR

LA English

DT Article

DE Computational Biology; Glioma; Models; Statistical; Oxidative Stress;

Prognosis

ID DEPENDENT APOPTOSIS; CELL-DEATH; MUTATIONS; MITOCHONDRIA; TEMOZOLOMIDE;

DYSFUNCTION; PROGRESSION; ASTROCYTES; RESISTANCE; NOMOGRAM

AB Background: Gliomas are primary aggressive brain tumors with poor prognoses. Oxidative stress plays a crucial role in the tumorigenesis and drug resistance of gliomas. The aim of the present study was to use integrated bioinformatics analyses to evaluate the prognostic value of oxidative stress-related genes (OSRGs) in glioma. Material/Methods: Disease- and prognosis-associated OSRGs were identified using microarray and clinical data from the Chinese Glioma Genome Atlas database. Functional enrichment, gene-gene interaction, protein-protein interaction, and survival analyses were performed in screened OSRGs. The protein expression was validated by the Human Protein Atlas database. A risk score model was constructed and verified through Cox regression, receiver operating characteristic curve, principal component, and stratified analyses. The Cancer Genome Atlas (TCGA) database was used for external validation. A nomogram was constructed to facilitate the clinical application. Results: Twenty-one disease-associated and 14 prognosis-associated OSRGs were identified. Enrichment analyses indicated that these signature OSRGs were involved in tumorigenesis and drug resistance of glioma. The risk score model demonstrated a significant difference in overall survival between the high- and low-risk groups. The area under the curve and hazard ratio (1.296) revealed the independent prognostic value of the model. The model exhibited good predictive efficacy in the TCGA cohort. A clinical nomogram was constructed to calculate survival rates in glioma patients at 1, 3, and 5 years. Conclusions: Our comprehensive study indicated that OSRGs were valuable for prognosis prediction in glioma, which provides a novel insight into the relationship between oxidative stress and glioma and a potential therapeutic strategy for glioma patients.

C1 [Lu, Di; Yang, Ning; Wang, Shuai; Liu, Wenyu; Zhang, Di; Wang, Jian; Huang, Bin; Li, Xingang] Shandong Univ, Dept Neurosurg, Qilu Hosp, Cheeloo Coll Med, Jinan, Shandong, Peoples R China.

[Lu, Di; Yang, Ning; Wang, Shuai; Liu, Wenyu; Zhang, Di; Wang, Jian; Huang, Bin; Li, Xingang] Shandong Univ, Inst Brain & Brain Inspired Sci, Jinan, Shandong, Peoples R China.

[Lu, Di; Yang, Ning; Wang, Shuai; Liu, Wenyu; Zhang, Di; Wang, Jian; Huang, Bin; Li, Xingang] Shandong Univ, Key Lab Brain Funct Remodeling, Qilu Hosp, Jinan, Shandong, Peoples R China.

[Wang, Jian] Univ Bergen, Dept Biomed, Bergen, Norway.

C3 Shandong University; Shandong University; Shandong University;

University of Bergen

RP Huang, B; Li, XG (corresponding author), Shandong Univ, Dept Neurosurg, Qilu Hosp, Cheeloo Coll Med, Jinan, Shandong, Peoples R China.; Huang, B; Li, XG (corresponding author), Shandong Univ, Inst Brain & Brain Inspired Sci, Jinan, Shandong, Peoples R China.; Huang, B; Li, XG (corresponding author), Shandong Univ, Key Lab Brain Funct Remodeling, Qilu Hosp, Jinan, Shandong, Peoples R China.

EM hb@sdu.edu.cn; lixg@sdu.edu.cn

RI Li, Xingang/P-1522-2017; Lu, Di/HPB-9415-2023; Wang, Jing/B-3198-2009;

Wang, Shuai/HZJ-7466-2023; Huang, Bin/AAT-4514-2021

OI Lu, Di/0009-0002-5499-260X; Wang, Shuai/0000-0002-0454-5281; Wang,

Jian/0000-0002-9482-5227

FU National Natural Science Foundation of China [81702474, 81701329,

81972351]; Department of Science & Technology of Shandong Province

[ZR2019ZD33, 2018CXGC1503]; Special Foundation for Taishan Scholars

[ts20110814, tshw201502056, tsqn20161067]; Jinan Science and Technology

Bureau of Shandong Province [2019GXRC006]; Clinical Research Center of

Shandong University [2020SDUCRCB002]; Shandong Development and Reform

Commission; Shandong Research Institute of Industrial Technology

FX This work was supported by the National Natural Science Foundation of

China (81702474, 81701329, and 81972351), the Department of Science &

Technology of Shandong Province (ZR2019ZD33 and 2018CXGC1503), the

Special Foundation for Taishan Scholars (ts20110814, tshw201502056, and

tsqn20161067), the Jinan Science and Technology Bureau of Shandong

Province (2019GXRC006), Clinical Research Center of Shandong University

(No. 2020SDUCRCB002), Shandong Development and Reform Commission, and

the Shandong Research Institute of Industrial Technology

CR BALIN AK, 1984, J EXP MED, V160, P152, DOI 10.1084/jem.160.1.152

Brown SL, 2007, J CLIN INVEST, V117, P258, DOI 10.1172/JCI29159

Butterfield DA, 2019, NAT REV NEUROSCI, V20, P148, DOI 10.1038/s41583-019-0132-6

Cairncross G, 2008, CANCER J, V14, P352, DOI 10.1097/PPO.0b013e31818d8178

Chen HJ, 2021, J CELL PHYSIOL, V236, P2988, DOI 10.1002/jcp.30060

Cheng X, 2020, CELL METAB, V32, P229, DOI 10.1016/j.cmet.2020.06.002

Cheung HC, 2008, BMC GENOMICS, V9, DOI 10.1186/1471-2164-9-216

Datta K, 2002, INT J BIOCHEM CELL B, V34, P148, DOI 10.1016/S1357-2725(01)00106-6

Degterev A, 2005, NAT CHEM BIOL, V1, P112, DOI 10.1038/nchembio711

Duan SB, 2018, ONCOTARGETS THER, V11, P6395, DOI 10.2147/OTT.S173244

Everhard S, 2006, ANN NEUROL, V60, P740, DOI 10.1002/ana.21044

FAWELL S, 1994, P NATL ACAD SCI USA, V91, P664, DOI 10.1073/pnas.91.2.664

Golstein P, 2007, TRENDS BIOCHEM SCI, V32, P37, DOI 10.1016/j.tibs.2006.11.001

Han MZ, 2020, NEURO-ONCOLOGY, V22, P729, DOI 10.1093/neuonc/noaa027

Han Y, 2018, MOL CARCINOGEN, V57, P722, DOI 10.1002/mc.22793

Hao CS, 2020, STEM CELL RES THER, V11, DOI 10.1186/s13287-020-01759-8

Hayes JD, 2020, CANCER CELL, V38, P167, DOI 10.1016/j.ccell.2020.06.001

Iasonos A, 2008, J CLIN ONCOL, V26, P1364, DOI 10.1200/JCO.2007.12.9791

Kant S, 2020, CELL DEATH DIS, V11, DOI 10.1038/s41419-020-2449-5

LAPINSKAS PJ, 1995, MOL CELL BIOL, V15, P1382

Lapointe S, 2018, LANCET, V392, P432, DOI 10.1016/S0140-6736(18)30990-5

Leek JT, 2012, BIOINFORMATICS, V28, P882, DOI 10.1093/bioinformatics/bts034

Leek JT, 2010, NAT REV GENET, V11, P733, DOI 10.1038/nrg2825

Louis DN, 2016, ACTA NEUROPATHOL, V131, P803, DOI 10.1007/s00401-016-1545-1

Luo SL, 2018, OXID MED CELL LONGEV, V2018, DOI 10.1155/2018/9146528

Massi P, 2006, CELL MOL LIFE SCI, V63, P2057, DOI 10.1007/s00018-006-6156-x

Orrenius S, 2015, BIOCHEM BIOPH RES CO, V460, P72, DOI 10.1016/j.bbrc.2015.01.137

Ortmann B, 2014, CELL MOL LIFE SCI, V71, P3569, DOI 10.1007/s00018-014-1645-9

Ostrom QT, 2020, NEURO-ONCOLOGY, V22, P1, DOI 10.1093/neuonc/noaa200

Pedrotti S, 2012, NUCLEIC ACIDS RES, V40, P1021, DOI 10.1093/nar/gkr819

Pistollato F, 2007, MOL CELL NEUROSCI, V35, P424, DOI 10.1016/j.mcn.2007.04.003

Ritchie ME, 2015, NUCLEIC ACIDS RES, V43, DOI 10.1093/nar/gkv007

Shannon P, 2003, GENOME RES, V13, P2498, DOI 10.1101/gr.1239303

Silber JR, 2002, CLIN CANCER RES, V8, P3008

Simon MP, 1997, NAT GENET, V15, P95, DOI 10.1038/ng0197-95

Soon BH, 2017, FRONT PHYSIOL, V8, DOI 10.3389/fphys.2017.00231

Sternlicht MD, 1999, CELL, V98, P137, DOI 10.1016/S0092-8674(00)81009-0

Subramanian A, 2005, P NATL ACAD SCI USA, V102, P15545, DOI 10.1073/pnas.0506580102

Szklarczyk D, 2019, NUCLEIC ACIDS RES, V47, pD607, DOI [10.1093/nar/gky1131, 10.1093/nar/gkac1000]

Tsai KL, 1997, J PHYSIOL-LONDON, V502, P161, DOI 10.1111/j.1469-7793.1997.161bl.x

Uhlen M, 2017, SCIENCE, V357, P660, DOI 10.1126/science.aan2507

Uhlen M, 2015, SCIENCE, V347, DOI 10.1126/science.1260419

Varambally S, 2002, NATURE, V419, P624, DOI 10.1038/nature01075

Violi F, 2017, ANTIOXID REDOX SIGN, V27, P1083, DOI 10.1089/ars.2016.6963

Wang K, 2020, FRONT PHARMACOL, V11, DOI 10.3389/fphar.2020.00835

Warde-Farley D, 2010, NUCLEIC ACIDS RES, V38, pW214, DOI 10.1093/nar/gkq537

Wätjen W, 2004, BIOMETALS, V17, P65, DOI 10.1023/A:1024405119018

Wu LQ, 2019, J CELL BIOCHEM, V120, P19044, DOI 10.1002/jcb.29227

Xia SL, 2005, CANCER RES, V65, P5248, DOI 10.1158/0008-5472.CAN-04-4332

Yan H, 2009, NEW ENGL J MED, V360, P765, DOI 10.1056/NEJMoa0808710

Yaribeygi H, 2020, OXID MED CELL LONGEV, V2020, DOI 10.1155/2020/8609213

You J, 2018, BIOMED RES INT, V2018, DOI 10.1155/2018/2109865

Yu GC, 2012, OMICS, V16, P284, DOI 10.1089/omi.2011.0118

Zhang HY, 2009, TOXICOL SCI, V110, P376, DOI 10.1093/toxsci/kfp101

Zhang JM, 2020, BRIT J HAEMATOL, V190, P67, DOI 10.1111/bjh.16505

NR 55

TC 8

Z9 8

U1 1

U2 12

PU INT SCIENTIFIC INFORMATION, INC

PI MELVILLE

PA 150 BROADHOLLOW RD, STE 114, MELVILLE, NY 11747 USA

EI 1643-3750

J9 MED SCI MONITOR

JI Med. Sci. Monitor

PD NOV 27

PY 2021

VL 27

AR e934161

DI 10.12659/MSM.934161

PG 22

WC Medicine, Research & Experimental

WE Science Citation Index Expanded (SCI-EXPANDED)

SC Research & Experimental Medicine

GA XF1IL

UT WOS:000723830700001

PM 34836934

OA Green Published

DA 2025-04-09

ER

PT J

AU Allani, SK

Weissbach, H

Lopez-Toledano, MA

AF Allani, S. K.

Weissbach, H.

Lopez-Toledano, M. A.

TI Sulindac induces differentiation of glioblastoma stem cells making them

more sensitive to oxidative stress

SO NEOPLASMA

LA English

DT Article

DE glioblastoma; stem cells; sulindac; oxidative stress; differentiation

ID ACUTE PROMYELOCYTIC LEUKEMIA; SUBVENTRICULAR ZONE CELLS; TRANS-RETINOIC

ACID; BRAIN-TUMOR; CANCER-CELLS; IN-VIVO; NEURONAL DIFFERENTIATION;

DENTATE GYRUS; GROWTH-FACTOR; THERAPEUTIC STRATEGIES

AB Glioblastoma tumors (GBM) are very heterogeneous, being comprised of several cell subtypes, including glioblastoma stem cells (GSC). These tumors have a high rate of recurrence after initial treatment and one of the most prevalent theories to explain this is the cancer stem cell theory, which proposes that glioblastomas arise from mutations that transform normal neural stem cells (NSC) into GSC, which are highly resistant to oxidative stress and anti-cancer therapies. Sulindac is a non-steroidal anti-inflammatory drug (NSAID) that has been shown to protect the normal cells against oxidative damage by initiating a preconditioning response, but selectively sensitizes several cancer cell lines to agents that affect mitochondrial respiration, resulting in enhanced killing of the cancer cells. These effects of sulindac are independent of its NSAID activity. There is little information on the effect of sulindac on normal and cancer stem cells. To study the effect of sulindac on both normal and cancer stem cells, we have isolated normal neural stem cells (NSC) from mice hippocampi and glioblastoma stem cells (GSC) from a glioma cell line, U87.

As expected from previous studies, sulindac can protect normal astrocytes against oxidative stress. Sulindac induces differentiation of both NSC and GSC cells and sulindac upregulates neurogenesis in NSC. The differentiated NSC are also protected from oxidative stress damage, whereas the differentiation of GSC by sulindac increases the sensitivity of these cells to agents that cause oxidative stress. The S epimer of sulindac is more effective than the R epimer in inducing neuronal differentiation in both NSC and GSC. These results indicate that the ability of sulindac to induce GSC differentiation may have therapeutic value in preventing tumor recurrence.

C1 [Allani, S. K.; Weissbach, H.; Lopez-Toledano, M. A.] Florida Atlantic Univ, Charles E Schmidt Coll Sci, Ctr Mol Biol & Biotechnol, Jupiter, FL 33458 USA.

[Lopez-Toledano, M. A.] SCI, 2129 North Congress Ave, Riviera Beach, FL 33404 USA.

C3 State University System of Florida; Florida Atlantic University

RP Allani, SK (corresponding author), Florida Atlantic Univ, Charles E Schmidt Coll Sci, Ctr Mol Biol & Biotechnol, Jupiter, FL 33458 USA.

EM skesaraj@fau.edu

RI Allani, Shailaja/AGP-4437-2022; Lopez-Toledano, Miguel A./C-3471-2008

OI Lopez-Toledano, Miguel A./0000-0002-5743-3750

FU Florida Atlantic University Foundation; College of Science Seed Grant

FX The work was supported by funds from the Florida Atlantic University

Foundation to HW and the College of Science Seed Grant to MLT. We thank

Ms. Xiaoping Wu for her assistance in carrying out some of the Western

blot analyses.

CR Achanta P, 2010, ANTI-CANCER AGENT ME, V10, P121, DOI 10.2174/187152010790909290

Ahmad IM, 2005, J BIOL CHEM, V280, P4254, DOI 10.1074/jbc.M411662200

ALTMAN J, 1965, NATURE, V207, P953, DOI 10.1038/207953a0

ARCANGELI A, 1993, P NATL ACAD SCI USA, V90, P5858, DOI 10.1073/pnas.90.12.5858

Aykin-Burns N, 2009, BIOCHEM J, V418, P29, DOI 10.1042/BJ20081258

Ayyanathan K, 2012, PLOS ONE, V7, DOI 10.1371/journal.pone.0039949

BAYER SA, 1982, SCIENCE, V216, P890, DOI 10.1126/science.7079742

Bazán E, 2004, HISTOL HISTOPATHOL, V19, P1261, DOI 10.14670/HH-19.1261

BAZAN E, 1998, UNDERSTANDING GLIAL, P133

Bhola NE, 2013, J CLIN INVEST, V123, P1348, DOI 10.1172/JCI65416

Brunell D, 2011, DRUG METAB DISPOS, V39, P1014, DOI 10.1124/dmd.110.037663

Carey JO, 1996, BLOOD, V87, P4316, DOI 10.1182/blood.V87.10.4316.bloodjournal87104316

Chen J, 2012, NATURE, V488, P522, DOI 10.1038/nature11287

Cheng L, 2010, BIOCHEM PHARMACOL, V80, P654, DOI 10.1016/j.bcp.2010.04.035

Cho DY, 2013, CELL TRANSPLANT, V22, P731, DOI 10.3727/096368912X655136

Das A, 2010, P NATL ACAD SCI USA, V107, P18202, DOI 10.1073/pnas.1006965107

DAVIS AA, 1994, NATURE, V372, P263, DOI 10.1038/372263a0

de-Medeiros BC, 1998, BRAZ J MED BIOL RES, V31, P1537, DOI 10.1590/S0100-879X1998001200005

Demeter K, 2005, NEUROSCI RES, V53, P331, DOI 10.1016/j.neures.2005.08.003

Diabira S, 2008, MED HYPOTHESES, V70, P96, DOI 10.1016/j.mehy.2007.04.024

Dirks PB, 2008, PHILOS T R SOC B, V363, P139, DOI 10.1098/rstb.2006.2017

Dirks PB, 2010, MOL ONCOL, V4, P420, DOI 10.1016/j.molonc.2010.08.001

Dunn GP, 2012, GENE DEV, V26, P756, DOI 10.1101/gad.187922.112

Finlan LE, 2006, EUR J CANCER, V42, P1283, DOI 10.1016/j.ejca.2006.01.047

Fornazari M, 2011, J BIOENERG BIOMEMBR, V43, P531, DOI 10.1007/s10863-011-9374-3

GOLDMAN JE, 1995, J NEURO-ONCOL, V24, P61, DOI 10.1007/BF01052660

Gritti A, 1999, J NEUROSCI, V19, P3287

Gruber BM, 2010, MED SCI MONITOR, V16, pBR45

Guichet PO, 2013, GLIA, V61, P225, DOI 10.1002/glia.22429

Hemmati HD, 2003, P NATL ACAD SCI USA, V100, P15178, DOI 10.1073/pnas.2036535100

Holmberg J, 2011, PLOS ONE, V6, DOI 10.1371/journal.pone.0018454

Iacopino F, 2014, PLOS ONE, V9, DOI 10.1371/journal.pone.0105166

Izes JK, 1996, UROLOGY, V47, P756, DOI 10.1016/S0090-4295(96)00026-X

Jin F, 2008, NEUROSCIENCE, V154, P541, DOI 10.1016/j.neuroscience.2008.03.054

KAPLAN MS, 1984, J NEUROSCI, V4, P1429

Kikuta M, 2013, J PHARMACOL SCI, V121, P74, DOI 10.1254/jphs.12162FP

Kuhn HG, 1996, J NEUROSCI, V16, P2027

Laukaitis CM, 2011, BEST PRACT RES CL GA, V25, P495, DOI 10.1016/j.bpg.2011.09.007

Le Belle JE, 2011, CELL STEM CELL, V8, P59, DOI 10.1016/j.stem.2010.11.028

Ligon KL, 2007, NEURON, V53, P503, DOI 10.1016/j.neuron.2007.01.009

Lim DA, 2007, NEURO-ONCOLOGY, V9, P424, DOI 10.1215/15228517-2007-023

Limoli CL, 2004, P NATL ACAD SCI USA, V101, P16052, DOI 10.1073/pnas.0407065101

Liu W, 2008, J INT MED RES, V36, P890, DOI 10.1177/147323000803600504

Lobo MVT, 2003, J HISTOCHEM CYTOCHEM, V51, P89, DOI 10.1177/002215540305100111

Lois C, 1996, SCIENCE, V271, P978, DOI 10.1126/science.271.5251.978

LOIS C, 1993, P NATL ACAD SCI USA, V90, P2074, DOI 10.1073/pnas.90.5.2074

López-Toledano MA, 2004, J NEUROSCI, V24, P5439, DOI 10.1523/JNEUROSCI.0974-04.2004

Lopez-Toledano MA, 2007, J ALZHEIMERS DIS, V12, P229

LUSKIN MB, 1993, NEURON, V11, P173, DOI 10.1016/0896-6273(93)90281-U

Malagelada C, 2011, J NEUROSCI, V31, P3186, DOI 10.1523/JNEUROSCI.4011-10.2011

Marchetti M, 2009, PLOS ONE, V4, DOI 10.1371/journal.pone.0005804

Meyskens FL, 2008, CANCER PREV RES, V1, P32, DOI 10.1158/1940-6207.CAPR-08-0042

Modarresi Farzaneh, 2011, Int J Alzheimers Dis, V2011, P929042, DOI 10.4061/2011/929042

Moench I, 2009, P NATL ACAD SCI USA, V106, P19611, DOI 10.1073/pnas.0911046106

Moon CM, 2014, INT J CANCER, V134, P519, DOI 10.1002/ijc.28381

Nelson R, 2005, LANCET NEUROL, V4, P17, DOI 10.1016/S1474-4422(04)00955-X

Noble M, 2011, CELL STEM CELL, V8, P1, DOI 10.1016/j.stem.2010.12.005

Okugawa Y, 2013, BRIT J CANCER, V108, P121, DOI 10.1038/bjc.2012.499

Panopoulos AD, 2012, CELL RES, V22, P168, DOI 10.1038/cr.2011.177

Patani N, 2011, CANCER CELL INT, V11, DOI 10.1186/1475-2867-11-23

Pattabiraman DR, 2014, NAT REV DRUG DISCOV, V13, P497, DOI 10.1038/nrd4253

Persano L, 2013, BIOCHEM PHARMACOL, V85, P612, DOI 10.1016/j.bcp.2012.10.001

Piccirillo SGM, 2007, EXPERT OPIN BIOL TH, V7, P1129, DOI 10.1517/14712598.7.8.1129

Prabhakaran P, 2013, FRONT ONCOL, V3, DOI 10.3389/fonc.2013.00134

Ralph SJ, 2006, RECENT PAT ANTI-CANC, V1, P327, DOI 10.2174/157489206778776952

RAY J, 1994, J NEUROSCI, V14, P3548

Resnick L, 2009, J DRUGS DERMATOL, V8, P29

REYNOLDS BA, 1992, SCIENCE, V255, P1707, DOI 10.1126/science.1553558

Sanchez-Martin M, 2008, CURR STEM CELL RES T, V3, P197, DOI 10.2174/157488808785740370

Scheper MA, 2007, NEOPLASIA, V9, P192, DOI 10.1593/neo.06781

Sharma V, 2011, NEUROCHEM INT, V59, P567, DOI 10.1016/j.neuint.2011.06.018

SHIFF SJ, 1995, J CLIN INVEST, V96, P491, DOI 10.1172/JCI118060

Shyh-Chang N, 2013, DEVELOPMENT, V140, P2535, DOI 10.1242/dev.091777

Singh R, 2011, APOPTOSIS, V16, P889, DOI 10.1007/s10495-011-0624-y

STANFIELD BB, 1988, EXP BRAIN RES, V72, P399

Sturt NJH, 2004, CANCER, V101, P652, DOI 10.1002/cncr.20416

Suganuma M, 1999, CANCER RES, V59, P44

Sur A, 2014, P NATL ACAD SCI USA, V111, P16754, DOI 10.1073/pnas.1419576111

Sutter R, 2007, BBA-REV CANCER, V1776, P125, DOI 10.1016/j.bbcan.2007.07.006

Vescovi AL, 2006, NAT REV CANCER, V6, P425, DOI 10.1038/nrc1889

Walton NM, 2012, PLOS ONE, V7, DOI 10.1371/journal.pone.0035264

Wang Y, 2009, CANCER CELL, V15, P514, DOI 10.1016/j.ccr.2009.04.001

WARBURG O, 1956, SCIENCE, V123, P309, DOI 10.1126/science.123.3191.309

Weiss S, 1996, J NEUROSCI, V16, P7599

Wolanczyk M, 2010, FOLIA NEUROPATHOL, V48, P27

WU H, 1991, DIFFERENTIATION, V48, P51, DOI 10.1111/j.1432-0436.1991.tb00242.x

Yip-Schneider MT, 2007, MOL CANCER THER, V6, P1736, DOI 10.1158/1535-7163.MCT-06-0794

Yoneyama M, 2010, NEUROCHEM INT, V56, P740, DOI 10.1016/j.neuint.2009.11.018

Yu JJ, 2006, EXPERT OPIN BIOL TH, V6, P1255, DOI 10.1517/14712598.6.12.1255

Yu SC, 2008, CANCER LETT, V265, P124, DOI 10.1016/j.canlet.2008.02.010

Zaidi HA, 2009, J NEURO-ONCOL, V93, P49, DOI 10.1007/s11060-009-9856-x

Zheng HW, 2010, CANCER CELL, V17, P497, DOI 10.1016/j.ccr.2010.03.020

Zhou GB, 2007, PHILOS T R SOC B, V362, P959, DOI 10.1098/rstb.2007.2026

Zhu ZW, 2001, CLIN CANCER RES, V7, P105

NR 94

TC 7

Z9 7

U1 0

U2 3

PU AEPRESS SRO

PI BRATISLAVA

PA BAJZOVA 7, BRATISLAVA, 821 08, SLOVAKIA

SN 0028-2685

EI 1338-4317

J9 NEOPLASMA

JI Neoplasma

PY 2018

VL 65

IS 3

BP 376

EP 388

DI 10.4149/neo_2018_170404N245

PG 13

WC Oncology

WE Science Citation Index Expanded (SCI-EXPANDED)

SC Oncology

GA GM3DV

UT WOS:000437979300008

PM 29788733

OA Bronze

DA 2025-04-09

ER

PT J

AU Elmaci, I

Altinoz, MA

AF Elmaci, Ilhan

Altinoz, Meric A.

TI Thymoquinone: An edible redox-active quinone for the pharmacotherapy of

neurodegenerative conditions and glial brain tumors. A short review

SO BIOMEDICINE & PHARMACOTHERAPY

LA English

DT Review

DE Thymoquinone; Glial tumor; Glioblastoma; Neurodegenerative disease;

Oxidative injury

ID OXIDATIVE STRESS; NIGELLA-SATIVA; RAT HIPPOCAMPUS; ISCHEMIA; LEVEL;

MICE; OIL

AB There exist few efficient agents in the neurological and neurosurgical armamentarium for treatment of neurotrauma, refractory seizures and high grade glial tumors. Pathophysiological conditions of diverse neural injuries have converging common pathways including oxidative stress and apoptosis. Targeted therapies have been throughly investigated, but limited success has been achieved until now. Phytochemical drugs may provide easily achievable and cheap adjunctive sources. Thymoquinone is an edible quinone obtained from Nigella sativa seed oil and exerts powerful antiinflammatory, antioxidant and antitumor activities in experimental models. Recently emerging studies conducted with animal models suggest that thymoquinone - bearing a very simple molecular structure - significantly crosses the blood brain barrier and exerts neuromodulatory activities. Indeed, in animal studies, the following actions of thymoquinone were demonstrated: 1-Protection against ischemic brain damage. 2-Reduction of epileptic seizures and associated cerebral oxidative injury. 3-Reduction of morphine tolerance and associated oxidative brain damage. 4-Anxiolytic effects and reduction of immobility stress-associated cerebral oxidative injury. 5-Reduction of diabetes-induced cerebral oxidative stress, 6-Reduction of cerebral oxidative injuries induced by noxious exposures including toluene, lead and ionizing radiation. Substantial in vitro data suggest that thymoquinone may be beneficial in treatment of glial tumors. However, there is no clinical study investigating its antitumor effects. In fact, thymoquinone suppresses growth and invasion, and induces apoptosis of glial tumor cells via degrading tubulins and inhibiting 20S proteasome, telomerase, autophagy, FAK and metalloproteinases. A simple and easily available agent may be a promising adjunctive treatment option in neurological and neurosurgical practice. (C) 2016 Elsevier Masson SAS. All rights reserved.

C1 [Elmaci, Ilhan] Mem Hosp, Dept Neurosurg, Istanbul, Turkey.

[Altinoz, Meric A.] Istanbul Univ, DETAE, Dept Immunol, Istanbul, Turkey.

C3 Memorial Healthcare Group; Istanbul University

RP Altinoz, MA (corresponding author), Guven Sk 5 D 6, Istanbul, Turkey.

EM maltinoz@gmail.com

RI Elmaci, Ilhan/AAF-3459-2021

OI Elmaci, Ilhan/0000-0001-9433-0307; Altinoz, Meric/0000-0001-7804-4087

CR Abdel-Zaher AO, 2013, EUR J PHARMACOL, V702, P62, DOI 10.1016/j.ejphar.2013.01.036

Ahlatci A, 2014, PHYTOMEDICINE, V21, P740, DOI 10.1016/j.phymed.2013.10.023

Al-Majed AA, 2006, EUR J PHARMACOL, V543, P40, DOI 10.1016/j.ejphar.2006.05.046

Alhosin M, 2012, INVEST NEW DRUG, V30, P1813, DOI 10.1007/s10637-011-9734-1

Cecarini V, 2010, FEBS J, V277, P2128, DOI 10.1111/j.1742-4658.2010.07629.x

Dariani S, 2013, J MOL NEUROSCI, V51, P679, DOI 10.1007/s12031-013-0043-3

Gilhotra N, 2011, PHARMACOL REP, V63, P660, DOI 10.1016/S1734-1140(11)70577-1

Gurung RL, 2010, PLOS ONE, V5, DOI 10.1371/journal.pone.0012124

Hamdy NM, 2009, PHARMACOLOGY, V84, P127, DOI 10.1159/000234466

Hosseinzadeh H, 2007, PHYTOMEDICINE, V14, P621, DOI 10.1016/j.phymed.2006.12.005

Kanter M, 2011, J MOL HISTOL, V42, P39, DOI 10.1007/s10735-010-9305-3

Kolli-Bouhafs K, 2012, INVEST NEW DRUG, V30, P2121, DOI 10.1007/s10637-011-9777-3

Racoma IO, 2013, PLOS ONE, V8, DOI 10.1371/journal.pone.0072882

Radad K, 2014, EXP TOXICOL PATHOL, V66, P13, DOI 10.1016/j.etp.2013.07.002

Ullah I, 2015, NEUROMOL MED, V17, P35, DOI 10.1007/s12017-014-8337-3

NR 15

TC 34

Z9 38

U1 0

U2 14

PU ELSEVIER FRANCE-EDITIONS SCIENTIFIQUES MEDICALES ELSEVIER

PI PARIS

PA 23 RUE LINOIS, 75724 PARIS, FRANCE

SN 0753-3322

EI 1950-6007

J9 BIOMED PHARMACOTHER

JI Biomed. Pharmacother.

PD OCT

PY 2016

VL 83

BP 635

EP 640

DI 10.1016/j.biopha.2016.07.018

PG 6

WC Medicine, Research & Experimental; Pharmacology & Pharmacy

WE Science Citation Index Expanded (SCI-EXPANDED)

SC Research & Experimental Medicine; Pharmacology & Pharmacy

GA EF6IC

UT WOS:000390433400076

PM 27459120

DA 2025-04-09

ER

PT J

AU Hambarde, S

Pandey, A

Baskin, D

Helekar, S

AF Hambarde, Shashank

Pandey, Arvind

Baskin, David

Helekar, Santosh

TI ONCOMAGNETIC TREATMENT SELECTIVELY KILLS GLIOMA CANCER CELLS BY INDUCING

OXIDATIVE STRESS AND DNA DAMAGE

SO NEURO-ONCOLOGY

LA English

DT Meeting Abstract

CT 27th Annual Scientific Meeting and Education Day of the

Society-for-Neuro-Oncology (SNO)

CY NOV 16-20, 2022

CL Tampa, FL

SP Soc Neuro Oncol

C1 [Hambarde, Shashank; Pandey, Arvind; Helekar, Santosh] Houston Methodist Res Inst, Houston, TX USA.

[Baskin, David] Houston Methodist Res Inst, Dept Neurosurg, Peak Ctr, Houston, TX USA.

C3 Houston Methodist; Houston Methodist

NR 0

TC 0

Z9 0

U1 0

U2 0

PU OXFORD UNIV PRESS INC

PI CARY

PA JOURNALS DEPT, 2001 EVANS RD, CARY, NC 27513 USA

SN 1522-8517

EI 1523-5866

J9 NEURO-ONCOLOGY

JI Neuro-Oncology

PD NOV

PY 2022

VL 24

SU 7

MA EXTH-68

BP 224

EP 225

PG 2

WC Oncology; Clinical Neurology

WE Science Citation Index Expanded (SCI-EXPANDED); Conference Proceedings Citation Index - Science (CPCI-S)

SC Oncology; Neurosciences & Neurology

GA 6M0ME

UT WOS:000888571001180

DA 2025-04-09

ER

PT J

AU Escoll, M

Lastra, D

Robledinos-Antón, N

Wandosell, F

Antón, IM

Cuadrado, A

AF Escoll, Maribel

Lastra, Diego

Robledinos-Anton, Natalia

Wandosell, Francisco

Anton, Ines Maria

Cuadrado, Antonio

TI WIP Modulates Oxidative Stress through NRF2/KEAP1 in Glioblastoma Cells

SO ANTIOXIDANTS

LA English

DT Article

DE oxidative stress; redox; antioxidants; cytoskeleton

ID TRANSCRIPTION FACTOR NRF2; CUL3-BASED E3 LIGASE; ACTIN CYTOSKELETON;

REACTIVE OXYGEN; STEM-CELLS; N-WASP; PROTEIN; KEAP1; DEGRADATION;

ACTIVATION

AB Due to their high metabolic rate, tumor cells produce exacerbated levels of reactive oxygen species that need to be under control. Wiskott-Aldrich syndrome protein (WASP)-interacting protein (WIP) is a scaffold protein with multiple yet poorly understood functions that participates in tumor progression and promotes cancer cell survival. However, its participation in the control of oxidative stress has not been addressed yet. We show that WIP depletion increases the levels of reactive oxygen species and reduces the levels of transcription factor NRF2, the master regulator of redox homeostasis. We found that WIP stabilizes NRF2 by restraining the activity of its main NRF2 repressor, the E3 ligase adapter KEAP1, because the overexpression of a NRF2(Delta ETGE)mutant that is resistant to targeted proteasome degradation by KEAP1 or the knock-down of KEAP1 maintains NRF2 levels in the absence of WIP. Mechanistically, we show that the increased KEAP1 activity in WIP-depleted cells is not due to the protection of KEAP1 from autophagic degradation, but is dependent on the organization of the Actin cytoskeleton, probably through binding between KEAP1 and F-Actin. Our study provides a new role of WIP in maintaining the oxidant tolerance of cancer cells that may have therapeutic implications.

C1 [Escoll, Maribel; Lastra, Diego; Robledinos-Anton, Natalia; Cuadrado, Antonio] Autonomous Univ Madrid UAM, Med Coll, Dept Biochem, Arzobispo Morcillo 4, Madrid 28029, Spain.

[Escoll, Maribel; Lastra, Diego; Robledinos-Anton, Natalia; Cuadrado, Antonio] UAM, CSIC, Inst Invest Biomed Alberto Sols, Arturo Duperier 4, Madrid 28029, Spain.

[Escoll, Maribel; Lastra, Diego; Robledinos-Anton, Natalia; Cuadrado, Antonio] Inst Invest Sanitaria La Paz IdiPaz, Pedro Rico 6, Madrid 28029, Spain.

[Escoll, Maribel; Lastra, Diego; Robledinos-Anton, Natalia; Wandosell, Francisco; Anton, Ines Maria; Cuadrado, Antonio] Ctr Invest Biomed Red Enfermedades Neurodegenerat, Valderrebollo 5, Madrid 28049, Spain.

[Wandosell, Francisco] Univ Autonoma Madrid, UAM, CSIC, Ctr Biol Mol Severo Ochoa, Nicolas Cabrera 1, Madrid 28049, Spain.

[Anton, Ines Maria] CSIC, Dept Cellular & Mol Biol, CNB, Ctr Nacl Biotecnol, Darwin 3, Madrid 28049, Spain.

C3 Autonomous University of Madrid; Consejo Superior de Investigaciones

Cientificas (CSIC); CSIC - Instituto de Investigaciones Biomedicas

Alberto Sols (IIBM); Autonomous University of Madrid; CIBERNED;

Autonomous University of Madrid; Consejo Superior de Investigaciones

Cientificas (CSIC); CSIC - Centro de Biologia Molecular Severo Ochoa

(CBM); Consejo Superior de Investigaciones Cientificas (CSIC); CSIC -

Centro Nacional de Biotecnologia (CNB)

RP Cuadrado, A (corresponding author), Autonomous Univ Madrid UAM, Med Coll, Dept Biochem, Arzobispo Morcillo 4, Madrid 28029, Spain.; Cuadrado, A (corresponding author), UAM, CSIC, Inst Invest Biomed Alberto Sols, Arturo Duperier 4, Madrid 28029, Spain.; Cuadrado, A (corresponding author), Inst Invest Sanitaria La Paz IdiPaz, Pedro Rico 6, Madrid 28029, Spain.; Cuadrado, A (corresponding author), Ctr Invest Biomed Red Enfermedades Neurodegenerat, Valderrebollo 5, Madrid 28049, Spain.

EM mescoll@iib.uam.es; diegolastra@iib.uam.es; nrobledinos@iib.uam.es;

fwandosell@cbm.csic.es; ianton@cnb.csic.es; antonio.cuadrado@uam.es

RI Wandosell, Francisco/AAV-6391-2020; Anton, Ines/G-6090-2015; Wandosell,

Francisco/G-6105-2015

OI Wandosell, Francisco/0000-0001-6537-2321; Cuadrado,

Antonio/0000-0002-4039-7140

FU Spanish Ministry of Economy and Competiveness [PID2019-110061RB-I00,

RTI2018-096303-B-C31, SAF2017-82436R]; European Regional Development

Fund, Competitiveness Operational Program 2014-2020 [P_37_732/2016];

Comunidad Autonoma de Madrid [B2017/BMD-3827]; Juan de la Cierva; MINECO

FX This work was supported by PID2019-110061RB-I00, RTI2018-096303-B-C31

and SAF2017-82436R of the Spanish Ministry of Economy and Competiveness;

and by the P_37_732/2016 grant (REDBRAIN) financed by the European

Regional Development Fund, Competitiveness Operational Program

2014-2020, and Comunidad Autonoma de Madrid (grant B2017/BMD-3827). M.E.

was recipient of a postdoctoral contract Juan de la Cierva; D.L. and

N.R.-A. enjoied a FPU contract of MINECO.

CR Antón IM, 2006, EUR J CELL BIOL, V85, P295, DOI 10.1016/j.ejcb.2005.08.004

Antón IM, 2002, IMMUNITY, V16, P193, DOI 10.1016/S1074-7613(02)00268-6

Antón IM, 2007, TRENDS CELL BIOL, V17, P555, DOI 10.1016/j.tcb.2007.08.005

Banon-Rodriguez I, 2013, PLOS ONE, V8, DOI 10.1371/journal.pone.0070364

Cairns RA, 2011, NAT REV CANCER, V11, P85, DOI 10.1038/nrc2981

Chiang AC, 2008, NEW ENGL J MED, V359, P2814, DOI 10.1056/NEJMra0805239

Chou HC, 2006, CURR BIOL, V16, P2337, DOI 10.1016/j.cub.2006.10.037

Cuadrado A, 2019, NAT REV DRUG DISCOV, V18, P295, DOI 10.1038/s41573-018-0008-x

Cuadrado A, 2018, PHARMACOL REV, V70, P348, DOI 10.1124/pr.117.014753

Cuadrado A, 2015, FREE RADICAL BIO MED, V88, P147, DOI 10.1016/j.freeradbiomed.2015.04.029

Cullinan SB, 2004, MOL CELL BIOL, V24, P8477, DOI 10.1128/MCB.24.19.8477-8486.2004

Diaz B., 2009, SCI SIGNAL, V2

Escoll M, 2017, ONCOGENE, V36, P3515, DOI 10.1038/onc.2016.518

Escoll M, 2020, REDOX BIOL, V30, DOI 10.1016/j.redox.2019.101425

Fan WL, 2010, AUTOPHAGY, V6, P614, DOI 10.4161/auto.6.5.12189

Fedorova M, 2010, J PROTEOME RES, V9, P1598, DOI 10.1021/pr901099e

García E, 2012, EUR J CELL BIOL, V91, P869, DOI 10.1016/j.ejcb.2012.06.002

Gargini R, 2016, CELL REP, V17, P1962, DOI 10.1016/j.celrep.2016.10.064

Gorrini C, 2013, NAT REV DRUG DISCOV, V12, P931, DOI 10.1038/nrd4002

Haider N, 2020, CELL COMMUN SIGNAL, V18, DOI 10.1186/s12964-019-0506-4

Jain A, 2010, J BIOL CHEM, V285, P22576, DOI 10.1074/jbc.M110.118976

Jaramillo MC, 2013, GENE DEV, V27, P2179, DOI 10.1101/gad.225680.113

Kanamori M, 2015, NEURO-ONCOLOGY, V17, P555, DOI 10.1093/neuonc/nou282

Kang MI, 2004, P NATL ACAD SCI USA, V101, P2046, DOI 10.1073/pnas.0308347100

Kitamura H, 2018, CANCER SCI, V109, P900, DOI 10.1111/cas.13537

Kobayashi A, 2004, MOL CELL BIOL, V24, P7130, DOI 10.1128/MCB.24.16.7130-7139.2004

Komatsu M, 2010, NAT CELL BIOL, V12, P213, DOI 10.1038/ncb2021

Lee PP, 2017, NAT COMMUN, V8, DOI 10.1038/s41467-017-01676-0

Lehtimäki J, 2017, HANDB EXP PHARMACOL, V235, P123, DOI 10.1007/164_2016_28

Li XC, 2020, INT J BIOL SCI, V16, P2014, DOI 10.7150/ijbs.44943

MANDERS EMM, 1993, J MICROSC-OXFORD, V169, P375, DOI 10.1111/j.1365-2818.1993.tb03313.x

Martinez-Quiles N, 2001, NAT CELL BIOL, V3, P484, DOI 10.1038/35074551

Menegon S, 2016, TRENDS MOL MED, V22, P578, DOI 10.1016/j.molmed.2016.05.002

Moldogazieva NT, 2018, CANCER RES, V78, P6040, DOI 10.1158/0008-5472.CAN-18-0980

Moon DO, 2010, CANCER LETT, V288, P204, DOI 10.1016/j.canlet.2009.07.002

Pajares M, 2018, AUTOPHAGY, V14, P1310, DOI 10.1080/15548627.2018.1474992

Pajares M, 2016, AUTOPHAGY, V12, P1902, DOI 10.1080/15548627.2016.1208889

Pan Y, 2018, J EXP CLIN CANC RES, V37, DOI 10.1186/s13046-018-0848-6

Pani G, 2010, CANCER METAST REV, V29, P351, DOI 10.1007/s10555-010-9225-4

Panieri E, 2013, FREE RADICAL BIO MED, V57, P176, DOI 10.1016/j.freeradbiomed.2012.12.024

Rada P, 2011, MOL CELL BIOL, V31, P1121, DOI 10.1128/MCB.01204-10

Robledinos-Antón N, 2017, REDOX BIOL, V13, P393, DOI 10.1016/j.redox.2017.06.010

Rojo AI, 2010, GLIA, V58, P588, DOI 10.1002/glia.20947

Rotty JD, 2013, NAT REV MOL CELL BIO, V14, P7, DOI 10.1038/nrm3492

Seetharaman S, 2020, TRENDS CELL BIOL, V30, P720, DOI 10.1016/j.tcb.2020.06.004

Sokolik CG, 2020, BIOMOLECULES, V10, DOI 10.3390/biom10071084

Staub E, 2009, J MOL MED, V87, P633, DOI 10.1007/s00109-009-0467-y

Tang Z, 2013, PLOS ONE, V8, DOI 10.1371/journal.pone.0075436

Tebay LE, 2015, FREE RADICAL BIO MED, V88, P108, DOI 10.1016/j.freeradbiomed.2015.06.021

van der Kammen R, 2017, DEVELOPMENT, V144, P4588, DOI 10.1242/dev.156323

Velichkova M, 2003, CELL MOTIL CYTOSKEL, V56, P109, DOI 10.1002/cm.10138

Vurusaner B, 2012, FREE RADICAL BIO MED, V52, P7, DOI 10.1016/j.freeradbiomed.2011.09.035

Weinberg F, 2010, P NATL ACAD SCI USA, V107, P8788, DOI 10.1073/pnas.1003428107

Yu WD, 2020, CELL DEATH DIS, V11, DOI 10.1038/s41419-020-2701-z

Ziv-Av A, 2015, ONCOTARGET, V6, P19826, DOI 10.18632/oncotarget.4471

NR 55

TC 5

Z9 6

U1 1

U2 11

PU MDPI

PI BASEL

PA ST ALBAN-ANLAGE 66, CH-4052 BASEL, SWITZERLAND

EI 2076-3921

J9 ANTIOXIDANTS-BASEL

JI Antioxidants

PD SEP

PY 2020

VL 9

IS 9

AR 773

DI 10.3390/antiox9090773

PG 13

WC Biochemistry & Molecular Biology; Chemistry, Medicinal; Food Science &

Technology

WE Science Citation Index Expanded (SCI-EXPANDED)

SC Biochemistry & Molecular Biology; Pharmacology & Pharmacy; Food Science

& Technology

GA OD7NG

UT WOS:000580035200001

PM 32825452

OA gold, Green Published

DA 2025-04-09

ER

PT J

AU Gökçek-Saraç, Ç

Simsek, T

Karakurt, S

AF Gokcek-Sarac, Cigdem

Simsek, Tugce

Karakurt, Serdar

TI Cytoprotective effects of low-frequency pulsed electromagnetic field

against oxidative stress in glioblastoma cells

SO GENERAL PHYSIOLOGY AND BIOPHYSICS

LA English

DT Article

DE Pulsed electromagnetic field; U87-MG cells; Oxidative stress; Reactive

oxygen species; Antioxidants

ID ELF-MF; EXPOSURE; HZ; APOPTOSIS; PEMF

AB The low-frequency pulsed electromagnetic field (PEMF) may have possible cytoprotec-tive effects against the destructive effects of oxidative stress. The goal was to investigate if short-term low-frequency PEMF has cytoprotective effects in glioblastoma cell line following high-dose hydrogen peroxide (H2O2) treatment. U87-MG cells were divided into four groups: Sham-control group; PEMF group (cells exposed to PEMF); H2O2 group (cells treated with H2O2 at time intervals 30 min and 48 h, respectively); H2O2+PEMF group (cells exposed to PEMF after H2O2 treatment at time intervals 30 min and 48 h, respectively). The cell viability, levels of reactive oxygen species, glutathione peroxidase activity, and the amount of glutathione were measured. The cytoprotective effect of PEMF against deleterious effects of oxidative stress triggered by different time interval of H2O2 treatment might be mediated by the increase in the cell viability, the elevation in the antioxi-dant enzyme activity/amount, and the decrease in the reactive oxygen species level. In addition, the cytoprotective effect of PEMF varies depending on different time intervals of H2O2 treatment. In the light of these findings, further in vivo and/or in vitro studies on neurophysiological effects of PEMFs and their underlying molecular mechanisms are needed to elucidate neurotoxic or neuroprotective role against antioxidant defense mechanisms.

C1 [Gokcek-Sarac, Cigdem; Simsek, Tugce] Akdeniz Univ, Fac Engn, Dept Biomed Engn, Antalya, Turkiye.

[Karakurt, Serdar] Selcuk Univ, Fac Sci, Dept Biochem, Antalya, Turkiye.

C3 Akdeniz University; Selcuk University

RP Gökçek-Saraç, Ç (corresponding author), Akdeniz Univ, Fac Engn, Dept Biomed Engn, TR-07058 Antalya, Turkiye.

EM gokcekcigdem@gmail.com

RI Gokcek-Sarac, Cigdem/HJP-3652-2023

FU Scientific Research Projects Coordination Unit of Akdeniz University,

Turkey [FBA-2020-5388]

FX Funding. This work was supported by The Scientific Research Projects

Coordination Unit of Akdeniz University, Turkey (Grant Number:

FBA-2020-5388) .

CR Ahmadi-Zeidabadi M, 2019, ELECTROMAGN BIOL MED, V38, P198, DOI 10.1080/15368378.2019.1625784

Akbarnejad Z, 2017, ELECTROMAGN BIOL MED, V36, P238, DOI 10.1080/15368378.2016.1251452

Akdag MZ, 2013, INT J RADIAT BIOL, V89, P1053, DOI 10.3109/09553002.2013.817705

Azad Meghan B., 2014, Terminology of European education and training policy: a selection of 130 key terms. 2nd ed. Luxembourg: Publications Office, V4, P195, DOI 10.4161/auto.5278

Capone F, 2017, SCI REP-UK, V7, DOI 10.1038/s41598-017-12371-x

Ceccarelli G, 2013, BIORESEARCH OPEN ACC, V2, P283, DOI 10.1089/biores.2013.0016

Chen YX, 2020, CELL PROLIFERAT, V53, DOI 10.1111/cpr.12781

Dong S., Environmental Pollution, V159, P212, DOI [10.1016/j.envpol.2010.09.004, DOI 10.1016/J.ENVPOL.2021.116843]

Ehnert S, 2019, J CLIN MED, V8, DOI 10.3390/jcm8122028

Ehnert S, 2017, SCI REP-UK, V7, DOI 10.1038/s41598-017-14983-9

Falone S, 2008, INT J BIOCHEM CELL B, V40, P2762, DOI 10.1016/j.biocel.2008.05.022

Falone S, 2016, INT J RADIAT BIOL, V92, P281, DOI 10.3109/09553002.2016.1150619

Gessi S, 2019, J CELL PHYSIOL, V234, P15089, DOI 10.1002/jcp.28149

Hug K, 2012, BIOELECTROMAGNETICS, V33, P95, DOI 10.1002/bem.20703

Ighodaro OM, 2018, ALEX J MED, V54, P287, DOI 10.1016/j.ajme.2017.09.001

Iwasa K, 2018, TISSUE ENG PART B-RE, V24, P144, DOI [10.1089/ten.teb.2017.0294, 10.1089/ten.TEB.2017.0294]

Kang KA, 2014, J RADIAT RES, V55, P265, DOI 10.1093/jrr/rrt116

Karaman O, 2018, INT J APPL ELECTROM, V57, P427, DOI 10.3233/JAE-170129

Kazlauskaite Agne, 2014, CHAOS SOLITONS AMP F, V4, P130213, DOI [10.1098/rsob.130213, DOI 10.1016/J.CHAOS.2019.109405]

Kim GH, 2015, EXP NEUROBIOL, V24, P325, DOI 10.5607/en.2015.24.4.325

Kim JH, 2019, BIOMOL THER, V27, P265, DOI 10.4062/biomolther.2018.152

Kinnula VL, 2004, FEBS LETT, V569, P1, DOI 10.1016/j.febslet.2004.05.045

Kivrak Elfide Gizem, 2017, J Microsc Ultrastruct, V5, P167, DOI 10.1016/j.jmau.2017.07.003

Kohli H, 2022, IETE J RES, V68, P2723, DOI 10.1080/03772063.2020.1725661

Kohli Himani, 2020, TRANSL NEUROSCI, V68, P2723, DOI [DOI https://doi.org/10.1080/00131857.2020.1759194, 10.1080/03772063.2020.1725661, DOI 10.1515/TNSCI-2020-0007]

Konyalioglu S, 2013, NEURAL REGEN RES, V8, P485, DOI 10.3969/j.issn.1673-5374.2013.06.001

Li Meng, 2016, ANTIOXIDANTS-BASEL, V17, P332, DOI [10.3390/ijms17030332, DOI 10.3390/ANTIOX9070597]

Makinistian L, 2019, BMC CANCER, V19, DOI 10.1186/s12885-019-5376-z

Makinistian Leonardo, 2019, BMC CANCER, V19, DOI [10.1186/s12885-019-5376-z, DOI 10.1186/S12885-019-5376-Z]

Martínez-Sámano J, 2012, ARCH MED RES, V43, P183, DOI 10.1016/j.arcmed.2012.04.003

McLean KateC., 2015, The Oxford handbook of identity development

Nadin SB, 2001, J HISTOCHEM CYTOCHEM, V49, P1183, DOI 10.1177/002215540104900912

Omuro A, 2013, JAMA-J AM MED ASSOC, V310, P1842, DOI 10.1001/jama.2013.280319

Osera C, 2015, BIOELECTROMAGNETICS, V36, P219, DOI 10.1002/bem.21900

Osera Cecilia, 2015, BIOELECTROMAGNETICS, V36, P219, DOI [10.1002/bem.21900, DOI 10.1002/BEM.21900]

Palumbo R, 2006, BIOELECTROMAGNETICS, V27, P159, DOI 10.1002/bem.20199

Palumbo R., 2006, BIOELECTROMAGNETICS, V27, P159, DOI [10.1002/bem.20199, DOI 10.1002/BEM.20199]

Pi Y, 2019, MOL MED REP, V19, P4129, DOI 10.3892/mmr.2019.10079

Ranamukhaarachchi S. A., 2016, SCI REP-UK, V6, DOI [10.1038/srep32074, DOI 10.1038/SREP32074, 10.1038/nature03120, DOI 10.1038/S41388-020-01591-7]

Sakawa Hideaki, 2022, Coastal Engineering Proceedings, V54, P5373, DOI [10.1080/00036846.2022.2044996, DOI 10.9753/ICCE.V17.8, 10.15560/10.2.453]

Salazar-Ramiro A, 2016, FRONT IMMUNOL, V7, DOI 10.3389/fimmu.2016.00156

Salazar-Ramiro Aleli, 2016, CURR NEUROL NEUROSCI, V7, DOI [10.3389/fimmu.2016.00156, DOI 10.1007/S11910-012-0282-7, 10.3389/fpls.2021.700928, DOI 10.1038/S41522-020-00155-7]

Sepehrimanesh M., 2016, Online Journal of Veterinary Research, V20, P617

SU C, 2019, MOL MED REP, DOI DOI 10.3892/MMR10.3892/MMR.2019.10667

Sulpizio M, 2011, J CELL BIOCHEM, V112, P3797, DOI 10.1002/jcb.23310

Tsai YR, 2018, INT J MOL SCI, V19, DOI 10.3390/ijms19103252

Veronesi F, 2014, J ORTHOP RES, V32, P677, DOI 10.1002/jor.22584

Vincenzi F, 2017, J CELL PHYSIOL, V232, P1200, DOI 10.1002/jcp.25606

Vincenzi F, 2013, PLOS ONE, V8, DOI 10.1371/journal.pone.0065561

Vincenzi Fabrizio, 2016, J CELL PHYSIOL, V232, P1200, DOI [10.1002/jcp.25606, DOI 10.1002/JCP.27782]

Zastko L, 2020, BIOELECTROMAGNETICS, V41, P649, DOI 10.1002/bem.22302

Zhang LX, 2019, REDOX BIOL, V26, DOI 10.1016/j.redox.2019.101284

Zhao Fei, 2015, HDB SPORTS MED SCI, V27, P4351, DOI [10.1002/adma.201501867, DOI 10.1002/9781119227045.CH8]

Zhao XY, 2001, AM J PHYSIOL-LUNG C, V281, pL879, DOI 10.1152/ajplung.2001.281.4.L879

NR 54

TC 6

Z9 6

U1 2

U2 12

PU AEPRESS SRO

PI BRATISLAVA

PA BAJZOVA 7, BRATISLAVA, 821 08, SLOVAKIA

SN 0231-5882

EI 1338-4325

J9 GEN PHYSIOL BIOPHYS

JI Gen. Physiol. Biophys.

PY 2023

VL 42

IS 1

BP 97

EP 106

DI 10.4149/gpb_2022056

PG 10

WC Biochemistry & Molecular Biology; Biophysics; Physiology

WE Science Citation Index Expanded (SCI-EXPANDED)

SC Biochemistry & Molecular Biology; Biophysics; Physiology

GA J8NZ4

UT WOS:001012150000010

PM 36705309

OA gold

DA 2025-04-09

ER

PT J

AU Youn, P

Chen, YZ

Furgeson, DY

AF Youn, Pilju

Chen, Yizhe

Furgeson, Darin Y.

TI Cytoprotection against beta-amyloid (Aβ) peptide-mediated oxidative

damage and autophagy by Keap1 RNAi in human glioma U87mg cells

SO NEUROSCIENCE RESEARCH

LA English

DT Article

DE Neurodegenerative disorders (NDD); Oxidative stress; Keapl-Nrf2 pathway;

RNA interference (RNAi); Beta-amyloid (A beta) peptide; Autophagy

ID VITAMIN-E SUPPLEMENTATION; ALZHEIMERS-DISEASE; NRF2; DEPOSITION;

MEDICINE; PATHWAY; TARGET; DEATH; SIRNA; LC3

AB Extensive oxidative stress has been considered a primary pathological factor for many neurodegenerative disorders (NDDs). We speculated that the oxidative damage to brain cells can be managed by promoting the endogenous cellular antioxidants through the RNA interference (RNAi) against Keap1 (kelch-like ECH-associated protein). Keap1 acts as a negative regulator of Nrf2 (NF-E2-related factor 2) that represses the activation of the antioxidant responsive element (ARE). Here, we investigated whether Keap1 knockdown enhances the cellular antioxidant capacity and provides the neuroprotection against oxidative stress from hydrogen peroxide and beta-amyloid (A beta) peptide in U87mg cells. We found that the Keap1 siRNA pre-treated group displayed higher expression of diverse antioxidant genes and an increased antioxidant capacity compared to the control group. Moreover, the Keap1 RNAi exerted a cytoprotective effect against H2O2 treatment. In A beta peptide treatment experiments, the Keap1 siRNA pre-treated groups maintained acceptable cell viability, relatively intact cellular morphology, and controlled oxidative damage levels while the control groups suffered from A beta peptide-mediated neurotoxicity. Keap1 RNAi also attenuated the oxidative stress-mediated autophagy as well. These findings suggest that Keap1 RNAi can serve as a therapeutic strategy for relieving oxidative stress-associated symptoms in many NDDs. (C) 2015 Elsevier Ireland Ltd and the Japan Neuroscience Society. All rights reserved.

C1 [Youn, Pilju; Chen, Yizhe; Furgeson, Darin Y.] Univ Utah, Dept Pharmaceut & Pharmaceut Chem, Salt Lake City, UT 84112 USA.

C3 Utah System of Higher Education; University of Utah

RP Furgeson, DY (corresponding author), Univ Utah, Dept Pharmaceut & Pharmaceut Chem, Salt Lake City, UT 84112 USA.

EM darin.furgeson@gmail.com

FU University of Utah start-up funds

FX We appreciate the financial support from University of Utah start-up

funds.

CR Andersen JK, 2004, NAT MED, V10, pS18, DOI 10.1038/nrn1434

Barnham KJ, 2004, NAT REV DRUG DISCOV, V3, P205, DOI 10.1038/nrd1330

BEHL C, 1994, CELL, V77, P817, DOI 10.1016/0092-8674(94)90131-7

Boudreau RL, 2011, HUM MOL GENET, V20, pR21, DOI 10.1093/hmg/ddr137

Calkins MJ, 2009, ANTIOXID REDOX SIGN, V11, P497, DOI [10.1089/ars.2008.2242, 10.1089/ARS.2008.2242]

Chen Y, 2008, CELL DEATH DIFFER, V15, P171, DOI 10.1038/sj.cdd.4402233

de Vries HE, 2008, FREE RADICAL BIO MED, V45, P1375, DOI 10.1016/j.freeradbiomed.2008.09.001

Devling TWP, 2005, P NATL ACAD SCI USA, V102, P7280, DOI 10.1073/pnas.0501475102

Drake J, 2003, NEUROBIOL AGING, V24, P415, DOI 10.1016/S0197-4580(02)00225-7

DYRKS T, 1992, J BIOL CHEM, V267, P18210

Dysken MW, 2014, JAMA-J AM MED ASSOC, V311, P33, DOI 10.1001/jama.2013.282834

Hara T, 2006, NATURE, V441, P885, DOI 10.1038/nature04724

Hardy J, 2002, SCIENCE, V297, P353, DOI 10.1126/science.1072994

Itoh K, 1997, BIOCHEM BIOPH RES CO, V236, P313, DOI 10.1006/bbrc.1997.6943

Jung KA, 2013, OXID MED CELL LONGEV, V2013, DOI 10.1155/2013/423965

Kabeya Y, 2000, EMBO J, V19, P5720, DOI 10.1093/emboj/19.21.5720

Kobayashi A, 2004, MOL CELL BIOL, V24, P7130, DOI 10.1128/MCB.24.16.7130-7139.2004

Li WG, 2006, J BIOL CHEM, V281, P27251, DOI 10.1074/jbc.M602746200

Melo A, 2011, OXID MED CELL LONGEV, V2011, DOI 10.1155/2011/467180

Miller ER, 2005, ANN INTERN MED, V142, P37, DOI 10.7326/0003-4819-142-1-200501040-00110

Mizushima N, 2007, AUTOPHAGY, V3, P542, DOI 10.4161/auto.4600

MORTIMORE GE, 1977, NATURE, V270, P174, DOI 10.1038/270174a0

Nixon RA, 2005, J NEUROPATH EXP NEUR, V64, P113, DOI 10.1093/jnen/64.2.113

Ramsey CP, 2007, J NEUROPATH EXP NEUR, V66, P75, DOI 10.1097/nen.0b013e31802d6da9

Salloway S, 2014, NEW ENGL J MED, V370, P322, DOI 10.1056/NEJMoa1304839

Sung S, 2004, FASEB J, V18, DOI 10.1096/fj.03-0961fje

Thies W, 2013, ALZHEIMERS DEMENT, V9, P208, DOI 10.1016/j.jalz.2013.02.003

Wakabayashi N, 2003, NAT GENET, V35, P238, DOI 10.1038/ng1248

Wang HM, 2010, J ALZHEIMERS DIS, V21, P597, DOI 10.3233/JAD-2010-091207

Yatin SM, 1999, NEUROBIOL AGING, V20, P325

Youn P, 2014, MOL PHARMACEUT, V11, P486, DOI 10.1021/mp400446v

Yu WH, 2005, J CELL BIOL, V171, P87, DOI 10.1083/jcb.200505082

Zhang HY, 2009, TOXICOL SCI, V110, P376, DOI 10.1093/toxsci/kfp101

NR 33

TC 7

Z9 9

U1 0

U2 12

PU ELSEVIER IRELAND LTD

PI CLARE

PA ELSEVIER HOUSE, BROOKVALE PLAZA, EAST PARK SHANNON, CO, CLARE, 00000,

IRELAND

SN 0168-0102

EI 1872-8111

J9 NEUROSCI RES

JI Neurosci. Res.

PD MAY

PY 2015

VL 94

BP 70

EP 78

DI 10.1016/j.neures.2014.12.015

PG 9

WC Neurosciences

WE Science Citation Index Expanded (SCI-EXPANDED)

SC Neurosciences & Neurology

GA CH9FM

UT WOS:000354341400008

PM 25612817

DA 2025-04-09

ER

PT J

AU Yulyana, Y

Tovmasyan, A

Ho, IAW

Sia, KC

Newman, JP

Ng, WH

Guo, CM

Hui, KM

Batinic-Haberle, I

Lam, PYP

AF Yulyana, Yulyana

Tovmasyan, Artak

Ho, Ivy A. W.

Sia, Kian Chuan

Newman, Jennifer P.

Ng, Wai Hoe

Guo, Chang Ming

Hui, Kam Man

Batinic-Haberle, Ines

Lam, Paula Y. P.

TI Redox-Active Mn Porphyrin-based Potent SOD Mimic,

MnTnBuOE-2-PyP<SUP>5+</SUP>, Enhances Carbenoxolone-Mediated

TRAIL-Induced Apoptosis in Glioblastoma Multiforme

SO STEM CELL REVIEWS AND REPORTS

LA English

DT Article

DE Carbenoxolone; TRAIL-modified human mesenchymal stem cells; Manganese

porphyrin; Glioma; SOD mimic; Ascorbate; NAC

ID MESENCHYMAL STEM-CELLS; MANGANESE SUPEROXIDE-DISMUTASE; DEATH RECEPTOR

5; HYDROGEN-SULFIDE; OXIDATIVE STRESS; UP-REGULATION; CANCER-CELLS; P38

MAPK; INHIBITION; PROOXIDANT

AB Glioblastoma multiforme is the most malignant tumor of the brain and is challenging to treat due to its highly invasive nature and heterogeneity. Malignant brain tumor displays high metabolic activity which perturbs its redox environment and in turn translates to high oxidative stress. Thus, pushing the oxidative stress level to achieve the maximum tolerable threshold that induces cell death is a potential strategy for cancer therapy. Previously, we have shown that gap junction inhibitor, carbenoxolone (CBX), is capable of enhancing tumor necrosis factor-related apoptosis-inducing ligand (TRAIL) -induced apoptosis in glioma cells. Since CBX is known to induce oxidative stress, we hypothesized that the addition of another potent mediator of oxidative stress, powerful SODmimicMnTnBuOE-2-PyP5+ (MnBuOE), could further enhance TRAIL-driven therapeutic efficacy in glioma cells. Our results showed that combining TRAIL + CBX with MnBuOE significantly enhances cell death of glioma cell lines and this enhancement could be further potentiated by CBX pretreatment. MnBuOE-driven cytotoxicity is due to its ability to take advantage of oxidative stress imposed by CBX + TRAIL system, and enhance it in the presence of endogenous reductants, ascorbate and thiol, thereby producing cytotoxic H2O2, and in turn inducing death of glioma cells but not normal astrocytes. Most importantly, combination treatment significantly reduces viability of TRAIL-resistant Asian patient-derived glioma cells, thus demonstrating the potential clinical use of our therapeutic system. It was reported that H2O2 is involved in membrane depolarization-based sensitization of cancer cells toward TRAIL. MnBuOE is entering Clinical Trials as a normal brain radioprotector in glioma patients at Duke University increasing Clinical relevance of our studies.

C1 [Yulyana, Yulyana; Ho, Ivy A. W.; Sia, Kian Chuan; Newman, Jennifer P.; Lam, Paula Y. P.] Natl Canc Ctr, Humphrey Oei Inst Canc Res, Cellular & Mol Res Div, Lab Canc Gene Therapy, 11 Hosp Dr, Singapore 169610, Singapore.

[Tovmasyan, Artak; Batinic-Haberle, Ines] Duke Univ, Med Ctr, Dept Radiat Oncol, Res Dr 281b-285 MSRB I,Box 3455, Durham, NC 27710 USA.

[Ng, Wai Hoe] Natl Inst Neurosci, Dept Neurosurg, Singapore, Singapore.

[Guo, Chang Ming] Singapore Gen Hosp, Dept Orthoped, Singapore, Singapore.

[Hui, Kam Man] Natl Canc Ctr Singapore, Humphrey Oei Inst Canc Res, Cellular & Mol Res Div, Bek Chai Heah Lab Canc Genom, Singapore, Singapore.

[Hui, Kam Man; Lam, Paula Y. P.] Duke NUS Grad Med Sch, Canc & Stem Cells Biol Program, Singapore, Singapore.

[Hui, Kam Man] Natl Univ Singapore, Yong Loo Lin Sch Med, Dept Biochem, Singapore 117595, Singapore.

[Hui, Kam Man] ASTAR, Inst Mol & Cell Biol, Proteos, Singapore.

[Batinic-Haberle, Ines] Duke Univ, Med Ctr, Duke Canc Inst, Durham, NC USA.

[Lam, Paula Y. P.] Natl Univ Singapore, Yong Loo Lin Sch Med, Dept Physiol, Singapore 117595, Singapore.

[Ho, Ivy A. W.] Natl Inst Neurosci, Singapore, Singapore.

[Sia, Kian Chuan] Natl Univ Singapore, Singapore, Singapore.

C3 National Cancer Centre Singapore (NCCS); Duke University; National

Neuroscience Institute (NNI); Singapore General Hospital; National

Cancer Centre Singapore (NCCS); National University of Singapore;

National University of Singapore; Agency for Science Technology &

Research (A*STAR); A*STAR - Institute of Molecular & Cell Biology

(IMCB); Duke University; National University of Singapore; National

Neuroscience Institute (NNI); National University of Singapore

RP Lam, PYP (corresponding author), Natl Canc Ctr, Humphrey Oei Inst Canc Res, Cellular & Mol Res Div, Lab Canc Gene Therapy, 11 Hosp Dr, Singapore 169610, Singapore.; Batinic-Haberle, I (corresponding author), Duke Univ, Med Ctr, Dept Radiat Oncol, Res Dr 281b-285 MSRB I,Box 3455, Durham, NC 27710 USA.; Lam, PYP (corresponding author), Duke NUS Grad Med Sch, Canc & Stem Cells Biol Program, Singapore, Singapore.; Batinic-Haberle, I (corresponding author), Duke Univ, Med Ctr, Duke Canc Inst, Durham, NC USA.; Lam, PYP (corresponding author), Natl Univ Singapore, Yong Loo Lin Sch Med, Dept Physiol, Singapore 117595, Singapore.

EM ibatinic@duke.edu; cmrlyp@nccs.com.sg

RI Sia, Kian/AAI-4036-2021; Ho, Ivy/T-4396-2019; Ming, Guo-li/J-7880-2013;

Tovmasyan, Artak/D-9101-2014; Hui, Kam/C-6186-2011

OI Ho, Ivy/0000-0002-2948-3726; Hui, Kam/0000-0003-1820-1399; Sia, Kian

Chuan/0000-0002-2729-6345

FU National Cancer Centre Research Funds; National Medical Research

Council, Singapore; NIH [1R03-NS082704-01]; BioMimetix JV LLC; Duke

University School of Medicine-National University of Singapore

FX We would like to thank the Food Industry Research and Development

Institute, Bioresource Collection and Research Center for providing

primary GBM lines, GBM8401, GBM8901 and G5T/VGH. This research is

supported by institutional fund supports from the National Cancer Centre

Research Funds and the National Medical Research Council, Singapore. Dr.

Batinic-Haberle and Dr. Tovmasyan are grateful for the support from NIH

1R03-NS082704-01 and BioMimetix JV LLC. Dr. Batinic-Haberle acknowledges

Duke University School of Medicine-National University of Singapore

travel grant.

CR Akita M, 2014, INT J ONCOL, V45, P1901, DOI 10.3892/ijo.2014.2608

Ali DK, 2013, REDOX BIOL, V1, P457, DOI 10.1016/j.redox.2013.09.005

[Anonymous], 58 ANN M RAD RES SOC

Ashcraft K. A., 2015, INT J RAD ONCOLOGY B

Azarashvili T, 2011, AM J PHYSIOL-CELL PH, V300, pC707, DOI 10.1152/ajpcell.00061.2010

Bangert A, 2012, ONCOGENE, V31, P4677, DOI 10.1038/onc.2011.614

Batinic-Haberle I, 2015, REDOX BIOL, V5, P43, DOI 10.1016/j.redox.2015.01.017

Batinic-Haberle I, 2014, ANTIOXID REDOX SIGN, V20, P2372, DOI 10.1089/ars.2012.5147

Batinic-Haberle I, 2012, AMINO ACIDS, V42, P95, DOI 10.1007/s00726-010-0603-6

Bhuiyan AI, 2015, MOLECULES, V20, P1731, DOI 10.3390/molecules20011731

Das A, 2002, J NEURO-ONCOL, V60, P117, DOI 10.1023/A:1020622415786

Evans MK, 2014, FREE RADICAL BIO MED, V68, P302, DOI 10.1016/j.freeradbiomed.2013.11.031

Finn NA, 2012, MOL BIOSYST, V8, P650, DOI 10.1039/c1mb05315a

Halliwell B., 2000, FREE RADICAL BIO MED, V3rd

Hempel N, 2011, ANTI-CANCER AGENT ME, V11, P191, DOI 10.2174/187152011795255911

Hingtgen S, 2008, MOL CANCER THER, V7, P3575, DOI 10.1158/1535-7163.MCT-08-0640

Ho IAW, 2008, CANCER GENE THER, V15, P553, DOI 10.1038/cgt.2008.27

Ho IAW, 2013, HISTOL HISTOPATHOL, V28, P1427, DOI 10.14670/HH-28.1427

Holley AK, 2014, ANTIOXID REDOX SIGN, V20, P2347, DOI 10.1089/ars.2013.5204

HOLZER T, 1993, J COMPUT ASSIST TOMO, V17, P681

Jaramillo MC, 2015, FREE RADICAL BIO MED, V83, P89, DOI 10.1016/j.freeradbiomed.2015.01.031

Jaramillo MC, 2012, FREE RADICAL BIO MED, V52, P1272, DOI 10.1016/j.freeradbiomed.2012.02.001

Jaramillo MC, 2009, CANCER RES, V69, P5450, DOI 10.1158/0008-5472.CAN-08-4031

Jin Z, 2015, J PHYSIOL PHARMACOL, V66, P169

Jung EM, 2006, CARCINOGENESIS, V27, P2008, DOI 10.1093/carcin/bgl026

Kang SG, 2008, CHILD NERV SYST, V24, P293, DOI 10.1007/s00381-007-0515-2

Kim KY, 2001, J BIOL CHEM, V276, P40591, DOI 10.1074/jbc.M100975200

Kim SM, 2008, CANCER RES, V68, P9614, DOI 10.1158/0008-5472.CAN-08-0451

Kim WS, 2008, J DERMATOL SCI, V49, P133, DOI 10.1016/j.jdermsci.2007.08.004

Koh LWH, 2013, ANTIOXID REDOX SIGN, V19, P2261, DOI 10.1089/ars.2012.4999

Kusano C, 2000, Hum Cell, V13, P213

Leu D., 2014, 60 ANN M RAD RES SOC

Lin HD, 2014, STEM CELL REV REP, V10, P573, DOI 10.1007/s12015-014-9514-3

Loebinger MR, 2009, CANCER RES, V69, P4134, DOI 10.1158/0008-5472.CAN-08-4698

Miriyala S, 2012, BBA-MOL BASIS DIS, V1822, P794, DOI 10.1016/j.bbadis.2011.12.002

Mohr A, 2008, J CELL MOL MED, V12, P2628, DOI 10.1111/j.1582-4934.2008.00317.x

Monti DA, 2012, PLOS ONE, V7, DOI 10.1371/journal.pone.0029794

Monticone M, 2014, PLOS ONE, V9, DOI 10.1371/journal.pone.0090085

Moosavi MA, 2011, DARU, V19, P455

Nakamizo A, 2005, CANCER RES, V65, P3307, DOI 10.1158/0008-5472.CAN-04-1874

Nechushtan H, 2015, ONCOLOGIST, V20, P366, DOI 10.1634/theoncologist.2014-0424

Pivato LS, 2006, J BIOCHEM MOL TOXIC, V20, P230, DOI 10.1002/jbt.20139

Qanungo S, 2004, J BIOL CHEM, V279, P50455, DOI 10.1074/jbc.M406749200

Rajic Z, 2012, FREE RADICAL BIO MED, V52, P1828, DOI 10.1016/j.freeradbiomed.2012.02.006

Salvi M, 2005, ENDOCRINOLOGY, V146, P2306, DOI 10.1210/en.2004-1128

Sato A, 2014, STEM CELL RES, V12, P119, DOI 10.1016/j.scr.2013.09.012

SHEARMAN DJC, 1979, ANNU REV MED, V30, P61, DOI 10.1146/annurev.me.30.020179.000425

Spasojevic I, 2013, FREE RADICAL BIO MED, V65, pS132, DOI 10.1016/j.freeradbiomed.2013.10.728

Suzuki-Karasaki Y, 2014, FRONT ONCOL, V4, DOI 10.3389/fonc.2014.00128

Thakur P, 2015, MOL NEUROBIOL, V51, P209, DOI 10.1007/s12035-014-8769-7

Tovmasyan A., 2015, FREE RADICAL BIOL ME

Tovmasyan A, 2015, FREE RADICAL BIO MED, V86, P308, DOI 10.1016/j.freeradbiomed.2015.05.018

Tovmasyan A, 2014, INORG CHEM, V53, P11467, DOI 10.1021/ic501329p

Tovmasyan A, 2014, ANTIOXID REDOX SIGN, V20, P2416, DOI 10.1089/ars.2013.5576

Valle-Prieto A, 2010, STEM CELLS DEV, V19, P1885, DOI 10.1089/scd.2010.0093

Weitzel DH, 2015, MOL CANCER THER, V14, P70, DOI 10.1158/1535-7163.MCT-14-0343

Welsh JL, 2013, CANCER CHEMOTH PHARM, V71, P765, DOI 10.1007/s00280-013-2070-8

Woo JS, 2013, BIOCHEM BIOPH RES CO, V431, P354, DOI 10.1016/j.bbrc.2012.11.134

Ye XD, 2011, FREE RADICAL RES, V45, P1289, DOI 10.3109/10715762.2011.616199

Yoshida T, 2005, CANCER RES, V65, P5662, DOI 10.1158/0008-5472.CAN-05-0693

Yulyana Y, 2013, STEM CELLS DEV, V22, P1870, DOI 10.1089/scd.2012.0529

Zafarullah M, 2003, CELL MOL LIFE SCI, V60, P6, DOI 10.1007/s000180300001

Zündorf G, 2007, J NEUROCHEM, V102, P508, DOI 10.1111/j.1471-4159.2007.04509.x

NR 63

TC 30

Z9 33

U1 0

U2 13

PU SPRINGER

PI NEW YORK

PA ONE NEW YORK PLAZA, SUITE 4600, NEW YORK, NY, UNITED STATES

SN 2629-3269

EI 2629-3277

J9 STEM CELL REV REP

JI Stem Cell Rev. Rep.

PD FEB

PY 2016

VL 12

IS 1

BP 140

EP 155

DI 10.1007/s12015-015-9628-2

PG 16

WC Cell & Tissue Engineering; Cell Biology; Medicine, Research &

Experimental

WE Science Citation Index Expanded (SCI-EXPANDED)

SC Cell Biology; Research & Experimental Medicine

GA DK0DE

UT WOS:000374582000012

PM 26454429

OA Green Accepted

DA 2025-04-09

ER

PT J

AU Shandiz, SZ

Erfani, B

Hashemy, SI

AF Shandiz, Sara Zarei

Erfani, Bahareh

Hashemy, Seyed Isaac

TI Protective effects of silymarin in glioblastoma cancer cells through

redox system regulation

SO MOLECULAR BIOLOGY REPORTS

LA English

DT Article

DE Glioblastoma multiforme; Oxidative stress; Antioxidant; Silymarin; Nrf2

pathway

ID ANTIOXIDANT; APOPTOSIS; THIOREDOXIN; NRF2; SILIBININ; PATHWAY

AB BackgroundGlioblastoma multiforme, a deadly form of brain tumor, is characterized by aggressive growth and poor prognosis. Oxidative stress, a disruption in the balance between antioxidants and oxidants, is a crucial factor in its pathogenesis. Silymarin, a flavonoid extracted from milk thistle, has shown therapeutic potential in inhibiting cancer cell growth, promoting apoptosis, and reducing inflammation. It also regulates oxidative stress. This study aims to investigate the regulatory effects of silymarin on oxidative stress parameters, especially the transcription factor Nrf2 and its related enzymes in GBM cancer cells, to develop a new anti-cancer compound with low toxicity.Methods and resultsFirst, the cytotoxicity of silymarin on U-87 MG cells was investigated by MTT and the results showed an IC50 of 264.6 mu M. Then, some parameters of the redox system were measured with commercial kits, and the obtained results showed that silymarin increased the activity of catalase and superoxide dismutase enzymes, as well as the total antioxidant capacity levels; while the malondialdehyde level that is an indicator of lipid peroxidation was decreased by this compound. The expression level of Nrf2 and HO-1 and glutaredoxin and thioredoxin enzymes were checked by real-time PCR method, and the expression level increased significantly after treatment.ConclusionsOur findings suggest that silymarin may exert its cytotoxic and anticancer effects by enhancing the Nrf2/HO-1 pathway through antioxidant mechanisms in U-87 MG cells.

C1 [Shandiz, Sara Zarei] Islamic Azad Univ, Dept Biol, Mashhad Branch, Mashhad, Iran.

[Erfani, Bahareh; Hashemy, Seyed Isaac] Mashhad Univ Med Sci, Fac Med, Dept Clin Biochem, Mashhad, Iran.

[Erfani, Bahareh] Mashhad Univ Med Sci, Student Res Comm, Mashhad, Iran.

[Hashemy, Seyed Isaac] Mashhad Univ Med Sci, Surg Oncol Res Ctr, Mashhad, Iran.

C3 Islamic Azad University; Mashhad University of Medical Sciences; Mashhad

University of Medical Sciences; Mashhad University of Medical Sciences

RP Hashemy, SI (corresponding author), Mashhad Univ Med Sci, Fac Med, Dept Clin Biochem, Mashhad, Iran.; Hashemy, SI (corresponding author), Mashhad Univ Med Sci, Surg Oncol Res Ctr, Mashhad, Iran.

EM hashemyi@mums.ac.ir

RI ; Hashemy, Seyed Isaac/A-2693-2017

OI Erfani Karimzadeh Tousi, Bahareh/0000-0001-5490-0256; Hashemy, Seyed

Isaac/0000-0002-1323-5250

FU Research Council, Mashhad University of Medical Sciences

FX No Statement Available

CR ABASCAL K., 2003, Alt Comp Ther, V9, P251, DOI DOI 10.1089/107628003322490698

Bai ZL, 2018, BIOMED RES INT-UK, V2018, DOI 10.1155/2018/6165192

Chakrabarti M, 2016, APOPTOSIS, V21, P312, DOI 10.1007/s10495-015-1198-x

Chaulagain D., 2023, Int J Cancer, V18, P32, DOI [10.22141/2224-0713.18.8.2022.987, DOI 10.22141/2224-0713.18.8.2022.987]

Czarnik-Kwasniak J, 2020, NUTRIENTS, V12, DOI 10.3390/nu12010096

Dizaji MZ, 2012, NEUROCHEM RES, V37, P370, DOI 10.1007/s11064-011-0620-1

Ghahremani F, 2021, IRAN J BASIC MED SCI, V24, P499, DOI 10.22038/ijbms.2021.52902.11945

Godoy PRDV, 2020, OXID MED CELL LONGEV, V2020, DOI 10.1155/2020/2534643

Grochans S, 2022, CANCERS, V14, DOI 10.3390/cancers14102412

Hanif Farina, 2017, Asian Pac J Cancer Prev, V18, P3

Hardiany NS, 2012, MED J INDONES, V21, P122

Hosseinabadi T, 2019, PHYTOTHER RES, V33, P2849, DOI 10.1002/ptr.6470

Jaganjac M, 2020, ANTIOXIDANTS-BASEL, V9, DOI 10.3390/antiox9111151

Ji XJ, 2014, INT J CANCER, V135, P574, DOI 10.1002/ijc.28699

Karimi G, 2011, IRAN J BASIC MED SCI, V14, P308

Kemerdere R, 2013, J NEUROL SURG PART A, V74, P234, DOI 10.1055/s-0032-1333422

Kiruthiga PV, 2007, BASIC CLIN PHARMACOL, V100, P414, DOI 10.1111/j.1742-7843.2007.00069.x

Koltai T, 2022, J EVID-BASED INTEGR, V27, DOI 10.1177/2515690X211068826

Kusaczuk M, 2024, BBA-REV CANCER, V1879, DOI 10.1016/j.bbcan.2023.189054

Li L, 2017, FOOD CHEM TOXICOL, V102, P93, DOI 10.1016/j.fct.2017.01.021

Lu D., 2021, Med Sci Monit Int Med J Exp Clin Res, DOI [10.12659/2FMSM.934161, DOI 10.12659/2FMSM.934161]

Ohka F, 2012, NEUROL RES INT, V2012, DOI 10.1155/2012/878425

Pan H, 2013, NEUROL RES, V35, P71, DOI 10.1179/1743132812Y.0000000094

Ramakrishnan G, 2009, CELL PROLIFERAT, V42, P229, DOI 10.1111/j.1365-2184.2008.00581.x

Ramasamy K, 2008, CANCER LETT, V269, P352, DOI 10.1016/j.canlet.2008.03.053

Rao GM, 2000, CLIN CHIM ACTA, V296, P203, DOI 10.1016/S0009-8981(00)00219-9

Salazar-Ramiro A, 2016, FRONT IMMUNOL, V7, DOI 10.3389/fimmu.2016.00156

Serviddio G, 2014, FREE RADICAL BIO MED, V73, P117, DOI 10.1016/j.freeradbiomed.2014.05.002

Sferrazzo G, 2020, J CLIN MED, V9, DOI 10.3390/jcm9051562

Song QX, 2021, YONSEI MED J, V62, P843, DOI 10.3349/ymj.2021.62.9.843

Surai PF, 2015, ANTIOXIDANTS-BASEL, V4, P204, DOI 10.3390/antiox4010204

Svobodová A, 2007, J DERMATOL SCI, V46, P21, DOI 10.1016/j.jdermsci.2006.12.009

Thakkar JP, 2014, CANCER EPIDEM BIOMAR, V23, P1985, DOI 10.1158/1055-9965.EPI-14-0275

Toklu HZ, 2007, BURNS, V33, P908, DOI 10.1016/j.burns.2006.10.407

Vargas-Mendoza N, 2020, BIOMEDICINES, V8, DOI 10.3390/biomedicines8050122

Waris Gulam, 2006, J Carcinog, V5, P14

Yao MJ, 2018, CELL MOL IMMUNOL, V15, P737, DOI 10.1038/cmi.2017.159

Yassin Nour Y S, 2021, Oxid Med Cell Longev, V2021, P7665169, DOI 10.1155/2021/7665169

NR 38

TC 1

Z9 1

U1 1

U2 3

PU SPRINGER

PI DORDRECHT

PA VAN GODEWIJCKSTRAAT 30, 3311 GZ DORDRECHT, NETHERLANDS

SN 0301-4851

EI 1573-4978

J9 MOL BIOL REP

JI Mol. Biol. Rep.

PD DEC

PY 2024

VL 51

IS 1

AR 723

DI 10.1007/s11033-024-09658-4

PG 7

WC Biochemistry & Molecular Biology

WE Science Citation Index Expanded (SCI-EXPANDED)

SC Biochemistry & Molecular Biology

GA TA9L3

UT WOS:001238652300001

PM 38833199

DA 2025-04-09

ER

PT J

AU Sahin, B

Ergül, M

AF Sahin, Bilal

Ergul, Mustafa

TI The Role of The Inflammatory Pathway in the Protective Effects of

Captopril, an Angiotensin-Converting an Enzyme inhibitor, Against

Hydrogen Peroxide-Induced Oxidative Stress in C6 Glioma Cells

SO ACTA PHYSIOLOGICA

LA English

DT Meeting Abstract

DE Captopril; Inflammation; Hydrogen Peroxide; Oxidative Stress; Apoptosis;

C6 Rat Glioma

C1 [Sahin, Bilal] Sivas Cumhuriyet Univ, Dept Physiol, Sch Med, Sivas, Turkey.

[Ergul, Mustafa] Sivas Cumhuriyet Univ, Dept Biochem, Sch Pharm, Sivas, Turkey.

C3 Cumhuriyet University; Cumhuriyet University

NR 0

TC 0

Z9 0

U1 0

U2 0

PU WILEY

PI HOBOKEN

PA 111 RIVER ST, HOBOKEN 07030-5774, NJ USA

SN 1748-1708

EI 1748-1716

J9 ACTA PHYSIOL

JI Acta Physiol.

PD FEB

PY 2022

VL 234

SU 724

SI SI

MA OC-11

BP 17

EP 17

PG 1

WC Physiology

WE Science Citation Index Expanded (SCI-EXPANDED)

SC Physiology

GA YG2SG

UT WOS:000742342700020

DA 2025-04-09

ER

PT J

AU He, ZZ

Liu, Z

Wang, Q

Sima, X

Zhao, W

He, CM

Yang, WJ

Chen, H

Gong, B

Song, SY

Wang, Y

AF He, Zongze

Liu, Zheng

Wang, Qi

Sima, Xingjian

Zhao, Wei

He, Chunmei

Yang, Wenjie

Chen, Han

Gong, Bo

Song, Siyuan

Wang, Yi

TI Single-cell and spatial transcriptome assays reveal heterogeneity in

gliomas through stress responses and pathway alterations

SO FRONTIERS IN IMMUNOLOGY

LA English

DT Article

DE glioma; oligodendrocyte precursor cells (OPCs); oxidative stress

response; single-cell RNA sequencing; spatial transcriptomics;

heterogeneity

ID OXIDATIVE STRESS; CANCER; GLIOBLASTOMA; ACTIVATION; EXPRESSION;

APOPTOSIS; MIGRATION

AB Background Glioma is a highly heterogeneous malignancy of the central nervous system. This heterogeneity is driven by various molecular processes, including neoplastic transformation, cell cycle dysregulation, and angiogenesis. Among these biomolecular events, inflammation and stress pathways in the development and driving factors of glioma heterogeneity have been reported. However, the mechanisms of glioma heterogeneity under stress response remain unclear, especially from a spatial aspect.Methods This study employed single-cell RNA sequencing (scRNA-seq) and spatial transcriptomics (ST) to explore the impact of oxidative stress response genes in oligodendrocyte precursor cells (OPCs). Our analysis identified distinct pathways activated by oxidative stress in two different types of gliomas: high- and low- grade (HG and LG) gliomas.Results In HG gliomas, oxidative stress induced a metabolic shift from oxidative phosphorylation to glycolysis, promoting cell survival by preventing apoptosis. This metabolic reprogramming was accompanied by epithelial-to-mesenchymal transition (EMT) and an upregulation of stress response genes. Furthermore, SCENIC (Single-Cell rEgulatory Network Inference and Clustering) analysis revealed that oxidative stress activated the AP1 transcription factor in HG gliomas, thereby enhancing tumor cell survival and proliferation.Conclusion Our findings provide a novel perspective on the mechanisms of oxidative stress responses across various grades of gliomas. This insight enhances our comprehension of the evolutionary processes and heterogeneity within gliomas, potentially guiding future research and therapeutic strategies.

C1 [He, Zongze; Wang, Qi; Yang, Wenjie; Chen, Han] Univ Elect Sci & Technol China, Sichuan Prov Peoples Hosp, Dept Neurosurg, Chengdu, Peoples R China.

[Liu, Zheng] Baylor Coll Med, Dept Med, Houston, TX USA.

[Sima, Xingjian] Huazhong Univ Sci & Technol, Tongji Med Coll, Med Sch, Wuhan, Peoples R China.

[Zhao, Wei; Wang, Yi] Univ Elect Sci & Technol China, Sichuan Prov Peoples Hosp, Ctr Crit Care Med, Sch Med, Chengdu, Peoples R China.

[He, Chunmei] Chongqing Gen Hosp Chinese Peoples Armed Police Fo, Dept Otolaryngol, Chongqing, Peoples R China.

[Gong, Bo] Univ Elect Sci & Technol China, Sichuan Prov Peoples Hosp, Dept Hlth Management, Chengdu, Sichuan, Peoples R China.

[Gong, Bo] Univ Elect Sci & Technol China, Sichuan Prov Peoples Hosp, Key Lab Human Dis Gene Study Sichuan Prov, Chengdu, Sichuan, Peoples R China.

[Gong, Bo] Univ Elect Sci & Technol China, Sichuan Prov Peoples Hosp, Inst Lab Med, Chengdu, Sichuan, Peoples R China.

[Song, Siyuan] Baylor Coll Med, Dept Neurosurg, Houston, TX 77030 USA.

[Wang, Yi] Sichuan Acad Med Sci & Sichuan Prov Peoples Hosp, Key Lab Sichuan Prov, Clin Immunol Translat Med, Chengdu, Peoples R China.

C3 University of Electronic Science & Technology of China; Sichuan

Provincial People's Hospital; Baylor College of Medicine; Huazhong

University of Science & Technology; University of Electronic Science &

Technology of China; Sichuan Provincial People's Hospital; Sichuan

Provincial People's Hospital; University of Electronic Science &

Technology of China; University of Electronic Science & Technology of

China; Sichuan Provincial People's Hospital; University of Electronic

Science & Technology of China; Sichuan Provincial People's Hospital;

Baylor College of Medicine; Sichuan Provincial People's Hospital

RP Wang, Y (corresponding author), Univ Elect Sci & Technol China, Sichuan Prov Peoples Hosp, Ctr Crit Care Med, Sch Med, Chengdu, Peoples R China.; Gong, B (corresponding author), Univ Elect Sci & Technol China, Sichuan Prov Peoples Hosp, Dept Hlth Management, Chengdu, Sichuan, Peoples R China.; Gong, B (corresponding author), Univ Elect Sci & Technol China, Sichuan Prov Peoples Hosp, Key Lab Human Dis Gene Study Sichuan Prov, Chengdu, Sichuan, Peoples R China.; Gong, B (corresponding author), Univ Elect Sci & Technol China, Sichuan Prov Peoples Hosp, Inst Lab Med, Chengdu, Sichuan, Peoples R China.; Song, SY (corresponding author), Baylor Coll Med, Dept Neurosurg, Houston, TX 77030 USA.; Wang, Y (corresponding author), Sichuan Acad Med Sci & Sichuan Prov Peoples Hosp, Key Lab Sichuan Prov, Clin Immunol Translat Med, Chengdu, Peoples R China.

EM gongbo2007@hotmail.com; si-yuan.song@bcm.edu; w_yi2022@163.com

RI yang, wenjie/IXN-6840-2023

OI Liu, Zheng/0000-0003-3264-7119

FU Natural Science Foundation of China [81802504]; Health Commission Of

Sichuan Provincial, China [23LCYJ035]; Sichuan Science and Technology

Program, China [2023YFS0107, 2023YFS0106]

FX The author(s) declare financial support was received for the research,

authorship, and/or publication of this article. This study is supported

by the Natural Science Foundation of China (81802504), the grant from

Health Commission Of Sichuan Provincial, China (No. 23LCYJ035), Sichuan

Science and Technology Program, China (2023YFS0107, 2023YFS0106).

CR Agnihotri S, 2016, NEURO-ONCOLOGY, V18, P160, DOI 10.1093/neuonc/nov125

Aran D, 2019, NAT IMMUNOL, V20, P163, DOI 10.1038/s41590-018-0276-y

Bartoschek M, 2018, NAT COMMUN, V9, DOI 10.1038/s41467-018-07582-3

Berglund E, 2018, NAT COMMUN, V9, DOI 10.1038/s41467-018-04724-5

Berthenet K, 2020, CELL REP, V31, DOI 10.1016/j.celrep.2020.107731

Burns JS, 2017, INT J MOL SCI, V18, DOI 10.3390/ijms18122755

Butler A, 2018, NAT BIOTECHNOL, V36, P411, DOI 10.1038/nbt.4096

Chen WQ, 2016, CA-CANCER J CLIN, V66, P115, DOI 10.3322/caac.21338

Chen W, 2019, J MOL NEUROSCI, V68, P304, DOI 10.1007/s12031-019-01306-y

Chhipa RR, 2018, NAT CELL BIOL, V20, P823, DOI 10.1038/s41556-018-0126-z

Combs SE, 2016, CANCERS, V8, DOI 10.3390/cancers8010015

De Falco A, 2023, NAT COMMUN, V14, DOI 10.1038/s41467-023-36790-9

de la Vega MR, 2018, CANCER CELL, V34, P21, DOI 10.1016/j.ccell.2018.03.022

Dobin A, 2013, BIOINFORMATICS, V29, P15, DOI 10.1093/bioinformatics/bts635

Dodson M, 2019, REDOX BIOL, V23, DOI 10.1016/j.redox.2019.101107

Fan F, 2021, CELL ONCOL, V44, P917, DOI 10.1007/s13402-021-00612-1

Gao RL, 2021, NAT BIOTECHNOL, V39, P599, DOI 10.1038/s41587-020-00795-2

Garofano L, 2021, NAT CANCER, V2, DOI 10.1038/s43018-020-00159-4

Guntuku L, 2016, CURR NEUROPHARMACOL, V14, P567, DOI 10.2174/1570159X14666160121115641

Hafemeister C, 2019, GENOME BIOL, V20, DOI 10.1186/s13059-019-1874-1

Hayes JD, 2020, CANCER CELL, V38, P167, DOI 10.1016/j.ccell.2020.06.001

He C, 2021, FRONT CELL DEV BIOL, V9, DOI 10.3389/fcell.2021.779319

Jang M, 2013, EXP MOL MED, V45, DOI 10.1038/emm.2013.85

Johnson KC, 2021, NAT GENET, V53, P1456, DOI 10.1038/s41588-021-00926-8

Korsunsky I, 2019, NAT METHODS, V16, P1289, DOI 10.1038/s41592-019-0619-0

Kuehne A, 2015, MOL CELL, V59, P359, DOI 10.1016/j.molcel.2015.06.017

Laug D, 2018, NAT REV NEUROSCI, V19, P393, DOI 10.1038/s41583-018-0014-3

Lazarev VF, 2016, BIOCHEM BIOPH RES CO, V470, P766, DOI 10.1016/j.bbrc.2015.12.076

Liang J, 2017, CELL RES, V27, P329, DOI 10.1038/cr.2016.159

Liu ZG, 2016, ONCOTARGET, V7, P65946, DOI 10.18632/oncotarget.11779

Mabbott NA, 2013, BMC GENOMICS, V14, DOI 10.1186/1471-2164-14-632

Macosko EZ, 2015, CELL, V161, P1202, DOI 10.1016/j.cell.2015.05.002

McBrayer SK, 2018, CELL, V175, P101, DOI 10.1016/j.cell.2018.08.038

Miska J, 2019, CELL REP, V27, P226, DOI 10.1016/j.celrep.2019.03.029

Moncada R, 2020, NAT BIOTECHNOL, V38, P333, DOI 10.1038/s41587-019-0392-8

Müller S, 2017, GENOME BIOL, V18, DOI 10.1186/s13059-017-1362-4

Nabors LB, 2017, J NATL COMPR CANC NE, V15, P1331, DOI 10.6004/jnccn.2017.0166

Neftel C, 2019, CELL, V178, P835, DOI 10.1016/j.cell.2019.06.024

Norouzi-Barough L, 2018, J CELL PHYSIOL, V233, P4546, DOI 10.1002/jcp.26289

Ochocka N, 2021, NAT COMMUN, V12, DOI 10.1038/s41467-021-21407-w

Ostrom QT, 2019, NEURO-ONCOLOGY, V21, pV1, DOI 10.1093/neuonc/noz150

Park HK, 2019, CANCER RES, V79, P1369, DOI 10.1158/0008-5472.CAN-18-2558

Patel AP, 2019, LANCET NEUROL, V18, P376, DOI 10.1016/S1474-4422(18)30468-X

Patel AP, 2014, SCIENCE, V344, P1396, DOI 10.1126/science.1254257

Reczek CR, 2017, NAT CHEM BIOL, V13, P1274, DOI [10.1038/NCHEMBIO.2499, 10.1038/nchembio.2499]

Reuter S, 2010, FREE RADICAL BIO MED, V49, P1603, DOI 10.1016/j.freeradbiomed.2010.09.006

Sánchez-Pérez Y, 2017, CNS NEUROL DISORD-DR, V16, P1090, DOI 10.2174/1871527317666180110124645

Sharanek A, 2020, NAT COMMUN, V11, DOI 10.1038/s41467-020-17885-z

Shen JL, 2020, GUT, V69, P329, DOI 10.1136/gutjnl-2019-318668

Sies H, 2020, NAT REV MOL CELL BIO, V21, P363, DOI 10.1038/s41580-020-0230-3

Slyper M, 2020, NAT MED, V26, P792, DOI 10.1038/s41591-020-0844-1

Ståhl PL, 2016, SCIENCE, V353, P78, DOI 10.1126/science.aaf2403

Takebe N, 2015, NAT REV CLIN ONCOL, V12, P445, DOI 10.1038/nrclinonc.2015.61

Venteicher AS, 2017, SCIENCE, V355, DOI 10.1126/science.aai8478

Wesseling P, 2018, NEUROPATH APPL NEURO, V44, P139, DOI 10.1111/nan.12432

Wu LQ, 2019, J CELL BIOCHEM, V120, P19044, DOI 10.1002/jcb.29227

Xu SC, 2020, CANCER LETT, V476, P1, DOI 10.1016/j.canlet.2020.02.002

Yalamarty SSK, 2023, CANCERS, V15, DOI 10.3390/cancers15072116

Zhai Y, 2020, FRONT IMMUNOL, V11, DOI 10.3389/fimmu.2020.581209

Zhang ZQ, 2021, ONCOL REP, V46, DOI 10.3892/or.2021.8153

Zheng LJ, 2017, MOL NEUROBIOL, V54, P3492, DOI 10.1007/s12035-016-9926-y

Zhou XP, 2012, J NEURO-ONCOL, V110, P9, DOI 10.1007/s11060-012-0933-1

NR 62

TC 0

Z9 0

U1 15

U2 16

PU FRONTIERS MEDIA SA

PI LAUSANNE

PA AVENUE DU TRIBUNAL FEDERAL 34, LAUSANNE, CH-1015, SWITZERLAND

SN 1664-3224

J9 FRONT IMMUNOL

JI Front. Immunol.

PD AUG 27

PY 2024

VL 15

AR 1452172

DI 10.3389/fimmu.2024.1452172

PG 16

WC Immunology

WE Science Citation Index Expanded (SCI-EXPANDED)

SC Immunology

GA F3C0R

UT WOS:001308624300001

PM 39257581

OA gold

DA 2025-04-09

ER

PT J

AU Cholia, RP

Kumari, S

Kumar, S

Kaur, M

Kaur, M

Kumar, R

Dhiman, M

Mantha, AK

AF Cholia, Ravi P.

Kumari, Sanju

Kumar, Saurabh

Kaur, Manpreet

Kaur, Manbir

Kumar, Raj

Dhiman, Monisha

Mantha, Anil K.

TI An in vitro study ascertaining the role of

H2O2 and glucose oxidase in modulation of

antioxidant potential and cancer cell survival mechanisms in

glioblastoma U-87 MG cells

SO METABOLIC BRAIN DISEASE

LA English

DT Article

DE Oxidative stress; Antioxidants; APE1; Curcumin; Quercetin; iNOS

ID OXIDATIVE STRESS; SUPEROXIDE-DISMUTASE; NITRIC-OXIDE; CYCLOOXYGENASE-2

EXPRESSION; GLUTATHIONE-PEROXIDASE; HYDROGEN-PEROXIDE; COX-2 EXPRESSION;

DOWN-REGULATION; APE1/REF-1; QUERCETIN

AB Glial cells protect themselves from the elevated reactive oxygen species (ROS) via developing unusual mechanisms to maintain the genomic stability, and reprogramming of the cellular antioxidant system to cope with the adverse effects. In the present study non-cytotoxic dose of oxidants, H2O2 (100 mu M) and GO (10 mu U/ml) was used to induce moderate oxidative stress via generating ROS in human glioblastoma cell line U-87 MG cells, which showed a marked increase in the antioxidant capacity as studied by measuring the modulation in expression levels and activities of superoxide dismutase (SOD1 and SOD2) and catalase (CAT) enzymes, and the GSH content. However, pretreatment (3 h) of Curcumin and Quercetin (10 mu M) followed by the treatment of oxidants enhanced the cell survival, and the levels/activities of the antioxidants studied. Oxidative stress also resulted in an increase in the nitrite levels in the culture supernatants, and further analysis by immunocytochemistry showed an increase in iNOS expression. In addition, phytochemical pretreatment decreased the nitrite level in the culture supernatants of oxidatively stressed U-87 MG cells. Elevated ROS also increased the expression of COX-2 and APE1 enzymes and pretreatment of Curcumin and Quercetin decreased COX-2 expression and increased APE1 expression in the oxidatively stressed U-87 MG cells. The immunocytochemistry also indicates for APE1 enhanced stress-dependent subcellular localization to the nuclear compartment, which advocates for enhanced DNA repair and redox functions of APE1 towards survival of U-87 MG cells. It can be concluded that intracellular oxidants activate the key enzymes involved in antioxidant mechanisms, NO-dependent survival mechanisms, and also in the DNA repair pathways for glial cell survival in oxidative-stress micro-environment.

C1 [Cholia, Ravi P.; Mantha, Anil K.] Cent Univ Punjab, Sch Basic & Appl Sci, Ctr Anim Sci, Bathinda 151001, Punjab, India.

[Kumari, Sanju; Kumar, Saurabh; Kaur, Manpreet; Kaur, Manbir] Cent Univ Punjab, Sch Basic & Appl Sci, Ctr Biosci, Bathinda, Punjab, India.

[Dhiman, Monisha] Cent Univ Punjab, Sch Basic & Appl Sci, Ctr Biochem & Microbial Sci, Bathinda, Punjab, India.

[Kumar, Raj] Cent Univ Punjab, Sch Basic & Appl Sci, Ctr Pharmaceut Sci & Nat Prod, Bathinda, Punjab, India.

C3 Central University of Punjab; Central University of Punjab; Central

University of Punjab; Central University of Punjab

RP Mantha, AK (corresponding author), Cent Univ Punjab, Sch Basic & Appl Sci, Ctr Anim Sci, Bathinda 151001, Punjab, India.

EM anilmantha@gmail.com

RI Kaur, Manpreet/HJZ-4398-2023; Mantha, Anil/AAM-5035-2021; Cholia,

Ravi/AAH-8077-2021; Kumar, Raj/AAY-1522-2021; Kumar, Saurabh/M-5890-2017

OI , Raj/0000-0001-5113-6627; Cholia, Ravi/0000-0002-6694-3955; Dhiman,

Monisha/0000-0001-5923-3384; Kumar, Saurabh/0000-0003-2878-7426

FU BSR-startup grant from the University Grants Commission (UGC), New

Delhi, India; scheme Research Seed Money (RSM) from the Central

University of Punjab, Bathinda (CUPB); Indian Council for Medical

Research (ICMR), New Delhi, India

FX This work being supported to A. K. M. by the BSR-startup grant received

from the University Grants Commission (UGC), New Delhi, India, and the

funds received under the scheme Research Seed Money (RSM) from the

Central University of Punjab, Bathinda (CUPB). R.P.C. acknowledges

financial support in the form of senior research fellowship (SRF) from

the Indian Council for Medical Research (ICMR), New Delhi, India. The

confocal laser scanning microscope (Olympus) facility of the Central

Instrumentation Laboratory (CIL), CUPB is thankfully acknowledged.

Because of the limited focus of the article, many relevant and

appropriate references could not be included, for which the authors

apologize.

CR Alía M, 2006, TOXICOL APPL PHARM, V212, P110, DOI 10.1016/j.taap.2005.07.014

Ambs S, 1998, NAT MED, V4, P1371, DOI 10.1038/3957

BEAUCHAM.C, 1971, ANAL BIOCHEM, V44, P276, DOI 10.1016/0003-2697(71)90370-8

Bhakat KK, 2009, ANTIOXID REDOX SIGN, V11, P621, DOI [10.1089/ars.2008.2198, 10.1089/ARS.2008.2198]

Birben E, 2012, WORLD ALLERGY ORGAN, V5, P9, DOI 10.1097/WOX.0b013e3182439613

Bryan NS, 2007, FREE RADICAL BIO MED, V43, P645, DOI 10.1016/j.freeradbiomed.2007.04.026

Caneba CA, 2014, CELL DEATH DIS, V5, DOI 10.1038/cddis.2014.264

Carper D, 2001, FREE RADICAL BIO MED, V31, P90, DOI 10.1016/S0891-5849(01)00561-5

Che MX, 2016, DRUG DISCOV TODAY, V21, P143, DOI 10.1016/j.drudis.2015.10.001

Chen TJ, 2006, TOXICOLOGY, V223, P113, DOI 10.1016/j.tox.2006.03.007

Dhandapani KM, 2007, J NEUROCHEM, V102, P522, DOI 10.1111/j.1471-4159.2007.04633.x

Dhar SK, 2012, FREE RADICAL BIO MED, V52, P2209, DOI 10.1016/j.freeradbiomed.2012.03.009

Dhiman M, 2012, PLOS ONE, V7, DOI 10.1371/journal.pone.0028449

Dhiman M, 2009, CLIN VACCINE IMMUNOL, V16, P660, DOI 10.1128/CVI.00019-09

Dokic I, 2012, GLIA, V60, P1785, DOI 10.1002/glia.22397

Duthie SJ, 1997, MUTAT RES-GEN TOX EN, V393, P223, DOI 10.1016/S1383-5718(97)00107-1

FENG L, 1995, J CLIN INVEST, V95, P1669, DOI 10.1172/JCI117842

Ferraresi R, 2005, FREE RADICAL RES, V39, P1249, DOI 10.1080/10715760500306935

Glynn SA, 2010, J CLIN INVEST, V120, P3843, DOI 10.1172/JCI42059

Goel A, 2001, CANCER LETT, V172, P111, DOI 10.1016/S0304-3835(01)00655-3

Haar CP, 2012, NEUROCHEM RES, V37, P1192, DOI 10.1007/s11064-011-0701-1

Harris IS, 2015, CANCER CELL, V27, P211, DOI 10.1016/j.ccell.2014.11.019

Heo HJ, 2004, J AGR FOOD CHEM, V52, P7514, DOI 10.1021/jf049243r

Holland EC, 2001, NAT REV GENET, V2, P120, DOI 10.1038/35052535

Kim GN, 2009, ANN NY ACAD SCI, V1171, P530, DOI 10.1111/j.1749-6632.2009.04690.x

Knerr S, 2006, MOL NUTR FOOD RES, V50, P378, DOI 10.1002/mnfr.200500183

Kuo CP, 2011, ANESTHESIOLOGY, V115, P1229, DOI 10.1097/ALN.0b013e31823306f0

Mantha AK, 2012, J NEUROSCI RES, V90, P1230, DOI 10.1002/jnr.23018

Martindale JL, 2002, J CELL PHYSIOL, V192, P1, DOI 10.1002/jcp.10119

Milligan SA, 1996, AM J PHYSIOL-LUNG C, V271, pL114, DOI 10.1152/ajplung.1996.271.1.L114

Minghetti L, 2004, J NEUROPATH EXP NEUR, V63, P901, DOI 10.1093/jnen/63.9.901

Montaldi AP, 2015, MUTAT RES-GEN TOX EN, V793, P19, DOI 10.1016/j.mrgentox.2015.06.001

Murakami A, 2008, CANCER LETT, V269, P315, DOI 10.1016/j.canlet.2008.03.046

Naidu MD, 2010, J RADIAT RES, V51, P393, DOI 10.1269/jrr.09077

Park IJ, 2009, ANN NY ACAD SCI, V1171, P538, DOI 10.1111/j.1749-6632.2009.04698.x

Preusser M, 2011, ANN NEUROL, V70, P9, DOI 10.1002/ana.22425

Qu J, 2007, NUCLEIC ACIDS RES, V35, P2522, DOI 10.1093/nar/gkl1163

Rahman I, 2006, NAT PROTOC, V1, P3159, DOI 10.1038/nprot.2006.378

Ravindran J, 2009, AAPS J, V11, P495, DOI 10.1208/s12248-009-9128-x

Sen S, 2012, FREE RADICAL BIO MED, V53, P1541, DOI 10.1016/j.freeradbiomed.2012.06.030

Shanmugam MK, 2015, MOLECULES, V20, P2728, DOI 10.3390/molecules20022728

Shono T, 2001, CANCER RES, V61, P4375

Siddiqui MA, 2011, HUM EXP TOXICOL, V30, P192, DOI 10.1177/0960327110371696

Silber JR, 2002, CLIN CANCER RES, V8, P3008

Singh S, 2012, FREE RADICAL BIO MED, V53, P1782, DOI 10.1016/j.freeradbiomed.2012.07.025

Sosa V, 2013, AGEING RES REV, V12, P376, DOI 10.1016/j.arr.2012.10.004

Surh YJ, 2001, MUTAT RES-FUND MOL M, V480, P243, DOI 10.1016/S0027-5107(01)00183-X

Tell G, 2010, CELL MOL LIFE SCI, V67, P3589, DOI 10.1007/s00018-010-0486-4

Tell G, 2009, ANTIOXID REDOX SIGN, V11, P601, DOI 10.1089/ars.2008.2194

Thakur S, 2014, EXP MOL MED, V46, DOI 10.1038/emm.2014.42

Tran AN, 2017, ANTIOXID REDOX SIGN, V26, P986, DOI 10.1089/ars.2016.6820

Weydert CJ, 2010, NAT PROTOC, V5, P51, DOI 10.1038/nprot.2009.197

Yoo DG, 2008, LUNG CANCER, V60, P277, DOI 10.1016/j.lungcan.2007.10.015

NR 53

TC 14

Z9 16

U1 0

U2 23

PU SPRINGER/PLENUM PUBLISHERS

PI NEW YORK

PA 233 SPRING ST, NEW YORK, NY 10013 USA

SN 0885-7490

EI 1573-7365

J9 METAB BRAIN DIS

JI Metab. Brain Dis.

PD OCT

PY 2017

VL 32

IS 5

BP 1705

EP 1716

DI 10.1007/s11011-017-0057-6

PG 12

WC Endocrinology & Metabolism; Neurosciences

WE Science Citation Index Expanded (SCI-EXPANDED)

SC Endocrinology & Metabolism; Neurosciences & Neurology

GA FG9BD

UT WOS:000410732300036

PM 28676971

DA 2025-04-09

ER

PT J

AU Chen, X

Yu, MC

Xu, W

Kun, P

Wan, WB

Yuhong, X

Ye, J

Liu, Y

Luo, J

AF Chen, Xiang

Yu, Mingchuan

Xu, Wei

Kun, Peng

Wan, Wenbing

Yuhong, Xiao

Ye, Jing

Liu, Yu

Luo, Jun

TI RETRACTED: PCBP2 Reduced Oxidative Stress-Induced Apoptosis in Glioma

through cGAS/STING Pathway by METTL3-Mediated m6A Modification

(Retracted Article)

SO OXIDATIVE MEDICINE AND CELLULAR LONGEVITY

LA English

DT Article; Retracted Publication

ID RNA; CELLS; GLIOBLASTOMA; PROGRESSION; ACTIVATION; EXPRESSION; CANCER;

DAMAGE

AB Purpose. The most prevalent primary malignant tumor of CNS is glioma, which has a dismal prognosis. The theory of oxidative stress is one of the important theories in the study of its occurrence and development mechanism. In this study, the impacts of PCBP2 on glioma sufferers and the possible mechanisms were examined. Methods. Patients with glioma were obtained from May 2017 to July 2018. Quantitative PCR, microarray analysis, western blot analysis, and immunofluorescence were used in this experiment. Results. PCBP2 mRNA expression level and protein expression in patients with glioma were upregulated compared with paracancerous tissue. OS and DFS of PCBP2 low expression in patients with glioma were higher than those of PCBP2 high expression. PCBP2 promoted the progression and metastasis of glioma. PCBP2 reduced oxidative stress-induced apoptosis of glioma. PCBP2 suppressed the cGAS/STING pathway of glioma. PCBP2 protein interlinked with cGAS and cGAS was one target for PCBP2. METTL3-mediated m6A modification increases PCBP2 stability. Conclusion. Along the cGAS-STING signal pathway, PCBP2 decreased the apoptosis that oxidative stress-induced glioma caused, which might be a potential target to suppress oxidative stress-induced apoptosis of glioma.

C1 [Chen, Xiang; Yu, Mingchuan; Yuhong, Xiao; Ye, Jing; Liu, Yu; Luo, Jun] Nanchang Univ, Affiliated Hosp 2, Dept Rehabil Med, 1 Minde Rd, Nanchang, Jiangxi, Peoples R China.

[Xu, Wei] Nanchang Univ, Affiliated Hosp 2, Dept Gastrointestinal Surg, Nanchang, Jiangxi, Peoples R China.

[Kun, Peng; Wan, Wenbing] Nanchang Univ, Affiliated Hosp 2, Dept Orthoped, Nanchang, Jiangxi, Peoples R China.

C3 Nanchang University; Nanchang University; Nanchang University

RP Luo, J (corresponding author), Nanchang Univ, Affiliated Hosp 2, Dept Rehabil Med, 1 Minde Rd, Nanchang, Jiangxi, Peoples R China.

EM 1106346331@qq.com; 784632642@qq.com; 2194353272@qq.com;

1227901459@qq.com; 1216209756@qq.com; 651229742@qq.com;

2818470865@qq.com; 906293809@qq.com; junluo888@hotmail.com

RI YE, Jing/ABQ-1898-2022

FU National Key Research and Development Program of China [2020YFC2005800];

Regional Funds of the National Natural Science Foundation of China

[81760408, 8156036]; Project of the Science and Technology Department of

Jiangxi Province, China [20181BCG42001]; Natural Science Foundation of

Jiangxi Province, China [20113BCB22005]

FX This study is supported by the National Key Research and Development

Program of China (2020YFC2005800); Regional Funds of the National

Natural Science Foundation of China (81760408 and 8156036); Project of

the Science and Technology Department of Jiangxi Province, China

(20181BCG42001); and Natural Science Foundation of Jiangxi Province,

China (20113BCB22005).

CR Bridge G, 2014, CANCERS, V6, P1597, DOI 10.3390/cancers6031597

Chang JY, 2007, J NEURO-ONCOL, V84, P9, DOI 10.1007/s11060-007-9347-x

Chang YZ, 2021, CANCER LETT, V511, P36, DOI 10.1016/j.canlet.2021.04.020

Chen BJ, 2021, FRONT PHARMACOL, V12, DOI 10.3389/fphar.2021.719644

Chen RH, 2021, JHEP REP, V3, DOI 10.1016/j.jhepr.2021.100324

Cheng X, 2020, CELL METAB, V32, P229, DOI 10.1016/j.cmet.2020.06.002

Cong PL, 2021, FRONT CELL DEV BIOL, V9, DOI 10.3389/fcell.2021.709022

Dixit D, 2021, CANCER DISCOV, V11, P480, DOI 10.1158/2159-8290.CD-20-0331

Du HS, 2021, BIOMED PHARMACOTHER, V133, DOI 10.1016/j.biopha.2020.110972

Du JY, 2021, BRIEF BIOINFORM, V22, DOI 10.1093/bib/bbab013

Elmadany N, 2022, BIOMEDICINES, V10, DOI 10.3390/biomedicines10071738

Gu HY, 2022, NAT COMMUN, V13, DOI 10.1038/s41467-022-29266-9

Guarnaccia M, 2022, LIFE-BASEL, V12, DOI 10.3390/life12070956

Huang K, 2022, BIOLOGY-BASEL, V11, DOI 10.3390/biology11081115

Ishii T, 2020, J BIOL CHEM, V295, P12247, DOI 10.1074/jbc.RA119.011870

Jiang ML, 2020, J HEMATOL ONCOL, V13, DOI 10.1186/s13045-020-00916-z

Kim SH, 2014, J NEUROSCI RES, V92, P1419, DOI 10.1002/jnr.23431

Li YK, 2021, THERANOSTICS, V11, P2182, DOI 10.7150/thno.53102

Lam LHT, 2022, CANCERS, V14, DOI 10.3390/cancers14143492

Mao JH, 2020, J CELL PHYSIOL, V235, P3280, DOI 10.1002/jcp.29104

Muoio B, 2022, DIAGNOSTICS, V12, DOI 10.3390/diagnostics12071665

Ortiz-Rivera J, 2022, BRAIN SCI, V12, DOI 10.3390/brainsci12070893

Ostrowski RP, 2022, NEUROCHEM INT, V154, DOI 10.1016/j.neuint.2022.105281

Pu ZC, 2020, MOL THER-NUCL ACIDS, V22, P310, DOI 10.1016/j.omtn.2020.08.023

Tao NR, 2022, CELL DEATH DISCOV, V8, DOI 10.1038/s41420-022-01075-5

Tomkova S, 2018, BBA-MOL CELL RES, V1865, P616, DOI 10.1016/j.bbamcr.2018.01.012

Wang XN, 2021, MOL CANCER RES, V19, P86, DOI 10.1158/1541-7786.MCR-20-0390

Wang Y, 2020, MOL CANCER, V19, DOI 10.1186/s12943-020-01247-w

Weenink B, 2019, SCI REP-UK, V9, DOI 10.1038/s41598-019-51063-6

Wu LQ, 2019, J CELL BIOCHEM, V120, P19044, DOI 10.1002/jcb.29227

Xu SC, 2021, EVID-BASED COMPL ALT, V2021, DOI 10.1155/2021/6521953

Yabe-Wada T, 2020, FEBS OPEN BIO, V10, P407, DOI 10.1002/2211-5463.12794

Yao PS, 2014, BIOCHEM BIOPH RES CO, V450, P295, DOI 10.1016/j.bbrc.2014.05.120

Zhang WW, 2021, AGING-US, V13, P22588, DOI 10.18632/aging.203572

Zhang YH, 2020, J EXP CLIN CANC RES, V39, DOI 10.1186/s13046-020-01706-8

Zheng LJ, 2017, MOL NEUROBIOL, V54, P3492, DOI 10.1007/s12035-016-9926-y

Zhou HX, 2020, OXID MED CELL LONGEV, V2020, DOI 10.1155/2020/7126976

NR 37

TC 11

Z9 12

U1 2

U2 16

PU HINDAWI LTD

PI LONDON

PA ADAM HOUSE, 3RD FLR, 1 FITZROY SQ, LONDON, W1T 5HF, ENGLAND

SN 1942-0900

EI 1942-0994

J9 OXID MED CELL LONGEV

JI Oxidative Med. Cell. Longev.

PD OCT 11

PY 2022

VL 2022

AR 9049571

DI 10.1155/2022/9049571

PG 11

WC Cell Biology

WE Science Citation Index Expanded (SCI-EXPANDED)

SC Cell Biology

GA 5U4FQ

UT WOS:000876505200006

PM 36267817

OA hybrid, Green Published

DA 2025-04-09

ER

PT J

AU Means, JC

Gerdes, BC

Koulen, P

AF Means, John C.

Gerdes, Bryan C.

Koulen, Peter

TI Distinct Mechanisms Underlying Resveratrol-Mediated Protection from

Types of Cellular Stress in C6 Glioma Cells

SO INTERNATIONAL JOURNAL OF MOLECULAR SCIENCES

LA English

DT Article

DE caspase; central nervous system; DNA damage; glia; neurofibrillary

tangles; oxidative stress; tauopathy

ID OXIDATIVE STRESS; ALZHEIMER-DISEASE; TAU; IMPAIRMENT; CLEAVAGE; TANGLES;

DAMAGE

AB The polyphenolic phytostilbene, trans-resveratrol, is found in high amounts in several types and tissues of plants, including grapes, and has been proposed to have beneficial effects in the central nervous system due to its activity as an antioxidant. The objective of the present study was to identify the mechanisms underlying the protective effects of resveratrol under conditions of oxidative stress or DNA damage, induced by the extracellularly applied oxidant, tert-butyl hydrogen peroxide, or UV-irradiation, respectively. In C6 glioma cells, a model system for glial cell biology and pharmacology, resveratrol was protective against both types of insult. Prevention of tau protein cleavage and of the formation of neurofibrillary tangles were identified as mechanisms of action of resveratrol-mediated protection in both paradigms of cellular damage. However, depending on the type of insult, resveratrol exerted its protective activity differentially: under conditions of chemically induced oxidative stress, inhibition of caspase activity, while with DNA damage, resveratrol regulated tau phosphorylation at Ser(422). Results advance our understanding of resveratrol's complex impact on cellular signaling pathway and contribute to the notion of resveratrol's role as a pleiotropic therapeutic agent.

C1 [Means, John C.; Gerdes, Bryan C.; Koulen, Peter] Univ Missouri, Sch Med, Dept Ophthalmol, Vis Res Ctr, 2411 Holmes St, Kansas City, MO 64108 USA.

[Koulen, Peter] Univ Missouri, Sch Med, Dept Biomed Sci, 2411 Holmes St, Kansas City, MO 64108 USA.

C3 University of Missouri System; University of Missouri Kansas City;

University of Missouri System; University of Missouri Kansas City

RP Koulen, P (corresponding author), Univ Missouri, Sch Med, Dept Ophthalmol, Vis Res Ctr, 2411 Holmes St, Kansas City, MO 64108 USA.; Koulen, P (corresponding author), Univ Missouri, Sch Med, Dept Biomed Sci, 2411 Holmes St, Kansas City, MO 64108 USA.

EM meanscj@umkc.edu; gerdesbc@umkc.edu; koulenp@umkc.edu

FU NIH [AG022550, AG027956]; NIH/NIA [RR027093]; NIH/NCRR; NIH/NEI

[EY022774]; Felix and Carmen Sabates Missouri Endowed Chair in Vision

Research; Vision Research Foundation of Kansas City; Research to Prevent

Blindness

FX The research presented in the present publication was supported in part

by NIH grants AG022550 and AG027956 from NIH/NIA, RR027093 from NIH/NCRR

and EY022774 from NIH/NEI (PK). The content is solely the responsibility

of the authors and does not necessarily represent the official views of

the National Institutes of Health. Additional support by the Felix and

Carmen Sabates Missouri Endowed Chair in Vision Research, the Vision

Research Foundation of Kansas City and a departmental challenge grant by

Research to Prevent Blindness (PK) is gratefully acknowledged. The

authors thank Margaret, Richard and Sara Koulen for generous support and

encouragement.

CR Alonso AD, 2001, P NATL ACAD SCI USA, V98, P6923, DOI 10.1073/pnas.121119298

Alonso AD, 2008, CURR ALZHEIMER RES, V5, P375, DOI 10.2174/156720508785132307

Anna Brozyna, 2007, Expert Rev Dermatol, V2, P451, DOI 10.1586/17469872.2.4.451

Barbagallo M, 2015, MEDIAT INFLAMM, V2015, DOI 10.1155/2015/624801

Baur JA, 2006, NAT REV DRUG DISCOV, V5, P493, DOI 10.1038/nrd2060

BOWLING AC, 1995, LIFE SCI, V56, P1151, DOI 10.1016/0024-3205(95)00055-B

Chen XP, 2012, NEURAL REGEN RES, V7, P376, DOI 10.3969/j.issn.1673-5374.2012.05.009

Coppedè F, 2009, CURR ALZHEIMER RES, V6, P36, DOI 10.2174/156720509787313970

dos Santos AQ, 2006, ARCH BIOCHEM BIOPHYS, V453, P161, DOI 10.1016/j.abb.2006.06.025

Foster NL, 1997, ANN NEUROL, V41, P706, DOI 10.1002/ana.410410606

Frémont L, 2000, LIFE SCI, V66, P663, DOI 10.1016/S0024-3205(99)00410-5

Gamblin TC, 2003, P NATL ACAD SCI USA, V100, P10032, DOI 10.1073/pnas.1630428100

Gandhi S, 2012, OXID MED CELL LONGEV, V2012, DOI 10.1155/2012/428010

Iqbal K, 2010, CURR ALZHEIMER RES, V7, P656, DOI 10.2174/156720510793611592

Kaja S, 2011, NEUROSCIENCE, V175, P281, DOI 10.1016/j.neuroscience.2010.11.010

Kaja S, 2015, J PHARMACOL TOX MET, V73, P1, DOI 10.1016/j.vascn.2015.02.001

Kim GH, 2015, EXP NEUROBIOL, V24, P325, DOI 10.5607/en.2015.24.4.325

Kim YA, 2006, INT J MOL MED, V17, P1069

Kim Y, 2016, NEUROBIOL DIS, V87, P19, DOI 10.1016/j.nbd.2015.12.006

Konyalioglu S, 2013, NEURAL REGEN RES, V8, P485, DOI 10.3969/j.issn.1673-5374.2013.06.001

Koukourakis MI, 2012, BRIT J RADIOL, V85, P313, DOI 10.1259/bjr/16386034

Means JC, 2016, NEUROCHEM RES, V41, P2278, DOI 10.1007/s11064-016-1942-9

Mouser PE, 2006, AM J PATHOL, V168, P936, DOI 10.2353/ajpath.2006.050798

NISHIMURA M, 1995, AM J PATHOL, V146, P1052

Pervaiz S, 2004, DRUG RESIST UPDATE, V7, P333, DOI 10.1016/j.drup.2004.11.001

Prager I, 2016, BRAIN BEHAV, V6, DOI 10.1002/brb3.548

Quincozes-Santos A, 2013, PLOS ONE, V8, DOI 10.1371/journal.pone.0064372

Rege SD, 2014, FRONT AGING NEUROSCI, V6, DOI 10.3389/fnagi.2014.00218

Saiko P, 2008, MUTAT RES-REV MUTAT, V658, P68, DOI 10.1016/j.mrrev.2007.08.004

Shi QL, 2007, ALZ DIS ASSOC DIS, V21, P276, DOI 10.1097/WAD.0b013e31815721c3

Ulakcsai Z, 2015, CROAT MED J, V56, P78, DOI 10.3325/cmj.2015.56.78

Villafiores OB, 2012, TAIWAN J OBSTET GYNE, V51, P554, DOI 10.1016/j.tjog.2012.09.010

Wang MJ, 2001, J NEUROIMMUNOL, V112, P28, DOI 10.1016/S0165-5728(00)00374-X

Wang Q, 2002, BRAIN RES, V958, P439, DOI 10.1016/S0006-8993(02)03543-6

Zhao Y, 2013, OXID MED CELL LONGEV, V2013, DOI 10.1155/2013/316523

NR 35

TC 22

Z9 24

U1 0

U2 5

PU MDPI

PI BASEL

PA ST ALBAN-ANLAGE 66, CH-4052 BASEL, SWITZERLAND

EI 1422-0067

J9 INT J MOL SCI

JI Int. J. Mol. Sci.

PD JUL

PY 2017

VL 18

IS 7

AR 1521

DI 10.3390/ijms18071521

PG 12

WC Biochemistry & Molecular Biology; Chemistry, Multidisciplinary

WE Science Citation Index Expanded (SCI-EXPANDED)

SC Biochemistry & Molecular Biology; Chemistry

GA FF2SJ

UT WOS:000408746800190

PM 28708069

OA Green Published, Green Submitted, gold

DA 2025-04-09

ER

PT J

AU Lu, Z

Sun, GF

He, KY

Zhang, Z

Han, XH

Qu, XH

Wan, DF

Yao, DY

Tou, FF

Han, XJ

Wang, T

AF Lu, Zhuo

Sun, Gui-Feng

He, Kai-Yi

Zhang, Zhen

Han, Xin-Hao

Qu, Xin-Hui

Wan, Deng-Feng

Yao, Dongyuan

Tou, Fang -Fang

Han, Xiao-Jian

Wang, Tao

TI Targeted inhibition of branched-chain amino acid metabolism drives

apoptosis of glioblastoma by facilitating ubiquitin degradation of Mfn2

and oxidative stress

SO BIOCHIMICA ET BIOPHYSICA ACTA-MOLECULAR BASIS OF DISEASE

LA English

DT Article

DE BCATc Inhibitor 2; Apoptosis; Mfn2; Oxidative stress; PI3K/AKT/mTOR

signaling; Glioblastoma

ID MITOCHONDRIAL BIOGENESIS; BCAT1 PROMOTES; CANCER; SAFETY; CELLS

AB Glioblastoma is one of the most challenging malignancies with high aggressiveness and invasiveness and its development and progression of glioblastoma highly depends on branched -chain amino acid (BCAA) metabolism. The study aimed to investigate effects of inhibition of BCAA metabolism with cytosolic branched -chain amino acid transaminase (BCATc) Inhibitor 2 on glioblastoma, elucidate its underlying mechanisms, and explore therapeutic potential of targeting BCAA metabolism. The expression of BCATc was upregulated in glioblastoma and BCATc Inhibitor 2 precipitated apoptosis both in vivo and in vitro with the activation of Bax/Bcl2/Caspase3/Caspase-9 axis. In addition, BCATc Inhibitor 2 promoted K63 -linkage ubiquitination of mitofusin 2 (Mfn2), which subsequently caused lysosomal degradation of Mfn2, and then oxidative stress, mitochondrial fission and loss of mitochondrial membrane potential. Furthermore, BCATc Inhibitor 2 treatment resulted in metabolic reprogramming, and significant inhibition of expression of ATP5A, UQCRC2, SDHB and COX II, indicative of suppressed oxidative phosphorylation. Moreover, Mfn2 overexpression or scavenging mitochondria -originated reactive oxygen species (ROS) with mito-TEMPO ameliorated BCATc Inhibitor 2 -induced oxidative stress, mitochondrial membrane potential disruption and mitochondrial fission, and abrogated the inhibitory effect of BCATc Inhibitor 2 on glioblastoma cells through PI3K/AKT/mTOR signaling. All of these findings indicate suppression of BCAA metabolism promotes glioblastoma cell apoptosis via disruption of Mfn2-mediated mitochondrial dynamics and inhibition of PI3K/AKT/mTOR pathway, and suggest that BCAA metabolism can be targeted for developing therapeutic agents to treat glioblastoma.

C1 [Sun, Gui-Feng; Han, Xin-Hao; Tou, Fang -Fang; Han, Xiao-Jian; Wang, Tao] Nanchang Med Coll, Jiangxi Prov Peoples Hosp, Affiliated Hosp 1, Inst Geriatr, 152 Aiguo Rd, Nanchang 330006, Jiangxi, Peoples R China.

[Lu, Zhuo] Nanchang Univ, Affiliated Hosp 1, Jiangxi Med Coll, Dept Thorac Surg, Nanchang 330006, Jiangxi, Peoples R China.

[He, Kai-Yi; Han, Xiao-Jian] Nanchang Univ, Jiangxi Med Coll, Sch Pharmaceut Sci, Dept Pharmacol, Nanchang 330006, Jiangxi, Peoples R China.

[Zhang, Zhen; Han, Xiao-Jian] Nanchang Med Coll, Jiangxi Prov Peoples Hosp, Affiliated Hosp 1, Inst Clin Med, Nanchang 330006, Jiangxi, Peoples R China.

[Qu, Xin-Hui] Nanchang Med Coll, Jiangxi Prov Peoples Hosp, Affiliated Hosp 1, Dept Neurol 2, Nanchang 330006, Jiangxi, Peoples R China.

[Wan, Deng-Feng] Nanchang Med Coll, Jiangxi Prov Peoples Hosp, Affiliated Hosp 1, Dept Neurosurg, Nanchang 330006, Jiangxi, Peoples R China.

[Yao, Dongyuan] Nanchang Med Coll, Jiangxi Prov Peoples Hosp, Affiliated Hosp 1, Neurol Inst Jiangxi Prov,Dept Neurol, Nanchang 330006, Jiangxi, Peoples R China.

C3 Nanchang Medical College; Nanchang University; Nanchang University;

Nanchang Medical College; Nanchang Medical College; Nanchang Medical

College; Nanchang Medical College

RP Han, XJ; Wang, T (corresponding author), Nanchang Med Coll, Jiangxi Prov Peoples Hosp, Affiliated Hosp 1, Inst Geriatr, 152 Aiguo Rd, Nanchang 330006, Jiangxi, Peoples R China.

EM hanxiaojian@hotmail.com; wangtaoalepellis@gmail.com

RI Wang, Tao/AIF-0681-2022

OI Wang, Tao/0000-0003-4535-3830

FU Science and Technology Research Project from Department of Education of

Jiangxi Province [GJJ2203555]; National Natural Science Foundation of

China [82303229]; Jiangxi Provincial Natural Science Foundation

[20232BAB216078]; Science and Technology Plan Project of Health

Commission of Jiangxi Province [202310001]; Science and Technology Plan

Project of Administration of Traditional Chinese Medicine of Jiangxi

Province [2022B063, 82060177, 81472371]; Key Science and Technology

Innovation Project of Jiangxi Provincial Health Com- mission

[2023ZD001]; Research Fund for Jiangxi Geriatric Clinical Medical

Research Center [2020BCG74003]; Natural Science Foundation of Jiangxi

Province [20224ACB206014]; Department of Education of Jiangxi Province

[GJJ218902]

FX This work was supported by Science and Technology Research Project from

Department of Education of Jiangxi Province (GJJ2203555) , National

Natural Science Foundation of China (82303229) , Jiangxi Provincial

Natural Science Foundation (20232BAB216078) , Science and Technology

Plan Project of Health Commission of Jiangxi Province (202310001) ,

Science and Technology Plan Project of Administration of Traditional

Chinese Medicine of Jiangxi Province (2022B063) granted to Tao Wang,

National Natural Science Foundation of China (82060177, 81472371) , The

Key Science and Technology Innovation Project of Jiangxi Provincial

Health Com- mission (2023ZD001) , the Research Fund for Jiangxi

Geriatric Clinical Medical Research Center (2020BCG74003) , Natural

Science Foundation of Jiangxi Province (20224ACB206014) , and the Key

Projects from Department of Education of Jiangxi Province (GJJ218902)

granted to Xiao-Jian Han.

CR Ashraf R, 2022, CELL MOL LIFE SCI, V79, DOI 10.1007/s00018-022-04595-6

Balaban RS, 2005, CELL, V120, P483, DOI 10.1016/j.cell.2005.02.001

Benischke AS, 2017, SCI REP-UK, V7, DOI 10.1038/s41598-017-06523-2

Bi JB, 2019, REDOX BIOL, V20, P296, DOI 10.1016/j.redox.2018.10.019

Caballero J, 2009, MOL DIVERS, V13, P493, DOI 10.1007/s11030-009-9140-1

Chan DC, 2020, ANNU REV PATHOL-MECH, V15, P235, DOI 10.1146/annurev-pathmechdis-012419-032711

Cooper AJL, 2002, ANAL BIOCHEM, V308, P100, DOI 10.1016/S0003-2697(02)00243-9

Dasgupta A, 2021, FASEB J, V35, DOI 10.1096/fj.202100361R

Detmer SA, 2007, NAT REV MOL CELL BIO, V8, P870, DOI 10.1038/nrm2275

Fang CL, 2017, J CANCER, V8, P1153, DOI 10.7150/jca.17986

Han TY, 2018, AUTOPHAGY, V14, P2035, DOI 10.1080/15548627.2018.1491493

Hu LY, 2006, BIOORG MED CHEM LETT, V16, P2337, DOI 10.1016/j.bmcl.2005.07.058

Kumar Sandeep, 2022, Breast Dis, V41, P241, DOI 10.3233/BD-210066

Lai Q, 2021, BASIC RES CARDIOL, V116, DOI 10.1007/s00395-021-00844-0

Lamoral-Theys D, 2010, NEOPLASIA, V12, P69, DOI 10.1593/neo.91360

Leboucher GP, 2012, MOL CELL, V47, P547, DOI 10.1016/j.molcel.2012.05.041

Lei MZ, 2020, SIGNAL TRANSDUCT TAR, V5, DOI 10.1038/s41392-020-0168-0

Li J, 2022, RESP PHYSIOL NEUROBI, V306, DOI 10.1016/j.resp.2022.103961

Liu SY, 2021, BIOMED PHARMACOTHER, V141, DOI 10.1016/j.biopha.2021.111810

Livak KJ, 2001, METHODS, V25, P402, DOI 10.1006/meth.2001.1262

Lu Z, 2022, FRONT PHARMACOL, V13, DOI 10.3389/fphar.2022.1025551

Luo C, 2021, BRIT J NEUROSURG, V35, P555, DOI 10.1080/02688697.2021.1907306

Mayers JR, 2016, SCIENCE, V353, P1161, DOI 10.1126/science.aaf5171

McLelland GL, 2018, ELIFE, V7, DOI 10.7554/eLife.32866

Mishra P, 2016, J CELL BIOL, V212, P379, DOI 10.1083/jcb.201511036

Morio A, 2021, DIABETOL METAB SYNDR, V13, DOI 10.1186/s13098-021-00755-z

Nguyen TTT, 2021, NAT COMMUN, V12, DOI 10.1038/s41467-021-25501-x

Nie C., 2018, Int J Mol Sci, Branched Chain Amino Acids, P19

Olson KC, 2014, OBESITY, V22, P1212, DOI 10.1002/oby.20691

Pungsrinont T, 2021, INT J MOL SCI, V22, DOI 10.3390/ijms222011088

Silva LS, 2017, EMBO REP, V18, P2172, DOI 10.15252/embr.201744154

Soria JC, 2017, EUR J CANCER, V86, P186, DOI 10.1016/j.ejca.2017.08.027

Tönjes M, 2013, NAT MED, V19, P901, DOI 10.1038/nm.3217

Vansteenkiste JF, 2015, J THORAC ONCOL, V10, P1319, DOI 10.1097/JTO.0000000000000607

Wai T, 2016, TRENDS ENDOCRIN MET, V27, P105, DOI 10.1016/j.tem.2015.12.001

Wei Z, 2018, NAT COMMUN, V9, DOI 10.1038/s41467-018-06812-y

Wirsching Hans-Georg, 2016, Handb Clin Neurol, V134, P381, DOI 10.1016/B978-0-12-802997-8.00023-2

You MH, 2021, SCI REP-UK, V11, DOI 10.1038/s41598-021-81469-0

Zhang B, 2022, CANCER RES, V82, P2388, DOI 10.1158/0008-5472.CAN-21-3868

Zhang B, 2021, CELL MOL LIFE SCI, V78, P195, DOI 10.1007/s00018-020-03483-1

Zhang L, 2017, BIOCHEM BIOPH RES CO, V486, P224, DOI 10.1016/j.bbrc.2017.02.101

Zhu KR, 2022, CELLS-BASEL, V11, DOI 10.3390/cells11162508

Zou GP, 2021, J CANCER, V12, P7358, DOI 10.7150/jca.61379

NR 43

TC 8

Z9 8

U1 4

U2 7

PU ELSEVIER

PI AMSTERDAM

PA RADARWEG 29, 1043 NX AMSTERDAM, NETHERLANDS

SN 0925-4439

EI 1879-260X

J9 BBA-MOL BASIS DIS

JI Biochim. Biophys. Acta-Mol. Basis Dis.

PD JUN

PY 2024

VL 1870

IS 5

AR 167220

DI 10.1016/j.bbadis.2024.167220

EA MAY 2024

PG 13

WC Biochemistry & Molecular Biology; Biophysics; Cell Biology

WE Science Citation Index Expanded (SCI-EXPANDED)

SC Biochemistry & Molecular Biology; Biophysics; Cell Biology

GA TG6L6

UT WOS:001240151400001

PM 38718847

DA 2025-04-09

ER

PT J

AU Rezaei, S

Darban, RA

Javid, H

Hashemy, SI

AF Rezaei, Soodabeh

Darban, Reza Assaran

Javid, Hossein

Hashemy, Seyed Isaac

TI The Therapeutic Potential of Aprepitant in Glioblastoma Cancer Cells

through Redox Modification

SO BIOMED RESEARCH INTERNATIONAL

LA English

DT Article

ID NEUROKININ-1 RECEPTOR ANTAGONIST; TOTAL ANTIOXIDANT CAPACITY;

CHEMOTHERAPY-INDUCED NAUSEA; OXIDATIVE STRESS; SYSTEM; INFLAMMATION;

DAMAGE

AB Although there is no doubt regarding the involvement of oxidative stress in the development of glioblastoma, many questions remained unanswered about signaling cascades that regulate the redox status. Given the importance of the substance P (SP)/neurokinin 1 receptor (NK1R) system in different cancers, it was of particular interest to evaluate whether the stimulation of this cascade in glioblastoma-derived U87 cells is associated with the induction of oxidative stress. Our results showed that SP-mediated activation of NK1R not only increased the intracellular levels of malondialdehyde (MDA) and reactive oxygen species (ROS) but also reduced the concentration of thiol in U87 cells. We also found that upon SP addition, there was a significant reduction in the cells' total antioxidant capacity (TAC), revealing that the SP/NK1R axis may be involved in the regulation of oxidative stress in glioblastoma cells. The significant role of SP/NK1R in triggering oxidative stress in glioblastoma has become more evident when we found that the abrogation of the axis using aprepitant reduced cell survival, probably through exerting antioxidant effects. The results showed that both MDA and ROS concentrations were significantly reduced in the presence of aprepitant, and the number of antioxidant components of the redox system increased. Overall, these findings suggest that aprepitant might exert its anticancer effect on U87 cells through shifting the balance of oxidant and antioxidant components of the redox system.

C1 [Rezaei, Soodabeh; Darban, Reza Assaran] Islamic Azad Univ, Fac Sci, Dept Biol, Mashhad Branch, Mashhad, Razavi Khorasan, Iran.

[Javid, Hossein; Hashemy, Seyed Isaac] Mashhad Univ Med Sci, Fac Med, Dept Clin Biochem, Mashhad, Razavi Khorasan, Iran.

[Javid, Hossein] Varastegan Inst Med Sci, Dept Med Lab Sci, Mashhad, Razavi Khorasan, Iran.

[Hashemy, Seyed Isaac] Mashhad Univ Med Sci, Surg Oncol Res Ctr, Mashhad, Razavi Khorasan, Iran.

C3 Islamic Azad University; Mashhad University of Medical Sciences; Mashhad

University of Medical Sciences

RP Hashemy, SI (corresponding author), Mashhad Univ Med Sci, Fac Med, Dept Clin Biochem, Mashhad, Razavi Khorasan, Iran.; Hashemy, SI (corresponding author), Mashhad Univ Med Sci, Surg Oncol Res Ctr, Mashhad, Razavi Khorasan, Iran.

EM hashemyi@mums.ac.ir

RI Hashemy, Seyed Isaac/A-2693-2017; Assaran-Darban, Reza/S-3572-2017

OI Hashemy, Seyed Isaac/0000-0002-1323-5250; Assaran-Darban,

Reza/0000-0001-9126-5618; Javid, Dr. Hossein/0000-0002-2248-7708

CR Baek SM, 2016, MOL VIS, V22, P1015

Batash R, 2017, CURR MED CHEM, V24, P3002, DOI 10.2174/0929867324666170516123206

Bayati S, 2016, EUR J PHARMACOL, V791, P274, DOI 10.1016/j.ejphar.2016.09.006

Berger M, 2014, J HEPATOL, V60, P985, DOI 10.1016/j.jhep.2013.12.024

Blair IA, 2001, EXP GERONTOL, V36, P1473, DOI 10.1016/S0531-5565(01)00133-4

Chmielinska JJ, 2020, MOL CELL BIOCHEM, V465, P175, DOI 10.1007/s11010-019-03677-7

Filomeni G, 2015, CELL DEATH DIFFER, V22, P377, DOI 10.1038/cdd.2014.150

Ge CT, 2019, P NATL ACAD SCI USA, V116, P19635, DOI 10.1073/pnas.1908998116

Ghahremani F, 2021, IRAN J BASIC MED SCI, V24, P499, DOI 10.22038/ijbms.2021.52902.11945

Ghahremanloo A, 2021, BIOMED RES INT, V2021, DOI 10.1155/2021/1383878

Hanif Farina, 2017, Asian Pac J Cancer Prev, V18, P3

Hardiany NS, 2012, MED J INDONES, V21, P122

Hargreaves R, 2011, ANN NY ACAD SCI, V1222, P40, DOI 10.1111/j.1749-6632.2011.05961.x

Hashemian P, 2020, INT J CANCER MANAG, V13, DOI 10.5812/ijcm.100717

Hashemy SI, 2011, IRAN J BASIC MED SCI, V14, P191

Inda MD, 2014, CANCERS, V6, P226, DOI 10.3390/cancers6010226

Javid H, 2021, BIOMED RES INT, V2021, DOI 10.1155/2021/8808214

Javid H, 2020, MOL BIOL REP, V47, P2253, DOI 10.1007/s11033-020-05330-9

Javid H, 2019, J PHYSIOL BIOCHEM, V75, P415, DOI 10.1007/s13105-019-00697-1

Kangari Parisa, 2018, Asian Pac J Cancer Prev, V19, P3511

Kast RE, 2016, J NEURO-ONCOL, V126, P425, DOI 10.1007/s11060-015-1996-6

Kim EK, 2019, ANTIOXIDANTS-BASEL, V8, DOI 10.3390/antiox8100471

Korfi F, 2021, BIOCHEM RES INT, V2021, DOI 10.1155/2021/6620708

Mantha AK, 2006, BIOGERONTOLOGY, V7, P1, DOI 10.1007/s10522-005-6043-0

Mohammadi F, 2019, CANCER CHEMOTH PHARM, V84, P925, DOI 10.1007/s00280-019-03912-4

Muñoz M, 2020, J CLIN MED, V9, DOI 10.3390/jcm9061659

Muñoz M, 2015, J BIOSCIENCES, V40, P441, DOI 10.1007/s12038-015-9530-8

Muñoz M, 2014, INT J ONCOL, V45, P1658, DOI 10.3892/ijo.2014.2565

Muñoz M, 2014, INT J ONCOL, V44, P137, DOI 10.3892/ijo.2013.2164

Pelicano H, 2004, DRUG RESIST UPDATE, V7, P97, DOI 10.1016/j.drup.2004.01.004

Poli-Bigelli S, 2003, CANCER-AM CANCER SOC, V97, P3090, DOI 10.1002/cncr.11433

Rashad Yara A, 2014, Sci Pharm, V82, P129, DOI 10.3797/scipharm.1306-18

Rasool M, 2016, PLOS ONE, V11, DOI 10.1371/journal.pone.0167149

Santiago-Arteche R, 2012, MOL BIOL REP, V39, P9355, DOI 10.1007/s11033-012-1760-3

Schieber M, 2014, CURR BIOL, V24, pR453, DOI 10.1016/j.cub.2014.03.034

Sener DE, 2007, CELL BIOCHEM FUNCT, V25, P377, DOI 10.1027/cbf.1308

Serafini M, 2012, INT J CANCER, V131, pE544, DOI 10.1002/ijc.27347

Suvas S, 2017, J IMMUNOL, V199, P1543, DOI 10.4049/jimmunol.1601751

Tosoni A, 2016, CURR TREAT OPTION ON, V17, DOI 10.1007/s11864-016-0422-4

Tuzgen S, 2007, CLIN ONCOL-UK, V19, P177, DOI 10.1016/j.clon.2006.11.012

Wang F, 2019, BIOCHEM BIOPH RES CO, V514, P1210, DOI 10.1016/j.bbrc.2019.05.092

Wu W, 2020, TRANSL ONCOL, V13, DOI 10.1016/j.tranon.2020.100748

Zhang J, 2018, MOL CLIN ONCOL, V8, P391, DOI 10.3892/mco.2018.1549

Zhang R, 2020, LIFE SCI, V250, DOI 10.1016/j.lfs.2020.117583

NR 44

TC 24

Z9 25

U1 0

U2 1

PU HINDAWI LTD

PI LONDON

PA ADAM HOUSE, 3RD FLR, 1 FITZROY SQ, LONDON, W1T 5HF, ENGLAND

SN 2314-6133

EI 2314-6141

J9 BIOMED RES INT

JI Biomed Res. Int.

PD MAR 3

PY 2022

VL 2022

AR 8540403

DI 10.1155/2022/8540403

PG 8

WC Biotechnology & Applied Microbiology; Medicine, Research & Experimental

WE Science Citation Index Expanded (SCI-EXPANDED)

SC Biotechnology & Applied Microbiology; Research & Experimental Medicine

GA 0K5IE

UT WOS:000780821200011

PM 35281606

OA hybrid, Green Published

DA 2025-04-09

ER

PT J

AU Lazarev, VF

Nikotina, AD

Mikhaylova, ER

Nudler, E

Polonik, SG

Guzhova, IV

Margulis, BA

AF Lazarev, Vladimir F.

Nikotina, Alina D.

Mikhaylova, Elena R.

Nudler, Evgeny

Polonik, Sergey G.

Guzhova, Irina V.

Margulis, Boris A.

TI Hsp70 chaperone rescues C6 rat glioblastoma cells from oxidative stress

by sequestration of aggregating GAPDH

SO BIOCHEMICAL AND BIOPHYSICAL RESEARCH COMMUNICATIONS

LA English

DT Article

DE Oxidative stress; Cancer cells; Hsp70; GAPDH; Protein-protein

interaction

ID HEAT-SHOCK; GLYCERALDEHYDE-3-PHOSPHATE DEHYDROGENASE; ALZHEIMER-DISEASE;

DEATH; PROTEINS; TRIPTOLIDE; INHIBITOR; MECHANISM; THERAPY; PEPTIDE

AB The Hsp70 chaperone is known to elicit cytoprotective activity and this protection has a negative impact in anti-tumor therapy. In cancer cells subjected to oxidative stress Hsp70 may bind damaged poly peptides and proteins involved in apoptosis signaling. Since one of the important targets of oxidative stress is glyceraldehyde-3-phospate dehydrogenase (GAPDH) we suggested that Hsp70 might elicit its protective effect by binding GAPDH. Microscopy data show that in C6 rat glioma cells subjected to hydrogen peroxide treatment a considerable proportion of the GAPDH molecules are denatured and according to dot ultrafiltration data they form SDS-insoluble aggregates. Using two newly developed assays we show that Hsp70 can bind oxidized GAPDH in an ATP-dependent manner. Pharmacological up or down-regulation of Hsp70 with the aid of U133 echinochrome or triptolide, respectively, reduced or increased the number of C6 glioma cells containing GAPDH aggregates and dying due to treatment with hydrogen peroxide. Using immunoprecipitation we found that Hsp70 is able to sequester aggregation prone GAPDH and this may explain the anti-oxidative power of the chaperone. The results of this study led us to conclude that in cancer cells constantly exposed to conditions of oxidative stress, the protective power of Hsp70 should be abolished by specific inhibitors of Hsp70 expression. (C) 2015 Elsevier Inc. All rights reserved.

C1 [Lazarev, Vladimir F.; Nikotina, Alina D.; Mikhaylova, Elena R.; Guzhova, Irina V.; Margulis, Boris A.] Russian Acad Sci, Inst Cytol, Tikhoretsky Pr 4, St Petersburg 194064, Russia.

[Nudler, Evgeny] NYU, Sch Med, Dept Biochem & Mol Pharmacol, 550 First Ave, New York, NY USA.

[Polonik, Sergey G.] Russian Acad Sci, Pacific Inst Bioorgan Chem, Pr 100 Let Vladivostoku 159, Vladivostok 690022, Russia.

C3 Russian Academy of Sciences; St. Petersburg Scientific Centre of the

Russian Academy of Sciences; Institute of Cytology RAS; New York

University; Russian Academy of Sciences; Elyakov Pacific Institute of

Bioorganic Chemistry

RP Lazarev, VF (corresponding author), Russian Acad Sci, Inst Cytol, Tikhoretsky Pr 4, St Petersburg 194064, Russia.

EM vl.lazarev@gmail.com

RI Polonik, Sergey/N-4118-2013; Mikhaylova, Elena/AAN-2169-2020; Lazarev,

Vladimir/O-9813-2017; Nikotina, Alina/AAN-1775-2020; Guzhova,

Irina/R-5702-2016; Nikotina, Alina/K-7798-2015

OI Nikotina, Alina/0000-0002-0458-036X; Lazarev,

Vladimir/0000-0002-7117-6789; Nudler, Evgeny/0000-0002-8811-3071

FU Russian Scientific Foundation [14-50-00068]; Russian Science Foundation

[14-50-00068] Funding Source: Russian Science Foundation

FX This work was supported by the Russian Scientific Foundation (Grant #

14-50-00068).

CR BRADFORD MM, 1976, ANAL BIOCHEM, V72, P248, DOI 10.1016/0003-2697(76)90527-3

Butterfield DA, 2013, ANTIOXID REDOX SIGN, V19, P823, DOI 10.1089/ars.2012.5027

Castro JP, 2012, FREE RADICAL BIO MED, V53, P916, DOI 10.1016/j.freeradbiomed.2012.06.005

Cumming RC, 2005, FASEB J, V19, P2060, DOI 10.1096/fj.05-4195fje

Cunningham TJ, 2015, REJUV RES, V18, P136, DOI 10.1089/rej.2014.1620

Damalas A, 2011, INT J CANCER, V128, P1989, DOI 10.1002/ijc.25510

Eremenko E M, 2010, Tsitologiia, V52, P235

Grigorieva JA, 1999, ARCH BIOCHEM BIOPHYS, V369, P252, DOI 10.1006/abbi.1999.1341

Guzhova IV, 2013, INT J HYPERTHER, V29, P399, DOI 10.3109/02656736.2013.807439

Guzhova IV, 2011, HUM MOL GENET, V20, P3953, DOI 10.1093/hmg/ddr314

Itakura M, 2015, J BIOL CHEM, V290, P26072, DOI 10.1074/jbc.M115.669291

Itakura M, 2015, BIOCHEM BIOPH RES CO, V467, P373, DOI 10.1016/j.bbrc.2015.09.150

Joly AL, 2010, J INNATE IMMUN, V2, P238, DOI 10.1159/000296508

Kaniuk NA, 2007, DIABETES, V56, P930, DOI 10.2337/db06-1160

Lazarev VF, 2011, BIOCHEMISTRY-MOSCOW+, V76, P590, DOI 10.1134/S0006297911050099

Lazarev VF, 2015, FEBS LETT, V589, P581, DOI 10.1016/j.febslet.2015.01.018

Leak RK, 2014, J CELL COMMUN SIGNAL, V8, P293, DOI 10.1007/s12079-014-0243-9

Luo Q, 2011, FREE RADICAL RES, V45, P1355, DOI 10.3109/10715762.2011.627330

Nakajima H, 2007, J BIOL CHEM, V282, DOI 10.1074/jbc.M704199200

Nakajima H, 2009, J BIOL CHEM, V284, P34331, DOI 10.1074/jbc.M109.027698

Rodrigues JR, 2012, ANTICANCER RES, V32, P2721

Sherman MY, 2015, ONCOGENE, V34, P4153, DOI 10.1038/onc.2014.349

Shevtsov MA, 2014, ONCOTARGET, V5, P3101, DOI 10.18632/oncotarget.1820

Tsai KL, 1997, J PHYSIOL-LONDON, V502, P161, DOI 10.1111/j.1469-7793.1997.161bl.x

Verbeke P, 2001, FREE RADICAL BIO MED, V31, P1593, DOI 10.1016/S0891-5849(01)00752-3

Westerheide SD, 2006, J BIOL CHEM, V281, P9616, DOI 10.1074/jbc.M512044200

Zhou ZL, 2012, NAT PROD REP, V29, P457, DOI 10.1039/c2np00088a

NR 27

TC 29

Z9 33

U1 0

U2 13

PU ACADEMIC PRESS INC ELSEVIER SCIENCE

PI SAN DIEGO

PA 525 B ST, STE 1900, SAN DIEGO, CA 92101-4495 USA

SN 0006-291X

EI 1090-2104

J9 BIOCHEM BIOPH RES CO

JI Biochem. Biophys. Res. Commun.

PD FEB 12

PY 2016

VL 470

IS 3

BP 766

EP 771

DI 10.1016/j.bbrc.2015.12.076

PG 6

WC Biochemistry & Molecular Biology; Biophysics

WE Science Citation Index Expanded (SCI-EXPANDED)

SC Biochemistry & Molecular Biology; Biophysics

GA DE2OR

UT WOS:000370467100046

PM 26713364

DA 2025-04-09

ER

PT J

AU Ahmet, A

Hakan, KA

Derya, K

Mustafa, C

Cansu, OK

Adem, D

AF Ahmet, Albayrak

Hakan, Kurt Akif

Derya, Kilicaslan

Mustafa, Celik

Cansu, Oztabag Kara

Adem, Doganer

TI Protective Effects of Quercetin in Combination with Donepezil against

H2O2-Induced Oxidative Stress in Glioblastoma

Cells

SO PHARMACEUTICAL CHEMISTRY JOURNAL

LA English

DT Article

DE quercetin; donepezil; Alzheimer's disease; oxidative stress; U-118 MG

glioblastoma

ID ACETYLCHOLINESTERASE INHIBITORS; ALZHEIMERS-DISEASE; NEUROPROTECTION;

CYTOTOXICITY; FLAVONOIDS; RECEPTORS; PATHOLOGY; TOXICITY; DEATH; MODEL

AB Polyphenols are natural antioxidants found in the human diet, which are known to have protective effects against neurodegenerative diseases by scavenging free radicals. Recently, combination therapies including cholinesterase (ChE) inhibitors and neuroprotective agents have been shown to be more efficacious in the prevention and treatment of Alzheimer's disease. Therefore, the purpose of this work was to assess the neuroprotective impact of the combination of quercetin, a major polyphenolic compound, and donepezil, a cholinesterase inhibitor, against hydrogen peroxide-induced oxidative damage of glioblastoma (U-118 MG) cells. Following pretreatment with quercetin and donepezil, the glioblastoma cell line was subjected to H2O2-induced oxidative stress damage via application of 250 mu MH2O2 and incubation for a period of 24 h. In this study, four test groups were selected for the investigation of quercetin and donepezil as protective agents against H2O2 oxidative stress. First, quercetin and donepezil were examined in terms of their effects on glioblastoma cell viability. Next, pre- and post-application of quercetin and donepezil were used to determine their concentrations effective against the H2O2-induced oxidative damage in the U-118 MG cell line. Finally, the effect of the combined application of quercetin and donepezil on the cell viability was investigated. Viability testing of the cell lines was carried out via the advanced XTT assay using sulfonated tetrazolium. As a result of testing, no protective or therapeutic effects of donepezil alone were observed on the cell damage caused by oxidative stress. On the other hand, quercetin and combination applications exhibited protective or therapeutic effects on the cell damage. Thus, the neuroprotective effect of quercetin and its combination with donepezil provide a new approach to the search for drugs in the clinical treatment of neurodegenerative diseases.

C1 [Ahmet, Albayrak] Kahramanmaras Sutcu Imam Univ, Inst Nat & Appl Sci, Dept Bioengn & Sci, Kahramanmaras, Turkiye.

[Hakan, Kurt Akif] Bolu Abant Izzet Baysal Univ, Fac Med, Dept Pharmacol, Bolu, Turkiye.

[Derya, Kilicaslan] Kahramanmaras Sutcu Imam Univ, Afsin Vocat Sch, Dept Chem & Chem Proc Technol, Kahramanmaras, Turkiye.

[Mustafa, Celik] Kahramanmaras Sutcu Imam Univ, Fac Med, Dept Med Genet, Kahramanmaras, Turkiye.

[Cansu, Oztabag Kara] Bolu Abant Izzet Baysal Univ, Inst Hlth Sci, Dept Interdisciplinary Neurosci, Bolu, Turkiye.

[Adem, Doganer] Kahramanmaras Sutcu Imam Univ, Fac Med, Dept Biostat & Med Informat, Kahramanmaras, Turkiye.

C3 Kahramanmaras Sutcu Imam University; Abant Izzet Baysal University;

Kahramanmaras Sutcu Imam University; Kahramanmaras Sutcu Imam

University; Abant Izzet Baysal University; Kahramanmaras Sutcu Imam

University

RP Hakan, KA (corresponding author), Bolu Abant Izzet Baysal Univ, Fac Med, Dept Pharmacol, Bolu, Turkiye.

EM farma1975@hotmail.com

RI ÇELİK, Mustafa/HJY-8381-2023; Doganer, Adem/GPX-5935-2022; Albayrak,

Ahmet/MGU-0558-2025; Kılıçaslan, Derya/JVZ-8872-2024

FU Kahramanmaras Sutcu Imam University [2019/4 - 22D]

FX The Kahramanmaras Sutcu Imam University supported this work (Project No:

2019/4 - 22D). We would also like to thank Ali Raif Ilac Sanayi (ARIS)

for their support by supplying the donepezil hydrochloride.

CR Akaike A, 2006, ALZ DIS ASSOC DIS, V20, pS8, DOI 10.1097/01.wad.0000213802.74434.d6

Akasofu S, 2008, CHEM-BIOL INTERACT, V175, P222, DOI 10.1016/j.cbi.2008.04.045

Alok S., 2014, TROP BIOMED, V1, P84

Ansari MA, 2009, J NUTR BIOCHEM, V20, P269, DOI 10.1016/j.jnutbio.2008.03.002

Arias E, 2005, J PHARMACOL EXP THER, V315, P1346, DOI 10.1124/jpet.105.090365

Arredondo F, 2010, FREE RADICAL BIO MED, V49, P738, DOI 10.1016/j.freeradbiomed.2010.05.020

Baakman AC, 2019, ALZH DEMENT-TRCI, V5, P89, DOI 10.1016/j.trci.2019.02.002

Costa LG, 2016, OXID MED CELL LONGEV, V2016, DOI 10.1155/2016/2986796

Dinc E, 2017, CURR EYE RES, V42, P1659, DOI 10.1080/02713683.2017.1368085

Dong HX, 2009, BRAIN RES, V1303, P169, DOI 10.1016/j.brainres.2009.09.097

El-Horany HE, 2016, J BIOCHEM MOL TOXIC, V30, P360, DOI 10.1002/jbt.21821

González-Sarrías A, 2017, J AGR FOOD CHEM, V65, P752, DOI 10.1021/acs.jafc.6b04538

Gugnani KS, 2018, TOXICOL APPL PHARM, V340, P67, DOI 10.1016/j.taap.2017.12.014

Haider S, 2014, AGE, V36, P1291, DOI 10.1007/s11357-014-9653-0

Heo HJ, 2004, J AGR FOOD CHEM, V52, P7514, DOI 10.1021/jf049243r

Jakubowicz-Gill J, 2008, ACTA NEUROBIOL EXP, V68, P139

Jantan I., 2015, PLANT SCI, V6, P655

Jantas D, 2021, INT J MOL SCI, V22, DOI 10.3390/ijms221910363

Khan H, 2020, BIOMOLECULES, V10, DOI 10.3390/biom10010059

Kim SH, 2017, BRIT J PHARMACOL, V174, P4224, DOI 10.1111/bph.14030

Kimura M, 2005, BRAIN RES, V1047, P72, DOI 10.1016/j.brainres.2005.04.014

Li X, 2016, AM J TRANSL RES, V8, P3558

Loy C, 2006, COCHRANE DB SYST REV, DOI 10.1002/14651858.CD001747.pub3

Sabogal-Guáqueta AM, 2015, NEUROPHARMACOLOGY, V93, P134, DOI 10.1016/j.neuropharm.2015.01.027

Meunier J, 2006, BRIT J PHARMACOL, V149, P998, DOI 10.1038/sj.bjp.0706927

Noh MY, 2013, J NEUROCHEM, V127, P562, DOI 10.1111/jnc.12319

Noh MY, 2009, J NEUROCHEM, V108, P1116, DOI 10.1111/j.1471-4159.2008.05837.x

Ossola B, 2009, EXPERT OPIN DRUG SAF, V8, P397, DOI 10.1517/14740330903026944

Pamies D, 2017, ALTEX-ALTERN ANIM EX, V34, P95, DOI 10.14573/altex.1607121

Racchi M, 2004, PHARMACOL RES, V50, P441, DOI 10.1016/j.phrs.2003.12.027

Rezaei-Sadabady R, 2016, ARTIF CELL NANOMED B, V44, P128, DOI 10.3109/21691401.2014.926456

Rishitha N, 2018, LIFE SCI, V199, P80, DOI 10.1016/j.lfs.2018.03.010

Riss T. L., EXPERT OPIN DRUG DIS

Rogers SL, 1996, DEMENTIA, V7, P293, DOI 10.1159/000106895

Ross JA, 2002, ANNU REV NUTR, V22, P19, DOI 10.1146/annurev.nutr.22.111401.144957

Rowland JP, 2007, Adv Psychiatr Treat, V13, P178, DOI DOI 10.1192/APT.BP.106.002725

Sandhir R, 2013, BBA-MOL BASIS DIS, V1832, P421, DOI 10.1016/j.bbadis.2012.11.018

Sasaki N, 2003, CHEM-BIOL INTERACT, V145, P101, DOI 10.1016/S0009-2797(02)00248-X

Shen H, 2010, BRIT J PHARMACOL, V161, P127, DOI 10.1111/j.1476-5381.2010.00894.x

Silveira AC, 2019, CURR NEUROPHARMACOL, V17, P590, DOI 10.2174/1570159X16666180803162059

Singh Sujata, 2011, Toxicol Int, V18, P140, DOI 10.4103/0971-6580.84267

Solntseva EI, 2014, BRAIN RES BULL, V106, P56, DOI 10.1016/j.brainresbull.2014.06.002

Takada Y, 2003, J PHARMACOL EXP THER, V306, P772, DOI 10.1124/jpet.103.050104

Takada-Takatori Y, 2009, BIOL PHARM BULL, V32, P318, DOI 10.1248/bpb.32.318

Yoshiyama Y, 2010, J ALZHEIMERS DIS, V22, P295, DOI 10.3233/JAD-2010-100681

Yu X., 2020, BIOMED RES INT

Zimmermann M, 2013, BRIT J PHARMACOL, V170, P953, DOI 10.1111/bph.12359

NR 47

TC 1

Z9 1

U1 1

U2 6

PU SPRINGER

PI NEW YORK

PA ONE NEW YORK PLAZA, SUITE 4600, NEW YORK, NY, UNITED STATES

SN 0091-150X

EI 1573-9031

J9 PHARM CHEM J+

JI Pharm. Chem. J.

PD MAR

PY 2023

VL 56

IS 12

BP 1577

EP 1586

DI 10.1007/s11094-023-02830-3

EA MAR 2023

PG 10

WC Chemistry, Medicinal; Pharmacology & Pharmacy

WE Science Citation Index Expanded (SCI-EXPANDED)

SC Pharmacology & Pharmacy

GA C2LC0

UT WOS:000952951500001

DA 2025-04-09

ER

PT J

AU Ostrowski, RP

Pucko, EB

AF Ostrowski, Robert P.

Pucko, Emanuela B.

TI Harnessing oxidative stress for anti-glioma therapy

SO NEUROCHEMISTRY INTERNATIONAL

LA English

DT Article

DE Glioma; Glioblastoma; Oxidative stress; Nitrosative stress

ID GLIOBLASTOMA-MULTIFORME CELLS; DOUBLE-EDGED-SWORD; GLIOMA STEM-CELLS;

NITRIC-OXIDE; REACTIVE OXYGEN; HYPERBARIC-OXYGEN; NITROSATIVE STRESS;

MALIGNANT GLIOMA; S-NITROSYLATION; APOPTOTIC MECHANISM

AB Glioma cells use intermediate levels of reactive oxygen species (ROS) and reactive nitrogen species (RNS) for growth and invasion, and suppressing these reactive molecules thus may compromise processes that are vital for glioma survival. Increased oxidative stress has been identified in glioma cells, in particular in glioma stem-like cells. Studies have shown that these cells harbor potent antioxidant defenses, although endogenous protection against nitrosative stress remains understudied. The enhancement of oxidative or nitrosative stress offers a potential target for triggering glioma cell death, but whether oxidative and nitrosative stresses can be combined for therapeutic effects requires further research. The optimal approach of harnessing oxidative stress for anti-glioma therapy should include the induction of free radical-induced oxidative damage and the suppression of antioxidant defense mechanisms selectively in glioma cells. However, selective induction of oxidative/nitrosative stress in glioma cells remains a therapeutic challenge, and research into selective drug delivery systems is ongoing. Because of multifactorial mechanisms of glioma growth, progression, and invasion, prospective oncological therapies may include not only therapeutic oxidative/nitrosative stress but also inhibition of oncogenic kinases, antioxidant molecules, and programmed cell death mediators.

C1 [Ostrowski, Robert P.; Pucko, Emanuela B.] Polish Acad Sci, Mossakowski Med Res Inst, Dept Expt & Clin Neuropathol, Warsaw, Poland.

C3 Polish Academy of Sciences; Mossakowski Medical Research Institute of

the Polish Academy of Sciences

RP Ostrowski, RP (corresponding author), MMRI, Dept Expt & Clin Neuropathol, 5 Pawi nskiego St, PL-02106 Warsaw, Poland.

EM rostrowski@imdik.pan.pl

RI Ostrowski, R./AAH-6756-2019

OI Ostrowski, Robert P./0000-0003-1373-4452

FU Foundation for the Development of Diagnostic and Therapy, Warsaw

FX This work was supported by the Foundation for the Development of

Diagnostic and Therapy, Warsaw.

CR Acharya A, 2010, OXID MED CELL LONGEV, V3, P23, DOI 10.4161/oxim.3.1.10095

Ali M., 2013, MYELOPEROXIDASE INHI

Andújar I, 2013, PLANTA MED, V79, P1685, DOI 10.1055/s-0033-1350934

[Anonymous], 2014, AUSTIN J NUCL MED RA

Aquilano K, 2014, FRONT PHARMACOL, V5, DOI 10.3389/fphar.2014.00196

Atefeh Z, 2016, CURR RADIOPHARM, V9, P258, DOI 10.2174/1874471009666160813232031

Atukeren P., 2013, Clinical Management and Evolving Novel Therapeutic Strategies for Patients with Brain Tumors

Babykutty S, 2012, CLIN EXP METASTAS, V29, P471, DOI 10.1007/s10585-012-9464-6

Baguley BC, 2003, CURR MED CHEM, V10, P2643, DOI 10.2174/0929867033456332

Baird L, 2020, MOL CELL BIOL, V40, DOI 10.1128/MCB.00099-20

Bajor M, 2016, PLOS ONE, V11, DOI 10.1371/journal.pone.0154822

Bak DH, 2016, EXP THER MED, V11, P2153, DOI 10.3892/etm.2016.3196

Berthier S, 2017, J TRACE ELEM MED BIO, V44, P161, DOI 10.1016/j.jtemb.2017.04.012

Betti M, 2006, FREE RADICAL BIO MED, V41, P464, DOI 10.1016/j.freeradbiomed.2006.04.012

Bhandary B, 2013, INT J MOL SCI, V14, P434, DOI 10.3390/ijms14010434

Bishayee A, 2010, CANCER LETT, V294, P1, DOI 10.1016/j.canlet.2010.01.030

Böttcher M, 2018, ONCOIMMUNOLOGY, V7, DOI 10.1080/2162402X.2018.1445454

Bonavida B, 2010, Nitric oxide (no) and cancer: Prognosis, prevention, and therapy, P459, DOI [10.1007/978-1-4419-1432, DOI 10.1007/978-1-4419-1432]

Resende FFB, 2018, ONCOL LETT, V15, P4891, DOI 10.3892/ol.2018.7917

Butturini E, 2021, CELLS-BASEL, V10, DOI 10.3390/cells10113149

Cao SS, 2014, ANTIOXID REDOX SIGN, V21, P396, DOI 10.1089/ars.2014.5851

Cao WQ, 2020, PHARM BIOL, V58, P72, DOI 10.1080/13880209.2019.1703756

Cao ZW, 2017, CANCER CELL, V31, P110, DOI 10.1016/j.ccell.2016.11.010

Chamaon K, 2005, J NEURO-ONCOL, V72, P11, DOI 10.1007/s11060-004-2158-4

Chang KY, 2018, CANCER RES, V78, DOI 10.1158/1538-7445.AM2018-4887

Chen CH, 2012, ANN SURG ONCOL, V19, P3097, DOI 10.1245/s10434-012-2324-4

Chen LY, 2010, AM J PATHOL, V177, P2671, DOI 10.2353/ajpath.2010.100025

Chien CH, 2019, J BIOMED SCI, V26, DOI 10.1186/s12929-019-0565-2

Chitambar CR, 2018, MOL CANCER THER, V17, P1240, DOI 10.1158/1535-7163.MCT-17-1009

Chiu WT, 2010, NEUROBIOL DIS, V37, P118, DOI 10.1016/j.nbd.2009.09.015

Cobbs CS, 2003, CANCER RES, V63, P8670

Daniele S, 2015, SCI REP-UK, V5, DOI 10.1038/srep15556

Di H, 2018, ONCOL LETT, V15, P5131, DOI 10.3892/ol.2018.7932

Dittmann LM, 2012, ONCOGENE, V31, P3409, DOI 10.1038/onc.2011.513

Doblas S, 2008, FREE RADICAL BIO MED, V44, P63, DOI 10.1016/j.freeradbiomed.2007.09.006

Dokic I, 2012, GLIA, V60, P1785, DOI 10.1002/glia.22397

Elmaci I, 2019, METAB BRAIN DIS, V34, P687, DOI 10.1007/s11011-019-00412-5

Erdi F, 2018, TURK NEUROSURG, V28, P7, DOI 10.5137/1019-5149.JTN.18991-16.2

Esen H, 2015, J NEURO-ONCOL, V121, P451, DOI 10.1007/s11060-014-1661-5

Eyler CE, 2011, CELL, V146, P53, DOI 10.1016/j.cell.2011.06.006

Fack F, 2017, EMBO MOL MED, V9, P1681, DOI 10.15252/emmm.201707729

Fahey JM, 2018, J BIOL CHEM, V293, P5345, DOI 10.1074/jbc.RA117.000443

Fan WG, 2020, INT J ONCOL, V57, P1293, DOI 10.3892/ijo.2020.5134

Feng J, 2016, ONCOL REP, V35, P1395, DOI 10.3892/or.2015.4477

Forrester MT, 2006, ACS CHEM BIOL, V1, P355, DOI 10.1021/cb600244c

Friedmann-Morvinski D, 2016, SCI ADV, V2, DOI 10.1126/sciadv.1501292

Fu XD, 2015, CELL METAB, V22, P508, DOI 10.1016/j.cmet.2015.06.009

Galano A, 2016, MOLECULES, V21, DOI 10.3390/molecules21111442

Gandini NA, 2014, TUMOR BIOL, V35, P2803, DOI 10.1007/s13277-013-1373-z

Garcia-Gomez P., 2019, NOX4 REGULATES TGF

Garrido-Armas M, 2018, TOXICOL IN VITRO, V51, P63, DOI 10.1016/j.tiv.2018.04.014

Gieryng A, 2017, LAB INVEST, V97, P498, DOI 10.1038/labinvest.2017.19

Gilmore TD, 2006, ONCOGENE, V25, P6887, DOI 10.1038/sj.onc.1209982

Girotti A.W., 2020, J CANC METASTASIS TR, V6, P52

Glod B.K., 2009, POST CHROMATOGR, V1, P1

GRAY LH, 1953, BRIT J RADIOL, V26, P638, DOI 10.1259/0007-1285-26-312-638

Grech N, 2020, CUREUS J MED SCIENCE, V12, DOI 10.7759/cureus.8195

Greenberg M., 2010, HDB NEUROSURGERY

Grégoire H, 2020, FRONT PHARMACOL, V11, DOI 10.3389/fphar.2020.00368

Griguer CE, 2006, CANCER RES, V66, P2257, DOI 10.1158/0008-5472.CAN-05-3364

Guequen A, 2019, FRONT PHYSIOL, V10, DOI 10.3389/fphys.2019.00988

Gupta A, 2017, MOL CELL ONCOL, V4, DOI 10.1080/23723556.2017.1329692

Gupta P, 2015, ANTIOXID REDOX SIGN, V23, P665, DOI 10.1089/ars.2014.5973

Ha E.T., 2014, Neuroimmunol. Neuroinflamm., P66, DOI [DOI 10.4103/2347-8659.139717, 10.4103/2347-8659.139717]

Haas B, 2018, INT J MOL SCI, V19, DOI 10.3390/ijms19102874

Hacioglu C, 2021, MED ONCOL, V38, DOI 10.1007/s12032-021-01476-z

Hambardzumyan D, 2016, NAT NEUROSCI, V19, P20, DOI 10.1038/nn.4185

Hanif Farina, 2017, Asian Pac J Cancer Prev, V18, P3

Hardee ME, 2012, AM J PATHOL, V181, P1126, DOI 10.1016/j.ajpath.2012.06.030

Hardiany NS, 2017, MED J INDONES, V26, P19, DOI 10.13181/mji.v26i1.1511

Hays E, 2019, ANTIOXIDANTS-BASEL, V8, DOI 10.3390/antiox8090407

Heckler M, 2017, TUMOR BIOL, V39, P1, DOI 10.1177/1010428317703922

Heinrich TA, 2013, BRIT J PHARMACOL, V169, P1417, DOI 10.1111/bph.12217

Holland EC, 2001, J NEURO-ONCOL, V51, P265, DOI 10.1023/A:1010609114564

Hsieh CH, 2010, ONCOL REP, V24, P1629, DOI 10.3892/or_00001027

Huang J, 2017, ONCOTARGET, V8, P70366, DOI 10.18632/oncotarget.19705

Huang L, 2018, MED GAS RES, V8, P24, DOI 10.4103/2045-9912.229600

Ishii K, 2009, CLIN NEUROPATHOL, V28, P445

Jaganjac M, 2020, ANTIOXIDANTS-BASEL, V9, DOI 10.3390/antiox9111151

Jahani-Asl A, 2013, CURR MOL MED, V13, P1241, DOI 10.2174/1566524011313080002

Jelluma N, 2006, MOL CANCER RES, V4, P319, DOI 10.1158/1541-7786.MCR-05-0061

Ramírez-Expósito MJ, 2019, CURR NEUROPHARMACOL, V17, P342, DOI 10.2174/1570159X16666180302120925

Jin HO, 2006, J CELL PHYSIOL, V206, P477, DOI 10.1002/jcp.20488

Jin L, 2018, INT J MOL MED, V41, P1339, DOI 10.3892/ijmm.2017.3334

Jin L, 2019, ONCOL LETT, V17, P4213, DOI 10.3892/ol.2019.10112

Jones DP, 2008, AM J PHYSIOL-CELL PH, V295, pC849, DOI 10.1152/ajpcell.00283.2008

Jung T, 2014, REDOX BIOL, V2, P388, DOI 10.1016/j.redox.2013.12.029

Kacar S, 2021, TOXICOL IN VITRO, V73, DOI 10.1016/j.tiv.2021.105135

Kandil Eman I, 2018, Tumour Biol, V40, p1010428317749676, DOI 10.1177/1010428317749676

Kar F, 2021, J BIOCHEM MOL TOXIC, V35, DOI 10.1002/jbt.22742

Kathagen A, 2013, ACTA NEUROPATHOL, V126, P763, DOI 10.1007/s00401-013-1173-y

Keatley K, 2019, INT J MOL SCI, V20, DOI 10.3390/ijms20133364

Keir ST, 2011, ANTI-CANCER AGENT ME, V11, P202

Kennedy BC, 2013, J ONCOL, V2013, DOI 10.1155/2013/486912

Kesanakurti D, 2011, PLOS ONE, V6, DOI 10.1371/journal.pone.0019341

Khaw AK, 2012, GENE CHROMOSOME CANC, V51, P961, DOI 10.1002/gcc.21979

Kilburn L, 2010, CANCER-AM CANCER SOC, V116, P2242, DOI 10.1002/cncr.25006

Kim SH, 2014, J NEUROSCI RES, V92, P1419, DOI 10.1002/jnr.23431

Kim TH, 2014, NEURO-ONCOLOGY, V16, P1354, DOI 10.1093/neuonc/nou088

Kim TH, 2012, PLOS ONE, V7, DOI 10.1371/journal.pone.0042818

Kirches E, 2009, ANTI-CANCER AGENT ME, V9, P55, DOI 10.2174/187152009787047725

Klingelhoeffer C, 2012, BMC COMPLEM ALTERN M, V12, DOI 10.1186/1472-6882-12-61

Koh LWH, 2013, ANTIOXID REDOX SIGN, V19, P2261, DOI 10.1089/ars.2012.4999

Konaté MM, 2020, ANTIOXID REDOX SIGN, V33, P435, DOI 10.1089/ars.2020.8046

Kopacz A, 2020, FREE RADICAL BIO MED, V157, P63, DOI 10.1016/j.freeradbiomed.2020.03.023

Koppula P, 2018, CANCER COMMUN, V38, DOI 10.1186/s40880-018-0288-x

Kowalski S, 2020, MOLECULES, V25, DOI 10.3390/molecules25071757

Krawczynski K, 2020, ANTIOXIDANTS-BASEL, V9, DOI 10.3390/antiox9080747

Kumar R., 2018, Key Heterocycle Cores for Designing Multitargeting Molecules Ed, P53

Latini A, 2003, EUR J NEUROSCI, V17, P2017, DOI 10.1046/j.1460-9568.2003.02639.x

Lee KH, 2020, INT J MOL SCI, V21, DOI 10.3390/ijms21197152

Lee Y, 2017, ACTA NEUROPATHOL COM, V5, DOI 10.1186/s40478-017-0465-1

Legendre C, 2015, TRENDS ENDOCRIN MET, V26, P322, DOI 10.1016/j.tem.2015.03.008

Leone R, 2015, MOL BIOSYST, V11, P1612, DOI 10.1039/c4mb00725e

Li F, 2015, MOL CELL, V60, P661, DOI 10.1016/j.molcel.2015.10.017

Li J, 2016, NEUROREPORT, V27, P110, DOI 10.1097/WNR.0000000000000506

Li YS, 2014, OXID MED CELL LONGEV, V2014, DOI 10.1155/2014/581732

Liu JG, 2016, INT J CLIN EXP PATHO, V9, P3152

Liu JM, 2013, BIOCHEM BIOPH RES CO, V437, P87, DOI 10.1016/j.bbrc.2013.06.042

Liu RL, 2019, NAT COMMUN, V10, DOI 10.1038/s41467-019-08921-8

Liu YY, 2016, J NEURO-ONCOL, V128, P217, DOI 10.1007/s11060-016-2107-z

Liu Y, 2017, ONCOTARGETS THER, V10, P4023, DOI 10.2147/OTT.S136821

Llaguno SRA, 2016, BRIT J CANCER, V115, P1445, DOI 10.1038/bjc.2016.354

Loboda A, 2016, CELL MOL LIFE SCI, V73, P3221, DOI 10.1007/s00018-016-2223-0

Borges CRLD, 2019, CHEM PHYS LIPIDS, V225, DOI 10.1016/j.chemphyslip.2019.104828

López-Valero I, 2018, BIOCHEM PHARMACOL, V157, P266, DOI 10.1016/j.bcp.2018.09.007

Louis DN, 2021, NEURO-ONCOLOGY, V23, P1231, DOI 10.1093/neuonc/noab106

Maccallini C, 2020, CHEMMEDCHEM, V15, P339, DOI 10.1002/cmdc.201900580

Masiulionyte B, 2019, SCI REP-UK, V9, DOI 10.1038/s41598-019-41974-9

Massi P, 2006, CELL MOL LIFE SCI, V63, P2057, DOI 10.1007/s00018-006-6156-x

Mazurek M, 2021, NEUROCHEM INT, V150, DOI 10.1016/j.neuint.2021.105172

Meenambal R, 2020, NEUROCHEM INT, V140, DOI 10.1016/j.neuint.2020.104851

Mehrian-Shai R, 2015, BMC MED GENOMICS, V8, DOI 10.1186/s12920-015-0137-6

Missall TA, 2004, EUKARYOT CELL, V3, P835, DOI 10.1128/EC.3.4.835-846.2004

Moldogazieva NT, 2018, CANCER RES, V78, P6040, DOI 10.1158/0008-5472.CAN-18-0980

Molinaro AM, 2019, NAT REV NEUROL, V15, P405, DOI 10.1038/s41582-019-0220-2

Mondesir J, 2016, J BLOOD MED, V7, P171, DOI 10.2147/JBM.S70716

Morfouace M, 2012, J BIOL CHEM, V287, P33664, DOI 10.1074/jbc.M111.320028

Morgan LL, 2015, INT J ONCOL, V46, P1865, DOI 10.3892/ijo.2015.2908

Muhammad SA, 2021, NEUROCHEM INT, V144, DOI 10.1016/j.neuint.2021.104955

Muz B, 2015, HYPOXIA, V3, P83, DOI 10.2147/HP.S93413

Nakagawa J, 2007, INT J ONCOL, V30, P803

Narla RK, 2001, CLIN CANCER RES, V7, P2124

Neth B.J., 2021, NEUROLOGIST

Ogunrinu TA, 2010, J BIOL CHEM, V285, P37716, DOI 10.1074/jbc.M110.161190

Ostrom QT, 2014, NEURO-ONCOLOGY, V16, P896, DOI 10.1093/neuonc/nou087

Osuka S, 2017, J CLIN INVEST, V127, P415, DOI 10.1172/JCI89587

Palumbo P, 2020, CANCER CELL INT, V20, DOI 10.1186/s12935-020-01250-7

Palumbo P, 2017, ONCOTARGET, V8, P25582, DOI 10.18632/oncotarget.16106

Paolini A, 2015, INT J ONCOL, V46, P1491, DOI 10.3892/ijo.2015.2864

Perillo B, 2020, EXP MOL MED, V52, P192, DOI 10.1038/s12276-020-0384-2

Philips A, 2018, J ENVIRON PUBLIC HEA, V2018, DOI 10.1155/2018/7910754

Pogoda JM, 2009, ANN EPIDEMIOL, V19, P148, DOI 10.1016/j.annepidem.2008.12.011

Polewski MD, 2017, STEM CELLS DEV, V26, P1236, DOI 10.1089/scd.2017.0123

Postovit L, 2018, OXID MED CELL LONGEV, V2018, DOI 10.1155/2018/6135739

Puar YR, 2018, BIOMEDICINES, V6, DOI 10.3390/biomedicines6030082

Pucko EB, 2020, FOLIA NEUROPATHOL, V58, P287, DOI 10.5114/fn.2020.102430

Qiu HY, 2018, CHEM BIOL DRUG DES, V91, P681, DOI 10.1111/cbdd.13141

Qiu JG, 2017, DRUG DISCOV TODAY, V22, P148, DOI 10.1016/j.drudis.2016.09.017

Ramaiah MJ, 2021, MOL BIOL REP, V48, P4813, DOI 10.1007/s11033-021-06462-2

Reuter S, 2010, FREE RADICAL BIO MED, V49, P1603, DOI 10.1016/j.freeradbiomed.2010.09.006

Rezaee L, 2020, REP PRACT ONCOL RADI, V25, P68, DOI 10.1016/j.rpor.2019.12.014

Rice T, 2016, NEURO-ONCOL PRACT, V3, P10, DOI 10.1093/nop/npv026

Ridnour LA, 2004, BIOL CHEM, V385, P1, DOI 10.1515/BC.2004.001

Rinaldi M, 2016, INT J MOL SCI, V17, DOI 10.3390/ijms17060984

Rocha CRR, 2014, CELL DEATH DIS, V5, DOI 10.1038/cddis.2014.465

Salazar-Ramiro A, 2016, FRONT IMMUNOL, V7, DOI 10.3389/fimmu.2016.00156

Santangelo R, 2020, ACS OMEGA, V5, P17900, DOI 10.1021/acsomega.9b04483

Sarfraz I, 2020, BIOFACTORS, V46, P550, DOI 10.1002/biof.1624

Schiavone S, 2019, INT J MOL SCI, V20, DOI 10.3390/ijms20051242

Schiffer D, 2010, ANTICANCER RES, V30, P1977

Scholz N, 2020, FRONT ONCOL, V10, DOI 10.3389/fonc.2020.574011

Schroeder AB, 2020, J BIOMED OPT, V25, DOI 10.1117/1.JBO.25.3.036502

Sedgwick AJ, 2020, FRONT IMMUNOL, V11, DOI 10.3389/fimmu.2020.01549

Sharma V, 2007, MOL CANCER THER, V6, P2544, DOI 10.1158/1535-7163.MCT-06-0788

Sheikh T, 2018, J BIOL CHEM, V293, P4767, DOI 10.1074/jbc.M117.816785

Shen XL, 2016, NAT IMMUNOL, V17, P1282, DOI 10.1038/ni.3545

Shen YM, 2006, CHEM RES TOXICOL, V19, P1441, DOI 10.1021/tx0601206

Shendge AK, 2021, MOL BIOL REP, V48, P539, DOI 10.1007/s11033-020-06087-x

Shimada K, 2018, CELL CHEM BIOL, V25, P585, DOI 10.1016/j.chembiol.2018.02.010

Shinawi T, 2013, EPIGENETICS-US, V8, P149, DOI 10.4161/epi.23398

Shono T, 2008, INT J CANCER, V123, P787, DOI 10.1002/ijc.23569

Sies H, 2017, ANNU REV BIOCHEM, V86, P715, DOI 10.1146/annurev-biochem-061516-045037

Singer E, 2015, CELL DEATH DIS, V6, DOI 10.1038/cddis.2014.566

Singh Gyanesh, 2015, F1000Res, V4, P176, DOI 10.12688/f1000research.6665.1

Singh K, 2018, SAUDI PHARM J, V26, P177, DOI 10.1016/j.jsps.2017.12.013

Smith PS, 2007, FREE RADICAL BIO MED, V42, P787, DOI 10.1016/j.freeradbiomed.2006.11.032

Smith-Pearson PS, 2008, FREE RADICAL BIO MED, V45, P1178, DOI 10.1016/j.freeradbiomed.2008.07.015

Snezhkina AV, 2019, OXID MED CELL LONGEV, V2019, DOI 10.1155/2019/6175804

Song K, 2019, J INT MED RES, DOI 10.1177/0300060519872898

Soubannier V, 2017, BIOMEDICINES, V5, DOI 10.3390/biomedicines5020029

Souza FD, 2020, INT J MOL SCI, V21, DOI 10.3390/ijms21218395

Speed N, 2011, CANCER METAST REV, V30, P437, DOI 10.1007/s10555-011-9298-8

Stepien K, 2016, MED ONCOL, V33, DOI 10.1007/s12032-016-0814-0

Strickland M, 2017, FRONT CELL DEV BIOL, V5, DOI 10.3389/fcell.2017.00043

Stump TA, 2017, J PHARM PHARMACOL, V69, P907, DOI 10.1111/jphp.12718

Tandon V., 2005, JK SCI J MED ED RES, V7

Tang XY, 2020, MOL CANCER THER, V19, P221, DOI 10.1158/1535-7163.MCT-19-0103

Tatarkova Z, 2012, Klin Onkol, V25, P421

Tavana E, 2020, BIOFACTORS, V46, P356, DOI 10.1002/biof.1605

Teitelbaum AM, 2012, CANCER CHEMOTH PHARM, V69, P1519, DOI 10.1007/s00280-012-1855-5

Tonelli C, 2018, ANTIOXID REDOX SIGN, V29, P1727, DOI 10.1089/ars.2017.7342

Tong X, 2013, ANTI-CANCER AGENT ME, V13, P971, DOI 10.2174/18715206113139990119

Tran AN, 2017, ANTIOXID REDOX SIGN, V26, P986, DOI 10.1089/ars.2016.6820

Varlamova EG, 2021, INT J MOL SCI, V22, DOI 10.3390/ijms22157798

Vitovcova B, 2020, INT J MOL SCI, V21, DOI 10.3390/ijms21155324

Wanandi S. I., 2018, Asian J. Pharm. Clin. Res., V11, P48

Wang D, 2024, CANCER BIOTHER RADIO, V39, P125, DOI 10.1089/cbr.2020.4283

Wang Q, 2012, INT J CLIN EXP PATHO, V5, P555

Wang WJ, 2022, MED GAS RES, V12, P1, DOI 10.4103/2045-9912.324589

Wang Y, 2020, ACTA PHARMACOL SIN B, V10, P207, DOI 10.1016/j.apsb.2019.08.001

Wang YG, 2015, ONCOL LETT, V10, P189, DOI 10.3892/ol.2015.3244

Ward NP, 2017, PLOS ONE, V12, DOI 10.1371/journal.pone.0180061

Wenzl T, 2011, RADIAT ONCOL, V6, DOI 10.1186/1748-717X-6-171

Wiestler B, 2013, ACTA NEUROPATHOL, V126, P443, DOI 10.1007/s00401-013-1156-z

Wilson JX, 1997, CAN J PHYSIOL PHARM, V75, P1149, DOI 10.1139/cjpp-75-10-11-1149

Wisastra R, 2014, CANCERS, V6, P1500, DOI 10.3390/cancers6031500

Wong SC, 2021, NUTRIENTS, V13, DOI 10.3390/nu13030950

Woroniecka Karolina, 2018, Oncotarget, V9, P35287, DOI 10.18632/oncotarget.26228

Wu DD, 2018, INT C NUMER SIMUL, P71, DOI 10.1109/NUSOD.2018.8570279

Xue T, 2021, MED GAS RES, V11, P155, DOI 10.4103/2045-9912.318861

Yakovlev VA, 2007, BIOCHEMISTRY-US, V46, P11671, DOI 10.1021/bi701107z

Yang JT, 2014, PLOS ONE, V9, DOI 10.1371/journal.pone.0094180

Yang TQ, 2017, ONCOTARGETS THER, V10, P4305, DOI 10.2147/OTT.S144014

Yao AQ, 2020, MOL NEUROBIOL, V57, P2889, DOI 10.1007/s12035-020-01928-z

Yousfi N, 2015, PLOS ONE, V10, DOI 10.1371/journal.pone.0120435

Yue YY, 2021, SCI REP-UK, V11, DOI 10.1038/s41598-021-88485-0

Zembrzuska K, 2019, ONCOL REP, V41, P2703, DOI 10.3892/or.2019.7064

Zeng XF, 2019, J NANOBIOTECHNOL, V17, DOI 10.1186/s12951-019-0483-1

Zhang FJ, 2010, PHARM BIOL, V48, P883, DOI 10.3109/13880200903311102

Zhang H, 2019, ONCOL LETT, V17, P1826, DOI 10.3892/ol.2018.9806

Zhang LP, 2020, PTERIDINES, V31, P142, DOI 10.1515/pteridines-2020-0019

Zhang Y., 2020, Frontiers in Genetics, V11

Zhang YM, 2013, J PINEAL RES, V55, P121, DOI 10.1111/jpi.12052

Zhou Y, 2020, ARCH PHARM RES, V43, P187, DOI 10.1007/s12272-020-01205-6

Zhu ZL, 2018, J NEUROCHEM, V144, P93, DOI 10.1111/jnc.14250

Ziegler DS, 2008, J CLIN INVEST, V118, P3109, DOI 10.1172/JCI34120

Zong H, 2012, EXPERT REV MOL DIAGN, V12, P383, DOI [10.1586/erm.12.30, 10.1586/ERM.12.30]

NR 238

TC 19

Z9 20

U1 7

U2 50

PU PERGAMON-ELSEVIER SCIENCE LTD

PI OXFORD

PA THE BOULEVARD, LANGFORD LANE, KIDLINGTON, OXFORD OX5 1GB, ENGLAND

SN 0197-0186

EI 1872-9754

J9 NEUROCHEM INT

JI Neurochem. Int.

PD MAR

PY 2022

VL 154

AR 105281

DI 10.1016/j.neuint.2022.105281

EA JAN 2022

PG 15

WC Biochemistry & Molecular Biology; Neurosciences

WE Science Citation Index Expanded (SCI-EXPANDED)

SC Biochemistry & Molecular Biology; Neurosciences & Neurology

GA 0O9ED

UT WOS:000783825300002

PM 35038460

DA 2025-04-09

ER

PT J

AU Wei, JW

Wang, ZF

Wang, WW

Liu, XG

Wan, JH

Yuan, YJ

Li, XY

Ma, LW

Liu, XZ

AF Wei, Jianwei

Wang, Zhengfeng

Wang, Weiwei

Liu, Xiaoge

Wan, Junhu

Yuan, Yongjie

Li, Xueyuan

Ma, Liwei

Liu, Xianzhi

TI Oxidative Stress Activated by Sorafenib Alters the Temozolomide

Sensitivity of Human Glioma Cells Through Autophagy and JAK2/STAT3-AIF

Axis

SO FRONTIERS IN CELL AND DEVELOPMENTAL BIOLOGY

LA English

DT Article

DE JAK2; STAT3; AIF; sorafenib; TMZ; glioma cells

ID HUMAN GLIOBLASTOMA CELLS; DOWN-REGULATION; HEPATOCELLULAR-CARCINOMA;

INDUCED APOPTOSIS; MITOCHONDRIAL; INHIBITION; DEATH; CYTOTOXICITY; AIF

AB The development of temozolomide (TMZ) resistance in glioma leads to poor patient prognosis. Sorafenib, a novel diaryl urea compound and multikinase inhibitor, has the ability to effectively cross the blood-brain barrier. However, the effect of sorafenib on glioma cells and the molecular mechanism underlying the ability of sorafenib to enhance the antitumor effects of TMZ remain elusive. Here, we found that sorafenib could enhance the cytotoxic effects of TMZ in glioma cells in vitro and in vivo. Mechanistically, the combination of sorafenib and TMZ induced mitochondrial depolarization and apoptosis inducing factor (AIF) translocation from mitochondria to nuclei, and this process was dependent on STAT3 inhibition. Moreover, the combination of sorafenib and TMZ inhibited JAK2/STAT3 phosphorylation and STAT3 translocation to mitochondria. Inhibition of STAT3 activation promoted the autophagy-associated apoptosis induced by the combination of sorafenib and TMZ. Furthermore, the combined sorafenib and TMZ treatment induced oxidative stress while reactive oxygen species (ROS) clearance reversed the treatment-induced inhibition of JAK2/STAT3. The results indicate that sorafenib enhanced the temozolomide sensitivity of human glioma cells by inducing oxidative stress-mediated autophagy and JAK2/STAT3-AIF axis.

C1 [Wei, Jianwei; Wang, Zhengfeng; Li, Xueyuan; Liu, Xianzhi] Zhengzhou Univ, Dept Neurosurg, Affiliated Hosp 1, Zhengzhou, Peoples R China.

[Wang, Weiwei] Zhengzhou Univ, Dept Pathol, Affiliated Hosp 1, Zhengzhou, Peoples R China.

[Liu, Xiaoge] Zhengzhou Univ, Dept Magnet Resonance Imaging, Affiliated Hosp 1, Zhengzhou, Peoples R China.

[Wan, Junhu; Ma, Liwei] Zhengzhou Univ, Dept Clin Lab, Affiliated Hosp 1, Zhengzhou, Peoples R China.

[Yuan, Yongjie] Zhengzhou Univ, Dept Intervent Neurol, Affiliated Hosp 1, Zhengzhou, Peoples R China.

C3 Zhengzhou University; Zhengzhou University; Zhengzhou University;

Zhengzhou University; Zhengzhou University

RP Liu, XZ (corresponding author), Zhengzhou Univ, Dept Neurosurg, Affiliated Hosp 1, Zhengzhou, Peoples R China.; Ma, LW (corresponding author), Zhengzhou Univ, Dept Clin Lab, Affiliated Hosp 1, Zhengzhou, Peoples R China.

EM liweimalg@126.com; xianzhiliu910@126.com

RI Li, Xueyuan/HZM-1127-2023; Wan, Junhu/V-3895-2019; Wei,

Jianwei/AAH-3502-2020

FU National Natural Science Foundation of China [82002751, U1804172];

Natural Science Foundation of Henan province of China [202300410392];

Science and Technology Project of Henan province of China [202102310442,

202102310112]

FX The present study was supported by grants from the National Natural

Science Foundation of China (Grant Nos. 82002751 and U1804172), the

Natural Science Foundation of Henan province of China (Grant No.

202300410392), the Science and Technology Project of Henan province of

China (Grant No. 2018020051), and the Science and Technology Project of

Henan province of China (Grant Nos. 202102310442 and 202102310112).

CR Booth L, 2020, BIOCHEM PHARMACOL, V178, DOI 10.1016/j.bcp.2020.114067

Bull VH, 2012, J PROTEOME RES, V11, P1609, DOI 10.1021/pr200790e

Cabral LKD, 2020, CANCERS, V12, DOI 10.3390/cancers12061576

Cao Y, 2020, J EXP CLIN CANC RES, V39, DOI 10.1186/s13046-020-01621-y

Chong DQ, 2015, BMC CANCER, V15, DOI 10.1186/s12885-015-1191-3

Choudhury S, 2010, CARDIOVASC RES, V85, P28, DOI 10.1093/cvr/cvp261

Collins KL, 2020, CANCERS, V12, DOI 10.3390/cancers12051152

Costa B, 2013, PLOS ONE, V8, DOI 10.1371/journal.pone.0072281

Crawley O, 2019, NAT COMMUN, V10, DOI 10.1038/s41467-019-12804-3

Ganipineni LP, 2018, J CONTROL RELEASE, V281, P42, DOI 10.1016/j.jconrel.2018.05.008

Garama DJ, 2015, MOL CELL BIOL, V35, P3646, DOI 10.1128/MCB.00541-15

Hage C, 2019, HEPATOLOGY, V70, P1280, DOI 10.1002/hep.30666

Hainsworth JD, 2010, CANCER-AM CANCER SOC, V116, P3663, DOI 10.1002/cncr.25275

He HC, 2020, J CELL MOL MED, V24, P50, DOI 10.1111/jcmm.14295

Hlavac M, 2019, CELL ONCOL, V42, P287, DOI 10.1007/s13402-019-00425-3

Hu L, 2020, CELL DEATH DIS, V11, DOI 10.1038/s41419-020-2476-2

Huang CY, 2013, INT J RADIAT ONCOL, V86, P456, DOI 10.1016/j.ijrobp.2013.01.025

Hung MH, 2014, WORLD J GASTROENTERO, V20, P15269, DOI 10.3748/wjg.v20.i41.15269

Ignarro RS, 2016, MOL CELL BIOCHEM, V418, P167, DOI 10.1007/s11010-016-2742-x

Jakubowicz-Gil J, 2017, PHARMACOL REP, V69, P779, DOI 10.1016/j.pharep.2017.03.008

Kim BH, 2020, CELLS-BASEL, V9, DOI 10.3390/cells9030722

Kuwana T, 2020, J BIOL CHEM, V295, P1623, DOI 10.1074/jbc.RA119.011635

Lan YL, 2020, CANCER BIOL MED, V17, P112, DOI 10.20892/j.issn.2095-3941.2019.0164

Li H, 2018, J HEMATOL ONCOL, V11, DOI 10.1186/s13045-018-0618-0

Li JW, 2020, TALANTA, V216, DOI 10.1016/j.talanta.2020.120983

Liu BY, 2020, CELL DEATH DIS, V11, DOI 10.1038/s41419-020-2540-y

Liu XY, 2015, MOL MED REP, V11, P1516, DOI 10.3892/mmr.2014.2811

Luo HM, 2020, J CELL MOL MED, V24, P6426, DOI 10.1111/jcmm.15288

Ma JB, 2020, CELL DEATH DIS, V11, DOI 10.1038/s41419-020-2671-1

Ma LW, 2019, J EXP CLIN CANC RES, V38, DOI 10.1186/s13046-019-1090-6

Macias E, 2014, J INVEST DERMATOL, V134, P1971, DOI 10.1038/jid.2014.68

Maycotte P, 2014, CANCER RES, V74, P2579, DOI 10.1158/0008-5472.CAN-13-3470

Miyazaki T, 2020, BRAIN TUMOR PATHOL, V37, P41, DOI 10.1007/s10014-020-00357-z

Nabors LB, 2011, NEURO-ONCOLOGY, V13, P1324, DOI 10.1093/neuonc/nor145

Nawara HM, 2020, CANCERS, V12, DOI 10.3390/cancers12061360

París-Coderch L, 2020, CELL DEATH DIS, V11, DOI 10.1038/s41419-020-02986-w

Perazzoli G, 2015, PLOS ONE, V10, DOI 10.1371/journal.pone.0140131

Radhakrishnan H, 2017, CANCER LETT, V403, P231, DOI 10.1016/j.canlet.2017.06.020

Riedel M, 2016, ONCOTARGET, V7, P61988, DOI 10.18632/oncotarget.11328

Siegelin MD, 2010, NEUROSCI LETT, V478, P165, DOI 10.1016/j.neulet.2010.05.009

Silva MC, 2020, NAT COMMUN, V11, DOI 10.1038/s41467-020-16984-1

Solà-Riera C, 2020, PLOS PATHOG, V16, DOI 10.1371/journal.ppat.1008297

Su YX, 2020, FRONT CELL DEV BIOL, V8, DOI 10.3389/fcell.2020.00362

Su YX, 2020, FRONT CELL DEV BIOL, V8, DOI 10.3389/fcell.2020.00274

Tang JB, 2011, NEURO-ONCOLOGY, V13, P471, DOI 10.1093/neuonc/nor011

Vicinanza M, 2020, DEV CELL, V53, P619, DOI 10.1016/j.devcel.2020.05.030

Xie L, 2018, INT J BIOL SCI, V14, P577, DOI 10.7150/ijbs.22220

Yan XH, 2018, BIOCHEM PHARMACOL, V150, P72, DOI 10.1016/j.bcp.2018.01.031

Yang HQ, 2020, INT J NANOMED, V15, P7791, DOI 10.2147/IJN.S267120

Yuan XP, 2015, EXP CELL RES, V330, P267, DOI 10.1016/j.yexcr.2014.09.006

Zhang CG, 2017, J BIOL CHEM, V292, P15105, DOI 10.1074/jbc.M117.783175

Zhang Q, 2020, CELL DEATH DIS, V11, DOI 10.1038/s41419-020-2696-5

Zhang XF, 2021, ECOTOX ENVIRON SAFE, V208, DOI 10.1016/j.ecoenv.2020.111391

Zhao HW, 2016, CANCER LETT, V374, P136, DOI 10.1016/j.canlet.2016.01.055

Zhou JP, 2020, CELL ONCOL, V43, P461, DOI 10.1007/s13402-020-00502-y

Zhu N, 2020, P NATL ACAD SCI USA, V117, P15047, DOI 10.1073/pnas.1910278117

Zimmerman MA, 2020, INT J MED SCI, V17, P2831, DOI 10.7150/ijms.40159

Zustovich F, 2013, ANTICANCER RES, V33, P3487

NR 58

TC 21

Z9 21

U1 0

U2 17

PU FRONTIERS MEDIA SA

PI LAUSANNE

PA AVENUE DU TRIBUNAL FEDERAL 34, LAUSANNE, CH-1015, SWITZERLAND

SN 2296-634X

J9 FRONT CELL DEV BIOL

JI Front. Cell. Dev. Biol.

PD JUN 14

PY 2021

VL 09

AR 660005

DI 10.3389/fcell.2021.660005

PG 17

WC Cell Biology; Developmental Biology

WE Science Citation Index Expanded (SCI-EXPANDED)

SC Cell Biology; Developmental Biology

GA TA5ND

UT WOS:000667294800001

PM 34277607

OA gold, Green Published

DA 2025-04-09

ER

PT J

AU Marconi, GD

Gallorini, M

Carradori, S

Guglielmi, P

Cataldi, A

Zara, S

AF Marconi, Guya Diletta

Gallorini, Marialucia

Carradori, Simone

Guglielmi, Paolo

Cataldi, Amelia

Zara, Susi

TI The Up-Regulation of Oxidative Stress as a Potential Mechanism of Novel

MAO-B Inhibitors for Glioblastoma Treatment

SO MOLECULES

LA English

DT Article

DE Glioblastoma; MAO-B inhibitors; oxidative stress; migration

ID MONOAMINE-OXIDASE-B; CELLULAR PROLIFERATION; CANCER-CELLS; GLIOMA;

MIGRATION; PARGYLINE; INVASION

AB Gliomas are malignant brain tumors characterized by rapid spread and growth into neighboring tissues and graded I-IV by the World Health Organization. Glioblastoma is the fastest growing and most devastating IV glioma. The aim of this paper is to evaluate the biological effects of two potent and selective Monoamine Oxidase B (MAO-B) inhibitors, Cmp3 and Cmp5, in C6 glioma cells and in CTX/TNA2 astrocytes in terms of cell proliferation, apoptosis occurrence, inflammatory events and cell migration. These compounds decrease C6 glioma cells viability sparing normal astrocytes. Cell cycle analysis, the Mitochondrial Membrane Potential (MMP) and Reactive Oxygen Species (ROS) production were detected, revealing that Cmp3 and Cmp5 induce a G1 or G2/M cell cycle arrest, as well as a MMP depolarization and an overproduction of ROS; moreover, they inhibit the expression level of inducible nitric oxide synthase 2, thus contributing to fatal drug-induced oxidative stress. Cmp5 notably reduces glioma cell migration via down-regulating Matrix Metalloproteinases 2 and 9. This study demonstrated that our novel MAO-B inhibitors increase the oxidative stress level resulting in a cell cycle arrest and markedly reduces glioma cells migration thus reinforcing the hypothesis of a critical role-played by MAO-B in mediating oncogenesis in high-grade gliomas.

C1 [Marconi, Guya Diletta; Gallorini, Marialucia; Carradori, Simone; Cataldi, Amelia; Zara, Susi] Univ G dAnnunzio, Dept Pharm, Via Vestini 31, I-66100 Chieti, Italy.

[Guglielmi, Paolo] Sapienza Univ Rome, Dept Drug Chem & Technol, Ple A Moro 5, I-00185 Rome, Italy.

C3 G d'Annunzio University of Chieti-Pescara; Sapienza University Rome

RP Carradori, S (corresponding author), Univ G dAnnunzio, Dept Pharm, Via Vestini 31, I-66100 Chieti, Italy.

EM guya.marconi@virgilio.it; marialucia.gallorini@unich.it;

simone.carradori@uniroma1.it; paolo.guglielmi@uniroma1.it;

amelia.cataldi@unich.it; susi.zara@unich.it

RI Guglielmi, Paolo/AAH-7911-2021; Carradori, Simone/S-4776-2019

OI Carradori, Simone/0000-0002-8698-9440; Gallorini,

Marialucia/0000-0002-2283-4159; GUGLIELMI, PAOLO/0000-0001-5701-6925;

Zara, Susi/0000-0003-1705-6943; CATALDI, Amelia/0000-0002-4989-6210

FU FABBR 2018; FAR2017; Cataldi RES

FX This research has been financed by Cataldi RES, FABBR 2018 Zara Susi and

FAR2017 Zara Susi.

CR Azzalin A, 2017, NEOPLASIA, V19, P364, DOI 10.1016/j.neo.2017.02.009

Callado LF, 2011, TUMOR CENT NERV SYST, V1, P53, DOI 10.1007/978-94-007-0344-5_6

Chandrika G, 2016, SCI REP-UK, V6, DOI 10.1038/srep22455

Finberg JPM, 2016, FRONT PHARMACOL, V7, DOI 10.3389/fphar.2016.00340

Gabilondo AM, 2008, NEUROCHEM INT, V52, P230, DOI 10.1016/j.neuint.2007.05.015

Gào X, 2017, ONCOTARGET, V8, P51888, DOI 10.18632/oncotarget.17128

He X, 2018, TECHNOL CANCER RES T, V17, DOI [DOI 10.1177/1533034618770305, 10.1177/153303461877030529756546]

Kimura H, 2000, BLOOD, V95, P189, DOI 10.1182/blood.V95.1.189.001k05_189_197

Kramer N, 2013, MUTAT RES-REV MUTAT, V752, P10, DOI 10.1016/j.mrrev.2012.08.001

Lan FM, 2016, INT J ONCOL, V48, P559, DOI 10.3892/ijo.2015.3271

Lee HT, 2013, ONCOL REP, V30, P1587, DOI 10.3892/or.2013.2635

Lee HT, 2012, MOL CELL TOXICOL, V8, P393, DOI 10.1007/s13273-012-0048-y

Liu H, 2015, TOXICOL LETT, V232, P349, DOI 10.1016/j.toxlet.2014.11.011

Meskelevicius D, 2016, ANTI-CANCER AGENT ME, V16, P1190, DOI 10.2174/1871520616666160204113217

Niu HF, 2018, J BUON, V23, P1840

Perazzoli G, 2015, PLOS ONE, V10, DOI 10.1371/journal.pone.0140131

Schumacker PT, 2006, CANCER CELL, V10, P175, DOI 10.1016/j.ccr.2006.08.015

Secci D, 2019, CURR ANAL CHEM, V15, P313, DOI 10.2174/1573411014666180502105225

Secci D, 2019, J ENZYM INHIB MED CH, V34, P597, DOI 10.1080/14756366.2019.1571272

Sun Y, 2009, CARCINOGENESIS, V30, P93, DOI 10.1093/carcin/bgn242

Tipton KF, 2004, CURR MED CHEM, V11, P1965, DOI 10.2174/0929867043364810

Tuettenberg J, 2006, CRIT REV ONCOL HEMAT, V59, P181, DOI 10.1016/j.critrevonc.2006.01.004

Ulasov I, 2014, BBA-REV CANCER, V1846, P113, DOI 10.1016/j.bbcan.2014.03.002

Vannini F, 2015, REDOX BIOL, V6, P334, DOI 10.1016/j.redox.2015.08.009

Webb AH, 2017, BMC CANCER, V17, DOI 10.1186/s12885-017-3418-y

Weller M, 2015, NAT REV DIS PRIMERS, V1, DOI 10.1038/nrdp.2015.17

Zhang JF, 2017, ONCOL REP, V38, P2033, DOI 10.3892/or.2017.5926

NR 27

TC 26

Z9 27

U1 0

U2 8

PU MDPI

PI BASEL

PA ST ALBAN-ANLAGE 66, CH-4052 BASEL, SWITZERLAND

SN 1420-3049

J9 MOLECULES

JI Molecules

PD MAY 2

PY 2019

VL 24

IS 10

AR 2005

DI 10.3390/molecules24102005

PG 16

WC Biochemistry & Molecular Biology; Chemistry, Multidisciplinary

WE Science Citation Index Expanded (SCI-EXPANDED)

SC Biochemistry & Molecular Biology; Chemistry

GA IC5GU

UT WOS:000470996600163

PM 31130597

OA gold, Green Published, Green Submitted

DA 2025-04-09

ER

PT J

AU Ye, ZPP

Ai, XL

Yang, KL

Yang, ZN

Fei, F

Liao, XL

Qiu, ZX

Gimple, RC

Yuan, HR

Huang, H

Gong, YQ

Xiao, CX

Yue, J

Huang, L

Saulnier, O

Wang, W

Zhang, PD

Dai, LZ

Wang, X

Wang, XX

Ha Ahn, Y

You, C

Xu, JG

Wan, XX

Taylor, MD

Zhao, LJ

Rich, JN

Zhou, ST

AF Ye, Zengpanpan

Ai, Xiaolin

Yang, Kailin

Yang, Zhengnan

Fei, Fan

Liao, Xiaoling

Qiu, Zhixin

Gimple, Ryan C.

Yuan, Huairui

Huang, Hao

Gong, Yanqiu

Xiao, Chaoxin

Yue, Jing

Huang, Liang

Saulnier, Olivier

Wang, Wei

Zhang, Peidong

Dai, Lunzhi

Wang, Xin

Wang, Xiuxing

Ha Ahn, Young

You, Chao

Xu, Jianguo

Wan, Xiaoxiao

Taylor, Michael D.

Zhao, Linjie

Rich, Jeremy N.

Zhou, Shengtao

TI Targeting Microglial Metabolic Rewiring Synergizes with

Immune-Checkpoint Blockade Therapy for Glioblastoma

SO CANCER DISCOVERY

LA English

DT Article

ID T-CELLS; MACROPHAGE POLARIZATION; OXIDATIVE STRESS; CANCER;

TEMOZOLOMIDE; REGRESSION; RECEPTORS; RADIATION; REVEALS; PACKAGE

AB Glioblastoma (GBM) constitutes the most lethal primary brain tumor for which immunotherapy has provided limited benefit. The unique brain immune landscape is reflected in a complex tumor immune microenvironment (TIME) in GBM. Here, single-cell sequencing of the GBM TIME revealed that microglia were under severe oxidative stress, which induced nuclear recep-tor subfamily 4 group A member 2 (NR4A2)-dependent transcriptional activity in microglia. Heterozy-gous Nr4a2 (Nr4a2+/-) or CX3CR1+ myeloid cell-specific Nr4a2 (Nr4a2fl/flCx3cr1Cre) genetic targeting reshaped microglia plasticity in vivo by reducing alternatively activated microglia and enhancing anti-gen presentation capacity for CD8+ T cells in GBM. In microglia, NR4A2 activated squalene monooxy-genase (SQLE) to dysregulate cholesterol homeostasis. Pharmacologic NR4A2 inhibition attenuated the protumorigenic TIME, and targeting the NR4A2 or SQLE enhanced the therapeutic effi cacy of immune-checkpoint blockade in vivo . Collectively, oxidative stress promotes tumor growth through NR4A2-SQLE activity in microglia, informing novel immune therapy paradigms in brain cancer.SIGNIFICANCE: Metabolic reprogramming of microglia in GBM informs synergistic vulnerabilities for immune-checkpoint blockade therapy in this immunologically cold brain tumor.

C1 [Ye, Zengpanpan; Ai, Xiaolin; Yang, Zhengnan; Xiao, Chaoxin; Yue, Jing; Huang, Liang; Zhang, Peidong; You, Chao; Xu, Jianguo; Zhou, Shengtao] Sichuan Univ, West China Hosp 2, West China Hosp, Dept Neurosurg,Dept Obstet & Gynecol,Key Lab Birth, Chengdu, Peoples R China.

[Ye, Zengpanpan; Ai, Xiaolin; Yang, Zhengnan; Xiao, Chaoxin; Yue, Jing; Huang, Liang; Zhang, Peidong; You, Chao; Xu, Jianguo; Zhou, Shengtao] Collaborat Innovat Ctr, Chengdu, Peoples R China.

[Yang, Kailin] Cleveland Clin, Taussig Canc Ctr, Dept Radiat Oncol, Cleveland, OH USA.

[Fei, Fan; Liao, Xiaoling] Sichuan Peoples Hosp, Dept Neurosurg, Chengdu, Sichuan, Peoples R China.

[Qiu, Zhixin; Yuan, Huairui; Zhao, Linjie; Rich, Jeremy N.] Univ Pittsburgh, Med Ctr Hillman Canc Ctr, Pittsburgh, PA 15232 USA.

[Gimple, Ryan C.] Case Western Reserve Univ, Dept Pathol, Cleveland, OH USA.

[Huang, Hao] Southeast Univ, Sch Biol Sci & Med Engn, Nanjing, Peoples R China.

[Gong, Yanqiu; Dai, Lunzhi] Sichuan Univ, West China Hosp, Natl Clin Res Ctr Geriatr, Chengdu, Peoples R China.

[Gong, Yanqiu; Dai, Lunzhi] Sichuan Univ, West China Hosp, Dept Gen Practice, State Key Lab Biotherapy, Chengdu, Peoples R China.

[Gong, Yanqiu; Dai, Lunzhi] Collaborat Innovat Ctr Biotherapy, Chengdu, Peoples R China.

[Saulnier, Olivier; Taylor, Michael D.] Hosp Sick Children, Dev & Stem Cell Biol Program, Toronto, ON, Canada.

[Saulnier, Olivier; Taylor, Michael D.] Hosp Sick Children, Arthur & Sonia Labatt Brain Tumour Res Ctr, Toronto, ON, Canada.

[Wang, Wei] Huzhou Matern & Child Hlth Care Hosp, Dept Gynecol, Huzhou, Zhejiang, Peoples R China.

Chinese Univ Hong Kong, Prince Wales Hosp, Dept Surg, Shatin, Hong Kong, Peoples R China.

Nanjing Med Univ, Sch Basic Med Sci, Nanjing, Jiangsu, Peoples R China.

Korea Res Inst Biosci & Biotechnol KRIBB, Immunotherapy Convergence Res Ctr, Daejeon, South Korea.

Washington Univ, Sch Med, Dept Pathol & Immunol, Div Immunobiol, St Louis, MO USA.

Hosp Sick Children, Div Neurosurg, Toronto, ON, Canada.

Univ Pittsburgh, Med Ctr, Dept Neurol, Pittsburgh, PA USA.

C3 Sichuan University; Cleveland Clinic Foundation; Pennsylvania

Commonwealth System of Higher Education (PCSHE); University of

Pittsburgh; University System of Ohio; Case Western Reserve University;

Southeast University - China; Sichuan University; Sichuan University;

University of Toronto; Hospital for Sick Children (SickKids); University

of Toronto; Hospital for Sick Children (SickKids); Chinese University of

Hong Kong; Prince of Wales Hospital; Nanjing Medical University; Korea

Research Institute of Bioscience & Biotechnology (KRIBB); Washington

University (WUSTL); University of Toronto; Hospital for Sick Children

(SickKids); Pennsylvania Commonwealth System of Higher Education

(PCSHE); University of Pittsburgh

RP Zhou, ST (corresponding author), Sichuan Univ, West China Hosp 2, West China Hosp, Dept Neurosurg,Dept Obstet & Gynecol,Key Lab Birth, Chengdu, Peoples R China.; Zhou, ST (corresponding author), Collaborat Innovat Ctr, Chengdu, Peoples R China.; Zhao, LJ; Rich, JN (corresponding author), Univ Pittsburgh, Med Ctr Hillman Canc Ctr, Pittsburgh, PA 15232 USA.

EM linjiezhao89@gmail.com; drjeremyrich@gmail.com; taotaovip2005@163.com

RI Rich, Jeremy/A-9501-2012; Yang, Zhengnan/ABG-4280-2021; fei,

fan/KZU-0743-2024; Qiu, Zhixin/CAG-1575-2022; Wang,

Xiuxing/HGU-0583-2022; Zhang, Peidong/AAA-7564-2022; Ai,

Xiaolin/S-5709-2019; Huang, Liang/H-2671-2015; Gimple,

Ryan/AAU-8944-2020; Wang, Xin/N-8865-2018; jiang, lei/IWE-1124-2023;

Yang, Kailin/L-2205-2013

OI Zhou, Shengtao/0000-0001-8322-5536; Liao, xiaoling/0000-0001-6779-3322;

Gimple, Ryan/0000-0002-7189-3243; Fei, Fan/0000-0003-4437-3186;

Saulnier, Olivier/0000-0003-4111-1017; Huang, Hao/0000-0002-5570-6145;

Wang, Xiuxing/0000-0001-7115-729X; yang, zhengnan/0000-0003-1076-3530;

Yang, Kailin/0000-0001-5968-6738; Qiu, Zhixin/0000-0001-7103-8004; Dai,

Lunzhi/0000-0002-3003-8910

FU National Natural Science Foundation of China [82273255, 81822034,

81821002, 81773119, 82103127, 82202889]; National Key Research and

Development Program of China [2022YFA1106600, 2017YFA0106800,

2018YFA0109200]; Sichuan Science-Technology Project [22ZYZYTS0070,

2019YFH0144]; West China Second Hospital, Sichuan University [KS021,

K1907]; NIH [CA197718, NS103434, CA268634, CA238662]; Computational

Genomic Epidemiology of Cancer Program at Case Comprehensive Cancer

Center [T32CA094186]; ASCO Conquer Cancer Foundation; RSNA Research

Resident Grant

FX The authors thank Bo Peng from Fudan University, China, for helpful

discussion and constructive suggestions. This work was supported by the

National Natural Science Foundation of China (grants #82273255,

#81822034, #81821002, and #81773119 to S. Zhou; grant #82103127 to Z.

Ye; and grant #82202889 to X. Ai) , the National Key Research and

Development Program of China (grants #2022YFA1106600, #2017YFA0106800,

and #2018YFA0109200 to S. Zhou) , Sichuan Science-Technology Project

(grants #22ZYZYTS0070 and #2019YFH0144 to S. Zhou) , Direct Scientific

Research Grants from West China Second Hospital, Sichuan University

(grants #KS021 and #K1907 to S. Zhou) , the NIH (grants CA197718,

NS103434, CA268634, and CA238662 to J.N. Rich) , the Computational

Genomic Epidemiology of Cancer Program at Case Comprehensive Cancer

Center (grant #T32CA094186 to K. Yang) , a Young Investigator Award in

Glioblastoma from the ASCO Conquer Cancer Foundation (to K. Yang) , and

an RSNA Research Resident Grant (to K. Yang) . The publication costs of

this article were defrayed in part by the payment of publication fees.

Therefore, and solely to indicate this fact, this article is hereby

marked advertisement in accordance with 18 USC section 1734.

CR Aldape K, 2019, NAT REV CLIN ONCOL, V16, P509, DOI 10.1038/s41571-019-0177-5

Antunes ARP, 2021, NAT NEUROSCI, V24, P595, DOI 10.1038/s41593-020-00789-y

Brown AJ, 2019, NAT COMMUN, V10, DOI 10.1038/s41467-019-08866-y

Brown CE, 2016, NEW ENGL J MED, V375, P2561, DOI 10.1056/NEJMoa1610497

Chen J, 2019, NATURE, V567, P530, DOI 10.1038/s41586-019-0985-x

Chen YX, 2017, GIGASCIENCE, V7, DOI 10.1093/gigascience/gix120

Chin L, 2008, NATURE, V455, P1061, DOI 10.1038/nature07385

Cloughesy TF, 2019, NAT MED, V25, P477, DOI 10.1038/s41591-018-0337-7

Darmanis S, 2017, CELL REP, V21, P1399, DOI 10.1016/j.celrep.2017.10.030

Desjardins A, 2018, NEW ENGL J MED, V379, P150, DOI 10.1056/NEJMoa1716435

Dobin A, 2013, BIOINFORMATICS, V29, P15, DOI 10.1093/bioinformatics/bts635

Doi Y, 2008, P NATL ACAD SCI USA, V105, P8381, DOI 10.1073/pnas.0803454105

Dumas AA, 2020, EMBO J, V39, DOI 10.15252/embj.2019103790

Fang WR, 2018, THERANOSTICS, V8, P3530, DOI 10.7150/thno.24475

Felsenstein M, 2020, CANCERS, V12, DOI 10.3390/cancers12071882

Field CS, 2020, CELL METAB, V31, P422, DOI 10.1016/j.cmet.2019.11.021

Forman HJ, 2021, NAT REV DRUG DISCOV, V20, P689, DOI 10.1038/s41573-021-00233-1

Gao XH, 2021, ADV MATER, V33, DOI 10.1002/adma.202006116

Garcia-Bermudez J, 2019, NATURE, V567, P118, DOI 10.1038/s41586-019-0945-5

Gimple RC, 2019, CANCER DISCOV, V9, P1248, DOI 10.1158/2159-8290.CD-19-0061

Ginhoux F, 2010, SCIENCE, V330, P841, DOI 10.1126/science.1194637

Goldmann T, 2016, NAT IMMUNOL, V17, P797, DOI 10.1038/ni.3423

Hanahan D, 2022, CANCER DISCOV, V12, P31, DOI 10.1158/2159-8290.CD-21-1059

Hao YH, 2021, CELL, V184, P3573, DOI 10.1016/j.cell.2021.04.048

Hara T, 2021, CANCER CELL, V39, P779, DOI 10.1016/j.ccell.2021.05.002

Hayes JD, 2020, CANCER CELL, V38, P167, DOI 10.1016/j.ccell.2020.06.001

Hibino S, 2018, CANCER RES, V78, P3027, DOI 10.1158/0008-5472.CAN-17-3102

Hilf N, 2019, NATURE, V565, P240, DOI 10.1038/s41586-018-0810-y

Hodges TR, 2017, NEURO-ONCOLOGY, V19, P1047, DOI 10.1093/neuonc/nox026

Hoeffel G, 2015, IMMUNITY, V42, P665, DOI 10.1016/j.immuni.2015.03.011

Hong ZP, 2022, CANCER RES, V82, P1298, DOI 10.1158/0008-5472.CAN-21-2229

Jarry U, 2013, EUR J IMMUNOL, V43, P1173, DOI 10.1002/eji.201243040

Jing CY, 2021, ONCOGENE, V40, P2910, DOI 10.1038/s41388-021-01705-9

Johnson KC, 2021, NAT GENET, V53, P1456, DOI 10.1038/s41588-021-00926-8

Kalogirou C, 2021, NAT COMMUN, V12, DOI 10.1038/s41467-021-25325-9

Kim JE, 2017, CLIN CANCER RES, V23, P124, DOI 10.1158/1078-0432.CCR-15-1535

Klemm F, 2020, CELL, V181, P1643, DOI 10.1016/j.cell.2020.05.007

Kuleshov MV, 2016, NUCLEIC ACIDS RES, V44, pW90, DOI 10.1093/nar/gkw377

Langmead B, 2012, NAT METHODS, V9, P357, DOI [10.1038/NMETH.1923, 10.1038/nmeth.1923]

Le WD, 2003, NAT GENET, V33, P85, DOI 10.1038/ng1066

Lee EQ, 2015, CLIN CANCER RES, V21, P3610, DOI 10.1158/1078-0432.CCR-14-3220

Li CG, 2022, GUT, V71, P2253, DOI 10.1136/gutjnl-2021-325851

Liu DB, 2018, SCI TRANSL MED, V10, DOI 10.1126/scitranslmed.aap9840

Liu HL, 2021, CELL REP, V36, DOI 10.1016/j.celrep.2021.109718

Liu ZY, 2022, J CLIN INVEST, V132, DOI 10.1172/JCI159229

Lu-Emerson C, 2015, J CLIN ONCOL, V33, P1197, DOI 10.1200/JCO.2014.55.9575

Luo M, 2018, CELL METAB, V28, P69, DOI 10.1016/j.cmet.2018.06.006

Mahoney CE, 2019, NAT COMMUN, V10, DOI 10.1038/s41467-018-07959-4

Maj T, 2017, NAT IMMUNOL, V18, P1332, DOI 10.1038/ni.3868

Martínez-Reyes I, 2021, NAT REV CANCER, V21, P669, DOI 10.1038/s41568-021-00378-6

McEvoy C, 2017, FRONT IMMUNOL, V8, DOI 10.3389/fimmu.2017.00007

Montarolo F, 2019, TRANSL PSYCHIAT, V9, DOI 10.1038/s41398-019-0544-0

Neftel C, 2019, CELL, V178, P835, DOI 10.1016/j.cell.2019.06.024

Ochocka N, 2021, NAT COMMUN, V12, DOI 10.1038/s41467-021-21407-w

Ostrom QT, 2017, NEURO-ONCOLOGY, V19, pV1, DOI 10.1093/neuonc/nox158

Pei LM, 2006, NAT MED, V12, P1048, DOI 10.1038/nm1471

Pyonteck SM, 2013, NAT MED, V19, P1264, DOI 10.1038/nm.3337

Reardon DA, 2020, JAMA ONCOL, V6, P1003, DOI 10.1001/jamaoncol.2020.1024

Saha D, 2017, CANCER CELL, V32, P253, DOI 10.1016/j.ccell.2017.07.006

Sarkar S, 2014, NAT NEUROSCI, V17, P46, DOI 10.1038/nn.3597

Sawant DV, 2019, NAT IMMUNOL, V20, P724, DOI 10.1038/s41590-019-0346-9

Schalper KA, 2019, NAT MED, V25, P470, DOI 10.1038/s41591-018-0339-5

Sekiya T, 2013, NAT IMMUNOL, V14, P230, DOI 10.1038/ni.2520

Sekiya T, 2011, NAT COMMUN, V2, DOI 10.1038/ncomms1272

Sheng JP, 2015, IMMUNITY, V43, P382, DOI 10.1016/j.immuni.2015.07.016

Shi RC, 2020, MEDCOMM, V1, P47, DOI 10.1002/mco2.6

Stupp R, 2009, LANCET ONCOL, V10, P459, DOI 10.1016/S1470-2045(09)70025-7

Su H, 2021, CANCER CELL, V39, P678, DOI 10.1016/j.ccell.2021.02.016

Sun HS, 2021, EMBO REP, V22, DOI 10.15252/embr.202152537

Takahashi N, 2018, CANCER CELL, V33, P985, DOI 10.1016/j.ccell.2018.05.001

Villa GR, 2016, CANCER CELL, V30, P683, DOI 10.1016/j.ccell.2016.09.008

Wang J, 2016, BMC CANCER, V16, DOI 10.1186/s12885-016-2291-4

Weinberg SE, 2019, NATURE, V565, P495, DOI 10.1038/s41586-018-0846-z

Weller M, 2015, NAT REV DIS PRIMERS, V1, DOI 10.1038/nrdp.2015.17

Wiel C, 2019, CELL, V178, P330, DOI 10.1016/j.cell.2019.06.005

Willems S, 2020, COMMUN CHEM, V3, DOI 10.1038/s42004-020-0331-0

Xie Y, 2021, JCI INSIGHT, V6, DOI 10.1172/jci.insight.150861

Yang RS, 2020, ONCOGENE, V39, P6340, DOI 10.1038/s41388-020-01439-0

Yang W, 2016, NATURE, V531, P651, DOI 10.1038/nature17412

Yang ZN, 2021, SCI ADV, V7, DOI 10.1126/sciadv.abb0737

Yeini E, 2021, NAT COMMUN, V12, DOI 10.1038/s41467-021-22186-0

Yoshioka H, 2020, P NATL ACAD SCI USA, V117, P7150, DOI 10.1073/pnas.1915923117

Yu GC, 2015, BIOINFORMATICS, V31, P2382, DOI 10.1093/bioinformatics/btv145

Yu GC, 2012, OMICS, V16, P284, DOI 10.1089/omi.2011.0118

Yu K, 2020, NATL SCI REV, V7, P1306, DOI 10.1093/nsr/nwaa099

Zabala A, 2018, EMBO MOL MED, V10, DOI 10.15252/emmm.201708743

Zagani R, 2009, GASTROENTEROLOGY, V137, P1358, DOI 10.1053/j.gastro.2009.06.039

Zhail K, 2021, NAT CANCER, V2, P1136, DOI 10.1038/s43018-021-00267-9

Zhao LJ, 2018, GENOME BIOL, V19, DOI 10.1186/s13059-018-1412-6

Zhao LJ, 2017, CANCER RES, V77, P1369, DOI 10.1158/0008-5472.CAN-16-1615

NR 90

TC 40

Z9 40

U1 9

U2 64

PU AMER ASSOC CANCER RESEARCH

PI PHILADELPHIA

PA 615 CHESTNUT ST, 17TH FLOOR, PHILADELPHIA, PA 19106-4404 USA

SN 2159-8274

EI 2159-8290

J9 CANCER DISCOV

JI Cancer Discov.

PD APR

PY 2023

VL 13

IS 4

BP 974

EP 1001

DI 10.1158/2159-8290.CD-22-0455

PG 28

WC Oncology

WE Science Citation Index Expanded (SCI-EXPANDED)

SC Oncology

GA E7MG0

UT WOS:000977335700001

PM 36649564

OA Green Submitted, Green Accepted

DA 2025-04-09

ER

PT J

AU Gilbert, MR

Liu, YX

Neltner, J

Pu, H

Morris, A

Sunkara, M

Pittman, T

Kyprianou, N

Horbinski, C

AF Gilbert, Misty R.

Liu, Yinxing

Neltner, Janna

Pu, Hong

Morris, Andrew

Sunkara, Manjula

Pittman, Thomas

Kyprianou, Natasha

Horbinski, Craig

TI Autophagy and oxidative stress in gliomas with IDH1 mutations

SO ACTA NEUROPATHOLOGICA

LA English

DT Article

DE IDH1; Autophagy; Oxidative stress; Apoptosis; Glioma

ID ISOCITRATE DEHYDROGENASE 1; IN-VIVO; D-2-HYDROXYGLUTARIC ACID; CELLS;

EXPRESSION; GROWTH

AB IDH1 mutations in gliomas associate with longer survival. Prooxidant and antiproliferative effects of IDH1 mutations and its d-2-hydroxyglutarate (2-HG) product have been described in vitro, but inconsistently observed. It is also unclear whether overexpression of mutant IDH1 in wild-type cells accurately phenocopies the effects of endogenous IDH1-mutations on tumor apoptosis and autophagy. Herein we investigated the effects of 2-HG and mutant IDH1 overexpression on proliferation, apoptosis, oxidative stress, and autophagy in IDH1 wild-type glioma cells, and compared those results with patient-derived tumors. 2-HG reduced viability and proliferation of U87MG and LN18 cells, triggered apoptosis in LN18 cells, and autophagy in U87MG cells. In vitro studies and flank xenografts of U87MG cells overexpressing R132H IDH1 exhibited increased oxidative stress, including increases of both manganese superoxide dismutase (MnSOD) and p62. Patient-derived IDH1-mutant tumors showed no significant differences in apoptosis or autophagy, but showed p62 accumulation and actually trended toward reduced MnSOD expression. These data indicate that mutant IDH1 and 2-HG can induce oxidative stress, autophagy, and apoptosis, but these effects vary greatly according to cell type.

C1 [Gilbert, Misty R.; Liu, Yinxing; Neltner, Janna; Kyprianou, Natasha; Horbinski, Craig] Univ Kentucky, Dept Pathol & Lab Med, Lexington, KY 40536 USA.

[Pu, Hong; Morris, Andrew; Sunkara, Manjula; Kyprianou, Natasha; Horbinski, Craig] Univ Kentucky, Dept Mol & Cellular Biochem, Lexington, KY 40536 USA.

[Pittman, Thomas] Univ Kentucky, Dept Neurosurg, Lexington, KY 40536 USA.

C3 University of Kentucky; University of Kentucky; University of Kentucky

RP Horbinski, C (corresponding author), Univ Kentucky, Dept Pathol & Lab Med, 307 Combs Bldg, Lexington, KY 40536 USA.

EM craig.horbinski@uky.edu

RI Gilbert, Mark/J-7494-2016; sunkara, Manjula/H-7944-2016; sunkara,

Manjula/G-9326-2013

OI sunkara, Manjula/0000-0001-6151-1929

FU Peter and Carmen Lucia Buck Training Program in Translational Clinical

Oncology; University of Kentucky College of Medicine Physician Scientist

Program; Markey Cancer Center Free Radical Biology in Cancer (FRBC)

Shared Resource Facility; NIH [S10 RR026827-01A1]; University of

Kentucky Markey Cancer Center [P30CA177558]; [K08 CA155764]; [2P20

RR020171 COBRE]

FX C.H. was supported by K08 CA155764 (National Cancer Institute), 2P20

RR020171 COBRE pilot grant (National Institute of General Medical

Sciences), The Peter and Carmen Lucia Buck Training Program in

Translational Clinical Oncology, and the University of Kentucky College

of Medicine Physician Scientist Program. The Markey Biospecimen and

Tissue Procurement (BSTP) Shared Resource Facility facilitated the

construction of tissue microarrays and immunohistochemical studies.

Special thanks to Dana Napier for her excellent histologic expertise.

Study data were collected and managed using REDCap electronic data

capture tools hosted at the University of Kentucky. This research was

also supported by the Markey Cancer Center Free Radical Biology in

Cancer (FRBC) Shared Resource Facility. Flow cytometry and cell sorting

was carried out at the University of Kentucky Flow Cytometry and Cell

Sorting (FCCS) Core Facility, which is supported in part by the Office

of the Vice President for Research, the Markey Cancer Center and a grant

from the NIH Shared Instrument Program (S10 RR026827-01A1). The BSTP,

FRBC, and FCCS Shared Resource Facilities are all supported by the

University of Kentucky Markey Cancer Center (P30CA177558). We thank Dr.

Hai Yan of Duke University Medical Center for supplying us with

pEGFP-N1-IDH1 and pEGFP-N1-IDH1R132H plasmids. We thank Dr. Haining Zhu

and Dr. Jozsef Gal, Department of Molecular and Cellular Biochemistry of

the University of Kentucky, for providing the GFP-LC3 plasmid. We thank

Dr. Daret St. Clair, Department of Toxicology, for her excellent

suggestions involving MnSOD experiments. We also thank Drs. Jeremy Rich

and Monica Venere from the Cleveland Clinic Foundation for their

generous help and training with the patient-derived glioma cultures.

CR Bleeker FE, 2010, ACTA NEUROPATHOL, V119, P487, DOI 10.1007/s00401-010-0645-6

Bralten LBC, 2011, ANN NEUROL, V69, P455, DOI 10.1002/ana.22390

Brauburger K, 2011, J INHERIT METAB DIS, V34, P477, DOI 10.1007/s10545-010-9268-2

Cherra SJ, 2010, NEUROPATH APPL NEURO, V36, P125, DOI [10.1111/j.1365-2990.2009.01062.x, 10.1111/j.1365-2990.2010.01062.x]

Choi AMK, 2013, NEW ENGL J MED, V368, P1845, DOI [10.1056/NEJMra1205406, 10.1056/NEJMc1303158]

Chowdhury R, 2011, EMBO REP, V12, P463, DOI 10.1038/embor.2011.43

Dang L, 2009, NATURE, V462, P739, DOI 10.1038/nature08617

Dhar SK, 2012, FREE RADICAL BIO MED, V52, P2209, DOI 10.1016/j.freeradbiomed.2012.03.009

Haapasalo H, 2003, BRAIN PATHOL, V13, P155

Horbinski C, 2013, ACTA NEUROPATHOL, V125, P621, DOI 10.1007/s00401-013-1106-9

Ishii N, 1999, BRAIN PATHOL, V9, P469, DOI 10.1111/j.1750-3639.1999.tb00536.x

Jane EP, 2011, MOL CANCER THER, V10, P198, DOI 10.1158/1535-7163.MCT-10-0725

Jin GL, 2013, CANCER RES, V73, P496, DOI 10.1158/0008-5472.CAN-12-2852

Jin GL, 2011, PLOS ONE, V6, DOI 10.1371/journal.pone.0016812

Klink B, 2013, PLOS ONE, V8, DOI 10.1371/journal.pone.0059773

Klionsky DJ, 2008, AUTOPHAGY, V4, P151, DOI 10.4161/auto.5338

Ladoire S, 2012, AUTOPHAGY, V8, P1175, DOI 10.4161/auto.20353

Latini A, 2005, MOL GENET METAB, V86, P188, DOI 10.1016/j.ymgme.2005.05.002

Latini A, 2003, EUR J NEUROSCI, V17, P2017, DOI 10.1046/j.1460-9568.2003.02639.x

Lazovic J, 2012, NEURO-ONCOLOGY, V14, P1465, DOI 10.1093/neuonc/nos258

Leonardi R, 2012, J BIOL CHEM, V287, P14615, DOI 10.1074/jbc.C112.353946

Li SC, 2013, NEURO-ONCOLOGY, V15, P57, DOI 10.1093/neuonc/nos261

Li XY, 2013, J HEMATOL ONCOL, V6, DOI 10.1186/1756-8722-6-19

Losman JA, 2013, SCIENCE, V339, P1621, DOI 10.1126/science.1231677

Luchman HA, 2012, NEURO-ONCOLOGY, V14, P184, DOI 10.1093/neuonc/nor207

Metallo CM, 2012, NATURE, V481, P380, DOI 10.1038/nature10602

Mohrenz IV, 2013, APOPTOSIS, V18, P1416, DOI 10.1007/s10495-013-0877-8

Mullen AR, 2012, NATURE, V481, P385, DOI 10.1038/nature10642

Nezis IP, 2012, ANTIOXID REDOX SIGN, V17, P786, DOI 10.1089/ars.2011.4394

Piaskowski S, 2011, BRIT J CANCER, V104, P968, DOI 10.1038/bjc.2011.27

Poon HF, 2004, NEUROSCIENCE, V126, P915, DOI 10.1016/j.neuroscience.2004.04.046

Reitman ZJ, 2011, P NATL ACAD SCI USA, V108, P3270, DOI 10.1073/pnas.1019393108

Shibata T, 2011, AM J PATHOL, V178, P1395, DOI 10.1016/j.ajpath.2010.12.011

Struys EA, 2004, CLIN CHEM, V50, P1391, DOI 10.1373/clinchem.2004.033399

Sultana R, 2008, METHOD ENZYMOL, V440, P309, DOI 10.1016/S0076-6879(07)00820-8

Valadez JG, 2013, CANCER LETT, V328, P297, DOI 10.1016/j.canlet.2012.10.002

Wise DR, 2011, P NATL ACAD SCI USA, V108, P19611, DOI 10.1073/pnas.1117773108

Xu W, 2011, CANCER CELL, V19, P17, DOI 10.1016/j.ccr.2010.12.014

Zhu J, 2013, J MOL NEUROSCI, V50, P165, DOI 10.1007/s12031-012-9890-6

NR 39

TC 59

Z9 64

U1 1

U2 15

PU SPRINGER

PI NEW YORK
[truncated: 3,734,866 more chars]
