# Supplementary material for: Recent status and trends regarding oxidative stress in gliomas (2013 - 2025): a systematic review and bibliometric analysis
Source: Front Oncol. 2025 May 16;15:1586515. doi: 10.3389/fonc.2025.1586515 (PMC12122519; doi:10.3389/fonc.2025.1586515)
Supplement: Supplementary file 3 [file Table3.docx]

FN Clarivate Analytics Web of Science

VR 1.0

PT J

AU Li, GZ

Wang, ZQ

Gao, BX

Dai, K

Niu, XW

Li, X

Wang, YJ

Li, LY

Wu, X

Li, HY

Yu, ZQ

Wang, Z

Chen, G

AF Li, Guangzhao

Wang, Zongqi

Gao, Bixi

Dai, Kun

Niu, Xiaowang

Li, Xiang

Wang, Yunjiang

Li, Longyuan

Wu, Xin

Li, Haiying

Yu, Zhengquan

Wang, Zhong

Chen, Gang

TI ANKZF1 knockdown inhibits glioblastoma progression by promoting

intramitochondrial protein aggregation through mitoRQC

SO CANCER LETTERS

LA English

DT Article

DE Glioblastoma; Ribosome related quality control; CAT-Tail; Ankyrin repeat

and zinc-finger domain-con; taining protein 1; Mitochondrial unfolded

protein response; Protein aggresome

ID QUALITY-CONTROL; MITOCHONDRIAL DYSFUNCTION; HYDROGEN-PEROXIDE; OXIDATIVE

STRESS; IMPORT; MECHANISMS; PATHWAY; RQC

AB Protein homeostasis is fundamental to the development of tumors. Ribosome-associated quality-control (RQC) is able to add alanine and threonine to the stagnant polypeptide chain C-terminal (CAT-tail) when protein translation is hindered, while Ankyrin repeat and zinc-finger domain-containing-protein 1 (ANKZF1) can counteract the formation of the CAT-tail, preventing the aggregation of polypeptide chains. In particular, ANKZF1 plays an important role in maintaining mitochondrial protein homeostasis by mitochondrial RQC (mitoRQC) after translation stagnation of precursor proteins targeting mitochondria. However, the role of ANKZF1 in glioblastoma is unclear. Therefore, the current study was aimed to investigate the effects of ANKZF1 in glioblastoma cells and a nude mouse glioblastoma xenograft model. Here, we reported that knockdown of ANKZF1 in glioblastoma cells resulted in the accumulation of CAT-tail in mitochondria, leading to the activated mitochondrial unfolded protein response (UPRmt) and inhibits glioblastoma malignant progression. Excessive CAT-tail sequestered mitochondrial chaperones HSP60, mtHSP70 and proteases LONP1 as well as mitochondrial respiratory chain subunits ND1, Cytb, mtCO2 and ATP6, leading to mitochondrial oxidative phosphorylation dysfunction, membrane potential impairment, and mitochondrial apoptotic pathway activation. Our study highlights ANKZF1 as a valuable target for glioblastoma intervention and provides an innovative insight for the treatment of glioblastoma through the regulating of mitochondrial protein homeostasis.

C1 [Li, Guangzhao; Wang, Zongqi; Gao, Bixi; Dai, Kun; Niu, Xiaowang; Li, Xiang; Wang, Yunjiang; Li, Longyuan; Wu, Xin; Li, Haiying; Yu, Zhengquan; Wang, Zhong; Chen, Gang] Soochow Univ, Affiliated Hosp 1, Dept Neurosurg, 188 Shizi St, Suzhou 215006, Peoples R China.

[Li, Guangzhao; Wang, Zongqi; Gao, Bixi; Dai, Kun; Niu, Xiaowang; Li, Xiang; Wang, Yunjiang; Li, Longyuan; Wu, Xin; Li, Haiying; Yu, Zhengquan; Wang, Zhong; Chen, Gang] Soochow Univ, Affiliated Hosp 1, Brain & Nerve Res Lab, 188 Shizi St, Suzhou 215006, Peoples R China.

[Li, Guangzhao; Wang, Zongqi; Gao, Bixi; Dai, Kun; Niu, Xiaowang; Li, Xiang; Wang, Yunjiang; Li, Longyuan; Wu, Xin; Li, Haiying; Yu, Zhengquan; Wang, Zhong; Chen, Gang] Soochow Univ, Inst Stroke Res, Suzhou 215006, Peoples R China.

[Li, Guangzhao] Hefei First Peoples Hosp, Dept Neurosurg, Hefei 230031, Peoples R China.

C3 Soochow University - China; Soochow University - China; Soochow

University - China

RP Wang, Z; Chen, G (corresponding author), Soochow Univ, Affiliated Hosp 1, Dept Neurosurg, 188 Shizi St, Suzhou 215006, Peoples R China.; Wang, Z; Chen, G (corresponding author), Soochow Univ, Affiliated Hosp 1, Brain & Nerve Res Lab, 188 Shizi St, Suzhou 215006, Peoples R China.

EM wangz8761@163.com; gangchen@suda.edu.cn

RI li, haiying/KJL-3941-2024; Li, Guang-Zhao/LDE-6198-2024; Wang,

Zongqi/KHC-3331-2024; Dai, Kun/MIN-7933-2025

CR Ahmed AU, 2006, EUKARYOT CELL, V5, P1314, DOI 10.1128/EC.00386-05

Ahmed AU, 2009, INT REV CEL MOL BIO, V273, P49, DOI 10.1016/S1937-6448(08)01802-9

Ahmed A, 2021, HUM GENET, V140, P579, DOI 10.1007/s00439-020-02226-3

Brancolini C, 2020, CANCERS, V12, DOI 10.3390/cancers12092385

Brandman O, 2016, NAT STRUCT MOL BIOL, V23, P7, DOI 10.1038/nsmb.3147

Burugu S, 2018, SEMIN CANCER BIOL, V52, P39, DOI 10.1016/j.semcancer.2017.10.001

Carreno G, 2021, NEUROPATH APPL NEURO, V47, P359, DOI 10.1111/nan.12689

Chen SH, 2020, BIOSCIENCE REP, V40, DOI 10.1042/BSR20201427

Choe YJ, 2016, NATURE, V531, P191, DOI 10.1038/nature16973

Chojak R, 2023, J NEURO-ONCOL, DOI 10.1007/s11060-023-04387-3

Chu J, 2009, P NATL ACAD SCI USA, V106, P2097, DOI 10.1073/pnas.0812819106

Criscuolo D, 2021, FRONT ONCOL, V11, DOI 10.3389/fonc.2021.797265

Defenouillère Q, 2017, CURR GENET, V63, P997, DOI 10.1007/s00294-017-0708-5

Deng P, 2017, SEMIN CANCER BIOL, V47, P43, DOI 10.1016/j.semcancer.2017.05.002

Desai N, 2020, SCIENCE, V370, P1105, DOI 10.1126/science.abc7782

Filbeck S, 2022, MOL CELL, V82, P1451, DOI 10.1016/j.molcel.2022.03.038

Fletcher-Sananikone E, 2021, CANCER RES, V81, P5935, DOI 10.1158/0008-5472.CAN-21-0752

GILLE JJP, 1992, MUTAT RES, V275, P405, DOI 10.1016/0921-8734(92)90043-O

Guang MHZ, 2019, CANCERS, V11, DOI 10.3390/cancers11010066

Guo KX, 2021, FRONT ONCOL, V11, DOI 10.3389/fonc.2021.605810

Hilal T, 2015, MOL CELL, V57, P389, DOI 10.1016/j.molcel.2015.01.029

Howard CJ, 2021, CRIT REV BIOCHEM MOL, V56, P603, DOI 10.1080/10409238.2021.1938507

Huang H, 2023, QJM-INT J MED, V116, P463, DOI 10.1093/qjmed/hcad030

Huang JL, 2017, NAT COMMUN, V8, DOI 10.1038/ncomms15144

Inigo JR, 2021, TRENDS CANCER, V7, P1050, DOI 10.1016/j.trecan.2021.08.008

Izawa T, 2017, CELL, V171, P890, DOI 10.1016/j.cell.2017.10.002

Ramírez-Expósito MJ, 2019, CURR NEUROPHARMACOL, V17, P342, DOI 10.2174/1570159X16666180302120925

Jiang T, 2021, ADV MATER, V33, DOI 10.1002/adma.202104779

Joazeiro CAP, 2019, NAT REV MOL CELL BIO, V20, P368, DOI 10.1038/s41580-019-0118-2

Jovaisaite V, 2014, J EXP BIOL, V217, P137, DOI 10.1242/jeb.090738

Keerthiga R, 2021, CELL BIOSCI, V11, DOI 10.1186/s13578-021-00696-0

Keiten-Schmitz J, 2020, MOL CELL, V79, P54, DOI 10.1016/j.molcel.2020.05.017

Kong JJ, 2022, BMC CANCER, V22, DOI 10.1186/s12885-022-09209-9

Kostova KK, 2017, SCIENCE, V357, P414, DOI 10.1126/science.aam7787

Kreft SG, 2018, TRENDS CELL BIOL, V28, P3, DOI 10.1016/j.tcb.2017.11.003

Kuroha K, 2018, MOL CELL, V72, P286, DOI 10.1016/j.molcel.2018.08.022

Lamech L, 2017, DEV CELL, V43, P259, DOI 10.1016/j.devcel.2017.10.022

Li HZ, 2017, TURK NEUROSURG, V27, P894, DOI 10.5137/1019-5149.JTN.17746-16.1

Li ZT, 2021, MEDICINE, V100, DOI 10.1097/MD.0000000000027374

Liu Y, 2019, ONCOGENE, V38, P1815, DOI 10.1038/s41388-018-0525-4

Liu YJ, 2020, NANO LETT, V20, P1637, DOI 10.1021/acs.nanolett.9b04683

Liu ZZ, 2022, HUM GENOMICS, V16, DOI 10.1186/s40246-022-00377-0

Lu BW, 2020, TRENDS CELL BIOL, V30, P317, DOI 10.1016/j.tcb.2020.01.008

Martin PB, 2020, NAT COMMUN, V11, DOI 10.1038/s41467-020-18327-6

Melber A, 2018, CELL RES, V28, P281, DOI 10.1038/cr.2018.16

Mishra R, 2021, MOL NEUROBIOL, V58, P6593, DOI 10.1007/s12035-021-02564-x

Mukhopadhyay A, 2004, BIOCHEM J, V382, P385, DOI 10.1042/BJ20040065

Osuna BA, 2017, ELIFE, V6, DOI 10.7554/eLife.27949

Ramalingam M, 2014, FREE RADICAL RES, V48, P347, DOI 10.3109/10715762.2013.869588

Rendón OZ, 2018, NAT COMMUN, V9, DOI 10.1038/s41467-018-04564-3

Sajadi M, 2022, CANCER CELL INT, V22, DOI 10.1186/s12935-022-02751-3

Salam R, 2023, NAT COMMUN, V14, DOI 10.1038/s41467-023-36124-9

Schulz JA, 2022, BMC CANCER, V22, DOI 10.1186/s12885-022-09910-9

Shin CS, 2021, NAT COMMUN, V12, DOI 10.1038/s41467-020-20597-z

Sitron CS, 2020, PLOS ONE, V15, DOI 10.1371/journal.pone.0227841

Sitron CS, 2019, NAT STRUCT MOL BIOL, V26, P450, DOI 10.1038/s41594-019-0230-1

Stein KC, 2022, NATURE, V601, P637, DOI 10.1038/s41586-021-04295-4

Sutandy FXR, 2023, NATURE, V618, P849, DOI 10.1038/s41586-023-06142-0

Thrun A, 2021, MOL CELL, V81, P2112, DOI 10.1016/j.molcel.2021.03.004

Tian X, 2022, FRONT PHARMACOL, V13, DOI 10.3389/fphar.2022.939542

Udagawa T, 2021, STAR PROTOC, V2, DOI 10.1016/j.xpro.2021.100615

Udagawa T, 2021, CELL REP, V34, DOI 10.1016/j.celrep.2020.108599

van Haaften-Visser DY, 2017, J BIOL CHEM, V292, P7904, DOI 10.1074/jbc.M116.772038

Vendramin R, 2018, NAT STRUCT MOL BIOL, V25, P1035, DOI 10.1038/s41594-018-0143-4

Verma R, 2018, NATURE, V557, P446, DOI 10.1038/s41586-018-0022-5

VERNER K, 1993, TRENDS BIOCHEM SCI, V18, P366, DOI 10.1016/0968-0004(93)90090-A

Wang G, 2022, CELL BIOSCI, V12, DOI 10.1186/s13578-022-00747-0

Wang W, 2016, SCI TRANSL MED, V8, DOI 10.1126/scitranslmed.aac7410

Wilkening A, 2018, J BIOL CHEM, V293, P11537, DOI 10.1074/jbc.RA118.002122

Wu ZH, 2019, MOL CELL, V75, P835, DOI 10.1016/j.molcel.2019.06.031

Xing QW, 2021, BMC CANCER, V21, DOI 10.1186/s12885-021-08111-0

Xu FS, 2020, BMC CANCER, V20, DOI 10.1186/s12885-020-07702-7

Zhang HY, 2021, SCI ROBOT, V6, DOI 10.1126/scirobotics.aaz9519

Zhou X, 2019, FUTURE ONCOL, V15, P2093, DOI 10.2217/fon-2018-0920

Zhu L, 2021, FREE RADICAL BIO MED, V163, P125, DOI 10.1016/j.freeradbiomed.2020.12.013

NR 75

TC 4

Z9 4

U1 1

U2 2

PU ELSEVIER IRELAND LTD

PI CLARE

PA ELSEVIER HOUSE, BROOKVALE PLAZA, EAST PARK SHANNON, CO, CLARE, 00000,

IRELAND

SN 0304-3835

EI 1872-7980

J9 CANCER LETT

JI Cancer Lett.

PD JUN 1

PY 2024

VL 591

AR 216895

DI 10.1016/j.canlet.2024.216895

EA APR 2024

PG 17

WC Oncology

WE Science Citation Index Expanded (SCI-EXPANDED)

SC Oncology

GA SU6H5

UT WOS:001236994800001

PM 38670305

DA 2025-04-09

ER

PT J

AU Bountali, A

Tonge, DP

Mourtada-Maarabouni, M

AF Bountali, Aikaterini

Tonge, Daniel P.

Mourtada-Maarabouni, Mirna

TI RNA sequencing reveals a key role for the long non-coding RNA MIAT in

regulating neuroblastoma and glioblastoma cell fate

SO INTERNATIONAL JOURNAL OF BIOLOGICAL MACROMOLECULES

LA English

DT Article

DE MIAT; RNA sequencing; Oxidative stress; Apoptosis; Neuroblastoma;

Glioblastoma

ID INVASION; GLIOMA; PERSPECTIVES; MECHANISMS; EXPRESSION; MIGRATION;

GENETICS; HYPOXIA; SP1

AB Myocardial Infarction Associated Transcript (MIAT) is a subnuclear lncRNA that interferes with alternative splicing and is associated with increased risk of various heart conditions and nervous system tumours. The current study aims to elucidate the role of MIAT in cell survival, apoptosis and migration in neuroblastoma and glioblastoma multiforme. To this end, MIAT was silenced by MIAT-specific siRNAs in neuroblastoma and glioblastoma cell lines, and RNA sequencing together with a series of functional assays were performed. The RNA sequencing has revealed that the expression of an outstanding number of genes is altered, including genes involved in cancer-related processes, such as cell growth and survival, apoptosis, reactive oxygen species (ROS) production and migration. Furthermore, the functional studies have confirmed the RNA sequencing leads, with our key findings suggesting that MEAT knockdown eliminates long-term survival and migration and increases basal apoptosis in neuroblastoma and glioblastoma cell lines. Taken together with the recent demonstration of the involvement of MIAT in glioblastoma, our observations suggest that MIAT could possess tumour-promoting properties, thereby acting as an oncogene, and has the potential to be used as a reliable biomarker for neuroblastoma and glioblastoma and be employed for prognostic, predictive and, potentially, therapeutic purposes for these cancers. (C) 2019 Elsevier B.V. All rights reserved.

C1 [Bountali, Aikaterini; Tonge, Daniel P.; Mourtada-Maarabouni, Mirna] Keele Univ, Fac Nat Sci, Sch Life Sci, Newcastle Under Lyme ST5 5BG, England.

C3 Keele University

RP Mourtada-Maarabouni, M (corresponding author), Keele Univ, Fac Nat Sci, Sch Life Sci, Newcastle Under Lyme ST5 5BG, England.

EM m.m.maarabouni@keele.ac.uk

OI /0000-0001-8324-1325; Tonge, Daniel/0000-0002-3499-2752

FU Faculty of Natural Sciences, Keele University

FX Aikaterini Bountali is supported by a studentship awarded by the Faculty

of Natural Sciences, Keele University.

CR Alipoor FJ, 2018, J CELL BIOCHEM, V119, P6470, DOI 10.1002/jcb.26678

Almnaseer ZA, 2018, BIOSCIENCE REP, V38, DOI 10.1042/BSR20180704

Amaral PP, 2008, MAMM GENOME, V19, P454, DOI 10.1007/s00335-008-9136-7

Blackshaw S, 2004, PLOS BIOL, V2, P1411, DOI 10.1371/journal.pbio.0020247

Boon RA, 2016, J AM COLL CARDIOL, V67, P1214, DOI 10.1016/j.jacc.2015.12.051

Cabili MN, 2011, GENE DEV, V25, P1915, DOI 10.1101/gad.17446611

Cao JN, 2014, BIOL PROCED ONLINE, V16, DOI 10.1186/1480-9222-16-11

Cheng L, 2016, SCI CHINA LIFE SCI, V59, P236, DOI 10.1007/s11427-016-5012-y

Choudhry H, 2014, EMBO REP, V15, P70, DOI 10.1002/embr.201337642

Clark BS, 2014, FRONT GENET, V5, DOI 10.3389/fgene.2014.00164

Crocoli A, 2015, JNCI-J NATL CANCER I, V15, P79

Davies KJA, 1999, IUBMB LIFE, V48, P41, DOI 10.1080/152165499307404

Dhamija S, 2016, INT J CANCER, V139, P269, DOI 10.1002/ijc.30039

Domingo-Fernandez R, 2013, PEDIATR SURG INT, V29, P101, DOI 10.1007/s00383-012-3239-7

Dong J, 2016, TUMOR BIOL, V37, P1379, DOI 10.1007/s13277-015-4457-0

Dong QS, 2014, PLOS ONE, V9, DOI 10.1371/journal.pone.0098651

Fang YW, 2016, GENOM PROTEOM BIOINF, V14, P42, DOI 10.1016/j.gpb.2015.09.006

Fenoglio C, 2013, INT J MOL SCI, V14, P20427, DOI 10.3390/ijms141020427

Galardi S, 2016, EMBO REP, V17, P1872, DOI 10.15252/embr.201541489

Goodenberger ML, 2012, CANCER GENET-NY, V205, P613, DOI 10.1016/j.cancergen.2012.10.009

Guan HY, 2012, INT J CANCER, V130, P593, DOI 10.1002/ijc.26049

Guo H, 2015, GENE, V554, P114, DOI 10.1016/j.gene.2014.10.038

Hara J, 2012, INT J CLIN ONCOL, V17, P196, DOI 10.1007/s10147-012-0417-5

Hedrick E, 2016, ONCOTARGET, V7, P22245, DOI 10.18632/oncotarget.7925

Hoffman B, 2008, ONCOGENE, V27, P6462, DOI 10.1038/onc.2008.312

Ishii N, 2006, J HUM GENET, V51, P1087, DOI 10.1007/s10038-006-0070-9

Li XY, 2016, ONCOTARGET, V7, P22031, DOI 10.18632/oncotarget.8247

Liao JQ, 2016, GENE, V578, P158, DOI 10.1016/j.gene.2015.12.032

Liu ZX, 2018, CANCER CELL INT, V18, DOI 10.1186/s12935-017-0477-8

Luksch R, 2016, CRIT REV ONCOL HEMAT, V107, P163, DOI 10.1016/j.critrevonc.2016.10.001

McMahon SB, 2014, CSH PERSPECT MED, V4, DOI 10.1101/cshperspect.a014407

Melissari MT, 2016, PFLUG ARCH EUR J PHY, V468, P945, DOI 10.1007/s00424-016-1804-y

Mohanty V, 2015, BRIEF FUNCT GENOMICS, V14, P115, DOI 10.1093/bfgp/elu034

O'Hagan HM, 2011, CANCER CELL, V20, P606, DOI 10.1016/j.ccr.2011.09.012

Pandey GK, 2015, ONCOTARGET, V6, P18265, DOI 10.18632/oncotarget.4251

Pelicano H, 2004, DRUG RESIST UPDATE, V7, P97, DOI 10.1016/j.drup.2004.01.004

Qu Y, 2018, CELL PHYSIOL BIOCHEM, V48, P1075, DOI 10.1159/000491974

Quan MY, 2015, INT J MOL SCI, V16, P5467, DOI 10.3390/ijms16035467

Qureshi IA, 2010, BRAIN RES, V1338, P20, DOI 10.1016/j.brainres.2010.03.110

Ramos AD, 2016, NEUROSCI LETT, V625, P70, DOI 10.1016/j.neulet.2015.12.025

Rao AKDM, 2017, MOL BIOL REP, V44, P203, DOI 10.1007/s11033-017-4103-6

Safe S, 2018, CANCER PREV RES, V11, P371, DOI 10.1158/1940-6207.CAPR-17-0407

Salazar BM, 2017, INT J MOL SCI, V18, DOI 10.3390/ijms18010037

Sattari A, 2016, ONCOTARGET, V7, P54174, DOI 10.18632/oncotarget.11099

Schmitz SU, 2016, CELL MOL LIFE SCI, V73, P2491, DOI 10.1007/s00018-016-2174-5

Shen Y, 2016, J CELL MOL MED, V20, P537, DOI 10.1111/jcmm.12755

Sone M, 2007, J CELL SCI, V120, P2498, DOI 10.1242/jcs.009357

Tsuiji H, 2011, GENES CELLS, V16, P479, DOI 10.1111/j.1365-2443.2011.01502.x

Wapinski O, 2011, TRENDS CELL BIOL, V21, P354, DOI 10.1016/j.tcb.2011.04.001

Watters KM, 2013, BMC CANCER, V13, DOI 10.1186/1471-2407-13-184

Wu RF, 2016, MOL GENET GENOMICS, V291, P1013, DOI 10.1007/s00438-016-1179-y

Zhang HY, 2017, GENE, V633, P61, DOI 10.1016/j.gene.2017.08.009

Zhang XQ, 2014, NEUROCHEM INT, V77, P78, DOI 10.1016/j.neuint.2014.05.008

Zhang XQ, 2013, NEUROBIOL DIS, V58, P123, DOI 10.1016/j.nbd.2013.05.011

Zhang ZL, 2018, BIOCHEM BIOPH RES CO, V503, P1530, DOI 10.1016/j.bbrc.2018.07.075

2002, MOL CELL, V9, P1031

2015, CANCERS, V7, P1406

NR 57

TC 33

Z9 35

U1 0

U2 10

PU ELSEVIER SCIENCE BV

PI AMSTERDAM

PA PO BOX 211, 1000 AE AMSTERDAM, NETHERLANDS

SN 0141-8130

EI 1879-0003

J9 INT J BIOL MACROMOL

JI Int. J. Biol. Macromol.

PD JUN 1

PY 2019

VL 130

BP 878

EP 891

DI 10.1016/j.ijbiomac.2019.03.005

PG 14

WC Biochemistry & Molecular Biology; Chemistry, Applied; Polymer Science

WE Science Citation Index Expanded (SCI-EXPANDED)

SC Biochemistry & Molecular Biology; Chemistry; Polymer Science

GA HV8SE

UT WOS:000466253000093

PM 30836187

DA 2025-04-09

ER

PT J

AU Winter, SF

Loebel, F

Dietrich, J

AF Winter, Sebastian F.

Loebel, Franziska

Dietrich, Jorg

TI Role of ketogenic metabolic therapy in malignant glioma: A systematic

review

SO CRITICAL REVIEWS IN ONCOLOGY HEMATOLOGY

LA English

DT Review

DE Malignant glioma; Glioblastoma multiforme; Cancer metabolism; Ketogenic

diet; Low glycemic diet; Metabolic therapy; Adjunctive cancer therapy

ID GROWTH-FACTOR-I; TARGETING ENERGY-METABOLISM; IGF-BINDING PROTEIN-3;

OXIDATIVE STRESS; KETONE-BODIES; PROSTATE-CANCER; BREAST-CANCER; CALORIE

RESTRICTION; PROLONGS SURVIVAL; ADJUVANT THERAPY

AB Background: Coined as the "Warburg effect" and a recognized hallmark of cancer, energy metabolism is aberrantly geared towards aerobic glycolysis in most human cancers, including malignant glioma. Ketogenic metabolic therapy (KMT), i.e. nutritional intervention with ketogenic or low-glycemic diets, has been proposed as an anti-neoplastic strategy in glioma patients.

Materials and methods: We here review the rationale and existing data investigating KMT in management of patients with malignant glioma and discuss the promise and potential challenges of this novel strategy. Results from published clinical studies and ongoing clinical trials on the topic are systematically reviewed, including 6 published original articles and 10 ongoing clinical trials. Search criteria for this review entailed the databases MEDLINE, EMBASE, Cochrane CENTRAL, and Google Scholar, as well as ICTRP (WHO) and ClinicalTrials.gov (NIH) registries:

Results: A substantial amount of preclinical literature demonstrates KMT efficacy and safety in model systems of malignant glioma. Clinical literature indicates KMT safety and feasibility; 2 clinical studies suggest KMT-associated anti-neoplastic efficacy and clinical benefit. Ongoing clinical trials address KMT safety and metabolic impact, patient compliance, and patient clinical/survival benefit.

Conclusions: While clinical evidence is still limited in this evolving field, increasing numbers of ongoing clinical trials suggest that KMT is emerging as a potential therapeutic option and might be combinable with existing anti-neoplastic treatments for malignant glioma. Emerging clinical data will help answer questions concerning safety and efficacy of KMT, and are aiming to identify the most promising KMT regimen, compatibility with other anti-cancer treatments, ethical aspects, and impact on quality of life of cancer patients. (C) 2017 Elsevier B.V. All rights reserved.

C1 [Winter, Sebastian F.; Dietrich, Jorg] Harvard Med Sch, Massachusetts Gen Hosp, Dept Neurol, 55 Fruit St, Boston, MA 02114 USA.

[Winter, Sebastian F.; Loebel, Franziska] Charite, Fac Med, Charitepl 1, D-10117 Berlin, Germany.

[Loebel, Franziska] Charite, Dept Neurosurg, Augustenburger Pl 1, D-13353 Berlin, Germany.

[Dietrich, Jorg] Harvard Med Sch, Massachusetts Gen Hosp, MGH Canc Ctr, 55 Fruit St,Yawkey 9E, Boston, MA 02114 USA.

[Dietrich, Jorg] Harvard Med Sch, Massachusetts Gen Hosp, Ctr Regenerat Med, 55 Fruit St,Yawkey 9E, Boston, MA 02114 USA.

C3 Harvard University; Harvard Medical School; Harvard University Medical

Affiliates; Massachusetts General Hospital; Berlin Institute of Health;

Free University of Berlin; Humboldt University of Berlin; Charite

Universitatsmedizin Berlin; Berlin Institute of Health; Free University

of Berlin; Humboldt University of Berlin; Charite Universitatsmedizin

Berlin; Harvard University; Harvard Medical School; Harvard University

Medical Affiliates; Massachusetts General Hospital; Harvard University;

Harvard University Medical Affiliates; Massachusetts General Hospital;

Harvard Medical School

RP Dietrich, J (corresponding author), Massachusetts Gen Hosp, Canc Ctr, 55 Fruit St,Yawkey 9E, Boston, MA 02114 USA.

EM sebastian-friedrich.winter@charite.de; franziskaJoebel@charite.de;

Dietrich.Jorg@mgh.harvard.edu

RI Winter, Sebastian/ABF-8919-2020

OI Winter, Sebastian F./0000-0001-8836-0235

FU German National Academic Foundation (Studienstiftung des Deutschen

Volkes)

FX S.F.W. received a travel grant from the German National Academic

Foundation (Studienstiftung des Deutschen Volkes).

CR Abdelwahab MG, 2012, PLOS ONE, V7, DOI 10.1371/journal.pone.0036197

Alifieris C, 2015, PHARMACOL THERAPEUT, V152, P63, DOI 10.1016/j.pharmthera.2015.05.005

Allen BG, 2013, CLIN CANCER RES, V19, P3905, DOI 10.1158/1078-0432.CCR-12-0287

[Anonymous], ACTA ONCOL

[Anonymous], MOL NEUROBIOL

[Anonymous], NEUROLOGY S

[Anonymous], IR ISL REP ID IRCT20

[Anonymous], ID ACTRN126140010566

Anton K, 2012, HEMATOL ONCOL CLIN N, V26, P825, DOI 10.1016/j.hoc.2012.04.006

Arismendi-Morillo GJ, 2008, J ELECTRON MICROSC, V57, P33, DOI 10.1093/jmicro/dfm038

Aum DJ, 2014, NEUROSURG FOCUS, V37, DOI 10.3171/2014.9.FOCUS14521

Bastien JIL, 2015, CANCER-AM CANCER SOC, V121, P502, DOI 10.1002/cncr.28968

Bowers LW, 2015, FRONT ENDOCRINOL, V6, DOI 10.3389/fendo.2015.00077

Brandsma D, 2008, LANCET ONCOL, V9, P453, DOI 10.1016/S1470-2045(08)70125-6

Brennan CW, 2013, CELL, V155, P462, DOI 10.1016/j.cell.2013.09.034

Cairns RA, 2011, NAT REV CANCER, V11, P85, DOI 10.1038/nrc2981

Campbell TC, 2007, AM J CLIN NUTR, V85, P1667, DOI 10.1093/ajcn/85.6.1667

Carlsson SK, 2014, EMBO MOL MED, V6, P1359, DOI 10.15252/emmm.201302627

Cervenka Mackenzie C, 2013, Continuum (Minneap Minn), V19, P756, DOI 10.1212/01.CON.0000431396.23852.56

Champ CE, 2014, J NEURO-ONCOL, V117, P125, DOI 10.1007/s11060-014-1362-0

Chan JM, 1998, SCIENCE, V279, P563, DOI 10.1126/science.279.5350.563

Chang HT, 2013, NUTR METAB, V10, DOI 10.1186/1743-7075-10-47

Chang SM, 2005, JAMA-J AM MED ASSOC, V293, P557, DOI 10.1001/jama.293.5.557

Chinot OL, 2014, NEW ENGL J MED, V370, P709, DOI 10.1056/NEJMoa1308345

Crespo I, 2015, AM J PATHOL, V185, P1820, DOI 10.1016/j.ajpath.2015.02.023

Crocetti E, 2012, EUR J CANCER, V48, P1532, DOI 10.1016/j.ejca.2011.12.013

Deighton RF, 2014, J NEURO-ONCOL, V118, P247, DOI 10.1007/s11060-014-1430-5

Derr RL, 2009, J CLIN ONCOL, V27, P1082, DOI 10.1200/JCO.2008.19.1098

Dietrich J, 2010, ADV EXP MED BIOL, V678, P77

Dietrich J, 2008, ONCOLOGIST, V13, P1285, DOI 10.1634/theoncologist.2008-0130

Dietrich Jorg, 2011, Expert Rev Clin Pharmacol, V4, P233, DOI 10.1586/ecp.11.1

Draznin B, 2012, HORM METAB RES, V44, P650, DOI 10.1055/s-0032-1312656

Duggan C, 2011, J CLIN ONCOL, V29, P32, DOI 10.1200/JCO.2009.26.4473

Eloqayli H, 2011, NEUROCHEM RES, V36, P1566, DOI 10.1007/s11064-011-0485-3

Fack F, 2015, ACTA NEUROPATHOL, V129, P115, DOI 10.1007/s00401-014-1352-5

Freeman John M, 2010, Adv Pediatr, V57, P315, DOI 10.1016/j.yapd.2010.08.003

Frosina G, 2015, CRIT REV ONCOL HEMAT, V96, P257, DOI 10.1016/j.critrevonc.2015.05.013

Fulda S, 2010, NAT REV DRUG DISCOV, V9, P447, DOI 10.1038/nrd3137

Gilbert MR, 2014, NEW ENGL J MED, V370, P699, DOI 10.1056/NEJMoa1308573

Giovannucci E, 2000, CANCER EPIDEM BIOMAR, V9, P345

Goodwin PJ, 2002, J CLIN ONCOL, V20, P42, DOI 10.1200/JCO.20.1.42

Guntuku L, 2016, CURR NEUROPHARMACOL, V14, P567, DOI 10.2174/1570159X14666160121115641

Han Lijiang, 2014, Zhonghua Yi Xue Za Zhi, V94, P2129

Hanahan D, 2011, CELL, V144, P646, DOI 10.1016/j.cell.2011.02.013

Harman SM, 2000, J CLIN ENDOCR METAB, V85, P4258, DOI 10.1210/jc.85.11.4258

Harris D, 2013, CURR ONCOL, V20, pE532, DOI 10.3747/co.20.1499

Hart MG, 2013, COCHRANE DB SYST REV, DOI 10.1002/14651858.CD007415.pub2

Hussain TA, 2012, NUTRITION, V28, P1016, DOI 10.1016/j.nut.2012.01.016

Jelluma N, 2006, MOL CANCER RES, V4, P319, DOI 10.1158/1541-7786.MCR-05-0061

Jiang YS, 2013, J NEURO-ONCOL, V114, P25, DOI 10.1007/s11060-013-1154-y

Kaiser J, 2014, CORTEX, V54, P33, DOI 10.1016/j.cortex.2014.01.010

Kang HC, 2004, EPILEPSIA, V45, P1116, DOI 10.1111/j.0013-9580.2004.10004.x

Khasraw M, 2014, COCHRANE DB SYST REV, DOI 10.1002/14651858.CD008218.pub3

Kyritsis AP, 2011, NUTR CANCER, V63, P174, DOI 10.1080/01635581.2011.523807

Lee C, 2012, SCI TRANSL MED, V4, DOI 10.1126/scitranslmed.3003293

Lee WH, 2010, INT J RADIAT BIOL, V86, P132, DOI 10.3109/09553000903419346

Lukins MB, 2005, ANESTH ANALG, V100, P1129, DOI 10.1213/01.ANE.0000146943.45445.55

Lussier DM, 2016, BMC CANCER, V16, DOI 10.1186/s12885-016-2337-7

Ma J, 1999, J NATL CANCER I, V91, P620, DOI 10.1093/jnci/91.7.620

Ma J, 2008, LANCET ONCOL, V9, P1039, DOI 10.1016/S1470-2045(08)70235-3

Maalouf M, 2007, NEUROSCIENCE, V145, P256, DOI 10.1016/j.neuroscience.2006.11.065

Maalouf M, 2009, BRAIN RES REV, V59, P293, DOI 10.1016/j.brainresrev.2008.09.002

Maroon J, 2013, J CHILD NEUROL, V28, P1002, DOI 10.1177/0883073813488670

Maroon Joseph C, 2015, Surg Neurol Int, V6, P61, DOI 10.4103/2152-7806.155259

Marsh J, 2008, NUTR METAB, V5, DOI 10.1186/1743-7075-5-33

Martuscello RT, 2014, NEURO-ONCOLOGY, V16, DOI 10.1093/neuonc/nou278.12

Matsubara J, 2008, CLIN CANCER RES, V14, P3022, DOI 10.1158/1078-0432.CCR-07-1898

Maurer GD, 2011, BMC CANCER, V11, DOI 10.1186/1471-2407-11-315

Mayer A, 2014, STRAHLENTHER ONKOL, V190, P933, DOI 10.1007/s00066-014-0696-z

McGirt MJ, 2008, NEUROSURGERY, V63, P286, DOI 10.1227/01.NEU.0000315282.61035.48

Meidenbauer JJ, 2015, NUTR METAB, V12, DOI 10.1186/s12986-015-0009-2

Milder J, 2012, EPILEPSY RES, V100, P295, DOI 10.1016/j.eplepsyres.2011.09.021

Tieu MT, 2015, J NEURO-ONCOL, V124, P119, DOI 10.1007/s11060-015-1815-0

Moher D, 2009, ANN INTERN MED, V151, P264, DOI [10.7326/0003-4819-151-4-200908180-00135, 10.1016/j.ijsu.2010.07.299, 10.1186/2046-4053-4-1, 10.1371/journal.pmed.1000097, 10.1136/bmj.b2700, 10.1136/bmj.b2535, 10.1016/j.ijsu.2010.02.007, 10.1136/bmj.i4086]

Monje M, 2012, BEHAV BRAIN RES, V227, P376, DOI 10.1016/j.bbr.2011.05.012

Monje ML, 2007, ANN NEUROL, V62, P515, DOI 10.1002/ana.21214

Moreno B, 2014, ENDOCRINE, V47, P793, DOI 10.1007/s12020-014-0192-3

Morscher RJ, 2015, PLOS ONE, V10, DOI 10.1371/journal.pone.0129802

Mukherjee P, 2008, MOL CANCER, V7, DOI 10.1186/1476-4598-7-37

Nathan J, 2011, EPILEPSIA, V52, P162

Neal EG, 2008, LANCET NEUROL, V7, P500, DOI 10.1016/S1474-4422(08)70092-9

NEBELING LC, 1995, J AM COLL NUTR, V14, P202

Newman JC, 2014, TRENDS ENDOCRIN MET, V25, P42, DOI 10.1016/j.tem.2013.09.002

Ostrom Q.T, 2015, NEURO-ONCOLOGY, V17, P1

Oudard S, 1996, BRIT J CANCER, V74, P839, DOI 10.1038/bjc.1996.446

Oudard S, 1997, ANTICANCER RES, V17, P1903

Panosyan EH, 2016, J NEURO-ONCOL, V128, P57, DOI 10.1007/s11060-016-2073-5

Parsons DW, 2008, SCIENCE, V321, P1807, DOI 10.1126/science.1164382

Pasternak JJ, 2004, J NEUROSURG ANESTH, V16, P122, DOI 10.1097/00008506-200404000-00003

Pazmandi J, 2015, CANCER RES, V75, DOI 10.1158/1538-7445.AM2015-240

Pitter KL, 2016, BRAIN, V139, P1458, DOI 10.1093/brain/aww046

Poff AM, 2015, PLOS ONE, V10, DOI 10.1371/journal.pone.0127407

Poff AM, 2014, INT J CANCER, V135, P1711, DOI 10.1002/ijc.28809

Poff AM, 2013, PLOS ONE, V8, DOI 10.1371/journal.pone.0065522

Prados MD, 2015, NEURO-ONCOLOGY, V17, P1051, DOI 10.1093/neuonc/nov031

Prust MJ, 2015, NEUROLOGY, V85, P683, DOI 10.1212/WNL.0000000000001861

Raffaghello L, 2008, P NATL ACAD SCI USA, V105, P8215, DOI 10.1073/pnas.0708100105

Renda L, 2015, CANCER RES, V75, DOI 10.1158/1538-7445.AM2015-CT213

Renehan AG, 2004, LANCET, V363, P1346, DOI 10.1016/S0140-6736(04)16044-3

Rieger J, 2014, INT J ONCOL, V44, P1843, DOI 10.3892/ijo.2014.2382

Rossi AP, 2015, CANCER RES, V75, DOI 10.1158/1538-7445.AM2015-3346

Roth P, 2010, CURR OPIN NEUROL, V23, P597, DOI 10.1097/WCO.0b013e32833e5a5d

Ru Peng, 2013, Cancers (Basel), V5, P1469, DOI 10.3390/cancers5041469

Safdie F, 2012, PLOS ONE, V7, DOI 10.1371/journal.pone.0044603

Sanzey M, 2015, PLOS ONE, V10, DOI 10.1371/journal.pone.0123544

Scheck AC, 2012, EPILEPSY RES, V100, P327, DOI 10.1016/j.eplepsyres.2011.09.022

Schiff D, 2015, NEURO-ONCOLOGY, V17, P488, DOI 10.1093/neuonc/nou304

Schwartz K, 2015, CANCER METAB, V3, DOI 10.1186/s40170-015-0129-1

Seyfried BTN, 2009, J CANCER RES THER, V5, P7, DOI 10.4103/0973-1482.55134

Seyfried TN, 2008, EPILEPSIA, V49, P114, DOI 10.1111/j.1528-1167.2008.01853.x

Seyfried TN, 2015, CANCER LETT, V356, P289, DOI 10.1016/j.canlet.2014.07.015

Seyfried TN, 2014, CARCINOGENESIS, V35, P515, DOI 10.1093/carcin/bgt480

Seyfried TN, 2012, EPILEPSY RES, V100, P310, DOI 10.1016/j.eplepsyres.2011.06.017

Seyfried TN, 2011, BBA-BIOENERGETICS, V1807, P577, DOI 10.1016/j.bbabio.2010.08.009

Seyfried TN, 2010, LANCET ONCOL, V11, P811, DOI 10.1016/S1470-2045(10)70166-2

Seyfried TN, 2003, BRIT J CANCER, V89, P1375, DOI 10.1038/sj.bjc.6601269

Shelton LM, 2010, ASN NEURO, V2, DOI 10.1042/AN20100002

Shelton LM, 2010, INT J CANCER, V127, P2478, DOI 10.1002/ijc.25431

Shi RH, 2004, INT J CANCER, V111, P418, DOI 10.1002/ijc.20233

Shimazu T, 2013, SCIENCE, V339, P211, DOI 10.1126/science.1227166

Sichani MM, 2010, SAUDI J KIDNEY DIS T, V21, P69

Simone BA, 2013, FUTURE ONCOL, V9, P959, DOI [10.2217/fon.13.31, 10.2217/FON.13.31]

Singh DH, 2005, STRAHLENTHER ONKOL, V181, P507, DOI 10.1007/s00066-005-1320-z

SOKOLOFF L, 1973, ANNU REV MED, V24, P271, DOI 10.1146/annurev.me.24.020173.001415

Spitz DR, 2000, ANN NY ACAD SCI, V899, P349, DOI 10.1111/j.1749-6632.2000.tb06199.x

Stafford P, 2010, NUTR METAB, V7, DOI 10.1186/1743-7075-7-74

Strowd RE, 2015, NEURO-ONCOL PRACT, V2, P127, DOI 10.1093/nop/npv010

Strowd RE, 2015, CURR TREAT OPTION ON, V16, DOI 10.1007/s11864-015-0356-2

Stupp R, 2005, NEW ENGL J MED, V352, P987, DOI 10.1056/NEJMoa043330

Suhrcke M, 2008, Caring for people with chronic conditions: A health system perspective, P43

Sullivan PG, 2004, ANN NEUROL, V55, P576, DOI 10.1002/ana.20062

Tanaka K, 2015, J CLIN INVEST, V125, P1591, DOI 10.1172/JCI78239

Tanaka S, 2013, NAT REV CLIN ONCOL, V10, P14, DOI 10.1038/nrclinonc.2012.204

Varshneya K, 2015, CUREUS J MED SCIENCE, V7, DOI 10.7759/cureus.251

Venkataramanaa Neelam K, 2013, Asian J Neurosurg, V8, P9, DOI 10.4103/1793-5482.110274

Venur VA, 2015, CANCER TREAT RES, V163, P103, DOI 10.1007/978-3-319-12048-5_7

Wang YG, 2015, ONCOL LETT, V10, P189, DOI 10.3892/ol.2015.3244

Warburg O, 1924, BIOCHEM Z, V152, P309

Warburg O. H, 1930, METABOLISM TUMOURS

Wen PY, 2008, NEW ENGL J MED, V359, P492, DOI 10.1056/NEJMra0708126

Wolpin BM, 2009, J CLIN ONCOL, V27, P176, DOI 10.1200/JCO.2008.17.9945

Wong ET, 2015, BRIT J CANCER, V113, P232, DOI 10.1038/bjc.2015.238

Woolf EC, 2015, PLOS ONE, V10, DOI 10.1371/journal.pone.0130357

Woolf EC, 2015, J LIPID RES, V56, P5, DOI 10.1194/jlr.R046797

Woolf Eric C, 2012, CNS Oncol, V1, P7, DOI 10.2217/cns.12.9

Yang CD, 2009, CANCER RES, V69, P7986, DOI 10.1158/0008-5472.CAN-09-2266

Ye ZC, 1999, CANCER RES, V59, P4383

Yeung YT, 2013, BRIT J PHARMACOL, V168, P591, DOI 10.1111/bph.12008

Yip S, 2009, CLIN CANCER RES, V15, P4622, DOI 10.1158/1078-0432.CCR-08-3012

Zakikhani M, 2006, CANCER RES, V66, P10269, DOI 10.1158/0008-5472.CAN-06-1500

Zhou WH, 2007, NUTR METAB, V4, DOI 10.1186/1743-7075-4-5

Zu K, 2013, CANCER EPIDEM BIOMAR, V22, P1984, DOI 10.1158/1055-9965.EPI-13-0349

Zuccoli G, 2010, NUTR METAB, V7, DOI 10.1186/1743-7075-7-33

NR 153

TC 65

Z9 68

U1 2

U2 27

PU ELSEVIER SCIENCE INC

PI NEW YORK

PA STE 800, 230 PARK AVE, NEW YORK, NY 10169 USA

SN 1040-8428

EI 1879-0461

J9 CRIT REV ONCOL HEMAT

JI Crit. Rev. Oncol./Hematol.

PD APR

PY 2017

VL 112

BP 41

EP 58

DI 10.1016/j.critrevonc.2017.02.016

PG 18

WC Oncology; Hematology

WE Science Citation Index Expanded (SCI-EXPANDED)

SC Oncology; Hematology

GA ER4GO

UT WOS:000398757300005

PM 28325264

DA 2025-04-09

ER

PT J

AU Wang, YJ

Cheng, D

He, JJ

Liu, SJ

Wang, XL

Wang, M

AF Wang, Yingjie

Cheng, Dai

He, Jingjing

Liu, Sijia

Wang, Xinlu

Wang, Meng

TI Magnolol protects C6 glioma cells against neurotoxicity of FB1 via

modulating PI3K/Akt and mitochondria-associated apoptosis signaling

pathways

SO ENVIRONMENTAL POLLUTION

LA English

DT Article

DE Fumonisin B1; Magnolol; Neurotoxicity; PI3K/Akt pathway; Apoptosis

ID CYCLE ARREST; IN-VITRO; ACTIVATION; CURCUMIN; INSULIN; DAMAGE

AB Fumonisin B1 (FB1) is a contaminant commonly occurring in crops and food. Mycotoxin contamination, including FB1, has been progressively shown to be an important risk factor in mediating neurotoxicity and neurodegenerative diseases. Studies have found that magnolol (MAG) exhibits favorable pharmacological effects in the central nervous system. However, the protective effects of MAG against FB1-induced neurotoxicity and the molecular pathways involved have not been fully elucidated. Our study aimed to investigate the neuroprotective effects of MAG on FB1-exposed C6 cells and to identify the underlying mechanisms. A model of FB1-induced cytotoxicity in C6 glial cells was established. C6 cells were treated with MAG (40, 80 and 160 mu M) in the presence/absence of FB1 (15 mu M) and then assessed for cell viability, cellular and mitochondrial morphology and oxidative stress. The mechanism of action of MAG was revealed using a variety of means including RNA-seq, qRT-PCR, Western blot, immunofluorescence, scanning electron microscopy analysis and agonist validation experiments. Our results indicated that MAG significantly alleviated AFB1-induced C6 astroglial cytotoxicity, as evidenced by elevated cell viability and restoration of overall cellular and mitochondrial morphology. Meanwhile, MAG also alleviated oxidative stress in FB1-exposed C6 cells, with 80 mu M MAG showing the best effect. Transcriptome analysis showed that PI3K/Akt and apoptosis involved in it might be the key pathway for MAG to treat FB1 neurotoxicity. MAG suppressed FB1-induced mitochondria-dependent apoptosis in C6 cells, primarily manifested by reduced apoptosis rate and reversal of apoptosis-associated protein expression. It was verified that MAG restored the expression of p-PI3K and p-Akt in FB1-treated cells and reversed the downstream effectors IKKa and NF-KB via measurement of related protein levels. The rescue experiment using Akt pathway activator (SC79) was further confirmed that activation of the PI3K/Akt signaling pathway is an effective strategy for MAG to mitigate FB1-induced cytotoxicity in C6 astroglial cells.

C1 [Wang, Yingjie; Cheng, Dai; He, Jingjing; Liu, Sijia] Tianjin Univ Sci & Technol, State Key Lab Food Nutr & Safety, Tianjin 300457, Peoples R China.

[Wang, Xinlu; Wang, Meng] Beijing Acad Agr & Forestry Sci, Inst Qual Stand & Testing Technol, Beijing 100097, Peoples R China.

C3 Tianjin University Science & Technology; Beijing Academy of Agriculture

& Forestry Sciences (BAAFS)

RP Cheng, D (corresponding author), Tianjin Univ Sci & Technol, State Key Lab Food Nutr & Safety, Tianjin 300457, Peoples R China.; Wang, M (corresponding author), Beijing Acad Agr & Forestry Sci, Inst Qual Stand & Testing Technol, Beijing 100097, Peoples R China.

EM dcheng@tust.edu.cn; wangm@iqstt.cn

FU Beijing Natural Science Foundation [6222012]; National Key Research and

Development Program of China [2022YFF1102800]; Project of Tianjin

Science and Technology Program [22JCYBJC00360, 21ZYJDJC00060]

FX This work was supported by the Beijing Natural Science Foundation (No

6222012) , the National Key Research and Development Program of China

under Grant (2022YFF1102800) , the Project of Tianjin Science and

Technology Program (22JCYBJC00360, 21ZYJDJC00060) . Addi-tional

appreciation was extended to the Research Centre of Modern Analytical

Technology for the support provided.

CR Bashan N, 2009, PHYSIOL REV, V89, P27, DOI 10.1152/physrev.00014.2008

Boyman L, 2020, TRENDS MOL MED, V26, P21, DOI 10.1016/j.molmed.2019.10.007

Chen XR, 2023, TOXINS, V15, DOI 10.3390/toxins15110653

Chen XR, 2023, TOXINS, V15, DOI 10.3390/toxins15090577

Chipuk JE, 2010, MOL CELL, V37, P299, DOI 10.1016/j.molcel.2010.01.025

Chu Q, 2020, J HAZARD MATER, V393, DOI 10.1016/j.jhazmat.2020.122364

Chuang DY, 2013, J NEUROINFLAMM, V10, DOI 10.1186/1742-2094-10-15

Cianciulli A, 2016, INT IMMUNOPHARMACOL, V36, P282, DOI 10.1016/j.intimp.2016.05.007

Du HN, 2024, ENVIRON INT, V184, DOI 10.1016/j.envint.2024.108445

Fakhri S, 2021, PHYTOMEDICINE, V91, DOI 10.1016/j.phymed.2021.153664

Fan X, 2023, ACS NANO, V18, P229, DOI 10.1021/acsnano.3c05732

Fischer M, 2022, TRENDS BIOCHEM SCI, V47, P1009, DOI 10.1016/j.tibs.2022.06.007

Gao F, 2002, CIRCULATION, V105, P1497, DOI 10.1161/01.CIR.0000012529.00367.0F

Gao ZC, 2023, ENVIRON POLLUT, V320, DOI 10.1016/j.envpol.2023.121065

Geng Z, 2023, CNS NEUROSCI THER, V29, P3121, DOI 10.1111/cns.14344

Hakimuddin F, 2006, J AGR FOOD CHEM, V54, P7912, DOI 10.1021/jf060834m

Hong TY, 2024, J HAZARD MATER, V480, DOI 10.1016/j.jhazmat.2024.136364

Hou Y, 2023, PHYTOMEDICINE, V109, DOI 10.1016/j.phymed.2022.154568

Khan MI, 2023, LIFE SCI, V312, DOI 10.1016/j.lfs.2022.121202

Kim DJ, 2016, NEUROTOXICOLOGY, V53, P173, DOI 10.1016/j.neuro.2016.01.001

Krishnaswamy K, 2024, J FOOD SCI, V89, P1280, DOI 10.1111/1750-3841.16869

Larsen BD, 2010, P NATL ACAD SCI USA, V107, P4230, DOI 10.1073/pnas.0913089107

Li N, 2024, AGEING RES REV, V99, DOI 10.1016/j.arr.2024.102398

Li SH, 2024, ENVIRON POLLUT, V363, DOI 10.1016/j.envpol.2024.125210

Li TT, 2024, J ADV RES, V60, P13, DOI 10.1016/j.jare.2023.08.001

Li TT, 2022, FOOD RES INT, V152, DOI 10.1016/j.foodres.2021.110927

Lin MM, 2022, ACTA PHARMACOL SIN, V43, P2439, DOI 10.1038/s41401-022-00879-6

Liu CL, 2019, FOOD FUNCT, V10, P3491, DOI 10.1039/c8fo02557f

Liu T, 2017, SIGNAL TRANSDUCT TAR, V2, DOI 10.1038/sigtrans.2017.23

Liu YL, 2022, BIOMED PHARMACOTHER, V156, DOI 10.1016/j.biopha.2022.113941

Lu XL, 2017, BIOMED PHARMACOTHER, V85, P658, DOI 10.1016/j.biopha.2016.11.077

Ma L, 2020, FOOD FUNCT, V11, P10774, DOI [10.1039/d0fo02165b, 10.1039/D0FO02165B]

Uranga RM, 2013, J BIOL CHEM, V288, P19773, DOI 10.1074/jbc.M113.457622

Meca G, 2010, J AGR FOOD CHEM, V58, P1359, DOI 10.1021/jf9028255

Melo-Silveira RF, 2019, CARBOHYD POLYM, V210, P245, DOI 10.1016/j.carbpol.2019.01.073

Mobio TA, 2000, TOXICOL APPL PHARM, V164, P91, DOI 10.1006/taap.2000.8893

Mortiboys H, 2008, ANN NEUROL, V64, P555, DOI 10.1002/ana.21492

Nolfi-Donegan D, 2020, REDOX BIOL, V37, DOI 10.1016/j.redox.2020.101674

Park HS, 2012, FOOD CHEM TOXICOL, V50, P2407, DOI 10.1016/j.fct.2012.04.034

Patergnani S, 2022, NEURAL REGEN RES, V17, P2563, DOI 10.4103/1673-5374.339473

Pei Xingyao, 2021, Oxid Med Cell Longev, V2021, P9967334, DOI 10.1155/2021/9967334

Pei XY, 2021, FOOD CHEM TOXICOL, V151, DOI 10.1016/j.fct.2021.112134

Pérez-García MJ, 2004, J BIOL CHEM, V279, P6132, DOI 10.1074/jbc.M308367200

Rong Y, 2008, ANNU REV PHYSIOL, V70, P73, DOI 10.1146/annurev.physiol.70.021507.105852

Sampath D, 2003, ONCOGENE, V22, P9063, DOI 10.1038/sj.onc.1207229

Scheideler L, 2013, ACTA BIOMATER, V9, P8740, DOI 10.1016/j.actbio.2013.02.020

Sinha K, 2013, ARCH TOXICOL, V87, P1157, DOI 10.1007/s00204-013-1034-4

Song C, 2024, ENVIRON POLLUT, V356, DOI 10.1016/j.envpol.2024.124359

Stockmann-Juvala H, 2004, TOXICOLOGY, V202, P173, DOI 10.1016/j.tox.2004.05.002

Sun YH, 2023, J HAZARD MATER, V443, DOI 10.1016/j.jhazmat.2022.130252

Wang HL, 2023, SIGNAL TRANSDUCT TAR, V8, DOI 10.1038/s41392-023-01347-1

Wang M, 2011, EUR J PHARMACOL, V650, P41, DOI 10.1016/j.ejphar.2010.09.049

Wang Xinlu, 2024, Chemosphere, V359, P142300, DOI 10.1016/j.chemosphere.2024.142300

Wang YJ, 2024, ECOTOX ENVIRON SAFE, V270, DOI 10.1016/j.ecoenv.2023.115831

Wei ZJ, 2021, ENVIRON POLLUT, V273, DOI 10.1016/j.envpol.2020.115919

Yan FJ, 2017, FOOD RES INT, V102, P213, DOI 10.1016/j.foodres.2017.10.009

Yang DD, 2023, J AGR FOOD CHEM, V71, P16752, DOI 10.1021/acs.jafc.3c05501

Yang DD, 2024, CRIT REV FOOD SCI, V64, P3660, DOI 10.1080/10408398.2022.2134290

Yu S, 2021, SCI TOTAL ENVIRON, V787, DOI 10.1016/j.scitotenv.2021.147405

Zhang FL, 2024, J HAZARD MATER, V468, DOI 10.1016/j.jhazmat.2024.133836

Zhang WF, 2023, SIGNAL TRANSDUCT TAR, V8, DOI 10.1038/s41392-023-01486-5

Zhao L, 2021, FOOD CHEM TOXICOL, V153, DOI 10.1016/j.fct.2021.112256

Zhou FF, 2019, BRAIN BEHAV IMMUN, V77, P161, DOI 10.1016/j.bbi.2018.12.018

NR 63

TC 0

Z9 0

U1 0

U2 0

PU ELSEVIER SCI LTD

PI London

PA 125 London Wall, London, ENGLAND

SN 0269-7491

EI 1873-6424

J9 ENVIRON POLLUT

JI Environ. Pollut.

PD MAY 1

PY 2025

VL 372

AR 126017

DI 10.1016/j.envpol.2025.126017

EA MAR 2025

PG 13

WC Environmental Sciences

WE Science Citation Index Expanded (SCI-EXPANDED)

SC Environmental Sciences & Ecology

GA 0CL5V

UT WOS:001444073600001

PM 40057167

DA 2025-04-09

ER

PT J

AU Kim, JE

Kim, H

An, SSA

Maeng, EH

Kim, MK

Song, YJ

AF Kim, Jung-Eun

Kim, Hyejin

An, Seong Soo A.

Maeng, Eun Ho

Kim, Meyoung-Kon

Song, Yoon-Jae

TI In vitro cytotoxicity of SiO2 or ZnO nanoparticles with

different sizes and surface charges on U373MG human glioblastoma cells

SO INTERNATIONAL JOURNAL OF NANOMEDICINE

LA English

DT Article

DE apoptosis

ID ZINC-OXIDE NANOPARTICLES; AMORPHOUS SILICA NANOPARTICLES; HUMAN

ENDOTHELIAL-CELLS; OXIDATIVE STRESS; DNA-DAMAGE; P53 PATHWAY; APOPTOSIS;

BIOCOMPATIBILITY; TOXICITY; LIVER

AB Silicon dioxide (SiO2) and zinc oxide (ZnO) nanoparticles are widely used in various applications, raising issues regarding the possible adverse effects of these metal oxide nanoparticles on human cells. In this study, we determined the cytotoxic effects of differently charged SiO2 and ZnO nanoparticles, with mean sizes of either 100 or 20 nm, on the U373MG human glioblastoma cell line. The overall cytotoxicity of ZnO nanoparticles against U373MG cells was significantly higher than that of SiO2 nanoparticles. Neither the size nor the surface charge of the ZnO nanoparticles affected their cytotoxicity against U373MG cells. The 20 nm SiO2 nanoparticles were more toxic than the 100 nm nanoparticles against U373MG cells, but the surface charge had little or no effect on their cytotoxicity. Both SiO2 and ZnO nanoparticles activated caspase-3 and induced DNA fragmentation in U373MG cells, suggesting the induction of apoptosis. Thus, SiO2 and ZnO nanoparticles appear to exert cytotoxic effects against U373MG cells, possibly via apoptosis.

C1 [Kim, Jung-Eun; Kim, Hyejin; Song, Yoon-Jae] Gachon Univ, Dept Life Sci, Songnam 461701, South Korea.

[An, Seong Soo A.] Gachon Univ, Dept Bionano Technol, Songnam 461701, South Korea.

[Maeng, Eun Ho] Korea Testing & Res Inst, Seoul, South Korea.

[Kim, Meyoung-Kon] Korea Univ, Med Sch & Coll, Dept Biochem & Mol Biol, Seoul, South Korea.

C3 Gachon University; Gachon University; Korea University; Korea University

Medicine (KU Medicine)

RP Song, YJ (corresponding author), Gachon Univ, Dept Life Sci, San 65,Bokjeong Dong, Songnam 461701, South Korea.

EM jerry@korea.ac.kr; songyj@gachon.ac.kr

RI An, Seong Soo/AFR-4832-2022

FU Ministry of Food and Drug Safety [10182MFDS991]

FX This research was supported by a grant (10182MFDS991) from the Ministry

of Food and Drug Safety in 2010-2011.

CR Ahamed M, 2013, HUM EXP TOXICOL, V32, P186, DOI 10.1177/0960327112459206

Ahamed M, 2011, NANOMED-NANOTECHNOL, V7, P904, DOI 10.1016/j.nano.2011.04.011

Bae HC, 2011, MOL CELL TOXICOL, V7, P333, DOI 10.1007/s13273-011-0042-9

Bauer AT, 2011, BIOMATERIALS, V32, P8385, DOI 10.1016/j.biomaterials.2011.07.078

Cho WS, 2010, ENVIRON HEALTH PERSP, V118, P1699, DOI 10.1289/ehp.1002201

Chung JH, 2010, PLOS GENET, V6, DOI 10.1371/journal.pgen.1000863

Corbalan JJ, 2011, INT J NANOMED, V6, P2821, DOI 10.2147/IJN.S25071

Cuddihy AR, 2003, INT REV CYTOL, V222, P99

Deng ZJ, 2009, NANOTECHNOLOGY, V20, DOI 10.1088/0957-4484/20/45/455101

Duan JC, 2013, PLOS ONE, V8, DOI 10.1371/journal.pone.0062087

Ekkapongpisit M, 2012, INT J NANOMED, V7, P4147, DOI 10.2147/IJN.S33803

Fan ZY, 2005, J NANOSCI NANOTECHNO, V5, P1561, DOI 10.1166/jnn.2005.182

Fine D, 2013, ADV HEALTHC MATER, V2, P632, DOI 10.1002/adhm.201200214

Greish K, 2012, NANOTOXICOLOGY, V6, P713, DOI 10.3109/17435390.2011.604442

Hsiao IL, 2011, SCI TOTAL ENVIRON, V409, P1219, DOI 10.1016/j.scitotenv.2010.12.033

Kermanizadeh A, 2013, NANOTOXICOLOGY, V7, P301, DOI 10.3109/17435390.2011.653416

Kim KM, 2014, PHYSICOCHEM IN PRESS

Kim Kyoung-Min, 2012, Toxicology and Environmental Health Sciences, V4, P121, DOI 10.1007/s13530-012-0126-5

Lee SH, 2012, MOL CELL TOXICOL, V8, P113, DOI 10.1007/s13273-012-0014-8

Lundqvist M, 2011, ACS NANO, V5, P7503, DOI 10.1021/nn202458g

Ma HB, 2013, ENVIRON POLLUT, V172, P76, DOI 10.1016/j.envpol.2012.08.011

Malvindi MA, 2012, NANOSCALE, V4, P486, DOI 10.1039/c1nr11269d

McNamee LM, 2009, GENETICS, V182, P423, DOI 10.1534/genetics.109.102327

Meyer K, 2011, TOXICOL IN VITRO, V25, P1721, DOI 10.1016/j.tiv.2011.08.011

Nasir A, 2010, CLIN DERMATOL, V28, P581, DOI 10.1016/j.clindermatol.2009.06.006

Okoturo-Evans O, 2013, PLOS ONE, V8, DOI 10.1371/journal.pone.0072363

Ou YH, 2005, MOL BIOL CELL, V16, P1684, DOI 10.1091/mbc.E04-08-0689

Park YH, 2013, MOL CELL TOXICOL, V9, P67, DOI 10.1007/s13273-013-0010-7

Pujalté I, 2011, PART FIBRE TOXICOL, V8, DOI 10.1186/1743-8977-8-10

Rabolli V, 2010, NANOTOXICOLOGY, V4, P307, DOI 10.3109/17435390.2010.482749

Sharma V, 2012, APOPTOSIS, V17, P852, DOI 10.1007/s10495-012-0705-6

Sharma V, 2012, MUTAT RES-GEN TOX EN, V745, P84, DOI 10.1016/j.mrgentox.2011.12.009

Sharma V, 2011, J BIOMED NANOTECHNOL, V7, P98, DOI 10.1166/jbn.2011.1220

Song YJ, 2005, J VIROL, V79, P2597, DOI 10.1128/JVI.79.4.2597-2603.2005

Speidel D, 2010, TRENDS CELL BIOL, V20, P14, DOI 10.1016/j.tcb.2009.10.002

Uskokovic V, 2013, J BIOMED NANOTECHNOL, V9, P1441, DOI 10.1166/jbn.2013.1642

Wilhelmi V, 2013, PLOS ONE, V8, DOI 10.1371/journal.pone.0065704

Xia T, 2008, ACS NANO, V2, P2121, DOI 10.1021/nn800511k

Xu ZL, 2012, J APPL TOXICOL, V32, P358, DOI 10.1002/jat.1710

NR 39

TC 36

Z9 38

U1 1

U2 33

PU DOVE MEDICAL PRESS LTD

PI ALBANY

PA PO BOX 300-008, ALBANY, AUCKLAND 0752, NEW ZEALAND

SN 1178-2013

J9 INT J NANOMED

JI Int. J. Nanomed.

PD DEC 15

PY 2014

VL 9

SU 2

BP 235

EP 241

DI 10.2147/IJN.S57936

PG 7

WC Nanoscience & Nanotechnology; Pharmacology & Pharmacy

WE Science Citation Index Expanded (SCI-EXPANDED)

SC Science & Technology - Other Topics; Pharmacology & Pharmacy

GA AW7KO

UT WOS:000346443200022

PM 25565841

OA Green Submitted, gold, Green Published

DA 2025-04-09

ER

PT J

AU Bhowmick, R

Sarkar, RR

AF Bhowmick, Rupa

Sarkar, Ram Rup

TI Differential suitability of reactive oxygen species and the role of

glutathione in regulating paradoxical behavior in gliomas: A

mathematical perspective

SO PLOS ONE

LA English

DT Article

ID OXIDATIVE STRESS; GLIOBLASTOMA CELLS; HYDROGEN-PEROXIDE; REDOX

REGULATION; CANCER; ROS; PROLIFERATION; RATIO; MITOCHONDRIA; CONNECTION

AB Manipulative strategies of ROS in cancer are often exhibited as changes in the redox and thiol ratio of the cells. Cellular responses to oxidative insults are generated in response to these changes which are triggered due to the rerouting of the metabolic framework to maintain survival under stress. However, mechanisms of these metabolic re-routing are not clearly understood and remained debatable. In the present work, we have designed a context-based dynamic metabolic model to establish that the coordinated functioning of glutathione peroxidase (GTHP), glutathione oxidoreductase (GTHO) and NADPH oxidase (NOX) is crucial in determining cancerous transformation, specifically in gliomas. Further, we propose that the puzzling duality of ROS (represented by changes inh(2)o(2)in the present model) in exhibiting varying cellular fates can be determined by considering simultaneous changes innadph/nadp(+)andgsh/gssgthat occur during the reprogramming of metabolic reactions. This will be helpful in determining the pro-apoptotic or anti-apoptotic fate of gliomas and can be useful in designing effective pro-oxidant and/or anti-oxidant therapeutic approaches against gliomas.

C1 [Bhowmick, Rupa; Sarkar, Ram Rup] CSIR Natl Chem Lab, Chem Engn & Proc Dev Div, Pune, Maharashtra, India.

[Bhowmick, Rupa; Sarkar, Ram Rup] Acad Sci & Innovat Res AcSIR, Ghaziabad, India.

C3 Council of Scientific & Industrial Research (CSIR) - India; CSIR -

National Chemical Laboratory (NCL); Academy of Scientific & Innovative

Research (AcSIR)

RP Sarkar, RR (corresponding author), CSIR Natl Chem Lab, Chem Engn & Proc Dev Div, Pune, Maharashtra, India.; Sarkar, RR (corresponding author), Acad Sci & Innovat Res AcSIR, Ghaziabad, India.

EM rr.sarkar@ncl.res.in

OI Bhowmick, Rupa/0000-0001-5812-0513; SARKAR, RAM RUP/0000-0001-7115-163X

FU SERB, Department of Science and Technology, Govt. of India

[EMR/2016/000516, DST/ICPS/EDA/2018]; Council of Scientific & Industrial

Research (CSIR)

FX We thank SERB, Department of Science and Technology, Govt. of India

(File No. EMR/2016/000516) and (DST/ICPS/EDA/2018), for providing

financial support to Ram Rup Sarkar. Rupa Bhowmick acknowledges the

Council of Scientific & Industrial Research (CSIR) for the Senior

Research Fellowship. The funders had no role in study design, data

collection and analysis, decision to publish, or preparation of the

manuscript.

CR Agledal L, 2010, REDOX REP, V15, P2, DOI 10.1179/174329210X12650506623122

Amelio I, 2014, TRENDS BIOCHEM SCI, V39, P191, DOI 10.1016/j.tibs.2014.02.004

[Anonymous], 2017, LANCET HIV, V2017, pPE475

[Anonymous], 2015, ALZHEIMERS DEMENTI S, V11, pP121

Aoyama K, 2013, INT J MOL SCI, V14, P21021, DOI 10.3390/ijms141021021

Aquilano K, 2014, FRONT PHARMACOL, V5, DOI 10.3389/fphar.2014.00196

Aylett SB, 2013, NEUROCHEM INT, V63, P750, DOI 10.1016/j.neuint.2013.10.002

Bansal A, 2018, J CELL BIOL, V217, P2291, DOI 10.1083/jcb.201804161

Bhowmick Rupa, 2015, Syst Synth Biol, V9, P159, DOI 10.1007/s11693-015-9183-9

Bienert GP, 2006, BBA-BIOMEMBRANES, V1758, P994, DOI 10.1016/j.bbamem.2006.02.015

Blacker TS, 2016, FREE RADICAL BIO MED, V100, P53, DOI 10.1016/j.freeradbiomed.2016.08.010

Blanchetot C, 2008, CRIT REV EUKAR GENE, V18, P35, DOI 10.1615/CritRevEukarGeneExpr.v18.i1.30

Blanco G., 2017, Medical biochemistry

Borbély G, 2010, J MED CHEM, V53, P6758, DOI 10.1021/jm1004368

Dokic I, 2012, GLIA, V60, P1785, DOI 10.1002/glia.22397

Galadari S, 2017, FREE RADICAL BIO MED, V104, P144, DOI 10.1016/j.freeradbiomed.2017.01.004

Galanis A, 2008, CANCER LETT, V266, P12, DOI 10.1016/j.canlet.2008.02.028

Gali RR, 1997, BIOCHEM J, V321, P207, DOI 10.1042/bj3210207

Gould RL, 2019, NUTRIENTS, V11, DOI 10.3390/nu11051056

Haack F, 2015, PLOS COMPUT BIOL, V11, DOI 10.1371/journal.pcbi.1004106

Halliwell B, 2000, LANCET, V355, P1179, DOI 10.1016/S0140-6736(00)02075-4

Herrera B, 2001, FASEB J, V15, P741, DOI 10.1096/fj.00-0267com

Hole PS, 2013, BLOOD, V122, P3322, DOI 10.1182/blood-2013-04-491944

Hosios AM, 2018, J BIOL CHEM, V293, P7490, DOI 10.1074/jbc.TM117.000239

Huster D, 2000, NEUROCHEM INT, V36, P461, DOI 10.1016/S0197-0186(99)00149-7

Janes KA, 2013, J CELL SCI, V126, P1913, DOI 10.1242/jcs.112045

Jeon SM, 2012, NATURE, V485, P661, DOI 10.1038/nature11066

Kong Q, 2000, MED HYPOTHESES, V55, P29, DOI 10.1054/mehy.1999.0982

Lewerenz J, 2013, ANTIOXID REDOX SIGN, V18, P522, DOI 10.1089/ars.2011.4391

Liao BR, 1996, ARCH BIOCHEM BIOPHYS, V327, P53, DOI 10.1006/abbi.1996.0092

Lim JKM, 2019, P NATL ACAD SCI USA, V116, P9433, DOI 10.1073/pnas.1821323116

Maldonado EN, 2014, MITOCHONDRION, V19, P78, DOI 10.1016/j.mito.2014.09.002

Martin KR, 2002, HUM EXP TOXICOL, V21, P71, DOI 10.1191/0960327102ht213oa

MEIER B, 1989, BIOCHEM J, V263, P539, DOI 10.1042/bj2630539

MORIKOFERZWEZ S, 1989, BIOCHEM J, V259, P117

Pereira EJ, 2016, FRONT PHARMACOL, V7, DOI 10.3389/fphar.2016.00457

PIERCE GB, 1991, DIFFERENTIATION, V46, P181, DOI 10.1111/j.1432-0436.1991.tb00880.x

Pollak N, 2007, BIOCHEM J, V402, P205, DOI 10.1042/BJ20061638

Qu Y, 2011, J CLIN INVEST, V121, P212, DOI 10.1172/JCI43144

Raftos JE, 2010, J BIOL CHEM, V285, P23557, DOI 10.1074/jbc.M109.067017

Rao GM, 2000, CLIN CHIM ACTA, V296, P203, DOI 10.1016/S0009-8981(00)00219-9

Ren JG, 2014, SCI REP-UK, V4, DOI 10.1038/srep05414

Rinaldi M, 2016, INT J MOL SCI, V17, DOI 10.3390/ijms17060984

Ruiz-Ginés JA, 2000, J CARDIOVASC PHARM, V35, P109, DOI 10.1097/00005344-200001000-00014

Sauer H, 2001, CELL PHYSIOL BIOCHEM, V11, P173, DOI 10.1159/000047804

Schumacker PT, 2006, CANCER CELL, V10, P175, DOI 10.1016/j.ccr.2006.08.015

Sharma P, 2008, IMMUNITY, V29, P551, DOI 10.1016/j.immuni.2008.07.019

Sheeran FL, 2010, BBA-BIOENERGETICS, V1797, P1138, DOI 10.1016/j.bbabio.2010.04.002

Shono T, 2008, INT J CANCER, V123, P787, DOI 10.1002/ijc.23569

Simon HU, 2000, APOPTOSIS, V5, P415, DOI 10.1023/A:1009616228304

Smith GR, 2013, BMC SYST BIOL, V7, DOI 10.1186/1752-0509-7-41

Stankova J, 2008, CURR PHARM DESIGN, V14, P1143, DOI 10.2174/138161208784246171

SZATROWSKI TP, 1991, CANCER RES, V51, P794

Tantama M, 2013, NAT COMMUN, V4, DOI 10.1038/ncomms3550

Tedeschi PM, 2016, CLIN CANCER RES, V22, P5189, DOI 10.1158/1078-0432.CCR-16-1129

Wang J, 2008, CANCER BIOL THER, V7, P1875, DOI 10.4161/cbt.7.12.7067

Wang YP, 2016, MOL CELL, V64, P673, DOI 10.1016/j.molcel.2016.09.028

Ye ZC, 1999, CANCER RES, V59, P4383

Ye ZC, 1999, J NEUROSCI, V19, P10767, DOI 10.1523/JNEUROSCI.19-24-10767.1999

Zhang HY, 2009, TOXICOL SCI, V110, P376, DOI 10.1093/toxsci/kfp101

Zhong WX, 1999, FREE RADICAL BIO MED, V27, P1334, DOI 10.1016/S0891-5849(99)00181-1

Zhu ZL, 2018, J NEUROCHEM, V144, P93, DOI 10.1111/jnc.14250

Zitka O, 2012, ONCOL LETT, V4, P1247, DOI 10.3892/ol.2012.931

NR 63

TC 9

Z9 9

U1 2

U2 6

PU PUBLIC LIBRARY SCIENCE

PI SAN FRANCISCO

PA 1160 BATTERY STREET, STE 100, SAN FRANCISCO, CA 94111 USA

SN 1932-6203

J9 PLOS ONE

JI PLoS One

PD JUN 25

PY 2020

VL 15

IS 6

AR e0235204

DI 10.1371/journal.pone.0235204

PG 23

WC Multidisciplinary Sciences

WE Science Citation Index Expanded (SCI-EXPANDED)

SC Science & Technology - Other Topics

GA MG0TI

UT WOS:000545747200066

PM 32584884

OA Green Published, gold

DA 2025-04-09

ER

PT J

AU Wang, ZX

Liu, F

Liao, WL

Yu, LZ

Hu, ZW

Li, MC

Xia, HL

AF Wang, Zexia

Liu, Fei

Liao, Wenli

Yu, Liangzhu

Hu, Zhenwu

Li, Mincai

Xia, Hongli

TI Curcumin suppresses glioblastoma cell proliferation by p-AKT/mTOR

pathway and increases the PTEN expression

SO ARCHIVES OF BIOCHEMISTRY AND BIOPHYSICS

LA English

DT Article

DE Glioblastoma; Curcumin; PTEN; AKT; Mechanism

ID DIETARY PHYTOCHEMICALS; OXIDATIVE STRESS; CANCER; APOPTOSIS; AUTOPHAGY;

INFLAMMATION; P53

AB Background: Glioblastoma (GB) is the most common neoplasm in the brain. Curcumin, as a known polyphenolic compound extracted from turmeric, is a chemotherapy used in some cancer treatments in China. However, the effect of curcumin on the survivability of GB cells remains to be elucidated.

Methods: We performed a CCK8 assay to detect the viability of GB cells following treatments with curcumin and examined the migration and invasion the ability of these cells using the wound-healing and transwell invasion assays. The cell proliferation and apoptotic proteins were detected by Western blot analyses. We utilized a glioblastoma-xenograft mouse model to assess cell proliferation following curcumin treatment.

Results: We found that curcumin inhibited the proliferation, migration, and invasion of U251 and U87 GB cells. We detected that curcumin decreased p-AKT and p-mTOR protein expression, and promoted the apoptosis of U251 and U87 GB cells. Further, we found that curcumin promoted the PTEN and p53 expression, as the tumor suppressor genes. In addition, we administered curcumin to nude mice and found that curcumin decreased the tumor volume, caused necrosis of tumor tissue, and significantly enhanced the PTEN and p53 expression in vivo.

Conclusions: These results indicated that curcumin inhibited proliferation by decreasing the p-AKT/p-mTOR pathway and promoted apoptosis by increasing the PTEN and p53 expression. Our study provided the molecular mechanisms by which curcumin inhibited glioblastoma and its targeted interventions.

C1 [Wang, Zexia; Liu, Fei; Liao, Wenli; Yu, Liangzhu; Hu, Zhenwu; Li, Mincai; Xia, Hongli] Hubei Univ Sci & Technol, Sch Basic Med Sci, Xianning 437100, Peoples R China.

[Liu, Fei; Li, Mincai] Hubei Univ Sci & Technol, Sch Pharm, Xianning 437100, Peoples R China.

[Xia, Hongli] Hubei Univ Sci & Technol, Cent Hosp Xianning, Xianning 437100, Peoples R China.

[Wang, Zexia] Henan Shengde Hosp, Dept Pharm, Xinyang 464000, Peoples R China.

C3 Hubei University of Science & Technology; Hubei University of Science &

Technology; Hubei University of Science & Technology

RP Li, MC (corresponding author), Hubei Univ Sci & Technol, Sch Basic Med Sci, Xianning 437100, Peoples R China.; Xia, HL (corresponding author), Hubei Univ Sci & Technol, Cent Hosp Xianning, Xianning 437100, Peoples R China.

EM 454544454@qq.com; 2440032910@qq.com; roxana822@163.com;

yuliangyu73@163.com; huzhenwu@hotmail.com; mincaili@163.com;

mobei_hu@126.com

RI yu, liangzhu/AAE-1941-2019

FU Fund of the Science and Technology Department of Hubei Province

[2018CFB737]; Fund of Hubei University of Science and Technology

[2019-21GP12,2019-20xz01]

FX This work was supported by the grants from the Fund of the Science and

Technology Department of Hubei Province (2018CFB737) and the Fund of

Hubei University of Science and Technology (2019-21GP12,2019-20xz01). No

specific funding was received for this study.

CR Bhattacharya K, 2016, ONCOGENESIS, V5, DOI 10.1038/oncsis.2016.34

Chikara S, 2018, CANCER LETT, V413, P122, DOI 10.1016/j.canlet.2017.11.002

Daniele S, 2015, SCI REP-UK, V5, DOI 10.1038/srep09956

Goodenberger ML, 2012, CANCER GENET-NY, V205, P613, DOI 10.1016/j.cancergen.2012.10.009

Gupta SC, 2011, ANN NY ACAD SCI, V1215, P150, DOI 10.1111/j.1749-6632.2010.05852.x

Hassan M, 2014, BIOMED RES INT, V2014, DOI 10.1155/2014/150845

Huang ZQ, 2015, MOL MED REP, V11, P4678, DOI 10.3892/mmr.2015.3322

Jiao DM, 2016, MOL THER-ONCOLYTICS, V3, DOI 10.1038/mto.2016.18

Kang R, 2011, CELL DEATH DIFFER, V18, P571, DOI 10.1038/cdd.2010.191

Lee MS, 2015, NAT COMMUN, V6, DOI 10.1038/ncomms8769

Lee YR, 2018, NAT REV MOL CELL BIO, V19, P547, DOI 10.1038/s41580-018-0015-0

Li MC, 2016, CARDIOVASC TOXICOL, V16, P172, DOI 10.1007/s12012-015-9326-y

Li MC, 2012, EUR J PHARMACOL, V696, P203, DOI 10.1016/j.ejphar.2012.09.033

Li MC, 2010, EXP MOL PATHOL, V88, P250, DOI 10.1016/j.yexmp.2009.12.001

Maiti P, 2019, PLOS ONE, V14, DOI 10.1371/journal.pone.0225660

Miao YP, 2016, BRAIN RES BULL, V121, P9, DOI 10.1016/j.brainresbull.2015.11.019

Quail DF, 2017, CANCER CELL, V31, P326, DOI 10.1016/j.ccell.2017.02.009

Rana C, 2015, MOL CELL BIOCHEM, V402, P225, DOI 10.1007/s11010-015-2330-5

Ravindranathan P, 2018, SCI REP-UK, V8, DOI 10.1038/s41598-018-32267-8

Shang N, 2018, CELL DEATH DIS, V9, DOI 10.1038/s41419-018-0617-7

Shankar S, 2007, INT J ONCOL, V30, P905

Shukla S, 2014, CANCER LETT, V355, P9, DOI 10.1016/j.canlet.2014.09.017

Tian BQ, 2017, J DRUG TARGET, V25, P626, DOI 10.1080/1061186X.2017.1306535

Tu SP, 2012, CANCER PREV RES, V5, P205, DOI 10.1158/1940-6207.CAPR-11-0247

Tu XK, 2014, INFLAMMATION, V37, P1544, DOI 10.1007/s10753-014-9881-6

Tveden-Nyborg P, 2018, BASIC CLIN PHARMACOL, V123, P233, DOI 10.1111/bcpt.13059

Wang AL, 2017, EXP THER MED, V14, P5075, DOI 10.3892/etm.2017.5172

Wang SS, 2018, CELL DEATH DIS, V9, DOI 10.1038/s41419-018-1036-5

Wirawan E, 2010, CELL DEATH DIS, V1, DOI 10.1038/cddis.2009.16

Zhang HH, 2018, MOL CARCINOGEN, V57, P44, DOI 10.1002/mc.22718

Zhang PL, 2017, ONCOL LETT, V13, P1789, DOI 10.3892/ol.2017.5654

Zhong WH, 2016, BIOMED PHARMACOTHER, V83, P302, DOI 10.1016/j.biopha.2016.06.036

NR 32

TC 75

Z9 78

U1 1

U2 24

PU ELSEVIER SCIENCE INC

PI NEW YORK

PA STE 800, 230 PARK AVE, NEW YORK, NY 10169 USA

SN 0003-9861

EI 1096-0384

J9 ARCH BIOCHEM BIOPHYS

JI Arch. Biochem. Biophys.

PD AUG 15

PY 2020

VL 689

AR 108412

DI 10.1016/j.abb.2020.108412

PG 7

WC Biochemistry & Molecular Biology; Biophysics

WE Science Citation Index Expanded (SCI-EXPANDED)

SC Biochemistry & Molecular Biology; Biophysics

GA ML3GS

UT WOS:000549359300006

PM 32445778

DA 2025-04-09

ER

PT J

AU Conway, GE

He, ZL

Hutanu, AL

Cribaro, GP

Manaloto, E

Casey, A

Traynor, D

Milosavljevic, V

Howe, O

Barcia, C

Murray, JT

Cullen, PJ

Curtin, JF

AF Conway, Gillian E.

He, Zhonglei

Hutanu, Ana Lacramioara

Cribaro, George Paul

Manaloto, Eline

Casey, Alan

Traynor, Damien

Milosavljevic, Vladimir

Howe, Orla

Barcia, Carlos

Murray, James T.

Cullen, Patrick J.

Curtin, James F.

TI Cold Atmospheric Plasma induces accumulation of lysosomes and

caspase-independent cell death in U373MG glioblastoma multiforme cells

SO SCIENTIFIC REPORTS

LA English

DT Article

ID MEMBRANE PERMEABILIZATION; NOMENCLATURE COMMITTEE; ADJUVANT

TEMOZOLOMIDE; MOLECULAR-MECHANISMS; OXIDATIVE STRESS; AUTOPHAGY;

RECOMMENDATIONS; RADIOTHERAPY; 3-METHYLADENINE; APOPTOSIS

AB Room temperature Cold Atmospheric Plasma (CAP) has shown promising efficacy for the treatment of cancer but the exact mechanisms of action remain unclear. Both apoptosis and necrosis have been implicated as the mode of cell death in various cancer cells. We have previously demonstrated a caspase-independent mechanism of cell death in p53-mutated glioblastoma multiforme (GBM) cells exposed to plasma. The purpose of this study was to elucidate the molecular mechanisms involved in caspase-independent cell death induced by plasma treatment. We demonstrate that plasma induces rapid cell death in GBM cells, independent of caspases. Accumulation of vesicles was observed in plasma treated cells that stained positive with acridine orange. Western immunoblotting confirmed that autophagy is not activated following plasma treatment. Acridine orange intensity correlates closely with the lysosomal marker Lyso Tracker (TM) Deep Red. Further investigation using isosurface visualisation of confocal imaging confirmed that lysosomal accumulation occurs in plasma treated cells. The accumulation of lysosomes was associated with concomitant cell death following plasma treatment. In conclusion, we observed rapid accumulation of acidic vesicles and cell death following CAP treatment in GBM cells. We found no evidence that either apoptosis or autophagy, however, determined that a rapid accumulation of late stage endosomes/lysosomes precedes membrane permeabilisation, mitochondrial membrane depolarisation and caspase independent cell death.

C1 [Conway, Gillian E.; He, Zhonglei; Manaloto, Eline; Cullen, Patrick J.; Curtin, James F.] Technol Univ Dublin, Sch Food Sci & Environm Hlth, Dublin, Ireland.

[Conway, Gillian E.; He, Zhonglei; Manaloto, Eline; Casey, Alan; Traynor, Damien; Milosavljevic, Vladimir; Howe, Orla; Cullen, Patrick J.; Curtin, James F.] Technol Univ Dublin, FOCAS Res Inst, Dublin, Ireland.

[Conway, Gillian E.; He, Zhonglei; Manaloto, Eline; Howe, Orla; Cullen, Patrick J.; Curtin, James F.] Technol Univ Dublin, Environm Sustainabil & Hlth Inst, Dublin, Ireland.

[Casey, Alan; Milosavljevic, Vladimir] Technol Univ Dublin, Sch Phys & Clin & Optometr Sci, Dublin, Ireland.

[Howe, Orla] Technol Univ Dublin, Sch Biol & Hlth Sci, Dublin, Ireland.

[Conway, Gillian E.] Swansea Univ, Med Sch, Inst Life Sci, In Vitro Toxicol Grp, Singleton Pk, Swansea, W Glam, Wales.

[Hutanu, Ana Lacramioara; Murray, James T.] Trinity Coll Dublin, Sch Biochem & Immunol, Trinity Biomed Sci Inst, Dublin, Ireland.

[Cribaro, George Paul; Barcia, Carlos] Univ Autonoma Barcelona, Sch Med, Inst Neurociencies, Barcelona, Spain.

[Cribaro, George Paul; Barcia, Carlos] Univ Autonoma Barcelona, Sch Med, Dept Biochem & Mol Biol, Barcelona, Spain.

[Cullen, Patrick J.] Univ Sydney, Sch Chem & Biomol Engn, Darlington, Australia.

C3 Swansea University; Trinity College Dublin; Autonomous University of

Barcelona; Autonomous University of Barcelona; University of Sydney

RP Conway, GE; Curtin, JF (corresponding author), Technol Univ Dublin, Sch Food Sci & Environm Hlth, Dublin, Ireland.; Conway, GE; Curtin, JF (corresponding author), Technol Univ Dublin, FOCAS Res Inst, Dublin, Ireland.; Conway, GE; Curtin, JF (corresponding author), Technol Univ Dublin, Environm Sustainabil & Hlth Inst, Dublin, Ireland.; Conway, GE (corresponding author), Swansea Univ, Med Sch, Inst Life Sci, In Vitro Toxicol Grp, Singleton Pk, Swansea, W Glam, Wales.

EM gillian.conway@swansea.ac.uk; james.curtin@tudublin.ie

RI Cullen, PJ/W-9341-2019; He, Zhonglei/AAK-6100-2021; Curtin,

James/B-1669-2008; Barcia, Carlos/I-8205-2014

OI Hutanu, Ana Lacramioara/0009-0006-0012-5585; Conway,

Gillian/0000-0002-5991-0960; He, Zhonglei/0000-0001-6533-4974; Curtin,

James/0000-0002-9320-9254; Casey, Alan/0000-0001-9082-0579; Barcia,

Carlos/0000-0003-0976-4245

FU Irish Research Council IRCSET grant; DIT Fiosraigh Research Scholarship

programme; Science Foundation Ireland [11/PI/08, 14/IA/2626]; Spanish

Ministry of Economy and Competitiveness; European Regional Development

Fund [SAF2015-64123-P]; Science Foundation Ireland (SFI) [14/IA/2626]

Funding Source: Science Foundation Ireland (SFI)

FX This work is supported by Irish Research Council IRCSET grant (G.E.C.),

DIT Fiosraigh Research Scholarship programme (Z.H., E.M.), Science

Foundation Ireland Grant Number 11/PI/08 (A.C.), Science Foundation

Ireland Grant Number 14/IA/2626 (V.M., P.C. and J.C.), Spanish Ministry

of Economy and Competitiveness and European Regional Development Fund

Grant number SAF2015-64123-P (C.B. and G.P.C.). The authors also thank

the FOCAS Research Institute, TU Dublin and Institut de Neurociencies at

UAB for the use of facilities.

CR Adachi T, 2015, FREE RADICAL BIO MED, V79, P28, DOI 10.1016/j.freeradbiomed.2014.11.014

Ahn HJ, 2014, PLOS ONE, V9, DOI 10.1371/journal.pone.0086173

Ahn HJ, 2011, PLOS ONE, V6, DOI 10.1371/journal.pone.0028154

Aits S, 2013, J CELL SCI, V126, P1905, DOI 10.1242/jcs.091181

Altman BJ, 2012, CSH PERSPECT BIOL, V4, DOI 10.1101/cshperspect.a008763

Babington P, 2015, BIOINTERPHASES, V10, DOI 10.1116/1.4915264

Begg AC, 2011, NAT REV CANCER, V11, P239, DOI 10.1038/nrc3007

Boehm D., 2017, ANTICANCER AGENTS ME, V17

Boehm D, 2016, SCI REP-UK, V6, DOI 10.1038/srep21464

Booth LA, 2014, CELL SIGNAL, V26, P549, DOI 10.1016/j.cellsig.2013.11.028

Boya P, 2008, ONCOGENE, V27, P6434, DOI 10.1038/onc.2008.310

BUJA LM, 1993, ARCH PATHOL LAB MED, V117, P1208

Conway GE, 2016, BRIT J CANCER, V114, P435, DOI 10.1038/bjc.2016.12

Curtin J. F., 2003, ESSAYS BIOCH, V39

Datta K, 2002, INT J BIOCHEM CELL B, V34, P148, DOI 10.1016/S1357-2725(01)00106-6

Declercq W, 2009, CELL, V138, P229, DOI 10.1016/j.cell.2009.07.006

Elmore SP, 2001, FASEB J, V15, P2286, DOI 10.1096/fj.01-0206fje

Elmore SA, 2016, TOXICOL PATHOL, V44, P173, DOI 10.1177/0192623315625859

Fietta P., RIV BIOL, V99, P69

Fine HA, 2005, NAT CLIN PRACT ONCOL, V2, P334, DOI 10.1038/ncponc0204

Galluzzi L, 2012, CELL DEATH DIFFER, V19, P107, DOI 10.1038/cdd.2011.96

Galluzzi L, 2018, CELL DEATH DIFFER, V25, P486, DOI 10.1038/s41418-017-0012-4

Guicciardi ME, 2004, ONCOGENE, V23, P2881, DOI 10.1038/sj.onc.1207512

He ZL, 2018, SCI REP-UK, V8, DOI 10.1038/s41598-018-23262-0

Hirst AM, 2015, BRIT J CANCER, V112, P1536, DOI 10.1038/bjc.2015.113

Hou J, 2015, BMC GENOMICS, V16, DOI 10.1186/s12864-015-1644-8

Ishaq M, 2014, MOL BIOL CELL, V25, P1523, DOI 10.1091/mbc.E13-10-0590

Ito S, 2007, INT J ONCOL, V31, P261

Kalghatgi S, 2011, PLOS ONE, V6, DOI 10.1371/journal.pone.0016270

Karna P, 2010, J BIOL CHEM, V285, P18737, DOI 10.1074/jbc.M109.091694

Katsuragi Y, 2015, FEBS J, V282, P4672, DOI 10.1111/febs.13540

Keidar M, 2011, BRIT J CANCER, V105, P1295, DOI 10.1038/bjc.2011.386

Keidar M, 2013, PHYS PLASMAS, V20, DOI 10.1063/1.4801516

Kirkegaard T, 2009, BBA-MOL CELL RES, V1793, P746, DOI 10.1016/j.bbamcr.2008.09.008

Köritzer J, 2013, PLOS ONE, V8, DOI 10.1371/journal.pone.0064498

Kondo Y, 2005, NAT REV CANCER, V5, P726, DOI 10.1038/nrc1692

Kong MG, 2011, J PHYS D APPL PHYS, V44, DOI 10.1088/0022-3727/44/17/174018

Kroemer G, 2005, CELL DEATH DIFFER, V12, P1463, DOI 10.1038/sj.cdd.4401724

Kroemer G, 2009, CELL DEATH DIFFER, V16, P3, DOI 10.1038/cdd.2008.150

Kroemer G, 2007, PHYSIOL REV, V87, P99, DOI 10.1152/physrev.00013.2006

Kurz T, 2008, BBA-GEN SUBJECTS, V1780, P1291, DOI 10.1016/j.bbagen.2008.01.009

Lee J, 2012, BIOCHEM J, V441, P523, DOI 10.1042/BJ20111451

Leng SL, 2013, INT J CANCER, V133, P2781, DOI 10.1002/ijc.28301

Levine B, 2008, CELL, V132, P27, DOI 10.1016/j.cell.2007.12.018

Ma YH, 2014, PLOS ONE, V9, DOI 10.1371/journal.pone.0091947

Milosavljevic V, 2017, EUR PHYS J-APPL PHYS, V80, DOI 10.1051/epjap/2017170201

Mizushima N, 2008, NATURE, V451, P1069, DOI 10.1038/nature06639

Mpoke SS, 1997, J HISTOCHEM CYTOCHEM, V45, P675, DOI 10.1177/002215549704500505

Paglin S, 2001, CANCER RES, V61, P439

Petiot A, 2000, J BIOL CHEM, V275, P992, DOI 10.1074/jbc.275.2.992

Proskuryakov SY, 2003, EXP CELL RES, V283, P1, DOI 10.1016/S0014-4827(02)00027-7

Pugsley HR, 2017, METHODS, V112, P147, DOI 10.1016/j.ymeth.2016.05.022

Repnik U, 2012, BBA-PROTEINS PROTEOM, V1824, P22, DOI 10.1016/j.bbapap.2011.08.016

Saito T, 2015, CIRC RES, V116, P1477, DOI 10.1161/CIRCRESAHA.116.303790

Serrano-Puebla A, 2016, ANN NY ACAD SCI, V1371, P30, DOI 10.1111/nyas.12966

Siu A, 2015, PLOS ONE, V10, DOI 10.1371/journal.pone.0126313

Stoffels E, 2003, J PHYS D APPL PHYS, V36, P2908, DOI 10.1088/0022-3727/36/23/007

Stupp R, 2005, NEW ENGL J MED, V352, P987, DOI 10.1056/NEJMoa043330

Tait SWG, 2008, ONCOGENE, V27, P6452, DOI 10.1038/onc.2008.311

Thakor AS, 2013, CA-CANCER J CLIN, V63, P395, DOI 10.3322/caac.21199

Vandamme M, 2012, INT J CANCER, V130, P2185, DOI 10.1002/ijc.26252

Vanden Berghe T, 2013, METHODS, V61, P117, DOI 10.1016/j.ymeth.2013.02.011

von Woedtke T, 2013, PHYS REP, V530, P291, DOI 10.1016/j.physrep.2013.05.005

Wang FJ, 2018, TRAFFIC, V19, P918, DOI 10.1111/tra.12613

Wu YT, 2010, J BIOL CHEM, V285, P10850, DOI 10.1074/jbc.M109.080796

NR 65

TC 36

Z9 36

U1 0

U2 22

PU NATURE PORTFOLIO

PI BERLIN

PA HEIDELBERGER PLATZ 3, BERLIN, 14197, GERMANY

SN 2045-2322

J9 SCI REP-UK

JI Sci Rep

PD SEP 9

PY 2019

VL 9

AR 12891

DI 10.1038/s41598-019-49013-3

PG 12

WC Multidisciplinary Sciences

WE Science Citation Index Expanded (SCI-EXPANDED)

SC Science & Technology - Other Topics

GA IW0MZ

UT WOS:000484657300015

PM 31501494

OA Green Published, gold

DA 2025-04-09

ER

PT J

AU Chen, YH

McGowan, LD

Cimino, PJ

Dahiya, S

Leonard, JR

Lee, DY

Gutmann, DH

AF Chen, Yi-Hsien

McGowan, Lucy D'Agostino

Cimino, Patrick J.

Dahiya, Sonika

Leonard, Jeffrey R.

Lee, Da Yong

Gutmann, David H.

TI Mouse Low-Grade Gliomas Contain Cancer Stem Cells with Unique Molecular

and Functional Properties

SO CELL REPORTS

LA English

DT Article

ID ENDOPLASMIC-RETICULUM STRESS; ACUTE MYELOID-LEUKEMIA; RADIAL GLIA CELLS;

OPTIC PATHWAY; IN-VITRO; PILOCYTIC ASTROCYTOMA; TUBEROUS SCLEROSIS;

CHOLESTEROL EFFLUX; HUMAN GLIOBLASTOMA; OXIDATIVE STRESS

AB The availability of adult malignant glioma stem cells (GSCs) has provided unprecedented opportunities to identify the mechanisms underlying treatment resistance. Unfortunately, there is a lack of comparable reagents for the study of pediatric low-grade glioma (LGG). Leveraging a neurofibromatosis 1 (Nf1) genetically engineered mouse LGG model, we report the isolation of CD133(+) multi-potent low-grade glioma stem cells (LG-GSCs), which generate gliomalike lesions histologically similar to the parent tumor following injection into immunocompetent hosts. In addition, we demonstrate that these LG-GSCs harbor selective resistance to currently employed conventional and biologically targeted anti-cancer agents, which reflect the acquisition of new targetable signaling pathway abnormalities. Using transcriptomic analysis to identify additional molecular properties, we discovered that mouse and human LG-GSCs harbor high levels of Abcg1 expression critical for protecting against ER-stress-induced mouse LG-GSC apoptosis. Collectively, these findings establish that LGG cancer stem cells have unique molecular and functional properties relevant to brain cancer treatment.

C1 [Chen, Yi-Hsien; McGowan, Lucy D'Agostino; Lee, Da Yong; Gutmann, David H.] Washington Univ, Sch Med, Dept Neurol, St Louis, MO 63110 USA.

[Cimino, Patrick J.; Dahiya, Sonika] Washington Univ, Sch Med, Dept Pathol, St Louis, MO 63110 USA.

[Leonard, Jeffrey R.] Washington Univ, Sch Med, Dept Neurosurg, St Louis, MO 63110 USA.

C3 Washington University (WUSTL); Washington University (WUSTL); Washington

University (WUSTL)

RP Gutmann, DH (corresponding author), Washington Univ, Sch Med, Dept Neurol, St Louis, MO 63110 USA.

EM gutmannd@neuro.wustl.edu

OI Dahiya, Sonika/0000-0002-5585-0964; Gutmann, David/0000-0002-3127-5045;

D'Agostino McGowan, Lucy/0000-0001-7297-9359; Cimino,

P.J./0000-0003-0441-4502

FU Department of Defense [NF120032]; NIH [R01-NS065547-01]; American Brain

Tumor Association - Emily Dorfman Foundation for Children

FX We thank Dr. Sara Taylor and Scott Gianino for technical assistance, Dr.

Jason Weber for providing HeLa cells, and the Broad Institute RNAi

Consortium (TRC), the Children's Discovery Institute (CDI), and The

Genome Institute at Washington University (TGI). This work was funded by

grants from the Department of Defense (NF120032 to D.H.G.) and the NIH

(R01-NS065547-01 to D.H.G.). Y.-H.C. is a recipient of the fellowship

from the American Brain Tumor Association supported by the Emily Dorfman

Foundation for Children in memory of Emily Ann Dorfman.

CR Bajenaru ML, 2003, CANCER RES, V63, P8573

Bao SD, 2006, NATURE, V444, P756, DOI 10.1038/nature05236

Beier D, 2011, MOL CANCER, V10, DOI 10.1186/1476-4598-10-128

Bleau AM, 2009, CELL STEM CELL, V4, P226, DOI 10.1016/j.stem.2009.01.007

Bonnet D, 1997, NAT MED, V3, P730, DOI 10.1038/nm0797-730

Booth L, 2014, MOL CANCER THER, V13, P2384, DOI 10.1158/1535-7163.MCT-14-0172

Carrière A, 2008, CURR BIOL, V18, P1269, DOI 10.1016/j.cub.2008.07.078

Chen J, 2012, NATURE, V488, P522, DOI 10.1038/nature11287

Cho HY, 2014, MOL CANCER THER, V13, P2004, DOI 10.1158/1535-7163.MCT-13-0964

Ciechomska IA, 2013, ONCOGENE, V32, P1518, DOI 10.1038/onc.2012.174

Daginakatte GC, 2007, HUM MOL GENET, V16, P1098, DOI 10.1093/hmg/ddm059

Dasgupta B, 2005, J NEUROSCI, V25, P5584, DOI 10.1523/JNEUROSCI.4693-04.2005

Erbay E, 2009, NAT MED, V15, P1383, DOI 10.1038/nm.2067

Galli R, 2004, CANCER RES, V64, P7011, DOI 10.1158/0008-5472.CAN-04-1364

Golebiewska A, 2013, BRAIN, V136, P1462, DOI 10.1093/brain/awt025

Guillamo JS, 2003, BRAIN, V126, P152, DOI 10.1093/brain/awg016

Hale JS, 2014, STEM CELLS, V32, P1746, DOI 10.1002/stem.1716

Hegedus B, 2008, CANCER RES, V68, P1520, DOI 10.1158/0008-5472.CAN-07-5916

Hegedus B, 2009, J NEUROPATH EXP NEUR, V68, P542, DOI 10.1097/NEN.0b013e3181a3240b

Hope KJ, 2004, NAT IMMUNOL, V5, P738, DOI 10.1038/ni1080

Horiguchi S, 2004, J NEUROSCI RES, V75, P817, DOI 10.1002/jnr.20046

Johnson GG, 2014, NEURO-ONCOLOGY, V16, P1086, DOI 10.1093/neuonc/nou012

Kaul A, 2012, GENE DEV, V26, P2561, DOI 10.1101/gad.200907.112

Kennedy MA, 2005, CELL METAB, V1, P121, DOI 10.1016/j.cmet.2005.01.002

Kim CFB, 2005, CELL, V121, P823, DOI 10.1016/j.cell.2005.03.032

Kim KY, 2010, NEUROSCIENCE, V170, P178, DOI 10.1016/j.neuroscience.2010.06.017

Kim YT, 2006, EXP NEUROL, V199, P222, DOI 10.1016/j.expneurol.2006.03.015

Klucken J, 2000, P NATL ACAD SCI USA, V97, P817, DOI 10.1073/pnas.97.2.817

Kwiatkowski DJ, 2002, HUM MOL GENET, V11, P525, DOI 10.1093/hmg/11.5.525

Lathia JD, 2014, CELL REP, V6, P117, DOI 10.1016/j.celrep.2013.11.043

Lathia JD, 2010, CELL STEM CELL, V6, P421, DOI 10.1016/j.stem.2010.02.018

Lee DY, 2012, CANCER CELL, V22, P131, DOI 10.1016/j.ccr.2012.05.036

Lee DY, 2010, GENE DEV, V24, P2317, DOI 10.1101/gad.1957110

Li JZ, 2006, CURR MOL MED, V6, P45, DOI 10.2174/156652406775574523

Lito P, 2014, CANCER CELL, V25, P697, DOI 10.1016/j.ccr.2014.03.011

Liu GT, 2006, MOL CANCER, V5, DOI 10.1186/1476-4598-5-67

Liu H, 1997, J BIOL CHEM, V272, P21751, DOI 10.1074/jbc.272.35.21751

Liu WT, 2013, NEURO-ONCOLOGY, V15, P1127, DOI 10.1093/neuonc/not073

Louis DN, 2007, ACTA NEUROPATHOL, V114, P547, DOI 10.1007/s00401-007-0278-6

Luo B, 2013, ONCOGENE, V32, P805, DOI 10.1038/onc.2012.130

Ma L, 2005, CELL, V121, P179, DOI 10.1016/j.cell.2005.02.031

Mahoney DH, 2000, NEURO-ONCOLOGY, V2, P213, DOI 10.1093/neuonc/2.4.213

Manning BD, 2002, MOL CELL, V10, P151, DOI 10.1016/S1097-2765(02)00568-3

Nakata S, 2013, BRAIN PATHOL, V23, P60, DOI 10.1111/j.1750-3639.2012.00618.x

Özcan U, 2006, SCIENCE, V313, P1137, DOI 10.1126/science.1128294

Pace A, 2003, ANN ONCOL, V14, P1722, DOI 10.1093/annonc/mdg502

PACKER RJ, 1993, J CLIN ONCOL, V11, P850, DOI 10.1200/JCO.1993.11.5.850

Packer RJ, 1997, J NEUROSURG, V86, P747, DOI 10.3171/jns.1997.86.5.0747

Parry PV, 2014, NEUROSURGERY, V74, pN14, DOI 10.1227/01.neu.0000442976.61335.f6

Pietras A, 2014, CELL STEM CELL, V14, P357, DOI 10.1016/j.stem.2014.01.005

Pong WW, 2013, ANN NEUROL, V73, P303, DOI 10.1002/ana.23813

Raabe EH, 2011, CLIN CANCER RES, V17, P3590, DOI 10.1158/1078-0432.CCR-10-3349

Ricci-Vitiani L, 2007, NATURE, V445, P111, DOI 10.1038/nature05384

Sakariassen PO, 2007, NEOPLASIA, V9, P882, DOI 10.1593/neo.07658

See WL, 2012, CANCER RES, V72, P3350, DOI 10.1158/0008-5472.CAN-12-0334

Singh SK, 2003, CANCER RES, V63, P5821

Singh SK, 2004, NATURE, V432, P396, DOI 10.1038/nature03128

Son MJ, 2009, CELL STEM CELL, V4, P440, DOI 10.1016/j.stem.2009.03.003

Suzuki K, 2013, NEURO-ONCOLOGY, V15, P1186, DOI 10.1093/neuonc/not062

Tarling EJ, 2011, P NATL ACAD SCI USA, V108, P19719, DOI 10.1073/pnas.1113021108

Tarling EJ, 2010, ARTERIOSCL THROM VAS, V30, P1174, DOI 10.1161/ATVBAHA.110.205617

Taylor MD, 2005, CANCER CELL, V8, P323, DOI 10.1016/j.ccr.2005.09.001

Tchoghandjian A, 2010, BRAIN PATHOL, V20, P211, DOI 10.1111/j.1750-3639.2009.00269.x

Tchoghandjian A, 2009, BRAIN, V132, P1523, DOI 10.1093/brain/awp048

Vermeulen L, 2008, P NATL ACAD SCI USA, V105, P13427, DOI 10.1073/pnas.0805706105

Wang DL, 2014, ONCOL REP, V31, P41, DOI 10.3892/or.2013.2826

Wang N, 2004, P NATL ACAD SCI USA, V101, P9774, DOI 10.1073/pnas.0403506101

Wojcik AJ, 2008, J IMMUNOL, V180, P4273, DOI 10.4049/jimmunol.180.6.4273

Xue JH, 2013, J PHYSIOL SCI, V63, P435, DOI 10.1007/s12576-013-0281-8

Yeh TH, 2009, GLIA, V57, P1239, DOI 10.1002/glia.20845

Yvan-Charvet L, 2007, J CLIN INVEST, V117, P3900, DOI 10.1172/JCI33372

Yvan-Charvet L, 2010, CIRC RES, V106, P1861, DOI 10.1161/CIRCRESAHA.110.217281

Zinszner H, 1998, GENE DEV, V12, P982, DOI 10.1101/gad.12.7.982

NR 73

TC 41

Z9 43

U1 0

U2 12

PU CELL PRESS

PI CAMBRIDGE

PA 50 HAMPSHIRE ST, FLOOR 5, CAMBRIDGE, MA 02139 USA

SN 2211-1247

J9 CELL REP

JI Cell Reports

PD MAR 24

PY 2015

VL 10

IS 11

BP 1899

EP 1912

DI 10.1016/j.celrep.2015.02.041

PG 14

WC Cell Biology

WE Science Citation Index Expanded (SCI-EXPANDED)

SC Cell Biology

GA CE4CY

UT WOS:000351779600011

PM 25772366

OA Green Published, Green Accepted, gold

DA 2025-04-09

ER

PT J

AU Berthier, S

Larrouquère, L

Champelovier, P

Col, E

Lefebvre, C

Cottet-Rouselle, C

Arnaud, J

Garrel, C

Laporte, F

Boutonnat, J

Faure, P

Hazane-Puch, F

AF Berthier, Sylvie

Larrouquere, Louis

Champelovier, Pierre

Col, Edwige

Lefebvre, Christine

Cottet-Rouselle, Cecile

Arnaud, Josiane

Garrel, Catherine

Laporte, Francois

Boutonnat, Jean

Faure, Patrice

Hazane-Puch, Florence

TI A New Patient-Derived Metastatic Glioblastoma Cell Line:

Characterisation and Response to Sodium Selenite Anticancer Agent

SO CANCERS

LA English

DT Article

DE Glioblastoma; cancer stem cells; new cell line; sodium selenite;

xenograft; cell death; epigenetics

ID BRAIN-TUMOR CELLS; CANCER STEM-CELL; HISTONE METHYLTRANSFERASE; CHEMICAL

FORM; TEMOZOLOMIDE; RESISTANCE; EXPRESSION; SUPPLEMENTATION;

IDENTIFICATION; RADIOTHERAPY

AB Glioblastoma multiform (GBM) tumors are very heterogeneous, organized in a hierarchical pattern, including cancer stem cells (CSC), and are responsible for development, maintenance, and cancer relapse. Therefore, it is relevant to establish new GBM cell lines with CSC characteristics to develop new treatments. A new human GBM cell line, named R2J, was established from the cerebro-spinal fluid (CSF) of a patient affected by GBM with leptomeningeal metastasis. R2J cells exhibits an abnormal karyotype and form self-renewable spheres in a serum-free medium. Original tumor, R2J, cultured in monolayer (2D) and in spheres showed a persistence expression of CD44, CD56 (except in monolayer), EGFR, Ki67, Nestin, and vimentin. The R2J cell line is tumorigenic and possesses CSC properties. We tested in vitro the anticancer effects of sodium selenite (SS) compared to temozolomide TMZ. SS was absorbed by R2J cells, was cytotoxic, induced an oxidative stress, and arrested cell growth in G2M before inducing both necrosis and apoptosis via caspase-3. SS also modified dimethyl-histone-3-lysine-9 (H3K9m2) levels and decreased histone deacetylase (HDAC) activity, suggesting anti-invasiveness potential. This study highlights the value of this new GBM cell line for preclinical modeling of clinically relevant, patient specific GBM and opens a therapeutic window to test SS to target resistant and recurrent GBM.

C1 [Berthier, Sylvie; Champelovier, Pierre] Grenoble Alpes Hosp, Inst Biol & Pathol, Cytometry Platform, CS10217, Grenoble 9, France.

[Larrouquere, Louis] INSERM U1205, BrainTech Lab, F-38000 Grenoble, France.

[Larrouquere, Louis] Grenoble Alpes Hosp, Med Oncol Dept, CS10217, Grenoble 9, France.

[Col, Edwige; Boutonnat, Jean] Grenoble Alpes Hosp, Inst Biol & Pathol, Unit Anatomopathol, CS10217, Grenoble 9, France.

[Lefebvre, Christine] Grenoble Alpes Hosp, Inst Biol & Pathol, Lab Hematol Oncogenet & Immunol, CS10217, Grenoble 9, France.

[Cottet-Rouselle, Cecile; Arnaud, Josiane] Univ Grenoble Alpes, Lab Fundamental & Appl Bioenerget LBFA, Inserm U1055, F-38000 Grenoble, France.

[Cottet-Rouselle, Cecile; Arnaud, Josiane] Univ Grenoble Alpes, Inserm U1055, SFR BEeSy, F-38000 Grenoble, France.

[Arnaud, Josiane; Garrel, Catherine; Laporte, Francois; Faure, Patrice; Hazane-Puch, Florence] Grenoble Alpes Hosp, Inst Biol & Pathol, Unit Nutr & Hormonal Biochem, CS10217, F-38043 Grenoble 9, France.

[Faure, Patrice] Univ Grenoble Alpes, Hypoxia Physiopathol Lab HP2, Inserm U1042, F-38000 Grenoble, France.

C3 Institut National de la Sante et de la Recherche Medicale (Inserm);

Institut National de la Sante et de la Recherche Medicale (Inserm);

Communaute Universite Grenoble Alpes; Universite Grenoble Alpes (UGA);

Communaute Universite Grenoble Alpes; Universite Grenoble Alpes (UGA);

Institut National de la Sante et de la Recherche Medicale (Inserm);

Communaute Universite Grenoble Alpes; Universite Grenoble Alpes (UGA);

Institut National de la Sante et de la Recherche Medicale (Inserm)

RP Hazane-Puch, F (corresponding author), Grenoble Alpes Hosp, Inst Biol & Pathol, Unit Nutr & Hormonal Biochem, CS10217, F-38043 Grenoble 9, France.

EM SBerthier@chu-grenoble.fr; LLarrouquere@chu-grenoble.fr;

pierre.champelovier@wanadoo.fr; ECol@chu-grenoble.fr;

CLefebvre@chu-grenoble.fr; cecile.cottet@univ-grenoble-alpes.fr;

JArnaud@chu-grenoble.fr; CGarrel@chu-grenoble.fr;

francois.laporte@cegetel.net; JBoutonnat@chu-grenoble.fr;

PFaure@chu-grenoble.fr; FPuch@chu-grenoble.fr

RI Lefebvre, Christine/G-7280-2014

OI PUCH, Florence/0000-0003-2770-289X; arnaud, josiane/0000-0001-9101-3330;

Larrouquere, Louis/0000-0002-5287-6712; Laporte,

francois/0000-0002-8194-8973; COTTET-ROUSSELLE,

Cecile/0000-0003-1208-8911

FU Direction de la Recherche Clinique (DRC) of the Centre Hospitalier

Universitaire Grenoble Alpes (CHUGA, France); Groupement des Entreprises

Francaises de Lutte contre le Cancer (GEFLUC)

FX This research was funded by grants from the Direction de la Recherche

Clinique (DRC) of the Centre Hospitalier Universitaire Grenoble Alpes

(CHUGA, France) and by the Groupement des Entreprises Francaises de

Lutte contre le Cancer (GEFLUC).

CR Beier D, 2011, MOL CANCER, V10, DOI 10.1186/1476-4598-10-128

Berthier S, 2017, J TRACE ELEM MED BIO, V44, P161, DOI 10.1016/j.jtemb.2017.04.012

Bonomi Serena, 2013, Int J Cell Biol, V2013, P962038, DOI 10.1155/2013/962038

CAVALIERI RR, 1966, J NUCL MED, V7, P197

Ceccarelli M, 2016, CELL, V164, P550, DOI 10.1016/j.cell.2015.12.028

Chen J, 2012, NATURE, V488, P522, DOI 10.1038/nature11287

Chen MW, 2010, CANCER RES, V70, P7830, DOI 10.1158/0008-5472.CAN-10-0833

Choi SA, 2012, CANCER LETT, V324, P221, DOI 10.1016/j.canlet.2012.05.026

Ciechomska IA, 2016, SCI REP-UK, V6, DOI 10.1038/srep38723

Coles-Takabe BLK, 2008, STEM CELLS, V26, P2938, DOI 10.1634/stemcells.2008-0558

de Narvajas AAM, 2013, MOL CELL BIOL, V33, P3983, DOI 10.1128/MCB.00813-13

Giulietti A, 2001, METHODS, V25, P386, DOI 10.1006/meth.2001.1261

Harabin-Slowinska M, 1998, FOLIA NEUROPATHOL, V36, P179

Hazane-Puch F., 2017, UNIT NUTR HORM UNPUB

Hazane-Puch F, 2016, ANTI-CANCER AGENT ME, V16, P490, DOI 10.2174/1871520615666150819095426

Hazane-Puch F, 2014, METALLOMICS, V6, P1683, DOI 10.1039/c4mt00040d

Hazane-Puch F, 2013, BIOL TRACE ELEM RES, V154, P288, DOI 10.1007/s12011-013-9709-5

Hong X, 2012, INT J ONCOL, V41, P1693, DOI 10.3892/ijo.2012.1592

Iacopino F, 2014, PLOS ONE, V9, DOI 10.1371/journal.pone.0105166

Jhaveri N, 2016, CANCER LETT, V371, P240, DOI 10.1016/j.canlet.2015.11.040

Jin X, 2013, BIOCHEM BIOPH RES CO, V433, P496, DOI 10.1016/j.bbrc.2013.03.021

Kim EH, 2007, CANCER RES, V67, P6314, DOI 10.1158/0008-5472.CAN-06-4217

Kong DS, 2009, CANCER-AM CANCER SOC, V115, P140, DOI 10.1002/cncr.23972

Lee SY, 2016, GENES DIS, V3, P198, DOI 10.1016/j.gendis.2016.04.007

Loja T, 2009, ONCOL REP, V21, P119, DOI 10.3892/or_00000198

Louis DN, 2016, ACTA NEUROPATHOL, V131, P803, DOI 10.1007/s00401-016-1545-1

Lunoe K, 2011, METALLOMICS, V3, P162, DOI 10.1039/c0mt00091d

Mahabir R, 2014, NEURO-ONCOLOGY, V16, P671, DOI 10.1093/neuonc/not239

Mao X, 2000, CANCER GENET CYTOGEN, V122, P87, DOI 10.1016/S0165-4608(00)00278-8

May CD, 2011, BREAST CANCER RES, V13, DOI 10.1186/bcr2789

Mimeault M, 2014, CANCER EPIDEM BIOMAR, V23, P234, DOI 10.1158/1055-9965.EPI-13-0785

Okamoto R, 2002, JPN J CANCER RES, V93, P93, DOI 10.1111/j.1349-7006.2002.tb01205.x

Olm E, 2009, P NATL ACAD SCI USA, V106, P11400, DOI 10.1073/pnas.0902204106

Onda K, 1999, J NEURO-ONCOL, V41, P247, DOI 10.1023/A:1006172608019

Park CK, 2013, J NEURO-ONCOL, V112, P277, DOI 10.1007/s11060-013-1060-3

Pollard SM, 2009, CELL STEM CELL, V4, P568, DOI 10.1016/j.stem.2009.03.014

Rooprai HK, 2007, INT J ONCOL, V30, P1263

SEHESTED J, 1974, HUMANGENETIK, V21, P55, DOI 10.1007/BF00278565

Seligson DB, 2009, AM J PATHOL, V174, P1619, DOI 10.2353/ajpath.2009.080874

Singh A, 2010, ONCOGENE, V29, P4741, DOI 10.1038/onc.2010.215

Singh SK, 2003, CANCER RES, V63, P5821

Singh SK, 2004, NATURE, V432, P396, DOI 10.1038/nature03128

Stupp R, 2009, LANCET ONCOL, V10, P459, DOI 10.1016/S1470-2045(09)70025-7

THERMAN E, 1984, CANCER GENET CYTOGEN, V11, P185, DOI 10.1016/0165-4608(84)90113-4

Thiery JP, 2006, NAT REV MOL CELL BIO, V7, P131, DOI 10.1038/nrm1835

Verhaak RGW, 2010, CANCER CELL, V17, P98, DOI 10.1016/j.ccr.2009.12.020

Vermeulen K, 2003, CELL PROLIFERAT, V36, P131, DOI 10.1046/j.1365-2184.2003.00266.x

Vivanco I, 2002, NAT REV CANCER, V2, P489, DOI 10.1038/nrc839

Weekley CM, 2011, J AM CHEM SOC, V133, P18272, DOI 10.1021/ja206203c

WESTPHAL M, 1994, ACTA NEUROCHIR, V126, P17, DOI 10.1007/BF01476489

Wilting RH, 2012, DRUG RESIST UPDATE, V15, P21, DOI 10.1016/j.drup.2012.01.008

Yin D, 2007, CLIN CANCER RES, V13, P1045, DOI 10.1158/1078-0432.CCR-06-1261

Yu SC, 2008, CANCER LETT, V265, P124, DOI 10.1016/j.canlet.2008.02.010

Yu ZR, 2012, INT J BIOCHEM CELL B, V44, P2144, DOI 10.1016/j.biocel.2012.08.022

Zang LL, 2018, FRONT MOL NEUROSCI, V11, DOI 10.3389/fnmol.2018.00408

ZHANG ZH, 1995, BIOL TRACE ELEM RES, V48, P45, DOI 10.1007/BF02789078

NR 56

TC 8

Z9 8

U1 0

U2 4

PU MDPI

PI BASEL

PA ST ALBAN-ANLAGE 66, CH-4052 BASEL, SWITZERLAND

EI 2072-6694

J9 CANCERS

JI Cancers

PD JAN

PY 2019

VL 11

IS 1

AR 12

DI 10.3390/cancers11010012

PG 28

WC Oncology

WE Science Citation Index Expanded (SCI-EXPANDED)

SC Oncology

GA HJ5RS

UT WOS:000457240200013

PM 30583471

OA Green Published, gold, Green Submitted

DA 2025-04-09

ER

PT J

AU Dixit, D

Ghildiyal, R

Anto, NP

Sen, E

AF Dixit, D.

Ghildiyal, R.

Anto, N. P.

Sen, E.

TI Chaetocin-induced ROS-mediated apoptosis involves ATM-YAP1 axis and

JNK-dependent inhibition of glucose metabolism

SO CELL DEATH & DISEASE

LA English

DT Article

DE Glioblastoma; ROS; ATM; YAP; JNK; metabolism

ID YES-ASSOCIATED PROTEIN; KAPPA-B AXIS; GLIOMA-CELLS; DNA-DAMAGE;

GLIOBLASTOMA CELLS; UP-REGULATION; ACTIVATION; CANCER; GROWTH; YAP

AB Oxidative stress serves as an important regulator of both apoptosis and metabolic reprogramming in tumor cells. Chaetocin, a histone methyltransferase inhibitor, is known to induce ROS generation. As elevating basal ROS level sensitizes glioma cells to apoptosis, the ability of Chaetocin in regulating apoptotic and metabolic adaptive responses in glioma was investigated. Chaetocin induced glioma cell apoptosis in a ROS-dependent manner. Increased intracellular ROS induced (i) Yes-associated protein 1 (YAP1) expression independent of the canonical Hippo pathway as well as (ii) ATM and JNK activation. Increased interaction of YAP1 with p73 and p300 induced apoptosis in an ATM-dependent manner. Chaetocin induced JNK modulated several metabolic parameters like glucose uptake, lactate production, ATP generation, and activity of glycolytic enzymes hexokinase and pyruvate kinase. However, JNK had no effect on ATM or YAP1 expression. Coherent with the in vitro findings, Chaetocin reduced tumor burden in heterotypic xenograft glioma mouse model. Chaetocin-treated tumors exhibited heightened ROS, pATM, YAP1 and pJNK levels. Our study highlights the coordinated control of glioma cell proliferation and metabolism by ROS through (i) ATM-YAP1-driven apoptotic pathway and (ii) JNK-regulated metabolic adaptation. The elucidation of these newfound connections and the roles played by ROS to simultaneously shift metabolic program and induce apoptosis could provide insights toward the development of new anti-glioma strategies.

C1 [Dixit, D.; Ghildiyal, R.; Anto, N. P.; Sen, E.] Natl Brain Res Ctr, Cellular & Mol Neurosci Div, Gurgaon 122051, Haryana, India.

C3 Department of Biotechnology (DBT) India; National Brain Research Centre

(NBRC)

RP Dixit, D (corresponding author), Natl Brain Res Ctr, Cellular & Mol Neurosci Div, NH-8, Gurgaon 122051, Haryana, India.

EM dev25@nbrc.ac.in; ellora@nbrc.ac.in

OI Anto, Nikhil/0000-0001-9449-5654; Dixit, Deobrat/0000-0003-4459-3904;

Ghildiyal, Ruchi/0000-0001-5867-0072; Sen, Ellora/0000-0001-6842-7850

FU Department of Biotechnology (DBT, Government of India)

[BT/PR5818/Med/30/839/2012]; Council of Scientific and Industrial

Research (CSIR, Government of India)

FX The work was supported by a research grant from the Department of

Biotechnology (DBT, Government of India No. BT/PR5818/Med/30/839/2012)

to ES. DD is supported by a research fellowship from Council of

Scientific and Industrial Research (CSIR, Government of India). We

acknowledge Shanker Dutt Joshi for technical assistance with the animal

experiments. We acknowledge Dr. Inderjeet Yadav for his help and support

with animal facility.

CR Anastasiou D, 2011, SCIENCE, V334, P1278, DOI 10.1126/science.1211485

Basu S, 2003, MOL CELL, V11, P11, DOI 10.1016/S1097-2765(02)00776-1

Burma S, 2001, J BIOL CHEM, V276, P42462, DOI 10.1074/jbc.c100466200

Cairns RA, 2011, NAT REV CANCER, V11, P85, DOI 10.1038/nrc2981

Chaib H, 2012, LEUKEMIA, V26, P662, DOI 10.1038/leu.2011.271

Chambers JW, 2011, J BIOL CHEM, V286, DOI 10.1074/jbc.M111.223602

Cherblanc FL, 2013, NAT CHEM BIOL, V9, P136, DOI 10.1038/nchembio.1187

Chervona Y, 2012, FREE RADICAL BIO MED, V53, P1041, DOI 10.1016/j.freeradbiomed.2012.07.020

Chiu WH, 2012, BIOCHEM PHARMACOL, V83, P1159, DOI 10.1016/j.bcp.2012.01.016

Cosentino C, 2011, EMBO J, V30, P546, DOI 10.1038/emboj.2010.330

Dixit D, 2012, CELL DEATH DIS, V3, DOI 10.1038/cddis.2012.10

Dixit D, 2013, CANCER LETT, V336, P347, DOI 10.1016/j.canlet.2013.03.025

Dixit D, 2009, FREE RADICAL BIO MED, V47, P364, DOI 10.1016/j.freeradbiomed.2009.04.031

Fausti F, 2013, CELL DEATH DIFFER, V20, P1498, DOI 10.1038/cdd.2013.101

Greiner D, 2005, NAT CHEM BIOL, V1, P143, DOI 10.1038/nchembio721

Grüning NM, 2011, CELL METAB, V14, P415, DOI 10.1016/j.cmet.2011.06.017

Gupta P, 2013, CARCINOGENESIS, V34, P388, DOI 10.1093/carcin/bgs352

Hanahan D, 2011, CELL, V144, P646, DOI 10.1016/j.cell.2011.02.013

Hsu PP, 2008, CELL, V134, P703, DOI 10.1016/j.cell.2008.08.021

Isham CR, 2012, BRIT J CANCER, V106, P314, DOI 10.1038/bjc.2011.522

Isham CR, 2007, BLOOD, V109, P2579, DOI 10.1182/blood-2006-07-027326

Kang MA, 2012, CELL DEATH DIS, V3, DOI 10.1038/cddis.2011.134

Lai JM, 2009, EUR J PHARMACOL, V623, P1, DOI 10.1016/j.ejphar.2009.08.031

Lemire J, 2008, PLOS ONE, V3, DOI 10.1371/journal.pone.0001550

Levy D, 2008, MOL CELL, V29, P350, DOI 10.1016/j.molcel.2007.12.022

Mukherjee J, 2013, PLOS ONE, V8, DOI 10.1371/journal.pone.0057610

Okazaki T, 2012, CANCER LETT, V323, P199, DOI 10.1016/j.canlet.2012.04.013

Orr BA, 2011, J NEUROPATH EXP NEUR, V70, P568, DOI 10.1097/NEN.0b013e31821ff8d8

Pastorino JG, 2002, J BIOL CHEM, V277, P7610, DOI 10.1074/jbc.M109950200

Rundlöf AK, 2004, ANTIOXID REDOX SIGN, V6, P41, DOI 10.1089/152308604771978336

Schneider JG, 2006, CELL METAB, V4, P377, DOI 10.1016/j.cmet.2006.10.002

Sharma V, 2007, MOL CANCER THER, V6, P2544, DOI 10.1158/1535-7163.MCT-06-0788

Sinha S, 2013, CELL DEATH DIS, V4, DOI 10.1038/cddis.2013.128

Strano S, 2005, MOL CELL, V18, P447, DOI 10.1016/j.molcel.2005.04.008

Swa HLF, 2012, MOL CELL PROTEOMICS, V11, P381, DOI 10.1074/mcp.M111.011205

Tewari R, 2012, J MOL MED, V90, P67, DOI 10.1007/s00109-011-0807-6

Tibodeau JD, 2009, ANTIOXID REDOX SIGN, V11, P1097, DOI [10.1089/ars.2008.2318, 10.1089/ARS.2008.2318]

Tomlinson V, 2010, CELL DEATH DIS, V1, DOI 10.1038/cddis.2010.7

Tournier C, 2000, SCIENCE, V288, P870, DOI 10.1126/science.288.5467.870

Trachootham D, 2006, CANCER CELL, V10, P241, DOI 10.1016/j.ccr.2006.08.009

Venneti S, 2013, J NEUROPATH EXP NEUR, V72, P298, DOI 10.1097/NEN.0b013e3182898113

Wang W, GENES DEV, V26, P1959

Wang ZQ, 2009, J CELL PHYSIOL, V221, P213, DOI 10.1002/jcp.21844

Wolf A, 2010, ONCOTARGET, V1, P552, DOI 10.18632/oncotarget.190

Wolf A, 2011, J EXP MED, V208, P313, DOI 10.1084/jem.20101470

Wu RX, 2012, EMBO MOL MED, V4, P633, DOI 10.1002/emmm.201200240

Yang WW, 2011, NATURE, V480, P118, DOI 10.1038/nature10598

Zagurovskaya M, 2009, ONCOGENE, V28, P1121, DOI 10.1038/onc.2008.461

Zhao B, 2007, GENE DEV, V21, P2747, DOI 10.1101/gad.1602907

Zhao F, 2010, ONCOGENE, V29, P2962, DOI 10.1038/onc.2010.67

Zhao YH, 2011, CANCER RES, V71, P4585, DOI 10.1158/0008-5472.CAN-11-0127

Zhou YF, 2012, CANCER RES, V72, P304, DOI 10.1158/0008-5472.CAN-11-1674

NR 52

TC 87

Z9 93

U1 2

U2 34

PU NATURE PUBLISHING GROUP

PI LONDON

PA MACMILLAN BUILDING, 4 CRINAN ST, LONDON N1 9XW, ENGLAND

SN 2041-4889

J9 CELL DEATH DIS

JI Cell Death Dis.

PD MAY

PY 2014

VL 5

SI SI

AR e1212

DI 10.1038/cddis.2014.179

PG 13

WC Cell Biology

WE Science Citation Index Expanded (SCI-EXPANDED)

SC Cell Biology

GA AI9DN

UT WOS:000337229300012

PM 24810048

OA Green Published, gold

DA 2025-04-09

ER

PT J

AU Williams, M

Tietzel, I

Quick, QA

AF Williams, Musa

Tietzel, Illya

Quick, Quincy A.

TI 1′-Acetoxychavicol acetate promotes caspase 3-activated glioblastoma

cell death by overcoming enhanced cytokine expression

SO ONCOLOGY LETTERS

LA English

DT Article

DE glioblastomas; acetoxychavichol acetate; caspase 3; cytokines

ID XANTHINE-OXIDASE INHIBITOR; HUMAN BRAIN-TUMORS; 1-ACETOXYCHAVICOL

ACETATE; GLUTATHIONE-PEROXIDASE; OXIDATIVE STRESS; APOPTOSIS; CANCER;

GROWTH; CARCINOGENESIS; INTERLEUKIN-6

AB The brain consumes similar to 20% of the oxygen utilized in the human body, meaning that brain tumors are vulnerable to paradoxical physiological effects from free radical generation. In the present study, 1'-acetoxychavicol acetate (ACA), a naturally derived antioxidant that inhibits xanthine oxidase, was evaluated for its role as an anti-tumorigenic agent in glioblastomas. The study revealed that ACA inhibited glioblastoma cell proliferation as a consequence of promoting apoptotic cell death by enhancing caspase 3 activity. It was also shown that ACA impaired the migratory ability of glioblastoma cells by decreasing their adhesive properties. Additionally, ACA increased the protein expression levels of the pro-survival signaling cytokines, IL-6 and IL-1 alpha, established cell protectors and survival molecules in brain tumors. Together, these results demonstrate that, despite enhanced expression of compensatory signaling molecules that contribute to tumor cell survival, ACA is an effective pro-apoptotic inducing agent in glioblastomas.

C1 [Williams, Musa; Tietzel, Illya; Quick, Quincy A.] Southern Univ New Orleans, Dept Biol, New Orleans, LA 70126 USA.

C3 Southern University System; Southern University New Orleans

RP Quick, QA (corresponding author), Southern Univ New Orleans, Dept Biol, 6400 Press Dr, New Orleans, LA 70126 USA.

EM qquick@suno.edu

OI Tietzel, Illya/0000-0003-0966-4498

FU Louisiana Board of Regents [LEQSF 2012-13-ENH-UG-32]

FX This study was supported by a grant from the Louisiana Board of Regents

(LEQSF 2012-13-ENH-UG-32). The ACA was kindly provided by Dr Heather

Kleiner of the Department of Pharmacology, LSU Health Sciences Center

(Shreveport, LA, USA).

CR Acharya A, 2010, OXID MED CELL LONGEV, V3, P23, DOI 10.4161/oxim.3.1.10095

Atukeren P, 2010, NEUROL RES, V32, P492, DOI 10.1179/174313209X459075

Bissonnette CJ, 2004, NEUROSCI LETT, V361, P40, DOI 10.1016/j.neulet.2004.01.005

Cabello CM, 2007, CURR OPIN INVEST DR, V8, P1022

Campbell CT, 2007, TOXICOL LETT, V173, P151, DOI 10.1016/j.toxlet.2007.07.008

Dokic I, 2012, GLIA, V60, P1785, DOI 10.1002/glia.22397

Fang J, 2009, ADV DRUG DELIVER REV, V61, P290, DOI 10.1016/j.addr.2009.02.005

Higashida M, 2009, AMINO ACIDS, V36, P107, DOI 10.1007/s00726-008-0038-5

Ichikawa H, 2005, J IMMUNOL, V174, P7383, DOI 10.4049/jimmunol.174.11.7383

In LLA, 2012, BMC COMPLEM ALTERN M, V12, DOI 10.1186/1472-6882-12-179

Ito K, 2005, BIOCHEM BIOPH RES CO, V338, P1702, DOI 10.1016/j.bbrc.2005.10.153

Ito K, 2005, CANCER RES, V65, P4417, DOI 10.1158/0008-5472.CAN-05-0072

Ito K, 2004, CLIN CANCER RES, V10, P2120, DOI 10.1158/1078-0432.CCR-1142-03

KOKOGLU E, 1990, CANCER LETT, V50, P179, DOI 10.1016/0304-3835(90)90262-V

Li RH, 2010, ONCOL REP, V23, P1553, DOI 10.3892/or_00000795

Liu QL, 2010, J NEURO-ONCOL, V100, P165, DOI 10.1007/s11060-010-0158-0

Michaud-Levesque J, 2012, EXP CELL RES, V318, P925, DOI 10.1016/j.yexcr.2012.02.017

Moffatt J, 2000, CARCINOGENESIS, V21, P2151, DOI 10.1093/carcin/21.12.2151

Montero AJ, 2011, DRUGS, V71, P1385, DOI 10.2165/11592590-000000000-00000

Muangnoi P, 2007, PLANTA MED, V73, P748, DOI 10.1055/s-2007-981542

Naik E, 2011, J EXP MED, V208, P417, DOI 10.1084/jem.20110367

Ohnishi M, 1996, JPN J CANCER RES, V87, P349, DOI 10.1111/j.1349-7006.1996.tb00229.x

Pacher P, 2006, PHARMACOL REV, V58, P87, DOI 10.1124/pr.58.1.6

Pu PY, 1996, J NEURO-ONCOL, V29, P121

Saidi A, 2009, INT J CANCER, V125, P1054, DOI 10.1002/ijc.24380

Tanaka T, 1997, JPN J CANCER RES, V88, P821, DOI 10.1111/j.1349-7006.1997.tb00457.x

Tanriverdi T, 2007, J CANCER RES CLIN, V133, P627, DOI 10.1007/s00432-007-0212-2

Valko M, 2006, CHEM-BIOL INTERACT, V160, P1, DOI 10.1016/j.cbi.2005.12.009

Wang J, 2008, CANCER BIOL THER, V7, P1875, DOI 10.4161/cbt.7.12.7067

Wu WS, 2008, CANCER METAST REV, V27, P303, DOI 10.1007/s10555-008-9112-4

Wu WS, 2006, CANCER METAST REV, V25, P695, DOI 10.1007/s10555-006-9037-8

NR 31

TC 6

Z9 9

U1 2

U2 10

PU SPANDIDOS PUBL LTD

PI ATHENS

PA POB 18179, ATHENS, 116 10, GREECE

SN 1792-1074

EI 1792-1082

J9 ONCOL LETT

JI Oncol. Lett.

PD JUN

PY 2013

VL 5

IS 6

BP 1968

EP 1972

DI 10.3892/ol.2013.1292

PG 5

WC Oncology

WE Science Citation Index Expanded (SCI-EXPANDED)

SC Oncology

GA 223OT

UT WOS:000324816500042

PM 23833677

OA Green Published, gold, Green Submitted

DA 2025-04-09

ER

PT J

AU Ranjit, M

Hirano, M

Aoki, K

Okuno, Y

Ohka, F

Yamamichi, A

Kato, A

Maeda, S

Motomura, K

Matsuo, K

Enomoto, A

Ino, Y

Todo, T

Takahashi, M

Wakabayashi, T

Kato, T

Natsume, A

AF Ranjit, Melissa

Hirano, Masaki

Aoki, Kosuke

Okuno, Yusuke

Ohka, Fumiharu

Yamamichi, Akane

Kato, Akira

Maeda, Sachi

Motomura, Kazuya

Matsuo, Keitaro

Enomoto, Atsushi

Ino, Yasushi

Todo, Tomoki

Takahashi, Masahide

Wakabayashi, Toshihiko

Kato, Takuya

Natsume, Atsushi

TI Aberrant Active cis-Regulatory Elements Associated with

Downregulation of RET Finger Protein Overcome Chemoresistance in

Glioblastoma

SO CELL REPORTS

LA English

DT Article

ID POLY(ADP-RIBOSE) POLYMERASE-1 INHIBITOR; ADJUVANT TEMOZOLOMIDE;

BINDING-PROTEIN; CANCER-CELLS; EXPRESSION; SENSITIVITY; GLIOMA;

METHYLTRANSFERASE; RADIOTHERAPY; CONCOMITANT

AB RET finger protein (RFP) forms a complex with histone deacetylase 1, resulting in aberrant deacetylation of H3K27ac and dysregulation of cis-regulatory elements. We evaluated the modulatory effects of RFP knockdown on cis-regulatory elements, gene expression, and chemosensitivity to temozolomide both in glioblastoma cells and in an intracranial glioblastoma model. The combination of RFP knockdown and temozolomide treatment markedly suppressed the glioblastoma cell growth due to oxidative stress and aberrant cell cycle and increased survival time in mice with glioblastoma. ChIP-seq and RNA-seq revealed that RFP knockdown increased or decreased activity of numerous cis-regulatory elements that lie adjacent to genes that control functions such as apoptosis, mitosis, DNA replication, and cell cycle: FOXO1, TBP2, and PARPBP. This study suggests that RFP contributes to chemoresistance via aberrant deacetylation of histone H3 at K27, whereas dysregulation of RFP-associated cis-regulatory elements in glioma and RFP knockdown combined with temozolomide is an effective treatment strategy for lethal glioma.

C1 [Ranjit, Melissa; Hirano, Masaki; Aoki, Kosuke; Ohka, Fumiharu; Yamamichi, Akane; Kato, Akira; Maeda, Sachi; Motomura, Kazuya; Wakabayashi, Toshihiko; Natsume, Atsushi] Nagoya Univ, Dept Neurosurg, Sch Med, Nagoya, Aichi, Japan.

[Okuno, Yusuke] Nagoya Univ Hosp, Ctr Adv Med & Clin Res, Nagoya, Aichi, Japan.

[Matsuo, Keitaro] Aichi Canc Ctr, Div Canc Epidemiol & Prevent, Res Inst, Nagoya, Aichi, Japan.

[Matsuo, Keitaro] Nagoya Univ, Dept Epidemiol, Sch Med, Nagoya, Aichi, Japan.

[Enomoto, Atsushi; Takahashi, Masahide] Nagoya Univ, Dept Pathol, Sch Med, Nagoya, Aichi, Japan.

[Ino, Yasushi; Todo, Tomoki] Univ Tokyo, Div Innovat Canc Therapy, Adv Clin Res Ctr, Inst Med Sci, Tokyo, Japan.

[Kato, Takuya] Kitasato Univ, Dept Pathol, Sch Med, Sagamihara, Kanagawa, Japan.

C3 Nagoya University; Nagoya University; Aichi Cancer Center; Nagoya

University; Nagoya University; University of Tokyo; Kitasato University

RP Natsume, A (corresponding author), Nagoya Univ, Dept Neurosurg, Sch Med, Nagoya, Aichi, Japan.; Kato, T (corresponding author), Kitasato Univ, Dept Pathol, Sch Med, Sagamihara, Kanagawa, Japan.

EM katot@med.kitasato-u.ac.jp; anatsume@med.nagoya-u.ac.jp

RI Ohka, Fumiharu/A-3420-2016; Motomura, Kazuya/AAF-8776-2021; Takahashi,

Masahide/AAN-4770-2020; Matsuo, Keitaro/H-6758-2019; Okuno,

Yusuke/C-9082-2009; Enomoto, Atsushi/I-7272-2014; Natsume,

Atsushi/I-7364-2014

OI Motomura, Kazuya/0000-0002-4376-1104; Enomoto,

Atsushi/0000-0002-9206-6116; Ohka, Fumiharu/0000-0002-5569-5626; Matsuo,

Keitaro/0000-0003-1761-6314; Natsume, Atsushi/0000-0002-9113-0470;

Takahashi, Masahide/0000-0002-2803-2683

FU JSPS KAKENHI [17928985]

FX Computations were partially performed on the NIG supercomputer at ROIS

National Institute of Genetics. This work was supported by a

Grant-in-Aid for Scientific Research on Innovative Areas "Chemistry for

Multimolecular Crowding Biosystems'' (JSPS KAKENHI Grant 17928985).

CR Blough MD, 2007, CANCER RES, V67, P580, DOI 10.1158/0008-5472.CAN-06-2782

Bobustuc GC, 2010, NEURO-ONCOLOGY, V12, P917, DOI 10.1093/neuonc/noq044

Cao TY, 1998, J CELL SCI, V111, P1319

Ceccarelli M, 2016, CELL, V164, P550, DOI 10.1016/j.cell.2015.12.028

Creyghton MP, 2010, P NATL ACAD SCI USA, V107, P21931, DOI 10.1073/pnas.1016071107

Curtin NJ, 2004, CLIN CANCER RES, V10, P881, DOI 10.1158/1078-0432.CCR-1144-3

Goldman M, 2013, NUCLEIC ACIDS RES, V41, pD949, DOI 10.1093/nar/gks1008

Hasegawa N, 1996, BIOCHEM BIOPH RES CO, V225, P627, DOI 10.1006/bbrc.1996.1221

Heinz S, 2015, NAT REV MOL CELL BIO, V16, P144, DOI 10.1038/nrm3949

Heinz S, 2010, MOL CELL, V38, P576, DOI 10.1016/j.molcel.2010.05.004

Hermisson M, 2006, J NEUROCHEM, V96, P766, DOI 10.1111/j.1471-4159.2005.03583.x

Horio M, 2012, CANCER MED-US, V1, P218, DOI 10.1002/cam4.32

Ikushima H, 2009, CELL STEM CELL, V5, P504, DOI 10.1016/j.stem.2009.08.018

ISOMURA T, 1992, NUCLEIC ACIDS RES, V20, P5305, DOI 10.1093/nar/20.20.5305

Iwakoshi A, 2012, PATHOL INT, V62, P324, DOI 10.1111/j.1440-1827.2012.02797.x

Kato T, 2009, CANCER RES, V69, P3597, DOI 10.1158/0008-5472.CAN-08-4368

Khan MM, 2001, MOL CELL, V7, P1233, DOI 10.1016/S1097-2765(01)00257-X

Kim D, 2013, GENOME BIOL, V14, DOI 10.1186/gb-2013-14-4-r36

Langmead B, 2012, NAT METHODS, V9, P357, DOI [10.1038/NMETH.1923, 10.1038/nmeth.1923]

Marks PA, 2003, CURR OPIN PHARMACOL, V3, P344, DOI 10.1016/S1471-4892(03)00084-5

Matsuura T, 2005, EXP CELL RES, V308, P65, DOI 10.1016/j.yexcr.2005.04.022

Mcnab FW, 2011, CURR OPIN IMMUNOL, V23, P46, DOI 10.1016/j.coi.2010.10.021

Mottet D, 2008, CLIN EXP METASTAS, V25, P183, DOI 10.1007/s10585-007-9131-5

Piao L, 2011, GENE CHROMOSOME CANC, V50, P13, DOI 10.1002/gcc.20828

Reymond A, 2001, EMBO J, V20, P2140, DOI 10.1093/emboj/20.9.2140

Shimono Y, 2000, J BIOL CHEM, V275, P39411, DOI 10.1074/jbc.M006585200

Stupp R, 2005, NEW ENGL J MED, V352, P987, DOI 10.1056/NEJMoa043330

Stupp R, 2009, LANCET ONCOL, V10, P459, DOI 10.1016/S1470-2045(09)70025-7

TAKAHASHI M, 1988, MOL CELL BIOL, V8, P1853, DOI 10.1128/MCB.8.4.1853

TAKAHASHI M, 1985, CELL, V42, P581, DOI 10.1016/0092-8674(85)90115-1

Tentori L, 2003, CLIN CANCER RES, V9, P5370

Tezel G, 1999, PATHOL INT, V49, P881, DOI 10.1046/j.1440-1827.1999.00957.x

Trapnell C, 2013, NAT BIOTECHNOL, V31, P46, DOI 10.1038/nbt.2450

Tsukamoto H, 2009, CANCER SCI, V100, P1895, DOI 10.1111/j.1349-7006.2009.01278.x

Yang Y, 2009, NEOPLASIA, V11, P313, DOI 10.1593/neo.81358

Yin ZX, 2018, ONCOTARGET, V9, P1885, DOI 10.18632/oncotarget.21277

Zhu JC, 2009, NAT METHODS, V6, P239, DOI 10.1038/nmeth0409-239

NR 37

TC 9

Z9 11

U1 0

U2 2

PU CELL PRESS

PI CAMBRIDGE

PA 50 HAMPSHIRE ST, FLOOR 5, CAMBRIDGE, MA 02139 USA

SN 2211-1247

J9 CELL REP

JI Cell Reports

PD FEB 26

PY 2019

VL 26

IS 9

BP 2274

EP +

DI 10.1016/j.celrep.2019.01.109

PG 13

WC Cell Biology

WE Science Citation Index Expanded (SCI-EXPANDED)

SC Cell Biology

GA HN6EV

UT WOS:000460279100003

PM 30811978

OA gold

DA 2025-04-09

ER

PT J

AU Sheikh, T

Gupta, P

Gowda, P

Patrick, S

Sen, E

AF Sheikh, Touseef

Gupta, Piyushi

Gowda, Pruthvi

Patrick, Shruti

Sen, Ellora

TI Hexokinase 2 and nuclear factor erythroid 2-related factor 2

transcriptionally coactivate xanthine oxidoreductase expression in

stressed glioma cells

SO JOURNAL OF BIOLOGICAL CHEMISTRY

LA English

DT Article

ID HYPOXIA-INDUCIBLE FACTOR; OXIDATIVE STRESS; CANCER-CELLS;

SACCHAROMYCES-CEREVISIAE; GLUCOSE-HOMEOSTASIS; NLRP3 INFLAMMASOME;

GLIOBLASTOMA CELLS; SIGNALING PATHWAY; ROS PRODUCTION; NRF2

AB A dynamic network of metabolic adaptations, inflammatory responses, and redox homeostasis is known to drive tumor progression. A considerable overlap among these processes exists, but several of their key regulators remain unknown. To this end, here we investigated the role of the proinflammatory cytokine IL-1 beta in connecting these processes in glioma cells. We found that glucose starvation sensitizes glioma cells to IL-1 beta-induced apoptosis in a manner that depended on reactive oxygen species (ROS). Although IL-1 beta-induced JNK had no effect on cell viability under glucose deprivation, it mediated nuclear translocation of hexokinase 2 (HK2). This event was accompanied by increases in the levels of sirtuin 6 (SIRT6), nuclear factor erythroid 2-related factor 2 (Nrf2), and xanthine oxidoreductase (XOR). SIRT6 not only induced ROS-mediated cell death but also facilitated nuclear Nrf2-HK2 interaction. Recruitment of the Nrf2-HK2 complex to the ARE site on XOR promoter regulated its expression. Importantly, HK2 served as transcriptional coactivator of Nrf2 to regulate XOR expression, indicated by decreased XOR levels in siRNA-mediated Nrf2 and HK2 knockdown experiments. Our results highlight a non-metabolic role of HK2 as transcriptional coactivator of Nrf2 to regulate XOR expression under conditions of proinflammatory and metabolic stresses. Our insights also underscore the importance of nuclear activities of HK2 in the regulation of genes involved in redox homeostasis.

C1 [Sheikh, Touseef; Gupta, Piyushi; Gowda, Pruthvi; Patrick, Shruti; Sen, Ellora] Natl Brain Res Ctr, Manesar 122051, Haryana, India.

C3 Department of Biotechnology (DBT) India; National Brain Research Centre

(NBRC)

RP Sen, E (corresponding author), Natl Brain Res Ctr, Manesar 122051, Haryana, India.

EM ellora@nbrc.ac.in

OI Patrick, Shruti/0000-0003-4007-6547; Sen, Ellora/0000-0001-6842-7850;

Gowda, Pruthvi/0000-0002-3928-2442; Sheikh, Dr Touseef

Ahmad/0000-0002-5608-8705

FU Department of Biotechnology (Government of India)

[BT/MED/30/SP11016/2015]

FX The work was supported by Department of Biotechnology (Government of

India) Research Grant BT/MED/30/SP11016/2015 (to E. S.). The authors

declare that they have no conflicts of interest with the contents of

this article.

CR Ahmad F, 2016, CELL DEATH DIS, V7, DOI 10.1038/cddis.2016.117

Ahuatzi D, 2004, J BIOL CHEM, V279, P14440, DOI 10.1074/jbc.M313431200

Bauer I, 2012, J BIOL CHEM, V287, P40924, DOI 10.1074/jbc.M112.405837

del Rey A, 2006, P NATL ACAD SCI USA, V103, P16039, DOI 10.1073/pnas.0607076103

Dixit D, 2014, CELL DEATH DIS, V5, DOI 10.1038/cddis.2014.179

Dixit D, 2009, FREE RADICAL BIO MED, V47, P364, DOI 10.1016/j.freeradbiomed.2009.04.031

Ghosh S, 2016, EXP CELL RES, V340, P116, DOI 10.1016/j.yexcr.2015.11.016

Ghosh S, 2013, MOL CELL BIOL, V33, P2718, DOI 10.1128/MCB.01254-12

Graham NA, 2012, MOL SYST BIOL, V8, DOI 10.1038/msb.2012.20

Griguer CE, 2006, CANCER RES, V66, P2257, DOI 10.1158/0008-5472.CAN-05-3364

Gupta P, 2017, EXP CELL RES, V357, P98, DOI 10.1016/j.yexcr.2017.05.005

Herrero P, 1998, FEBS LETT, V434, P71, DOI 10.1016/S0014-5793(98)00872-2

Huber HJ, 2011, MOL SYST BIOL, V7, DOI 10.1038/msb.2011.2

Ives A, 2015, NAT COMMUN, V6, DOI 10.1038/ncomms7555

Jaramillo MC, 2013, GENE DEV, V27, P2179, DOI 10.1101/gad.225680.113

John S, 2011, PLOS ONE, V6, DOI 10.1371/journal.pone.0017674

Kim TH, 2011, CANCER RES, V71, P2260, DOI 10.1158/0008-5472.CAN-10-3007

Kovac S, 2015, BBA-GEN SUBJECTS, V1850, P794, DOI 10.1016/j.bbagen.2014.11.021

Maksin-Matveev A, 2015, EXP CELL RES, V330, P81, DOI 10.1016/j.yexcr.2014.07.013

Malhotra D, 2010, NUCLEIC ACIDS RES, V38, P5718, DOI 10.1093/nar/gkq212

Martinez-Outschoorn UE, 2013, CELL CYCLE, V12, P2580, DOI 10.4161/cc.25510

Mathupala SP, 2001, J BIOL CHEM, V276, P43407, DOI 10.1074/jbc.M108181200

Mergenthaler P, 2012, P NATL ACAD SCI USA, V109, P1518, DOI 10.1073/pnas.1108225109

Mitsuishi Y, 2012, CANCER CELL, V22, P66, DOI 10.1016/j.ccr.2012.05.016

Neary CL, 2010, BIOCHEM BIOPH RES CO, V394, P1075, DOI 10.1016/j.bbrc.2010.03.129

Nguyen T, 2009, J BIOL CHEM, V284, P13291, DOI 10.1074/jbc.R900010200

O'Neill LAJ, 2013, NATURE, V493, P346, DOI 10.1038/nature11862

Pan HZ, 2016, CELL RES, V26, P190, DOI 10.1038/cr.2016.4

Peláez R, 2009, J BIOL CHEM, V284, P20548, DOI 10.1074/jbc.M109.013730

Reichard JF, 2007, NUCLEIC ACIDS RES, V35, P7074, DOI 10.1093/nar/gkm638

Sanman LE, 2016, ELIFE, V5, DOI 10.7554/eLife.13663

Schroder K, 2010, SCIENCE, V327, P296, DOI 10.1126/science.1184003

Sharma V, 2007, MOL CANCER THER, V6, P2544, DOI 10.1158/1535-7163.MCT-06-0788

Sharma V, 2011, J MOL MED, V89, P123, DOI 10.1007/s00109-010-0683-5

SZATROWSKI TP, 1991, CANCER RES, V51, P794

Theodore M, 2008, J BIOL CHEM, V283, P8984, DOI 10.1074/jbc.M709040200

Vega M, 2016, J BIOL CHEM, V291, P7267, DOI 10.1074/jbc.M115.711408

Wolf A, 2011, J EXP MED, V208, P313, DOI 10.1084/jem.20101470

Wolf AJ, 2016, CELL, V166, P624, DOI 10.1016/j.cell.2016.05.076

Wu RX, 2012, EMBO MOL MED, V4, P633, DOI 10.1002/emmm.201200240

Yang WW, 2011, NATURE, V480, P118, DOI 10.1038/nature10598

Zhong L, 2010, CELL, V140, P280, DOI 10.1016/j.cell.2009.12.041

Zhou RB, 2011, NATURE, V469, P221, DOI 10.1038/nature09663

Zhou YF, 2011, J BIOL CHEM, V286, P32843, DOI 10.1074/jbc.M111.260935

NR 44

TC 24

Z9 26

U1 1

U2 15

PU AMER SOC BIOCHEMISTRY MOLECULAR BIOLOGY INC

PI BETHESDA

PA 9650 ROCKVILLE PIKE, BETHESDA, MD 20814-3996 USA

SN 0021-9258

EI 1083-351X

J9 J BIOL CHEM

JI J. Biol. Chem.

PD MAR 30

PY 2018

VL 293

IS 13

BP 4767

EP 4777

DI 10.1074/jbc.M117.816785

PG 11

WC Biochemistry & Molecular Biology

WE Science Citation Index Expanded (SCI-EXPANDED)

SC Biochemistry & Molecular Biology

GA GB1YT

UT WOS:000428848300018

PM 29414774

OA Green Published

DA 2025-04-09

ER

PT J

AU Li, J

Ren, SY

Liu, YJ

Lian, ZG

Dong, B

Yao, YQ

Xu, YH

AF Li, Jun

Ren, Siyang

Liu, Yongjian

Lian, Zhigang

Dong, Bin

Yao, Yiqun

Xu, Yinghui

TI Knockdown of NUPR1 inhibits the proliferation of glioblastoma cells via

ERK1/2, p38 MAPK and caspase-3

SO JOURNAL OF NEURO-ONCOLOGY

LA English

DT Article

DE Globlastoma; NUPR1; Proliferation; Migration; Apoptosis

ID NUCLEAR-PROTEIN 1; TRANSCRIPTION FACTORS; OXIDATIVE STRESS;

GLIOMA-CELLS; P8 PROTEIN; IN-VITRO; CANCER; EXPRESSION; APOPTOSIS;

CARCINOMA

AB Nuclear protein-1 (NUPR1), located on chromosome 16p11.2, is a stress response factor that plays an important role in the growth and migration of human malignant tumor cells. However, the role of NUPR1 in glioblastoma remains poorly understood. The expression level of NUPR1 was detected by quantitative real-time PCR and immunohistochemistry (IHC). Wound healing, MTT, cell counting and BrdU assays were used to analyze the migration and proliferation of glioblastoma cells after down-regulating NUPR1 expression using a lentiviral vector. FACS analysis and a signaling antibody array kit were used to detect the mechanism by which NUPR1 modulates cell cycle and apoptosis activities in glioblastoma cells. We confirmed that NUPR1 was up-regulated in glioblastoma tissues compared to NB tissues. Down-regulation of NUPR1 suppressed cell migration and proliferation, arrested the cell cycle in the G0/G1 phase and promoted apoptosis in U251 and U87 cells in vitro. Furthermore, the expression levels of phosphorylated ERK1/2, p38 MAPK and cleaved caspase-3 were decreased upon silencing NUPR1 expression in U251 and U87 cells. In summary, NUPR1 plays an important role in the growth and migration of human glioblastoma cells. Knockdown of NUPR1 suppressed glioblastoma cell growth by arresting the cell cycle and inducing cell apoptosis via decreases in the expression of ERK1/2, p38 MAPK and caspase-3.

C1 [Li, Jun; Ren, Siyang; Lian, Zhigang; Dong, Bin; Yao, Yiqun; Xu, Yinghui] Dalian Med Univ, Dept Neurosurg, Affiliated Hosp 1, 222 Zhong Shan Rd, Dalian 116011, Peoples R China.

[Liu, Yongjian] Dalian Med Univ, Affiliated Hosp 1, Dept Intervent Therapy, Dalian, Peoples R China.

C3 Dalian Medical University; Dalian Medical University

RP Yao, YQ; Xu, YH (corresponding author), Dalian Med Univ, Dept Neurosurg, Affiliated Hosp 1, 222 Zhong Shan Rd, Dalian 116011, Peoples R China.

EM quince58@163.com; xuyh_dl@126.com

RI ren, siyang/GYJ-0039-2022; Xu, Yinghui/JBS-3453-2023

FU Natural Science Foundation of China (NSFC) [81172180]

FX The study was supported by the Natural Science Foundation of China

(NSFC) (No. 81172180).

CR Bak Y, 2015, BIOCHEM BIOPH RES CO, V466, P676, DOI 10.1016/j.bbrc.2015.09.082

Brat DJ, 2007, BRAIN PATHOL, V17, P319, DOI 10.1111/j.1750-3639.2007.00082.x

Cano CE, 2014, GUT, V63, P984, DOI 10.1136/gutjnl-2013-305221

Cano CE, 2011, J CELL PHYSIOL, V226, P1439, DOI 10.1002/jcp.22324

Carracedo A, 2006, FEBS LETT, V580, P1571, DOI 10.1016/j.febslet.2006.01.084

Clark DW, 2008, CURR CANCER DRUG TAR, V8, P421, DOI 10.2174/156800908785133196

Cui YT, 2016, FOOD CHEM TOXICOL, V92, P26, DOI 10.1016/j.fct.2016.03.013

Di Martino MT, 2015, ONCOTARGET, V6, P19132, DOI 10.18632/oncotarget.4302

Dolka I, 2016, RES VET SCI, V105, P124, DOI 10.1016/j.rvsc.2016.02.004

Encinar JA, 2001, J BIOL CHEM, V276, P2742, DOI 10.1074/jbc.M008594200

Garcia-Montero A, 2001, EUR J CELL BIOL, V80, P720, DOI 10.1078/0171-9335-00209

Goruppi S, 2007, MOL CELL BIOL, V27, P993, DOI 10.1128/MCB.00996-06

Goruppi S, 2010, J BIOL CHEM, V285, P1577, DOI 10.1074/jbc.R109.080887

Guo XT, 2012, ANAT REC, V295, P2114, DOI 10.1002/ar.22571

Hoffmeister A, 2002, J BIOL CHEM, V277, P22314, DOI 10.1074/jbc.M201657200

Hu WM, 2015, MEDICINE, V94, DOI 10.1097/MD.0000000000000979

Huang Hongyan, 2015, Xi Bao Yu Fen Zi Mian Yi Xue Za Zhi, V31, P782

Ito Y, 2005, ANTICANCER RES, V25, P833

Ito Y, 2005, ANTICANCER RES, V25, P3419

Jiang T, 2016, CANCER LETT, V375, P263, DOI 10.1016/j.canlet.2016.01.024

Jiang WG, 2006, INT J MOL MED, V18, P981

Jung SH, 2012, BMC CANCER, V12, DOI 10.1186/1471-2407-12-382

Lee YK, 2015, BMB REP, V48, P597, DOI 10.5483/BMBRep.2015.48.11.180

Lee YS, 2015, EVID-BASED COMPL ALT, V2015, DOI 10.1155/2015/520578

Lefranc F, 2006, EXPERT REV ANTICANC, V6, P719, DOI 10.1586/14737140.6.5.719

Liu XR, 2015, EUR REV MED PHARMACO, V19, P4068

Liu YJ, 2016, SCI REP-UK, V6, DOI 10.1038/srep20642

Liu Y, 2015, J BIOL CHEM, V290, P11843, DOI 10.1074/jbc.M114.629931

Lorente M, 2009, GLIA, V57, P1374, DOI 10.1002/glia.20856

Malicet C, 2006, P NATL ACAD SCI USA, V103, P2671, DOI 10.1073/pnas.0508955103

Mallo GV, 1997, J BIOL CHEM, V272, P32360, DOI 10.1074/jbc.272.51.32360

Ohgaki H, 2004, CANCER RES, V64, P6892, DOI 10.1158/0008-5472.CAN-04-1337

Pedrola N, 2015, CLIN EXP METASTAS, V32, P467, DOI 10.1007/s10585-015-9720-7

Sathornsumetee S, 2007, NEUROL CLIN, V25, P1111, DOI 10.1016/j.ncl.2007.07.004

Su SB, 2001, CLIN CANCER RES, V7, P1320

Veerla S, 2008, GENE CHROMOSOME CANC, V47, P368, DOI 10.1002/gcc.20542

Xu L, 2016, TUMOR BIOL, V2016, P1, DOI DOI 10.1080/13607863.2016.1181708

Zeng Z, 2015, PLOS ONE, V10, DOI 10.1371/journal.pone.0119912

Zhang L, 2015, MED SCI MONITOR, V21, P3629, DOI 10.12659/MSM.894006

NR 39

TC 40

Z9 44

U1 1

U2 9

PU SPRINGER

PI NEW YORK

PA 233 SPRING ST, NEW YORK, NY 10013 USA

SN 0167-594X

EI 1573-7373

J9 J NEURO-ONCOL

JI J. Neuro-Oncol.

PD MAR

PY 2017

VL 132

IS 1

BP 15

EP 26

DI 10.1007/s11060-016-2337-0

PG 12

WC Oncology; Clinical Neurology

WE Science Citation Index Expanded (SCI-EXPANDED)

SC Oncology; Neurosciences & Neurology

GA EQ4MV

UT WOS:000398052800003

PM 28000106

DA 2025-04-09

ER

PT J

AU Im, CN

Yun, HH

Lee, JH

AF Im, Chang-Nim

Yun, Hye Hyeon

Lee, Jeong-Hwa

TI Heat Shock Factor 1 Depletion Sensitizes A172 Glioblastoma Cells to

Temozolomide via Suppression of Cancer Stem Cell-Like Properties

SO INTERNATIONAL JOURNAL OF MOLECULAR SCIENCES

LA English

DT Article

DE BIS; HSF1; glioblastoma; temozolomide; apoptosis

ID MESENCHYMAL TRANSITION; OXIDATIVE STRESS; BAG3 EXPRESSION; MGMT

EXPRESSION; BREAST-CANCER; PROTEIN; HSF1; RESISTANCE; BIS; INHIBITION

AB Heat shock factor 1 (HSF1), a transcription factor activated by various stressors, regulates proliferation and apoptosis by inducing expression of target genes, such as heat shock proteins and Bcl-2 (B-cell lymphoma 2) interacting cell death suppressor (BIS). HSF1 also directly interacts with BIS, although it is still unclear whether this interaction is critical in the regulation of glioblastoma stem cells (GSCs). In this study, we examined whether small interfering RNA-mediated BIS knockdown decreased protein levels of HSF1 and subsequent nuclear localization under GSC-like sphere (SP)-forming conditions. Consistent with BIS depletion, HSF1 knockdown also reduced sex determining region Y (SRY)-box 2 (SOX2) expression, a marker of stemness, accompanying the decrease in SP-forming ability and matrix metalloprotease 2 (MMP2) activity. When HSF1 or BIS knockdown was combined with temozolomide (TMZ) treatment, a standard drug used in glioblastoma therapy, apoptosis increased, as measured by an increase in poly (ADP-ribose) polymerase (PARP) cleavage, whereas cancer stem-like properties, such as colony-forming activity and SOX2 protein expression, decreased. Taken together, our findings suggest that targeting BIS or HSF1 could be a viable therapeutic strategy for GSCs resistant to conventional TMZ treatment.

C1 [Im, Chang-Nim; Yun, Hye Hyeon; Lee, Jeong-Hwa] Catholic Univ Korea, Dept Biochem, Seoul 06591, South Korea.

[Im, Chang-Nim; Yun, Hye Hyeon; Lee, Jeong-Hwa] Catholic Univ Korea, Inst Aging & Metab Dis, Seoul 06591, South Korea.

[Im, Chang-Nim; Lee, Jeong-Hwa] Catholic Univ Korea, Coll Med, Canc Evolut Res Ctr, Seoul 06591, South Korea.

C3 Catholic University of Korea; Catholic University of Korea; Catholic

University of Korea

RP Im, CN; Lee, JH (corresponding author), Catholic Univ Korea, Dept Biochem, Seoul 06591, South Korea.; Im, CN; Lee, JH (corresponding author), Catholic Univ Korea, Inst Aging & Metab Dis, Seoul 06591, South Korea.; Im, CN; Lee, JH (corresponding author), Catholic Univ Korea, Coll Med, Canc Evolut Res Ctr, Seoul 06591, South Korea.

EM milybud@gmail.com; nice1205@hanmail.net; leejh@catholic.ac.kr

RI Lee, Jeong/JFA-4725-2023

OI Im, Chang-Nim/0000-0002-7566-7075

FU Basic Science Research Program through the National Research Foundation

of Korea (NRF) - Ministry of Science, ICT and future Planning

[NRF-2014R1A1A1006961, NRF-2012R1A5A2047939]

FX This research was supported by Basic Science Research Program through

the National Research Foundation of Korea (NRF) funded by the Ministry

of Science, ICT and future Planning (NRF-2014R1A1A1006961 to Chang-Nim

Im and NRF-2012R1A5A2047939 to Jeong-Hwa Lee).

CR Boiani M, 2013, J BIOL CHEM, V288, P6980, DOI 10.1074/jbc.M112.414177

Chen Y, 2013, MOL CELL PROTEOMICS, V12, P2804, DOI 10.1074/mcp.M112.025882

Chuma M, 2014, CARCINOGENESIS, V35, P272, DOI 10.1093/carcin/bgt343

Ciurea ME, 2014, INT J MOL SCI, V15, P8169, DOI 10.3390/ijms15058169

Cui MN, 2016, MOL CELL TOXICOL, V12, P63, DOI 10.1007/s13273-016-0009-y

Dai C, 2007, CELL, V130, P1005, DOI 10.1016/j.cell.2007.07.020

Du ZX, 2009, J CELL PHYSIOL, V218, P631, DOI 10.1002/jcp.21634

Erice O, 2015, MOL CANCER THER, V14, P1236, DOI 10.1158/1535-7163.MCT-14-0810

Franceschelli S, 2008, J CELL PHYSIOL, V215, P575, DOI 10.1002/jcp.21397

Gabelloni P, 2010, NEUROSCIENCE, V168, P514, DOI 10.1016/j.neuroscience.2010.03.064

Garros-Regulez L, 2016, EXPERT OPIN THER TAR, V20, P393, DOI 10.1517/14728222.2016.1151002

Gentilella A, 2011, J BIOL CHEM, V286, P9205, DOI 10.1074/jbc.M110.175836

He YC, 2014, INT J MOL SCI, V15, P8335, DOI 10.3390/ijms15058335

Hong J, 2016, EXP MOL MED, V48, DOI 10.1038/emm.2016.2

Im CN, 2016, ONCOTARGET, V7, P35056, DOI 10.18632/oncotarget.9039

Im CN, 2016, IUBMB LIFE, V68, P173, DOI 10.1002/iub.1475

Im CN, 2015, ONCOL REP, V34, P920, DOI 10.3892/or.2015.4021

Im Chang-Nim, 2013, Genomics & Informatics, V11, P245, DOI 10.5808/GI.2013.11.4.245

Im CN, 2010, ANGEW CHEM INT EDIT, V49, P7497, DOI 10.1002/anie.201002463

Jacobs AT, 2009, J BIOL CHEM, V284, P9176, DOI 10.1074/jbc.M808656200

Jin YH, 2015, BIOCHEM BIOPH RES CO, V464, P561, DOI 10.1016/j.bbrc.2015.07.006

Kim HY, 2016, EXP MOL MED, V48, DOI 10.1038/emm.2016.84

Kohsaka S, 2012, MOL CANCER THER, V11, P1289, DOI 10.1158/1535-7163.MCT-11-0801

Lang BJ, 2012, CELL STRESS CHAPERON, V17, P765, DOI 10.1007/s12192-012-0349-z

Lee JH, 1999, ONCOGENE, V18, P6183, DOI 10.1038/sj.onc.1203043

Liu XD, 1997, EMBO J, V16, P6466, DOI 10.1093/emboj/16.21.6466

Mani SA, 2008, CELL, V133, P704, DOI 10.1016/j.cell.2008.03.027

Matsuda Y, 2015, CANCER LETT, V357, P602, DOI 10.1016/j.canlet.2014.12.030

Mendillo ML, 2012, CELL, V150, P549, DOI 10.1016/j.cell.2012.06.031

Messaoudi K, 2015, DRUG DISCOV TODAY, V20, P899, DOI 10.1016/j.drudis.2015.02.011

Rodríguez AE, 2016, AUTOPHAGY, V12, P287, DOI 10.1080/15548627.2015.1124225

Rosati A, 2012, BBA-REV CANCER, V1826, P365, DOI 10.1016/j.bbcan.2012.06.001

Santagata S, 2011, P NATL ACAD SCI USA, V108, P18378, DOI 10.1073/pnas.1115031108

Vihervaara A, 2014, J CELL SCI, V127, P261, DOI 10.1242/jcs.132605

Villalva C, 2012, INT J MOL SCI, V13, P6983, DOI 10.3390/ijms13066983

Wang B, 2015, BREAST CANCER RES TR, V153, P57, DOI 10.1007/s10549-015-3521-1

Yasui K, 2013, THYROID, V23, P989, DOI 10.1089/thy.2012.0319

Yoo HJ, 2014, KOREAN J PHYSIOL PHA, V18, P403, DOI 10.4196/kjpp.2014.18.5.403

NR 38

TC 18

Z9 21

U1 0

U2 2

PU MDPI

PI BASEL

PA ST ALBAN-ANLAGE 66, CH-4052 BASEL, SWITZERLAND

EI 1422-0067

J9 INT J MOL SCI

JI Int. J. Mol. Sci.

PD FEB

PY 2017

VL 18

IS 2

AR 468

DI 10.3390/ijms18020468

PG 12

WC Biochemistry & Molecular Biology; Chemistry, Multidisciplinary

WE Science Citation Index Expanded (SCI-EXPANDED)

SC Biochemistry & Molecular Biology; Chemistry

GA EM6YD

UT WOS:000395457700234

PM 28241425

OA Green Published, gold, Green Submitted

DA 2025-04-09

ER

PT J

AU Zhang, YM

Liu, Q

Wang, FW

Ling, EA

Liu, SM

Wang, LY

Yang, Y

Yao, LL

Chen, XR

Wang, F

Shi, W

Gao, M

Hao, AJ

AF Zhang, Yanmin

Liu, Qian

Wang, Fuwu

Ling, Eng-Ang

Liu, Shangming

Wang, Liyan

Yang, Yang

Yao, Linli

Chen, Xueran

Wang, Fen

Shi, Wei

Gao, Ming

Hao, Aijun

TI Melatonin antagonizes hypoxia-mediated glioblastoma cell migration and

invasion via inhibition of HIF-1α

SO JOURNAL OF PINEAL RESEARCH

LA English

DT Article

DE glioblastoma multiforme; HIF-1 alpha; hypoxia; invasion; melatonin;

migration

ID ASTROCYTIC GLIOMA; OXIDATIVE STRESS; DOWN-REGULATION; TUMOR HYPOXIA;

HIF-ALPHA; ANGIOGENESIS; CANCER; GROWTH; EXPRESSION; PROGRESSION

AB Hypoxia is a crucial factor in tumor aggressiveness and resistance to therapy, especially in glioblastoma. Our previous results have shown that melatonin exerts antimigratory and anti-invasive action in glioblastoma cells under normoxia. However, the effect of melatonin on migration and invasion of glioblastoma cells under hypoxic condition remains poorly understood. Here, we show that melatonin strongly reduced hypoxia-mediated invasion and migration of U251 and U87 glioblastoma cells. In addition, we found that melatonin significantly blocked HIF-1 alpha protein expression and suppressed the expression of downstream target genes, matrix metalloproteinase 2 (MMP-2) and vascular endothelial growth factor (VEGF). Furthermore, melatonin destabilized hypoxia-induced HIF-1 alpha protein via its antioxidant activity against ROS produced by glioblastoma cells in response to hypoxia. Along with this, HIF-1 alpha silencing by small interfering RNA markedly inhibited glioblastoma cell migration and invasion, and this appeared to be associated with MMP-2 and VEGF under hypoxia. Taken together, our findings suggest that melatonin suppresses hypoxia-induced glioblastoma cell migration and invasion via inhibition of HIF-1 alpha. Considering the fact that overexpression of the HIF-1 alpha protein is often detected in glioblastoma multiforme, melatonin may prove to be a potent therapeutic agent for this tumor.

C1 [Zhang, Yanmin; Liu, Qian; Wang, Fuwu; Liu, Shangming; Wang, Liyan; Yang, Yang; Yao, Linli; Chen, Xueran; Wang, Fen; Shi, Wei; Gao, Ming; Hao, Aijun] Shandong Univ Sch Med, Dept Histol & Embryol, Key Lab, Minist Educ Expt Teratol,Shandong Prov Key Lab Me, Jinan 250012, Shandong, Peoples R China.

[Ling, Eng-Ang] Natl Univ Singapore, Yong Loo Lin Sch Med, Key Dept Anat, Singapore 117595, Singapore.

C3 Shandong University; National University of Singapore

RP Hao, AJ (corresponding author), Shandong Univ Sch Med, Dept Histol & Embryol, Key Lab, Minist Educ Expt Teratol, 44 Wenhua Xi Rd, Jinan 250012, Shandong, Peoples R China.

EM Aijunhao@sdu.edu.cn

RI Shi, Wei/P-2846-2019; Chen, Xueran/AAC-2599-2019; Zhang,

Yinyan/S-7675-2019

OI chen, xue ran/0000-0003-4710-8054; Zhang, Yanmin/0009-0004-8093-0095

FU Science Foundation of Ministry of Education of China [20110131110036];

National Natural Science Foundation of China [81071057, 81271451];

Independent Innovation Foundation of Shandong University [2012JC006];

Natural Science Foundation of Shandong Province [21300005201209,

2012GSF11842]

FX This work was supported by Science Foundation of Ministry of Education

of China (No. 20110131110036); National Natural Science Foundation of

China (No. 81071057, 81271451); Independent Innovation Foundation of

Shandong University (No. 2012JC006); Natural Science Foundation of

Shandong Province (No. 21300005201209, 2012GSF11842).

CR Alqawi O, 2006, PROSTATE CANCER P D, V9, P126, DOI 10.1038/sj.pcan.4500852

Bar EE, 2010, AM J PATHOL, V177, P1491, DOI 10.2353/ajpath.2010.091021

Blázquez C, 2008, CANCER RES, V68, P1945, DOI 10.1158/0008-5472.CAN-07-5176

Chan DA, 2005, MOL CELL BIOL, V25, P6415, DOI 10.1128/MCB.25.15.6415-6426.2005

Chan DA, 2002, J BIOL CHEM, V277, P40112, DOI 10.1074/jbc.M206922200

Chandel NS, 1998, P NATL ACAD SCI USA, V95, P11715, DOI 10.1073/pnas.95.20.11715

Claes A, 2007, ACTA NEUROPATHOL, V114, P443, DOI 10.1007/s00401-007-0293-7

Cos S, 1998, CANCER RES, V58, P4383

Furnari FB, 2007, GENE DEV, V21, P2683, DOI 10.1101/gad.1596707

Galano A, 2013, J PINEAL RES, V54, P245, DOI 10.1111/jpi.12010

Galano A, 2011, J PINEAL RES, V51, P1, DOI 10.1111/j.1600-079X.2011.00916.x

Gao C, 2012, J PINEAL RES, V52, P305, DOI 10.1111/j.1600-079X.2011.00944.x

Gondi CS, 2004, NEURON GLIA BIOL, V1, P165, DOI 10.1017/S1740925X04000237

Guo P, 2005, AM J PATHOL, V166, P877, DOI 10.1016/S0002-9440(10)62308-5

Harris AL, 2002, NAT REV CANCER, V2, P38, DOI 10.1038/nrc704

Hlobilkova A, 2009, NEOPLASMA, V56, P284, DOI 10.4149/neo_2009_04_284

Hockel M, 1996, CANCER RES, V56, P4509

Ivan M, 2001, SCIENCE, V292, P464, DOI 10.1126/science.1059817

Jaakkola P, 2001, SCIENCE, V292, P468, DOI 10.1126/science.1059796

Jensen RL, 2009, J NEURO-ONCOL, V92, P317, DOI 10.1007/s11060-009-9827-2

Kaur B, 2005, NEURO-ONCOLOGY, V7, P134, DOI 10.1215/S1152851704001115

Kim KJ, 2013, J PINEAL RES, V54, P264, DOI 10.1111/j.1600-079X.2012.01030.x

Krishnamachary B, 2003, CANCER RES, V63, P1138

Lanoix D, 2008, J PINEAL RES, V45, P50, DOI 10.1111/j.1600-079X.2008.00555.x

Lee OH, 2008, ONCOGENE, V27, P1310, DOI 10.1038/sj.onc.1210731

Lefranc F, 2009, ADV TECH STAND NEURO, V34, P3, DOI 10.1007/978-3-211-78741-0_1

Liao D, 2007, CANCER METAST REV, V26, P281, DOI 10.1007/s10555-007-9066-y

Liu Y, 2012, J PINEAL RES, V52, P47, DOI 10.1111/j.1600-079X.2011.00917.x

Mao LL, 2010, BREAST CANCER RES, V12, DOI 10.1186/bcr2794

Mashiko R, 2011, J NEURO-ONCOL, V102, P43, DOI 10.1007/s11060-010-0292-8

Mauriz JL, 2013, J PINEAL RES, V54, P1, DOI 10.1111/j.1600-079X.2012.01014.x

Melillo G, 2006, CANCER RES, V66, P4558, DOI 10.1158/0008-5472.CAN-06-0069

Muñoz-Nájar UM, 2006, ONCOGENE, V25, P2379, DOI 10.1038/sj.onc.1209273

Ortíz-López L, 2009, J PINEAL RES, V46, P15, DOI 10.1111/j.1600-079X.2008.00600.x

Park SY, 2010, J PINEAL RES, V48, P178, DOI 10.1111/j.1600-079X.2009.00742.x

Roskoski R, 2007, CRIT REV ONCOL HEMAT, V62, P179, DOI 10.1016/j.critrevonc.2007.01.006

Semenza GL, 2012, TRENDS PHARMACOL SCI, V33, P207, DOI 10.1016/j.tips.2012.01.005

Soeda A, 2009, ONCOGENE, V28, P3949, DOI 10.1038/onc.2009.252

Swarnakar S, 2011, J PINEAL RES, V50, P8, DOI 10.1111/j.1600-079X.2010.00812.x

Venegas C, 2012, J PINEAL RES, V52, P217, DOI 10.1111/j.1600-079X.2011.00931.x

Vijayalaxmi, 2002, J CLIN ONCOL, V20, P2575, DOI 10.1200/JCO.2002.11.004

Wang JT, 2012, J PINEAL RES, V53, P180, DOI 10.1111/j.1600-079X.2012.00985.x

Warnecke C, 2004, FASEB J, V18, P1462, DOI 10.1096/fj.04-1640fje

Wu CC, 2012, J PINEAL RES, V52, P460, DOI 10.1111/j.1600-079X.2011.00960.x

Yang CS, 2007, J NEUROINFLAMM, V4, DOI 10.1186/1742-2094-4-27

Yu F, 2001, P NATL ACAD SCI USA, V98, P9630, DOI 10.1073/pnas.181341498

Zagzag D, 2003, J CELL PHYSIOL, V196, P394, DOI 10.1002/jcp.10306

Zagzag D, 2000, CANCER-AM CANCER SOC, V88, P2606, DOI 10.1002/1097-0142(20000601)88:11<2606::AID-CNCR25>3.0.CO;2-W

Zhang XX, 2011, J BIOL CHEM, V286, P1429, DOI 10.1074/jbc.M110.146530

Zhang YM, 2011, CANCER SCI, V102, P1991, DOI 10.1111/j.1349-7006.2011.02059.x

NR 50

TC 83

Z9 88

U1 0

U2 24

PU WILEY

PI HOBOKEN

PA 111 RIVER ST, HOBOKEN 07030-5774, NJ USA

SN 0742-3098

EI 1600-079X

J9 J PINEAL RES

JI J. Pineal Res.

PD SEP

PY 2013

VL 55

IS 2

BP 121

EP 130

DI 10.1111/jpi.12052

PG 10

WC Endocrinology & Metabolism; Neurosciences; Physiology

WE Science Citation Index Expanded (SCI-EXPANDED)

SC Endocrinology & Metabolism; Neurosciences & Neurology; Physiology

GA 196AF

UT WOS:000322744600002

PM 23551342

DA 2025-04-09

ER

PT J

AU McKelvey, KJ

Wilson, EB

Short, S

Melcher, AA

Biggs, M

Diakos, C

Howell, VM

AF McKelvey, Kelly J.

Wilson, Erica B.

Short, Susan

Melcher, Alan A.

Biggs, Michael

Diakos, Connie, I

Howell, Viive M.

TI Glycolysis and Fatty Acid Oxidation Inhibition Improves Survival in

Glioblastoma

SO FRONTIERS IN ONCOLOGY

LA English

DT Article

DE glioblastoma; cancer metabolism; ranolazine; dichloroacetate; radiation;

temozolomide

ID CELLS; TEMOZOLOMIDE; METABOLISM; SUPPRESSOR; MODELS; CANCER

AB Glioblastoma (GBM) is the most aggressive adult glioma with a median survival of 14 months. While standard treatments (safe maximal resection, radiation, and temozolomide chemotherapy) have increased the median survival in favorable O(6)-methylguanine-DNA methyltransferase (MGMT)-methylated GBM (similar to 21 months), a large proportion of patients experience a highly debilitating and rapidly fatal disease. This study examined GBM cellular energetic pathways and blockade using repurposed drugs: the glycolytic inhibitor, namely dicholoroacetate (DCA), and the partial fatty acid oxidation (FAO) inhibitor, namely ranolazine (Rano). Gene expression data show that GBM subtypes have similar glucose and FAO pathways, and GBM tumors have significant upregulation of enzymes in both pathways, compared to normal brain tissue (p < 0.01). DCA and the DCA/Rano combination showed reduced colony-forming activity of GBM and increased oxidative stress, DNA damage, autophagy, and apoptosis in vitro. In the orthotopic Gl261 and CT2A syngeneic murine models of GBM, DCA, Rano, and DCA/Rano increased median survival and induced focal tumor necrosis and hemorrhage. In conclusion, dual targeting of glycolytic and FAO metabolic pathways provides a viable treatment that warrants further investigation concurrently or as an adjuvant to standard chemoradiation for GBM.

C1 [McKelvey, Kelly J.; Diakos, Connie, I; Howell, Viive M.] Univ Sydney, Fac Med & Hlth, Bill Walsh Translat Canc Res Lab, St Leonards, NSW, Australia.

[Wilson, Erica B.; Short, Susan] Univ Leeds, Translat Neurooncol, Leeds Inst Med Res St Jamess, Leeds, W Yorkshire, England.

[Melcher, Alan A.] Inst Canc Res, Div Radiotherapy & Imaging, Translat Immunotherapy, London, England.

[Biggs, Michael] North Shore Private Hosp, Dept Neurosurg, St Leonards, NSW, Australia.

[Diakos, Connie, I] Royal North Shore Hosp, Northern Sydney Canc Ctr, Dept Med Oncol, St Leonards, NSW, Australia.

[Diakos, Connie, I] Univ Sydney, Fac Med & Hlth, Northern Clin Sch, St Leonards, NSW, Australia.

C3 University of Sydney; University of Leeds; University of London;

Institute of Cancer Research - UK; Royal Marsden NHS Foundation Trust;

Royal North Shore Hospital; University of Sydney

RP McKelvey, KJ (corresponding author), Univ Sydney, Fac Med & Hlth, Bill Walsh Translat Canc Res Lab, St Leonards, NSW, Australia.

EM keliy.mckelvey@sydney.edu.au

OI McKelvey, Kelly/0000-0002-4923-791X; Wilson, Erica/0000-0002-0441-728X;

Short, Susan/0000-0003-4423-7256

FU Matt Callander Beanie for Brain Cancer Hunter Medical Research Institute

(HMRI) Fellowship - Mark Hughes Foundation (MHF) [HMRI 780]; MHF

Innovation Grant [HMRI 1357]

FX KM was supported by the Matt Callander Beanie for Brain Cancer Hunter

Medical Research Institute (HMRI) Fellowship funded by the Mark Hughes

Foundation (MHF; HMRI 780) and the work supported by an MHF Innovation

Grant (HMRI 1357).

CR Aldasoro M, 2016, PLOS ONE, V11, DOI 10.1371/journal.pone.0150619

Begolly S, 2018, GLIA, V66, P846, DOI 10.1002/glia.23288

CHOU TC, 1984, ADV ENZYME REGUL, V22, P27, DOI 10.1016/0065-2571(84)90007-4

Chou TC, 2006, PHARMACOL REV, V58, P621, DOI 10.1124/pr.58.3.10

de Hoon MJL, 2004, BIOINFORMATICS, V20, P1453, DOI 10.1093/bioinformatics/bth078

Dolecek TA, 2012, NEURO-ONCOLOGY, V14, pv1, DOI 10.1093/neuonc/nos218

Dunbar EM, 2014, INVEST NEW DRUG, V32, P452, DOI 10.1007/s10637-013-0047-4

Duraj T, 2021, CELLS-BASEL, V10, DOI 10.3390/cells10020202

Eriksson M, 2017, MOL CELL BIOL, V37, DOI 10.1128/MCB.00328-17

Fereidoonnezhad M, 2016, INDIAN J PHARM EDUC, V50, pS32, DOI 10.5530/ijper.50.2.15

Gilbert MR, 2013, J CLIN ONCOL, V31, P4085, DOI 10.1200/JCO.2013.49.6968

Gnanapradeepan K, 2018, FRONT ENDOCRINOL, V9, DOI 10.3389/fendo.2018.00124

Goetzman ES, 2018, FRONT ENDOCRINOL, V9, DOI 10.3389/fendo.2018.00129

Health AIo Welfare, 2020, CANC DATA AUSTR

Hossain F, 2015, CANCER IMMUNOL RES, V3, P1236, DOI 10.1158/2326-6066.CIR-15-0036

Jiang W, 2016, ONCOTARGET, V7, P56456, DOI 10.18632/oncotarget.10919

Kant S, 2020, CELL DEATH DIS, V11, DOI 10.1038/s41419-020-2449-5

Keil C., 2016, TREEVIEW 3 BETA 1 VI

Kim J, 2019, CANCER CELL, V35, P191, DOI 10.1016/j.ccell.2018.12.012

Lin H, 2017, NEURO-ONCOLOGY, V19, P43, DOI 10.1093/neuonc/now128

Ma YB, 2020, SCI REP-UK, V10, DOI 10.1038/s41598-020-58334-7

McKelvey KJ, 2020, PLOS ONE, V15, DOI 10.1371/journal.pone.0226444

McKelvey KJ, 2020, FRONT ONCOL, V9, DOI 10.3389/fonc.2019.01504

Michelakis ED, 2010, SCI TRANSL MED, V2, DOI 10.1126/scitranslmed.3000677

Moher D, 2009, ANN INTERN MED, V151, P264, DOI [10.7326/0003-4819-151-4-200908180-00135, 10.1016/j.ijsu.2010.07.299, 10.1186/2046-4053-4-1, 10.1371/journal.pmed.1000097, 10.1136/bmj.b2700, 10.1136/bmj.b2535, 10.1016/j.ijsu.2010.02.007, 10.1136/bmj.i4086]

Oh T, 2014, J TRANSL MED, V12, DOI 10.1186/1479-5876-12-107

Olar A, 2014, J PATHOL, V232, P165, DOI 10.1002/path.4282

Parker NR, 2016, SCI REP-UK, V6, DOI 10.1038/srep22477

Prabhu AH, 2019, NEURO-ONCOLOGY, V21, P337, DOI 10.1093/neuonc/noy185

Roos WP, 2007, ONCOGENE, V26, P186, DOI 10.1038/sj.onc.1209785

Sánchez-Martín P, 2018, J CELL SCI, V131, DOI 10.1242/jcs.222836

Shen H, 2015, MOL CANCER THER, V14, P1794, DOI 10.1158/1535-7163.MCT-15-0247

Shen H, 2015, J EXP CLIN CANC RES, V34, DOI 10.1186/s13046-015-0130-0

Stupp R, 2005, NEW ENGL J MED, V352, P987, DOI 10.1056/NEJMoa043330

Tang ZF, 2017, NUCLEIC ACIDS RES, V45, pW98, DOI 10.1093/nar/gkx247

Verhaak RGW, 2010, CANCER CELL, V17, P98, DOI 10.1016/j.ccr.2009.12.020

Xie Y, 2015, EBIOMEDICINE, V2, P1351, DOI 10.1016/j.ebiom.2015.08.026

NR 37

TC 38

Z9 39

U1 3

U2 9

PU FRONTIERS MEDIA SA

PI LAUSANNE

PA AVENUE DU TRIBUNAL FEDERAL 34, LAUSANNE, CH-1015, SWITZERLAND

SN 2234-943X

J9 FRONT ONCOL

JI Front. Oncol.

PD MAR 29

PY 2021

VL 11

AR 633210

DI 10.3389/fonc.2021.633210

PG 18

WC Oncology

WE Science Citation Index Expanded (SCI-EXPANDED)

SC Oncology

GA RL7HR

UT WOS:000639139900001

PM 33854970

OA Green Published, gold

DA 2025-04-09

ER

PT J

AU Pinho, RA

Muller, AP

Marqueze, LF

Radak, Z

Arida, RM

AF Pinho, R. A.

Muller, A. P.

Marqueze, L. F.

Radak, Z.

Arida, R. M.

TI Physical exercise-mediated neuroprotective mechanisms in Parkinson's

disease, Alzheimer's disease, and epilepsy

SO BRAZILIAN JOURNAL OF MEDICAL AND BIOLOGICAL RESEARCH

LA English

DT Article

DE Physical exercise; Neuroprotection; Parkinson's disease; Alzheimer's

disease; Glioblastoma; Epilepsy

ID CEREBRAL-BLOOD-FLOW; OXIDATIVE STRESS; ELECTROENCEPHALOGRAPHIC

RESPONSES; NEUROTROPHIC FACTOR; BRAIN; RATS; INSULIN; MEMORY; MODEL;

INFLAMMATION

AB Research suggests that physical exercise is associated with prevention and management of chronic diseases. The influence of physical exercise on brain function and metabolism and the mechanisms involved are well documented in the literature. This review provides a comprehensive overview of the potential implications of physical exercise and the molecular benefits of exercise in Parkinson's disease, Alzheimer's disease, and epilepsy. Here, we present an overview of the effects of exercise on various aspects of metabolism and brain function. To this end, we conducted an extensive literature search of the PubMed, Web of Science, and Google Scholar databases to identify articles published in the past two decades. This review delves into key aspects including the modulation of neuroinflammation, neurotrophic factors, and synaptic plasticity. Moreover, we explored the potential role of exercise in advancing therapeutic strategies for these chronic diseases. In conclusion, the review highlights the importance of regular physical exercise as a complementary non-pharmacological treatment for individuals with neurological disorders such as Alzheimer's, Parkinson's disease, and epilepsy.

C1 [Pinho, R. A.; Marqueze, L. F.] Pontificia Univ Catolica Parana, Lab Bioquim Exercicio Saude, Programa Posgrad Ciencias Saude, Escola Med & Ciencias Vida, Curitiba, PR, Brazil.

[Pinho, R. A.; Arida, R. M.] Rede Nacl Neurociencia & Atividade Fis, Rio De Janeiro, Brazil.

[Muller, A. P.] Univ Fed Santa Catarina, Dept Bioquim, Florianopolis, SC, Brazil.

[Radak, Z.] Hungarian Univ Sport Sci, Res Inst Sport Sci, Budapest, Hungary.

[Arida, R. M.] Univ Fed Sao Paulo, Dept Fisiol, Botucatu, SP, Brazil.

C3 Pontificia Universidade Catolica do Parana; Universidade Federal de

Santa Catarina (UFSC); Universidade Federal de Sao Paulo (UNIFESP)

RP Pinho, RA (corresponding author), Pontificia Univ Catolica Parana, Lab Bioquim Exercicio Saude, Programa Posgrad Ciencias Saude, Escola Med & Ciencias Vida, Curitiba, PR, Brazil.; Pinho, RA (corresponding author), Rede Nacl Neurociencia & Atividade Fis, Rio De Janeiro, Brazil.

EM rapinho12@gmail.com

RI Arida, Ricardo/P-7408-2016; Marqueze, Luis/AAX-8574-2021; A. Pinho,

Ricardo/G-2643-2012

OI A. Pinho, Ricardo/0000-0003-3116-4553; Marqueze,

Luis/0000-0002-4738-1440; Muller, alexandre/0000-0002-9961-8614

FU National Council for Scientific and Technological Development, CNPq

[442941/2023-4, 302689/2022-2, 308734/2021-1]; Coordination for the

Improvement of Higher Education Personnel, CAPES [001]; So Paulo

Research Foundation, FAPESP [2022/10696-1]

FX The authors would like to thank Brazilian agencies: National Council for

Scientific and Technological Development, CNPq (R.A. Pinho

442941/2023-4; R.M. Arida 302689/2022-2; A.P. Muller 308734/2021-1) ,

Coordination for the Improvement of Higher Education Personnel, CAPES

(Finance Code 001) , and the S & atilde;o Paulo Research Foundation,

FAPESP (R.M. Arida 2022/10696-1) for their support.

CR Aguiar AS, 2011, MECH AGEING DEV, V132, P560, DOI 10.1016/j.mad.2011.09.005

Silva LFA, 2013, J NEUROTRAUM, V30, P1278, DOI 10.1089/neu.2012.2577

Andrews SJ, 2023, EBIOMEDICINE, V90, DOI 10.1016/j.ebiom.2023.104511

Arida RM, 2009, EPILEPSY BEHAV, V16, P381, DOI 10.1016/j.yebeh.2009.08.023

Arida RM, 2007, PROG NEURO-PSYCHOPH, V31, P814, DOI 10.1016/j.pnpbp.2007.01.021

Arida RM, 2021, BBA-MOL BASIS DIS, V1867, DOI 10.1016/j.bbadis.2020.165979

Arida RM, 2013, CURR PHARM DESIGN, V19, P6720

Arida RM, 2010, EPILEPSY BEHAV, V17, P432, DOI 10.1016/j.yebeh.2010.01.013

Arida RM, 2004, PHYSIOL BEHAV, V83, P165, DOI 10.1016/j.physbeh.2004.08.008

Arida RM, 2003, PHYSIOL BEHAV, V79, P789, DOI 10.1016/S0031-9384(03)00204-X

Azevedo CV, 2023, FRONT NEUROSCI-SWITZ, V17, DOI 10.3389/fnins.2023.1131214

Bergman J, 2000, PSYCHOPHARMACOLOGY, V153, P67, DOI 10.1007/s002130000567

Bettio L, 2019, INT REV NEUROBIOL, V147, P295, DOI 10.1016/bs.irn.2019.07.002

Bloem BR, 2022, J PARKINSON DIS, V12, pS1, DOI 10.3233/JPD-229005

Brown C, 2022, J APPL PHYSIOL, V132, P824, DOI 10.1152/japplphysiol.00866.2021

Burtscher J, 2021, INT J MOL SCI, V22, DOI 10.3390/ijms22126479

Capovilla G, 2016, EPILEPSIA, V57, P6, DOI 10.1111/epi.13261

CENDES F, 1993, NEUROLOGY, V43, P1083, DOI 10.1212/WNL.43.6.1083

Chan WS, 2024, SCI SIGNAL, V17, DOI 10.1126/scisignal.adh2783

Chen C, 2023, AGEING RES REV, V86, DOI 10.1016/j.arr.2023.101868

Choi SH, 2018, SCIENCE, V361, P991, DOI 10.1126/science.aan8821

Craft S, 2020, JAMA NEUROL, V77, P1099, DOI 10.1001/jamaneurol.2020.1840

de Farias JM, 2021, MOL NEUROBIOL, DOI 10.1007/s12035-021-02411-z

de Freitas GB, 2020, J NEUROCHEM, V155, P602, DOI 10.1111/jnc.15039

De la Rosa A, 2019, SCI REP-UK, V9, DOI 10.1038/s41598-019-40040-8

de Lima C, 2011, EPILEPSY BEHAV, V22, P718, DOI 10.1016/j.yebeh.2011.08.033

Dienel GA, 2023, EPILEPSIA, V64, P29, DOI 10.1111/epi.17412

Drouin-Ouellet J, 2023, NAT REV NEUROSCI, V24, P193, DOI 10.1038/s41583-023-00676-y

Dworak M, 2007, NEUROSCIENCE, V150, P789, DOI 10.1016/j.neuroscience.2007.09.062

El Hayek L, 2019, J NEUROSCI, V39, P2369, DOI 10.1523/JNEUROSCI.1661-18.2019

Erickson KI, 2011, P NATL ACAD SCI USA, V108, P3017, DOI 10.1073/pnas.1015950108

ERIKSEN HR, 1994, EPILEPSIA, V35, P1256, DOI 10.1111/j.1528-1157.1994.tb01797.x

Ernst M, 2023, COCHRANE DB SYST REV, DOI [10.1002/14651858.CD013856.pub2, 10.1002/14651858.CD013856.pub3]

Fabisiak T, 2022, FRONT CELL DEV BIOL, V10, DOI 10.3389/fcell.2022.976953

Fisher RS, 2014, EPILEPSIA, V55, P475, DOI 10.1111/epi.12550

Goodman AM, 2021, NEUROTHERAPEUTICS, V18, P811, DOI 10.1007/s13311-021-01049-y

GOTZE W, 1967, DIS NERV SYST, V28, P664

Häfele CA, 2021, EPILEPSY BEHAV, V117, DOI 10.1016/j.yebeh.2021.107904

Hamer M, 2009, PSYCHOL MED, V39, P3, DOI 10.1017/S0033291708003681

Ide K, 2000, PROG NEUROBIOL, V61, P397, DOI 10.1016/S0301-0082(99)00057-X

Ionescu-Tucker A, 2021, NEUROBIOL AGING, V107, P86, DOI 10.1016/j.neurobiolaging.2021.07.014

Sutachan JJ, 2012, NUTR NEUROSCI, V15, P120, DOI 10.1179/1476830511Y.0000000033

Jensen CS, 2019, EXP GERONTOL, V121, P91, DOI 10.1016/j.exger.2019.04.003

Jin ZJ, 2021, REDOX BIOL, V46, DOI 10.1016/j.redox.2021.102076

Kellar D, 2020, LANCET NEUROL, V19, P758, DOI 10.1016/S1474-4422(20)30231-3

Khaksari K, 2024, NEUROTHERAPEUTICS, V21, DOI 10.1016/j.neurot.2024.e00323

Koo JH, 2017, NEUROSCIENCE, V356, P102, DOI 10.1016/j.neuroscience.2017.05.016

Kuijer A., 1980, Advances in Epileptology: The 10th Epilepsy International Symposium, P543

Kullmann S, 2020, NAT COMMUN, V11, DOI 10.1038/s41467-020-15686-y

Ledreux A, 2019, J ALZHEIMERS DIS, V71, P1245, DOI 10.3233/JAD-190756

Lee YM, 2008, EPILEPSIA, V49, P685, DOI 10.1111/j.1528-1167.2007.01522.x

Liu JX, 2023, SCI REP-UK, V13, DOI 10.1038/s41598-023-33063-9

Lu Y, 2023, TRANSL NEURODEGENER, V12, DOI 10.1186/s40035-023-00341-5

Bosch BM, 2021, SCI REP-UK, V11, DOI 10.1038/s41598-021-93813-5

Masters CL, 2015, NAT REV DIS PRIMERS, V1, DOI 10.1038/nrdp.2015.56

McAuley JW, 2001, EPILEPSY BEHAV, V2, P592, DOI 10.1006/ebeh.2001.0271

Mee-Inta O., 2019, CELLS-BASEL, V8, DOI DOI 10.3390/cells8070691

Merighi S, 2022, INT J MOL SCI, V23, DOI 10.3390/ijms232112990

Misrani A, 2021, FRONT AGING NEUROSCI, V13, DOI 10.3389/fnagi.2021.617588

Moon HY, 2016, CELL METAB, V24, P332, DOI 10.1016/j.cmet.2016.05.025

Moos WH, 2023, MITOCHONDRION, V72, P84, DOI 10.1016/j.mito.2023.08.002

Morris JK, 2017, PLOS ONE, V12, DOI 10.1371/journal.pone.0170547

Muller AP, 2012, J ALZHEIMERS DIS, V30, P889, DOI 10.3233/JAD-2012-112066

Muller AP, 2011, HIPPOCAMPUS, V21, P1082, DOI 10.1002/hipo.20822

Muzio L, 2021, FRONT NEUROSCI-SWITZ, V15, DOI 10.3389/fnins.2021.742065

NAKKEN KO, 1990, EPILEPSIA, V31, P88, DOI 10.1111/j.1528-1157.1990.tb05365.x

Ngugi AK, 2010, EPILEPSIA, V51, P883, DOI 10.1111/j.1528-1167.2009.02481.x

Nilsson J, 2020, SCI REP-UK, V10, DOI 10.1038/s41598-020-60124-0

Nuic D, 2024, EUR J NEUROL, V31, DOI 10.1111/ene.16055

Ogoh S, 2005, AM J PHYSIOL-HEART C, V288, pH1461, DOI 10.1152/ajpheart.00948.2004

Ogoh S, 2009, EXERC SPORT SCI REV, V37, P123, DOI 10.1097/JES.0b013e3181aa64d7

Otáhal J, 2014, INT REV NEUROBIOL, V114, P209, DOI 10.1016/B978-0-12-418693-4.00009-1

Packer L, 2007, FREE RADICAL RES, V41, P951, DOI 10.1080/10715760701490975

Padilha C, 2023, PLOS ONE, V18, DOI 10.1371/journal.pone.0293826

Pajares M, 2020, CELLS-BASEL, V9, DOI 10.3390/cells9071687

Pedata F, 2001, ANN NY ACAD SCI, V939, P74

Pedersen BK, 2019, NAT REV ENDOCRINOL, V15, P383, DOI 10.1038/s41574-019-0174-x

Pinho RA, 2019, ANTIOXIDANTS-BASEL, V8, DOI 10.3390/antiox8110529

Rafie F, 2023, NEUROPEPTIDES, V101, DOI 10.1016/j.npep.2023.102357

Rahman S, 2015, EPILEPSY BEHAV, V49, P71, DOI 10.1016/j.yebeh.2015.05.003

Rahman S, 2013, DEV MED CHILD NEUROL, V55, P23, DOI 10.1111/j.1469-8749.2012.04406.x

Ramdeo KR, 2023, JMIR RES PROTOC, V12, DOI 10.2196/50030

Rathore C, 2014, EPILEPSY RES, V108, P1306, DOI 10.1016/j.eplepsyres.2014.06.012

Rho JM, 2022, NAT REV NEUROL, V18, P333, DOI 10.1038/s41582-022-00651-8

Ruiz-González D, 2021, NEUROSCI BIOBEHAV R, V128, P394, DOI 10.1016/j.neubiorev.2021.05.025

Sachdev S, 2008, FREE RADICAL BIO MED, V44, P215, DOI 10.1016/j.freeradbiomed.2007.07.019

Sanganahalli BG, 2013, J CEREBR BLOOD F MET, V33, P1115, DOI 10.1038/jcbfm.2013.61

Sato K, 2011, J PHYSIOL-LONDON, V589, P2847, DOI 10.1113/jphysiol.2010.204461

Scheffer IE, 2017, EPILEPSIA, V58, P512, DOI 10.1111/epi.13709

Seo DY, 2019, INT NEUROUROL J, V23, pS82

Sevigny J, 2016, NATURE, V537, P50, DOI 10.1038/nature19323

Sheoran S, 2023, GEROSCIENCE, V45, P1837, DOI 10.1007/s11357-023-00732-6

Sleiman SF, 2016, ELIFE, V5, DOI 10.7554/eLife.15092

Song XH, 2020, FRONT NEUROSCI-SWITZ, V14, DOI 10.3389/fnins.2020.00267

Steen E, 2005, J ALZHEIMERS DIS, V7, P63, DOI 10.3233/jad-2005-7107

Subramaniam SR, 2013, PROG NEUROBIOL, V106, P17, DOI 10.1016/j.pneurobio.2013.04.004

Thakur S, 2023, INFLAMMATION, V46, P1, DOI 10.1007/s10753-022-01721-1

Thirupathi A, 2024, NEUROCHEM RES, V49, P1643, DOI 10.1007/s11064-024-04152-6

Tönnies E, 2017, J ALZHEIMERS DIS, V57, P1105, DOI 10.3233/JAD-161088

Tuon T, 2014, BRAIN RES BULL, V108, P106, DOI 10.1016/j.brainresbull.2014.09.006

Tuon T, 2012, NEUROSCIENCE, V227, P305, DOI 10.1016/j.neuroscience.2012.09.063

Tuon T, 2015, OXID MED CELL LONGEV, V2015, DOI 10.1155/2015/261809

Vancini RL, 2010, EPILEPSY BEHAV, V19, P504, DOI 10.1016/j.yebeh.2010.09.007

Vezzani A, 2005, EPILEPSIA, V46, P1724, DOI 10.1111/j.1528-1167.2005.00298.x

Vezzani A, 2011, NAT REV NEUROL, V7, P31, DOI 10.1038/nrneurol.2010.178

Vilela TC, 2020, NEURAL REGEN RES, V15, P1981, DOI 10.4103/1673-5374.282237

Vilela TC, 2017, MOL NEUROBIOL, V54, P7928, DOI 10.1007/s12035-016-0272-x

Vissing J, 1996, J CEREBR BLOOD F MET, V16, P729, DOI 10.1097/00004647-199607000-00025

Wang R, 2020, SCI CHINA LIFE SCI, V63, P1850, DOI 10.1007/s11427-020-1756-9

Wu Y, 2015, NEUROSCIENCE, V287, P175, DOI 10.1016/j.neuroscience.2014.06.046

Wu ZR, 2021, P NATL ACAD SCI USA, V118, DOI 10.1073/pnas.2100986118

Yan SK, 2023, FOODS, V12, DOI 10.3390/foods12203800

Yang Y, 2016, PARKINSONS DIS-US, V2016, DOI 10.1155/2016/6734678

Yao W, 2021, TRANSL PSYCHIAT, V11, DOI 10.1038/s41398-021-01261-6

Ye H, 2023, ANNU REV PATHOL-MECH, V18, P95, DOI 10.1146/annurev-pathmechdis-031521-034145

Yu F, 2021, J ALZHEIMERS DIS, V80, P233, DOI 10.3233/JAD-201100

Zhang JM, 2007, INT ANESTHESIOL CLIN, V45, P27, DOI 10.1097/AIA.0b013e318034194e

NR 117

TC 0

Z9 0

U1 8

U2 8

PU ASSOC BRAS DIVULG CIENTIFICA

PI RIBEIRAO PRETO

PA FACULDADE MEDICINA, CASA 10, 14049 RIBEIRAO PRETO, RIBEIRAO PRETO, SP

14049, BRAZIL

SN 0100-879X

EI 1414-431X

J9 BRAZ J MED BIOL RES

JI Brazilian J. Med. Biol. Res.

PY 2024

VL 57

AR e14094

DI 10.1590/1414-431X2024e14094

PG 12

WC Biology; Medicine, Research & Experimental

WE Science Citation Index Expanded (SCI-EXPANDED)

SC Life Sciences & Biomedicine - Other Topics; Research & Experimental

Medicine

GA O2U2R

UT WOS:001369736700001

PM 39607205

OA gold

DA 2025-04-09

ER

PT J

AU Zwierello, W

Maruszewska, A

Skorka-Majewicz, M

Gutowska, I

AF Zwierello, Wojciech

Maruszewska, Agnieszka

Skorka-Majewicz, Marta

Gutowska, Izabela

TI Fluoride in the Central Nervous System and Its Potential Influence on

the Development and Invasiveness of Brain Tumours-A Research Hypothesis

SO INTERNATIONAL JOURNAL OF MOLECULAR SCIENCES

LA English

DT Review

DE fluoride; brain tumour; glioblastoma; invasiveness; multidrug

resistance; environmental pollution

ID NF-KAPPA-B; HEDGEHOG SIGNALING PATHWAY; MESSENGER-RNA EXPRESSION;

GLIOMA-CELL MIGRATION; GLIOBLASTOMA-MULTIFORME; TNF-ALPHA; CHRONIC

FLUOROSIS; BREAST-CANCER; TEMOZOLOMIDE RESISTANCE; MAMMALIAN TARGETS

AB The purpose of this review is to attempt to outline the potential role of fluoride in the pathogenesis of brain tumours, including glioblastoma (GBM). In this paper, we show for the first time that fluoride can potentially affect the generally accepted signalling pathways implicated in the formation and clinical course of GBM. Fluorine compounds easily cross the blood-brain barrier. Enhanced oxidative stress, disruption of multiple cellular pathways, and microglial activation are just a few examples of recent reports on the role of fluoride in the central nervous system (CNS). We sought to present the key mechanisms underlying the development and invasiveness of GBM, as well as evidence on the current state of knowledge about the pleiotropic, direct, or indirect involvement of fluoride in the regulation of these mechanisms in various tissues, including neural and tumour tissue. The effects of fluoride on the human body are still a matter of controversy. However, given the growing incidence of brain tumours, especially in children, and numerous reports on the effects of fluoride on the CNS, it is worth taking a closer look at these mechanisms in the context of brain tumours, including gliomas.

C1 [Zwierello, Wojciech; Skorka-Majewicz, Marta; Gutowska, Izabela] Pomeranian Med Univ, Dept Med Chem, Powstancow Wlkp 71 St, PL-70111 Szczecin, Poland.

[Maruszewska, Agnieszka] Univ Szczecin, Inst Biol, Dept Physiol & Biochem, Felczaka 3c St, PL-71412 Szczecin, Poland.

[Maruszewska, Agnieszka] Univ Szczecin, Inst Biol, Mol Biol & Biotechnol Ctr, Waska 13 St, PL-71415 Szczecin, Poland.

C3 Pomeranian Medical University; University of Szczecin; University of

Szczecin

RP Zwierello, W; Gutowska, I (corresponding author), Pomeranian Med Univ, Dept Med Chem, Powstancow Wlkp 71 St, PL-70111 Szczecin, Poland.

EM wojciech.zwierello@pum.edu.pl; izabela.gutowska@pum.edu.pl

RI Gutowska, Izabela/I-3960-2014

OI Zwierello, Wojciech/0000-0001-5987-7120; Maruszewska,

Agnieszka/0000-0003-0687-9717

FU Department of Medical Chemistry Pomeranian Medical University in

Szczecin, Poland

FX This study was supported by the statutory budget of the Department of

Medical Chemistry Pomeranian Medical University in Szczecin, Poland.

CR Adedara IA, 2017, CAN J PHYSIOL PHARM, V95, P1019, DOI 10.1139/cjpp-2016-0641

Agalakova NI, 2020, CRIT REV TOXICOL, V50, P28, DOI 10.1080/10408444.2020.1722061

Aggarwal BB, 2003, NAT REV IMMUNOL, V3, P745, DOI 10.1038/nri1184

Al-Saleh I, 2001, BIOL TRACE ELEM RES, V79, P197, DOI 10.1385/BTER:79:3:197

Alfonso JCL, 2017, J R SOC INTERFACE, V14, DOI 10.1098/rsif.2017.0490

Allen EA, 2020, CELL DEATH DIFFER, V27, P903, DOI 10.1038/s41418-020-0497-0

García JA, 2016, METALLOMICS, V8, P1090, DOI 10.1039/c6mt00100a

ANDRASI E, 1993, SCI TOTAL ENVIRON, V140, P399, DOI 10.1016/0048-9697(93)90036-6

[Anonymous], 2019, Exposure to cadmium: A major public health concern

Arora A, 2019, CANCER BIOL THER, V20, P1083, DOI 10.1080/15384047.2019.1599662

Ashur-Fabian O, 2013, ANTI-CANCER DRUG, V24, P315, DOI 10.1097/CAD.0b013e32835c7a47

Aulestia FJ, 2020, SCI SIGNAL, V13, DOI 10.1126/scisignal.aay0086

Avci NG, 2020, SCI REP-UK, V10, DOI 10.1038/s41598-020-70392-5

Balkwill F, 2006, CANCER METAST REV, V25, P409, DOI 10.1007/s10555-006-9005-3

Bao ZY, 2019, TRANSL ONCOL, V12, P1155, DOI 10.1016/j.tranon.2019.04.016

Barrow-McGee R, 2016, NAT COMMUN, V7, DOI 10.1038/ncomms11942

Bartos M, 2019, INT J TOXICOL, V38, P405, DOI 10.1177/1091581819857558

Bashash M, 2017, ENVIRON HEALTH PERSP, V125, DOI 10.1289/EHP655

Basu Sayon, 2018, F1000Res, V7, DOI 10.12688/f1000research.15782.1

Bazzoni R, 2019, CANCERS, V11, DOI 10.3390/cancers11030292

Becker AP, 2021, CANCERS, V13, DOI 10.3390/cancers13040761

Beretta F, 2009, EUR J NEUROSCI, V30, P25, DOI 10.1111/j.1460-9568.2009.06804.x

Berghoff AS, 2020, EUR J CANCER, V135, P150, DOI 10.1016/j.ejca.2020.05.011

Bhat KPL, 2013, CANCER CELL, V24, P331, DOI 10.1016/j.ccr.2013.08.001

Binda E, 2017, CANCER RES, V77, P996, DOI 10.1158/0008-5472.CAN-16-1693

Biswas S, 2017, PHARMACOL THERAPEUT, V173, P118, DOI 10.1016/j.pharmthera.2017.02.011

Bombik E, 2020, ENVIRON MONIT ASSESS, V192, DOI 10.1007/s10661-020-8143-3

Broadbent JM, 2015, AM J PUBLIC HEALTH, V105, P72, DOI 10.2105/AJPH.2013.301857

Cao JL, 2016, CHEMOSPHERE, V161, P292, DOI 10.1016/j.chemosphere.2016.06.106

Cao K, 2019, ALZHEIMERS RES THER, V11, DOI 10.1186/s13195-019-0490-3

Carpenter RL, 2012, DISCOV MED, V13, P105

Chandra S, 2016, J NEURO-ONCOL, V127, P33, DOI 10.1007/s11060-015-2022-8

Chao M., 2020, FRONT IMMUNOL, V11, DOI [10.3389/fimmu.2020.592080, DOI 10.3389/FIMMU.2020.592080]

Chen JJ, 2016, AQUAT TOXICOL, V171, P48, DOI 10.1016/j.aquatox.2015.12.010

Chen LL, 2019, BIOL TRACE ELEM RES, V189, P157, DOI 10.1007/s12011-018-1458-z

Chen Q, 2010, INT J HYG ENVIR HEAL, V213, P381, DOI 10.1016/j.ijheh.2010.06.002

Chen R, 2017, INFLAMMATION, V40, P1123, DOI 10.1007/s10753-017-0556-y

Chen SX, 2013, BMC PUBLIC HEALTH, V13, DOI 10.1186/1471-2458-13-156

Chopra S, 2019, CELL MOL LIFE SCI, V76, P3083, DOI 10.1007/s00018-019-03171-9

Choudhry Z, 2014, ANN NEUROSCI, V21, P28, DOI 10.5214/ans.0972.7531.210109

Cilliers K, 2020, ANAT REC, V303, P1293, DOI 10.1002/ar.24254

Claus EB, 2015, NEUROSURG FOCUS, V38, DOI 10.3171/2014.10.FOCUS12367

Colman H, 2010, NEURO-ONCOLOGY, V12, P49, DOI 10.1093/neuonc/nop007

Coniglio S, 2016, JOVE-J VIS EXP, DOI 10.3791/53990

Coombs MRP, 2015, EXP MOL PATHOL, V99, P262, DOI 10.1016/j.yexmp.2015.07.008

Cuddapah VA, 2014, NAT REV NEUROSCI, V15, P455, DOI 10.1038/nrn3765

Cuny E, 2002, J NEUROSURG, V96, P294, DOI 10.3171/jns.2002.96.2.0294

D'Amico M, 2022, CANCER DRUG RESIST, V5, P939, DOI 10.20517/cdr.2022.46

Davis PJ, 2014, ONCOTARGETS THER, V7, P1619, DOI 10.2147/OTT.S67393

de Groot J, 2011, GLIA, V59, P1181, DOI 10.1002/glia.21113

Dec K, 2020, BIOMOLECULES, V10, DOI 10.3390/biom10030422

Deng CN, 2021, J ORTHOP SURG RES, V16, DOI 10.1186/s13018-021-02287-8

Deng HD, 2017, ONCOTARGET, V8, P114428, DOI 10.18632/oncotarget.22826

Doheny D, 2020, ONCOGENE, V39, P6589, DOI 10.1038/s41388-020-01454-1

Farmus L, 2021, ENVIRON RES, V200, DOI 10.1016/j.envres.2021.111315

Feldheim J, 2019, CANCERS, V11, DOI 10.3390/cancers11121837

Fianco G, 2017, ELIFE, V6, DOI 10.7554/eLife.22593

Florianczyk B., 2007, Journal of Pre-Clinical and Clinical Research, V1, P89

Fluegge K, 2016, J WATER HEALTH, V14, P864, DOI 10.2166/wh.2016.012

FOURNIER T, 1995, J IMMUNOL, V155, P2123

Fraser J, 2017, ESSAYS BIOCHEM, V61, P597, DOI 10.1042/EBC20170091

Friedmann-Morvinski D, 2016, SCI ADV, V2, DOI 10.1126/sciadv.1501292

Ghanbarian M, 2022, ENVIRON GEOCHEM HLTH, V44, P771, DOI 10.1007/s10653-021-00982-3

Ghosh D, 2020, INT J PHARM SCI RES, V11, P2011, DOI 10.13040/IJPSR.0975-8232.11(5).2011-17

Godlewski J, 2010, CELL CYCLE, V9, P2742, DOI 10.4161/cc.9.14.12248

Godlewski J, 2010, MOL CELL, V37, P620, DOI 10.1016/j.molcel.2010.02.018

Gong YY, 2016, NEURO-ONCOLOGY, V18, P48, DOI 10.1093/neuonc/nov096

Grandjean P, 2019, ENVIRON HEALTH-GLOB, V18, DOI 10.1186/s12940-019-0551-x

Gray GK, 2014, EXPERT REV NEUROTHER, V14, P1293, DOI 10.1586/14737175.2014.964211

Green R, 2019, JAMA PEDIATR, V173, P940, DOI 10.1001/jamapediatrics.2019.1729

Gu XL, 2016, ONCOTARGET, V7, P65218, DOI 10.18632/oncotarget.11573

Guan ZZ, 1998, NEUROTOXICOL TERATOL, V20, P537, DOI 10.1016/S0892-0362(97)00136-0

Guo Q, 2020, CHEMOSPHERE, V241, DOI 10.1016/j.chemosphere.2019.124861

Gupte A, 2009, CANCER TREAT REV, V35, P32, DOI 10.1016/j.ctrv.2008.07.004

Guth S, 2020, ARCH TOXICOL, V94, P1375, DOI 10.1007/s00204-020-02725-2

Gutowska I, 2015, TOXICOL IN VITRO, V29, P1661, DOI 10.1016/j.tiv.2015.06.024

Habib A, 2022, STEM CELL REV REP, V18, P691, DOI 10.1007/s12015-021-10297-6

Hadler-Olsen E, 2013, TUMOR BIOL, V34, P2041, DOI 10.1007/s13277-013-0842-8

Hakuno F, 2018, J MOL ENDOCRINOL, V61, pT69, DOI 10.1530/JME-17-0311

Han HJ, 2015, CHEMOSPHERE, V135, P297, DOI 10.1016/j.chemosphere.2015.04.012

Han N, 2017, ONCOTARGET, V8, P88059, DOI 10.18632/oncotarget.21409

Höring E, 2012, ACTA NEUROPATHOL, V124, P83, DOI 10.1007/s00401-011-0940-x

Hu CY, 2012, BIOL TRACE ELEM RES, V150, P297, DOI 10.1007/s12011-012-9482-x

Iano FG, 2014, J FLUORINE CHEM, V168, P212, DOI 10.1016/j.jfluchem.2014.09.029

Ichikawa T, 2016, NEUROL MED-CHIR, V56, P387, DOI 10.2176/nmc.ra.2016-0077

Inda MD, 2014, CANCERS, V6, P226, DOI 10.3390/cancers6010226

Jaudenes JR, 2020, APPL SCI-BASEL, V10, DOI 10.3390/app10186582

Jha SK, 2011, REV ENVIRON CONTAM T, V211, P121, DOI 10.1007/978-1-4419-8011-3_4

Ji M, 2018, ONCOTARGETS THER, V11, P3671, DOI 10.2147/OTT.S163535

Jiang CY, 2014, NEUROMOL MED, V16, P94, DOI 10.1007/s12017-013-8260-z

Jiang LL, 2013, PLOS ONE, V8, DOI 10.1371/journal.pone.0055527

Kahlert UD, 2012, CANCER LETT, V325, P42, DOI 10.1016/j.canlet.2012.05.024

Kamino M, 2011, CANCER SCI, V102, P540, DOI 10.1111/j.1349-7006.2010.01815.x

Kanamori M, 2007, J NEUROSURG, V106, P417, DOI 10.3171/jns.2007.106.3.417

Kanderi T., STATPEARLS

Katsuno Y, 2013, CURR OPIN ONCOL, V25, P76, DOI 10.1097/CCO.0b013e32835b6371

Kheradpisheh Z, 2018, SCI REP-UK, V8, DOI 10.1038/s41598-018-20696-4

Kim Y, 2021, ACTA NEUROPATHOL COM, V9, DOI 10.1186/s40478-021-01151-4

Koh I, 2018, SCI REP-UK, V8, DOI 10.1038/s41598-018-22681-3

Kuang P, 2018, AGING-US, V10, P1649, DOI 10.18632/aging.101499

Kuo HW, 2002, BIOL TRACE ELEM RES, V89, P1, DOI 10.1385/BTER:89:1:1

Kupnicka P, 2020, INT J MOL SCI, V21, DOI 10.3390/ijms21072361

Latour M, 2021, INT J MOL SCI, V22, DOI 10.3390/ijms22168428

Lee Y, 2016, LAB INVEST, V96, P137, DOI 10.1038/labinvest.2015.140

Lei Shuang, 2016, Shanghai Kou Qiang Yi Xue, V25, P426

Levy JMM, 2017, NAT REV CANCER, V17, P528, DOI 10.1038/nrc.2017.53

Li JL, 2016, J EXP CLIN CANC RES, V35, DOI 10.1186/s13046-016-0463-3

Li XY, 2019, MOL CELL BIOCHEM, V454, P77, DOI 10.1007/s11010-018-3454-1

Li YY, 2021, CHEMOSPHERE, V263, DOI 10.1016/j.chemosphere.2020.128178

Liu H, 2014, INT J HYG ENVIR HEAL, V217, P413, DOI 10.1016/j.ijheh.2013.08.001

Liu PH, 2021, ECOTOX ENVIRON SAFE, V222, DOI 10.1016/j.ecoenv.2021.112506

Liu TR, 2018, NAT COMMUN, V9, DOI 10.1038/s41467-018-05982-z

Liu XL, 2015, J HUAZHONG U SCI-MED, V35, P712, DOI 10.1007/s11596-015-1495-1

Liu XL, 2012, BIOL TRACE ELEM RES, V148, P117, DOI 10.1007/s12011-012-9333-9

Liu YJ, 2010, TOXICOL LETT, V192, P324, DOI 10.1016/j.toxlet.2009.11.002

Lobo JGVM, 2015, J DENT RES, V94, P990, DOI 10.1177/0022034515581186

Lombarte M, 2013, J ENDOCRINOL, V218, P99, DOI 10.1530/JOE-13-0067

Lopes GO, 2020, INT J MOL SCI, V21, DOI 10.3390/ijms21197297

Lukomska A, 2021, INT J MOL SCI, V22, DOI 10.3390/ijms22010391

Luo KK, 2021, TOXICOL SCI, V182, P275, DOI 10.1093/toxsci/kfab054

Luo Ping-Ping, 2018, Shanghai Kou Qiang Yi Xue, V27, P22

Luo Q, 2017, ONCOTARGET, V8, P80192, DOI 10.18632/oncotarget.19006

Lupo M, 2011, BIOL TRACE ELEM RES, V140, P198, DOI 10.1007/s12011-010-8690-5

Lütfioglu M, 2012, CLIN ORAL INVEST, V16, P1563, DOI 10.1007/s00784-011-0652-6

Ma L, 2021, CHEM-BIOL INTERACT, V349, DOI 10.1016/j.cbi.2021.109659

Malin AJ, 2015, ENVIRON HEALTH-GLOB, V14, DOI 10.1186/s12940-015-0003-1

Manini I, 2018, INT J MOL SCI, V19, DOI 10.3390/ijms19010147

Mathieu Patricia, 2013, Biomol Concepts, V4, P465, DOI 10.1515/bmc-2013-0006

McFarland BC, 2013, MOL CANCER RES, V11, P494, DOI 10.1158/1541-7786.MCR-12-0528

Medjedovic Eida, 2015, Mater Sociomed, V27, P395, DOI 10.5455/msm.2015.27.395-398

Moeller LC, 2005, MOL ENDOCRINOL, V19, P2955, DOI 10.1210/me.2004-0542

Mori Y, 2015, GLIA, V63, P906, DOI 10.1002/glia.22792

Mu N, 2018, THERANOSTICS, V8, P1527, DOI 10.7150/thno.22699

Mulware Stephen Juma, 2013, J Biophys, V2013, P192026, DOI 10.1155/2013/192026

Nabors LLB, 2003, CANCER RES, V63, P4181

Nadei OV, 2020, BIOL TRACE ELEM RES, V197, P495, DOI 10.1007/s12011-019-01993-z

Nagendra AH, 2021, MOL BIOL REP, V48, P5661, DOI 10.1007/s11033-021-06523-6

Nakamoto T, 2018, J CLIN PEDIATR DENT, V42, P325, DOI 10.17796/1053-4625-42.5.1

Nauman P, 2015, ENDOKRYNOL POL, V66, P444, DOI 10.5603/EP.2015.0055

Niu Q, 2018, ENVIRON POLLUT, V233, P889, DOI 10.1016/j.envpol.2017.09.015

Nogueira L, 2011, ONCOTARGET, V2, P646, DOI 10.18632/oncotarget.322

O'Mullane DM, 2016, COMMUNITY DENT HLTH, V33, P69, DOI 10.1922/CDH_3707O'Mullane31

Opydo-Szymaczek J, 2007, FLUORIDE, V40, P46

Ostrakhovitch EA, 2004, ARCH BIOCHEM BIOPHYS, V423, P351, DOI 10.1016/j.abb.2004.01.004

Ostrom QT, 2018, NEURO-ONCOLOGY, V20, P1, DOI 10.1093/neuonc/noy131

Ostrom QT, 2016, NEURO-ONCOLOGY, V18, P1, DOI 10.1093/neuonc/nov297

Ouyang T, 2021, ENVIRON TOXICOL, V36, P1817, DOI 10.1002/tox.23302

Paganoni R, 2021, INT J MOL SCI, V22, DOI 10.3390/ijms22084097

Pain G., 2018, FLUORIDE CAUSES DIAB

Pan XL, 2020, CHEM-BIOL INTERACT, V315, DOI 10.1016/j.cbi.2019.108875

Patel AP, 2014, SCIENCE, V344, P1396, DOI 10.1126/science.1254257

Patel S, 2014, CHILD NERV SYST, V30, P147, DOI 10.1007/s00381-013-2307-1

Paw I, 2015, CANCER LETT, V362, P1, DOI 10.1016/j.canlet.2015.03.015

Peng L, 2011, EUR J ORAL SCI, V119, P41, DOI 10.1111/j.1600-0722.2011.00880.x

Phipps O, 2021, NUTR REV, V79, P88, DOI 10.1093/nutrit/nuaa040

Piao YJ, 2009, NEURO-ONCOLOGY, V11, P260, DOI 10.1215/15228517-2008-094

Pietrobono S, 2019, FRONT GENET, V10, DOI 10.3389/fgene.2019.00556

Prionisti I, 2019, FRONT PHARMACOL, V10, DOI 10.3389/fphar.2019.00506

Qiao LC, 2021, INT J MOL SCI, V22, DOI 10.3390/ijms222111932

Quadri JA, 2018, TOXICOLOGY, V406, P44, DOI 10.1016/j.tox.2018.05.012

Raaschou-Nielsen O, 2006, BRIT J CANCER, V95, P416, DOI 10.1038/sj.bjc.6603278

Raina R, 2015, BIOL TRACE ELEM RES, V166, P157, DOI 10.1007/s12011-015-0263-1

Rao TP, 2010, CIRC RES, V106, P1798, DOI 10.1161/CIRCRESAHA.110.219840

Ravanan P, 2017, LIFE SCI, V188, P53, DOI 10.1016/j.lfs.2017.08.029

Raychaudhuri B, 2007, J NEURO-ONCOL, V85, P39, DOI 10.1007/s11060-007-9390-7

Razaghi A, 2021, EUR J CANCER, V155, P256, DOI 10.1016/j.ejca.2021.07.013

Reddy PY., 2011, J. Med. Allied Sci, V1, P30

Refsnes M, 2014, J INFLAMM RES, V7, P169, DOI 10.2147/JIR.S69646

Riddell JK, 2021, INT J ENV RES PUB HE, V18, DOI 10.3390/ijerph18126203

Ritchie CK, 2000, J CELL PHYSIOL, V184, P214, DOI 10.1002/1097-4652(200008)184:2<214::AID-JCP9>3.0.CO;2-Z

Rodón L, 2014, CANCER DISCOV, V4, P1230, DOI 10.1158/2159-8290.CD-14-0275

Rogalska A, 2017, NEUROTOX RES, V31, P436, DOI 10.1007/s12640-017-9709-x

Roomi MW, 2017, ONCOL REP, V37, P1907, DOI 10.3892/or.2017.5391

Russ TC, 2020, BRIT J PSYCHIAT, V216, P29, DOI 10.1192/bjp.2018.287

Ryu J, 2011, ANTICANCER RES, V31, P4223

Saeed M, 2020, ENVIRON SCI POLLUT R, V27, P2566, DOI 10.1007/s11356-019-06938-6

SAKUMA S, 1993, J NEURO-ONCOL, V15, P197, DOI 10.1007/BF01050066

Salmaggi A, 2021, J PERS MED, V11, DOI 10.3390/jpm11050390

Sana S, 2017, INT J NANOMED, V12, P4059, DOI 10.2147/IJN.S124119

Sarkar S, 2009, J NEURO-ONCOL, V91, P157, DOI 10.1007/s11060-008-9695-1

Schiera G, 2021, CANCERS, V13, DOI 10.3390/cancers13112693

Schlenska-Lange A, 2008, ANTICANCER RES, V28, P1055

Semenov MV, 2007, CELL, V131, DOI 10.1016/j.cell.2007.12.011

Serna J, 2020, NUTRIENTS, V12, DOI 10.3390/nu12103001

Sharma V, 2011, NEUROCHEM INT, V59, P567, DOI 10.1016/j.neuint.2011.06.018

Shashi A., 2013, World Journal of Medical Sciences, V8, P67

Shen QF, 2019, ARCH MED SCI, V15, P457, DOI 10.5114/aoms.2019.83294

Shusterman K, 2014, CELLS TISSUES ORGANS, V199, P159, DOI 10.1159/000367840

Sinceviciute R, 2018, INT J CLIN EXP PATHO, V11, P3010

Singh N, 2014, SPRINGERPLUS, V3, DOI 10.1186/2193-1801-3-7

Singh N, 2021, CANCER DRUG RESIST, V4, P17, DOI 10.20517/cdr.2020.79

Sirkisoon SR, 2020, ONCOGENE, V39, P64, DOI 10.1038/s41388-019-0959-3

Slompo Camila, 2012, Braz. Dent. J., V23, P629

Smith MA, 1998, J NATL CANCER I, V90, P1269, DOI 10.1093/jnci/90.17.1269

Sohrabi M, 2018, BIOL TRACE ELEM RES, V183, P1, DOI 10.1007/s12011-017-1099-7

Song C, 2017, SCI REP-UK, V7, DOI 10.1038/s41598-017-00796-3

Soubannier V, 2017, BIOMEDICINES, V5, DOI 10.3390/biomedicines5020029

Stachowska E, 2005, FLUORIDE, V38, P297

Stepien M, 2017, CARCINOGENESIS, V38, P699, DOI 10.1093/carcin/bgx051

Strazielle N, 2013, MOL PHARMACEUT, V10, P1473, DOI 10.1021/mp300518e

Strunecka A, 2020, APPL SCI-BASEL, V10, DOI 10.3390/app10207100

Stupp R, 2005, NEW ENGL J MED, V352, P987, DOI 10.1056/NEJMoa043330

Sun J, 2006, AM J PHYSIOL-ENDOC M, V291, pE315, DOI 10.1152/ajpendo.00590.2005

Sun SC, 2011, CELL RES, V21, P71, DOI 10.1038/cr.2010.177

Sun Z, 2018, HUM EXP TOXICOL, V37, P87, DOI 10.1177/0960327117693067

Suzuki M, 2014, J DENT RES, V93, P1022, DOI 10.1177/0022034514545629

Suzuki M, 2014, BBA-MOL BASIS DIS, V1842, P245, DOI 10.1016/j.bbadis.2013.11.023

Takebe N, 2014, PHARMACOL THERAPEUT, V141, P140, DOI 10.1016/j.pharmthera.2013.09.005

Tang Sha, 2017, Wei Sheng Yan Jiu, V46, P472

Tian XL, 2020, ARCH TOXICOL, V94, P749, DOI 10.1007/s00204-019-02651-y

Tian YH, 2016, CHEMOSPHERE, V161, P89, DOI 10.1016/j.chemosphere.2016.06.035

Till C, 2021, PEDIATR RES, V90, P1093, DOI 10.1038/s41390-020-0973-8

Tirrò E, 2021, FRONT ONCOL, V10, DOI 10.3389/fonc.2020.612385

Tiwari S, 2004, CALCIFIED TISSUE INT, V75, P313, DOI 10.1007/s00223-004-0225-7

Torti SV, 2018, ANNU REV NUTR, V38, P97, DOI 10.1146/annurev-nutr-082117-051732

Trevizol JS, 2020, CHEMOSPHERE, V254, DOI 10.1016/j.chemosphere.2020.126602

TRIVEDI N, 1993, DIABETOLOGIA, V36, P826, DOI 10.1007/BF00400357

Tu W, 2018, TOXICOL APPL PHARM, V347, P60, DOI 10.1016/j.taap.2018.03.030

Turner CH, 1997, CALCIFIED TISSUE INT, V61, P77, DOI 10.1007/s002239900299

ULLER RP, 1973, J CLIN ENDOCR METAB, V37, P741, DOI 10.1210/jcem-37-5-741

Urut F, 2021, BIOL TRACE ELEM RES, V199, P3700, DOI 10.1007/s12011-020-02491-3

Verma RJ, 2002, FOOD CHEM TOXICOL, V40, P1781, DOI 10.1016/S0278-6915(02)00170-9

Vithanage M, 2015, ENVIRON CHEM LETT, V13, P131, DOI 10.1007/s10311-015-0496-4

Wandzilak A, 2015, SPECTROCHIM ACTA B, V114, P52, DOI 10.1016/j.sab.2015.10.002

Wang HW, 2017, BIOL TRACE ELEM RES, V178, P253, DOI 10.1007/s12011-016-0929-3

Wang JX, 2021, SCI TOTAL ENVIRON, V760, DOI 10.1016/j.scitotenv.2020.143376

Wang K, 2017, CANCER CELL INT, V17, DOI 10.1186/s12935-017-0491-x

Wang K, 2010, NEUROL RES, V32, P975, DOI 10.1179/016164110X12681290831360

Wang XY, 2017, TOXICOL IN VITRO, V44, P57, DOI 10.1016/j.tiv.2017.06.026

Wang X, 2015, ONCOL LETT, V9, P2586, DOI 10.3892/ol.2015.3130

Wang YJ, 2021, ECOTOX ENVIRON SAFE, V217, DOI 10.1016/j.ecoenv.2021.112225

Wang Y, 2017, BIOL TRACE ELEM RES, V176, P367, DOI 10.1007/s12011-016-0833-x

Wang YQ, 2003, ENDOCRINOLOGY, V144, P2164, DOI 10.1210/en.2002-220740

White E, 2015, J CLIN INVEST, V125, P42, DOI 10.1172/JCI73941

Wickström M, 2015, NAT COMMUN, V6, DOI 10.1038/ncomms9904

World Health Organization (WHO), 2021, CureAll framework: WHO global initiative for childhood cancer: increasing access, advancing quality, saving lives

Wu CX, 2006, FLUORIDE, V39, P274

Xi SH, 2012, MEDIAT INFLAMM, V2012, DOI 10.1155/2012/102954

Xie JW, 2001, P NATL ACAD SCI USA, V98, P9255, DOI 10.1073/pnas.151173398

Xing ZY, 2015, CLIN NEUROL NEUROSUR, V131, P54, DOI 10.1016/j.clineuro.2015.01.018

Xu BY, 2011, ENVIRON TOXICOL, V26, P86, DOI 10.1002/tox.20543

Xu L, 2021, FRONT PHYSIOL, V12, DOI 10.3389/fphys.2021.603848

Yamamoto K, 2020, NATURE, V581, P100, DOI 10.1038/s41586-020-2229-5

Yamini B, 2018, CELLS-BASEL, V7, DOI 10.3390/cells7090125

Yan L, 2013, MEDIAT INFLAMM, V2013, DOI 10.1155/2013/895975

Yan N, 2016, MOL NEUROBIOL, V53, P4449, DOI 10.1007/s12035-015-9380-2

Yang C, 2017, PLOS ONE, V12, DOI 10.1371/journal.pone.0170674

Yang CY, 2020, FRONT ONCOL, V10, DOI 10.3389/fonc.2020.590931

Yang L, 2018, NEUROTOXICOLOGY, V69, P108, DOI 10.1016/j.neuro.2018.09.006

Ye XZ, 2012, J IMMUNOL, V189, P444, DOI 10.4049/jimmunol.1103248

Yeung YT, 2013, BRIT J PHARMACOL, V168, P591, DOI 10.1111/bph.12008

Yeung YT, 2012, J NEURO-ONCOL, V109, P35, DOI 10.1007/s11060-012-0875-7

Yi GZ, 2019, BRAIN, V142, P2352, DOI 10.1093/brain/awz202

Yu X, 2019, LIFE SCI, V236, DOI 10.1016/j.lfs.2019.116917

Yuzhalin AE, 2014, INTERLEUKINS IN CANCER BIOLOGY: THEIR HETEROGENEOUS ROLE, P1

Zeng Qi-bing, 2019, Yingyong Shengtai Xuebao, V30, P37, DOI 10.13287/j.1001-9332.201901.023

Zhang CZ, 2020, BIOL TRACE ELEM RES, V193, P502, DOI 10.1007/s12011-019-01735-1

Zhang H, 2019, ONCOL LETT, V17, P1826, DOI 10.3892/ol.2018.9806

Zhang JF, 2017, ONCOL REP, V38, P2033, DOI 10.3892/or.2017.5926

Zhang JH, 2017, J AGR FOOD CHEM, V65, P8966, DOI 10.1021/acs.jafc.7b03822

Zhang J, 2011, EXP TOXICOL PATHOL, V63, P407, DOI 10.1016/j.etp.2010.02.017

Zhang M, 2010, P NATL ACAD SCI USA, V107, P3522, DOI 10.1073/pnas.0910179107

Zhang M, 2008, TOXICOL LETT, V179, P1, DOI 10.1016/j.toxlet.2008.03.002

Zhang S, 2015, TOXICOL SCI, V144, P238, DOI 10.1093/toxsci/kfu311

Zhang XH, 2012, CANCER SCI, V103, P181, DOI 10.1111/j.1349-7006.2011.02154.x

Zhang Xueli, 2012, Hua Xi Kou Qiang Yi Xue Za Zhi, V30, P434

Zhao LN, 2014, TOXICOL LETT, V225, P318, DOI 10.1016/j.toxlet.2013.12.022

Zhao YF, 2018, ARCH TOXICOL, V92, P3277, DOI 10.1007/s00204-018-2305-x

Zhao YF, 2018, FOOD CHEM TOXICOL, V115, P26, DOI 10.1016/j.fct.2018.02.065

Zhou GY, 2019, TOXICOL APPL PHARM, V378, DOI 10.1016/j.taap.2019.114608

Zhou W, 2019, BIOMED PHARMACOTHER, V118, DOI 10.1016/j.biopha.2019.109369

Zhu H, 2014, CANCER LETT, V343, P51, DOI 10.1016/j.canlet.2013.09.014

Zhu H, 2010, CURR GENOMICS, V11, P238, DOI 10.2174/138920210791233108

[朱志坚 Zhu Zhijian], 2018, [中国公共卫生, China Journal of Public Health], V34, P241

Zhuang GS, 1996, BIOL TRACE ELEM RES, V53, P45, DOI 10.1007/BF02784543

NR 275

TC 16

Z9 16

U1 3

U2 12

PU MDPI

PI BASEL

PA ST ALBAN-ANLAGE 66, CH-4052 BASEL, SWITZERLAND

SN 1661-6596

EI 1422-0067

J9 INT J MOL SCI

JI Int. J. Mol. Sci.

PD JAN

PY 2023

VL 24

IS 2

AR 1558

DI 10.3390/ijms24021558

PG 28

WC Biochemistry & Molecular Biology; Chemistry, Multidisciplinary

WE Science Citation Index Expanded (SCI-EXPANDED)

SC Biochemistry & Molecular Biology; Chemistry

GA 8C5HS

UT WOS:000917639800001

PM 36675073

OA Green Published, gold

DA 2025-04-09

ER

PT J

AU Dong, JH

Qian, YM

Zhang, W

Xu, JY

Wang, LP

Fan, ZW

Jia, MX

Wei, LJ

Yang, H

Luo, X

Wang, YJ

Jiang, YY

Huang, ZH

Wang, Y

AF Dong, Jianhong

Qian, Yiming

Zhang, Wei

Xu, Jiayun

Wang, Lipei

Fan, Ziwei

Jia, Mengxian

Wei, Lijia

Yang, Hui

Luo, Xuan

Wang, Yongjie

Jiang, Yuanyuan

Huang, Zhihui

Wang, Ying

TI Tenacissoside H repressed the progression of glioblastoma by inhibiting

the PI3K/Akt/mTOR signaling pathway

SO EUROPEAN JOURNAL OF PHARMACOLOGY

LA English

DT Article

DE Apoptosis; Glioblastoma; Migration; mTOR; Proliferation; Tenacissoside H

ID DRUG TARGET IDENTIFICATION; MARSDENIA-TENACISSIMA; OXIDATIVE STRESS; WEB

SERVER; PROMOTES; INJURY

AB Glioblastoma (GBM) is one of the most common intracranial primary malignancies with the highest mortality rate, and there is a lack of effective treatments. In this study, we examined the anti-GBM activity of Tenacissoside H (TH), an active component isolated from the traditional Chinese medicine Marsdenia tenacissima (Roxb.) Wight & Arn (MT), and investigated the potential mechanism. Firstly, we found that TH decreased the viability of GBM cells by inducing cell cycle arrest and apoptosis, and inhibited the migration of GBM cells. Furthermore, combined with the Gene Expression Omnibus database (GEO) and network pharmacology as well as molecular docking, TH was shown to inhibit GBM progression by directly regulating the PI3K/Akt/mTOR pathway, which was further validated in vitro. In addition, the selective PI3K agonist 740 y-p partially restored the inhibitory effects of TH on GBM cells. Finally, TH inhibited GBM progression in an orthotopic transplantation model by inactivating the PI3K/Akt/mTOR pathway in vivo. Conclusively, our results suggest that TH represses GBM progression by inhibiting the PI3K/Akt/mTOR signaling pathway in vitro and in vivo, and provides new insight for the treatment of GBM patients.

C1 [Dong, Jianhong; Qian, Yiming; Wang, Ying] Zhejiang Univ, Affiliated Hangzhou Peoples Hosp 1, Dept Clin Res Ctr, Sch Med, Hangzhou 310053, Zhejiang, Peoples R China.

[Dong, Jianhong; Qian, Yiming; Zhang, Wei; Xu, Jiayun; Wei, Lijia; Yang, Hui; Luo, Xuan; Wang, Yongjie; Jiang, Yuanyuan; Huang, Zhihui] Hangzhou Normal Univ, Sch Pharm, Hangzhou 311121, Zhejiang, Peoples R China.

[Dong, Jianhong; Qian, Yiming; Zhang, Wei; Xu, Jiayun; Wei, Lijia; Yang, Hui; Luo, Xuan; Wang, Yongjie; Jiang, Yuanyuan; Huang, Zhihui] Hangzhou Normal Univ, Key Lab Elemene Class Anticanc Chinese Med, Hangzhou 311121, Zhejiang, Peoples R China.

[Wang, Lipei] Hangzhou Normal Univ, Sch Basic Med Sci, Hangzhou 310030, Zhejiang, Peoples R China.

[Fan, Ziwei; Jia, Mengxian] Wenzhou Med Univ, Affiliated Hosp 1, Dept Orthoped Spine Surg, Wenzhou 325035, Zhejiang, Peoples R China.

[Huang, Zhihui] Hangzhou Normal Univ, Hangzhou, Peoples R China.

[Wang, Ying] Affiliated Hangzhou First Peoples Hosp, Hangzhou, Peoples R China.

C3 Zhejiang University; Hangzhou Normal University; Hangzhou Normal

University; Hangzhou Normal University; Wenzhou Medical University;

Hangzhou Normal University

RP Huang, ZH (corresponding author), Hangzhou Normal Univ, Hangzhou, Peoples R China.; Wang, Y (corresponding author), Affiliated Hangzhou First Peoples Hosp, Hangzhou, Peoples R China.

EM huang0069@hznu.edu.cn; nancywangying@163.com

RI Qian, Yiming/LUA-1368-2024; xu, jiayun/LVR-9624-2024; , Wang

Yongjie/JVZ-3756-2024

OI huang, zhihui/0000-0002-1927-0946; , Wang Yongjie/0000-0002-0306-3088

FU Ministry of Science and Technology China Brain Initiative Grant

(STI2030-Major Projects) [2022ZD0204700]

FX This work was supported by the Ministry of Science and Technology China

Brain Initiative Grant (STI2030-Major Projects, 2022ZD0204700) provided

funding for this research.

CR Arrieta VA, 2023, J CLIN INVEST, V133, DOI 10.1172/JCI163447

Banerji U, 2018, CLIN CANCER RES, V24, P2050, DOI 10.1158/1078-0432.CCR-17-2260

Berger TR, 2022, JAMA ONCOL, V8, P1493, DOI 10.1001/jamaoncol.2022.2844

Butowski N, 2016, NEURO-ONCOLOGY, V18, P557, DOI 10.1093/neuonc/nov245

Daina A, 2019, NUCLEIC ACIDS RES, V47, pW357, DOI 10.1093/nar/gkz382

Fruman DA, 2017, CELL, V170, P605, DOI 10.1016/j.cell.2017.07.029

Haider SA, 2020, NEUROLOGY, V95, pE1575, DOI 10.1212/WNL.0000000000010263

Hong ZS, 2020, EVID-BASED COMPL ALT, V2020, DOI 10.1155/2020/2824984

Hsieh AC, 2012, NATURE, V485, P55, DOI 10.1038/nature10912

Huang DW, 2009, NAT PROTOC, V4, P44, DOI 10.1038/nprot.2008.211

Jia YS, 2015, EVID-BASED COMPL ALT, V2015, DOI 10.1155/2015/464937

Jiang S, 2016, CARBOHYD POLYM, V137, P52, DOI 10.1016/j.carbpol.2015.10.056

KOYAMA S, 1993, CELL, V72, P945, DOI 10.1016/0092-8674(93)90582-B

Kundu M, 2019, PHYTOTHER RES, V33, P2571, DOI 10.1002/ptr.6426

Lambert AW, 2017, CELL, V168, P670, DOI 10.1016/j.cell.2016.11.037

Lawrence MS, 2014, NATURE, V505, P495, DOI 10.1038/nature12912

Lin JT, 2021, DOSE-RESPONSE, V19, DOI 10.1177/15593258211011023

Liu XF, 2010, NUCLEIC ACIDS RES, V38, pW609, DOI 10.1093/nar/gkq300

Louis DN, 2021, NEURO-ONCOLOGY, V23, P1231, DOI 10.1093/neuonc/noab106

Manning BD, 2017, CELL, V169, P381, DOI 10.1016/j.cell.2017.04.001

Martina JA, 2012, AUTOPHAGY, V8, P903, DOI 10.4161/auto.19653

McCormack F.X., N. Engl. J. Med., V364, P1595

Menon S, 2008, Oncogene, V27 Suppl 2, pS43, DOI 10.1038/onc.2009.352

Ostrom QT, 2023, NEURO-ONCOLOGY, V25, DOI 10.1093/neuonc/noad149

Ostrom QT, 2015, NEURO-ONCOLOGY, V16, P1, DOI [10.1093/neuonc/nou327, 10.1093/neuonc/nou223, 10.1093/neuonc/nov189, 10.1093/neuonc/nox158]

Peeters MCM, 2020, J NEURO-ONCOL, V146, P293, DOI 10.1007/s11060-019-03373-y

Robitaille AM, 2013, SCIENCE, V339, P1320, DOI 10.1126/science.1228771

Sherman BT, 2022, NUCLEIC ACIDS RES, V50, pW216, DOI 10.1093/nar/gkac194

Shi MZ, 2019, J PHARMACEUT BIOMED, V174, P728, DOI 10.1016/j.jpba.2019.07.003

Sun ZQ, 2020, J ALZHEIMERS DIS, V76, P1513, DOI 10.3233/JAD-191032

Tan AC, 2020, CA-CANCER J CLIN, V70, P299, DOI 10.3322/caac.21613

Wang PL, 2018, AM J CHINESE MED, V46, P1449, DOI 10.1142/S0192415X18500751

Wang X, 2017, NUCLEIC ACIDS RES, V45, pW356, DOI 10.1093/nar/gkx374

Wick W, 2017, NEW ENGL J MED, V377, P1954, DOI 10.1056/NEJMoa1707358

Wick W, 2016, CLIN CANCER RES, V22, P4797, DOI 10.1158/1078-0432.CCR-15-3153

Wu ZL, 2022, J ETHNOPHARMACOL, V294, DOI 10.1016/j.jep.2022.115353

Xie B, 2019, J ETHNOPHARMACOL, V235, P309, DOI 10.1016/j.jep.2019.02.028

Xie CN, 2020, J NEUROSCI, V40, P2644, DOI 10.1523/JNEUROSCI.2229-19.2020

Yang M, 2021, FRONT PHARMACOL, V12, DOI 10.3389/fphar.2021.666368

Ye BG, 2014, PLANTA MED, V80, P29, DOI 10.1055/s-0033-1360128

Zhang R, 2021, CLIN EXP PHARMACOL P, V48, P757, DOI [10.1111/1440-1681.13398, 10.1080/09588221.2021.1888752, 10.1080/09588221.2021.1880441]

Zhang YQ, 2017, CANCER CELL, V31, P820, DOI 10.1016/j.ccell.2017.04.013

Zhou XQ, 2022, FRONT PHARMACOL, V13, DOI 10.3389/fphar.2022.1023314

Zou J, 2018, J CELL MOL MED, V22, P2692, DOI 10.1111/jcmm.13552

NR 44

TC 0

Z9 0

U1 2

U2 9

PU ELSEVIER

PI AMSTERDAM

PA RADARWEG 29, 1043 NX AMSTERDAM, NETHERLANDS

SN 0014-2999

EI 1879-0712

J9 EUR J PHARMACOL

JI Eur. J. Pharmacol.

PD APR 5

PY 2024

VL 968

AR 176401

DI 10.1016/j.ejphar.2024.176401

EA FEB 2024

PG 15

WC Pharmacology & Pharmacy

WE Science Citation Index Expanded (SCI-EXPANDED)

SC Pharmacology & Pharmacy

GA NF6N7

UT WOS:001199078700001

PM 38331340

DA 2025-04-09

ER

PT J

AU Solomou, G

Finch, A

Asghar, A

Bardella, C

AF Solomou, Georgios

Finch, Alina

Asghar, Asim

Bardella, Chiara

TI Mutant IDH in Gliomas: Role in Cancer and Treatment Options

SO CANCERS

LA English

DT Review

DE isocitrate dehydrogenase; cancer metabolism; hydroxyglutarate; gliomas;

oncometabolites

ID ISOCITRATE DEHYDROGENASE 1; ACUTE MYELOID-LEUKEMIA; INDUCED OXIDATIVE

STRESS; DNA-REPAIR ENZYMES; 2 MUTATIONS; ONCOMETABOLITE

2-HYDROXYGLUTARATE; INTRAHEPATIC CHOLANGIOCARCINOMAS; PROMOTES

DIFFERENTIATION; LACTATE-DEHYDROGENASE; HISTONE DEMETHYLATION

AB Altered metabolism is a common feature of many cancers and, in some cases, is a consequence of mutation in metabolic genes, such as the ones involved in the TCA cycle. Isocitrate dehydrogenase (IDH) is mutated in many gliomas and other cancers. Physiologically, IDH converts isocitrate to a-ketoglutarate (a-KG), but when mutated, IDH reduces a-KG to D2-hydroxyglutarate (D2-HG). D2-HG accumulates at elevated levels in IDH mutant tumours, and in the last decade, a massive effort has been made to develop small inhibitors targeting mutant IDH. In this review, we summarise the current knowledge about the cellular and molecular consequences of IDH mutations and the therapeutic approaches developed to target IDH mutant tumours, focusing on gliomas.

C1 [Solomou, Georgios; Finch, Alina; Asghar, Asim; Bardella, Chiara] Univ Birmingham, Inst Canc & Genom Sci, Coll Med & Dent Sci, Birmingham B15 2TT, England.

[Solomou, Georgios] Univ Cambridge, Dept Clin Neurosci, Div Acad Neurosurg, Cambridge CB2 0QQ, England.

[Solomou, Georgios] Univ Cambridge, Wellcome MRC Cambridge Stem Cell Inst, Cambridge CB2 0AW, England.

C3 University of Birmingham; University of Cambridge; University of

Cambridge

RP Bardella, C (corresponding author), Univ Birmingham, Inst Canc & Genom Sci, Coll Med & Dent Sci, Birmingham B15 2TT, England.

EM c.bardella@bham.ac.uk

RI bardella, chiara/AAG-6272-2021

OI Bardella, Chiara/0000-0002-7780-2541; Solomou,

Georgios/0000-0002-9795-0517

FU Royal Society [RGS\R2\212424]

FX C.B. thanks the Royal Society for funding (RGS\R2\212424).

CR Abbas S, 2010, BLOOD, V116, P2122, DOI 10.1182/blood-2009-11-250878

Al-Khallaf H, 2017, CELL BIOSCI, V7, DOI 10.1186/s13578-017-0165-3

Alston CL, 2012, J MED GENET, V49, P569, DOI 10.1136/jmedgenet-2012-101146

Amankulor NM, 2017, GENE DEV, V31, P774, DOI 10.1101/gad.294991.116

Amary MF, 2011, J PATHOL, V224, P334, DOI 10.1002/path.2913

Andronesi OC, 2018, NAT COMMUN, V9, DOI 10.1038/s41467-018-03905-6

Andronesi OC, 2012, SCI TRANSL MED, V4, DOI 10.1126/scitranslmed.3002693

Aptowitzer I, 1997, J PEDIATR GASTR NUTR, V24, P599, DOI 10.1097/00005176-199705000-00019

Arai M, 2012, BRAIN TUMOR PATHOL, V29, P201, DOI 10.1007/s10014-012-0085-1

Armstrong TS, 2016, NEURO-ONCOLOGY, V18, P779, DOI 10.1093/neuonc/nov269

Badur MG, 2018, CELL REP, V25, P1680, DOI 10.1016/j.celrep.2018.10.099

Bai H, 2016, NAT GENET, V48, P59, DOI 10.1038/ng.3457

Baldock AL, 2014, NEURO-ONCOLOGY, V16, P779, DOI 10.1093/neuonc/nou027

Balss J, 2008, ACTA NEUROPATHOL, V116, P597, DOI 10.1007/s00401-008-0455-2

Bardella C, 2016, CANCER CELL, V30, P578, DOI 10.1016/j.ccell.2016.08.017

BARNES LD, 1971, BIOCHEMISTRY-US, V10, P3939, DOI 10.1021/bi00797a022

Behrend L, 2003, BIOCHEM SOC T, V31, P1441

Bello L, 2004, CANC TREAT, V117, P263

Borger DR, 2012, ONCOLOGIST, V17, P72, DOI 10.1634/theoncologist.2011-0386

Borodovsky A, 2013, ONCOTARGET, V4, P1737, DOI 10.18632/oncotarget.1408

Borodovsky A, 2012, CURR OPIN ONCOL, V24, P83, DOI 10.1097/CCO.0b013e32834d816a

Burris H., 2015, MOL CANCER THER, V14, pPL04, DOI DOI 10.1158/1535-7163.TARG-15-PL04-05

Cai SJ, 2019, CELL BIOSCI, V9, DOI 10.1186/s13578-019-0309-8

Cairns RA, 2012, BLOOD, V119, P1901, DOI 10.1182/blood-2011-11-391748

Carrillo JA, 2012, AM J NEURORADIOL, V33, P1349, DOI 10.3174/ajnr.A2950

Chatterjee N, 2017, ENVIRON MOL MUTAGEN, V58, P235, DOI 10.1002/em.22087

Chaumeil MM, 2016, NEUROIMAGE-CLIN, V12, P180, DOI 10.1016/j.nicl.2016.06.018

Chen FY, 2017, CHEM RES TOXICOL, V30, P1102, DOI 10.1021/acs.chemrestox.7b00009

Chen H, 2017, NEUROLOGY, V88, P1805, DOI 10.1212/WNL.0000000000003911

Chen J, 2016, MINI-REV MED CHEM, V16, P1344, DOI 10.2174/1389557516666160609085520

Chesnelong C, 2014, NEURO-ONCOLOGY, V16, P686, DOI 10.1093/neuonc/not243

Chiang S, 2016, CANCER RES, V76, P7118, DOI 10.1158/0008-5472.CAN-16-0298

Cho YS, 2017, ACS MED CHEM LETT, V8, P1116, DOI 10.1021/acsmedchemlett.7b00342

Chowdhury R, 2011, EMBO REP, V12, P463, DOI 10.1038/embor.2011.43

Christensen BC, 2011, JNCI-J NATL CANCER I, V103, DOI 10.1093/jnci/djq497

Christians A, 2019, ACTA NEUROPATHOL COM, V7, DOI 10.1186/s40478-019-0817-0

Cleven AHG, 2017, CLIN SARCOMA RES, V7, DOI 10.1186/s13569-017-0074-6

Colvin H, 2016, SCI REP-UK, V6, DOI 10.1038/srep36289

Correia CE, 2021, NEURO-ONCOL ADV, V3, DOI 10.1093/noajnl/vdab146

Cui DM, 2016, INT J BIOCHEM CELL B, V73, P72, DOI 10.1016/j.biocel.2016.02.007

Dang L, 2009, NATURE, V462, P739, DOI 10.1038/nature08617

de la Fuente MI, 2023, NEURO-ONCOLOGY, V25, P146, DOI 10.1093/neuonc/noac139

Deng GJ, 2015, J BIOL CHEM, V290, P762, DOI 10.1074/jbc.M114.608497

Dhillon S, 2018, DRUGS, V78, P1509, DOI 10.1007/s40265-018-0978-3

DiNardo CD, 2018, NEW ENGL J MED, V378, P2386, DOI 10.1056/NEJMoa1716984

DiNardo CD, 2023, J CANCER RES CLIN, V149, P1145, DOI 10.1007/s00432-022-03983-6

Doherty JR, 2013, J CLIN INVEST, V123, P3685, DOI 10.1172/JCI69741

Doi A, 2009, NAT GENET, V41, P1350, DOI 10.1038/ng.471

Duncan CG, 2012, GENOME RES, V22, P2339, DOI 10.1101/gr.132738.111

Elhammali A, 2014, CANCER DISCOV, V4, P828, DOI 10.1158/2159-8290.CD-13-0572

Elpeleg ON, 1997, MUSCLE NERVE, V20, P238

Emadi A, 2014, EXP HEMATOL, V42, P247, DOI 10.1016/j.exphem.2013.12.001

Fack F, 2017, EMBO MOL MED, V9, P1681, DOI 10.15252/emmm.201707729

Fan B, 2020, INVEST NEW DRUG, V38, P433, DOI 10.1007/s10637-019-00771-x

Farshidfar F, 2017, CELL REP, V18, P2780, DOI [10.1016/j.celrep.2017.02.033, 10.1016/j.celrep.2017.06.008]

Fathi AT, 2014, ONCOLOGIST, V19, P602, DOI 10.1634/theoncologist.2013-0417

Fattal-Valevski A, 2017, NEUROGENETICS, V18, P57, DOI 10.1007/s10048-016-0507-z

Figueroa ME, 2010, CANCER CELL, V18, P553, DOI 10.1016/j.ccr.2010.11.015

Flavahan WA, 2016, NATURE, V529, P110, DOI 10.1038/nature16490

Friedrich M, 2021, NAT CANCER, V2, P723, DOI 10.1038/s43018-021-00201-z

Gaal J, 2010, J CLIN ENDOCR METAB, V95, P1274, DOI 10.1210/jc.2009-2170

GABRIEL JL, 1986, METABOLISM, V35, P661, DOI 10.1016/0026-0495(86)90175-7

Garrett M, 2018, CANCER METAB, V6, DOI 10.1186/s40170-018-0177-4

Garten A, 2015, NAT REV ENDOCRINOL, V11, P535, DOI 10.1038/nrendo.2015.117

Gatto L, 2021, MOL DIAGN THER, V25, P457, DOI 10.1007/s40291-021-00537-3

GELLERA C, 1990, NEUROLOGY, V40, P495, DOI 10.1212/WNL.40.3_Part_1.495

Gilbert MR, 2014, ACTA NEUROPATHOL, V127, P221, DOI 10.1007/s00401-013-1194-6

Golub D, 2019, FRONT ONCOL, V9, DOI 10.3389/fonc.2019.00417

Gronbaek K, 2007, APMIS, V115, P1039, DOI 10.1111/j.1600-0463.2007.apm_636.xml.x

Gross S, 2010, J EXP MED, V207, P339, DOI 10.1084/jem.20092506

Gupta SK, 2016, JNCI-J NATL CANCER I, V108, DOI 10.1093/jnci/djv369

Hansen E, 2014, BLOOD, V124, DOI 10.1182/blood.V124.21.3734.3734

Hartman DJ, 2014, AM J SURG PATHOL, V38, P1147, DOI 10.1097/PAS.0000000000000239

Hartmann C, 2009, ACTA NEUROPATHOL, V118, P469, DOI 10.1007/s00401-009-0561-9

Hartong DT, 2008, NAT GENET, V40, P1230, DOI 10.1038/ng.223

Hemerly JP, 2010, EUR J ENDOCRINOL, V163, P747, DOI 10.1530/EJE-10-0473

Heredia V., 2017, Ann Oncol, V28, pv538, DOI DOI 10.1093/ANNONC/MDX387.049

Herman MA, 2011, PLOS ONE, V6, DOI 10.1371/journal.pone.0026501

Heuser M, 2020, LEUKEMIA, V34, P2903, DOI 10.1038/s41375-020-0996-5

Hinsch A, 2018, WORLD J UROL, V36, P877, DOI 10.1007/s00345-018-2225-7

Hirata M, 2015, P NATL ACAD SCI USA, V112, P2829, DOI 10.1073/pnas.1424400112

Huang J, 2019, FRONT ONCOL, V9, DOI 10.3389/fonc.2019.00506

HURLEY JH, 1991, BIOCHEMISTRY-US, V30, P8671, DOI 10.1021/bi00099a026

Hvinden IC, 2021, CELL REP MED, V2, DOI 10.1016/j.xcrm.2021.100469

Ichimura K, 2009, NEURO-ONCOLOGY, V11, P341, DOI 10.1215/15228517-2009-025

Itsumi M, 2015, CELL DEATH DIFFER, V22, P1837, DOI 10.1038/cdd.2015.38

Jackson CB, 2014, J MED GENET, V51, P170, DOI 10.1136/jmedgenet-2013-101932

Jiao YC, 2013, NAT GENET, V45, P1470, DOI 10.1038/ng.2813

Jo SH, 2001, J BIOL CHEM, V276, P16168, DOI 10.1074/jbc.M010120200

Johannessen TCA, 2016, MOL CANCER RES, V14, P976, DOI 10.1158/1541-7786.MCR-16-0141

Johnson BE, 2014, SCIENCE, V343, P189, DOI 10.1126/science.1239947

Joseph JW, 2006, J BIOL CHEM, V281, P35624, DOI 10.1074/jbc.M602606200

Kalluri AL, 2023, INT J MOL SCI, V24, DOI 10.3390/ijms24032020

Kaneko MK, 2014, CANCER SCI, V105, P744, DOI 10.1111/cas.12413

Khurshed M, 2017, ONCOTARGET, V8, P49165, DOI 10.18632/oncotarget.17106

Kickingereder P, 2015, SCI REP-UK, V5, DOI 10.1038/srep16238

Kimura H, 2013, J HUM GENET, V58, P439, DOI 10.1038/jhg.2013.66

Kipp BR, 2012, HUM PATHOL, V43, P1552, DOI 10.1016/j.humpath.2011.12.007

Kizilbash SH, 2019, J CLIN ONCOL, V37, DOI 10.1200/JCO.2019.37.15_suppl.TPS2075

Koh HJ, 2004, J BIOL CHEM, V279, P39968, DOI 10.1074/jbc.M402260200

Kohanbash G, 2017, J CLIN INVEST, V127, P1425, DOI 10.1172/JCI90644

Kohli RM, 2013, NATURE, V502, P472, DOI 10.1038/nature12750

Koivunen P, 2012, NATURE, V483, P485, DOI 10.1038/nature10898

Kölker S, 2002, EUR J NEUROSCI, V16, P21, DOI 10.1046/j.1460-9568.2002.02055.x

Konteatis Z, 2020, ACS MED CHEM LETT, V11, P101, DOI 10.1021/acsmedchemlett.9b00509

Kopinja J, 2017, SCI REP-UK, V7, DOI 10.1038/s41598-017-14065-w

Krell D, 2011, PLOS ONE, V6, DOI 10.1371/journal.pone.0019868

Kurek KC, 2013, AM J PATHOL, V182, P1494, DOI 10.1016/j.ajpath.2013.01.012

Lai A, 2011, J CLIN ONCOL, V29, P4482, DOI 10.1200/JCO.2010.33.8715

Lange F, 2021, CELLS-BASEL, V10, DOI 10.3390/cells10051226

Le A, 2010, P NATL ACAD SCI USA, V107, P2037, DOI 10.1073/pnas.0914433107

Lee JH, 2017, WORLD J SURG ONCOL, V15, DOI 10.1186/s12957-017-1144-1

Lee SH, 2004, INT J RADIAT BIOL, V80, P635, DOI 10.1080/09553000400007680

LEIGHTON F, 1969, J CELL BIOL, V41, P521, DOI 10.1083/jcb.41.2.521

Li W, 2022, NAT COMMUN, V13, DOI 10.1038/s41467-022-35199-0

Li-Chang HH, 2015, BMC CANCER, V15, DOI 10.1186/s12885-015-1021-7

Linninger A, 2018, NEURO-ONCOLOGY, V20, P1197, DOI 10.1093/neuonc/noy051

Liu XJ, 2021, SIGNAL TRANSDUCT TAR, V6, DOI 10.1038/s41392-021-00774-2

Liu XJ, 2020, AM J CANCER RES, V10, P3212

Liu Y, 2019, JNCI-J NATL CANCER I, V111, P1033, DOI 10.1093/jnci/djy230

Liubinas SV, 2014, EPILEPSIA, V55, P1438, DOI 10.1111/epi.12662

Lokker NA, 2002, CANCER RES, V62, P3729

Lopez GY, 2010, BIOCHEM BIOPH RES CO, V398, P585, DOI 10.1016/j.bbrc.2010.06.125

Losman JA, 2013, GENE DEV, V27, P836, DOI 10.1101/gad.217406.113

Lozada JR, 2018, HISTOPATHOLOGY, V73, P339, DOI 10.1111/his.13522

Lu C, 2013, GENE DEV, V27, P1986, DOI 10.1101/gad.226753.113

Lu C, 2012, NATURE, V483, P474, DOI 10.1038/nature10860

Lu J, 2019, J BUON, V24, P2458

Lugowska I, 2018, J CANCER, V9, P998, DOI 10.7150/jca.22915

Ma D, 2021, CANCER LETT, V517, P35, DOI 10.1016/j.canlet.2021.05.038

Ma R, 2018, BIOCHEM BIOPH RES CO, V503, P2912, DOI 10.1016/j.bbrc.2018.08.068

Ma TF, 2017, SCI REP-UK, V7, DOI 10.1038/srep41882

Malta TM, 2018, NEURO-ONCOLOGY, V20, P608, DOI 10.1093/neuonc/nox183

Marcucci G, 2010, J CLIN ONCOL, V28, P2348, DOI 10.1200/JCO.2009.27.3730

Mardis ER, 2009, NEW ENGL J MED, V361, P1058, DOI 10.1056/NEJMoa0903840

Matre P, 2016, ONCOTARGET, V7, P79708, DOI 10.18632/oncotarget.12944

Matteo DA, 2017, J BIOL CHEM, V292, P7971, DOI 10.1074/jbc.M117.776179

Maus A, 2017, AMINO ACIDS, V49, P21, DOI 10.1007/s00726-016-2342-9

May JL, 2019, SCI ADV, V5, DOI 10.1126/sciadv.aat0456

McBrayer SK, 2018, CELL, V175, P101, DOI 10.1016/j.cell.2018.08.038

McDonough MA, 2010, CURR OPIN STRUC BIOL, V20, P659, DOI 10.1016/j.sbi.2010.08.006

Mellinghoff I., 2018, NEURO-ONCOLOGY, V20, pvi18, DOI [10.1093/neuonc/noy148.064, DOI 10.1093/NEUONC/NOY148.064]

Mellinghoff IK, 2019, NEURO-ONCOLOGY, V21, P2, DOI 10.1093/neuonc/noz126.004

Mellinghoff IK., 2020, J CLIN ONCOL, V38, DOI [DOI 10.1200/JCO.2020.38.15_SUPPL.TPS2574, 10.1200/JCO.2020.38.15_suppl.TPS2574]

Metellus P, 2011, J NEURO-ONCOL, V105, P591, DOI 10.1007/s11060-011-0625-2

Mohrenz IV, 2013, APOPTOSIS, V18, P1416, DOI 10.1007/s10495-013-0877-8

Molenaar RJ, 2015, LEUKEMIA, V29, P2134, DOI 10.1038/leu.2015.91

Molenaar RJ, 2015, CANCER RES, V75, P4790, DOI 10.1158/0008-5472.CAN-14-3603

Molenaar RJ, 2014, BBA-REV CANCER, V1846, P326, DOI 10.1016/j.bbcan.2014.05.004

Morales JC, 2014, CRIT REV EUKAR GENE, V24, P15, DOI 10.1615/CritRevEukaryotGeneExpr.2013006875

Mortazavi A, 2022, NEURO-ONCOLOGY, V24, P1423, DOI 10.1093/neuonc/noac003

Mukherjee J, 2018, CANCER RES, V78, P2966, DOI 10.1158/0008-5472.CAN-17-2269

Murugan AK, 2010, BIOCHEM BIOPH RES CO, V393, P555, DOI 10.1016/j.bbrc.2010.02.095

Nagaraj R, 2017, CELL, V168, P210, DOI 10.1016/j.cell.2016.12.026

Natsume A, 2023, NEURO-ONCOLOGY, V25, P326, DOI 10.1093/neuonc/noac155

Nepal C, 2018, HEPATOLOGY, V68, P949, DOI 10.1002/hep.29764

Nicolay B, 2017, NEURO-ONCOLOGY, V19, P86

Norsworthy KJ, 2019, CLIN CANCER RES, V25, P3205, DOI 10.1158/1078-0432.CCR-18-3749

Notarangelo G, 2022, SCIENCE, V377, P1519, DOI 10.1126/science.abj5104

Noushmehr H, 2010, CANCER CELL, V17, P510, DOI 10.1016/j.ccr.2010.03.017

Odejide O, 2014, BLOOD, V123, P1293, DOI 10.1182/blood-2013-10-531509

Ohka F, 2014, TUMOR BIOL, V35, P5911, DOI 10.1007/s13277-014-1784-5

Okamoto K, 2019, CELLS-BASEL, V8, DOI 10.3390/cells8020107

Okoye-Okafor UC, 2015, NAT CHEM BIOL, V11, P878, DOI 10.1038/nchembio.1930

Parsons DW, 2008, SCIENCE, V321, P1807, DOI 10.1126/science.1164382

Paschka P, 2010, J CLIN ONCOL, V28, P3636, DOI 10.1200/JCO.2010.28.3762

Peter VG, 2019, OPHTHALMIC GENET, V40, P177, DOI 10.1080/13816810.2019.1605391

Pierrache LHM, 2017, OPHTHALMOLOGY, V124, P992, DOI 10.1016/j.ophtha.2017.03.010

Platten M, 2021, NATURE, V592, P463, DOI 10.1038/s41586-021-03363-z

Polivka Jiri Jr, 2018, Oncotarget, V9, P16462, DOI 10.18632/oncotarget.24536

Polychronidou G, 2017, FUTURE ONCOL, V13, P637, DOI 10.2217/fon-2016-0226

Poon Candice C, 2019, Oncotarget, V10, P3129, DOI 10.18632/oncotarget.26863

Pope WB, 2012, J NEURO-ONCOL, V107, P197, DOI 10.1007/s11060-011-0737-8

Popovici-Muller J, 2018, ACS MED CHEM LETT, V9, P300, DOI 10.1021/acsmedchemlett.7b00421

Pusch S, 2017, ACTA NEUROPATHOL, V133, P629, DOI 10.1007/s00401-017-1677-y

Pusch S, 2014, ACTA NEUROPATHOL COM, V2, DOI 10.1186/2051-5960-2-19

RAMACHANDRAN N, 1980, J BIOL CHEM, V255, P8859

Rastogi RP, 2010, J NUCLEIC ACIDS, V2010, DOI 10.4061/2010/592980

Reitman ZJ, 2011, P NATL ACAD SCI USA, V108, P3270, DOI 10.1073/pnas.1019393108

Reyhanoglu G, 2020, CUREUS J MED SCIENCE, V12, DOI 10.7759/cureus.12042

Rohle D, 2013, SCIENCE, V340, P626, DOI 10.1126/science.1236062

Rosenberg MJ, 2002, NAT GENET, V32, P175, DOI 10.1038/ng948

Ross JS, 2014, ONCOLOGIST, V19, P235, DOI 10.1634/theoncologist.2013-0352

Saha SK, 2014, NATURE, V513, P110, DOI 10.1038/nature13441

Sakata-Yanagimoto M, 2014, NAT GENET, V46, P171, DOI 10.1038/ng.2872

Sasaki M, 2012, GENE DEV, V26, P2038, DOI 10.1101/gad.198200.112

Sasaki M, 2012, NATURE, V488, P656, DOI 10.1038/nature11323

Schnittger S, 2010, BLOOD, V116, P5486, DOI 10.1182/blood-2010-02-267955

Schumacher T, 2014, NATURE, V512, P324, DOI 10.1038/nature13387

Seltzer MJ, 2010, CANCER RES, V70, P8981, DOI 10.1158/0008-5472.CAN-10-1666

Shany E, 1999, BIOCHEM BIOPH RES CO, V262, P163, DOI 10.1006/bbrc.1999.1133

Shi JL, 2015, TUMOR BIOL, V36, P655, DOI 10.1007/s13277-014-2644-z

Spiegel K, 2012, AM J HUM GENET, V90, P518, DOI 10.1016/j.ajhg.2012.01.009

Spiegel R, 2009, ANN NEUROL, V66, P419, DOI 10.1002/ana.21752

Stein EM, 2018, FUTURE ONCOL, V14, P23, DOI 10.2217/fon-2017-0392

Sulkowski PL, 2017, SCI TRANSL MED, V9, DOI 10.1126/scitranslmed.aal2463

Tallegas M, 2019, HUM PATHOL, V84, P183, DOI 10.1016/j.humpath.2018.09.015

Tang F, 2022, NEUROSCI BULL, V38, P1069, DOI 10.1007/s12264-022-00866-1

Tateishi K, 2017, CANCER RES, V77, P4102, DOI 10.1158/0008-5472.CAN-16-2263

Tateishi K, 2015, CANCER CELL, V28, P773, DOI 10.1016/j.ccell.2015.11.006

Tesileanu CMS, 2021, ACTA NEUROPATHOL, V141, P945, DOI 10.1007/s00401-021-02291-6

Tian WQ, 2022, FRONT PHARMACOL, V13, DOI 10.3389/fphar.2022.982424

Toth LN, 2018, HUM PATHOL, V78, P138, DOI 10.1016/j.humpath.2018.04.014

Tsukada Y, 2006, NATURE, V439, P811, DOI 10.1038/nature04433

Turcan S, 2018, NAT GENET, V50, P62, DOI 10.1038/s41588-017-0001-z

Turcan S, 2013, ONCOTARGET, V4, P1729, DOI 10.18632/oncotarget.1412

Turcan S, 2012, NATURE, V483, P479, DOI 10.1038/nature10866

Unruh D, 2019, SCI REP-UK, V9, DOI 10.1038/s41598-019-45346-1

van Lith SAM, 2014, NEURO-ONCOLOGY, V16, P1669, DOI 10.1093/neuonc/nou152

Wahner HCW, 2020, RADIAT ONCOL, V15, DOI 10.1186/s13014-020-01728-8

Waitkus MS, 2018, CANCER CELL, V34, P186, DOI 10.1016/j.ccell.2018.04.011

Waitkus MS, 2018, CANCER RES, V78, P36, DOI 10.1158/0008-5472.CAN-17-1352

Walsby-Tickle J, 2020, COMMUN BIOL, V3, DOI 10.1038/s42003-020-0957-6

Wang C, 2015, BLOOD, V126, P1741, DOI 10.1182/blood-2015-05-644591

Wang F, 2013, SCIENCE, V340, P622, DOI 10.1126/science.1234769

Wang HY, 2016, J EXP CLIN CANC RES, V35, DOI 10.1186/s13046-016-0362-7

Wang J, 2018, J SURG RES, V231, P116, DOI 10.1016/j.jss.2018.04.056

Wang P, 2013, ONCOGENE, V32, P3091, DOI 10.1038/onc.2012.315

Wang P, 2015, CELL REP, V13, P2353, DOI 10.1016/j.celrep.2015.11.029

Ward PS, 2012, ONCOGENE, V31, P2491, DOI 10.1038/onc.2011.416

Ward PS, 2010, CANCER CELL, V17, P225, DOI 10.1016/j.ccr.2010.01.020

Watanabe T, 2009, AM J PATHOL, V174, P1149, DOI 10.2353/ajpath.2009.080958

Wen H, 2015, J NEUROCHEM, V132, P183, DOI 10.1111/jnc.12950

Williams SC, 2011, ACTA NEUROPATHOL, V121, P279, DOI 10.1007/s00401-010-0790-y

Xu W, 2011, CANCER CELL, V19, P17, DOI 10.1016/j.ccr.2010.12.014

Xu X, 2004, J BIOL CHEM, V279, P33946, DOI 10.1074/jbc.M404298200

Yalaza C, 2017, ANN CLIN LAB SCI, V47, P362

Yan H, 2009, NEW ENGL J MED, V360, P765, DOI 10.1056/NEJMoa0808710

Yang B, 2010, CELL RES, V20, P1188, DOI 10.1038/cr.2010.145

Yang CD, 2009, CANCER RES, V69, P7986, DOI 10.1158/0008-5472.CAN-09-2266

Yang QJ, 2022, MOL THER, V30, P1188, DOI 10.1016/j.ymthe.2022.01.007

Yen K, 2018, CANCER RES, V78, DOI 10.1158/1538-7445.AM2018-4956

Yen K, 2018, MOL CANCER THER, V17, DOI 10.1158/1535-7163.TARG-17-B126

Yen K, 2017, CANCER DISCOV, V7, P478, DOI 10.1158/2159-8290.CD-16-1034

Zeidner JF, 2020, CLIN CANCER RES, V26, P4174, DOI 10.1158/1078-0432.CCR-20-1820

Zeng L, 2015, ONCOGENE, V34, P4758, DOI 10.1038/onc.2014.411

Zhang DX, 2015, CELL REP, V10, P1335, DOI 10.1016/j.celrep.2015.02.006

Zhang JM, 2020, CELL BIOSCI, V10, DOI 10.1186/s13578-020-00391-6

Zhang LJ, 2018, CLIN CANCER RES, V24, P5381, DOI 10.1158/1078-0432.CCR-17-3855

Zhang XR, 2016, NEURO-ONCOLOGY, V18, P1402, DOI 10.1093/neuonc/now061

Zhao SM, 2009, SCIENCE, V324, P261, DOI 10.1126/science.1170944

Zhou LN, 2019, J PROTEOME RES, V18, P960, DOI 10.1021/acs.jproteome.8b00663

NR 242

TC 15

Z9 15

U1 2

U2 11

PU MDPI

PI BASEL

PA ST ALBAN-ANLAGE 66, CH-4052 BASEL, SWITZERLAND

EI 2072-6694

J9 CANCERS

JI Cancers

PD MAY 23

PY 2023

VL 15

IS 11

AR 2883

DI 10.3390/cancers15112883

PG 31

WC Oncology

WE Science Citation Index Expanded (SCI-EXPANDED)

SC Oncology

GA I6NJ9

UT WOS:001003929100001

PM 37296846

OA Green Published, gold

DA 2025-04-09

ER

PT J

AU Hsieh, FY

Zhilenkov, AV

Voronov, II

Khakina, EA

Mischenko, DV

Troshin, PA

Hsu, SH

AF Hsieh, Fu-Yu

Zhilenkov, A. V.

Voronov, I. I.

Khakina, E. A.

Mischenko, D. V.

Troshin, Pavel A.

Hsu, Shan-hui

TI Water-Soluble Fullerene Derivatives as Brain Medicine: Surface Chemistry

Determines If They Are Neuroprotective and Antitumor

SO ACS APPLIED MATERIALS & INTERFACES

LA English

DT Article

DE water-soluble C-60 fullerene derivatives; neural repair; glioblastoma;

surface functionalization; antitumor

ID HUMAN SKIN KERATINOCYTES; CHLOROFULLERENE C60CL6; OXIDATIVE STRESS;

IN-VIVO; ZEBRAFISH; CARBOXYFULLERENES; CYTOTOXICITY; ANTIOXIDANTS;

ACTIVATION; PRECURSOR

AB Delivering drugs to the central nervous system (CNS) is a major challenge in treating CNS-related diseases. Nanoparticles that can cross blood-brain barrier (BBB) are potential tools. In this study, water-soluble C60 fullerene derivatives with different types of linkages between the fullerene cage and the solubilizing addend were synthesized (compounds 1-3: C-C bonds, compounds 4-5: C-S bonds, compound 6: C-P bonds, and compounds 7-9: C-N bonds). Fullerene derivatives 1-6 were observed to induce neural stem cell (NSC) proliferation in vitro and rescue the function of injured CNS in zebrafish. Fullerene derivatives 7-9 were found to inhibit glioblastoma cell proliferation in vitro and reduce glioblastoma formation in zebrafish. These effects were correlated with the cell metabolic changes. Particularly, compound 3 bearing residues of phenylbutiryc acids significantly promoted NSC proliferation and neural repair without causing tumor growth. Meanwhile, compound 7 with phenylalanine appendages significantly inhibited glioblastoma growth without retarding the neural repair. We conclude that the surface functional group determines the properties as well as the interactions of C60 with NSCs and glioma cells, producing either a neuroprotective or antitumor effect for possible treatment of CNS-related diseases

C1 [Hsieh, Fu-Yu; Hsu, Shan-hui] Natl Taiwan Univ, Inst Polymer Sci & Engn, 1,Sec 4 Roosevelt Rd, Taipei 10617, Taiwan.

[Hsu, Shan-hui] Natl Taiwan Univ, Res & Dev Ctr Med Devices, Taipei 10617, Taiwan.

[Zhilenkov, A. V.; Voronov, I. I.; Khakina, E. A.; Mischenko, D. V.; Troshin, Pavel A.] Russian Acad Sci, Inst Problems Chem Phys, Semenov Prospect 1, Chernogolovka 142432, Russia.

[Troshin, Pavel A.] Skolkovo Inst Sci & Technol, Moscow 143005, Russia.

[Hsu, Shan-hui] Natl Hlth Res Inst, Inst Cellular & Syst Med, Zhunan 35053, Taiwan.

C3 National Taiwan University; National Taiwan University; Russian Academy

of Sciences; Institute of Problems of Chemical Physics of the Russian

Academy of Sciences; Skolkovo Institute of Science & Technology;

National Health Research Institutes - Taiwan

RP Hsu, SH (corresponding author), Natl Taiwan Univ, Inst Polymer Sci & Engn, 1,Sec 4 Roosevelt Rd, Taipei 10617, Taiwan.; Troshin, PA (corresponding author), Russian Acad Sci, Inst Problems Chem Phys, Semenov Prospect 1, Chernogolovka 142432, Russia.; Troshin, PA (corresponding author), Skolkovo Inst Sci & Technol, Moscow 143005, Russia.

EM troshin2003@inbox.ru; shhsu@ntu.edu.tw

RI Zhilenkov, Alexander/D-8968-2014; Khakina, Ekaterina/GOG-7819-2022;

Mishchenko, Denis/H-3054-2011; Troshin, Pavel/A-5128-2014

OI Khakina, Ekaterina/0000-0001-6194-5073; Zhilenkov,

Alexander/0000-0003-4985-5088; Mishchenko, Denis/0000-0003-3779-3211;

Troshin, Pavel/0000-0001-9957-4140

FU bilateral Taiwanese-Russian research project (RFBR) [16-53-52030, MOST

105-2923-E-002-003 -MY3]

FX This work was supported by the bilateral Taiwanese-Russian research

project (RFBR No. 16-53-52030; MOST 105-2923-E-002-003 -MY3). We are

obliged to Dr. Ing-Ming Chiu (Institute of Cellular and Systems

Medicine, National Health Research Institutes, Zhunan, Taiwan) for

providing murine NSCs. We also appreciate the Taiwan Zebrafish Core

Facility located in National Taiwan University (NTU-ERP-104R8600) for

the technical and facility supports.

CR Ali SS, 2008, NANOMED-NANOTECHNOL, V4, P283, DOI 10.1016/j.nano.2008.05.003

Ali SS, 2004, FREE RADICAL BIO MED, V37, P1191, DOI 10.1016/j.freeradbiomed.2004.07.002

Bakry R, 2007, INT J NANOMED, V2, P639

Ball P, 2004, NATURE, V431, P729, DOI 10.1038/431729b

Burger C, 2004, J COLLOID INTERF SCI, V275, P632, DOI 10.1016/j.jcis.2004.02.048

Cai XQ, 2010, TOXICOL APPL PHARM, V243, P27, DOI 10.1016/j.taap.2009.11.009

Carew Jennifer S, 2002, Mol Cancer, V1, P9, DOI 10.1186/1476-4598-1-9

Chandel NS, 2000, J BIOL CHEM, V275, P25130, DOI 10.1074/jbc.M001914200

Chen ZY, 2012, THERANOSTICS, V2, P238, DOI 10.7150/thno.3509

Chiu IM, 2000, ONCOGENE, V19, P6229, DOI 10.1038/sj.onc.1204021

Dugan LL, 2001, PARKINSONISM RELAT D, V7, P243, DOI 10.1016/S1353-8020(00)00064-X

Dugan LL, 1997, P NATL ACAD SCI USA, V94, P9434, DOI 10.1073/pnas.94.17.9434

Fedorova NE, 2012, MENDELEEV COMMUN, V22, P254, DOI 10.1016/j.mencom.2012.09.009

Gharbi N, 2005, NANO LETT, V5, P2578, DOI 10.1021/nl051866b

Ghose AK, 1999, J COMB CHEM, V1, P55, DOI 10.1021/cc9800071

Hsu YC, 2009, DEV DYNAM, V238, P302, DOI 10.1002/dvdy.21753

Khakina EA, 2012, CHEM COMMUN, V48, P7158, DOI 10.1039/c2cc32517a

Kim GW, 2002, STROKE, V33, P809, DOI 10.1161/hs0302.103745

KIMMEL CB, 1995, DEV DYNAM, V203, P253, DOI 10.1002/aja.1002030302

Klaunig JE, 2010, TOXICOL PATHOL, V38, P96, DOI 10.1177/0192623309356453

Kobzar OL, 2015, MENDELEEV COMMUN, V25, P199, DOI 10.1016/j.mencom.2015.05.013

Kornev AB, 2012, CHEM COMMUN, V48, P5461, DOI 10.1039/c2cc00071g

Lipinski CA, 2000, J PHARMACOL TOX MET, V44, P235, DOI 10.1016/S1056-8719(00)00107-6

Liu QL, 2013, ACS APPL MATER INTER, V5, P11101, DOI 10.1021/am4033372

Pardridge WM, 2002, DRUG DISCOV TODAY, V7, P5, DOI 10.1016/S1359-6446(01)02082-7

Pardridge WM, 2001, JPN J PHARMACOL, V87, P97, DOI 10.1254/jjp.87.97

Partha Ranga, 2007, Journal of Nanobiotechnology, V5, P6, DOI 10.1186/1477-3155-5-6

Sano M, 2008, CIRC RES, V103, P1191, DOI 10.1161/CIRCRESAHA.108.189092

Santos SM, 2014, TOXICOL SCI, V138, P117, DOI 10.1093/toxsci/kft327

Sayes CM, 2004, NANO LETT, V4, P1881, DOI 10.1021/nl0489586

Schnorr JM, 2011, CHEM MATER, V23, P646, DOI 10.1021/cm102406h

Sonsalla PK, 2013, EXP NEUROL, V250, P376, DOI 10.1016/j.expneurol.2013.10.014

Troshina OA, 2007, ORG BIOMOL CHEM, V5, P2783, DOI 10.1039/b705331b

Usenko CY, 2007, CARBON, V45, P1891, DOI 10.1016/j.carbon.2007.04.021

Wu CC, 2014, PLOS ONE, V9, DOI [10.1371/journal.pone.0097902, 10.1371/journal.pone.0105362]

Xiao L, 2006, BIOORG MED CHEM LETT, V16, P1590, DOI 10.1016/j.bmcl.2005.12.011

Xiao L, 2005, BIOMED PHARMACOTHER, V59, P351, DOI 10.1016/j.biopha.2005.02.004

Xiao L, 2010, BIOMATERIALS, V31, P5976, DOI 10.1016/j.biomaterials.2010.04.032

Xie J, 2010, BMC DEV BIOL, V10, DOI 10.1186/1471-213X-10-76

Yurkova AA, 2012, CHEM COMMUN, V48, P8916, DOI 10.1039/c2cc34338j

Zhou SQ, 2005, J PHYS CHEM B, V109, P19741, DOI 10.1021/jp053978x

Zhou SQ, 2001, SCIENCE, V291, P1944, DOI 10.1126/science.291.5510.1944

NR 42

TC 85

Z9 86

U1 1

U2 77

PU AMER CHEMICAL SOC

PI WASHINGTON

PA 1155 16TH ST, NW, WASHINGTON, DC 20036 USA

SN 1944-8244

J9 ACS APPL MATER INTER

JI ACS Appl. Mater. Interfaces

PD APR 5

PY 2017

VL 9

IS 13

BP 11482

EP 11492

DI 10.1021/acsami.7b01077

PG 11

WC Nanoscience & Nanotechnology; Materials Science, Multidisciplinary

WE Science Citation Index Expanded (SCI-EXPANDED)

SC Science & Technology - Other Topics; Materials Science

GA ER4JE

UT WOS:000398764100020

PM 28263053

DA 2025-04-09

ER

PT J

AU Li, ZX

Ren, LX

Zhang, L

AF Li, Zongxi

Ren, Lingxuan

Zhang, Lie

TI Knockdown of PRR11 Induces Autophagy in Glioma Cells by Inhibiting

Akt/mTOR Signaling Pathway

SO NEUROCHEMICAL JOURNAL

LA English

DT Article

DE glioma; PRR11; cell cycle; autophagy; Akt/mTOR pathway

ID NALOXONE-PRECIPITATED WITHDRAWAL; 5-HT1A RECEPTOR ANTAGONIST; SUPPRESSES

BEHAVIORAL SENSITIZATION; BRAIN OXIDATIVE STRESS; DORSAL RAPHE NUCLEUS;

MORPHINE-WITHDRAWAL; CORTISOL SECRETION; OPIOID RECEPTORS; SEROTONIN;

EXPRESSION

AB Glioma is the most common type of primary craniocerebral tumor. Understanding the molecular mechanisms of glioma occurrence and development will provide strategies for effectively treating glioma. Proline-rich protein 11 (PRR11) is a protein which is widely overexpressed in different tumors. TCGA data analysis showed that PRR11 expression was up-regulated in glioma tissues, but its role still needs to be further studied. Here, the role of PRR11 in glioma progression and the mechanism were investigated. We found PRR11 was overexpressed in glioma cells. Depletion of PRR11 suppressed the growth of glioma cells as well as induced cell cycle arrest. We further found PRR11 ablation induced the autophagy of glioma cells. Furthermore, knockdown of PRR11 restrained the activation of Akt/mTOR pathway, thereby suppressing the proliferation of glioma cells. We thought PRR11 could serve as a target for glioma therapy.

C1 [Li, Zongxi; Zhang, Lie] Chengdu Med Coll, Dept Neurosurg, Affiliated Hosp 1, Chengdu 610500, Sichuan, Peoples R China.

[Ren, Lingxuan] ChengDu XinDu Maternal & Child Hlth Care Hosp, Dept Child Healthcare, Chengdu 610000, Sichuan, Peoples R China.

C3 Chengdu Medical College

RP Zhang, L (corresponding author), Chengdu Med Coll, Dept Neurosurg, Affiliated Hosp 1, Chengdu 610500, Sichuan, Peoples R China.

EM zhanglie3082501@163.com

FU Funding Project of Sichuan Collaborative Innovation Center for Elderly

Careand Elderly Health, Chengdu Medical College [YLZBZ2014]

FX This work was supported by the Funding Project of Sichuan Collaborative

Innovation Center for Elderly Careand Elderly Health, Chengdu Medical

College (GrantNo. YLZBZ2014).

CR Abdel-Zaher AO, 2013, BEHAV BRAIN RES, V247, P17, DOI 10.1016/j.bbr.2013.02.034

Abdel-Zaher AO, 2013, EUR J PHARMACOL, V702, P62, DOI 10.1016/j.ejphar.2013.01.036

Afshar S., 2023, Univer. Med. Sci, V28, P40

Afshar S, 2019, J CHEM NEUROANAT, V96, P140, DOI 10.1016/j.jchemneu.2019.01.008

Albert PR, 2011, MOL BRAIN, V4, DOI 10.1186/1756-6606-4-21

Arborelius L, 1996, N-S ARCH PHARMACOL, V353, P630, DOI 10.1007/BF00167182

Ballantyne JC, 2021, PAIN, V162, P2315, DOI 10.1097/j.pain.0000000000002280

Bardoni R, 2019, CURR NEUROPHARMACOL, V17, P1133, DOI 10.2174/1570159X17666191001123900

Bearn J, 2001, ADDICT BIOL, V6, P157, DOI 10.1080/13556210020040235

Blier P, 2003, BIOL PSYCHIAT, V53, P193, DOI 10.1016/S0006-3223(02)01643-8

Bohn LM, 2010, CRIT REV BIOCHEM MOL, V45, P555, DOI 10.3109/10409238.2010.516741

Broderick PA, 2008, J NEURAL TRANSM, V115, P7, DOI 10.1007/s00702-007-0809-2

Celada P, 2004, J PSYCHIATR NEUROSCI, V29, P252

Charkhpour M, 2014, Drug Res (Stuttg), V64, P393, DOI 10.1055/s-0033-1358728

Colpaert FC, 2006, J PHARMACOL EXP THER, V316, P892, DOI 10.1124/jpet.105.095109

Darmani NA, 1996, PHARMACOL BIOCHEM BE, V55, P1, DOI 10.1016/0091-3057(96)00072-X

DRUST EG, 1979, PHARMACOLOGY, V18, P299, DOI 10.1159/000137269

Ebadi M, 1998, J PINEAL RES, V24, P193, DOI 10.1111/j.1600-079X.1998.tb00532.x

ELKADI AOS, 1995, LIFE SCI, V57, P511, DOI 10.1016/0024-3205(95)00284-D

Fink KB, 2007, PHARMACOL REV, V59, P360, DOI 10.1124/pr.107.07103

FORSTER EA, 1995, EUR J PHARMACOL, V281, P81, DOI 10.1016/0014-2999(95)00234-C

Gao J, 2020, NEUROPHARMACOLOGY, V162, DOI 10.1016/j.neuropharm.2019.107848

GELLERT VF, 1978, J PHARMACOL EXP THER, V205, P536

GULATI A, 1990, EUR J PHARMACOL, V182, P253, DOI 10.1016/0014-2999(90)90284-D

Jabeen HD, 2018, PHARMACOL RES, V134, P212, DOI 10.1016/j.phrs.2018.06.030

Johansson L, 1997, J PHARMACOL EXP THER, V283, P216

Kirby LG, 2011, NEUROPHARMACOLOGY, V61, P421, DOI 10.1016/j.neuropharm.2011.03.022

KOOB GF, 1992, TRENDS NEUROSCI, V15, P186, DOI 10.1016/0166-2236(92)90171-4

Lanfumey L, 2008, NEUROSCI BIOBEHAV R, V32, P1174, DOI 10.1016/j.neubiorev.2008.04.006

LESCH KP, 1990, J CLIN ENDOCR METAB, V70, P670, DOI 10.1210/jcem-70-3-670

Li JX, 2013, PSYCHOPHARMACOLOGY, V225, P791, DOI 10.1007/s00213-012-2870-2

Lovallo WR, 2006, INT J PSYCHOPHYSIOL, V59, P195, DOI 10.1016/j.ijpsycho.2005.10.007

Lutz PE, 2013, TRENDS NEUROSCI, V36, P195, DOI 10.1016/j.tins.2012.11.002

Lutz PE, 2011, EUR NEUROPSYCHOPHARM, V21, P835, DOI 10.1016/j.euroneuro.2011.02.002

Mahmoodi M, 2014, PSICOTHEMA, V26, P511, DOI 10.7334/psicothema2014.36

MALDONADO R, 1992, J PHARMACOL EXP THER, V261, P669

Maroteaux L, 2019, BIOCHIMIE, V161, P23, DOI 10.1016/j.biochi.2019.01.009

Matinfar Mahdieh, 2013, Adv Biomed Res, V2, P80, DOI 10.4103/2277-9175.120868

McLean TH, 2006, J MED CHEM, V49, P5794, DOI 10.1021/jm060656o

Misane I, 2003, NEUROPSYCHOPHARMACOL, V28, P253, DOI 10.1038/sj.npp.1300024

Morgan MM, 2011, BRIT J PHARMACOL, V164, P1322, DOI 10.1111/j.1476-5381.2011.01335.x

Mori T, 2014, EUR J PHARMACOL, V740, P160, DOI 10.1016/j.ejphar.2014.07.009

Motaghinejad M, 2016, IRAN J MED SCI, V41, P53

Motaghinejad Majid, 2014, Adv Biomed Res, V3, P171, DOI 10.4103/2277-9175.139181

Motavalli M, 2018, PARASITOL RES, V117, P2957, DOI 10.1007/s00436-018-5992-6

Müller CP, 2007, PROG NEUROBIOL, V81, P133, DOI 10.1016/j.pneurobio.2007.01.001

Müller CP, 2015, BEHAV BRAIN RES, V277, P146, DOI 10.1016/j.bbr.2014.04.007

Naumenko VS, 2010, GENES BRAIN BEHAV, V9, P519, DOI 10.1111/j.1601-183X.2010.00581.x

Nunez C, 2007, J NEUROCHEM, V101, P1060, DOI 10.1111/j.1471-4159.2006.04421.x

Ozdemir E., 2017, Int. J. Basic & Clin. Pharmacol., V6, P217, DOI [10.18203/2319-2003.ijbcp20170312, DOI 10.18203/2319-2003.IJBCP20170312]

Palchaudhuri M, 2005, CELL TISSUE RES, V321, P159, DOI 10.1007/s00441-005-1112-x

Pang G, 2016, FRONT PHARMACOL, V7, DOI 10.3389/fphar.2016.00514

POTHOS E, 1991, BRAIN RES, V566, P348, DOI 10.1016/0006-8993(91)91724-F

Raymond JR, 1999, BRIT J PHARMACOL, V127, P1751, DOI 10.1038/sj.bjp.0702723

Richardson-Jones JW, 2010, NEURON, V65, P40, DOI 10.1016/j.neuron.2009.12.003

Rojas-Corrales MO, 2005, EUR J PHARMACOL, V511, P21, DOI 10.1016/j.ejphar.2005.02.006

Ross Svante B., 1999, CNS Drug Reviews, V5, P213

ROSSETTI ZL, 1992, EUR J PHARMACOL, V221, P227, DOI 10.1016/0014-2999(92)90706-A

SAMANIN R, 1980, LIFE SCI, V27, P1141, DOI 10.1016/0024-3205(80)90464-6

Samarghandian S, 2014, INT J CLIN EXP MED, V7, P1449

Sánchez-Brualla I, 2018, NEUROSCIENCE, V387, P48, DOI 10.1016/j.neuroscience.2017.08.033

Schechter LE, 2002, CURR PHARM DESIGN, V8, P139, DOI 10.2174/1381612023396483

Schiavone S, 2016, TOXICOL LETT, V258, P29, DOI 10.1016/j.toxlet.2016.06.002

Shahidi S, 2014, NEUROSCI LETT, V578, P27, DOI 10.1016/j.neulet.2014.06.027

Tao R, 1998, J PHARMACOL EXP THER, V286, P481

Trajkovska V, 2009, EXP NEUROL, V218, P83, DOI 10.1016/j.expneurol.2009.04.008

WAY EL, 1968, SCIENCE, V162, P1290, DOI 10.1126/science.162.3859.1290

Wu X, 2015, NEUROSCI LETT, V607, P23, DOI 10.1016/j.neulet.2015.09.013

Xu XJ, 2003, TRENDS PHARMACOL SCI, V24, P634, DOI 10.1016/j.tips.2003.10.005

Yan CZ, 2004, ACTA PHARMACOL SIN, V25, P1285

You IJ, 2016, NEUROPSYCHOPHARMACOL, V41, P1210, DOI 10.1038/npp.2015.268

Zhang GL, 2016, NEUROPHARMACOLOGY, V101, P246, DOI 10.1016/j.neuropharm.2015.09.031

Zhang GL, 2015, FRONT PHARMACOL, V6, DOI 10.3389/fphar.2015.00225

NR 73

TC 0

Z9 0

U1 3

U2 4

PU MAIK NAUKA/INTERPERIODICA/SPRINGER

PI NEW YORK

PA 233 SPRING ST, NEW YORK, NY 10013-1578 USA

SN 1819-7124

EI 1819-7132

J9 NEUROCHEM J+

JI Neurochem. J.

PD JUN

PY 2024

VL 18

IS 2

BP 264

EP 270

DI 10.1134/S1819712424020077

PG 7

WC Neurosciences

WE Science Citation Index Expanded (SCI-EXPANDED)

SC Neurosciences & Neurology

GA SG8G7

UT WOS:001233389200003

DA 2025-04-09

ER

PT J

AU Inal, BB

Emre, HO

Baran, O

Ahmedov, M

Ozdemir, AF

Kemerdere, R

Ates, S

Tanriverdi, T

AF Inal, Berrin Bercik

Emre, Humeyra Ozturk

Baran, Oguz

Ahmedov, Merdin.

Ozdemir, Ahmet Faruk

Kemerdere, Rahsan

Ates, Seda

Tanriverdi, Taner

TI Dynamic thiol-disulphide homeostasis in low-grade gliomas: Preliminary

results in serum

SO CLINICAL NEUROLOGY AND NEUROSURGERY

LA English

DT Article

DE Disulfide bond; Gliomas; Native thiol; Oxidation; Total thiol

ID TOTAL ANTIOXIDANT CAPACITY; OXIDATIVE STRESS MARKER; TRANSITIONAL

MENINGIOMA; GLIOBLASTOMA-MULTIFORME; DNA-DAMAGE; DISEASE; TUMORS

AB Objective: Maintaining of precise balance between oxidation and anti-oxidation is important in both physiological and pathological states. Knowledge about this balance may give an idea about the process of the disease. The aim of this study was to investigate dynamic thiol-disulfide homeostasis in patients with low-grade gliomas.

Patients and methods: Serial serum samples were collected in 13 patients operated on low-grade gliomas before and after surgery. Control serum samples were obtained from venous cord blood from 13 healthy women during cesarean section. Total thiol, native thiol, and disulfide bond formation were measured and compared with the controls.

Results: Total thiols, native thiols, and disulfide bond formation were significantly elevated in patients before the surgery compared to the controls (p < 0.05). Even after the surgery, these three parameters were still high in patients, and the differences were significant (p < 0.05). Although no significant difference was found between patients and controls regarding the ratios of disulfide/total thiol, disulfide/native thiol, and native thiol/total thiol (p > 0.05), the balance seemed to shift to oxidative side.

Conclusions: Thiol-disulfide homeostasis was disrupted in patients with low-grade gliomas, and oxidation may play a role in the process of this disease. Supplementation with antioxidants before and after surgery may be taken into consideration.

C1 [Inal, Berrin Bercik] Istanbul Res & Training Res Hosp, Clin Biochem Lab, Istanbul, Turkey.

[Emre, Humeyra Ozturk] Haseki Training & Res Hosp, Clin Biochem Lab, Istanbul, Turkey.

[Baran, Oguz] Istanbul Res & Training Res Hosp, Neurosurg Clin, Istanbul, Turkey.

[Ahmedov, Merdin.; Ozdemir, Ahmet Faruk; Kemerdere, Rahsan; Tanriverdi, Taner] Istanbul Univ, Cerrahpasa Med Fac, Dept Neurosurg, Cerrahpasa, Turkey.

[Ates, Seda] Bezm i Alem Vakif Univ, Med Fac, Dept Obstet & Gynecol, Istanbul, Turkey.

C3 Istanbul Haseki Training & Research Hospital; Istanbul University -

Cerrahpasa; Istanbul University; Bezmialem Vakif University

RP Baran, O (corresponding author), Istanbul Res & Training Res Hosp, Neurosurg Clin, Istanbul, Turkey.

EM oguzbaran@gmail.com

RI Özdemir, Ahmet/AAE-7569-2021; BARAN, Oguz/AAG-3278-2019; Ahmedov,

Merdin/IQU-7056-2023; Kemerdere, Rahsan/D-6667-2019; BercikInal,

Berrin/MIK-5967-2025; Tanriverdi, Taner/C-9003-2019; OZTURK EMRE,

HUMEYRA/KCZ-1257-2024

OI Kemerdere, Rahsan/0000-0002-9947-3486; OZTURK EMRE,

HUMEYRA/0000-0003-0422-4549; Ozdemir, Ahmet Faruk/0000-0002-7919-1609;

Baran, Oguz/0000-0002-2345-4318

CR Bicíková M, 2006, CLIN CHEM LAB MED, V44, P978, DOI 10.1515/CCLM.2006.170

Erel O, 2014, CLIN BIOCHEM, V47, P326, DOI 10.1016/j.clinbiochem.2014.09.026

Gumusyayla S, 2016, ACTA NEUROPSYCHIATR, V28, P315, DOI 10.1017/neu.2016.13

Gumusyayla S, 2016, NEUROL SCI, V37, P1311, DOI 10.1007/s10072-016-2592-z

Hanimoglu H, 2007, CLIN NEUROL NEUROSUR, V109, P561, DOI 10.1016/j.clineuro.2007.04.007

Lopaczynski W, 2001, NUTR RES, V21, P295, DOI 10.1016/S0271-5317(00)00288-8

Louis DN, 2016, ACTA NEUROPATHOL, V131, P803, DOI 10.1007/s00401-016-1545-1

McBean GJ, 2015, REDOX BIOL, V5, P186, DOI 10.1016/j.redox.2015.04.004

Prakash M., 2009, OJHAS, V8, P1

Ratnayake S, 2013, J PROTEOMICS, V92, P160, DOI 10.1016/j.jprot.2013.06.019

Rossi R., 2008, J CELLULAR MOL MED, V10, P1582

Tanriverdi T, 2007, J CANCER RES CLIN, V133, P627, DOI 10.1007/s00432-007-0212-2

Tuzgen S, 2007, CLIN ONCOL-UK, V19, P177, DOI 10.1016/j.clon.2006.11.012

NR 13

TC 5

Z9 5

U1 0

U2 5

PU ELSEVIER SCIENCE BV

PI AMSTERDAM

PA PO BOX 211, 1000 AE AMSTERDAM, NETHERLANDS

SN 0303-8467

EI 1872-6968

J9 CLIN NEUROL NEUROSUR

JI Clin. Neurol. Neurosurg.

PD OCT

PY 2017

VL 161

BP 17

EP 21

DI 10.1016/j.clineuro.2017.08.002

PG 5

WC Clinical Neurology; Surgery

WE Science Citation Index Expanded (SCI-EXPANDED)

SC Neurosciences & Neurology; Surgery

GA FI8ME

UT WOS:000412255900004

PM 28804016

DA 2025-04-09

ER

PT J

AU Moon, H

Jang, JH

Jang, TC

Park, GH

AF Moon, Hyewon

Jang, Jung-Hee

Jang, Tae Chang

Park, Gyu Hwan

TI Carbon Monoxide Ameliorates 6-Hydroxydopamine-Induced Cell Death in C6

Glioma Cells

SO BIOMOLECULES & THERAPEUTICS

LA English

DT Article

DE CO; PD; Neuroprotection; Nrf2; HO-1; SOD

ID OXIDATIVE STRESS; THERAPEUTIC TARGET; INDUCED INFLAMMATION;

PARKINSONS-DISEASE; HYDROGEN-SULFIDE; NEURAL MESSENGER; HEME OXYGENASE;

NITRIC-OXIDE; APOPTOSIS; INJURY

AB Carbon monoxide (CO) is well-known as toxic gas and intrinsic signaling molecule such as neurotransmitter and blood vessel relaxant. Recently, it has been reported that low concentration of CO exerts therapeutic actions under various pathological conditions including liver failure, heart failure, gastric cancer, and cardiac arrest. However, little has been known about the effect of CO in neurodegenerative diseases like Parkinson's disease (PD). To test whether CO could exert a beneficial action during oxidative cell death in PD, we examined the effects of CO on 6-hydroxydopamine (6-OHDA)-induced cell death in C6 glioma cells. Treatment of CO-releasing molecule-2 (CORM-2) significantly attenuated 6-OHDA-induced apoptotic cell death in a dose-dependent manner. CORM-2 treatment decreased Bax/Bcl2 ratio and caspase-3 activity, which had been increased by 6-OHDA. CORM-2 increased phosphorylation of NF-E2-related factor 2 (Nrf2) which is a transcription factor regulating antioxidant proteins. Subsequently, CORM-2 also increased the expression of heme oxygenase-1 and superoxide dismutases (CuZnSOD and MnSOD), which were antioxidant enzymes regulated by Nrf2. These results suggest that CO released by CORM-2 treatment may have protective effects against oxidative cell death in PD through the potentiation of cellular adaptive survival responses via activation of Nrf2 and upregulation of heme oxygenase-1, leading to increasing antioxidant defense capacity.

C1 [Moon, Hyewon; Park, Gyu Hwan] Kyungpook Natl Univ, Res Inst Pharmaceut Sci, Coll Pharm, Daegu 41566, South Korea.

[Jang, Jung-Hee] Keimyung Univ, Sch Med, Dept Pharmacol, Daegu 42601, South Korea.

[Jang, Tae Chang] Daegu Catholic Univ, Sch Med, Dept Emergency Med, Daegu 42472, South Korea.

C3 Kyungpook National University (KNU); Keimyung University; Catholic

University of Daegu

RP Park, GH (corresponding author), Kyungpook Natl Univ, Res Inst Pharmaceut Sci, Coll Pharm, Daegu 41566, South Korea.; Jang, TC (corresponding author), Daegu Catholic Univ, Sch Med, Dept Emergency Med, Daegu 42472, South Korea.

EM emzzang@cu.ac.kr; park014@knu.ac.kr

OI Jang, Tae Chang/0000-0002-0895-5990

FU Research Institute of Medical Science, Catholic University of Daegu

FX This work was supported by the grant of Research Institute of Medical

Science, Catholic University of Daegu (2016).

CR Babu D, 2015, TOXICOL APPL PHARM, V288, P161, DOI 10.1016/j.taap.2015.07.007

Basuroy S, 2013, AM J PHYSIOL-CELL PH, V304, pC1105, DOI 10.1152/ajpcell.00023.2013

Blandini Fabio, 2008, Parkinsonism Relat Disord, V14 Suppl 2, pS124, DOI 10.1016/j.parkreldis.2008.04.015

Caumartin Y, 2011, KIDNEY INT, V79, P1080, DOI 10.1038/ki.2010.542

Chapman JT, 2001, AM J PHYSIOL-LUNG C, V281, pL209, DOI 10.1152/ajplung.2001.281.1.L209

Chi PL, 2015, MOL NEUROBIOL, V52, P277, DOI 10.1007/s12035-014-8869-4

Choi YK, 2018, BIOMOL THER, V26, P93, DOI 10.4062/biomolther.2017.144

Christie AE, 2014, GEN COMP ENDOCR, V202, P76, DOI 10.1016/j.ygcen.2014.04.003

Fledderus JO, 2013, NEPHROL DIAL TRANSPL, V28, P1969, DOI 10.1093/ndt/gft202

Ghattas MH, 2002, INT J BIOCHEM CELL B, V34, P1619, DOI 10.1016/S1357-2725(02)00097-3

Halliwell B, 2006, J NEUROCHEM, V97, P1634, DOI 10.1111/j.1471-4159.2006.03907.x

Herman ZS, 1997, POL J PHARMACOL, V49, P1

Hettiarachchi NT, 2014, CELL DEATH DIS, V5, DOI 10.1038/cddis.2014.529

Innamorato NG, 2008, J IMMUNOL, V181, P680, DOI 10.4049/jimmunol.181.1.680

Joshi Gururaj, 2012, Recent Pat CNS Drug Discov, V7, P218

Kaizaki A, 2006, BRAIN RES, V1108, P39, DOI 10.1016/j.brainres.2006.06.011

Kalia LV, 2016, NAT REV NEUROL, V12, P65, DOI 10.1038/nrneurol.2015.249

Kikuchi A, 2002, NEUROBIOL DIS, V9, P244, DOI 10.1006/nbdi.2002.0466

Kim DS, 2009, IMMUNOPHARM IMMUNOT, V31, P64, DOI 10.1080/08923970802354762

Lian S, 2016, TOXICOLOGY, V361, P24, DOI 10.1016/j.tox.2016.07.003

Magierowski M, 2016, DIGEST DIS SCI, V61, P3176, DOI 10.1007/s10620-016-4280-5

McCoole MD, 2012, COMP BIOCHEM PHYS D, V7, P124, DOI 10.1016/j.cbd.2012.01.001

MICHIELS C, 1994, FREE RADICAL BIO MED, V17, P235, DOI 10.1016/0891-5849(94)90079-5

Mizuguchi S, 2010, FREE RADICAL BIO MED, V49, P1534, DOI 10.1016/j.freeradbiomed.2010.08.017

Onyiah JC, 2013, GASTROENTEROLOGY, V144, P789, DOI 10.1053/j.gastro.2012.12.025

Pietrus Milosz, 2015, Przegl Lek, V72, P482

Qin SY, 2015, INFLAMM RES, V64, P537, DOI 10.1007/s00011-015-0834-9

Ruvolo PP, 1998, J BIOL CHEM, V273, P25436, DOI 10.1074/jbc.273.39.25436

Schipper HM, 1998, EXP NEUROL, V150, P60, DOI 10.1006/exnr.1997.6752

Schipper HM, 1999, NEUROTOX RES, V1, P57, DOI 10.1007/BF03033339

SHIRAGA H, 1993, NEUROCHEM INT, V23, P561, DOI 10.1016/0197-0186(93)90104-D

Soni H, 2011, TOXICOL APPL PHARM, V253, P70, DOI 10.1016/j.taap.2011.03.013

Suliman HB, 2007, J CLIN INVEST, V117, P3730, DOI 10.1172/JCI32967

TENHUNEN R, 1968, P NATL ACAD SCI USA, V61, P748, DOI 10.1073/pnas.61.2.748

Uddin MJ, 2016, CELL MOL IMMUNOL, V13, P170, DOI 10.1038/cmi.2015.02

VERMA A, 1993, SCIENCE, V259, P381, DOI 10.1126/science.7678352

Wegiel B, 2013, CANCER RES, V73, P7009, DOI 10.1158/0008-5472.CAN-13-1075

Wei YW, 2010, BMC GASTROENTEROL, V10, DOI 10.1186/1471-230X-10-42

Xie ZX, 2016, CELL MOL NEUROBIOL, V36, P1343, DOI 10.1007/s10571-016-0333-8

Yang YC, 2014, PLOS ONE, V9, DOI [10.1371/journal.pone.0100677, 10.1371/journal.pone.0087749, 10.1371/journal.pone.0102942, 10.1371/journal.pone.0085863]

Yao L, 2015, J CARDIOVASC PHARM T, V20, P330, DOI 10.1177/1074248414559837

Zhou SN, 2013, CRIT REV ONCOL HEMAT, V88, P706, DOI 10.1016/j.critrevonc.2013.09.001

NR 42

TC 14

Z9 14

U1 1

U2 18

PU KOREAN SOC APPLIED PHARMACOLOGY

PI SEOUL

PA RM 805, KOREAN FEDERATION SCIENCE & TECHNOLOGY B/D, 635-4 YEOKSAM-DONG,

KANGNAM-GU, SEOUL, 135-703, SOUTH KOREA

SN 1976-9148

EI 2005-4483

J9 BIOMOL THER

JI Biomol. Ther.

PD MAR

PY 2018

VL 26

IS 2

BP 175

EP 181

DI 10.4062/biomolther.2018.009

PG 7

WC Biochemistry & Molecular Biology; Pharmacology & Pharmacy

WE Science Citation Index Expanded (SCI-EXPANDED)

SC Biochemistry & Molecular Biology; Pharmacology & Pharmacy

GA FZ7BO

UT WOS:000427754700011

PM 29429149

OA Green Published, Green Submitted

DA 2025-04-09

ER

PT J

AU Quéré, M

Alberto, JM

Broly, F

Hergalant, S

Christov, C

Gauchotte, G

Guéant, JL

Namour, F

Battaglia-Hsu, SF

AF Quere, Maelle

Alberto, Jean-Marc

Broly, Franck

Hergalant, Sebastien

Christov, Christo

Gauchotte, Guillaume

Gueant, Jean-Louis

Namour, Fares

Battaglia-Hsu, Shyue-Fang

TI ALDH1L2 Knockout in U251 Glioblastoma Cells Reduces Tumor Sphere

Formation by Increasing Oxidative Stress and Suppressing Methionine

Dependency

SO NUTRIENTS

LA English

DT Article

DE tumor sphere; glioblastoma; methionine; ROS

ID CANCER STEM-CELLS; METABOLISM; ASSOCIATION; RESISTANCE; FOLATE

AB Previously, the in vitro growth of cancer stem cells in the form of tumor spheres from five different brain cancer cell lines was found to be methionine-dependent. As this earlier work indicated that ALDH1L2, a folate-dependent mitochondria aldehyde dehydrogenase gene, is upregulated in glioblastoma stem cells, we invalidated this gene using CRISPR-cas 9 technique in this present work. We reported here that this invalidation was effective in U251 glioblastoma cells, and no cas9 off target site could be detected by genome sequencing of the two independent knockout targeting either exon I or exon III. The knockout of ALDH1L2 gene in U251 cells rendered the growth of the cancer stem cells of U251 methionine independent. In addition, a much higher ROS (reactive oxygen radicals) level can be detected in the knockout cells compared to the wild type cells. Our evidence here linked the excessive ROS level of the knockout cells to reduced total cellular NADPH. Our evidence suggested also that the cause of the slower growth of the knockout turmor sphere may be related to its partial differentiation.

C1 [Quere, Maelle; Alberto, Jean-Marc; Hergalant, Sebastien; Christov, Christo; Gauchotte, Guillaume; Gueant, Jean-Louis; Namour, Fares; Battaglia-Hsu, Shyue-Fang] Univ Lorraine, INSERM, NGERE Nutr Genet & Environm Risk Exposure, U1256, F-54500 Nancy, France.

[Broly, Franck] Ctr Hosp Reg, Ctr Biol Pathol Genet, Serv Genopathies, F-59037 Lille, France.

[Broly, Franck] Univ Lille, F-59037 Lille, France.

[Gauchotte, Guillaume] CHRU Nancy, Dept Biopathol, Rue Morvan, F-54511 Vandoeuvre Les Nancy, France.

[Gueant, Jean-Louis; Namour, Fares; Battaglia-Hsu, Shyue-Fang] Univ Hosp Nancy, Div Biochem Mol Biol Nutr & Metab, Dept Mol Med, F-54505 Vandoeuvre Les Nancy, France.

C3 Universite de Lorraine; Institut National de la Sante et de la Recherche

Medicale (Inserm); Universite de Lille; CHU Lille; Universite de Lille;

CHU de Nancy; Universite de Lorraine; CHU de Nancy

RP Namour, F; Battaglia-Hsu, SF (corresponding author), Univ Lorraine, INSERM, NGERE Nutr Genet & Environm Risk Exposure, U1256, F-54500 Nancy, France.; Namour, F; Battaglia-Hsu, SF (corresponding author), Univ Hosp Nancy, Div Biochem Mol Biol Nutr & Metab, Dept Mol Med, F-54505 Vandoeuvre Les Nancy, France.

EM maelle.quere@univ-lorraine.fr; jean-marc.alberto@univ-lorraine.fr;

franck.broly@chru-lille.fr; sebastien.hergalant@inserm.fr;

christo.christov@univ-lorraine.fr; guillaume.gauchotte@univ-lorraine.fr;

jean-louis.gueant@univ-lorraine.fr; bernard.namour@univ-lorraine.fr;

shyue-fang.battaglia@univ-lorraine.fr

RI Gueant, Jean-Louis/N-7298-2016; Hergalant, Sebastien/G-8129-2018

OI Gueant, Jean-Louis/0000-0002-5067-042X; ALBERTO,

Jean-Marc/0000-0002-5126-2890; Battaglia-Hsu,

Shyue-fang/0000-0002-5881-6251; Hergalant,

Sebastien/0000-0001-8456-7992; GAUCHOTTE, Guillaume/0000-0001-5585-9195

FU La Ligue Contre Le Cancer, Universite de Lorraine (BMS, projet incitatif

2019); INSERM; Universite de Lorraine

FX We thank Dejian Ren (Department of Biology, University of Pennsylvania)

for his help in the construction of the CRISPR knockouts. We also thank

La Ligue Contre Le Cancer, Universite de Lorraine (BMS, projet incitatif

2019), as well as INSERM for the financial supports. The PhD fellowship

granted to Maelle Quere was given by the Universite de Lorraine

(2018-2021).

CR Bigarella CL, 2014, DEVELOPMENT, V141, P4206, DOI 10.1242/dev.107086

Buccarelli M, 2021, J EXP CLIN CANC RES, V40, DOI 10.1186/s13046-021-02031-4

Chacko BK, 2014, CLIN SCI, V127, P367, DOI 10.1042/CS20140101

Ciavardelli D, 2014, CELL DEATH DIS, V5, DOI 10.1038/cddis.2014.285

Diehn M, 2009, NATURE, V458, P780, DOI 10.1038/nature07733

Duong HQ, 2012, INT J ONCOL, V41, P855, DOI 10.3892/ijo.2012.1516

Emmink BL, 2013, J PROTEOMICS, V91, P84, DOI 10.1016/j.jprot.2013.06.027

Fan J, 2014, NATURE, V510, P298, DOI 10.1038/nature13236

Galluzzi L, 2010, MOL ASPECTS MED, V31, P1, DOI 10.1016/j.mam.2009.08.002

Janiszewska M, 2012, GENE DEV, V26, P1926, DOI 10.1101/gad.188292.112

Ju HQ, 2020, SIGNAL TRANSDUCT TAR, V5, DOI 10.1038/s41392-020-00326-0

Kirsch M, 2001, FASEB J, V15, P1569, DOI 10.1096/fj.00-0823hyp

Kroemer G, 2008, CANCER CELL, V13, P472, DOI 10.1016/j.ccr.2008.05.005

Lamb R, 2015, ONCOTARGET, V6, P4585, DOI 10.18632/oncotarget.3278

Liao NS, 2019, STEM CELL RES THER, V10, DOI 10.1186/s13287-019-1404-9

Loureiro R, 2017, SEMIN CANCER BIOL, V47, P18, DOI 10.1016/j.semcancer.2017.06.012

Mertens J, 2021, CELL STEM CELL, V28, P1533, DOI 10.1016/j.stem.2021.04.004

Muralikrishnan V, 2020, CANCERS, V12, DOI 10.3390/cancers12040961

Piskounova E, 2015, NATURE, V527, P186, DOI 10.1038/nature15726

Pourbagher R, 2021, REP BIOCHEM MOL BIOL, V10, P105, DOI 10.52547/rbmb.10.1.105

Rausch V, 2010, CANCER RES, V70, P5004, DOI 10.1158/0008-5472.CAN-10-0066

Sancho P, 2015, CELL METAB, V22, P590, DOI 10.1016/j.cmet.2015.08.015

Sanderson SM, 2019, SCI ADV, V5, DOI 10.1126/sciadv.aav7769

Sarret C, 2019, NPJ GENOM MED, V4, DOI 10.1038/s41525-019-0092-9

Shiraki N, 2014, CELL METAB, V19, P780, DOI 10.1016/j.cmet.2014.03.017

Tanei T, 2009, CLIN CANCER RES, V15, P4234, DOI 10.1158/1078-0432.CCR-08-1479

Toledano MB, 2015, MOL CELL, V59, P517, DOI 10.1016/j.molcel.2015.08.003

Vassalli G, 2019, STEM CELLS INT, V2019, DOI 10.1155/2019/3904645

Weiswald LB, 2015, NEOPLASIA, V17, P1, DOI 10.1016/j.neo.2014.12.004

Yang M, 2016, NAT REV CANCER, V16, P650, DOI 10.1038/nrc.2016.81

Ye XQ, 2011, INT J CANCER, V129, P820, DOI 10.1002/ijc.25944

Zgheib R, 2019, CELL DEATH DIS, V10, DOI 10.1038/s41419-019-1836-2

Zhou YF, 2011, J BIOL CHEM, V286, P32843, DOI 10.1074/jbc.M111.260935

NR 33

TC 4

Z9 4

U1 0

U2 3

PU MDPI

PI BASEL

PA ST ALBAN-ANLAGE 66, CH-4052 BASEL, SWITZERLAND

EI 2072-6643

J9 NUTRIENTS

JI Nutrients

PD MAY

PY 2022

VL 14

IS 9

AR 1887

DI 10.3390/nu14091887

PG 11

WC Nutrition & Dietetics

WE Science Citation Index Expanded (SCI-EXPANDED)

SC Nutrition & Dietetics

GA 1F8ZH

UT WOS:000795449400001

PM 35565854

OA gold, Green Published

DA 2025-04-09

ER

PT J

AU Kim, TH

Song, J

Kim, SH

Parikh, AK

Mo, XK

Palanichamy, K

Kaur, B

Yu, JH

Yoon, SO

Nakano, I

Kwon, CH

AF Kim, Tae Hyong

Song, Jieun

Kim, Sung-Hak

Parikh, Arav Krishnavadan

Mo, Xiaokui

Palanichamy, Kamalakannan

Kaur, Balveen

Yu, Jianhua

Yoon, Sung Ok

Nakano, Ichiro

Kwon, Chang-Hyuk

TI Piperlongumine treatment inactivates peroxiredoxin 4, exacerbates

endoplasmic reticulum stress, and preferentially kills high-grade glioma

cells

SO NEURO-ONCOLOGY

LA English

DT Article

DE Endoplasmic reticulum stress; high-grade glioma; piperlongumine;

peroxiredoxin 4; reactive oxygen species

ID OXIDATIVE STRESS; CANCER-CELLS; STEM-CELLS; PROLIFERATION; APOPTOSIS;

PEROXIDE; TUMORS; DEATH

AB Backgrounds. Piperlongumine, a natural plant product, kills multiple cancer types with little effect on normal cells. Piperlongumine raises intracellular levels of reactive oxygen species (ROS), a phenomenon that may underlie the cancer-cell killing. Although these findings suggest that piperlongumine could be useful for treating cancers, the mechanism by which the drug selectively kills cancer cells remains unknown.

Methods. We treated multiple high-grade glioma (HGG) sphere cultures with piperlongumine and assessed its effects on ROS and cell-growth levels as well as changes in downstream signaling. We also examined the levels of putative piperlongumine targets and their roles in HGG cell growth.

Results. Piperlongumine treatment increased ROS levels and preferentially killed HGG cells with little effect in normal brain cells. Piperlongumine reportedly increases ROS levels after interactions with several redox regulators. We found that HGG cells expressed higher levels of the putative piperlongumine targets than did normal neural stem cells (NSCs). Furthermore, piperlongumine treatment in HGG cells, but not in normal NSCs, increased oxidative inactivation of peroxiredoxin 4 (PRDX4), an ROS-reducing enzyme that is over-expressed in HGGs and facilitates proper protein folding in the endoplasmic reticulum (ER). Moreover, piperlongumine exacerbated intracellular ER stress, an effect that was mimicked by suppressing PRDX4 expression.

Conclusions. Our results reveal that the mechanism by which piperlongumine preferentially kills HGG cells involves PRDX4 inactivation, thereby inducing ER stress. Therefore, piperlongumine treatment could be considered as a novel therapeutic option for HGG treatment.

C1 [Kim, Tae Hyong; Song, Jieun; Kim, Sung-Hak; Parikh, Arav Krishnavadan; Kaur, Balveen; Nakano, Ichiro; Kwon, Chang-Hyuk] Ohio State Univ, Dept Neurol Surg, Dardinger Neurooncol Ctr, Columbus, OH 43210 USA.

[Kim, Tae Hyong; Song, Jieun; Parikh, Arav Krishnavadan; Kwon, Chang-Hyuk] James Comprehens Canc Ctr, Solid Tumor Program, Columbus, OH USA.

[Mo, Xiaokui] Ohio State Univ, Ctr Biostat, Columbus, OH 43210 USA.

[Palanichamy, Kamalakannan] Ohio State Univ, Dept Radiat Oncol, Columbus, OH 43210 USA.

[Yu, Jianhua] Ohio State Univ, Dept Internal Med, Div Hematol, Columbus, OH 43210 USA.

[Yoon, Sung Ok] Ohio State Univ, Wexner Med Ctr, Dept Mol & Cellular Biochem, Columbus, OH 43210 USA.

C3 University System of Ohio; Ohio State University; James Cancer Hospital

& Solove Research Institute; University System of Ohio; Ohio State

University; University System of Ohio; Ohio State University; University

System of Ohio; Ohio State University; University System of Ohio; Ohio

State University; University System of Ohio; Ohio State University

RP Kwon, CH (corresponding author), 820 Biomed Res Tower,460 W Twelfth Ave, Columbus, OH 43210 USA.

EM chang-hyuk.kwon@osumc.edu

RI Yoon, Sung/C-5992-2012; mo, xiao/KCY-0371-2024; Nakano,

Ichiro/AAR-9562-2020; kaur, Balveen/E-3355-2011; Palanichamy,

Kamalakannan/Z-1376-2019; Wan, Kim/AAI-8744-2020; Kwon,

Chang-hyuk/E-3450-2011; Song, Ji Eun/A-3567-2013; Kaur,

Balveen/LFU-6745-2024

OI Song, Ji Eun/0000-0001-7886-1765; Kaur, Balveen/0000-0001-7738-0804

FU NIH [R01NS064607, R01CA150153, P01 CA163205, P30NS045758]; American

Cancer Society Institutional Seed Grant; Ohio State University (OSU)

Comprehensive Cancer Center (CCC) Intramural Research Program Idea

Grant; OSU CCC Start-up Fund

FX NIH R01NS064607, R01CA150153, P01 CA163205, and P30NS045758 to B. K. and

American Cancer Society Institutional Seed Grant, Ohio State University

(OSU) Comprehensive Cancer Center (CCC) Intramural Research Program Idea

Grant, and OSU CCC Start-up Fund to C.-H.K.

CR Badr CE, 2013, JNCI-J NATL CANCER I, V105, P643, DOI 10.1093/jnci/djt037

Bezerra DP, 2007, TOXICOL IN VITRO, V21, P1, DOI 10.1016/j.tiv.2006.07.007

Bilimoria Parizad M, 2008, CSH Protoc, V2008, DOI 10.1101/pdb.prot5107

Chin L, 2008, NATURE, V455, P1061, DOI 10.1038/nature07385

Day AM, 2012, MOL CELL, V45, P398, DOI 10.1016/j.molcel.2011.11.027

Dittmann LM, 2012, ONCOGENE, V31, P3409, DOI 10.1038/onc.2011.513

Han SS, 2013, LEUKEMIA RES, V37, P146, DOI 10.1016/j.leukres.2012.11.009

HOLM S, 1979, SCAND J STAT, V6, P65

Iuchi Y, 2009, BIOCHEM J, V419, P149, DOI 10.1042/BJ20081526

Jarvis RM, 2012, FREE RADICAL BIO MED, V53, P1522, DOI 10.1016/j.freeradbiomed.2012.08.001

Karihtala P, 2011, BMC CANCER, V11, DOI 10.1186/1471-2407-11-262

Kim TH, 2012, PLOS ONE, V7, DOI 10.1371/journal.pone.0042818

Kwon CH, 2008, CANCER RES, V68, P3286, DOI 10.1158/0008-5472.CAN-07-6867

Lee J, 2006, CANCER CELL, V9, P391, DOI 10.1016/j.ccr.2006.03.030

Liu HL, 2013, J CHROMATOGR B, V928, P78, DOI 10.1016/j.jchromb.2013.03.021

Liu JM, 2013, BIOCHEM BIOPH RES CO, V437, P87, DOI 10.1016/j.bbrc.2013.06.042

Liu LD, 2012, NATURE, V483, P608, DOI 10.1038/nature10927

Liu XD, 2009, CANCER INVEST, V27, P345, DOI 10.1080/07357900802438577

Llaguno SA, 2009, CANCER CELL, V15, P45, DOI 10.1016/j.ccr.2008.12.006

Mao P, 2013, P NATL ACAD SCI USA, V110, P8644, DOI 10.1073/pnas.1221478110

Marciniak SJ, 2004, GENE DEV, V18, P3066, DOI 10.1101/gad.1250704

Papadia S, 2008, NAT NEUROSCI, V11, P476, DOI 10.1038/nn2071

Pennington JD, 2005, DRUG RESIST UPDATE, V8, P322, DOI 10.1016/j.drup.2005.09.002

Pritchard C, 2009, CANCER RES, V69, P1739, DOI 10.1158/0008-5472.CAN-07-6817

Raj L, 2011, NATURE, V475, P231, DOI 10.1038/nature10167

Schildge S, 2013, JOVE-J VIS EXP, DOI 10.3791/50079

Schönthal AH, 2013, BIOCHEM PHARMACOL, V85, P653, DOI 10.1016/j.bcp.2012.09.012

Shah A, 2013, CELL DEATH DIS, V4, DOI 10.1038/cddis.2013.374

Singh SK, 2004, NATURE, V432, P396, DOI 10.1038/nature03128

Souza DG, 2013, PLOS ONE, V8, DOI 10.1371/journal.pone.0060282

Stupp R, 2005, NEW ENGL J MED, V352, P987, DOI 10.1056/NEJMoa043330

Suh DH, 2012, ANN NY ACAD SCI, V1271, P20, DOI 10.1111/j.1749-6632.2012.06739.x

Szegezdi E, 2006, EMBO REP, V7, P880, DOI 10.1038/sj.embor.7400779

Tavender TJ, 2010, J CELL SCI, V123, P2672, DOI 10.1242/jcs.067843

Zhang JY, 2012, PLOS ONE, V7, DOI 10.1371/journal.pone.0040433

Ziech D, 2011, MUTAT RES-FUND MOL M, V711, P167, DOI 10.1016/j.mrfmmm.2011.02.015

Zito E, 2010, MOL CELL, V40, P787, DOI 10.1016/j.molcel.2010.11.010

NR 37

TC 54

Z9 61

U1 8

U2 38

PU OXFORD UNIV PRESS INC

PI CARY

PA JOURNALS DEPT, 2001 EVANS RD, CARY, NC 27513 USA

SN 1522-8517

EI 1523-5866

J9 NEURO-ONCOLOGY

JI Neuro-Oncology

PD OCT

PY 2014

VL 16

IS 10

BP 1354

EP 1364

DI 10.1093/neuonc/nou088

PG 11

WC Oncology; Clinical Neurology

WE Science Citation Index Expanded (SCI-EXPANDED)

SC Oncology; Neurosciences & Neurology

GA AR6CP

UT WOS:000343671000007

PM 24879047

OA Green Published, Bronze

DA 2025-04-09

ER

PT J

AU Zhang, L

Fu, C

Li, J

Zhao, ZZ

Hou, YX

Zhou, W

Fu, AL

AF Zhang, Le

Fu, Chen

Li, Jin

Zhao, Zizhen

Hou, Yixue

Zhou, Wei

Fu, Ailing

TI Discovery of a Ruthenium Complex for the Theranosis of Glioma through

Targeting the Mitochondrial DNA with Bioinformatic Methods

SO INTERNATIONAL JOURNAL OF MOLECULAR SCIENCES

LA English

DT Article

DE organometallic complexes; glioma; mtDNA mutation; computation docking

ID SENSITIVITY; METABOLISM; SPEED

AB Glioma is the most aggressive and lethal brain tumor in humans. Mutations of mitochondrial DNA (mtDNA) are commonly found in tumor cells and are closely associated with tumorigenesis and progress. However, glioma-specific inhibitors that reflect the unique feature of tumor cells are rare. Here we uncover RC-7, a ruthenium complex with strong red fluorescence, could bind with glioma mtDNA and then inhibited the growth of human glioma cells but not that of neuronal cells, liver, or endothelial cells. RC-7 significantly reduced energy production and increased the oxidative stress in the glioma cells. Administration of RC-7 into mice not only could be observed in the glioma mass of brain by fluorescence imaging, but also obviously prevented the growth of xenograft glioma and prolonged mouse survival days. The findings suggested the theranostic application of a novel type of complex through targeting the tumor mtDNA.

C1 [Zhang, Le; Li, Jin] Southwest Univ, Coll Comp & Informat Sci, Chongqing 400715, Peoples R China.

[Zhang, Le] Sichuan Univ, Coll Comp Sci, Chengdu 610065, Sichuan, Peoples R China.

[Fu, Chen; Zhao, Zizhen; Hou, Yixue; Zhou, Wei; Fu, Ailing] Southwest Univ, Coll Pharmaceut Sci, Chongqing 400715, Peoples R China.

C3 Southwest University - China; Sichuan University; Southwest University -

China

RP Fu, AL (corresponding author), Southwest Univ, Coll Pharmaceut Sci, Chongqing 400715, Peoples R China.

EM zhangle06@scu.edu.cn; fuchen0794@swu.edu.cn; eddyblue@swu.edu.cn;

zhaozizhen0512@hotmail.com; yixue6577@163.com; zw2678615937@163.com;

fal@swu.edu.cn

RI Zhao, Zizhen/MNO-2162-2025; Zhang, Le/AAD-9104-2019

OI Zhang, Le/0000-0002-3708-1727; Fu, Chen/0000-0002-9924-0797; ZHAO,

ZiZhen/0000-0001-9729-644X

FU Natural Science Foundation of China [61372138, 21601146, 81501002];

National Science and Technology Major Project; Chinese Chongqing

Distinguish Youth Funding; Chongqing Research Program of Basic Research

and Frontier Technology [cstc2018jcyjAX0612]; Major Innovation Project

of Southwest Hospital [SWH2016ZDCX1011]; National Science and Technology

Major Project [2018ZX10201002]; Chinese Chongqing Distinguish Youth

Funding [cstc2014jcyjjq40003]

FX The work was funded by the Natural Science Foundation of China

(61372138, 21601146, 81501002), the National Science and Technology

Major Project and Chinese Chongqing Distinguish Youth Funding. This work

was also funded by Chongqing Research Program of Basic Research and

Frontier Technology (No. cstc2018jcyjAX0612), and the Major Innovation

Project of Southwest Hospital (No. SWH2016ZDCX1011), the National

Science and Technology Major Project (2018ZX10201002), and Chinese

Chongqing Distinguish Youth Funding (cstc2014jcyjjq40003].

CR Bachmann M, 2018, INT J MOL SCI, V19, DOI 10.3390/ijms19072060

Boynton AN, 2016, J AM CHEM SOC, V138, P5020, DOI 10.1021/jacs.6b02022

Cahill D, 2018, SEMIN NEUROL, V38, P5, DOI 10.1055/s-0037-1620238

Chen R, 2017, NEUROTHERAPEUTICS, V14, P284, DOI 10.1007/s13311-017-0519-x

Chu Y, 2019, BIOCONJUGATE CHEM, V30, P1642, DOI 10.1021/acs.bioconjchem.9b00375

Coe BJ, 2000, COORDIN CHEM REV, V203, P5, DOI 10.1016/S0010-8545(99)00184-8

Devall M, 2015, BIOTECHNIQUES, V59, P241, DOI 10.2144/000114343

Du Q, 2019, INORG CHEM, V58, P5956, DOI 10.1021/acs.inorgchem.9b00282

Ernst RJ, 2011, BIOCHEMISTRY-US, V50, P10919, DOI 10.1021/bi2015822

Ganeshpandian M, 2014, DALTON T, V43, P1203, DOI 10.1039/c3dt51641e

Guntuku L, 2016, CURR NEUROPHARMACOL, V14, P567, DOI 10.2174/1570159X14666160121115641

Gusyatiner O, 2018, SEMIN CANCER BIOL, V51, P50, DOI 10.1016/j.semcancer.2017.11.010

Herst PM, 2018, CANCER METAST REV, V37, P643, DOI 10.1007/s10555-018-9769-2

Hopkins JF, 2018, GASTROENTEROLOGY, V154, P1620, DOI 10.1053/j.gastro.2018.01.029

Jiang GB, 2019, SPECTROCHIM ACTA A, V220, DOI 10.1016/j.saa.2019.05.037

Kalyanaraman B, 2018, REDOX BIOL, V14, P316, DOI 10.1016/j.redox.2017.09.020

Karges J, 2019, J INORG BIOCHEM, V198, DOI 10.1016/j.jinorgbio.2019.110752

Li DY, 2017, J EXP CLIN CANC RES, V36, DOI 10.1186/s13046-017-0638-6

Li GY, 2016, DALTON T, V45, P13261, DOI 10.1039/c6dt01624c

Liu CA, 2018, INT J MOL SCI, V19, DOI 10.3390/ijms19041115

Lu QR, 2019, WIRES DEV BIOL, V8, DOI 10.1002/wdev.342

Ma DL, 2019, MOLECULES, V24, DOI 10.3390/molecules24152739

Ma DL, 2016, DALTON T, V45, P2762, DOI 10.1039/c5dt04338g

Mirzaei H, 2015, J CHEM THEORY COMPUT, V11, P1063, DOI 10.1021/ct500155t

Montanini L, 2005, J NEURO-ONCOL, V74, P87, DOI 10.1007/s11060-004-4036-5

Nano A, 2017, J AM CHEM SOC, V139, P17301, DOI 10.1021/jacs.7b10639

Neagu M, 2019, FRONT ONCOL, V9, DOI 10.3389/fonc.2019.00348

Ohata J, 2018, DALTON T, V47, P14855, DOI 10.1039/c8dt03032d

Perrone AM, 2018, INT J MOL SCI, V19, DOI 10.3390/ijms19072048

Peters U, 2003, ANTICANCER RES, V23, P1249

Qian MJ, 2017, ADV EXP MED BIOL, V1038, P23, DOI 10.1007/978-981-10-6674-0_3

Riccardi C, 2017, EUR J ORG CHEM, V2017, P1100, DOI 10.1002/ejoc.201600943

Shiomi T, 2006, EUR J ORG CHEM, V2006, P5594, DOI 10.1002/ejoc.200600722

Song XD, 2017, EUR J MED CHEM, V138, P246, DOI 10.1016/j.ejmech.2017.06.038

Sun B, 2018, TALANTA, V179, P658, DOI 10.1016/j.talanta.2017.11.047

Sun C, 2019, THERANOSTICS, V9, P3595, DOI 10.7150/thno.33100

Thota S, 2018, J MED CHEM, V61, P5805, DOI 10.1021/acs.jmedchem.7b01689

Trott O, 2010, J COMPUT CHEM, V31, P455, DOI 10.1002/jcc.21334

Weidmann AG, 2014, COMMENT INORG CHEM, V34, P114, DOI 10.1080/02603594.2014.890099

Wumaier M, 2019, J INORG BIOCHEM, V196, DOI 10.1016/j.jinorgbio.2019.03.021

Yeung KY, 2014, ACTA NEUROPATHOL COM, V2, DOI 10.1186/2051-5960-2-1

Yusoff AAM, 2017, ONCOL LETT, V14, P5179, DOI 10.3892/ol.2017.6851

Zhao M, 2018, INT J NANOMED, V13, P1601, DOI 10.2147/IJN.S157019

Zong WX, 2016, MOL CELL, V61, P667, DOI 10.1016/j.molcel.2016.02.011

NR 44

TC 13

Z9 15

U1 0

U2 18

PU MDPI

PI BASEL

PA ST ALBAN-ANLAGE 66, CH-4052 BASEL, SWITZERLAND

EI 1422-0067

J9 INT J MOL SCI

JI Int. J. Mol. Sci.

PD SEP 2

PY 2019

VL 20

IS 18

AR 4643

DI 10.3390/ijms20184643

PG 16

WC Biochemistry & Molecular Biology; Chemistry, Multidisciplinary

WE Science Citation Index Expanded (SCI-EXPANDED)

SC Biochemistry & Molecular Biology; Chemistry

GA JC2IK

UT WOS:000489100500322

PM 31546801

OA Green Published, gold

DA 2025-04-09

ER

PT J

AU Colovic, M

Yang, H

Merkens, H

Colpo, N

Bénard, F

Schaffer, P

AF Colovic, Milena

Yang, Hua

Merkens, Helen

Colpo, Nadine

Benard, Francois

Schaffer, Paul

TI The Effect of Chirality on the Application of

5-[<SUP>18</SUP>F]Fluoro-Aminosuberic Acid ([<SUP>18</SUP>F]FASu) for

Oxidative Stress Imaging

SO MOLECULAR IMAGING AND BIOLOGY

LA English

DT Article

DE Oxidative stress imaging; Cystine transporter; FASu; Fluoroaminosuberic

acid; Chirality; Isomers; Cancer PET imaging; Prostate cancer;

Glioblastoma; TNBC

ID POSITRON-EMISSION-TOMOGRAPHY; CYSTINE/GLUTAMATE ANTIPORTER; AMINO-ACIDS;

D-ISOMERS; TRANSPORT; EXPRESSION; LAT1; BIODISTRIBUTION; INDUCTION; XCT

AB Purpose The cystine transporter, system x(C)(-), plays a crucial role in sustaining redox homeostasis and is reported to be overexpressed in several cancer subtypes. 5-[F-18]Fluoroaminosuberic acid ([F-18]FASu) is a novel positron emission tomography (PET) tracer, which exhibits specific uptake via system x(C)(-). [F-18]FASu synthesis by the commonly used Kryptofix 2.2.2/K2CO3-facilitated fluorination method results in four diastereomers, as a result of 2 chiral centers at positions 2- and 5- of the tracer. We recently reported the synthesis of the optically pure 2S-[F-18]FASu from chiral precursors. Our preliminary results indicated preferential uptake of the 2S-isomer by tumor cells compared to 2R-[F-18]FASu. Few studies have investigated the biodistribution of chiral F-18-labeled amino acids. The aim of this study was to evaluate the imaging utility and biodistribution of the 5-position diastereomers as well as the racemic (2S,5R/S-) mixture in three different tumor models. Procedures In vitro tracer uptake experiments and Western blotting were performed in breast cancer (MDA-MB-231), glioblastoma (U-87), and prostate (PC-3) cancer cell lines. PET imaging and biodistribution studies were conducted in xenograft-bearing immunocompromised Rag2M female mice. Results All three tracer conformations allowed for the visualization of tumor xenografts at 1 h (for U-87 and PC-3 tumors) or 2 h (in the case of MDA-MB-231 xenografts) post-injection, with the racemate (2S,5R/S-) displaying similar image contrast as compared to the 5- position diastereomers and the 2S,5S-[F-18]FASu conformation exhibiting relatively higher contrast for imaging U-87 and PC-3 xenografts. Tumor uptake of the isomers was blocked by an excess of the non-radioactive standard, aminosuberic acid (ASu), confirming target specificity. All three isomers were excreted via the renal pathway. Biodistribution analyses showed that PC-3 tumors had the highest tracer uptake, and the accumulation (%ID/g) of the 2S,5R/S-, 2S,5S-, and 2S,5R- isomers was 9.19 +/- 1.14, 8.00 +/- 1.41, and 7.16 +/- 2.13 at 1 h post-injection, respectively. This gave corresponding tumor-to-muscle ratios of 33.68 +/- 9.52, 31.42 +/- 4.54, and 25.33 +/- 4.97, respectively. Conclusion Our data suggest that pure 2S-[F-18]FASu can be used to noninvasively image system x(C)(-) in a variety of cancers, either as the racemic mixture (2S,5R/S-) or optically pure form. Furthermore, this work shows potential utility of [F-18]FASu for detection of glioblastoma and prostate cancer.

C1 [Colovic, Milena; Yang, Hua; Schaffer, Paul] TRIUMF, Life Sci Div, 4004 Wesbrook Mall, Vancouver, BC V6T 2A3, Canada.

[Colovic, Milena; Merkens, Helen; Colpo, Nadine; Benard, Francois] British Columbia Canc Res Ctr, Mol Oncol, 675 W 10th Ave, Vancouver, BC V5Z 1L3, Canada.

[Benard, Francois; Schaffer, Paul] Univ British Columbia, Fac Med, Dept Radiol, Vancouver, BC, Canada.

[Schaffer, Paul] Simon Fraser Univ, Fac Sci, Dept Chem, Vancouver, BC, Canada.

C3 University of British Columbia; British Columbia Cancer Agency;

University of British Columbia; Simon Fraser University

RP Schaffer, P (corresponding author), TRIUMF, Life Sci Div, 4004 Wesbrook Mall, Vancouver, BC V6T 2A3, Canada.; Bénard, F (corresponding author), British Columbia Canc Res Ctr, Mol Oncol, 675 W 10th Ave, Vancouver, BC V5Z 1L3, Canada.; Bénard, F; Schaffer, P (corresponding author), Univ British Columbia, Fac Med, Dept Radiol, Vancouver, BC, Canada.; Schaffer, P (corresponding author), Simon Fraser Univ, Fac Sci, Dept Chem, Vancouver, BC, Canada.

EM fbenard@bccrc.ca; pschaffer@triumf.ca

RI Schaffer, Paul/AAW-7654-2021; Benard, Francois/M-7720-2015

OI Benard, Francois/0000-0001-7995-3581; Colovic,

Milena/0000-0001-8686-0084

FU CIHR [201403COP, 329895]; NSERC CREATE IsoSiM fellowship [448110]; BC

Leading Edge Endowment Fund; National Research Council of Canada

FX This study is financially supported by the CIHR (201403COP, 329895).

TRIUMF receives federal funding via a contribution agreement with the

National Research Council of Canada. M.C. is supported by the NSERC

CREATE IsoSiM fellowship, grant no. 448110, competition year 2014. This

work was supported in part by the BC Leading Edge Endowment Fund.

CR Altan B, 2018, CANCER CHEMOTH PHARM, V81, P141, DOI 10.1007/s00280-017-3477-4

Bauwens M, 2007, NUCL MED COMMUN, V28, P823, DOI 10.1097/MNM.0b013e3282e7d731

Bridges RJ, 2012, BRIT J PHARMACOL, V165, P20, DOI 10.1111/j.1476-5381.2011.01480.x

Colovic M, 2019, MOL IMAGING BIOL, V21, P1107, DOI 10.1007/s11307-019-01331-8

Colovic M, 2018, BIOORG MED CHEM LETT, V28, P3579, DOI 10.1016/j.bmcl.2018.06.014

Conrad M, 2012, AMINO ACIDS, V42, P231, DOI 10.1007/s00726-011-0867-5

Ganapathy V, 2009, PHARMACOL THERAPEUT, V121, P29, DOI 10.1016/j.pharmthera.2008.09.005

Heiss P, 1999, J NUCL MED, V40, P1367

Huang CF, 2013, J NUCL MED, V54, P1007, DOI 10.2967/jnumed.112.113100

ISHII T, 1992, ANN NY ACAD SCI, V663, P497, DOI 10.1111/j.1749-6632.1992.tb38714.x

Kersemans V, 2006, EUR J NUCL MED MOL I, V33, P919, DOI 10.1007/s00259-005-0043-9

Langen KJ, 2005, J CEREBR BLOOD F MET, V25, P607, DOI 10.1038/sj.jcbfm.9600065

Liu F, 2018, MOL PHARMACEUT, V15, P3448, DOI 10.1021/acs.molpharmaceut.8b00430

Martarello L, 2002, J MED CHEM, V45, P2250, DOI 10.1021/jm010242p

McConathy J, 2008, CANCER METAST REV, V27, P555, DOI 10.1007/s10555-008-9154-7

Nakanishi T, 2011, J PHARM SCI-US, V100, P3731, DOI 10.1002/jps.22576

Piroth MD, 2011, INT J RADIAT ONCOL, V80, P176, DOI 10.1016/j.ijrobp.2010.01.055

Qu WC, 2011, J AM CHEM SOC, V133, P1122, DOI 10.1021/ja109203d

Sasaki H, 2002, J BIOL CHEM, V277, P44765, DOI 10.1074/jbc.M208704200

Timmerman LA, 2013, CANCER CELL, V24, P450, DOI 10.1016/j.ccr.2013.08.020

Toyoda M, 2014, BRIT J CANCER, V110, P2506, DOI 10.1038/bjc.2014.178

Tsukada H, 2006, EUR J NUCL MED MOL I, V33, P1017, DOI 10.1007/s00259-006-0076-8

Urakami T, 2009, NUCL MED BIOL, V36, P295, DOI 10.1016/j.nucmedbio.2008.12.012

Wang Q, 2015, AM J CANCER RES, V5, P1281

Webster JM, 2014, J NUCL MED, V55, P657, DOI 10.2967/jnumed.113.126664

Yanagisawa N, 2012, J CLIN PATHOL, V65, P1019, DOI 10.1136/jclinpath-2012-200826

Yang H, 2017, CHEM-EUR J, V23, P11100, DOI 10.1002/chem.201702007

Yang H, 2017, J NUCL MED, V58, P367, DOI 10.2967/jnumed.116.180661

Zhang ZW, 2018, J RADIOANAL NUCL CH, V316, P153, DOI 10.1007/s10967-018-5753-0

NR 29

TC 6

Z9 6

U1 0

U2 7

PU SPRINGER

PI NEW YORK

PA ONE NEW YORK PLAZA, SUITE 4600, NEW YORK, NY, UNITED STATES

SN 1536-1632

EI 1860-2002

J9 MOL IMAGING BIOL

JI Mol. Imaging. Biol.

PD AUG

PY 2020

VL 22

IS 4

BP 873

EP 882

DI 10.1007/s11307-019-01450-2

EA DEC 2019

PG 10

WC Radiology, Nuclear Medicine & Medical Imaging

WE Science Citation Index Expanded (SCI-EXPANDED)

SC Radiology, Nuclear Medicine & Medical Imaging

GA MH4UD

UT WOS:000499965700004

PM 31792837

DA 2025-04-09

ER

PT J

AU Asemi, Z

Behnam, M

Pourattar, MA

Mirzaei, H

Razavi, ZS

Tamtaji, OR

AF Asemi, Zatollah

Behnam, Mohammad

Pourattar, Mohammad Ali

Mirzaei, Hamed

Razavi, Zahra Sadat

Tamtaji, Omid Reza

TI Therapeutic Potential of Berberine in the Treatment of Glioma: Insights

into Its Regulatory Mechanisms

SO CELLULAR AND MOLECULAR NEUROBIOLOGY

LA English

DT Review

DE Berberine; Glioma; Apoptosis; Autophagy; Cell cycle arrest

ID CELL-CYCLE ARREST; N-ACETYLTRANSFERASE ACTIVITY; GLIOBLASTOMA T98G

CELLS; MALIGNANT GLIOMA; APOPTOSIS PATHWAY; GENE-EXPRESSION; CANCER

CELLS; AUTOPHAGY; INHIBITION; DEATH

AB Glioma is known as one of the most common primary intracranial tumors accounting for four-fifths of malignant brain tumors. There are several biological pathways that play a synergistic, pathophysiological role in glioma, including apoptosis, autophagy, oxidative stress, and cell cycle arrest. According to previous rese arches, the drugs used in the treatment of glioma have been associated with significant limitations. Therefore, improved and/or new therapeutic platforms are required. In this regard, multiple flavonoids and alkaloids have been extensively studied in the treatment of glioma. Berberine is a protoberberine alkaloid with wide range of pharmacological activities, applicable to various pathological conditions. Few studies have reported beneficial roles of berberine in glioma. Berberine exerts its pharmacological functions in glioma by controlling different molecular and cellular pathways. We reviewed the existing knowledge supporting the use of berberine in the treatment of glioma and its effects on molecular and cellular mechanisms.

C1 [Asemi, Zatollah; Mirzaei, Hamed; Tamtaji, Omid Reza] Kashan Univ Med Sci, Inst Basic Sci, Res Ctr Biochem & Nutr Metab Dis, Kashan, Iran.

[Behnam, Mohammad] FDA, Halal Res Ctr IRI, Tehran, Iran.

[Pourattar, Mohammad Ali] Iran Univ Med Sci, Dept Radiobiol, Tehran, Iran.

[Razavi, Zahra Sadat] Kashan Univ Med Sci, Student Res Comm, Kashan, Iran.

C3 Iran University of Medical Sciences

RP Tamtaji, OR (corresponding author), Kashan Univ Med Sci, Inst Basic Sci, Res Ctr Biochem & Nutr Metab Dis, Kashan, Iran.

EM Tamtaji.or@gmail.com

RI asemi, zatollah/J-2677-2018; mirzaei, hamed/X-2374-2018; tamtaji,

M/KWU-3655-2024

CR Annovazzi L, 2009, ANTICANCER RES, V29, P3087

Ashkenazi A, 2008, CYTOKINE GROWTH F R, V19, P325, DOI 10.1016/j.cytogfr.2008.04.001

Badiga AV, 2011, PLOS ONE, V6, DOI 10.1371/journal.pone.0020614

Balmanno K, 2009, CELL DEATH DIFFER, V16, P368, DOI 10.1038/cdd.2008.148

Besson A, 2001, ONCOGENE, V20, P7398, DOI 10.1038/sj.onc.1204899

Bhutada P, 2010, EPILEPSY BEHAV, V18, P207, DOI 10.1016/j.yebeh.2010.03.007

Butcher NJ, 2012, PHARMACOL REV, V64, P147, DOI 10.1124/pr.110.004275

Chen DB, 2003, MOL CELL ENDOCRINOL, V200, P141, DOI 10.1016/S0303-7207(02)00379-9

CHEN KT, 1994, CHINESE MED J-PEKING, V107, P808

Chen TC, 2009, INT J ONCOL, V34, P1681, DOI 10.3892/ijo_00000299

Cholia RP, 2018, METAB BRAIN DIS, V33, P1307, DOI 10.1007/s11011-018-0233-3

Chung JG, 1999, FOOD CHEM TOXICOL, V37, P319, DOI 10.1016/S0278-6915(99)00016-2

da Silva AB, 2020, BRAIN BEHAV IMMUN, V85, P170, DOI 10.1016/j.bbi.2019.05.003

De Luca A, 2012, EXPERT OPIN THER TAR, V16, pS17, DOI 10.1517/14728222.2011.639361

Delaney G, 2005, CANCER-AM CANCER SOC, V104, P1129, DOI 10.1002/cncr.21324

Dong C, 2002, ANNU REV IMMUNOL, V20, P55, DOI 10.1146/annurev.immunol.20.091301.131133

Durairajan SSK, 2012, NEUROBIOL AGING, V33, P2903, DOI 10.1016/j.neurobiolaging.2012.02.016

Egeblad M, 2002, NAT REV CANCER, V2, P161, DOI 10.1038/nrc745

Eom KS, 2010, BIOL PHARM BULL, V33, P1644, DOI 10.1248/bpb.33.1644

Eom KS, 2008, BIOL PHARM BULL, V31, P558, DOI 10.1248/bpb.31.558

Fan QW, 2011, AUTOPHAGY, V7, P536, DOI 10.4161/auto.7.5.14779

Filippi-Chiela EC, 2011, PLOS ONE, V6, DOI 10.1371/journal.pone.0020849

Forsyth PA, 1999, BRIT J CANCER, V79, P1828, DOI 10.1038/sj.bjc.6690291

Fukuda K, 1999, J ETHNOPHARMACOL, V66, P227, DOI 10.1016/S0378-8741(98)00162-7

Fulda S, 2006, ONCOGENE, V25, P4798, DOI 10.1038/sj.onc.1209608

Ghavami S, 2009, J MED GENET, V46, P497, DOI 10.1136/jmg.2009.066944

Guo YW, 2019, FRONT MOL NEUROSCI, V12, DOI 10.3389/fnmol.2019.00125

Howlader N., 2011, SEER cancer statistics review, 1975-2008

Jacques-Silva MC, 2004, ONCOLOGY-BASEL, V67, P450, DOI 10.1159/000082930

Kabir S, 2018, J CANC RES PRACT, V5, P131, DOI [10.1016/j.jcrpr.2018.07.001, DOI 10.1016/J.JCRPR.2018.07.001]

Kan LK, 2019, J NEUROIMMUNOL, V332, P138, DOI 10.1016/j.jneuroim.2019.04.010

Kang R, 2011, CELL DEATH DIFFER, V18, P571, DOI 10.1038/cdd.2010.191

Kim HS, 2010, HORM METAB RES, V42, P165, DOI 10.1055/s-0029-1243190

Kim M, 2014, INT J MOL MED, V33, P870, DOI 10.3892/ijmm.2014.1656

Kim S, 2012, J SURG RES, V176, pE21, DOI 10.1016/j.jss.2011.11.1041

Kim S, 2008, MOLECULES, V13, P2975, DOI 10.3390/molecules13122975

Kondo Y, 2005, NAT REV CANCER, V5, P726, DOI 10.1038/nrc1692

Laperriere N, 2002, RADIOTHER ONCOL, V64, P259, DOI 10.1016/S0167-8140(02)00078-6

Lee WC, 2005, NEUROCHEM RES, V30, P263, DOI 10.1007/s11064-005-2449-y

Leist M, 1997, MOL MED, V3, P750, DOI 10.1007/BF03401713

Li TY, 2012, CELL, V149, P1269, DOI 10.1016/j.cell.2012.04.026

Lin CC, 2005, ANTICANCER RES, V25, P4149

Lin JP, 2008, IN VIVO, V22, P223

Lin JP, 2006, WORLD J GASTROENTERO, V12, P21, DOI 10.3748/wjg.v12.i1.21

Lin TH, 2008, BMC CANCER, V8, DOI 10.1186/1471-2407-8-58

Liu Q, 2015, MOL CANCER THER, V14, P355, DOI 10.1158/1535-7163.MCT-14-0634

Liu Q, 2011, PLOS ONE, V6, DOI 10.1371/journal.pone.0023427

Luo SL, 2018, OXID MED CELL LONGEV, V2018, DOI 10.1155/2018/9146528

Mantena SK, 2006, MOL CANCER THER, V5, P296, DOI 10.1158/1535-7163.MCT-05-0448

Marconi GD, 2019, MOLECULES, V24, DOI 10.3390/molecules24102005

Masoudi MS, 2018, J CELL BIOCHEM, V119, P1285, DOI 10.1002/jcb.26300

Meier F, 2005, FRONT BIOSCI-LANDMRK, V10, P2986, DOI 10.2741/1755

Mizushima N, 2007, GENE DEV, V21, P2861, DOI 10.1101/gad.1599207

Mooney J, 2019, WORLD NEUROSURG

Nyormoi O, 2003, CELL DEATH DIFFER, V10, P558, DOI 10.1038/sj.cdd.4401209

Ostrom QT, 2017, NEURO-ONCOLOGY, V19, pV1, DOI 10.1093/neuonc/nox158

Palumbo S, 2012, J CELL BIOCHEM, V113, P2308, DOI 10.1002/jcb.24102

PARDEE AB, 1989, SCIENCE, V246, P603, DOI 10.1126/science.2683075

Pece S, 2000, J BIOL CHEM, V275, P41227, DOI 10.1074/jbc.M006578200

Peng PL, 2008, INT J RADIAT ONCOL, V70, P529, DOI 10.1016/j.ijrobp.2007.08.034

Picart T, 2019, NEUROCHIRURGIE, V65, P164, DOI 10.1016/j.neuchi.2019.04.005

Russell RC, 2013, NAT CELL BIOL, V15, P741, DOI 10.1038/ncb2757

Saadatpour L, 2016, CANCER GENE THER, V23, P415, DOI 10.1038/cgt.2016.48

Sawaya RE, 1996, CLIN EXP METASTAS, V14, P35, DOI 10.1007/BF00157684

Schild L, 2010, PHYTOMEDICINE, V17, P589, DOI 10.1016/j.phymed.2009.12.002

SEE WL, 2018, HDB BRAIN TUMOR CHEM, P323

Senft C, 2011, LANCET ONCOL, V12, P997, DOI 10.1016/S1470-2045(11)70196-6

Sim E, 2014, BRIT J PHARMACOL, V171, P2705, DOI 10.1111/bph.12598

Song JH, 2003, BRAIN PATHOL, V13, P539

Stupp R, 2009, LANCET ONCOL, V10, P459, DOI 10.1016/S1470-2045(09)70025-7

Sun YX, 2018, BIOMED PHARMACOTHER, V102, P699, DOI 10.1016/j.biopha.2018.03.132

SYMONDS H, 1994, CELL, V78, P703, DOI 10.1016/0092-8674(94)90534-7

Thorns V, 2003, ANTICANCER RES, V23, P3937

Tian CH, 2019, INT J BIOL MACROMOL, V136, P143, DOI 10.1016/j.ijbiomac.2019.06.060

Tong L, 2019, FRONT ONCOL, V9, DOI 10.3389/fonc.2019.00364

Ulasov I, 2013, CANCER MED-US, V2, P457, DOI 10.1002/cam4.104

WALKER MD, 1980, NEW ENGL J MED, V303, P1323, DOI 10.1056/NEJM198012043032303

Wang DY, 2002, NEUROCHEM RES, V27, P883, DOI 10.1023/A:1020335430016

Wang JW, 2016, ONCOTARGET, V7, P66944, DOI 10.18632/oncotarget.11396

Wang N, 2010, J CELL BIOCHEM, V111, P1426, DOI 10.1002/jcb.22869

Windmill KF, 2000, TOXICOL SCI, V54, P19, DOI 10.1093/toxsci/54.1.19

Wong RSY, 2011, J EXP CLIN CANC RES, V30, DOI 10.1186/1756-9966-30-87

Wu CC, 2013, ANTIOXID REDOX SIGN, V19, P546, DOI 10.1089/ars.2012.4905

Wu HL, 2019, J AGR FOOD CHEM, V67, P2212, DOI 10.1021/acs.jafc.8b07126

Wu YT, 2009, AUTOPHAGY, V5, P824, DOI 10.4161/auto.9099

Yount Garret, 2004, J Exp Ther Oncol, V4, P137

Yu R, 2014, CANCER CELL INT, V14, DOI 10.1186/1475-2867-14-49

Zou YH, 2014, MOL MED REP, V10, P411, DOI 10.3892/mmr.2014.2151

NR 88

TC 12

Z9 12

U1 2

U2 14

PU SPRINGER/PLENUM PUBLISHERS

PI NEW YORK

PA 233 SPRING ST, NEW YORK, NY 10013 USA

SN 0272-4340

EI 1573-6830

J9 CELL MOL NEUROBIOL

JI Cell. Mol. Neurobiol.

PD AUG

PY 2021

VL 41

IS 6

BP 1195

EP 1201

DI 10.1007/s10571-020-00903-5

EA JUN 2020

PG 7

WC Cell Biology; Neurosciences

WE Science Citation Index Expanded (SCI-EXPANDED)

SC Cell Biology; Neurosciences & Neurology

GA SY4EK

UT WOS:000541219000001

PM 32557203

DA 2025-04-09

ER

PT J

AU Tai, GM

Gu, P

Zhang, HW

He, XJ

Du, J

Huang, JF

Yu, JH

Cai, J

Liu, FJ

AF Tai, Guomei

Gu, Pei

Zhang, Haowen

He, Xiaojun

Du, Jie

Huang, Jianfeng

Yu, Jiahua

Cai, Jing

Liu, Fenju

TI TP53 induced glycolysis and apoptosis regulator knockdown

radiosensitizes glioma cells through oxidative stress-induced excessive

autophagy

SO INTERNATIONAL JOURNAL OF CLINICAL AND EXPERIMENTAL PATHOLOGY

LA English

DT Article

DE TIGAR; NADPH; autophagy; glioma; radiosensitization

ID TIGAR; P53

AB TP53 induced glycolysis and apoptosis regulator (TIGAR) knockdown has been suggested to be a feasible solution to radiosensitize glioma cells. The mechanisms inside it were not fully understood. In this study, human malignant glioma cells, A172 and T98G, were treated with TIGAR siRNA and the relationship between TIGAR interfering-induced radiosensitization and autophagy promotion in irradiated glioma cells was investigated. In order to explore whether TIGAR knockdown-promoted autophagy was protective or not, glioma cells were treated with 3-methyladenine. It was indicated that inhibition of autophagy diminished the radiosensitizing effect of TIGAR knockdown. NADPH content was determined to demonstrate the mechanism of TIGAR interfering-induced autophagy in irradiated glioma cells. It was revealed that N-Acetylcysteine treatment abrogated TIGAR knockdown-induced NADPH depletion and inhibited the autophagy activity, while buthionine sulfoximine further increased the autophagy flux in cells suffered by TIGAR interfering and irradiation. In conclusion, our data revealed an excessive autophagy was induced by TIGAR knockdown in irradiated glioma cells, which was dependent on NADPH depletion.

C1 [Tai, Guomei; Gu, Pei; Zhang, Haowen; Du, Jie; Huang, Jianfeng; Yu, Jiahua; Liu, Fenju] Soochow Univ, Coll Med,CICRM, Sch Radiat Med & Protect,Jiangsu Higher Educ Inst, Dept Radiobiol,Sch Radiol & Interdisciplinary Sci, Suzhou, Peoples R China.

[Tai, Guomei; Gu, Pei; He, Xiaojun; Cai, Jing] Nantong Univ, Affiliated Tumor Hosp, Nantong Tumor Hosp, Dept Radiat Oncol, Nantong 226321, Peoples R China.

C3 Soochow University - China; Nantong University

RP Cai, J (corresponding author), Nantong Univ, Affiliated Tumor Hosp, Nantong Tumor Hosp, Dept Radiat Oncol, Nantong 226321, Peoples R China.; Liu, FJ (corresponding author), Soochow Univ, Sch Radiol & Interdisciplinary Sci, Sch Radiat Med & Protect, Dept Radiobiol,Coll Med, Suzhou 215123, Peoples R China.

EM cj7227@sina.com; fangsh@suda.edu.cn

RI HE, Xiaojun/D-8511-2012; Cai, Jing/LMN-7521-2024; zhang,

haowen/JNE-7429-2023

FU National Science Foundation of China [31270897, 81271682]; Graduate

Education Innovation Project of Jiangsu Province; Priority Academic

Program Development of Jiangsu Higher Education Institutions (PAPD);

Collaborative Innovation Center of Radiological Medicine of Jiangsu

Higher Education Institutions; Nantong science foundation of health

bureau for youth [WQ2014045]

FX This work was supported by grants from the National Science Foundation

of China (No. 31270897 and 81271682), Graduate Education Innovation

Project of Jiangsu Province, Priority Academic Program Development of

Jiangsu Higher Education Institutions (PAPD) and Collaborative

Innovation Center of Radiological Medicine of Jiangsu Higher Education

Institutions and Nantong science foundation of health bureau for youth

(WQ2014045).

CR Artero-Castro A, 2015, AUTOPHAGY, V11, P1499, DOI 10.1080/15548627.2015.1063764

Bensaad K, 2006, CELL, V126, P107, DOI 10.1016/j.cell.2006.05.036

Bensaad K, 2009, EMBO J, V28, P3015, DOI 10.1038/emboj.2009.242

Kimura T, 2013, CANCER RES, V73, P3, DOI 10.1158/0008-5472.CAN-12-2464

Komatsu M, 2007, CELL, V131, P1149, DOI 10.1016/j.cell.2007.10.035

Kuoa SY, 2015, P NATL ACAD SCI USA, V112, pE4281, DOI 10.1073/pnas.1512289112

Li H, 2009, J BIOL CHEM, V284, P1748, DOI 10.1074/jbc.M807821200

Lui VWY, 2011, ONCOGENE, V30, P1127, DOI 10.1038/onc.2010.490

Ohtsuka T, 2004, ONCOGENE, V23, P5405, DOI 10.1038/sj.onc.1207693

Palumbo S, 2013, J CELL PHYSIOL, V228, P1, DOI 10.1002/jcp.24118

Pattingre S, 2008, BIOCHIMIE, V90, P313, DOI 10.1016/j.biochi.2007.08.014

Peña-Rico MA, 2011, RADIOTHER ONCOL, V101, P132, DOI 10.1016/j.radonc.2011.07.002

Perera R, 2015, NATURE, V524, P361, DOI 10.1038/nature14587

Tanida I, 2004, INT J BIOCHEM CELL B, V36, P2503, DOI 10.1016/j.biocel.2004.05.009

Thomas AA, 2014, JAMA NEUROL, V71, P1437, DOI 10.1001/jamaneurol.2014.1701

Tibbetts RS, 1999, GENE DEV, V13, P152, DOI 10.1101/gad.13.2.152

Van Meir EG, 2010, CA-CANCER J CLIN, V60, P166, DOI 10.3322/caac.20069

Zhang HW, 2014, FREE RADICAL BIO MED, V69, P239, DOI 10.1016/j.freeradbiomed.2014.01.034

NR 18

TC 0

Z9 0

U1 0

U2 6

PU E-CENTURY PUBLISHING CORP

PI MADISON

PA 40 WHITE OAKS LN, MADISON, WI 53711 USA

SN 1936-2625

J9 INT J CLIN EXP PATHO

JI Int. J. Clin. Exp. Pathol.

PY 2016

VL 9

IS 12

BP 12535

EP 12542

PG 8

WC Oncology; Pathology

WE Science Citation Index Expanded (SCI-EXPANDED)

SC Oncology; Pathology

GA EF5YP

UT WOS:000390406200038

DA 2025-04-09

ER

PT J

AU Polewski, MD

Reveron-Thornton, RF

Cherryholmes, GA

Marinov, GK

Cassady, K

Aboody, KS

AF Polewski, Monika D.

Reveron-Thornton, Rosyli F.

Cherryholmes, Gregory A.

Marinov, Georgi K.

Cassady, Kaniel

Aboody, Karen S.

TI Increased Expression of System xc<SUP>-</SUP> in Glioblastoma

Confers an Altered Metabolic State and Temozolomide Resistance

SO MOLECULAR CANCER RESEARCH

LA English

DT Article

ID CYSTINE/GLUTAMATE ANTIPORTER; IN-VITRO; CANCER; XCT; GLIOMAS; GROWTH;

CELLS; SULFASALAZINE; GLUTATHIONE; PROGRESSION

AB Glioblastoma multiforme is the most aggressive malignant primary brain tumor in adults. Several studies have shown that glioma cells upregulate the expression of xCT ( SLC7A11), the catalytic subunit of system x(c)(-), a transporter involved in cystine import, that modulates glutathione production and glioma growth. However, the role of system x(c)(-) in regulating the sensitivity of glioma cells to chemotherapy is currently debated. Inhibiting system x(c)(-) with sulfasalazine decreased glioma growth and survival via redox modulation, and use of the chemotherapeutic agent temozolomide together with sulfasalazine had a synergistic effect on cell killing. To better understand the functional consequences of system x(c)(-) in glioma, stable SLC7A11-knockdown and -overexpressing U251 glioma cells were generated. Modulation of SLC7A11 did not alter cellar proliferation but overexpression did increase anchorage-independent cell growth. Knockdown of SLC7A11 increased basal reactive oxygen species (ROS) and decreased glutathione generation resulting in increased cell death under oxidative and genotoxic stress. Overexpression of SLC7A11 resulted in increased resistance to oxidative stress and decreased chemosensitivity to temozolomide. In addition, SLC7A11 overexpression was associated with altered cellular metabolism including increased mitochondrial biogenesis, oxidative phosphorylation, and ATP generation. These results suggest that expression of SLC7A11 in the context of glioma contributes to tumorigenesis, tumor progression, and resistance to standard chemotherapy. (C) 2016 AACR.

C1 [Polewski, Monika D.; Reveron-Thornton, Rosyli F.; Aboody, Karen S.] City Hope Natl Med Ctr, Dept Neurosci, 1500 East Duarte Rd, Duarte, CA 91010 USA.

[Polewski, Monika D.; Reveron-Thornton, Rosyli F.; Cherryholmes, Gregory A.; Cassady, Kaniel; Aboody, Karen S.] Beckman Res Inst, 1500 East Duarte Rd, Duarte, CA 91010 USA.

[Polewski, Monika D.; Cherryholmes, Gregory A.; Cassady, Kaniel] City Hope Natl Med Ctr, Irell & Manella Grad Sch Biol Sci, 1500 East Duarte Rd, Duarte, CA 91010 USA.

[Reveron-Thornton, Rosyli F.] Calif State Univ San Bernardino, Dept Biol Sci, San Bernardino, CA 92407 USA.

[Cherryholmes, Gregory A.] City Hope Natl Med Ctr, Dept Canc Immunotherapeut & Tumor Immunol, 1500 E Duarte Rd, Duarte, CA 91010 USA.

[Marinov, Georgi K.] CALTECH, Div Biol, Pasadena, CA 91125 USA.

[Cassady, Kaniel] City Hope Natl Med Ctr, Dept Diabet Res, 1500 E Duarte Rd, Duarte, CA 91010 USA.

[Cassady, Kaniel] City Hope Natl Med Ctr, Dept Hematol Hematopoiet Cell Transplantat, 1500 E Duarte Rd, Duarte, CA 91010 USA.

[Aboody, Karen S.] City Hope Natl Med Ctr, Div Neurosurg, 1500 East Duarte Rd, Duarte, CA 91010 USA.

C3 City of Hope; City of Hope; Beckman Research Institute of City of Hope;

City of Hope; California State University System; California State

University San Bernardino; City of Hope; California Institute of

Technology; City of Hope; City of Hope; City of Hope

RP Polewski, MD; Aboody, KS (corresponding author), City Hope Natl Med Ctr, Dept Neurosci, 1500 East Duarte Rd, Duarte, CA 91010 USA.; Polewski, MD; Aboody, KS (corresponding author), Beckman Res Inst, 1500 East Duarte Rd, Duarte, CA 91010 USA.; Polewski, MD (corresponding author), City Hope Natl Med Ctr, Irell & Manella Grad Sch Biol Sci, 1500 East Duarte Rd, Duarte, CA 91010 USA.; Aboody, KS (corresponding author), City Hope Natl Med Ctr, Div Neurosurg, 1500 East Duarte Rd, Duarte, CA 91010 USA.

EM monika.polewski@gmail.com; kaboody@coh.org

RI Cassady, Kaniel/R-2575-2019

OI Marinov, Georgi/0000-0003-1822-7273; Cassady, Kaniel/0000-0001-8949-3507

FU California Institute of Regenerative Medicine [TG2-01150]; Rosalinde and

Arthur Gilbert Foundation; STOP Cancer; Cancer Center Support Grant

[P30CA033572]

FX This work was supported by funding from the California Institute of

Regenerative Medicine (TG2-01150), the Rosalinde and Arthur Gilbert

Foundation, STOP Cancer, and the Cancer Center Support Grant

(P30CA033572).

CR Anders S, 2010, GENOME BIOL, V11, DOI 10.1186/gb-2010-11-10-r106

Banjac A, 2008, ONCOGENE, V27, P1618, DOI 10.1038/sj.onc.1210796

Berriz GF, 2009, BIOINFORMATICS, V25, P3043, DOI 10.1093/bioinformatics/btp498

Brand MD, 2011, BIOCHEM J, V435, P297, DOI 10.1042/BJ20110162

Brandes AA, 2008, NEURO-ONCOLOGY, V10, P361, DOI 10.1215/15228517-2008-008

Brown CE, 2009, CANCER RES, V69, P8886, DOI 10.1158/0008-5472.CAN-09-2687

Chen LY, 2015, ONCOL REP, V33, P1465, DOI 10.3892/or.2015.3712

Chen RS, 2009, ONCOGENE, V28, P599, DOI 10.1038/onc.2008.414

Chou TC, 2006, PHARMACOL REV, V58, P621, DOI 10.1124/pr.58.3.10

Chung WJ, 2005, J NEUROSCI, V25, P7101, DOI 10.1523/JNEUROSCI.5258-04.2005

COLVIN OM, 1993, ADV ENZYME REGUL, V33, P19

Conrad M, 2012, AMINO ACIDS, V42, P231, DOI 10.1007/s00726-011-0867-5

Gupta SC, 2012, ANTIOXID REDOX SIGN, V16, P1295, DOI 10.1089/ars.2011.4414

Huang Y, 2005, CANCER RES, V65, P7446, DOI 10.1158/0008-5472.CAN-04-4267

Langmead B, 2009, GENOME BIOL, V10, DOI 10.1186/gb-2009-10-3-r25

Lee HC, 2004, INT J MOL MED, V13, P883

Lin CJ, 2012, FREE RADICAL BIO MED, V52, P377, DOI 10.1016/j.freeradbiomed.2011.10.487

Liou GY, 2010, FREE RADICAL RES, V44, P479, DOI 10.3109/10715761003667554

Lo M, 2008, BRIT J CANCER, V99, P464, DOI 10.1038/sj.bjc.6604485

Lo M, 2008, J CELL PHYSIOL, V215, P593, DOI 10.1002/jcp.21366

Ogunrinu TA, 2010, J BIOL CHEM, V285, P37716, DOI 10.1074/jbc.M110.161190

Okuno S, 2003, BRIT J CANCER, V88, P951, DOI 10.1038/sj.bjc.6600786

Oliva CR, 2011, PLOS ONE, V6, DOI 10.1371/journal.pone.0024665

Pham AN, 2010, J PHARMACOL EXP THER, V332, P949, DOI 10.1124/jpet.109.162248

Robe PA, 2004, CLIN CANCER RES, V10, P5595, DOI 10.1158/1078-0432.CCR-03-0392

Robe PA, 2009, BMC CANCER, V9, DOI 10.1186/1471-2407-9-372

Robert SM, 2015, SCI TRANSL MED, V7, DOI 10.1126/scitranslmed.aaa8103

Roberts A, 2013, NAT METHODS, V10, P71, DOI [10.1038/NMETH.2251, 10.1038/nmeth.2251]

Savaskan NE, 2008, NAT MED, V14, P629, DOI 10.1038/nm1772

Singer E, 2015, CELL DEATH DIS, V6, DOI 10.1038/cddis.2014.566

Stupp R, 2005, NEW ENGL J MED, V352, P987, DOI 10.1056/NEJMoa043330

Takeuchi S, 2013, NEUROSURGERY, V72, P33, DOI 10.1227/NEU.0b013e318276b2de

Trachootham D, 2009, NAT REV DRUG DISCOV, V8, P579, DOI 10.1038/nrd2803

Traverso N, 2013, OXID MED CELL LONGEV, V2013, DOI 10.1155/2013/972913

Villeneuve LM, 2008, P NATL ACAD SCI USA, V105, P9047, DOI 10.1073/pnas.0803623105

Wallace DC, 2012, NAT REV CANCER, V12, P685, DOI 10.1038/nrc3365

Watkins S, 2012, TRENDS NEUROSCI, V35, P546, DOI 10.1016/j.tins.2012.05.001

NR 37

TC 95

Z9 110

U1 1

U2 15

PU AMER ASSOC CANCER RESEARCH

PI PHILADELPHIA

PA 615 CHESTNUT ST, 17TH FLOOR, PHILADELPHIA, PA 19106-4404 USA

SN 1541-7786

EI 1557-3125

J9 MOL CANCER RES

JI Mol. Cancer Res.

PD DEC

PY 2016

VL 14

IS 12

BP 1229

EP 1242

DI 10.1158/1541-7786.MCR-16-0028

PG 14

WC Oncology; Cell Biology

WE Science Citation Index Expanded (SCI-EXPANDED)

SC Oncology; Cell Biology

GA EE5GE

UT WOS:000389632700007

PM 27658422

OA Bronze, Green Accepted, Green Submitted

DA 2025-04-09

ER

PT J

AU König, S

Strassheimer, F

Brandner, NI

Schröder, JH

Urban, H

Harwart, LF

Hehlgans, S

Steinbach, JP

Ronellenfitsch, MW

Luger, AL

AF Koenig, Sven

Strassheimer, Florian

Brandner, Nadja I.

Schroeder, Jan-Hendrik

Urban, Hans

Harwart, Leander F.

Hehlgans, Stephanie

Steinbach, Joachim P.

Ronellenfitsch, Michael W.

Luger, Anna-Luisa

TI Superoxide dismutase 1 mediates adaptation to the tumor microenvironment

of glioma cells via mammalian target of rapamycin complex 1

SO CELL DEATH DISCOVERY

LA English

DT Article

ID CANCER STEM-CELLS; PHASE-II; PROTECTS GLIOMA; HYPOXIA; TEMOZOLOMIDE;

GROWTH; SOD1; TETRATHIOMOLYBDATE; GLIOBLASTOMA; INHIBITION

AB In glioblastoma (GB) cells oxidative stress is induced by both, conditions of the tumor microenvironment as well as by therapeutic interventions. Upregulation of superoxide dismutase 1 (SOD1), a key enzyme for oxidative defense and downstream target of mammalian target of rapamycin complex 1 (mTORC1) is a candidate mechanism to sustain survival and proliferation of tumor cells. SOD1 was inhibited by shRNA mediated gene suppression, CRISPR/Cas9 knockout and pharmacological inhibition in human (primary) GB cells. SOD1 activity was determined by SOD1/2 activity assay. ROS levels, cell death and the NADPH/NADP-ratio were measured under normal and starvation conditions. To study the mTORC1-SOD1 axis, mTORC1 activated TSC2 knockdown cells (TSC2sh) were analyzed. Genetic and pharmacological inhibition of SOD1 correlated with decreased SOD1 activity, increased ROS and enhanced the sensitivity of glioma cells towards starvation- and hypoxia-induced cell death. This was accompanied by a decreased NADPH/NADP-ratio. Furthermore, combination therapy of SOD1 and mTORC1 inhibition partially rescued the protective effect of mTORC1 inhibitor monotherapy. SOD1 mediates adaptation of GB cells to stress conditions in the tumor microenvironment in a mTORC1-dependent manner. Moreover, SOD1 activation contributes to the cell death resistance conferred by mTORC1 inhibitors under hypoxic conditions.

C1 [Koenig, Sven; Strassheimer, Florian; Brandner, Nadja I.; Schroeder, Jan-Hendrik; Urban, Hans; Harwart, Leander F.; Steinbach, Joachim P.; Ronellenfitsch, Michael W.; Luger, Anna-Luisa] Goethe Univ Frankfurt, Univ Hosp, Dr Senckenberg Inst Neurooncol, Frankfurt, Germany.

[Koenig, Sven; Strassheimer, Florian; Brandner, Nadja I.; Schroeder, Jan-Hendrik; Urban, Hans; Harwart, Leander F.; Steinbach, Joachim P.; Ronellenfitsch, Michael W.; Luger, Anna-Luisa] German Canc Consortium DKTK, Partner Site Frankfurt Mainz, Frankfurt, Germany.

[Koenig, Sven; Strassheimer, Florian; Brandner, Nadja I.; Schroeder, Jan-Hendrik; Urban, Hans; Harwart, Leander F.; Steinbach, Joachim P.; Ronellenfitsch, Michael W.; Luger, Anna-Luisa] Goethe Univ Frankfurt, Frankfurt Canc Inst FCI, Frankfurt, Germany.

[Koenig, Sven; Strassheimer, Florian; Brandner, Nadja I.; Schroeder, Jan-Hendrik; Urban, Hans; Harwart, Leander F.; Steinbach, Joachim P.; Ronellenfitsch, Michael W.; Luger, Anna-Luisa] Goethe Univ Frankfurt, Univ Hosp, Univ Canc Ctr UCT, Frankfurt, Germany.

[Hehlgans, Stephanie] Goethe Univ Frankfurt, Univ Hosp, Dept Radiotherapy & Oncol, Frankfurt, Germany.

C3 Goethe University Frankfurt; Goethe University Frankfurt Hospital;

Helmholtz Association; German Cancer Research Center (DKFZ); Goethe

University Frankfurt; Goethe University Frankfurt; Goethe University

Frankfurt Hospital; Goethe University Frankfurt; Goethe University

Frankfurt Hospital

RP Luger, AL (corresponding author), Goethe Univ Frankfurt, Univ Hosp, Dr Senckenberg Inst Neurooncol, Frankfurt, Germany.; Luger, AL (corresponding author), German Canc Consortium DKTK, Partner Site Frankfurt Mainz, Frankfurt, Germany.; Luger, AL (corresponding author), Goethe Univ Frankfurt, Frankfurt Canc Inst FCI, Frankfurt, Germany.; Luger, AL (corresponding author), Goethe Univ Frankfurt, Univ Hosp, Univ Canc Ctr UCT, Frankfurt, Germany.

EM a.luger@med.uni-frankfurt.de

RI Thiepold, Anna-Luisa/AAW-8310-2021; Ronellenfitsch, Michael

W./AAW-8201-2020

OI Ronellenfitsch, Michael W./0000-0002-1402-6290; Konig,

Sven/0000-0002-7904-1483

FU Mildred Scheel Career Center Frankfurt (Deutsche Krebshilfe)

FX The results shown in Fig. 1A, B are based upon data generated by the

TCGA Research Network: https://www.cancer.gov/tcga.

CR Agrawal K, 2023, CANCERS, V15, DOI 10.3390/cancers15204920

Bowman RL, 2017, NEURO-ONCOLOGY, V19, P139, DOI 10.1093/neuonc/now247

Chan N, 2017, CLIN CANCER RES, V23, P666, DOI 10.1158/1078-0432.CCR-16-1326

Che MX, 2016, DRUG DISCOV TODAY, V21, P143, DOI 10.1016/j.drudis.2015.10.001

Cheung EC, 2022, NAT REV CANCER, V22, P280, DOI 10.1038/s41568-021-00435-0

Doñate F, 2008, BRIT J CANCER, V98, P776, DOI 10.1038/sj.bjc.6604226

Eckerich C, 2009, J NEUROCHEM, V109, P969, DOI 10.1111/j.1471-4159.2009.06027.x

Elchuri S, 2005, ONCOGENE, V24, P367, DOI 10.1038/sj.onc.1208207

Engel AL, 2020, BRIT J CANCER, V122, P1391, DOI 10.1038/s41416-020-0794-x

Epperly MW, 2003, RADIAT RES, V160, P568, DOI 10.1667/RR3081

Foltyn M, 2019, BRIT J CANCER, V120, P481, DOI 10.1038/s41416-018-0368-3

Gao Z, 2008, FREE RADICAL BIO MED, V45, P1501, DOI 10.1016/j.freeradbiomed.2008.08.009

Glasauer A, 2014, J CLIN INVEST, V124, P117, DOI 10.1172/JCI71714

GRADY JE, 1960, CANCER RES, V20, P1114

Harris AL, 2002, NAT REV CANCER, V2, P38, DOI 10.1038/nrc704

Harter PN, 2015, PLOS ONE, V10, DOI 10.1371/journal.pone.0127123

Hayes JD, 2020, CANCER CELL, V38, P167, DOI 10.1016/j.ccell.2020.06.001

Heinzen D, 2019, INT J MOL SCI, V20, DOI 10.3390/ijms20184474

Iqbal MJ, 2024, CELL COMMUN SIGNAL, V22, DOI 10.1186/s12964-023-01398-5

Kim D, 2015, NATURE, V520, P363, DOI 10.1038/nature14363

Kraboth Z, 2020, PATHOL ONCOL RES, V26, P2035, DOI 10.1007/s12253-019-00705-1

Krawczynski K, 2020, ANTIOXIDANTS-BASEL, V9, DOI 10.3390/antiox9080747

Laplante M, 2012, CELL, V149, P274, DOI 10.1016/j.cell.2012.03.017

Lathia JD, 2011, CELL STEM CELL, V8, P482, DOI 10.1016/j.stem.2011.04.013

Lin JQ, 2013, UROL ONCOL-SEMIN ORI, V31, P581, DOI 10.1016/j.urolonc.2011.04.009

Ling M, 2022, FRONT ONCOL, V12, DOI 10.3389/fonc.2022.937444

Lorenz NI, 2021, SCI REP-UK, V11, DOI 10.1038/s41598-021-93663-1

Lowndes SA, 2008, CLIN CANCER RES, V14, P7526, DOI 10.1158/1078-0432.CCR-08-0315

Luger AL, 2020, CANCERS, V12, DOI 10.3390/cancers12082144

Miao L, 2009, FREE RADICAL BIO MED, V47, P344, DOI 10.1016/j.freeradbiomed.2009.05.018

Nakamura H, 2021, CANCER SCI, V112, P3945, DOI 10.1111/cas.15068

Norambuena A, 2022, NEUROBIOL DIS, V169, DOI 10.1016/j.nbd.2022.105737

Omuro A, 2013, JAMA-J AM MED ASSOC, V310, P1842, DOI 10.1001/jama.2013.280319

Papa L., 2014, GENES CANC, V5, P15, DOI DOI 10.18632/GENESANDCANCER.4

Ronellenfitsch MW, 2018, ACTA NEUROPATHOL COM, V6, DOI 10.1186/s40478-018-0583-4

Ronellenfitsch MW, 2009, BRAIN, V132, P1509, DOI 10.1093/brain/awp093

Sahoo BM, 2022, ANTI-CANCER AGENT ME, V22, P215, DOI 10.2174/1871520621666210608095512

Sauer B, 2024, CELL DEATH DISCOV, V10, DOI 10.1038/s41420-023-01779-2

Schneider BJ, 2013, INVEST NEW DRUG, V31, P435, DOI 10.1007/s10637-012-9864-0

Somwar R, 2011, P NATL ACAD SCI USA, V108, P16375, DOI 10.1073/pnas.1113554108

Steinbach JP, 2004, CANCER RES, V64, P1575, DOI 10.1158/0008-5472.CAN-03-3775

Steinbach JP, 2003, CELL DEATH DIFFER, V10, P823, DOI 10.1038/sj.cdd.4401252

STUDER A, 1985, ACTA NEUROPATHOL, V66, P208, DOI 10.1007/BF00688585

Stupp R, 2005, NEW ENGL J MED, V352, P987, DOI 10.1056/NEJMoa043330

Stupp R, 2017, JAMA-J AM MED ASSOC, V318, P2306, DOI 10.1001/jama.2017.18718

Thiepold AL, 2017, BRAIN, V140, P2623, DOI 10.1093/brain/awx196

Tsang CK, 2018, MOL CELL, V70, P502, DOI 10.1016/j.molcel.2018.03.029

Tsang CK, 2014, NAT COMMUN, V5, DOI 10.1038/ncomms4446

Urban H, 2020, CANCERS, V12, DOI 10.3390/cancers12103050

Vandesompele J, 2002, GENOME BIOL, V3, DOI 10.1186/gb-2002-3-7-research0034

Venere M, 2011, GLIA, V59, P1148, DOI 10.1002/glia.21185

Wang YW, 2021, THERANOSTICS, V11, P4839, DOI 10.7150/thno.56747

Wanka C, 2012, ONCOGENE, V31, P3764, DOI 10.1038/onc.2011.530

Weydert CJ, 2010, NAT PROTOC, V5, P51, DOI 10.1038/nprot.2009.197

Wick W, 2016, CLIN CANCER RES, V22, P4797, DOI 10.1158/1078-0432.CCR-15-3153

Wischhusen J, 2003, ONCOGENE, V22, P8233, DOI 10.1038/sj.onc.1207198

Xu J, 2022, ANTIOXIDANTS-BASEL, V11, DOI 10.3390/antiox11020427

Yang YC, 2023, FRONT IMMUNOL, V14, DOI 10.3389/fimmu.2023.1259797

NR 58

TC 1

Z9 1

U1 2

U2 2

PU SPRINGERNATURE

PI LONDON

PA CAMPUS, 4 CRINAN ST, LONDON, N1 9XW, ENGLAND

EI 2058-7716

J9 CELL DEATH DISCOV

JI Cell Death Discov.

PD AUG 26

PY 2024

VL 10

IS 1

AR 379

DI 10.1038/s41420-024-02145-6

PG 12

WC Cell Biology

WE Science Citation Index Expanded (SCI-EXPANDED)

SC Cell Biology

GA D7G9F

UT WOS:001297840800002

PM 39187509

OA gold

DA 2025-04-09

ER

PT J

AU Sharanek, A

Burban, A

Laaper, M

Heckel, E

Joyal, JS

Soleimani, VD

Jahani-Asl, A

AF Sharanek, Ahmad

Burban, Audrey

Laaper, Matthew

Heckel, Emilie

Joyal, Jean-Sebastien

Soleimani, Vahab D.

Jahani-Asl, Arezu

TI OSMR controls glioma stem cell respiration and confers resistance of

glioblastoma to ionizing radiation

SO NATURE COMMUNICATIONS

LA English

DT Article

ID ONCOSTATIN-M RECEPTOR; COMPLEX-I; OXIDATIVE STRESS; MITOCHONDRIAL;

METABOLISM; MUTATIONS; TEMOZOLOMIDE; ASTROCYTES; PATHWAYS; DYNAMICS

AB Glioblastoma contains a rare population of self-renewing brain tumor stem cells (BTSCs) which are endowed with properties to proliferate, spur the growth of new tumors, and at the same time, evade ionizing radiation (IR) and chemotherapy. However, the drivers of BTSC resistance to therapy remain unknown. The cytokine receptor for oncostatin M (OSMR) regulates BTSC proliferation and glioblastoma tumorigenesis. Here, we report our discovery of a mitochondrial OSMR that confers resistance to IR via regulation of oxidative phosphorylation, independent of its role in cell proliferation. Mechanistically, OSMR is targeted to the mitochondrial matrix via the presequence translocase-associated motor complex components, mtHSP70 and TIM44. OSMR interacts with NADH ubiquinone oxidoreductase 1/2 (NDUFS1/2) of complex I and promotes mitochondrial respiration. Deletion of OSMR impairs spare respiratory capacity, increases reactive oxygen species, and sensitizes BTSCs to IR-induced cell death. Importantly, suppression of OSMR improves glioblastoma response to IR and prolongs lifespan. The suppression of the receptor for oncostatin M (OSMR) can prevent glioblastoma cell growth. Here, the authors demonstrate a role for OSMR in modulating glioma stem cell respiration and its impact on resistance to ionizing radiation.

C1 [Sharanek, Ahmad; Burban, Audrey; Laaper, Matthew; Soleimani, Vahab D.; Jahani-Asl, Arezu] Jewish Gen Hosp, Lady Davis Inst Med Res, 3755 Chemin Cote St Catherine, Montreal, PQ H3T 1E2, Canada.

[Jahani-Asl, Arezu] Montreal Neurol Inst, Integrated Program Neurosci, 3801 Univ St, Montreal, PQ H3A 2B4, Canada.

[Heckel, Emilie; Joyal, Jean-Sebastien] Univ Montreal, Dept Pediat, CHU St Justine, Montreal, PQ H3T 1C5, Canada.

[Heckel, Emilie; Joyal, Jean-Sebastien] Univ Montreal, Dept Pharmacol, CHU St Justine, Montreal, PQ H3T 1C5, Canada.

[Heckel, Emilie; Joyal, Jean-Sebastien] Univ Montreal, Dept Ophthalmol, CHU St Justine, Montreal, PQ H3T 1C5, Canada.

[Heckel, Emilie; Joyal, Jean-Sebastien] McGill Univ, Dept Pharmacol & Therapeut, Montreal, PQ H3G 1Y6, Canada.

[Soleimani, Vahab D.] McGill Univ, Dept Human Genet, 3640 Rue Univ, Montreal, PQ H3A 0C7, Canada.

[Jahani-Asl, Arezu] McGill Univ, Gerald Bronfman Dept Oncol, 5100 Maisonneuve Blvd West,Suite 720, Montreal, PQ H4A 3T2, Canada.

[Jahani-Asl, Arezu] McGill Univ, Div Expt Med, 5100 Maisonneuve Blvd West,Suite 720, Montreal, PQ H4A 3T2, Canada.

C3 Lady Davis Institute; Universite de Montreal; Centre Hospitalier

Universitaire Sainte-Justine; Universite de Montreal; Centre Hospitalier

Universitaire Sainte-Justine; Universite de Montreal; Centre Hospitalier

Universitaire Sainte-Justine; McGill University; McGill University;

McGill University; McGill University

RP Jahani-Asl, A (corresponding author), Jewish Gen Hosp, Lady Davis Inst Med Res, 3755 Chemin Cote St Catherine, Montreal, PQ H3T 1E2, Canada.; Jahani-Asl, A (corresponding author), Montreal Neurol Inst, Integrated Program Neurosci, 3801 Univ St, Montreal, PQ H3A 2B4, Canada.; Jahani-Asl, A (corresponding author), McGill Univ, Gerald Bronfman Dept Oncol, 5100 Maisonneuve Blvd West,Suite 720, Montreal, PQ H4A 3T2, Canada.; Jahani-Asl, A (corresponding author), McGill Univ, Div Expt Med, 5100 Maisonneuve Blvd West,Suite 720, Montreal, PQ H4A 3T2, Canada.

EM arezu.jahani@mcgill.ca

RI Sharanek, Ahmad/HJY-6216-2023

OI Sharanek, Ahmad/0000-0002-0558-3626; Burban, Audrey/0009-0000-4114-0403;

Jahani-Asl, Arezu/0000-0003-4002-3381

FU Canadian Institute of Health Research [PJT 148986, PJT 145449, PJT

162198]; Brain Tumor Charity [497225]; FRQS postdoctoral fellowship

FX This work was supported by grants from the Canadian Institute of Health

Research # PJT 148986, PJT 145449, PJT 162198, and The Brain Tumor

Charity, #497225 to AJA. AJA is an Fonds de la recherche en sante du

Quebec (FRQS) scholar in Glioblastoma Biology. AB is supported by an

FRQS postdoctoral fellowship. We thank Dr. Samual Weiss at the

University of Calgary for sharing BTSC73, 147, 12, and Dr. Keith Ligon

at Harvard Medical School for the generation of BTSC112, 145, and 172.

We thank Christian Young at the Lady Davis Institute for Medical

Research - Jewish General Hospital - core facility for help with the

Fluorescence-Activated Cell Sorting (FACS). We thank staff at the Lady

Davis Institute Animal Core Facility for assistance with studies

involving mice. We thank Perrine Gaub for technical assistance with the

Seahorse bioenergetic analysis, and Felicia Lazure for technical help in

IR experiments.

CR Acín-Pérez R, 2004, MOL CELL, V13, P805, DOI 10.1016/S1097-2765(04)00124-8

Acin-Pérez R, 2008, MOL CELL, V32, P529, DOI 10.1016/j.molcel.2008.10.021

Agnihotri S, 2016, NEURO-ONCOLOGY, V18, P160, DOI 10.1093/neuonc/nov125

Arita K, 2008, AM J HUM GENET, V82, P73, DOI 10.1016/j.ajhg.2007.09.002

Arnold CR, 2020, FRONT ONCOL, V10, DOI 10.3389/fonc.2020.00164

Azzam EI, 2012, CANCER LETT, V327, P48, DOI 10.1016/j.canlet.2011.12.012

Bao SD, 2006, NATURE, V444, P756, DOI 10.1038/nature05236

Bauer DE, 2015, JOVE-J VIS EXP, DOI 10.3791/52118

Burban A, 2018, FREE RADICAL BIO MED, V115, P166, DOI 10.1016/j.freeradbiomed.2017.11.017

Caffarel MM, 2014, J PATHOL, V232, P386, DOI 10.1002/path.4305

Cao XY, 2011, MOL CANCER, V10, DOI 10.1186/1476-4598-10-26

Case AJ, 2017, REDOX BIOL, V11, P82, DOI 10.1016/j.redox.2016.11.011

Chacinska A, 2009, CELL, V138, P628, DOI 10.1016/j.cell.2009.08.005

Che TF, 2015, ONCOTARGET, V6, P37349, DOI 10.18632/oncotarget.5736

Chen J, 2012, NATURE, V488, P522, DOI 10.1038/nature11287

Chen J, 2012, CELL, V149, P36, DOI 10.1016/j.cell.2012.03.009

Demory ML, 2009, J BIOL CHEM, V284, P36592, DOI 10.1074/jbc.M109.000760

Diehn M, 2009, NATURE, V458, P780, DOI 10.1038/nature07733

Ding Y, 2012, NAT COMMUN, V3, DOI 10.1038/ncomms2236

Elkholi R, 2019, MOL CELL, V74, P452, DOI 10.1016/j.molcel.2019.02.012

Esparza-Molto PB, 2020, ANTIOXID REDOX SIGN, V33, P927, DOI 10.1089/ars.2019.7988

Feichtinger RG, 2017, OXID MED CELL LONGEV, V2017, DOI 10.1155/2017/7202589

Galli R, 2004, CANCER RES, V64, P7011, DOI 10.1158/0008-5472.CAN-04-1364

Guo L, 2013, ONCOGENE, V32, P5272, DOI 10.1038/onc.2012.573

Heinrich PC, 1998, BIOCHEM J, V334, P297, DOI 10.1042/bj3340297

Hermanns HM, 2015, CYTOKINE GROWTH F R, V26, P545, DOI 10.1016/j.cytogfr.2015.07.006

Hsu PP, 2008, CELL, V134, P703, DOI 10.1016/j.cell.2008.08.021

Hu YF, 2009, J IMMUNOL METHODS, V347, P70, DOI 10.1016/j.jim.2009.06.008

Iuso A, 2006, J BIOL CHEM, V281, P10374, DOI 10.1074/jbc.M513387200

Jahani-Asl A, 2016, NAT NEUROSCI, V19, P798, DOI 10.1038/nn.4295

Kan CE, 2011, CANCER RES, V71, P6930, DOI 10.1158/0008-5472.CAN-10-3860

Khacho M, 2016, CELL STEM CELL, V19, P232, DOI 10.1016/j.stem.2016.04.015

Kirby DM, 2004, J CLIN INVEST, V114, P837, DOI 10.1172/JCI200420683

Laaper M, 2017, JOVE-J VIS EXP, DOI 10.3791/55871

Lapuente-Brun E, 2013, SCIENCE, V340, P1567, DOI 10.1126/science.1230381

Lee MJ, 2013, INT J BIOCHEM CELL B, V45, P1869, DOI 10.1016/j.biocel.2013.05.027

Lopez-Fabuel I, 2016, P NATL ACAD SCI USA, V113, P13063, DOI 10.1073/pnas.1613701113

Louis DN, 2016, ACTA NEUROPATHOL, V131, P803, DOI 10.1007/s00401-016-1545-1

Mandegar MA, 2016, CELL STEM CELL, V18, P541, DOI 10.1016/j.stem.2016.01.022

Mazurek M, 2020, CANCERS, V12, DOI 10.3390/cancers12010210

Meier JA, 2014, SEMIN IMMUNOL, V26, P20, DOI 10.1016/j.smim.2013.12.005

Miller TW, 2015, J BIOL CHEM, V290, P24858, DOI 10.1074/jbc.M115.665752

Molina JR, 2018, NAT MED, V24, P1036, DOI 10.1038/s41591-018-0052-4

Moreno-Lastres D, 2012, CELL METAB, V15, P324, DOI 10.1016/j.cmet.2012.01.015

Naguib A, 2018, CELL REP, V23, P58, DOI 10.1016/j.celrep.2018.03.032

Natesh K, 2015, NEOPLASIA, V17, P225, DOI 10.1016/j.neo.2015.01.001

Ni Y, 2019, CELLS-BASEL, V8, DOI 10.3390/cells8101149

Poteet E, 2013, J BIOL CHEM, V288, P9153, DOI 10.1074/jbc.M112.440354

Qazi MA, 2017, ANN ONCOL, V28, P1448, DOI 10.1093/annonc/mdx169

Schaefer LK, 2000, CYTOKINE, V12, P1647, DOI 10.1006/cyto.2000.0774

Shen H, 2015, MOL CANCER THER, V14, P1794, DOI 10.1158/1535-7163.MCT-15-0247

Shi YF, 2019, NATURE, V567, P341, DOI 10.1038/s41586-019-0993-x

Sica V, 2020, INT J CANCER, V146, P10, DOI 10.1002/ijc.32616

Singh SK, 2004, NATURE, V432, P396, DOI 10.1038/nature03128

Soubannier V, 2009, BBA-MOL CELL RES, V1793, P154, DOI 10.1016/j.bbamcr.2008.07.008

Spinazzi M, 2012, NAT PROTOC, V7, P1235, DOI 10.1038/nprot.2012.058

Strickland M, 2017, FRONT CELL DEV BIOL, V5, DOI 10.3389/fcell.2017.00043

Stupp R, 2005, NEW ENGL J MED, V352, P987, DOI 10.1056/NEJMoa043330

Tanaka M, 2003, BLOOD, V102, P3154, DOI 10.1182/blood-2003-02-0367

Tang L, 2018, J EXP CLIN CANC RES, V37, DOI 10.1186/s13046-018-0758-7

Tucker EJ, 2013, PLOS GENET, V9, DOI 10.1371/journal.pgen.1004034

Urra FA, 2017, FRONT ONCOL, V7, DOI 10.3389/fonc.2017.00118

Van Wagoner NJ, 2000, J NEUROCHEM, V75, P563, DOI 10.1046/j.1471-4159.2000.0750563.x

Velpula KK, 2017, ONCOTARGET, V8, P35639, DOI 10.18632/oncotarget.16767

Vlashi E, 2011, P NATL ACAD SCI USA, V108, P16062, DOI 10.1073/pnas.1106704108

Wegrzyn J, 2009, SCIENCE, V323, P793, DOI 10.1126/science.1164551

Westermann B, 2010, NAT REV MOL CELL BIO, V11, P872, DOI 10.1038/nrm3013

Wheaton WW, 2014, ELIFE, V3, DOI 10.7554/eLife.02242

Zhou DH, 2014, ADV CANCER RES, V122, P1, DOI 10.1016/B978-0-12-420117-0.00001-3

Zickermann V, 2015, SCIENCE, V347, P44, DOI 10.1126/science.1259859

NR 70

TC 53

Z9 59

U1 1

U2 17

PU NATURE PORTFOLIO

PI BERLIN

PA HEIDELBERGER PLATZ 3, BERLIN, 14197, GERMANY

EI 2041-1723

J9 NAT COMMUN

JI Nat. Commun.

PD AUG 17

PY 2020

VL 11

IS 1

AR 4116

DI 10.1038/s41467-020-17885-z

PG 16

WC Multidisciplinary Sciences

WE Science Citation Index Expanded (SCI-EXPANDED)

SC Science & Technology - Other Topics

GA NF8TK

UT WOS:000563565300007

PM 32807793

OA gold, Green Published

DA 2025-04-09

ER

PT J

AU Liu, JM

Pan, F

Li, L

Liu, QR

Chen, Y

Xiong, XX

Cheng, KJ

Bin Yu, S

Shi, Z

Yu, ACH

Chen, XQ

AF Liu, Ju Mei

Pan, Feng

Li, Li

Liu, Qian Rong

Chen, Yong

Xiong, Xin Xin

Cheng, Kejun

Bin Yu, Shang

Shi, Zhi

Yu, Albert Cheung-Hoi

Chen, Xiao Qian

TI Piperlongumine selectively kills glioblastoma multiforme cells via

reactive oxygen species accumulation dependent JNK and p38 activation

SO BIOCHEMICAL AND BIOPHYSICAL RESEARCH COMMUNICATIONS

LA English

DT Article

DE Piplartine; Glioma; Oxidative stress; Apoptosis; Cancer therapy; Brain

tumor

ID CANCER-CELLS; NEUROGLOBIN; 14-3-3-GAMMA; APOPTOSIS

AB Piperlongumine (PL), a natural alkaloid isolated from the long pepper, may have anti-cancer properties. It selectively targets and kills cancer cells but leaves normal cells intact. Here, we reported that PL selectively killed glioblastoma multiforme (GBM) cells via accumulating reactive oxygen species (ROS) to activate JNK and p38. PL at 20 mu M could induce severe cell death in three GBM cell lines (LN229, U87 and 8MG) but not astrocytes in cultures. PL elevated ROS prominently and reduced glutathione levels in LN229 and 1387 cells. Antioxidant N-acetyl-L-cysteine (NAC) completely reversed PL-induced ROS accumulation and prevented cell death in LN229 and U87 cells. In LN229 and 1387 cells, PL-treatment activated JNK and p38 but not Erk and Akt, in a dosage-dependent manner. These activations could be blocked by NAC pre-treatment. JNK and p38 specific inhibitors, SB203580 and SP600125 respectively, significantly blocked the cytotoxic effects of PL in LN229 and 1387 cells. Our data first suggests that PL may have therapeutic potential for one of the most malignant and refractory tumors GBM. (c) 2013 Elsevier Inc. All rights reserved.

C1 [Liu, Ju Mei; Pan, Feng; Li, Li; Liu, Qian Rong; Chen, Yong; Xiong, Xin Xin; Bin Yu, Shang; Chen, Xiao Qian] Huazhong Univ Sci & Technol, Sch Basic Med, Dept Pathophysiol,Minist Educ, Key Lab Neurol Dis,Hubei Prov Key Lab Neurolog Di, Wuhan 430030, Peoples R China.

[Pan, Feng] Huazhong Univ Sci & Technol, Union Hosp, Dept Urol, Wuhan 430030, Peoples R China.

[Cheng, Kejun] Lishui Inst Agr Sci, Chem Biol Ctr, Lishui, Zhejiang 323300, Peoples R China.

[Cheng, Kejun] Vatalis Tech LLC, Houston, TX 77054 USA.

[Shi, Zhi] Jinan Univ, Dept Cell Biol, Guangzhou 510632, Guangdong, Peoples R China.

[Shi, Zhi] Jinan Univ, Coll Life Sci, Inst Biomed, Guangzhou 510632, Guangdong, Peoples R China.

[Yu, Albert Cheung-Hoi] Peking Univ, Neurosci Res Inst, Beijing 100191, Peoples R China.

[Yu, Albert Cheung-Hoi] Peking Univ, Sch Basic Med Sci, Key Lab Neurosci, Natl Hlth & Family Planning Commiss,Minist Educ, Beijing 100191, Peoples R China.

C3 Huazhong University of Science & Technology; Huazhong University of

Science & Technology; Jinan University; Jinan University; Peking

University; Peking University

RP Chen, XQ (corresponding author), Huazhong Univ Sci & Technol, Sch Basic Med, Dept Pathophysiol,Minist Educ, Key Lab Neurol Dis,Hubei Prov Key Lab Neurolog Di, Wuhan 430030, Peoples R China.

EM chenxiaoqian66@gmail.com

RI Chen, Jinyan/JJF-7875-2023; Liu, Di/HSH-1255-2023

OI Cheng, Kejun/0000-0002-1936-9129

FU National Natural Science Foundation of China [30570555, 81070937,

81172397, 81201726, 31271444]

FX We thank Dr. Ann Y.K. Wong for her helpful comments on the manuscript.

This work was supported by the National Natural Science Foundation of

China (Grants 30570555, 81070937, 81172397, 81201726 and 31271444).

CR Adams DJ, 2012, P NATL ACAD SCI USA, V109, P15115, DOI 10.1073/pnas.1212802109

Bezerra DP, 2013, EUR J PHARM SCI, V48, P453, DOI 10.1016/j.ejps.2012.12.003

Chen XQ, 2005, GLIA, V50, P182, DOI 10.1002/glia.20147

Chen XQ, 2003, GLIA, V42, P315, DOI 10.1002/glia.10185

Chen XQ, 2002, BIOCHEM BIOPH RES CO, V296, P657

Chin L, 2008, NATURE, V455, P1061, DOI 10.1038/nature07385

Evans SM, 2004, CLIN CANCER RES, V10, P8177, DOI 10.1158/1078-0432.CCR-04-1081

Gomez-Lazaro M, 2007, MOL PHARMACOL, V71, P736, DOI 10.1124/mol.106.030718

Jarvius M, 2013, BIOCHEM BIOPH RES CO, V431, P117, DOI 10.1016/j.bbrc.2013.01.017

Kumar S, 1999, BIOCHEM BIOPH RES CO, V263, P825, DOI 10.1006/bbrc.1999.1454

Liu HL, 2013, J CHROMATOGR B, V928, P78, DOI 10.1016/j.jchromb.2013.03.021

Raj L, 2011, NATURE, V475, P231, DOI 10.1038/nature10167

Randhawa H, 2013, TOXICOL IN VITRO, V27, P1626, DOI 10.1016/j.tiv.2013.04.006

Schwer CI, 2010, MOL PHARMACOL, V77, P660, DOI 10.1124/mol.109.059519

Son DJ, 2012, BIOCHEM BIOPH RES CO, V427, P349, DOI 10.1016/j.bbrc.2012.09.061

Stupp R, 2005, NEW ENGL J MED, V352, P987, DOI 10.1056/NEJMoa043330

Trachootham D, 2009, NAT REV DRUG DISCOV, V8, P579, DOI 10.1038/nrd2803

Wang T, 2001, J BIOL CHEM, V276, P20999, DOI 10.1074/jbc.M101355200

Xiao D, 2011, MOL PHARMACOL, V79, P499, DOI 10.1124/mol.110.068551

Ye SQ, 2009, ACTA PHARMACOL SIN, V30, P913, DOI 10.1038/aps.2009.70

Zhang J, 2013, MOL PHARMACOL, V83, P1109, DOI 10.1124/mol.112.083634

Zhao SM, 2009, SCIENCE, V324, P261, DOI 10.1126/science.1170944

NR 22

TC 88

Z9 91

U1 0

U2 44

PU ACADEMIC PRESS INC ELSEVIER SCIENCE

PI SAN DIEGO

PA 525 B ST, STE 1900, SAN DIEGO, CA 92101-4495 USA

SN 0006-291X

EI 1090-2104

J9 BIOCHEM BIOPH RES CO

JI Biochem. Biophys. Res. Commun.

PD JUL 19

PY 2013

VL 437

IS 1

BP 87

EP 93

DI 10.1016/j.bbrc.2013.06.042

PG 7

WC Biochemistry & Molecular Biology; Biophysics

WE Science Citation Index Expanded (SCI-EXPANDED)

SC Biochemistry & Molecular Biology; Biophysics

GA 190PP

UT WOS:000322353000015

PM 23796709

DA 2025-04-09

ER

PT J

AU Lu, GH

Wang, XJ

Li, F

Wang, S

Zhao, JW

Wang, JY

Liu, J

Lyu, CL

Ye, P

Tan, H

Li, WP

Ma, GH

Wei, W

AF Lu, Guihong

Wang, Xiaojun

Li, Feng

Wang, Shuang

Zhao, Jiawei

Wang, Jinyi

Liu, Jing

Lyu, Chengliang

Ye, Peng

Tan, Hui

Li, Weiping

Ma, Guanghui

Wei, Wei

TI Engineered biomimetic nanoparticles achieve targeted delivery and

efficient metabolism-based synergistic therapy against glioblastoma

SO NATURE COMMUNICATIONS

LA English

DT Article

ID OXIDATIVE STRESS; DRUG-DELIVERY; LACTATE; CELLS

AB Targeting cancer-associated metabolism is evolving as a promising approach for cancer therapy. Here, the authors generate cancer cell-membrane encapsulated nanoparticles to induce cell cycle arrest and cytotoxicity in lactate-high cancer cells, reducing tumourigensis in glioblastoma cell-line and patient-derived models.

Glioblastoma multiforme (GBM) is an aggressive brain cancer with a poor prognosis and few treatment options. Here, building on the observation of elevated lactate (LA) in resected GBM, we develop biomimetic therapeutic nanoparticles (NPs) that deliver agents for LA metabolism-based synergistic therapy. Because our self-assembling NPs are encapsulated in membranes derived from glioma cells, they readily penetrate the blood-brain barrier and target GBM through homotypic recognition. After reaching the tumors, lactate oxidase in the NPs converts LA into pyruvic acid (PA) and hydrogen peroxide (H2O2). The PA inhibits cancer cell growth by blocking histones expression and inducing cell-cycle arrest. In parallel, the H2O2 reacts with the delivered bis[2,4,5-trichloro-6-(pentyloxycarbonyl)phenyl] oxalate to release energy, which is used by the co-delivered photosensitizer chlorin e6 for the generation of cytotoxic singlet oxygen to kill glioma cells. Such a synergism ensures strong therapeutic effects against both glioma cell-line derived and patient-derived xenograft models.

C1 [Lu, Guihong; Wang, Jinyi; Liu, Jing; Tan, Hui; Li, Weiping] Shenzhen Univ, Shenzhen Peoples Hosp 2, Hlth Sci Ctr, Dept Neurosurg,Affiliated Hosp 1, Shenzhen 518035, Peoples R China.

[Lu, Guihong; Wang, Xiaojun; Li, Feng; Wang, Shuang; Zhao, Jiawei; Lyu, Chengliang; Ye, Peng; Ma, Guanghui; Wei, Wei] Chinese Acad Sci, Inst Proc Engn, State Key Lab Biochem Engn, Beijing 100190, Peoples R China.

[Li, Feng; Ma, Guanghui; Wei, Wei] Univ Chinese Acad Sci, Sch Chem Engn, Beijing 100049, Peoples R China.

[Tan, Hui] Shenzhen Childrens Hosp, Pneumol Dept, Shenzhen 518026, Peoples R China.

C3 Shenzhen University; Chinese Academy of Sciences; Institute of Process

Engineering, CAS; Chinese Academy of Sciences; University of Chinese

Academy of Sciences, CAS; Shenzhen Children's Hospital

RP Tan, H; Li, WP (corresponding author), Shenzhen Univ, Shenzhen Peoples Hosp 2, Hlth Sci Ctr, Dept Neurosurg,Affiliated Hosp 1, Shenzhen 518035, Peoples R China.; Ma, GH; Wei, W (corresponding author), Chinese Acad Sci, Inst Proc Engn, State Key Lab Biochem Engn, Beijing 100190, Peoples R China.; Ma, GH; Wei, W (corresponding author), Univ Chinese Acad Sci, Sch Chem Engn, Beijing 100049, Peoples R China.; Tan, H (corresponding author), Shenzhen Childrens Hosp, Pneumol Dept, Shenzhen 518026, Peoples R China.

EM huitan@email.szu.edu.cn; wpli@szu.edu.cn; ghma@ipe.ac.cn;

weiwei@ipe.ac.cn

RI tan, hui/KYP-1763-2024; Wang, Xiao-Jun/A-6036-2012; LI,

WEI/ISS-1208-2023; ye, peng/LWI-6154-2024; lu, guihong/GXH-8869-2022;

Wang, Shuang/LDG-0730-2024

OI Wang, Shuang/0000-0002-9189-602X; lu, guihong/0000-0002-9160-9588; Wang,

Xiaojun/0000-0001-8983-3233; Zhao, Jiawei/0000-0003-3343-4676; Li,

Feng/0000-0002-0045-1501; ye, peng/0000-0001-6265-0074; Lyu,

Chengliang/0000-0002-3999-3199

FU National Natural Science Foundation of China [32030062, U2001224,

32000988]; Science and Technology Innovation Commission of Shenzhen

[ZDSYS20200811142600003, ZDSYS20140509173142601]; China Postdoctoral

Science Foundation [2019TQ0212, 2020M672832]; Natural Science Foundation

of Guangdong Province [2021A1515010720, 2019A1515011750]; Basic and

Applied Basic Research Foundation Guangdong Province [2019A1515110876]

FX This work was supported by the National Natural Science Foundation of

China (32030062 to G.M., U2001224 to W.W., and 32000988 to G.L.),

Science and Technology Innovation Commission of Shenzhen

(ZDSYS20200811142600003 and ZDSYS20140509173142601 to H.T.), China

Postdoctoral Science Foundation (2019TQ0212, 2020M672832 to G.L.),

Natural Science Foundation of Guangdong Province (2021A1515010720 and

2019A1515011750 to H.T.), Basic and Applied Basic Research Foundation

Guangdong Province (2019A1515110876 to G.L.).

CR Barbosa BJAP, 2015, NEUROSURG REV, V38, P217, DOI 10.1007/s10143-014-0592-0

Alexander BM, 2018, CLIN CANCER RES, V24, P737, DOI 10.1158/1078-0432.CCR-17-0764

Alifieris C, 2015, PHARMACOL THERAPEUT, V152, P63, DOI 10.1016/j.pharmthera.2015.05.005

Avni D, 2003, MOL CELL, V12, P735, DOI 10.1016/S1097-2765(03)00355-1

Bagul PK, 2015, J NUTR BIOCHEM, V26, P1298, DOI 10.1016/j.jnutbio.2015.06.006

Chen QW, 2020, ANGEW CHEM INT EDIT, V59, P21562, DOI 10.1002/anie.202002649

Chen XH, 2019, ADV FUNCT MATER, V29, DOI 10.1002/adfm.201905785

Chen ZX, 2018, ADV FUNCT MATER, V28, DOI 10.1002/adfm.201803498

Cheng Y, 2014, ADV DRUG DELIVER REV, V66, P42, DOI 10.1016/j.addr.2013.09.006

Corbet C, 2018, NAT COMMUN, V9, DOI 10.1038/s41467-018-03525-0

Corbin Z, 2017, CURR ONCOL REP, V19, DOI 10.1007/s11912-017-0637-y

De Pasquale D, 2020, MATER DESIGN, V192, DOI 10.1016/j.matdes.2020.108742

Ding JJ, 2021, ADV MATER, V33, DOI 10.1002/adma.202005562

Doherty JR, 2013, J CLIN INVEST, V123, P3685, DOI 10.1172/JCI69741

Fan WP, 2016, CHEM SOC REV, V45, P6488, DOI 10.1039/c6cs00616g

Faubert B, 2020, SCIENCE, V368, P152, DOI 10.1126/science.aaw5473

Furtado D, 2018, ADV MATER, V30, DOI 10.1002/adma.201801362

Gao F, 2019, ADV MATER, V31, DOI 10.1002/adma.201904639

Gao SS, 2019, ADV SCI, V6, DOI 10.1002/advs.201801733

He CS, 2018, ADV FUNCT MATER, V28, DOI 10.1002/adfm.201705668

HEINTZ N, 1983, MOL CELL BIOL, V3, P539, DOI 10.1128/MCB.3.4.539

Hirschhaeuser F, 2011, CANCER RES, V71, P6921, DOI 10.1158/0008-5472.CAN-11-1457

Hui S, 2017, NATURE, V551, P115, DOI 10.1038/nature24057

Idris NM, 2012, NAT MED, V18, P1580, DOI 10.1038/nm.2933

Jia YL, 2019, ACS NANO, V13, P386, DOI 10.1021/acsnano.8b06556

Jia Y, 2016, ADV MATER, V28, P1312, DOI 10.1002/adma.201502581

Khan JA, 2007, EXPERT OPIN THER TAR, V11, P695, DOI 10.1517/14728222.11.5.695

Le A, 2010, P NATL ACAD SCI USA, V107, P2037, DOI 10.1073/pnas.0914433107

Lee DC, 2015, CELL, V161, P595, DOI 10.1016/j.cell.2015.03.011

Li JL, 2017, NAT COMMUN, V8, DOI 10.1038/s41467-017-01327-4

Lovett ML, 2020, ADV FUNCT MATER, V30, DOI 10.1002/adfm.201909146

Lu GH, 2019, CHEM SCI, V10, P4847, DOI 10.1039/c9sc00324j

Ma R, 2019, NUCLEIC ACIDS RES, V47, P11132, DOI 10.1093/nar/gkz864

Mao D, 2017, CHEM-US, V3, P991, DOI 10.1016/j.chempr.2017.10.002

Miao TT, 2019, ADV FUNCT MATER, V29, DOI 10.1002/adfm.201900259

Nacarelli T, 2019, NAT CELL BIOL, V21, P397, DOI 10.1038/s41556-019-0287-4

Palmieri EM, 2020, NAT COMMUN, V11, DOI 10.1038/s41467-020-14433-7

Park JH, 2020, CELLS-BASEL, V9, DOI 10.3390/cells9102308

Peng JH, 2021, ONCOL LETT, V21, DOI 10.3892/ol.2021.12630

Pérez-Tomás R, 2020, CANCERS, V12, DOI 10.3390/cancers12113244

Reichel D, 2020, ACS NANO, V14, P8392, DOI 10.1021/acsnano.0c02509

Sanai N, 2018, NAT REV CLIN ONCOL, V15, P112, DOI 10.1038/nrclinonc.2017.171

Shergalis A, 2018, PHARMACOL REV, V70, P412, DOI 10.1124/pr.117.014944

Sonveaux P, 2008, J CLIN INVEST, V118, P3930, DOI 10.1172/JCI36843

Tang W, 2019, CHEM SOC REV, V48, P2967, DOI 10.1039/c8cs00805a

Teh DBL, 2020, ADV MATER, V32, DOI 10.1002/adma.202001459

Tian ZM, 2019, SMALL, V15, DOI 10.1002/smll.201903746

Wang CX, 2020, ADV FUNCT MATER, V30, DOI 10.1002/adfm.201909369

Wang H, 2020, NAT CHEM, V12, P1102, DOI 10.1038/s41557-020-00587-w

Wu MY, 2018, NAT COMMUN, V9, DOI 10.1038/s41467-018-07250-6

Xue JW, 2017, NAT NANOTECHNOL, V12, P692, DOI [10.1038/nnano.2017.54, 10.1038/NNANO.2017.54]

Yi HG, 2019, NAT BIOMED ENG, V3, P509, DOI 10.1038/s41551-019-0363-x

Yin SY, 2020, SCI TRANSL MED, V12, DOI 10.1126/scitranslmed.aaz1723

Yu ZZ, 2018, NAT COMMUN, V9, DOI 10.1038/s41467-018-07197-8

Zanders ED, 2019, DRUG DISCOV TODAY, V24, P1193, DOI 10.1016/j.drudis.2019.03.008

Zhang J, 2021, NAT NANOTECHNOL, V16, P538, DOI 10.1038/s41565-020-00843-7

NR 56

TC 117

Z9 120

U1 27

U2 304

PU NATURE PORTFOLIO

PI BERLIN

PA HEIDELBERGER PLATZ 3, BERLIN, 14197, GERMANY

EI 2041-1723

J9 NAT COMMUN

JI Nat. Commun.

PD JUL 21

PY 2022

VL 13

IS 1

AR 4214

DI 10.1038/s41467-022-31799-y

PG 17

WC Multidisciplinary Sciences

WE Science Citation Index Expanded (SCI-EXPANDED)

SC Science & Technology - Other Topics

GA 3C6AG

UT WOS:000828703800003

PM 35864093

OA Green Published, gold

HC Y

HP N

DA 2025-04-09

ER

PT J

AU Lu, B

Wang, ZQ

Ding, Y

Wang, XZ

Lu, S

Wang, CC

He, C

Piao, MH

Chi, GF

Luo, YA

Ge, PF

AF Lu, Bin

Wang, Zongqi

Ding, Ye

Wang, Xuanzhong

Lu, Shan

Wang, Chongcheng

He, Chuan

Piao, Meihua

Chi, Guangfan

Luo, Yinan

Ge, Pengfei

TI RIP1 and RIP3 contribute to shikonin-induced glycolysis suppression in

glioma cells via increase of intracellular hydrogen peroxide

SO CANCER LETTERS

LA English

DT Article

DE Glycolysis; RIP1; RIP3; Hydrogen peroxide; Glioma

ID CHAPERONE-MEDIATED AUTOPHAGY; OXIDATIVE STRESS; PYRUVATE-KINASE;

BREAST-CANCER; NECROPTOSIS; APOPTOSIS; PHOSPHORYLATION; METABOLISM;

KNOCKDOWN; DEATH

AB RIP1 and RIP3 are necroptosis initiators, but their roles in regulation of glycolysis remain elusive, In this study, we found shikonin activated RIP1 and RIP3 in glioma cells in vitro and in vivo, which was accompanied with glycolysis suppression. Further investigation revealed that shikonin-induced decreases of glucose-6-phosphate and pyruvate and downregulation of HK II and PKM2 were significantly prevented when RIP1 or RIP3 was pharmacologically inhibited or genetically knocked down with SiRNA. Moreover, shikonin also triggered accumulation of intracellular H2O2 and depletion of GSH and cysteine. Mitigation of intracellular H2O2 via supplement of GSH reversed shikonin-induced glycolysis suppression. The role of intracellular H2O2 in regulation of glycolysis suppression was further confirmed in the cells treated with exogenous H2O2. Notably, inhibition of RIP1 or RIP3 prevented intracellular H2O2 accumulation, which was correlated with preventing shikonin-induced downregulation of x-CT and depletion of GSH and cysteine. In addition, supplement of pyruvate effectively inhibited shikonin- or exogenous H2O2-induced accumulation of intracellular H2O2 and glioma cell death. Taken together, we demonstrated in this study that RIP1 and RIP3 contributed to shikonin-induced glycolysis suppression via increasing intracellular H2O2. (C) 2018 Elsevier B.V. All rights reserved.

C1 [Lu, Bin; Wang, Zongqi; Ding, Ye; Wang, Xuanzhong; Lu, Shan; Wang, Chongcheng; He, Chuan; Luo, Yinan; Ge, Pengfei] Jilin Univ, Hosp 1, Dept Neurosurg, 71 Xinmin Ave, Changchun 130021, Jilin, Peoples R China.

[Lu, Bin; Wang, Zongqi; Ding, Ye; Wang, Xuanzhong; Lu, Shan; Wang, Chongcheng; He, Chuan; Luo, Yinan; Ge, Pengfei] Jilin Univ, Hosp 1, Res Ctr Neurosci, Changchun 130021, Jilin, Peoples R China.

[Piao, Meihua] Jilin Univ, Hosp 1, Dept Anesthesiol, Changchun 130021, Jilin, Peoples R China.

[Chi, Guangfan] Jilin Univ, Minist Educ, Key Lab Pathobiol, Changchun 130021, Jilin, Peoples R China.

C3 Jilin University; Jilin University; Jilin University; Jilin University

RP Ge, PF (corresponding author), Jilin Univ, Hosp 1, Dept Neurosurg, 71 Xinmin Ave, Changchun 130021, Jilin, Peoples R China.

EM gepf@jlu.edu.cn

RI Zhang, Xuan/HTM-2094-2023; Wang, Zongqi/KHC-3331-2024; He,

Chuan/HNO-9469-2023; Lu, Bin/JTS-4256-2023

OI Wang, Zongqi/0000-0003-4559-3420

FU National Nature and Science Foundation of China [81372697, 81772669];

Changbaishan Scholar Project of Jilin Province [2013026]; Scientific

Research Foundation of Jilin province [20160101127JC]

FX This work was supported by National Nature and Science Foundation of

China (81372697, 81772669), Changbaishan Scholar Project of Jilin

Province (2013026), and Scientific Research Foundation of Jilin province

(20160101127JC).

CR Bonapace L, 2010, J CLIN INVEST, V120, P1310, DOI 10.1172/JCI39987

Byun YJ, 2009, NEUROSCI LETT, V461, P131, DOI 10.1016/j.neulet.2009.06.011

Chen CH, 2012, ANN SURG ONCOL, V19, P3097, DOI 10.1245/s10434-012-2324-4

Chen J, 2011, ONCOGENE, V30, P4297, DOI 10.1038/onc.2011.137

Chen W, 2014, CELL DEATH DIFFER, V21, P1061, DOI 10.1038/cdd.2014.25

Chu BB, 2015, MOL MED REP, V12, P4358, DOI 10.3892/mmr.2015.3943

Dengler MA, 2011, PLOS ONE, V6, DOI 10.1371/journal.pone.0025139

Dixit D, 2014, CELL DEATH DIS, V5, DOI 10.1038/cddis.2014.179

Fu ZZ, 2013, BMC CANCER, V13, DOI 10.1186/1471-2407-13-580

Guo DQ, 2016, J MOL CELL CARDIOL, V91, P179, DOI 10.1016/j.yjmcc.2016.01.009

Heiden MGV, 2009, SCIENCE, V324, P1029, DOI 10.1126/science.1160809

Huang CJ, 2013, PLOS ONE, V8, DOI 10.1371/journal.pone.0066326

Iida M, 1997, J CANCER RES CLIN, V123, P619, DOI 10.1007/s004320050115

Kaushik N, 2015, SCI REP-UK, V5, DOI 10.1038/srep08726

Kim YS, 2007, MOL CELL, V26, P675, DOI 10.1016/j.molcel.2007.04.021

Lennicke C, 2015, CELL COMMUN SIGNAL, V13, DOI 10.1186/s12964-015-0118-6

Li JX, 2012, CELL, V150, P339, DOI 10.1016/j.cell.2012.06.019

Li L, 2017, EUR J PHARMACOL, V815, P274, DOI 10.1016/j.ejphar.2017.09.034

Li XB, 2015, THORAC CANCER, V6, P17, DOI 10.1111/1759-7714.12148

Lu B, 2017, ACTA PHARMACOL SIN, V38, P1543, DOI 10.1038/aps.2017.112

Luan WK, 2015, ONCOTARGET, V6, P13006, DOI 10.18632/oncotarget.3514

Lv L, 2011, MOL CELL, V42, P719, DOI 10.1016/j.molcel.2011.04.025

Ma EL, 2017, FREE RADICAL BIO MED, V113, P36, DOI 10.1016/j.freeradbiomed.2017.09.008

McCabe KE, 2014, CELL DEATH DIS, V5, DOI 10.1038/cddis.2014.448

McQuade T, 2013, BIOCHEM J, V456, P409, DOI 10.1042/BJ20130860

Melo-Lima Sara, 2014, Oncoscience, V1, P649

Miao Y, 2016, ONCOL RES, V24, P463, DOI 10.3727/096504016X14685034103671

Miki Y, 2015, LASER MED SCI, V30, P1739, DOI 10.1007/s10103-015-1783-9

Mukherjee J, 2013, PLOS ONE, V8, DOI 10.1371/journal.pone.0057610

Najafov A, 2017, TRENDS CANCER, V3, P294, DOI 10.1016/j.trecan.2017.03.002

Pasupuleti N, 2013, J PHARMACOL EXP THER, V344, P600, DOI 10.1124/jpet.112.200519

Rodemeister S, 2017, BIOCHEM BIOPH RES CO, V493, P1184, DOI 10.1016/j.bbrc.2017.09.138

Shahsavari Z, 2016, TUMOR BIOL, V37, P4479, DOI 10.1007/s13277-015-4258-5

Shimada K, 2016, MOL CELL ONCOL, V3, DOI 10.1080/23723556.2015.1091059

Silginer M, 2017, CELL DEATH DIS, V8, DOI 10.1038/cddis.2017.171

Stavrovskaya AA, 2016, BIOCHEMISTRY-MOSCOW+, V81, P91, DOI 10.1134/S0006297916020036

Tekade RK, 2017, DRUG DISCOV TODAY, V22, P1637, DOI 10.1016/j.drudis.2017.08.003

Varma SD, 2015, MOL CELL BIOCHEM, V403, P149, DOI 10.1007/s11010-015-2345-y

Wada N, 2015, INT J ONCOL, V46, P963, DOI 10.3892/ijo.2014.2804

Wang HY, 2014, MOL CELL, V54, P133, DOI 10.1016/j.molcel.2014.03.003

Wolf A, 2011, J EXP MED, V208, P313, DOI 10.1084/jem.20101470

Wu RX, 2012, EMBO MOL MED, V4, P633, DOI 10.1002/emmm.201200240

Xia HG, 2015, J CELL BIOL, V210, P705, DOI 10.1083/jcb.201503044

Zhang DW, 2009, SCIENCE, V325, P332, DOI 10.1126/science.1172308

Zhang L, 2015, TOXICOL LETT, V236, P43, DOI 10.1016/j.toxlet.2015.04.015

Zhao YH, 2011, CANCER RES, V71, P4585, DOI 10.1158/0008-5472.CAN-11-0127

Zheng LJ, 2017, MOL NEUROBIOL, V54, P3492, DOI 10.1007/s12035-016-9926-y

Zhou ZJ, 2017, CANCER LETT, V390, P77, DOI 10.1016/j.canlet.2017.01.004

Zou X, 2015, ONCOL REP, V34, P1895, DOI 10.3892/or.2015.4147

NR 49

TC 38

Z9 41

U1 1

U2 59

PU ELSEVIER IRELAND LTD

PI CLARE

PA ELSEVIER HOUSE, BROOKVALE PLAZA, EAST PARK SHANNON, CO, CLARE, 00000,

IRELAND

SN 0304-3835

EI 1872-7980

J9 CANCER LETT

JI Cancer Lett.

PY 2018

VL 425

BP 31

EP 42

DI 10.1016/j.canlet.2018.03.046

PG 12

WC Oncology

WE Science Citation Index Expanded (SCI-EXPANDED)

SC Oncology

GA GG5UJ

UT WOS:000432760900004

PM 29608987

DA 2025-04-09

ER

PT J

AU Shahbaz, U

Yu, XB

AF Shahbaz, Umar

Yu, Xiao bin

TI An analysis of brain tumor trace elements and their effects

SO TRACE ELEMENTS AND ELECTROLYTES

LA English

DT Review

DE brain tumor; trace elements; blood-brain barrier; reactive oxygen

species; glioma; benign; malignant; Parkinson's disease

ID X-RAY-FLUORESCENCE; COPPER HOMEOSTASIS; BARRIER SYSTEMS; IRON; ZINC;

LEAD; CALCIUM; EXPRESSION; LOCALIZATION; CADMIUM

AB Each year, there are more than 10 million new cancer cases, resulting in 12% of worldwide deaths are due to cancer. Trace elements are naturally found in the environment, and human exposure comes from various resources, including food, drinking water, and air. A slight quantity of trace elements have protective properties, excess of trace elements causes oxidative stress, that is why trace element homeostasis is critical for the appropriate performance of the human brain. Trace elements have been reported as having risk factors for various types of cancers such as stomach, brain, breast, lung, urinary bladder, and prostate, since trace element levels differ between noncancerous and cancerous tissues, various cancer types, and distinct malignancy grade. The few studies describing trace element concentrations in human brain tumor tissues showed the highest level of magnesium, low level of copper, and varying results for zinc as compared to normal brain tissues. However, a higher malignancy grade is associated with a higher concentration of lead, manganese, zinc, and mercury and a lower concentration of iron, calcium, sulfur, phosphorus, and cadmium. Furthermore, in this review, we present a summary of trace element concentration values found by various research groups.

C1 [Shahbaz, Umar; Yu, Xiao bin] Jiangnan Univ, Sch Biotechnol, Key Lab Carbohydrate Chem & Biotechnol, Minist Educ, 1800 Lihu Rd, Wuxi 214122, Jiangsu, Peoples R China.

C3 Jiangnan University

RP Yu, XB (corresponding author), Jiangnan Univ, Sch Biotechnol, Key Lab Carbohydrate Chem & Biotechnol, Minist Educ, 1800 Lihu Rd, Wuxi 214122, Jiangsu, Peoples R China.

EM xbyu@jiangnan.edu.cn

RI SHAHBAZ, UMAR/AAE-6492-2019

FU Chinese government scholarship

FX This work was supported by the Chinese government scholarship.

CR Akagi Y, 2018, BRAIN TUMOR PATHOL, V35, P81, DOI 10.1007/s10014-018-0313-4

Al-Saleh I, 2001, BIOL TRACE ELEM RES, V79, P197, DOI 10.1385/BTER:79:3:197

ANDRASI E, 1993, SCI TOTAL ENVIRON, V140, P399, DOI 10.1016/0048-9697(93)90036-6

Ansari KI, 2009, DALTON T, P8525, DOI 10.1039/b905276c

Asano K, 2018, BRAIN TUMOR PATHOL, V35, P131, DOI 10.1007/s10014-018-0320-5

Aschner Judy L., 2005, Molecular Aspects of Medicine, V26, P353, DOI 10.1016/j.mam.2005.07.003

Aschner M, 2005, CRIT REV TOXICOL, V35, P1, DOI 10.1080/10408440590905920

ASCHNER M, 1990, BRAIN RES BULL, V24, P857, DOI 10.1016/0361-9230(90)90152-P

Baldari S, 2020, INT J MOL SCI, V21, DOI 10.3390/ijms21031069

Barth RF, 2009, J NEURO-ONCOL, V94, P299, DOI 10.1007/s11060-009-9875-7

Bartzokis G, 2007, NEUROBIOL AGING, V28, P414, DOI 10.1016/j.neurobiolaging.2006.02.005

Bauer S, 2013, PHYS MED BIOL, V58, pR97, DOI 10.1088/0031-9155/58/13/R97

Becker JS, 2010, TRAC-TREND ANAL CHEM, V29, P966, DOI 10.1016/j.trac.2010.06.009

Becker JS, 2007, INT J MASS SPECTROM, V261, P68, DOI 10.1016/j.ijms.2006.07.016

Becker JS, 2005, ANAL CHEM, V77, P3208, DOI 10.1021/ac040184q

Bhatti P, 2011, OCCUP ENVIRON MED, V68, P4, DOI 10.1136/oem.2009.048132

Burdo JR, 2003, BIOMETALS, V16, P63, DOI 10.1023/A:1020718718550

Calzolari A, 2007, BLOOD CELL MOL DIS, V39, P82, DOI 10.1016/j.bcmd.2007.02.003

Chandra S, 1997, AM J PHYSIOL-RENAL, V273, pF939, DOI 10.1152/ajprenal.1997.273.6.F939

Chandra S, 2000, ANAL CHEM, V72, p104A, DOI 10.1021/ac002716i

Chandra S, 2016, J NEURO-ONCOL, V127, P33, DOI 10.1007/s11060-015-2022-8

Collingwood J, 2006, J ALZHEIMERS DIS, V10, P215

Cui Y, 2006, CANCER EPIDEM BIOMAR, V15, P1427, DOI 10.1158/1055-9965.EPI-06-0075

Davies KM, 2013, METALLOMICS, V5, P43, DOI 10.1039/c2mt20151h

Dehnhardt M, 2008, J TRACE ELEM MED BIO, V22, P17, DOI 10.1016/j.jtemb.2007.08.002

DEMAYO A, 1982, CRIT REV ENV CONTR, V12, P257, DOI 10.1080/10643388209381698

Depboylu C, 2007, NEURODEGENER DIS, V4, P218, DOI 10.1159/000101846

Dobrowolska J, 2008, TALANTA, V74, P717, DOI 10.1016/j.talanta.2007.06.051

Ebrahim AM, 2007, SCI TOTAL ENVIRON, V383, P52, DOI 10.1016/j.scitotenv.2007.04.047

Ferlay J, 2019, INT J CANCER, V144, P1941, DOI 10.1002/ijc.31937

Feske S, 2012, NAT REV IMMUNOL, V12, P532, DOI 10.1038/nri3233

Florianczyk B., 2007, J PRECLIN CLIN RES, V1, P16

FREDERICKSON CJ, 1989, INT REV NEUROBIOL, V31, P145

GERHARDSSON L, 1995, J TRACE ELEM MED BIO, V9, P136, DOI 10.1016/S0946-672X(11)80037-4

Góes P, 2018, J NEURO-ONCOL, V137, P331, DOI 10.1007/s11060-017-2721-4

Grochowski C, 2019, FRONT CHEM, V7, DOI 10.3389/fchem.2019.00115

Huang SQ, 2009, J INORG BIOCHEM, V103, P282, DOI 10.1016/j.jinorgbio.2008.10.019

IARC Working Group on the Evaluation of Carcinogenic Risks to Humans, 2006, IARC Monogr Eval Carcinog Risks Hum, V87, P1

Iuchi T, 2018, BRAIN TUMOR PATHOL, V35, P71, DOI 10.1007/s10014-018-0309-0

Jahnen-Dechent W, 2012, CLIN KIDNEY J, V5, P3, DOI 10.1093/ndtplus/sfr163

KONO Y, 1983, J BIOL CHEM, V258, P6015

Koriyama S, 2018, BRAIN TUMOR PATHOL, V35, P159, DOI 10.1007/s10014-018-0324-1

Krebs N, 2014, J TRACE ELEM MED BIO, V28, P1, DOI 10.1016/j.jtemb.2013.09.006

Kuwahara K, 2019, BRAIN TUMOR PATHOL, V36, P135, DOI 10.1007/s10014-019-00348-9

Law NA, 1999, ADV INORG CHEM, V46, P305

Lee TG, 2008, APPL SURF SCI, V255, P1241, DOI 10.1016/j.apsusc.2008.05.156

Leibold EA, 2001, HISTOCHEM CELL BIOL, V115, P195

Leskovjan AC, 2009, NEUROIMAGE, V47, P1215, DOI 10.1016/j.neuroimage.2009.05.063

Li SJ, 2014, PLOS ONE, V9, DOI 10.1371/journal.pone.0093900

Li Y, 2013, ONCOL REP, V29, P1805, DOI 10.3892/or.2013.2333

Liu YP, 2008, J NEUROCHEM, V105, P137, DOI 10.1111/j.1471-4159.2007.05118.x

Logeswari T., 2010, J Cancer Res Exp Oncol, V2, P006, DOI DOI 10.5897/JCREO2010.0185

López E, 2006, FREE RADICAL BIO MED, V40, P940, DOI 10.1016/j.freeradbiomed.2005.10.062

Louis DN, 2018, BRAIN TUMOR PATHOL, V35, P49, DOI 10.1007/s10014-018-0315-2

Louis DN, 2016, ACTA NEUROPATHOL, V131, P803, DOI 10.1007/s00401-016-1545-1

Luk E, 2003, P NATL ACAD SCI USA, V100, P10353, DOI 10.1073/pnas.1632471100

Maklad A, 2019, CANCERS, V11, DOI 10.3390/cancers11020145

Martinez-Finley EJ, 2013, FREE RADICAL BIO MED, V62, P65, DOI 10.1016/j.freeradbiomed.2013.01.032

Mittal M, 2019, APPL SOFT COMPUT, V78, P346, DOI 10.1016/j.asoc.2019.02.036

Mocchegiani E, 2005, PROG NEUROBIOL, V75, P367, DOI 10.1016/j.pneurobio.2005.04.005

Monteith GR, 2017, NAT REV CANCER, V17, P367, DOI 10.1038/nrc.2017.18

Monteith GR, 2012, J BIOL CHEM, V287, P31666, DOI 10.1074/jbc.R112.343061

Moos T, 1996, J COMP NEUROL, V375, P675, DOI 10.1002/(SICI)1096-9861(19961125)375:4<675::AID-CNE8>3.0.CO;2-Z

Mulware Stephen Juma, 2013, J Biophys, V2013, P192026, DOI 10.1155/2013/192026

Nimmrich V, 2013, BRIT J PHARMACOL, V169, P1203, DOI 10.1111/bph.12240

Nishioka H, 2018, BRAIN TUMOR PATHOL, V35, P57, DOI 10.1007/s10014-017-0307-7

Pal A, 2016, INDIAN J CLIN BIOCHE, V31, P93, DOI 10.1007/s12291-015-0503-3

Parent ME, 2017, ENVIRON HEALTH-GLOB, V16, DOI 10.1186/s12940-017-0300-y

Park SH, 2017, J PATHOL TRANSL MED, V51, P205, DOI 10.4132/jptm.2017.03.08

Pizzino G, 2014, REDOX BIOL, V2, P686, DOI 10.1016/j.redox.2014.05.003

Popescu BFG, 2009, PHYS MED BIOL, V54, P651, DOI 10.1088/0031-9155/54/3/012

Prevarskaya N, 2014, PHILOS T R SOC B, V369, DOI 10.1098/rstb.2013.0097

Ramos P, 2014, BIOL TRACE ELEM RES, V161, P190, DOI 10.1007/s12011-014-0093-6

Rodriguez A, 2007, J HISTOCHEM CYTOCHEM, V55, P85, DOI 10.1369/jhc.6A7031.2006

Roth JA, 2006, BIOL RES, V39, P45

Scheiber IF, 2013, NEUROCHEM INT, V62, P556, DOI 10.1016/j.neuint.2012.08.017

Shahbaz U, 2020, CURR PHARM BIOTECHNO, V21, P1433, DOI 10.2174/1389201021666200605104939

Sharma P, 2015, INDIAN J CLIN BIOCHE, V30, P1, DOI 10.1007/s12291-015-0480-6

Sharma S, 2017, NEUROCHEM INT, V108, P481, DOI 10.1016/j.neuint.2017.06.011

Shibuya M, 2018, BRAIN TUMOR PATHOL, V35, P62, DOI 10.1007/s10014-018-0311-6

Sian-Hülsmann J, 2011, J NEUROCHEM, V118, P939, DOI 10.1111/j.1471-4159.2010.07132.x

Singh L, 2012, LECT NOTES COMPUT SC, V7632, P94, DOI 10.1007/978-3-642-34123-6_9

Squitti R, 2012, J TRACE ELEM MED BIO, V26, P93, DOI 10.1016/j.jtemb.2012.04.012

Steenland K, 2019, OCCUP ENVIRON MED, V76, P603, DOI 10.1136/oemed-2019-105786

Strazielle N, 2013, MOL PHARMACEUT, V10, P1473, DOI 10.1021/mp300518e

Szczerbowska-Boruchowska M, 2011, J BIOL INORG CHEM, V16, P1217, DOI 10.1007/s00775-011-0810-y

SZERDAHELYI P, 1986, HISTOCHEMISTRY, V85, P341, DOI 10.1007/BF00493487

Takeda A, 2003, BRAIN RES, V965, P170, DOI 10.1016/S0006-8993(02)04165-3

Takeda A, 2001, CANCER RES, V61, P5065

Takeda A, 2000, BRAIN RES, V859, P352, DOI 10.1016/S0006-8993(00)02027-8

Tan CL, 2018, BRAIN TUMOR PATHOL, V35, P202, DOI 10.1007/s10014-018-0327-y

Tiiman A, 2013, J PEPT SCI, V19, P386, DOI 10.1002/psc.2513

Tougu V, 2009, J NEUROCHEM, V110, P1784, DOI 10.1111/j.1471-4159.2009.06269.x

TURECKY L, 1984, KLIN WOCHENSCHR, V62, P187, DOI 10.1007/BF01731643

Vuolo Laura, 2012, Front Endocrinol (Lausanne), V3, P58, DOI 10.3389/fendo.2012.00058

Wandzilak A, 2015, SPECTROCHIM ACTA B, V114, P52, DOI 10.1016/j.sab.2015.10.002

Wong RW, 2007, RETINA-J RET VIT DIS, V27, P997, DOI 10.1097/IAE.0b013e318074c290

Yamasaki T, 2018, BRAIN TUMOR PATHOL, V35, P209, DOI 10.1007/s10014-018-0326-z

Yuan Y, 2018, SCI REP-UK, V8, DOI 10.1038/s41598-018-27106-9

Zecca L, 2004, NAT REV NEUROSCI, V5, P863, DOI 10.1038/nrn1537

Zheng W, 2003, TOXICOL APPL PHARM, V192, P1, DOI 10.1016/S0041-008X(03)00251-5

Zheng W, 2012, PHARMACOL THERAPEUT, V133, P177, DOI 10.1016/j.pharmthera.2011.10.006

NR 102

TC 0

Z9 0

U1 2

U2 14

PU DUSTRI-VERLAG DR KARL FEISTLE

PI DEISENHOFEN-MUENCHEN

PA BAHNHOFSTRASSE 9 POSTFACH 49, D-82032 DEISENHOFEN-MUENCHEN, GERMANY

SN 0946-2104

J9 TRACE ELEM ELECTROLY

JI Trace Elem. Electrolytes

PY 2021

VL 39

IS 1

BP 32

EP 42

DI 10.5414/TEX01680

PG 11

WC Biochemistry & Molecular Biology; Endocrinology & Metabolism

WE Science Citation Index Expanded (SCI-EXPANDED)

SC Biochemistry & Molecular Biology; Endocrinology & Metabolism

GA XV0CL

UT WOS:000734621200005

DA 2025-04-09

ER

PT J

AU Chen, H

Li, CL

Hu, HY

Zhang, B

AF Chen, Hao

Li, Chunlin

Hu, Haiyang

Zhang, Bin

TI Activated TRPA1 plays a therapeutic role in TMZ resistance in

glioblastoma by altering mitochondrial dynamics

SO BMC MOLECULAR AND CELL BIOLOGY

LA English

DT Article

DE Glioblastoma; Temozolomide; TRPA1; Oxidative stress; Mitochondrial

dysfunction

ID OXIDATIVE STRESS; GLIOMA-CELLS; TEMOZOLOMIDE; CHANNELS; MULTIFORME;

EXPRESSION; APOPTOSIS; SURVIVAL

AB Background Glioblastoma (GBM) represents nearly one-half of primary brain tumors, and the median survival of patients with GBM is only 14.6 months. Surgery followed by radiation with concomitant temozolomide (TMZ) therapy is currently the standard of care. However, an increasing body of evidence suggests that GBM acquires resistance to TMZ, compromising the effect of the drug. Thus, further exploration into the mechanism underlying this resistance is urgently needed. Studies have demonstrated that TMZ resistance is associated with DNA damage, followed by altered reactive oxygen species (ROS) production in mitochondria. Studies have also showed that Ca2+-related transient receptor potential (TRP) channels participate in GBM cell proliferation and metastasis, but the detailed mechanism of their involvement remain to be studied. The present study demonstrates the role played by TRPA1 in TMZ resistance in GBM and elucidates the mechanism of resistance. Methods U251 and SHG-44 cells were analyzed in vitro. A CCK-8 assay was performed to verify the effect of TMZ toxicity on GBM cells. Intracellular ROS levels were detected by DCFH-DA assay. A MitoSOX Red assay was performed to determine the mitochondrial ROS levels. Intracellular Ca2+ levels in the cells were determined with a Fluo-4 AM calcium assay kit. Intracellular GSH levels were determined with GSH and GSSG Assay Kit. MGMT protein, Mitochondrial fission- and fusion-, apoptosis- and motility-related protein expression was detected by western blot assay. A recombinant lentiviral vector was used to infect human U251 cells to overexpress shRNA and generate TRPA1(+/+) and negative control cells. All experiments were repeated. Results In the U251 and SHG-44 cells, TMZ induced a small increase in the apoptosis rate and intracellular and mitochondrial ROS levels. The expression of antioxidant genes and antioxidants in these cells was also increased by TMZ. However, pretreatment with a TRPA1 agonist significantly decreased the level of antioxidant gene and antioxidants expression and enhanced intracellular and mitochondrial ROS levels. Also TMZ induced the level of MGMT protein increased, and pretreatment with a TRPA1 agonist decreased the MGMT expression. Moreover, Ca2+ influx, mitochondrial damage and cell apoptosis were promoted, and the balance between mitochondrial fission and fusion protein expression was disrupted in these GBM cells. Pretreatment with a TRPA1 inhibitor slightly enhanced the level of antioxidant gene expression and reduced the apoptosis rate. TRPA1 gene overexpression in the U251 cells was similar to that after inhibitor intervention, confirming the aforementioned experimental results. Conclusion The present study proved that activating TRPA1 in glioma cells, which leads to mitochondrial damage and dysfunction and ultimately to apoptosis, may decrease the TMZ resistance of GBM cells.

C1 [Chen, Hao; Zhang, Bin] Jining Med Univ, Dept Neurosurg, Affiliated Hosp, Jining, Peoples R China.

[Hu, Haiyang] Shanghai Jiao Tong Univ Affiliated Peoples Hosp 6, Dept Vasc Surg, 600 Yishan Rd, Shanghai 200233, Peoples R China.

[Li, Chunlin] Shanghai Jiao Tong Univ, Sch Med, Shanghai Gen Hosp, Trauma Ctr, Shanghai 201620, Peoples R China.

C3 Jining Medical University; Shanghai Jiao Tong University; Shanghai Jiao

Tong University

RP Zhang, B (corresponding author), Jining Med Univ, Dept Neurosurg, Affiliated Hosp, Jining, Peoples R China.; Hu, HY (corresponding author), Shanghai Jiao Tong Univ Affiliated Peoples Hosp 6, Dept Vasc Surg, 600 Yishan Rd, Shanghai 200233, Peoples R China.

EM huhaiyang1988@163.com; zhangbin8515@163.com

RI Hu, Haiyang/F-7544-2016; li, chunlin/KFS-0761-2024

FU Supporting Funds for Teacher's Research of Jining Medical University;

Shanghai Jiao Tong University Medical & Engineering Cross Fund

[YG2019QNA65]

FX This work was supported by grants from the Supporting Funds for

Teacher's Research of Jining Medical University (No.JYFC2019FKJ046 to

Bin Zhang) and Shanghai Jiao Tong University Medical & Engineering Cross

Fund (YG2019QNA65).

CR Alptekin M, 2015, TUMOR BIOL, V36, P9209, DOI 10.1007/s13277-015-3577-x

Aravamudan B, 2017, J CELL PHYSIOL, V232, P1053, DOI 10.1002/jcp.25508

Bautista DM, 2013, ANNU REV PHYSIOL, V75, P181, DOI 10.1146/annurev-physiol-030212-183811

Bi YM, 2018, CANCER LETT, V435, P66, DOI 10.1016/j.canlet.2018.07.040

Bu X, 2021, INT J BIOL SCI, V17, P3013, DOI 10.7150/ijbs.60894

Chien CH, 2019, J BIOMED SCI, V26, DOI 10.1186/s12929-019-0565-2

Chinigò G, 2021, FRONT CELL DEV BIOL, V9, DOI 10.3389/fcell.2021.617801

Deveci HA, 2019, BIOMED PHARMACOTHER, V111, P292, DOI 10.1016/j.biopha.2018.12.077

Gees M, 2010, CSH PERSPECT BIOL, V2, DOI 10.1101/cshperspect.a003962

Green DR, 2004, SCIENCE, V305, P626, DOI 10.1126/science.1099320

Guo RY, 2018, BIOMED J, V41, P9, DOI 10.1016/j.bj.2017.12.001

He C, 2021, ACTA PHARMACOL SIN, V42, P1324, DOI 10.1038/s41401-021-00663-y

Holland EC, 2000, P NATL ACAD SCI USA, V97, P6242, DOI 10.1073/pnas.97.12.6242

Johnson DR, 2012, J NEURO-ONCOL, V107, P359, DOI 10.1007/s11060-011-0749-4

Kang MJ, 2016, TUBERC RESPIR DIS, V79, P207, DOI 10.4046/trd.2016.79.4.207

Larson-Casey JL, 2020, REDOX BIOL, V33, DOI 10.1016/j.redox.2020.101426

Linz U, 2010, CANCER-AM CANCER SOC, V116, P1844, DOI 10.1002/cncr.24950

Louhivuori LM, 2009, J CELL PHYSIOL, V221, P67, DOI 10.1002/jcp.21828

Macpherson LJ, 2007, NATURE, V445, P541, DOI 10.1038/nature05544

Naziroglu M, 2017, FRONT PHYSIOL, V8, DOI 10.3389/fphys.2017.01040

Naziroglu M, 2012, J RECEPT SIG TRANSD, V32, P134, DOI 10.3109/10799893.2012.672994

Özkal B, 2020, CHILD NERV SYST, V36, P1283, DOI 10.1007/s00381-020-04567-w

Ozyerli-Goknar E, 2019, CELL DEATH DIS, V10, DOI 10.1038/s41419-019-2107-y

Pandey A, 2021, CANCERS, V13, DOI 10.3390/cancers13205114

Pang DJ, 2019, OXID MED CELL LONGEV, V2019, DOI 10.1155/2019/1805635

Park J, 2016, PHYTOMEDICINE, V23, P324, DOI 10.1016/j.phymed.2016.01.009

Poteser M, 2011, TRP CHANNELS, P91

Rehman FU, 2022, J CONTROL RELEASE, V345, P696, DOI 10.1016/j.jconrel.2022.03.036

Roos WP, 2007, ONCOGENE, V26, P186, DOI 10.1038/sj.onc.1209785

Samanta A, 2018, SUBCELL BIOCHEM, V87, P141, DOI 10.1007/978-981-10-7757-9_6

Sonoda Y, 2010, INT J CLIN ONCOL, V15, P352, DOI 10.1007/s10147-010-0065-6

Spiegl-Kreinecker S, 2010, NEURO-ONCOLOGY, V12, P28, DOI 10.1093/neuonc/nop003

Tai SH, 2021, TRANSL CANCER RES, V10, P3906, DOI 10.21037/tcr-20-3426

Takahashi N, 2018, CANCER CELL, V33, P985, DOI 10.1016/j.ccell.2018.05.001

Valtorta S, 2017, ONCOTARGET, V8, P113090, DOI 10.18632/oncotarget.23028

Wang MY, 2019, FREE RADICAL BIO MED, V134, P229, DOI 10.1016/j.freeradbiomed.2019.01.004

Wick W, 2014, NAT REV NEUROL, V10, P372, DOI 10.1038/nrneurol.2014.100

NR 37

TC 13

Z9 13

U1 0

U2 8

PU BMC

PI LONDON

PA CAMPUS, 4 CRINAN ST, LONDON N1 9XW, ENGLAND

EI 2661-8850

J9 BMC MOL CELL BIOL

JI BMC Mol. Cell Biol.

PD AUG 19

PY 2022

VL 23

IS 1

AR 38

DI 10.1186/s12860-022-00438-1

PG 14

WC Cell Biology

WE Science Citation Index Expanded (SCI-EXPANDED)

SC Cell Biology

GA 3V6DE

UT WOS:000841751000001

PM 35982414

OA Green Published, gold

DA 2025-04-09

ER

PT J

AU Murnan, KM

Horbinski, C

Stegh, AH

AF Murnan, Kevin M. M.

Horbinski, Craig

Stegh, Alexander H. H.

TI Redox Homeostasis and Beyond: The Role of Wild-Type Isocitrate

Dehydrogenases for the Pathogenesis of Glioblastoma

SO ANTIOXIDANTS & REDOX SIGNALING

LA English

DT Review

DE glioma; isocitrate dehydrogenases; metabolism; cancer immunology; small

molecule inhibitor

ID INTEGRATED GENOMIC ANALYSIS; HYPOXIA-INDUCIBLE FACTORS; CANCER

STEM-CELLS; OUTER RADIAL GLIA; ALPHA-KETOGLUTARATE;

GLUTAMINE-METABOLISM; SUBVENTRICULAR ZONE; TARGETED THERAPIES; OXIDATIVE

DAMAGE; PROMOTES GROWTH

AB Significance: Glioblastoma is an aggressive and devastating brain tumor characterized by a dismal prognosis and resistance to therapeutic intervention. To support catabolic processes critical for unabated cellular growth and defend against harmful reactive oxygen species, glioblastoma tumors upregulate the expression of wild-type isocitrate dehydrogenases (IDHs). IDH enzymes catalyze the oxidative decarboxylation of isocitrate to alpha-ketoglutarate (alpha-KG), NAD(P)H, and CO2. On molecular levels, IDHs epigenetically control gene expression through effects on alpha-KG-dependent dioxygenases, maintain redox balance, and promote anaplerosis by providing cells with NADPH and precursor substrates for macromolecular synthesis.Recent Advances: While gain-of-function mutations in IDH1 and IDH2 represent one of the most comprehensively studied mechanisms of IDH pathogenic effects, recent studies identified wild-type IDHs as critical regulators of normal organ physiology and, when transcriptionally induced or down regulated, as contributing to glioblastoma progression.Critical Issues: Here, we will discuss molecular mechanisms of how wild-type IDHs control glioma pathogenesis, including the regulation of oxidative stress and de novo lipid biosynthesis, and provide an overview of current and future research directives that aim to fully characterize wild-type IDH-driven metabolic reprogramming and its contribution to the pathogenesis of glioblastoma.Future Directions: Future studies are required to further dissect mechanisms of metabolic and epigenomic reprogramming in tumors and the tumor microenvironment, and to develop pharmacological approaches to inhibit wild-type IDH function.

C1 [Murnan, Kevin M. M.] Northwestern Univ, Malnati Brain Tumor Inst, Robert H Lurie Comprehens Canc Ctr, Ken & Ruth Davee Dept Neurol,Feinberg Sch Med, Chicago, IL USA.

[Horbinski, Craig] Northwestern Univ, Malnati Brain Tumor Inst, Robert H Lurie Comprehens Canc Ctr, Dept Pathol,Feinberg Sch Med, Chicago, IL USA.

[Horbinski, Craig] Northwestern Univ, Malnati Brain Tumor Inst, Robert H Lurie Comprehens Canc Ctr, Dept Neurol Surg,Feinberg Sch Med, Chicago, IL USA.

[Stegh, Alexander H. H.] Washington Univ, Brain Tumor Ctr, Alvin J Siteman Comprehens Canc Ctr, Dept Neurol Surg,Sch Med, 4590 Childrens Pl,Suite 8200, St Louis, MO 63130 USA.

C3 Northwestern University; Feinberg School of Medicine; Ann & Robert H.

Lurie Children's Hospital of Chicago; Robert H. Lurie Comprehensive

Cancer Center; Northwestern University; Feinberg School of Medicine; Ann

& Robert H. Lurie Children's Hospital of Chicago; Robert H. Lurie

Comprehensive Cancer Center; Ann & Robert H. Lurie Children's Hospital

of Chicago; Northwestern University; Feinberg School of Medicine; Robert

H. Lurie Comprehensive Cancer Center; Washington University (WUSTL);

Siteman Cancer Center

RP Stegh, AH (corresponding author), Washington Univ, Brain Tumor Ctr, Alvin J Siteman Comprehens Canc Ctr, Dept Neurol Surg,Sch Med, 4590 Childrens Pl,Suite 8200, St Louis, MO 63130 USA.

EM stegh@wustl.edu

OI Stegh, Alexander/0000-0003-2395-7967

FU Northwestern University Brain Tumor SPORE grant [R01NS129123]; NIH

[P50CA221747, R01NS118039, R01NS117104, R01NS102669]; [T32CA009560]

FX This research was supported by R01NS129123 (to Alexander H. Stegh), the

Northwestern University Brain Tumor SPORE grant (P50CA221747) to Craig

Horbinski and Alexander H.Stegh; R01NS118039, R01NS117104, R01NS102669

(to Craig Horbinski), and an NIH T32CA009560 (to Kevin M. Murnan).

CR Alcantara Llaguno Sheila R, 2016, Cold Spring Harb Symp Quant Biol, V81, P31, DOI 10.1101/sqb.2016.81.030973

Altman BJ, 2016, NAT REV CANCER, V16, P619, DOI 10.1038/nrc.2016.71

Amankulor NM, 2017, GENE DEV, V31, P774, DOI 10.1101/gad.294991.116

Anderson DD, 2012, J BIOL CHEM, V287, P7051, DOI 10.1074/jbc.M111.333120

BANNAI S, 1986, J BIOL CHEM, V261, P2256

Bao SD, 2006, NATURE, V444, P756, DOI 10.1038/nature05236

Bar EE, 2010, AM J PATHOL, V177, P1491, DOI 10.2353/ajpath.2010.091021

Bélanger M, 2011, CELL METAB, V14, P724, DOI 10.1016/j.cmet.2011.08.016

Bernal A, 2018, CELL MOL LIFE SCI, V75, P2177, DOI 10.1007/s00018-018-2794-z

Bhaduri A, 2020, CELL STEM CELL, V26, P48, DOI 10.1016/j.stem.2019.11.015

Brat DJ, 2004, CANCER RES, V64, P920, DOI 10.1158/0008-5472.CAN-03-2073

Brennan CW, 2013, CELL, V155, P462, DOI 10.1016/j.cell.2013.09.034

Büchler P, 2003, PANCREAS, V26, P56, DOI 10.1097/00006676-200301000-00010

Bzymek KP, 2007, BIOCHEMISTRY-US, V46, P5391, DOI 10.1021/bi700061t

Calver AR, 1998, NEURON, V20, P869, DOI 10.1016/S0896-6273(00)80469-9

Calvert AE, 2017, CELL REP, V19, P1858, DOI 10.1016/j.celrep.2017.05.014

Campos B, 2016, ONCOGENE, V35, P5819, DOI 10.1038/onc.2016.85

Carey BW, 2015, NATURE, V518, P413, DOI 10.1038/nature13981

Carmeliet P, 2011, NAT REV DRUG DISCOV, V10, P417, DOI 10.1038/nrd3455

Carta G, 2017, FRONT PHYSIOL, V8, DOI 10.3389/fphys.2017.00902

Cejalvo T, 2020, CANCERS, V12, DOI 10.3390/cancers12113230

Chakravarti A, 2006, CLIN CANCER RES, V12, P4738, DOI 10.1158/1078-0432.CCR-06-0596

Chen J, 2012, NATURE, V488, P522, DOI 10.1038/nature11287

Chen RH, 2014, P NATL ACAD SCI USA, V111, P14217, DOI 10.1073/pnas.1409653111

Chen Y, 2005, J BIOL CHEM, V280, P33766, DOI 10.1074/jbc.M504604200

Cheng CM, 2018, CANCER COMMUN, V38, DOI 10.1186/s40880-018-0301-4

Cheng X, 2018, CURR TOP MED CHEM, V18, P484, DOI 10.2174/1568026618666180523104541

Choi EJ, 2014, BMC CANCER, V14, DOI 10.1186/1471-2407-14-17

Chung C, 2020, CANCER CELL, V38, P334, DOI 10.1016/j.ccell.2020.07.008

Claes A, 2007, ACTA NEUROPATHOL, V114, P443, DOI 10.1007/s00401-007-0293-7

Cloughesy TF, 2014, ANNU REV PATHOL-MECH, V9, P1, DOI 10.1146/annurev-pathol-011110-130324

Cuddapah VA, 2014, NAT REV NEUROSCI, V15, P455, DOI 10.1038/nrn3765

Dahmane N, 1999, DEVELOPMENT, V126, P3089

Dang L, 2009, NATURE, V462, P739, DOI 10.1038/nature08617

Deng GJ, 2015, J BIOL CHEM, V290, P762, DOI 10.1074/jbc.M114.608497

Dirkse A, 2019, NAT COMMUN, V10, DOI 10.1038/s41467-019-09853-z

ENOCH HG, 1976, J BIOL CHEM, V251, P5095

Glasauer A, 2013, CURR BIOL, V23, pR100, DOI 10.1016/j.cub.2012.12.011

Gonzalez FJ, 2018, NAT REV ENDOCRINOL, V15, P21, DOI 10.1038/s41574-018-0096-z

GOPAL K, 1963, Acta Neurochir (Wien), V11, P333, DOI 10.1007/BF01402012

Guo DL, 2009, SCI SIGNAL, V2, DOI 10.1126/scisignal.2000446

HALL TR, 1993, J BIOL CHEM, V268, P3092

Hanahan D, 2011, CELL, V144, P646, DOI 10.1016/j.cell.2011.02.013

Hansen DV, 2010, NATURE, V464, P554, DOI 10.1038/nature08845

Hatoum A, 2019, CANCER MANAG RES, V11, P1843, DOI 10.2147/CMAR.S186142

Hwang IY, 2016, CELL METAB, V24, P494, DOI 10.1016/j.cmet.2016.06.014

Imayoshi I, 2010, J NEUROSCI, V30, P3489, DOI 10.1523/JNEUROSCI.4987-09.2010

Itsumi M, 2015, CELL DEATH DIFFER, V22, P1837, DOI 10.1038/cdd.2015.38

Jackson EL, 2006, NEURON, V51, P187, DOI 10.1016/j.neuron.2006.06.012

Jakob CG, 2018, J MED CHEM, V61, P6647, DOI 10.1021/acs.jmedchem.8b00305

Jiang L, 2016, NATURE, V532, P255, DOI 10.1038/nature17393

Jiang XJ, 2021, NAT REV MOL CELL BIO, V22, P266, DOI 10.1038/s41580-020-00324-8

Jo SH, 2001, J BIOL CHEM, V276, P16168, DOI 10.1074/jbc.M010120200

Jue TR, 2016, J NEURO-ONCOL, V127, P427, DOI 10.1007/s11060-016-2080-6

Kaur B, 2005, NEURO-ONCOLOGY, V7, P134, DOI 10.1215/S1152851704001115

Keith B, 2007, CELL, V129, P465, DOI 10.1016/j.cell.2007.04.019

Koh HJ, 2004, J BIOL CHEM, V279, P39968, DOI 10.1074/jbc.M402260200

Koh KP, 2011, CELL STEM CELL, V8, P200, DOI 10.1016/j.stem.2011.01.008

Kong MJ, 2018, CELL DEATH DIS, V9, DOI 10.1038/s41419-018-0537-6

Koundouros N, 2020, BRIT J CANCER, V122, P4, DOI 10.1038/s41416-019-0650-z

Krebs HA, 1937, BIOCHEM J, V31, P645, DOI 10.1042/bj0310645

Krell D, 2011, PLOS ONE, V6, DOI 10.1371/journal.pone.0019868

Lai A, 2011, J CLIN ONCOL, V29, P4482, DOI 10.1200/JCO.2010.33.8715

Lai K, 2003, NAT NEUROSCI, V6, P21, DOI 10.1038/nn983

Lang XT, 2019, CANCER DISCOV, V9, P1673, DOI 10.1158/2159-8290.CD-19-0338

Lathia JD, 2015, GENE DEV, V29, P1203, DOI 10.1101/gad.261982.115

Lee SM, 2002, FREE RADICAL BIO MED, V32, P1185, DOI 10.1016/S0891-5849(02)00815-8

Li Z, 2009, CANCER CELL, V15, P501, DOI 10.1016/j.ccr.2009.03.018

Liao P, 2022, CANCER CELL, V40, P365, DOI 10.1016/j.ccell.2022.02.003

Liou GY, 2010, FREE RADICAL RES, V44, P479, DOI 10.3109/10715761003667554

Liu C, 2011, CELL, V146, P209, DOI 10.1016/j.cell.2011.06.014

Llaguno SA, 2019, NAT NEUROSCI, V22, P545, DOI 10.1038/s41593-018-0333-8

Llaguno SRA, 2016, BRIT J CANCER, V115, P1445, DOI 10.1038/bjc.2016.354

Llaguno SRA, 2015, CANCER CELL, V28, P429, DOI 10.1016/j.ccell.2015.09.007

Louis DN, 2016, ACTA NEUROPATHOL, V131, P803, DOI 10.1007/s00401-016-1545-1

Lukas RV, 2019, ONCOLOGY-NY, V33, P91

Magtanong L, 2019, CELL CHEM BIOL, V26, P420, DOI 10.1016/j.chembiol.2018.11.016

Martínez-Reyes I, 2020, NAT COMMUN, V11, DOI 10.1038/s41467-019-13668-3

Mashimo T, 2014, CELL, V159, P1603, DOI 10.1016/j.cell.2014.11.025

Matsuzaka T, 2007, NAT MED, V13, P1193, DOI 10.1038/nm1662

May JL, 2019, SCI ADV, V5, DOI 10.1126/sciadv.aat0456

McKinnon C, 2021, BMJ-BRIT MED J, V374, DOI 10.1136/bmj.n1560

Mehrmohamadi M, 2014, CELL REP, V9, P1507, DOI 10.1016/j.celrep.2014.10.026

Metallo CM, 2012, NATURE, V481, P380, DOI 10.1038/nature10602

Monteiro AR, 2017, CELLS-BASEL, V6, DOI 10.3390/cells6040045

Monticone M, 2014, PLOS ONE, V9, DOI 10.1371/journal.pone.0090085

Moran-Crusio K, 2011, CANCER CELL, V20, P11, DOI 10.1016/j.ccr.2011.06.001

Mu LY, 2018, FRONT MOL NEUROSCI, V11, DOI 10.3389/fnmol.2018.00082

Nagaraj R, 2017, CELL, V168, P210, DOI 10.1016/j.cell.2016.12.026

Neftel C, 2019, CELL, V178, P835, DOI 10.1016/j.cell.2019.06.024

Nishiyama A, 2009, NAT REV NEUROSCI, V10, P9, DOI 10.1038/nrn2495

Ohgaki H, 2005, ACTA NEUROPATHOL, V109, P93, DOI 10.1007/s00401-005-0991-y

Ohgaki H, 2013, CLIN CANCER RES, V19, P764, DOI 10.1158/1078-0432.CCR-12-3002

Olar A, 2014, J PATHOL, V232, P165, DOI 10.1002/path.4282

Osswald M, 2015, NATURE, V528, P93, DOI 10.1038/nature16071

Palazon A, 2014, IMMUNITY, V41, P518, DOI 10.1016/j.immuni.2014.09.008

Parsons DW, 2008, SCIENCE, V321, P1807, DOI 10.1126/science.1164382

Patel AP, 2014, SCIENCE, V344, P1396, DOI 10.1126/science.1254257

Pavlova NN, 2022, CELL METAB, V34, P355, DOI 10.1016/j.cmet.2022.01.007

Peng JC, 2009, CELL, V139, P1290, DOI 10.1016/j.cell.2009.12.002

Phillips HS, 2006, CANCER CELL, V9, P157, DOI 10.1016/j.ccr.2006.02.019

Piccirillo SGM, 2006, NATURE, V444, P761, DOI 10.1038/nature05349

Pistollato F, 2007, MOL CELL NEUROSCI, V35, P424, DOI 10.1016/j.mcn.2007.04.003

Pollen AA, 2015, CELL, V163, P55, DOI 10.1016/j.cell.2015.09.004

PRINGLE NP, 1993, DEVELOPMENT, V117, P525

Qattan AT, 2012, J PROTEOME RES, V11, P6080, DOI 10.1021/pr300736v

Qi F, 2008, BBA-PROTEINS PROTEOM, V1784, P1641, DOI 10.1016/j.bbapap.2008.07.001

Qin EY, 2017, CELL, V170, P845, DOI 10.1016/j.cell.2017.07.016

Roth AD, 2016, ADV EXP MED BIOL, V949, P167, DOI 10.1007/978-3-319-40764-7_8

SCHERER H. J., 1938, AMER JOUR CANCER, V34, P333

Seidel S, 2010, BRAIN, V133, P983, DOI 10.1093/brain/awq042

Sharma S, 2017, PLOS GENET, V13, DOI [10.1371/journal.pgen.1007019, 10.1371/journal.pgen.1006804]

Shechter I, 2003, J LIPID RES, V44, P2169, DOI 10.1194/jlr.M300285-JLR200

Shi JL, 2014, NEUROL SCI, V35, P839, DOI 10.1007/s10072-013-1607-2

Shi Y, 2017, NAT COMMUN, V8, DOI 10.1038/ncomms15080

Sies H, 2022, NAT REV MOL CELL BIO, V23, P499, DOI 10.1038/s41580-022-00456-z

Singh SK, 2003, CANCER RES, V63, P5821

Snaebjornsson MT, 2020, CELL METAB, V31, P62, DOI 10.1016/j.cmet.2019.11.010

Soda Y, 2011, P NATL ACAD SCI USA, V108, P4274, DOI 10.1073/pnas.1016030108

Soeda A, 2009, ONCOGENE, V28, P3949, DOI 10.1038/onc.2009.252

Stockwell BR, 2020, TRENDS CELL BIOL, V30, P478, DOI 10.1016/j.tcb.2020.02.009

Stockwell BR, 2020, CELL CHEM BIOL, V27, P365, DOI 10.1016/j.chembiol.2020.03.013

Stricker SH, 2013, GENE DEV, V27, P654, DOI 10.1101/gad.212662.112

Sullivan BA, 2004, NAT STRUCT MOL BIOL, V11, P1076, DOI 10.1038/nsmb845

Suvà ML, 2020, CANCER CELL, V37, P630, DOI 10.1016/j.ccell.2020.04.001

Suvà ML, 2014, CELL, V157, P580, DOI 10.1016/j.cell.2014.02.030

Tan AC, 2020, CA-CANCER J CLIN, V70, P299, DOI 10.3322/caac.21613

TeSlaa T, 2016, CELL METAB, V24, P485, DOI 10.1016/j.cmet.2016.07.002

Tönjes M, 2013, NAT MED, V19, P901, DOI 10.1038/nm.3217

Tommasini-Ghelfi S, 2019, SCI ADV, V5, DOI 10.1126/sciadv.aaw4543

Tsai JW, 2007, NAT NEUROSCI, V10, P970, DOI 10.1038/nn1934

Tsukada Y, 2006, NATURE, V439, P811, DOI 10.1038/nature04433

van Tilborg E, 2018, GLIA, V66, P221, DOI 10.1002/glia.23256

Vander Heiden MG, 2017, CELL, V168, DOI 10.1016/j.cell.2016.12.039

Vaziri-Gohar A, 2022, NAT CANCER, V3, P852, DOI 10.1038/s43018-022-00393-y

Verhaak RGW, 2010, CANCER CELL, V17, P98, DOI 10.1016/j.ccr.2009.12.020

Wahl DR, 2017, CANCER RES, V77, P960, DOI 10.1158/0008-5472.CAN-16-2008

Walport LJ, 2016, NAT COMMUN, V7, DOI 10.1038/ncomms11974

Wang B, 2020, EMBO REP, V21, DOI 10.15252/embr.201948183

Wang QH, 2017, CANCER CELL, V32, P42, DOI [10.1016/j.ccell.2017.06.003, 10.1016/j.ccell.2017.12.012]

Ward PS, 2013, J BIOL CHEM, V288, P3804, DOI 10.1074/jbc.M112.435495

Westphal M, 2011, NAT REV NEUROSCI, V12, P495, DOI 10.1038/nrn3060

Wise DR, 2011, P NATL ACAD SCI USA, V108, P19611, DOI 10.1073/pnas.1117773108

Xiong ZJ, 2021, FRONT ONCOL, V11, DOI 10.3389/fonc.2021.782043

Xu X, 2004, J BIOL CHEM, V279, P33946, DOI 10.1074/jbc.M404298200

Yan H, 2009, NEW ENGL J MED, V360, P765, DOI 10.1056/NEJMoa0808710

Yang H, 2012, CLIN CANCER RES, V18, P5562, DOI 10.1158/1078-0432.CCR-12-1773

Yin F, 2017, FRONT AGING NEUROSCI, V9, DOI 10.3389/fnagi.2017.00209

You MX, 2017, NAT NANOTECHNOL, V12, P453, DOI [10.1038/NNANO.2017.23, 10.1038/nnano.2017.23]

Zong H, 2015, CSH PERSPECT BIOL, V7, DOI 10.1101/cshperspect.a020610

NR 150

TC 4

Z9 4

U1 2

U2 8

PU MARY ANN LIEBERT, INC

PI NEW ROCHELLE

PA 140 HUGUENOT STREET, 3RD FL, NEW ROCHELLE, NY 10801 USA

SN 1523-0864

EI 1557-7716

J9 ANTIOXID REDOX SIGN

JI Antioxid. Redox Signal.

PD NOV 1

PY 2023

VL 39

IS 13-15

BP 923

EP 941

DI 10.1089/ars.2023.0262

EA JUN 2023

PG 19

WC Biochemistry & Molecular Biology; Endocrinology & Metabolism

WE Science Citation Index Expanded (SCI-EXPANDED)

SC Biochemistry & Molecular Biology; Endocrinology & Metabolism

GA Y4IZ0

UT WOS:001011177700001

PM 37132598

OA Green Published

DA 2025-04-09

ER

PT J

AU Tan, JY

Duan, XH

Zhang, F

Ban, XH

Mao, JJ

Cao, MH

Han, SS

Shuai, XT

Shen, J

AF Tan, Junyi

Duan, Xiaohui

Zhang, Fang

Ban, Xiaohua

Mao, Jiaji

Cao, Minghui

Han, Shisong

Shuai, Xintao

Shen, Jun

TI Theranostic Nanomedicine for Synergistic Chemodynamic Therapy and

Chemotherapy of Orthotopic Glioma

SO ADVANCED SCIENCE

LA English

DT Article

DE chemodynamic therapy; chemotherapy; glioma; magnetic resonance imaging;

tumor microenvironment

ID ALBUMIN-MNO2 NANOPARTICLES; TUMOR MICROENVIRONMENT; CO-DELIVERY;

HYPOXIA; OXYGEN; OPPORTUNITIES; TEMOZOLOMIDE; DOXORUBICIN; EXPRESSION;

STRATEGIES

AB Glioma is a common primary brain malignancy with a poor prognosis. Chemotherapy is the first-line treatment for brain tumors but low efficiency of drugs in crossing the blood-brain barrier (BBB) and drug resistance related to tumor hypoxia thwart its efficacy. Herein, a theranostic nanodrug (iRPPA@TMZ/MnO) is developed by incorporating oleic acid-modified manganese oxide (MnO) and temozolomide (TMZ) into a polyethylene glycol-poly(2-(diisopropylamino)ethyl methacrylate-based polymeric micelle containing internalizing arginine-glycine-aspartic acid (iRGD). The presence of iRGD provides the nanodrug with a high capacity of crossing the BBB and penetrating the tumor tissue. After accumulation in glioma, the nanodrug responds to the tumor microenvironment to simultaneously release TMZ, Mn2+, and O-2. The released TMZ induces tumor cell apoptosis and the released Mn2+ causes intracellular oxidative stress that kill tumor cells via a Fenton-like reaction. The O-2 produced in situ alleviates tumor hypoxia and enhances the chemotherapy/chemodynamic therapeutic effects against glioma. The Mn2+ can also serve as a magnetic resonance imaging (MRI) contrast agent for tumor imaging during therapy. The study demonstrates the great potential of this multifunctional nanodrug for MRI-visible therapy of brain glioma.

C1 [Tan, Junyi; Duan, Xiaohui; Zhang, Fang; Mao, Jiaji; Cao, Minghui; Shuai, Xintao; Shen, Jun] Sun Yat Sen Univ, Sun Yat Sen Mem Hosp, Dept Radiol, Guangzhou 510120, Peoples R China.

[Tan, Junyi; Han, Shisong; Shuai, Xintao] Sun Yat Sen Univ, Sch Mat Sci & Engn, PCFM Lab, Minist Educ, Guangzhou 510275, Peoples R China.

[Duan, Xiaohui; Zhang, Fang; Mao, Jiaji; Cao, Minghui; Shen, Jun] Sun Yat Sen Univ, Sun Yat Sen Mem Hosp, Guangdong Prov Key Lab Malignant Tumour Epigenet, Guangzhou 510120, Peoples R China.

[Ban, Xiaohua] Sun Yat Sen Univ, Ctr Canc, Dept Radiol, Guangzhou 510060, Peoples R China.

C3 Sun Yat Sen University; Sun Yat Sen University; Sun Yat Sen University;

Sun Yat Sen University

RP Shuai, XT; Shen, J (corresponding author), Sun Yat Sen Univ, Sun Yat Sen Mem Hosp, Dept Radiol, Guangzhou 510120, Peoples R China.; Shuai, XT (corresponding author), Sun Yat Sen Univ, Sch Mat Sci & Engn, PCFM Lab, Minist Educ, Guangzhou 510275, Peoples R China.; Shen, J (corresponding author), Sun Yat Sen Univ, Sun Yat Sen Mem Hosp, Guangdong Prov Key Lab Malignant Tumour Epigenet, Guangzhou 510120, Peoples R China.

EM shuaixt@mail.sysu.edu.cn; shenjun@mail.sysu.edu.cn

RI Shuai, Xintao/C-5819-2008; Han, Shisong/P-7380-2018; Shen,

Jun/C-9471-2019

OI Han, Shisong/0000-0001-6769-5810; Duan, Xiaohui/0000-0002-2224-0887;

Shuai, Xintao/0000-0003-4271-0310; Shen, Jun/0000-0001-7746-5285

FU National Natural Science Foundation of China [81701764, U1801681,

81801756, 51933011, 31971296, 21805314]; Key Areas Research and

Development Program of Guangdong [2019B020235001]; National Basic

Research Program of China [2015CB755500]; Natural Science Foundation of

Guangdong Province [2017A030310221, 2014A030312018]; Guangdong Basic and

Applied Basic Research Foundation [2020A1515010523]; Guangdong

Innovative and Entrepreneurial Research Team Program [2013S086];

Guangdong Province Universities and Colleges Pearl River Scholar Funded

Scheme; Fundamental Research Funds for the Central Universities

[19ykpy107, 19lgjc05]

FX J.T., X.D., and F.Z. contributed equally to this work. This research was

supported by the National Natural Science Foundation of China (81701764,

U1801681, 81801756, 51933011, 31971296, and 21805314), the Key Areas

Research and Development Program of Guangdong (2019B020235001), the

National Basic Research Program of China (2015CB755500), the Natural

Science Foundation of Guangdong Province (2017A030310221 and

2014A030312018), the Guangdong Basic and Applied Basic Research

Foundation (2020A1515010523), the Guangdong Innovative and

Entrepreneurial Research Team Program (2013S086), the Guangdong Province

Universities and Colleges Pearl River Scholar Funded Scheme (2017), and

the Fundamental Research Funds for the Central Universities (19ykpy107

and 19lgjc05).

CR Arvanitis CD, 2020, NAT REV CANCER, V20, P26, DOI 10.1038/s41568-019-0205-x

Chang L, 2017, J DRUG TARGET, V25, P235, DOI 10.1080/1061186X.2016.1238091

Chen Q, 2016, ADV MATER, V28, P7129, DOI 10.1002/adma.201601902

Chen WC, 2014, SMALL, V10, P2678, DOI 10.1002/smll.201303951

Chen YL, 2016, CELL ONCOL, V39, P411, DOI 10.1007/s13402-016-0285-5

Choi JS, 2010, J AM CHEM SOC, V132, P11015, DOI 10.1021/ja104503g

Chu YC, 2017, INT J NANOMED, V12, P1353, DOI 10.2147/IJN.S126865

Danhier F, 2012, MOL PHARMACEUT, V9, P2961, DOI 10.1021/mp3002733

Fan WP, 2015, ADV MATER, V27, P4155, DOI 10.1002/adma.201405141

Fu CP, 2019, ADV HEALTHC MATER, V8, DOI 10.1002/adhm.201900047

Furtado D, 2018, ADV MATER, V30, DOI 10.1002/adma.201801362

Gao HL, 2017, J NEUROIMMUNE PHARM, V12, P6, DOI 10.1007/s11481-016-9687-4

Gao HL, 2016, ACTA PHARM SIN B, V6, P268, DOI 10.1016/j.apsb.2016.05.013

Ge X, 2018, CELL DEATH DIS, V9, DOI 10.1038/s41419-018-1176-7

Hatley RJD, 2018, ANGEW CHEM INT EDIT, V57, P3298, DOI 10.1002/anie.201707948

Hsu BYW, 2016, ADV HEALTHC MATER, V5, P721, DOI 10.1002/adhm.201500908

Jia Y, 2016, ADV MATER, V28, P1312, DOI 10.1002/adma.201502581

Kuang J, 2018, ADV FUNCT MATER, V28, DOI 10.1002/adfm.201800025

Kumar V, 2014, IMMUNOLOGY, V143, P512, DOI 10.1111/imm.12380

Lapointe S, 2018, LANCET, V392, P432, DOI 10.1016/S0140-6736(18)30990-5

Lee CY, 2017, ONCOTARGETS THER, V10, P265, DOI 10.2147/OTT.S120662

Li JJ, 2015, CHEM SOC REV, V44, P7855, DOI 10.1039/c4cs00476k

Lim M, 2018, NAT REV CLIN ONCOL, V15, P422, DOI 10.1038/s41571-018-0003-5

Lin LS, 2018, ANGEW CHEM INT EDIT, V57, P4902, DOI 10.1002/anie.201712027

Liu CP, 2017, SMALL, V13, DOI 10.1002/smll.201700278

Lu L, 2020, BIOMATERIALS, V230, DOI 10.1016/j.biomaterials.2019.119666

Monteiro AR, 2017, CELLS-BASEL, V6, DOI 10.3390/cells6040045

Pan DPJ, 2011, WIRES NANOMED NANOBI, V3, P162, DOI 10.1002/wnan.116

Patel A, 2016, BIOTECHNOL ADV, V34, P803, DOI 10.1016/j.biotechadv.2016.04.005

Prasad P, 2014, ACS NANO, V8, P3202, DOI 10.1021/nn405773r

Rankin EB, 2016, SCIENCE, V352, P175, DOI 10.1126/science.aaf4405

Robey RW, 2018, NAT REV CANCER, V18, P452, DOI 10.1038/s41568-018-0005-8

Sanai N, 2018, NAT REV CLIN ONCOL, V15, P112, DOI 10.1038/nrclinonc.2017.171

Shen ZY, 2018, ACS NANO, V12, P11355, DOI 10.1021/acsnano.8b06201

Shergalis A, 2018, PHARMACOL REV, V70, P412, DOI 10.1124/pr.117.014944

Stojanov D, 2016, NEURORADIOLOGY, V58, P433, DOI 10.1007/s00234-016-1658-1

Su C, 2019, ADV DRUG DELIVER REV, V143, P97, DOI 10.1016/j.addr.2019.06.008

Tang ZM, 2019, ANGEW CHEM INT EDIT, V58, P946, DOI 10.1002/anie.201805664

Wang NB, 2017, BIOMED PHARMACOTHER, V90, P368, DOI 10.1016/j.biopha.2017.03.083

Wilson WR, 2011, NAT REV CANCER, V11, P393, DOI 10.1038/nrc3064

Xiang Y, 2019, CHEM ENG J, V374, P392, DOI 10.1016/j.cej.2019.05.186

Xie YY, 2018, NANOMEDICINE-UK, V13, P887, DOI 10.2217/nnm-2017-0395

Yu M, 2019, ADV SCI, V6, DOI 10.1002/advs.201900037

Zhou LH, 2018, SMALL, V14, DOI 10.1002/smll.201801008

Zou MZ, 2018, SMALL, V14, DOI 10.1002/smll.201801120

NR 45

TC 88

Z9 88

U1 13

U2 204

PU WILEY

PI HOBOKEN

PA 111 RIVER ST, HOBOKEN 07030-5774, NJ USA

EI 2198-3844

J9 ADV SCI

JI Adv. Sci.

PD DEC

PY 2020

VL 7

IS 24

AR 2003036

DI 10.1002/advs.202003036

EA NOV 2020

PG 16

WC Chemistry, Multidisciplinary; Nanoscience & Nanotechnology; Materials

Science, Multidisciplinary

WE Science Citation Index Expanded (SCI-EXPANDED)

SC Chemistry; Science & Technology - Other Topics; Materials Science

GA PH0WZ

UT WOS:000588706300001

PM 33344142

OA Green Published, gold

DA 2025-04-09

ER

PT J

AU Zhao, XH

Liu, YH

Zhen, J

Liu, XB

Chen, JJ

Liu, LB

Wang, P

Xue, YX

AF Zhao, Xihe

Liu, Yunhui

Zhen, Jian

Liu, Xiaobai

Chen, Jiajia

Liu, Libo

Wang, Ping

Xue, Yixue

TI GAS5 suppresses malignancy of human glioma stem cells via a

miR-196a-5p/FOXO1 feedback loop

SO BIOCHIMICA ET BIOPHYSICA ACTA-MOLECULAR CELL RESEARCH

LA English

DT Article

DE lncRNA; GAS5; Glioma; Stem cell; miR-196a-5p

ID LONG NONCODING RNA; HUMAN GLIOBLASTOMA; TUMOR-SUPPRESSOR;

COLORECTAL-CANCER; OXIDATIVE STRESS; INHIBITORY GENE; POOR-PROGNOSIS;

EXPRESSION; GROWTH; MIIP

AB Glioma stem cells (GSCs) make up highly tumorigenic subpopulations within gliomas, and aberrant expression of GSC genes is a major underlying cause of glioma pathogenesis and treatment failure. The present study characterized the expression and function of long non-coding RNA growth arrest specific 5 (GAS5) in GSCs in order to elucidate the molecular mechanisms by which GAS5 contributes to glioma pathogenesis. We demonstrate that GAS5 suppresses GSC malignancy by binding to miR-196a-5p. miR-196a-5p, an onco-miRNA, stimulates GSC proliferation, migration, and invasion, in addition to reducing levels of apoptosis. miR-196a-5p specifically downregulates the expression of forkhead box protein O1 (FOXO1) by targeting its 3' untranslated region (3'-UTR). FOX)1 upregulates expression of phosphotyrosine interaction domain containing 1 (PID1), thereby inhibiting GSC tumorigenicity and growth. FOXOL also upregulates migration and invasion inhibitory protein (WHIP), resulting in attenuation of migration and invasion activities. Interestingly, we also show that FOXO1 promotes GAS5 transcription, thus forminga positive feedback loop. These data provide insights into potential new pathways for GSC molecular therapy and suggest that GAS5 may be an efficacious target for glioma treatments.

C1 [Zhao, Xihe; Chen, Jiajia; Liu, Libo; Wang, Ping; Xue, Yixue] China Med Univ, Coll Basic Med, Dept Neurobiol, Shenyang 110122, Liaoning, Peoples R China.

[Zhao, Xihe; Chen, Jiajia; Liu, Libo; Wang, Ping; Xue, Yixue] China Med Univ, Inst Pathol & Pathophysiol, Shenyang 110122, Liaoning, Peoples R China.

[Liu, Yunhui; Zhen, Jian; Liu, Xiaobai] China Med Univ, Shengjing Hosp, Dept Neurosurg, Shenyang 110004, Liaoning, Peoples R China.

[Liu, Yunhui; Zhen, Jian; Liu, Xiaobai] Liaoning Res Ctr Clin Med Nervous Syst Dis, Shenyang 110004, Liaoning, Peoples R China.

[Liu, Yunhui; Zhen, Jian; Liu, Xiaobai] Key Lab Neurooncol Liaoning Prov, Shenyang 110004, Liaoning, Peoples R China.

C3 China Medical University; China Medical University; China Medical

University

RP Xue, YX (corresponding author), China Med Univ, Coll Basic Med, Dept Neurobiol, Shenyang 110122, Liaoning, Peoples R China.

EM xueyixue888@163.com

OI Zhao, Xihe/0009-0005-2032-9166; Zhao, Xi-he/0000-0003-2458-6395

FU National Natural Science Foundation of China [81573010, 81672511];

Liaoning Science and Technology Plan Project [2015225007]; Shenyang

Science and Technology Plan Projects [F15-199-1-30, F15-199-1-57]

FX This work is supported by grants from the National Natural Science

Foundation of China (Nos. 81573010 and 81672511), Liaoning Science and

Technology Plan Project (No. 2015225007), and Shenyang Science and

Technology Plan Projects (Nos. F15-199-1-30 and F15-199-1-57).

CR ALONSO K, 1984, CANCER-AM CANCER SOC, V54, P2475, DOI 10.1002/1097-0142(19841201)54:11<2475::AID-CNCR2820541127>3.0.CO;2-Z

Bao SD, 2006, NATURE, V444, P756, DOI 10.1038/nature05236

Bonala S, 2013, MOL ENDOCRINOL, V27, P1518, DOI 10.1210/me.2013-1048

Cao SH, 2014, INT J CLIN EXP PATHO, V7, P6776

Chang L, 2016, MOL MED REP, V13, P1541, DOI 10.3892/mmr.2015.4716

Chen CY, 2011, J CELL MOL MED, V15, P14, DOI 10.1111/j.1582-4934.2010.01219.x

Chen J, 2014, TUMOR BIOL, V35, P7195, DOI 10.1007/s13277-014-1913-1

Cheng C., 2016, MOL MED REP

COCCIA EM, 1992, MOL CELL BIOL, V12, P3514, DOI 10.1128/MCB.12.8.3514

Di Gesualdo F, 2014, ONCOTARGET, V5, P10976, DOI 10.18632/oncotarget.2770

Diederichs S, 2010, PATHOLOGE, V31, P258, DOI 10.1007/s00292-010-1336-8

Erdreich-Epstein A, 2014, CLIN CANCER RES, V20, P827, DOI 10.1158/1078-0432.CCR-13-2053

Flemming A, 2015, NAT REV DRUG DISCOV, V14, P165, DOI 10.1038/nrd4560

Furukawa-Hibi Y, 2005, ANTIOXID REDOX SIGN, V7, P752, DOI 10.1089/ars.2005.7.752

Galasso M, 2014, GENOME MED, V6, DOI 10.1186/s13073-014-0076-4

Gallego-Perez D, 2016, NANO LETT, V16, P5326, DOI 10.1021/acs.nanolett.6b00902

Goodenberger ML, 2012, CANCER GENET-NY, V205, P613, DOI 10.1016/j.cancergen.2012.10.009

Gross DN, 2009, CURR DIABETES REP, V9, P208, DOI 10.1007/s11892-009-0034-5

Guan YL, 2015, INT J CLIN EXP PATHO, V8, P6576

Hezova R, 2012, WORLD J GASTROENTERO, V18, P2827, DOI 10.3748/wjg.v18.i22.2827

Ji P, 2010, ONCOGENE, V29, P3501, DOI 10.1038/onc.2010.114

Ke J, 2015, ONCOTARGET, V6, P21934, DOI 10.18632/oncotarget.4290

Koldemir O, 2017, BIOMED REP, V6, P358, DOI 10.3892/br.2017.848

Lau CJ, 2009, CANCER RES, V69, P5433, DOI 10.1158/0008-5472.CAN-08-4540

Laurent G, 2008, CELL METAB, V7, P113, DOI 10.1016/j.cmet.2007.12.010

Li J, 2016, EXP BIOL MED, V241, P644, DOI 10.1177/1535370215622708

Li L, 2015, PLOS ONE, V10, DOI 10.1371/journal.pone.0120550

Li W., 2016, ONCOTARGET, DOI 10.18632/oncotarget.8413

Liu J, 2014, BIOCHEM PHARMACOL, V89, P477, DOI 10.1016/j.bcp.2014.04.014

Lu YC, 2014, MOL CANCER, V13, DOI 10.1186/1476-4598-13-218

Ma J, 2014, CANCER LETT, V355, P85, DOI 10.1016/j.canlet.2014.09.012

Maass PG, 2014, J MOL MED, V92, P337, DOI 10.1007/s00109-014-1131-8

Mourtada-Maarabouni M, 2009, ONCOGENE, V28, P195, DOI 10.1038/onc.2008.373

Mueller DW, 2011, INT J CANCER, V129, P1064, DOI 10.1002/ijc.25768

Reis M, 2012, J EXP MED, V209, P1611, DOI 10.1084/jem.20111580

Sanai N, 2005, NEW ENGL J MED, V353, P811, DOI 10.1056/NEJMra043666

Song FF, 2010, CANCER RES, V70, P1024, DOI 10.1158/0008-5472.CAN-09-3742

Sun M, 2014, BMC CANCER, V14, DOI 10.1186/1471-2407-14-319

Tili E, 2013, IMMUNOL REV, V253, P167, DOI 10.1111/imr.12050

Tu ZQ, 2014, INT J CLIN EXP PATHO, V7, P4303

Vicente-Dueñas C, 2015, SEMIN CANCER BIOL, V32, P3, DOI 10.1016/j.semcancer.2014.02.001

Wen J, 2016, ONCOTARGET, V7, P9118, DOI 10.18632/oncotarget.7001

Wu Y, 2010, J BIOL CHEM, V285, P3554, DOI 10.1074/jbc.M109.063354

Yan W, 2014, ONCOTARGET, V5, P12908, DOI 10.18632/oncotarget.2679

Yao YL, 2015, MOL ONCOL, V9, P640, DOI 10.1016/j.molonc.2014.11.003

Yao YL, 2015, CANCER LETT, V359, P75, DOI 10.1016/j.canlet.2014.12.051

Yin DD, 2014, MED ONCOL, V31, DOI 10.1007/s12032-014-0253-8

Zhao XH, 2015, MOL THER, V23, P1899, DOI 10.1038/mt.2015.170

Zhao Y, 2010, NAT CELL BIOL, V12, P665, DOI 10.1038/ncb2069

Zheng J, 2016, MOL THER

Zheng J, 2015, ONCOTARGET, V6, P25339, DOI 10.18632/oncotarget.4509

Zhou BBS, 2009, NAT REV DRUG DISCOV, V8, P806, DOI 10.1038/nrd2137

NR 52

TC 78

Z9 82

U1 0

U2 17

PU ELSEVIER SCIENCE BV

PI AMSTERDAM

PA PO BOX 211, 1000 AE AMSTERDAM, NETHERLANDS

SN 0167-4889

EI 0006-3002

J9 BBA-MOL CELL RES

JI Biochim. Biophys. Acta-Mol. Cell Res.

PD OCT

PY 2017

VL 1864

IS 10

BP 1605

EP 1617

DI 10.1016/j.bbamcr.2017.06.020

PG 13

WC Biochemistry & Molecular Biology; Cell Biology

WE Science Citation Index Expanded (SCI-EXPANDED)

SC Biochemistry & Molecular Biology; Cell Biology

GA FH4YG

UT WOS:000411168100008

PM 28666797

DA 2025-04-09

ER

PT J

AU Kim, SS

Seong, S

Lim, SH

Kim, SY

AF Kim, Sung Su

Seong, Sin

Lim, Seong Hyeon

Kim, Sung Young

TI Biliverdin reductase plays a crucial role in hypoxia-induced

chemoresistance in human glioblastoma

SO BIOCHEMICAL AND BIOPHYSICAL RESEARCH COMMUNICATIONS

LA English

DT Article

DE Biliverdin reductase; Glioblastoma; Hypoxia; Chemoresistance;

Temozolomide; Paclitaxel; Reactive oxygen species

ID HEME OXYGENASE-1; CANCER-CELLS; ACTIVATION; ANGIOGENESIS; PROTECTION;

RESISTANCE; APOPTOSIS

AB Hypoxia-induced alterations in the cellular redox status play a critical role in the development of hypoxia-induced chemoresistance in cancer cells. Human biliverdin reductase (hBVR), an enzyme involved in the conversion of biliverdin into bilirubin in heme metabolism, was recently identified as an important cytoprotectant against oxidative stress and hypoxia. However, the role of hBVR on hypoxia-induced drug resistance has not been previously investigated. Using human glioblastoma cell lines, we evaluated the potential role of hBVR in hypoxia-induced drug resistance. We found that hypoxia caused a significant increase in hBVR expression in glioblastoma cells that was accompanied by chemoresistance. We also observed that siRNA-based targeting of hBVR genes attenuated the hypoxia-induced chemoresistance. Furthermore, knocking down hBVR induced a marked increase in the levels of intracellular reactive oxygen species under hypoxic conditions, and the chemosensitizing effect of hBVR depletion was reversed by pretreatment with the antioxidant N-acetylcysteine. These findings suggest that hBVR significantly contributes to the modulation of hypoxia-induced chemoresistance of glioblastoma cells by adjusting their cellular redox status. (C) 2013 Elsevier Inc. All rights reserved.

C1 [Kim, Sung Su; Seong, Sin] Kyung Hee Univ, Coll Oriental Med, Seoul 130701, South Korea.

[Lim, Seong Hyeon] Peking Univ, Hlth Sci Ctr, Beijing 100871, Peoples R China.

[Kim, Sung Young] Konkuk Univ, Sch Med, Dept Biochem, Seoul 143701, South Korea.

C3 Kyung Hee University; Peking University; Konkuk University; Konkuk

University Medical Center

RP Kim, SY (corresponding author), Konkuk Univ, Sch Med, Dept Biochem, Seoul 143701, South Korea.

EM palelamp@kku.ac.kr

RI Kim, Sung Young/GMW-7968-2022

OI Kim, Sung Young/0000-0003-4702-8265

CR Ahmad Z, 2002, J BIOL CHEM, V277, P9226, DOI 10.1074/jbc.M108239200

Barañano DE, 2002, P NATL ACAD SCI USA, V99, P16093, DOI 10.1073/pnas.252626999

DAUMASDUPORT C, 1988, CANCER-AM CANCER SOC, V62, P2152, DOI 10.1002/1097-0142(19881115)62:10<2152::AID-CNCR2820621015>3.0.CO;2-T

Florczyk U, 2011, CANCER LETT, V300, P40, DOI 10.1016/j.canlet.2010.09.003

Gozzelino R, 2010, ANNU REV PHARMACOL, V50, P323, DOI 10.1146/annurev.pharmtox.010909.105600

Haar CP, 2012, NEUROCHEM RES, V37, P1192, DOI 10.1007/s11064-011-0701-1

Kweon MH, 2006, J BIOL CHEM, V281, P33761, DOI 10.1074/jbc.M604748200

Lluis JM, 2007, CANCER RES, V67, P7368, DOI 10.1158/0008-5472.CAN-07-0515

Lu KH, 2005, J ORTHOP RES, V23, P988, DOI 10.1016/j.orthres.2005.01.018

Ma JJ, 2009, ARCH GYNECOL OBSTET, V279, P149, DOI 10.1007/s00404-008-0690-8

Miyake M, 2010, ANTICANCER RES, V30, P2145

Oliva CR, 2011, PLOS ONE, V6, DOI 10.1371/journal.pone.0024665

Rong Y, 2006, J NEUROPATH EXP NEUR, V65, P529, DOI 10.1097/00005072-200606000-00001

Sedlak TW, 2004, PEDIATRICS, V113, P1776, DOI 10.1542/peds.113.6.1776

Selvendiran K, 2009, INT J CANCER, V125, P2198, DOI 10.1002/ijc.24601

Vaupel P, 2007, CANCER METAST REV, V26, P225, DOI 10.1007/s10555-007-9055-1

Wouters A, 2007, ONCOLOGIST, V12, P690, DOI 10.1634/theoncologist.12-6-690

Xia C, 2007, CANCER RES, V67, P10823, DOI 10.1158/0008-5472.CAN-07-0783

NR 18

TC 19

Z9 21

U1 0

U2 4

PU ACADEMIC PRESS INC ELSEVIER SCIENCE

PI SAN DIEGO

PA 525 B ST, STE 1900, SAN DIEGO, CA 92101-4495 USA

SN 0006-291X

EI 1090-2104

J9 BIOCHEM BIOPH RES CO

JI Biochem. Biophys. Res. Commun.

PD NOV 1

PY 2013

VL 440

IS 4

BP 658

EP 663

DI 10.1016/j.bbrc.2013.09.120

PG 6

WC Biochemistry & Molecular Biology; Biophysics

WE Science Citation Index Expanded (SCI-EXPANDED)

SC Biochemistry & Molecular Biology; Biophysics

GA 251CQ

UT WOS:000326905800035

PM 24113378

DA 2025-04-09

ER

PT J

AU Fahey, JM

Korytowski, W

Girotti, AW

AF Fahey, Jonathan M.

Korytowski, Witold

Girotti, Albert W.

TI Upstream signaling events leading to elevated production of pro-survival

nitric oxide in photodynamically-challenged glioblastoma cells

SO FREE RADICAL BIOLOGY AND MEDICINE

LA English

DT Article

DE Photodynamic therapy; Glioblastoma; Oxidative stress; Nitric oxide;

Inducible nitric oxide synthase; Stress signaling

ID NF-KAPPA-B; MALIGNANT BRAIN-TUMORS; HISTONE ACETYLTRANSFERASE;

UP-REGULATION; CANCER-CELLS; THERAPY; SYNTHASE; AKT; TRANSCRIPTION;

PATHWAY

AB Nitric oxide (NO) generated endogenously by inducible nitric oxide synthase (iNOS) promotes growth and migration/invasion of glioblastoma cells and also fosters resistance to chemotherapy and ionizing radiotherapy. Our recent studies revealed that glioblastoma cell iNOS/NO also opposes the cytotoxic effects of non-ionizing photodynamic therapy (PDT), and moreover stimulates growth/migration aggressiveness of surviving cells. These negative responses, which depended on PI3K/Akt/NF-kappa B activation, were strongly suppressed by blocking iNOS transcription with JQ1, a BET bromodomain inhibitor. In the present study, we sought to identify additional molecular events that precede iNOS transcriptional upregulation. Akt activation, iNOS induction, and viability loss in PDT-challenged glioblastoma U87 cells were all strongly inhibited by added L-histidine, consistent with primary involvement of photogenerated singlet oxygen (O-1(2)). Transacetylase p300 not only underwent greater Akt-dependent activation after PDT, but greater interaction with NF-kappa B subunit p65, which in turn exhibited greater K310 acetylation. In addition, PDT promoted intramolecular disulfide formation and inactivation of tumor suppressor PTEN, thereby favoring Akt and p300 activation leading to iNOS upregulation. Importantly, deacetylase Sirt1 was down-regulated by PDT stress, consistent with the observed increase in p65-acK310 level, which fostered iNOS transcription. This study provides new mechanistic insights into how glioblastoma tumors can exploit iNOS/NO to not only resist PDT, but to attain a more aggressive survival phenotype.

C1 [Fahey, Jonathan M.; Girotti, Albert W.] Med Coll Wisconsin, Dept Biochem, Milwaukee, WI 53226 USA.

[Korytowski, Witold] Jagiellonian Univ, Dept Biophys, Krakow, Poland.

C3 Medical College of Wisconsin; Jagiellonian University

RP Girotti, AW (corresponding author), Med Coll Wisconsin, Dept Biochem, Milwaukee, WI 53226 USA.

EM agirotti@mcw.edu

RI Girotti, Albert/AAF-6910-2020

FU NIH/NCI from the Advancing a Healthier Wisconsin Research and Education

Program [CA70823, 5520347]; BSC from the MCW Cancer Center

[3308239-FP12605]; NCN [2017/27/B/NZ5/02620]

FX This work was supported by the following grants: NIH/NCI Grant CA70823,

Grant 5520347 from the Advancing a Healthier Wisconsin Research and

Education Program, and BSC Grant 3308239-FP12605 from the MCW Cancer

Center (to A.W.G.); and NCN grant 2017/27/B/NZ5/02620 (to W.K.). The

authors thank Dr. Brian C Smith for providing the human Sirt1 and Sirt2

antibodies along with a sample of recombinant human Sirt1. We also thank

Jerzy Bazak and Chuanwu Xia for their assistance in finalizing the

figures.

CR Agostinis P, 2011, CA-CANCER J CLIN, V61, P250, DOI 10.3322/caac.20114

Bazak J, 2017, FREE RADICAL BIO MED, V102, P111, DOI 10.1016/j.freeradbiomed.2016.11.034

Bechet D, 2014, CANCER TREAT REV, V40, P229, DOI 10.1016/j.ctrv.2012.07.004

Behin A, 2003, LANCET, V361, P323, DOI 10.1016/S0140-6736(03)12328-8

Benov L, 2015, MED PRIN PRACT, V24, P14, DOI 10.1159/000362416

Bhowmick R, 2014, CANCER LETT, V343, P115, DOI 10.1016/j.canlet.2013.09.025

Bhowmick R, 2013, FREE RADICAL BIO MED, V57, P39, DOI 10.1016/j.freeradbiomed.2012.12.005

Bhowmick R, 2011, PHOTOCHEM PHOTOBIOL, V87, P378, DOI 10.1111/j.1751-1097.2010.00877.x

Bhowmick R, 2010, FREE RADICAL BIO MED, V48, P1296, DOI 10.1016/j.freeradbiomed.2010.01.040

Bhowmick R, 2009, FREE RADICAL BIO MED, V47, P731, DOI 10.1016/j.freeradbiomed.2009.06.009

Brazil DP, 2001, TRENDS BIOCHEM SCI, V26, P657, DOI 10.1016/S0968-0004(01)01958-2

Burke AJ, 2013, CARCINOGENESIS, V34, P503, DOI 10.1093/carcin/bgt034

Cantley LC, 1999, P NATL ACAD SCI USA, V96, P4240, DOI 10.1073/pnas.96.8.4240

Chen LF, 2002, EMBO J, V21, P6539, DOI 10.1093/emboj/cdf660

Crowell JA, 2003, MOL CANCER THER, V2, P815

Dougherty TJ, 1998, JNCI-J NATL CANCER I, V90, P889, DOI 10.1093/jnci/90.12.889

Ekmekcioglu S, 2017, HUM VACC IMMUNOTHER, V13, P1105, DOI 10.1080/21645515.2016.1276682

Fahey JM, 2018, J BIOL CHEM, V293, P5345, DOI 10.1074/jbc.RA117.000443

Fahey JM, 2017, NITRIC OXIDE-BIOL CH, V62, P52, DOI 10.1016/j.niox.2016.12.003

Fahey JM, 2016, PHOTOCHEM PHOTOBIOL, V92, P842, DOI 10.1111/php.12636

Fahey JM, 2015, NITRIC OXIDE-BIOL CH, V49, P47, DOI 10.1016/j.niox.2015.05.006

Fionda C, 2016, CURR MED CHEM, V23, P2618, DOI 10.2174/0929867323666160727105101

FOOTE CS, 1968, SCIENCE, V162, P963, DOI 10.1126/science.162.3857.963

FOOTE CS, 1991, PHOTOCHEM PHOTOBIOL, V54, P659, DOI 10.1111/j.1751-1097.1991.tb02071.x

Fukumura D, 1998, CANCER METAST REV, V17, P77, DOI 10.1023/A:1005908805527

Girotti AW, 2008, FREE RADICAL BIO MED, V44, P956, DOI 10.1016/j.freeradbiomed.2007.12.004

Girotti AW, 2015, PHOTOCH PHOTOBIO SCI, V14, P1425, DOI 10.1039/c4pp00470a

Goodman RH, 2000, GENE DEV, V14, P1553

Hawkins PT, 2006, BIOCHEM SOC T, V34, P647, DOI 10.1042/BST0340647

Henderson BW, 1999, PHOTOCHEM PHOTOBIOL, V70, P64, DOI 10.1562/0031-8655(1999)070<0064:POPTAA>2.3.CO;2

Hogg N., 2017, ANTIOXIDANTS REDOX S, V17, P969

Huang B, 2009, MOL CELL BIOL, V29, P1375, DOI 10.1128/MCB.01365-08

Huang WC, 2005, MOL CELL BIOL, V25, P6592, DOI 10.1128/MCB.25.15.6592-6602.2005

JENKINS DC, 1995, P NATL ACAD SCI USA, V92, P4392, DOI 10.1073/pnas.92.10.4392

Kauppinen A, 2013, CELL SIGNAL, V25, P1939, DOI 10.1016/j.cellsig.2013.06.007

KENNEDY JC, 1992, J PHOTOCH PHOTOBIO B, V14, P275, DOI 10.1016/1011-1344(92)85108-7

Kleszcz R, 2015, PHARMACOL REP, V67, P1068, DOI 10.1016/j.pharep.2015.03.021

Korbelik M, 2000, BRIT J CANCER, V82, P1835, DOI 10.1054/bjoc.2000.1157

Kostourou V, 2011, BRIT J CANCER, V104, P83, DOI 10.1038/sj.bjc.6606034

Lee SR, 2002, J BIOL CHEM, V277, P20336, DOI 10.1074/jbc.M111899200

Leslie NR, 2003, EMBO J, V22, P5501, DOI 10.1093/emboj/cdg513

Lin Zhenghong, 2013, Genes Cancer, V4, P97, DOI 10.1177/1947601912475079

Myers MP, 1998, P NATL ACAD SCI USA, V95, P13513, DOI 10.1073/pnas.95.23.13513

Olzowy B, 2002, J NEUROSURG, V97, P970, DOI 10.3171/jns.2002.97.4.0970

Palumbo P, 2018, INT J MOL SCI, V19, DOI 10.3390/ijms19092801

Peng Q, 1997, PHOTOCHEM PHOTOBIOL, V65, P235, DOI 10.1111/j.1751-1097.1997.tb08549.x

Quirk BJ, 2015, PHOTODIAGN PHOTODYN, V12, P530, DOI 10.1016/j.pdpdt.2015.04.009

Ribas V, 2016, CLIN TRANSL MED, V5, DOI 10.1186/s40169-016-0106-5

Sengupta R, 2007, BIOCHEMISTRY-US, V46, P8472, DOI 10.1021/bi700449x

Shikama N, 1997, TRENDS CELL BIOL, V7, P230, DOI 10.1016/S0962-8924(97)01048-9

Spikes JD., 1989, The Science of Photobiology, P79, DOI DOI 10.1007/978-1-4615-8061-43

Switzer CH, 2011, TRENDS PHARMACOL SCI, V32, P644, DOI 10.1016/j.tips.2011.07.001

Tetard MC, 2014, PHOTODIAGN PHOTODYN, V11, P319, DOI 10.1016/j.pdpdt.2014.04.004

Thomas DD, 2008, FREE RADICAL BIO MED, V45, P18, DOI 10.1016/j.freeradbiomed.2008.03.020

Thomas DD, 2012, ANTIOXID REDOX SIGN, V17, P934, DOI 10.1089/ars.2012.4669

Toker Alex, 2014, Advances in Biological Regulation, V55, P28, DOI 10.1016/j.jbior.2014.04.001

Tran AN, 2017, ANTIOXID REDOX SIGN, V26, P986, DOI 10.1089/ars.2016.6820

Tsai YJ, 2015, FREE RADICAL BIO MED, V86, P118, DOI 10.1016/j.freeradbiomed.2015.05.009

Vannini F, 2015, REDOX BIOL, V6, P334, DOI 10.1016/j.redox.2015.08.009

Vivanco I, 2002, NAT REV CANCER, V2, P489, DOI 10.1038/nrc839

Wang F, 2013, CELL MOL LIFE SCI, V70, P3989, DOI 10.1007/s00018-012-1254-4

Wen PY, 2008, NEW ENGL J MED, V359, P492, DOI 10.1056/NEJMra0708126

Whelan HT, 2012, J NATL COMPR CANC NE, V10, pS31, DOI 10.6004/jnccn.2012.0171

WILKINSON F, 1995, J PHYS CHEM REF DATA, V24, P663, DOI 10.1063/1.555965

XIE QW, 1994, J BIOL CHEM, V269, P4705

Xue LY, 2001, ONCOGENE, V20, P3420, DOI 10.1038/sj.onc.1204441

Yeung F, 2004, EMBO J, V23, P2369, DOI 10.1038/sj.emboj.7600244

Yuan TL, 2008, ONCOGENE, V27, P5497, DOI 10.1038/onc.2008.245

Zhang Y, 2017, INT J MOL SCI, V18, DOI 10.3390/ijms18050982

Zhuang T, 2013, BMC CANCER, V13, DOI 10.1186/1471-2407-13-590

Zou Z, 2014, ONCOGENE, V33, P2395, DOI 10.1038/onc.2013.179

NR 71

TC 27

Z9 27

U1 0

U2 13

PU ELSEVIER SCIENCE INC

PI NEW YORK

PA STE 800, 230 PARK AVE, NEW YORK, NY 10169 USA

SN 0891-5849

EI 1873-4596

J9 FREE RADICAL BIO MED

JI Free Radic. Biol. Med.

PD JUN

PY 2019

VL 137

BP 37

EP 45

DI 10.1016/j.freeradbiomed.2019.04.013

PG 9

WC Biochemistry & Molecular Biology; Endocrinology & Metabolism

WE Science Citation Index Expanded (SCI-EXPANDED)

SC Biochemistry & Molecular Biology; Endocrinology & Metabolism

GA HY1WT

UT WOS:000467908800004

PM 30991141

OA Green Accepted

DA 2025-04-09

ER

PT J

AU Rupprecht, A

Theisen, U

Wendt, F

Frank, M

Hinz, B

AF Rupprecht, Anne

Theisen, Ulrike

Wendt, Franziska

Frank, Marcus

Hinz, Burkhard

TI The Combination of Δ<SUP>9</SUP>-Tetrahydrocannabinol and Cannabidiol

Suppresses Mitochondrial Respiration of Human Glioblastoma Cells via

Downregulation of Specific Respiratory Chain Proteins

SO CANCERS

LA English

DT Article

DE cannabinoids; Delta(9)-tetrahydrocannabinol; cannabidiol; glioblastoma

cells; mitochondria; oxygen consumption rate; electron transport chain

complex proteins

ID OXIDATIVE STRESS; TISSUE INHIBITOR; COMPLEX I; CANNABINOIDS; ACTIVATION;

AUTOPHAGY; DEATH; PROLIFERATION; TEMOZOLOMIDE; DEGRADATION

AB Phytocannabinoids represent a promising approach in glioblastoma therapy. Previous work has shown that a combined treatment of glioblastoma cells with submaximal effective concentrations of psychoactive Delta(9)-tetrahydrocannabinol (THC) and non-psychoactive cannabidiol (CBD) greatly increases cell death. In the present work, the glioblastoma cell lines U251MG and U138MG were used to investigate whether the combination of THC and CBD in a 1:1 ratio is associated with a disruption of cellular energy metabolism, and whether this is caused by affecting mitochondrial respiration. Here, the combined administration of THC and CBD (2.5 mu M each) led to an inhibition of oxygen consumption rate and energy metabolism. These effects were accompanied by morphological changes to the mitochondria, a release of mitochondrial cytochrome c into the cytosol and a marked reduction in subunits of electron transport chain complexes I (NDUFA9, NDUFB8) and IV (COX2, COX4). Experiments with receptor antagonists and inhibitors showed that the degradation of NDUFA9 occurred independently of the activation of the cannabinoid receptors CB1, CB2 and TRPV1 and of usual degradation processes mediated via autophagy or the proteasomal system. In summary, the results describe a previously unknown mitochondria-targeting mechanism behind the toxic effect of THC and CBD on glioblastoma cells that should be considered in future cancer therapy, especially in combination strategies with other chemotherapeutics.

C1 [Rupprecht, Anne; Theisen, Ulrike; Wendt, Franziska; Hinz, Burkhard] Rostock Univ Med Ctr, Inst Pharmacol & Toxicol, D-18057 Rostock, Germany.

[Frank, Marcus] Rostock Univ Med Ctr, Electron Microscopy Ctr, D-18057 Rostock, Germany.

[Frank, Marcus] Univ Rostock, Dept Life Light & Matter, D-18059 Rostock, Germany.

C3 University of Rostock

RP Hinz, B (corresponding author), Rostock Univ Med Ctr, Inst Pharmacol & Toxicol, D-18057 Rostock, Germany.

EM rupprechta31@gmail.com; ulrike.theisen@med.uni-rostock.de;

franziska.wendt@med.uni-rostock.de; marcus.frank@med.uni-rostock.de;

burkhard.hinz@med.uni-rostock.de

RI Wendt, Frank/AAQ-3456-2021

FU FORUN programme (Rostock University Medical Centre)

FX This work was supported by the FORUN programme (Rostock University

Medical Centre).

CR Abate M, 2020, SEMIN CELL DEV BIOL, V98, P139, DOI 10.1016/j.semcdb.2019.05.022

Alharris Esraah, 2019, Oncotarget, V10, P45, DOI 10.18632/oncotarget.26534

Armstrong JL, 2015, J INVEST DERMATOL, V135, P1629, DOI 10.1038/jid.2015.45

Bansal S, 2014, REDOX BIOL, V2, P273, DOI 10.1016/j.redox.2013.07.004

Barth S, 2010, J PATHOL, V221, P117, DOI 10.1002/path.2694

Batara DCR, 2021, CELLS-BASEL, V10, DOI 10.3390/cells10061411

Bénard G, 2012, NAT NEUROSCI, V15, P558, DOI 10.1038/nn.3053

Bezawork-Geleta A, 2015, SCI REP-UK, V5, DOI 10.1038/srep17397

Böckmann S, 2020, CELLS-BASEL, V9, DOI 10.3390/cells9071703

Bogenhagen DF, 2020, J BIOL CHEM, V295, P2544, DOI 10.1074/jbc.RA119.011791

Bolisetty S, 2013, AM J PHYSIOL-RENAL, V305, pF255, DOI 10.1152/ajprenal.00160.2013

Bonekamp NA, 2021, LIFE SCI ALLIANCE, V4, DOI 10.26508/lsa.202101034

Bonvini P, 2007, LEUKEMIA, V21, P838, DOI 10.1038/sj.leu.2404528

Bruns I, 2019, J BIOL CHEM, V294, P3037, DOI 10.1074/jbc.RA118.006993

Chan JZ, 2021, CELLS-BASEL, V10, DOI 10.3390/cells10051251

Chen YQ, 2007, J CELL SCI, V120, P4155, DOI 10.1242/jcs.011163

Chiang SK, 2019, INT J MOL SCI, V20, DOI 10.3390/ijms20010039

Fisar Z, 2014, TOXICOL LETT, V231, P62, DOI 10.1016/j.toxlet.2014.09.002

Furukawa R, 2012, FEBS OPEN BIO, V2, P145, DOI 10.1016/j.fob.2012.06.001

Galve-Roperh I, 2000, NAT MED, V6, P313, DOI 10.1038/73171

Gross C, 2021, FRONT PHARMACOL, V12, DOI 10.3389/fphar.2021.725136

Hebert-Chatelain E, 2014, MOL METAB, V3, P495, DOI 10.1016/j.molmet.2014.03.007

Hinz B, 2022, BRIT J CANCER, V127, P1, DOI 10.1038/s41416-022-01727-4

Huang TF, 2021, AUTOPHAGY, V17, P3592, DOI 10.1080/15548627.2021.1885203

Hull TD, 2016, JCI INSIGHT, V1, DOI 10.1172/jci.insight.85817

Jacobsson SOP, 2001, J PHARMACOL EXP THER, V299, P951

Jeong S, 2019, CELL DEATH DIS, V10, DOI 10.1038/s41419-019-2001-7

Jeong S, 2019, CANCERS, V11, DOI 10.3390/cancers11060781

Kabeya Y, 2000, EMBO J, V19, P5720, DOI 10.1093/emboj/19.21.5720

Kim SR, 2006, J IMMUNOL, V177, P4322, DOI 10.4049/jimmunol.177.7.4322

Kimura S, 2009, METHOD ENZYMOL, V452, P1, DOI 10.1016/S0076-6879(08)03601-X

Kosgodage US, 2018, FRONT PHARMACOL, V9, DOI 10.3389/fphar.2018.00889

Krämer L, 2021, FEBS LETT, V595, P1223, DOI 10.1002/1873-3468.14010

Krzysik-Walker SM, 2013, MOL PHARMACOL, V83, P157, DOI 10.1124/mol.112.082651

Lavie J, 2018, CELL REP, V23, P2852, DOI 10.1016/j.celrep.2018.05.013

Lebeau J, 2018, INT REV CEL MOL BIO, V340, P79, DOI 10.1016/bs.ircmb.2018.05.003

Lee SY, 2016, GENES DIS, V3, P198, DOI 10.1016/j.gendis.2016.04.007

Lin CJ, 2012, PLOS ONE, V7, DOI 10.1371/journal.pone.0038706

López-Valero I, 2018, BIOCHEM PHARMACOL, V157, P266, DOI 10.1016/j.bcp.2018.09.007

López-Valero I, 2018, BIOCHEM PHARMACOL, V157, P275, DOI 10.1016/j.bcp.2018.08.023

Marcu JP, 2010, MOL CANCER THER, V9, P180, DOI 10.1158/1535-7163.MCT-09-0407

Nabissi M, 2016, ONCOTARGET, V7, P77543, DOI 10.18632/oncotarget.12721

Navarro E, 2017, ANTIOXID REDOX SIGN, V27, P93, DOI 10.1089/ars.2016.6698

Olivas-Aguirre M, 2019, CELL DEATH DIS, V10, DOI 10.1038/s41419-019-2024-0

Piantadosi CA, 2008, CIRC RES, V103, P1232, DOI 10.1161/01.RES.0000338597.71702.ad

Prüser JL, 2021, MOL CANCER THER, V20, P787, DOI 10.1158/1535-7163.MCT-20-0589

Pryde KR, 2016, CELL REP, V17, P2522, DOI 10.1016/j.celrep.2016.11.027

Ramer R, 2008, J NATL CANCER I, V100, P59, DOI 10.1093/jnci/djm268

Ramer R, 2021, CANCERS, V13, DOI 10.3390/cancers13225701

Ramer R, 2014, BIOCHEM PHARMACOL, V91, P202, DOI 10.1016/j.bcp.2014.06.017

Rayamajhi N, 2013, OXID MED CELL LONGEV, V2013, DOI 10.1155/2013/154279

Rimmerman N, 2013, CELL DEATH DIS, V4, DOI 10.1038/cddis.2013.471

Salazar M, 2009, J CLIN INVEST, V119, P1359, DOI 10.1172/JCI37948

Sánchez C, 2001, CANCER RES, V61, P5784

Scott KA, 2015, ANTICANCER RES, V35, P5827

Scott KA, 2014, MOL CANCER THER, V13, P2955, DOI 10.1158/1535-7163.MCT-14-0402

Shangguan FG, 2021, FRONT CELL DEV BIOL, V9, DOI 10.3389/fcell.2021.697832

Shin CS, 2021, NAT COMMUN, V12, DOI 10.1038/s41467-020-20597-z

Stroud DA, 2016, NATURE, V538, P123, DOI 10.1038/nature19754

Stroud DA, 2013, J BIOL CHEM, V288, P1685, DOI 10.1074/jbc.C112.436766

Suliman HB, 2017, JCI INSIGHT, V2, DOI 10.1172/jci.insight.89676

Szczepanowska K, 2021, BBA-BIOENERGETICS, V1862, DOI 10.1016/j.bbabio.2020.148365

Tan AC, 2020, CA-CANCER J CLIN, V70, P299, DOI 10.3322/caac.21613

Tang JX, 2020, INT J MOL SCI, V21, DOI 10.3390/ijms21113820

Thomas HE, 2018, CELL REP, V24, P2404, DOI 10.1016/j.celrep.2018.07.101

Timón-Gómez A, 2018, SEMIN CELL DEV BIOL, V76, P163, DOI 10.1016/j.semcdb.2017.08.055

Torres S, 2011, MOL CANCER THER, V10, P90, DOI 10.1158/1535-7163.MCT-10-0688

Twelves C, 2021, BRIT J CANCER, V124, P1379, DOI 10.1038/s41416-021-01259-3

Wu HY, 2018, FREE RADICAL BIO MED, V124, P311, DOI 10.1016/j.freeradbiomed.2018.06.023

Wu ZH, 2022, NEUROMOL MED, V24, P18, DOI 10.1007/s12017-021-08678-8

Yamamoto A, 1998, CELL STRUCT FUNCT, V23, P33, DOI 10.1247/csf.23.33

NR 71

TC 19

Z9 19

U1 0

U2 4

PU MDPI

PI BASEL

PA ST ALBAN-ANLAGE 66, CH-4052 BASEL, SWITZERLAND

EI 2072-6694

J9 CANCERS

JI Cancers

PD JUL

PY 2022

VL 14

IS 13

AR 3129

DI 10.3390/cancers14133129

PG 22

WC Oncology

WE Science Citation Index Expanded (SCI-EXPANDED)

SC Oncology

GA 2Y2GV

UT WOS:000825709300001

PM 35804909

OA gold, Green Published

DA 2025-04-09

ER

PT J

AU Peng, F

Wang, XX

Shu, MT

Yang, MF

Wang, LG

Ouyang, ZR

Shen, C

Hou, X

Zhao, BY

Wang, XZ

Zhang, LW

Liu, YH

Zhao, SG

AF Peng, Eel

Wang, Xiaoxiong

Shu, Mengting

Yang, Mingfei

Wang, Ligang

Ouyang, Zhongrui

Shen, Chen

Hou, Xu

Zhao, Boyan

Wang, Xinzhuang

Zhang, Linda Wei

Liu, Yaohua

Zhao, Shiguang

TI Raddeanin a Suppresses Glioblastoma Growth by Inducing ROS Generation

and Subsequent JNK Activation to Promote Cell Apoptosis

SO CELLULAR PHYSIOLOGY AND BIOCHEMISTRY

LA English

DT Article

DE Raddeanin A; Glioblastoma; Apoptosis; ROS/JNK signaling pathway;

Autophagy

ID REACTIVE OXYGEN; MATRIX METALLOPROTEINASES; SIGNAL-TRANSDUCTION;

OXIDATIVE STRESS; GLIOMA-CELLS; CANCER-CELLS; AUTOPHAGY; DEATH; PATHWAY;

KINASE

AB Background/Aims: Raddeanin A (RA), an active pharmacological ingredient from Anemone raddeana Regel, plays an important role in tumor suppression. In this study, we assessed the potentially therapeutic effect of RA on glioblastoma and its underlying mechanisms. Methods: Cell viability was examined using the MTT assay. Invasive and migratory capacities were examined using Transwell and wound healing assays. Apoptosis was determined by Hoechst staining, flow cytometry, DCFH-fluorescent probe and immunohistochemical staining. Autophagy was detected by transmission electron microscopy and western blotting. A U251 glioma xenograft model was established to evaluate the effect of RA in vivo. Results: The data demonstrated that RA inhibited viability, and abrogated the invasive/migratory abilities of glioblastoma cells. In addition, RA induced apoptosis by reactive oxygen species (ROS)/Jun N-terminal kinase (JNK) signaling in glioblastoma. Conversely, the antioxidant N-Acetyl-L-cysteine (NAC) and pan-caspase inhibitor z-VAD-fmk attenuated RA-induced apoptosis by scavenging ROS and inactivating caspase-3. Furthermore, the inhibition of autophagy by 3-MA exacerbated apoptosis through ROS generation and JNK phosphorylation. In vivo, RA exhibited a curative effect on U251-derived xenografts in nude mice. Conclusions: The results of this study suggest that RA suppressed the growth of glioblastoma, thus serving as a promising and potential strategy for glioblastoma chemotherapy. (C) 2018 The Author(s) Published by S. Karger AG, Basel

C1 [Peng, Eel; Wang, Xiaoxiong; Shu, Mengting; Yang, Mingfei; Wang, Ligang; Ouyang, Zhongrui; Shen, Chen; Hou, Xu; Zhao, Boyan; Wang, Xinzhuang; Liu, Yaohua; Zhao, Shiguang] Harbin Med Univ, Affiliated Hosp 1, Dept Neurosurg, Youzheng St 23, Harbin, Heilongjiang, Peoples R China.

[Peng, Eel; Wang, Xiaoxiong; Shu, Mengting; Yang, Mingfei; Wang, Ligang; Ouyang, Zhongrui; Shen, Chen; Hou, Xu; Zhao, Boyan; Wang, Xinzhuang; Liu, Yaohua; Zhao, Shiguang] Harbin Med Univ, Inst Brain Sci, Harbin, Heilongjiang, Peoples R China.

[Zhang, Linda Wei] Fudan Univ, Shanghai Med Coll, Shanghai, Peoples R China.

[Liu, Yaohua] Shanghai Jiao Tong Univ, Shanghai Peoples Hosp 1, Sch Med, Dept Neurosurg, Shanghai, Peoples R China.

C3 Harbin Medical University; Harbin Medical University; Fudan University;

Shanghai Jiao Tong University

RP Liu, YH; Zhao, SG (corresponding author), Harbin Med Univ, Affiliated Hosp 1, Dept Neurosurg, Youzheng St 23, Harbin, Heilongjiang, Peoples R China.

EM liuyaohua_harbin@hotmail.com; guangsz@hotmail.com

RI Zhao, Shiguang/KBQ-5809-2024; Yang, mingfei/LWI-0908-2024; Hou,

Xu/AAY-4921-2021

OI Wang, Xiaoxiong/0000-0002-8949-8970

FU National Natural Science Foundation of China [81272788, 81472368,

81372701]; First Affiliated Hospital of Harbin Medical University

Foundation [2016B006]

FX This study was primarily supported by the National Natural Science

Foundation of China (Grant No. 81272788, 81472368, 81372701) and the

First Affiliated Hospital of Harbin Medical University Foundation (Grant

No. 2016B006).

CR Baik JS, 2016, J MICROBIOL BIOTECHN, V26, P309, DOI 10.4014/jmb.1507.07090

Davies C, 2012, BIOCHEM SOC T, V40, P85, DOI 10.1042/BST20110641

Davis RJ, 2000, CELL, V103, P239, DOI 10.1016/S0092-8674(00)00116-1

de Bruin EC, 2008, CANCER TREAT REV, V34, P737, DOI 10.1016/j.ctrv.2008.07.001

Denton D, 2012, CELL DEATH DIFFER, V19, P87, DOI 10.1038/cdd.2011.146

Dolado I, 2007, CANCER CELL, V11, P191, DOI 10.1016/j.ccr.2006.12.013

Egeblad M, 2002, NAT REV CANCER, V2, P161, DOI 10.1038/nrc745

Eisenberg-Lerner A, 2009, CELL DEATH DIFFER, V16, P966, DOI 10.1038/cdd.2009.33

Fleury C, 2002, BIOCHIMIE, V84, P131, DOI 10.1016/S0300-9084(02)01369-X

Fox JL, 2016, BRIT J CANCER, V115, P5, DOI 10.1038/bjc.2016.111

Ghobrial IM, 2005, CA-CANCER J CLIN, V55, P178, DOI 10.3322/canjclin.55.3.178

Gilbert MR, 2014, NEW ENGL J MED, V370, P699, DOI 10.1056/NEJMoa1308573

Guan YY, 2015, PHYTOMEDICINE, V22, P103, DOI 10.1016/j.phymed.2014.11.008

Hanahan D, 2000, CELL, V100, P57, DOI 10.1016/S0092-8674(00)81683-9

He CC, 2009, ANNU REV GENET, V43, P67, DOI 10.1146/annurev-genet-102808-114910

Herman-Antosiewicz A, 2006, CANCER RES, V66, P5828, DOI 10.1158/0008-5472.CAN-06-0139

Hu PF, 2015, CELL PHYSIOL BIOCHEM, V37, P697, DOI 10.1159/000430388

Jelassi B, 2013, CARCINOGENESIS, V34, P1487, DOI 10.1093/carcin/bgt099

Kaza N, 2012, BRAIN PATHOL, V22, P89, DOI 10.1111/j.1750-3639.2011.00544.x

LAZEBNIK YA, 1994, NATURE, V371, P346, DOI 10.1038/371346a0

Lei YY, 2014, ASIAN PAC J CANCER P, V15, P8539, DOI 10.7314/APJCP.2014.15.20.8539

Li CG, 2015, CELL PHYSIOL BIOCHEM, V35, P1303, DOI 10.1159/000373952

Li JN, 2017, BIOCHEM BIOPH RES CO, V485, P335, DOI 10.1016/j.bbrc.2017.02.079

Liu XR, 2015, EUR REV MED PHARMACO, V19, P4068

Louis DN, 2007, ACTA NEUROPATHOL, V114, P547, DOI 10.1007/s00401-007-0278-6

Luan X, 2013, J CHROMATOGR B, V923, P43, DOI 10.1016/j.jchromb.2013.01.019

Maiuri MC, 2007, NAT REV MOL CELL BIO, V8, P741, DOI 10.1038/nrm2239

Mizushima N, 2010, NAT CELL BIOL, V12, P823, DOI 10.1038/ncb0910-823

Mora R, 2009, AUTOPHAGY, V5, P419, DOI 10.4161/auto.5.3.7881

Nakada M, 2003, FRONT BIOSCI, V8, pE261, DOI 10.2741/1016

Nikoletopoulou V, 2013, BBA-MOL CELL RES, V1833, P3448, DOI 10.1016/j.bbamcr.2013.06.001

Paiva C, 2015, PLOS ONE, V10, DOI 10.1371/journal.pone.0143685

Pankiv S, 2007, J BIOL CHEM, V282, P24131, DOI 10.1074/jbc.M702824200

Patel AP, 2014, SCIENCE, V344, P1396, DOI 10.1126/science.1254257

Pelicano H, 2004, DRUG RESIST UPDATE, V7, P97, DOI 10.1016/j.drup.2004.01.004

Ray PD, 2012, CELL SIGNAL, V24, P981, DOI 10.1016/j.cellsig.2012.01.008

Reed John C., 1995, Current Opinion in Oncology, V7, P541, DOI 10.1097/00001622-199511000-00012

Reuter S, 2010, FREE RADICAL BIO MED, V49, P1603, DOI 10.1016/j.freeradbiomed.2010.09.006

Rodríguez CE, 2015, PLOS ONE, V10, DOI 10.1371/journal.pone.0137920

Shen HM, 2006, FREE RADICAL BIO MED, V40, P928, DOI 10.1016/j.freeradbiomed.2005.10.056

Song B, 2015, PLOS ONE, V10, DOI 10.1371/journal.pone.0120694

Stupp R, 2015, JAMA-J AM MED ASSOC, V314, P2535, DOI 10.1001/jama.2015.16669

Teng YH, 2016, EVID-BASED COMPL ALT, V2016, DOI 10.1155/2016/9406758
[truncated: 4,346,263 more chars]
