# Supplementary material for: Recent status and trends regarding oxidative stress in gliomas (2013 - 2025): a systematic review and bibliometric analysis
Source: Front Oncol. 2025 May 16;15:1586515. doi: 10.3389/fonc.2025.1586515 (PMC12122519; doi:10.3389/fonc.2025.1586515)
Supplement: Supplementary file 4 [file Table4.docx]

FN Clarivate Analytics Web of Science

VR 1.0

PT J

AU Guerriero, C

Manfredelli, M

Matera, C

Iuzzolino, A

Conti, L

Dallanoce, C

De Amici, M

Trisciuoglio, D

Tata, AM

AF Guerriero, Claudia

Manfredelli, Marianna

Matera, Carlo

Iuzzolino, Angela

Conti, Luciano

Dallanoce, Clelia

De Amici, Marco

Trisciuoglio, Daniela

Tata, Ada Maria

TI M2 Muscarinic Receptor Stimulation Induces Autophagy in Human

Glioblastoma Cancer Stem Cells via mTOR Complex-1 Inhibition

SO CANCERS

LA English

DT Article

DE glioblastoma; cancer stem cells; M2 muscarinic receptor; orthosteric and

dualsteric muscarinic agonism; autophagy; apoptosis; mTORC1

ID ACETYLCHOLINE-RECEPTORS; PHOSPHORYLATION; SURVIVAL; GROWTH; AMPK;

ACTIVATION; MECHANISM; PATHOLOGY

AB Simple Summary Tumor cells use autophagy as a pro-survival strategy. However, several studies have shown that excessive stimulation of the autophagic process can promote cell death; in this context, autophagy acquires an antitumor effect. To further explore the consequences of the cytotoxic effects induced by M2 muscarinic receptor activation that we have previously described both in glioblastoma multiforme (GBM) stable cell lines and in human GBM cancer stem cells, here we investigated the involvement of autophagy and apoptosis in the cell death engendered by treatment with M2 muscarinic agonists. Moreover, we compared the effects mediated by orthosteric and dualsteric M2 muscarinic agonists in the modulation of these different mechanisms of cell death.Abstract Background: Although autophagy is a pro-survival process of tumor cells, it can stimulate cell death in particular conditions and when differently regulated by specific signals. We previously demonstrated that the selective stimulation of the M2 muscarinic receptor subtype (mAChR) negatively controls cell proliferation and survival and causes oxidative stress and cytotoxic and genotoxic effects in both GBM cell lines and GBM stem cells (GSCs). In this work, we have evaluated whether autophagy was induced as a downstream mechanism of the observed cytotoxic processes induced by M2 mAChR activation by the orthosteric agonist APE or the dualsteric agonist N8-Iper (N8). Methods: To assess the activation of autophagy, we analyzed the expression of LC3B using Western blot analysis and in LC3B-EGFP transfected cell lines. Apoptosis was assessed by measuring the protein expression of Caspases 3 and 9. Results: Our data indicate that activation of M2 mAChR by N8 promotes autophagy in both U251 and GB7 cell lines as suggested by the LC3B-II expression level and analysis of the transfected cells by fluorescence microscopy. Autophagy induction by M2 mAChRs is regulated by the decreased activity of the PI3K/AKT/mTORC1 pathway and upregulated by pAMPK expression. Downstream of autophagy activation, an increase in apoptosis was also observed in both cell lines after treatment with the two M2 agonists. Conclusions: N8 treatment causes autophagy via pAMPK upregulation, followed by apoptosis in both investigated cell lines. In contrast, the absence of autophagy in APE-treated GSC cells seems to indicate that cell death could be triggered by mechanisms alternative to those observed for N8.

C1 [Guerriero, Claudia; Manfredelli, Marianna; Iuzzolino, Angela; Tata, Ada Maria] Sapienza Univ Rome, Dept Biol & Biotechnol Charles Darwin, I-00185 Rome, Italy.

[Matera, Carlo; Dallanoce, Clelia; De Amici, Marco] Univ Milan, Dept Pharmaceut Sci, I-20133 Milan, Italy.

[Iuzzolino, Angela; Trisciuoglio, Daniela] CNR, Inst Mol Biol & Pathol, I-00185 Rome, Italy.

[Conti, Luciano] Univ Trento, Dept Cellular Computat & Integrat Biol CIBIO, I-38123 Trento, Italy.

[Tata, Ada Maria] Sapienza Univ Rome, Res Ctr Neurobiol Daniel Bovet, I-00185 Rome, Italy.

[Tata, Ada Maria] Univ Ferrara, Consortium Interuniv Biotechnol CIB, I-44121 Ferrara, Italy.

C3 Sapienza University Rome; University of Milan; Consiglio Nazionale delle

Ricerche (CNR); Istituto di Biologia e Patologia Molecolari (IBPM-CNR);

University of Trento; Sapienza University Rome; University of Ferrara

RP Tata, AM (corresponding author), Sapienza Univ Rome, Dept Biol & Biotechnol Charles Darwin, I-00185 Rome, Italy.; Tata, AM (corresponding author), Sapienza Univ Rome, Res Ctr Neurobiol Daniel Bovet, I-00185 Rome, Italy.; Tata, AM (corresponding author), Univ Ferrara, Consortium Interuniv Biotechnol CIB, I-44121 Ferrara, Italy.

EM claudia.guerriero@uniroma1.it; manfredellimarianna@gmail.com;

carlo.matera@unimi.it; angela.iuzzolino@uniroma1.it;

luciano.conti@unitn.it; clelia.dallanoce@unimi.it;

marco.deamici@unimi.it; daniela.trisciuoglio@uniroma1.it;

adamaria.tata@uniroma1.it

RI trisciuoglio, daniela/AAL-4002-2021; De Amici, Marco/F-9684-2015;

Trisciuoglio, Daniela/H-2131-2016; Conti, Luciano/H-4184-2012; Matera,

Carlo/G-3808-2015

OI TATA, Ada Maria/0000-0003-4868-5435; De Amici,

Marco/0000-0002-0236-0662; Trisciuoglio, Daniela/0000-0002-7007-7914;

Conti, Luciano/0000-0002-2050-9846; Dallanoce,

Clelia/0000-0002-7383-1484; Matera, Carlo/0000-0001-6939-3859

FU Ateneo Sapienza Funds

FX No Statement Available

CR Alessandrini F, 2015, INT IMMUNOPHARMACOL, V29, P105, DOI 10.1016/j.intimp.2015.05.032

Aoki H, 2008, AUTOPHAGY, V4, P467, DOI 10.4161/auto.5668

Baronchelli S, 2013, PLOS ONE, V8, DOI 10.1371/journal.pone.0057462

Bento CF, 2016, ANNU REV BIOCHEM, V85, P685, DOI 10.1146/annurev-biochem-060815-014556

Bhutia SK, 2013, ADV CANCER RES, V118, P61, DOI 10.1016/B978-0-12-407173-5.00003-0

Bock Andreas, 2013, Drug Discov Today Technol, V10, pe245, DOI 10.1016/j.ddtec.2012.12.003

Bock A, 2012, NAT COMMUN, V3, DOI 10.1038/ncomms2028

Calaf GM, 2022, CANCERS, V14, DOI 10.3390/cancers14092322

Cao WY, 2021, B CANCER, V108, P304, DOI 10.1016/j.bulcan.2020.11.004

Cristofaro I, 2020, CELLS-BASEL, V9, DOI 10.3390/cells9030657

Cristofaro I, 2018, NEUROCHEM INT, V118, P52, DOI 10.1016/j.neuint.2018.04.010

Davis AA, 2010, J NEUROSCI, V30, P4190, DOI 10.1523/JNEUROSCI.6393-09.2010

Di Bari M, 2021, CELLS-BASEL, V10, DOI 10.3390/cells10071727

Escamilla-Ramírez A, 2020, PHARMACEUTICALS-BASE, V13, DOI 10.3390/ph13070156

Eskelinen EL, 2019, INT J BIOCHEM CELL B, V111, P1, DOI 10.1016/j.biocel.2019.03.010

Español AJ, 2020, PLOS ONE, V15, DOI 10.1371/journal.pone.0226450

Feng YC, 2014, CELL RES, V24, P24, DOI 10.1038/cr.2013.168

Ferretti M, 2013, J CELL MOL MED, V17, P552, DOI 10.1111/jcmm.12038

Guerriero C, 2021, CELLS-BASEL, V10, DOI 10.3390/cells10081877

Gwinn DM, 2008, MOL CELL, V30, P214, DOI 10.1016/j.molcel.2008.03.003

Inoki K, 2006, CELL, V126, P955, DOI 10.1016/j.cell.2006.06.055

Inoki K, 2012, ANNU REV PHARMACOL, V52, P381, DOI 10.1146/annurev-pharmtox-010611-134537

Jeon J, 2010, J NEUROSCI, V30, P2396, DOI 10.1523/JNEUROSCI.3843-09.2010

Jia BX, 2018, ONCOL LETT, V16, P3509, DOI 10.3892/ol.2018.9131

Jung S, 2020, EXP MOL MED, V52, P921, DOI 10.1038/s12276-020-0455-4

Kocaturk NM, 2019, EUR J PHARM SCI, V134, P116, DOI 10.1016/j.ejps.2019.04.011

Kruse AC, 2014, NAT REV DRUG DISCOV, V13, P549, DOI 10.1038/nrd4295

Li B, 2018, ONCOL LETT, V15, P2477, DOI 10.3892/ol.2017.7537

Li XY, 2011, J NEURO-ONCOL, V103, P453, DOI 10.1007/s11060-010-0424-1

Lucianò AM, 2020, INT J MOL SCI, V21, DOI 10.3390/ijms21228433

Mecca C, 2018, DIS MARKERS, V2018, DOI 10.1155/2018/9230479

Medeiros R, 2011, AM J PATHOL, V179, P980, DOI 10.1016/j.ajpath.2011.04.041

Mizushima N, 2010, CURR OPIN CELL BIOL, V22, P132, DOI 10.1016/j.ceb.2009.12.004

MOSMANN T, 1983, J IMMUNOL METHODS, V65, P55, DOI 10.1016/0022-1759(83)90303-4

Pirtoli L, 2009, AUTOPHAGY, V5, P930, DOI 10.4161/auto.5.7.9227

Pollard SM, 2009, CELL STEM CELL, V4, P568, DOI 10.1016/j.stem.2009.03.014

Runwal G, 2019, SCI REP-UK, V9, DOI 10.1038/s41598-019-46657-z

Sales ME, 2019, CURR CLIN PHARMACOL, V14, P91, DOI 10.2174/1574884714666181203095437

Shah N, 2009, AM J PHYSIOL-CELL PH, V296, pC221, DOI 10.1152/ajpcell.00514.2008

Shukla S, 2014, J BIOL CHEM, V289, P22306, DOI 10.1074/jbc.M114.567032

Song P, 2007, CANCER RES, V67, P3936, DOI 10.1158/0008-5472.CAN-06-2484

Taggi M, 2022, J CELL BIOCHEM, V123, P1440, DOI 10.1002/jcb.30303

Vial D, 2016, MOL CARCINOGEN, V55, P1118, DOI 10.1002/mc.22346

Wess J, 2007, NAT REV DRUG DISCOV, V6, P721, DOI 10.1038/nrd2379

Xi HY, 2022, ONCOL REP, V48, DOI 10.3892/or.2022.8423

NR 45

TC 3

Z9 3

U1 2

U2 4

PU MDPI

PI BASEL

PA ST ALBAN-ANLAGE 66, CH-4052 BASEL, SWITZERLAND

EI 2072-6694

J9 CANCERS

JI Cancers

PD JAN

PY 2024

VL 16

IS 1

AR 25

DI 10.3390/cancers16010025

PG 19

WC Oncology

WE Science Citation Index Expanded (SCI-EXPANDED)

SC Oncology

GA EL8L6

UT WOS:001139173000001

PM 38201453

OA Green Published, gold

DA 2025-04-09

ER

PT J

AU Murota, Y

Tabu, K

Taga, T

AF Murota, Yoshitaka

Tabu, Kouichi

Taga, Tetsuya

TI Requirement of ABC transporter inhibition and Hoechst 33342 dye

deprivation for the assessment of side population-defined C6 glioma stem

cell metabolism using fluorescent probes

SO BMC CANCER

LA English

DT Article

DE Cancer stem cells; Side population; Fluorescent probes; ABC

transporters; JC-1; Hoechst 33342

ID CANCER-CELLS; SUBPOPULATION; GLIOBLASTOMA; REVEALS; TUMORS; JC-1; LINE

AB Background: Elucidating the precise properties of cancer stem cells (CSCs) is indispensable for the development of effective therapies against tumors, because CSCs are key drivers of tumor development, metastasis and relapse. We previously reported that the Hoechst 33342 dye-low staining side population (SP) method can enrich for CSCs in the C6 glioma cell line, and that the positively stained main population (MP) cells are non-CSCs. Presence of cancer stem-like SP cells is reported in various types of cancer. Although altered cellular energy metabolism is a hallmark of cancer, very little has been studied on the applicability of fluorescent probes for the understanding of CSC energy metabolism.

Methods: The metabolic status of C6 SP and MP cells are evaluated by CellROX, MitoTracker Green (MTG) and JC-1 for cellular oxidative stress, mitochondrial amount, and mitochondrial membrane potential, respectively.

Results: SP cells were found to exhibit significantly lower fluorescent intensities of CellROX and MTG than MP cells. However, inhibition of ATP binding cassette (ABC) transporters by verapamil enhanced the intensities of these probes in SP cells to the levels similar to those in MP cells, indicating that SP cells expel the probes outside of the cells through ABC transporters. Next, SP cells were stained with JC-1 dye which exhibits membrane potential dependent accumulation in mitochondrial matrix, followed by formation of aggregates. The mitochondrial membrane potential indicated by the aggregates of JC-1 was 5.0-fold lower in SP cells than MP cells. Inhibition of ABC transporters enhanced the fluorescent intensities of the JC-1 aggregates in both SP and MP cells, the former of which was still 2.2-fold lower than the latter. This higher JC-1 signal in MP cells was further found to be due to the Hoechst 33342 dye existing in MP cells. When SP and MP cells were recultured to deprive the intracellular Hoechst 33342 dye and then stained with JC-1 in the presence of verapamil, the intensities of JC-1 aggregates in such SP and MP cells became comparable.

Conclusion: Inhibiting ABC transporters and depriving Hoechst 33342 dye are required for the accurate assessment of side population-defined C6 glioma stem cell metabolism using fluorescent probes.

C1 [Murota, Yoshitaka; Tabu, Kouichi; Taga, Tetsuya] Tokyo Med & Dent Univ, Med Res Inst, Dept Stem Cell Regulat, Bunkyo Ku, Tokyo 1138510, Japan.

C3 Institute of Science Tokyo; Tokyo Medical & Dental University (TMDU)

RP Taga, T (corresponding author), Tokyo Med & Dent Univ, Med Res Inst, Dept Stem Cell Regulat, Bunkyo Ku, Tokyo 1138510, Japan.

EM taga.scr@mri.tmd.ac.jp

RI Tabu, Kouichi/AAW-1225-2020

OI Murota, Yoshitaka/0000-0003-1197-652X; Taga, Tetsuya/0000-0002-1625-0122

FU MEXT KAKENHI [22130008]; JSPS KAKENHI [15H04292]; Joint Usage/Research

Program of Medical Research Institute, TMDU; Grants-in-Aid for

Scientific Research [15H04292, 22130008, 15K20970] Funding Source: KAKEN

FX This work was supported by MEXT KAKENHI Grant Number 22130008 (TT), JSPS

KAKENHI Grant number 15H04292 (TT), and Joint Usage/Research Program of

Medical Research Institute, TMDU (KT, TT).

CR Agnello M, 2008, CYTOTECHNOLOGY, V56, P145, DOI 10.1007/s10616-008-9143-2

Gil MA, 2012, MOL REPROD DEV, V79, P651, DOI 10.1002/mrd.22071

Cairns RA, 2011, NAT REV CANCER, V11, P85, DOI 10.1038/nrc2981

Chen JC, 2004, ANN CLIN LAB SCI, V34, P458

Chiba T, 2006, HEPATOLOGY, V44, P240, DOI 10.1002/hep.21227

Dean M, 2005, NAT REV CANCER, V5, P275, DOI 10.1038/nrc1590

Diehn M, 2009, NATURE, V458, P780, DOI 10.1038/nature07733

Duan JJ, 2013, STEM CELLS DEV, V22, P2221, DOI 10.1089/scd.2012.0613

Haraguchi N, 2006, STEM CELLS, V24, P506, DOI 10.1634/stemcells.2005-0282

Ho MM, 2007, CANCER RES, V67, P4827, DOI 10.1158/0008-5472.CAN-06-3557

Jain S, 2013, BIOMATERIALS, V34, P4891, DOI 10.1016/j.biomaterials.2013.03.055

Johnson I, 1998, HISTOCHEM J, V30, P123, DOI 10.1023/A:1003287101868

Keil VC, 2011, PFLUG ARCH EUR J PHY, V462, P693, DOI 10.1007/s00424-011-1012-8

Kokubu Y, 2016, GENES CELLS, V21, P241, DOI 10.1111/gtc.12333

Kondo T, 2004, P NATL ACAD SCI USA, V101, P781, DOI 10.1073/pnas.0307618100

Kuhnel JM, 1997, LEUKEMIA, V11, P1147, DOI 10.1038/sj.leu.2400698

Landreville S, 2011, PIGM CELL MELANOMA R, V24, P430, DOI 10.1111/j.1755-148X.2011.00841.x

Marques-Santos LF, 2003, BIOSCIENCE REP, V23, P199, DOI 10.1023/B:BIRE.0000007693.33521.18

Muramatsu S, 2013, HEPATOLOGY, V58, P218, DOI 10.1002/hep.26345

Nakai E, 2009, CANCER INVEST, V27, P901, DOI 10.3109/07357900801946679

Patrawala L, 2005, CANCER RES, V65, P6207, DOI 10.1158/0008-5472.CAN-05-0592

Strouse JJ, 2013, ANAL BIOCHEM, V437, P77, DOI 10.1016/j.ab.2013.02.018

Tabu K, 2016, STEM CELLS, V34, P1151, DOI 10.1002/stem.2299

Visvader JE, 2008, NAT REV CANCER, V8, P755, DOI 10.1038/nrc2499

Vlashi E, 2011, P NATL ACAD SCI USA, V108, P16062, DOI 10.1073/pnas.1106704108

Wu CL, 2007, CANCER RES, V67, P8216, DOI 10.1158/0008-5472.CAN-07-0999

Ye XQ, 2011, INT J CANCER, V129, P820, DOI 10.1002/ijc.25944

Zhou S, 2001, NAT MED, V7, P1028, DOI 10.1038/nm0901-1028

Zhou YF, 2011, J BIOL CHEM, V286, P32843, DOI 10.1074/jbc.M111.260935

NR 29

TC 17

Z9 18

U1 0

U2 17

PU BIOMED CENTRAL LTD

PI LONDON

PA 236 GRAYS INN RD, FLOOR 6, LONDON WC1X 8HL, ENGLAND

SN 1471-2407

J9 BMC CANCER

JI BMC Cancer

PD NOV 4

PY 2016

VL 16

AR 847

DI 10.1186/s12885-016-2895-8

PG 7

WC Oncology

WE Science Citation Index Expanded (SCI-EXPANDED)

SC Oncology

GA EB8KJ

UT WOS:000387639600001

PM 27814696

OA Green Published, gold

DA 2025-04-09

ER

PT J

AU Caffo, M

Curcio, A

Rajiv, K

Caruso, G

Venza, M

Germano, A

AF Caffo, Maria

Curcio, Antonello

Rajiv, Kumar

Caruso, Gerardo

Venza, Mario

Germano, Antonino

TI Potential Role of Carbon Nanomaterials in the Treatment of Malignant

Brain Gliomas

SO CANCERS

LA English

DT Review

DE blood-brain barrier; brain drug delivery; carbon nanomaterials; cerebral

gliomas; glioblastoma; nanoparticles

ID CENTRAL-NERVOUS-SYSTEM; GRAPHENE OXIDE; DRUG-DELIVERY; IN-VITRO;

ENGINEERED NANOPARTICLES; ADJUVANT TEMOZOLOMIDE; MOLECULAR-MECHANISMS;

ACHETA-DOMESTICUS; ANTICANCER DRUGS; OXIDATIVE STRESS

AB Simple Summary: Nanomaterials are one of the most promising discoveries of this millennium. Thanks to the widest range of applications, this field has spread to all scientific disciplines. As well, interest has significantly increased in the medical sector. Although there are many different families of nanoparticles, carbon-based nanoparticles have only recently come to light. An infinite number of nanoparticles of various shapes and sizes can be produced by taking advantage of the chemical bonding properties of carbon, which also allows for the modification of their chemical, thermal, and physical properties. This review examines the biomolecular aspects of the theoretical and practical challenges involved in creating nanoparticles with biological activity, identifying the benefits and drawbacks of each approach, and summarizing the most recent research on carbon-based nanoparticles conceptualized and developed to date. Although it is a very promising area of study, more pharmacokinetic and toxicological research is still required.Malignant gliomas are the most common primary brain tumors in adults up to an extent of 78% of all primary malignant brain tumors. However, total surgical resection is almost unachievable due to the considerable infiltrative ability of glial cells. The efficacy of current multimodal therapeutic strategies is, furthermore, limited by the lack of specific therapies against malignant cells, and, therefore, the prognosis of these in patients is still very unfavorable. The limitations of conventional therapies, which may result from inefficient delivery of the therapeutic or contrast agent to brain tumors, are major reasons for this unsolved clinical problem. The major problem in brain drug delivery is the presence of the blood-brain barrier, which limits the delivery of many chemotherapeutic agents. Nanoparticles, thanks to their chemical configuration, are able to go through the blood-brain barrier carrying drugs or genes targeted against gliomas. Carbon nanomaterials show distinct properties including electronic properties, a penetrating capability on the cell membrane, high drug-loading and pH-dependent therapeutic unloading capacities, thermal properties, a large surface area, and easy modification with molecules, which render them as suitable candidates for deliver drugs. In this review, we will focus on the potential effectiveness of the use of carbon nanomaterials in the treatment of malignant gliomas and discuss the current progress of in vitro and in vivo researches of carbon nanomaterials-based drug delivery to brain.

C1 [Caffo, Maria; Curcio, Antonello; Caruso, Gerardo; Venza, Mario; Germano, Antonino] Univ Messina, Dept Biomed & Dent Sci & Morphofunct Imaging, Neurosurg Clin, I-98125 Messina, Italy.

[Rajiv, Kumar] Natl Inst Med Sci, NIET, New Delhi 110007, India.

[Rajiv, Kumar] Univ Delhi, New Delhi 110007, India.

C3 University of Messina; University of Delhi

RP Caruso, G (corresponding author), Univ Messina, Dept Biomed & Dent Sci & Morphofunct Imaging, Neurosurg Clin, I-98125 Messina, Italy.

EM crcnnl91s28e977t@studenti.unime.it; gcaruso@unime.it

RI Venza, Mario/LRU-9496-2024; Caruso, Gerardo/H-5312-2019; Caffo,

Maria/AAP-1678-2021; KUMAR, RAJIV/I-7831-2013

OI Curcio, Antonello/0000-0003-1783-9710; Caffo, Maria/0000-0002-3240-0506;

Caruso, Gerardo/0000-0001-8505-817X; Venza, Mario/0000-0002-4221-8863

CR Akbarzadeh A, 2013, NANOSCALE RES LETT, V8, DOI 10.1186/1556-276X-8-102

Akçan R, 2020, TURK J MED SCI, V50, P1180, DOI 10.3906/sag-1912-209

Aktas Y, 2005, BIOCONJUGATE CHEM, V16, P1503, DOI 10.1021/bc050217o

Alshehri R, 2016, J MED CHEM, V59, P8149, DOI 10.1021/acs.jmedchem.5b01770

Baldrighi M, 2016, FRONT NEUROSCI-SWITZ, V10, DOI 10.3389/fnins.2016.00250

Bar-Zeev M, 2017, DRUG RESIST UPDATE, V31, P15, DOI 10.1016/j.drup.2017.05.002

Bardi G, 2013, PLOS ONE, V8, DOI 10.1371/journal.pone.0080964

Barhoum A, 2022, NANOMATERIALS-BASEL, V12, DOI 10.3390/nano12020177

Behzadi S, 2017, CHEM SOC REV, V46, P4218, DOI 10.1039/c6cs00636a

Benos L, 2019, COMPUT METH PROG BIO, V172, P79, DOI 10.1016/j.cmpb.2019.02.008

Berg JM., 2002, MICHAELIS MENTEN MOD

Caffo M., 2013, EVOLUTION MOL BIOL B

Caffo M, 2023, ADV EXP MED BIOL, V1394, P181, DOI 10.1007/978-3-031-14732-6_11

Caruso G., 2017, Nano- and microscale drug delivery systems, P95, DOI [10.1016/B978-0-323-52727-9.00006-6, DOI 10.1016/B978-0-323-52727-9.00006-6]

Caruso G., 2014, Innovative Brain Tumor Therapy. Nanoparticle-Based Therapeutic Strategies, P1

Caruso G, 2011, NANOMED-NANOTECHNOL, V7, P744, DOI 10.1016/j.nano.2011.02.008

Chadha U, 2022, ECS J SOLID STATE SC, V11, DOI 10.1149/2162-8777/ac5c83

Cheng WW, 2011, INT J BIOCHEM CELL B, V43, P564, DOI 10.1016/j.biocel.2010.12.013

Chowdhury SM, 2015, NANOMED-NANOTECHNOL, V11, P109, DOI 10.1016/j.nano.2014.08.001

Cooke MS, 2003, FASEB J, V17, P1195, DOI 10.1096/fj.02-0752rev

Costa Pedro Miguel, 2018, Nanotheranostics, V2, P168, DOI 10.7150/ntno.23125

Deng CY, 2008, BIOSENS BIOELECTRON, V23, P1272, DOI 10.1016/j.bios.2007.11.009

Dubin C.H., 2004, MECH ENG, V126, pS10

Dworak N, 2014, CARBON, V68, P763, DOI 10.1016/j.carbon.2013.11.067

Dziewiecka M, 2016, J HAZARD MATER, V305, P30, DOI 10.1016/j.jhazmat.2015.11.021

Facciolà A, 2019, ENVIRON TOXICOL PHAR, V65, P23, DOI 10.1016/j.etap.2018.11.006

Fan ZY, 2020, CURR OPIN BIOTECH, V66, P131, DOI 10.1016/j.copbio.2020.07.006

Firme CP, 2010, NANOMED-NANOTECHNOL, V6, P245, DOI 10.1016/j.nano.2009.07.003

Friedman HS, 2009, J CLIN ONCOL, V27, P4733, DOI 10.1200/JCO.2008.19.8721

Guo X, 2013, BIOMATERIALS, V34, P4544, DOI 10.1016/j.biomaterials.2013.02.071

Gupta SK, 2011, J BIOMED NANOTECHNOL, V7, P179, DOI 10.1166/jbn.2011.1258

Hettiarachchi SD, 2019, NANOSCALE, V11, P6192, DOI 10.1039/c8nr08970a

Hone J, 2002, APPL PHYS A-MATER, V74, P339, DOI 10.1007/s003390201277

Horie M, 2021, FREE RADICAL RES, V55, P331, DOI 10.1080/10715762.2020.1859108

Hsieh FY, 2017, ACS APPL MATER INTER, V9, P11482, DOI 10.1021/acsami.7b01077

Hurt RH, 2006, CARBON, V44, P1028, DOI 10.1016/j.carbon.2005.12.023

Jiang YQ, 2013, PLOS ONE, V8, DOI 10.1371/journal.pone.0065756

Jiwanti PK, 2022, MOLECULES, V27, DOI 10.3390/molecules27217578

Jovic D, 2020, NANOMATERIALS-BASEL, V10, DOI 10.3390/nano10081508

Jung YS, 2009, ENVIRON TECHNOL, V30, P183, DOI 10.1080/09593330802468848

Kafa H, 2015, BIOMATERIALS, V53, P437, DOI 10.1016/j.biomaterials.2015.02.083

Kagan VE, 2006, TOXICOL LETT, V165, P88, DOI 10.1016/j.toxlet.2006.02.001

Kang S, 2015, J NANOPART RES, V17, DOI 10.1007/s11051-015-3181-4

Karpeta-Kaczmarek J, 2018, ENVIRON RES, V166, P602, DOI 10.1016/j.envres.2018.05.027

Ke WL, 2009, BIOMATERIALS, V30, P6976, DOI 10.1016/j.biomaterials.2009.08.049

Kim M, 2020, NEUROTOXICOLOGY, V77, P30, DOI 10.1016/j.neuro.2019.12.011

Kumar P, 2007, NATURE, V448, P39, DOI 10.1038/nature05901

Kutty RV, 2014, NANOMEDICINE-UK, V9, P561, DOI [10.2217/NNM.14.19, 10.2217/nnm.14.19]

Kwon GS, 2003, CRIT REV THER DRUG, V20, P357, DOI 10.1615/CritRevTherDrugCarrierSyst.v20.i5.20

Lee H, 2021, BIOMATERIALS, V279, DOI 10.1016/j.biomaterials.2021.121232

Li SH, 2016, COLLOID SURFACE B, V145, P251, DOI 10.1016/j.colsurfb.2016.05.007

Li TF, 2019, ACTA BIOMATER, V86, P381, DOI 10.1016/j.actbio.2019.01.020

Li Y, 2012, BIOMATERIALS, V33, P402, DOI 10.1016/j.biomaterials.2011.09.091

Liu GD, 2013, ACS APPL MATER INTER, V5, P6909, DOI 10.1021/am402128s

Liu HY, 2017, J MATER CHEM B, V5, P6437, DOI 10.1039/c7tb00891k

Liu YC, 2017, SEMIN IMMUNOL, V34, P25, DOI 10.1016/j.smim.2017.09.011

Liyanage PY, 2020, NANOSCALE, V12, P7927, DOI 10.1039/d0nr01647k

Louis DN, 2021, NEURO-ONCOLOGY, V23, P1231, DOI 10.1093/neuonc/noab106

Lu CT, 2014, INT J NANOMED, V9, P2241, DOI 10.2147/IJN.S61288

Lu SS, 2016, INT J NANOMED, V11, P6325, DOI 10.2147/IJN.S119252

Lu YJ, 2012, INT J NANOMED, V7, P1737, DOI 10.2147/IJN.S29376

Magdolenova Z, 2014, NANOTOXICOLOGY, V8, P233, DOI 10.3109/17435390.2013.773464

Manke A, 2013, BIOMED RES INT, V2013, DOI 10.1155/2013/942916

Manshian BB, 2013, NANOTOXICOLOGY, V7, P144, DOI 10.3109/17435390.2011.647928

Mao Z, 2011, INT J MOL SCI, V12, P114, DOI 10.3390/ijms12010114

Maria C, 2015, NANOMEDICINE-UK, V10, P615, DOI 10.2217/nnm.14.195

Martins SM, 2013, EUR J PHARM BIOPHARM, V85, P488, DOI 10.1016/j.ejpb.2013.08.011

McCallion C, 2016, EUR J PHARM BIOPHARM, V104, P235, DOI 10.1016/j.ejpb.2016.04.015

Mendes RG, 2013, J MATER CHEM B, V1, P401, DOI 10.1039/c2tb00085g

Mengesha A.E., 2013, Diamond-Based Materials for Biomedical Applications, P186, DOI 10.1533/9780857093516.2.186

Mohajeri M, 2019, J CELL PHYSIOL, V234, P298, DOI 10.1002/jcp.26899

Mrdanovic J, 2009, MUTAT RES-GEN TOX EN, V680, P25, DOI 10.1016/j.mrgentox.2009.08.008

Novoselov KS, 2004, SCIENCE, V306, P666, DOI 10.1126/science.1102896

Orozco F, 2022, POLYMER, V260, DOI 10.1016/j.polymer.2022.125365

Ostrom QT, 2018, NEURO-ONCOLOGY, V20, P1, DOI 10.1093/neuonc/noy131

Ou LL, 2016, PART FIBRE TOXICOL, V13, DOI 10.1186/s12989-016-0168-y

Pacurari M, 2008, ENVIRON HEALTH PERSP, V116, P1211, DOI 10.1289/ehp.10924

Paital B, 2013, ECOTOX ENVIRON SAFE, V87, P33, DOI 10.1016/j.ecoenv.2012.10.006

Porto LS, 2019, REV ANAL CHEM, V38, DOI 10.1515/revac-2019-0017

Power AC, 2018, NANOTECHNOL REV, V7, P19, DOI 10.1515/ntrev-2017-0160

Qin Y, 2010, J DRUG TARGET, V18, P536, DOI 10.3109/10611861003587235

Rapoport N, 2000, ARCH BIOCHEM BIOPHYS, V384, P100, DOI 10.1006/abbi.2000.2104

Rauti R, 2019, CARBON, V143, P430, DOI 10.1016/j.carbon.2018.11.026

Ren JF, 2012, BIOMATERIALS, V33, P3324, DOI 10.1016/j.biomaterials.2012.01.025

Rinaldi M, 2016, INT J MOL SCI, V17, DOI 10.3390/ijms17060984

Roman JA, 2011, J NEUROTRAUM, V28, P2349, DOI 10.1089/neu.2010.1409

Salahudeen MS, 2017, SAUDI PHARM J, V25, P165, DOI 10.1016/j.jsps.2016.07.002

Saleemi MA, 2021, CHEM RES TOXICOL, V34, P24, DOI 10.1021/acs.chemrestox.0c00172

Samadian H, 2020, MUTAT RES-REV MUTAT, V783, DOI 10.1016/j.mrrev.2020.108296

Samanta PN, 2017, J MOL GRAPH MODEL, V72, P187, DOI 10.1016/j.jmgm.2017.01.009

Scida K, 2011, ANAL CHIM ACTA, V691, P6, DOI 10.1016/j.aca.2011.02.025

Shang WH, 2014, NANOSCALE, V6, P5799, DOI 10.1039/c3nr06433f

Shapira C, 2022, ACS NANO, V16, P15760, DOI 10.1021/acsnano.2c03613

Sharma A, 2021, COLLOID SURFACE B, V200, DOI 10.1016/j.colsurfb.2021.111572

Shvedova AA, 2003, J TOXICOL ENV HEAL A, V66, P1909, DOI 10.1080/713853956

Simpson S, 2019, APPL SCI-BASEL, V9, DOI 10.3390/app9010087

Song MM, 2017, MAT SCI ENG C-MATER, V77, P904, DOI 10.1016/j.msec.2017.03.309

Stupp R, 2005, NEW ENGL J MED, V352, P987, DOI 10.1056/NEJMoa043330

Stupp R, 2009, LANCET ONCOL, V10, P459, DOI 10.1016/S1470-2045(09)70025-7

Suk JS, 2016, ADV DRUG DELIVER REV, V99, P28, DOI 10.1016/j.addr.2015.09.012

Sumi N, 2019, MUTAT RES-GEN TOX EN, V847, DOI 10.1016/j.mrgentox.2019.503104

Szczepaniak J, 2022, MATERIALS, V15, DOI 10.3390/ma15175843

Szczepaniak J, 2021, INT J MOL SCI, V22, DOI 10.3390/ijms22020515

Takakura A, 2019, NAT COMMUN, V10, DOI 10.1038/s41467-019-10959-7

Tykhomyrov AA, 2008, TOXICOLOGY, V246, P158, DOI 10.1016/j.tox.2008.01.005

Ulbrich K, 2009, EUR J PHARM BIOPHARM, V71, P251, DOI 10.1016/j.ejpb.2008.08.021

Valcárcel M, 2008, TRAC-TREND ANAL CHEM, V27, P34, DOI 10.1016/j.trac.2007.10.012

Wang C, 2015, NANO RES, V8, P1975, DOI 10.1007/s12274-015-0707-0

Wang HH, 2013, ENVIRON SCI TECHNOL, V47, P13212, DOI 10.1021/es404527d

Wang SS, 2017, BIOMATERIALS, V141, P29, DOI 10.1016/j.biomaterials.2017.05.040

Wei XL, 2014, ACTA PHARM SIN B, V4, P193, DOI 10.1016/j.apsb.2014.03.001

Weller M, 2017, LANCET ONCOL, V18, pE315, DOI 10.1016/S1470-2045(17)30194-8

Wilson B, 2014, INT J PHARMACEUT, V473, P73, DOI 10.1016/j.ijpharm.2014.05.056

Wu C, 2014, NANOSCALE RES LETT, V9, DOI 10.1186/1556-276X-9-447

Yamawaki H, 2006, AM J PHYSIOL-CELL PH, V290, pC1495, DOI 10.1152/ajpcell.00481.2005

Yang CB, 2017, NANO RES, V10, P3049, DOI 10.1007/s12274-017-1521-7

Yang ST, 2012, THERANOSTICS, V2, P271, DOI 10.7150/thno.3618

Yang XL, 2019, ENVIRON TOXICOL, V34, P415, DOI 10.1002/tox.22695

Yao HY, 2020, ENVIRON SCI-NANO, V7, P782, DOI 10.1039/c9en01295h

You YY, 2019, DALTON T, V48, P1569, DOI 10.1039/c8dt03948h

Yousefi M, 2017, MAT SCI ENG C-MATER, V74, P568, DOI 10.1016/j.msec.2016.12.125

Yuan X, 2019, PART FIBRE TOXICOL, V16, DOI 10.1186/s12989-019-0299-z

Zhang MF, 2019, ACS APPL NANO MATER, V2, P4293, DOI 10.1021/acsanm.9b00757

Zhang TT, 2016, BIOMATER SCI-UK, V4, P219, DOI 10.1039/c5bm00383k

Zhang XL, 2017, ENVIRON SCI TECHNOL, V51, P7861, DOI 10.1021/acs.est.7b01922

Zhao DC, 2011, CLIN CANCER RES, V17, P771, DOI 10.1158/1078-0432.CCR-10-2444

NR 126

TC 6

Z9 7

U1 3

U2 20

PU MDPI

PI BASEL

PA ST ALBAN-ANLAGE 66, CH-4052 BASEL, SWITZERLAND

EI 2072-6694

J9 CANCERS

JI Cancers

PD APR 30

PY 2023

VL 15

IS 9

AR 2575

DI 10.3390/cancers15092575

PG 24

WC Oncology

WE Science Citation Index Expanded (SCI-EXPANDED)

SC Oncology

GA G1YW4

UT WOS:000987206700001

PM 37174040

OA Green Submitted, gold, Green Published

DA 2025-04-09

ER

PT J

AU Bekeschus, S

Ispirjan, M

Freund, E

Kinnen, F

Moritz, J

Saadati, F

Eckroth, J

Singer, D

Stope, MB

Wende, K

Ritter, CA

Schroeder, HWS

Marx, S

AF Bekeschus, Sander

Ispirjan, Mikael

Freund, Eric

Kinnen, Frederik

Moritz, Juliane

Saadati, Fariba

Eckroth, Jacqueline

Singer, Debora

Stope, Matthias B.

Wende, Kristian

Ritter, Christoph A.

Schroeder, Henry W. S.

Marx, Sascha

TI Gas Plasma Exposure of Glioblastoma Is Cytotoxic and Immunomodulatory in

Patient-Derived GBM Tissue

SO CANCERS

LA English

DT Article

DE brain tumor; chemokines; cytokines; cold physical plasma; reactive

oxygen species

ID COLD ATMOSPHERIC PLASMA; OXIDATIVE STRESS; EGFR INHIBITION;

GLIOMA-CELLS; EXPRESSION; TUMOR; PROLIFERATION; MULTIFORME; RESISTANCE;

RECEPTOR

AB Simple Summary Despite treatment advances, glioblastoma multiforme (GBM) remains an often-fatal disease, motivating novel therapeutic avenues. Gas plasma is a technology that has been recently employed in preclinical oncology research and acts primarily via reactive oxygen-species-induced cell death. In addition, the modulation of immune processes and inflammation have been ascribed to gas plasma exposure. This is the first study that extends those observations from in vitro investigations to a set of 16 patient-derived GBM tumor biopsies analyzed after gas plasma treatment ex vivo. Besides cell culture results showing cell cycle arrest and apoptosis induction, an immunomodulatory potential was identified for gas plasma exposure in vitro and cultured GBM tissues. The proapoptotic action shown in this study might be an important step forward to the first clinical observational studies on the future discovery of gas plasma technology's potential in neurosurgery and neuro-oncology. Glioblastoma multiforme (GBM) is the most common primary malignant adult brain tumor. Therapeutic options for glioblastoma are maximal surgical resection, chemotherapy, and radiotherapy. Therapy resistance and tumor recurrence demand, however, new strategies. Several experimental studies have suggested gas plasma technology, a partially ionized gas that generates a potent mixture of reactive oxygen species (ROS), as a future complement to the existing treatment arsenal. However, aspects such as immunomodulation, inflammatory consequences, and feasibility studies using GBM tissue have not been addressed so far. In vitro, gas plasma generated ROS that oxidized cells and led to a treatment time-dependent metabolic activity decline and G2 cell cycle arrest. In addition, peripheral blood-derived monocytes were co-cultured with glioblastoma cells, and immunomodulatory surface expression markers and cytokine release were screened. Gas plasma treatment of either cell type, for instance, decreased the expression of the M2-macrophage marker CD163 and the tolerogenic molecule SIGLEC1 (CD169). In patient-derived GBM tissue samples exposed to the plasma jet kINPen ex vivo, apoptosis was significantly increased. Quantitative chemokine/cytokine release screening revealed gas plasma exposure to significantly decrease 5 out of 11 tested chemokines and cytokines, namely IL-6, TGF-beta, sTREM-2, b-NGF, and TNF-alpha involved in GBM apoptosis and immunomodulation. In summary, the immuno-modulatory and proapoptotic action shown in this study might be an important step forward to first clinical observational studies on the future discovery of gas plasma technology's potential in neurosurgery and neuro-oncology especially in putative adjuvant or combinatory GBM treatment settings.

C1 [Bekeschus, Sander; Ispirjan, Mikael; Freund, Eric; Kinnen, Frederik; Moritz, Juliane; Saadati, Fariba; Eckroth, Jacqueline; Singer, Debora; Wende, Kristian] Leibniz Inst Plasma Sci & Technol INP, ZIK Plasmatis, Felix Hausdorff Str 2, D-17489 Greifswald, Germany.

[Ispirjan, Mikael; Kinnen, Frederik; Schroeder, Henry W. S.; Marx, Sascha] Greifswald Univ, Med Ctr, Dept Neurosurg, Ferdinand Sauerbruch Str, D-17475 Greifswald, Germany.

[Freund, Eric] Greifswald Univ, Med Ctr, Dept Gen Visceral Thorac & Vasc Surg, Ferdinand Sauerbruch Str, D-17475 Greifswald, Germany.

[Saadati, Fariba] Rostock Univ, Med Ctr, Clin & Policlin Dermatol & Venerol, Strempelstr 13, D-18057 Rostock, Germany.

[Stope, Matthias B.] Univ Bonn, Med Ctr, Dept Gynecol & Gynecol Oncol, Venusberg Campus 1, D-53127 Bonn, Germany.

[Ritter, Christoph A.] Univ Greifswald, Dept Clin Pharmaceut, Felix Hausdorff Str 1, D-17489 Greifswald, Germany.

[Marx, Sascha] Harvard Med Sch, Dana Farber Canc Inst, Dept Canc Immunol & Virol, Boston, MA 02215 USA.

C3 Leibniz Institut fur Plasmaforschung und Technologie; University of

Rostock; University of Bonn; Universitat Greifswald; Harvard University;

Harvard Medical School; Harvard University Medical Affiliates;

Dana-Farber Cancer Institute

RP Bekeschus, S (corresponding author), Leibniz Inst Plasma Sci & Technol INP, ZIK Plasmatis, Felix Hausdorff Str 2, D-17489 Greifswald, Germany.

EM sander.bekeschus@inp-greifswald.de;

mikael.ispirjan@stud.uni-greifswald.de; eric.freund@inp-greifswald.de;

fk133993@uni-greifswald.de; juliane.moritz@inp-greifswald.de;

fariba.saadati@inp-greifswald.de; jacqueline.eckroth@inp-greifswald.de;

debora.singer@inp-greifswald.de; matthias.Stope@ukbonn.de;

kristian.wende@inp-greifswald.de; ritter@uni-greifswald.de;

henry.schroeder@uni-greifswald.de; sascha.marx@uni-greifswald.de

RI Wende, Kristian/AAE-8810-2020; Bekeschus, Sander/KSM-1629-2024

OI Marx, Sascha/0000-0002-6451-5404; Bekeschus, Sander/0000-0002-8773-8862;

Wende, Kristian/0000-0001-5217-0683; Singer, Debora/0000-0003-2335-1607;

Freund, Eric/0000-0003-2950-0984

FU German Federal Ministry of Education and Research [03Z22DN11]; Gerhard

Domagk Foundation Greifswald; Lieselotte-Beutel Foundation (Berlin,

Germany)

FX This research was funded by the German Federal Ministry of Education and

Research, grant number 03Z22DN11 (to S.B.), the Gerhard Domagk

Foundation Greifswald (to S.M.), and the Lieselotte-Beutel Foundation

(Berlin, Germany).

CR Achyut BR, 2017, SCI REP-UK, V7, DOI 10.1038/s41598-017-14079-4

Adhikari M, 2020, CURR PHARM DESIGN, V26, P2195, DOI 10.2174/1381612826666200302105715

Affandi AJ, 2021, FRONT IMMUNOL, V12, DOI 10.3389/fimmu.2021.697840

Akbari Z, 2021, APPL SCI-BASEL, V11, DOI 10.3390/app11104527

Akter M, 2021, CELLS-BASEL, V10, DOI 10.3390/cells10020236

Akter M, 2020, CANCERS, V12, DOI 10.3390/cancers12010245

Ali S, 2021, PLOS ONE, V16, DOI 10.1371/journal.pone.0246646

Apetoh L, 2007, IMMUNOL REV, V220, P47, DOI 10.1111/j.1600-065X.2007.00573.x

Bald T, 2014, CANCER DISCOV, V4, P674, DOI 10.1158/2159-8290.CD-13-0458

Balermpas P, 2014, BRIT J CANCER, V111, P1509, DOI 10.1038/bjc.2014.446

Bedin AS, 2021, J INFECT DIS, V223, P562, DOI 10.1093/infdis/jiaa724

Bekeschus S, 2014, FREE RADICAL RES, V48, P542, DOI 10.3109/10715762.2014.892937

Bekeschus S, 2020, APPL SCI-BASEL, V10, DOI 10.3390/app10061971

Bekeschus S, 2017, JOVE-J VIS EXP, DOI 10.3791/56331

Bekeschus S, 2016, CLIN PLASMA MED, V4, P19, DOI 10.1016/j.cpme.2016.01.001

Bekeschus S, 2016, OXID MED CELL LONGEV, V2016, DOI 10.1155/2016/5910695

Bekeschus S, 2013, PLASMA PROCESS POLYM, V10, P706, DOI 10.1002/ppap.201300008

Benner B, 2019, J IMMUNOTHER CANCER, V7, DOI 10.1186/s40425-019-0622-0

BORSELLINO N, 1995, CANCER RES, V55, P4633

Buechler C, 2000, J LEUKOCYTE BIOL, V67, P97, DOI 10.1002/jlb.67.1.97

Canning M, 2019, FRONT CELL DEV BIOL, V7, DOI 10.3389/fcell.2019.00052

Cantrell JN, 2019, MAYO CLIN PROC, V94, P1278, DOI 10.1016/j.mayocp.2018.11.031

Carson BS, 2002, J NEURO-ONCOL, V60, P151, DOI 10.1023/A:1020626419269

Charles NA, 2012, GLIA, V60, P502, DOI 10.1002/glia.21264

Chávez-Galán L, 2015, FRONT IMMUNOL, V6, DOI 10.3389/fimmu.2015.00263

Chen ZT, 2017, CANCERS, V9, DOI 10.3390/cancers9060061

Cheng X, 2020, CELL METAB, V32, P229, DOI 10.1016/j.cmet.2020.06.002

Cheng XQ, 2014, PLOS ONE, V9, DOI 10.1371/journal.pone.0098652

Conway GE, 2019, SCI REP-UK, V9, DOI 10.1038/s41598-019-49013-3

Daeschlein G, 2018, IEEE T RADIAT PLASMA, V2, P153, DOI 10.1109/TRPMS.2018.2789659

Dokic I, 2012, GLIA, V60, P1785, DOI 10.1002/glia.22397

Dubuc A, 2018, THER ADV MED ONCOL, V10, P1, DOI 10.1177/1758835918786475

Gentry JJ, 2004, PROG BRAIN RES, V146, P25, DOI 10.1016/S0079-6123(03)46002-0

Gjika E, 2020, SCI REP-UK, V10, DOI 10.1038/s41598-020-73457-7

Goswami S, 1998, J NEUROCHEM, V71, P1837

Guo G, 2019, NEURO-ONCOLOGY, V21, P1529, DOI 10.1093/neuonc/noz127

Guo G, 2017, NAT NEUROSCI, V20, P1074, DOI 10.1038/nn.4584

Hänsch MAC, 2015, J PHYS D APPL PHYS, V48, DOI 10.1088/0022-3727/48/45/454001

Ishaq M, 2014, MOL BIOL CELL, V25, P1523, DOI 10.1091/mbc.E13-10-0590

Kore RA, 2014, BIOCHEM BIOPH RES CO, V453, P326, DOI 10.1016/j.bbrc.2014.09.068

Krakstad C, 2010, MOL CANCER, V9, DOI 10.1186/1476-4598-9-135

Lafontaine J, 2020, CANCERS, V12, DOI 10.3390/cancers12020348

Lailler C, 2019, BIOSCIENCE REP, V39, DOI 10.1042/BSR20191433

Lisi L, 2017, NEUROSCI LETT, V645, P106, DOI 10.1016/j.neulet.2017.02.076

Lu Y, 2011, ONCOL REP, V25, P1329, DOI 10.3892/or.2011.1195

Manaloto E, 2020, ARCH BIOCHEM BIOPHYS, V689, DOI 10.1016/j.abb.2020.108462

Markovic DS, 2005, J NEUROPATH EXP NEUR, V64, P754, DOI 10.1097/01.jnen.0000178445.33972.a9

Metelmann HR, 2018, CLIN PLASMA MED, V9, P6, DOI 10.1016/j.cpme.2017.09.001

Noch EK, 2018, WORLD NEUROSURG, V116, P505, DOI 10.1016/j.wneu.2018.04.022

Osuka S, 2017, J CLIN INVEST, V127, P415, DOI 10.1172/JCI89587

Park JC, 2018, J KOREAN NEUROSURG S, V61, P441, DOI 10.3340/jkns.2017.0219

Pasqual-Melo G, 2020, INT J MOL SCI, V21, DOI 10.3390/ijms21041379

Privat-Maldonado A, 2019, OXID MED CELL LONGEV, V2019, DOI 10.1155/2019/9062098

Privat-Maldonado A, 2018, CANCERS, V10, DOI 10.3390/cancers10110394

Reuter S, 2018, J PHYS D APPL PHYS, V51, DOI 10.1088/1361-6463/aab3ad

Saadati F, 2021, INT J MOL SCI, V22, DOI 10.3390/ijms222111446

Schiffer D, 2019, CANCERS, V11, DOI 10.3390/cancers11010005

Schwamborn J, 2003, BMC GENOMICS, V4, DOI 10.1186/1471-2164-4-46

Segherlou ZH, 2021, J CELL PHYSIOL, V236, P5564, DOI 10.1002/jcp.30289

Sharma I, 2018, J BIOMED SCI, V25, DOI 10.1186/s12929-018-0464-y

Sies H, 2015, REDOX BIOL, V4, P180, DOI 10.1016/j.redox.2015.01.002

Siu A, 2015, PLOS ONE, V10, DOI 10.1371/journal.pone.0126313

Soni V, 2021, CANCERS, V13, DOI 10.3390/cancers13174485

Southwell DG, 2018, J NEUROSURG, V129, P567, DOI 10.3171/2017.5.JNS17166

Strik H, 1999, J NEUROL NEUROSUR PS, V67, P763, DOI 10.1136/jnnp.67.6.763

Tanaka H, 2019, SCI REP-UK, V9, DOI 10.1038/s41598-019-50136-w

Tchirkov A, 2001, BRIT J CANCER, V85, P518, DOI 10.1054/bjoc.2001.1942

Toler Steven M, 2006, Neurosurg Focus, V21, pE10

Vandamme M, 2012, INT J CANCER, V130, P2185, DOI 10.1002/ijc.26252

Vera C, 2014, J OVARIAN RES, V7, DOI 10.1186/s13048-014-0082-6

Wang QR, 2018, NAT COMMUN, V9, DOI 10.1038/s41467-018-03050-0

Wang XQ, 2016, ONCOTARGET, V7, P2354, DOI 10.18632/oncotarget.6221

Warta R, 2017, NAT NEUROSCI, V20, P1035, DOI 10.1038/nn.4605

Wen PY, 2008, NEW ENGL J MED, V359, P492, DOI 10.1056/NEJMra0708126

Wende K, 2014, PLASMA PROCESS POLYM, V11, P655, DOI 10.1002/ppap.201300172

Xiao AY, 2019, REDOX BIOL, V26, DOI 10.1016/j.redox.2019.101220

Yan DY, 2020, SCI REP-UK, V10, DOI 10.1038/s41598-020-68585-z

Ye XZ, 2012, J IMMUNOL, V189, P444, DOI 10.4049/jimmunol.1103248

Zhu VF, 2012, CANCER LETT, V316, P139, DOI 10.1016/j.canlet.2011.11.001

NR 79

TC 10

Z9 10

U1 1

U2 7

PU MDPI

PI BASEL

PA ST ALBAN-ANLAGE 66, CH-4052 BASEL, SWITZERLAND

EI 2072-6694

J9 CANCERS

JI Cancers

PD FEB

PY 2022

VL 14

IS 3

AR 813

DI 10.3390/cancers14030813

PG 17

WC Oncology

WE Science Citation Index Expanded (SCI-EXPANDED)

SC Oncology

GA ZC1TI

UT WOS:000757310800001

PM 35159079

OA Green Published, gold

DA 2025-04-09

ER

PT J

AU Saha, A

Mondal, I

Kumari, A

Sonkar, AK

Mishra, R

Kulshreshtha, R

Patra, AK

AF Saha, Abhijit

Mondal, Indranil

Kumari, Anuj

Sonkar, Avinash Kumar

Mishra, Ramranjan

Kulshreshtha, Ritu

Patra, Ashis K.

TI Hyphenation of lipophilic ruthenium(ii)-diphosphine core with

5-fluorouracil: an effective metallodrug against glioblastoma brain

cancer cells

SO DALTON TRANSACTIONS

LA English

DT Article

ID POLYPYRIDYL COMPLEXES; OXIDATIVE STRESS; ANTICANCER; DRUG; RU

AB Glioblastoma multiforme (GBM) is the most common highly aggressive malignant brain tumor, with a very limited chance for survival post-diagnosis and post-treatment. Despite significant advancement in GBM genomics implicated in molecularly targeted chemotherapies, the prognosis remains poor and requires new drug discovery approaches. We used fluoropyrimidine 5-fluorouracil (5-FU), an antimetabolite anticancer drug conjugated or 'caged' within a lipophilic Ru(II)-diphosphine (dppe) core formulated as [Ru-II(dppe)(2)(5-FU)]PF6 (Ru-DPPE-5FU), where dppe = 1,2-bis(diphenylphosphino)ethane, and evaluated its in vitro cytotoxicity in depth with aggressive GBM cells (LN229). The hydrophilic nature of 5-FU limits its passage through the blood-brain barrier (BBB), which prevents its effective accumulation and efficacy for GBM tumors. Herein, we attempted to modulate the lipophilicity of 5-FU by inserting it within a well-designed lipophilic {Ru(dppe)(2)}-core with anticipated higher efficiency towards GBM. The physicochemical properties of [Ru-II(dppe)(2)(5-FU)]PF6 (Ru-DPPE-5FU) were studied using various spectroscopic and analytical techniques. The molecular structure was determined using X-ray crystallography, showing a distorted {RuP4NO} octahedral geometry with bidentate (N, O) binding of 5-FU and its aromatization in the Ru(II)-bound form. The P-31-NMR spectra of Ru-DPPE-5FU showed four closely spaced distinct P-31-signals, indicating four unique chemical environments around P, and the strong coupling constants between them make it a second-order spectrum. The Ru-II/Ru-III redox potential in Ru-DPPE-5FU shifted by similar to 0.91 V towards the anodic region as compared to its precursor complex cis-[Ru(dppe)(2)Cl-2] (Ru-DPPE-Cl). DFT-based theoretical calculations have been performed to correlate the experimental electronic absorption spectra and redox behaviours of the complexes. The electrostatic potential (ESP) plots indicate the delocalization of the charge density on the O-/F-atom from the 5-FU ligand towards Ru(II) upon its complexation. The antioxidant properties of all the compounds were quantified by a 2,2-diphenyl-1-picrylhydrazyl (DPPH) radical scavenging assay. The hyphenation of the 5-fluorouracil (5-FU) ligand to the lipophilic {Ru(dppe)(2)}-core endowed lipophilicity to Ru-DPPE-5FU with higher in vitro cytotoxicity (IC50 = 2.37 mu M) against the LN229 GBM cells as compared to the hydrophilic 5-FU, suggesting efficient cellular uptake. Further biological assays indicated that the complex is highly potent in inhibiting significant proliferation and spheroid formation and restricting the migratory potentials of the GBM cells. Increased caspase 3/7 activity and the presence of apoptotic bodies at the center of 3-D GBM spheroids as revealed by AO/EB dual staining indicated a deeper penetration of the lipophilic complex. The Ru-DPPE-5FU complex displayed lower cytotoxicity in HaCaT normal cells (IC50 = 7.27 mu M) in comparison to LN229 cancer cells with a selectivity index (S.I.) of >= 3. Overall, the synergism and caging of 5-FU within the hydrophobic {Ru(dppe)(2)}-core improves the pharmacokinetic profile of Ru-DPPE-5FU as a potent anticancer agent for glioblastoma.

C1 [Saha, Abhijit; Sonkar, Avinash Kumar; Mishra, Ramranjan; Patra, Ashis K.] Indian Inst Technol Kanpur, Dept Chem, Kanpur 208016, Uttar Pradesh, India.

[Mondal, Indranil; Kumari, Anuj; Kulshreshtha, Ritu] Indian Inst Technol Delhi, Dept Biochem Engn & Biotechnol, New Delhi 110016, India.

C3 Indian Institute of Technology System (IIT System); Indian Institute of

Technology (IIT) - Kanpur; Indian Institute of Technology System (IIT

System); Indian Institute of Technology (IIT) - Delhi

RP Patra, AK (corresponding author), Indian Inst Technol Kanpur, Dept Chem, Kanpur 208016, Uttar Pradesh, India.

EM akpatra@iitk.ac.in

RI Mondal, Indranil/LRU-0908-2024

OI Kulshreshtha, Ritu/0000-0003-0488-5048; Saha,

Abhijit/0000-0003-4838-902X

FU Ministry of Human Resource Development; Ministry of Human Resource and

Development (MHRD) [01(3048)/21/EMR-II]; Council of Scientific and

Industrial Research (CSIR) [2020-2677]; Indian Council of Medical

Research (ICMR) [CRG/2020/004640]; Science and Engineering Research

Board (SERB), Government of India [DBT/2020/IIT-D/1484, HPC-2013];

Department of Biotechnology (DBT)

FX A. S. is grateful to Ministry of Human Resource and Development (MHRD)

for the fellowship, R. M. thanks the Council of Scientific and

Industrial Research (CSIR), A. K. P. acknowledge Council of Scientific

and Industrial Research (CSIR) for financial support (No.

01(3048)/21/EMR-II) and Indian Council of Medical Research (ICMR)

(Project No. 2020-2677), R. K. thanks the Science and Engineering

Research Board (SERB), Government of India for the financial support

(CRG/2020/004640). I. M. is thankful to Ministry of Human Resource and

Development (MHRD), Govt. of India for the award of Senior Research

Fellowship. A. K. acknowledges the Department of Biotechnology (DBT),

Govt. of India for the award of Junior Research Fellowship

(DBT/2020/IIT-D/1484), and A. S. is thankful to Dr Sharad K. Sachan,

Dept. of Chemistry, IITK for the suggestions in crystal structure

analysis, and we also acknowledged Computer Centre IIT Kanpur for

providing High Performance Computing (HPC-2013) facility for the DFT

calculations.

CR Alessio E, 2017, EUR J INORG CHEM, P1549, DOI 10.1002/ejic.201600986

Allardyce CS, 2016, DALTON T, V45, P3201, DOI 10.1039/c5dt03919c

ANDRAE D, 1990, THEOR CHIM ACTA, V77, P123, DOI 10.1007/BF01114537

[Anonymous], 2003, SMART SAINT SOFTWARE

[Anonymous], 1999, APEX2 V20124

Bai RY, 2011, TRENDS MOL MED, V17, P301, DOI 10.1016/j.molmed.2011.01.011

Baka E, 2008, J PHARMACEUT BIOMED, V46, P335, DOI 10.1016/j.jpba.2007.10.030

BAUTISTA MT, 1991, J AM CHEM SOC, V113, P4876, DOI 10.1021/ja00013a025

BLOIS MS, 1958, NATURE, V181, P1199, DOI 10.1038/1811199a0

Chen J, 2012, CELL, V149, P36, DOI 10.1016/j.cell.2012.03.009

da Silva MM, 2021, INORG CHEM, V60, P14174, DOI 10.1021/acs.inorgchem.1c01539

FALLON L, 1973, ACTA CRYSTALLOGR B, V29, P2549, DOI 10.1107/S0567740873006989

Foti MC, 2015, J AGR FOOD CHEM, V63, P8765, DOI 10.1021/acs.jafc.5b03839

Gorrini C, 2013, NAT REV DRUG DISCOV, V12, P931, DOI 10.1038/nrd4002

Guo Z., 1999, ANGEW CHEM-GER EDIT, V11, P1512

Karmakar S, 2015, DALTON T, V44, P18607, DOI 10.1039/c5dt02585k

Kenny RG, 2019, CHEM REV, V119, P1058, DOI 10.1021/acs.chemrev.8b00271

Kumar P, 2020, DALTON T, V49, P13294, DOI 10.1039/d0dt02167a

Lakkadwala S, 2018, J PHARM SCI-US, V107, P2902, DOI 10.1016/j.xphs.2018.07.020

Lau ATY, 2008, J CELL BIOCHEM, V104, P657, DOI 10.1002/jcb.21655

Li SC, 2012, CANCER CELL INT, V12, DOI 10.1186/1475-2867-12-41

Li Z. J., 2015, PLOS ONE, V10

Liu KG, 2012, INORG CHIM ACTA, V388, P78, DOI 10.1016/j.ica.2012.02.046

Mari C, 2015, CHEM SCI, V6, P2660, DOI 10.1039/c4sc03759f

Mjos KD, 2014, CHEM REV, V114, P4540, DOI 10.1021/cr400460s

Mu CH, 2018, INORG CHEM, V57, P15247, DOI 10.1021/acs.inorgchem.8b02542

Polivka J, 2017, ANTICANCER RES, V37, P21, DOI 10.21873/anticanres.11285

Poynton FE, 2017, CHEM SOC REV, V46, P7706, DOI 10.1039/c7cs00680b

Ribeiro GH, 2020, INORG CHEM, V59, P15004, DOI 10.1021/acs.inorgchem.0c01835

Saha S, 2020, J MATER CHEM B, V8, P4318, DOI 10.1039/c9tb02700a

Sayre LM, 2008, CHEM RES TOXICOL, V21, P172, DOI 10.1021/tx700210j

Sheldrick G., 2000, SHELXTL 6 14

Sheldrick G.M., 2001, SADABS: Area Detector Absorption Correction

Sheldrick GM, 2015, ACTA CRYSTALLOGR C, V71, P3, DOI [10.1107/S2053273314026370, 10.1107/S2053229614024218, 10.1107/S0108767307043930]

Silva VR, 2018, SCI REP-UK, V8, DOI 10.1038/s41598-017-18639-6

Srivastava P, 2019, POLYHEDRON, V172, P132, DOI 10.1016/j.poly.2019.04.009

Stupp R, 2005, NEW ENGL J MED, V352, P987, DOI 10.1056/NEJMoa043330

SULLIVAN BP, 1982, INORG CHEM, V21, P1037, DOI 10.1021/ic00133a033

Thota S, 2018, J MED CHEM, V61, P5805, DOI 10.1021/acs.jmedchem.7b01689

Trondl R, 2014, CHEM SCI, V5, P2925, DOI 10.1039/c3sc53243g

Valko M, 2007, INT J BIOCHEM CELL B, V39, P44, DOI 10.1016/j.biocel.2006.07.001

Vlcek A, 2007, COORDIN CHEM REV, V251, P258, DOI 10.1016/j.ccr.2006.05.021

WALKER N, 1983, ACTA CRYSTALLOGR A, V39, P158, DOI 10.1107/S0108767383000252

Wang M., 2018, Glioma, V1, P79

NR 44

TC 9

Z9 9

U1 0

U2 4

PU ROYAL SOC CHEMISTRY

PI CAMBRIDGE

PA THOMAS GRAHAM HOUSE, SCIENCE PARK, MILTON RD, CAMBRIDGE CB4 0WF, CAMBS,

ENGLAND

SN 1477-9226

EI 1477-9234

J9 DALTON T

JI Dalton Trans.

PD JAN 23

PY 2024

VL 53

IS 4

BP 1551

EP 1567

DI 10.1039/d3dt02941g

EA DEC 2023

PG 17

WC Chemistry, Inorganic & Nuclear

WE Science Citation Index Expanded (SCI-EXPANDED)

SC Chemistry

GA FN1V0

UT WOS:001134717000001

PM 38164612

OA Bronze

DA 2025-04-09

ER

PT J

AU Haron, S

Kilmister, EJ

Davis, PF

Stylli, SS

Mantamadiotis, T

Kaye, AH

Hall, SR

Tan, ST

Wickremesekera, AC

AF Haron, Simon

Kilmister, Ethan J.

Davis, Paul F.

Stylli, Stanley S.

Mantamadiotis, Theo

Kaye, Andrew H.

Hall, Sean R.

Tan, Swee T.

Wickremesekera, Agadha C.

TI The renin-angiotensin system in central nervous system tumors and

degenerative diseases

SO FRONTIERS IN BIOSCIENCE-LANDMARK

LA English

DT Review

DE Central nervous system; Renin-angiotensin system; Pluripotency;

Glioblastoma; Parkinson's disease; Cerebral organoids

ID CONVERTING ENZYME-INHIBITOR; CELL-LIKE POPULATION; (PRO)RENIN RECEPTOR;

GLIOBLASTOMA-MULTIFORME; MONOCLONAL-ANTIBODIES; PARKINSONS-DISEASE;

PRORENIN RECEPTOR; ACE-INHIBITORS; CATHEPSIN-B; MOUSE MODEL

AB Despite their differences, central nervous system (CNS) tumors and degenerative diseases share important molecular mechanisms underlying their pathologies, due to their common anatomy. Here we review the role of the renin-angiotensin system (RAS) in CNS tumors and degenerative diseases, to highlight common molecular features and examine the potential merits in repurposing drugs that inhibit the RAS, its bypass loops, and converging signaling pathways. The RAS consists of key components, including angiotensinogen, (pro)renin receptor (PRR), angiotensin-converting enzyme 1 (ACE1), angiotensin-converting enzyme 2 (ACE2), angiotensin I (ATI), angiotensin II (ATII), ATII receptor 1 (AT(1)R), ATII receptor 2 (AT(2)R) and the Mas receptor (MasR). The RAS is integral to systemic and cellular pathways that regulate blood pressure and body fluid equilibrium and cellular homeostasis. The main effector of the RAS is ATII which exerts its effect by binding to AT(1)R and AT(2)R through two competitive arms: an ACE1/ATII/AT(1)R axis, which is involved in regulat-ing oxidative stress and neuroinflammation pathways, and an ATII/AT(2)R and/or ATII/ACE2/Ang(1-7)/MasR axis that potentiates neuroprotection pathways. Alterations of these axes are associated with cellular dysfunction linked to CNS diseases. The generation of ATII is also influenced by proteases that constitute bypass loops of the RAS. These by-pass loops include cathepsins B, D and G and chymase and aminopeptidases. The RAS is also influenced by converging pathways such as the Wnt/beta-catenin pathway which sits upstream of the RAS via PRR, a key component of the RAS. We also discuss the co-expression of components of the RAS and markers of pluripotency, such as OCT4 and SOX2, in Parkinson's disease and glioblastoma, and their potential influences on transduction pathways involving the Wnt/beta-catenin, MAPK/ERK, PI3K/AKT and vacuolar (H+) adenosine triphosphatase (V-ATPase) signaling cascades. Further research investigating modulation of the ACE1/ATII/AT(1)R and ACE2/Ang(1-7)/MasR axes with RAS inhibitors may lead to novel treatment of CNS tumors and degenerative diseases. The aim of this review article is to discuss and highlight experimental and epidemiological evidence for the role of the RAS, its bypass loops and convergent signaling pathways in the pathogenesis of CNS tumors and degenerative diseases, to direct research that may lead to the development of novel therapy.

C1 [Haron, Simon; Wickremesekera, Agadha C.] Wellington Reg Hosp, Dept Neurosurg, Wellington 6242, New Zealand.

[Kilmister, Ethan J.; Davis, Paul F.; Hall, Sean R.; Tan, Swee T.; Wickremesekera, Agadha C.] Gillies McIndoe Res Inst, Wellington 6242, New Zealand.

[Stylli, Stanley S.; Mantamadiotis, Theo; Tan, Swee T.; Wickremesekera, Agadha C.] Univ Melbourne, Royal Melbourne Hosp, Dept Surg, Parkville, Vic 3050, Australia.

[Stylli, Stanley S.] Royal Melbourne Hosp, Dept Neurosurg, Parkville, Vic 3050, Australia.

[Kaye, Andrew H.] Hadassah Hebrew Univ, Dept Neurosurg, Med Ctr, IL-91120 Jerusalem, Israel.

[Tan, Swee T.] Hutt Hosp, Maxillofacial Burns Unit, Wellington Reg Plast, Lower Hutt 5040, New Zealand.

C3 Melbourne Health; Royal Melbourne Hospital; University of Melbourne;

Melbourne Health; Royal Melbourne Hospital; Hebrew University of

Jerusalem; Hadassah University Medical Center

RP Wickremesekera, AC (corresponding author), Wellington Reg Hosp, Dept Neurosurg, Wellington 6242, New Zealand.; Tan, ST; Wickremesekera, AC (corresponding author), Gillies McIndoe Res Inst, Wellington 6242, New Zealand.; Tan, ST; Wickremesekera, AC (corresponding author), Univ Melbourne, Royal Melbourne Hosp, Dept Surg, Parkville, Vic 3050, Australia.; Tan, ST (corresponding author), Hutt Hosp, Maxillofacial Burns Unit, Wellington Reg Plast, Lower Hutt 5040, New Zealand.

EM swee.tan@gmri.org.nz; agadha.wickremesekera@ccdhb.org.nz

RI stylli, stanley/R-7540-2019; Mantamadiotis, Theo/AAM-1346-2020;

Kilmister, Ethan/AAW-8060-2021

OI Australian Plastic and Reconstructive Surgery Research Network,

ASPS/0000-0002-3818-308X; Mantamadiotis, Theo/0000-0003-3971-5380

CR Abdul-Muneer PM, 2018, MOL NEUROBIOL, V55, P5901, DOI 10.1007/s12035-017-0812-z

Abiodun OA, 2020, SAUDI J BIOL SCI, V27, P905, DOI 10.1016/j.sjbs.2020.01.026

Adilijiang A, 2019, MOLECULES, V24, DOI 10.3390/molecules24173046

ALHENCGELAS F, 1989, J HYPERTENS, V7, pS9, DOI 10.1097/00004872-198909007-00003

Ameratunga M, 2018, COCHRANE DB SYST REV, DOI 10.1002/14651858.CD008218.pub4

ARIZA A, 1988, AM J CLIN PATHOL, V90, P437, DOI 10.1093/ajcp/90.4.437

Arundhathi A, 2016, ONCOTARGET, V7, P55437, DOI 10.18632/oncotarget.10583

Baltatu O, 1997, BRAIN RES, V752, P269, DOI 10.1016/S0006-8993(96)01474-6

Balyasnikova IV, 2005, PULM PHARMACOL THER, V18, P251, DOI 10.1016/j.pupt.2004.12.008

Balyasnikova IV, 2005, TISSUE ANTIGENS, V65, P240, DOI 10.1111/j.1399-0039.2005.00364.x

Bengoa-Vergniory N, 2014, STEM CELLS, V32, P3196, DOI 10.1002/stem.1807

Bennion DM, 2015, CURR HYPERTENS REP, V17, DOI 10.1007/s11906-014-0512-2

BICKERTON R, 1961, P SOC EXP BIOL MED, V106, P834

Bodiga VL, 2013, ASIAN J NEUROSCI, V2013, DOI [10.1155/2013/102602, DOI 10.1155/2013/102602]

Bradshaw A, 2016, FRONT SURG, V3, DOI 10.3389/fsurg.2016.00048

Bradshaw A, 2016, FRONT SURG, V3, DOI 10.3389/fsurg.2016.00021

Bradshaw AR, 2016, FRONT SURG, V3, DOI 10.3389/fsurg.2016.00051

Carpentier AF, 2012, EUR J NEUROL, V19, P1337, DOI 10.1111/j.1468-1331.2012.03766.x

Carvalho C, 2018, FRONT PHYSIOL, V9, DOI 10.3389/fphys.2018.00806

CASSIS LA, 1988, HYPERTENSION, V11, P591, DOI 10.1161/01.HYP.11.6.591

Chiang YY, 2014, J CLIN HYPERTENS, V16, P27, DOI 10.1111/jch.12228

Costa-Besada MA, 2018, MOL NEUROBIOL, V55, P5847, DOI 10.1007/s12035-017-0805-y

Cruciat CM, 2010, SCIENCE, V327, P459, DOI 10.1126/science.1179802

Cui CM, 2019, REDOX BIOL, V26, DOI 10.1016/j.redox.2019.101295

Dai YN, 2015, CANCER CAUSE CONTROL, V26, P1245, DOI 10.1007/s10552-015-0617-1

Danser AHJ, 2015, AM J HYPERTENS, V28, P1187, DOI 10.1093/ajh/hpv045

de Morais SDB, 2018, CURR HYPERTENS REP, V20, DOI 10.1007/s11906-018-0810-1

Dell'Italia LJ, 2018, CIRC RES, V122, P319, DOI 10.1161/CIRCRESAHA.117.310978

DESCHEPPER CF, 1986, BRAIN RES, V374, P195, DOI 10.1016/0006-8993(86)90411-7

Di Lullo E, 2017, NAT REV NEUROSCI, V18, P573, DOI 10.1038/nrn.2017.107

Di Stadio A, 2020, MULT SCLER RELAT DIS, V46, DOI 10.1016/j.msard.2020.102540

Dong YF, 2011, FASEB J, V25, P2911, DOI 10.1096/fj.11-182873

Du ZH, 2009, GLIA, V57, P724, DOI 10.1002/glia.20800

DZAU VJ, 1986, HYPERTENSION, V8, P544, DOI 10.1161/01.HYP.8.6.544

Farag E, 2017, J CLIN NEUROSCI, V46, P1, DOI 10.1016/j.jocn.2017.08.055

Featherston T, PRS-GLOB OPEN, V8, pE3042

Featherston T, 2017, FRONT MED-LAUSANNE, V4, DOI 10.3389/fmed.2017.00100

Ferone G, 2016, CANCER CELL, V30, P519, DOI 10.1016/j.ccell.2016.09.001

Ferreira R, 2011, J NEUROINFLAMM, V8, DOI 10.1186/1742-2094-8-169

Follmer C, 2020, MOVEMENT DISORD, V35, P1495, DOI 10.1002/mds.28231

Gangemi RMR, 2009, STEM CELLS, V27, P40, DOI 10.1634/stemcells.2008-0493

Gao Q, 2017, ONCOTARGET, V8, P24099, DOI 10.18632/oncotarget.15732

Garrido-Gil P, 2013, BRAIN STRUCT FUNCT, V218, P373, DOI 10.1007/s00429-012-0402-9

Garros-Regulez L, 2016, FRONT ONCOL, V6, DOI 10.3389/fonc.2016.00222

Gebre AK, 2018, FRONT PHARMACOL, V9, DOI 10.3389/fphar.2018.00440

Geller S, 2017, EUR J NEUROSCI, V46, P2596, DOI 10.1111/ejn.13732

Gheblawi M, 2020, CIRC RES, V126, P1456, DOI 10.1161/CIRCRESAHA.120.317015

Guimond Marie-Odile, 2012, Front Endocrinol (Lausanne), V3, P164, DOI 10.3389/fendo.2012.00164

Guo XL, 2017, AM J PATHOL, V187, P2876, DOI 10.1016/j.ajpath.2017.08.012

Hamming I, 2004, J PATHOL, V203, P631, DOI 10.1002/path.1570

Hanahan D, 2000, CELL, V100, P57, DOI 10.1016/S0092-8674(00)81683-9

Hanahan D, 2011, CELL, V144, P646, DOI 10.1016/j.cell.2011.02.013

Happold C, 2018, J NEURO-ONCOL, V138, P163, DOI 10.1007/s11060-018-2786-8

Harrison PJ, 2021, BRIT J PSYCHIAT, V218, P283, DOI 10.1192/bjp.2020.249

HIROSE S, 1981, J BIOL CHEM, V256, P5572

Hirose T, 2019, J CLIN INVEST, V129, P2145, DOI 10.1172/JCI79990

Houck Alexander L, 2018, Curr Aging Sci, V11, P77, DOI 10.2174/1874609811666180223154436

Humphries H, 2019, PLAST RECONSTR SURG, V144, P372, DOI 10.1097/PRS.0000000000005867

Itinteang T, 2015, FRONT SURG, V2, DOI [10.3389/fssurg.2015.00026, 10.3389/fsurg.2015.00026]

Ito M, 2002, J CLIN ENDOCR METAB, V87, P1871, DOI 10.1210/jc.87.4.1871

Iwasaki Y, 2003, NEUROL RES, V25, P301, DOI 10.1179/016164103101201382

Jackson L, 2018, INT J MOL SCI, V19, DOI 10.3390/ijms19030876

Jansen EJR, 2012, CURR PROTEIN PEPT SC, V13, P124

Januel E, 2015, EUR J NEUROL, V22, P1304, DOI 10.1111/ene.12746

Jensen B L, 1991, Ugeskr Laeger, V153, P2547

Jerabek S, 2014, BBA-GENE REGUL MECH, V1839, P138, DOI 10.1016/j.bbagrm.2013.10.001

Joglar B, 2009, J NEUROCHEM, V109, P656, DOI 10.1111/j.1471-4159.2009.05999.x

Juillerat-Jeanneret L, 2004, BRIT J CANCER, V90, P1059, DOI 10.1038/sj.bjc.6601646

Jutras I, 1999, FEBS LETT, V443, P48, DOI 10.1016/S0014-5793(98)01672-X

Kast RE, 2014, ONCOTARGET, V5, P8052, DOI 10.18632/oncotarget.2408

Kaur C, 2017, HISTOL HISTOPATHOL, V32, P879, DOI 10.14670/HH-11-881

Kawai T, 2017, PHARMACOL RES, V125, P4, DOI 10.1016/j.phrs.2017.05.008

KLICKSTEIN LB, 1982, J BIOL CHEM, V257, P5042

Koh SP, 2017, FRONT SURG, V4, DOI 10.3389/fsurg.2017.00028

Kotfis K, 2020, CRIT CARE, V24, DOI 10.1186/s13054-020-02882-x

Kouchi M, 2017, J NEUROSURG, V127, P819, DOI 10.3171/2016.9.JNS16431

Kourilsky A, 2016, J NEUROL, V263, P524, DOI 10.1007/s00415-015-8016-9

Labandeira-Garcia JL, 2017, FRONT AGING NEUROSCI, V9, DOI 10.3389/fnagi.2017.00129

Lavoie JL, 2004, HYPERTENSION, V43, P1116, DOI 10.1161/01.HYP.0000125143.73301.94

Lebouvier T, 2020, EXPERT REV NEUROTHER, V20, P175, DOI 10.1080/14737175.2020.1708195

Levin VA, 2017, J NEURO-ONCOL, V134, P325, DOI 10.1007/s11060-017-2528-3

Li NC, 2010, BMJ-BRIT MED J, V340, DOI 10.1136/bmj.b5465

Li QJ, 2014, NEURAL REGEN RES, V9, P959, DOI 10.4103/1673-5374.133140

Li Y, 2017, CELL STEM CELL, V20, P385, DOI 10.1016/j.stem.2016.11.017

Liu B, 2015, ONCOL REP, V33, P2583, DOI 10.3892/or.2015.3813

Liu H, 2017, CLIN CANCER RES, V23, P5959, DOI 10.1158/1078-0432.CCR-17-0256

Liu J, 2019, AM J PHYSIOL-LUNG C, V317, pL202, DOI 10.1152/ajplung.00295.2018

Liu TR, 2018, CANCER RES, V78, P6632, DOI 10.1158/0008-5472.CAN-18-0650

Liu Y, 2014, PLOS ONE, V9, DOI 10.1371/journal.pone.0111343

Louis DN, 2007, ACTA NEUROPATHOL, V114, P97, DOI 10.1007/s00401-007-0243-4

Makar GA, 2014, JNCI-J NATL CANCER I, V106, DOI 10.1093/jnci/djt374

Matsubara H, 1998, Nihon Rinsho, V56, P1912

Mehrotra S, 2021, LYMPHAT RES BIOL, V19, P347, DOI 10.1089/lrb.2020.0047

Mehrotra S, 2018, FRONT SURG, V5, DOI 10.3389/fsurg.2018.00040

Miyata S, 2015, FRONT NEUROSCI-SWITZ, V9, DOI 10.3389/fnins.2015.00390

Morita-Takemure S, 2019, NEUROCHEM INT, V128, P135, DOI 10.1016/j.neuint.2019.04.007

MORRIS BJ, 1978, J CLIN ENDOCR METAB, V46, P153, DOI 10.1210/jcem-46-1-153

Motawi TK, 2020, MOL CELL BIOCHEM, V465, P89, DOI 10.1007/s11010-019-03670-0

Mukerjee S, 2019, HYPERTENSION, V74, P1181, DOI 10.1161/HYPERTENSIONAHA.119.13133

Nakagawa P, 2020, CURR HYPERTENS REP, V22, DOI 10.1007/s11906-019-1011-2

Nakagawa P, 2017, HYPERTENSION, V70, P10, DOI 10.1161/HYPERTENSIONAHA.117.08550

NAKAI H, 1988, CYTOGENET CELL GENET, V47, P90, DOI 10.1159/000132515

Naseem RH, 2005, BASIC RES CARDIOL, V100, P139, DOI 10.1007/s00395-004-0499-3

Naughton SX, 2020, J ALZHEIMERS DIS, V76, P21, DOI 10.3233/JAD-200537

Neftel C, 2019, CELL, V178, P835, DOI 10.1016/j.cell.2019.06.024

Neves FAR, 1996, HYPERTENSION, V27, P514, DOI 10.1161/01.HYP.27.3.514

Ohrui T, 2004, NEUROLOGY, V63, P1324, DOI 10.1212/01.WNL.0000140705.23869.E9

Oscanoa TJ, 2020, CURR CLIN PHARMACOL

Pan Y, 2015, Z NATURFORSCH C, V70, P151, DOI 10.1515/znc-2014-4158

Pantelis C, 2021, AUST NZ J PSYCHIAT, V55, P750, DOI 10.1177/0004867420961472

Paterson C, 2019, PLAST RECONSTR SURG, V144, P1338, DOI 10.1097/PRS.0000000000006275

Paul M, 2006, PHYSIOL REV, V86, P747, DOI 10.1152/physrev.00036.2005

Ocaranza MP, 2020, NAT REV CARDIOL, V17, P116, DOI 10.1038/s41569-019-0244-8

Perez-Lloret S, 2017, EXPERT OPIN INV DRUG, V26, P1163, DOI 10.1080/13543784.2017.1371133

Pinter M, 2018, CLIN CANCER RES, V24, P3803, DOI 10.1158/1078-0432.CCR-18-0236

Rabie MA, 2018, BIOCHEM PHARMACOL, V151, P126, DOI 10.1016/j.bcp.2018.01.047

Rahman RMA, 2019, FRONT SURG, V6, DOI 10.3389/fsurg.2019.00006

Reardon KA, 2000, AUST NZ J MED, V30, P48, DOI 10.1111/j.1445-5994.2000.tb01054.x

Ren LW, 2019, CURR HYPERTENS REP, V21, DOI 10.1007/s11906-019-0937-8

Ricci-Vitiani L, 2008, CELL DEATH DIFFER, V15, P1491, DOI 10.1038/cdd.2008.72

Ricci-Vitiani L, 2010, NATURE, V468, P824, DOI 10.1038/nature09557

Riquier-Brison ADM, 2018, AM J PHYSIOL-RENAL, V315, pF521, DOI 10.1152/ajprenal.00029.2018

Rodríguez EM, 2005, INT REV CYTOL, V247, P89, DOI 10.1016/S0074-7696(05)47003-5

Rosenthal T, 2019, ANTICANCER RES, V39, P4597, DOI 10.21873/anticanres.13639

Roth IM, 2019, FRONT ONCOL, V9, DOI 10.3389/fonc.2019.00745

Rykl J, 2006, J HYPERTENS, V24, P1797, DOI 10.1097/01.hjh.0000242404.91332.be

Ryskalin L, 2019, INT J MOL SCI, V20, DOI 10.3390/ijms20153824

Scotti L, 2021, PHARMACOL RES, V166, DOI 10.1016/j.phrs.2021.105515

Seo J, 2020, CELL MOL LIFE SCI, V77, P2659, DOI 10.1007/s00018-019-03428-3

Shen J, 2016, J RENIN-ANGIO-ALDO S, V17, DOI 10.1177/1470320316656679

Siltari A, 2016, PEPTIDES, V85, P46, DOI 10.1016/j.peptides.2016.09.001

Sparks MA, 2014, COMPR PHYSIOL, V4, P1201, DOI 10.1002/cphy.c130040

Stegbauer J, 2009, P NATL ACAD SCI USA, V106, P14942, DOI 10.1073/pnas.0903602106

Stern JE, 2016, HYPERTENSION, V68, P1483, DOI 10.1161/HYPERTENSIONAHA.116.07747

Sterne JAC, 2020, JAMA-J AM MED ASSOC, V324, P1330, DOI 10.1001/jama.2020.17023

Stone RE, 2020, J NEUROIMMUNE PHARM, V15, P249, DOI 10.1007/s11481-019-09894-7

Straub RH, 2000, J NEUROCHEM, V75, P2464, DOI 10.1046/j.1471-4159.2000.0752464.x

Stupp R, 2005, NEW ENGL J MED, V352, P987, DOI 10.1056/NEJMoa043330

Takahashi K, 2006, CELL, V126, P663, DOI 10.1016/j.cell.2006.07.024

Tamarat R, 2002, LAB INVEST, V82, P747, DOI 10.1097/01.LAB.0000017372.76297.EB

Tan DCH, 2019, CELLS-BASEL, V8, DOI 10.3390/cells8111364

Tan K, 2018, PRS-GLOB OPEN, V6, DOI 10.1097/GOX.0000000000001686

Tao MX, 2018, NEUROSCI LETT, V687, P16, DOI 10.1016/j.neulet.2018.09.024

TAUGNER R, 1987, HISTOCHEMISTRY, V86, P249

Testa U, 2020, MED SCI, V6, P85

Torika N, 2016, J NEUROIMMUNE PHARM, V11, P774, DOI 10.1007/s11481-016-9703-8

van der Knaap R, 2008, CANCER-AM CANCER SOC, V112, P748, DOI 10.1002/cncr.23215

van Schaijik B, 2019, J CLIN NEUROSCI, V61, P5, DOI 10.1016/j.jocn.2018.12.019

Venkataramani V, 2019, NATURE, V573, P532, DOI 10.1038/s41586-019-1564-x

Venkatesh HS, 2019, NATURE, V573, P539, DOI 10.1038/s41586-019-1563-y

Verdecchia P, 2020, EUR J INTERN MED, V76, P14, DOI 10.1016/j.ejim.2020.04.037

Verhaak RGW, 2010, CANCER CELL, V17, P98, DOI 10.1016/j.ccr.2009.12.020

Villapol S, 2015, BRAIN, V138, DOI 10.1093/brain/awv172

Villapol S, 2015, AM J HYPERTENS, V28, P289, DOI 10.1093/ajh/hpu197

Wagner L, 2015, J NEUROCHEM, V135, P1019, DOI 10.1111/jnc.13378

Wang J, 2020, CELL COMMUN SIGNAL, V18, DOI 10.1186/s12964-020-0531-3

Wesseling P, 2018, NEUROPATH APPL NEURO, V44, P139, DOI 10.1111/nan.12432

Wiesmann M, 2017, J CEREBR BLOOD F MET, V37, P2396, DOI 10.1177/0271678X16667364

Windus LCE, 2011, CELL MOL LIFE SCI, V68, P3233, DOI 10.1007/s00018-011-0630-9

Wolf G, 2002, KIDNEY INT, V61, P1986, DOI 10.1046/j.1523-1755.2002.00365.x

Wright JW, 2013, PFLUG ARCH EUR J PHY, V465, P133, DOI 10.1007/s00424-012-1102-2

Xu JX, 2019, HYPERTENSION, V73, P1266, DOI 10.1161/HYPERTENSIONAHA.119.12832

Xu JX, 2018, CELL MOL NEUROBIOL, V38, P1235, DOI 10.1007/s10571-018-0591-8

Xu JX, 2017, CIRC RES, V121, P43, DOI 10.1161/CIRCRESAHA.116.310509

Xu QB, 2016, PHARMACOL THERAPEUT, V164, P126, DOI 10.1016/j.pharmthera.2016.04.006

Yamada K, 2010, BRAIN RES, V1352, P176, DOI 10.1016/j.brainres.2010.07.006

Yang LQ, 2020, SIGNAL TRANSDUCT TAR, V5, DOI 10.1038/s41392-020-0110-5

Yue YL, 2020, BRAIN RES, V1735, DOI 10.1016/j.brainres.2020.146726

Zhao HR, 2015, NEUROCHEM RES, V40, P854, DOI 10.1007/s11064-015-1536-y

NR 169

TC 9

Z9 9

U1 0

U2 10

PU IMR PRESS

PI ROBINSON

PA 112 ROBINSON RD, ROBINSON, SINGAPORE

SN 2768-6701

EI 2768-6698

J9 FRONT BIOSCI-LANDMRK

JI Front. Biosci.

PD SEP 30

PY 2021

VL 26

IS 9

BP 628

EP 642

DI 10.52586/4972

PG 15

WC Biochemistry & Molecular Biology; Cell Biology

WE Science Citation Index Expanded (SCI-EXPANDED)

SC Biochemistry & Molecular Biology; Cell Biology

GA WD5VR

UT WOS:000705008400019

PM 34590472

OA gold

DA 2025-04-09

ER

PT J

AU Jiang, XZ

Qiao, SY

Jiang, T

Yan, Y

Xu, Y

Wu, T

AF Jiang, Xi-Zhong

Qiao, Shi-Yu

Jiang, Tong

Yan, Ying

Xu, Ying

Wu, Tong

TI Targeting Ferroptosis to Enhance Radiosensitivity of Glioblastoma

SO PROGRESS IN BIOCHEMISTRY AND BIOPHYSICS

LA English

DT Article

DE glioblastoma, irradiation, ferroptosis, radiosensitization

ID IRON; RADIOTHERAPY; TEMOZOLOMIDE; AUTOPHAGY; ACSL4

AB Glioblastoma (GBM), one of the most common malignant tumors in the central nervous system (CNS), is characterized by diffuse and invasive growth as well as resistance to various combination therapies.GBM is the most prevalent type with the highest degree of malignancy and the worst prognosis. While current clinical treatments include surgical resection, radiotherapy, temozolomide chemotherapy, novel molecular targeted therapy, and immunotherapy, the median survival time of GBM patients is only about one year. Radiotherapy is one of the important treatment modalities for GBM, which relies on ionizing radiation to eradicate tumor cells.Approximately 60% to 70% of patients need to receive radiotherapy as postoperative radiotherapy or neoadjuvant radiotherapy during the treatment process. However, during radiotherapy, the radioresistant effect caused by DNA repair activation and cell apoptosis inhibition impedes the therapeutic effect of malignant glioblastoma.Ferroptosis was first proposed by Dr. Brent R. Stockwell in 2012. It is an iron-dependent mode of cell death induced by excessive lipid peroxidation. Although the application of ferroptosis in tumor therapy is still in the exploratory stage, it provides a completely new idea for tumor therapy as a novel form of cell death. Ferroptosis has played a significant role in the treatment of GBM. Specifically, research has revealed the key processes of ferroptosis occurrence, including intracellular iron accumulation, reactive oxygen species (ROS) generation, lipid peroxidation, and a decrease in the activity of the antioxidant system. Among them, glutathione peroxidase 4(GPX4) in the cytoplasm and mitochondria, ferroptosis suppressor protein 1(FSP1) on the plasma membrane, and dihydroorotate dehydrogenase (DHODH) in the mitochondria constitute an antioxidant protection system against ferroptosis. In iron metabolism, nuclear receptor coactivator 4(NCOA4) can mediate ferritin autophagy to regulate intracellular iron balance based on intracellular iron content. Heme oxygenase1(HMOX1) catalyzes heme degradation to release iron and regulate ferroptosis. Radiation can trigger ferroptosis by generating ROS,inhibiting the signaling axis of the antioxidant system, depleting glutathione, upregulating acyl-CoA synthase long chain family member 4(ACSL4), and inducing autophagy. Interestingly, some articles has documented that exposure to low doses of radiation (6 Gy for 24 h or 8 Gy for 4-12 h) can induce the expression of SLC7A11 and GPX4 in breast cancer and lung cancer cells, leading to radiation resistance, while radiation-induced ferroptosis occurs after 48 h. In contrast, high doses of ionizing radiation (20 Gy and 50 Gy) increase lipid peroxidation after 24 h. This suggests that radiation-induced oxidative stress is a double-edged sword that can regulate ferroptosis in both directions, and the ultimate fate of cells after radiation exposure--developing resistance and achieving homeostasis or undergoing ferroptosis--depends on the degree and duration of membrane lipid damage caused by the radiation dose. In addition,during the process of radiotherapy,methods such as inducing iron overload,damaging the antioxidant system,and disrupting mitochondrial function are used to target ferroptosis,thereby enhancing the radiosensitivity of glioblastoma. By promoting the occurrence of ferroptosis in tumor cells as a strategy to improve radiotherapy sensitivity, we can enhance the killing effect of ionizing radiation on tumor cells,thus providing more treatment options for patients with glioblastoma.

In this paper, we reviewed ferroptosis and its mechanism, analyzed the molecular mechanism of radiation-induced ferroptosis, and discussed the effective strategies to regulate ferroptosis in enhancing the sensitivity of radiotherapy, with a view to providing an important reference value for improving the current status of glioblastoma treatment

C1 [Jiang, Xi-Zhong; Qiao, Shi-Yu] China Med Univ, Grad Training Base Gen Hosp Northern Theater Comma, Shenyang 110016, Peoples R China.

[Jiang, Tong; Yan, Ying; Xu, Ying; Wu, Tong] Gen Hosp Northern Theater Command, Dept Radiat Oncol, Shenyang 110016, Peoples R China.

C3 China Medical University

RP Yan, Y (corresponding author), Gen Hosp Northern Theater Command, Dept Radiat Oncol, Shenyang 110016, Peoples R China.

EM yanyingdoctor@sina.com

RI Jiang, Xizhong/JNE-3481-2023

FX This work was supported by a grant from China Postdoctoral Science

Foundation (2023M734296)

CR Adjemian S, 2020, CELL DEATH DIS, V11, DOI 10.1038/s41419-020-03209-y

Chen X, 2021, CELL DEATH DIFFER, V28, P2843, DOI 10.1038/s41418-021-00859-z

Coates TD, 2014, FREE RADICAL BIO MED, V72, P23, DOI 10.1016/j.freeradbiomed.2014.03.039

Costa I, 2023, PHARMACOL THERAPEUT, V244, DOI 10.1016/j.pharmthera.2023.108373

Dixon SJ, 2012, CELL, V149, P1060, DOI 10.1016/j.cell.2012.03.042

Doll S, 2019, NATURE, V575, P693, DOI 10.1038/s41586-019-1707-0

Doll S, 2017, NAT CHEM BIOL, V13, P91, DOI [10.1038/NCHEMBIO.2239, 10.1038/nchembio.2239]

Florean C, 2019, FREE RADICAL BIO MED, V134, P177, DOI 10.1016/j.freeradbiomed.2019.01.008

Galluzzi L, 2018, CELL DEATH DIFFER, V25, P486, DOI 10.1038/s41418-017-0012-4

Hassannia B, 2019, CANCER CELL, V35, P830, DOI 10.1016/j.ccell.2019.04.002

Hou W, 2016, AUTOPHAGY, V12, P1425, DOI 10.1080/15548627.2016.1187366

Huang WP, 2023, INT J RADIAT BIOL, V99, P1908, DOI 10.1080/09553002.2023.2235433

Ivanov SD, 2015, B EXP BIOL MED+, V158, P800, DOI 10.1007/s10517-015-2865-1

Ivanov S. D. ., 2013, Radiatsionnaya Biologiya Radioekologiya, V53, P296, DOI 10.7868/S0869803113030065

Klionsky DJ, 2000, SCIENCE, V290, P1717, DOI 10.1126/science.290.5497.1717

Koeberle SC, 2023, MED RES REV, V43, P614, DOI 10.1002/med.21933

Kuno S, 2022, EMBO REP, V23, DOI 10.15252/embr.202154278

Lang XT, 2019, CANCER DISCOV, V9, P1673, DOI 10.1158/2159-8290.CD-19-0338

Lei G, 2020, CELL RES, V30, P146, DOI 10.1038/s41422-019-0263-3

Li DS, 2020, SIGNAL TRANSDUCT TAR, V5, DOI 10.1038/s41392-020-00216-5

Liu R, 2023, J NEUROCHEM, V167, P347, DOI 10.1111/jnc.15969

Liu R, 2022, FRONT CELL DEV BIOL, V9, DOI 10.3389/fcell.2021.772380

Liu YF, 2023, REDOX REP, V28, DOI 10.1080/13510002.2023.2260646

Mao C, 2021, NATURE, V593, P586, DOI 10.1038/s41586-021-03539-7

Murphy MP, 2009, BIOCHEM J, V417, P1, DOI 10.1042/BJ20081386

Orlando UD, 2012, PLOS ONE, V7, DOI 10.1371/journal.pone.0040794

Santivasi WL, 2014, ANTIOXID REDOX SIGN, V21, P251, DOI 10.1089/ars.2013.5668

Stupp R, 2005, NEW ENGL J MED, V352, P987, DOI 10.1056/NEJMoa043330

Su J, 2022, FRONT CELL DEV BIOL, V10, DOI 10.3389/fcell.2022.951116

Wen WW, 2023, GUT MICROBES, V15, DOI 10.1080/19490976.2023.2265578

Wu Y, 2023, MOL CANCER, V22, DOI 10.1186/s12943-023-01801-2

Xu HY, 2023, DRUG DELIV, V30, P1, DOI 10.1080/10717544.2022.2152911

Yang PF, 2023, CELL DEATH DIFFER, V30, P2432, DOI 10.1038/s41418-023-01230-0

Yang QY, 2020, LIFE SCI, V258, DOI 10.1016/j.lfs.2020.118135

Ye LF, 2020, ACS CHEM BIOL, V15, P469, DOI 10.1021/acschembio.9b00939

Yu F, 2022, CELL DISCOV, V8, DOI 10.1038/s41421-022-00390-6

Yu KX, 2023, ACS APPL MATER INTER, V15, P44689, DOI 10.1021/acsami.3c07379

Zeng L, 2023, THERANOSTICS, V13, P3725, DOI 10.7150/thno.81999

Zhang HL, 2022, NAT CELL BIOL, V24, P88, DOI 10.1038/s41556-021-00818-3

Zhang SL, 2022, FREE RADICAL BIO MED, V193, P202, DOI 10.1016/j.freeradbiomed.2022.10.004

Zhang YL, 2021, ONCOGENE, V40, P1425, DOI 10.1038/s41388-020-01622-3

Zhou BR, 2020, SEMIN CANCER BIOL, V66, P89, DOI 10.1016/j.semcancer.2019.03.002

Zhou H, 2022, REDOX BIOL, V55, DOI 10.1016/j.redox.2022.102413

Zou ZZ, 2017, APOPTOSIS, V22, P1321, DOI 10.1007/s10495-017-1424-9

NR 44

TC 0

Z9 0

U1 6

U2 10

PU CHINESE ACAD SCIENCES, INST BIOPHYSICS

PI BEIJING

PA 15 DATUN RD, CHAOYAND DISTRICT, BEIJING, 100101, PEOPLES R CHINA

SN 1000-3282

J9 PROG BIOCHEM BIOPHYS

JI Prog. Biochem. Biophys.

PD JUN

PY 2024

VL 51

IS 6

BP 1284

EP 1291

DI 10.16476/j.pibb.2023.0342

PG 8

WC Biochemistry & Molecular Biology; Biophysics

WE Science Citation Index Expanded (SCI-EXPANDED)

SC Biochemistry & Molecular Biology; Biophysics

GA XN0L6

UT WOS:001262245500006

DA 2025-04-09

ER

PT J

AU Gregory, M

Sarmento, B

Duarte, S

Martins, JA

Silva, C

Coutinho, O

Dias, A

AF Gregory, M.

Sarmento, B.

Duarte, S.

Martins, J. A.

Silva, C.

Coutinho, O.

Dias, A.

TI Curcumin loaded MPEG-PCL di-block copolymer nanoparticles protect glioma

cells from oxidative damage

SO PLANTA MEDICA

LA English

DT Meeting Abstract

CT 62nd International Congress and Annual Meeting of the

Society-of-Medicinal-Plant-and-Natural-Product-Research

CY AUG 31-SEP 04, 2014

CL Guimaraes, PORTUGAL

SP Soc Med Plant & Nat Prod Res

DE curcumin; nanoparticles; neuroprotection

ID MICELLES; DELIVERY

C1 [Gregory, M.; Duarte, S.; Coutinho, O.; Dias, A.] Univ Minho, Dept Biol, AgroBioPlant Grp, Ctr Res & Technol Agroenvironm & Biol Sci CITAB U, P-4719 Braga, Portugal.

[Sarmento, B.] Inst Super Ciencias Saude Norte, IINFACTS, CESPU, Gandra Prd, Portugal.

[Sarmento, B.] Univ Porto, INEB Inst Engn Biomed, P-4100 Oporto, Portugal.

[Martins, J. A.; Silva, C.] Univ Minho, Dept Chem, P-4710057 Braga, Portugal.

C3 Universidade do Minho; Universidade do Porto; Universidade do Minho

RI Sarmento, Bruno/J-6265-2013; Martins, jose/A-4230-2013; Silva, Carlos

Jorge Ribeiro da/A-3176-2013; Dias, Alberto/K-5834-2013; Gregory,

Marslin/H-1218-2012

OI Martins, jose/0000-0001-9323-3978; Silva, Carlos Jorge Ribeiro

da/0000-0001-6211-3295; Dias, Alberto/0000-0003-3641-3248; Gregory,

Marslin/0000-0002-2383-2382

CR Anand P, 2008, BIOCHEM PHARMACOL, V76, P1590, DOI 10.1016/j.bcp.2008.08.008

Gong CY, 2013, BIOMATERIALS, V34, P1413, DOI 10.1016/j.biomaterials.2012.10.068

Mohanty C, 2010, NANOMEDICINE-UK, V5, P433, DOI 10.2217/NNM.10.9

Rejinold NS, 2011, J COLLOID INTERF SCI, V360, P39, DOI 10.1016/j.jcis.2011.04.006

NR 4

TC 2

Z9 3

U1 0

U2 4

PU GEORG THIEME VERLAG KG

PI STUTTGART

PA RUDIGERSTR 14, D-70469 STUTTGART, GERMANY

SN 0032-0943

EI 1439-0221

J9 PLANTA MED

JI Planta Med.

PD NOV

PY 2014

VL 80

IS 16

MA P2N13

BP 1468

EP 1468

PG 1

WC Plant Sciences; Chemistry, Medicinal; Integrative & Complementary

Medicine; Pharmacology & Pharmacy

WE Science Citation Index Expanded (SCI-EXPANDED); Conference Proceedings Citation Index - Science (CPCI-S)

SC Plant Sciences; Pharmacology & Pharmacy; Integrative & Complementary

Medicine

GA AU4AB

UT WOS:000345550400359

DA 2025-04-09

ER

PT J

AU Yan, T

Zhuang, JX

He, L

AF Yan, Tao

Zhuang, Junxue

He, Lu

TI Carboxamide derivatives induce apoptosis in the U251 glioma cell line

SO ONCOLOGY LETTERS

LA English

DT Article

DE carboxamide derivatives; apoptosis; oxidative damage; glioma

ID IN-VITRO; TEMOZOLOMIDE; EXPRESSION

AB Glioma is a malignant tumor that is frequently treated using chemotherapy. The aim of the present study was to examine the antitumor activity of two novel carboxamide derivatives in glioma, and investigate the underlying mechanisms. Two previously designed and synthesized carboxamide derivatives were selected and their effects on glioma cells were evaluated. Specifically, assays to evaluate proliferation, apoptosis, oxidation, caspase-3, -8 and -9 activity, and the expression of Bcl-2 and surviving in glioma cells were conducted. The carboxamide derivatives were revealed to inhibit proliferation, as well as to induce apoptosis and oxidative damage in glioma U251 cells. In addition, the carboxamide derivatives increased the activity of caspase-3, -8 and -9, and suppressed the expression of Bcl-2 and survivin. These findings demonstrate that the carboxamide derivatives displayed antitumor activity against glioma in vitro, which may have been mediated via the induction of oxidative damage and apoptosis.

C1 [Yan, Tao] Tianjin Huanhu Hosp, Dept Pharm, Tianjin 300350, Peoples R China.

[Zhuang, Junxue] Tianjin Med Univ, Baodi Clin Coll, Tianjin Baodi Hosp, Dept Pharm, Tianjin 301800, Peoples R China.

[He, Lu] Tianjin Med Univ, Sch Basic Med Sci, Dept Anat & Histol, 22 Qixiangtai Rd, Tianjin 300070, Peoples R China.

C3 Tianjin Medical University; Tianjin Medical University

RP He, L (corresponding author), Tianjin Med Univ, Sch Basic Med Sci, Dept Anat & Histol, 22 Qixiangtai Rd, Tianjin 300070, Peoples R China.

EM helu@tmu.edu.cn

FU National Natural Science Foundation of China [81200957]

FX The present study was supported by the National Natural Science

Foundation of China (grant no. 81200957).

CR Bae IS, 2017, BIOMED REP, V6, P396, DOI 10.3892/br.2017.861

Bureta C, 2019, ONCOL REP, V41, P3404, DOI 10.3892/or.2019.7100

Chen TC, 2015, J BIOMED SCI, V22, DOI 10.1186/s12929-015-0175-6

Cioca Andreea, 2016, Asian Pac J Cancer Prev, V17, P4205

Iorio AL, 2017, ONCOTARGET, V8, P89595, DOI 10.18632/oncotarget.19875

Jiang B, 2018, J HAZARD MATER, V343, P1, DOI 10.1016/j.jhazmat.2017.09.015

Karpel-Massler G, 2016, ONCOTARGET, V7, P12791, DOI 10.18632/oncotarget.7302

Kobylinska LI, 2017, CROAT MED J, V58, P150, DOI 10.3325/cmj.2017.58.150

Li C, 2016, J NEURO-ONCOL, V130, P11, DOI 10.1007/s11060-016-2233-7

Livak KJ, 2001, METHODS, V25, P402, DOI 10.1006/meth.2001.1262

Nakayama N, 2016, J MOL NEUROSCI, V58, P297, DOI 10.1007/s12031-015-0680-9

Narayan RS, 2017, BMC CANCER, V17, DOI 10.1186/s12885-017-3193-9

Piano V, 2015, ACS CHEM BIOL, V10, P2589, DOI 10.1021/acschembio.5b00466

Sharma N, 2016, ASN NEURO, V8, DOI 10.1177/1759091416662808

Sousa F, 2018, ADV PROTEIN CHEM STR, V112, P61, DOI 10.1016/bs.apcsb.2018.03.001

Tang JQ, 2018, CANCER LETT, V423, P16, DOI 10.1016/j.canlet.2018.03.002

Tarozzi A, 2012, INT J MOL SCI, V13, P10899, DOI 10.3390/ijms130910899

Vellanki SH, 2019, ANTICANCER RES, V39, P1197, DOI 10.21873/anticanres.13230

Wang NB, 2017, BIOMED PHARMACOTHER, V90, P368, DOI 10.1016/j.biopha.2017.03.083

Xu N, 2018, J MICROBIOL, V56, P838, DOI 10.1007/s12275-018-8327-5

Yan HQ, 2011, BRIT J NUTR, V106, P1779, DOI 10.1017/S0007114511002315

Yang B, 2018, BIOMED REP, V8, P235, DOI 10.3892/br.2018.1051

Zhu Y, 2018, ONCOL LETT, V16, P1431, DOI 10.3892/ol.2018.8873

Zhu Y, 2014, ONCOL REP, V32, P431, DOI 10.3892/or.2014.3189

NR 24

TC 2

Z9 2

U1 0

U2 6

PU SPANDIDOS PUBL LTD

PI ATHENS

PA POB 18179, ATHENS, 116 10, GREECE

SN 1792-1074

EI 1792-1082

J9 ONCOL LETT

JI Oncol. Lett.

PD AUG

PY 2019

VL 18

IS 2

BP 1409

EP 1414

DI 10.3892/ol.2019.10434

PG 6

WC Oncology

WE Science Citation Index Expanded (SCI-EXPANDED)

SC Oncology

GA IL0MM

UT WOS:000476990900050

PM 31423205

OA gold, Green Published

DA 2025-04-09

ER

PT J

AU Liu, WJ

Yin, YB

Sun, JY

AF Liu, W. J.

Yin, Y. B.

Sun, J. Y.

TI Natural borneol is a novel chemosensitizer that enhances

temozolomide-induced anticancer efficiency against human glioma by

triggering mitochondrial dysfunction and reactive oxide species-mediated

oxidative damage (vol 11, pg 5429, 2018)

SO ONCOTARGETS AND THERAPY

LA English

DT Correction

CR WJ L, 2018, ONCOTARGETS THER, V11, P5429

NR 1

TC 0

Z9 0

U1 0

U2 5

PU DOVE MEDICAL PRESS LTD

PI ALBANY

PA PO BOX 300-008, ALBANY, AUCKLAND 0752, NEW ZEALAND

SN 1178-6930

J9 ONCOTARGETS THER

JI OncoTargets Ther.

PY 2018

VL 11

BP 8273

EP 8274

DI 10.2147/OTT.S193911

PG 2

WC Biotechnology & Applied Microbiology; Oncology

WE Science Citation Index Expanded (SCI-EXPANDED)

SC Biotechnology & Applied Microbiology; Oncology

GA HB7VF

UT WOS:000451288800002

OA Green Published, Green Submitted, gold

DA 2025-04-09

ER

PT J

AU Chen, W

Hao, PD

Song, QL

Feng, XT

Zhao, X

Wu, JC

Gong, ZX

Zhang, JL

Fu, XY

Wang, XJ

AF Chen, Wang

Hao, Pida

Song, Qile

Feng, Xiaotong

Zhao, Xuan

Wu, Jincheng

Gong, Zixiang

Zhang, Jinli

Fu, Xiaoyan

Wang, Xianjun

TI Methylseleninic acid inhibits human glioma growth in vitro and

in vivo by triggering ROS-dependent oxidative damage and

apoptosis

SO METABOLIC BRAIN DISEASE

LA English

DT Article

DE Selenium; Methylseleninic acid; Glioma; Reactive oxygen species;

Oxidative damage; Apoptosis

ID MITOCHONDRIAL-FUNCTION; CANCER GROWTH; HYPERGLYCEMIA; GLIOBLASTOMA;

RESISTANCE; MECHANISMS; SELENIUM; CELLS

AB Selenium-containing agents showed novel anticancer activity by triggering pro-oxidative mechanism. Studies confirmed that methylseleninic acid (MeSe) displayed broad-spectrum anti-tumor activity against kinds of human cancers. However, the anticancer effects and mechanism of MeSe against human glioma growth have not been explored yet. Herein, the present study showed that MeSeA dose-dependently inhibited U251 and U87 human glioma cells growth in vitro. Flow cytometry analysis indicated that MeSe induced significant U251 cells apoptosis with a dose-dependent manner, followed by the activation of caspase-7, caspase-9 and caspase-3. Immunofluorescence staining revealed that MeSe time-dependently caused reactive oxide species (ROS) accumulation and subsequently resulted in oxidative damage, as convinced by the increased phosphorylation level of Ser428-ATR, Ser1981-ATM, Ser15-p53 and Ser139-histone. ROS inhibition by glutathione (GSH) effectively attenuated MeSe-induced ROS generation, oxidative damage, caspase-3 activation and cytotoxicity, indicating that ROS was an upstream factor involved in MeSe-mediated anticancer mechanism in glioma. Importantly, MeSe administration in nude mice significantly inhibited glioma growth in vivo by inducing apoptosis through triggering oxidative damage. Taken together, our findings validated the possibility that MeSe as a selenium-containing can act as potential tumor chemotherapy agent for therapy of human glioma.

C1 [Chen, Wang; Zhao, Xuan; Wu, Jincheng; Gong, Zixiang; Wang, Xianjun] Peoples Hosp Linyi, Dept Neurol, Linyi 276000, Shandong, Peoples R China.

[Hao, Pida] Linyi Third Peoples Hosp, Dept Neurol, Linyi 276023, Shandong, Peoples R China.

[Song, Qile; Feng, Xiaotong; Fu, Xiaoyan] Shandong First Med Univ, Affiliated Hosp 2, Dept Neurol, Tai An 271000, Shandong, Peoples R China.

[Zhang, Jinli] Feixian Peoples Hosp, Dept Neurol, Linyi 273400, Shandong, Peoples R China.

[Fu, Xiaoyan] Shandong First Med Univ Taian, Affiliated Hosp 2, Shandong Key Lab TCM Multitarget Intervent & Dis C, Tai An 271000, Shandong, Peoples R China.

C3 Shandong First Medical University & Shandong Academy of Medical Sciences

RP Wang, XJ (corresponding author), Peoples Hosp Linyi, Dept Neurol, Linyi 276000, Shandong, Peoples R China.; Fu, XY (corresponding author), Shandong First Med Univ, Affiliated Hosp 2, Dept Neurol, Tai An 271000, Shandong, Peoples R China.; Zhang, JL (corresponding author), Feixian Peoples Hosp, Dept Neurol, Linyi 273400, Shandong, Peoples R China.; Fu, XY (corresponding author), Shandong First Med Univ Taian, Affiliated Hosp 2, Shandong Key Lab TCM Multitarget Intervent & Dis C, Tai An 271000, Shandong, Peoples R China.

EM cwsjnk@163.com; haopida@163.com; sqlbangong@163.com; ann2067@163.com;

zhaoxuan071@163.com; wujincheng1998@163.com; docg13455959373@163.com;

zhangjinliwsy@sina.com; txyfu66@163.com; wangxianjun008@163.com

RI Feng, Xiaotong/H-3777-2018; Fu, Xiaoyan/C-2573-2012

OI Song, Qile/0009-0006-9840-3431; Feng, Xiaotong/0009-0006-2818-8561;

wang, xianjun/0000-0002-1782-5514

FU Natural Science Foundation of Shandong

FX No Statement Available

CR Anderson SL, 2018, AM J VET RES, V79, P424, DOI 10.2460/ajvr.79.4.424

Behera C, 2022, EUR J PHARM SCI, V176, DOI 10.1016/j.ejps.2022.106238

Brenneisen Peter, 2005, Molecular Aspects of Medicine, V26, P256, DOI 10.1016/j.mam.2005.07.004

Diogo CV, 2013, INT J BIOCHEM CELL B, V45, P114, DOI 10.1016/j.biocel.2012.07.004

Hu WL, 2021, MOL CARCINOGEN, V60, P746, DOI 10.1002/mc.23340

Ip C, 2000, CANCER RES, V60, P2882

Jiang T, 2021, CANCER LETT, V499, P60, DOI 10.1016/j.canlet.2020.10.050

Lipinski B, 2019, MINI-REV MED CHEM, V19, P720, DOI 10.2174/1389557517666161104125022

Liu CA, 2018, INT J MOL SCI, V19, DOI 10.3390/ijms19041115

Manea SA, 2018, REDOX BIOL, V16, P332, DOI 10.1016/j.redox.2018.03.011

Messaoudi K, 2015, DRUG DISCOV TODAY, V20, P899, DOI 10.1016/j.drudis.2015.02.011

Miller KD, 2021, CA-CANCER J CLIN, V71, P381, DOI 10.3322/caac.21693

Okuno T, 2014, BIOL PHARM BULL, V37, P1831, DOI 10.1248/bpb.b14-00453

Ostrom QT, 2021, NEURO-ONCOLOGY, V23, P1, DOI 10.1093/neuonc/noab200

Ou A, 2021, INT J MOL SCI, V22, DOI 10.3390/ijms22010351

Palmeira CM, 2007, TOXICOL APPL PHARM, V225, P214, DOI 10.1016/j.taap.2007.07.015

Qiu CW, 2019, REPROD SCI, V26, P829, DOI 10.1177/1933719118815582

Rayman MP, 2012, LANCET, V379, P1256, DOI 10.1016/S0140-6736(11)61452-9

Rolo AP, 2006, TOXICOL APPL PHARM, V212, P167, DOI 10.1016/j.taap.2006.01.003

Sampson JH, 2020, NAT REV CANCER, V20, P12, DOI 10.1038/s41568-019-0224-7

Spallholz JE, 2004, BIOCHEM PHARMACOL, V67, P547, DOI 10.1016/j.bcp.2003.09.004

Tarrado-Castellarnau M, 2015, PHARMACOL RES, V102, P218, DOI 10.1016/j.phrs.2015.09.009

Varlamova EG, 2021, INT J MOL SCI, V22, DOI 10.3390/ijms22126614

Wang L, 2014, NUTR CANCER, V66, P295, DOI 10.1080/01635581.2014.868911

Wang XJ, 2018, CELL DEATH DISCOV, V4, DOI 10.1038/s41420-018-0114-x

Yang KY, 2022, MOL CANCER, V21, DOI 10.1186/s12943-022-01513-z

Zhu ZL, 2018, J NEUROCHEM, V144, P93, DOI 10.1111/jnc.14250

NR 27

TC 1

Z9 1

U1 2

U2 4

PU SPRINGER/PLENUM PUBLISHERS

PI NEW YORK

PA 233 SPRING ST, NEW YORK, NY 10013 USA

SN 0885-7490

EI 1573-7365

J9 METAB BRAIN DIS

JI Metab. Brain Dis.

PD APR

PY 2024

VL 39

IS 4

BP 625

EP 633

DI 10.1007/s11011-024-01344-5

EA FEB 2024

PG 9

WC Endocrinology & Metabolism; Neurosciences

WE Science Citation Index Expanded (SCI-EXPANDED)

SC Endocrinology & Metabolism; Neurosciences & Neurology

GA OG0N8

UT WOS:001173105600001

PM 38416338

DA 2025-04-09

ER

PT J

AU Marslin, G

Franklin, G

Sarmento, B

Dias, AC

AF Marslin, G.

Franklin, G.

Sarmento, B.

Dias, A. C.

TI Withania somnifera leaf extract delivery as a nanoparticle

protect the glioma cells from oxidative damage

SO PLANTA MEDICA

LA English

DT Meeting Abstract

CT 63rd International Congress and Annual Meeting of the

Society-for-Medicinal-Plant-and-Natural-Product-Research (GA)

CY AUG 23-27, 2015

CL Budapest, HUNGARY

C1 [Marslin, G.; Franklin, G.; Dias, A. C.] Univ Minho, Dept Biol, Ctr Res & Technol Agroenvironm & Biol Sci CITAB U, AgroBioPlant Grp, Braga, Portugal.

[Sarmento, B.] Inst Super Ciencias Saude Norte, Gandra PRD, CESPU, IINFACTS Inst Invest & Formacao Avancada Ciencias, Gandra, Portugal.

[Sarmento, B.] Univ Porto, INEB Inst Engn Biomed, P-4100 Oporto, Portugal.

C3 Universidade do Minho; Universidade do Porto

RI Sarmento, Bruno/J-6265-2013; Gregory, Marslin/H-1218-2012; Gregory,

Franklin/AAM-6132-2020; Dias, Alberto/K-5834-2013

OI Dias, Alberto/0000-0003-3641-3248

NR 0

TC 2

Z9 2

U1 0

U2 2

PU GEORG THIEME VERLAG KG

PI STUTTGART

PA RUDIGERSTR 14, D-70469 STUTTGART, GERMANY

SN 0032-0943

EI 1439-0221

J9 PLANTA MED

JI Planta Med.

PD NOV

PY 2015

VL 81

IS 16

MA PW-131

BP 1528

EP 1529

PG 2

WC Plant Sciences; Chemistry, Medicinal; Integrative & Complementary

Medicine; Pharmacology & Pharmacy

WE Science Citation Index Expanded (SCI-EXPANDED); Conference Proceedings Citation Index - Science (CPCI-S)

SC Plant Sciences; Pharmacology & Pharmacy; Integrative & Complementary

Medicine

GA DA1LU

UT WOS:000367558100480

DA 2025-04-09

ER

PT J

AU Zhong, S

Xue, J

Cao, JJ

Sun, BM

Sun, QF

Bian, LG

Hu, LY

Pan, SJ

AF Zhong, Shan

Xue, Jun

Cao, Jiao-Jiao

Sun, Bomin

Sun, Qing-Fang

Bian, Liu-Guan

Hu, Liang-Yun

Pan, Si-Jian

TI The therapeutic value of XL388 in human glioma cells

SO AGING-US

LA English

DT Article

DE glioma; mTOR; Akt; XL388; MAFG

ID MAMMALIAN TARGET; MTOR INHIBITORS; PHASE-II; CANCER; PATHWAY; GROWTH;

PREVENTION; RAPAMYCIN; CCI-779; COMPLEX

AB XL388 is a highly efficient and orally-available ATP-competitive PI3K-mTOR dual inhibitor. Its activity against glioma cells was studied here. In established and primary human glioma cells, XL388 potently inhibited cell survival and proliferation as well as cell migration, invasion and cell cycle progression. The dual inhibitor induced significant apoptosis activation in glioma cells. In A172 cells and primary human glioma cells, XL388 inhibited Akt-mTORC1/2 activation by blocking phosphorylation of Akt and S6K1. XL388-induced glioma cell death was only partially attenuated by a constitutively-active mutant Akt1. Furthermore, it was cytotoxic against Akt1-knockout A172 glioma cells. XL388 downregulated MAF bZIP transcription factor G (MAFG) and inhibited Nrf2 signaling, causing oxidative injury in glioma cells. Conversely, antioxidants, n-acetylcysteine, pyrrolidine dithiocarbamate and AGI-106, alleviated XL388-induced cytotoxicity and apoptosis in glioma cells. Oral administration of XL388 inhibited subcutaneous A172 xenograft growth in severe combined immunodeficient mice. Akt-S6K1 inhibition and MAFG downregulation were detected in XL388-treated A172 xenograft tissues. Collectively, XL388 efficiently inhibits human glioma cell growth, through Akt-mTORdependent and-independent mechanisms.

C1 [Zhong, Shan; Xue, Jun; Sun, Qing-Fang; Bian, Liu-Guan; Pan, Si-Jian] Shanghai Jiao Tong Univ, Sch Med, Rui Jin Hosp, Dept Neurosurg, Shanghai, Peoples R China.

[Cao, Jiao-Jiao; Sun, Bomin; Hu, Liang-Yun] Shanghai Jiao Tong Univ, Sch Med, Rui Jin Hosp, Dept Stereotact & Funct Neurosurg, Shanghai, Peoples R China.

C3 Shanghai Jiao Tong University; Shanghai Jiao Tong University

RP Pan, SJ (corresponding author), Shanghai Jiao Tong Univ, Sch Med, Rui Jin Hosp, Dept Neurosurg, Shanghai, Peoples R China.; Hu, LY (corresponding author), Shanghai Jiao Tong Univ, Sch Med, Rui Jin Hosp, Dept Stereotact & Funct Neurosurg, Shanghai, Peoples R China.

EM hly40829@rjh.com.cn; psj11629@rjh.com.cn

RI zhong, shan/HZJ-4647-2023

FU Fund of Shanghai Municipal Health Bureau [201640210]; Fund of Shanghai

Charitable Cancer Research Center; Medical Cross Foundation of Shanghai

Jiao-Tong University [YG2016MS59]

FX This project was supported by the the Fund of Shanghai Municipal Health

Bureau (201640210) to S.-J.P, and the Fund of Shanghai Charitable Cancer

Research Center to S.-J.P, and the Medical Cross Foundation of Shanghai

Jiao-Tong University (YG2016MS59) to S.-J.P. The funders had no role in

study design, data collection and analysis, decision to publish, or

preparation of the manuscript.

CR [Anonymous], 2016, OXID MED CELL LONGEV, DOI DOI 10.1155/2016/1958174

Brooks MM, 2013, MOL VIS, V19, P1406

Brunn GJ, 1996, EMBO J, V15, P5256, DOI 10.1002/j.1460-2075.1996.tb00911.x

Celeghini ECC, 2021, ANIM BIOTECHNOL, V32, P77, DOI 10.1080/10495398.2019.1654485

Chang SM, 2005, INVEST NEW DRUG, V23, P357, DOI 10.1007/s10637-005-1444-0

Chen P, 2019, AGING-US, V11, P7339, DOI 10.18632/aging.101733

Cheng FY, 2019, AGING-US, V11, P9875, DOI 10.18632/aging.102437

Choi S, 2020, NEUROSCI LETT, V718, DOI 10.1016/j.neulet.2020.134742

Crunkhorn S, 2012, NAT REV DRUG DISCOV, V11, P96, DOI 10.1038/nrd3655

Cui YW, 2016, PLOS ONE, V11, DOI 10.1371/journal.pone.0161780

Doherty L, 2006, NEUROLOGY, V67, P156, DOI 10.1212/01.wnl.0000223844.77636.29

Fan QW, 2011, CURR TOP MICROBIOL, V347, P279, DOI 10.1007/82_2010_67

Fang MG, 2014, MOL CELL, V55, P904, DOI 10.1016/j.molcel.2014.08.010

Franks AM, 2006, ANN PHARMACOTHER, V40, P66, DOI 10.1345/aph.1G142

Galanis E, 2005, J CLIN ONCOL, V23, P5294, DOI 10.1200/JCO.2005.23.622

Guertin DA, 2007, CANCER CELL, V12, P9, DOI 10.1016/j.ccr.2007.05.008

He XY, 2013, BIOCHEM BIOPH RES CO, V435, P397, DOI 10.1016/j.bbrc.2013.04.099

Huang TT, 2009, NEUROTHERAPEUTICS, V6, P500, DOI 10.1016/j.nurt.2009.04.008

Jiang H, 2016, PLOS ONE, V11, DOI 10.1371/journal.pone.0161017

Kondapaka SB, 2003, MOL CANCER THER, V2, P1093

Krajka-Kuzniak V, 2017, PHARMACOL REP, V69, P393, DOI 10.1016/j.pharep.2016.12.011

Lamming DW, 2013, J CLIN INVEST, V123, P980, DOI 10.1172/JCI64099

Laplante M, 2012, CELL, V149, P274, DOI 10.1016/j.cell.2012.03.017

Lefranc F, 2009, ADV TECH STAND NEURO, V34, P3, DOI 10.1007/978-3-211-78741-0_1

Li K, 2013, PLOS ONE, V8, DOI [10.1371/journal.pone.0084171, 10.1371/journal.pone.0068157, 10.1371/journal.pone.0057542]

Liu T, 2018, GASTROENTEROLOGY, V155, P557, DOI 10.1053/j.gastro.2018.04.032

Liu WY, 2017, NUTRIENTS, V9, DOI 10.3390/nu9121312

Ni W, 2019, AGING-US, V11, P1427, DOI 10.18632/aging.101843

Pan SJ, 2018, CANCER LETT, V412, P21, DOI 10.1016/j.canlet.2017.09.051

Pan SJ, 2015, INT J MOL SCI, V16, P5363, DOI 10.3390/ijms16035363

Pan SJ, 2015, BIOCHEM BIOPH RES CO, V458, P476, DOI 10.1016/j.bbrc.2015.01.128

Qin LS, 2015, J EXP CLIN CANC RES, V34, DOI 10.1186/s13046-015-0174-1

Sabatini DM, 2006, NAT REV CANCER, V6, P729, DOI 10.1038/nrc1974

Saxton RA, 2017, CELL, V168, P960, DOI [10.1016/j.cell.2017.02.004, 10.1016/j.cell.2017.03.035]

Siegel RL, 2020, CA-CANCER J CLIN, V70, P7, DOI 10.3322/caac.21590

Sporn MB, 2012, NAT REV CANCER, V12, P564, DOI 10.1038/nrc3278

Sun XF, 2016, HEPATOLOGY, V63, P173, DOI 10.1002/hep.28251

Sung HK, 2019, SCI REP-UK, V9, DOI 10.1038/s41598-019-41111-6

Takeuchi CS, 2013, J MED CHEM, V56, P2218, DOI 10.1021/jm3007933

Taki-Nakano N, 2014, BBA-GEN SUBJECTS, V1840, P3413, DOI 10.1016/j.bbagen.2014.09.003

Tardif JC, 2003, AM J CARDIOL, V91, p41A

Vera-Puente O, 2018, TRANSL RES, V200, P1, DOI 10.1016/j.trsl.2018.06.005

Wang HY, 2019, AGING-US, V11, P11329, DOI 10.18632/aging.102531

Wang SS, 2019, CANCER LETT, V443, P13, DOI 10.1016/j.canlet.2018.11.028

Wen PY, 2016, NAT REV NEUROL, V12, P69, DOI 10.1038/nrneurol.2015.242

Wen PY, 2012, NEURO-ONCOLOGY, V14, P819, DOI 10.1093/neuonc/nos117

Westphal M, 2011, NAT REV NEUROSCI, V12, P495, DOI 10.1038/nrn3060

Xiong ZQ, 2017, ONCOTARGET, V8, P30151, DOI 10.18632/oncotarget.15620

Xu XZ, 2019, FREE RADICAL BIO MED, V143, P387, DOI 10.1016/j.freeradbiomed.2019.08.024

Xue J, 2019, CELL DEATH DIS, V10, DOI 10.1038/s41419-019-2093-0

Yang L, 2015, EXP CELL RES, V332, P47, DOI 10.1016/j.yexcr.2014.12.017

Zhang D, 2016, TUMOR BIOL, V37, P1327, DOI 10.1007/s13277-015-3922-0

Zhao HF, 2017, MOL CANCER, V16, DOI 10.1186/s12943-017-0670-3

Zhou LN, 2019, AGING-US, V11, P11136, DOI 10.18632/aging.102515

Zhu JL, 2019, MOL CELL BIOCHEM, V461, P81, DOI 10.1007/s11010-019-03592-x

Zhu YR, 2016, ONCOTARGET, V7, P49527, DOI 10.18632/oncotarget.10389

NR 56

TC 4

Z9 4

U1 0

U2 8

PU IMPACT JOURNALS LLC

PI ORCHARD PARK

PA 6666 E QUAKER ST, STE 1, ORCHARD PARK, NY 14127 USA

SN 1945-4589

J9 AGING-US

JI Aging-US

PD NOV 30

PY 2020

VL 12

IS 22

BP 22550

EP 22563

PG 14

WC Cell Biology; Geriatrics & Gerontology

WE Science Citation Index Expanded (SCI-EXPANDED)

SC Cell Biology; Geriatrics & Gerontology

GA PA8UY

UT WOS:000595905600016

PM 33159013

OA Green Published, gold

DA 2025-04-09

ER

PT J

AU Fang, Y

Zhang, Z

AF Fang, Yi

Zhang, Zhen

TI Arsenic trioxide as a novel anti-glioma drug: a review

SO CELLULAR & MOLECULAR BIOLOGY LETTERS

LA English

DT Review

DE Arsenic trioxide; Glioma; Anti-cancer mechanism

ID AUTOPHAGIC CELL-DEATH; PHASE-II TRIAL; ACUTE PROMYELOCYTIC LEUKEMIA;

MALIGNANT GLIOMA-CELLS; TRANS-RETINOIC ACID; INDUCED APOPTOSIS;

RADIATION-THERAPY; DOWN-REGULATION; HEPATOCELLULAR-CARCINOMA;

GENE-EXPRESSION

AB Arsenic trioxide has shown a strong anti-tumor effect with little toxicity when used in the treatment of acute promyelocytic leukemia (APL). An effect on glioma has also been shown. Its mechanisms include regulation of apoptosis and autophagy; promotion of the intracellular production of reactive oxygen species, causing oxidative damage; and inhibition of tumor stem cells. However, glioma cells and tissues from other sources show different responses to arsenic trioxide. Researchers are working to enhance its efficacy in anti-glioma treatments and reducing any adverse reactions. Here, we review recent research on the efficacy and mechanisms of action of arsenic trioxide in the treatment of gliomas to provide guidance for future studies.

C1 [Fang, Yi; Zhang, Zhen] China Med Univ, Dept Ultrasound, Affiliated Hosp 1, Shenyang 110001, Liaoning, Peoples R China.

C3 China Medical University

RP Zhang, Z (corresponding author), China Med Univ, Dept Ultrasound, Affiliated Hosp 1, Shenyang 110001, Liaoning, Peoples R China.

EM 2662898158@qq.com

OI yi, fang/0000-0003-0329-5273

FU National Natural Science Foundation of China [81971639]

FX This work was supported by the National Natural Science Foundation of

China (Grant No. 81971639).

CR Alamolhodaei NS, 2015, ENVIRON TOXICOL PHAR, V40, P1005, DOI 10.1016/j.etap.2015.08.030

Ardalan B, 2010, CLIN CANCER RES, V16, P3019, DOI 10.1158/1078-0432.CCR-09-2590

Becker KP, 2012, CANCER J, V18, P12, DOI 10.1097/PPO.0b013e318244d7eb

Beer TM, 2006, CANCER-AM CANCER SOC, V106, P2624, DOI 10.1002/cncr.21925

Bell JB, 2018, MOL CANCER RES, V16, P32, DOI 10.1158/1541-7786.MCR-17-0397

Beurel E, 2006, PROG NEUROBIOL, V79, P173, DOI 10.1016/j.pneurobio.2006.07.006

Bureta C, 2019, ONCOL REP, V41, P3404, DOI 10.3892/or.2019.7100

Bursch W, 2000, J CELL SCI, V113, P1189

Bursch W, 2000, ANN NY ACAD SCI, V926, P1, DOI 10.1111/j.1749-6632.2000.tb05594.x

Cao KM, 2018, METALLOMICS, V10, P1564, DOI 10.1039/c8mt00202a

Chen JD, 2016, CSH PERSPECT MED, V6, DOI 10.1101/cshperspect.a026104

Cheng TJ, 2007, FOOD CHEM TOXICOL, V45, P1027, DOI 10.1016/j.fct.2006.12.014

Cheng Y, 2009, AUTOPHAGY, V5, P430, DOI 10.4161/auto.5.3.7896

Cheng Y, 2016, ONCOTARGET, V7, P12682, DOI 10.18632/oncotarget.7259

Chiu HW, 2011, J MOL MED, V89, P927, DOI 10.1007/s00109-011-0763-1

Chiu HW, 2009, AUTOPHAGY, V5, P472, DOI 10.4161/auto.5.4.7759

Chow SKY, 2004, J CELL BIOCHEM, V93, P173, DOI 10.1002/jcb.20102

Dalby KN, 2010, AUTOPHAGY, V6, P322, DOI 10.4161/auto.6.3.11625

Ding DC, 2014, ACTA NEUROPATHOL COM, V2, DOI 10.1186/2051-5960-2-31

Dizaji MZ, 2012, NEUROCHEM RES, V37, P370, DOI 10.1007/s11064-011-0620-1

Falnoga I, 2012, BIOL TRACE ELEM RES, V149, P331, DOI 10.1007/s12011-012-9431-8

GERLACH M, 1994, J NEUROCHEM, V63, P793, DOI 10.1046/j.1471-4159.1994.63030793.x

Grimm SA, 2012, J NEURO-ONCOL, V110, P237, DOI 10.1007/s11060-012-0957-6

Gülden M, 2017, FOOD CHEM TOXICOL, V105, P486, DOI 10.1016/j.fct.2017.04.035

Haga N, 2005, CANCER SCI, V96, P825, DOI 10.1111/j.1349-7006.2005.00114.x

Hai JJ, 2015, ANN HEMATOL, V94, P501, DOI 10.1007/s00277-014-2174-1

Hoyer-Hansen M, 2008, AUTOPHAGY, V4, P574, DOI 10.4161/auto.5921

Huilgol NG, 2006, INT J HYPERTHER, V22, P391, DOI 10.1080/02656730600722685

Imeryuz N, 2007, J HEPATOL, V47, P851, DOI 10.1016/j.jhep.2007.06.018

Jiang CC, 2007, CANCER RES, V67, P5880, DOI 10.1158/0008-5472.CAN-07-0213

Jiang JH, 2006, GLYCOBIOLOGY, V16, P1045, DOI 10.1093/glycob/cwl027

Jiao YH, 2015, J MEMBRANE BIOL, V248, P1071, DOI 10.1007/s00232-015-9818-5

Kanzawa T, 2003, CANCER RES, V63, P2103

Kanzawa T, 2005, ONCOGENE, V24, P980, DOI 10.1038/sj.onc.1208095

Karsy M, 2014, TUMOR BIOL, V35, P4567, DOI 10.1007/s13277-013-1601-6

Kim EH, 2008, CANCER RES, V68, P266, DOI 10.1158/0008-5472.CAN-07-2444

Kindler HL, 2008, AM J CLIN ONCOL-CANC, V31, P553, DOI 10.1097/COC.0b013e318178e4cd

Klauser E, 2014, FOOD CHEM TOXICOL, V67, P212, DOI 10.1016/j.fct.2014.02.039

Kumthekar P, 2017, J NEURO-ONCOL, V133, P589, DOI 10.1007/s11060-017-2469-x

Lai Yuen-Liang, 2003, Anticancer Drugs, V14, P825

Lee SJ, 2010, MOL BRAIN, V3, DOI 10.1186/1756-6606-3-30

Lew YS, 1999, CANCER RES, V59, P6033

Lew YS, 2002, CANCER RES, V62, P4202

Li YC, 2009, CANCER LETT, V284, P208, DOI 10.1016/j.canlet.2009.04.035

Lin CC, 2007, INVEST NEW DRUG, V25, P77, DOI 10.1007/s10637-006-9004-9

Lin CC, 2008, UROL ONCOL-SEMIN ORI, V26, P659, DOI 10.1016/j.urolonc.2008.02.018

Lin TH, 2008, BMC CANCER, V8, DOI 10.1186/1471-2407-8-58

Linder B, 2019, CANCERS BASEL, V11

Liu B, 2009, CANCER LETT, V275, P54, DOI 10.1016/j.canlet.2008.09.042

Liu J, 2008, J PHARMACOL EXP THER, V326, P363, DOI 10.1124/jpet.108.139543

Liu SY, 2010, TOXICOL SCI, V116, P183, DOI 10.1093/toxsci/kfq113

Liu YH, 2011, J NEURO-ONCOL, V104, P449, DOI 10.1007/s11060-010-0513-1

Lu YP, 2018, INT J NANOMED, V13, P5937, DOI 10.2147/IJN.S175418

Mesbahi Y, 2018, EUR J PHARMACOL, V820, P274, DOI 10.1016/j.ejphar.2017.12.041

Milano MT, 2010, J NEURO-ONCOL, V98, P83, DOI 10.1007/s11060-009-0054-7

Ng G, 2005, MOL CARCINOGEN, V43, P183, DOI 10.1002/mc.20097

Ning SC, 2004, INT J RADIAT ONCOL, V60, P197, DOI 10.1016/j.ijrobp.2004.02.013

Ning SC, 2006, INT J RADIAT ONCOL, V65, P493, DOI 10.1016/j.ijrobp.2005.12.015

PERILLO NL, 1995, NATURE, V378, P736, DOI 10.1038/378736a0

Podolsky L, 2011, ACTA ONCOL, V50, P602, DOI 10.3109/0284186X.2010.524934

Price Richard Lee, 2014, Neurosurgery, V61 Suppl 1, P74, DOI 10.1227/NEU.0000000000000390

Primon M, 2013, EXP CELL RES, V319, P2637, DOI 10.1016/j.yexcr.2013.08.011

SCHWEICHEL JU, 1973, TERATOLOGY, V7, P253, DOI 10.1002/tera.1420070306

Segerman A, 2016, CELL REP, V17, P2994, DOI 10.1016/j.celrep.2016.11.056

Shay JW, 2008, BRIT J CANCER, V98, P677, DOI 10.1038/sj.bjc.6604209

Shidfar F, 2016, ANTI-CANCER AGENT ME, V16, P247, DOI 10.2174/1871520615666150629100752

Soignet SL, 1998, NEW ENGL J MED, V339, P1341, DOI 10.1056/NEJM199811053391901

Song JH, 2006, J NEUROSCI, V26, P3299, DOI 10.1523/JNEUROSCI.5572-05.2006

Sun YY, 2018, CELL MOL BIOL LETT, V23, DOI 10.1186/s11658-018-0074-4

Tao JY, 2019, MOL PHARMACEUT, V16, P786, DOI 10.1021/acs.molpharmaceut.8b01056

Vuky J, 2002, INVEST NEW DRUG, V20, P327, DOI 10.1023/A:1016270206374

Wahl GM, 2001, NAT CELL BIOL, V3, pE277, DOI 10.1038/ncb1201-e277

Walker Alice M, 2016, J Cancer Sci Ther, V8, P1

Wang CL, 2014, EUR J MED RES, V19, DOI 10.1186/s40001-014-0049-5

Wang GB, 2017, EUR REV MED PHARMACO, V21, P4875

Wang J, 2015, J MOL NEUROSCI, V55, P985, DOI 10.1007/s12031-014-0455-8

Wei YY, 2008, CANCER LETT, V267, P96, DOI 10.1016/j.canlet.2008.03.019

White E, 2015, J CLIN INVEST, V125, P42, DOI 10.1172/JCI73941

Wu JN, 2013, TOXICOL LETT, V220, P61, DOI 10.1016/j.toxlet.2013.03.019

Xu YY, 2007, J CELL BIOCHEM, V100, P773, DOI 10.1002/jcb.21088

Yoshimura Y, 2015, PLOS ONE, V10, DOI 10.1371/journal.pone.0128288

Zhang XW, 2010, SCIENCE, V328, P240, DOI 10.1126/science.1183424

Zhang XY, 2012, J CELL BIOCHEM, V113, P3528, DOI 10.1002/jcb.24230

Zhao SG, 2008, MOL BIOL REP, V35, P421, DOI 10.1007/s11033-007-9102-6

Zhen YB, 2010, CANCER LETT, V292, P64, DOI 10.1016/j.canlet.2009.11.005

Zhou WC, 2015, ONCOTARGET, V6, P37300, DOI 10.18632/oncotarget.5836

Zhou X, 2008, CARCINOGENESIS, V29, P1831, DOI 10.1093/carcin/bgn063

NR 87

TC 45

Z9 48

U1 3

U2 36

PU BMC

PI LONDON

PA CAMPUS, 4 CRINAN ST, LONDON N1 9XW, ENGLAND

SN 1425-8153

EI 1689-1392

J9 CELL MOL BIOL LETT

JI Cell. Mol. Biol. Lett.

PD SEP 24

PY 2020

VL 25

IS 1

AR 44

DI 10.1186/s11658-020-00236-7

PG 13

WC Biochemistry & Molecular Biology; Cell Biology

WE Science Citation Index Expanded (SCI-EXPANDED)

SC Biochemistry & Molecular Biology; Cell Biology

GA NY1DA

UT WOS:000576137600002

PM 32983240

OA Green Published, gold

DA 2025-04-09

ER

PT J

AU Barciszewska, AM

Giel-Pietraszuk, M

Perrigue, PM

Naskret-Barciszewska, M

AF Barciszewska, Anna-Maria

Giel-Pietraszuk, Malgorzata

Perrigue, Patrick M.

Naskret-Barciszewska, Miroslawa

TI Total DNA Methylation Changes Reflect Random Oxidative DNA Damage in

Gliomas

SO CELLS

LA English

DT Article

DE 8-oxo-deoxyguanosine; 5-methylcytosine; glioma; biomarker; oxidative

damage

ID CENTRAL-NERVOUS-SYSTEM; FREE-RADICALS; STRESS; ROS; CLASSIFICATION;

8-OXOGUANINE; 8-OXO-7,8-DIHYDROGUANINE; 5-METHYLCYTOSINE; MECHANISMS;

EXPRESSION

AB DNA modifications can be used to monitor pathological processes. We have previously shown that estimating the amount of the main DNA epigenetic mark, 5-methylcytosine (m(5)C), is an efficient and reliable way to diagnose brain tumors, hypertension, and other diseases. Abnormal increases of reactive oxygen species (ROS) are a driving factor for mutations that lead to changes in m(5)C levels and cancer evolution. 8-oxo-deoxyguanosine (8-oxo-dG) is a specific marker of ROS-driven DNA-damage, and its accumulation makes m(5)C a hotspot for mutations. It is unknown how m(5)C and 8-oxo-dG correlate with the malignancy of gliomas. We analyzed the total contents of m(5)C and 8-oxo-dG in DNA from tumor tissue and peripheral blood samples from brain glioma patients. We found an opposite relationship in the amounts of m(5)C and 8-oxo-dG, which correlated with glioma grade in the way that low level of m(5)C and high level of 8-oxo-dG indicated increased glioma malignancy grade. Our results could be directly applied to patient monitoring and treatment protocols for gliomas, as well as bolster previous findings, suggesting that spontaneously generated ROS react with m(5)C. Because of the similar mechanisms of m(5)C and guanosine oxidation, we concluded that 8-oxo-dG could also predict glioma malignancy grade and global DNA demethylation in cancer cells.

C1 [Barciszewska, Anna-Maria] Poznan Univ Med Sci, Intraoperat Imaging Unit, Chair & Clin Neurosurg & Neurotraumatol, Przybyszewskiego 49, PL-60355 Poznan, Poland.

[Barciszewska, Anna-Maria] Heliodor Swiecicki Clin Hosp, Dept Neurosurg & Neurotraumatol, Przybyszewskiego 49, PL-60355 Poznan, Poland.

[Giel-Pietraszuk, Malgorzata; Perrigue, Patrick M.; Naskret-Barciszewska, Miroslawa] Polish Acad Sci, Inst Bioorgan Chem, Noskowskiego 12-14, PL-61704 Poznan, Poland.

C3 Poznan University of Medical Sciences; Polish Academy of Sciences

RP Barciszewska, AM (corresponding author), Poznan Univ Med Sci, Intraoperat Imaging Unit, Chair & Clin Neurosurg & Neurotraumatol, Przybyszewskiego 49, PL-60355 Poznan, Poland.; Barciszewska, AM (corresponding author), Heliodor Swiecicki Clin Hosp, Dept Neurosurg & Neurotraumatol, Przybyszewskiego 49, PL-60355 Poznan, Poland.

EM abarcisz@man.poznan.pl

RI Barciszewska, Anna-Maria/J-5978-2018

OI Barciszewska, Anna-Maria/0000-0002-7179-1474; Perrigue,

Patrick/0000-0001-6501-118X

FU SONATA 11 grant from the National Science Center, Poland

[2016/21/D/NZ3/00641]

FX P.M.P. is supported by SONATA 11 (2016/21/D/NZ3/00641) grant from the

National Science Center, Poland.

CR André P, 2017, INT J COSMETIC SCI, V39, P355, DOI 10.1111/ics.12386

[Anonymous], 2006, J. Carcinog., DOI 10.1186/1477-3163-5-14

Armstrong DA, 2014, FASEB J, V28, P2088, DOI 10.1096/fj.13-238402

Barciszewska AM, 2018, BIOSCIENCE REP, V38, DOI 10.1042/BSR20180731

Barciszewska AM, 2015, PLOS ONE, V10, DOI 10.1371/journal.pone.0136669

Barciszewska AM, 2014, PLOS ONE, V9, DOI 10.1371/journal.pone.0092599

Barciszewska MZ, 2007, BIOGERONTOLOGY, V8, P673, DOI 10.1007/s10522-007-9109-3

Bhattacharjee R, 2018, ANALYST, V143, P4802, DOI 10.1039/c8an01348a

Birben E, 2012, WORLD ALLERGY ORGAN, V5, P9, DOI 10.1097/WOX.0b013e3182439613

Cadet J, 2013, CSH PERSPECT BIOL, V5, DOI 10.1101/cshperspect.a012559

Chen LX, 2017, SCIENCE, V355, P752, DOI 10.1126/science.aai8690

Cheng G, 2018, J BIOL CHEM, V293, P10363, DOI 10.1074/jbc.RA118.003044

Conti Alfredo, 2010, Cancers (Basel), V2, P693, DOI 10.3390/cancers2020693

Cooper David N, 2010, Hum Genomics, V4, P406

Dabrowska N, 2017, ADV CLIN EXP MED, V26, P155, DOI 10.17219/acem/43272

Dong YY, 2014, BIOMED REP, V2, P326, DOI 10.3892/br.2014.237

Ehrlich M, 2013, ADV EXP MED BIOL, V754, P31, DOI 10.1007/978-1-4419-9967-2_2

Feil R, 2012, NAT REV GENET, V13, P97, DOI 10.1038/nrg3142

Feinberg AP, 2004, NAT REV CANCER, V4, P143, DOI 10.1038/nrc1279

Fleming AM, 2017, DNA REPAIR, V56, P75, DOI 10.1016/j.dnarep.2017.06.009

Fleming AM, 2017, P NATL ACAD SCI USA, V114, P2604, DOI 10.1073/pnas.1619809114

Gedik CM, 2005, FASEB J, V19, P82, DOI 10.1096/fj.04-1767fje

GROLLMAN AP, 1993, TRENDS GENET, V9, P246, DOI 10.1016/0168-9525(93)90089-Z

Hardiany NS, 2012, MED J INDONES, V21, P122

Hashimoto H, 2014, NATURE, V506, P391, DOI 10.1038/nature12905

Horvath S, 2013, GENOME BIOL, V14, DOI 10.1186/gb-2013-14-10-r115

Iida T, 2001, NEURO-ONCOLOGY, V3, P73, DOI 10.1093/neuonc/3.2.73

Jha P, 2014, NEURO-ONCOLOGY, V16, P1607, DOI 10.1093/neuonc/nou113

Kafer GR, 2016, CELL REP, V14, P1283, DOI 10.1016/j.celrep.2016.01.035

Khan IN, 2018, SEMIN CANCER BIOL, V52, P85, DOI 10.1016/j.semcancer.2017.07.004

Kurkjian C, 2008, CURR PROB CANCER, V32, P185, DOI 10.1016/j.currproblcancer.2008.08.002

Levine AJ, 2010, SCIENCE, V330, P1340, DOI 10.1126/science.1193494

Lewandowska-Gnatowska E, 2014, PLANT PHYSIOL BIOCH, V82, P202, DOI 10.1016/j.plaphy.2014.06.003

Liou GY, 2010, FREE RADICAL RES, V44, P479, DOI 10.3109/10715761003667554

Lokk K, 2014, GENOME BIOL, V15, DOI 10.1186/gb-2014-15-4-r54

López-Moyado IF, 2019, P NATL ACAD SCI USA, V116, P16933, DOI 10.1073/pnas.1903059116

Louis DN, 2016, ACTA NEUROPATHOL, V131, P803, DOI 10.1007/s00401-016-1545-1

Madugundu GS, 2014, NUCLEIC ACIDS RES, V42, P7450, DOI 10.1093/nar/gku334

Michalak M, 2013, PLOS ONE, V8, DOI 10.1371/journal.pone.0070693

Moore LD, 2013, NEUROPSYCHOPHARMACOL, V38, P23, DOI 10.1038/npp.2012.112

Nakabeppu Y, 2014, INT J MOL SCI, V15, P12543, DOI 10.3390/ijms150712543

Nakada Mitsutoshi, 2011, Cancers (Basel), V3, P3242, DOI 10.3390/cancers3033242

Nowak S, 1999, Neurol Neurochir Pol, V33, P1339

Ohno M, 2014, SCI REP-UK, V4, DOI 10.1038/srep04689

PARK EM, 1992, P NATL ACAD SCI USA, V89, P3375, DOI 10.1073/pnas.89.8.3375

Pfaffeneder T, 2014, NAT CHEM BIOL, V10, P574, DOI [10.1038/NCHEMBIO.1532, 10.1038/nchembio.1532]

Poprac P, 2017, TRENDS PHARMACOL SCI, V38, P592, DOI 10.1016/j.tips.2017.04.005

Puri T, 2010, NEUROL INDIA, V58, P20, DOI 10.4103/0028-3886.60389

Radak Z, 2010, FREE RADICAL BIO MED, V49, P587, DOI 10.1016/j.freeradbiomed.2010.05.008

Sabharwal SS, 2014, NAT REV CANCER, V14, P709, DOI 10.1038/nrc3803

Salazar-Ramiro A, 2016, FRONT IMMUNOL, V7, DOI 10.3389/fimmu.2016.00156

Sassa A, 2014, J BIOL CHEM, V289, P13996, DOI 10.1074/jbc.M114.557769

Singh K, 2018, SAUDI PHARM J, V26, P177, DOI 10.1016/j.jsps.2017.12.013

Siomek A, 2007, ANTIOXID REDOX SIGN, V9, P143, DOI 10.1089/ars.2007.9.143

SZATROWSKI TP, 1991, CANCER RES, V51, P794

Trachootham D, 2009, NAT REV DRUG DISCOV, V8, P579, DOI 10.1038/nrd2803

TURK PW, 1995, CARCINOGENESIS, V16, P1253, DOI 10.1093/carcin/16.5.1253

Umeno A, 2017, FREE RADICAL RES, V51, P413, DOI 10.1080/10715762.2017.1315114

Umer M, 2013, ANTIOXID REDOX SIGN, V18, P1972, DOI 10.1089/ars.2012.4923

Valko M, 2006, CHEM-BIOL INTERACT, V160, P1, DOI 10.1016/j.cbi.2005.12.009

Venter JC, 2001, SCIENCE, V291, P1304, DOI 10.1126/science.1058040

WEITZMAN SA, 1994, P NATL ACAD SCI USA, V91, P1261, DOI 10.1073/pnas.91.4.1261

Wesseling P, 2018, NEUROPATH APPL NEURO, V44, P139, DOI 10.1111/nan.12432

Winterbourn CC, 2008, NAT CHEM BIOL, V4, P278, DOI 10.1038/nchembio.85

Wu QH, 2015, CURR DRUG TARGETS, V16, P13, DOI 10.2174/1389450116666150113121054

Xu GL, 2014, BMB REP, V47, P609, DOI 10.5483/BMBRep.2014.47.11.223

Yang HT, 2018, J EXP CLIN CANC RES, V37, DOI 10.1186/s13046-018-0909-x

You CJ, 2014, SCI REP-UK, V4, DOI 10.1038/srep07052

Zhou XL, 2016, CELL SIGNAL, V28, P1163, DOI 10.1016/j.cellsig.2016.05.021

Zukiel R, 2004, MOL CANCER RES, V2, P196

NR 70

TC 35

Z9 37

U1 0

U2 13

PU MDPI

PI BASEL

PA ST ALBAN-ANLAGE 66, CH-4052 BASEL, SWITZERLAND

EI 2073-4409

J9 CELLS-BASEL

JI Cells

PD SEP

PY 2019

VL 8

IS 9

AR 1065

DI 10.3390/cells8091065

PG 14

WC Cell Biology

WE Science Citation Index Expanded (SCI-EXPANDED)

SC Cell Biology

GA JC2JR

UT WOS:000489103800124

PM 31514401

OA gold, Green Published

DA 2025-04-09

ER

PT J

AU Chen, WL

Turlova, E

Sun, CLF

Kim, JS

Huang, S

Zhong, X

Guan, YY

Wang, GL

Rutka, JT

Feng, ZP

Sun, HS

AF Chen, Wen-Liang

Turlova, Ekaterina

Sun, Christopher L. F.

Kim, Ji-Sun

Huang, Sammen

Zhong, Xiao

Guan, Yong-Yuan

Wang, Guan-Lei

Rutka, James T.

Feng, Zhong-Ping

Sun, Hong-Shuo

TI Xyloketal B Suppresses Glioblastoma Cell Proliferation and Migration

in Vitro through Inhibiting TRPM7-Regulated PI3K/Akt and MEK/ERK

Signaling Pathways

SO MARINE DRUGS

LA English

DT Article

ID GROWTH-FACTOR RECEPTOR; VASCULAR ENDOTHELIAL-CELLS; MARINE COMPOUND;

TRPM7 CHANNELS; PROTEIN-KINASE; CANCER; GLIOMA; OVEREXPRESSION;

DERIVATIVES; THERAPIES

AB Glioblastoma, the most common and aggressive type of brain tumors, has devastatingly proliferative and invasive characteristics. The need for finding a novel and specific drug target is urgent as the current approaches have limited therapeutic effects in treating glioblastoma. Xyloketal B is a marine compound obtained from mangrove fungus Xylaria sp. (No. 2508) from the South China Sea, and has displayed antioxidant activity and protective effects on endothelial and neuronal oxidative injuries. In this study, we used a glioblastoma U251 cell line to (1) explore the effects of xyloketal B on cell viability, proliferation, and migration; and (2) investigate the underlying molecular mechanisms and signaling pathways. MTT assay, colony formation, wound healing, western blot, and patch clamp techniques were employed. We found that xyloketal B reduced cell viability, proliferation, and migration of U251 cells. In addition, xyloketal B decreased p-Akt and p-ERK1/2 protein expressions. Furthermore, xyloketal B blocked TRPM7 currents in HEK-293 cells overexpressing TRPM7. These effects were confirmed by using a TRPM7 inhibitor, carvacrol, in a parallel experiment. Our findings indicate that TRPM7-regulated PI3K/Akt and MEK/ERK signaling is involved in anti-proliferation and migration effects of xyloketal B on U251 cells, providing in vitro evidence for the marine compound xyloketal B to be a potential drug for treating glioblastoma.

C1 [Chen, Wen-Liang; Turlova, Ekaterina; Kim, Ji-Sun; Huang, Sammen; Zhong, Xiao; Feng, Zhong-Ping; Sun, Hong-Shuo] Univ Toronto, Fac Med, Dept Physiol, Toronto, ON M5S 1A8, Canada.

[Chen, Wen-Liang; Zhong, Xiao; Rutka, James T.; Sun, Hong-Shuo] Univ Toronto, Dept Surg, Fac Med, Toronto, ON M5S 1A8, Canada.

[Chen, Wen-Liang; Sun, Hong-Shuo] Univ Toronto, Dept Pharmacol, Fac Med, Toronto, ON M5S 1A8, Canada.

[Sun, Christopher L. F.] Univ Toronto, Fac Appl Sci & Engn, Toronto, ON M5S 1A4, Canada.

[Guan, Yong-Yuan; Wang, Guan-Lei] Sun Yat Sen Univ, Zhongshan Sch Med, Dept Pharmacol, Guangzhou 510080, Guangdong, Peoples R China.

[Wang, Guan-Lei] Sun Yat Sen Univ, Dept Educ Guangdong Prov, Key Lab Funct Mol Ocean Microorganisms, Guangzhou 510080, Guangdong, Peoples R China.

[Sun, Hong-Shuo] Univ Toronto, Inst Med Sci, Fac Med, Toronto, ON M5S 1A8, Canada.

C3 University of Toronto; University of Toronto; University of Toronto;

University of Toronto; Sun Yat Sen University; Sun Yat Sen University;

University of Toronto

RP Feng, ZP (corresponding author), Univ Toronto, Fac Med, Dept Physiol, Toronto, ON M5S 1A8, Canada.

EM wenliang.chen@utoronto.ca; e.turlova@mail.utoronto.ca;

christopher.sun@mail.utoronto.ca; jsk.kim@mail.utoronto.ca;

sammen.huang@mail.utoronto.ca; 16822832@qq.com; guanyy@mail.sysu.edu.cn;

wangglei@mail.sysu.edu.cn; james.rutka@sickkids.ca; zp.feng@utoronto.ca;

hss.sun@utoronto.ca

FU Natural Sciences and Engineering Research Council of Canada (NSERC)

[RGPIN 249962, RGPIN 402733]; Canadian Institutes of Health Research

(CIHR) China-Canada Joint Health Research Initiative (CIHR) [132571];

Ontario Graduate Scholarships

FX This work was supported by the following grants: Natural Sciences and

Engineering Research Council of Canada (NSERC) Discovery Grants to ZPF

(RGPIN 249962) and to HSS (RGPIN 402733); Canadian Institutes of Health

Research (CIHR) China-Canada Joint Health Research Initiative to HSS

(CIHR, FRN #132571), Ontario Graduate Scholarships to ET. WC, a lecturer

for Department of Pharmacology, School of Pharmaceutical Sciences,

Guangzhou Medical University, China, is currently a Postdoctoral Fellow

at the University of Toronto.

CR Aarts M, 2003, CELL, V115, P863, DOI 10.1016/S0092-8674(03)01017-1

Alicino I, 2012, PAIN, V153, P245, DOI 10.1016/j.pain.2011.10.002

López-Guerrero JA, 2015, CHIN J CANCER, V34, P41, DOI 10.5732/cjc.014.10278

Berridge MJ, 2003, NAT REV MOL CELL BIO, V4, P517, DOI 10.1038/nrm1155

Chen WL, 2012, PLOS ONE, V7, DOI 10.1371/journal.pone.0049758

Chen WL, 2009, BIOCHEM PHARMACOL, V78, P941, DOI 10.1016/j.bcp.2009.05.029

Cheng YJ, 2014, AM J NUCL MED MOLEC, V4, P385

Clark K, 2008, J MOL BIOL, V378, P790, DOI 10.1016/j.jmb.2008.02.057

Cuddapah VA, 2010, J BIOL CHEM, V285, P11188, DOI 10.1074/jbc.M109.097675

De Luca A, 2012, EXPERT OPIN THER TAR, V16, pS17, DOI 10.1517/14728222.2011.639361

Deason-Towne F, 2012, CELL SIGNAL, V24, P2070, DOI 10.1016/j.cellsig.2012.06.015

Fang L, 2013, TOXICOL APPL PHARM, V272, P713, DOI 10.1016/j.taap.2013.08.009

FLEMING TP, 1992, CANCER RES, V52, P4550

Franken NAP, 2006, NAT PROTOC, V1, P2315, DOI 10.1038/nprot.2006.339

Furnari FB, 2007, GENE DEV, V21, P2683, DOI 10.1101/gad.1596707

Goldberg L, 2006, CANCER RES, V66, P11709, DOI 10.1158/0008-5472.CAN-06-1878

Hottinger AF, 2014, CHIN J CANCER, V33, P32, DOI 10.5732/cjc.013.10207

Huang LY, 2009, CARCINOGENESIS, V30, P737, DOI 10.1093/carcin/bgp034

Inoue K, 2009, CARDIOVASC RES, V83, P547, DOI 10.1093/cvr/cvp153

Jin J, 2008, SCIENCE, V322, P756, DOI 10.1126/science.1163493

Klingler-Hoffmann M, 2003, INT J CANCER, V105, P331, DOI 10.1002/ijc.11085

Langeslag M, 2007, J BIOL CHEM, V282, P232, DOI 10.1074/jbc.M605300200

Lehen'kyi V, 2011, AM J PHYSIOL-CELL PH, V301, pC1281, DOI 10.1152/ajpcell.00249.2011

Leng TD, 2015, CNS NEUROSCI THER, V21, P252, DOI 10.1111/cns.12354

LEON SP, 1994, NEUROSURGERY, V34, P708, DOI 10.1227/00006123-199404000-00021

Li SC, 2013, MAR DRUGS, V11, P5159, DOI 10.3390/md11125159

Li ZX, 2013, MAR DRUGS, V11, P504, DOI 10.3390/md11020504

Liang CC, 2007, NAT PROTOC, V2, P329, DOI 10.1038/nprot.2007.30

Lin YC, 2001, J ORG CHEM, V66, P6252, DOI 10.1021/jo015522r

Liu ML, 2014, CELL SIGNAL, V26, P2773, DOI 10.1016/j.cellsig.2014.08.020

Lu XL, 2010, BRAIN RES, V1332, P110, DOI 10.1016/j.brainres.2010.03.071

McDowell KA, 2011, CURR PHARM DESIGN, V17, P2411

Mellinghoff IK, 2005, NEW ENGL J MED, V353, P2012, DOI 10.1056/NEJMoa051918

Meng XJ, 2013, CANCER LETT, V333, P96, DOI 10.1016/j.canlet.2013.01.031

Parnas M, 2009, CELL CALCIUM, V45, P300, DOI 10.1016/j.ceca.2008.11.009

Pelloski CE, 2006, CLIN CANCER RES, V12, P3935, DOI 10.1158/1078-0432.CCR-05-2202

Pettigrew JD, 2006, J ORG CHEM, V71, P1620, DOI 10.1021/jo052371+

Rich JN, 2004, NAT REV DRUG DISCOV, V3, P430, DOI 10.1038/nrd1380

Rybarczyk P, 2012, INT J CANCER, V131, pE851, DOI 10.1002/ijc.27487

Sathornsumetee S, 2008, ANN NY ACAD SCI, V1142, P108, DOI 10.1196/annals.1444.009

Sathornsumetee S, 2007, CANCER-AM CANCER SOC, V110, P13, DOI 10.1002/cncr.22741

Soni D, 2005, J CLIN NEUROSCI, V12, P1, DOI 10.1016/j.jocn.2004.04.001

Stupp R, 2009, LANCET ONCOL, V10, P459, DOI 10.1016/S1470-2045(09)70025-7

Su JH, 2014, DRUG DES DEV THER, V8, P2555, DOI 10.2147/DDDT.S73476

Sun HS, 2009, NAT NEUROSCI, V12, P1300, DOI 10.1038/nn.2395

Sunayama J, 2010, STEM CELLS, V28, P1930, DOI 10.1002/stem.521

Wang J, 2014, BIOCHEM BIOPH RES CO, V454, P547, DOI 10.1016/j.bbrc.2014.10.118

Wang J, 2014, ASIAN PAC J CANCER P, V15, P3955, DOI 10.7314/APJCP.2014.15.9.3955

Wen PY, 2012, NEURO-ONCOLOGY, V14, P819, DOI 10.1093/neuonc/nos117

WONG AJ, 1992, P NATL ACAD SCI USA, V89, P2965, DOI 10.1073/pnas.89.7.2965

Xiao AJ, 2015, MAR DRUGS, V13, P29, DOI 10.3390/md13010029

Xu ZL, 2010, J MED CHEM, V53, P4642, DOI 10.1021/jm1001502

Yajima I, 2012, DERMAT RES PRACT, V2012, DOI 10.1155/2012/354191

Yang Yong Ryoul, 2013, Advances in Biological Regulation, V53, P232, DOI 10.1016/j.jbior.2013.08.003

Yee Nelson S, 2014, Cells, V3, P751, DOI 10.3390/cells3030751

Yu MZ, 2013, INFLAMM RES, V62, P961, DOI 10.1007/s00011-013-0653-9

Zeng Z, 2015, AM J PHYSIOL-CELL PH, V308, pC308, DOI 10.1152/ajpcell.00275.2013

Zhao J, 2009, BRAIN RES, V1302, P240, DOI 10.1016/j.brainres.2009.09.034

NR 58

TC 62

Z9 70

U1 0

U2 29

PU MDPI

PI BASEL

PA ST ALBAN-ANLAGE 66, CH-4052 BASEL, SWITZERLAND

EI 1660-3397

J9 MAR DRUGS

JI Mar. Drugs

PD APR

PY 2015

VL 13

IS 4

BP 2505

EP 2525

DI 10.3390/md13042505

PG 21

WC Chemistry, Medicinal; Pharmacology & Pharmacy

WE Science Citation Index Expanded (SCI-EXPANDED)

SC Pharmacology & Pharmacy

GA CH0NA

UT WOS:000353715900047

PM 25913706

OA Green Submitted, gold, Green Published

DA 2025-04-09

ER

PT J

AU Huang, ZB

Shi, XY

Li, M

Huang, QM

Xie, LM

AF Huang, Zhibing

Shi, Xiaoyi

Li, Meng

Huang, Qiming

Xie, Liuming

TI Monacolin K Induces Apoptosis of Human Glioma U251 Cells by Triggering

ROS-Mediated Oxidative Damage and Regulating MAPKs and NF-?B Pathways

SO ACS CHEMICAL NEUROSCIENCE

LA English

DT Article

DE monacolin K; Monascus; glioma; apoptosis; intracellular

reduction-oxidation; antitumor

AB Monacolin K (MK), a polyketo secondary metabolic compound of the mold genus Monascus, can promote the apoptosis of malignant cancer cells, possessing potential antitumor properties. However, its mechanism of action on gliomas remains unclear. Here, we explored and investigated the potential of the monacolin K's antitumor effect on human glioma U251 cells and its possible molecular mechanism. Results showed that the application of 10 mu M monacolin K inhibited the proliferation of U251 cells, with an inhibitory rate of up to 53.4%. Additionally, monacolin K induced the generation of reactive oxygen species and activated mitochondria-mediated pathways, including decreased MMP, activation of caspase3/caspase9, decreased Na+/K+-ATPase and Ca2+-ATPase activities, and disruption of the antioxidant system, resulting in the disruption of intracellular reduction-oxidation homeostasis. Monacolin K also activated MAPK and NF -KB pathways, upregulating P38 activity and downregulating JNK/ERK/ P65/IKB alpha expression, ultimately leading to apoptosis of U251 cells. Importantly, monacolin K was not cytotoxic to normal human cells, hUC-MSCs. We concluded that monacolin K can induce apoptosis in U251 cells by triggering ROS-mediated oxidative damage and regulating MAPKs and NF -KB pathways.

C1 [Huang, Zhibing; Shi, Xiaoyi; Li, Meng; Xie, Liuming] Nanchang Univ, State Key Lab Food Sci & Technol, Nanchang 330047, Peoples R China.

[Huang, Zhibing; Shi, Xiaoyi; Li, Meng; Xie, Liuming] Nanchang Univ, Sino German Joint Res Inst, Nanchang 330047, Peoples R China.

[Huang, Qiming] Nanchang Univ, Coll Life Sci, Nanchang 330031, Peoples R China.

[Huang, Qiming] Nanchang Univ, Inst Translat Med, Natl Engn Res Ctr Bioengn Drugs & Technol, Nanchang 330031, Peoples R China.

C3 Nanchang University; Nanchang University; Nanchang University; Nanchang

University

RP Huang, ZB (corresponding author), Nanchang Univ, State Key Lab Food Sci & Technol, Nanchang 330047, Peoples R China.; Huang, ZB (corresponding author), Nanchang Univ, Sino German Joint Res Inst, Nanchang 330047, Peoples R China.

EM hzbchem@ncu.edu.cn

RI Xie, Liuming/ABC-2239-2021

FU Natural Science Foundation of Jiangxi, China [20224ACB205011]; Training

Plan for Academic Leaders of the Main Subject of Jiangxi Province

[20172BCB22006, 20204BCJ22035, 20192ACB70004]; Key Research Project

[20192BBH80015]

FX This work was financially supported by the Natural Science Foundation of

Jiangxi, China (20224ACB205011) , the Training Plan for Academic Leaders

of the Main Subject of Jiangxi Province (No. 20172BCB22006 and

20204BCJ22035) , the Key Program (No. 20192ACB70004) , and the Key

Research Project (No. 20192BBH80015) of Jiangxi Province.

CR Conklin KA, 2004, J NUTR, V134, p3201S, DOI 10.1093/jn/134.11.3201S

Dhillon AS, 2007, ONCOGENE, V26, P3279, DOI 10.1038/sj.onc.1210421

Duan DZ, 2014, FREE RADICAL BIO MED, V70, P182, DOI 10.1016/j.freeradbiomed.2014.02.016

Duan EZ, 2021, RES VET SCI, V138, P30, DOI 10.1016/j.rvsc.2021.05.011

Fan HT, 2021, EXP MOL PATHOL, V118, DOI 10.1016/j.yexmp.2020.104571

Fan Y, 2022, J AGR FOOD CHEM, V70, P10847, DOI 10.1021/acs.jafc.2c02619

Ferreira P, 2010, CLIN NUTR, V29, P819, DOI 10.1016/j.clnu.2010.06.008

Frontinan-Rubio J., RADIOTHER ONCOL

Gong LJ, 2022, TOXICOL APPL PHARM, V454, DOI 10.1016/j.taap.2022.116249

Han XC, 2020, LIFE SCI, V253, DOI 10.1016/j.lfs.2020.117675

Hong MY, 2008, J NUTR BIOCHEM, V19, P448, DOI 10.1016/j.jnutbio.2007.05.012

Hossain KR, 2020, BBA-BIOMEMBRANES, V1862, DOI 10.1016/j.bbamem.2019.183138

HUANG F, 2022, NATURE, V249, P33

Ibraheem F, 2019, MATER LETT, V234, P129, DOI 10.1016/j.matlet.2018.09.075

Ji LN, 2009, LIFE SCI, V85, P788, DOI 10.1016/j.lfs.2009.10.008

Jung J, 2018, INT J MOL SCI, V19, DOI 10.3390/ijms19061657

Kalaivani P, 2014, EUR J MED CHEM, V82, P584, DOI 10.1016/j.ejmech.2014.05.075

Kwak AW, 2022, PHYTOMEDICINE, V105, DOI 10.1016/j.phymed.2022.154383

Liu HQ, 2021, CELL DEATH DIS, V12, DOI 10.1038/s41419-021-03897-0

LIU J, 2022, ONCOL REP, V93

Ma Y. S., MOL THER-ONCOLYTICS

Mahato S, 2023, FOOD CHEM, V399, DOI 10.1016/j.foodchem.2022.133914

McCormack A, 2022, BEST PRACT RES CL EN, V36, DOI 10.1016/j.beem.2022.101713

Mohideen K., CONTEMP CLIN DENT

Pan J, 2018, J AGR FOOD CHEM, V66, P5871, DOI 10.1021/acs.jafc.8b02243

Qin T, 2016, AM J EMERG MED, V34, P1944, DOI 10.1016/j.ajem.2016.06.084

Sanli T, 2011, J THORAC ONCOL, V6, P439, DOI 10.1097/JTO.0b013e3182049d8b

Sreelatha S, 2011, FOOD CHEM TOXICOL, V49, P1270, DOI 10.1016/j.fct.2011.03.006

Wang C, 2020, J HAZARD MATER, V383, DOI 10.1016/j.jhazmat.2019.121157

Wang TY, 2022, PHYTOMEDICINE, V104, DOI 10.1016/j.phymed.2022.154317

Wang XY, 2018, INT J BIOL MACROMOL, V108, P625, DOI 10.1016/j.ijbiomac.2017.12.006

Wozniak B, 2007, J NEURO-ONCOL, V81, P21, DOI 10.1007/s11060-006-9202-5

Xiao HF, 2013, J AGR FOOD CHEM, V61, P1509, DOI 10.1021/jf3050268

Xiong ZX, 2019, FOOD CHEM TOXICOL, V131, DOI 10.1016/j.fct.2019.110585

Xu L, 2022, J AGR FOOD CHEM, DOI 10.1021/acs.jafc.2c04298

Yamawaki R, 2021, CLIN NEUROL NEUROSUR, V208, DOI 10.1016/j.clineuro.2021.106824

Yao JQ, 2022, NANOMED-NANOTECHNOL, V43, DOI 10.1016/j.nano.2022.102554

Zhang BB, 2018, J BIOSCI BIOENG, V125, P333, DOI 10.1016/j.jbiosc.2017.10.011

Zhang L, 2019, GENE, V681, P15, DOI 10.1016/j.gene.2018.09.040

NR 39

TC 0

Z9 0

U1 4

U2 22

PU AMER CHEMICAL SOC

PI WASHINGTON

PA 1155 16TH ST, NW, WASHINGTON, DC 20036 USA

SN 1948-7193

J9 ACS CHEM NEUROSCI

JI ACS Chem. Neurosci.

PD APR 5

PY 2023

VL 14

IS 7

BP 1331

EP 1341

DI 10.1021/acschemneuro.3c00104

EA MAR 2023

PG 11

WC Biochemistry & Molecular Biology; Chemistry, Medicinal; Neurosciences

WE Science Citation Index Expanded (SCI-EXPANDED)

SC Biochemistry & Molecular Biology; Pharmacology & Pharmacy; Neurosciences

& Neurology

GA D0BD1

UT WOS:000953939900001

DA 2025-04-09

ER

PT J

AU Balaji, EV

Kumar, N

Satarker, S

Nampoothiri, M

AF Balaji, Vignesh E.

Kumar, Nitesh

Satarker, Sairaj

Nampoothiri, Madhavan

TI Zinc as a plausible epigenetic modulator of glioblastoma multiforme

SO EUROPEAN JOURNAL OF PHARMACOLOGY

LA English

DT Review

DE Epigenetics; Glioblastoma multiforme; Zinc; Artificial intelligence; DNA

methylation; Histone modification

ID DNA METHYLATION; O-6-METHYLGUANINE-DNA METHYLTRANSFERASE; PROMOTER

METHYLATION; GENE PROMOTER; MGMT GENE; EXPRESSION; EGFR;

HYPERMETHYLATION; CHROMATIN; BINDING

AB Glioblastoma Multiforme (GBM) is an aggressive brain tumor (WHO grade 4 astrocytoma) with unknown causes and is associated with a reduced life expectancy. The available treatment options namely radiotherapy, surgery and chemotherapy have failed to improve life expectancy. Out of the various therapeutic approaches, epigenetic therapy is one of the most studied. Epigenetic therapy is involved in the effective treatment of GBM by inhibiting DNA methyltransferase, histone deacetylation and non-coding RNA. It also promotes the expression of the tumor suppressor gene and is involved in the suppression of the oncogene. Various targets are being studied to implement proper epigenetic regulation to control GBM effectively. Zinc is one of the micronutrients which is considered to maintain epigenetic regulation by promoting the proper DNA folding, protecting genetic material from the oxidative damage and controlling the enzyme activation involved in the epigenetic regulation. Here, we are discussing the importance of zinc in regulating the epigenetic modifications and assessing its role in glioblastoma research. The discussion also highlights the importance of artificial intelligence using epigenetics for envisaging the glioma progression, diagnosis and its management.

C1 [Balaji, Vignesh E.; Kumar, Nitesh; Satarker, Sairaj; Nampoothiri, Madhavan] Manipal Acad Higher Educ, Dept Pharmacol, Manipal Coll Pharmaceut Sci, Manipal 576104, Karnataka, India.

C3 Manipal Academy of Higher Education (MAHE)

RP Nampoothiri, M (corresponding author), Manipal Acad Higher Educ, Dept Pharmacol, Manipal Coll Pharmaceut Sci, Manipal 576104, Karnataka, India.

EM madhavan.ng@manipal.edu

RI Satarker, Sairaj/KGL-7967-2024; Kumar, Nitesh/JGE-0321-2023; Kumar,

Nitesh/V-4466-2019

OI Nampoothiri g, Madhavan/0000-0003-2218-2004; Satarker,

Sairaj/0000-0002-4213-2664; Kumar, Nitesh/0000-0002-4929-3954; Reis,

AlessanRSS/0000-0001-8486-7469; , VIGNESH BALAJI E/0000-0002-6394-5381

CR Adamson C, 2009, EXPERT OPIN INV DRUG, V18, P1061, DOI 10.1517/13543780903052764

Allen BK, 2015, J CELL BIOCHEM, V116, P351, DOI 10.1002/jcb.24990

An ZY, 2018, ONCOGENE, V37, P1561, DOI 10.1038/s41388-017-0045-7

[Anonymous], 2019, NAT GENET, V51, P1, DOI 10.1038/s41588-018-0328-0

Baeza N, 2003, ACTA NEUROPATHOL, V106, P479, DOI 10.1007/s00401-003-0748-4

Bafaro E, 2017, SIGNAL TRANSDUCT TAR, V2, DOI 10.1038/sigtrans.2017.29

Benitez JA, 2017, NAT COMMUN, V8, DOI 10.1038/ncomms15223

Beyer S, 2017, CANCERS, V9, DOI 10.3390/cancers9070085

Bhat AA, 2017, CANCER INVEST, V35, P116, DOI 10.1080/07357907.2016.1271887

Binder H, 2019, ACTA NEUROPATHOL COM, V7, DOI 10.1186/s40478-019-0704-8

Cabrini G, 2015, INT J ONCOL, V47, P417, DOI 10.3892/ijo.2015.3026

Capper D, 2018, ACTA NEUROPATHOL, V136, P181, DOI 10.1007/s00401-018-1879-y

Cassandri M, 2017, CELL DEATH DISCOV, V3, DOI 10.1038/cddiscovery.2017.71

Chen JR, 2016, MEDICINE, V95, DOI 10.1097/MD.0000000000002583

Chinopoulos C, 2018, ASN NEURO, V10, DOI 10.1177/1759091418818261

Chistiakov DA, 2017, EUR J PHARMACOL, V810, P70, DOI 10.1016/j.ejphar.2017.05.064

Choi S, 2018, FASEB J, V32, P404, DOI 10.1096/fj.201700227RRR

Chrun ES, 2017, PATHOL RES PRACT, V213, P1329, DOI 10.1016/j.prp.2017.06.013

Dabrowski MJ, 2019, INT J MOL SCI, V20, DOI 10.3390/ijms20143478

Delcuve GP, 2012, CLIN EPIGENETICS, V4, DOI 10.1186/1868-7083-4-5

Deng L, 2018, ONCOL LETT, V16, P5405, DOI 10.3892/ol.2018.9317

Dimitrov L, 2015, INT J MED SCI, V12, P201, DOI 10.7150/ijms.11047

Etcheverry A, 2010, BMC GENOMICS, V11, DOI 10.1186/1471-2164-11-701

Fernandes J, 2018, TOXICOL MECH METHOD, V28, P328, DOI 10.1080/15376516.2017.1411412

Forloni M, 2016, CELL REP, V16, P457, DOI 10.1016/j.celrep.2016.05.087

Gao YT, 2016, SCI REP-UK, V6, DOI 10.1038/srep32972

Ginisty A, 2019, EUR J PHARMACOL, V855, P30, DOI 10.1016/j.ejphar.2019.04.039

Gömöri É, 2012, DIAGN PATHOL, V7, DOI 10.1186/1746-1596-7-8

Greenberg MVC, 2019, NAT REV MOL CELL BIO, V20, P590, DOI 10.1038/s41580-019-0159-6

Guan Y, 2019, INT J CLIN EXP MED, V12, P8725

Hanif Farina, 2017, Asian Pac J Cancer Prev, V18, P3

Hatae R, 2016, PLOS ONE, V11, DOI 10.1371/journal.pone.0160489

He CC, 2018, NUTR NEUROSCI, V21, P478, DOI [10.1080/1028415X.2017.1312090, 10.1080/1028415x.2017.1312090]

Ho E, 2002, P NATL ACAD SCI USA, V99, P16770, DOI 10.1073/pnas.222679399

Holder LB, 2017, EPIGENETICS-US, V12, P505, DOI 10.1080/15592294.2017.1329068

Hu WL, 2017, BMC MED GENOMICS, V10, DOI 10.1186/s12920-017-0307-9

Jarome TJ, 2018, J NEUROSCI, V38, P7635, DOI 10.1523/JNEUROSCI.0538-18.2018

Jovanovic N, 2019, MEDICINA-LITHUANIA, V55, DOI 10.3390/medicina55020034

Kalkan R, 2016, CRIT REV EUKAR GENE, V26, P137, DOI 10.1615/CritRevEukaryotGeneExpr.2016015964

Kambe T, 2015, PHYSIOL REV, V95, P749, DOI 10.1152/physrev.00035.2014

Kaminska K, 2019, MOL DIAGN THER, V23, P83, DOI 10.1007/s40291-018-0371-7

Kan SF, 2019, ONCOL LETT, V18, P1679, DOI 10.3892/ol.2019.10512

Katsushima K, 2014, FRONT GENET, V5, DOI 10.3389/fgene.2014.00014

Kaur P, 2019, BIORESOUR BIOPROCESS, V6, DOI 10.1186/s40643-019-0237-9

Kazim Zakia, 2019, Asian Pac J Cancer Prev, V20, P269

Ladeira C, 2017, NUTR METAB INSIGHTS, V10, DOI 10.1177/1178638816684666

Lee CY, 2019, TOXICOEPIGENETICS: CORE PRINCIPLES AND APPLICATIONS, P3, DOI 10.1016/B978-0-12-812433-8.00001-0

Li YX, 2016, CSH PERSPECT MED, V6, DOI 10.1101/cshperspect.a026831

Li YX, 2015, MEDCHEMCOMM, V6, P613, DOI 10.1039/c4md00401a

Li ZZ, 2019, J EXP CLIN CANC RES, V38, DOI 10.1186/s13046-019-1371-0

Lim M, 2018, NAT REV CLIN ONCOL, V15, P422, DOI 10.1038/s41571-018-0003-5

Liu S, 2016, MOL CANCER THER, V15, P2977, DOI 10.1158/1535-7163.MCT-16-0320

Lv Xinwen, 2019, Eur J Pharmacol, V863, P172643, DOI 10.1016/j.ejphar.2019.172643

Lyu X, 2019, THER ADV CHRONIC DIS, V10, DOI 10.1177/2040622319862697

Maret W, 2017, INT J MOL SCI, V18, DOI 10.3390/ijms18112285

Marfavi ZH, 2019, ARTIF CELL NANOMED B, V47, P2783, DOI 10.1080/21691401.2019.1577889

Mattiroli F, 2017, SCIENCE, V357, P609, DOI 10.1126/science.aaj1849

Maury E, 2017, EPIGENETICS-US, V12, P353, DOI 10.1080/15592294.2016.1278095

Mirmohammadsadegh A, 2006, CANCER RES, V66, P6546, DOI 10.1158/0008-5472.CAN-06-0384

Mohr SE, 2018, G3-GENES GENOM GENET, V8, P631, DOI 10.1534/g3.117.300447

Molinari F, 2014, FRONT ONCOL, V3, DOI 10.3389/fonc.2013.00326

Montgomery M, 2019, ADV NUTR, V10, P1012, DOI 10.1093/advances/nmz046

Narciso L, 2016, NEURAL PLAST, V2016, DOI 10.1155/2016/3619274

Nowacka-Zawisza M, 2017, ONCOL REP, V38, P2587, DOI 10.3892/or.2017.5972

Núñez FJ, 2019, SCI TRANSL MED, V11, DOI 10.1126/scitranslmed.aaq1427

Ohashi W, 2019, DIGEST DIS SCI, V64, P2404, DOI 10.1007/s10620-019-05561-2

Ozata DM, 2019, NAT REV GENET, V20, P89, DOI 10.1038/s41576-018-0073-3

Padula G, 2017, IN VITRO CELL DEV-AN, V53, P586, DOI 10.1007/s11626-017-0146-1

Pan ZY, 2015, GENET MOL RES, V14, P9813, DOI 10.4238/2015.August.19.14

Parsons C., 2018, Journal of Cancer Metastasis and Treatment, V4, P1, DOI DOI 10.20517/2394-4722.2018.11

Peng Y, 2016, SIGNAL TRANSDUCT TAR, V1, DOI 10.1038/sigtrans.2015.4

Petrescu GED, 2019, J EXP CLIN CANC RES, V38, DOI 10.1186/s13046-019-1180-5

Pfaender S, 2016, NEURAL PLAST, V2016, DOI 10.1155/2016/3760702

Powrózek T, 2016, TRANSL CANCER RES, V5, pS1531, DOI 10.21037/tcr.2016.12.51

Prasad AS, 2002, CANCER METAST REV, V21, P291, DOI 10.1023/A:1021215111729

Radke J, 2019, ACTA NEUROPATHOL COM, V7, DOI 10.1186/s40478-019-0745-z

Rehman AU, 2017, TUMOR BIOL, V39, DOI 10.1177/1010428317705770

Reon BJ, 2016, PLOS MED, V13, DOI 10.1371/journal.pmed.1002192

Rolle K, 2015, ACTA BIOCHIM POL, V62, P353, DOI 10.18388/abp.2015_1072

Saadeh FS, 2018, INT J BIOL MARKER, V33, P22, DOI 10.5301/ijbm.5000301

Sadli N, 2012, CELL PHYSIOL BIOCHEM, V29, P87, DOI 10.1159/000337590

Sahu P, 2019, PSYCHOPHARMACOLOGY, V236, P1829, DOI 10.1007/s00213-019-5166-y

Santangelo A, 2017, ANN TRANSL MED, V5, DOI 10.21037/atm.2017.06.15

Sasaki T, 2019, SCI REP-UK, V9, DOI 10.1038/s41598-019-50849-y

Sato K, 2002, VIRCHOWS ARCH, V440, P160, DOI 10.1007/s004280100499

Shergalis A, 2018, PHARMACOL REV, V70, P412, DOI 10.1124/pr.117.014944

Shukla S, 2013, CANCER RES, V73, P6563, DOI 10.1158/0008-5472.CAN-13-0298

Smrdel U, 2016, RADIOL ONCOL, V50, P394, DOI 10.1515/raon-2015-0041

Song Y, 2009, J NUTR, V139, P1626, DOI 10.3945/jn.109.106369

Sotoudeh H, 2019, FRONT ONCOL, V9, DOI 10.3389/fonc.2019.00768

Staedtke V, 2016, TRENDS CANCER, V2, P338, DOI 10.1016/j.trecan.2016.06.003

Storey K., 2018, BIORXIV, DOI [10.1101/317636., DOI 10.1101/317636]

Strzelczyk JK, 2018, J CANCER, V9, P1896, DOI 10.7150/jca.24477

Suzuki Hiromu, 2013, Frontiers in Genetics, V4, P258, DOI 10.3389/fgene.2013.00258

Tan BL, 2018, FRONT PHARMACOL, V9, DOI 10.3389/fphar.2018.01162

Tang HL, 2015, J CANCER RES THER, V11, P630, DOI 10.4103/0973-1482.146121

Taylor OG, 2019, FRONT ONCOL, V9, DOI 10.3389/fonc.2019.00963

Teplyuk NM, 2016, EMBO MOL MED, V8, P268, DOI 10.15252/emmm.201505495

Tommasini-Ghelfi S, 2019, SCI ADV, V5, DOI 10.1126/sciadv.aaw4543

Toraih EA, 2017, TUMOR BIOL, V39, p1010428317726842, DOI [10.1177/1010428317726842, DOI 10.1177/1010428317726842]

Urbanovska I, 2019, PATHOL ONCOL RES, V25, P971, DOI 10.1007/s12253-018-0413-9

Valdebenito S, 2019, CANCER REP-US, V2, DOI 10.1002/cnr2.1220

Vasilatou D, 2013, EPIGENETICS-US, V8, P561, DOI 10.4161/epi.24897

Vecera M, 2018, INT J MOL SCI, V19, DOI 10.3390/ijms19092754

Venkatesh S, 2015, NAT REV MOL CELL BIO, V16, P178, DOI 10.1038/nrm3941

Wang DF, 2007, J ORG CHEM, V72, P5446, DOI 10.1021/jo070739s

Wang F, 2019, ROY SOC OPEN SCI, V6, DOI 10.1098/rsos.190338

Wang FF, 2018, EBIOMEDICINE, V37, P68, DOI 10.1016/j.ebiom.2018.10.024

Wei JW, 2017, ONCOL REP, V37, P3, DOI 10.3892/or.2016.5236

Weller M, 2015, NAT REV DIS PRIMERS, V1, DOI 10.1038/nrdp.2015.17

Weng XL, 2015, SCI REP-UK, V5, DOI 10.1038/srep10154

Wenger A, 2019, NEURO-ONCOLOGY, V21, P616, DOI 10.1093/neuonc/noz011

Wiencke JK, 2007, NEURO-ONCOLOGY, V9, P271, DOI 10.1215/15228517-2007-003

Xi YB, 2018, J MAGN RESON IMAGING, V47, P1380, DOI 10.1002/jmri.25860

Xie Q, 2018, CELL, V175, P1228, DOI 10.1016/j.cell.2018.10.006

Yang ZJ, 2016, J CANCER RES THER, V12, P233, DOI 10.4103/0973-1482.200745

Yelton Caleb J, 2018, Neuroimmunol Neuroinflamm, V5, DOI 10.20517/2347-8659.2018.58

Yu XM, 2015, TUMOR BIOL, V36, P967, DOI 10.1007/s13277-014-2709-z

Zhang BH, 2007, DEV BIOL, V302, P1, DOI 10.1016/j.ydbio.2006.08.028

Zhang J, 2019, FRONT ONCOL, V9, DOI 10.3389/fonc.2019.01214

Zhang L, 2018, J ENZYM INHIB MED CH, V33, P714, DOI 10.1080/14756366.2017.1417274

Zhang SA, 2019, ACM COMPUT SURV, V52, DOI 10.1145/3285029

Zhang W, 2017, BIOMARK RES, V5, DOI 10.1186/s40364-017-0081-z

Zhang XN, 2016, J THORAC DIS, V8, P2185, DOI 10.21037/jtd.2016.07.21

Zhang ZX, 2017, MEDICINE, V96, DOI 10.1097/MD.0000000000006708

Zyba SJ, 2017, AM J CLIN NUTR, V105, P343, DOI 10.3945/ajcn.116.135327

NR 126

TC 13

Z9 13

U1 1

U2 17

PU ELSEVIER

PI AMSTERDAM

PA RADARWEG 29, 1043 NX AMSTERDAM, NETHERLANDS

SN 0014-2999

EI 1879-0712

J9 EUR J PHARMACOL

JI Eur. J. Pharmacol.

PD NOV 15

PY 2020

VL 887

AR 173549

DI 10.1016/j.ejphar.2020.173549

PG 10

WC Pharmacology & Pharmacy

WE Science Citation Index Expanded (SCI-EXPANDED)

SC Pharmacology & Pharmacy

GA OH6SG

UT WOS:000582725000022

PM 32926916

DA 2025-04-09

ER

PT J

AU Yuan, QY

Yang, WC

Zhang, SX

Li, TF

Zuo, MR

Zhou, XW

Li, JH

Li, M

Xia, XQ

Chen, MN

Liu, YH

AF Yuan, Qiuyun

Yang, Wanchun

Zhang, Shuxin

Li, Tengfei

Zuo, Mingrong

Zhou, Xingwang

Li, Junhong

Li, Mao

Xia, Xiaoqiang

Chen, Mina

Liu, Yanhui

TI Inhibition of mitochondrial carrier homolog 2 (MTCH2) suppresses tumor

invasion and enhances sensitivity to temozolomide in malignant glioma

SO MOLECULAR MEDICINE

LA English

DT Article

DE MTCH2; Glioma; Temozolomide; Mitochondria; Cell migration; invasion;

Cell death

AB BackgroundMalignant glioma exerts a metabolic shift from oxidative phosphorylation (OXPHOs) to aerobic glycolysis, with suppressed mitochondrial functions. This phenomenon offers a proliferation advantage to tumor cells and decrease mitochondria-dependent cell death. However, the underlying mechanism for mitochondrial dysfunction in glioma is not well elucidated. MTCH2 is a mitochondrial outer membrane protein that regulates mitochondrial metabolism and related cell death. This study aims to clarify the role of MTCH2 in glioma.MethodsBioinformatic analysis from TCGA and CGGA databases were used to investigate the association of MTCH2 with glioma malignancy and clinical significance. The expression of MTCH2 was verified from clinical specimens using real-time PCR and western blots in our cohorts. siRNA-mediated MTCH2 knockdown were used to assess the biological functions of MTCH2 in glioma progression, including cell invasion and temozolomide-induced cell death. Biochemical investigations of mitochondrial and cellular signaling alternations were performed to detect the mechanism by which MTCH2 regulates glioma malignancy.ResultsBioinformatic data from public database and our cohort showed that MTCH2 expression was closely associated with glioma malignancy and poor patient survival. Silencing of MTCH2 expression impaired cell migration/invasion and enhanced temozolomide sensitivity of human glioma cells. Mechanistically, MTCH2 knockdown may increase mitochondrial OXPHOs and thus oxidative damage, decreased migration/invasion pathways, and repressed pro-survival AKT signaling.ConclusionOur work establishes the relationship between MTCH2 expression and glioma malignancy, and provides a potential target for future interventions.

C1 [Yuan, Qiuyun; Yang, Wanchun; Zhang, Shuxin; Li, Tengfei; Zuo, Mingrong; Zhou, Xingwang; Li, Junhong; Li, Mao; Xia, Xiaoqiang; Chen, Mina; Liu, Yanhui] Sichuan Univ, West China Hosp, State Key Lab Biotherapy, Dept Neurosurg, Chengdu 610041, Peoples R China.

C3 Sichuan University

RP Chen, MN; Liu, YH (corresponding author), Sichuan Univ, West China Hosp, State Key Lab Biotherapy, Dept Neurosurg, Chengdu 610041, Peoples R China.

EM chenmina2010@scu.edu.cn; liuyh@scu.edu.cn

RI XIA, XIAOQIANG/JCO-6775-2023; zuo, mingrong/IST-0923-2023; Yang,

Wanchun/GRX-1427-2022; Wang, Leilei/P-4178-2014; liu,

yanhui/AAC-5936-2021; Chen, Mina/GQO-9722-2022

OI Chen, Mina/0000-0002-7446-3978; Yang, Wanchun/0000-0003-4852-1887

FU Sichuan Province Science and Technology Support Program [2017SZ0006];

National Natural Science Foundation of China [31501155, 81571195]

FX This work was supported by the grant from Sichuan Province Science and

Technology Support Program (2017SZ0006 to Yanhui Liu), and the National

Natural Science Foundation of China (31501155 to Wanchun Yang and

81571195 to Mina Chen).

CR Agnihotri S, 2016, NEURO-ONCOLOGY, V18, P160, DOI 10.1093/neuonc/nov125

Alcalá S, 2008, ONCOGENE, V27, P44, DOI 10.1038/sj.onc.1210600

Arigoni M, 2013, AM J PATHOL, V182, P2058, DOI 10.1016/j.ajpath.2013.02.046

Bahat A, 2018, NAT COMMUN, V9, DOI 10.1038/s41467-018-07519-w

Buzaglo-Azriel L, 2017, CELL REP, V18, P1335, DOI 10.1016/j.celrep.2017.01.046

Cogliati S, 2010, CELL RES, V20, P863, DOI 10.1038/cr.2010.100

Deighton RF, 2014, J NEURO-ONCOL, V118, P247, DOI 10.1007/s11060-014-1430-5

Eckel-Passow JE, 2015, NEW ENGL J MED, V372, P2499, DOI 10.1056/NEJMoa1407279

ESTERBAUER H, 1985, BIOCHEM J, V228, P363, DOI 10.1042/bj2280363

Franceschi S, 2018, EBIOMEDICINE, V37, P56, DOI 10.1016/j.ebiom.2018.10.008

Jacinto E, 2004, NAT CELL BIOL, V6, P1122, DOI 10.1038/ncb1183

Kaech S, 2006, NAT PROTOC, V1, P2406, DOI 10.1038/nprot.2006.356

Katz C, 2012, J BIOL CHEM, V287, P15016, DOI 10.1074/jbc.M111.328377

Keatley K, 2019, INT J MOL SCI, V20, DOI 10.3390/ijms20133364

Khan DH, 2020, BLOOD, V136, P81, DOI 10.1182/blood.2019000106

Laug D, 2018, NAT REV NEUROSCI, V19, P393, DOI 10.1038/s41583-018-0014-3

Leibowitz-Amit R, 2006, CANCER RES, V66, P8687, DOI 10.1158/0008-5472.CAN-05-2294

Li TF, 2020, CANCER CELL INT, V20, DOI 10.1186/s12935-020-1145-y

Lokody I, 2014, NAT REV CANCER, V14, P706, DOI 10.1038/nrc3840

Lytovchenko O, 2017, BBA-BIOENERGETICS, V1858, P641, DOI 10.1016/j.bbabio.2017.03.006

Martinez-Outschoorn UE, 2017, NAT REV CLIN ONCOL, V14, P113, DOI 10.1038/nrclinonc.2017.1

Maryanovich M, 2015, NAT COMMUN, V6, DOI 10.1038/ncomms8901

Matsuzaki H, 1999, J NEUROCHEM, V73, P2037

Michelakis ED, 2010, SCI TRANSL MED, V2, DOI 10.1126/scitranslmed.3000677

Moriyama K, 1996, GENES CELLS, V1, P73, DOI 10.1046/j.1365-2443.1996.05005.x

Omuro A, 2013, JAMA-J AM MED ASSOC, V310, P1842, DOI 10.1001/jama.2013.280319

Puchalski RB, 2018, SCIENCE, V360, P660, DOI 10.1126/science.aaf2666

Robinson AJ, 2012, EXP CELL RES, V318, P1316, DOI 10.1016/j.yexcr.2012.01.026

Ruggiero A, 2017, SCI REP-UK, V7, DOI 10.1038/srep44401

Schiapparelli P, 2017, EBIOMEDICINE, V21, P94, DOI 10.1016/j.ebiom.2017.06.020

Strickland M, 2017, FRONT CELL DEV BIOL, V5, DOI 10.3389/fcell.2017.00043

Stupp R, 2009, LANCET ONCOL, V10, P459, DOI 10.1016/S1470-2045(09)70025-7

Tan HL, 2020, CELL DEATH DIFFER, V27, P1369, DOI 10.1038/s41418-019-0422-6

Venneti S, 2017, ANNU REV PATHOL-MECH, V12, P515, DOI 10.1146/annurev-pathol-012615-044329

Veresov VG, 2014, CELL SIGNAL, V26, P370, DOI 10.1016/j.cellsig.2013.11.016

Vyas S, 2016, CELL, V166, P555, DOI 10.1016/j.cell.2016.07.002

Warburg O, 1927, J GEN PHYSIOL, V8, P519, DOI 10.1085/jgp.8.6.519

Xie Q, 2015, NAT NEUROSCI, V18, P501, DOI 10.1038/nn.3960

Zaltsman Y, 2010, NAT CELL BIOL, V12, P553, DOI 10.1038/ncb2057

NR 39

TC 19

Z9 20

U1 2

U2 10

PU SPRINGER

PI NEW YORK

PA ONE NEW YORK PLAZA, SUITE 4600, NEW YORK, NY, UNITED STATES

SN 1076-1551

EI 1528-3658

J9 MOL MED

JI Mol. Med.

PD JAN 28

PY 2021

VL 27

IS 1

AR 7

DI 10.1186/s10020-020-00261-4

PG 13

WC Biochemistry & Molecular Biology; Cell Biology; Medicine, Research &

Experimental

WE Science Citation Index Expanded (SCI-EXPANDED)

SC Biochemistry & Molecular Biology; Cell Biology; Research & Experimental

Medicine

GA QE3WL

UT WOS:000616139400001

PM 33509092

OA gold, Green Published, Green Submitted

DA 2025-04-09

ER

PT J

AU Wu, HL

Fu, XY

Cao, WG

Xiang, WZ

Hou, YJ

Ma, JK

Wang, Y

Fan, CD

AF Wu, Hua-lian

Fu, Xiao-yan

Cao, Wen-giang

Xiang, Wen-zhou

Hou, Ya-jun

Ma, Jin-kui

Wang, Ying

Fan, Cun-dong

TI Induction of Apoptosis in Human Glioma Cells by Fucoxanthin via

Triggering of ROS-Mediated Oxidative Damage and Regulation of MAPKs and

PI3K-AKT Pathways

SO JOURNAL OF AGRICULTURAL AND FOOD CHEMISTRY

LA English

DT Article

DE fucoxanthin; glioma cells; reactive oxygen species; apoptosis; oxidative

damage

ID CYCLE ARREST; TEMOZOLOMIDE; INHIBITION

AB Fucoxanthin, a natural carotenoid derived from algae, exhibits novel anticancer potential. However, fucoxanthin with high purity is hard to prepare, and the anticancer mechanism remains elusive. In the present study, fucoxanthin with high purity was prepared and purified from the marine microalgae Nitzschia sp. by silica-gel column chromatography (SGCC), and the underlying mechanism against human glioma cells was evaluated. The results showed that fucoxanthin time- and dose-dependently inhibited U251-human-glioma-cell growth by induction of apoptosis (64.4 +/- 4.8, P < 0.01) accompanied by PARP cleavage and caspase activation (244 +/- 14.2, P < 0.01). Mechanically, fucoxanthin time-dependently triggered reactive-oxygen-species (ROS)-mediated DNA damage (100 +/- 7.38, P < 0.01), as evidenced by the phosphorylation activation of Ser1981-ATM, Ser428-ATR, Ser15-p53, and Ser139-histone. Moreover, fucoxanthin treatment also time-dependently caused dysfunction of MAPKs and PI3K-AKT pathways, as demonstrated by the phosphorylation activation of Thr183-JNK, Thr180-p38, and Thr202-ERK and the phosphorylation inactivation of Ser473-AKT. The addition of kinase inhibitors further confirmed the importance of MAPKs and PI3K-AKT pathways in fucoxanthin-induced cell-growth inhibition (32.5 +/- 3.6, P < 0.01). However, ROS inhibition by the antioxidant glutathione (GSH) effectively inhibited fucoxanthin-induced DNA damage, attenuated the dysfunction of MAPKs and PI3K-AKT pathways, and eventually blocked fucoxanthin-induced cytotoxicity (54.3 +/- 5.6, P < 0.05) and cell apoptosis (32.7 +/- 2.5, P < 0.05), indicating that ROS production, an early apoptotic event, is involved in the fucoxanthin-mediated anticancer mechanism. Taken together, these results suggested that fucoxanthin induced U251-human-glioma-cell apoptosis by triggering ROS-mediated oxidative damage and dysfunction of MAPKs and PI3K-AKT pathways, which validated that fucoxanthin may be a candidate for potential applications in cancer chemotherapy and chemoprevention.

C1 [Wu, Hua-lian; Xiang, Wen-zhou] Chinese Acad Sci, CAS Key Lab Trop Marine Bioresources & Ecol LMB C, Guangdong Key Lab Marine Mat Med LMMM GD, South China Sea Inst Oceanol, Guangzhou 510301, Guangdong, Peoples R China.

[Fu, Xiao-yan; Hou, Ya-jun; Wang, Ying; Fan, Cun-dong] Taishan Med Univ, Key Lab Cerebral Microcirculat Univ Shandong, Tai An 271000, Shandong, Peoples R China.

[Cao, Wen-giang] Zhuhai Hopegenes Med & Phamaceut Inst, Hengqin New Area, Zhuhai 519000, Guangdong, Peoples R China.

[Ma, Jin-kui] Zhaoqing Univ, Sch Food & Pharmaceut Engn, Zhaoqing 526061, Guangdong, Peoples R China.

C3 Chinese Academy of Sciences; South China Sea Institute of Oceanology,

CAS; Shandong First Medical University & Shandong Academy of Medical

Sciences; Zhaoqing University

RP Wang, Y; Fan, CD (corresponding author), Taishan Med Univ, Key Lab Cerebral Microcirculat Univ Shandong, Tai An 271000, Shandong, Peoples R China.

EM yingw9@163.com; tcdfan66@163.com

RI ma, jinkui/AAC-7492-2019; Fan, Cundong/ABG-5600-2021; Fu,

Xiaoyan/C-2573-2012

OI , Jin-Kui/0000-0001-9346-2076; Fan, Cun-dong/0000-0003-4485-1344

FU National Natural Science Foundation of China [31402320, 81701178,

81501106]; Knowledge Innovation Program of the Chinese Academy of

Sciences [SQ201213]

FX This work was financially supported by the National Natural Science

Foundation of China (Nos. 31402320, 81701178, and 81501106) and the

Knowledge Innovation Program of the Chinese Academy of Sciences (No.

SQ201213).

CR [Anonymous], 2018, NITZSCHIA LAEVIS MAR

[Anonymous], 2012, Curr. Bioact. Comp, DOI DOI 10.2174/157340712801784787

Beppu F, 2009, J TOXICOL SCI, V34, P501, DOI 10.2131/jts.34.501

Burri SH, 2015, J NEURO-ONCOL, V123, P259, DOI 10.1007/s11060-015-1793-2

Dembitsky VM, 2007, PROG LIPID RES, V46, P328, DOI 10.1016/j.plipres.2007.07.001

Fei HR, 2012, MOL BIOL REP, V39, P131, DOI 10.1007/s11033-011-0721-6

Goodenberger ML, 2012, CANCER GENET-NY, V205, P613, DOI 10.1016/j.cancergen.2012.10.009

Hou LL, 2013, ACTA PHARMACOL SIN, V34, P1403, DOI 10.1038/aps.2013.90

Ius T, 2014, J NEUROSURG, V120, P12, DOI 10.3171/2013.9.JNS13728

Jang EJ, 2018, BMC COMPLEM ALTERN M, V18, DOI 10.1186/s12906-018-2164-2

Jemal A, 2007, CA-CANCER J CLIN, V57, P43, DOI 10.3322/canjclin.57.1.43

Kasiappan R., 2016, React. Oxyg. Species. (Apex), V1, DOI [10.20455/ros.2016.805, DOI 10.20455/ROS.2016.805]

Kawee-ai A, 2014, NAT PROD COMMUN, V9, P921

Kim KN, 2013, ENVIRON TOXICOL PHAR, V35, P39, DOI 10.1016/j.etap.2012.10.002

Kim KN, 2010, TOXICOL IN VITRO, V24, P1648, DOI 10.1016/j.tiv.2010.05.023

Kim SM, 2012, APPL BIOCHEM BIOTECH, V166, P1843, DOI 10.1007/s12010-012-9602-2

Liu YG, 2016, NEUROCHEM RES, V41, P2728, DOI 10.1007/s11064-016-1989-7

Ong JYH, 2015, LIFE SCI, V135, P158, DOI 10.1016/j.lfs.2015.03.019

Peng J, 2011, MAR DRUGS, V9, P1806, DOI 10.3390/md9101806

Rwigemera A, 2014, CELL BIOL TOXICOL, V30, P157, DOI 10.1007/s10565-014-9277-2

Satomi Y, 2012, ANTICANCER RES, V32, P807

STAUBER JL, 1988, J PHYCOL, V24, P158

Stupp R, 2005, NEW ENGL J MED, V352, P987, DOI 10.1056/NEJMoa043330

Wang LJ, 2018, MAR DRUGS, V16, DOI 10.3390/md16010033

Wang LB, 2014, ACTA BIOCH BIOPH SIN, V46, P877, DOI 10.1093/abbs/gmu080

Wang S, 2018, MICROB CELL FACT, V17, DOI 10.1186/s12934-018-0957-0

Wu HL, 2016, CHIN J OCEANOL LIMN, V34, P391, DOI 10.1007/s00343-015-4325-1

Yamamoto K, 2011, CANCER LETT, V300, P225, DOI 10.1016/j.canlet.2010.10.016

Yu J, 2014, INT J BIOL MACROMOL, V68, P67, DOI 10.1016/j.ijbiomac.2014.04.018

Yu RX, 2011, EUR J PHARMACOL, V657, P10, DOI 10.1016/j.ejphar.2010.12.006

NR 30

TC 62

Z9 65

U1 1

U2 68

PU AMER CHEMICAL SOC

PI WASHINGTON

PA 1155 16TH ST, NW, WASHINGTON, DC 20036 USA

SN 0021-8561

EI 1520-5118

J9 J AGR FOOD CHEM

JI J. Agric. Food Chem.

PD FEB 27

PY 2019

VL 67

IS 8

BP 2212

EP 2219

DI 10.1021/acs.jafc.8b07126

PG 8

WC Agriculture, Multidisciplinary; Chemistry, Applied; Food Science &

Technology

WE Science Citation Index Expanded (SCI-EXPANDED)

SC Agriculture; Chemistry; Food Science & Technology

GA HN5CG

UT WOS:000460200000012

PM 30688446

DA 2025-04-09

ER

PT J

AU Kara, ID

Cakir, A

Ozaslan, M

Kili, IH

Tepe, B

Akdogan, E

Kazaz, C

AF Kara, Isik Didem

Cakir, Ahmet

Ozaslan, Mehmet

Kili, Ibrahim Halil

Tepe, Bektas

Akdogan, Ebru

Kazaz, Cavit

TI Anticancer Agents from Xanthium strumarium Fruits Against C6

Glioma Cells

SO INTERNATIONAL JOURNAL OF PHARMACOLOGY

LA English

DT Article

DE Xanthium strumarium; cocklebur; xanthanolides; glioma; apoptosis; lipid

peroxidation; radiotherapy

ID CYCLE ARREST; IN-VITRO; SESQUITERPENE LACTONES; INDUCED APOPTOSIS;

CANCER-CELLS; XANTHATIN; PROTEIN; INHIBITION; EXPRESSION; FLAVONOIDS

AB Background and Objective: Glioma is known to be highly resistant to radiotherapy and chemotherapy. Therefore, new therapeutic agents for glioma are being extensively researched. In this study, we aimed to investigate anti-tumoural effects of Xanthium strumarium L. (Asteraceae family) extracts and its pure constituents on C6 glioma cells. Materials and Methods: In this study, cytotoxicity, lipid peroxidation, apoptotic effect, phosphatidylserine externalization, cell cycle analysis, invasion, kinase activity, COX-2 expression and micronucleus tests were used. Results: As a result of IR, H-1-NMR, C-13-NMR, 1D and 2D NMR analysis, 5 known compounds were characterized as xanthinosin (1), stigmasterol (2), xanthatin (3), xanthinin (4) and xanthanol (5). Among them, the most cytotoxic xanthanolide was xanthinin (4) with an IC50 value of 7.5 mu M. It was thought that this effect may be due to the oxidative damage and protein kinase activity of this compound. While xanthinosin (1) and xanthanol (5) showed the selective cytotoxic effect on C6 glioma cells with low IC50 values (22.46 and 40.12 mu M, respectively), it was determined that their toxicity on Human Umbilical Vein Endothelial (HUVEC) cells was lower (IC50 >75 mu M). Conclusion: It was concluded that xanthanolides isolated from X. strumarium could be used as target molecules in future studies as chemotherapeutic agents on glioma.

C1 [Kara, Isik Didem; Ozaslan, Mehmet; Kili, Ibrahim Halil; Akdogan, Ebru] Gaziantep Univ, Dept Biol, TR-27310 Sehitkamil, Gaziantep, Turkey.

[Cakir, Ahmet] Kilis 7 Aralik Univ, Dept Chem, Fac Art & Sci, Kilis, Turkey.

[Cakir, Ahmet; Tepe, Bektas] Kilis 7 Aralik Univ, Adv Technol Applicat & Res Ctr ATARC, Kilis, Turkey.

[Tepe, Bektas] Kilis 7 Aralik Univ, Dept Mol Biol & Genet, Fac Art & Sci, Kilis, Turkey.

[Kazaz, Cavit] Ataturk Univ, Fac Sci, Dept Chem, Erzurum, Turkey.

C3 Gaziantep University; Kilis 7 Aralik University; Kilis 7 Aralik

University; Kilis 7 Aralik University; Ataturk University

RP Ozaslan, M (corresponding author), Gaziantep Univ, Dept Biol, TR-27310 Sehitkamil, Gaziantep, Turkey.

RI TEPE, Bektas/GZM-5592-2022; Kazaz, Cavit/AGG-7269-2022; ÇAKIR,

Ahmet/R-1884-2019; OZASLAN, Mehmet/T-5549-2018; Karagöz,

Işık/AAG-5473-2020; Ozaslan, Mehmet/J-5494-2018

OI Ozaslan, Mehmet/0000-0001-9380-4902

FU Scientific and Technological Research Council of Turkey (TUBITAK)

[114Z574]

FX The authors are thankful to the Scientific and Technological Research

Council of Turkey (TUBITAK) for financial support (114Z574).

CR Aldibekova D.A., 2018, OnLine J Biol Sci, V18, P237, DOI DOI 10.3844/OJBSCI.2018.237.246

Alorfi HS, 2020, PHARMACOGN MAG, V16, pS391, DOI 10.4103/pm.pm_585_19

[Anonymous], 2017, CURR ENZYME INHIB, DOI DOI 10.2174/1573408013666161128144216

Armstrong JS, 2001, BIOCHEM BIOPH RES CO, V289, P973, DOI 10.1006/bbrc.2001.6054

Arslan MA, 2006, CURR CANCER DRUG TAR, V6, P623, DOI 10.2174/156800906778742479

ASCO, 2021, BRAIN TUMORS STAT

Barros GP, 2012, INT J GEOPHYS, V2012, DOI [10.1155/2012/459497, 10.5402/2012/137289]

Behrend L, 2003, BIOCHEM SOC T, V31, P1441

Bui VB, 2012, PHYTOCHEM LETT, V5, P685, DOI 10.1016/j.phytol.2012.07.008

Araújo LCC, 2013, PLOS ONE, V8, DOI 10.1371/journal.pone.0081973

Cragg GM, 2006, J NAT PROD, V69, P488, DOI 10.1021/np0581216

Dogan A.L., 2004, ACTA MEDICA CORDOBA, V35, P34

Ermert M, 2003, LAB INVEST, V83, P333, DOI 10.1097/01.LAB.0000059924.47118.88

Feng CP, 2016, J ETHNOPHARMACOL, V188, P70, DOI 10.1016/j.jep.2016.04.042

Fu J, 2021, ORG CHEM FRONT, V8, P1288, DOI 10.1039/d0qo01541e

Gabriely G, 2008, MOL CELL BIOL, V28, P5369, DOI 10.1128/MCB.00479-08

Grobben B, 2002, CELL TISSUE RES, V310, P257, DOI 10.1007/s00441-002-0651-7

Joki T, 2000, CANCER RES, V60, P4926

Kamal N., 2016, Journal of Applied Pharmaceutical Science, V6, P102

Khan Y., 2020, INT J SCI ENG RES, V11, P587

Kim YS, 2003, PLANTA MED, V69, P375, DOI 10.1055/s-2003-38879

Kovács A, 2009, Z NATURFORSCH C, V64, P343

Li WD, 2013, PHYTOMEDICINE, V20, P865, DOI 10.1016/j.phymed.2013.03.006

Li YJ, 2016, PLANT CELL PHYSIOL, V57, P630, DOI 10.1093/pcp/pcw019

Linden A, 2008, TOXICOL IN VITRO, V22, P1371, DOI 10.1016/j.tiv.2008.02.003

Luzhna Lidiya, 2013, Frontiers in Genetics, V4, P131, DOI 10.3389/fgene.2013.00131

Mobio TA, 2000, TOXICOL APPL PHARM, V164, P91, DOI 10.1006/taap.2000.8893

Morales-Ramirez P, 2017, MUTAGENESIS, V32, P411, DOI 10.1093/mutage/gex010

Murillo-Alvarez JI, 2001, PHARM BIOL, V39, P445, DOI 10.1076/phbi.39.6.445.5877

Nayak P.S., 2015, International Journal of Pharmacy and Pharmaceutical Sciences, V7, P25

Oliver L, 2016, TRANSL CANCER RES, V5, pS1073, DOI 10.21037/tcr.2016.11.20

Pelicano H, 2004, DRUG RESIST UPDATE, V7, P97, DOI 10.1016/j.drup.2004.01.004

Perillo B, 2020, EXP MOL MED, V52, P192, DOI 10.1038/s12276-020-0384-2

Ferrer JP, 2016, NAT PROD COMMUN, V11, P371

Rajaratnam V, 2020, CANCERS, V12, DOI 10.3390/cancers12040937

Ramírez-Erosa I, 2007, CAN J PHYSIOL PHARM, V85, P1160, DOI 10.1139/Y07-104

Ranjini HS, 2017, ADV SCI LETT, V23, P1889, DOI 10.1166/asl.2017.8509

Riss T. L., 2016, Assay Guid. Manual, V22, P978

Roy S., 2008, Open Leuk J, V1, P1, DOI [10.2174/1876816400801010001, DOI 10.2174/1876816400801010001]

Schumacker PT, 2006, CANCER CELL, V10, P175, DOI 10.1016/j.ccr.2006.08.015

Serpeloni JM, 2015, J ETHNOPHARMACOL, V176, P345, DOI 10.1016/j.jep.2015.11.003

Shi L, 2010, BRAIN RES, V1352, P255, DOI 10.1016/j.brainres.2010.07.009

Shono T, 2001, CANCER RES, V61, P4375

Sun ZL, 2017, ONCOTARGET, V8, P58823, DOI 10.18632/oncotarget.19221

Takeda S, 2011, CHEM RES TOXICOL, V24, P855, DOI 10.1021/tx200046s

Tong C, 2020, NAT PROD COMMUN, V15, DOI 10.1177/1934578X20945541

Tong S, 2013, ONCOL REP, V30, P2171, DOI 10.3892/or.2013.2694

Ventura Juan-Jose, 2006, Clin Transl Oncol, V8, P153, DOI 10.1007/s12094-006-0005-0

Wang CZ, 2013, INT J ONCOL, V42, P1018, DOI 10.3892/ijo.2013.1777

Wätjen W, 2004, BIOMETALS, V17, P65, DOI 10.1023/A:1024405119018

Wu QG, 2016, TOXICOL LETT, V253, P55, DOI 10.1016/j.toxlet.2016.04.016

Wu WS, 2006, CANCER METAST REV, V25, P695, DOI 10.1007/s10555-006-9037-8

Wu XX, 2018, FRONT PHARMACOL, V9, DOI 10.3389/fphar.2018.01466

Xu KM, 2014, ONCOTARGET, V5, P1241, DOI 10.18632/oncotarget.1370

Yaglioglu Muhammet Samil, 2017, Anatolian Bryology, V3, P31

Zhang L, 2012, MOLECULES, V17, P3736, DOI 10.3390/molecules17043736

NR 56

TC 3

Z9 4

U1 4

U2 24

PU ASIAN NETWORK SCIENTIFIC INFORMATION-ANSINET

PI FAISALABAD

PA 308-LASANI TOWN, SARGODHA RD, FAISALABAD, 38090, PAKISTAN

SN 1811-7775

EI 1812-5700

J9 INT J PHARMACOL

JI Int. J. Pharmacol.

PY 2022

VL 18

IS 3

BP 437

EP 454

DI 10.3923/ijp.2022.437.454

PG 18

WC Pharmacology & Pharmacy

WE Science Citation Index Expanded (SCI-EXPANDED)

SC Pharmacology & Pharmacy

GA 0U3NI

UT WOS:000787558900006

DA 2025-04-09

ER

PT J

AU Tu, YY

Wang, Z

Wang, X

Yang, HW

Zhang, PX

Johnson, M

Liu, N

Liu, H

Jin, WL

Zhang, YS

Cui, DX

AF Tu, Yanyang

Wang, Zhen

Wang, Xin

Yang, Hongwei

Zhang, Pengxing

Johnson, Mark

Liu, Nan

Liu, Hui

Jin, Weilin

Zhang, Yongsheng

Cui, Daxiang

TI Birth of MTH1 as a therapeutic target for glioblastoma: MTH1 is

indispensable for gliomatumorigenesis

SO AMERICAN JOURNAL OF TRANSLATIONAL RESEARCH

LA English

DT Article

DE GBM; MTH1; reactive oxygen species; viability; stemness

ID HUMAN MUTT HOMOLOG; DNA-DAMAGE; OXIDIZED FORMS; CANCER-CELLS; REPAIR;

ROS; 8-OXOGUANINE; MECHANISMS; NUCLEOTIDE; PROTEIN

AB Malignant glioma is the most common primary tumor of the central nervous system. Chemotherapy and radiotherapy are the most common therapeutic approaches in glioma therapy. Both processes mainly kill cancer cells through generating high Reactive Oxygen Species (ROS) and lead to oxidative DNA damage. However, tumor resistance to ROS is always a challenge for cancer treatment. Human Mut T homolog 1 (MTH1, also known as NUDT1) is regarded as a protector of nucleotides against oxidization. Recent reports have verified that overexpression of MTH1 could remove oxidized dNTP pools. Here, we find that MTH1 is overexpressed both at mRNA and protein levels in GBM. MTH1 silencing inhibits colony formation; tumor spheres formation and xenograft tumor growth, and more importantly, the viability of glioma cells is significantly decreased in H2O2 after MTH1 was knocked down in glioma. PI staining show that H2O2 cause more glioma cell death after MTH1 silencing. So we speculate that overexpression of MTH1 is crucial for glioma survival, suppression of its expression can inhibit cancer cell survival in vitro and in vivo, MTH1 may be a potential target for human glioma therapy in future.

C1 [Tu, Yanyang; Wang, Zhen; Zhang, Pengxing; Liu, Nan; Liu, Hui; Zhang, Yongsheng] Fourth Mil Med Univ, Tangdu Hosp, Dept Expt Surg, Xian 710038, Peoples R China.

[Jin, Weilin; Cui, Daxiang] Shanghai Jiao Tong Univ, Inst Nano Biomed & Engn, Sch Elect Informat & Elect Engn,Minist Educ, Dept Instrument Sci & Engn,Key Lab Thin Film & Mi, Shanghai 200240, Peoples R China.

[Tu, Yanyang; Wang, Xin; Yang, Hongwei; Johnson, Mark] Harvard Med Sch, Brigham & Womens Hosp, Dept Neurosurg, Boston, MA 02115 USA.

C3 Air Force Medical University; Shanghai Jiao Tong University; Harvard

University; Harvard University Medical Affiliates; Brigham & Women's

Hospital; Harvard Medical School

RP Zhang, YS (corresponding author), Fourth Mil Med Univ, Tangdu Hosp, Dept Expt Surg, Xian 710038, Peoples R China.; Jin, WL; Cui, DX (corresponding author), Shanghai Jiao Tong Univ, Inst Nano Biomed & Engn, Sch Elect Informat & Elect Engn,Minist Educ, Dept Instrument Sci & Engn,Key Lab Thin Film & Mi, Shanghai 200240, Peoples R China.

EM weilin-jin@sjtu.edu.cn; zhangys_td@163.com; dxcui@sjtu.edu.cn

RI yang, huaju/P-1578-2015; Jin, Wei-Lin/A-8355-2013; wang,

sijia/IYJ-0510-2023; wang, zhenhui/JMQ-0550-2023

OI Jin, Wei-Lin/0000-0001-8011-2405

FU National Natural Science Foundation of China [81272801, 81272419,

81572983]; Social Development of Technology Research Projects in Shaanxi

Province [2015SF027]; Natural Scientific Foundation of Shaanxi Province

[2014JM4148]; Beijing Key Laboratory of Brain Major Diseases Open

Project

FX This work was supported by National Natural Science Foundation of China

to Weilin Jin (No. 81272801), National Natural Scientific Foundation of

China for Yongsheng Zhang (No. 81272419), National Natural Scientific

Foundation of China for Yanyang Tu (No. 81572983), Social Development of

Technology Research Projects in Shaanxi Province for Pengxing Zhang (No.

2015SF027), Natural Scientific Foundation of Shaanxi Province for

Yanyang Tu (No. 2014JM4148) and Beijing Key Laboratory of Brain Major

Diseases Open Project for Yanyang Tu (2015).

CR [Anonymous], 2015, CANC TRANSL MED, DOI [DOI 10.4103/2395-3977.155924, 10.4103/2395-3977.151470]

de Souza-Pinto NC, 2001, CANCER RES, V61, P5378

Dianov G, 1998, J BIOL CHEM, V273, P33811, DOI 10.1074/jbc.273.50.33811

Dizdaroglu M, 2012, CANCER LETT, V327, P26, DOI 10.1016/j.canlet.2012.01.016

Duarte V, 1999, NUCLEIC ACIDS RES, V27, P496, DOI 10.1093/nar/27.2.496

Fortini P, 2003, MUTAT RES-FUND MOL M, V531, P127, DOI 10.1016/j.mrfmmm.2003.07.004

Frosina G, 2009, MOL CANCER RES, V7, P989, DOI 10.1158/1541-7786.MCR-09-0030

Fujikawa K, 1999, J BIOL CHEM, V274, P18201, DOI 10.1074/jbc.274.26.18201

Gad H, 2014, NATURE, V508, P215, DOI 10.1038/nature13181

Gupta SC, 2012, ANTIOXID REDOX SIGN, V16, P1295, DOI 10.1089/ars.2011.4414

Hour MJ, 2014, AM J CANCER RES, V4, P474

Huber KVM, 2014, NATURE, V508, P222, DOI 10.1038/nature13194

Khan MI, 2012, BIOMATERIALS, V33, P1477, DOI 10.1016/j.biomaterials.2011.10.080

Liu H, 2014, MED ONCOL, V31, DOI 10.1007/s12032-014-0077-6

Maiti AK, 2014, CHEMOTHERAPY, V1, pe119

Maiti AK, 2012, INT J CANCER, V130, P1, DOI 10.1002/ijc.26306

Malecki Marek, 2008, Proc S Dak Acad Sci, V87, P249

MO JY, 1992, P NATL ACAD SCI USA, V89, P11021, DOI 10.1073/pnas.89.22.11021

Nakabeppu Y, 2001, MUTAT RES-FUND MOL M, V477, P59, DOI 10.1016/S0027-5107(01)00096-3

Nakabeppu Y, 2014, INT J MOL SCI, V15, P12543, DOI 10.3390/ijms150712543

Nakabeppu Y, 2010, MUTAT RES-GEN TOX EN, V703, P51, DOI 10.1016/j.mrgentox.2010.06.006

Omuro A, 2013, JAMA-J AM MED ASSOC, V310, P1842, DOI 10.1001/jama.2013.280319

Peng XH, 2012, THER DELIV, V3, P823, DOI 10.4155/TDE.12.61

Salles B, 2006, PATHOL BIOL, V54, P185, DOI 10.1016/j.patbio.2006.01.012

Sanchis-Gomar F, 2014, DNA REPAIR, V21, P163, DOI 10.1016/j.dnarep.2014.04.010

Saraswat V, 2002, BIOCHEMISTRY-US, V41, P15566, DOI 10.1021/bi020552p

Takagi Y, 2012, J BIOL CHEM, V287, P21541, DOI 10.1074/jbc.M112.363010

Trachootham D, 2009, NAT REV DRUG DISCOV, V8, P579, DOI 10.1038/nrd2803

Tu YY, 2013, CANCER RES, V73, P6046, DOI 10.1158/0008-5472.CAN-13-0358

Wolf A, 2010, ONCOTARGET, V1, P552, DOI 10.18632/oncotarget.190

Wu WS, 2006, CANCER METAST REV, V25, P695, DOI 10.1007/s10555-006-9037-8

Xu P, 2003, J BIOL CHEM, V278, P48422, DOI 10.1074/jbc.M307888200

Yamaguchi H, 2006, CELL DEATH DIFFER, V13, P551, DOI 10.1038/sj.cdd.4401788

Zhang WB, 2015, AM J CANCER RES, V5, P2788

NR 34

TC 31

Z9 34

U1 0

U2 13

PU E-CENTURY PUBLISHING CORP

PI MADISON

PA 40 WHITE OAKS LN, MADISON, WI 53711 USA

SN 1943-8141

J9 AM J TRANSL RES

JI Am. J. Transl. Res.

PY 2016

VL 8

IS 6

BP 2803

EP 2811

PG 9

WC Oncology; Medicine, Research & Experimental

WE Science Citation Index Expanded (SCI-EXPANDED)

SC Oncology; Research & Experimental Medicine

GA DQ0RL

UT WOS:000378907500031

PM 27398163

DA 2025-04-09

ER

PT J

AU Li, J

Zhao, YH

Tian, SF

Xu, CS

Cai, YX

Li, K

Cheng, YB

Wang, ZF

Li, ZQ

AF Li, Jie

Zhao, Yu-Hang

Tian, Su-Fang

Xu, Cheng-Shi

Cai, Yu-Xiang

Li, Kai

Cheng, Yan-Bing

Wang, Ze-Fen

Li, Zhi-Qiang

TI Genetic alteration and clonal evolution of primary glioblastoma into

secondary gliosarcoma

SO CNS NEUROSCIENCE & THERAPEUTICS

LA English

DT Article

DE clonal evolution; mutation signature; primary glioblastoma;

radiotherapy; secondary gliosarcoma

ID MULTIFORME; MUTATIONS; SURVIVAL; SARCOMA

AB Aims Secondary gliosarcoma (SGS) rarely arises post treatment of primary glioblastoma multiforme (GBM), and contains gliomatous and sarcomatous components. The origin and clonal evolution of SGS sarcomatous components remain uncharacterized. Therapeutic radiation is mutagenic and can induce sarcomas in patients with other tumor phenotypes, but possible causal relationships between radiotherapy and induction of SGS sarcomatous components remain unexplored. Herein, we investigated the clonal origin of SGS in a patient with primary GBM progressing into SGS post-radiochemotherapy. Methods Somatic mutation profile in GBM and SGS was examined using whole-genome sequencing and deep-whole-exome sequencing. Mutation signatures were characterized to investigate relationships between radiochemotherapy and SGS pathogenesis. Results A mutation cluster containing two founding mutations in tumor-suppressor genes NF1 (variant allele frequency [VAF]: 50.0% in GBM and 51.1% in SGS) and TP53 (VAF: 26.7% in GBM and 50.8% in SGS) was shared in GBM and SGS. SGS exhibited an overpresented C>A (G>T) transversion (oxidative DNA damage signature) but no signature 11 mutations (alkylating-agents - exposure signature). Since radiation induces DNA lesions by generating reactive oxygen species, the mutations observed in this case of SGS were likely the result of radiotherapy rather than chemotherapy. Conclusions Secondary gliosarcoma components likely have a monoclonal origin, and the clone possessing mutations in NF1 and TP53 was likely the founding clone in this case of SGS.

C1 [Li, Jie; Zhao, Yu-Hang; Xu, Cheng-Shi; Li, Kai; Wang, Ze-Fen; Li, Zhi-Qiang] Wuhan Univ, Zhongnan Hosp, Brain Glioma Ctr, Wuhan, Peoples R China.

[Li, Jie; Wang, Ze-Fen] Wuhan Univ, Sch Basic Med Sci, Dept Physiol, Wuhan, Peoples R China.

[Tian, Su-Fang; Cai, Yu-Xiang] Wuhan Univ, Zhongnan Hosp, Dept Pathol, Wuhan, Peoples R China.

[Cheng, Yan-Bing] Wuhan Frasergen Bioinformat Co Ltd, Wuhan, Peoples R China.

C3 Wuhan University; Wuhan University; Wuhan University

RP Wang, ZF; Li, ZQ (corresponding author), Wuhan Univ, Zhongnan Hosp, Brain Glioma Ctr, Wuhan, Peoples R China.

EM wangzf@whu.edu.cn; lizhiqiang@whu.edu.cn

RI Cai, Yuxiang/ITU-3802-2023

FU Translational Medicine Fund of the Zhongnan Hospital of Wuhan University

[ZLYNXM202011, ZNLH201901]; Medical Science Advancement Program of Wuhan

University [TFJC2018003]; National Health Commission of China

[2018ZX--07S--011]

FX This work was funded in part by the Translational Medicine Fund of the

Zhongnan Hospital of Wuhan University (No. ZLYNXM202011, ZNLH201901),

Medical Science Advancement Program of Wuhan University (No.

TFJC2018003), and the National Health Commission of China

(2018ZX--07S--011)

CR Actor B, 2002, GENE CHROMOSOME CANC, V34, P416, DOI 10.1002/gcc.10087

Alexandrov LB, 2013, NATURE, V500, P415, DOI 10.1038/nature12477

Anderson KJ, 2020, CSH MOL CASE STUD, V6, DOI 10.1101/mcs.a004671

Beaumont TL, 2007, J NEURO-ONCOL, V83, P39, DOI 10.1007/s11060-006-9295-x

BIERNAT W, 1995, J NEUROPATH EXP NEUR, V54, P651, DOI 10.1097/00005072-199509000-00006

Boerman RH, 1996, J NEUROPATH EXP NEUR, V55, P973, DOI 10.1097/00005072-199609000-00004

Boeva V, 2012, BIOINFORMATICS, V28, P423, DOI 10.1093/bioinformatics/btr670

Brennan CW, 2013, CELL, V155, P462, DOI 10.1016/j.cell.2013.09.034

Cachia D, 2015, J NEURO-ONCOL, V125, P401, DOI 10.1007/s11060-015-1930-y

Deb P, 2006, CHILD NERV SYST, V22, P314, DOI 10.1007/s00381-005-1239-9

Díaz-Gay M, 2018, BMC BIOINFORMATICS, V19, DOI 10.1186/s12859-018-2234-y

do Valle IF, 2016, BMC BIOINFORMATICS, V17, DOI 10.1186/s12859-016-1190-7

FEIGIN IH, 1955, AM J PATHOL, V31, P633

Frandsen J, 2018, J NEUROSURG, V128, P1133, DOI 10.3171/2016.12.JNS162291

Frandsen S, 2019, FRONT ONCOL, V9, DOI 10.3389/fonc.2019.01425

Galanis E, 1998, J NEUROSURG, V89, P425, DOI 10.3171/jns.1998.89.3.0425

Georgescu MM, 2020, ACTA NEUROPATHOL COM, V8, DOI 10.1186/s40478-020-0889-x

Giannini L, 2018, FRONT ONCOL, V8, DOI 10.3389/fonc.2018.00449

GRANT JW, 1989, ACTA NEUROPATHOL, V79, P305, DOI 10.1007/BF00294666

Guo Q, 2019, CNS NEUROSCI THER, V25, P343, DOI 10.1111/cns.13072

HADDAD SF, 1992, J NEUROPATH EXP NEUR, V51, P493, DOI 10.1097/00005072-199209000-00003

Han SJ, 2010, J NEURO-ONCOL, V96, P313, DOI 10.1007/s11060-009-9973-6

Han SJ, 2010, J NEUROSURG, V112, P26, DOI 10.3171/2009.3.JNS081081

Huang RY, 2020, CNS NEUROSCI THER, V26, P791, DOI 10.1111/cns.13382

Hunter C, 2006, CANCER RES, V66, P3987, DOI 10.1158/0008-5472.CAN-06-0127

KOCHI N, 1987, ACTA NEUROPATHOL, V73, P124, DOI 10.1007/BF00693777

Lee D, 2012, J NEURO-ONCOL, V107, P643, DOI 10.1007/s11060-011-0790-3

Li H, 2009, BIOINFORMATICS, V25, P1094, DOI [10.1093/bioinformatics/btp100, 10.1093/bioinformatics/btp324]

Louis DN, 2016, ACTA NEUROPATHOL, V131, P803, DOI 10.1007/s00401-016-1545-1

MEIS JM, 1991, CANCER, V67, P2342, DOI 10.1002/1097-0142(19910501)67:9<2342::AID-CNCR2820670922>3.0.CO;2-B

Pain M, 2018, ONCOTARGET, V9, P2603, DOI 10.18632/oncotarget.23517

Persi E, 2021, NAT REV GENET, V22, P251, DOI 10.1038/s41576-020-00299-4

Rausch T, 2012, BIOINFORMATICS, V28, pI333, DOI 10.1093/bioinformatics/bts378

Reis RM, 2000, AM J PATHOL, V156, P425, DOI 10.1016/S0002-9440(10)64746-3

SCHIFFER D, 1984, ACTA NEUROPATHOL, V63, P108, DOI 10.1007/BF00697192

SCHRANTZ JL, 1972, ARCH PATHOL, V93, P26

SLOWIK F, 1985, ACTA NEUROPATHOL, V67, P201, DOI 10.1007/BF00687802

Smith DR, 2018, J NEURO-ONCOL, V137, P303, DOI 10.1007/s11060-017-2718-z

SPYK SLV, 2011, HUM GENOMICS, V5, P623, DOI DOI 10.1186/1479-7364-5-6-623

Viel A, 2017, EBIOMEDICINE, V20, P39, DOI 10.1016/j.ebiom.2017.04.022

Walker C, 2001, ACTA NEUROPATHOL, V101, P321

Wang KY, 2019, CNS NEUROSCI THER, V25, P965, DOI 10.1111/cns.13137

Wojtas B, 2019, CANCERS, V11, DOI 10.3390/cancers11030284

Xiong ZJ, 2020, CNS NEUROSCI THER, V26, P981, DOI 10.1111/cns.13396

Yates A, 2016, NUCLEIC ACIDS RES, V44, pD710, DOI 10.1093/nar/gkv1157

NR 45

TC 7

Z9 7

U1 1

U2 6

PU WILEY

PI HOBOKEN

PA 111 RIVER ST, HOBOKEN 07030-5774, NJ USA

SN 1755-5930

EI 1755-5949

J9 CNS NEUROSCI THER

JI CNS Neurosci. Ther.

PD DEC

PY 2021

VL 27

IS 12

BP 1483

EP 1492

DI 10.1111/cns.13740

EA OCT 2021

PG 10

WC Neurosciences; Pharmacology & Pharmacy

WE Science Citation Index Expanded (SCI-EXPANDED)

SC Neurosciences & Neurology; Pharmacology & Pharmacy

GA XC0WM

UT WOS:000703026200001

PM 34605602

OA Green Published, gold

DA 2025-04-09

ER

PT J

AU Zhang, LW

Yan, JW

Liu, Y

Zha, QY

Di, CX

Chao, S

Jie, L

Liu, YY

Zhang, H

AF Zhang, Luwei

Yan, Jiawei

Liu, Yang

Zha, Qiuyue

Di, Cuixia

Chao, Sun

Jie, Li

Liu, Yuanyuan

Zhang, Hong

TI Contribution of caspase-independent pathway to apoptosis in malignant

glioma induced by carbon ion beams

SO ONCOLOGY REPORTS

LA English

DT Article

DE apoptosis; CIB; caspase-independent pathway; apoptosis inducing factor;

PARP-1; glioma

ID POLYMERASE-1-DEPENDENT CELL-DEATH; IONIZING-RADIATION; FACTOR AIF;

AUTOPHAGY CONTRIBUTES; CARCINOMA-CELLS; CANCER-CELLS; DNA-DAMAGE;

HELA-CELLS; INHIBITION; PARP-1

AB High linear energy transfer (LET) carbon ion beam (CIB) is becoming the best tool for external radiotherapy of inoperable tumors because of its greater cell killing than conventional low LET gamma or X-rays. In the present study, whether the caspase-independent pathway exerts the important contribution in CIB-induced cell apoptosis was explored. Herein we showed, despite the absence of caspase activity using a pan caspase inhibitor Z-VAD-FMK, that apoptosis induced by high LET CIB were clearly observed in the glioma cells. Simultaneously, the increased 8-OHdG level, PARP-1 activity and AIF translocation occurred in response to CIB irradiation. Moreover, it was distinctly higher in the nuclear translocation frequency along with PARP-1 activation when the caspase protease cascade was suppressed in the irradiated glioma cells. Nuclear colocalization between PARP-1 and AIF as well as a positive association of the PARP-1 mRNA expression with AIF translocation frequency indicated that PARP-1 activation controlled the translocation of AIF to the nucleus. Our findings strongly demonstrated that caspase-independent cell apoptosis provided a prominent compensation in the glioma cell death involving the PARP-1/AIF signaling pathway at 24 h after CIB exposure, and likely triggered by oxidative damage to DNA. The knowledge on the molecular mechanism of AIF-mediated cell death may be very useful for the improvement of the therapeutic efficacy of malignant gliomas with heavy charged particles.

C1 [Zhang, Luwei; Yan, Jiawei; Liu, Yang; Zha, Qiuyue; Di, Cuixia; Chao, Sun; Jie, Li; Liu, Yuanyuan; Zhang, Hong] Chinese Acad Sci, Inst Modern Phys, Dept Heavy Ion Radiat Med, 509 Nanchang Rd, Lanzhou 730000, Gansu, Peoples R China.

[Zhang, Luwei; Yan, Jiawei; Liu, Yang; Zha, Qiuyue; Di, Cuixia; Chao, Sun; Liu, Yuanyuan; Zhang, Hong] Chinese Acad Sci, Key Lab Heavy Ion Radiat Biol & Med, Lanzhou 730000, Gansu, Peoples R China.

[Zhang, Luwei; Liu, Yang; Zha, Qiuyue; Di, Cuixia; Chao, Sun; Liu, Yuanyuan; Zhang, Hong] Key Lab Heavy Ion Radiat Med Gansu Prov, Lanzhou 730000, Gansu, Peoples R China.

[Yan, Jiawei] Univ Chinese Acad Sci, Beijing 100039, Peoples R China.

[Jie, Li] Lanzhou Univ, Sch Stomatol, Lanzhou 730000, Gansu, Peoples R China.

C3 Chinese Academy of Sciences; Institute of Modern Physics, CAS; Chinese

Academy of Sciences; Chinese Academy of Sciences; University of Chinese

Academy of Sciences, CAS; Lanzhou University

RP Zhang, H (corresponding author), Chinese Acad Sci, Inst Modern Phys, Dept Heavy Ion Radiat Med, 509 Nanchang Rd, Lanzhou 730000, Gansu, Peoples R China.

EM zhangh@impcas.ac.cn

RI Yan, Jiawei/ADG-8967-2022; zhang, xian/JAC-5480-2023

FU Key Program of National Natural Science Foundation of China [U1432248];

Ministry of Science and Technology National Key RD Project

[2016YFC0904602]; National Natural Science Foundation of China [1120521,

11305224, 11575262]

FX This work was supported by grants from the Key Program of National

Natural Science Foundation of China (U1432248), Ministry of Science and

Technology National Key R&D Project (2016YFC0904602) and National

Natural Science Foundation of China (nos. 1120521, 11305224 and

11575262).

CR Candé C, 2004, CELL DEATH DIFFER, V11, P591, DOI 10.1038/sj.cdd.4401400

Carruthers R, 2015, CANCER DRUG DISCOV D, V83, P533, DOI 10.1007/978-3-319-14151-0_23

Chen ZT, 2015, MOL MED REP, V12, P1868, DOI 10.3892/mmr.2015.3604

Cregan SP, 2002, J CELL BIOL, V158, P507, DOI 10.1083/jcb.200202130

Cummings BS, 2004, J PHARMACOL EXP THER, V310, P126, DOI 10.1124/jpet.104.065862

Di CX, 2013, GENE, V515, P208, DOI 10.1016/j.gene.2012.11.040

Ding W, 2009, J BIOL CHEM, V284, P6809, DOI 10.1074/jbc.M805566200

Ferrand-Drake M, 2003, J NEUROCHEM, V85, P1431, DOI 10.1046/j.1471-4159.2003.01794.x

Ghorai A, 2015, APOPTOSIS, V20, P562, DOI 10.1007/s10495-015-1107-3

Hangen E, 2010, TRENDS BIOCHEM SCI, V35, P278, DOI 10.1016/j.tibs.2009.12.008

Hong SJ, 2004, TRENDS PHARMACOL SCI, V25, P259, DOI 10.1016/j.tips.2004.03.005

Jänicke RU, 2001, ONCOGENE, V20, P5043, DOI 10.1038/sj.onc.1204659

Jin W, 2016, J NEUROL SCI, V366, P8, DOI 10.1016/j.jns.2016.04.038

Jo GH, 2015, CANCER RES TREAT, V47, P221, DOI 10.4143/crt.2013.159

Joza N, 2001, NATURE, V410, P549, DOI 10.1038/35069004

Kao GD, 2007, J BIOL CHEM, V282, P21206, DOI 10.1074/jbc.M703042200

Kolthur-Seetharam U, 2006, CELL CYCLE, V5, P873, DOI 10.4161/cc.5.8.2690

Kondo K, 2010, J BIOL CHEM, V285, P13079, DOI 10.1074/jbc.M109.044206

Linsenbardt AJ, 2012, J NEUROCHEM, V122, P175, DOI 10.1111/j.1471-4159.2012.07756.x

Liu B, 2008, EUR J OBSTET GYN R B, V138, P226, DOI 10.1016/j.ejogrb.2007.08.012

Oláh G, 2015, PLOS ONE, V10, DOI 10.1371/journal.pone.0134227

Park MT, 2005, BLOOD, V105, P1724, DOI 10.1182/blood-2004-07-2938

Prabhakaran K, 2004, TOXICOL APPL PHARM, V195, P194, DOI 10.1016/j.taap.2003.11.012

Susin SA, 1999, NATURE, V397, P441, DOI 10.1038/17135

Wang YF, 2011, SCI SIGNAL, V4, DOI 10.1126/scisignal.2000902

Wieler S, 2003, J BIOL CHEM, V278, P18914, DOI 10.1074/jbc.M211641200

Yu SW, 2002, SCIENCE, V297, P259, DOI 10.1126/science.1072221

Zhang H, 2012, BRIT J DERMATOL, V166, P1100, DOI 10.1111/j.1365-2133.2011.10764.x

Zhang J, 2009, CLIN CHIM ACTA, V401, P76, DOI 10.1016/j.cca.2008.11.016

Zhang N, 2011, AUTOPHAGY, V7, P598, DOI 10.4161/auto.7.6.15103

Zhang XP, 2002, J NEUROCHEM, V82, P181, DOI 10.1046/j.1471-4159.2002.00975.x

NR 31

TC 9

Z9 10

U1 0

U2 8

PU SPANDIDOS PUBL LTD

PI ATHENS

PA POB 18179, ATHENS, 116 10, GREECE

SN 1021-335X

EI 1791-2431

J9 ONCOL REP

JI Oncol. Rep.

PD MAY

PY 2017

VL 37

IS 5

BP 2994

EP 3000

DI 10.3892/or.2017.5529

PG 7

WC Oncology

WE Science Citation Index Expanded (SCI-EXPANDED)

SC Oncology

GA ET9LR

UT WOS:000400629500052

PM 28350112

OA Bronze

DA 2025-04-09

ER

PT J

AU Klattenhoff, AW

Thakur, M

Chu, CS

Ray, D

Habib, SL

Kidane, D

AF Klattenhoff, Alex W.

Thakur, Megha

Chu, Christopher S.

Ray, Debolina

Habib, Samy L.

Kidane, Dawit

TI Loss of NEIL3 DNA glycosylase markedly increases replication associated

double strand breaks and enhances sensitivity to ATR inhibitor in

glioblastoma cells

SO ONCOTARGET

LA English

DT Article

DE DNA glycosylase; ATR; replication stress

ID BASE EXCISION-REPAIR; OXIDIZED BASES; PROTEIN-A; S-PHASE; CISPLATIN

RESISTANCE; POLYMERASE INHIBITOR; HUMAN HOMOLOG; GENOME; EXPRESSION;

DAMAGE

AB DNA endonuclease eight-like glycosylase 3 (NEIL3) is one of the DNA glycosylases that removes oxidized DNA base lesions from single-stranded DNA (ssDNA) and non-B DNA structures. Approximately seven percent of human tumors have an altered NEIL3 gene. However, the role of NEIL3 in replication-associated repair and its impact on modulating treatment response is not known. Here, we report that NEIL3 is localized at the DNA double-strand break (DSB) sites during oxidative DNA damage and replication stress. Loss of NEIL3 significantly increased spontaneous replication-associated DSBs and recruitment of replication protein A (RPA). In contrast, we observed a marked decrease in Rad51 on nascent DNA strands at the replication fork, suggesting that HR-dependent repair is compromised in NEIL3-deficient cells. Interestingly, NEIL3-deficient cells were sensitive to ataxia-telangiectasia and Rad3 related protein (ATR) inhibitor alone or in combination with PARP1 inhibitor. This study elucidates the mechanism by which NEIL3 is critical to overcome oxidative and replication-associated genotoxic stress. Our findings may have important clinical implications to utilize ATR and PARP1 inhibitors to enhance cytotoxicity in tumors that carry altered levels of NEIL3.

C1 [Klattenhoff, Alex W.; Thakur, Megha; Chu, Christopher S.; Ray, Debolina; Kidane, Dawit] Univ Texas Austin, Dell Pediat Res Inst, Coll Pharm, Div Pharmacol & Toxicol, Austin, TX 78712 USA.

[Habib, Samy L.] Univ Texas Hlth Sci Ctr San Antonio, South Texas Vet Hlth Syst, San Antonio, TX 78229 USA.

[Habib, Samy L.] Univ Texas Hlth Sci Ctr San Antonio, Dept Cellular & Struct Biol, San Antonio, TX 78229 USA.

C3 University of Texas System; University of Texas Austin; University of

Texas System; University of Texas Health Science Center at San Antonio;

University of Texas System; University of Texas Health Science Center at

San Antonio

RP Kidane, D (corresponding author), Univ Texas Austin, Dell Pediat Res Inst, Coll Pharm, Div Pharmacol & Toxicol, Austin, TX 78712 USA.

EM dawit.kidane@austin.utexas.edu

RI Kidane, Dawit/AAQ-7109-2020

OI Thakur, Megha/0000-0003-2001-0065

FU University of Texas at Austin, College of Pharmacy

FX This work is supported by the Startup Fund from The University of Texas

at Austin, College of Pharmacy. We thank Dr Karen Vasquez for critical

scientific comments and reading the manuscript, and Miss Stephanie D.

Cote for editing the manuscript.

CR Almeida KH, 2007, DNA REPAIR, V6, P695, DOI 10.1016/j.dnarep.2007.01.009

Arai K, 1997, ONCOGENE, V14, P2857, DOI 10.1038/sj.onc.1201139

Arlt MF, 2011, P NATL ACAD SCI USA, V108, P17360, DOI 10.1073/pnas.1109272108

Aspinwall R, 1997, P NATL ACAD SCI USA, V94, P109, DOI 10.1073/pnas.94.1.109

Audeh MW, 2010, LANCET, V376, P245, DOI 10.1016/S0140-6736(10)60893-8

Banerjee D, 2011, J BIOL CHEM, V286, P6006, DOI 10.1074/jbc.M110.198796

Bar-Joseph Z, 2008, P NATL ACAD SCI USA, V105, P955, DOI 10.1073/pnas.0704723105

Bekker-Jensen S, 2006, J CELL BIOL, V173, P195, DOI 10.1083/jcb.200510130

BERTONCINI CRA, 1995, NUCLEIC ACIDS RES, V23, P2995, DOI 10.1093/nar/23.15.2995

Branzei D, 2010, NAT REV MOL CELL BIO, V11, P208, DOI 10.1038/nrm2852

Bryant HE, 2005, NATURE, V434, P913, DOI 10.1038/nature03443

Chaudhuri AR, 2012, NAT STRUCT MOL BIOL, V19, P417, DOI 10.1038/nsmb.2258

CHOU TC, 1984, ADV ENZYME REGUL, V22, P27, DOI 10.1016/0065-2571(84)90007-4

Cimprich KA, 2008, NAT REV MOL CELL BIO, V9, P616, DOI 10.1038/nrm2450

Cortez D, 2001, SCIENCE, V294, P1713, DOI 10.1126/science.1065521

Cortez D, 2015, DNA REPAIR, V32, P149, DOI 10.1016/j.dnarep.2015.04.026

Couch FB, 2013, GENE DEV, V27, P1610, DOI 10.1101/gad.214080.113

Dalhus B, 2009, FEMS MICROBIOL REV, V33, P1044, DOI 10.1111/j.1574-6976.2009.00188.x

de Sousa JF, 2017, TUMOR BIOL, V39, DOI 10.1177/1010428317694552

Donahue SL, 2006, P NATL ACAD SCI USA, V103, P11642, DOI 10.1073/pnas.0510741103

Dou H, 2003, J BIOL CHEM, V278, P49679, DOI 10.1074/jbc.M308658200

Dou H, 2008, J BIOL CHEM, V283, P3130, DOI 10.1074/jbc.M709186200

Eklund H, 2001, PROG BIOPHYS MOL BIO, V77, P177, DOI 10.1016/S0079-6107(01)00014-1

Fridlich R, 2015, DNA REPAIR, V30, P11, DOI 10.1016/j.dnarep.2015.03.002

Friedel AM, 2009, CURR OPIN CELL BIOL, V21, P237, DOI 10.1016/j.ceb.2009.01.017

Gadhikar MA, 2013, MOL CANCER THER, V12, P1860, DOI 10.1158/1535-7163.MCT-13-0157

González-Prieto R, 2013, EMBO J, V32, P1307, DOI 10.1038/emboj.2013.73

Hailer MK, 2005, DNA REPAIR, V4, P41, DOI 10.1016/j.dnarep.2004.07.006

Hayashi MT, 2011, CHROMOSOMA, V120, P39, DOI 10.1007/s00412-010-0287-4

Hazra TK, 2002, J BIOL CHEM, V277, P30417, DOI 10.1074/jbc.C200355200

Hegde ML, 2008, CELL RES, V18, P27, DOI 10.1038/cr.2008.8

Hegde ML, 2008, J BIOL CHEM, V283, P27028, DOI 10.1074/jbc.M802712200

Hegde ML, 2013, P NATL ACAD SCI USA, V110, pE3090, DOI 10.1073/pnas.1304231110

Henderson PT, 2003, BIOCHEMISTRY-US, V42, P9257, DOI 10.1021/bi0347252

Henry-Mowatt J, 2003, MOL CELL, V11, P1109, DOI 10.1016/S1097-2765(03)00132-1

Higuchi K, 2003, GENES CELLS, V8, P437, DOI 10.1046/j.1365-2443.2003.00646.x

Hildrestrand GA, 2009, BMC NEUROSCI, V10, DOI 10.1186/1471-2202-10-45

Huehls AM, 2012, MOL PHARMACOL, V82, P767, DOI 10.1124/mol.112.080614

Iftode C, 1999, CRIT REV BIOCHEM MOL, V34, P141, DOI 10.1080/10409239991209255

Ikeda S, 1998, J BIOL CHEM, V273, P21585, DOI 10.1074/jbc.273.34.21585

Jacobs AL, 2012, CHROMOSOMA, V121, P1, DOI 10.1007/s00412-011-0347-4

Kang MA, 2012, CELL DEATH DIS, V3, DOI 10.1038/cddis.2011.134

Kauffmann A, 2008, ONCOGENE, V27, P565, DOI 10.1038/sj.onc.1210700

Kedar PS, 2008, DNA REPAIR, V7, P1787, DOI 10.1016/j.dnarep.2008.07.006

Kidane D, 2014, ONCOGENESIS, V3, DOI 10.1038/oncsis.2014.42

Krejci L, 2003, PROG NUCLEIC ACID RE, V74, P159, DOI 10.1016/S0079-6603(03)01013-4

Krokan HE, 1997, BIOCHEM J, V325, P1

Lebedeva NA, 2009, BIOCHEMISTRY-MOSCOW+, V74, P1278, DOI 10.1134/S0006297909110157

Liu MM, 2013, MUTAT RES-FUND MOL M, V743, P4, DOI 10.1016/j.mrfmmm.2012.12.003

Liu MM, 2010, P NATL ACAD SCI USA, V107, P4925, DOI 10.1073/pnas.0908307107

Lossaint G, 2013, MOL CELL, V51, P678, DOI 10.1016/j.molcel.2013.07.023

Massaad MJ, 2016, J CLIN INVEST, V126, P4219, DOI 10.1172/JCI85647

Middleton FK, 2015, ONCOTARGET, V6, P32396, DOI 10.18632/oncotarget.6136

Mitra S, 2002, FREE RADICAL BIO MED, V33, P15, DOI 10.1016/S0891-5849(02)00819-5

Montaner B, 2007, EMBO REP, V8, P1074, DOI 10.1038/sj.embor.7401084

Morland I, 2002, NUCLEIC ACIDS RES, V30, P4926, DOI 10.1093/nar/gkf618

Nam EA, 2011, BIOCHEM J, V436, P527, DOI 10.1042/BJ20102162

Neurauter CG, 2012, DNA REPAIR, V11, P401, DOI 10.1016/j.dnarep.2012.01.007

Noël G, 2006, MOL CANCER THER, V5, P564, DOI 10.1158/1535-7163.MCT-05-0418

Oakley GG, 2010, FRONT BIOSCI-LANDMRK, V15, P883, DOI 10.2741/3652

Petermann E, 2010, MOL CELL, V37, P492, DOI 10.1016/j.molcel.2010.01.021

Pommier Y, 2013, ACS CHEM BIOL, V8, P82, DOI 10.1021/cb300648v

Prado F, 2017, GENES-BASEL, V8, DOI 10.3390/genes8020049

Raderschall E, 1999, P NATL ACAD SCI USA, V96, P1921, DOI 10.1073/pnas.96.5.1921

Ribeyre C, 2016, CELL REP, V15, P300, DOI 10.1016/j.celrep.2016.03.027

Sage E, 2011, MUTAT RES-FUND MOL M, V711, P123, DOI 10.1016/j.mrfmmm.2010.12.010

Sangster-Guity N, 2011, ONCOGENE, V30, P2526, DOI 10.1038/onc.2010.624

Seiler JA, 2007, MOL CELL BIOL, V27, P5806, DOI 10.1128/MCB.02278-06

Sejersted Y, 2011, P NATL ACAD SCI USA, V108, P18802, DOI 10.1073/pnas.1106880108

Shibutani S, 1997, J BIOL CHEM, V272, P13916, DOI 10.1074/jbc.272.21.13916

Sirbu BM, 2011, GENE DEV, V25, P1320, DOI 10.1101/gad.2053211

Sugiyama T, 1997, J BIOL CHEM, V272, P7940, DOI 10.1074/jbc.272.12.7940

Sung P, 1997, GENE DEV, V11, P1111, DOI 10.1101/gad.11.9.1111

Toledo LI, 2013, CELL, V155, P1088, DOI 10.1016/j.cell.2013.10.043

Torisu K, 2005, J BIOCHEM, V138, P763, DOI 10.1093/jb/mvi168

Trenz K, 2006, EMBO J, V25, P1764, DOI 10.1038/sj.emboj.7601045

Tutt A, 2010, LANCET, V376, P235, DOI 10.1016/S0140-6736(10)60892-6

Vassin VM, 2009, J CELL SCI, V122, P4070, DOI 10.1242/jcs.053702

Wallace SS, 2002, FREE RADICAL BIO MED, V33, P1, DOI 10.1016/S0891-5849(02)00827-4

Wallace SS, 2013, ENVIRON MOL MUTAGEN, V54, P691, DOI 10.1002/em.21820

Ward IM, 2001, J BIOL CHEM, V276, P47759, DOI 10.1074/jbc.C100569200

Whitfield ML, 2002, MOL BIOL CELL, V13, P1977, DOI 10.1091/mbc.02-02-0030

Xu B, 2011, PLOS ONE, V6, DOI 10.1371/journal.pone.0018618

Yang YG, 2004, ONCOGENE, V23, P3872, DOI 10.1038/sj.onc.1207491

Zellweger R, 2015, J CELL BIOL, V208, P563, DOI 10.1083/jcb.201406099

Zeman MK, 2014, NAT CELL BIOL, V16, P2, DOI 10.1038/ncb2897

Zharkov DO, 2008, CELL MOL LIFE SCI, V65, P1544, DOI 10.1007/s00018-008-7543-2

Zharkov DO, 2000, J BIOL CHEM, V275, P28607, DOI 10.1074/jbc.M002441200

NR 88

TC 26

Z9 32

U1 0

U2 2

PU IMPACT JOURNALS LLC

PI ORCHARD PARK

PA 6666 E QUAKER ST, STE 1, ORCHARD PARK, NY 14127 USA

EI 1949-2553

J9 ONCOTARGET

JI Oncotarget

PD DEC 22

PY 2017

VL 8

IS 68

BP 112942

EP 112958

DI 10.18632/oncotarget.22896

PG 17

WC Oncology; Cell Biology

WE Science Citation Index Expanded (SCI-EXPANDED)

SC Oncology; Cell Biology

GA FS1WP

UT WOS:000419569800066

PM 29348879

OA Green Published, Green Submitted, gold

DA 2025-04-09

ER

PT J

AU Qu, F

Wang, P

Zhang, K

Shi, Y

Li, YX

Li, CR

Lu, JH

Liu, QH

Wang, XB

AF Qu, Fei

Wang, Pan

Zhang, Kun

Shi, Yin

Li, Yixiang

Li, Chengren

Lu, Junhan

Liu, Quanhong

Wang, Xiaobing

TI Manipulation of Mitophagy by "All-in-One" nanosensitizer augments

sonodynamic glioma therapy

SO AUTOPHAGY

LA English

DT Article

DE Blood brain barrier; mitophagy manipulation; nanosonosensitizer;

orthotopic glioma; sonodynamic therapy

ID BLOOD-BRAIN-BARRIER; BREAST-CANCER CELLS; AUTOPHAGY INHIBITION;

SINOPORPHYRIN SODIUM; DEPENDENT ROS; APOPTOSIS; DELIVERY; LIPOSOMES;

ULTRASOUND; RESISTANT

AB Limited penetration of chemotherapeutic drugs through the blood brain barrier (BBB), and the increased chemo-resistance of glioma cells due to macroautophagy/autophagy, result in high tumor recurrence and extremely limited survival of glioma patients. Ultrasound-targeted microbubble destruction (UTMD) is a technique of transient and reversible BBB disruption, which greatly facilitates intracerebral drug delivery. In addition, sonodynamic therapy (SDT) based on ultrasound stimulation and a sonosensitizer, can be a safe and noninvasive strategy for treating glioma. We innovatively designed a smart "all-in-one" nanosensitizer platform by incorporating the sonoactive chlorin e6 (Ce6) and an autophagy inhibitor-hydroxychloroquine (HCQ) into angiopep-2 peptide-modified liposomes (designated as ACHL), which integrates multiple diagnostic and therapeutic functions. ACHL selectively accumulated in the brain tumors during the optimal time-window of transient UTMD-mediated BBB opening. The nanosensitizer then responded to a second ultrasonic stimulation, and simultaneously unloaded HCQ and generated ROS in the glioma cells. The sonotherapy triggered apoptosis as well as MAPK/p38-PINK1-PRKN-dependent mitophagy, in which the antioxidant relieved the sonotoxicity and MAPK/p38 activation, while the inhibition of MAPK/p38 attenuated the progression toward mitophagy by compromising redistribution of PRKN. Moreover, HCQ blocking autophagosome degradation, augmented intracellular ROS production and resulted in an oxidative-damage regenerative loop. ACHL-SDT treatment using this construct significantly inhibited the xenograft-tumor growth and prolonged the survival time of tumor-bearing mice, exhibiting an improved therapeutic efficiency. All together, we demonstrated a precision sonotherapy with simultaneous apoptosis induction and mitophagy inhibition, which served as an intelligently strategic sense of working alongside, providing new insights into the theranostics of brain tumors.

C1 [Qu, Fei; Wang, Pan; Zhang, Kun; Shi, Yin; Li, Yixiang; Lu, Junhan; Liu, Quanhong; Wang, Xiaobing] Shaanxi Normal Univ, Minist Educ, Coll Life Sci,Natl Engn Lab Resource Dev Endanger, Key Lab Med Resources & Nat Pharmaceut Chem, Xian 710119, Shaanxi, Peoples R China.

[Li, Chengren] Army Med Univ, Chongqing Key Lab Neurobiol, Dept Histol & Embryol, Chongqing, Peoples R China.

C3 Shaanxi Normal University; Army Medical University

RP Liu, QH; Wang, XB (corresponding author), Shaanxi Normal Univ, Minist Educ, Coll Life Sci,Natl Engn Lab Resource Dev Endanger, Key Lab Med Resources & Nat Pharmaceut Chem, Xian 710119, Shaanxi, Peoples R China.

EM lshaof@snnu.edu.cn; wangxiaobing@snnu.edu.cn

FU National Natural Science Foundation of China [81972900, 81571834,

81872497]; Natural Science Foundation of Shaanxi Province [2019JZ-13,

2017KJXX-78]; Natural Science Foundation of Guangdong Province

[2017A030313651]; Fundamental Research Funds for the Central

Universities [GK201802002, 16QNGG012]

FX This work was supported by National Natural Science Foundation of China

[81972900,81571834, 81872497], the Natural Science Foundation of Shaanxi

Province [2019JZ-13, 2017KJXX-78], the Natural Science Foundation of

Guangdong Province [2017A030313651], and the Fundamental Research Funds

for the Central Universities [GK201802002, 16QNGG012].

CR Apel A, 2008, CANCER RES, V68, P1485, DOI 10.1158/0008-5472.CAN-07-0562

Basit F, 2017, CELL DEATH DIS, V8, DOI 10.1038/cddis.2017.133

Carter KA, 2014, NAT COMMUN, V5, DOI 10.1038/ncomms4546

Chen L, 2017, ULTRASON SONOCHEM, V39, P654, DOI 10.1016/j.ultsonch.2017.05.013

Cook KL, 2014, CLIN CANCER RES, V20, P3222, DOI 10.1158/1078-0432.CCR-13-3227

Dai SC, 2014, ONCOL LETT, V8, P1675, DOI 10.3892/ol.2014.2419

Dewaele M, 2010, AUTOPHAGY, V6, P838, DOI 10.4161/auto.6.7.12113

Dolado I, 2007, CANCER CELL, V11, P191, DOI 10.1016/j.ccr.2006.12.013

Dolma S, 2016, CANCER CELL, V29, P859, DOI 10.1016/j.ccell.2016.05.002

Endo-Takahashi Y, 2016, BIOL PHARM BULL, V39, P977, DOI 10.1248/bpb.b15-00994

Furre IE, 2005, CANCER RES, V65, P11051, DOI 10.1158/0008-5472.CAN-05-0510

Garcia-Cano J, 2016, FRONT CELL DEV BIOL, V4, DOI 10.3389/fcell.2016.00069

Gonzales J, 2016, J BIOMED OPT, V21, DOI 10.1117/1.JBO.21.7.078002

Gorick CM, 2018, THERANOSTICS, V8, P2988, DOI 10.7150/thno.26025

Han W, 2017, J BIOMAT SCI-POLYM E, V28, P1695, DOI 10.1080/09205063.2017.1348739

Jarauta V, 2016, CANCER LETT, V382, P1, DOI 10.1016/j.canlet.2016.08.019

Kim I, 2011, ANTIOXID REDOX SIGN, V14, P1919, DOI 10.1089/ars.2010.3768

Kim JS, 2018, J CONTROL RELEASE, V269, P245, DOI 10.1016/j.jconrel.2017.11.026

Landhuis E, 2017, NATURE, V551, P257, DOI 10.1038/d41586-017-05479-7

Lapointe S, 2018, LANCET, V392, P432, DOI 10.1016/S0140-6736(18)30990-5

Levy JMM, 2017, NAT REV CANCER, V17, P528, DOI 10.1038/nrc.2017.53

Levy JMM, 2014, CANCER DISCOV, V4, P773, DOI 10.1158/2159-8290.CD-14-0049

Li J, 2013, CHEM SOC REV, V42, P662, DOI 10.1039/c2cs35249d

Li LL, 2015, CELL MOL NEUROBIOL, V35, P615, DOI 10.1007/s10571-015-0166-x

Li Q, 2018, STEM CELL RES THER, V9, DOI 10.1186/s13287-018-1029-4

Li YX, 2018, NANO RES, V11, P1038, DOI 10.1007/s12274-017-1719-8

Li YP, 2014, NAT COMMUN, V5, DOI 10.1038/ncomms5712

Lima S, 2018, AUTOPHAGY, V14, P942, DOI 10.1080/15548627.2018.1429875

Lin TT, 2016, ACS NANO, V10, P9999, DOI 10.1021/acsnano.6b04268

Lipsman N, 2018, NAT COMMUN, V9, DOI 10.1038/s41467-018-04529-6

Liu YC, 2016, ULTRASON SONOCHEM, V31, P437, DOI 10.1016/j.ultsonch.2016.01.038

Ma NN, 2013, J NANOSCI NANOTECHNO, V13, P6485, DOI 10.1166/jnn.2013.7525

Ma XW, 2011, ACS NANO, V5, P8629, DOI 10.1021/nn202155y

Mojzisova H, 2007, BBA-BIOMEMBRANES, V1768, P2748, DOI 10.1016/j.bbamem.2007.07.002

Moreau K, 2011, CELL, V146, P303, DOI 10.1016/j.cell.2011.06.023

Palikaras K, 2018, NAT CELL BIOL, V20, P1013, DOI 10.1038/s41556-018-0176-2

Radogna F, 2016, ONCOGENE, V35, P3839, DOI 10.1038/onc.2015.455

Scherz-Shouval R, 2011, TRENDS BIOCHEM SCI, V36, P30, DOI 10.1016/j.tibs.2010.07.007

Shen J, 2013, BRIT J CANCER, V109, P164, DOI 10.1038/bjc.2013.306

Song L, 2018, CELL PHYSIOL BIOCHEM, V49, P1825, DOI 10.1159/000493629

Su XM, 2014, GEN PHYSIOL BIOPHYS, V33, P295, DOI 10.4149/gpb_2014003

Sun XY, 2012, BIOMATERIALS, V33, P916, DOI 10.1016/j.biomaterials.2011.10.035

Sun Y, 2019, BIOMATER SCI-UK, V7, P985, DOI 10.1039/c8bm01187g

Tang W, 2019, CHEM SOC REV, V48, P2967, DOI 10.1039/c8cs00805a

Torchilin VP, 2005, NAT REV DRUG DISCOV, V4, P145, DOI 10.1038/nrd1632

van Rooy I, 2011, J CONTROL RELEASE, V150, P30, DOI 10.1016/j.jconrel.2010.11.014

Vera-Ramirez L, 2018, NAT COMMUN, V9, DOI 10.1038/s41467-018-04070-6

Viale A, 2014, NATURE, V514, P628, DOI 10.1038/nature13611

Wang XB, 2018, J CONTROL RELEASE, V286, P358, DOI 10.1016/j.jconrel.2018.07.048

Wang XB, 2013, TOXICOL IN VITRO, V27, P1247, DOI 10.1016/j.tiv.2012.12.023

Wang XB, 2011, ULTRASONICS, V51, P539, DOI 10.1016/j.ultras.2010.12.001

Wang XB, 2010, ULTRASOUND MED BIOL, V36, P1933, DOI 10.1016/j.ultrasmedbio.2010.06.022

Wang Y, 2016, AUTOPHAGY, V12, P949, DOI 10.1080/15548627.2016.1162930

Wang Y, 2017, ONCOTARGET, V8, P37511, DOI 10.18632/oncotarget.16400

Wang YQ, 2012, AUTOPHAGY, V8, P1462, DOI 10.4161/auto.21211

Wu H, 2015, ANTIOXID REDOX SIGN, V22, P1032, DOI 10.1089/ars.2014.6204

Xiao B, 2017, CELL DEATH DIS, V8, DOI 10.1038/cddis.2017.463

Xiao B, 2017, J BIOL CHEM, V292, P16697, DOI 10.1074/jbc.M117.787739

Xue JW, 2017, NAT NANOTECHNOL, V12, P692, DOI [10.1038/nnano.2017.54, 10.1038/NNANO.2017.54]

Xue Q, 2015, LASER MED SCI, V30, P1967, DOI 10.1007/s10103-015-1770-1

Yan C, 2017, CANCER LETT, V388, P34, DOI 10.1016/j.canlet.2016.11.018

Yeshurun L, 2016, ULTRASOUND MED BIOL, V42, P243, DOI 10.1016/j.ultrasmedbio.2015.09.004

Zhang H, 2018, NANO LETT, V18, P4985, DOI 10.1021/acs.nanolett.8b01818

Zhang YP, 2019, BIOMED PHARMACOTHER, V111, P1315, DOI 10.1016/j.biopha.2019.01.034

Zhao BX, 2018, ONCOTARGET, V9, P4897, DOI 10.18632/oncotarget.23527

Zhou J, 2015, AUTOPHAGY, V11, P1259, DOI 10.1080/15548627.2015.1056970

Zhu P, 2018, ACS NANO, V12, P3780, DOI 10.1021/acsnano.8b00999

NR 67

TC 137

Z9 143

U1 11

U2 206

PU TAYLOR & FRANCIS INC

PI PHILADELPHIA

PA 530 WALNUT STREET, STE 850, PHILADELPHIA, PA 19106 USA

SN 1554-8627

EI 1554-8635

J9 AUTOPHAGY

JI Autophagy

PD AUG 2

PY 2020

VL 16

IS 8

BP 1413

EP 1435

DI 10.1080/15548627.2019.1687210

EA NOV 2019

PG 23

WC Cell Biology

WE Science Citation Index Expanded (SCI-EXPANDED)

SC Cell Biology

GA NC2DU

UT WOS:000495311700001

PM 31674265

OA Bronze, Green Published

DA 2025-04-09

ER

PT J

AU Haapasalo, J

Nordfors, K

Granberg, KJ

Kivioja, T

Nykter, M

Haapasalo, H

Soini, Y

AF Haapasalo, Joonas

Nordfors, Kristiina

Granberg, Kirsi J.

Kivioja, Tomi

Nykter, Matti

Haapasalo, Hannu

Soini, Ylermi

TI NRF2, DJ1 and SNRX1 and their prognostic impact in astrocytic gliomas

SO HISTOLOGY AND HISTOPATHOLOGY

LA English

DT Article

DE NRF2; DJ1; SNXR1; Glioma; Prognosis

ID ANTIOXIDANT ENZYMES; EXPRESSION; GLIOBLASTOMA; ACTIVATION; PATHWAY;

CELLS; POLYMORPHISMS; MUTATIONS; SURVIVAL

AB Nuclear factor erythroid 2-related factor 2 (NRF2), DJ1 and sulfiredoxin 1 (SRXN1) are transcription factors which protect cells from the oxidative damage caused by reactive oxygen species and, on the other hand, are associated with resistance to cancer treatments. The immunohistochemical expression of NRF2, DJ1 and SRNX 1 was assessed in human grade II-IV astrocytic gliomas. Their association to clinicopathologic and essential molecular factors was evaluated. The RNA expression levels and genetic alterations were analyzed from publicly available datasets. All studied molecules were commonly expressed. The cytoplasmic NRF2 expression was higher in tumors with a higher malignancy grade, whereas the nuclear and cytoplasmic DJ1 expression was associated with a lower grade. The presence of the isocitrate dehyrdogenase 1 mutation (IDH1) was associated with an increasing cytoplasmic and nuclear expression of NRF2 and a nuclear DJ1 expression. When primary grade IV astrocytomas were compared to secondary glioblastomas, nuclear DJ1 was associated with secondary tumors. In grade II-IV tumors, the cytoplasmic NRF2 expression was associated with a poor prognosis, whereas nuclear NRF2 and both cytoplasmic and nuclear DJ1 were associated with a better patient prognosis. Recurrent homozygous deletions of DJ1 were observed, especially in the IDH wild-type samples. When only the glioblastomas were evaluated, nuclear NRF2 and SRNX1 predicted better survival. As a conclusion, NRF2, DJ1 and SNXR1 can be used as prognosticators in gliomas.

C1 [Haapasalo, Joonas; Kivioja, Tomi] Tampere Univ Hosp, Unit Neurosurg, Teiskontie 35, FI-33521 Tampere, Finland.

[Granberg, Kirsi J.; Kivioja, Tomi; Nykter, Matti] Tampere Univ Hosp, Sci Ctr, Tampere, Finland.

[Nordfors, Kristiina] Tampere Univ Hosp, Dept Pediat, Tampere, Finland.

[Nordfors, Kristiina] Tampere Univ Hosp, Tampere Ctr Child Hlth Res, Tampere, Finland.

[Granberg, Kirsi J.; Nykter, Matti] Univ Tampere, BioMediTech, Tampere, Finland.

[Granberg, Kirsi J.; Nykter, Matti] Univ Tampere, Fac Med & Life Sci, Tampere, Finland.

[Haapasalo, Hannu] Fimlab Labs, Dept Pathol, Tampere, Finland.

[Soini, Ylermi] Univ Eastern Finland, Canc Ctr Eastern Finland, Inst Clin Med Pathol & Forens Med, Kuopio, Finland.

[Soini, Ylermi] Kuopio Univ Hosp, Dept Clin Pathol, Kuopio, Finland.

C3 Tampere University; Tampere University Hospital; Tampere University;

Tampere University Hospital; Tampere University; Tampere University

Hospital; Tampere University; Tampere University Hospital; Tampere

University; Tampere University; University of Eastern Finland; Kuopio

University Hospital; University of Eastern Finland; University of

Eastern Finland Hospital

RP Haapasalo, J (corresponding author), Tampere Univ Hosp, Unit Neurosurg, Teiskontie 35, FI-33521 Tampere, Finland.

EM joonas.haapasalo@gmail.com

RI Rautajoki, Kirsi Johanna/LYP-1650-2024

OI Rautajoki, Kirsi Johanna/0000-0001-6549-7810; Nykter,

Matti/0000-0001-6956-2843

FU Finnish Anti-Tuberculosis Association; Finnish Cancer Society; Paivikki

ja Sakari Sohlberg Foundation; Lea and Arvo Ylppo Foundation; Finnish

Pediatric Research Foundation; Finnish Medical Foundation; Competitive

State Research Financing of the Expert Responsibility area of the

Tampere University Hospital [9T042, 9U041]

FX The Finnish Anti-Tuberculosis Association (Y.S), Finnish Cancer Society

(Y.S), Paivikki ja Sakari Sohlberg Foundation (K.G.), Lea and Arvo Ylppo

Foundation (KN), the Finnish Pediatric Research Foundation (KN), The

Finnish Medical Foundation (KN) and Competitive State Research Financing

of the Expert Responsibility area of the Tampere University Hospital

(J.H., H.H., grants 9T042 and 9U041 to M.N. and J.H.) are acknowledged

for their support to the study.

CR Brennan CW, 2013, CELL, V155, P462, DOI 10.1016/j.cell.2013.09.034

Cho HY, 2015, FREE RADICAL BIO MED, V88, P362, DOI 10.1016/j.freeradbiomed.2015.06.012

Haapasalo H, 2003, BRAIN PATHOL, V13, P155

Haapasalo H, 1999, NEUROPATH APPL NEURO, V25, P134

Hartikainen JM, 2012, CANCER RES, V72, P5537, DOI 10.1158/0008-5472.CAN-12-1474

Hayes JD, 2009, TRENDS BIOCHEM SCI, V34, P176, DOI 10.1016/j.tibs.2008.12.008

Järvelä S, 2010, BMC CANCER, V10, DOI 10.1186/1471-2407-10-104

Ji XJ, 2014, INT J CANCER, V135, P574, DOI 10.1002/ijc.28699

Ji XJ, 2013, NEUROL RES, V35, P1044, DOI 10.1179/1743132813Y.0000000251

Kanamori M, 2015, NEURO-ONCOLOGY, V17, P555, DOI 10.1093/neuonc/nou282

Karihtala P, 2007, APMIS, V115, P81, DOI 10.1111/j.1600-0463.2007.apm_514.x

Kensler TW, 2010, CARCINOGENESIS, V31, P90, DOI 10.1093/carcin/bgp231

Kim YR, 2010, J PATHOL, V220, P446, DOI 10.1002/path.2653

Lau A, 2008, PHARMACOL RES, V58, P262, DOI 10.1016/j.phrs.2008.09.003

Leinonen HM, 2014, ADV CANCER RES, V122, P281, DOI 10.1016/B978-0-12-420117-0.00008-6

Li SC, 2013, NEURO-ONCOLOGY, V15, P57, DOI 10.1093/neuonc/nos261

Louis DN., 2007, WHO CLASSIFICATION T

Lu C, 2012, NATURE, V483, P474, DOI 10.1038/nature10860

Miyajima Y, 2010, ANTICANCER RES, V30, P265

Mohrenz IV, 2013, APOPTOSIS, V18, P1416, DOI 10.1007/s10495-013-0877-8

Nordfors K, 2007, CLIN NEUROPATHOL, V26, P210

Nordfors K, 2015, J CLIN PATHOL, V68, P905, DOI 10.1136/jclinpath-2015-202868

Satoh H, 2013, CANCER RES, V73, P4158, DOI 10.1158/0008-5472.CAN-12-4499

Shi JL, 2014, NEUROL SCI, V35, P839, DOI 10.1007/s10072-013-1607-2

Smith-Pearson PS, 2008, FREE RADICAL BIO MED, V45, P1178, DOI 10.1016/j.freeradbiomed.2008.07.015

Stupp R, 2009, LANCET ONCOL, V10, P459, DOI 10.1016/S1470-2045(09)70025-7

Tsai WC, 2016, INT J MOL SCI, V17, DOI 10.3390/ijms17050722

Turcan S, 2012, NATURE, V483, P479, DOI 10.1038/nature10866

Wilhelmus MMM, 2012, FREE RADICAL BIO MED, V53, P983, DOI 10.1016/j.freeradbiomed.2012.05.040

Wilson MA, 2011, ANTIOXID REDOX SIGN, V15, P111, DOI 10.1089/ars.2010.3481

You A, 2011, ARCH BIOCHEM BIOPHYS, V507, P356, DOI 10.1016/j.abb.2010.12.034

NR 31

TC 16

Z9 16

U1 0

U2 3

PU F HERNANDEZ

PI MURCIA

PA PLAZA FUENSANTA 2-7 C, 30008 MURCIA, SPAIN

SN 0213-3911

EI 1699-5848

J9 HISTOL HISTOPATHOL

JI Histol. Histopath.

PD AUG

PY 2018

VL 33

IS 8

BP 791

EP 801

DI 10.14670/HH-11-973

PG 11

WC Cell Biology; Pathology

WE Science Citation Index Expanded (SCI-EXPANDED)

SC Cell Biology; Pathology

GA GK2LG

UT WOS:000435961000005

PM 29441509

DA 2025-04-09

ER

PT J

AU Park, JW

AF Park, Jong-Whi

TI Metabolic Rewiring in Adult-Type Diffuse Gliomas

SO INTERNATIONAL JOURNAL OF MOLECULAR SCIENCES

LA English

DT Review

DE glioma metabolism; isocitrate dehydrogenases (IDH); therapeutic

strategies

ID MITOCHONDRIAL PYRUVATE CARRIER; STEM-LIKE CELLS; ISOCITRATE

DEHYDROGENASE; AEROBIC GLYCOLYSIS; GLUTAMINE-METABOLISM; THERAPEUTIC

TARGET; ENERGY-METABOLISM; OXIDATIVE DAMAGE; IDH2 MUTATIONS;

BRAIN-TUMORS

AB Multiple metabolic pathways are utilized to maintain cellular homeostasis. Given the evidence that altered cell metabolism significantly contributes to glioma biology, the current research efforts aim to improve our understanding of metabolic rewiring between glioma's complex genotype and tissue context. In addition, extensive molecular profiling has revealed activated oncogenes and inactivated tumor suppressors that directly or indirectly impact the cellular metabolism that is associated with the pathogenesis of gliomas. The mutation status of isocitrate dehydrogenases (IDHs) is one of the most important prognostic factors in adult-type diffuse gliomas. This review presents an overview of the metabolic alterations in IDH-mutant gliomas and IDH-wildtype glioblastoma (GBM). A particular focus is placed on targeting metabolic vulnerabilities to identify new therapeutic strategies for glioma.

C1 [Park, Jong-Whi] Gachon Univ, Coll BioNano Technol, Dept Life Sci, Seongnam 13120, South Korea.

[Park, Jong-Whi] Gachon Univ, GAIHST, Dept Hlth Sci & Technol, Incheon 21999, South Korea.

[Park, Jong-Whi] Gachon Univ, Neurosci Res Inst, Incheon 21565, South Korea.

C3 Gachon University; Gachon University; Gachon University

RP Park, JW (corresponding author), Gachon Univ, Coll BioNano Technol, Dept Life Sci, Seongnam 13120, South Korea.

EM jpark@gachon.ac.kr

RI Park, Jong-Whi/AAR-2991-2021

OI Park, Jong-Whi/0000-0002-9890-9241

CR Ahmad F, 2016, CELL DEATH DIS, V7, DOI 10.1038/cddis.2016.117

Ahmad F, 2017, J NEUROCHEM, V143, P671, DOI 10.1111/jnc.14152

Amary MF, 2011, J PATHOL, V224, P334, DOI 10.1002/path.2913

Azzalin A, 2017, NEOPLASIA, V19, P364, DOI 10.1016/j.neo.2017.02.009

Babic I, 2013, CELL METAB, V17, P1000, DOI 10.1016/j.cmet.2013.04.013

Badur MG, 2018, CELL REP, V25, P1018, DOI 10.1016/j.celrep.2018.09.074

Banerjee S, 2011, P NATL ACAD SCI USA, V108, P15996, DOI 10.1073/pnas.1019012108

Bao L, 2018, NUCLEIC ACIDS RES, V46, P6576, DOI 10.1093/nar/gky449

Bélanger M, 2011, CELL METAB, V14, P724, DOI 10.1016/j.cmet.2011.08.016

Bettegowda C, 2011, SCIENCE, V333, P1453, DOI 10.1126/science.1210557

Bi JF, 2021, CELL REP, V37, DOI 10.1016/j.celrep.2021.109957

Brat DJ, 2015, NEW ENGL J MED, V372, P2481, DOI 10.1056/NEJMoa1402121

Brennan CW, 2013, CELL, V155, P462, DOI 10.1016/j.cell.2013.09.034

Bruce KD, 2017, FRONT ENDOCRINOL, V8, DOI 10.3389/fendo.2017.00060

Bunse L, 2018, NAT MED, V24, P1192, DOI 10.1038/s41591-018-0095-6

Chen RH, 2014, P NATL ACAD SCI USA, V111, P14217, DOI 10.1073/pnas.1409653111

Cheng CM, 2015, CANCER CELL, V28, P569, DOI 10.1016/j.ccell.2015.09.021

Colen CB, 2011, NEOPLASIA, V13, P620, DOI 10.1593/neo.11134

Cosset É, 2017, CANCER CELL, V32, P856, DOI 10.1016/j.ccell.2017.10.016

Dang CV, 2013, CSH PERSPECT MED, V3, DOI 10.1101/cshperspect.a014217

Dang L, 2009, NATURE, V462, P739, DOI 10.1038/nature08617

DeBerardinis RJ, 2007, P NATL ACAD SCI USA, V104, P19345, DOI 10.1073/pnas.0709747104

Dias-Santagata D, 2011, PLOS ONE, V6, DOI 10.1371/journal.pone.0017948

Dolinska M, 2003, NEUROCHEM INT, V43, P501, DOI 10.1016/S0197-0186(03)00040-8

Duan Y, 2013, ONCOTARGETS THER, V6, P189, DOI 10.2147/OTT.S40992

Dwarakanath BS, 2009, J CANCER RES THER, V5, P21, DOI 10.4103/0973-1482.55136

Elhammali A, 2014, CANCER DISCOV, V4, P828, DOI 10.1158/2159-8290.CD-13-0572

Elstrom RL, 2004, CANCER RES, V64, P3892, DOI 10.1158/0008-5472.CAN-03-2904

Fack F, 2017, EMBO MOL MED, V9, P1681, DOI 10.15252/emmm.201707729

Fu XD, 2015, CELL METAB, V22, P508, DOI 10.1016/j.cmet.2015.06.009

Garrett M, 2018, CANCER METAB, V6, DOI 10.1186/s40170-018-0177-4

Gimple RC, 2019, CANCER DISCOV, V9, P1248, DOI 10.1158/2159-8290.CD-19-0061

Gini B, 2013, CLIN CANCER RES, V19, P5722, DOI 10.1158/1078-0432.CCR-13-0527

Goyal MS, 2018, J PEDIATR GASTR NUTR, V66, pS46, DOI 10.1097/MPG.0000000000001875

Grassian AR, 2014, CANCER RES, V74, P3317, DOI 10.1158/0008-5472.CAN-14-0772-T

Gu YC, 2017, MOL CELL, V67, P128, DOI 10.1016/j.molcel.2017.05.030

Guo DL, 2009, SCI SIGNAL, V2, DOI 10.1126/scisignal.2000446

Hanahan D, 2022, CANCER DISCOV, V12, P31, DOI 10.1158/2159-8290.CD-21-1059

Hartmann C, 2009, ACTA NEUROPATHOL, V118, P469, DOI 10.1007/s00401-009-0561-9

Heiden MGV, 2009, SCIENCE, V324, P1029, DOI 10.1126/science.1160809

Hollinshead KER, 2018, CELL REP, V22, P3107, DOI 10.1016/j.celrep.2018.02.084

Hu J, 2016, CANCER CELL, V29, P49, DOI 10.1016/j.ccell.2015.12.005

HURLEY JH, 1991, BIOCHEMISTRY-US, V30, P8671, DOI 10.1021/bi00099a026

Intlekofer AM, 2015, CELL METAB, V22, P304, DOI 10.1016/j.cmet.2015.06.023

Ishida CT, 2018, CLIN CANCER RES, V24, P5392, DOI 10.1158/1078-0432.CCR-18-1040

Israelsen WJ, 2013, CELL, V155, P397, DOI 10.1016/j.cell.2013.09.025

Jing XM, 2019, MOL CANCER, V18, DOI 10.1186/s12943-019-1089-9

Jo SH, 2001, J BIOL CHEM, V276, P16168, DOI 10.1074/jbc.M010120200

Kang MK, 2007, STEM CELLS DEV, V16, P837, DOI 10.1089/scd.2007.0006

Kant S, 2020, CELL DEATH DIS, V11, DOI 10.1038/s41419-020-2449-5

Karsy M, 2019, J NEUROSURG, V130, P56, DOI 10.3171/2017.9.JNS172036

Kefas B, 2010, NEURO-ONCOLOGY, V12, P1102, DOI 10.1093/neuonc/noq080

Kickingereder P, 2015, SCI REP-UK, V5, DOI 10.1038/srep16238

Kim MM, 2016, NAT REV CLIN ONCOL, V13, P725, DOI 10.1038/nrclinonc.2016.108

Klemm F, 2020, CELL, V181, P1643, DOI 10.1016/j.cell.2020.05.007

Koch K, 2020, CELL DEATH DISCOV, V6, DOI 10.1038/s41420-020-0258-3

Koh HJ, 2004, J BIOL CHEM, V279, P39968, DOI 10.1074/jbc.M402260200

Koivunen P, 2012, NATURE, V483, P485, DOI 10.1038/nature10898

Kölker S, 2002, EUR J NEUROSCI, V16, P21, DOI 10.1046/j.1460-9568.2002.02055.x

Kruiswijk F, 2015, NAT REV MOL CELL BIO, V16, P393, DOI 10.1038/nrm4007

Kucharzewska P, 2015, PLOS ONE, V10, DOI 10.1371/journal.pone.0116740

Latini A, 2005, MOL GENET METAB, V86, P188, DOI 10.1016/j.ymgme.2005.05.002

Lee SM, 2002, FREE RADICAL BIO MED, V32, P1185, DOI 10.1016/S0891-5849(02)00815-8

Lee Y, 2017, ACTA NEUROPATHOL COM, V5, DOI 10.1186/s40478-017-0465-1

Lenting K, 2019, FASEB J, V33, P557, DOI 10.1096/fj.201800907RR

Lita A, 2021, NAT COMMUN, V12, DOI 10.1038/s41467-020-20752-6

Liu YY, 2016, BIOCHEM BIOPH RES CO, V480, P415, DOI 10.1016/j.bbrc.2016.10.064

Lloyd RE, 2015, NEURO-ONCOLOGY, V17, P942, DOI 10.1093/neuonc/nov020

Lunt SY, 2011, ANNU REV CELL DEV BI, V27, P441, DOI 10.1146/annurev-cellbio-092910-154237

Luoto S, 2018, CANCER RES, V78, P5574, DOI 10.1158/0008-5472.CAN-17-3714

Mai WX, 2017, NAT MED, V23, P1342, DOI 10.1038/nm.4418

Mair R, 2018, CANCER RES, V78, P5408, DOI 10.1158/0008-5472.CAN-18-0759

Mardis ER, 2009, NEW ENGL J MED, V361, P1058, DOI 10.1056/NEJMoa0903840

Marin-Valencia I, 2012, CELL METAB, V15, P827, DOI 10.1016/j.cmet.2012.05.001

McBrayer SK, 2018, CELL, V175, P101, DOI 10.1016/j.cell.2018.08.038

Michelakis ED, 2010, SCI TRANSL MED, V2, DOI 10.1126/scitranslmed.3000677

Miller JJ, 2021, NEURO-ONCOLOGY, V23, P53, DOI 10.1093/neuonc/noaa180

Molina JR, 2018, NAT MED, V24, P1036, DOI 10.1038/s41591-018-0052-4

Monteiro AR, 2017, CELLS-BASEL, V6, DOI 10.3390/cells6040045

Mudassar F, 2020, J EXP CLIN CANC RES, V39, DOI 10.1186/s13046-020-01724-6

Navis AC, 2013, ACTA NEUROPATHOL COM, V1, DOI 10.1186/2051-5960-1-18

Ohba S, 2016, CANCER RES, V76, P6680, DOI 10.1158/0008-5472.CAN-16-0696

Oliva CR, 2010, J BIOL CHEM, V285, P39759, DOI 10.1074/jbc.M110.147504

Ostrom QT, 2020, NEURO-ONCOLOGY, V22, P1, DOI 10.1093/neuonc/noaa200

Pang B, 2016, ONCOTARGET, V7, P45134, DOI 10.18632/oncotarget.9761

Park JW, 2023, ACTA NEUROPATHOL COM, V11, DOI 10.1186/s40478-023-01507-y

Parsons DW, 2008, SCIENCE, V321, P1807, DOI 10.1126/science.1164382

Philip B, 2018, CELL REP, V23, P1553, DOI 10.1016/j.celrep.2018.03.133

Plas David R, 2002, Trends Endocrinol Metab, V13, P75, DOI 10.1016/S1043-2760(01)00528-8

Prabhu A, 2015, NEURO-ONCOLOGY, V17, P1220, DOI 10.1093/neuonc/nou369

Reitman ZJ, 2011, P NATL ACAD SCI USA, V108, P3270, DOI 10.1073/pnas.1019393108

Robert SM, 2015, SCI TRANSL MED, V7, DOI 10.1126/scitranslmed.aaa8103

Ros S, 2013, CANCER METAB, V1, DOI 10.1186/2049-3002-1-8

Ru P, 2016, CELL REP, V16, P1527, DOI 10.1016/j.celrep.2016.07.017

Ruban A, 2012, INVEST NEW DRUG, V30, P2226, DOI [10.1007/s10637-012-9794-x, 10.1007/s10637-012-9799-5]

Ryall S, 2020, CANCER CELL, V37, P569, DOI 10.1016/j.ccell.2020.03.011

Schell JC, 2014, MOL CELL, V56, P400, DOI 10.1016/j.molcel.2014.09.026

Sekhar KR, 2002, ONCOGENE, V21, P6829, DOI 10.1038/sj.onc.1205905

Seltzer MJ, 2010, CANCER RES, V70, P8981, DOI 10.1158/0008-5472.CAN-10-1666

Shah SS, 2019, CANCERS, V11, DOI 10.3390/cancers11020159

Suzuki H, 2015, NAT GENET, V47, P458, DOI 10.1038/ng.3273

Taguchi K, 2011, GENES CELLS, V16, P123, DOI 10.1111/j.1365-2443.2010.01473.x

Tanaka K, 2015, J CLIN INVEST, V125, P1591, DOI 10.1172/JCI78239

Tardito S, 2015, NAT CELL BIOL, V17, P1556, DOI 10.1038/ncb3272

Tateishi K, 2016, CLIN CANCER RES, V22, P4452, DOI 10.1158/1078-0432.CCR-15-2274

Tateishi K, 2015, CANCER CELL, V28, P773, DOI 10.1016/j.ccell.2015.11.006

Taylor SR, 2022, NAT REV CANCER, V22, P452, DOI 10.1038/s41568-022-00485-y

Turcan S, 2018, NAT GENET, V50, P62, DOI 10.1038/s41588-017-0001-z

Venkataramani V, 2019, NATURE, V573, P532, DOI 10.1038/s41586-019-1564-x

Venkatesh HS, 2019, NATURE, V573, P539, DOI 10.1038/s41586-019-1563-y

Venneti S, 2017, ANNU REV PATHOL-MECH, V12, P515, DOI 10.1146/annurev-pathol-012615-044329

Venneti S, 2015, SCI TRANSL MED, V7, DOI 10.1126/scitranslmed.aaa1009

Veys K, 2020, CIRC RES, V127, P466, DOI 10.1161/CIRCRESAHA.119.316463

Villa GR, 2016, CANCER CELL, V30, P683, DOI 10.1016/j.ccell.2016.09.008

Wang P, 2013, ONCOGENE, V32, P3091, DOI 10.1038/onc.2012.315

WARBURG O, 1956, SCIENCE, V123, P309, DOI 10.1126/science.123.3191.309

Ward PS, 2012, CANCER CELL, V21, P297, DOI 10.1016/j.ccr.2012.02.014

Ward PS, 2010, CANCER CELL, V17, P225, DOI 10.1016/j.ccr.2010.01.020

Weller M, 2015, NAT REV DIS PRIMERS, V1, DOI 10.1038/nrdp.2015.17

Wise DR, 2008, P NATL ACAD SCI USA, V105, P18782, DOI 10.1073/pnas.0810199105

Wolf A, 2011, NEUROBIOL DIS, V44, P84, DOI 10.1016/j.nbd.2011.06.007

Wolf A, 2011, J EXP MED, V208, P313, DOI 10.1084/jem.20101470

Wu G, 2012, NAT GENET, V44, P251, DOI 10.1038/ng.1102

Wu XN, 2020, ISCIENCE, V23, DOI 10.1016/j.isci.2020.101569

Xu W, 2011, CANCER CELL, V19, P17, DOI 10.1016/j.ccr.2010.12.014

Yan H, 2009, NEW ENGL J MED, V360, P765, DOI 10.1056/NEJMoa0808710

Yang WW, 2012, CELL, V150, P685, DOI 10.1016/j.cell.2012.07.018

Yang WW, 2011, NATURE, V480, P118, DOI 10.1038/nature10598

Yuan SQ, 2013, STEM CELLS, V31, P23, DOI 10.1002/stem.1273

Zeng QQ, 2019, NATURE, V573, P526, DOI 10.1038/s41586-019-1576-6

Zhang LJ, 2018, CLIN CANCER RES, V24, P5381, DOI 10.1158/1078-0432.CCR-17-3855

Zhao SM, 2009, SCIENCE, V324, P261, DOI 10.1126/science.1170944

NR 132

TC 3

Z9 3

U1 0

U2 6

PU MDPI

PI BASEL

PA ST ALBAN-ANLAGE 66, CH-4052 BASEL, SWITZERLAND

SN 1661-6596

EI 1422-0067

J9 INT J MOL SCI

JI Int. J. Mol. Sci.

PD APR

PY 2023

VL 24

IS 8

AR 7348

DI 10.3390/ijms24087348

PG 16

WC Biochemistry & Molecular Biology; Chemistry, Multidisciplinary

WE Science Citation Index Expanded (SCI-EXPANDED)

SC Biochemistry & Molecular Biology; Chemistry

GA E7SE3

UT WOS:000977490000001

PM 37108511

OA Green Published, gold

DA 2025-04-09

ER

PT J

AU Deighton, RF

Le Bihan, T

Martin, SF

Gerth, AMJ

McCulloch, M

Edgar, JM

Kerr, LE

Whittle, IR

McCulloch, J

AF Deighton, Ruth F.

Le Bihan, Thierry

Martin, Sarah F.

Gerth, Alice M. J.

McCulloch, Mailis

Edgar, Julia M.

Kerr, Lorraine E.

Whittle, Ian R.

McCulloch, James

TI Interactions among mitochondrial proteins altered in glioblastoma

SO JOURNAL OF NEURO-ONCOLOGY

LA English

DT Article

DE Glioblastoma; Mitochondria; Clinical proteomics

ID KAPPA-B ACTIVATION; GLIOMA PATHOPHYSIOLOGY; CANCER; OVEREXPRESSION;

PEROXIREDOXIN; MUTATIONS; ALPHA; IDENTIFICATION; BIOENERGETICS;

THIOREDOXIN

AB Mitochondrial dysfunction is putatively central to glioblastoma (GBM) pathophysiology but there has been no systematic analysis in GBM of the proteins which are integral to mitochondrial function. Alterations in proteins in mitochondrial enriched fractions from patients with GBM were defined with label-free liquid chromatography mass spectrometry. 256 mitochondrially-associated proteins were identified in mitochondrial enriched fractions and 117 of these mitochondrial proteins were markedly (fold-change a parts per thousand yen2) and significantly altered in GBM (p a parts per thousand currency sign 0.05). Proteins associated with oxidative damage (including catalase, superoxide dismutase 2, peroxiredoxin 1 and peroxiredoxin 4) were increased in GBM. Protein-protein interaction analysis highlighted a reduction in multiple proteins coupled to energy metabolism (in particular respiratory chain proteins, including 23 complex-I proteins). Qualitative ultrastructural analysis in GBM with electron microscopy showed a notably higher prevalence of mitochondria with cristolysis in GBM. This study highlights the complex mitochondrial proteomic adjustments which occur in GBM pathophysiology.

C1 [Deighton, Ruth F.] Univ Edinburgh, Sch Biomed Sci, Ctr Integrat Physiol, Edinburgh EH8 9XD, Midlothian, Scotland.

[Le Bihan, Thierry; Martin, Sarah F.; Kerr, Lorraine E.] Univ Edinburgh, SynthSys Synthet & Syst Biol, Edinburgh EH8 9XD, Midlothian, Scotland.

[Gerth, Alice M. J.; McCulloch, James] Univ Edinburgh, Ctr Cognit & Neural Syst, Edinburgh EH8 9XD, Midlothian, Scotland.

[Edgar, Julia M.; McCulloch, James] Univ Glasgow, Coll Med Vet & Life Sci, Appl Neurobiol Grp, Inst Infect Immun & Inflammat, Glasgow, Lanark, Scotland.

[Whittle, Ian R.] Western Gen Hosp, Dept Clin Neurosci, Edinburgh EH4 2XU, Midlothian, Scotland.

C3 University of Edinburgh; University of Edinburgh; University of

Edinburgh; University of Glasgow; University of Edinburgh

RP Deighton, RF (corresponding author), Univ Edinburgh, Sch Biomed Sci, Ctr Integrat Physiol, Hugh Robson Bldg, Edinburgh EH8 9XD, Midlothian, Scotland.

EM ruth.deighton@ed.ac.uk

RI Le Bihan, Thierry/AAN-2534-2020; Edgar, Julia/U-8456-2019; whittle,

ian/AAX-7309-2021

OI Edgar, Julia/0000-0002-3869-0962; Le Bihan, Thierry/0000-0003-0498-8063;

Whittle, Ian/0000-0002-6074-0639; Gerth, Alice/0000-0002-9200-9154

FU Melville Trust; Brain Tumour Research Fund; Synthsys; BBSRC; EPSRC

[BB/D019621/1]; BBSRC [BB/D019621/1] Funding Source: UKRI

FX This work was supported in part by Grants from The Melville Trust and

Brain Tumour Research Fund. RD is the Melville Trust Research Fellow.

TLB, SFM, and LEK are funded by Synthsys which is a Centre for

Integrative Systems Biology (CISB) funded by BBSRC and EPSRC; reference

BB/D019621/1.

CR Acharya A, 2010, OXID MED CELL LONGEV, V3, P23, DOI 10.4161/oxim.3.1.10095

Alphey MS, 2005, J BIOL CHEM, V280, P3068, DOI 10.1074/jbc.M411069200

Anderson E, 2008, BRIT J NEUROSURG, V22, P339, DOI 10.1080/02688690701885603

Barsnes H, 2009, NAT BIOTECHNOL, V27, P598, DOI 10.1038/nbt0709-598

Benard G, 2008, ANTIOXID REDOX SIGN, V10, P1313, DOI 10.1089/ars.2007.2000

Brigelius-Flohé R, 2009, BBA-GEN SUBJECTS, V1790, P1555, DOI 10.1016/j.bbagen.2009.03.006

Sempere MC, 2008, CLIN TRANSL ONCOL, V10, P143, DOI 10.1007/s12094-008-0171-3

Cuezva JM, 2009, BBA-MOL BASIS DIS, V1792, P1145, DOI 10.1016/j.bbadis.2009.01.006

Cullen ME, 2006, CIRCULATION, V114, pI16, DOI 10.1161/CIRCULATIONAHA.105.000448

Dang LN, 2010, TRENDS MOL MED, V16, P387, DOI 10.1016/j.molmed.2010.07.002

Darsigny M, 2010, CANCER RES, V70, P9423, DOI 10.1158/0008-5472.CAN-10-1697

Deighton RF, 2010, BRAIN PATHOL, V20, P691, DOI 10.1111/j.1750-3639.2010.00376.x

Deighton RF, 2010, PROTEOMICS, V10, P1307, DOI 10.1002/pmic.200900112

Dennis G, 2003, GENOME BIOL, V4, DOI 10.1186/gb-2003-4-9-r60

Forner F, 2006, J PROTEOME RES, V5, P3277, DOI 10.1021/pr060361z

Fu YJ, 2011, BIOCHEM BIOPH RES CO, V410, P218, DOI 10.1016/j.bbrc.2011.05.108

Furnari FB, 2007, GENE DEV, V21, P2683, DOI 10.1101/gad.1596707

Gallegos AM, 2001, PROG LIPID RES, V40, P498, DOI 10.1016/S0163-7827(01)00015-7

GRIFFITHS IR, 1981, J NEUROCYTOL, V10, P847, DOI 10.1007/BF01262657

Griguer CE, 2011, CURR PHARM DESIGN, V17, P2421

Hempel N, 2011, ANTI-CANCER AGENT ME, V11, P191, DOI 10.2174/187152011795255911

Herrmann A, 2011, J NEURO-ONCOL, V104, P401, DOI 10.1007/s11060-010-0526-9

Herrmann AG, 2013, J CEREBR BLOOD F MET, V33, P673, DOI 10.1038/jcbfm.2012.204

Huang DW, 2009, NAT PROTOC, V4, P44, DOI 10.1038/nprot.2008.211

James R, 2012, J CEREBR BLOOD F MET, V32, P164, DOI 10.1038/jcbfm.2011.120

Jin DY, 1997, J BIOL CHEM, V272, P30952, DOI 10.1074/jbc.272.49.30952

Kanekura T, 2010, J DERMATOL SCI, V57, P149, DOI 10.1016/j.jdermsci.2009.12.008

Kang DW, 2011, CANCER RES, V71, P293, DOI 10.1158/0008-5472.CAN-10-2463

Kinnula VL, 2002, J PATHOL, V196, P316, DOI 10.1002/path.1042

Le Bihan T, 2010, RAPID COMMUN MASS SP, V24, P1093, DOI 10.1002/rcm.4487

Lee HJ, 2007, NAT CELL BIOL, V9, P1303, DOI 10.1038/ncb1650

Li ZY, 2011, BIOCHEM BIOPH RES CO, V414, P5, DOI 10.1016/j.bbrc.2011.09.046

Lüpertz R, 2008, J CELL BIOCHEM, V103, P1497, DOI 10.1002/jcb.21538

Miller C, 2011, BBA-MOL BASIS DIS, V1812, P625, DOI 10.1016/j.bbadis.2011.01.013

Moreno-Sánchez R, 2009, BIOFACTORS, V35, P209, DOI 10.1002/biof.31

Nikiforov MA, 2002, MOL CELL BIOL, V22, P5793, DOI 10.1128/MCB.22.16.5793-5800.2002

Noh DY, 2001, ANTICANCER RES, V21, P2085

Ordys BB, 2010, MOL NEUROBIOL, V42, P64, DOI 10.1007/s12035-010-8133-5

Park JH, 2006, RESPIROLOGY, V11, P269, DOI 10.1111/j.1440-1843.2006.00849.x

Payton JE, 2004, J MOL BIOL, V337, P1001, DOI 10.1016/j.jmb.2004.02.014

Rich PR, 2010, ESSAYS BIOCHEM, V47, P1, DOI [10.1042/bse0470001, 10.1042/BSE0470001]

Sacksteder KA, 2000, AM J HUM GENET, V66, P1736, DOI 10.1086/302919

Seyfried Thomas N, 2005, Nutr Metab (Lond), V2, P30, DOI 10.1186/1743-7075-2-30

Shaw RJ, 2006, CURR OPIN CELL BIOL, V18, P598, DOI 10.1016/j.ceb.2006.10.005

Tang W, 2004, MOL BIOL CELL, V15, P4043, DOI 10.1091/mbc.e04-05-0402

THORPE C, 1995, FASEB J, V9, P718, DOI 10.1096/fasebj.9.9.7601336

Venkatesan B, 2010, J MOL CELL CARDIOL, V49, P655, DOI 10.1016/j.yjmcc.2010.05.007

Warburg O., 1931, METABOLISM TUMOURS

Yan H, 2009, NEW ENGL J MED, V360, P765, DOI 10.1056/NEJMoa0808710

Yang ZR, 2002, STRUCTURE, V10, P951, DOI 10.1016/S0969-2126(02)00788-8

Ziegler DS, 2008, J CLIN ONCOL, V26, P493, DOI 10.1200/JCO.2007.13.9717

NR 51

TC 59

Z9 66

U1 0

U2 7

PU SPRINGER

PI NEW YORK

PA 233 SPRING ST, NEW YORK, NY 10013 USA

SN 0167-594X

EI 1573-7373

J9 J NEURO-ONCOL

JI J. Neuro-Oncol.

PD JUN

PY 2014

VL 118

IS 2

BP 247

EP 256

DI 10.1007/s11060-014-1430-5

PG 10

WC Oncology; Clinical Neurology

WE Science Citation Index Expanded (SCI-EXPANDED)

SC Oncology; Neurosciences & Neurology

GA AI6YN

UT WOS:000337024300004

PM 24728830

OA hybrid, Green Published

DA 2025-04-09

ER

PT J

AU Kim, SY

Yoo, YH

Park, JW

AF Kim, Sung Youl

Yoo, Young Hyun

Park, Jeen-Woo

TI Silencing of mitochondrial NADP<SUP>+</SUP>-dependent isocitrate

dehydrogenase gene enhances glioma radiosensitivity

SO BIOCHEMICAL AND BIOPHYSICAL RESEARCH COMMUNICATIONS

LA English

DT Article

DE Ionizing radiation; Glioma; Autophagy; Apoptosis; Redox status

ID INDUCED APOPTOSIS; AUTOPHAGY; CANCER; PATHWAY; RADIATION

AB Reactive oxygen species (ROS) levels are elevated in organisms that have been exposed to ionizing radiation and are protagonists in the induction of cell death. Recently, we demonstrated that the control of mitochondrial redox balance and the cellular defense against oxidative damage are primary functions of mitochondrial NADP(+)-dependent isocitrate dehydrogenase (IDPm) via the supply of NADPH for antioxidant systems. In the present study, we report an autophagic response to ionizing radiation in A172 glioma cells transfected with small interfering RNA (siRNA) targeting the IDPm gene. Autophagy in A172 transfectant cells was associated with enhanced autophagolysosome formation and GFP-LC3 punctuation/aggregation. Furthermore, we found that the inhibition of autophagy by chloroquine augmented apoptotic cell death of irradiated A172 cells transfected with IDPm siRNA. Taken together, our data suggest that autophagy functions as a survival mechanism in A172 cells against ionizing radiation-induced apoptosis and the sensitizing effect of IDPm siRNA and autophagy inhibitor on the ionizing radiation-induced apoptotic cell death of glioma cells offers a novel redox-active therapeutic strategy for the treatment of cancer. (C) 2013 Elsevier Inc. All rights reserved.

C1 [Kim, Sung Youl; Park, Jeen-Woo] Kyungpook Natl Univ, Coll Nat Sci, Sch Life Sci & Biotechnol, Taegu 702701, South Korea.

[Yoo, Young Hyun] Dong A Univ, Coll Med, Mitochondria Hub Regulat Ctr, Pusan, South Korea.

C3 Kyungpook National University (KNU); Dong A University

RP Park, JW (corresponding author), Kyungpook Natl Univ, Coll Nat Sci, Sch Life Sci & Biotechnol, Taegu 702701, South Korea.

EM parkjw@knu.ac.kr

RI park, jun yeon/GPX-5293-2022

FU National Research Foundation (NRF) of Korea; Korean Government (MEST)

[2011-0025802, 2012-0000891, 2012-0004881]

FX This research was supported by National Research Foundation (NRF) of

Korea Grant funded by the Korean Government (MEST) (2011-0025802,

2012-0000891 and 2012-0004881).

CR Ahmed KM, 2008, FREE RADICAL BIO MED, V44, P1, DOI 10.1016/j.freeradbiomed.2007.09.022

Amaravadi RK, 2007, CLIN CANCER RES, V13, P7271, DOI 10.1158/1078-0432.CCR-07-1595

Botti J, 2006, AUTOPHAGY, V2, P67, DOI 10.4161/auto.2.2.2458

Camphausen K, 2004, CANCER BIOL THER, V3, P247, DOI 10.4161/cbt.3.3.544

Cantley LC, 2002, SCIENCE, V296, P1655, DOI 10.1126/science.296.5573.1655

CERUTTI PA, 1985, SCIENCE, V227, P375, DOI 10.1126/science.2981433

Elbashir SM, 2001, NATURE, V411, P494, DOI 10.1038/35078107

Elliott A, 2008, TOXICOL APPL PHARM, V232, P169, DOI 10.1016/j.taap.2008.06.017

EWING D, 1987, ARCH BIOCHEM BIOPHYS, V254, P53, DOI 10.1016/0003-9861(87)90080-4

Fisher CJ, 2008, CANCER BIOL THER, V7, P1271, DOI 10.4161/cbt.7.8.6300

Goussetis DJ, 2010, J BIOL CHEM, V285, P29989, DOI 10.1074/jbc.M109.090530

Hennessy BT, 2005, NAT REV DRUG DISCOV, V4, P988, DOI 10.1038/nrd1902

Herman-Antosiewicz A, 2006, CANCER RES, V66, P5828, DOI 10.1158/0008-5472.CAN-06-0139

Hippert MM, 2006, CANCER RES, V66, P9349, DOI 10.1158/0008-5472.CAN-06-1597

Jo SH, 2001, J BIOL CHEM, V276, P16168, DOI 10.1074/jbc.M010120200

Kabeya Y, 2000, EMBO J, V19, P5720, DOI 10.1093/emboj/19.21.5720

Kirsch M, 2001, FASEB J, V15, P1569, DOI 10.1096/fj.00-0823hyp

Kondo Y, 2005, NAT REV CANCER, V5, P726, DOI 10.1038/nrc1692

Lee JH, 2007, J BIOL CHEM, V282, P13385, DOI 10.1074/jbc.M700303200

Levine B, 2004, DEV CELL, V6, P463, DOI 10.1016/S1534-5807(04)00099-1

Levine B, 2008, CELL, V132, P27, DOI 10.1016/j.cell.2007.12.018

Levine B, 2007, NATURE, V446, P745, DOI 10.1038/446745a

Liang JY, 2007, NAT CELL BIOL, V9, P218, DOI 10.1038/ncb1537

Longo L, 2008, MOL CANCER THER, V7, P2476, DOI 10.1158/1535-7163.MCT-08-0361

Louis DN, 2007, ACTA NEUROPATHOL, V114, P547, DOI 10.1007/s00401-007-0278-6

Lu Yiling, 2003, Rev Clin Exp Hematol, V7, P205

Mathew R, 2007, NAT REV CANCER, V7, P961, DOI 10.1038/nrc2254

Maycotte P, 2012, AUTOPHAGY, V8, P200, DOI 10.4161/auto.8.2.18554

Mikkelsen RB, 2003, ONCOGENE, V22, P5734, DOI 10.1038/sj.onc.1206663

Morgensztern D, 2005, ANTI-CANCER DRUG, V16, P797, DOI 10.1097/01.cad.0000173476.67239.3b

Nakamura H, 2005, ANTIOXID REDOX SIGN, V7, P823, DOI 10.1089/ars.2005.7.823

Nicholson KM, 2002, CELL SIGNAL, V14, P381, DOI 10.1016/S0898-6568(01)00271-6

Sevrioukova IF, 2011, ANTIOXID REDOX SIGN, V14, P2545, DOI 10.1089/ars.2010.3445

Tsuboi Y, 2009, J NEUROSURG, V110, P594, DOI 10.3171/2008.8.JNS17648

Vivanco I, 2002, NAT REV CANCER, V2, P489, DOI 10.1038/nrc839

NR 35

TC 12

Z9 12

U1 0

U2 12

PU ACADEMIC PRESS INC ELSEVIER SCIENCE

PI SAN DIEGO

PA 525 B ST, STE 1900, SAN DIEGO, CA 92101-4495 USA

SN 0006-291X

EI 1090-2104

J9 BIOCHEM BIOPH RES CO

JI Biochem. Biophys. Res. Commun.

PD APR 5

PY 2013

VL 433

IS 2

BP 260

EP 265

DI 10.1016/j.bbrc.2013.02.093

PG 6

WC Biochemistry & Molecular Biology; Biophysics

WE Science Citation Index Expanded (SCI-EXPANDED)

SC Biochemistry & Molecular Biology; Biophysics

GA 123FZ

UT WOS:000317375100018

PM 23500467

DA 2025-04-09

ER

PT J

AU Maurer, GD

Heller, S

Wanka, C

Rieger, J

Steinbach, JP

AF Maurer, Gabriele D.

Heller, Sonja

Wanka, Christina

Rieger, Johannes

Steinbach, Joachim P.

TI Knockdown of the TP53-Induced Glycolysis and Apoptosis Regulator (TIGAR)

Sensitizes Glioma Cells to Hypoxia, Irradiation and Temozolomide

SO INTERNATIONAL JOURNAL OF MOLECULAR SCIENCES

LA English

DT Article

DE TP53-induced glycolysis and apoptosis regulator; glioma; hypoxia;

irradiation; temozolomide; reactive oxygen species; hypoxia-inducible

factor

ID TUMOR-SUPPRESSOR; P53; GLIOBLASTOMA; RADIOSENSITIZATION;

MICROENVIRONMENT; IDENTIFICATION; PROGRESSION; PROGNOSIS; PROTECTS;

HIF-1

AB The TP53-induced glycolysis and apoptosis regulator (TIGAR) has been shown to decrease glycolysis, to activate the pentose phosphate pathway, and to provide protection against oxidative damage. Hypoxic regions are considered characteristic of glioblastoma and linked with resistance to current treatment strategies. Here, we established that LNT-229 glioma cell lines stably expressed shRNA constructs targeting TIGAR, and exposed them to hypoxia, irradiation and temozolomide. The disruption of TIGAR enhanced levels of reactive oxygen species and cell death under hypoxic conditions, as well as the effectiveness of irradiation and temozolomide. In addition, TIGAR was upregulated by HIF-1. As a component of a complex network, TIGAR contributes to the metabolic adjustments that arise from either spontaneous or therapy-induced changes in tumor microenvironment.

C1 [Maurer, Gabriele D.; Heller, Sonja; Wanka, Christina; Rieger, Johannes; Steinbach, Joachim P.] Goethe Univ, Univ Hosp Frankfurt, Dr Senckenberg Inst Neurooncol, D-60590 Frankfurt, Germany.

[Maurer, Gabriele D.; Heller, Sonja; Wanka, Christina; Rieger, Johannes; Steinbach, Joachim P.] Goethe Univ, Univ Hosp Frankfurt, Univ Canc Ctr UCT, D-60590 Frankfurt, Germany.

[Maurer, Gabriele D.; Heller, Sonja; Wanka, Christina; Rieger, Johannes; Steinbach, Joachim P.] German Canc Res Ctr DKFZ Heidelberg, D-60590 Frankfurt, Germany.

[Maurer, Gabriele D.; Heller, Sonja; Wanka, Christina; Rieger, Johannes; Steinbach, Joachim P.] German Canc Consortium DKTK, Partner Site Frankfurt Mainz, D-60590 Frankfurt, Germany.

[Rieger, Johannes] Eberhard Karls Univ Tubingen, Univ Hosp Tuebingen, Hertie Inst Clin Brain Res, Interdisciplinary Div Neurooncol, D-72076 Tubingen, Germany.

C3 Goethe University Frankfurt; Goethe University Frankfurt Hospital;

Goethe University Frankfurt; Goethe University Frankfurt Hospital;

Helmholtz Association; German Cancer Research Center (DKFZ); Helmholtz

Association; German Cancer Research Center (DKFZ); Eberhard Karls

University of Tubingen; Eberhard Karls University Hospital

RP Steinbach, JP (corresponding author), Goethe Univ, Univ Hosp Frankfurt, Dr Senckenberg Inst Neurooncol, D-60590 Frankfurt, Germany.; Steinbach, JP (corresponding author), Goethe Univ, Univ Hosp Frankfurt, Univ Canc Ctr UCT, D-60590 Frankfurt, Germany.; Steinbach, JP (corresponding author), German Canc Res Ctr DKFZ Heidelberg, D-60590 Frankfurt, Germany.; Steinbach, JP (corresponding author), German Canc Consortium DKTK, Partner Site Frankfurt Mainz, D-60590 Frankfurt, Germany.

EM gabriele.maurer@kgu.de; sonja.heller@kgu.de; christina.wanka@gmx.de;

j.rieger@uni-tuebingen.de; joachim.steinbach@med.uni-frankfurt.de

OI Rieger, Johannes/0000-0002-7918-1777

FU Dr. Senckenberg foundation [2014/SIN-02]

FX The Dr. Senckenberg Institute of Neurooncology is supported by the Dr.

Senckenberg foundation (grant number 2014/SIN-02).

CR Agnihotri S, 2016, NEURO-ONCOLOGY, V18, P160, DOI 10.1093/neuonc/nov125

[Anonymous], BIOMED RES INT

[Anonymous], 2017, J EXP MED

Barker HE, 2015, NAT REV CANCER, V15, P409, DOI 10.1038/nrc3958

Bartrons R, 2018, FRONT ONCOL, V8, DOI 10.3389/fonc.2018.00331

Bensaad K, 2006, CELL, V126, P107, DOI 10.1016/j.cell.2006.05.036

Berra E, 2003, EMBO J, V22, P4082, DOI 10.1093/emboj/cdg392

Brennan CW, 2013, CELL, V155, P462, DOI 10.1016/j.cell.2013.09.034

Carro MS, 2010, NATURE, V463, P318, DOI 10.1038/nature08712

Chandel NS, 1998, P NATL ACAD SCI USA, V95, P11715, DOI 10.1073/pnas.95.20.11715

Cheung EC, 2013, DEV CELL, V25, P463, DOI 10.1016/j.devcel.2013.05.001

Colwell N, 2017, NEURO-ONCOLOGY, V19, P887, DOI 10.1093/neuonc/now258

Corbet C, 2017, NAT REV CANCER, V17, P577, DOI 10.1038/nrc.2017.77

Dayal R, 2014, J CANCER RES THER, V10, P811, DOI 10.4103/0973-1482.146073

Diehn M, 2009, NATURE, V458, P780, DOI 10.1038/nature07733

Dyer BW, 2000, ANAL BIOCHEM, V282, P158, DOI 10.1006/abio.2000.4605

Fei PW, 2003, ONCOGENE, V22, P5774, DOI 10.1038/sj.onc.1206677

Geng J, 2019, FASEB J, V33, P6082, DOI 10.1096/fj.201802209R

Geng J, 2018, FREE RADICAL RES, V52, P1240, DOI 10.1080/10715762.2018.1489133

Gerin I, 2014, BIOCHEM J, V458, P439, DOI 10.1042/BJ20130841

Hammond EM, 2005, BIOCHEM BIOPH RES CO, V331, P718, DOI 10.1016/j.bbrc.2005.03.154

Henze AT, 2010, CANCER RES, V70, P357, DOI 10.1158/0008-5472.CAN-09-1876

Hermisson M, 2006, J NEUROCHEM, V96, P766, DOI 10.1111/j.1471-4159.2005.03583.x

Hofmann U, 2008, BIOTECHNOL BIOENG, V100, P344, DOI 10.1002/bit.21747

Hong M, 2016, LEUKEMIA RES, V50, P72, DOI 10.1016/j.leukres.2016.09.013

Humpton TJ, 2016, CSH PERSPECT MED, V6, DOI 10.1101/cshperspect.a026146

Ishii N, 1999, BRAIN PATHOL, V9, P469, DOI 10.1111/j.1750-3639.1999.tb00536.x

Josephy P.D., 2006, MOL TOXICOLOGY

Kastenhuber ER, 2017, CELL, V170, P1062, DOI 10.1016/j.cell.2017.08.028

Kim YH, 2014, CANCER LETT, V354, P132, DOI 10.1016/j.canlet.2014.07.048

Ko YH, 2016, J BIOL CHEM, V291, P26291, DOI 10.1074/jbc.M116.740209

Li H, 2009, J BIOL CHEM, V284, P1748, DOI 10.1074/jbc.M807821200

Lin CJ, 2012, FREE RADICAL BIO MED, V52, P377, DOI 10.1016/j.freeradbiomed.2011.10.487

Lin L, 2019, CANCER MANAG RES, V11, P263, DOI 10.2147/CMAR.S190272

Liu JB, 2018, HUM PATHOL, V77, P54, DOI 10.1016/j.humpath.2017.12.029

Livak KJ, 2001, METHODS, V25, P402, DOI 10.1006/meth.2001.1262

Mao Z, 2019, BRIT J PHARMACOL, V176, P919, DOI 10.1111/bph.14594

McGillicuddy LT, 2009, CANCER CELL, V16, P44, DOI 10.1016/j.ccr.2009.05.009

McKeown SR, 2014, BRIT J RADIOL, V87, DOI 10.1259/bjr.20130676

Moeller BJ, 2004, CANCER CELL, V5, P429, DOI 10.1016/S1535-6108(04)00115-1

Moloney JN, 2018, SEMIN CELL DEV BIOL, V80, P50, DOI 10.1016/j.semcdb.2017.05.023

Mycielska ME, 2018, CANCER RES, V78, P2513, DOI 10.1158/0008-5472.CAN-17-2959

Oliva CR, 2011, PLOS ONE, V6, DOI 10.1371/journal.pone.0024665

Peña-Rico MA, 2011, RADIOTHER ONCOL, V101, P132, DOI 10.1016/j.radonc.2011.07.002

Phillips HS, 2006, CANCER CELL, V9, P157, DOI 10.1016/j.ccr.2006.02.019

Province P, 2013, Evolution of the Molecular Biology of Brain Tumors and the Therapeutic Implications

Qian SX, 2016, J HEMATOL ONCOL, V9, DOI 10.1186/s13045-016-0360-4

Rajendran R, 2013, INT J ONCOL, V42, P1961, DOI 10.3892/ijo.2013.1907

Schieber M, 2014, CURR BIOL, V24, pR453, DOI 10.1016/j.cub.2014.03.034

Schneider CA, 2012, NAT METHODS, V9, P671, DOI 10.1038/nmeth.2089

Semenza GL, 2004, PHYSIOLOGY, V19, P176, DOI 10.1152/physiol.00001.2004

Sermeus A, 2011, CELL DEATH DIS, V2, DOI 10.1038/cddis.2011.48

Shen MQ, 2018, MOL CANCER, V17, DOI 10.1186/s12943-018-0839-4

Stupp R, 2005, NEW ENGL J MED, V352, P987, DOI 10.1056/NEJMoa043330

Tafani M, 2016, OXID MED CELL LONGEV, V2016, DOI 10.1155/2016/3907147

Turgeon MO, 2018, FRONT ONCOL, V8, DOI 10.3389/fonc.2018.00015

Uhlen M, 2017, SCIENCE, V357, P660, DOI 10.1126/science.aan2507

Verhaak RGW, 2010, CANCER CELL, V17, P98, DOI 10.1016/j.ccr.2009.12.020

Vousden K.H., 2018, FRANKF CANC C FRANK

Wang HH, 2017, SCI REP-UK, V7, DOI 10.1038/s41598-017-06851-3

Wanka C, 2012, J BIOL CHEM, V287, P33436, DOI 10.1074/jbc.M112.384578

Weinmann L, 2008, CELL DEATH DIFFER, V15, P718, DOI 10.1038/sj.cdd.4402301

Westergaard N, 2017, NEUROCHEM RES, V42, P1583, DOI 10.1007/s11064-016-2159-7

Wischhusen J, 2003, ONCOGENE, V22, P8233, DOI 10.1038/sj.onc.1207198

Wong EYL, 2015, ONCOL LETT, V9, P569, DOI 10.3892/ol.2014.2797

Xie JM, 2014, CANCER RES, V74, P5127, DOI 10.1158/0008-5472.CAN-13-3517

Yin CQ, 2016, ONCOTARGET, V7, P47494, DOI 10.18632/oncotarget.10202

Yin HT, 2014, ONCOL REP, V32, P1610, DOI 10.3892/or.2014.3342

Yu HP, 2015, SCI REP-UK, V5, DOI 10.1038/srep09853

Zhang HW, 2014, FREE RADICAL BIO MED, V69, P239, DOI 10.1016/j.freeradbiomed.2014.01.034

[张腾 Zhang Teng], 2014, [肿瘤, Tumor], V34, P303

NR 71

TC 17

Z9 18

U1 0

U2 9

PU MDPI

PI BASEL

PA ST ALBAN-ANLAGE 66, CH-4052 BASEL, SWITZERLAND

SN 1422-0067

J9 INT J MOL SCI

JI Int. J. Mol. Sci.

PD MAR 1

PY 2019

VL 20

IS 5

AR 1061

DI 10.3390/ijms20051061

PG 14

WC Biochemistry & Molecular Biology; Chemistry, Multidisciplinary

WE Science Citation Index Expanded (SCI-EXPANDED)

SC Biochemistry & Molecular Biology; Chemistry

GA HQ6QE

UT WOS:000462542300060

PM 30823646

OA Green Submitted, Green Published, gold

DA 2025-04-09

ER

PT J

AU Kayabolen, A

Yilmaz, E

Bagci-Onder, T

AF Kayabolen, Alisan

Yilmaz, Ebru

Bagci-Onder, Tugba

TI IDH Mutations in Glioma: Double-Edged Sword in Clinical

Applications?

SO BIOMEDICINES

LA English

DT Review

DE isocitrate dehydrogenase (IDH); mutations; glioma; glioblastoma;

therapeutics; clinical trials

ID DEPENDENT ISOCITRATE DEHYDROGENASE; PREDICT LONGER SURVIVAL; T-CELL

THERAPY; MUTANT IDH1; ONCOMETABOLITE 2-HYDROXYGLUTARATE; PROMOTES

DIFFERENTIATION; GLUTAMINE-METABOLISM; OXIDATIVE DAMAGE; INHIBITOR;

GROWTH

AB Discovery of point mutations in the genes encoding isocitrate dehydrogenases (IDH) in gliomas about a decade ago has challenged our view of the role of metabolism in tumor progression and provided a new stratification strategy for malignant gliomas. IDH enzymes catalyze the conversion of isocitrate to alpha-ketoglutarate (alpha-KG), an intermediate in the citric acid cycle. Specific mutations in the genes encoding IDHs cause neomorphic enzymatic activity that produces D-2-hydroxyglutarate (2-HG) and result in the inhibition of alpha-KG-dependent enzymes such as histone and DNA demethylases. Thus, chromatin structure and gene expression profiles in IDH-mutant gliomas appear to be different from those in IDH-wildtype gliomas. IDH mutations are highly common in lower grade gliomas (LGG) and secondary glioblastomas, and they are among the earliest genetic events driving tumorigenesis. Therefore, inhibition of mutant IDH enzymes in LGGs is widely accepted as an attractive therapeutic strategy. On the other hand, the metabolic consequences derived from IDH mutations lead to selective vulnerabilities within tumor cells, making them more sensitive to several therapeutic interventions. Therefore, instead of shutting down mutant IDH enzymes, exploiting the selective vulnerabilities caused by them might be another attractive and promising strategy. Here, we review therapeutic options and summarize current preclinical and clinical studies on IDH-mutant gliomas.

C1 [Kayabolen, Alisan; Yilmaz, Ebru; Bagci-Onder, Tugba] Koc Univ, Brain Canc Res & Therapy Lab, Sch Med, TR-34450 Istanbul, Turkey.

[Kayabolen, Alisan; Yilmaz, Ebru; Bagci-Onder, Tugba] Koc Univ, Res Ctr Translat Med KUTTAM, TR-34450 Istanbul, Turkey.

C3 Koc University; Koc University

RP Bagci-Onder, T (corresponding author), Koc Univ, Brain Canc Res & Therapy Lab, Sch Med, TR-34450 Istanbul, Turkey.; Bagci-Onder, T (corresponding author), Koc Univ, Res Ctr Translat Med KUTTAM, TR-34450 Istanbul, Turkey.

EM akayabolen@ku.edu.tr; eyilmaz20@ku.edu.tr; tuonder@ku.edu.tr

RI Kayabolen, Alisan/AAJ-8436-2020; Onder, Tugba/AAN-1392-2021

OI Bagci Onder, Tugba/0000-0003-3646-2613; KAYABOLEN,

ALISAN/0000-0001-5997-0894; Yilmaz, Ebru/0000-0003-1033-1797

FU Scientific and Technological Research Council of Turkey (TUBITAK)

[1001-219S882]

FX Financial support was obtained from the Scientific and Technological

Research Council of Turkey (TUBITAK) (grant No. 1001-219S882).

CR Altman BJ, 2016, NAT REV CANCER, V16, P619, DOI 10.1038/nrc.2016.71

Amankulor NM, 2017, GENE DEV, V31, P774, DOI 10.1101/gad.294991.116

Andronesi OC, 2018, NAT COMMUN, V9, DOI 10.1038/s41467-018-03905-6

Badur MG, 2018, CELL REP, V25, P1018, DOI 10.1016/j.celrep.2018.09.074

Bai H, 2016, NAT GENET, V48, P59, DOI 10.1038/ng.3457

Batsios G, 2019, SCI REP-UK, V9, DOI 10.1038/s41598-019-47021-x

Bender S, 2013, CANCER CELL, V24, P660, DOI 10.1016/j.ccr.2013.10.006

Berghoff AS, 2017, NEURO-ONCOLOGY, V19, P1460, DOI 10.1093/neuonc/nox054

Bettegowda C, 2011, SCIENCE, V333, P1453, DOI 10.1126/science.1210557

Borodovsky A, 2013, ONCOTARGET, V4, P1737, DOI 10.18632/oncotarget.1408

Bowman RL, 2017, NEURO-ONCOLOGY, V19, P139, DOI 10.1093/neuonc/now247

Brat DJ, 2015, NEW ENGL J MED, V372, P2481, DOI 10.1056/NEJMoa1402121

Buckner JC, 2016, NEW ENGL J MED, V374, P1344, DOI 10.1056/NEJMoa1500925

Bunse L, 2018, NAT MED, V24, P1192, DOI 10.1038/s41591-018-0095-6

Burki F, 2004, NAT GENET, V36, P1061, DOI 10.1038/ng1431

Carbonneau M, 2016, NAT COMMUN, V7, DOI 10.1038/ncomms12700

Chan SM, 2015, NAT MED, V21, P178, DOI 10.1038/nm.3788

Chen RH, 2014, P NATL ACAD SCI USA, V111, P14217, DOI 10.1073/pnas.1409653111

Chesnelong C, 2014, NEURO-ONCOLOGY, V16, P686, DOI 10.1093/neuonc/not243

Chowdhury R, 2011, EMBO REP, V12, P463, DOI 10.1038/embor.2011.43

Claus EB, 2015, NEUROSURG FOCUS, V38, DOI 10.3171/2014.10.FOCUS12367

DiNardo CD, 2018, NEW ENGL J MED, V378, P2386, DOI 10.1056/NEJMoa1716984

DiNardo CD, 2016, BLOOD, V128, DOI 10.1182/blood.V128.22.1073.1073

Dowdy T, 2020, CANCERS, V12, DOI 10.3390/cancers12102910

Fack F, 2017, EMBO MOL MED, V9, P1681, DOI 10.15252/emmm.201707729

Fan QW, 2006, CANCER CELL, V9, P341, DOI 10.1016/j.ccr.2006.03.029

Flavahan WA, 2016, NATURE, V529, P110, DOI 10.1038/nature16490

Fu XD, 2015, CELL METAB, V22, P508, DOI 10.1016/j.cmet.2015.06.009

Fujiwara S, 2007, INT J ONCOL, V30, P793

GABRIEL JL, 1986, METABOLISM, V35, P661, DOI 10.1016/0026-0495(86)90175-7

Garcia-Manero G, 2020, BLOOD, V136, P674, DOI 10.1182/blood.2019004143

Gelman SJ, 2018, CELL REP, V22, P512, DOI 10.1016/j.celrep.2017.12.050

Gillespie DL, 2007, CLIN CANCER RES, V13, P2441, DOI 10.1158/1078-0432.CCR-06-2692

Grassian AR, 2014, CANCER RES, V74, P3317, DOI 10.1158/0008-5472.CAN-14-0772-T

Hartmann C, 2010, ACTA NEUROPATHOL, V120, P707, DOI 10.1007/s00401-010-0781-z

Hartmann C, 2009, ACTA NEUROPATHOL, V118, P469, DOI 10.1007/s00401-009-0561-9

Hodges TR, 2017, NEURO-ONCOLOGY, V19, P1047, DOI 10.1093/neuonc/nox026

Holmgren A, 2010, BIOCHEM BIOPH RES CO, V396, P120, DOI 10.1016/j.bbrc.2010.03.083

Horton JR, 2016, J BIOL CHEM, V291, P2631, DOI 10.1074/jbc.M115.698449

Houillier C, 2010, NEUROLOGY, V75, P1560, DOI 10.1212/WNL.0b013e3181f96282

HURLEY JH, 1991, BIOCHEMISTRY-US, V30, P8671, DOI 10.1021/bi00099a026

De La Fuente MI, 2020, J CLIN ONCOL, V38

Intlekofer AM, 2015, CELL METAB, V22, P304, DOI 10.1016/j.cmet.2015.06.023

Jensen Randy L, 2006, Neurosurg Focus, V20, pE24, DOI 10.3171/foc.2006.20.4.16

Jo SH, 2001, J BIOL CHEM, V276, P16168, DOI 10.1074/jbc.M010120200

Johannessen TCA, 2016, MOL CANCER RES, V14, P976, DOI 10.1158/1541-7786.MCR-16-0141

Johnson BE, 2014, SCIENCE, V343, P189, DOI 10.1126/science.1239947

Kadiyala P, 2021, J CLIN INVEST, V131, DOI 10.1172/JCI139542

Karpel-Massler G, 2017, NAT COMMUN, V8, DOI 10.1038/s41467-017-00984-9

Kats LM, 2014, CELL STEM CELL, V14, P329, DOI 10.1016/j.stem.2013.12.016

Kayabolen A., 2020, COMBINED INHIBITION, DOI [10.1101/2020.11.26.400234, DOI 10.1101/2020.11.26.400234]

Kessler J, 2010, BMC CANCER, V10, DOI 10.1186/1471-2407-10-605

Kim ES, 2017, DRUGS, V77, P929, DOI 10.1007/s40265-017-0749-6

Koh HJ, 2004, J BIOL CHEM, V279, P39968, DOI 10.1074/jbc.M402260200

Kohanbash G, 2017, J CLIN INVEST, V127, P1425, DOI 10.1172/JCI90644

Koivunen P, 2012, NATURE, V483, P485, DOI 10.1038/nature10898

Kopinja J, 2017, SCI REP-UK, V7, DOI 10.1038/s41598-017-14065-w

Lai A, 2011, J CLIN ONCOL, V29, P4482, DOI 10.1200/JCO.2010.33.8715

Lee SM, 2002, FREE RADICAL BIO MED, V32, P1185, DOI 10.1016/S0891-5849(02)00815-8

Li SC, 2013, NEURO-ONCOLOGY, V15, P57, DOI 10.1093/neuonc/nos261

Lita A., 2020, IDH1 MUTAITONS INDUC, DOI [10.1101/2020.03.20.000414, DOI 10.1101/2020.03.20.000414]

Losman JA, 2013, GENE DEV, V27, P836, DOI 10.1101/gad.217406.113

Louis DN, 2016, ACTA NEUROPATHOL, V131, P803, DOI 10.1007/s00401-016-1545-1

Lu C, 2012, NATURE, V483, P474, DOI 10.1038/nature10860

Lu YX, 2017, CANCER RES, V77, P1709, DOI 10.1158/0008-5472.CAN-16-2773

McBrayer SK, 2018, CELL, V175, P101, DOI 10.1016/j.cell.2018.08.038

Mellinghoff IK, 2020, J CLIN ONCOL, V38, DOI 10.1200/JCO.19.03327

Metellus P, 2010, ACTA NEUROPATHOL, V120, P719, DOI 10.1007/s00401-010-0777-8

Miller JJ, 2021, NEURO-ONCOLOGY, V23, P53, DOI 10.1093/neuonc/noaa180

Molenaar RJ, 2015, CANCER RES, V75, P4790, DOI 10.1158/0008-5472.CAN-14-3603

Nagashima H, 2020, CANCER DISCOV, V10, P1672, DOI 10.1158/2159-8290.CD-20-0226

Natsume A, 2019, J CLIN ONCOL, V37, DOI 10.1200/JCO.2019.37.15_suppl.2004

Newsholme P, 2003, CELL BIOCHEM FUNCT, V21, P1, DOI 10.1002/cbf.1003

Nobusawa S, 2009, CLIN CANCER RES, V15, P6002, DOI 10.1158/1078-0432.CCR-09-0715

Noorani I, 2020, GENOME BIOL, V21, DOI 10.1186/s13059-020-02092-2

Noorani I, 2019, CANCERS, V11, DOI 10.3390/cancers11091335

Norsworthy KJ, 2019, CLIN CANCER RES, V25, P3205, DOI 10.1158/1078-0432.CCR-18-3749

O'Rourke DM, 2017, SCI TRANSL MED, V9, DOI 10.1126/scitranslmed.aaa0984

Oganesian A, 2013, BLOOD, V122, DOI 10.1182/blood.V122.21.2526.2526

Ohgaki H, 2004, CANCER RES, V64, P6892, DOI 10.1158/0008-5472.CAN-04-1337

Okoye-Okafor UC, 2015, NAT CHEM BIOL, V11, P878, DOI 10.1038/nchembio.1930

Ostrom QT, 2016, NEURO-ONCOLOGY, V18, pv1, DOI [10.1093/neuonc/nov189, 10.1093/neuonc/now207]

Ou XY, 2020, NAT COMMUN, V11, DOI 10.1038/s41467-020-15562-9

Pellegatta S, 2015, ACTA NEUROPATHOL COM, V3, DOI 10.1186/s40478-014-0180-0

Philip B, 2018, CELL REP, V23, P1553, DOI 10.1016/j.celrep.2018.03.133

Platten M, 2021, NATURE, V592, P463, DOI 10.1038/s41586-021-03363-z

Platten M, 2018, NEURO-ONCOLOGY, V20, P8

Puli S, 2010, NEUROCHEM RES, V35, P986, DOI 10.1007/s11064-010-0142-2

Pusch S, 2017, ACTA NEUROPATHOL, V133, P629, DOI 10.1007/s00401-017-1677-y

Qazi MA, 2017, ANN ONCOL, V28, P1448, DOI 10.1093/annonc/mdx169

Qi ST, 2012, CANCER SCI, V103, P269, DOI 10.1111/j.1349-7006.2011.02134.x

RAMACHANDRAN N, 1980, J BIOL CHEM, V255, P8859

Rohle D, 2013, SCIENCE, V340, P626, DOI 10.1126/science.1236062

Salamanca-Cardona L, 2017, CELL METAB, V26, P830, DOI 10.1016/j.cmet.2017.10.001

Sampson JH, 2014, CLIN CANCER RES, V20, P972, DOI 10.1158/1078-0432.CCR-13-0709

Sanson M, 2009, J CLIN ONCOL, V27, P4150, DOI 10.1200/JCO.2009.21.9832

Sasaki M, 2012, GENE DEV, V26, P2038, DOI 10.1101/gad.198200.112

Schumacher T, 2014, NATURE, V512, P324, DOI 10.1038/nature13387

Seltzer MJ, 2010, CANCER RES, V70, P8981, DOI 10.1158/0008-5472.CAN-10-1666

Senhaji N, 2017, BIOMED RES INT-UK, V2017, DOI 10.1155/2017/8045859

Shashidharan P, 2014, NEUROCHEM RES, V39, P460, DOI 10.1007/s11064-013-1227-5

Sulkowski PL, 2017, SCI TRANSL MED, V9, DOI 10.1126/scitranslmed.aal2463

Suzuki H, 2015, NAT GENET, V47, P458, DOI 10.1038/ng.3273

Taher MM, 2020, ONCOL LETT, V20, DOI 10.3892/ol.2020.12247

Tang JH, 2016, EXP CELL RES, V343, P148, DOI 10.1016/j.yexcr.2016.04.011

Tateishi K, 2017, CANCER RES, V77, P4102, DOI 10.1158/0008-5472.CAN-16-2263

Tateishi K, 2015, CANCER CELL, V28, P773, DOI 10.1016/j.ccell.2015.11.006

Tran AN, 2014, NEURO-ONCOLOGY, V16, P414, DOI 10.1093/neuonc/not198

Turcan S, 2018, NAT GENET, V50, P62, DOI 10.1038/s41588-017-0001-z

Turcan S, 2013, ONCOTARGET, V4, P1729, DOI 10.18632/oncotarget.1412

Turcan S, 2012, NATURE, V483, P479, DOI 10.1038/nature10866

Van Den Bent MJ, 2019, J CLIN ONCOL, V37, DOI 10.1200/JCO.2019.37.15_suppl.2000

van den Bent MJ, 2010, CLIN CANCER RES, V16, P1597, DOI 10.1158/1078-0432.CCR-09-2902

Viswanath P, 2018, CANCER RES, V78, P2290, DOI 10.1158/0008-5472.CAN-17-2926

Wakimoto H, 2014, CLIN CANCER RES, V20, P2898, DOI 10.1158/1078-0432.CCR-13-3052

Wang F, 2013, SCIENCE, V340, P622, DOI 10.1126/science.1234769

Wang YX, 2020, SCI ADV, V6, DOI 10.1126/sciadv.aaz3221

WARBURG O, 1956, SCIENCE, V123, P309, DOI 10.1126/science.123.3191.309

Watanabe T, 2009, AM J PATHOL, V174, P1149, DOI 10.2353/ajpath.2009.080958

Xiao W, 2020, CANCER-AM CANCER SOC, V126, P2093, DOI 10.1002/cncr.32764

Xu W, 2011, CANCER CELL, V19, P17, DOI 10.1016/j.ccr.2010.12.014

Xu X, 2004, J BIOL CHEM, V279, P33946, DOI 10.1074/jbc.M404298200

Yalaza C, 2017, ANN CLIN LAB SCI, V47, P362

Yamashita AS, 2019, NEURO-ONCOLOGY, V21, P189, DOI 10.1093/neuonc/noy146

Ye D, 2012, CELL RES, V22, P1102, DOI 10.1038/cr.2012.51

Yip S, 2012, J PATHOL, V226, P7, DOI 10.1002/path.2995

Zecchini V, 2017, BBA-BIOENERGETICS, V1858, P723, DOI 10.1016/j.bbabio.2016.12.003

Zhang Y, 2019, CANCER RES, V79, P4994, DOI 10.1158/0008-5472.CAN-19-0054

NR 128

TC 52

Z9 59

U1 1

U2 7

PU MDPI

PI BASEL

PA ST ALBAN-ANLAGE 66, CH-4052 BASEL, SWITZERLAND

EI 2227-9059

J9 BIOMEDICINES

JI Biomedicines

PD JUL

PY 2021

VL 9

IS 7

AR 799

DI 10.3390/biomedicines9070799

PG 21

WC Biochemistry & Molecular Biology; Medicine, Research & Experimental;

Pharmacology & Pharmacy

WE Science Citation Index Expanded (SCI-EXPANDED)

SC Biochemistry & Molecular Biology; Research & Experimental Medicine;

Pharmacology & Pharmacy

GA TN5TE

UT WOS:000676295900001

PM 34356864

OA gold, Green Published

DA 2025-04-09

ER

PT J

AU Liu, WJ

Yin, YB

Sun, JY

Feng, S

Ma, JK

Fu, XY

Hou, YJ

Yang, MF

Sun, BL

Fan, CD

AF Liu, Wen-Jian

Yin, Yi-Bo

Sun, Jing-Yi

Feng, Sai

Ma, Jin-Kui

Fu, Xiao-Yan

Hou, Ya-Jun

Yang, Ming-Feng

Sun, Bao-Liang

Fan, Cun-Dong

TI Natural borneol is a novel chemosensitizer that enhances

temozolomide-induced anticancer efficiency against human glioma by

triggering mitochondrial dysfunction and reactive oxide species-mediated

oxidative damage

SO ONCOTARGETS AND THERAPY

LA English

DT Article

DE glioma; borneol; temozolomide; DNA damage; apoptosis

ID CELL-CYCLE ARREST; INTESTINAL-ABSORPTION; MOLECULAR-MECHANISMS;

CANCER-CELLS; DNA-DAMAGE; APOPTOSIS; BRAIN; DEATH; ROS

AB Background: Temozolomide (TMZ)-based chemotherapy represents an effective way for treating human glioma. However, its clinical application is limited because of its side effects and resistance to standard chemotherapy. Hence, the search for novel chemosensitizers to augment their anticancer efficiency has attracted much attention. Natural borneol (NB) has been identified as a potential chemosensitizer in treating human cancers. However, the synergistic effect and mechanism of NB and TMZ in human glioma have not been investigated yet.

Materials and methods: U251 human glioma cells were cultured, and the cytotoxicity and apoptosis of NB and/or TMZ were examined by MTT assay, flow cytometric analysis and Western blot. Nude mice tumor model was also employed to evaluate the in vivo anticancer effect and mechanism.

Results: The results showed that the combined treatment of NB and TMZ more effectively inhibited human glioma growth via triggering mitochondria-mediated apoptosis in vitro, accompanied by the caspase activation. Combined treatment of NB and TMZ also caused mitochondrial dysfunction through disturbing Bcl-2 family expression. Further investigation revealed that NB enhanced TMZ-induced DNA damage through inducing reactive oxide species (ROS) overproduction. Moreover, glioma tumor xenograft growth in vivo was more effectively inhibited by the combined treatment with NB and TMZ through triggering apoptosis and anti-angiogenesis.

Conclusion: Taken together, our findings validated that the strategy of using NB and TMZ could be a highly efficient way to achieve anticancer synergism.

C1 [Liu, Wen-Jian; Yin, Yi-Bo; Sun, Bao-Liang] Taishan Med Univ, Dept Neurol, Affiliated Hosp, Tai An, Shandong, Peoples R China.

[Sun, Jing-Yi] Yonsei Univ, Wonju Severance Christian Hosp, Dept Orthopaed, Wonju Coll Med, Wonju, Gangwon, South Korea.

[Feng, Sai] Guangzhou New BenFu Technol Co Ltd, Guangzhou, Guangdong, Peoples R China.

[Ma, Jin-Kui] Akita Prefectural Univ, Fac Bioresource Sci, Akita, Akita, Japan.

[Fu, Xiao-Yan; Hou, Ya-Jun; Yang, Ming-Feng; Sun, Bao-Liang; Fan, Cun-Dong] Taishan Med Univ, Key Lab Cerebral Microcirculat Univ Shandong, Tai An, Shandong, Peoples R China.

C3 Shandong First Medical University & Shandong Academy of Medical

Sciences; Yonsei University; Akita Prefectural University; Shandong

First Medical University & Shandong Academy of Medical Sciences

RP Sun, BL; Fan, CD (corresponding author), Taishan Med Univ, Dept Neurol, Yingsheng East Rd 2, Tai An 271000, Shandong, Peoples R China.

EM tblsun66@163.com; tcdfan66@163.com

RI Fu, Xiaoyan/C-2573-2012; ma, jinkui/AAC-7492-2019; Fan,

Cundong/ABG-5600-2021; Sun, Baoliang/ACO-7110-2022

OI , Jin-Kui/0000-0001-9346-2076; Fan, Cun-dong/0000-0003-4485-1344

FU National Natural Science Foundation of China [81501106, 81471212]

FX This study was supported by the National Natural Science Foundation of

China No 81501106 to C-DF and No 81471212 to B-LS.

CR Abbott NJ, 2010, NEUROBIOL DIS, V37, P13, DOI 10.1016/j.nbd.2009.07.030

Attwell D, 2016, J CEREBR BLOOD F MET, V36, P451, DOI 10.1177/0271678X15610340

Berghiche H., 2005, Communications in Agricultural and Applied Biological Sciences, V70, P837

Boulaire J, 2000, PATHOL BIOL, V48, P190

Carlsson SK, 2014, EMBO MOL MED, V6, P1359, DOI 10.15252/emmm.201302627

Chen J, 2012, CELL, V149, P36, DOI 10.1016/j.cell.2012.03.009

Chen JP, 2015, FOOD FUNCT, V6, P740, DOI [10.1039/C4FO00807C, 10.1039/c4fo00807c]

Chen JP, 2014, PLOS ONE, V9, DOI 10.1371/journal.pone.0101277

Chen TF, 2008, BIOMED PHARMACOTHER, V62, P77, DOI 10.1016/j.biopha.2007.12.002

Chen WQ, 2016, CA-CANCER J CLIN, V66, P115, DOI 10.3322/caac.21338

Circu ML, 2010, FREE RADICAL BIO MED, V48, P749, DOI 10.1016/j.freeradbiomed.2009.12.022

Cui Q, 2007, ACTA PHARMACOL SIN, V28, P1057, DOI 10.1111/j.1745-7254.2007.00588.x

Edlich F, 2011, CELL, V145, P104, DOI 10.1016/j.cell.2011.02.034

Hart MG, 2013, COCHRANE DB SYST REV, DOI 10.1002/14651858.CD007415.pub2

Hikita H, 2009, HEPATOLOGY, V50, P1217, DOI 10.1002/hep.23126

Kaufmann SH, 2000, EXP CELL RES, V256, P42, DOI 10.1006/excr.2000.4838

Krantic S, 2007, PROG NEUROBIOL, V81, P179, DOI 10.1016/j.pneurobio.2006.12.002

Kruh Gary D., 1993, Current Opinion in Oncology, V5, P1029, DOI 10.1097/00001622-199311000-00014

Martinou JC, 2011, DEV CELL, V21, P92, DOI 10.1016/j.devcel.2011.06.017

Maule F, 2016, ONCOTARGET, V7, P54632, DOI 10.18632/oncotarget.10565

Mizutani H, 2007, YAKUGAKU ZASSHI, V127, P1837, DOI 10.1248/yakushi.127.1837

Ostrom QT, 2013, NEURO-ONCOLOGY, V15, P1, DOI 10.1093/neuonc/not151

Pelicano H, 2004, DRUG RESIST UPDATE, V7, P97, DOI 10.1016/j.drup.2004.01.004

Riedl SJ, 2004, NAT REV MOL CELL BIO, V5, P897, DOI 10.1038/nrm1496

Sancar A, 2004, ANNU REV BIOCHEM, V73, P39, DOI 10.1146/annurev.biochem.73.011303.073723

Shen Q, 2011, AAPS PHARMSCITECH, V12, P1044, DOI 10.1208/s12249-011-9672-4

Su JY, 2013, PLOS ONE, V8, DOI 10.1371/journal.pone.0063502

Trost A, 2016, FRONT CELL NEUROSCI, V10, DOI 10.3389/fncel.2016.00020

van Gurp M, 2003, BIOCHEM BIOPH RES CO, V304, P487, DOI 10.1016/S0006-291X(03)00621-1

Zhou YQ, 2010, ENVIRON TOXICOL PHAR, V29, P229, DOI 10.1016/j.etap.2010.01.004

NR 30

TC 28

Z9 30

U1 0

U2 12

PU DOVE MEDICAL PRESS LTD

PI ALBANY

PA PO BOX 300-008, ALBANY, AUCKLAND 0752, NEW ZEALAND

SN 1178-6930

J9 ONCOTARGETS THER

JI OncoTargets Ther.

PY 2018

VL 11

BP 5429

EP 5439

DI 10.2147/OTT.S174498

PG 11

WC Biotechnology & Applied Microbiology; Oncology

WE Science Citation Index Expanded (SCI-EXPANDED)

SC Biotechnology & Applied Microbiology; Oncology

GA GS6AW

UT WOS:000443763400002

PM 30233204

OA Green Published, Green Submitted, gold

DA 2025-04-09

ER

PT J

AU Rivera, M

Wu, Q

Hamerlik, P

Hjelmeland, AB

Bao, S

Rich, JN

AF Rivera, M.

Wu, Q.

Hamerlik, P.

Hjelmeland, A. B.

Bao, S.

Rich, J. N.

TI Acquisition of meiotic DNA repair regulators maintain genome stability

in glioblastoma

SO CELL DEATH & DISEASE

LA English

DT Article

ID MEIOSIS-SPECIFIC GENES; HOMOLOGOUS RECOMBINATION; IONIZING-RADIATION;

ADJUVANT TEMOZOLOMIDE; REPLICATION STRESS; OXIDATIVE DAMAGE; PRIMARY

BRAIN; RAD51; RADIOTHERAPY; SURVIVAL

AB Glioblastoma (GBM), the most prevalent type of primary intrinsic brain cancer in adults, remains universally fatal despite maximal therapy, including radiotherapy and chemotherapy. Cytotoxic therapy generates double-stranded DNA breaks (DSBs), most commonly repaired by homologous recombination (HR). We hypothesized that cancer cells coopt meiotic repair machinery as DSBs are generated during meiosis and repaired by molecular complexes distinct from genotoxic responses in somatic tissues. Indeed, we found that gliomas express meiotic repair genes and their expression informed poor prognosis. We interrogated the function of disrupted meiotic cDNA1 (DMC1), a homolog of RAD51, the primary recombinase used in mitotic cells to search and recombine with the homologous DNA template. DMC1, whose only known function is as an HR recombinase, was expressed by GBM cells and induced by radiation. Although targeting DMC1 in non-neoplastic cells minimally altered cell growth, DMC1 depletion in GBM cells decreased proliferation, induced activation of CHK1 and expression of p21(CIP1/WAF1), and increased RPA foci, suggesting increased replication stress. Combining loss of DMC1 with ionizing radiation inhibited activation of DNA damage responses and increased radiosensitivity. Furthermore, loss of DMC1 reduced tumor growth and prolonged survival in vivo. Our results suggest that cancers coopt meiotic genes to augment survival under genotoxic stress, offering molecular targets with high therapeutic indices.

C1 [Rivera, M.; Wu, Q.; Bao, S.; Rich, J. N.] Cleveland Clin Fdn, Lerner Res Inst, Dept Stem Cell Biol & Regenerat Med, Cleveland, OH 44118 USA.

[Rivera, M.; Rich, J. N.] Case Western Reserve Univ, Cleveland Clin, Lerner Coll Med, Dept Mol Med, Cleveland, OH 44106 USA.

[Hamerlik, P.] Danish Canc Soc Res Ctr, Dept Brain Tumor Biol, DK-2100 Copenhagen, Denmark.

[Hjelmeland, A. B.] Univ Alabama Birmingham, Dept Cell Dev & Integrat Biol, Birmingham, AL 35294 USA.

C3 Cleveland Clinic Foundation; University System of Ohio; Case Western

Reserve University; Cleveland Clinic Foundation; Danish Cancer Society;

University of Alabama System; University of Alabama Birmingham

RP Rich, JN (corresponding author), Cleveland Clin Fdn, Lerner Res Inst, Dept Stem Cell Biol & Regenerat Med, NE30,9500 Euclid Ave, Cleveland, OH 44118 USA.

EM drjeremyrich@gmail.com

RI Rich, Jeremy/AAM-1445-2021; Hamerlik, Petra/G-7398-2016

OI Hamerlik, Petra/0000-0002-5856-0161

FU National Institutes of Health [CA171652, CA154130, CA129958, NS087913,

NS089272, CA169117, CA151522, NS070315]; James S McDonnell Foundation

[T32-GM088088]; Danish Council for Independent Research/Medical Sciences

[ID4765/11-105457]; Czech Ministry of Health [NT11065-5];

Ludenbeckfonden; DCRC

FX We thank our funding sources: the National Institutes of Health grants

CA171652, CA154130, CA129958, NS087913, NS089272, CA169117 (JNR),

CA151522 (AH), NS070315 (SB); the James S McDonnell Foundation (JNR),

T32-GM088088 (MR), the Danish Council for Independent Research/Medical

Sciences ID4765/11-105457 (PH), Czech Ministry of Health (NT11065-5) and

Ludenbeckfonden and DCRC (PH). We also thank all the members of the Rich

Laboratory. We appreciate critical manuscript review by M Venere, J Yu

and M Summers; flow cytometry assistance by C Shemo and S O'Bryant;

imaging assistance by J Drazba and E Diskin; and animal support provided

by the Lerner Research Institute BRU.

CR Bao SD, 2006, NATURE, V444, P756, DOI 10.1038/nature05236

Bartkova J, 2010, ONCOGENE, V29, P5095, DOI 10.1038/onc.2010.249

BISHOP DK, 1994, CELL, V79, P1081, DOI 10.1016/0092-8674(94)90038-8

BISHOP DK, 1992, CELL, V69, P439, DOI 10.1016/0092-8674(92)90446-J

Bryant HE, 2005, NATURE, V434, P913, DOI 10.1038/nature03443

Bugreev DV, 2014, NAT COMMUN, V5, DOI 10.1038/ncomms5198

Bugreev DV, 2011, NAT STRUCT MOL BIOL, V18, P56, DOI 10.1038/nsmb.1946

Chang Julie E, 2007, Clin Adv Hematol Oncol, V5, P894

Chinot OL, 2014, NEW ENGL J MED, V370, P709, DOI 10.1056/NEJMoa1308345

Cloud V, 2012, SCIENCE, V337, P1222, DOI 10.1126/science.1219379

Costanzo V, 2011, DNA REPAIR, V10, P1060, DOI 10.1016/j.dnarep.2011.07.009

Davis AJ, 2013, TRANSL CANCER RES, V2, P130, DOI 10.3978/j.issn.2218-676X.2013.04.02

Farmer H, 2005, NATURE, V434, P917, DOI 10.1038/nature03445

Fong PC, 2009, NEW ENGL J MED, V361, P123, DOI 10.1056/NEJMoa0900212

Gilbert MR, 2014, NEW ENGL J MED, V370, P699, DOI 10.1056/NEJMoa1308573

Goudar RK, 2005, MOL CANCER THER, V4, P101

Gupta T, 2005, ACTA ONCOL, V44, P105, DOI 10.1080/02841860510007611

Hashimoto Y, 2010, NAT STRUCT MOL BIOL, V17, P1305, DOI 10.1038/nsmb.1927

Hawrylycz MJ, 2012, NATURE, V489, P391, DOI 10.1038/nature11405

Ianzini F, 2009, CANCER RES, V69, P2296, DOI 10.1158/0008-5472.CAN-08-3364

Iyama T, 2013, DNA REPAIR, V12, P620, DOI 10.1016/j.dnarep.2013.04.015

Jiang KC, 2003, J BIOL CHEM, V278, P25207, DOI 10.1074/jbc.M300070200

Kalejs M, 2006, BMC CANCER, V6, DOI 10.1186/1471-2407-6-6

Keeney Scott, 2008, V2, P81, DOI 10.1007/7050_2007_026

Konca K, 2003, MUTAT RES-GEN TOX EN, V534, P15, DOI 10.1016/S1383-5718(02)00251-6

Krex D, 2007, BRAIN, V130, P2596, DOI 10.1093/brain/awm204

Lambert S, 2010, MOL CELL, V39, P346, DOI 10.1016/j.molcel.2010.07.015

Lao JP, 2013, PLOS GENET, V9, DOI 10.1371/journal.pgen.1003978

Lim YC, 2014, MOL ONCOL, V8, P1603, DOI 10.1016/j.molonc.2014.06.012

Lim YC, 2012, MOL CANCER THER, V11, P1863, DOI 10.1158/1535-7163.MCT-11-1044

Lindsey SF, 2013, J SKIN CANCER, V2013, DOI 10.1155/2013/190109

Lord CJ, 2012, NATURE, V481, P287, DOI 10.1038/nature10760

Makishima H, 2011, CLIN CANCER RES, V17, P3913, DOI 10.1158/1078-0432.CCR-10-2900

Masson JY, 1999, EMBO J, V18, P6552, DOI 10.1093/emboj/18.22.6552

Morales A, 1998, INT J RADIAT ONCOL, V42, P191, DOI 10.1016/S0360-3016(98)00185-0

Moynahan ME, 2010, NAT REV MOL CELL BIO, V11, P196, DOI 10.1038/nrm2851

Nesic D, 2004, NATURE, V429, P429, DOI 10.1038/nature02532

Ostrom QT, 2013, NEURO-ONCOLOGY, V15, P1, DOI 10.1093/neuonc/not151

Rivera M, 2013, FRONT ONCOL, V3, DOI 10.3389/fonc.2013.00074

Romanelli P, 2009, NEUROSURG FOCUS, V27, DOI 10.3171/2009.9.FOCUS09187

Santivasi WL, 2013, CELL BIOSCI, V3, DOI 10.1186/2045-3701-3-10

Schwartzbaum JA, 2006, NAT CLIN PRACT NEURO, V2, P494, DOI 10.1038/ncpneuro0289

Shi Q, 2007, MOL CARCINOGEN, V46, P488, DOI 10.1002/mc.20297

Short SC, 2011, NEURO-ONCOLOGY, V13, P487, DOI 10.1093/neuonc/nor010

Sleeth KM, 2007, J MOL BIOL, V373, P38, DOI 10.1016/j.jmb.2007.07.068

Smith J, 2010, ADV CANCER RES, V108, P73, DOI 10.1016/S0065.230X(10)08002.4

Stupp R, 2005, NEW ENGL J MED, V352, P987, DOI 10.1056/NEJMoa043330

Stupp R, 2009, LANCET ONCOL, V10, P459, DOI 10.1016/S1470-2045(09)70025-7

van Gent DC, 2001, NAT REV GENET, V2, P196, DOI 10.1038/35056049

Welsh JW, 2009, INT J RADIAT ONCOL, V74, P1251, DOI 10.1016/j.ijrobp.2009.03.018

Westermark UK, 2011, NEURO-ONCOLOGY, V13, P1277, DOI 10.1093/neuonc/nor131

Yata K, 2012, MOL CELL, V45, P371, DOI 10.1016/j.molcel.2011.12.028

Zheng ZM, 2012, CANCER RES, V72, P1221, DOI 10.1158/0008-5472.CAN-11-2785

NR 53

TC 20

Z9 27

U1 0

U2 3

PU NATURE PUBLISHING GROUP

PI LONDON

PA MACMILLAN BUILDING, 4 CRINAN ST, LONDON N1 9XW, ENGLAND

SN 2041-4889

J9 CELL DEATH DIS

JI Cell Death Dis.

PD APR

PY 2015

VL 6

AR e1732

DI 10.1038/cddis.2015.75

PG 13

WC Cell Biology

WE Science Citation Index Expanded (SCI-EXPANDED)

SC Cell Biology

GA CM2LL

UT WOS:000357511500025

PM 25906155

OA Green Published, gold

DA 2025-04-09

ER

PT J

AU de Oliveira, CTP

Colenci, R

Pacheco, CC

Mariano, PM

do Prado, PR

Mamprin, GPR

Santana, MG

Gambero, A

Carvalho, PD

Priolli, DG

AF Parisi de Oliveira, Carlos Tadeu

Colenci, Renato

Pacheco, Cesar Cozar

Mariano, Patrick Moro

do Prado, Paula Ribeiro

Rosas Mamprin, Gustavo Pignatari

Santana, Maycon Giovani

Gambero, Alessandra

Carvalho, Patricia de Oliveira

Priolli, Denise Goncalves

TI Hydrolyzed Rutin Decreases Worsening of Anaplasia in Glioblastoma

Relapse

SO CNS & NEUROLOGICAL DISORDERS-DRUG TARGETS

LA English

DT Article

DE Glioblastoma; cancer; flavonoid; animal model; anaplasia; relapses

ID OXIDATIVE DNA-DAMAGE; SIGNALING PATHWAYS; QUERCETIN; CANCER; GLIOMA;

ANTIOXIDANT; BRAIN; EXPRESSION; FLAVONOIDS; APOPTOSIS

AB Background: Gliomas are aggressive and resilient tumors. Progression to advanced stages of malignancy, characterized by cell anaplasia, necrosis, and reduced response to conventional surgery or therapeutic adjuvant, are critical challenges in glioma therapy. Relapse of the disease poses a considerable challenge for management. Hence, new compounds are required to improve therapeutic response. As hydrolyzed rutin (HR). a compound modified via rutin deglycosylation, as well as some flavonoids demonstrated antiproliferative effect for glioblastoma, these are considered potential epigenetic drugs.

Objective: The purpose of this study was to determine the antitumor activity and evaluate the potential for modifying tumor aggressivity of rutin hydrolysates for treating both primary and relapsed glioblastoma.

Methods: The glioblastoma cell line, U251, was used for analyzing cell cycle inhibition and apoptosis and for establishing the GBM mouse model. Mice with GBM were treated with HR to verify antitumor activity. Histological analysis was used to evaluate HR interference in aggressive behavior and glioma grade. Immunohistochemistry, comet assay, and thiobarbituric acid reactive substance (TSARS) values were used to evaluate the mechanism of HR action.

Results: HR is an antiproliferative and antitumoral compound that inhibits the cell cycle via a p53-independent pathway. HR reduces tumor growth and aggression, mainly by decreasing mitosis and necrosis rates without genotoxicity, which is suggestive of epigenetic modulation.

Conclusion: HR possesses antitumor activity and decreases anaplasia in glioblastoma, inhibiting progression to malignant stages of the disease. HR can improve the effectiveness of response to conventional therapy, which has a crucial role in recurrent glioma.

C1 [Parisi de Oliveira, Carlos Tadeu; Colenci, Renato; Pacheco, Cesar Cozar; Mariano, Patrick Moro; do Prado, Paula Ribeiro; Rosas Mamprin, Gustavo Pignatari; Gambero, Alessandra; Carvalho, Patricia de Oliveira; Priolli, Denise Goncalves] Sao Francisco Univ, Med Sch, Braganca Paulista, SP, Brazil.

[Santana, Maycon Giovani] Sao Francisco Univ, Nurse Sch, Braganca Paulista, SP, Brazil.

[Gambero, Alessandra; Carvalho, Patricia de Oliveira; Priolli, Denise Goncalves] Sao Francisco Univ, Postgrad Program Hlth Sci, Med Sch, Av Sao Francisco Assis 218, BR-12916900 Braganca Paulista, SP, Brazil.

RP Priolli, DG (corresponding author), Multidisciplinary Lab, Av Sao Francisco Assis 218, BR-12916900 Braganca Paulista, SP, Brazil.

EM denise.priolli@usf.edu.br

RI Gambero, Alessandra/G-5792-2013; de Oliveira Carvalho,

Patricia/B-8579-2012; Santana, Maycon Giovani/AAW-1538-2020; Pacheco,

Cesar/KHU-4926-2024; priolli, denise/C-4490-2012

OI Gambero, Alessandra/0000-0002-4862-4328; Carvalho, Patricia

de/0000-0002-2681-7022; priolli, denise/0000-0003-3190-4013; Santana,

Maycon Giovani/0000-0003-2084-668X

FU Sao Paulo Research Foundation (FAPESP) [2015/07891-2, 2012/04634-1];

Vinnova [2012-04634] Funding Source: Vinnova; Swedish Research Council

[2012-04634] Funding Source: Swedish Research Council; Fundacao de

Amparo a Pesquisa do Estado de Sao Paulo (FAPESP) [12/04634-1] Funding

Source: FAPESP

FX Funding was provided by the Sao Paulo Research Foundation (FAPESP) to

DGP (grant numbers #2015/07891-2 and 2012/04634-1).

CR Aherne SA, 2000, FREE RADICAL BIO MED, V29, P507, DOI 10.1016/S0891-5849(00)00360-9

Amado NG, 2011, LIFE SCI, V89, P545, DOI 10.1016/j.lfs.2011.05.003

Baumann F, 2004, J NEURO-ONCOL, V67, P191, DOI 10.1023/B:NEON.0000021803.01170.03

Bie L, 2011, PLOS ONE, V6, DOI 10.1371/journal.pone.0025631

Duarte ACB, 2018, CNS NEUROL DISORD-DR, V17, P34, DOI 10.2174/1871527317666171221110139

Braganhol E, 2006, ANTI-CANCER DRUG, V17, P663, DOI 10.1097/01.cad.0000215063.23932.02

Cao GH, 1997, FREE RADICAL BIO MED, V22, P749, DOI 10.1016/S0891-5849(96)00351-6

Cengiz P, 2015, CHILD NERV SYST, V31, P1313, DOI 10.1007/s00381-015-2705-7

Chakravarti A, 2004, J CLIN ONCOL, V22, P1926, DOI 10.1200/JCO.2004.07.193

Davis ME, 2016, CLIN J ONCOL NURS, V20, P2, DOI 10.1188/16.CJON.S1.2-8

Gentile MT, 2015, PLOS ONE, V10, DOI 10.1371/journal.pone.0118864

Gokul S, 2017, CNS NEUROL DISORD-DR, V16, P636, DOI 10.2174/1871527316999170505104203

Hennessy BT, 2005, NAT REV DRUG DISCOV, V4, P988, DOI 10.1038/nrd1902

Jagtap S, 2009, CURR MED CHEM, V16, P1451, DOI 10.2174/092986709787909578

Ramírez-Expósito MJ, 2019, CNS NEUROL DISORD-DR, V18, P29, DOI 10.2174/1871527317666181029111739

Kim BH, 2013, MOL CELLS, V35, P410, DOI 10.1007/s10059-013-0031-z

Le UM, 2018, J DRUG TARGET, V26, P676, DOI 10.1080/1061186X.2017.1408114

Lin Q, 2018, J FUNCT FOODS, V46, P227, DOI 10.1016/j.jff.2018.05.007

Liu S, SCI REP, V7, P8302

Liu Tie Fu, 2013, Genes Cancer, V4, P135, DOI 10.1177/1947601913476948

Magalingam KB, 2013, INT J MOL MED, V32, P235, DOI 10.3892/ijmm.2013.1375

Majid S, 2008, CANCER RES, V68, P2736, DOI 10.1158/0008-5472.CAN-07-2290

Martins F, 2008, BR J NUTR, V101, P519

Martins I.J., 2017, J CLI EPIGENET, V3, P1, DOI [10.21767/2472-1158.100058, DOI 10.21767/2472-1158.100058]

de Araújo MEMB, 2013, FOOD CHEM, V141, P266, DOI 10.1016/j.foodchem.2013.02.127

Ostrom QT, 2014, NEURO-ONCOLOGY, V16, P896, DOI 10.1093/neuonc/nou087

Pan HC, 2015, NEUROCHEM INT, V80, P60, DOI 10.1016/j.neuint.2014.12.001

Panieri E, 2016, CELL DEATH DIS, V7, DOI 10.1038/cddis.2016.105

Piñeros M, 2016, CANCER EPIDEMIOL, V44, pS141, DOI 10.1016/j.canep.2016.04.007

Priolli DG, 2013, INT J COLORECTAL DIS, V28, P713, DOI 10.1007/s00384-013-1688-7

Qu Y, 2012, NEUROSCI LETT, V525, P168, DOI 10.1016/j.neulet.2012.07.025

Ribeiro ML, 2008, CLIN COLORECTAL CANC, V7, P267, DOI 10.3816/CCC.2008.n.034

Roy S, 2017, SOUTH ASIAN J CANCER, V6, P153, DOI 10.4103/2278-330X.221358

Sánchez-Pérez Y, 2017, CNS NEUROL DISORD-DR, V16, P1090, DOI 10.2174/1871527317666180110124645

Scalise Jose Ricardo, 2016, J Gastrointest Cancer, V47, P409

TOMAYKO MM, 1989, CANCER CHEMOTH PHARM, V24, P148, DOI 10.1007/BF00300234

Vauzour D, 2007, J NEUROCHEM, V103, P1355, DOI 10.1111/j.1471-4159.2007.04841.x

Wang LK, 2014, INT J MOL SCI, V15, P16226, DOI 10.3390/ijms150916226

Wang RH, 2008, MOL CELL, V32, P11, DOI 10.1016/j.molcel.2008.09.011

Wang RH, 2008, CANCER CELL, V14, P312, DOI 10.1016/j.ccr.2008.09.001

Yang ZJ, 2017, CNS NEUROL DISORD-DR, V16, P160, DOI 10.2174/1871527315666161018122909

Zappe K, 2018, OXID MED CELL LONGEV, V2018, DOI 10.1155/2018/3734250

Zhan TM, 2017, CNS NEUROL DISORD-DR, V16, P129, DOI 10.2174/1871527316666170113101559

Zhang PD, 2017, J NEURO-ONCOL, V132, P393, DOI 10.1007/s11060-017-2387-y

Zhang Y, 2018, CANCERS, V10, DOI 10.3390/cancers10090297

Zhang ZQ, 2017, CNS NEUROL DISORD-DR, V16, P346, DOI 10.2174/1871527315666160902144513

NR 46

TC 9

Z9 9

U1 0

U2 7

PU BENTHAM SCIENCE PUBL

PI BUSUM

PA PO BOX 294, BUSUM, 1400 AG, NETHERLANDS

SN 1871-5273

EI 1996-3181

J9 CNS NEUROL DISORD-DR

JI CNS Neurol. Disord.-Drug Targets

PY 2019

VL 18

IS 5

BP 405

EP 412

DI 10.2174/1871527318666190314103104

PG 8

WC Neurosciences; Pharmacology & Pharmacy

WE Science Citation Index Expanded (SCI-EXPANDED)

SC Neurosciences & Neurology; Pharmacology & Pharmacy

GA IZ8CB

UT WOS:000487329000005

PM 30868970

DA 2025-04-09

ER

PT J

AU Hayashima, K

Katoh, H

AF Hayashima, Kazuki

Katoh, Hironori

TI Expression of gamma-glutamyltransferase 1 in glioblastoma cells confers

resistance to cystine deprivation-induced ferroptosis

SO JOURNAL OF BIOLOGICAL CHEMISTRY

LA English

DT Article

ID HIPPO PATHWAY; DRUG-RESISTANCE; TRANSPEPTIDASE; INHIBITION; DEATH;

GLUTATHIONE

AB Ferroptosis is an iron-dependent mode of cell death caused by excessive oxidative damage to lipids. Lipid peroxidation is normally suppressed by glutathione peroxidase 4, which requires reduced glutathione. Cystine is a major resource for glutathione synthesis, especially in cancer cells. Therefore, cystine deprivation or inhibition of cystine uptake promotes ferroptosis in cancer cells. However, the roles of other molecules involved in cysteine deprivation-induced ferroptosis are unexplored. We report here that the expression of gamma-glutamyltransferase 1 (GGT1), an enzyme that cleaves extracellular glutathione, determines the sensitivity of glioblastoma cells to cystine deprivation-induced ferroptosis at high cell density (HD). In glioblastoma cells expressing GGT1, pharmacological inhibition or deletion of GGT1 suppressed the cell density-induced increase in intracellular glutathione levels and cell viability under cystine deprivation, which were restored by the addition of cysteinylglycine, the GGT product of glutathione cleavage. On the other hand, cystine deprivation induced glutathione depletion and ferroptosis in GGT1deficient glioblastoma cells even at an HD. Exogenous expression of GGT1 in GGT1-deficient glioblastoma cells inhibited cystine deprivation-induced glutathione depletion and ferroptosis at an HD. This suggests that GGT1 plays an important role in glioblastoma cell survival under cystine-limited and HD conditions. We conclude that combining GGT inhibitors with ferroptosis inducers may provide an effective therapeutic approach for treating glioblastoma.

C1 [Hayashima, Kazuki; Katoh, Hironori] Kyoto Univ, Grad Sch Biostudies, Lab Mol Neurobiol, Kyoto, Japan.

C3 Kyoto University

RP Katoh, H (corresponding author), Kyoto Univ, Grad Sch Biostudies, Lab Mol Neurobiol, Kyoto, Japan.

EM hirokato@pharm.kyoto-u.ac.jp

FU Japan Society for the Promotion of Science [18K06215, 21K06065];

Grants-in-Aid for Scientific Research [21K06065] Funding Source: KAKEN

FX We thank Prof Junichi Miyazaki (Osaka University, Osaka, Japan) for the

CAG promoter-containing vector. We also thank Dr Yohei Katoh and Prof

Kazuhisa Nakayama (Kyoto University) for providing peSpCAS9(1.1)-2xsgRNA

and pDonortBFP-NLS-Neo. This study was supported in part by Grants-inaid

for Scientific Research from the Japan Society for the Promotion of

Science (grant nos.: 18K06215 and 21K06065).

CR Bansal A, 2019, MOL CANCER RES, V17, P1881, DOI 10.1158/1541-7786.MCR-18-1204

Bansal A, 2018, J CELL BIOL, V217, P2291, DOI 10.1083/jcb.201804161

Batsios G, 2020, SCI REP-UK, V10, DOI 10.1038/s41598-020-63160-y

Briggs KJ, 2016, CELL, V166, P126, DOI 10.1016/j.cell.2016.05.042

Corti A, 2010, ANTICANCER RES, V30, P1169

Dixon SJ, 2019, ANNU REV CANC BIOL, V3, P35, DOI 10.1146/annurev-cancerbio-030518-055844

Dixon SJ, 2012, CELL, V149, P1060, DOI 10.1016/j.cell.2012.03.042

Dolecek TA, 2012, NEURO-ONCOLOGY, V14, pv1, DOI 10.1093/neuonc/nos218

Goji T, 2017, J BIOL CHEM, V292, P19721, DOI 10.1074/jbc.M117.814392

GRIFFITH OW, 1978, P NATL ACAD SCI USA, V75, P5405, DOI 10.1073/pnas.75.11.5405

Gujral TS, 2017, P NATL ACAD SCI USA, V114, pE3729, DOI 10.1073/pnas.1703096114

HANES CS, 1950, NATURE, V166, P288, DOI 10.1038/166288a0

HANIGAN MH, 1995, CARCINOGENESIS, V16, P181, DOI 10.1093/carcin/16.2.181

Hayashima K, 2021, BIOCHEM BIOPH RES CO, V539, P56, DOI 10.1016/j.bbrc.2020.12.075

Heisterkamp N, 2008, HUM GENET, V123, P321, DOI 10.1007/s00439-008-0487-7

Horie K, 2020, ONCOLOGY-BASEL, V98, P734, DOI 10.1159/000508688

Hughes CE, 2020, CELL, V180, P296, DOI 10.1016/j.cell.2019.12.035

Ji Y, 2016, AMINO ACIDS, V48, P149, DOI 10.1007/s00726-015-2071-5

Katoh Y, 2017, MOL BIOL CELL, V28, P898, DOI 10.1091/mbc.E17-01-0051

Kawakami K, 2017, BMC CANCER, V17, DOI 10.1186/s12885-017-3301-x

KOBAYASHI H, 1992, CANCER CHEMOTH PHARM, V31, P6, DOI 10.1007/BF00695987

Koppula P, 2021, PROTEIN CELL, V12, P599, DOI 10.1007/s13238-020-00789-5

Lewerenz J, 2013, ANTIOXID REDOX SIGN, V18, P522, DOI 10.1089/ars.2011.4391

Lieberman MW, 1996, P NATL ACAD SCI USA, V93, P7923, DOI 10.1073/pnas.93.15.7923

Liu XG, 2021, GENES DIS, V8, P731, DOI 10.1016/j.gendis.2020.11.010

MEISTER A, 1983, ANNU REV BIOCHEM, V52, P711, DOI 10.1146/annurev.bi.52.070183.003431

MEYTS ERD, 1992, TOXICOL APPL PHARM, V114, P56, DOI 10.1016/0041-008X(92)90096-B

Pavel M, 2018, NAT COMMUN, V9, DOI 10.1038/s41467-018-05388-x

Poltorack CD, 2022, FEBS J, V289, P374, DOI 10.1111/febs.15842

Reuven N, 2013, CELL DEATH DIFFER, V20, P1330, DOI 10.1038/cdd.2013.83

Shimomura T, 2021, ACS SENSORS, V6, P2125, DOI 10.1021/acssensors.1c00496

Suzuki H, 2002, J BIOL CHEM, V277, P43536, DOI 10.1074/jbc.M207680200

Tan SK, 2018, FRONT PHARMACOL, V9, DOI 10.3389/fphar.2018.00218

West MB, 2011, J BIOL CHEM, V286, P28876, DOI 10.1074/jbc.M111.248823

Westhoff MA, 2008, ONCOGENE, V27, P5169, DOI 10.1038/onc.2008.148

Wu J, 2019, NATURE, V572, P402, DOI 10.1038/s41586-019-1426-6

Yamaguchi I, 2020, J BIOL CHEM, V295, P6936, DOI 10.1074/jbc.RA119.012213

Yang M, 2016, NAT REV CANCER, V16, P650, DOI 10.1038/nrc.2016.81

Yang WH, 2020, MOL CANCER RES, V18, P79, DOI 10.1158/1541-7786.MCR-19-0691

Yang WH, 2019, CELL REP, V28, P2501, DOI 10.1016/j.celrep.2019.07.107

Zhao B, 2007, GENE DEV, V21, P2747, DOI 10.1101/gad.1602907

NR 41

TC 31

Z9 32

U1 0

U2 14

PU ELSEVIER

PI AMSTERDAM

PA RADARWEG 29, 1043 NX AMSTERDAM, NETHERLANDS

EI 1083-351X

J9 J BIOL CHEM

JI J. Biol. Chem.

PD MAR

PY 2022

VL 298

IS 3

AR 101703

DI 10.1016/j.jbc.2022.101703

EA MAR 2022

PG 11

WC Biochemistry & Molecular Biology

WE Science Citation Index Expanded (SCI-EXPANDED)

SC Biochemistry & Molecular Biology

GA 2R4AJ

UT WOS:000821054100004

PM 35148992

OA Green Published, gold

DA 2025-04-09

ER

PT J

AU Zaher, A

Mapuskar, KA

Sarkaria, JN

Spitz, DR

Petronek, MS

Allen, BG

AF Zaher, Amira

Mapuskar, Kranti A.

Sarkaria, Jann N.

Spitz, Douglas R.

Petronek, Michael S.

Allen, Bryan G.

TI Differential H2O2 Metabolism among Glioblastoma

Subtypes Confers Variable Responses to Pharmacological Ascorbate Therapy

Combined with Chemoradiation

SO INTERNATIONAL JOURNAL OF MOLECULAR SCIENCES

LA English

DT Article

DE glioblastoma; chemoradiation; glioblastoma subtypes; pharmacological

ascorbate; antioxidant therapy; prooxidant; hydrogen peroxide; DNA

damage

ID OXIDATIVE DNA-DAMAGE; HYDROGEN-PEROXIDE; CANCER-CELLS; ASSAY; ACID

AB Glioblastoma (GBM), a highly lethal and aggressive central nervous system malignancy, presents a critical need for targeted therapeutic approaches to improve patient outcomes in conjunction with standard-of-care (SOC) treatment. Molecular subtyping based on genetic profiles and metabolic characteristics has advanced our understanding of GBM to better predict its evolution, mechanisms, and treatment regimens. Pharmacological ascorbate (P-AscH(-)) has emerged as a promising supplementary cancer therapy, leveraging its pro-oxidant properties to selectively kill malignant cells when combined with SOC. Given the clinical challenges posed by the heterogeneity and resistance of various GBM subtypes to conventional SOC, our study assessed the response of classical, mesenchymal, and proneural GBM to P-AscH(-). P-AscH(-) (20 pmol/cell) combined with SOC (5 mu M temozolomide and 4 Gy of radiation) enhanced clonogenic cell killing in classical and mesenchymal GBM subtypes, with limited effects in the proneural subtype. Similarly, following exposure to P-AscH(-) (20 pmol/cell), single-strand DNA damage significantly increased in classical and mesenchymal but not proneural GBM. Moreover, proneural GBM exhibited increased hydrogen peroxide removal rates, along with increased catalase and glutathione peroxidase activities compared to mesenchymal and classical GBM, demonstrating an altered H2O2 metabolism that potentially drives differential P-AscH- toxicity. Taken together, these data suggest that P-AscH- may hold promise as an approach to improve SOC responsiveness in mesenchymal GBMs that are known for their resistance to SOC.

C1 [Zaher, Amira; Mapuskar, Kranti A.; Spitz, Douglas R.; Petronek, Michael S.; Allen, Bryan G.] Univ Iowa, Dept Radiat Oncol, Iowa City, IA 52242 USA.

[Sarkaria, Jann N.] Mayo Clin, Dept Radiat Oncol, Rochester, MN 55905 USA.

C3 University of Iowa; Mayo Clinic

RP Petronek, MS; Allen, BG (corresponding author), Univ Iowa, Dept Radiat Oncol, Iowa City, IA 52242 USA.

EM amira-zaher@uiowa.edu; krantiashok-mapuskar@uiowa.edu;

sarkaria.jann@mayo.edu; douglas-spitz@uiowa.edu;

michael-petronek@uiowa.edu; bryan-allen@uiowa.edu

RI Petronek, Michael/HLH-7071-2023; Zaher, Amira/JNT-0363-2023; Mapuskar,

Kranti/AAX-3923-2021

OI mapuskar, kranti/0000-0002-7311-7184; Zaher, Amira/0000-0001-7030-0576

FU National Institutes of Health; National Cancer Institute [P01CA244091]

Funding Source: NIH RePORTER

FX We would like to acknowledge Jann N. Sarkaria at the Mayo Clinic for

providing patient-derived GBM cell lines used in this study. We would

also like to acknowledge the Radiation Core, the Antioxidant Core, and

the Small Animal Imaging Core in the Holden Comprehensive Cancer Center

at the University of Iowa.

CR AEBI H, 1984, METHOD ENZYMOL, V105, P121

Ahmed MH, 2023, REV NEUROL-FRANCE, V179, P430, DOI 10.1016/j.neurol.2023.03.013

Aiyappa-Maudsley R, 2022, NEURO-ONCOL ADV, V4, DOI 10.1093/noajnl/vdac156

Alexander MS, 2018, CANCER RES, V78, P6838, DOI 10.1158/0008-5472.CAN-18-1680

Behnan J, 2019, BRAIN, V142, P847, DOI 10.1093/brain/awz044

Cannan WJ, 2016, J CELL PHYSIOL, V231, P3, DOI 10.1002/jcp.25048

Chen Q, 2005, P NATL ACAD SCI USA, V102, P13604, DOI 10.1073/pnas.0506390102

Chen Q, 2008, P NATL ACAD SCI USA, V105, P11105, DOI 10.1073/pnas.0804226105

Doskey CM, 2016, REDOX BIOL, V10, P274, DOI 10.1016/j.redox.2016.10.010

Du J, 2015, CANCER RES, V75, P3314, DOI 10.1158/0008-5472.CAN-14-1707

Duarte TL, 2007, TOXICOL LETT, V170, P57, DOI 10.1016/j.toxlet.2007.02.005

Fedele M, 2019, INT J MOL SCI, V20, DOI 10.3390/ijms20112746

Franken NAP, 2006, NAT PROTOC, V1, P2315, DOI 10.1038/nprot.2006.339

FREIFELDER D, 1969, BIOPOLYMERS, V7, P681, DOI 10.1002/bip.1969.360070506

Gutierrez-Quintana R, 2022, NEURO-ONCOL ADV, V4, DOI 10.1093/noajnl/vdab190

HEINZ-ERIAN P, 1985, Paediatrie und Paedologie, V20, P49

Henle ES, 1997, J BIOL CHEM, V272, P19095, DOI 10.1074/jbc.272.31.19095

Herst PM, 2012, FREE RADICAL BIO MED, V52, P1486, DOI 10.1016/j.freeradbiomed.2012.01.021

Kaffes I, 2019, ONCOIMMUNOLOGY, V8, DOI 10.1080/2162402X.2019.1655360

Lam MSY, 2023, SMALL, V19, DOI 10.1002/smll.202302280

LAWRENCE RA, 1976, BIOCHEM BIOPH RES CO, V71, P952, DOI 10.1016/0006-291X(76)90747-6

Li RQ, 2022, BIOMATER SCI-UK, V10, P892, DOI 10.1039/d1bm01401c

Ma EL, 2017, FREE RADICAL BIO MED, V113, P36, DOI 10.1016/j.freeradbiomed.2017.09.008

Nordberg J, 2001, FREE RADICAL BIO MED, V31, P1287, DOI 10.1016/S0891-5849(01)00724-9

Ostrom QT, 2021, NEURO-ONCOLOGY, V23, P1, DOI 10.1093/neuonc/noab200

Ostrom QT, 2019, NEURO-ONCOLOGY, V21, pV1, DOI 10.1093/neuonc/noz150

Ou A, 2021, INT J MOL SCI, V22, DOI 10.3390/ijms22010351

Petronek MS, 2023, REDOX BIOL, V62, DOI 10.1016/j.redox.2023.102651

Petronek MS, 2021, REDOX BIOL, V42, DOI 10.1016/j.redox.2021.101864

Petronek MS, 2024, CLIN CANCER RES, V30, P283, DOI 10.1158/1078-0432.CCR-22-3952

Phillips HS, 2006, CANCER CELL, V9, P157, DOI 10.1016/j.ccr.2006.02.019

REIBER H, 1993, CLIN CHIM ACTA, V217, P163, DOI 10.1016/0009-8981(93)90162-W

Rivière J, 2006, FREE RADICAL BIO MED, V40, P2071, DOI 10.1016/j.freeradbiomed.2006.02.003

Schoenfeld JD, 2019, SEMIN RADIAT ONCOL, V29, P25, DOI 10.1016/j.semradonc.2018.10.006

Schoenfeld JD, 2017, CANCER CELL, V31, P487, DOI 10.1016/j.ccell.2017.02.018

Stupp R, 2005, NEW ENGL J MED, V352, P987, DOI 10.1056/NEJMoa043330

Turner N, 2023, CRIT CARE RESUSC, V25, P175, DOI 10.1016/j.ccrj.2023.10.003

Turnquist C, 2020, NEURO-ONCOL ADV, V2, DOI 10.1093/noajnl/vdaa057

Verhaak RGW, 2010, CANCER CELL, V17, P98, DOI 10.1016/j.ccr.2009.12.020

Wagner BA, 2013, REDOX BIOL, V1, P210, DOI 10.1016/j.redox.2013.01.011

Wang QH, 2017, CANCER CELL, V32, P42, DOI [10.1016/j.ccell.2017.06.003, 10.1016/j.ccell.2017.12.012]

Wen PY, 2008, NEW ENGL J MED, V359, P492, DOI 10.1056/NEJMra0708126

Zaher A, 2022, FRONT IMMUNOL, V13, DOI 10.3389/fimmu.2022.989000

Zhou LF, 2020, FREE RADICAL RES, V54, P385, DOI 10.1080/10715762.2020.1744577

NR 44

TC 4

Z9 4

U1 0

U2 1

PU MDPI

PI BASEL

PA ST ALBAN-ANLAGE 66, CH-4052 BASEL, SWITZERLAND

SN 1661-6596

EI 1422-0067

J9 INT J MOL SCI

JI Int. J. Mol. Sci.

PD DEC

PY 2023

VL 24

IS 24

AR 17158

DI 10.3390/ijms242417158

PG 12

WC Biochemistry & Molecular Biology; Chemistry, Multidisciplinary

WE Science Citation Index Expanded (SCI-EXPANDED)

SC Biochemistry & Molecular Biology; Chemistry

GA DL4R2

UT WOS:001132189900001

PM 38138986

OA Green Published, gold

DA 2025-04-09

ER

PT J

AU Peeri, NC

Creed, JH

Anic, GM

Thompson, RC

Olson, JJ

LaRocca, RV

Chowdhary, SA

Brockman, JD

Gerke, TA

Nabors, LB

Egan, KM

AF Peeri, Noah C.

Creed, Jordan H.

Anic, Gabriella M.

Thompson, Reid C.

Olson, Jeffrey J.

LaRocca, Renato, V

Chowdhary, Sajeel A.

Brockman, John D.

Gerke, Travis A.

Nabors, L. Burton

Egan, Kathleen M.

TI Toenail selenium, genetic variation in selenoenzymes and risk and

outcome in glioma

SO CANCER EPIDEMIOLOGY

LA English

DT Article

DE Glioma; Selenium; Single nucleotide polymorphism; Case-control study; UK

biobank

ID GENOME-WIDE ASSOCIATION; CANCER RISK; BRAIN; TUMORS; BLOOD;

SELENOCYSTEINE; GROWTH; AGE; SE

AB Background: Selenium is an essential trace element obtained through diet that plays a critical role in DNA synthesis and protection from oxidative damage. Selenium intake and polymorphisms in selenoproteins have been linked to the risk of certain cancers though data for glioma are sparse.

Methods: In a case-control study of glioma, we examined the associations of selenium in toenails and genetic variants in the selenoenzyme pathway with the risk of glioma and patient survival. A total of 423 genetic variants in 29 candidate genes in the selenoenzyme pathway were studied in 1547 glioma cases and 1014 healthy controls. Genetic associations were also examined in the UK Biobank cohort comprised of 313,868 persons with 322 incident glioma cases. Toenail selenium was measured in a subcohort of 300 glioma cases and 300 age-matched controls from the case-control study.

Results: None of the 423 variants studied were consistently associated with glioma risk in the case-control and cohort studies. Moreover, toenail selenium in the case-control study had no significant association with glioma risk (p trend = 0.70) or patient survival among 254 patients with high grade tumors (p trend = 0.70).

Conclusion: The present study offers no support for the hypothesis that selenium plays a role in the onset of glioma or patient outcome.

C1 [Peeri, Noah C.; Creed, Jordan H.; Anic, Gabriella M.; Gerke, Travis A.; Egan, Kathleen M.] H Lee Moffitt Canc Ctr & Res Inst, Dept Canc Epidemiol, Tampa, FL 33612 USA.

[Thompson, Reid C.] Vanderbilt Univ, Med Ctr, Dept Neurol Surg, Nashville, TN 37232 USA.

[Nabors, L. Burton] Univ Alabama Birmingham, Neurooncol Program, Birmingham, AL 35294 USA.

[Olson, Jeffrey J.] Emory Sch Med, Dept Neurosurg, Atlanta, GA 30322 USA.

[LaRocca, Renato, V] Norton Canc Inst, Louisville, KY 40202 USA.

[Chowdhary, Sajeel A.] Lynn Canc Inst, Neurooncol Program, 701 NW 13th St, Boca Raton, FL 33486 USA.

[Brockman, John D.] Univ Missouri, Univ Missouri Res Reactor, Columbia, MO 65211 USA.

C3 H Lee Moffitt Cancer Center & Research Institute; Vanderbilt University;

University of Alabama System; University of Alabama Birmingham; Emory

University; University of Missouri System; University of Missouri

Columbia

RP Egan, KM (corresponding author), H Lee Moffitt Canc Ctr & Res Inst, Dept Canc Epidemiol, 12902 Magnolia Dr, Tampa, FL 33612 USA.

EM kathleen.egan@moffitt.org

RI Nabors, Louis/ABB-8223-2021

OI Peeri, Noah/0000-0003-4278-650X; Thompson, Reid/0000-0002-9747-5330;

Brockman, John/0000-0001-7419-5558

FU National Institutes of Health [R01 CA116174, R03 CA171612]

FX The research was supported by the National Institutes of Health [grant

number R01 CA116174 and R03 CA171612]. The work is based in part on the

UK Biobank Resource under application number 16944.

CR Amirian ES, 2016, CANCER MED-US, V5, P1352, DOI 10.1002/cam4.682

Amirian ES, 2016, CANCER EPIDEM BIOMAR, V25, P282, DOI 10.1158/1055-9965.EPI-15-0847

Anic GM, 2013, CANCER CAUSE CONTROL, V24, P2051, DOI 10.1007/s10552-013-0281-2

[Anonymous], 2017, J NATL CANC I

Arslan M, 2011, ASIAN PAC J CANCER P, V12, P447

Braganza MZ, 2012, NEURO-ONCOLOGY, V14, P1316, DOI 10.1093/neuonc/nos208

Bräuer AU, 2004, REV NEUROSCIENCE, V15, P19

Cardoso BR, 2014, J TRACE ELEM MED BIO, V28, P422, DOI 10.1016/j.jtemb.2014.08.009

Collins R, 2012, LANCET, V379, P1173, DOI [10.1016/S0140-6736(12)60404-8, 10.1016/S0140-6736(16)31357-5]

Cornelis MC, 2015, HUM MOL GENET, V24, P1469, DOI 10.1093/hmg/ddu546

Egan KM, 2012, J MED GENET, V49, P420, DOI 10.1136/jmedgenet-2012-100941

Egan KM, 2011, J NEURO-ONCOL, V104, P535, DOI 10.1007/s11060-010-0506-0

ELYAZIGI A, 1984, CLIN CHEM, V30, P1358

Farrell CJ, 2007, NEUROL CLIN, V25, P925, DOI 10.1016/j.ncl.2007.07.008

Freeman LEB, 2015, AM J EPIDEMIOL, V181, P488, DOI 10.1093/aje/kwu324

GARLAND M, 1993, CANCER EPIDEM BIOMAR, V2, P493

Gustavsson N., 2001, Geochemical landscapes of the conterminous United States - New map presentations for 22 elements

Hadaszadeh BM, 2006, PHYSIOLOGY, V21, P307, DOI 10.1152/physiol.00021.2006

Hesketh J, 2011, P NUTR SOC, V70, P365, DOI 10.1017/S0029665111000115

Hughes DJ, 2015, INT J CANCER, V136, P1149, DOI 10.1002/ijc.29071

Jain RB, 2015, J TRACE ELEM MED BIO, V30, P142, DOI 10.1016/j.jtemb.2014.12.004

Johansson L, 2005, BBA-GEN SUBJECTS, V1726, P1, DOI 10.1016/j.bbagen.2005.05.010

Kim Y, 2015, ANTICANCER RES, V35, P4983

Kohler BA, 2011, JNCI-J NATL CANCER I, V103, P714, DOI 10.1093/jnci/djr077

Krishnamachari B, 2014, CANCER CAUSE CONTROL, V25, P1007, DOI 10.1007/s10552-014-0400-8

Li HJ, 2004, JNCI-J NATL CANCER I, V96, P696, DOI 10.1093/jnci/djh125

Li JL, 2012, PLOS ONE, V7, DOI 10.1371/journal.pone.0035761

Little RB, 2017, CANCER CAUSE CONTROL, V28, P709, DOI 10.1007/s10552-017-0871-5

Little RB, 2013, CANCER CAUSE CONTROL, V24, P1025, DOI 10.1007/s10552-013-0178-0

Longnecker MP, 1996, EPIDEMIOLOGY, V7, P384, DOI 10.1097/00001648-199607000-00008

LONGNECKER MP, 1993, AM J CLIN NUTR, V57, P408, DOI 10.1093/ajcn/57.3.408

Lu SC, 2013, BBA-GEN SUBJECTS, V1830, P3143, DOI 10.1016/j.bbagen.2012.09.008

Mathers JC, 2010, INT J VITAM NUTR RES, V80, P314, DOI 10.1024/0300-9831/a000039

Melin BS, 2017, NAT GENET, V49, P789, DOI 10.1038/ng.3823

Méplan C, 2014, CANCER TREAT RES, V159, P145, DOI 10.1007/978-3-642-38007-5_9

Méplan C, 2009, ANTIOXID REDOX SIGN, V11, P2631, DOI [10.1089/ars.2009.2533, 10.1089/ARS.2009.2533]

Ostrom QT, 2014, NEURO-ONCOLOGY, V16, P896, DOI 10.1093/neuonc/nou087

Oudouhou F, 2017, BIOCHEMISTRY-US, V56, P2261, DOI 10.1021/acs.biochem.6b01116

Park K, 2011, NUTR RES PRACT, V5, P357, DOI 10.4162/nrp.2011.5.4.357

Philipov P, 1988, Zentralbl Neurochir, V49, P344

Rajaraman P, 2009, CANCER EPIDEM BIOMAR, V18, P1651, DOI 10.1158/1055-9965.EPI-08-1041

Rayman MP, 2012, LANCET, V379, P1256, DOI 10.1016/S0140-6736(11)61452-9

Rice T, 2016, NEURO-ONCOL PRACT, V3, P10, DOI 10.1093/nop/npv026

ROSENTHAL R, 1978, PSYCHOL BULL, V85, P185, DOI 10.1037/0033-2909.85.1.185

Savaskan NE, 2007, BIOL CHEM, V388, P1007, DOI 10.1515/BC.2007.126

Schweizer U, 2004, J NUTR, V134, P707, DOI 10.1093/jn/134.4.707

Slattery M. L., 2011, PLOS ONE, V7

Stupp R, 2005, NEW ENGL J MED, V352, P987, DOI 10.1056/NEJMoa043330

Sundaram N, 2000, J NEURO-ONCOL, V46, P125, DOI 10.1023/A:1006436326003

Sunde R.A., 2012, MODERN NUTR HLTH DIS

Tobe R, 2016, BIOCHEM J, V473, P2141, DOI 10.1042/BCJ20160393

Vinceti M, 2014, COCHRANE DB SYST REV, DOI 10.1002/14651858.CD005195.pub3

Winkel LHE, 2012, ENVIRON SCI TECHNOL, V46, P571, DOI 10.1021/es203434d

Yakubov E, 2014, BIOL TRACE ELEM RES, V161, P246, DOI 10.1007/s12011-014-0111-8

NR 54

TC 1

Z9 1

U1 0

U2 4

PU ELSEVIER SCI LTD

PI OXFORD

PA THE BOULEVARD, LANGFORD LANE, KIDLINGTON, OXFORD OX5 1GB, OXON, ENGLAND

SN 1877-7821

EI 1877-783X

J9 CANCER EPIDEMIOL

JI Cancer Epidemiol.

PD AUG

PY 2018

VL 55

BP 45

EP 51

DI 10.1016/j.canep.2018.05.002

PG 7

WC Oncology; Public, Environmental & Occupational Health

WE Science Citation Index Expanded (SCI-EXPANDED)

SC Oncology; Public, Environmental & Occupational Health

GA GN8NN

UT WOS:000439421300007

PM 29777993

OA Green Accepted

DA 2025-04-09

ER

PT J

AU Kutwin, M

Sawosz, E

Jaworski, S

Wierzbicki, M

Strojny, B

Grodzik, M

Chwalibog, A

AF Kutwin, Marta

Sawosz, Ewa

Jaworski, Slawomir

Wierzbicki, Mateusz

Strojny, Barbara

Grodzik, Marta

Chwalibog, Andre

TI Assessment of the proliferation status of glioblastoma cell and tumour

tissue after nanoplatinum treatment

SO PLOS ONE

LA English

DT Article

ID PLATINUM NANOPARTICLES; DNA-DAMAGE; CISPLATIN; GLIOMAS; RADIOTHERAPY;

APOPTOSIS; INHIBIT; MARKERS

AB Glioblastoma is one of the most frequent primary brain tumours of the central nervous system, with a poor survival time. With inefficient chemotherapy, it is urgent to develop new strategies for tumour therapy. The present approach is based on the inhibition of cell proliferation using platinum nanoparticles (NP-Pt). The aim of the study was to evaluate and compare the antiproliferative properties of NP-Pt and cisplatin against U87 and U118 glioma cell lines and U87 tumour tissue. NP-Pt and cisplatin were incubated with U87 and U118 glioma cells or administered directly into glioma tumour tissue. Cell morphology, the level of DNA synthesis, the migration of cells, protein expression levels of proliferating cell nuclear antigen (PCNA) and the level of DNA oxidation in glioma tumours were investigated. The results showed that NP-Pt treatment of U87 and U118 glioma cells decreased the level of DNA synthesis and the migration of cancer cells but also downregulated the level of PCNA protein expression in tumour tissue. Furthermore, NP-Pt caused oxidative DNA damage in tumour tissue to a higher degree than cisplatin. Consequently, NP-Pt can be considered as an effective inhibitor of glioblastoma tumour cell proliferation. However, the mechanism of action and potential side effects need to be elucidated further.

C1 [Kutwin, Marta; Sawosz, Ewa; Jaworski, Slawomir; Wierzbicki, Mateusz; Strojny, Barbara; Grodzik, Marta; Chwalibog, Andre] Warsaw Univ Life Sci, Fac Anim Sci, Dept Anim Nutr & Biotechnol, Warsaw, Poland.

[Chwalibog, Andre] Univ Copenhagen, Fac Hlth & Med Sci, Dept Vet & Anim Sci, Frederiksberg, Denmark.

C3 Warsaw University of Life Sciences; University of Copenhagen

RP Chwalibog, A (corresponding author), Univ Copenhagen, Fac Hlth & Med Sci, Dept Vet & Anim Sci, Frederiksberg, Denmark.

EM ach@sund.ku.dk

RI Grodzik, Marta/AAO-4319-2021; Wierzbicki, Mateusz/ABF-5482-2020;

Jaworski, Sławomir/AAI-2313-2020; Kutwin, Marta/AAI-6887-2020; Grodzik,

Marta/J-9404-2015

OI Sawosz Chwalibog, Ewa/0000-0002-0016-4721; Jaworski,

Slawomir/0000-0002-4619-941X; Kutwin, Marta/0000-0001-7059-7875;

Wierzbicki, Mateusz/0000-0003-3623-8929; Strojny,

Barbara/0000-0002-2642-5491; Chwalibog, Andre/0000-0001-8150-2392;

Grodzik, Marta/0000-0001-5359-1885

FU National Centre of Science, Poland [NCN 2013/09/N/NZ9/01895]; NCN

2013/09/N/NZ9/01895

FX This work was supported by the National Centre of Science, Poland, grant

NCN 2013/09/N/NZ9/01895 to MK.This work was supported by grant NCN

2013/09/N/NZ9/01895.

CR Alen M, 2016, SCI TRANSL MED, V354

Alshatwi AA, 2015, J MATER SCI-MATER M, V26, DOI 10.1007/s10856-015-5468-5

[Anonymous], J NANOSCALE RES LETT

Artelt S, 1999, SCI TOTAL ENVIRON, V228, P219, DOI 10.1016/S0048-9697(99)00049-2

Asharani PV, 2010, NANOMEDICINE-UK, V5, P51, DOI 10.2217/NNM.09.85

Bhattacharya R, 2007, ADV MATER, V19, P711, DOI 10.1002/adma.200602098

Butler JS, 2013, CURR OPIN CHEM BIOL, V17, P175, DOI 10.1016/j.cbpa.2013.01.004

Cemazar M, 1998, ANTI-CANCER DRUG, V9, P525

Chaney SG, 2005, CRIT REV ONCOL HEMAT, V53, P3, DOI 10.1016/j.critrevonc.2004.08.008

Cloughesy T, 2011, SEMIN ONCOL, V38, pS11, DOI 10.1053/j.seminoncol.2011.09.007

Cunningham JM, 1997, J NEUROSURG, V86, P121, DOI 10.3171/jns.1997.86.1.0121

Elder A, 2007, ADV MATER, V19, P3124, DOI 10.1002/adma.200701962

Enoiu M, 2012, NUCLEIC ACIDS RES, V40, P8953, DOI 10.1093/nar/gks670

Gehrke H, 2011, ARCH TOXICOL, V85, P799, DOI 10.1007/s00204-010-0636-3

Grodzik M, 2011, INT J NANOMED, V6, P3041, DOI 10.2147/IJN.S25528

Jawaid P, 2014, APOPTOSIS, V19, P1006, DOI 10.1007/s10495-014-0972-5

Kartalou M, 2001, MUTAT RES-FUND MOL M, V478, P23, DOI 10.1016/S0027-5107(01)00141-5

KONDO S, 1995, BRIT J CANCER, V71, P282, DOI 10.1038/bjc.1995.57

KUBBEN FJGM, 1994, GUT, V35, P530, DOI 10.1136/gut.35.4.530

Kutwin M, 2016, ARCH MED SCI

Li H, 2016, 2016 IEEE INTERNATIONAL CONFERENCE ON RECENT TRENDS IN ELECTRONICS, INFORMATION & COMMUNICATION TECHNOLOGY (RTEICT), P1, DOI 10.1109/RTEICT.2016.7807769

López T, 2010, EUR J MED CHEM, V45, P1982, DOI 10.1016/j.ejmech.2010.01.043

Mohammadi H, 2013, INT NANO LETT, V3, DOI 10.1186/2228-5326-3-28

Nakada M, 2007, CELL MOL LIFE SCI, V64, P458, DOI 10.1007/s00018-007-6342-5

Porcel E, 2010, NANOTECHNOLOGY, V21, DOI 10.1088/0957-4484/21/8/085103

Prasek M, 2013, NANOSCALE RES LETT, V8, DOI 10.1186/1556-276X-8-251

Strojnik T, 2010, ANTICANCER RES, V30, P4851

Stupp R, 2005, NEW ENGL J MED, V352, P987, DOI 10.1056/NEJMoa043330

Swift LH, 2014, INT J MOL SCI, V15, P3403, DOI 10.3390/ijms15033403

Urbanska K, 2015, NANOSCALE RES LETT, V10, P1, DOI 10.1186/s11671-015-0823-5

Ustymowicz K, 2009, ANTICANCER RES, V29, P3049

Walid Mohammad Sami, 2008, Perm J, V12, P45

Wen PY, 2008, NEW ENGL J MED, V359, P492, DOI 10.1056/NEJMra0708126

Yan T, 2011, BMC CANCER, V11, DOI 10.1186/1471-2407-11-524

Zhang X, 2012, EXP THER MED, V3, P9, DOI 10.3892/etm.2011.367

NR 35

TC 23

Z9 25

U1 0

U2 18

PU PUBLIC LIBRARY SCIENCE

PI SAN FRANCISCO

PA 1160 BATTERY STREET, STE 100, SAN FRANCISCO, CA 94111 USA

SN 1932-6203

J9 PLOS ONE

JI PLoS One

PD MAY 31

PY 2017

VL 12

IS 5

AR e0178277

DI 10.1371/journal.pone.0178277

PG 14

WC Multidisciplinary Sciences

WE Science Citation Index Expanded (SCI-EXPANDED)

SC Science & Technology - Other Topics

GA EW6GY

UT WOS:000402608700050

PM 28562655

OA gold, Green Published, Green Submitted

DA 2025-04-09

ER

PT J

AU Richard, SA

Sackey, M

Kortei, NK

AF Richard, Seidu A.

Sackey, Marian

Kortei, Nii Korley

TI Exploring the Pivotal Neurophysiologic and Therapeutic Potentials of

Vitamin C in Glioma

SO JOURNAL OF ONCOLOGY

LA English

DT Review

ID BLOOD-BRAIN-BARRIER; GLIOBLASTOMA-MULTIFORME CELLS; N-ACETYLTRANSFERASE

ACTIVITY; GLUTATHIONE-S-TRANSFERASES; GROWTH-FACTOR-I; ASCORBIC-ACID;

NITRIC-OXIDE; PHARMACOLOGICAL ASCORBATE; HYDROGEN-PEROXIDE; CANCER-CELLS

AB Gliomas represent solely primary brain cancers of glial cell or neuroepithelial origin. Gliomas are still the most lethal human cancers despite modern innovations in both diagnostic techniques as well as therapeutic regimes. Gliomas have the lowest overall survival rate compared to other cancers 5 years after definitive diagnosis. The dietary intake of vitamin C has protective effect on glioma risk. Vitamin C is an essential compound that plays a vital role in the regulation of lysyl and prolyl hydroxylase activity. Neurons store high levels of vitamin C via sodium dependent-vitamin C transporters (SVCTs) to protect them from oxidative ischemia-reperfusion injury. Vitamin C is a water-soluble enzyme, typically seen as a powerful antioxidant in plants as well as animals. The key function of vitamin C is the inhibition of redox imbalance from reactive oxygen species produced via the stimulation of glutamate receptors. Gliomas absorb vitamin C primarily via its oxidized dehydroascorbate form by means of GLUT 1, 3, and 4 and its reduced form, ascorbate, by SVCT2. Vitamin C is able to preserve prosthetic metal ions like Fe2+ and Cu+ in their reduced forms in several enzymatic reactions as well as scavenge free radicals in order to safeguard tissues from oxidative damage. Therapeutic concentrations of vitamin C are able to trigger H2O2 generation in glioma. High-dose combination of vitamin C and radiation has a much more profound cytotoxic effect on primary glioblastoma multiforme cells compared to normal astrocytes. Control trials are needed to validate the use of vitamin C and standardization of the doses of vitamin C in the treatment of patients with glioma.

C1 [Richard, Seidu A.] Princefield Univ, Dept Med, POB MA-128, Ho, Ghana.

[Sackey, Marian] Ho Teaching Hosp, Dept Pharm, POB MA-374, Ho, Ghana.

[Kortei, Nii Korley] Univ Hlth & Allied Sci, Sch Allied Hlth Sci, Dept Nutr & Dietet, Ho, Ghana.

RP Richard, SA (corresponding author), Princefield Univ, Dept Med, POB MA-128, Ho, Ghana.

EM gbepoo@gmail.com; sackeymarian60@gmail.com; nkkortei@uhas.edu.gh

RI Kortei, Nii/X-2537-2019; A. Richard, Seidu/H-7590-2017

OI Kortei, Nii Korley/0000-0002-8863-4694; A. Richard,

Seidu/0000-0003-3475-0363

CR [Anonymous], 2006, J. Carcinog., DOI 10.1186/1477-3163-5-14

Atlante A, 1997, J NEUROCHEM, V68, P2038, DOI 10.1046/j.1471-4159.1997.68052038.x

Bagley RG, 2003, CANCER RES, V63, P5866

BANKS WA, 1985, BRAIN RES BULL, V15, P287, DOI 10.1016/0361-9230(85)90153-4

Begley DJ, 1996, J PHARM PHARMACOL, V48, P136, DOI 10.1111/j.2042-7158.1996.tb07112.x

Beltrán FA, 2011, J CELL PHYSIOL, V226, P3286, DOI 10.1002/jcp.22674

BENADE L, 1969, ONCOLOGY-BASEL, V23, P33, DOI 10.1159/000224465

Bi DB, 2015, MED SCI MONITOR, V21, P2421, DOI 10.12659/MSM.893745

Bijur GN, 1999, ENVIRON MOL MUTAGEN, V33, P144, DOI 10.1002/(SICI)1098-2280(1999)33:2<144::AID-EM6>3.0.CO;2-V

Blowers L, 1997, CANCER CAUSE CONTROL, V8, P5, DOI 10.1023/A:1018437031987

BOEING H, 1993, INT J CANCER, V53, P561, DOI 10.1002/ijc.2910530406

Bompais H, 2004, BLOOD, V103, P2577, DOI 10.1182/blood-2003-08-2770

BOUTELLE MG, 1989, NEUROSCIENCE, V30, P11, DOI 10.1016/0306-4522(89)90349-7

Brada M, 2006, ANN ONCOL, V17, pX183, DOI 10.1093/annonc/mdl257

BUNIN GR, 1993, NEW ENGL J MED, V329, P536, DOI 10.1056/NEJM199308193290804

BUNIN GR, 1994, CANCER CAUSE CONTROL, V5, P177, DOI 10.1007/BF01830264

CAMERON E, 1974, CHEM-BIOL INTERACT, V9, P273, DOI 10.1016/0009-2797(74)90018-0

CAMERON E, 1976, P NATL ACAD SCI USA, V73, P3685, DOI 10.1073/pnas.73.10.3685

CAMERON E, 1974, CHEM-BIOL INTERACT, V9, P285, DOI 10.1016/0009-2797(74)90019-2

Castro M, 2001, J NEUROCHEM, V78, P815, DOI 10.1046/j.1471-4159.2001.00461.x

Castro ML, 2014, FREE RADICAL BIO MED, V74, P200, DOI 10.1016/j.freeradbiomed.2014.06.022

Castro MA, 2008, PFLUG ARCH EUR J PHY, V457, P519, DOI 10.1007/s00424-008-0526-1

Chen Q, 2005, P NATL ACAD SCI USA, V102, P13604, DOI 10.1073/pnas.0506390102

Chen Q, 2007, P NATL ACAD SCI USA, V104, P8749, DOI 10.1073/pnas.0702854104

Ciani E, 1996, BRAIN RES, V728, P1

Conklin Kenneth A, 2004, Integr Cancer Ther, V3, P294, DOI 10.1177/1534735404270335

COYLE JT, 1993, SCIENCE, V262, P689, DOI 10.1126/science.7901908

Cuddihy SL, 2008, FREE RADICAL BIO MED, V44, P1637, DOI 10.1016/j.freeradbiomed.2008.01.021

Daruwala R, 1999, FEBS LETT, V460, P480, DOI 10.1016/S0014-5793(99)01393-9

DILIBERTO EJ, 1987, ANN NY ACAD SCI, V498, P28, DOI 10.1111/j.1749-6632.1987.tb23749.x

Doskey CM, 2016, REDOX BIOL, V10, P274, DOI 10.1016/j.redox.2016.10.010

Du J, 2012, BBA-REV CANCER, V1826, P443, DOI 10.1016/j.bbcan.2012.06.003

Duan L, 2017, J EVID-BASED INTEGR, V22, P667, DOI 10.1177/2156587217708524

Duda DG, 2004, TRENDS MOL MED, V10, P143, DOI 10.1016/j.molmed.2004.02.001

Dulak J, 2000, ARTERIOSCL THROM VAS, V20, P659, DOI 10.1161/01.ATV.20.3.659

DYKENS JA, 1987, J NEUROCHEM, V49, P1222, DOI 10.1111/j.1471-4159.1987.tb10014.x

Ebert BL, 2003, ONCOLOGIST, V8, P69, DOI 10.1634/theoncologist.8-1-69

ELDRIDGE CF, 1987, J CELL BIOL, V105, P1023, DOI 10.1083/jcb.105.2.1023

Espey MG, 2011, FREE RADICAL BIO MED, V50, P1610, DOI 10.1016/j.freeradbiomed.2011.03.007

Flashman E, 2010, BIOCHEM J, V427, P135, DOI 10.1042/BJ20091609

Gallo O, 1998, J NATL CANCER I, V90, P587, DOI 10.1093/jnci/90.8.587

Garcia CG, 2018, MOL NEUROBIOL, V55, P6816, DOI 10.1007/s12035-018-0895-1

GILES GG, 1994, INT J CANCER, V59, P357, DOI 10.1002/ijc.2910590311

Gilloteaux J, 1998, SCANNING, V20, P564

Gilloteaux J, 2010, ULTRASTRUCT PATHOL, V34, P140, DOI 10.3109/01913121003662304

GLEMBOTSKI CC, 1987, ANN NY ACAD SCI, V498, P54, DOI 10.1111/j.1749-6632.1987.tb23750.x

Gokturk D, 2018, TURK NEUROSURG, V28, P13, DOI 10.5137/1019-5149.JTN.19111-16.1

Gratton JP, 2003, CANCER CELL, V4, P31, DOI 10.1016/S1535-6108(03)00168-5

GRUNEWALD RA, 1993, BRAIN RES REV, V18, P123, DOI 10.1016/0165-0173(93)90010-W

Hammarstrom L., 1967, Acta Physiologica Scandinavica, V70

Han SS, 2004, J CELL BIOCHEM, V93, P257, DOI 10.1002/jcb.20116

Herst PM, 2012, FREE RADICAL BIO MED, V52, P1486, DOI 10.1016/j.freeradbiomed.2012.01.021

HOLME JA, 1984, CELL BIOL TOXICOL, V1, P95, DOI 10.1007/BF00125568

Holotiuk V. V., 2019, Experimental Oncology, V41, P210

HORNIG D, 1975, ANN NY ACAD SCI, V258, P103, DOI 10.1111/j.1749-6632.1975.tb29271.x

Hu JF, 1999, INT J CANCER, V81, P20, DOI 10.1002/(SICI)1097-0215(19990331)81:1<20::AID-IJC4>3.0.CO;2-2

Hung CF, 2000, NEUROCHEM RES, V25, P845, DOI 10.1023/A:1007573609158

Hung CF, 2001, NEUROCHEM RES, V26, P1107, DOI 10.1023/A:1012314705007

JACOB HS, 1968, BLOOD, V32, P549, DOI 10.1182/blood.V32.4.549.549

Jadeski LC, 1999, AM J PATHOL, V155, P1381, DOI 10.1016/S0002-9440(10)65240-6

JENKINS DC, 1995, P NATL ACAD SCI USA, V92, P4392, DOI 10.1073/pnas.92.10.4392

Kaplan S, 1997, AM J EPIDEMIOL, V146, P832, DOI 10.1093/oxfordjournals.aje.a009201

Korones DN, 2003, CANCER, V97, P1963, DOI 10.1002/cncr.11260

Kume-Kick J, 1998, BRAIN RES, V803, P105, DOI 10.1016/S0006-8993(98)00628-3

KUO CH, 1978, JPN J PHARMACOL, V28, P789, DOI 10.1254/jjp.28.789

LAM DKC, 1986, Q J EXP PHYSIOL CMS, V71, P483, DOI 10.1113/expphysiol.1986.sp003007

LASZKIEWICZ I, 1992, METAB BRAIN DIS, V7, P157, DOI 10.1007/BF01000161

Lawenda BD, 2008, JNCI-J NATL CANCER I, V100, P773, DOI 10.1093/jnci/djn148

Lee M, 1997, CANCER CAUSE CONTROL, V8, P13, DOI 10.1023/A:1018470802969

Lee PC, 1999, AM J PHYSIOL-HEART C, V277, pH1600, DOI 10.1152/ajpheart.1999.277.4.H1600

Levine B, 2007, NATURE, V446, P745, DOI 10.1038/446745a

Lin SS, 2000, NEUROCHEM RES, V25, P1503, DOI 10.1023/A:1007632326953

Liu MG, 2006, CANCER RES, V66, P3593, DOI 10.1158/0008-5472.CAN-05-2912

Makino Y, 1999, ANTICANCER RES, V19, P3125

Mandl J, 2009, BRIT J PHARMACOL, V157, P1097, DOI 10.1111/j.1476-5381.2009.00282.x

Mardones L, 2011, BIOCHEM BIOPH RES CO, V410, P7, DOI 10.1016/j.bbrc.2011.05.070

Mikirova N, 2016, AM J CASE REP, V17, P774, DOI 10.12659/AJCR.899754

Mikirova NA, 2008, J TRANSL MED, V6, DOI 10.1186/1479-5876-6-50

MILBY K, 1982, NEUROSCI LETT, V28, P15, DOI 10.1016/0304-3940(82)90201-4

MILLER BT, 1987, BRAIN RES BULL, V19, P95, DOI 10.1016/0361-9230(87)90171-7

MILLER EC, 1949, CANCER RES, V9, P504

MITRA A, 1991, TOXICOL LETT, V58, P135, DOI 10.1016/0378-4274(91)90167-5

MITRA A, 1992, TOXICOL LETT, V60, P281, DOI 10.1016/0378-4274(92)90286-S

NAIDU AK, 1993, J NEURO-ONCOL, V16, P1, DOI 10.1007/BF01324828

NAIDU AK, 1993, ANTICANCER RES, V13, P1469

Naidu KA, 2001, J NEURO-ONCOL, V54, P15, DOI 10.1023/A:1012545311054

NEWELL SD, 1981, SURG NEUROL, V16, P161, DOI 10.1016/0090-3019(81)90125-7

Nualart Francisco, 2014, J Stem Cell Res Ther, V4, P209

ONEILL RD, 1984, NEUROSCI LETT, V52, P227, DOI 10.1016/0304-3940(84)90166-6

ORR CWM, 1967, BIOCHEMISTRY-US, V6, P2995, DOI 10.1021/bi00862a004

OWENS GC, 1989, GLIA, V2, P119, DOI 10.1002/glia.440020208

Pardridge WM, 1999, J NEUROVIROL, V5, P556, DOI 10.3109/13550289909021285

PARDRIDGE WM, 1988, ANNU REV PHARMACOL, V28, P25

Park S, 2013, NUTRIENTS, V5, P3496, DOI 10.3390/nu5093496

Payton NM, 2014, J PHARM SCI-US, V103, P3869, DOI 10.1002/jps.24171

Peichev M, 2000, BLOOD, V95, P952, DOI 10.1182/blood.V95.3.952.003k27_952_958

PETERKOFSKY B, 1977, J CELL PHYSIOL, V90, P61, DOI 10.1002/jcp.1040900109

Peyman GA, 2007, GRAEF ARCH CLIN EXP, V245, P1461, DOI 10.1007/s00417-007-0542-4

Portugal CC, 2009, J NEUROCHEM, V108, P507, DOI 10.1111/j.1471-4159.2008.05786.x

Pouliquen D, 2008, INT J CANCER, V123, P288, DOI 10.1002/ijc.23513

PRASAD KN, 1979, P NATL ACAD SCI USA, V76, P829, DOI 10.1073/pnas.76.2.829

PRASAD KN, 1980, LIFE SCI, V27, P275, DOI 10.1016/0024-3205(80)90194-0

REBEC GV, 1994, PROG NEUROBIOL, V43, P537, DOI 10.1016/0301-0082(94)90052-3

REIBER H, 1993, CLIN CHIM ACTA, V217, P163, DOI 10.1016/0009-8981(93)90162-W

Rice ME, 2000, TRENDS NEUROSCI, V23, P209, DOI 10.1016/S0166-2236(99)01543-X

RICE ME, 1995, J NEUROCHEM, V64, P1790

Rice ME, 1998, NEUROSCIENCE, V82, P1213

Richard S.A, 2019, NEUROQUANTOLOGY, V17, DOI [10.14704/nq.2019.17.6.2227, DOI 10.14704/NQ.2019.17.6.2227]

RIORDAN NH, 1995, MED HYPOTHESES, V44, P207, DOI 10.1016/0306-9877(95)90137-X

ROSE RC, 1993, FASEB J, V7, P1135, DOI 10.1096/fasebj.7.12.8375611

Rumsey SC, 1997, J BIOL CHEM, V272, P18982, DOI 10.1074/jbc.272.30.18982

Ryszawy D, 2019, LIFE SCI, V232, DOI 10.1016/j.lfs.2019.116657

Salmaso S, 2009, BIOCONJUGATE CHEM, V20, P2348, DOI 10.1021/bc900369d

SANDBERGNORDQVIST AC, 1993, CANCER RES, V53, P2475

Schoenfeld JD, 2017, CANCER CELL, V31, P487, DOI 10.1016/j.ccell.2017.02.018

Schofield CJ, 2004, NAT REV MOL CELL BIO, V5, P343, DOI 10.1038/nrm1366

Schwartzbaum JA, 2000, NUTR CANCER, V38, P40, DOI 10.1207/S15327914NC381_7

SHARMA SK, 1982, BIOCHEM J, V208, P43, DOI 10.1042/bj2080043

Siegel R, 2013, CA-CANCER J CLIN, V63, P11, DOI 10.3322/caac.21166

SMOLEN JE, 1974, J LIPID RES, V15, P273

Solis-Nolasco I.M., 2020, GLOBAL ADV HLTH MED, V9

Sorice A, 2014, MINI-REV MED CHEM, V14, P444, DOI 10.2174/1389557514666140428112602

SPECTOR R, 1977, NEW ENGL J MED, V296, P1393, DOI 10.1056/NEJM197706162962409

SPECTOR R, 1973, AM J PHYSIOL, V225, P757, DOI 10.1152/ajplegacy.1973.225.4.757

Spector R, 2000, PHARMACOLOGY, V60, P58, DOI 10.1159/000028349

Stegh AH, 2008, CELL CYCLE, V7, P2833, DOI 10.4161/cc.7.18.6759

Surawicz TS, 1998, J NEURO-ONCOL, V40, P151, DOI 10.1023/A:1006091608586

Telang S, 2007, NEOPLASIA, V9, P47, DOI 10.1593/neo.06664

Tsukaguchi H, 1999, NATURE, V399, P70, DOI 10.1038/19986

Vissers MCM, 2007, J LEUKOCYTE BIOL, V81, P1236, DOI 10.1189/jlb.0806541

Vissers MCM, 2007, FREE RADICAL BIO MED, V42, P765, DOI 10.1016/j.freeradbiomed.2006.11.023

Vita MF, 2011, INVEST NEW DRUG, V29, P1314, DOI 10.1007/s10637-010-9489-0

WALKER MD, 1978, J NEUROSURG, V49, P333, DOI 10.3171/jns.1978.49.3.0333

Wang YX, 2000, BIOCHEM BIOPH RES CO, V267, P488, DOI 10.1006/bbrc.1999.1929

WILSON CWM, 1975, ANN NY ACAD SCI, V258, P355, DOI 10.1111/j.1749-6632.1975.tb29295.x

Yao KC, 2003, J NEUROSURG, V98, P378, DOI 10.3171/jns.2003.98.2.0378

Zhong H, 1999, CANCER RES, V59, P5830

Zhou SR, 2015, NEUROEPIDEMIOLOGY, V44, P39, DOI 10.1159/000369814

Zhu JH, 2014, ONCOL REP, V32, P443, DOI 10.3892/or.2014.3259

Zumkeller W, 1996, INT J ONCOL, V9, P983

NR 140

TC 1

Z9 1

U1 0

U2 5

PU HINDAWI LTD

PI LONDON

PA ADAM HOUSE, 3RD FLR, 1 FITZROY SQ, LONDON, W1T 5HF, ENGLAND

SN 1687-8450

EI 1687-8469

J9 J ONCOL

JI J. Oncol.

PD DEC 14

PY 2021

VL 2021

AR 6141591

DI 10.1155/2021/6141591

PG 12

WC Oncology

WE Science Citation Index Expanded (SCI-EXPANDED)

SC Oncology

GA YB3LW

UT WOS:000738919200005

OA Green Published, gold

DA 2025-04-09

ER

PT J

AU Pekmez, M

Önay-Uçar, E

Arda, N

AF Pekmez, Murat

Onay-Ucar, Evren

Arda, Nazli

TI Effect of α-tocopheryl succinate on the molecular damage induced by

indomethacin in C6 glioma cells

SO EXPERIMENTAL AND THERAPEUTIC MEDICINE

LA English

DT Article

DE alpha-tocopheryl succinate; indomethacin; C6 Glioma; intracellular ROS

level; protein carbonyls

ID NONSTEROIDAL ANTIINFLAMMATORY DRUGS; VITAMIN-E; CYCLOOXYGENASE-2

EXPRESSION; PROGNOSTIC-SIGNIFICANCE; GENE-EXPRESSION; IN-VIVO; GROWTH;

CANCER; INHIBITION; ASPIRIN

AB Indomethacin is a member of the non-steroidal anti-inflammatory drug (NSAID) class, which has great potential for use in the treatment of glioma. However, it induces the generation of reactive oxygen species (ROS) and causes molecular damage while inducing its effects. Vitamin E is widely used in the complementary therapy of cancers. The main goal of the present study was to investigate the effects of alpha-tocopheryl succinate (alpha-TOS) against the oxidative damage induced by indomethacin in C6 glioma cells. Cells were treated with 10 piM alpha-TOS alone or in combination with 200 piM indomethacin for two days. The intracellular ROS level, molecular damage as revealed by lipid peroxidation and protein carbonyl formation, and the COX activity in C6 glioma cells were measured. Treatment of the cells with alpha-TOS and indomethacin, alone or in combination, caused the levels of ROS generation and protein damage to increase, but protected against lipid peroxidation and reduced COX activity.

C1 [Pekmez, Murat; Onay-Ucar, Evren; Arda, Nazli] Istanbul Univ, Fac Sci, Dept Mol Biol & Genet, TR-34134 Istanbul, Turkey.

C3 Istanbul University

RP Pekmez, M (corresponding author), Istanbul Univ, Fac Sci, Dept Mol Biol & Genet, Sehzadebasi St, TR-34134 Istanbul, Turkey.

EM mpekmez@istanbul.edu.tr

RI ARDA, NAZLI/A-9033-2018; UÇAR, Evren/A-8948-2018; Pekmez,

Murat/AAD-9065-2020

OI Arda, Nazli/0000-0002-1043-5652

FU Istanbul University Research Foundation [4120, 2675]

FX This study was supported by the Istanbul University Research Foundation

(Project No: 4120 and Project No: 2675).

CR Amin R, 2003, NEUROL RES, V25, P370, DOI 10.1179/016164103101201706

Azzi A, 2003, MOL ASPECTS MED, V24, P325, DOI 10.1016/S0098-2997(03)00028-1

Baek SJ, 2002, J PHARMACOL EXP THER, V301, P1126, DOI 10.1124/jpet.301.3.1126

Bernardi A, 2006, EUR J PHARMACOL, V532, P214, DOI 10.1016/j.ejphar.2006.01.008

Block KI, 2007, CANCER TREAT REV, V33, P407, DOI 10.1016/j.ctrv.2007.01.005

Conklin KA, 2000, NUTR CANCER, V37, P1, DOI 10.1207/S15327914NC3701_1

Constantinou C, 2008, INT J CANCER, V123, P739, DOI 10.1002/ijc.23689

Courade JP, 2001, LIFE SCI, V69, P1455, DOI 10.1016/S0024-3205(01)01228-0

Cuzick J, 2009, LANCET ONCOL, V10, P501, DOI 10.1016/S1470-2045(09)70035-X

DRAPER HH, 1990, METHOD ENZYMOL, V186, P421

Drisko JA, 2003, GYNECOL ONCOL, V88, P434, DOI 10.1016/S0090-8258(02)00067-7

FLOWER RJ, 1973, PROSTAGLANDINS, V4, P325, DOI 10.1016/0090-6980(73)90020-8

Gravel P., 2002, The Protein Protocols Handbook, P321

Grubbs CJ, 2000, CANCER RES, V60, P5599

Gupta RA, 2000, P NATL ACAD SCI USA, V97, P13275, DOI 10.1073/pnas.97.24.13275

Holt S, 2003, N-S ARCH PHARMACOL, V367, P237, DOI 10.1007/s00210-002-0686-z

Hrkal Z, 2003, Sb Lek, V104, P217

Ishibashi M, 2005, EXP CELL RES, V302, P244, DOI 10.1016/j.yexcr.2004.09.021

Kürzel F, 2002, ACTA NEUROCHIR, V144, P71, DOI 10.1007/s701-002-8276-9

Lamson D W, 2000, Altern Med Rev, V5, P152

Lee BS, 2010, BIOORG MED CHEM LETT, V20, P5262, DOI 10.1016/j.bmcl.2010.06.144

Mackerras D, 1999, BRIT J CANCER, V79, P1448, DOI 10.1038/sj.bjc.6690231

Maity P, 2009, J BIOL CHEM, V284, P3058, DOI 10.1074/jbc.M805329200

MOSMANN T, 1983, J IMMUNOL METHODS, V65, P55, DOI 10.1016/0022-1759(83)90303-4

Naito Y, 2006, REDOX REP, V11, P243, DOI 10.1179/135100006X155021

Nathoo N, 2004, J CLIN PATHOL, V57, P6, DOI 10.1136/jcp.57.1.6

Negre-Salvayre A, 2002, METHOD ENZYMOL, V352, P62

New Pamela, 2004, Cancer Control, V11, P152

OCALLAGHAN JP, 1988, NEUROTOXICOL TERATOL, V10, P445, DOI 10.1016/0892-0362(88)90006-2

Ottino P, 1997, NUTR RES, V17, P661, DOI 10.1016/S0271-5317(97)00036-5

RAMA BN, 1983, P SOC EXP BIOL MED, V174, P302

Rao PNP, 2008, J PHARM PHARM SCI, V11, P81, DOI 10.18433/J3T886

Ribeiro G, 2008, POLYHEDRON, V27, P1131, DOI 10.1016/j.poly.2007.12.011

Ristimäki A, 2002, CANCER RES, V62, P632

Salganik RI, 2000, CARCINOGENESIS, V21, P909, DOI 10.1093/carcin/21.5.909

Shiff SJ, 1996, EXP CELL RES, V222, P179, DOI 10.1006/excr.1996.0023

Shiff SJ, 2003, CURR OPIN PHARMACOL, V3, P352, DOI 10.1016/S1471-4892(03)00087-0

Shono T, 2001, CANCER RES, V61, P4375

Sivak-Sears NR, 2004, AM J EPIDEMIOL, V159, P1131, DOI 10.1093/aje/kwh153

Smith B.J., 2002, PROTEIN PROTOCOLS HD, V2nd, P237

Smith ML, 2000, EUR J CANCER, V36, P664, DOI 10.1016/S0959-8049(99)00333-0

Stapelberg M, 2005, J BIOL CHEM, V280, P25369, DOI 10.1074/jbc.M414498200

STOSCHECK CM, 1990, METHOD ENZYMOL, V182, P50

TEICHER BA, 1994, CANCER CHEMOTH PHARM, V34, P235

Wang MD, 2005, J NEURO-ONCOL, V72, P1, DOI 10.1007/s11060-004-1392-0

Weijl NI, 1997, CANCER TREAT REV, V23, P209, DOI 10.1016/S0305-7372(97)90012-8

Williams CS, 2000, CANCER RES, V60, P6045

Yasumaru M, 2003, CANCER RES, V63, P6726

NR 48

TC 5

Z9 5

U1 0

U2 5

PU SPANDIDOS PUBL LTD

PI ATHENS

PA POB 18179, ATHENS, 116 10, GREECE

SN 1792-0981

EI 1792-1015

J9 EXP THER MED

JI Exp. Ther. Med.

PD FEB

PY 2015

VL 9

IS 2

BP 585

EP 590

DI 10.3892/etm.2014.2101

PG 6

WC Medicine, Research & Experimental

WE Science Citation Index Expanded (SCI-EXPANDED)

SC Research & Experimental Medicine

GA CB3CX

UT WOS:000349506900053

PM 25574239

OA Green Submitted, Green Published, gold

DA 2025-04-09

ER

PT J

AU Barciszewska, AM

Belter, A

Gawronska, I

Giel-Pietraszuk, M

Naskret-Barciszewska, MZ

AF Barciszewska, Anna-Maria

Belter, Agnieszka

Gawronska, Iwona

Giel-Pietraszuk, Malgorzata

Naskret-Barciszewska, Miroslawa Z.

TI Cross-reactivity between histone demethylase inhibitor valproic acid and

DNA methylation in glioblastoma cell lines

SO FRONTIERS IN ONCOLOGY

LA English

DT Article

DE valproic acid; temozolomide; glioblastoma; DNA methylation;

8-oxo-deoxyguanosine

ID ANTIEPILEPTIC DRUGS; BRAIN-TUMORS; SURVIVAL; TEMOZOLOMIDE;

DIFFERENTIATION; HYPOMETHYLATION; MULTIFORME; RESISTANCE; EPIGENOME;

EORTC

AB Currently, valproic acid (VPA) is known as an inhibitor of histone deacetylase (epigenetic drug) and is used for the clinical treatment of epileptic events in the course of glioblastoma multiforme (GBM). Which improves the clinical outcome of those patients. We analyzed the level of 5-methylcytosine, a DNA epigenetic modulator, and 8-oxodeoxyguanosine, an cellular oxidative damage marker, affected with VPA administration, alone and in combination with temozolomide (TMZ), of glioma (T98G, U118, U138), other cancer (HeLa), and normal (HaCaT) cell lines. We observed the VPA dose-dependent changes in the total DNA methylation in neoplastic cell lines and the lack of such an effect in a normal cell line. VPA at high concentrations (250-500 mu M) induced hypermethylation of DNA in a short time frame. However, the exposition of GBM cells to the combination of VPA and TMZ resulted in DNA hypomethylation. At the same time, we observed an increase of genomic 8-oxo-dG, which as a hydroxyl radical reaction product with guanosine residue in DNA suggests a red-ox imbalance in the cancer cells and radical damage of DNA. Our data show that VPA as an HDAC inhibitor does not induce changes only in histone acetylation, but also changes in the state of DNA modification. It shows cross-reactivity between chromatin remodeling due to histone acetylation and DNA methylation. Finally, total DNA cytosine methylation and guanosine oxidation changes in glioma cell lines under VPA treatment suggest a new epigenetic mechanism of that drug action.

C1 [Barciszewska, Anna-Maria] Poznan Univ Med Sci, Chair & Dept Neurosurg & Neurotraumatol, Intraoperat Imaging Unit, Poznan, Poland.

[Barciszewska, Anna-Maria] Heliodor Swiecicki Clin Hosp, Dept Neurosurg & Neurotraumatol, Poznan, Poland.

[Belter, Agnieszka; Gawronska, Iwona; Giel-Pietraszuk, Malgorzata; Naskret-Barciszewska, Miroslawa Z.] Polish Acad Sci, Inst Bioorgan Chem, Poznan, Poland.

C3 Poznan University of Medical Sciences; Polish Academy of Sciences;

Institute of Bioorganic Chemistry of the Polish Academy of Sciences

RP Naskret-Barciszewska, MZ (corresponding author), Polish Acad Sci, Inst Bioorgan Chem, Poznan, Poland.

EM mbarcisz@ibch.poznan.pl

RI Belter, Agnieszka/W-6074-2018; Barciszewska, Anna-Maria/J-5978-2018

FU National Science Center Poland; [2020/37/B/NZ5/03249]

FX Funding This work was supported with funding from the National Science

Center Poland (grant nr.: 2020/37/B/NZ5/03249) to MZN-B.

CR Aggarwal V, 2019, BIOMOLECULES, V9, DOI 10.3390/biom9110735

Barciszewska AM, 2019, CELLS-BASEL, V8, DOI 10.3390/cells8091065

Barciszewska AM, 2018, BIOSCIENCE REP, V38, DOI 10.1042/BSR20180731

Barciszewska AM, 2015, PLOS ONE, V10, DOI 10.1371/journal.pone.0136669

Barciszewska AM, 2014, PLOS ONE, V9, DOI 10.1371/journal.pone.0092599

Barker CA, 2013, INT J RADIAT ONCOL, V86, P504, DOI 10.1016/j.ijrobp.2013.02.012

Baylin SB, 2011, NAT REV CANCER, V11, P726, DOI 10.1038/nrc3130

Belter A, 2020, PLOS ONE, V15, DOI 10.1371/journal.pone.0229534

Berendsen S, 2012, EXPERT OPIN INV DRUG, V21, P1391, DOI 10.1517/13543784.2012.694425

Chen JC, 2019, BMC CANCER, V19, DOI 10.1186/s12885-019-5843-6

Chen Y, 2012, J NEURO-ONCOL, V109, P23, DOI 10.1007/s11060-012-0871-y

Chou T., 2005, CompuSyn for drug combinations: PC software and users guide: a computer program for quantitation of synergism and antagonism in drug combinations, and the determination of IC50 and ED50 and LD50 values

CHOU TC, 1984, ADV ENZYME REGUL, V22, P27, DOI 10.1016/0065-2571(84)90007-4

Duenas-Gonzalez A, 2008, CANCER TREAT REV, V34, P206, DOI 10.1016/j.ctrv.2007.11.003

Eckert M, 2017, CELL PHYSIOL BIOCHEM, V44, P1591, DOI 10.1159/000485753

Eckschlager T, 2017, INT J MOL SCI, V18, DOI 10.3390/ijms18071414

Ehrlich M, 2013, EPIGENOMICS-UK, V5, P553, DOI 10.2217/epi.13.43

Ehrlich M, 2013, ADV EXP MED BIOL, V754, P31, DOI 10.1007/978-1-4419-9967-2_2

Englot Dario J, 2016, Handb Clin Neurol, V134, P267, DOI 10.1016/B978-0-12-802997-8.00016-5

Feil R, 2012, NAT REV GENET, V13, P97, DOI 10.1038/nrg3142

Göttlicher M, 2001, EMBO J, V20, P6969, DOI 10.1093/emboj/20.24.6969

Guthrie GD, 2013, J NEUROSURG, V118, P859, DOI 10.3171/2012.10.JNS12169

Han W, 2021, FRONT ONCOL, V11, DOI 10.3389/fonc.2021.687362

Hontecillas-Prieto L, 2020, FRONT GENET, V11, DOI 10.3389/fgene.2020.578011

Jeltsch A, 2016, NUCLEIC ACIDS RES, V44, P8556, DOI 10.1093/nar/gkw723

Johannessen CU, 2003, CNS DRUG REV, V9, P199

Kong DS, 2010, NEURO-ONCOLOGY, V12, P289, DOI 10.1093/neuonc/nop030

Kumar S, 2018, FRONT GENET, V9, DOI 10.3389/fgene.2018.00640

Löscher W, 1999, PROG NEUROBIOL, V58, P31, DOI 10.1016/S0301-0082(98)00075-6

Mahmood N, 2019, ADV EXP MED BIOL, V1164, P179, DOI 10.1007/978-3-030-22254-3_14

Mahmood N, 2018, ONCOTARGET, V9, P5169, DOI 10.18632/oncotarget.23704

Martin EM, 2018, ANNU REV PUBL HEALTH, V39, P309, DOI 10.1146/annurev-publhealth-040617-014629

Mehdipour P, 2020, PHARMACOL THERAPEUT, V205, DOI 10.1016/j.pharmthera.2019.107416

Michalak M, 2013, PLOS ONE, V8, DOI 10.1371/journal.pone.0070693

Milutinovic S, 2007, CARCINOGENESIS, V28, P560, DOI 10.1093/carcin/bgl167

Moore LD, 2013, NEUROPSYCHOPHARMACOL, V38, P23, DOI 10.1038/npp.2012.112

Nakada M, 2012, FRONT ONCOL, V2, DOI 10.3389/fonc.2012.00098

Nurk S, 2022, SCIENCE, V376, P44, DOI 10.1126/science.abj6987

NYCE J, 1989, CANCER RES, V49, P5829

Patsalos PN, 2017, EPILEPSIA, V58, P1234, DOI 10.1111/epi.13802

Riva G, 2016, ONCOL REP, V35, P2811, DOI 10.3892/or.2016.4665

Sarkar S, 2011, ANTICANCER RES, V31, P2723

Schritz A, 2021, NEURO-ONCOL ADV, V3, DOI 10.1093/noajnl/vdab052

SHEN DD, 1992, NEUROLOGY, V42, P582, DOI 10.1212/WNL.42.3.582

Silva MR, 2018, PHARMACOL BIOCHEM BE, V167, P17, DOI 10.1016/j.pbb.2018.02.001

Stupp R, 2009, LANCET ONCOL, V10, P459, DOI 10.1016/S1470-2045(09)70025-7

Tsai HC, 2021, FRONT ONCOL, V11, DOI 10.3389/fonc.2021.722754

Tsai HC, 2012, BRIT J NEUROSURG, V26, P347, DOI 10.3109/02688697.2011.638996

van den Bent MJ, 2011, CLIN CANCER RES, V17, P7148, DOI 10.1158/1078-0432.CCR-11-1274

Vecht CJ, 2014, ONCOLOGIST, V19, P751, DOI 10.1634/theoncologist.2014-0060

Veronezi GMB, 2017, PLOS ONE, V12, DOI 10.1371/journal.pone.0170740

Ververis K, 2013, BIOL-TARGETS THER, V7, P47, DOI 10.2147/BTT.S29965

Wang Y, 2017, ONCOTARGET, V8, P111866, DOI 10.18632/oncotarget.22942

WIESER HG, 1991, EPILEPSY RES, V9, P154, DOI 10.1016/0920-1211(91)90028-E

Yang HT, 2018, J EXP CLIN CANC RES, V37, DOI 10.1186/s13046-018-0909-x

Yang Y, 2014, SEIZURE-EUR J EPILEP, V23, P830, DOI 10.1016/j.seizure.2014.06.015

NR 56

TC 9

Z9 9

U1 0

U2 4

PU FRONTIERS MEDIA SA

PI LAUSANNE

PA AVENUE DU TRIBUNAL FEDERAL 34, LAUSANNE, CH-1015, SWITZERLAND

SN 2234-943X

J9 FRONT ONCOL

JI Front. Oncol.

PD NOV 16

PY 2022

VL 12

AR 1033035

DI 10.3389/fonc.2022.1033035

PG 13

WC Oncology

WE Science Citation Index Expanded (SCI-EXPANDED)

SC Oncology

GA 6Q4DF

UT WOS:000891563000001

PM 36465345

OA Green Published, gold

DA 2025-04-09

ER

PT J

AU Emsen, B

Aslan, A

Togar, B

Turkez, H

AF Emsen, Bugrahan

Aslan, Ali

Togar, Basak

Turkez, Hasan

TI In vitro antitumor activities of the lichen compounds olivetoric,

physodic and psoromic acid in rat neuron and glioblastoma cells

SO PHARMACEUTICAL BIOLOGY

LA English

DT Article

DE Cytotoxicity; genotoxicity; glioblastoma multiforme; oxidative status;

secondary metabolite

ID SECONDARY METABOLITES; HYPOGYMNIA-PHYSODES; CYTOTOXIC ACTIVITY; USNIC

ACID; ANTIMICROBIAL PROPERTIES; PSEUDEVERNIA-FURFURACEA; ANTIBACTERIAL

ACTIVITY; CHEMICAL-COMPOSITION; ANTICANCER ACTIVITY; EVERNIA-PRUNASTRI

AB Context Since methods utilised in the treatment of glioblastoma multiforme (GBM) are inadequate and have too many side effects, usage of herbal products in the treatment process comes into prominence. Lichens are symbiotic organisms used for medicinal purposes for many years. There are various anticancer treatments about components of two lichen species used in the present study.Objective Antitumor potential of three lichen secondary metabolites including olivetoric acid (OLA) and physodic acid (PHA) isolated from Pseudevernia furfuracea (L.) Zopf (Parmeliaceae) and psoromic acid (PSA) isolated from Rhizoplaca melanophthalma (DC.) Leuckert (Lecanoraceae) were investigated on human U87MG-GBM cell lines and primary rat cerebral cortex (PRCC) cells for the first time.Materials and methods PRCC cells used as healthy brain cells were obtained from Sprague-Dawley rats. The treatments were carried out on the cells cultured for 48h. Cytotoxic effects of different concentrations (2.5, 5, 10, 20 and 40mg/L) of metabolites on the cells were determined via 3-(4,5-dimethylthiazol-2-yl)-2,5-diphenyltetrazolium bromide (MTT) and lactate dehydrogenase (LDH) analyses. Total antioxidant capacity (TAC) and total oxidant status (TOS) parameters were used for assessing oxidative alterations. Oxidative DNA damage potentials of metabolites were investigated via evaluating 8-hydroxy-2-deoxyguanosine (8-OH-dG) levels.Results Median inhibitory concentration (IC50) values of OLA, PHA and PSA were 125.71, 698.19 and 79.40mg/L for PRCC cells and 17.55, 410.72 and 56.22mg/L for U87MG cells, respectively. It was revealed that cytotoxic effects of these metabolites showed positive correlation with concentration, LDH activity and oxidative DNA damage.Discussion and conclusion The present findings obtained in this study revealed that primarily OLA and then PSA had high potential for use in the treatment of GBM.

C1 [Emsen, Bugrahan] Karamanoglu Mehmetbey Univ, Dept Biol, Kamil Ozdag Fac Sci, TR-70100 Karaman, Turkey.

[Aslan, Ali] Ataturk Univ, Dept Biol Educ, Kazim Karabekir Fac Educ, Erzurum, Turkey.

[Togar, Basak] Ataturk Univ, Dept Biol, Fac Sci, Erzurum, Turkey.

[Turkez, Hasan] Erzurum Tech Univ, Dept Mol Biol & Genet, Fac Sci, Erzurum, Turkey.

C3 Karamanoglu Mehmetbey University; Ataturk University; Ataturk

University; Erzurum Technical University

RP Emsen, B (corresponding author), Karamanoglu Mehmetbey Univ, Dept Biol, Kamil Ozdag Fac Sci, TR-70100 Karaman, Turkey.

EM bugrahanemsen@gmail.com

RI aslan, ali/AAL-9580-2020; Emsen, Bugrahan/T-2000-2017; Türkez,

Hasan/AAQ-4905-2020

OI aslan, ali/0000-0002-5122-6646; Emsen, Bugrahan/0000-0002-9636-2596

FU Karamanoglu Mehmetbey University Scientific Research Projects Commission

[01-D-13]

FX There is no conflict of interest in any form between the authors. This

work was supported by Karamanoglu Mehmetbey University Scientific

Research Projects Commission with 01-D-13 project number.

CR Agar G, 2010, J MED PLANTS RES, V4, P2132

Alpsoy L, 2011, FRESEN ENVIRON BULL, V20, P1661

[Anonymous], 1971, CHEM LICHEN SUBSTANC

[Anonymous], PLANT GENET RESOUR

Aslan A, 2006, PHARM BIOL, V44, P247, DOI 10.1080/13880200600713808

Behera BC, 2012, PHARM BIOL, V50, P968, DOI 10.3109/13880209.2012.654396

Berridge MV, 2005, BIOTECHNOL ANN REV, V11, P127, DOI 10.1016/S1387-2656(05)11004-7

Bézivin C, 2003, PHYTOMEDICINE, V10, P499, DOI 10.1078/094471103322331458

Brodo IM, 2001, LICHENS LICHENS N AM

Celenza G, 2013, NAT PROD RES, V27, P1528, DOI 10.1080/14786419.2012.730043

Celikler Kasimogullari S, 2014, TURK J BIOL, V38, P940, DOI 10.3906/biy-1405-23

Cheng BK, 2013, PLOS ONE, V8, DOI 10.1371/journal.pone.0060770

Combs SE, 2005, STRAHLENTHER ONKOL, V181, P372, DOI 10.1007/s00066-005-1359-x

Correché ER, 2004, ATLA-ALTERN LAB ANIM, V32, P605, DOI 10.1177/026119290403200611

Culberson C.F., 1969, CHEM BOT GUIDE LICHE

Deng JY, 2009, CHEM RES TOXICOL, V22, P1817, DOI 10.1021/tx9002275

Dulger Basaran, 1998, Turkish Journal of Biology, V22, P111

Efdi M, 2007, BIOORGAN MED CHEM, V15, P3667, DOI 10.1016/j.bmc.2007.03.051

Efferth T, 2011, PHYTOMEDICINE, V18, P959, DOI 10.1016/j.phymed.2011.06.008

Erel O, 2004, CLIN BIOCHEM, V37, P277, DOI 10.1016/j.clinbiochem.2003.11.015

Erel O, 2005, CLIN BIOCHEM, V38, P1103, DOI 10.1016/j.clinbiochem.2005.08.008

Gan W, 2012, FREE RADICAL BIO MED, V52, P1700, DOI 10.1016/j.freeradbiomed.2012.02.016

Brandao LFG, 2013, CHEM PHARM BULL, V61, P176, DOI 10.1248/cpb.c12-00739

Gauden AJ, 2009, J CLIN NEUROSCI, V16, P1174, DOI 10.1016/j.jocn.2008.12.008

Ghate NB, 2013, PLOS ONE, V8, DOI 10.1371/journal.pone.0082293

Goldlust Samuel., 2008, Hospital Physician, P9

Grujicic D, 2014, CYTOTECHNOLOGY, V66, P803, DOI 10.1007/s10616-013-9629-4

Gülçin I, 2002, J ETHNOPHARMACOL, V79, P325, DOI 10.1016/S0378-8741(01)00396-8

Hahm SW, 2010, PLANT FOOD HUM NUTR, V65, P247, DOI 10.1007/s11130-010-0188-y

Halici M, 2005, PHYTOMEDICINE, V12, P656, DOI 10.1016/j.phymed.2004.06.021

Haslam G, 2000, CYTOTECHNOLOGY, V32, P63, DOI 10.1023/A:1008121125755

Jeong JC, 2011, PHYTOTHER RES, V25, P429, DOI 10.1002/ptr.3265

Jung HW, 2010, MOL MED REP, V3, P1041, DOI 10.3892/mmr.2010.376

Karagöz A, 2005, BIOLOGIA, V60, P281

Kim SY, 2008, BIOCHEM BIOPH RES CO, V366, P493, DOI 10.1016/j.bbrc.2007.11.178

Koparal AT, 2010, PHYTOTHER RES, V24, P754, DOI 10.1002/ptr.3035

Kosanic M, 2014, LWT-FOOD SCI TECHNOL, V59, P518, DOI 10.1016/j.lwt.2014.04.047

Kosanic M, 2013, FOOD CHEM TOXICOL, V53, P112, DOI 10.1016/j.fct.2012.11.034

Kosanic M, 2011, J MED FOOD, V14, P1624, DOI 10.1089/jmf.2010.0316

Kotan E, 2011, TOXICOL IND HEALTH, V27, P599, DOI 10.1177/0748233710394234

Lauinger IL, 2013, J NAT PROD, V76, P1064, DOI 10.1021/np400083k

Markiewicz-Zukowska R, 2013, J APIC SCI, V57, P147, DOI 10.2478/JAS-2013-0025

Markiewicz-Zukowska R, 2013, BMC COMPLEM ALTERN M, V13, DOI 10.1186/1472-6882-13-50

Mitrovic T, 2014, EXCLI J, V13, P938

Odabasoglu F, 2006, J ETHNOPHARMACOL, V103, P59, DOI 10.1016/j.jep.2005.06.043

OSAWA T, 1991, ENVIRON MOL MUTAGEN, V18, P35, DOI 10.1002/em.2850180107

Paudel B, 2012, BIOL RES, V45, P387, DOI 10.4067/S0716-97602012000400010

Pavlovic V, 2013, FOOD CHEM TOXICOL, V51, P160, DOI 10.1016/j.fct.2012.04.043

Purvis O.W., 1992, The Lichen Flora of Great Britain and Ireland

Rankovic B, 2014, MED CHEM RES, V23, P408, DOI 10.1007/s00044-013-0644-y

Russo A, 2010, CHEM-BIOL INTERACT, V184, P352, DOI 10.1016/j.cbi.2010.01.032

Russo A, 2006, ANTI-CANCER DRUG, V17, P1163, DOI 10.1097/01.cad.0000236310.66080.ed

Sepulveda B, 2013, J CHIL CHEM SOC, V58, P1750, DOI 10.4067/S0717-97072013000200024

Shen T, 2007, PHYTOCHEMISTRY, V68, P1331, DOI 10.1016/j.phytochem.2007.01.013

Singh N, 2013, NUTR CANCER, V65, P36, DOI 10.1080/01635581.2013.785007

Stojanovic IZ, 2014, FOLIA BIOL-PRAGUE, V60, P89

Süleyman H, 2003, PHYTOMEDICINE, V10, P552, DOI 10.1078/094471103322331539

Süleyman H, 2002, BIOL PHARM BULL, V25, P10, DOI 10.1248/bpb.25.10

Türk H, 2006, Z NATURFORSCH C, V61, P499

Urbanska K, 2014, WSPOLCZESNA ONKOL, V18, P307, DOI 10.5114/wo.2014.40559

Vartia K. O., 1973, LICHENS, P547

Wang SG, 2013, ACTA PHARMACOL SIN, V34, P1411, DOI 10.1038/aps.2013.98

[王小虎 Wang Xiaohu], 2010, [肿瘤, Tumor], V30, P1056

Wirth V., 1995, Die Flechten Baden-Wurttembergs-Teil 1 and 2

Wolterbeek HT, 2005, ASSAY DRUG DEV TECHN, V3, P675, DOI 10.1089/adt.2005.3.675

Yilmaz M, 2005, Z NATURFORSCH C, V60, P35

Zhang X, 2012, EXP THER MED, V3, P9, DOI 10.3892/etm.2011.367

NR 67

TC 51

Z9 53

U1 1

U2 39

PU TAYLOR & FRANCIS LTD

PI ABINGDON

PA 2-4 PARK SQUARE, MILTON PARK, ABINGDON OR14 4RN, OXON, ENGLAND

SN 1388-0209

EI 1744-5116

J9 PHARM BIOL

JI Pharm. Biol.

PD SEP

PY 2016

VL 54

IS 9

BP 1748

EP 1762

DI 10.3109/13880209.2015.1126620

PG 15

WC Plant Sciences; Medical Laboratory Technology; Pharmacology & Pharmacy

WE Science Citation Index Expanded (SCI-EXPANDED)

SC Plant Sciences; Medical Laboratory Technology; Pharmacology & Pharmacy

GA DU9XV

UT WOS:000382572400029

PM 26704132

OA hybrid, Green Submitted

DA 2025-04-09

ER

PT J

AU Sharma, H

AF Sharma, Horrick

TI Development of Novel Therapeutics Targeting Isocitrate Dehydrogenase

Mutations in Cancer

SO CURRENT TOPICS IN MEDICINAL CHEMISTRY

LA English

DT Review

DE Isocitrate dehydrogenase; mutation; Glioma; AML; cancer metabolism; mIDH

inhibitors; cancer

ID ACUTE MYELOID-LEUKEMIA; HEALTH-ORGANIZATION CLASSIFICATION; INTEGRATED

GENOMIC ANALYSIS; CENTRAL-NERVOUS-SYSTEM; CODON 132 MUTATION; IDH2

MUTATIONS; MUTANT IDH1; MYELODYSPLASTIC SYNDROMES;

PROGNOSTIC-SIGNIFICANCE; ONCOMETABOLITE 2-HYDROXYGLUTARATE

AB Isocitrate dehydrogenases 1 and 2 (IDH1 and IDH2) are key metabolic enzymes that catalyze the conversion of isocitrate to alpha-ketoglutarate (alpha KG). IDH1 and IDH2 regulate several cellular processes, including oxidative respiration, glutamine metabolism, lipogenesis, and cellular defense against oxidative damage. Mutations in IDH1 and IDH2 have recently been observed in multiple tumors, including gliomas, acute myeloid leukemia, myelodysplastic syndromes, and chondrosarcoma. IDH1 and IDH2 mutations involve a gain in neomorphic activity that catalyzes alpha KG conversion to (R)-2-hydroxyglutarate ((R)-2HG). IDH mutation-mediated accumulation of (R)-2HG results in epigenetic dysregulation, altered gene expression, and a block in cellular differentiation. Targeting mutant IDH by development of small molecule inhibitors is a rapidly emerging therapeutic approach as evidenced by the recent approval of the first selective mutant IDH2 inhibitor AG-221 (enasidenib) for the treatment of IDH2-mutated AML. This review will focus on mutant isocitrate dehydrogenase as a therapeutic drug target and provides an update on selective and pan-mutant IDH1/2 inhibitors in clinical trials and other mutant IDH inhibitors that are under development.

C1 [Sharma, Horrick] Southwestern Oklahoma State Univ, Dept Pharmaceut Sci, Coll Pharm, Weatherford, OK 73096 USA.

RP Sharma, H (corresponding author), Southwestern Oklahoma State Univ, Dept Pharmaceut Sci, Coll Pharm, Weatherford, OK 73096 USA.

EM horrick.sharma@swosu.edu

CR Abbas S, 2010, BLOOD, V116, P2122, DOI 10.1182/blood-2009-11-250878

Acker T, 2005, CANCER CELL, V8, P131, DOI 10.1016/j.ccr.2005.07.003

Agarwal S, 2013, NEURO-ONCOLOGY, V15, P718, DOI 10.1093/neuonc/not015

Amary MF, 2015, VIRCHOWS ARCH, V466, P217, DOI 10.1007/s00428-014-1685-4

Amary MF, 2011, J PATHOL, V224, P334, DOI 10.1002/path.2913

[Anonymous], MUTANT IDH1 AML ADV

[Anonymous], ENGL J MED

[Anonymous], 2016, BLOOD

[Anonymous], 2015, J CLIN ONCOL S

Badiali M, 2012, BRAIN PATHOL, V22, P841, DOI 10.1111/j.1750-3639.2012.00603.x

Balss J, 2008, ACTA NEUROPATHOL, V116, P597, DOI 10.1007/s00401-008-0455-2

Berger SL, 2007, NATURE, V447, P407, DOI 10.1038/nature05915

Birner P, 2014, CANCER-AM CANCER SOC, V120, P2440, DOI 10.1002/cncr.28732

Boissel N, 2011, BLOOD, V117, P3696, DOI 10.1182/blood-2010-11-320937

Boland F, 2017, J CLIN ONCOL, V35, DOI 10.1200/JCO.2017.35.15_suppl.e18128

Borger DR, 2012, ONCOLOGIST, V17, P72, DOI 10.1634/theoncologist.2011-0386

Bralten LBC, 2011, ANN NEUROL, V69, P455, DOI 10.1002/ana.22390

Brooks E, 2014, J BIOMOL SCREEN, V19, P1193, DOI 10.1177/1087057114541148

Cairncross G, 2013, J CLIN ONCOL, V31, P337, DOI 10.1200/JCO.2012.43.2674

Cairncross JG, 2014, J CLIN ONCOL, V32, P783, DOI 10.1200/JCO.2013.49.3726

Cairns RA, 2012, BLOOD, V119, P1901, DOI 10.1182/blood-2011-11-391748

Carmeliet P, 1998, NATURE, V394, P485, DOI 10.1038/28867

Ceccarelli M, 2016, CELL, V164, P550, DOI 10.1016/j.cell.2015.12.028

Chaturvedi A, 2017, LEUKEMIA, V31, P2020, DOI 10.1038/leu.2017.46

Chen RH, 2014, P NATL ACAD SCI USA, V111, P14217, DOI 10.1073/pnas.1409653111

Chesnelong C, 2014, NEURO-ONCOLOGY, V16, P686, DOI 10.1093/neuonc/not243

Chi P, 2010, NAT REV CANCER, V10, P457, DOI 10.1038/nrc2876

Cho YS, 2017, ACS MED CHEM LETT, V8, P1116, DOI 10.1021/acsmedchemlett.7b00342

Chotirat S, 2012, J HEMATOL ONCOL, V5, DOI 10.1186/1756-8722-5-5

Chou WC, 2011, LEUKEMIA, V25, P246, DOI 10.1038/leu.2010.267

Chowdhury R, 2011, EMBO REP, V12, P463, DOI 10.1038/embor.2011.43

Dang CV, 2012, GENE DEV, V26, P877, DOI 10.1101/gad.189365.112

Dang LN, 2010, TRENDS MOL MED, V16, P387, DOI 10.1016/j.molmed.2010.07.002

Dang L, 2009, NATURE, V462, P739, DOI 10.1038/nature08617

Davis MI, 2014, J BIOL CHEM, V289, P13717, DOI 10.1074/jbc.M113.511030

DeBerardinis RJ, 2008, CELL METAB, V7, P11, DOI 10.1016/j.cmet.2007.10.002

Deng GJ, 2015, J BIOL CHEM, V290, P762, DOI 10.1074/jbc.M114.608497

DiNardo CD, 2015, AM J HEMATOL, V90, P732, DOI 10.1002/ajh.24072

Doherty JR, 2013, J CLIN INVEST, V123, P3685, DOI 10.1172/JCI69741

Dunn GP, 2013, NEUROSURG FOCUS, V34, DOI 10.3171/2012.12.FOCUS12355

Ebrahimi A, 2016, ACTA NEUROPATHOL COM, V4, DOI 10.1186/s40478-016-0331-6

Emadi A, 2014, EXP HEMATOL, V42, P247, DOI 10.1016/j.exphem.2013.12.001

Esmaeili M, 2014, CANCER RES, V74, P4898, DOI 10.1158/0008-5472.CAN-14-0008

Fack F, 2017, EMBO MOL MED, V9, P1681, DOI 10.15252/emmm.201707729

Fan J, 2015, ACS CHEM BIOL, V10, P510, DOI 10.1021/cb500683c

Fan J, 2014, NATURE, V510, P298, DOI 10.1038/nature13236

Fang F, 2011, SCI TRANSL MED, V3, DOI 10.1126/scitranslmed.3001875

Fendt SM, 2013, NAT COMMUN, V4, DOI 10.1038/ncomms3236

Feng JH, 2012, AM J BLOOD RES, V2, P254

Figueroa ME, 2010, CANCER CELL, V18, P553, DOI 10.1016/j.ccr.2010.11.015

Fouad YA, 2017, AM J CANCER RES, V7, P1016

Ghiam AF, 2012, ONCOGENE, V31, P3826, DOI 10.1038/onc.2011.546

Gorovets D, 2012, CLIN CANCER RES, V18, P2490, DOI 10.1158/1078-0432.CCR-11-2977

Grassian AR, 2014, CANCER RES, V74, P3317, DOI 10.1158/0008-5472.CAN-14-0772-T

Green CL, 2011, BLOOD, V118, P409, DOI 10.1182/blood-2010-12-322479

Gross S, 2010, J EXP MED, V207, P339, DOI 10.1084/jem.20092506

Gupta A, 2017, J NEUROSCI RURAL PRA, V8, P629, DOI 10.4103/jnrp.jnrp_168_17

Heiden MGV, 2009, SCIENCE, V324, P1029, DOI 10.1126/science.1160809

Hoekstra AS, 2015, ONCOTARGET, V6, P38777, DOI 10.18632/oncotarget.6091

Holness MJ, 2003, BIOCHEM SOC T, V31, P1143

Im AP, 2014, LEUKEMIA, V28, P1774, DOI 10.1038/leu.2014.124

Isaacs JS, 2005, CANCER CELL, V8, P143, DOI 10.1016/j.ccr.2005.06.017

Ito S, 2011, SCIENCE, V333, P1300, DOI 10.1126/science.1210597

Iyer LM, 2009, CELL CYCLE, V8, P1698, DOI 10.4161/cc.8.11.8580

Izquierdo-Garcia JL, 2015, CANCER RES, V75, P2999, DOI 10.1158/0008-5472.CAN-15-0840

Izquierdo-Garcia JL, 2015, PLOS ONE, V10, DOI 10.1371/journal.pone.0118781

Jin J, 2014, PLOS ONE, V9, DOI 10.1371/journal.pone.0100206

Jones PA, 2007, CELL, V128, P683, DOI 10.1016/j.cell.2007.01.029

Jones S, 2016, J MED CHEM, V59, P11120, DOI 10.1021/acs.jmedchem.6b01320

Kang MR, 2009, INT J CANCER, V125, P353, DOI 10.1002/ijc.24379

Ko M, 2010, NATURE, V468, P839, DOI 10.1038/nature09586

Koivunen P, 2012, NATURE, V483, P485, DOI 10.1038/nature10898

Konopleva M, 2014, BLOOD, V124, DOI 10.1182/blood.V124.21.118.118

Kosmider O, 2010, LEUKEMIA, V24, P1094, DOI 10.1038/leu.2010.52

Kranendijk M, 2012, J INHERIT METAB DIS, V35, P571, DOI 10.1007/s10545-012-9462-5

Kroeze LI, 2014, BLOOD, V124, P1110, DOI 10.1182/blood-2013-08-518514

Lai A, 2011, J CLIN ONCOL, V29, P4482, DOI 10.1200/JCO.2010.33.8715

Lasho TL, 2012, BLOOD, V120, P4168, DOI 10.1182/blood-2012-05-429696

Lass U, 2012, PLOS ONE, V7, DOI 10.1371/journal.pone.0041298

Law JM, 2016, ACS MED CHEM LETT, V7, P944, DOI 10.1021/acsmedchemlett.6b00264

Leonardi R, 2012, J BIOL CHEM, V287, P14615, DOI 10.1074/jbc.C112.353946

Lewis CA, 2014, MOL CELL, V55, P253, DOI 10.1016/j.molcel.2014.05.008

Ley TJ, 2013, NEW ENGL J MED, V368, P2059, DOI 10.1056/NEJMoa1301689

Lin CC, 2014, AM J HEMATOL, V89, P137, DOI 10.1002/ajh.23596

Liu Z, 2014, J MED CHEM, V57, P8307, DOI 10.1021/jm500660f

Loenarz C, 2008, NAT CHEM BIOL, V4, P152, DOI 10.1038/nchembio0308-152

Losman JA, 2013, GENE DEV, V27, P836, DOI 10.1101/gad.217406.113

Losman JA, 2013, SCIENCE, V339, P1621, DOI 10.1126/science.1231677

Louis DN, 2007, ACTA NEUROPATHOL, V114, P97, DOI 10.1007/s00401-007-0243-4

Louis DN, 2016, ACTA NEUROPATHOL, V131, P803, DOI 10.1007/s00401-016-1545-1

Lu C, 2012, NATURE, V483, P474, DOI 10.1038/nature10860

Ma TF, 2017, BIOORGAN MED CHEM, V25, P6379, DOI 10.1016/j.bmc.2017.10.009

Mack FA, 2003, CANCER CELL, V3, P75, DOI 10.1016/S1535-6108(02)00240-4

Marcucci G, 2010, J CLIN ONCOL, V28, P2348, DOI 10.1200/JCO.2009.27.3730

Mardis ER, 2009, NEW ENGL J MED, V361, P1058, DOI 10.1056/NEJMoa0903840

Markolovic S, 2015, J BIOL CHEM, V290, P20712, DOI 10.1074/jbc.R115.662627

Mauzo SH, 2014, APPL IMMUNOHISTO M M, V22, P284, DOI 10.1097/PAI.0b013e3182649d1c

Medeiros BC, 2017, LEUKEMIA, V31, P272, DOI 10.1038/leu.2016.275

Mellinghoff IK, 2017, NEURO-ONCOLOGY, V19, P10

Metallo CM, 2012, NATURE, V481, P380, DOI 10.1038/nature10602

Molenaar RJ, 2015, CANCER RES, V75, P4790, DOI 10.1158/0008-5472.CAN-14-3603

Nagarajan A, 2016, TRENDS CANCER, V2, P365, DOI 10.1016/j.trecan.2016.06.002

Nobusawa S, 2009, CLIN CANCER RES, V15, P6002, DOI 10.1158/1078-0432.CCR-09-0715

Noushmehr H, 2010, CANCER CELL, V17, P510, DOI 10.1016/j.ccr.2010.03.017

Ohka F, 2014, TUMOR BIOL, V35, P5911, DOI 10.1007/s13277-014-1784-5

Okoye-Okafor UC, 2015, NAT CHEM BIOL, V11, P878, DOI 10.1038/nchembio.1930

Papaemmanuil E, 2016, NEW ENGL J MED, V374, P2209, DOI 10.1056/NEJMoa1516192

Pardanani A, 2010, LEUKEMIA, V24, P1370, DOI 10.1038/leu.2010.98

Parsons DW, 2008, SCIENCE, V321, P1807, DOI 10.1126/science.1164382

Paschka P, 2010, J CLIN ONCOL, V28, P3636, DOI 10.1200/JCO.2010.28.3762

Patay Z, 2012, AM J NEURORADIOL, V33, P940, DOI 10.3174/ajnr.A2869

Patel KP, 2011, AM J CLIN PATHOL, V135, P35, DOI 10.1309/AJCPD7NR2RMNQDVF

Patnaik MM, 2012, LEUKEMIA, V26, P101, DOI 10.1038/leu.2011.298

Pérez C, 2012, PLOS ONE, V7, DOI 10.1371/journal.pone.0031605

Pollak N, 2007, BIOCHEM J, V402, P205, DOI 10.1042/BJ20061638

Popovici-Muller J, 2012, ACS MED CHEM LETT, V3, P850, DOI 10.1021/ml300225h

Raval RR, 2005, MOL CELL BIOL, V25, P5675, DOI 10.1128/MCB.25.13.5675-5686.2005

Reitman ZJ, 2011, P NATL ACAD SCI USA, V108, P3270, DOI 10.1073/pnas.1019393108

Reitman ZJ, 2010, JNCI-J NATL CANCER I, V102, P932, DOI 10.1093/jnci/djq187

Rohle D, 2013, SCIENCE, V340, P626, DOI 10.1126/science.1236062

Ronnebaum SM, 2006, J BIOL CHEM, V281, P30593, DOI 10.1074/jbc.M511908200

Rzem R, 2007, J INHERIT METAB DIS, V30, P681, DOI 10.1007/s10545-007-0487-0

Saha SK, 2014, NATURE, V513, P110, DOI 10.1038/nature13441

Sanson M, 2009, J CLIN ONCOL, V27, P4150, DOI 10.1200/JCO.2009.21.9832

Sasaki M, 2012, GENE DEV, V26, P2038, DOI 10.1101/gad.198200.112

Selak MA, 2005, CANCER CELL, V7, P77, DOI 10.1016/j.ccr.2004.11.022

Sellner L, 2010, EUR J HAEMATOL, V85, P457, DOI 10.1111/j.1600-0609.2010.01505.x

Seltzer MJ, 2010, CANCER RES, V70, P8981, DOI 10.1158/0008-5472.CAN-10-1666

Sequist LV, 2011, ANN ONCOL, V22, P2616, DOI 10.1093/annonc/mdr489

Shi JL, 2015, TUMOR BIOL, V36, P655, DOI 10.1007/s13277-014-2644-z

Shih AH, 2014, BLOOD, V124, DOI 10.1182/blood.V124.21.437.437

Sjöblom T, 2006, SCIENCE, V314, P268, DOI 10.1126/science.1133427

Song LP, 2008, ONCOGENE, V27, P519, DOI 10.1038/sj.onc.1210670

Stein EM, 2018, FUTURE ONCOL, V14, P23, DOI 10.2217/fon-2017-0392

Stein EM, 2017, BLOOD, V130, P722, DOI 10.1182/blood-2017-04-779405

Struys EA, 2005, J INHERIT METAB DIS, V28, P921, DOI 10.1007/s10545-005-0114-x

Struys EA, 2004, CLIN CHEM, V50, P1391, DOI 10.1373/clinchem.2004.033399

Struys EA, 2004, FEBS LETT, V557, P115, DOI 10.1016/S0014-5793(03)01459-5

Tahiliani M, 2009, SCIENCE, V324, P930, DOI 10.1126/science.1170116

Tan FW, 2012, MOL CELL PROTEOMICS, V11, DOI 10.1074/mcp.M111.008821

Tang JY, 2012, KAOHSIUNG J MED SCI, V28, P138, DOI 10.1016/j.kjms.2011.10.023

Tefferi A, 2010, LEUKEMIA, V24, P1302, DOI 10.1038/leu.2010.113

Thol F, 2010, HAEMATOL-HEMATOL J, V95, P1668, DOI 10.3324/haematol.2010.025494

Toyota M, 1999, P NATL ACAD SCI USA, V96, P8681, DOI 10.1073/pnas.96.15.8681

van den Bent MJ, 2013, J CLIN ONCOL, V31, P344, DOI 10.1200/JCO.2012.43.2229

Vannucchi AM, 2013, LEUKEMIA, V27, P1861, DOI 10.1038/leu.2013.119

Verhaak RGW, 2010, CANCER CELL, V17, P98, DOI 10.1016/j.ccr.2009.12.020

Viswanath P, 2016, ONCOTARGET, V7, P34942, DOI 10.18632/oncotarget.9006

Wagner K, 2010, J CLIN ONCOL, V28, P2356, DOI 10.1200/JCO.2009.27.6899

Wakimoto H, 2014, CLIN CANCER RES, V20, P2898, DOI 10.1158/1078-0432.CCR-13-3052

Wang F, 2013, SCIENCE, V340, P622, DOI 10.1126/science.1234769

Wang P, 2013, ONCOGENE, V32, P3091, DOI 10.1038/onc.2012.315

Ward PS, 2013, J BIOL CHEM, V288, P3804, DOI 10.1074/jbc.M112.435495

Ward PS, 2012, CSH PERSPECT BIOL, V4, DOI 10.1101/cshperspect.a006783

Ward PS, 2010, CANCER CELL, V17, P225, DOI 10.1016/j.ccr.2010.01.020

Watanabe T, 2009, AM J PATHOL, V174, P1149, DOI 10.2353/ajpath.2009.080958

Williams SC, 2011, ACTA NEUROPATHOL, V121, P279, DOI 10.1007/s00401-010-0790-y

Wu FR, 2015, J MED CHEM, V58, P6899, DOI 10.1021/acs.jmedchem.5b00684

Xu W, 2011, CANCER CELL, V19, P17, DOI 10.1016/j.ccr.2010.12.014

Xu X, 2004, J BIOL CHEM, V279, P33946, DOI 10.1074/jbc.M404298200

Yan H, 2009, NEW ENGL J MED, V360, P765, DOI 10.1056/NEJMoa0808710

Yang B, 2010, CELL RES, V20, P1188, DOI 10.1038/cr.2010.145

Yen K, 2018, MOL CANCER THER, V17, DOI 10.1158/1535-7163.TARG-17-B126

Yip S, 2012, J PATHOL, V226, P7, DOI 10.1002/path.2995

Zdzisinska B, 2017, ARCH IMMUNOL THER EX, V65, P21, DOI 10.1007/s00005-016-0406-x

Zeng AL, 2015, ONCOTARGET, V6, P30232, DOI 10.18632/oncotarget.4920

Zhao SM, 2009, SCIENCE, V324, P261, DOI 10.1126/science.1170944

Zheng BS, 2013, ACS MED CHEM LETT, V4, P542, DOI 10.1021/ml400036z

Zheng QG, 2017, BIOORG MED CHEM LETT, V27, P5262, DOI 10.1016/j.bmcl.2017.10.029

NR 169

TC 24

Z9 26

U1 2

U2 27

PU BENTHAM SCIENCE PUBL LTD

PI SHARJAH

PA EXECUTIVE STE Y-2, PO BOX 7917, SAIF ZONE, 1200 BR SHARJAH, U ARAB

EMIRATES

SN 1568-0266

EI 1873-4294

J9 CURR TOP MED CHEM

JI Curr. Top. Med. Chem.

PY 2018

VL 18

IS 6

BP 505

EP 524

DI 10.2174/1568026618666180518091144

PG 20

WC Chemistry, Medicinal

WE Science Citation Index Expanded (SCI-EXPANDED)

SC Pharmacology & Pharmacy

GA GL2ZH

UT WOS:000436995100008

PM 29773061

DA 2025-04-09

ER

PT J

AU Hatziagapiou, K

Nikola, O

Marka, S

Koniari, E

Kakouri, E

Zografaki, ME

Mavrikou, SS

Kanakis, C

Flemetakis, E

Chrousos, GP

Kintzios, S

Lambrou, G

Kanaka-Gantenbein, C

Tarantilis, PA

AF Hatziagapiou, Kyriaki

Nikola, Olti

Marka, Sofia

Koniari, Eleni

Kakouri, Eleni

Zografaki, Maria-Eleftheria

Mavrikou, Sophie S.

Kanakis, Charalabos

Flemetakis, Emmanouil

Chrousos, George P.

Kintzios, Spyridon

Lambrou, George, I

Kanaka-Gantenbein, Christina

Tarantilis, Petros A.

TI An In Vitro Study of Saffron Carotenoids: The Effect of Crocin Extracts

and Dimethylcrocetin on Cancer Cell Lines

SO ANTIOXIDANTS

LA English

DT Article

DE crocins (CRCs); dimethylocrocetin (DMCRT); saffron; glioblastoma (GBM);

rhabdomyosarcoma; cytotoxicity

ID TO-MODERATE DEPRESSION; INDUCED OXIDATIVE DAMAGE; ETHANOL-INDUCED

IMPAIRMENT; SATIVUS STIGMA EXTRACT; HUMAN SERUM-ALBUMIN; DOUBLE-BLIND;

BREAST-CANCER; GASTRIC-CARCINOMA; SAFETY EVALUATION; PROMYELOCYTIC

LEUKEMIA

AB Crocus sativus L. has various pharmacological properties, known for over 3600 years. These properties are attributed mainly to biologically active substances, which belong to the terpenoid group and include crocins, picrocrocin and safranal. The aim of the current work was to examine the effects of crocins (CRCs) and their methyl ester derivate dimethylcrocetin (DMCRT) on glioblastoma and rhabdomyosarcoma cell lines, in terms of cytotoxicity and gene expression, implicated in proapoptotic and cell survival pathways. Cell cytotoxicity was assessed with Alamar Blue fluorescence assay after treatment with saffron carotenoids for 24, 48 and 72 h and concentrations ranging from 22.85 to 0.18 mg/mL for CRCs and 11.43 to 0.09 mg/mL for DMCRT. In addition, BAX, BID, BCL2, MYCN, SOD1, and GSTM1 gene expression was studied by qRT-PCR analysis. Both compounds demonstrated cytotoxic effects against glioblastoma and rhabdomyosarcoma cell lines, in a dose- and time-dependent manner. They induced apoptosis, via BAX and BID upregulation, MYCN and BCL-2, SOD1, GSTM1 downregulation. The current research denotes the possible anticancer properties of saffron carotenoids, which are considered safe phytochemicals, already tested in clinical trials for their health promoting properties.

C1 [Hatziagapiou, Kyriaki; Nikola, Olti; Lambrou, George, I; Kanaka-Gantenbein, Christina] Natl & Kapodistrian Univ Athens, Dept Pediat 1, Choremeio Res Lab, Thivon & Levadeias 8, Athens 11527, Greece.

[Hatziagapiou, Kyriaki] State Univ West Attica, Fac Hlth & Care Sci, Physiotherapy Dept, Agiou Spiridonos 28, Athens 12243, Greece.

[Marka, Sofia; Mavrikou, Sophie S.; Kintzios, Spyridon] Agr Univ Athens, EU CONEXUS European Univ, Dept Biotechnol, Lab Cell Technol, Athens 11855, Greece.

[Koniari, Eleni; Chrousos, George P.] Natl & Kapodistrian Univ Athens, Univ Res Inst Maternal & Child Hlth & Precis Med, UNESCO Chair Adolescent Hlth Care, Thivon & Levadeias 8, Athens 11527, Greece.

[Kakouri, Eleni; Kanakis, Charalabos; Tarantilis, Petros A.] Agr Univ Athens, EU CONEXUS European Univ, Sch Food Biotechnol & Dev, Dept Food Sci & Human Nutr,Lab Chem, Athens 11855, Greece.

[Zografaki, Maria-Eleftheria; Flemetakis, Emmanouil] Agr Univ Athens, EU CONEXUS European Univ, Dept Biotechnol, Lab Mol Biol, Athens 11855, Greece.

C3 National & Kapodistrian University of Athens; Agricultural University of

Athens; National & Kapodistrian University of Athens; Agricultural

University of Athens; Agricultural University of Athens

RP Hatziagapiou, K (corresponding author), Natl & Kapodistrian Univ Athens, Dept Pediat 1, Choremeio Res Lab, Thivon & Levadeias 8, Athens 11527, Greece.; Hatziagapiou, K (corresponding author), State Univ West Attica, Fac Hlth & Care Sci, Physiotherapy Dept, Agiou Spiridonos 28, Athens 12243, Greece.

EM khatziag@med.uoa.gr; onikola@med.uoa.gr; smarka@aua.gr;

hkoniari@med.uoa.gr; elenikakouri@aua.gr; mzografaki@aua.gr;

sophie_mav@aua.gr; chkanakis@aua.gr; mflem@aua.gr; chrousge@med.uoa.gr;

skin@aua.gr; ckanaka@med.uoa.gr; ptara@aua.gr

RI Chrousos, George/G-8702-2011; Tarantilis, Petros/ABE-4458-2020;

MAVRIKOU, SOFIA/HSC-2249-2023; Kakouri, Eleni/GLU-6579-2022; Flemetakis,

Emmanouil/KIG-5606-2024; HATZIAGAPIOU, KYRIAKI/E-6264-2018;

Kanaka-Gantenbein, Christina/AAP-3697-2020; Lambrou, George/F-9674-2011;

Kanakis, Charalabos/AFN-3581-2022

OI Lambrou, George/0000-0001-8389-1360; Kanakis,

Charalabos/0000-0001-7866-8279; MAVRIKOU, SOFIA/0000-0001-6565-3473;

KINTZIOS, SPYRIDON/0000-0003-2841-446X; Nikola,

Olti/0000-0001-9379-5946; Kakouri, Eleni/0000-0002-6233-2874;

Tarantilis, Petros A./0000-0002-5853-4780

FU European Union (European Social Fund) through the Operational Program

"Human Resources Development, Education and Lifelong Learning

2014-2020"; Program encoded EDBM103, titled "Support for researchers

with an emphasis on young researchers-cycle B" [MIS 5048464]

FX The Research Project is co-financed by Greece and the European Union

(European Social Fund) through the Operational Program "Human Resources

Development, Education and Lifelong Learning 2014-2020" and the Program

encoded EDBM103, titled "Support for researchers with an emphasis on

young researchers-cycle B" (MIS 5048464).

CR Abdullaev FI, 2003, TOXICOL IN VITRO, V17, P731, DOI 10.1016/S0887-2333(03)00098-5

ABDULLAEV FI, 1992, BIOFACTORS, V4, P43

Abdullaev FI, 2002, EXP BIOL MED, V227, P20, DOI 10.1177/153537020222700104

ABDULLAEV FI, 1994, TOXICOL LETT, V70, P243, DOI 10.1016/0378-4274(94)90168-6

Ahmadabadi F, 2021, PHYSIOL INT, V108, P19, DOI 10.1556/2060.2021.00009

Ajam M., 2014, ASIA PACIFIC J MED T, V3, P73

Akhondzadeh S, 2005, PHYTOTHER RES, V19, P148, DOI 10.1002/ptr.1647

Akhondzadeh S, 2010, J CLIN PHARM THER, V35, P581, DOI 10.1111/j.1365-2710.2009.01133.x

Akhondzadeh S, 2010, PSYCHOPHARMACOLOGY, V207, P637, DOI 10.1007/s00213-009-1706-1

Alavizadeh SH, 2014, FOOD CHEM TOXICOL, V64, P65, DOI 10.1016/j.fct.2013.11.016

Almodóvar P, 2020, EVID-BASED COMPL ALT, V2020, DOI 10.1155/2020/1575730

Amin A, 2021, MOLECULES, V26, DOI 10.3390/molecules26133855

Amin A, 2015, INT J MOL SCI, V16, P1544, DOI 10.3390/ijms16011544

Amin B, 2014, AN ACAD BRAS CIENC, V86, P1821, DOI 10.1590/0001-3765201420140067

An BZ, 2020, FOLIA NEUROPATHOL, V58, P201, DOI 10.5114/fn.2020.100063

[Anonymous], 2019, Basic Clinical Cancer Research, DOI DOI 10.18502/BCCR.V11I1.1646

[Anonymous], 2018, Pathobiology Research

Ashrafi M, 2015, DNA CELL BIOL, V34, P684, DOI 10.1089/dna.2015.2951

Assimiadis MK, 1998, APPL SPECTROSC, V52, P519, DOI 10.1366/0003702981944058

Assimopoulou AN, 2005, PHYTOTHER RES, V19, P997, DOI 10.1002/ptr.1749

Aung H H, 2007, Exp Oncol, V29, P175

Ayatollahi H, 2014, PHYTOTHER RES, V28, P539, DOI 10.1002/ptr.5021

Azmi AS, 2011, EXPERT OPIN EMERG DR, V16, P59, DOI 10.1517/14728214.2010.515210

Babaei A, 2014, AVICENNA J PHYTOMEDI, V4, P103

Bakshi H, 2010, ASIAN PAC J CANCER P, V11, P675

Bakshi HA, 2022, CELLS-BASEL, V11, DOI 10.3390/cells11091502

Bakshi HA, 2020, NUTRIENTS, V12, DOI 10.3390/nu12061901

Bakshi Hamid A, 2016, Asian Pac J Cancer Prev, V17, P1499

Bandegi AR, 2014, ADV PHARM BULL, V4, P493, DOI 10.5681/apb.2014.073

Basti AA, 2007, PROG NEURO-PSYCHOPH, V31, P439, DOI 10.1016/j.pnpbp.2006.11.010

Bathaie SZ, 2010, CRIT REV FOOD SCI, V50, P761, DOI 10.1080/10408390902773003

BELJEBBAR A, 1995, SPECTROSCOPY OF BIOLOGICAL MOLECULES, P475

Bi XQ, 2021, BIOENGINEERED, V12, P4569, DOI 10.1080/21655979.2021.1955175

Bijani Fatima, 2021, Dent Res J (Isfahan), V18, P70

Bolhassani A, 2014, BBA-REV CANCER, V1845, P20, DOI 10.1016/j.bbcan.2013.11.001

Bostan HB, 2017, IRAN J BASIC MED SCI, V20, P110, DOI 10.22038/ijbms.2017.8230

Broadhead GK, 2016, CRIT REV FOOD SCI, V56, P2767, DOI 10.1080/10408398.2013.879467

Brook, 2020, UROL J, P6331, DOI [10.22037/uj.v16i7.6331, DOI 10.22037/UJ.V16I7.6331]

Butnariu M, 2022, OXID MED CELL LONGEV, V2022, DOI 10.1155/2022/8214821

Caballero-Ortega H, 2007, FOOD CHEM, V100, P1126, DOI 10.1016/j.foodchem.2005.11.020

Carmona M, 2006, J AGR FOOD CHEM, V54, P973, DOI 10.1021/jf052297w

Chan SL, 2004, CLIN EXP PHARMACOL P, V31, P119, DOI 10.1111/j.1440-1681.2004.03975.x

Chen SS, 2015, TRANSL LUNG CANCER R, V4, P775, DOI 10.3978/j.issn.2218-6751.2015.11.03

Christodoulou E, 2019, J PHARM PHARMACOL, V71, P753, DOI 10.1111/jphp.13055

Christodoulou E, 2015, J PHARM PHARMACOL, V67, P1634, DOI 10.1111/jphp.12456

Chryssanthi DG, 2007, ANTICANCER RES, V27, P357

Chryssanthi DG, 2011, J PHARMACEUT BIOMED, V55, P563, DOI 10.1016/j.jpba.2011.02.018

Chryssanthi DG, 2011, PLANTA MED, V77, P146, DOI 10.1055/s-0030-1250178

Chu X, 2020, INT IMMUNOPHARMACOL, V84, DOI 10.1016/j.intimp.2020.106548

Colapietro A, 2020, INT J MOL SCI, V21, DOI 10.3390/ijms21020423

CRAGG GM, 1994, CIBA F SYMP, V185, P178

D'Alessandro AM, 2013, NUTR CANCER, V65, P930, DOI 10.1080/01635581.2013.767368

Das I, 2010, ACTA HISTOCHEM, V112, P317, DOI 10.1016/j.acthis.2009.02.003

Deng LY, 2019, J BIOCHEM MOL TOXIC, V33, DOI 10.1002/jbt.22292

Dhar A, 2009, MOL CANCER THER, V8, P315, DOI 10.1158/1535-7163.MCT-08-0762

Du JK, 2021, PSYCHOPHARMACOLOGY, V238, P2839, DOI 10.1007/s00213-021-05899-4

El Midaoui A, 2022, NUTRIENTS, V14, DOI 10.3390/nu14030597

Escribano J, 1996, CANCER LETT, V100, P23, DOI 10.1016/0304-3835(95)04067-6

Fagot D, 2018, INT J COSMETIC SCI, V40, P388, DOI 10.1111/ics.12472

Farokhnia M, 2014, HUM PSYCHOPHARM CLIN, V29, P351, DOI 10.1002/hup.2412

Feizzadeh Behzad, 2008, Urol J, V5, P161

Ferlay J, 2019, INT J CANCER, V144, P1941, DOI 10.1002/ijc.31937

Ferrucci V, 2016, N-S ARCH PHARMACOL, V389, P131, DOI 10.1007/s00210-015-1191-5

Fridlender M, 2015, FRONT PLANT SCI, V6, DOI 10.3389/fpls.2015.00799

García-Olmo DC, 1999, NUTR CANCER, V35, P120, DOI 10.1207/S15327914NC352_4

Geromichalos GD, 2014, FOOD CHEM TOXICOL, V74, P45, DOI 10.1016/j.fct.2014.09.001

Gezici S, 2019, J FOOD SCI TECH MYS, V56, P5435, DOI 10.1007/s13197-019-04014-y

Ghadrdoost B, 2011, EUR J PHARMACOL, V667, P222, DOI 10.1016/j.ejphar.2011.05.012

Ghajar A, 2017, PHARMACOPSYCHIATRY, V50, P152, DOI 10.1055/s-0042-116159

Ghobadi H, 2022, FRONT PHARMACOL, V13, DOI 10.3389/fphar.2022.884710

Giaccio M, 2004, CRIT REV FOOD SCI, V44, P155, DOI 10.1080/10408690490441433

Guo ZL, 2022, FRONT PHARMACOL, V12, DOI 10.3389/fphar.2021.745683

Gutheil WG, 2012, CURR PHARM BIOTECHNO, V13, P173

Hamid R, 2004, TOXICOL IN VITRO, V18, P703, DOI 10.1016/j.tiv.2004.03.012

Hashemi SA, 2020, AVICENNA J PHYTOMEDI, V10, P384, DOI 10.22038/ajp.2019.14372

Hoshyar R, 2017, J PHARM PHARMACOL, V69, P1419, DOI 10.1111/jphp.12776

Hoshyar R, 2013, DNA CELL BIOL, V32, P50, DOI 10.1089/dna.2012.1866

Hosseini A, 2018, EUR J DRUG METAB PH, V43, P383, DOI 10.1007/s13318-017-0449-3

Hosseinzadeh H, 2005, J PHARM PHARM SCI, V8, P394

Hosseinzadeh H, 2005, J PHARM PHARM SCI, V8, P387

Hosseinzadeh H., 2010, PHARMACOLOGYONLINE, V2, P943

Hosseinzadeh H, 2008, DNA CELL BIOL, V27, P657, DOI 10.1089/dna.2008.0767

Hosseinzadeh H, 2014, JUNDISHAPUR J NAT PH, V9, P1

Iqbal J, 2017, ASIAN PAC J TROP BIO, V7, P1129, DOI 10.1016/j.apjtb.2017.10.016

Jabini R, 2017, NUTR CANCER, V69, P911, DOI 10.1080/01635581.2017.1339816

Jafarisani M, 2018, J BIOMOL STRUCT DYN, V36, P1681, DOI 10.1080/07391102.2017.1331865

Jafarnia N, 2017, ARCH NEUROSCI, V4, DOI 10.5812/archneurosci.14332

Jahromi AS, 2021, MEDITERR J HEMATOL I, V13, DOI 10.4084/MJHID.2021.049

Jiang ZM, 2018, J PHOTOCH PHOTOBIO B, V180, P118, DOI 10.1016/j.jphotobiol.2018.01.013

Kakouri E, 2020, MOLECULES, V25, DOI 10.3390/molecules25225223

Kanakis CD, 2009, J PHOTOCH PHOTOBIO B, V95, P204, DOI 10.1016/j.jphotobiol.2009.03.006

Kanakis CD, 2007, J AGR FOOD CHEM, V55, P970, DOI 10.1021/jf062638l

Karimi G., 2004, Journal of Medicinal Plants, V3, P29

Karkoula E, 2018, J SEP SCI, V41, DOI 10.1002/jssc.201800516

Kim B, 2018, ONCOL REP, V39, P1883, DOI 10.3892/or.2018.6232

Kim B, 2017, J CELL BIOCHEM, V118, P3290, DOI 10.1002/jcb.25980

Kim SH, 2014, BIOCHEM CELL BIOL, V92, P105, DOI 10.1139/bcb-2013-0091

Laabich A, 2006, INVEST OPHTH VIS SCI, V47, P3156, DOI 10.1167/iovs.05-1621

Lautenschläger M, 2015, PHYTOMEDICINE, V22, P36, DOI 10.1016/j.phymed.2014.10.009

Li S, 2019, PHARMACOLOGY, V103, P263, DOI 10.1159/000487956

Li S, 2017, CANCER CELL INT, V17, DOI 10.1186/s12935-017-0468-9

Li S, 2015, ONCOL LETT, V9, P1254, DOI 10.3892/ol.2015.2869

Li XC, 2013, TOXICOL LETT, V221, P197, DOI 10.1016/j.toxlet.2013.06.233

Li XM, 2021, FRONT CELL DEV BIOL, V9, DOI 10.3389/fcell.2021.651434

LiakopoulouKyriakides M., 2002, Studies in natural products chemistry, V26, P293

Linardaki ZI, 2013, FOOD CHEM TOXICOL, V52, P163, DOI 10.1016/j.fct.2012.11.016

Livak KJ, 2001, METHODS, V25, P402, DOI 10.1006/meth.2001.1262

Lopresti AL, 2018, J AFFECT DISORDERS, V232, P349, DOI 10.1016/j.jad.2018.02.070

Lu PW, 2015, INT J CLIN EXP MED, V8, P20316

Luo YS, 2021, ONCOTARGETS THER, V14, P111, DOI 10.2147/OTT.S254167

Luo YS, 2020, INT J CLIN EXP PATHO, V13, P912

Luo YS, 2017, PAK J PHARM SCI, V30, P1629

Mahdizadeh S, 2016, DARU, V24, DOI 10.1186/s40199-016-0155-8

Masetti R, 2012, ADV THER, V29, P747, DOI 10.1007/s12325-012-0047-3

Mashmoul M, 2014, J FUNCT FOODS, V8, P180, DOI 10.1016/j.jff.2014.03.017

Mawson Anthony R, 2012, Cancer Manag Res, V4, P233, DOI 10.2147/CMAR.S32449

Mazidi Mohsen, 2016, J Complement Integr Med, V13, P195, DOI 10.1515/jcim-2015-0043

Medema Rene H, 2011, F1000 Biol Rep, V3, P10, DOI 10.3410/B3-10

Mehri S, 2020, WOODHEAD PUBL FOOD S, P517, DOI 10.1016/B978-0-12-818638-1.00035-6

Mehri S, 2015, IRAN J BASIC MED SCI, V18, P902

Mehri S, 2012, CELL MOL NEUROBIOL, V32, P227, DOI 10.1007/s10571-011-9752-8

Modaghegh MH, 2008, PHYTOMEDICINE, V15, P1032, DOI 10.1016/j.phymed.2008.06.003

Mohajeri SA, 2010, J SEP SCI, V33, P2302, DOI 10.1002/jssc.201000183

Mohamadpour AH, 2013, IRAN J BASIC MED SCI, V16, P39

Mollaei H, 2017, INT J CANCER MANAG, V10, DOI 10.5812/ijcm.11152

Mollaei H, 2017, BIOMED PHARMACOTHER, V94, P307, DOI 10.1016/j.biopha.2017.07.052

Moradzadeh M, 2019, J CELL BIOCHEM, V120, P4732, DOI 10.1002/jcb.27525

Moradzadeh M, 2019, J CELL BIOCHEM, V120, P1943, DOI 10.1002/jcb.27489

Moshiri E, 2006, PHYTOMEDICINE, V13, P607, DOI 10.1016/j.phymed.2006.08.006

Mostafavinia SE, 2016, DNA CELL BIOL, V35, P340, DOI 10.1089/dna.2015.3208

Mousavi B, 2015, AVICENNA J PHYTOMEDI, V5, P413

Naghizadeh Bahareh, 2008, Iran Biomed J, V12, P93

Nam KN, 2010, EUR J PHARMACOL, V648, P110, DOI 10.1016/j.ejphar.2010.09.003

Namayandeh Seyedeh Mahdieh, 2020, Asian Pac J Cancer Prev, V21, P1487, DOI 10.31557/APJCP.2020.21.5.1487

Nasimian A, 2020, BIOCHEM PHARMACOL, V177, DOI 10.1016/j.bcp.2020.113999

Nie ZG, 2019, MOL MED REP, V20, P401, DOI 10.3892/mmr.2019.10267

Nikbakht-Jam I, 2016, EUR J INTEGR MED, V8, P307, DOI 10.1016/j.eujim.2015.12.008

Noorbala AA, 2005, J ETHNOPHARMACOL, V97, P281, DOI 10.1016/j.jep.2004.11.004

Noureini SK, 2012, ASIAN PAC J CANCER P, V13, P2305, DOI 10.7314/APJCP.2012.13.5.2305

Papandreou MA, 2011, BEHAV BRAIN RES, V219, P197, DOI 10.1016/j.bbr.2011.01.007

Peng AM, 2013, CELL BIOSCI, V3, DOI 10.1186/2045-3701-3-20

Petrenko Yu. A., 2005, Ukrainskii Biokhimicheskii Zhurnal, V77, P100

Pitsikas N, 2006, BEHAV BRAIN RES, V173, P112, DOI 10.1016/j.bbr.2006.06.005

Pitsikas N, 2015, EVID-BASED COMPL ALT, V2015, DOI 10.1155/2015/926284

Premkumar K, 2003, PHYTOTHER RES, V17, P614, DOI 10.1002/ptr.1209

Qi Y, 2013, EXP EYE RES, V107, P44, DOI 10.1016/j.exer.2012.11.011

Rahaiee S, 2017, INT J BIOL MACROMOL, V99, P401, DOI 10.1016/j.ijbiomac.2017.02.095

Rahaiee S, 2015, J FOOD SCI TECH MYS, V52, P1881, DOI 10.1007/s13197-013-1238-x

Rahimi G, 2022, PHYTOTHER RES, V36, P2605, DOI 10.1002/ptr.7474

Ramadan A, 2012, J SAUDI CHEM SOC, V16, P13, DOI 10.1016/j.jscs.2010.10.012

Ramakers C, 2003, NEUROSCI LETT, V339, P62, DOI 10.1016/S0304-3940(02)01423-4

Raudvere U, 2019, NUCLEIC ACIDS RES, V47, pW191, DOI 10.1093/nar/gkz369

Ray P, 2016, SCI REP-UK, V6, DOI 10.1038/srep32979

Razavi BM, 2013, CHEM-BIOL INTERACT, V203, P547, DOI 10.1016/j.cbi.2013.03.010

Reynolds CP, 2003, CANCER LETT, V197, P185, DOI 10.1016/S0304-3835(03)00108-3

Reynolds CP, 2001, HEMATOL ONCOL CLIN N, V15, P867, DOI 10.1016/S0889-8588(05)70256-2

Salem M, 2022, SCI REP-UK, V12, DOI 10.1038/s41598-022-09109-9

Samarghandian S, 2013, BIOMED RES INT, V2013, DOI 10.1155/2013/417928

Samarghandian S, 2010, PHARMACOGN MAG, V6, P309, DOI 10.4103/0973-1296.71799

Schmidt M, 2007, WIEN MED WOCHENSCHR, V157, P315, DOI 10.1007/s10354-007-0428-4

Schramm A, 2016, HUM VACC IMMUNOTHER, V12, P2257, DOI 10.1080/21645515.2016.1171430

See SJ, 2004, NEURO-ONCOLOGY, V6, P253, DOI 10.1215/S1152851703000607

Sepahi S, 2018, AM J OPHTHALMOL, V190, P89, DOI 10.1016/j.ajo.2018.03.007

Serrano-Díaz J, 2012, J FOOD SCI, V77, pC1162, DOI 10.1111/j.1750-3841.2012.02926.x

Shahmansouri N, 2014, J AFFECT DISORDERS, V155, P216, DOI 10.1016/j.jad.2013.11.003

Shariat Razavi Seyedeh Mahya, 2020, Asian Pac J Cancer Prev, V21, P1959, DOI 10.31557/APJCP.2020.21.7.1959

Shi LP, 2018, MOL MED REP, V17, P7947, DOI 10.3892/mmr.2018.8835

Shoeb M., 2006, Bangladesh J. Pharmacol., V1, P35, DOI DOI 10.3329/BJP.V1I2.486

Siddiqui SA, 2022, MOLECULES, V27, DOI 10.3390/molecules27072076

Skowron, 1998, Int J Occup Saf Ergon, V4, P107

Soeda S, 2001, LIFE SCI, V69, P2887, DOI 10.1016/S0024-3205(01)01357-1

Song YN, 2021, FITOTERAPIA, V153, DOI 10.1016/j.fitote.2021.104969

Sugiura M, 1995, P JPN ACAD B-PHYS, V71, P319, DOI 10.2183/pjab.71.319

Sun J, 2011, ASIAN PAC J CANCER P, V12, P2679

Sun Y, 2015, W INDIAN MED J, V64, P473, DOI 10.7727/wimj.2016.053

Sun Y, 2013, EVID-BASED COMPL ALT, V2013, DOI 10.1155/2013/690164

Sung H, 2021, CA-CANCER J CLIN, V71, P209, DOI 10.3322/caac.21660

Taheri F., 2014, PATHOBIOLOGY RES, V17, P67

Tang Y, 2022, TOXICOL APPL PHARM, V437, DOI 10.1016/j.taap.2022.115892

Tarantilis PA, 1998, SPECTROCHIM ACTA A, V54, P651, DOI 10.1016/S1386-1425(98)00024-9

TARANTILIS PA, 1995, J CHROMATOGR A, V699, P107, DOI 10.1016/0021-9673(95)00044-N

TARANTILIS PA, 1994, ANTICANCER RES, V14, P1913

Tavakkol-Afshari J, 2008, FOOD CHEM TOXICOL, V46, P3443, DOI 10.1016/j.fct.2008.08.018

Trujillo-Jiménez F, 2004, ACTA HORTIC, P463, DOI 10.17660/ActaHortic.2004.650.56

Vali F, 2015, INT J BREAST CANCER, V2015, DOI 10.1155/2015/139349

van Vugt MATM, 2004, CELL CYCLE, V3, P1383, DOI 10.4161/cc.3.11.1248

Vazifedan V, 2017, IRAN J PHARM RES, V16, P230

Veisi A, 2020, IRAN J BASIC MED SCI, V23, P3, DOI [10.22038/IJBMS.2019.37821.8995, 10.22038/ijbms.2019.37821.8995]

Wang GF, 2018, EXP THER MED, V16, P5079, DOI 10.3892/etm.2018.6865

Wang H, 2012, ANTI-CANCER AGENT ME, V12, P1281, DOI 10.2174/187152012803833026

Wang K, 1996, GENE DEV, V10, P2859, DOI 10.1101/gad.10.22.2859

Winterhalter P, 2000, FOOD REV INT, V16, P39, DOI 10.1081/FRI-100100281

Xi L, 2007, PHYTOMEDICINE, V14, P633, DOI 10.1016/j.phymed.2006.11.028

Xia D, 2015, NAT PROD COMMUN, V10, P249

Xu GL, 2006, J ASIAN NAT PROD RES, V8, P79, DOI 10.1080/10286020500044732

Xu GL, 2007, BASIC CLIN PHARMACOL, V100, P31, DOI 10.1111/j.1742-7843.2007.00001.x

Xu Hui-Juan, 2012, Zhongguo Shi Yan Xue Ye Xue Za Zhi, V20, P57

Xu QX, 2022, CYTOKINE, V154, DOI 10.1016/j.cyto.2022.155888

Xu ZJ, 2022, J ETHNOPHARMACOL, V285, DOI 10.1016/j.jep.2021.114873

Yao C, 2018, ONCOTARGETS THER, V11, P2017, DOI 10.2147/OTT.S154586

Zhang B, 2004, BMC BIOINFORMATICS, V5, DOI 10.1186/1471-2105-5-16

Zhang J, 2020, ANN TRANSL MED, V8, DOI 10.21037/atm-20-5882

Zhang KP, 2018, BIOMED PHARMACOTHER, V99, P96, DOI 10.1016/j.biopha.2018.01.042

Zhang YG, 2021, J BIOCHEM MOL TOXIC, V35, DOI 10.1002/jbt.22608

ZHANG YX, 1994, BIOL PHARM BULL, V17, P217, DOI 10.1248/bpb.17.217

Zhang ZY, 2013, PHARM BIOL, V51, P920, DOI 10.3109/13880209.2013.771190

Zhao Pei, 2008, Zhongguo Zhong Yao Za Zhi, V33, P1869

Zheng J, 2016, NUTRIENTS, V8, DOI 10.3390/nu8080495

Zheng YQ, 2007, BRAIN RES, V1138, P86, DOI 10.1016/j.brainres.2006.12.064

NR 209

TC 7

Z9 8

U1 0

U2 9

PU MDPI

PI BASEL

PA ST ALBAN-ANLAGE 66, CH-4052 BASEL, SWITZERLAND

EI 2076-3921

J9 ANTIOXIDANTS-BASEL

JI Antioxidants

PD JUN

PY 2022

VL 11

IS 6

AR 1074

DI 10.3390/antiox11061074

PG 38

WC Biochemistry & Molecular Biology; Chemistry, Medicinal; Food Science &

Technology

WE Science Citation Index Expanded (SCI-EXPANDED)

SC Biochemistry & Molecular Biology; Pharmacology & Pharmacy; Food Science

& Technology

GA 2N5WK

UT WOS:000818448600001

PM 35739971

OA Green Published, gold

DA 2025-04-09

ER

PT J

AU Ainslie, AP

Klaver, M

Voshart, DC

Gerrits, E

den Dunnen, WFA

Eggen, BJL

Bergink, S

Barazzuol, L

AF Ainslie, Anna P.

Klaver, Myrthe

Voshart, Danielle C.

Gerrits, Emma

den Dunnen, Wilfred F. A.

Eggen, Bart J. L.

Bergink, Steven

Barazzuol, Lara

TI Glioblastoma and its treatment are associated with extensive accelerated

brain aging

SO AGING CELL

LA English

DT Article

DE aging hallmarks; cancer treatment side effects; cognitive decline;

glioblastoma; neurodegeneration

ID ENRICHMENT ANALYSIS; ALZHEIMERS-DISEASE; TUMORS; DYSFUNCTION; KNOWLEDGE;

SURVIVORS; THERAPY

AB Progressive neurocognitive dysfunction is the leading cause of a reduced quality of life in patients with primary brain tumors. Understanding how the human brain responds to cancer and its treatment is essential to improve the associated cognitive sequelae. In this study, we performed integrated transcriptomic and tissue analysis on postmortem normal-appearing non-tumor brain tissue from glioblastoma (GBM) patients that had received cancer treatments, region-matched brain tissue from unaffected control individuals and Alzheimer's disease (AD) patients. We show that normal-appearing non-tumor brain regions of patients with GBM display hallmarks of accelerated aging, in particular mitochondrial dysfunction, inflammation, and proteostasis deregulation. The extent and spatial pattern of this response decreased with distance from the tumor. Gene set enrichment analyses and a direct comparative analysis with an independent cohort of brain tissue samples from AD patients revealed a significant overlap in differentially expressed genes and a similar biological aging trajectory. Additionally, these responses were validated at the protein level showing the presence of increased lysosomal lipofuscin, phosphorylated microtubule-associated protein Tau, and oxidative DNA damage in normal-appearing brain areas of GBM patients. Overall, our data show that the brain of GBM patients undergoes accelerated aging and shared AD-like features, providing the basis for novel or repurposed therapeutic targets for managing brain tumor-related side effects.

In this study, we performed integrated transcriptomic and tissue analysis comparing postmortem non-tumor brain tissue from glioblastoma (GBM) patients that had received cancer treatments, with region-matched brain tissue from unaffected control individuals and Alzheimer's disease patients. We show that normal-appearing non-tumor brain regions of patients with GBM display hallmarks of accelerated aging, in particular mitochondrial dysfunction, inflammation, and proteostasis deregulation.dagger image

C1 [Ainslie, Anna P.; Klaver, Myrthe; Voshart, Danielle C.; Barazzuol, Lara] Univ Groningen, Univ Med Ctr Groningen, Dept Radiat Oncol, Groningen, Netherlands.

[Ainslie, Anna P.; Klaver, Myrthe; Voshart, Danielle C.; Gerrits, Emma; Eggen, Bart J. L.; Bergink, Steven; Barazzuol, Lara] Univ Groningen, Univ Med Ctr Groningen, Dept Biomed Sci Cells & Syst, Groningen, Netherlands.

[Ainslie, Anna P.; Klaver, Myrthe] Univ Groningen, Univ Med Ctr Groningen, European Res Inst Biol Ageing, Groningen, Netherlands.

[den Dunnen, Wilfred F. A.] Univ Groningen, Univ Med Ctr Groningen, Dept Pathol & Med Biol, Groningen, Netherlands.

[Bergink, Steven] Univ Groningen, Univ Coll Groningen, Groningen, Netherlands.

[Bergink, Steven; Barazzuol, Lara] A Deusinglaan 1, NL-9713AV Groningen, Netherlands.

C3 University of Groningen; University of Groningen; University of

Groningen; University of Groningen; University of Groningen

RP Bergink, S; Barazzuol, L (corresponding author), A Deusinglaan 1, NL-9713AV Groningen, Netherlands.

EM s.bergink@rug.nl; l.barazzuol@umcg.nl

RI Bergink, Steven/AAE-3020-2019; Gerrits, Emma/GPS-8515-2022; Dunnen,

Wilfred/A-4034-2009

OI Bergink, Steven/0000-0002-1142-869X; Klaver, Myrthe/0000-0002-9512-254X;

Voshart, Danielle/0009-0008-7370-0338; den Dunnen,

Wilfred/0000-0002-4168-1207; Eggen, Bart/0000-0001-8941-0353

FU KWF Kankerbestrijding; Department of Pathology at the UMCG [1116, 1834,

2463]; Department of Biomedical Sciences of Cells and Systems at the

UMCG

FX We thank the sequencing facility at the University Medical Center

Groningen (UMCG) for performing the bulk RNA-sequencing. We thank Wierd

Kooistra from the Department of Pathology at the UMCG for the DNA

extraction from tumor samples. We thank Demi van Egmond and Tom van

Wezel from the Department of Pathology at the University Medical Center

Leiden for the tumor status analysis. We thank Takuya Oshima from the

Department of Biomedical Sciences of Cells and Systems at the UMCG for

the batch effect correction script. Postmortem brain tissues (frozen and

fixed) were acquired from the NIH NeuroBioBank (request ID 1116 and 1834

and 2463). The schematic in Figure 1 was designed using BioRender.com.

CR Ainslie A, 2021, OPEN BIOL, V11, DOI 10.1098/rsob.200296

Ajithkumar T, 2017, LANCET ONCOL, V18, pE91, DOI 10.1016/S1470-2045(17)30030-X

Al Dahhan NZ, 2022, NEURON, V110, P2215, DOI 10.1016/j.neuron.2022.04.009

Barnes J, 2015, ALZHEIMERS DEMENT, V11, P1349, DOI 10.1016/j.jalz.2014.12.007

Belarbi K, 2013, CANCER RES, V73, P1201, DOI 10.1158/0008-5472.CAN-12-2989

Blalock EM, 2004, P NATL ACAD SCI USA, V101, P2173, DOI 10.1073/pnas.0308512100

Bottero V, 2021, FRONT MOL NEUROSCI, V14, DOI 10.3389/fnmol.2021.747798

Carroll JE, 2022, NAT REV CLIN ONCOL, V19, P173, DOI 10.1038/s41571-021-00580-3

Congdon EE, 2018, NAT REV NEUROL, V14, P399, DOI 10.1038/s41582-018-0013-z

Constanzo J, 2020, PROG NEURO-PSYCHOPH, V102, DOI 10.1016/j.pnpbp.2020.109954

DEANGELIS LM, 1989, NEUROLOGY, V39, P789, DOI 10.1212/WNL.39.6.789

Dietrich J, 2010, ADV EXP MED BIOL, V678, P77

Dumitriu A, 2016, BMC MED GENOMICS, V9, DOI 10.1186/s12920-016-0164-y

Ge SX, 2018, BMC BIOINFORMATICS, V19, DOI 10.1186/s12859-018-2486-6

Gibson EM, 2021, TRENDS NEUROSCI, V44, P441, DOI 10.1016/j.tins.2021.02.003

Gondi V, 2016, NAT REV NEUROL, V12, P334, DOI 10.1038/nrneurol.2016.70

Grissa D, 2022, DATABASE-OXFORD, V2022, DOI 10.1093/database/baac019

Hoffmann C, 2018, NEURO-ONCOLOGY, V20, P268, DOI 10.1093/neuonc/nox150

Huiting W., 2021, Genome Instab. Dis, V2, P1, DOI [10.1007/s42764-020-00027-6, DOI 10.1007/S42764-020-00027-6]

Huiting W, 2022, ELIFE, V11, DOI [10.7554/eLife.70726, 10.7554/eLife.70726.sa0, 10.7554/eLife.70726.sa1, 10.7554/eLife.70726.sa2]

Iqbal K, 2010, CURR ALZHEIMER RES, V7, P656, DOI 10.2174/156720510793611592

Kim M, 2021, NEURO-ONCOL ADV, V3, DOI 10.1093/noajnl/vdab125

Labadorf A, 2015, PLOS ONE, V10, DOI 10.1371/journal.pone.0143563

Ladomersky E, 2020, CLIN CANCER RES, V26, P5232, DOI 10.1158/1078-0432.CCR-19-3874

Lee JH, 2021, MOL CELL, V81, P1515, DOI 10.1016/j.molcel.2021.01.019

Liao YX, 2019, NUCLEIC ACIDS RES, V47, pW199, DOI 10.1093/nar/gkz401

Liberzon A, 2011, BIOINFORMATICS, V27, P1739, DOI 10.1093/bioinformatics/btr260

Lim Sungsu, 2018, Exp Mol Med, V50, P1, DOI 10.1038/s12276-017-0008-7

Lin XZ, 2020, INT J MOL SCI, V21, DOI 10.3390/ijms21051666

Liu R, 2009, NEURO-ONCOLOGY, V11, P330, DOI 10.1215/15228517-2008-093

Lowry JK, 1998, ARCH NEUROL-CHICAGO, V55, P922, DOI 10.1001/archneur.55.7.922

Lustberg MB, 2023, NAT REV CLIN ONCOL, V20, P527, DOI 10.1038/s41571-023-00776-9

Makale MT, 2017, NAT REV NEUROL, V13, P52, DOI 10.1038/nrneurol.2016.185

Mekkes N., 2022, MEDRXIV, DOI [10.1101/2022.09.22.22280158, DOI 10.1101/2022.09.22.22280158]

Montay-Gruel P, 2018, RADIOTHER ONCOL, V129, P582, DOI 10.1016/j.radonc.2018.08.016

Mootha VK, 2003, NAT GENET, V34, P267, DOI 10.1038/ng1180

Moreno-García A, 2018, FRONT NEUROSCI-SWITZ, V12, DOI 10.3389/fnins.2018.00464

Newman AM, 2019, NAT BIOTECHNOL, V37, P773, DOI 10.1038/s41587-019-0114-2

Noble W, 2013, FRONT NEUROL, V4, DOI 10.3389/fneur.2013.00083

Nonnekens J, 2017, EMBO MOL MED, V9, P4, DOI 10.15252/emmm.201607062

Ostrom QT, 2022, NEURO-ONCOLOGY, V24, piii1, DOI 10.1093/neuonc/noac161

Ostrom QT, 2021, NEURO-ONCOLOGY, V23, P1, DOI 10.1093/neuonc/noab200

Pletscher-Frankild S, 2015, METHODS, V74, P83, DOI 10.1016/j.ymeth.2014.11.020

Raudvere U, 2019, NUCLEIC ACIDS RES, V47, pW191, DOI 10.1093/nar/gkz369

Robinson MD, 2010, BIOINFORMATICS, V26, P139, DOI 10.1093/bioinformatics/btp616

Sánchez-Valle J, 2017, SCI REP-UK, V7, DOI 10.1038/s41598-017-04400-6

Schumacher B, 2021, NATURE, V592, P695, DOI 10.1038/s41586-021-03307-7

Siegel BI, 2019, TRANSL ONCOL, V12, P908, DOI 10.1016/j.tranon.2019.03.004

Simmons DA, 2019, RADIOTHER ONCOL, V139, P4, DOI 10.1016/j.radonc.2019.06.006

Subramanian A, 2005, P NATL ACAD SCI USA, V102, P15545, DOI 10.1073/pnas.0506580102

Sykora P, 2015, NUCLEIC ACIDS RES, V43, P943, DOI 10.1093/nar/gku1356

Venkataramani V, 2019, NATURE, V573, P532, DOI 10.1038/s41586-019-1564-x

Venkatesh HS, 2019, NATURE, V573, P539, DOI 10.1038/s41586-019-1563-y

Venkatesh HS, 2015, CELL, V161, P803, DOI 10.1016/j.cell.2015.04.012

Wefel JS, 2016, LANCET ONCOL, V17, pE97, DOI 10.1016/S1470-2045(15)00380-0

Williams JB, 2021, BRAIN COMMUN, V3, DOI 10.1093/braincomms/fcab123

NR 56

TC 5

Z9 5

U1 0

U2 3

PU WILEY

PI HOBOKEN

PA 111 RIVER ST, HOBOKEN 07030-5774, NJ USA

SN 1474-9718

EI 1474-9726

J9 AGING CELL

JI Aging Cell

PD MAR

PY 2024

VL 23

IS 3

DI 10.1111/acel.14066

EA JAN 2024

PG 14

WC Cell Biology; Geriatrics & Gerontology

WE Science Citation Index Expanded (SCI-EXPANDED)

SC Cell Biology; Geriatrics & Gerontology

GA KU9M6

UT WOS:001143857000001

PM 38234228

OA Green Published, gold

DA 2025-04-09

ER

PT J

AU He, ZL

Liu, KZ

Scally, L

Manaloto, E

Gunes, S

Ng, SW

Maher, M

Tiwari, B

Byrne, HJ

Bourke, P

Tian, FR

Cullen, PJ

Curtin, JF

AF He, Zhonglei

Liu, Kangze

Scally, Laurence

Manaloto, Eline

Gunes, Sebnem

Ng, Sing Wei

Maher, Marcus

Tiwari, Brijesh

Byrne, Hugh J.

Bourke, Paula

Tian, Furong

Cullen, Patrick J.

Curtin, James F.

TI Cold Atmospheric Plasma Stimulates Clathrin-Dependent Endocytosis to

Repair Oxidised Membrane and Enhance Uptake of Nanomaterial in

Glioblastoma Multiforme Cells

SO SCIENTIFIC REPORTS

LA English

DT Article

ID LIPID-PEROXIDATION; CELLULAR UPTAKE; NEURONAL DEGENERATION;

MOLECULAR-MECHANISMS; GOLD NANOPARTICLES; OXIDATION; TOXICITY;

CYTOTOXICITY; CHOLESTEROL; METABOLISM

AB Cold atmospheric plasma (CAP) enhances uptake and accumulation of nanoparticles and promotes synergistic cytotoxicity against cancer cells. However, the mechanisms are not well understood. In this study, we investigate the enhanced uptake of theranostic nanomaterials by CAP. Numerical modelling of the uptake of gold nanoparticle into U373MG Glioblastoma multiforme (GBM) cells predicts that CAP may introduce a new uptake route. We demonstrate that cell membrane repair pathways play the main role in this stimulated new uptake route, following non-toxic doses of dielectric barrier discharge CAP. CAP treatment induces cellular membrane damage, mainly via lipid peroxidation as a result of reactive oxygen species (ROS) generation. Membranes rich in peroxidised lipids are then trafficked into cells via membrane repairing endocytosis. We confirm that the enhanced uptake of nanomaterials is clathrin-dependent using chemical inhibitors and silencing of gene expression. Therefore, CAP-stimulated membrane repair increases endocytosis and accelerates the uptake of gold nanoparticles into U373MG cells after CAP treatment. We demonstrate the utility of CAP to model membrane oxidative damage in cells and characterise a previously unreported mechanism of membrane repair to trigger nanomaterial uptake. This knowledge will underpin the development of new delivery strategies for theranostic nanoparticles into cancer cells.

C1 [He, Zhonglei; Liu, Kangze; Scally, Laurence; Manaloto, Eline; Gunes, Sebnem; Ng, Sing Wei; Bourke, Paula; Tian, Furong; Cullen, Patrick J.; Curtin, James F.] Technol Univ Dublin, Sch Food Sci & Environm Hlth, BioPlasma Res Grp, Dublin, Ireland.

[He, Zhonglei; Liu, Kangze; Manaloto, Eline; Gunes, Sebnem; Maher, Marcus; Byrne, Hugh J.; Tian, Furong; Curtin, James F.] Technol Univ Dublin, FOCAS Res Inst, Nanolab, Dublin, Ireland.

[He, Zhonglei; Liu, Kangze; Ng, Sing Wei; Bourke, Paula; Tian, Furong; Curtin, James F.] Technol Univ Dublin, Environm Sustainabil & Hlth Res Inst, Dublin, Ireland.

[Tiwari, Brijesh] TEAGASC, Food Res Ctr, Dept Food Biosci, Dublin, Ireland.

[Bourke, Paula] Queens Univ Belfast, Sch Biol Sci, IGFS, Belfast, Antrim, North Ireland.

[Cullen, Patrick J.] Univ Sydney, Sch Chem & Biomol Engn, Sydney, NSW, Australia.

C3 Teagasc; Queens University Belfast; University of Sydney

RP Curtin, JF (corresponding author), Technol Univ Dublin, Sch Food Sci & Environm Hlth, BioPlasma Res Grp, Dublin, Ireland.; Curtin, JF (corresponding author), Technol Univ Dublin, FOCAS Res Inst, Nanolab, Dublin, Ireland.; Curtin, JF (corresponding author), Technol Univ Dublin, Environm Sustainabil & Hlth Res Inst, Dublin, Ireland.

EM james.curtin@tudublin.ie

RI Liu, Kangze/AGA-1994-2022; Scally, Laurence/ABB-7721-2021; He,

Zhonglei/AAK-6100-2021; Tian, Furong/AAA-6037-2020; Bourke,

Paula/Y-2709-2019; Curtin, James/B-1669-2008; Cullen, PJ/C-1901-2008;

Byrne, Hugh/AAD-9713-2020; Tiwari, Brijesh K/K-6227-2013; Liu,

Kangze/U-2681-2018; Byrne, Hugh/A-2301-2011

OI Tiwari, Brijesh K/0000-0002-4834-6831; Scally,

Laurence/0000-0002-3246-725X; Liu, Kangze/0000-0002-9644-0670; Maher,

Marcus/0000-0003-4926-4624; Bourke, Paula/0000-0002-5607-8021; Ng, Sing

Wei/0000-0002-2361-9557; Byrne, Hugh/0000-0002-1735-8610; Manaloto,

Eline/0000-0003-0142-3390

FU TU DUBLIN Fiosraigh Research Scholarship programme; Science Foundation

Ireland [14/IA/2626, 16/BBSRC/3391]; Science Foundation Ireland (SFI)

[14/IA/2626, 16/BBSRC/3391] Funding Source: Science Foundation Ireland

(SFI)

FX This work is supported by TU DUBLIN Fiosraigh Research Scholarship

programme (Z.H., E.M., K.L., L.S., S.G., M.M.), Science Foundation

Ireland Grant Numbers 14/IA/2626 (P.B., H.B., P.C. and J.C.) and

16/BBSRC/3391.

CR Adibhatla RM, 2008, BMB REP, V41, P560, DOI 10.5483/BMBRep.2008.41.8.560

Adibhatla RM, 2010, ANTIOXID REDOX SIGN, V12, P125, DOI [10.1089/ars.2009.2668, 10.1089/ARS.2009.2668]

Alkilany AM, 2010, J NANOPART RES, V12, P2313, DOI 10.1007/s11051-010-9911-8

Andrews NW, 2018, CURR BIOL, V28, pR392, DOI 10.1016/j.cub.2017.12.034

Babington P, 2015, BIOINTERPHASES, V10, DOI 10.1116/1.4915264

Begum A, 2013, AIP ADV, V3, DOI 10.1063/1.4811464

BORCHMAN D, 1992, LIPIDS, V27, P261, DOI 10.1007/BF02536472

BYRNE HJ, 2019, COMPUT TOXICOL, V12, DOI DOI 10.1002/JBIO.201800328

CHAO CC, 1994, J PHARMACOL EXP THER, V269, P198

CHATTERJEE SN, 1988, FREE RADICAL BIO MED, V4, P51, DOI 10.1016/0891-5849(88)90011-1

Cheng XQ, 2014, J PHYS D APPL PHYS, V47, DOI 10.1088/0022-3727/47/33/335402

Chithrani BD, 2006, NANO LETT, V6, P662, DOI 10.1021/nl052396o

Collet G, 2014, PLASMA SOURCES SCI T, V23, DOI 10.1088/0963-0252/23/1/012005

Conway GE, 2019, SCI REP-UK, V9, DOI 10.1038/s41598-019-49013-3

Conway GE, 2016, BRIT J CANCER, V114, P435, DOI 10.1038/bjc.2016.12

Dolman Nick J, 2013, Curr Protoc Cytom, VChapter 12, DOI 10.1002/0471142956.cy1230s65

Drummen GPC, 2004, FREE RADICAL BIO MED, V36, P1635, DOI 10.1016/j.freeradbiomed.2004.03.014

Freese C, 2012, PART FIBRE TOXICOL, V9, DOI 10.1186/1743-8977-9-23

Fridman G, 2008, PLASMA PROCESS POLYM, V5, P503, DOI 10.1002/ppap.200700154

GALLAGHER CJ, 2006, CANC IMMUN, V66

GOLDSTEIN IM, 1977, BIOCHEM BIOPH RES CO, V75, P604, DOI 10.1016/0006-291X(77)91515-7

Guerrero-Preston R, 2014, INT J MOL MED, V34, P941, DOI 10.3892/ijmm.2014.1849

HALLIWELL B, 1992, ANN RHEUM DIS, V51, P1261, DOI 10.1136/ard.51.11.1261

He Z, 2018, SCI REP, V8

He ZL, 2019, MICRO NANO TECHNOL, P191, DOI 10.1016/B978-0-12-814029-1.00008-9

Idone V, 2008, TRENDS CELL BIOL, V18, P552, DOI 10.1016/j.tcb.2008.09.001

Irani S, 2015, ARCH MED SCI, V11, P1286, DOI 10.5114/aoms.2015.48221

Iversen TG, 2011, NANO TODAY, V6, P176, DOI 10.1016/j.nantod.2011.02.003

Jacob RF, 2005, J BIOL CHEM, V280, P39380, DOI 10.1074/jbc.M507587200

Jinno M, 2016, ARCH BIOCHEM BIOPHYS, V605, P59, DOI 10.1016/j.abb.2016.04.013

Kim GC, 2009, J PHYS D APPL PHYS, V42, DOI 10.1088/0022-3727/42/3/032005

Kim W, 2017, SCI REP, V7, P1, DOI [10.1038/s41598-016-0028-x, DOI 10.1038/S41598-016-0028-X]

Koivusalo M, 2010, J CELL BIOL, V188, P547, DOI 10.1083/jcb.200908086

Kong MG, 2011, J PHYS D APPL PHYS, V44, DOI 10.1088/0022-3727/44/17/174018

Lakadamyali M, 2006, CELL, V124, P997, DOI 10.1016/j.cell.2005.12.038

Leopold JA, 2009, FREE RADICAL BIO MED, V47, P1673, DOI 10.1016/j.freeradbiomed.2009.09.009

Leutner S, 2001, J NEURAL TRANSM, V108, P955, DOI 10.1007/s007020170015

Lu P, 2017, PLASMA PROCESS POLYM, V14, DOI 10.1002/ppap.201600207

Maher MA, 2014, TOXICOL IN VITRO, V28, P1449, DOI 10.1016/j.tiv.2014.07.014

Mattson MP, 1999, ANN NY ACAD SCI, V893, P154, DOI 10.1111/j.1749-6632.1999.tb07824.x

Mattson MP, 1998, TRENDS NEUROSCI, V21, P53, DOI 10.1016/S0166-2236(97)01188-0

Moiseev T, 2014, PLASMA SOURCES SCI T, V23, DOI 10.1088/0963-0252/23/6/065033

Pelkmans L, 2002, SCIENCE, V296, P535, DOI 10.1126/science.1069784

Petrescu AD, 2001, J BIOL CHEM, V276, P36970, DOI 10.1074/jbc.M101939200

Recek N, 2015, PLOS ONE, V10, DOI 10.1371/journal.pone.0119111

Reuter S, 2010, FREE RADICAL BIO MED, V49, P1603, DOI 10.1016/j.freeradbiomed.2010.09.006

Robertson MJ, 2014, NAT PROTOC, V9, P1592, DOI 10.1038/nprot.2014.106

Ryan JA, 2007, ANAL CHEM, V79, P9150, DOI 10.1021/ac0715524

Schmidt-Bleker A, 2016, PLASMA SOURCES SCI T, V25, DOI 10.1088/0963-0252/25/1/015005

Schuessel K, 2006, FREE RADICAL BIO MED, V40, P850, DOI 10.1016/j.freeradbiomed.2005.10.041

Sevanian A, 2000, FREE RADICAL BIO MED, V29, P306, DOI 10.1016/S0891-5849(00)00342-7

Shi L, 2017, FREE RADICAL BIO MED, V108, P904, DOI 10.1016/j.freeradbiomed.2017.04.368

Souto GD, 2016, ANAL BIOANAL CHEM, V408, P5443, DOI 10.1007/s00216-016-9641-6

Stockwell BR, 2017, CELL, V171, P273, DOI 10.1016/j.cell.2017.09.021

Stoffels E, 2003, J PHYS D APPL PHYS, V36, P2908, DOI 10.1088/0022-3727/36/23/007

Tsoli M, 2005, SMALL, V1, P841, DOI 10.1002/smll.200500104

Tsoukou E., 2018, Plasma Med, V8, P299, DOI [DOI 10.1615/PLASMAMED.2018028261, 10.1615/PlasmaMed.2018028261]

Van der Paal J, 2016, CHEM SCI, V7, P489, DOI 10.1039/c5sc02311d

Verma A, 2008, NAT MATER, V7, P588, DOI 10.1038/nmat2202

WANG LH, 1993, J CELL BIOL, V123, P1107, DOI 10.1083/jcb.123.5.1107

Zhu W, 2016, SCI REP-UK, V6, DOI 10.1038/srep21974

Zivkovic M, 2007, CANCER LETT, V246, P100, DOI 10.1016/j.canlet.2006.02.002

2018, POLIT ASIA, P1

NR 63

TC 28

Z9 28

U1 0

U2 12

PU NATURE PORTFOLIO

PI BERLIN

PA HEIDELBERGER PLATZ 3, BERLIN, 14197, GERMANY

SN 2045-2322

J9 SCI REP-UK

JI Sci Rep

PD APR 24

PY 2020

VL 10

IS 1

AR 6985

DI 10.1038/s41598-020-63732-y

PG 12

WC Multidisciplinary Sciences

WE Science Citation Index Expanded (SCI-EXPANDED)

SC Science & Technology - Other Topics

GA NA4EP

UT WOS:000559768900009

PM 32332819

OA Green Published, Green Submitted, gold

DA 2025-04-09

ER

PT J

AU Morita, K

Itoh, M

Nishibori, N

Her, S

Lee, MS

AF Morita, Kyoji

Itoh, Mari

Nishibori, Naoyoshi

Her, Song

Lee, Mi-Sook

TI Spirulina Non-Protein Components Induce BDNF Gene Transcription via HO-1

Activity in C6 Glioma Cells

SO APPLIED BIOCHEMISTRY AND BIOTECHNOLOGY

LA English

DT Article

DE Blue-green algae; Spirulina protein-deprived extract; BDNF mRNA levels;

HO-1 expression; Glial cells

ID AMYOTROPHIC-LATERAL-SCLEROSIS; MESSENGER-RNA EXPRESSION; NEUROTROPHIC

FACTOR; HEME OXYGENASE-1; BRAIN; PLATENSIS; DISEASE; EXCITOTOXICITY;

NEURODEGENERATION; DISORDERS

AB Blue-green algae are known to contain biologically active proteins and non-protein substances and considered as useful materials for manufacturing the nutritional supplements. Particularly, Spirulina has been reported to contain a variety of antioxidants, such as flavonoids, carotenoids, and vitamin C, thereby exerting their protective effects against the oxidative damage to the cells. In addition to their antioxidant actions, polyphenolic compounds have been speculated to cause the protection of neuronal cells and the recovery of neurologic function in the brain through the production of brain-derived neurotrophic factor (BDNF) in glial cells. Then, the protein-deprived extract was prepared by removing the most part of protein components from aqueous extract of Spirulina platensis, and the effect of this extract on BDNF gene transcription was examined in C6 glioma cells. Consequently, the protein-deprived extract was shown to cause the elevation of BDNF mRNA levels following the expression of heme oxygenase-1 (HO-1) in the glioma cells. Therefore, the non-protein components of S. platensis are considered to stimulate BDNF gene transcription through the HO-1 induction in glial cells, thus proposing a potential ability of the algae to indirectly modulate the brain function through the glial cell activity.

C1 [Morita, Kyoji; Itoh, Mari] Shikoku Univ, Sch Hlth Sci, Dept Nursing, Neuropharmacol Lab, Tokushima 7711192, Japan.

[Nishibori, Naoyoshi] Shikoku Jr Coll, Dept Food Sci & Nutr, Lab Cell Biol & Toxicol, Tokushima 7711192, Japan.

[Her, Song; Lee, Mi-Sook] Korea Basic Sci Inst, Div Bioimaging, Chuncheon Ctr, Chunchon 200701, South Korea.

C3 Korea Basic Science Institute (KBSI)

RP Morita, K (corresponding author), Shikoku Univ, Sch Hlth Sci, Dept Nursing, Neuropharmacol Lab, Tokushima 7711192, Japan.

EM kmorita@shikoku-u.ac.jp

FU Kohken Co. Inc. (Sapporo, Japan); Shikoku Kakoki Co. Ltd. (Tokushima,

Japan)

FX This work was supported in part by the funds provided from Kohken Co.

Inc. (Sapporo, Japan) and Shikoku Kakoki Co. Ltd. (Tokushima, Japan).

CR Allaman I, 2011, PSYCHOPHARMACOLOGY, V216, P75, DOI 10.1007/s00213-011-2190-y

Bermejo-Bescós P, 2008, TOXICOL IN VITRO, V22, P1496, DOI 10.1016/j.tiv.2008.05.004

Bova R, 1998, MOL BRAIN RES, V57, P321, DOI 10.1016/S0169-328X(98)00092-8

BRADFORD MM, 1976, ANAL BIOCHEM, V72, P248, DOI 10.1016/0003-2697(76)90527-3

Caudle WM, 2009, EXP NEUROL, V220, P230, DOI 10.1016/j.expneurol.2009.09.027

CHOMCZYNSKI P, 1987, ANAL BIOCHEM, V162, P156, DOI 10.1016/0003-2697(87)90021-2

Chu WL, 2010, BMC COMPLEM ALTERN M, V10, DOI 10.1186/1472-6882-10-53

Corona JC, 2007, EXPERT OPIN THER TAR, V11, P1415, DOI 10.1517/14728222.11.11.1415

Cowan CM, 2006, CURR TOP DEV BIOL, V75, P25, DOI 10.1016/S0070-2153(06)75002-5

Dartsch PC, 2008, PHYTOTHER RES, V22, P627, DOI 10.1002/ptr.2310

Doron R, 2012, LIFE SCI, V90, P995, DOI 10.1016/j.lfs.2012.05.014

Hung SY, 2010, NEUROPHARMACOLOGY, V58, P321, DOI 10.1016/j.neuropharm.2009.11.003

Khan M, 2005, PHYTOTHER RES, V19, P1030, DOI 10.1002/ptr.1783

Kim NH, 2008, INT J NEUROSCI, V118, P1523, DOI 10.1080/00207450802325603

Koutsilleri E, 2007, PARKINSONISM RELAT D, V13, pS329, DOI 10.1016/S1353-8020(08)70025-7

Kulshreshtha A, 2008, CURR PHARM BIOTECHNO, V9, P400, DOI 10.2174/138920108785915111

Lu HK, 2006, EUR J APPL PHYSIOL, V98, P220, DOI 10.1007/s00421-006-0263-0

Lu J, 2010, BRIT J NUTR, V103, P1573, DOI 10.1017/S0007114509993758

Mao T K, 2000, J Med Food, V3, P135, DOI 10.1089/jmf.2000.3.135

Mao TK, 2005, J MED FOOD, V8, P27, DOI 10.1089/jmf.2005.8.27

Marton LS, 2000, AM J PHYSIOL-HEART C, V279, pH2405, DOI 10.1152/ajpheart.2000.279.5.H2405

Mattson MP, 2003, NEUROMOL MED, V3, P65, DOI 10.1385/NMM:3:2:65

Morita K, 1999, BRAIN RES, V830, P179, DOI 10.1016/S0006-8993(99)01292-5

Morita K, 2009, J MOL NEUROSCI, V38, P31, DOI 10.1007/s12031-008-9156-5

Park HJ, 2008, ANN NUTR METAB, V52, P322, DOI 10.1159/000151486

Schipper HM, 2006, NEUROBIOL AGING, V27, P252, DOI 10.1016/j.neurobiolaging.2005.01.016

Schipper HM, 2004, FREE RADICAL BIO MED, V37, P1995, DOI 10.1016/j.freeradbiomed.2004.09.015

Schipper HM, 2000, EXP GERONTOL, V35, P821, DOI 10.1016/S0531-5565(00)00148-0
[truncated: 113,160 more chars]
